# Cation Selectivity in Biological Cation Channels Using Experimental Structural Information and Statistical Mechanical Simulation

**DOI:** 10.1371/journal.pone.0138679

**Published:** 2015-10-13

**Authors:** Justin John Finnerty, Alexander Peyser, Paolo Carloni

**Affiliations:** 1 Computational Biophysics, German Research School for Simulation Sciences, 52425 Jülich, Germany; 2 Simulation Lab Neuroscience—Bernstein Facility for Simulation and Database Technology, Institute for Advanced Simulation, Jülich Aachen Research Alliance, Forschungszentrum Jülich, 52425 Jülich, Germany; 3 Computational Biomedicine, Institute for Neuroscience and Medicine (INM-9) and Institute for Advanced Simulation (IAS-5), Forschungszentrum Jülich, 52425 Jülich, Germany; Zhejiang University, CHINA

## Abstract

Cation selective channels constitute the gate for ion currents through the cell membrane. Here we present an improved statistical mechanical model based on atomistic structural information, cation hydration state and without tuned parameters that reproduces the selectivity of biological Na^+^ and Ca^2+^ ion channels. The importance of the inclusion of step-wise cation hydration in these results confirms the essential role partial dehydration plays in the bacterial Na^+^ channels. The model, proven reliable against experimental data, could be straightforwardly used for designing Na^+^ and Ca^2+^ selective nanopores.

## Introduction

**Fig 1:**
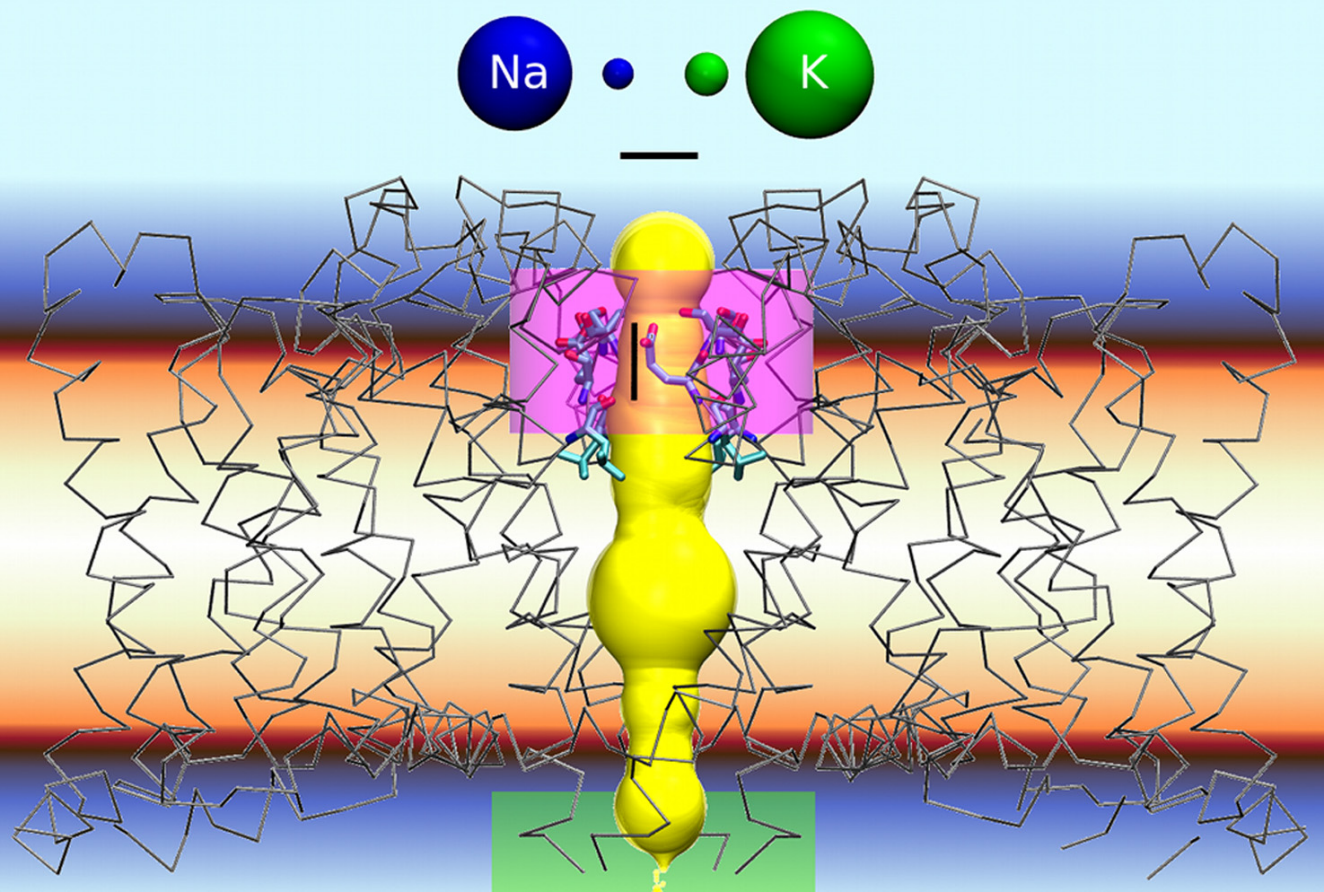
Cation channels from the voltage-gated like family [7] (represented here by NavAb; PDB 3RVY) [13] consist of a four domain transmembrane protein with an ion conduction pore (yellow), a cation selective filter at the extracellular pore mouth (SF, pink) and a gate region at the intracellular pore end (green). Inward pointing carbonyl oxygens from the backbone amide groups provide a lining of the SF [5, 6]. The charged side chains present in the SFs of the bacterial Na^+^ channels and the Ca^2+^ selective CavAb [18] structure are embedded in the pore lining. Hydrated (large) and fully dehydrated (small) Na^+^ (blue) and K^+^ (green) cations are shown to scale as spheres. Black bars are 5 Å.

Biological cation channels (Fig 1) allow the selective passage of Na^+^, K^+^ or Ca^2+^ through cell membranes [1–4] at passage rates near the diffusion limit. The cation discrimination occurs in the “selectivity filter” (SF, pink zone in Fig 1), a short, narrow section [5, 6] of the entire ion conduction pore (yellow volume in Fig 1).

In the voltage-gated-like (VGL) chanome [7] theory [8, 9], simulation [10] and experiment [11, 12] have shown the critical role that hydration plays in cation channels. It is now accepted that K^+^ channels transport K^+^ ions without a hydration shell [6]. Recent X-ray structures [13–15] and corresponding MD simulations [10, 16, 17] suggest that Na^+^ ions are transported with a partial hydration shell through bacterial Na^+^ selective ion channels. Additionally, the recent atomic structures of Na^+^ channels and a Ca^2+^ channel [18] show that the charged side-chains are embedded within the SF pore wall with only one of the oxygens of the carboxylic acid group exposed.

Here we investigate the role of hydration using simplified models. Simplified models provide complementary information [19, 20] to atomistic models, giving insights on the value of specific factors for selectivity. A major advantage of such methods is that by calculating properties using rigorous statistical thermodynamics, estimates of the value of a property have well-defined convergence due to the Central Limit Theorem. The top-down approach of simplified models means they may intrinsically identify the simplest and most robust physical principles responsible for a phenomenon, thus identifying the critical features needed to intentionally engineer artificial cation-selective nanopores.

The simplified model we focus on is based on the CSC Hamiltonian (Eq 1), [21–34] which has been successfully used to study the properties of mammalian Ca^2+^ channels; for example explaining the anomalous mole fraction effect [35, 36]. A major feature of the original CSC Hamiltonian is that the exact formulae for the included physics are used and it therefore has no parameters which are adjusted to tune the simulation results. This contrasts with classical molecular dynamics where a force field is parameterized to reproduce a set of representative physical properties. The key output of the CSC MC simulation is a population profile of ions within the simulation cell, and in particular within the SF. The competition between the cations for occupancy of the SF, which can be deduced from these profiles, determines the ion channel selectivity (*α*). This is measured experimentally as the ratio of the conductance of two ionic currents. We also compare concentration profiles for different cations from a single simulation, avoiding many of the problems associated with comparing absolute values from different simulations [37].

**Fig 2:**
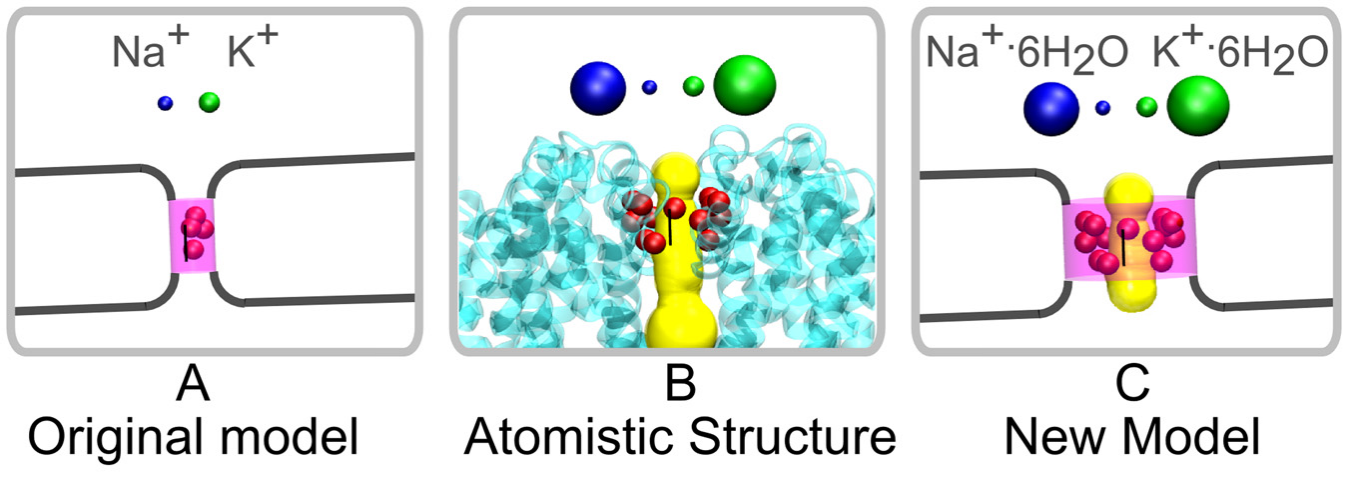
The geometry of the SF in the original CSC model (A, pink) was empirically parameterised to reproduce the cavity of the real SF (B, yellow). In the new model we use the atoms that line the pore (B and C, red balls) to reproduce the pore cavity (C, yellow). Using a realistic SF requires considering cation hydration (Hydrated(large) and fully dehydrated (small) Na^+^ (blue) and K^+^ (green) cations as spheres).

**Fig 3:**
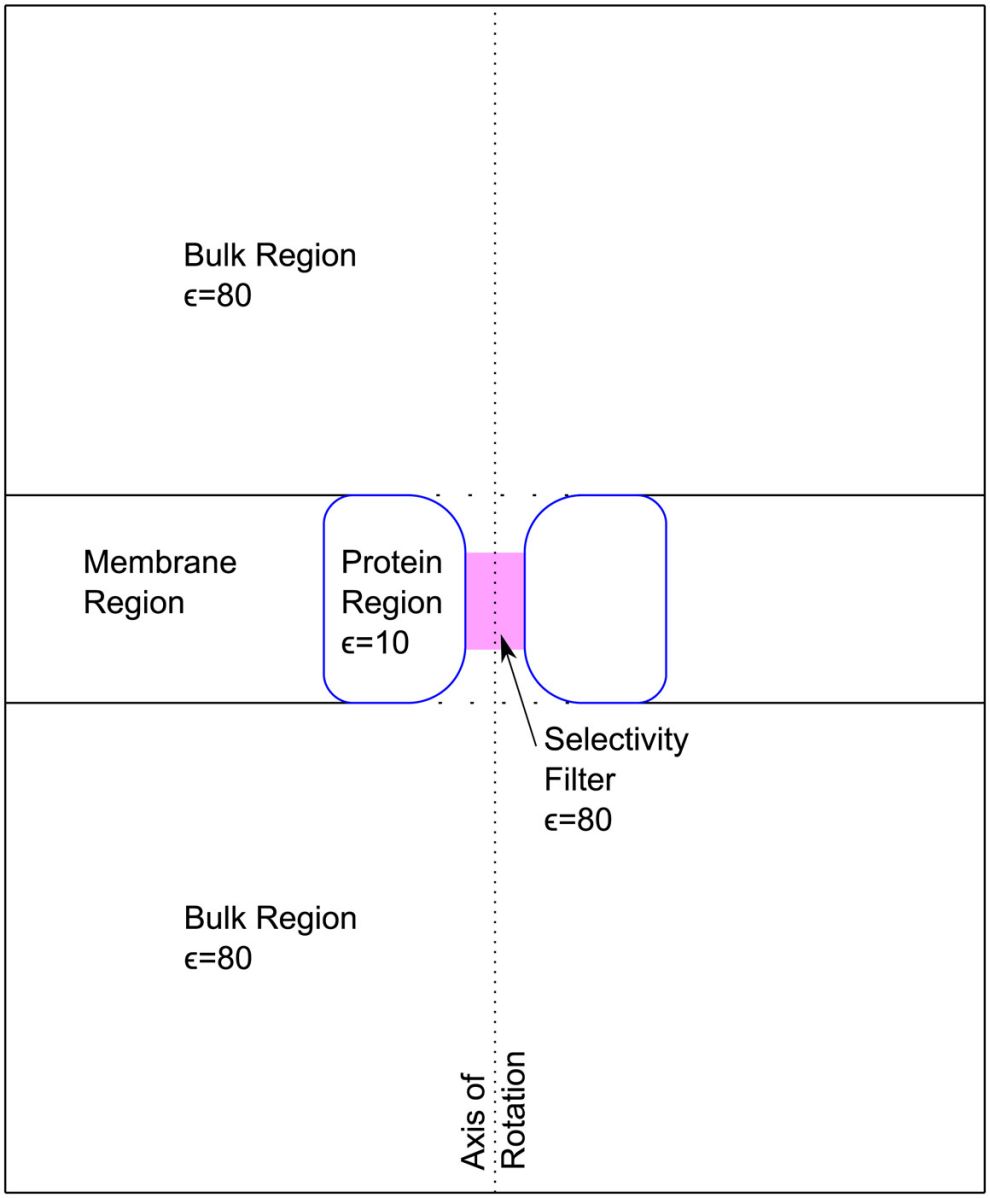
Schematic view of the simulation cell used here. The cell is a cylinder divided by a virtual membrane perpendicular to the cylinder’s axis of rotation. The model cation channel forms the only pore through the membrane and is located at the cylinder’s axis of rotation. The blue outline shows the dielectric boundary surface of the protein. The pink region shows where the spheres modelling the atoms lining the SF are located.

The core of the original CSC Hamiltonian is the competition between charge and space. The competition for space is modulated by a cylinder (pink regions in Figs 2 and 3) through which the spheres representing the solute ions must pass. The original CSC model used parameters for the cylinder deduced from experiments [11] combined with solute ion spheres with sizes that matched those used to deduce the cylinder parameters. The charge competition arises through charged spheres representing the side-chain charges, located within the cylinder, that interact with the solute ions as they pass through the cylinder (Fig 2A). To get closer to an atomistic representation of the pore (Fig 2B) means the existing representation can not be used directly. We have to either embed the side-chain charge into the cylinder representing the pore or adopt an alternative representation of the pore. Our recent modification of the CSC Hamiltonian [38] allows us reproduce the position and flexibility of any atom in an X-ray structure. Using this scheme we can build up an alternative representation of the pore from the atoms in the experimental structures that actually line the pore wall (Fig 2C). This would include charged atoms from side-chains in the Na^+^ and Ca^2+^ ion channels without needing to treat them as a special case. Hence the competition for space is now modulated by the volume defined by these pore wall spheres. Implicit in using such a volume to modulate the competition for space is that the spheres representing the solute ions also have sizes that correspond to the atomistic structure (Fig 2 balls at top). These solute ions vary in hydration state, being completely dehydrated in the KcsA channel [6] and partially dehydrated in the Na^+^ ion channels [10, 13–17]. Thus, determining the appropriate size of solute ions involves the consideration of hydration and how full and partial hydration will change the size of these ions as they take part in the competition for space within the SF pore (Fig 2C).

Here we add another important ingredient, cation hydration, to our simplified model. We describe our work to combine partial hydration and an atomistic representation of the SF pore wall in a manner conceptually consistent with the original CSC model. The good agreement between the presented model and experimental selectivity for Na^+^ and Ca^2+^ channels suggests that the included electrostatics, volume exclusion and hydration capture sufficient physics to explain selectivity for SFs containing charged residues, which are typical for Na^+^ and Ca^2+^ selective channels.

## Methods

The changes to the CSC model presented here change how the model is constructed but does not change the CSC Hamiltonian used in the simulation. In this paper we present the following:

- An atomistic representation of the SF pore wall. This requires modelling the oxygens of the carbonyl groups lining the SF using a partial charge on one particle as a representation of the carbonyl functional group and its dipole.
- A specific model of cation hydration tailored to the CSC cation channel simulations. It introduces no change to the CSC Hamiltonian, preserving the property of a model without adjustable parameters. The hydration model consists of three parts.
  ∘ The population distribution of the various hydrated cations in solution, calculated directly from the Boltzmann distribution and experimental partial dehydration energies.
  ∘ A Grand Canonical Monte Carlo move that swaps a cation between hydration states.
  ∘ A spherical approximation for the various hydrated cation states passing through the ion channel. The sphere diameters are Hille ‘gate-size” [11] and are based on geometric considerations and experimental data for hydrated cations in solution.

We chose the CSC Hamiltonian combined with grand canonical Monte Carlo simulations because they provide cation density profiles in the SF with well defined convergence characteristics from which selectivities can be derived. The CSC combines a Hamiltonian (Eq 1) and simulation set up that includes:

$$H=U_{C}+U_{IC}+U_{\text{mob}}+U_{\text{cp}}+U_{\text{overlap}}$$ (1)

- A cylindrical simulation cell divided into two equal halves by a virtual membrane (Fig 3). These halves represent the bulk solution either side of the membrane.
- A toroid shape, embedded in the virtual membrane, to represent the ion channel protein (Fig 3, blue outline).
  ∘ The surface of the toroid represents the dielectric boundary between the aqueous media and protein. The model computes the induced surface charges on this boundary surface (*U*_IC_) [39].
  ∘ The pore of the toroid represents the ion channel pore and provides a volume connecting the bulk volumes either side of the membrane.
  ∘ The radius and length of the narrowest part of the pore of the toroid are chosen to model the SF of the ion channel (more information in the supporting material). The dimensions of the SF are now fitted from the X-ray structure rather than using empirical estimates from literature.
- Selected atoms from the protein are placed in the narrow pore of the toroid to represent the pore wall and charge sites in the SF of the ion channel protein.
  ∘ These atoms are localized (*U*_mob_) with positions and flexibility taken directly from the coordinates and B-factors of the X-ray crystal structure [38].
- Charge-charge interactions (*U*_C_) are modeled using the Coulomb equation for dielectric media.
- Spatial interactions are handled using hard-body overlap (*U*_overlap_). With charged particles not being able to overlap each other or the volume contained within the protein and membrane.
- The concentration of the solute ions in the simulation is maintained by a fixed chemical potential (*U*_cp_; grand canonical *μVT* ensemble).

### Representation of the pore wall

Including charged and uncharged atoms that line the SF means we can embed charge sites into the pore wall and at the same time impose the same volume constraints that are present in the atomistic structure. These pore wall spheres are surrounded by a cylinder (shaded region in Fig 3), whose role in the competition for space is now to ensure solute ions can not go “outside” the pore wall spheres. For the ion channels studied here, the SF pore wall is composed of polar carbonyl oxygen atoms of the protein backbone [6, 13–15]. These could be represented using two spheres with opposite charges to form a dipole. However, as a first approximation we tested representing these groups using a single partial negative charge sphere. We considered that this approximation is a reasonable starting point as the influence of individual charges and asymmetry in charge reduces very rapidly with distance because our model ions exist in the strong dielectric media of water.

The embedding of the charged side groups in the SF pore wall also caused us to reconsider the earlier approach used in the CSC model of representing carboxylic acid groups as two half charged oxygen atoms. As an approaching cation would cause the charge to localize on the oxygen atom nearest to the pore wall, a single ion appeared to better capture these influential short-range electrostatic interactions better than a two ion representation. We therefore chose to represent the carboxylic group using a single charged atom in the position of the oxygen nearest to the pore wall.

### Hydration model

Each cation type passing through the channel may lose differing numbers of waters of hydration in order to fit through the narrowest part of SF. Our main assumption is that we can separate the hydration and dehydration reaction from the passage through the ion channel. For Na^+^ and K^+^ cations, this assumption is reasonable since the experimentally measured residence time of waters in the hydration shell of these cations (10^−10^s) [40] is two orders of magnitude shorter than cation passage through an ion channel (10^−8^s) [11]. This is not the case for divalent Ca^2+^. However the much higher energetic cost of removal of waters from the Ca^2+^ compared to monovalent ions (Table 1) means we can exclude dehydration from consideration when examining Ca^2+^ selective channels. Indeed, the CSC model without hydration has already provided accurate predictions of Ca^2+^ channel behavior [29, 30, 41].

**Table 1:** Model population factor ([A⌈*x*⌉]_bulk_[A]_bulk_) and cation diameters (*d*_*M*^+^,*x*_) for partially hydrated cations. Partial hydration energies used are experimental gas-phase data [42]. The base diameters of the hydrated cations were calculated from the experimental cation–water oxygen (*r*_*MO*_) and water oxygen–water oxygen (*r*_*OO*_) radial distribution functions in ionic solutions [40, 43]; see Fig 4, ${\text{d}}_{{\text{M}}^{+},6}=2\left(r_{MO}+\frac{1}{2}r_{OO}\right)$.

	K^+^		Na^+^		Ca^2+^	
	$\frac{{\left[{\text{K}}^{+}\left\lceil x\right\rceil \right]}_{\text{bulk}}}{{\left[{\text{K}}^{+}\right]}_{\text{bulk}}}$	*d*_K^+^,*x*_	$\frac{{\left[{\text{Na}}^{+}\left\lceil x\right\rceil \right]}_{\text{bulk}}}{{\left[{\text{Na}}^{+}\right]}_{\text{bulk}}}$	*d*_Na^+^,*x*_	$\frac{{\left[{\text{Ca}}^{2+}\left\lceil x\right\rceil \right]}_{\text{bulk}}}{{\left[{\text{Ca}}^{2+}\right]}_{\text{bulk}}}$	*d*_Ca^2+^,*x*_
*x* = 6	1	8.32	1	7.44	1	7.44
5	2.2×10^−2^	≈ 8.32	5.8×10^−3^	≈ 7.44	1.8×10^−16^	≈ 7.44
4	5.5×10^−5^	6.21	1.7×10^−5^	5.44	4.4×10^−34^	5.44
3	2.5×10^−8^	5.54	3.7×10^−10^	5.10	8.5×10^−53^	5.10
2	7.9×10^−13^	2.8	6.3×10^−17^	2.0		

#### Populations of partially hydrated cations

The major output of the CSC MC simulation is a population profile of ions within the simulation cell, and in particular within the SF. The separation of timescales between hydration and cation passage for Na^+^ and K^+^ ions means we can divide each cation population into hydration state subpopulations, all of which are present in solution:

$$\begin{array}{l} {}[A]=[A\cdot 0{\text{H}}_{2}\text{O}]+[A\cdot 1{\text{H}}_{2}\text{O}]+[A\cdot 2{\text{H}}_{2}\text{O}]\ldots \\ {}[B]=[B\cdot 0{\text{H}}_{2}\text{O}]+[B\cdot 1{\text{H}}_{2}\text{O}]+[B\cdot 2{\text{H}}_{2}\text{O}]\ldots \end{array}$$

where the total concentration of ions [*A*] and [*B*] are the sum of hydration state subpopulations. We can calculate these relative populations (see Table 1) using the Boltzmann equation and the relative dehydration free energies. This can also be applied to the SF to give:

$$\begin{array}{l} {}[A\cdot X]=[(A\cdot 0{\text{H}}_{2}\text{O})\cdot X]+[(A\cdot 1{\text{H}}_{2}\text{O})\cdot X]\ldots \\ {}[B\cdot X]=[(B\cdot 0{\text{H}}_{2}\text{O})\cdot X]+[(B\cdot 1{\text{H}}_{2}\text{O})\cdot X]\ldots \end{array}$$

where [*A*⋅*X*] is the sum of the interactions of hydration state subpopulations of *A* at some point within the SF (*X*).

A complete model calculation would involve a simulation containing all hydration states. In practice two factors limit the practicality of such simulations. Firstly, the sub-populations *B* ⋅ *m*H_2_O and *A* ⋅ *n*H_2_O decrease by several orders of magnitude as the cation hydration number, *m* and *n*, decreases resulting in 〈[(*B* ⋅ *m*H_2_O) ⋅ *X*]〉 and 〈[(*A* ⋅ *n*H_2_O) ⋅ *X*]〉 rapidly converging to zero due to the limits of finite sized simulation. On the other hand, only values of *m* and *n*, that allow *B* ⋅ *m*H_2_O or *A* ⋅ *n*H_2_O to fit within the SF and therefore pass through the ion channel will have non-zero contributions to [*B* ⋅ *X*] and [*A* ⋅ *X*]. We therefore perform preferential sampling of the critical hydration states by ignoring values of *m* and *n* for which the values of 〈[(*B* ⋅ *m*H_2_O) ⋅ *X*]〉 and 〈[(*A* ⋅ *n*H_2_O) ⋅ *X*]〉 will be zero. This involves determining the largest hydration numbers, ⌈*m*⌉ and ⌈*n*⌉, that allows the hydrated cation to fit in the SF. These maximum numbers and consecutively lower values of *m* and *n* which have populations within three orders of magnitude of the populations of ⌈*m*⌉ and ⌈*n*⌉ are the hydration states included in the final simulations.

In these simulations the sum of the partially hydrated ions in the bulk is still that of the original [*A*]_bulk_. This enhanced sampling of the critical hydration states then requires a rescaling factor of the ratio of the sum of the sub-populations used in the simulations to the full population of *A* to be applied:

$$\frac{{\left[A\left\lceil n\right\rceil \right]}_{\text{bulk}}}{{\left[A\right]}_{\text{bulk}}}=\frac{\sum_{j<n}\left[A\cdot j{\text{H}}_{2}\text{O}\right]}{\sum_{i<\infty }\left[A\cdot i{\text{H}}_{2}\text{O}\right]}$$ (2)

#### Grand Canonical Monte Carlo swap move

The difference in time scales between cation passage and dehydration for Na^+^ and K^+^ cations implies that not only can we model dehydration as a set of individual hydration states, but we can also include transitions between these states as a grand canonical Monte Carlo move. We therefore add a new grand canonical move into the CSC MC model that swaps cation hydration states to model variation in the hydration state profile. Such a move is a concatenation of two simple grand canonical moves; a grand canonical deletion of the particle in the original hydration state and a grand canonical addition of the particle in the new hydration state at the location of the original particle.

#### Model of partially hydrated cations

The CSC Hamiltonian represents a cation’s spacial component as a sphere with the cation’s charge component being a point charge in the centre of the sphere. Therefore, the only size parameter is the cation diameter (*d*_*B*,*m*_ and *d*_*A*,*n*_). We choose, as a first approximation, to ignore the dipolar interactions between the cation and its waters of hydration and model hydrated ions using this simple spherical model. This model also eliminates any consideration of the rotational entropy due to the potentially asymmetric shape of the partially hydrated cation cluster. We anticipate, however, that since the cluster has very little freedom of rotation while in the SF due to the size constraints of the narrow pore, a spherical model can be a reasonable first approximation. With a sphere, two choices for modeling the size seemed appropriate (i) ignore the shape of the partially hydrated cations and model the sphere based on the occupied volume, or (ii) maintain some shape information and ignore the occupied volume. The most critical spacial component will be the space the cation occludes during motion along the pore axis because the SF is such a narrow tube ions have limited lateral mobility. For example, if we consider a cation with zero, one or two waters of hydration, these water of hydration could orient along the channel axis and all three hydration states would effectively occlude the same minimal *cross-sectional area* along the direction of motion. This suggests that the resistance to motion along the pore axis will be in proportion to this cross-sectional area and not to the total volume of the cation complex. We thus take the critical size parameter as the area the partially hydrated cation water cluster occludes along the direction of motion. These diameters are thus Hille’s “gate size” [11] rather than, for example, an “effective” diameter determined from cation diffusion experiments in water.

**Fig 4:**
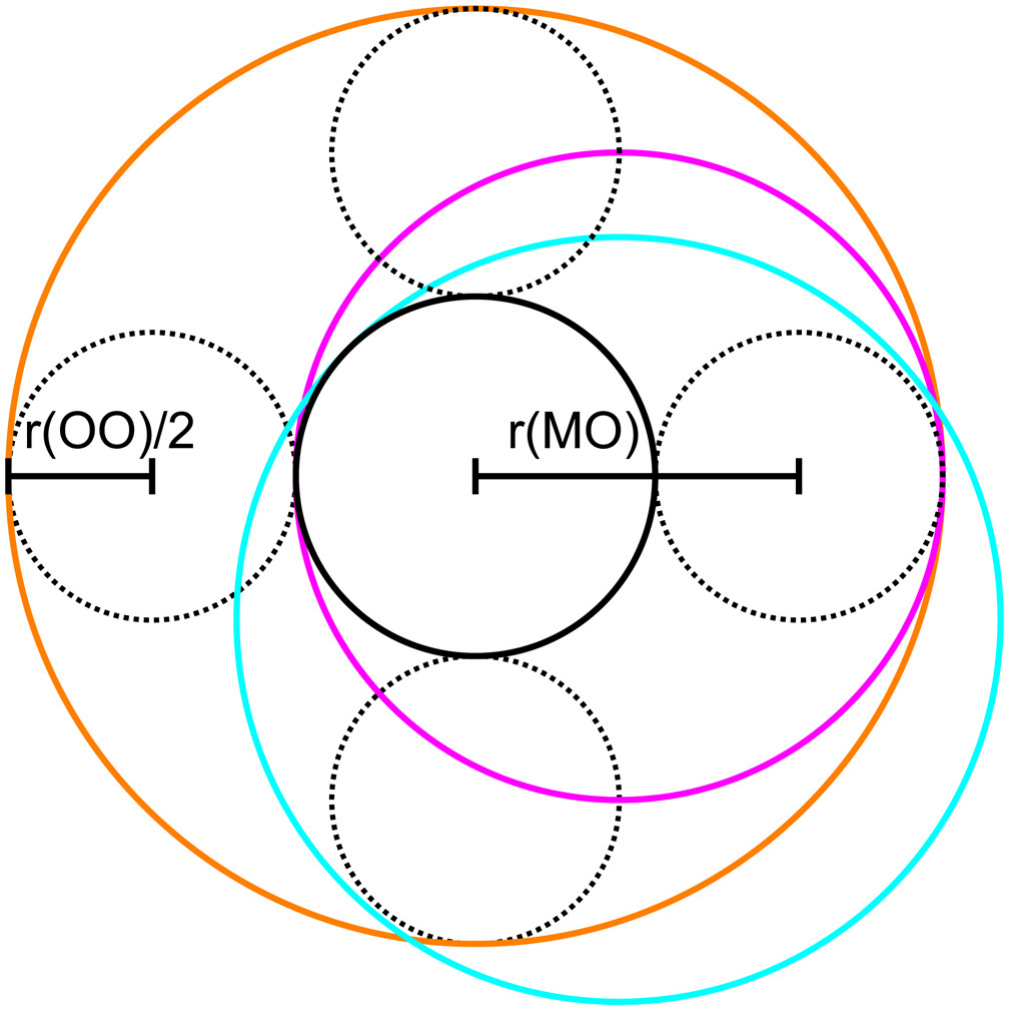
Schematic for the circumcircles that give the partially hydrated cation diameters, *d*_*M*^+^_, used in the CSC simulations (not drawn to scale). They follow the replacement of four waters of hydration (dashed circles) in a plane perpendicular to the channel axis. Removal of one water does not change the diameter (0, 1 = orange). Loss of more waters reduces the diameter (2 = blue, 3 = pink) to a minimum when four (or more) waters are lost (4 = black). The actual dimensions are calculated from the experimental cation–water oxygen distance (r(MO)) and water oxygen–water oxygen distance (r(OO)).

K^+^ and Na^+^ cations are normally hexa-coordinated in the first hydration shell [40, 42]. We assume that two of the waters of hydration can always be oriented along the SF pore axis and that they will be the last to be removed. This sets the four remaining coordination sites from which water is removed in a plane perpendicular to the SF axis (Fig 4). Using experimental cation–water oxygen (r_*MO*_) and water oxygen–water oxygen (r_*OO*_) distances [40, 43] we calculate *d*_*B*,*m*_ and *d*_*A*,*n*_ as the diameter of the smallest circumcircle around the cation and waters of hydration that occupy from zero to four of these planar sites (see Fig 4 and Table 1).

Note that this coordination constraint only applies to the water hydration shell. The model places no limitations on the coordination of ions with other species. In particular, the cations are free to coordinate with eight atoms in the SF.

### Simulation parameters

A major criterion when extending the model has been to keep the model as simple as possible and to use parameters directly derived from external sources, favoring experimental work. A major feature of the unmodified CSC Hamiltonian is that it had no parameters which are adjusted to tune the simulation results. The parameters that are in the CSC Hamiltonian are the fixed dielectric constant of water (80) and protein (10), the charge on an electron, Avogadro’s number, temperature and the Boltzmann factor. Input into the current model includes the atomic position and the B-factor of atoms from X-ray, the experimentally derived atomic radii and the geometry of the dielectric boundary surface between the aqueous and protein media. The dielectric boundary is modeled as a hard surface upon which an induced charge can develop. Previous work [30] has shown that the presence of the dielectric boundary is important but variation of the dielectric constant of the protein media (4–20) [44, 45] and the exact radius of the SF boundary surface [45] are less influential in determining selectivity.

In this work, we can not take the dimensions for the flattened toroid of the dielectric boundary representing the protein (blue line in Fig 3) directly from experiment. The most critical parameter is the diameter of the cylindrical part of the boundary surface in the SF (pink region in Fig 3). Previously, the dimensions of this boundary where critical for both control of pore volume and confinement of the electrostatic field. As we now represent the pore wall explicitly, the boundary surface no longer plays a crucial role in controlling pore volume. Additionally, previous work [45] showed that small changes in the diameter of the surface has a very small influence on the magnitude of the result. Our approach was therefore to maximise the freedom of the spheres representing the pore wall while avoiding solute cations being able to fit between these spheres and the boundary surface. Our initial estimate was a diameter slightly greater than the base atomic position plus particle radius of the spheres representing the pore wall of the SF. Test simulations were then performed to check if the diameter was too small and significantly truncated movement of the spheres of the pore wall or too large and allowed solute cations between these spheres and the boundary surface.

The parameter not taken directly from experiment or calculated through test simulations is the partial charge on the pore wall oxygen atoms. The impact of this charge will be most significant for the K^+^ channel model, where it is the only endogenous charge in the SF, compared to the Na^+^ and Ca^2+^ channel models that have fully charged residues in the SF. We therefore chose the value of −0.1 taken from the partial charge on the pore wall oxygen atoms seen from QM/MM MD simulations of the KcsA K^+^ channel [46]. This value was used universally for all pore wall oxygen atoms with no attempt to tune the parameter for specific selectivity.

### Software Availability

The software used to perform these simulations is available from https://github.com/charge-space-competition/ion-channel.

## Results

**Fig 5:**
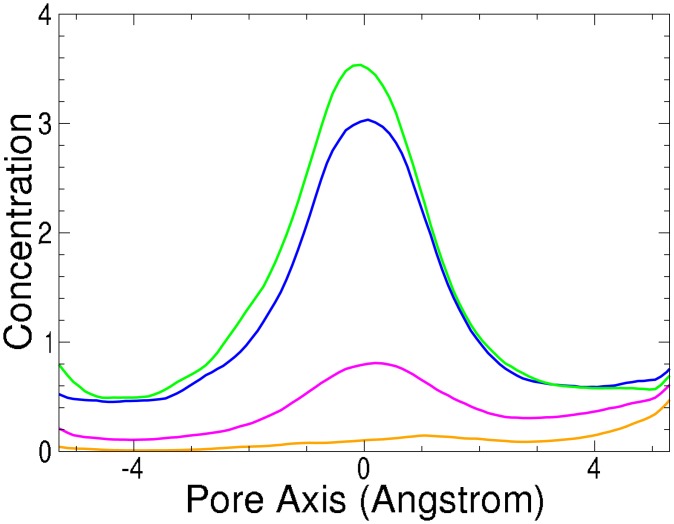
Simulated cation profile (as moving average over 2Å) in the Ca^2+^ (CavAb) ion channel SF. [Mn^2+^·*X*] green, [Ca^2+^·*X*] blue, [Na^+^·*X*] pink and [Ba^2+^·*X*] orange. Concentration minimum region used to calculate selectivity [−5.5 : −2.5].

**Fig 6:**
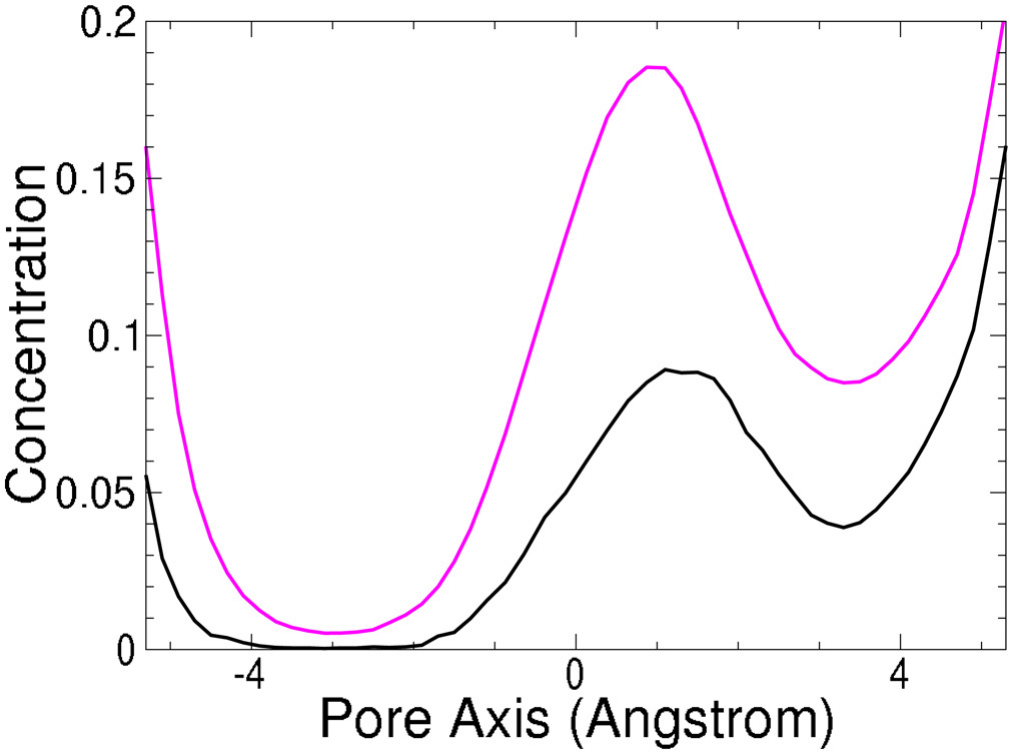
Simulated cation profile (as moving average over 2Å) in the Na^+^ (NavAb) ion channel SF. [Na^+^·*X*] pink and [K^+^·*X*] black. Concentration minimum region used to calculate selectivity [−4.0 : −2.0].

**Fig 7:**
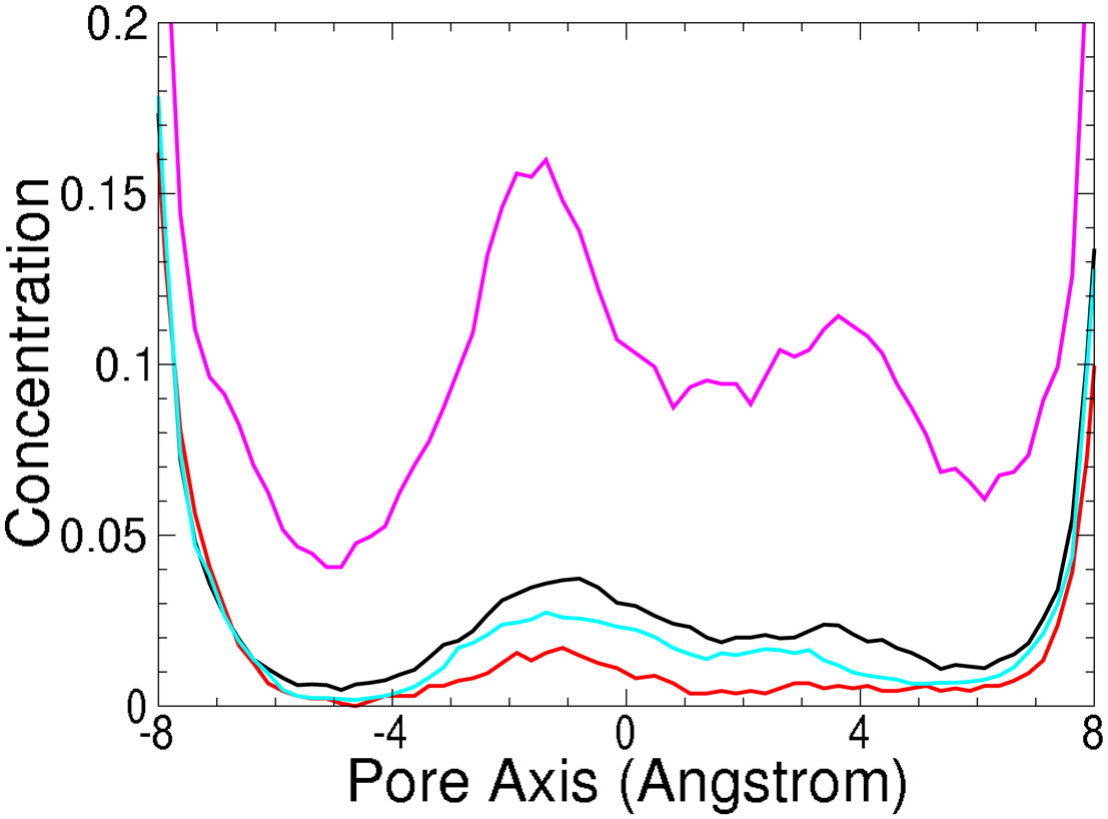
Simulated cation profile (as moving average over 2Å) in the K^+^ (KcsA) ion channel SF. [Na^+^·*X*] pink, [K^+^·*X*] black, [$\text{NH}{}_{4}^{+}$·*X*] red and [Rb^+^·*X*] or [Tl^+^·*X*] light blue. Concentration minimum region used to calculate selectivity [−6.5 : −2.5].

We simulated the SF from two bacterial Na^+^ channels (NavAb [13] (Fig 1) and NavMs [14]), a typical K^+^ channel (KcsA [6, 47]) and the Ca^2+^ selective mutant of the bacterial NavAb Na^+^ channel (CavAb) [18], the only Ca^2+^ selective channel in the VGL chanome [7] for which an X-ray structure has been determined. The simulated concentration profiles of solute cations within the SF have a shape characteristic for each ion channel ([*A* ⋅ *X*] and [*B* ⋅ *X*], Figs 5 and 6, 7). The location of the minima and maxima for a particular channel occur in similar ranges across cations. Interpreting selectivity from these density profiles requires application of a mechanism of cation passage. A single-barrier mechanism and the assumption the cations have similar average velocities is one of the simplest models and corresponds closely to what Hille describes as a single-ion pore [11]. The calculation of selectivity from this single-barrier mechanism is straightforward and involves comparing cation concentration ratios at the rate determining barrier in the SF (i.e. the minima in density: [*A* ⋅ *X*]min and [*B* ⋅ *X*]min) to the ratios in the bulk solution (for details see the supporting information):

$$\alpha_{\text{sim}}=\frac{{\left[A\right]}_{\text{bulk}}{\left[B\cdot X\right]}_{\text{min}}}{{\left[B\right]}_{\text{bulk}}{\left[A\cdot X\right]}_{\text{min}}}$$ (3)

When hydration is included we may have a only subset of partially hydrated cations in the bulk giving:

$${\hat{\alpha }}_{\text{sim}}=\frac{{\left[A\left\lceil n\right\rceil \right]}_{\text{bulk}}{\left[B\cdot X\right]}_{\text{min}}}{{\left[B\left\lceil m\right\rceil \right]}_{\text{bulk}}{\left[A\cdot X\right]}_{\text{min}}}$$ (4)

where [m] and [n] are the maximum hydration states that still allow the partially hydrated cations to fit within the SF. Correcting the bulk concentration by the population factors (Eq 2) leads to the following:

$$\alpha_{\text{hyd}}=\frac{{\left[B\left\lceil m\right\rceil \right]}_{\text{bulk}}{\left[A\right]}_{\text{bulk}}}{{\left[A\left\lceil n\right\rceil \right]}_{\text{bulk}}{\left[B\right]}_{\text{bulk}}}$$ (5)

$$\alpha_{\text{sim}}={\hat{\alpha }}_{\text{sim}}\times \alpha_{\text{hyd}}$$ (6)

Our calculated *α* values (Table 2) show quantitative agreement for the Ca^2+^ and Na^+^ channels and qualitative agreement for the K^+^ channel.

**Table 2:** Predicted equilibrium constant values from simulations based on the ion channel X-ray structures. Calculated selectivities are shown relative to the cation selected by the specific channel. (Eq 6: $\alpha_{\text{sim}}=\alpha_{\text{hyd}}\times {\hat{\alpha }}_{\text{sim}}$).

Channel	Ions *A*,*B*	Hyd.	*α*_hyd_	${\hat{\alpha }}_{\text{sim}}$	*α*_sim_	Expt
KcsA [6]	K^+^,Na^+^	*n*,*m* ≤ 2	49,000	0.1	4,900	> 170 [47]
NavMs [14]	Na^+^,K^+^	*n*,*m* ≤ 4	0.3	30	9	11–18 [10]
NavAb [13]	Na^+^,K^+^	*n*,*m* ≤ 4	0.3	12	2.4	6–30 [16]
CavAb [18]	Ca^2+^,Na^+^	(*n*,*m* ≤ 6)	1	600	600	380 [18]

A key finding is that our model reproduces selectivity in the bacterial Na^+^ channels. The hydration level of cations in the Na^+^ channels studied is in agreement with suggestions from recent X-ray [13–15] and corresponding MD simulation [10, 16, 17] studies. Our results complement these studies by highlighting the critical role that partial dehydration plays in the selectivity. In our simulations the largest occupancies in the SF are the fourth Na^+^ hydration state, (Na ⋅ 4(H_2_O))^+^, and the third K^+^ state, (K ⋅ 3(H_2_O))^+^. Thus, even though hydration states 4 and 3 are included in our simulations for both Na^+^ and K^+^, the (K ⋅ 4(H_2_O))^+^ cation is not present in the SF. This allows the value of ${\hat{\alpha }}_{\text{Na,K}}$ to overcome the population disadvantage (Na ⋅ 4(H_2_O))^+^ has over (K ⋅ 4(H_2_O))^+^ in a real aqueous solution (*α*_hyd:Na,K_ = 0.3), leading to Na^+^ selectivity overall.

We introduced a simple model of hydration that contributes two key factors into the calculation of selectivity: (i) The population of the partially dehydrated ions as set by their relative partial dehydration energies (*α*_sim,dehyd_). (ii) The representation of the partially dehydrated cation as a sphere of a certain radius.

The first test of the hydration model and the pore wall is if it can reproduce the behavior of the original CSC Hamiltonian for Ca^2+^ ion channels. In our simulations, Ca^2+^ would not be dehydrated because it has a much larger dehydration energy than the monovalent cations (for example the loss of two water molecules would give Na^+^ a huge advantage *α*_hyd,Ca,Na_ = 3×10^−29^). The Ca^2+^ *α*_sim,hyd_ values (Table 3) for fully hydrated ions are in good agreement with experiment and earlier CSC simulations that were based on an empirical model of the SF using cation sizes equivalent to the dehydrated ions. This shows that including atomistic pore wall and hydration in the CSC model continues to reproduce the selectivity behavior of Ca^2+^ ion channels as seen for simple CSC models without the new features.

**Table 3:** Predicted selectivity from simulations based on the CavAb [18] ion channel X-ray structures using cations with radii of the fully hydrated (${\hat{\alpha }}_{\text{sim,hyd}}$) and fully dehydrated (${\hat{\alpha }}_{\text{sim,dehyd}}$) species.

Ions	*α*_sim,hyd_	*α*_sim,dehyd_	Expt
Na^+^,Ca^2+^	600	100	380 [18]
Mn^2+^,Ca^2+^	0.4	0.8	Generally Mn^2+^ > Ca^2+^
Ba^2+^,Ca^2+^	200	3	Generally Ca^2+^ > Ba^2+^

To test if using hydrated ions is required we compared these selectivities with ones from simulations with cations at their fully dehydrated radii (Table 3: *α*_sim,dehyd_). All selectivities are reduced when using the smaller dehydrated radii cations, with the key selectivity between Ca^2+^ and Na^+^ falling below the experimental value. This can be explained from knowing that selectivity decreases as volume increases [45] and we assume the volume defined by the atomistic representation of the pore wall matches the size of fully hydrated cations rather than the size of dehydrated cations. Furthermore, in our model fully hydrated Ca^2+^ ions can not enter the Na^+^ selective channels whereas fully dehydrated Ca^2+^ ions could enter the Na^+^ selective channels which would lead to (results not shown) these channels being predicted as Ca^2+^ selective. This shows that including atomistic detail in the SF must be matched with realistically sized hydrated ions.

The second test of hydration and the representation of the pore wall is to see the results for the KcsA channel for ions with hydration energy close to that of K^+^, the selectivities (${\text{Tl}}^{+}>K^{+}>{\text{Rb}}^{+}>{\text{NH}}_{4}^{+}$) [48, 49] follow, in inverse order, the cation hydration energies (${\text{Tl}}^{+}<{\text{K}}^{+}<{\text{NH}}_{4}^{+}<{\text{Rb}}^{+}$) [50, 51] rather than the cation size (${\text{K}}^{+}<{\text{Tl}}^{+}≊{\text{Rb}}^{+}≊{\text{NH}}_{4}^{+}$) [52, 53]. From Table 4 we see that *α*_hyd_ is the dominant term in determining selectivity from simulation, resulting in selectivities that are *not* in agreement with experiment.

**Table 4:** Comparison of simulated and experimental [47] selectivity values for the KcsA [6] ion channel with cations of similar full hydration energies [51].

Ions	*α*_hyd_	${\hat{\alpha }}_{\text{sim}}$	*α*_sim_	Expt (P_K^+^_P_*X*^+^_)
$\text{NH}{}_{4}^{+}{\text{K}}^{+}$	0.02	4.0	0.08	5
Rb^+^,K^+^	0.0003	1.4	0.0004	1.2
Tl^+^,K^+^	7.5	1.4	10	0.3

**Fig 8:**
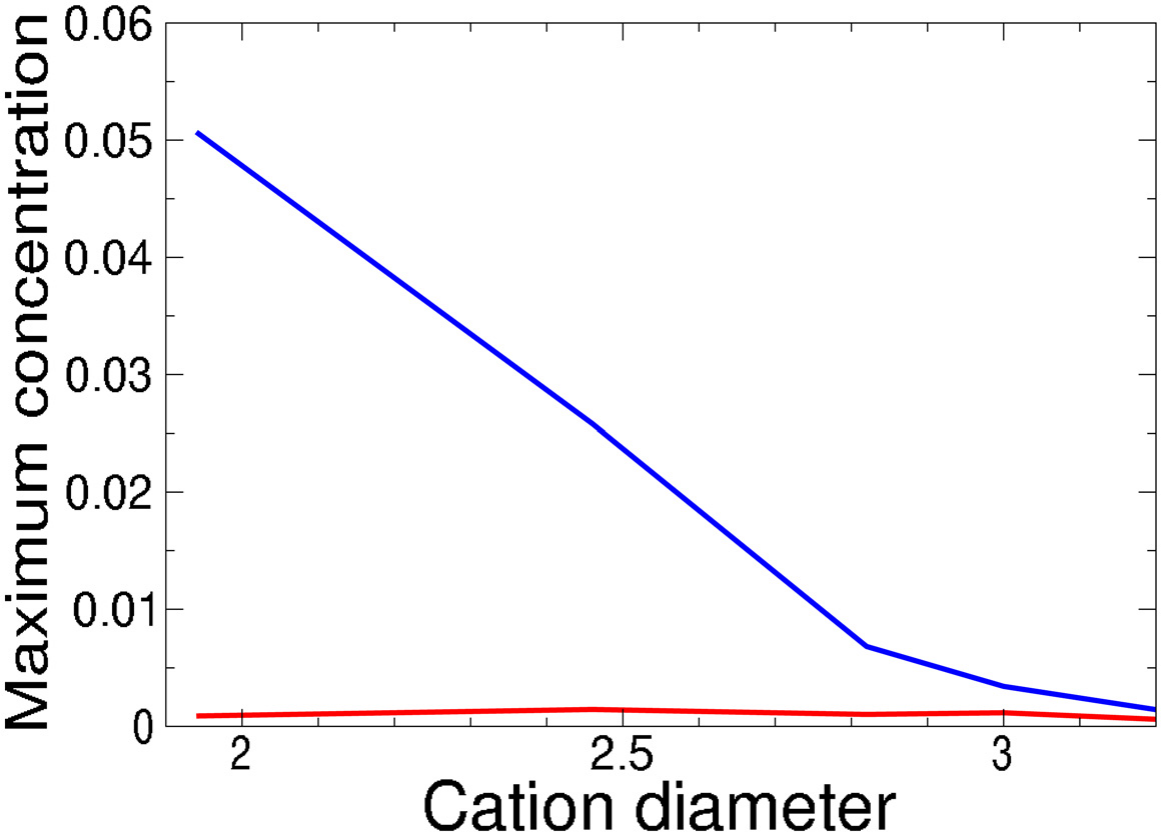
Variation of [A ⋅ *X*]_min_ (at maximum [A]_bulk_) in KcsA K^+^ channel simulation with “polarization” charge (red = 0:0, blue = −0.1) as a function of cation diameter.

The KcsA channel has no charged residues in the SF and so the dominant electrostatic interaction seen in the Na^+^ and Ca^2+^ channels is not present. The main attractive interaction is the interaction of the cations with the carbonyl oxygens, the geometry allowing up to eight atoms in the SF to coordinate with a single cation. This is represented in the CSC Hamiltonian only as an electrostatic interaction between the cation and the partial charge on the carbonyl oxygens. The ability of this attractive electrostatic interaction to mimic the attractive interaction inferred from the experimental results was evaluated by comparing simulations with different charges on the oxygens atoms in pore wall of the SF. When going from -0.1 to 0.0 (Table 5, Fig 8) the major impact appears to be to modulate the concentration of ions in the pore, with smaller ions benefitting the most. However, selectivity is derived from the relative concentrations and we find no correlation between the charge and selectivity in our small sample, contrary to earlier suggestions [54]. This points to less significance of this charge for determining selectivity. Therefore the principle of parsimony suggests that these charges should be left out in future simulations. The most telling problem is Rb^+^ and Tl^+^, which are modeled identically in our simulations except for their hydration energies. These energies are in inverse order to the experimental selectivities, putting selectivities for these cations in the K^+^ channel beyond the scope of our Hamiltonian. All this suggests that the non-electrostatic interaction between the cation and carbonyl oxygens that line the SF are important for selectivity in the K^+^ channel. Handling such interactions requires a more detailed treatment [55–57].

**Table 5:** Comparison of change in maximum concentration and selectivity from simulations based on the KcsA X-ray structure [6] with and without “polarization” charges on the SF oxygen atoms.

Ion (*A*)	diameter /Å	[*A* ⋅ *X*]_(max,0.0)_	[*A* ⋅ *X*]_(max,−0.1)_	$\frac{\alpha_{\left(\text{sim,K:}A,-0.1\right)}}{\alpha_{\left(\text{sim,K:}A,0.0\right)}}$
Na^+^	1.94	0.001	0.05	0.8
K^+^	2.82	0.001	0.006	
Rb^+^	3.00	0.001	0.003	1.1
$\text{NH}{}_{4}^{+}$	3.20	0.001	0.001	0.8

The simulation results for the KcsA model shows the limit of our Hamiltonian where volume, hydration and electrostatic interactions are assumed to be the most dominant terms and therefore neglecting attractive non-electrostatic interactions. Our results show this assumption is reasonable for ion channels that contain charged residues in the SF, such as Na^+^ and Ca^2+^ channels, but fails for the K^+^ channel which is without these charged residues. The results for the Na^+^ and Ca^2+^ channels demonstrate that the dehydration model presented here, while simple, appears to capture the main features of dehydration important for selectivity within the model.

### Limitations

We have presented here a model that is specific to the needs of simulating the confined geometry of ion channel SFs within the CSC method. As in any model, ours has advantages and disadvantages. Some of these have been described in previous sections. In addition, a limiting factor we have found are the limited number of published experimental values for partial hydration free energies over the full range of cations used in biological studies (for example the partial dehydration energies are not available for Cs^+^). Terms omitted from the current model include non-electrostatic attractive forces, a large number of details of the protein structure outside the SF, the bulk dynamics of the protein and the membrane voltage [58]. Explicit water molecules are not included, thus the complex networks of water molecules seen in atomistic simulations [59–66] are not reproduced. The model of partially hydrated cations as static spheres of discrete sizes and without dipoles can obviously be augmented with further terms, for example dynamic rearrangement of the waters of hydration affecting both the cluster shape and electrostatic field could be added.

### Concluding remarks

The augmented Hamiltonian leads to satisfactory results not only for Na^+^ and Ca^2+^ channels, but also for K^+^ because the ion-water interactions are critical to ion channel simulations. Close inspection of *α*_*hyd*_ (Table 1) reveals that selectivity based purely on hydration could vary over at least eight orders of magnitude. This implies that validation of the description of cation-water interactions must be a primary requirement for all models.

The CSC Hamiltonian used in this paper complements other studies by highlighting and isolating the most important contributions to selectivity. We confirm quantitatively and through robust simulation the critical role hydration, volume exclusion and charge sites play in selectivity, as first proposed by Eisenman [8, 9] in the 1960s. We have shown how partial dehydration plays a critical role in Na^+^ selectivity for bacterial Na^+^ ion channels, as only suggested by earlier studies. However, the physics included in the CSC model is insufficient to describe selectivity in K^+^ channels, possibly due to the absence of attractive non-electrostatic interactions in the current Hamiltonian.

We show that for selectivity, cation hydration states must to be taken into account for any Hamiltonian when using atomistic detail in the SF. We have also shown that the CSC Hamiltonian is limited to pores where the electrostatic interactions dominate. Within this limitation, the elements of the Hamiltonian isolated here gives us a robust basis to consider designing cation selective nanopores. Thus, not only does this model predict selectivity for a subset of biological cation channels, the model can also apply to the significant simplification required in forward-engineering similar functionality.

## Supporting Information

S1 File Supporting Information. Section A, Derivation of Eq 3 from single-barrier model and GHK equation. Section B, The mathematics of aggregating the results from sets of simulations into the single equilibrium constant. Section C, The mathematics of converting the X-ray B-factors into the harmonic potential terms used in the simulations. Section D, The pore geometry and atom positions used in the simulations. Section E, Tables of complete raw population data for Figs 5–7.
(PDF)

## Abbreviations

CSC: charge space competition

## References

[1] Bernstein J. Untersuchungen zur Thermodynamik der bioelektrischen Ströme. Pflüger’s Archiv für die gesamte Physiologie des Menschen und der Tiere. 1902;92(10–12):521–562. Available from: 10.1007/BF01790181.

[2] Cole K, Curtis H. Electric impedance of the squid giant axon during activity. J Gen Physiol. 1939 5;22(5):649–670. 10.1085/jgp.22.5.649 19873125 PMC2142006

[3] Hodgkin AL, Katz B. The effect of sodium ions on the electrical activity of giant axon of the squid. J Physiol. 1949 3;108(1):37–77. Available from: http://europepmc.org/abstract/MED/18128147. 10.1113/jphysiol.1949.sp004310 18128147 PMC1392331

[4] Hodgkin AL, Huxley AF. A quantitative description of membrane current and its application to conduction and excitation in nerve. J Physiol. 1952 8;117(4):500–544. 10.1113/jphysiol.1952.sp004764 12991237 PMC1392413

[5] Heginbotham L, Lu Z, Abramson T, MacKinnon R. Mutations in the K^+^ channel signature sequence. Biophys J. 1994;66(4):1061–1067. Available from: http://www.sciencedirect.com/science/article/pii/S0006349594808872. 10.1016/S0006-3495(94)80887-2 8038378 PMC1275813

[6] Doyle DA, Cabral JM, Pfuetzner RA, Kuo A, Gulbis JM, Cohen SL, et al The structure of the potassium channel: molecular basis of K^+^ conduction and selectivity. Science. 1998 4;280(5360):69–77. 10.1126/science.280.5360.69 9525859

[7] Yu FH, Catterall WA. The VGL-chanome: a protein superfamily specialized for electrical signaling and ionic homeostasis. Sci STKE. 2004 10;2004(253):re15 Available from: 10.1126/stke.2532004re15. 15467096

[8] Eisenman G. On the elementary atomic origin of equilibrium ionic specificity In: Symposium on membrane transport and metabolism. Academic Press, New York; 1961 p. 163–179.

[9] Eisenman G. Cation Selective Glass Electrodes and their Mode of Operation. Biophys J. 1962;2(2, Part 2):259–323. Available from: http://www.sciencedirect.com/science/article/pii/S0006349562869598. 10.1016/S0006-3495(62)86959-8 13889686 PMC1366487

[10] Ulmschneider MB, Bagnéris C, McCusker EC, DeCaen PG, Delling M, Clapham DE, et al Molecular dynamics of ion transport through the open conformation of a bacterial voltage-gated sodium channel. P Natl Acad Sci USA. 2013;110(16):6364–6369. Available from: http://www.pnas.org/content/110/16/6364.abstract. 10.1073/pnas.1214667110 PMC3631666 23542377

[11] Hille B. Ionic channels of excitable membranes. 2nd ed Sunderland, Massachusetts: Sinauer Associates, Inc; 1992.

[12] Collins KD. Sticky ions in biological systems. P Natl Acad Sci USA. 1995;92(12):5553–5557. 10.1073/pnas.92.12.5553 PMC41734 7539920

[13] Payandeh J, Scheuer T, Zheng N, Catterall WA. The crystal structure of a voltage-gated sodium channel. Nature. 2011;475:353–358. 10.1038/nature10238 21743477 PMC3266868

[14] McCusker EC, Bagnéris C, Naylor CE, Cole AR, D’Avanzo N, Nichols CG, et al Structure of a bacterial voltage-gated sodium channel pore reveals mechanisms of opening and closing. Nat Commun. 2012 10;3:1102–. Available from: 10.1038/ncomms2077. 10.1038/ncomms2077 23033078 PMC3493636

[15] Stock L, Delemotte L, Carnevale V, Treptow W, Klein ML. Conduction in a Biological Sodium Selective Channel. J Phys Chem B. 2013;p. 3782–3789. 10.1021/jp401403b 23452067

[16] Finol-Urdaneta RK, Wang Y, Al-Sabi A, Zhao C, Noskov SY, French RJ. Sodium channel selectivity and conduction: Prokaryotes have devised their own molecular strategy. J Gen Phys. 2014;143(2):157–171. Available from: http://jgp.rupress.org/content/143/2/157.abstract. 10.1085/jgp.201311037 PMC4001777 24420772

[17] Chakrabarti N, Ing C, Payandeh J, Zheng N, Catterall WA, Pomès R. Catalysis of Na+ permeation in the bacterial sodium channel NaVAb. P Natl Acad Sci USA. 2013;110(28):11331–11336. 10.1073/pnas.1309452110 PMC3710854 23803856

[18] Tang L, Gamal El-Din TM, Payandeh J, Martinez GQ, Heard TM, Scheuer T, et al Structural basis for Ca^2+^ selectivity of a voltage-gated calcium channel. Nature. 2014 1;505(7481):56–61. Available from: 10.1038/nature12775. 10.1038/nature12775 24270805 PMC3877713

[19] Kamerlin SCL, Vicatos S, Dryga A, Warshel A. Coarse-Grained (Multiscale) Simulations in Studies of Biophysical and Chemical Systems. Annu Rev Phys Chem. 2011;62(1):41–64. PMID: 21034218. Available from: 10.1146/annurev-physchem-032210-103335. 10.1146/annurev-physchem-032210-103335 21034218

[20] Roux B. Ion Binding Sites and Their Representations by Reduced Models. J Phys Chem B. 2012;116(23):6966–6979. Available from: http://pubs.acs.org/doi/abs/10.1021/jp3007365. 10.1021/jp3007365 22494321 PMC3718881

[21] Chen D, Eisenberg R. Exchange diffusion, single filing, and gating in macroscopic channels of one conformation. J Gen Physiol. 1992;100:9a.

[22] Chen DP, Eisenberg RS. Flux, coupling, and selectivity in ionic channels of one conformation. Biophys J. 1993;65(2):727–746. 10.1016/S0006-3495(93)81099-3 7693003 PMC1225775

[23] Eisenberg RS. From structure to permeation in open ionic channels. Biophys J. 1993;2(2):A22.

[24] Barcilon V, Chen D, Eisenberg RS, Ratner MA. Barrier crossing with concentration boundary conditions in biological channels and chemical reactions. J Chem Phys. 1993;98(2):1193–1212. Available from: http://link.aip.org/link/?JCP/98/1193/1. 10.1063/1.464342

[25] Chen D, Eisenberg R. Charges, currents, and potentials in ionic channels of one conformation. Biophys J. 1993 5;64(5):1405–1421. Available from: 10.1016/S0006-3495(93)81507-8. 10.1016/S0006-3495(93)81507-8 7686784 PMC1262466

[26] Nonner W, Eisenberg B. Ion Permeation and Glutamate Residues Linked by Poisson-Nernst-Planck Theory in L-type Calcium Channels. Biophys J. 1998;75:1287–1305. 10.1016/S0006-3495(98)74048-2 9726931 PMC1299804

[27] Nonner W, Catacuzzeno L, Eisenberg B. Binding and selectivity in L-type calcium channels: A mean spherical approximation. Biophys J. 2000 10;79(4):1976–1992. Available from: 10.1016/S0006-3495(00)76446-0. 10.1016/S0006-3495(00)76446-0 11023902 PMC1301088

[28] Schuss Z, Nadler B, Eisenberg RS. Derivation of Poisson and Nernst-Planck equations in a bath and channel from a molecular model. Physical Review E. 2001;64(3):036116 Available from: http://pre.aps.org/abstract/PRE/v64/i3/e036116. 10.1103/PhysRevE.64.036116 11580403

[29] Boda D, Busath D, Eisenberg B, Henderson D, Nonner W. Monte Carlo simulations of ion selectivity in a biological N^+^ channel: charge-space competition. Phys Chem Chem Physics. 2002;4:5154–5160. 10.1039/b203686j

[30] Boda D, Gillespie D, Nonner W, Henderson D, Eisenberg B. Computing induced charges in inhomogeneous dielectric media: application in a Monte Carlo simulation of complex ionic systems. Phys Rev E Stat Nonlin Soft Matter Phys. 2004 4;69(4 Pt 2):046702 10.1103/PhysRevE.69.046702 15169126

[31] Nadler B, Schuss Z, Singer A, Eisenberg RS. Ionic diffusion through confined geometries: from Langevin equations to partial differential equations. J Phys:Condens Matter. 2004;16(22):S2153 Available from: http://iopscience.iop.org/0953-8984/16/22/015.

[32] Nonner W, Peyser A, Gillespie D, Eisenberg B. Relating microscopic charge movement to macroscopic currents: the Ramo-Shockley theorem applied to ion channels. Biophys J. 2004 12;87(6):3716–3722. Available from: http://www.hubmed.org/display.cgi?uids = 15465857. 10.1529/biophysj.104.047548 15465857 PMC1304885

[33] Eisenberg RS. Atomic biology, electrostatics and ionic channels In: Elber R, editor. New Developments and Theoretical Studies of Proteins. vol. 7 Philadelphia: World Scientific Publishing; 2013 p. 269–357.

[34] Peyser A, Nonner W. Voltage sensing in ion channels: Mesoscale simulations of biological devices. Phys Rev E. 2012;86(1):011910 Available from: http://link.aps.org/doi/10.1103/PhysRevE.86.011910. 10.1103/PhysRevE.86.011910 23005455

[35] Nonner W, Chen DP, Eisenberg B. Anomalous Mole Fraction Effect, Electrostatics, and Binding in Ionic Channels. Biophys J. 1998;74(5):2327–2334. Available from: http://www.sciencedirect.com/science/article/pii/S0006349598779421. 10.1016/S0006-3495(98)77942-1 9591660 PMC1299576

[36] Gillespie D, Boda D. The Anomalous Mole Fraction Effect in Calcium Channels: A Measure of Preferential Selectivity. Biophys J. 2008;95(6):2658–2672. Available from: http://www.sciencedirect.com/science/article/pii/S000634950878411X. 10.1529/biophysj.107.127977 18515379 PMC2527270

[37] Bennett CH. Efficient estimation of free energy differences from Monte Carlo data. J Comput Phys. 1976;22(2):245–268. Available from: http://www.sciencedirect.com/science/article/pii/0021999176900784. 10.1016/0021-9991(76)90078-4

[38] Finnerty JJ, Eisenberg R, Carloni P. Localizing the Charged Side Chains of Ion Channels within the Crowded Charge Models. J Chem Theory Comput. 2013;9(1):766–773. Available from: http://pubs.acs.org/doi/abs/10.1021/ct300768j. 10.1021/ct300768j 26589069

[39] Allen R, Hansen JP, Melchionna S. Electrostatic potential inside ionic solutions confined by dielectrics: a variational approach. Phys Chem Chem Physics. 2001;3:4177–4186. 10.1039/b105176h

[40] Ohtaki H, Radnai T. Structure and Dynamics of Hydrated Ions. Chem Rev. 1993;93:1157–1204. 10.1021/cr00019a014

[41] Giri J, Fonseca JE, Boda D, Henderson D, Eisenberg B. Self-organized models of selectivity in calcium channels. Phys Biol. 2011;8(2):026004 Available from: http://stacks.iop.org/1478-3975/8/i=2/a=026004. 10.1088/1478-3975/8/2/026004 21263167

[42] Kebarle P. Ion Thermochemistry and Solvation from Gas Phase Ion Equilibria. Ann Rev Phys Chem. 1977;28:445–476. 10.1146/annurev.pc.28.100177.002305

[43] Marcus Y. Ionic radii in aqueous solutions. Chem Rev. 1988;88(8):1475–1498. Available from: http://pubs.acs.org/doi/abs/10.1021/cr00090a003. 10.1021/cr00090a003

[44] Boda D, Valiskó M, Eisenberg B, Nonner W, Henderson D, Gillespie D. The effect of protein dielectric coefficient on the ionic selectivity of a calcium channel. J Chem Phys. 2006 7;125(3):34901 Available from: 10.1063/1.2212423. 10.1063/1.2212423 16863379

[45] Boda D, Valiskó M, Eisenberg B, Nonner W, Henderson D, Gillespie D. Combined effect of pore radius and protein dielectric coefficient on the selectivity of a calcium channel. Phys Rev Lett. 2007 4;98(16):168102 Available from: http://www.hubmed.org/display.cgi?uids = 17501467. 10.1103/PhysRevLett.98.168102 17501467

[46] Bucher D, Raugei S, Guidoni L, Peraro MD, Rothlisberger U, Carloni P, et al Polarization effects and charge transfer in the KcsA potassium channel. Biophys Chem. 2006;124(3):292–301. Available from: http://www.sciencedirect.com/science/article/pii/S0301462206001232. 10.1016/j.bpc.2006.04.008 16737771

[47] LeMasurier M, Heginbotham L, Miller C. KcsA: It’s a Potassium Channels. The Journal of General Physiology. 2001;118(3):303–314. Available from: http://jgp.rupress.org/content/118/3/303.abstract. 10.1085/jgp.118.3.303 11524460 PMC2229506

[48] Bezanilla F, Armstrong CM. Negative Conductance Caused by Entry of Sodium and Cesium Ions into the Potassium Channels of Squid Axons. J Gen Physiol. 1972;60(5):588–608. Available from: http://jgp.rupress.org/content/60/5/588.abstract. 10.1085/jgp.60.5.588 4644327 PMC2226091

[49] Blatz AL, Magleby KL. Ion conductance and selectivity of single calcium-activated potassium channels in cultured rat muscle [Meeting Abstract]. Biophys J. 1984;45(2):A306. 10.1085/jgp.84.1.1 PMC2228730 6086805

[50] Rosseinsky DR. Electrode Potentials And Hydration Energies. Theories And Correlations [Review]. Chem Rev. 1965;65(4):467–490. 10.1021/cr60236a004

[51] Marcus Y. Thermodynamics of solvation of ions. Part 5.-Gibbs free energy of hydration at 298.15 K. J Chem Soc, Faraday Trans. 1991;87:2995–2999. Available from: 10.1039/FT9918702995. 10.1039/FT9918702995

[52] Shannon RD. Revised effective ionic radii and systematic studies of interatomic distances in halides and chalcogenides. Acta Crystallogr A. 1976 9;32(5):751–767. Available from: 10.1107/S0567739476001551. 10.1107/S0567739476001551

[53] Khan AA, Baur WH. Salt hydrates. VII. The crystal structures of sodium ammonium orthochromate dihydrate and magnesium diammonium bis(hydrogen orthophosphate) tetrahydrate and a discussion of the ammonium ion. Acta Crystallogr B. 1972 3;28(3):683–693. Available from: 10.1107/S0567740872003024. 10.1107/S0567740872003024

[54] Åqvist J, Alvarez O, Eisenman G. Ion-selective properties of a small ionophore in methanol studied by free energy perturbation simulations. J Phys Chem. 1992;96(24):10019–10025. 10.1021/j100203a079

[55] Varma S, Rogers DM, Pratt LR, Rempe SB. Design principles for K^+^ selectivity in membrane transport. J Gen Physiol. 2011;137(6):479–488. Available from: http://jgp.rupress.org/content/137/6/479.short. 10.1085/jgp.201010579 21624944 PMC3105521

[56] Rossi M, Tkatchenko A, Rempe SB, Varma S. Role of methyl-induced polarization in ion binding. P Natl Acad Sci USA. 2013;110(32):12978–12983. 10.1073/pnas.1302757110 PMC3740884 23878238

[57] Köpfer DA, Song C, Gruene T, Sheldrick GM, Zachariae U, de Groot BL. Ion permeation in K^+^ channels occurs by direct Coulomb knock-on. Science. 2014;346(6207):352–355. Available from: http://www.sciencemag.org/content/346/6207/352.abstract. 10.1126/science.1254840 25324389

[58] Peyser A, Nonner W. The sliding-helix voltage sensor: mesoscale views of a robust structure-function relationship. Eur Biophys J. 2012 9;41(9):705–721. Available from: http://www.hubmed.org/display.cgi?uids = 22907204. 10.1007/s00249-012-0847-z 22907204 PMC3448954

[59] Hofer TS, Randolf BR, Rode BM. Structure-breaking effects of solvated Rb(I) in dilute aqueous solution—An ab initio QM/MM MD approach. J Comput Chem. 2005;26(9):949–956. Available from: 10.1002/jcc.20232. 10.1002/jcc.20232 15858825

[60] Bostick DL, Brooks CL. Selectivity in K+ channels is due to topological control of the permeant ion’s coordinated state. P Natl Acad Sci USA. 2007;104(22):9260–9265. 10.1073/pnas.0700554104 PMC1890482 17519335

[61] Vchirawongkwin V, Hofer TS, Randolf BR, Rode BM. Tl(I)-the strongest structure-breaking metal ion in water? A quantum mechanical/molecular mechanical simulation study. J Comput Chem. 2007;28(6):1006–1016. Available from: 10.1002/jcc.20583. 10.1002/jcc.20583 17269122

[62] Azam SS, Hofer TS, Randolf BR, Rode BM. Hydration of Sodium(I) and Potassium(I) Revisited: A Comparative QM/MM and QMCF MD Simulation Study of Weakly Hydrated Ions. J Phys Chem A. 2009;113(9):1827–1834. PMID: 19203258. Available from: http://pubs.acs.org/doi/abs/10.1021/jp8093462. 10.1021/jp8093462 19203258

[63] Marcus Y. Effects of ions on the structure of water. Pure Appl Chem. 2010;82:1889–1899. 10.1351/PAC-CON-09-07-02

[64] Mähler J, Persson I. A study if the hydration of the alkali metals ions in aqueous solution. Inorg Chem. 2012;51:425–438. 10.1021/ic2018693 22168370 PMC3250073

[65] Ding Y, Hassanali AA, Parrinello M. Anomalous water diffusion in salt solutions. P Natl Acad Sci USA. 2014;111(9):3310–3315. Available from: http://www.pnas.org/content/111/9/3310.abstract. 10.1073/pnas.1400675111 PMC3948301 24522111

[66] Li H, Francisco JS, Zeng XC. Unraveling the mechanism of selective ion transport in hydrophobic subnanometer channels. P Natl Acad Sci USA. 2015;Available from: http://www.pnas.org/content/early/2015/08/13/1513718112.abstract. 10.1073/pnas.1513718112 PMC4568210 26283377

