# Supplementary material for: Cation Selectivity in Biological Cation Channels Using Experimental Structural Information and Statistical Mechanical Simulation
Source: PLoS One. 2015 Oct 13;10(10):e0138679. doi: 10.1371/journal.pone.0138679 (PMC4603898; doi:10.1371/journal.pone.0138679)
Supplement: S1 File — Section A, Derivation of Eq 3 from single-barrier model and GHK equation. Section B, The mathematics of aggregating the results from sets of simulations into the single equilibrium constant. Section C, The mathematics of converting the X-ray B-factors into the harmonic potential terms used in the simulations. Section D, The pore geometry and atom positions used in the simulations. Section E, Tables of complete raw population data for Figs 5–7. (PDF) [file pone.0138679.s001.pdf]

Supporting Information: Cation selectivity in biological cation channels using experimental structural information and statistical mechanical simulation.

Justin J. Finnerty

*Computational Biophysics, German Research School for Simulation Sciences, 52425 Jülich, Germany**

Alexander Peyser

Computational Biophysics, German Research School for Simulation Sciences, 52425 Jülich, Germany and Simulation Lab Neuroscience – Bernstein Facility for Simulation and Database Technology, Institute for Advanced Simulation, Jülich Aachen Research Alliance, Forschungszentrum Jülich, 52425 Jülich, Germany*

Paolo Carloni

Computational Biophysics, German Research School for Simulation Sciences, 52425 Jülich, Germany and Computational Biomedicine, Institute for Neuroscience and Medicine (INM-9) and Institute for Advanced Simulation (IAS-5), Forschungszentrum Jülich, 52425 Jülich, Germany*

* j.finnerty@grs-sim.de

The supporting information contains: (Section A) Derivation of selectivity from single-barrier model and GHK equation. (Section B) The mathematics of aggregating the results from sets of simulations into the single equilibrium constant. (Section C) The mathematics of converting the X-ray B-factors into the harmonic potential terms used in the simulations. (Section D) The pore geometry and atom positions used in the simulations. (Section E) Tables of raw population data for the key simulations.

CONTENTS

A. Relationship between channel occupancy and selectivity using a single-barrier model. 3

B. Selectivity by fitting data to dose response curves 4

C. Converting from B-Factor to CSC localization parameters 5

D. CSC model simulation conditions 7
- D.1. Parameters for KcsA K^+ channel 8
- D.2. Parameters for NavMs Bacterial Na^+ channel 10
- D.3. Parameters for NavAb Bacterial Na^+ channel 12
- D.4. Parameters for CavAb mutated bacterial Na^+ channel converted to a Ca^{2+} selective channel 14

References 16

E. Density profiles 16
- E.1. KCSA channel: Potassium and Sodium 16
- E.2. KCSA channel: Potassium and Ammonium 38
- E.3. KCSA channel: Potassium and Rubidium 59
- E.4. NavMs channel: Sodium and Potassium 80
- E.5. NavAb channel: Sodium and Potassium 125
- E.6. CavAb channel: Calcium and Sodium 170
- E.7. CavAb channel: Calcium and Barium 259
- E.8. CavAb channel: Calcium and Manganese 348

A. RELATIONSHIP BETWEEN CHANNEL OCCUPANCY AND SELECTIVITY USING A SINGLE-BARRIER MODEL.

This derivation follows from Hille's single-ion pores[1]. We describe below how his non-equilibrium equation can apply to our simulation under equilibrium conditions and zero voltage.

The selectivity of a cation channel for two cations A and B is usually as the ratio of the permeability rates of the two cations, $\alpha = \mathrm{P}_A/\mathrm{P}_B$. Experimental cation channel selectivity may be determined from electrophysiology voltage/current measurements of the ion channels using the Goldman-Hodgkins-Katz[2, 3] equation (a) for the reversal voltage (V_{rev}); the membrane voltage at which the net current reverses direction. For a two cation system under steady-state conditions, this is written as

$$V_{rev} = \frac{RT}{F} \ln \left(\frac{[B]_{out} + \alpha [A]_{out}}{[B]_{in} + \alpha [A]_{in}} \right) \tag{a}$$

where R is the gas constant, F is the Faraday constant, T the temperature, and the concentrations $[A]$ or $[B]$ are measured inside the cell (in) or in the extracellular (out) region.

Now we consider Hille's steady-state single-ion pore model[1] equation for permeation applied to our situation of the passage of A and B across the cell membrane at equilibrium:

$$A_{in} \underset{-k_A}{\overset{k_A}{\rightleftharpoons}} A_{out} \text{ and } B_{in} \underset{-k_B}{\overset{k_B}{\rightleftharpoons}} B_{out} \tag{b}$$

where k_A and k_B are the rate constants for the cations passing through the channel selectivity filter (SF). The SFs of cation channels are so narrow that we can consider ion passage as a pseudo one-dimensional system. We can therefore relate these rate constants directly to permeability because permeability ($\mathrm{P}_A = D_A/l_X$) is the diffusion constant (D_A) over the length of the channel (l_X), the diffusion constant is the passage length squared times by the passage rate ($D_A = l_X^2 \cdot k_\mathrm{A}$) and l_X, being the distance between bulk solutions, is independent of A or B giving $\alpha = {}^{k_\mathrm{A}}\!/\!_{k_\mathrm{B}}$.

This gives the rate of passage as the rate of formation of a rate-determining transition state complex $(A.X)^\ddagger$ or $(B.X)^\ddagger$ at the highest free energy point in the SF multiplied by the probability of moving to either side of the SF. We can then reformulate the Rxn. b as a reaction for some transition complex which does not reference which side of the SF a cation is approaching or leaving:

$$A + (B.X)^\ddagger \underset{k'_B}{\overset{k'_A}{\rightleftharpoons}} B + (A.X)^\ddagger \tag{c}$$

$[A.X^{\ddagger}]$ and $[B.X^{\ddagger}]$ can not be measured directly in experiment, but they can be estimated from simulation from the density minimum in the SF. Under our equilibrium conditions the probability of leaving the transition state in the direction of one side of the SF or the other should be equal so $2k_A = k'_A$. We can then write an $K_{B,A}$ for Rxn. c as

$$K_{A,B} = \frac{[A.X^{\ddagger}][B]}{[A][B.X^{\ddagger}]} = \frac{2k_{\text{A}}}{2k_{\text{B}}} = \alpha_{\text{sim}} \tag{d}$$

Hence α_{sim} can be determined from the ratio of $[A]$ and $[B]$ in the bulk solution and the ratio of the transition state complexes $[A.X^{\ddagger}]$ and $[B.X^{\ddagger}]$ in the SF.

$[A.X^{\ddagger}]$ and $[B.X^{\ddagger}]$ are estimated using the simulated density profile within the SF. We find the region containing the minima and maxima in the density profile for a particular ion channel are independent of the cation. After the simulation the density profiles are plotted and a region containing the minimum in density for all the ions is selected. The average density of cations in this region is then used as $[A.X^{\ddagger}]$ or $[B.X^{\ddagger}]$. Where multiple hydration states are present in the simulation the density profiles of the hydration states of an ion are first summed before determining $[A.X^{\ddagger}]$ or $[B.X^{\ddagger}]$.

On a side note about selectivity between monovalent and divalent cations. The equations used to derive selectivity from experiment are complicated by the need to account for the difference in charge between the two cations and the measured current. This complication is not present here as our values are derived from density which is independent of the charge carried.

B. SELECTIVITY BY FITTING DATA TO DOSE RESPONSE CURVES

Plotting the concentration profile along the channel axis for all the cations used with a particular ion channel allowed us to determine a region within the selectivity filter where the concentration/density of all the cations was lowest. The concentration/density of cations within this region ($[M \cdot X^{\ddagger}]$) was plotted against the ratio of the cation concentrations in the bulk solution ($[B]/[A]$), resulting in a sigmoid dose-response plot. We determine K_9 from Eqn. 11 in the main text when hydration is important and $K_{A,B}$ from Eqn. 7 in the main text without hydration from:

$$K = \frac{[A.X^{\ddagger}]}{[B.X^{\ddagger}]}\frac{[B]}{[A]}$$

From this formula it can be seen that the position where the plots of $[A.X^{\ddagger}]$ and $[B.X^{\ddagger}]$ cross (where the cations have equal concentration in the ion channel ${}^{[A.X^{\ddagger}]}/_{[B.X^{\ddagger}]} = 1$) has $K = {}^{[B]}/_{[A]}$. Thus K can be calculated from the entire data set by fitting the concentrations in the SF to the standard *dose-response curve* (both expressed in terms of $\mathrm{R} = [B]/[A]$):

$$[B \cdot X^{\ddagger}]_i = \frac{[B \cdot X^{\ddagger}]_{\text{max}}}{1 + 10^{(\log(\mathrm{R}_{50,\mathrm{B}}) - \log(\mathrm{R}_i))}} \text{ and } [A \cdot X^{\ddagger}]_i = \frac{[A \cdot X^{\ddagger}]_{\text{max}}}{1 + 10^{\left(\log(\mathrm{R}_i) - \log(\mathrm{R}_{50,\mathrm{A}})\right)}}$$

where $[B \cdot X^{\ddagger}]_{\text{max}}$ and $[A \cdot X^{\ddagger}]_{\text{max}}$ are the fitted maximum occupancies and $\mathrm{R}_{50,B}$ and $\mathrm{R}_{50,A}$ are the fitted ratios at fifty percent of maximum occupancy as ratios of $[B]/[A]$. From above we know that when $[B \cdot X^{\ddagger}] = [A \cdot X^{\ddagger}]$: $\mathrm{R}_i = K$ for both cations, giving:

$$\frac{[B \cdot X^{\ddagger}]_{\text{max}}}{1 + 10^{\left(\log(\mathrm{R}_{50,B}) - \log(K)\right)}} = \frac{[A \cdot X^{\ddagger}]_{\text{max}}}{1 + 10^{\left(\log(K) - \log(\mathrm{R}_{50,A})\right)}}$$

This gives the following quadratic after rearrangement:

$$0 = K^2 \cdot \frac{[B \cdot X^{\ddagger}]_{\text{max}}}{\mathrm{R}_{50,A}} + K \cdot \left([B \cdot X^{\ddagger}]_{\text{max}} - [A \cdot X^{\ddagger}]_{\text{max}}\right) - [A \cdot X^{\ddagger}]_{\text{max}} \cdot \mathrm{R}_{50,B}$$

C. CONVERTING FROM B-FACTOR TO CSC LOCALIZATION PARAMETERS

The localisation of atoms in the CSC model uses a spherical harmonic potential that mimics the normal distribution around the localization center. We match the normal distribution from which the X-ray B-factor is calculated based on the following:

$$\mathcal{U}(r) = \frac{k_f}{R_{f,i}^2} |r - r_{0,i}|^2 \tag{e}$$

where $\mathcal{U}(r)$ is the localization potential, and k_f is a global constant that scales for energy. Unique for each localized particle i, $R_{f,i}$ is both a maximum displacement cut off and spring constant component and $r_{0,i}$ is the center point of the localization potential.

A normal distribution has the following probability distribution:

$$P(x) = \frac{1}{\sigma\sqrt{2\pi}} \exp \frac{-|x - \mu|^2}{2\sigma^2}.$$

where μ is the mean, x is the independent coordinate, and σ is the standard deviation.

This translates to a potential in x in terms of the Boltzmann distribution:

$$\begin{aligned}\mathcal{U}(x) &= -kT\log\left(P(x)\right)\\ &= -kT\log\left(\frac{1}{\sigma\sqrt{2\pi}}\right) + kT\frac{|x-\mu|^2}{2\sigma^2}\\ &= kT\log\left(\sigma\sqrt{2\pi}\right) + kT\frac{|x-\mu|^2}{2\sigma^2}\end{aligned}$$

where kT is the Boltzmann constant.

Translating to our physical coordinate space, the mean μ is the per particle localization center $r_{0,i}$, and the free coordinate x is the test position r, which gives:

$$\mathcal{U}(r) = kT\log\left(\sigma\sqrt{2\pi}\right) + kT\frac{|r-r_{0,i}|^2}{2\sigma^2} + C.$$

As our boundary condition, we define $\mathcal{U}(r_{0,i}) = 0kT$ and thus $C = -kT\log\left(\sigma\sqrt{2\pi}\right)$, giving:

$$\mathcal{U}(r) = kT\frac{|r-r_{0,i}|^2}{2\sigma^2}. \tag{f}$$

By combining the CSC harmonic potential (Eq. e) with this Gaussian potential (Eq. f), we can solve for σ in terms of our harmonic constants:

$$\sigma = \frac{R_{f,i}}{\sqrt{2k_f/kT}}.$$

Since 3σ covers over $> 99\%$ of a normal population, we use this to define our cutoff $R_{f,i} = 3\sigma$, and so:

$$\begin{aligned}R_{f,i} &= \frac{3R_{f,i}}{\sqrt{2k_f/kT}},\\ k_f &= 4.5\,kT.\end{aligned}$$

The measured Debye-Waller factor[4] is reported in X-ray crystallography reports as the B-factor, which is defined as:

$$\mathcal{B} = 8\pi^2\langle(x-\mu)^2\rangle.$$

Since $\sigma^2 \approx \langle (x-\mu)^2 \rangle$ for a large number of samples, we get:

$$\sigma = \sqrt{(\mathcal{B}/8\pi^2)}.$$

Substituting the $R_{f,i} = 3\sigma$ that we defined as our cutoff, we can derive a relation between $R_{f,i}$ and $\mathcal{B}$ when setting $k_f = 4.5\,kT$:

$$\begin{aligned} R_{f,i} &= 3\sqrt{\mathcal{B}/8\pi^2} \\ &= \frac{3}{2\pi}\sqrt{\mathcal{B}/2} \end{aligned}$$

D. CSC MODEL SIMULATION CONDITIONS

The details of performing a CSC simulation have been reported previously[5, 6]. In brief, a series of simulations for each channel model was performed with various concentrations of the cations (as chloride salts) to be compared in the bulk regions of the simulation. The concentration ratio ($[B]/[A]$) in the bulk of the simulation for each cation pair was varied at least from 0.01 to 100.0 while the ionic strength was maintained at 220 mM. Approximately 10^9 simulation trials were performed at each concentration ratio. The concentration of $[B.X^\ddagger]$ and $[A.X^\ddagger]$ estimated from each simulation was plotted against $[B]/[A]$ to give a dose response curve for the series. The equilibrium constant is then determined (Section B) from this dose response curve.

The coordinates of the charged side groups within the ion channel selectivity filter used in the CSC model where taken from the X-ray structures. These coordinates were translated into the CSC model coordinate system which has the z-axis along the channel axis and the origin set to the center of the selectivity filter. We report here the PDB label, the atoms that where used, the translation matrix and the coordinate of each side-chain atom used within the CSC model. The localisation parameters are $k_f = 4.5\,kT$ and $R_{f,i}$ values calculated from the X-ray B-factors as described above (Section C).

The simulation is performed in a cylindrical box. The box is bisected into two bulk solution compartments by a virtual membrane. The model of the ion channel and selectivity filter is formed by rotating a rounded rectangle around an axis of rotation to form a toroid (See Fig. a). This toroid is embedded within the virtual membrane and represents the only passage between the two bulk regions. The virtual membrane is coincident with the left and right surfaces of the toroid. The

parameters of the rounded rectangle used to generate the toroid shape are listed for each channel.

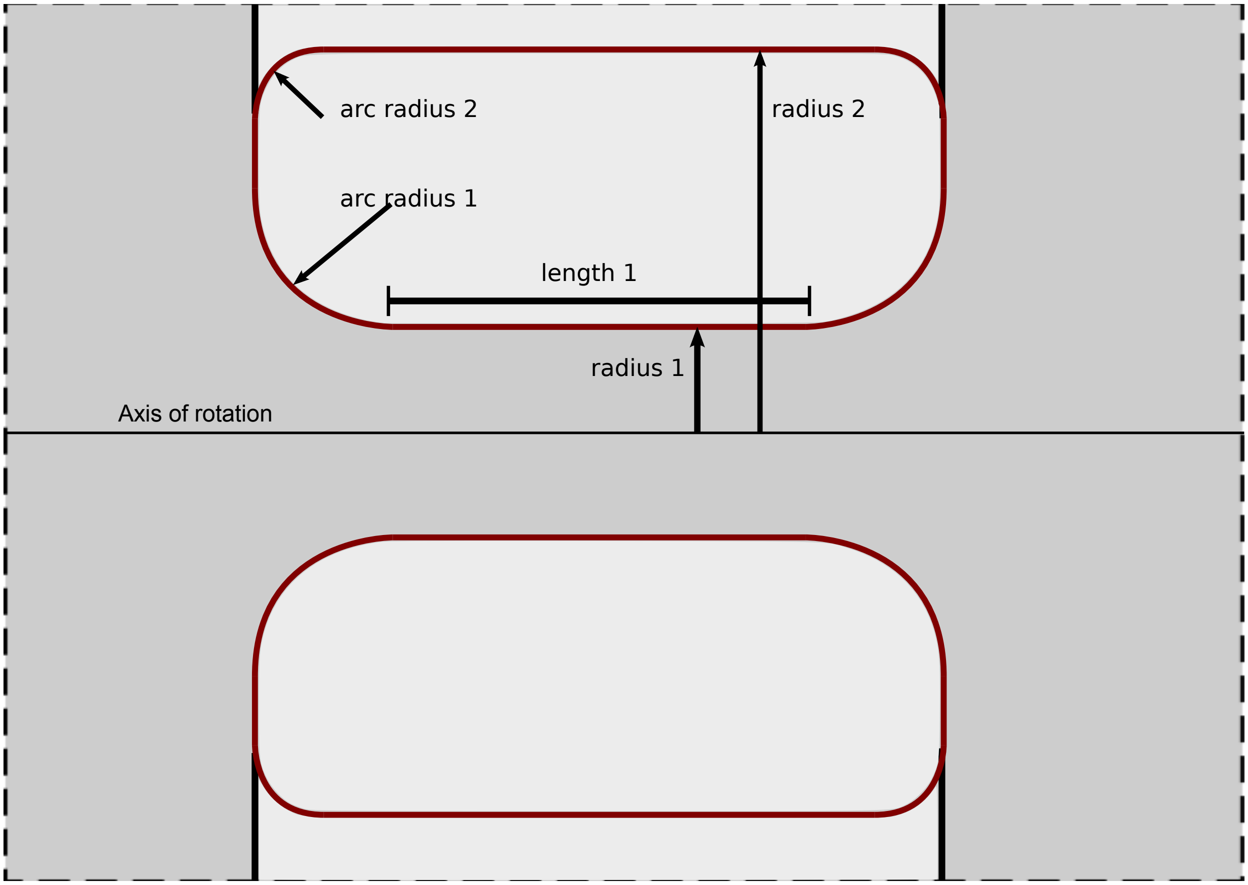

Figure a. Detail schematic (not to scale) of the pore through the membrane in the simulation. The model selectivity filter/ion channel is a toroid made by the shape outlined by the red line (light grey interior) rotated around the axis of symmetry of the simulation. The virtual membrane (black line with light grey interior) is coincident with the left and right surfaces of the toroid. Boundary conditions are satisfied for the system by the dielectric surface being closed to form a toriod with `radius 2` set to `radius 1` + ≈20Å.

D.1. Parameters for KcsA K^+ channel

The PDB used has accession number 1BL8. The parameters for defining the CSC model selectivity filter toroid are: `length 1` = 16.4Å, `radius 1` = 5.2Å, `radius 2` = 30Å, `arc radius 1` = 10Å and `arc radius 2` = 5Å (#JJF-GRS1275). Region found after the simulations for sampling $[M.X^{\ddagger}] = -6.5 : -3.5$.

The conversion matrix from the PDB X-ray coordinates to CSC model coordinates is:

$$\begin{pmatrix} -0.93 & 0.00 & 0.38 & 59.4666 \\ 0.00 & 1.00 & 0.00 & -26.6058 \\ -0.38 & 0.00 & -0.93 & 39.0358 \end{pmatrix}$$

Table of the PDB atoms and coordinates translated into the CSC coordinate system. The CSC maximum displacement radius, $R_{f,i}$, is listed along side the B-factor and corresponds to a CSC localization constant of $k_f = 4.5$.

ATOM	RES	CHAIN	SEQ	x_0	y_0	z_0	B-Factor	$R_{f,i}$
O	GLY	A	77	2.15	-0.37	2.93	148.34	4.11
O	GLY	B	77	-0.38	-2.15	2.91	130.62	3.86
O	GLY	C	77	-2.23	0.39	2.94	161.6	4.29
O	GLY	D	77	0.32	2.16	2.90	139.35	3.99
O	THR	A	75	2.74	-0.63	-4.12	71.55	2.86
O	THR	B	75	-0.55	-2.69	-4.15	73.54	2.90
O	THR	C	75	-2.62	0.60	-4.13	65.21	2.73
O	THR	D	75	0.63	2.66	-4.16	67.16	2.77
OG1	THR	A	75	2.83	1.34	-5.15	78.57	2.99
OG1	THR	B	75	1.41	-2.80	-5.16	51.43	2.42
OG1	THR	C	75	-2.70	-1.36	-5.15	51.91	2.43
OG1	THR	D	75	-1.32	2.75	-5.20	71.11	2.85
O	TYR	A	78	2.39	-1.68	6.07	62.83	2.68
O	TYR	B	78	-1.74	-2.40	6.03	53.28	2.46
O	TYR	C	78	-2.56	1.74	6.06	80	3.02
O	TYR	D	78	1.62	2.44	6.05	47.6	2.33
O	VAL	A	76	1.90	-1.42	0.34	146.85	4.09
O	VAL	B	76	-1.39	-1.88	0.31	146.59	4.09
O	VAL	C	76	-1.90	1.42	0.35	149.65	4.13
O	VAL	D	76	1.39	1.88	0.32	163.1	4.31

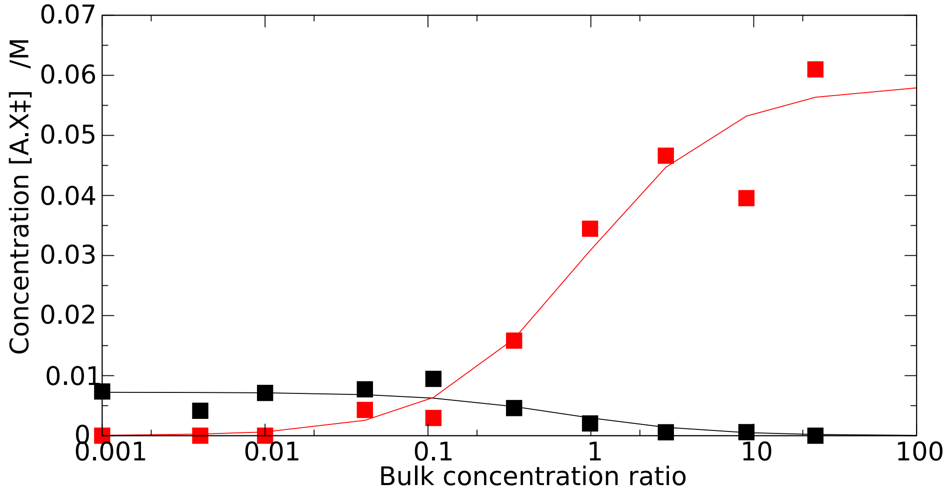

Figure b. Simulated (squares) and fitted (lines) values for $[A.X^{\ddagger}]$ within the KcsA channel. Black is [K^{+}.$X^{\ddagger}$] and red is [Na^{+}.$X^{\ddagger}$] ($K = 0.1$). Concentration ratio is bulk [Na^{+}]/[K^{+}].

D.2. Parameters for NavMs Bacterial Na $^{+}$ channel

The PDB used has accession number 4F4L. The parameters for defining the CSC model selectivity filter toroid are: `length 1` = 10.6Å, `radius 1` = 8.8Å, `radius 2` = 30Å, `arc radius 1` = 10Å and `arc radius 2` = 5Å. Region chosen for sampling $[M.X^{\ddagger}] = 1.5 : 4.5$ along channel axis.

The conversion matrix from the PDB X-ray coordinates to CSC model coordinates is:

$$\begin{pmatrix} 0.89 & 0.00 & -0.46 & -0.0814 \\ 0.00 & 1.00 & 0.00 & -15.6633 \\ 0.46 & 0.00 & 0.89 & 13.6886 \end{pmatrix}$$

Table of the PDB atoms and coordinates translated into the CSC coordinate system. The CSC maximum displacement radius, $R_{f,i}$, is listed along side the B-factor and corresponds to a CSC localization constant of $k_f = 4.5$. (‡ the charge position used for the carboxylic group was the average position of the two oxygen atoms.)

ATOM	RES	CHAIN	x_0	y_0	z_0	B-Factor	$R_{f,i}$
CA	GLU	A	-0.97	-4.73	-0.86	116.11	3.64
CA	GLU	B	1.04	4.73	-0.88	96.78	3.32
CA	GLU	C	-4.58	0.96	-0.87	102.65	3.42

ATOM	RES	CHAIN	x_0	y_0	z_0	B-Factor	$R_{f,i}$
CA	GLU	D	4.63	-0.89	-0.85	106.25	3.48
OE1	GLU	A	-2.40	-3.24	2.50	132.42	3.89
OE1	GLU	B	2.54	3.17	2.05	93.2	3.26
OE1	GLU	C	-3.89	2.77	2.50	30.36	1.86
OE1	GLU	D	3.40	-2.61	2.60	115.47	3.63
CA	GLY	A	2.47	-7.05	6.18	98.74	3.35
CA	GLY	B	-2.55	7.04	6.22	100.21	3.38
CA	GLY	C	-6.99	-2.46	6.16	103.49	3.43
CA	GLY	D	6.93	2.51	6.34	95.26	3.30
CB	LEU	A	1.79	-4.98	-4.84	113.45	3.60
CB	LEU	B	-1.91	5.02	-4.79	95.7	3.30
CB	LEU	C	-4.95	-1.97	-4.75	103.33	3.43
CB	LEU	D	4.80	1.84	-4.81	100.65	3.39
O	LEU	A	1.52	-4.49	-2.08	120.04	3.70
O	LEU	B	-1.49	4.34	-2.12	100.63	3.39
O	LEU	C	-4.31	-1.52	-2.10	109.28	3.53
O	LEU	D	4.35	1.58	-2.07	108.56	3.52
CB	MET	A	5.27	-3.60	5.65	93.28	3.26
CB	MET	B	-5.30	3.56	5.58	99.49	3.37
CB	MET	C	-3.52	-5.30	5.69	107.03	3.49
CB	MET	D	3.45	5.28	5.80	100.89	3.39
O	MET	A	-0.12	-8.12	-7.29	123.81	3.76
O	MET	B	0.22	8.01	-7.33	111.69	3.57
O	MET	C	-7.91	0.11	-7.30	115.75	3.63
O	MET	D	7.91	-0.06	-7.25	116.58	3.65
CA	SER	A	1.22	-5.02	2.27	111.21	3.56
CA	SER	B	-1.19	4.98	2.27	94.21	3.28
CA	SER	C	-4.90	-1.18	2.31	101.61	3.40
CA	SER	D	4.87	1.24	2.33	105.78	3.47

ATOM	RES	CHAIN	x_0	y_0	z_0	B-Factor	$R_{f,i}$
OG	SER	A	-0.07	-3.53	3.72	129.03	3.84
OG	SER	B	0.57	3.75	3.34	110.48	3.55
OG	SER	C	-3.50	0.06	3.89	118.87	3.68
OG	SER	D	3.53	-0.36	3.53	125.03	3.78
O	THR	A	-1.39	-4.77	-5.19	118.93	3.68
O	THR	B	1.36	4.67	-5.23	104.25	3.45
O	THR	C	-4.53	1.42	-5.15	108.18	3.51
O	THR	D	4.56	-1.20	-5.17	109.16	3.53

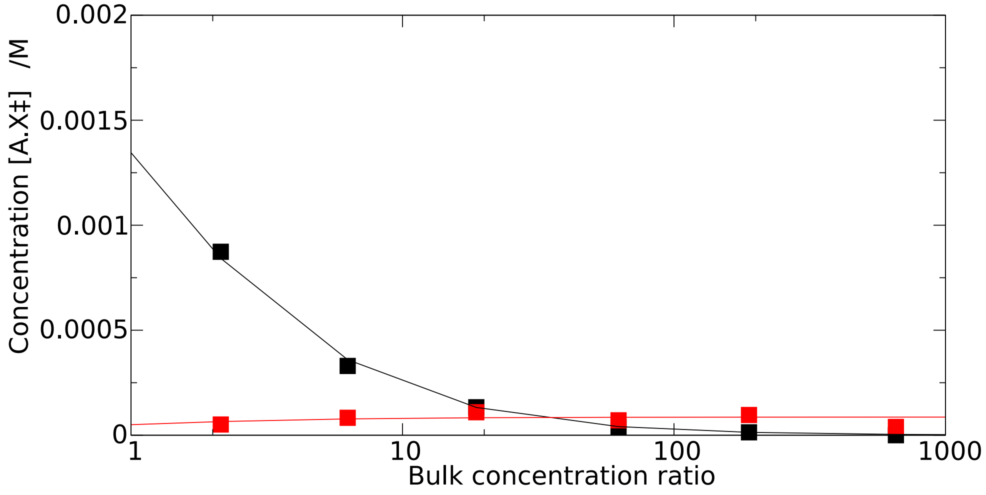

Figure c. Simulated (squares) and fitted (lines) values for $[A.X^{\ddagger}]$ within the NavAb channel. Red is [K^{+}.$X^{\ddagger}$] and black is [Na^{+}.$X^{\ddagger}$]. Concentration ratio is bulk [K^{+}]/[Na^{+}]. ($\alpha = 30$)

D.3. Parameters for NavAb Bacterial Na $^{+}$ channel

The PDB used has accession number 1BL8. The parameters for defining the CSC model selectivity filter toroid are: `length 1` = 10.6Å, `radius 1` = 8.8Å, `radius 2` = 30Å, `arc radius 1` = 10Å and `arc radius 2` = 5Å. Region chosen for sampling $[M.X^{\ddagger}] = -4.6 : -3.8$ along channel axis.

The conversion matrix from the PDB X-ray coordinates to CSC model coordinates is:

$$\begin{pmatrix} 1.00 & 0.00 & 0.00 & 62.7970 \\ 0.00 & 1.00 & 0.00 & 62.7415 \\ 0.00 & 0.00 & 1.00 & 82.7900 \end{pmatrix}$$

Table of the PDB atoms and coordinates translated into the CSC coordinate system. The CSC maximum displacement radius, $R_{f,i}$, is listed along side the B-factor and corresponds to a CSC localization constant of $k_f = 4.5$. (‡ the charge position used for the carboxylic group was the average position of the two oxygen atoms.)

ATOM	RES	CHAIN	SEQ	x_0	y_0	z_0	B-Factor	$R_{f,i}$
O	LEU	A	76	1.911	-4.3515	2.889	49.63	2.38
O	LEU	B	76	4.517	1.8605	2.782	51.48	2.42
O	LEU	C	76	-1.911	4.3515	2.889	49.63	2.38
O	LEU	D	76	-4.517	-1.8605	2.782	51.48	2.42
O	GLU	A	77	4.838	-4.2005	-0.119	51.48	2.42
O	GLU	B	77	4.237	4.6885	-0.142	49.58	2.38
O	GLU	C	77	-4.838	4.2005	-0.119	51.48	2.42
O	GLU	D	77	-4.237	-4.6885	-0.142	49.58	2.38
OE‡	GLU	A	77	5.5585	-0.8635	-1.3775	55.60	2.52
OE‡	GLU	B	77	0.787	5.511	-1.3895	58.04	2.57
OE‡	GLU	C	77	-5.5585	0.8635	-1.3775	55.60	2.52
OE‡	GLU	D	77	-0.787	-5.511	-1.3895	58.04	2.57
OG	SER	A	78	3.159	-2.3765	-2.889	51.59	2.42
OG	SER	B	78	2.351	3.0535	-2.852	52.8	2.45
OG	SER	C	78	-3.159	2.3765	-2.889	51.59	2.42
OG	SER	D	78	-2.351	-3.0535	-2.852	52.8	2.45

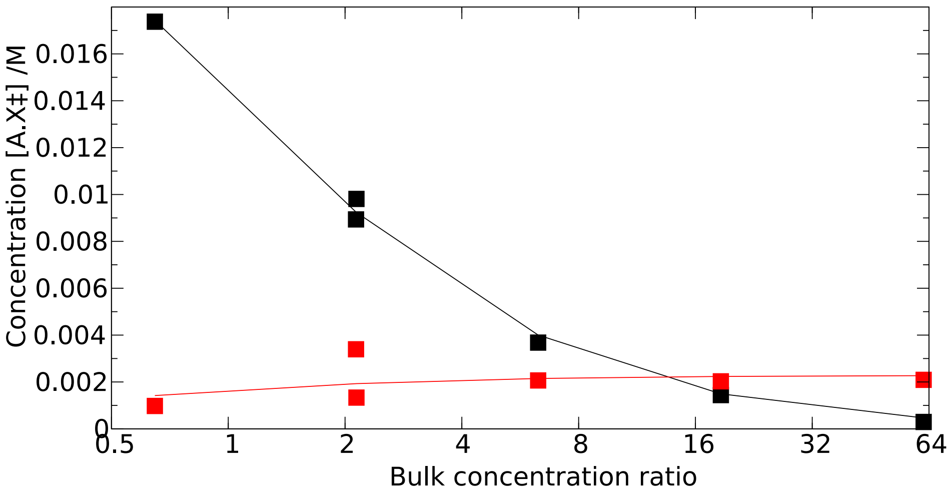

Figure d. Simulated (squares) and fitted (lines) values for $[A.X^{\ddagger}]$ within the NavAb channel. Red is [K$^+$.$X^{\ddagger}$] and black is [Na$^+$.$X^{\ddagger}$]. Concentration ratio is bulk [K$^+$]/[Na$^+$]. ($\alpha = 12$)

D.4. Parameters for CavAb mutated bacterial Na $^+$ channel converted to a Ca^{2+} selective channel

The PDB used has accession number 4MVQ. The parameters for defining the CSC model selectivity filter toroid are: `length 1` = 10.6Å, `radius 1` = 12.4Å, `radius 2` = 30Å, `arc radius 1` = 10Å and `arc radius 2` = 5Å. Region chosen for sampling $[M.X^{\ddagger}] = -5.0 : -3.0$ along channel axis.

The conversion matrix from the X-ray coordinates The conversion matrix from the PDB X-ray coordinates to CSC model coordinates is:

$$\begin{pmatrix} 1.00 & 0.00 & 0.00 & -177.8730 \\ 0.00 & 1.00 & 0.00 & 44.5615 \\ 0.00 & 0.00 & 1.00 & -10.8936 \end{pmatrix}$$

Table of the PDB atoms and coordinates translated into the CSC coordinate system. The CSC maximum displacement radius, $R_{f,i}$, is listed along side B-factor and corresponds to a CSC localization constant of $k_f = 4.5$.

ATOM	RES	CHAIN	SEQ	x_0	y_0	z_0	B-Factor	$R_{f,i}$
O	LEU	A	76	-1.3739	-4.1655	3.5736	40.37	2.15
O	LEU	B	76	-4.0970	1.4635	3.6828	37.48	2.07

ATOM	RES	CHAIN	SEQ	x_0	y_0	z_0	B-Factor	$R_{f,i}$
O	LEU	C	76	4.1763	-1.3855	3.6701	44.86	2.26
O	LEU	D	76	1.5048	4.1715	3.6318	40.57	2.15
OD1	ASP	A	77	2.7130	-3.4695	0.2465	54.59	2.49
OD1	ASP	B	77	-3.4025	-2.7425	0.3307	54.35	2.49
OD1	ASP	C	77	3.3595	2.6835	0.3951	51.63	2.43
OD1	ASP	D	77	-2.6419	3.4565	0.2801	59.02	2.59
CG	ASP	A	78	-0.8708	-4.1815	-3.6118	41.94	2.19
CG	ASP	B	78	-4.1846	0.8025	-3.6777	45.21	2.27
CG	ASP	C	78	4.1599	-0.8995	-3.5701	46.44	2.30
CG	ASP	D	78	0.7276	4.1835	-3.6828	43.28	2.22
CG	ASP	A	81	-6.6610	-2.5585	-4.6080	33.44	1.95
CG	ASP	B	81	-2.5854	6.6485	-4.5677	36.11	2.03
CG	ASP	C	81	2.6698	-6.5715	-4.5713	33.34	1.95
CG	ASP	D	81	6.5060	2.5645	-4.5776	35.9	2.02

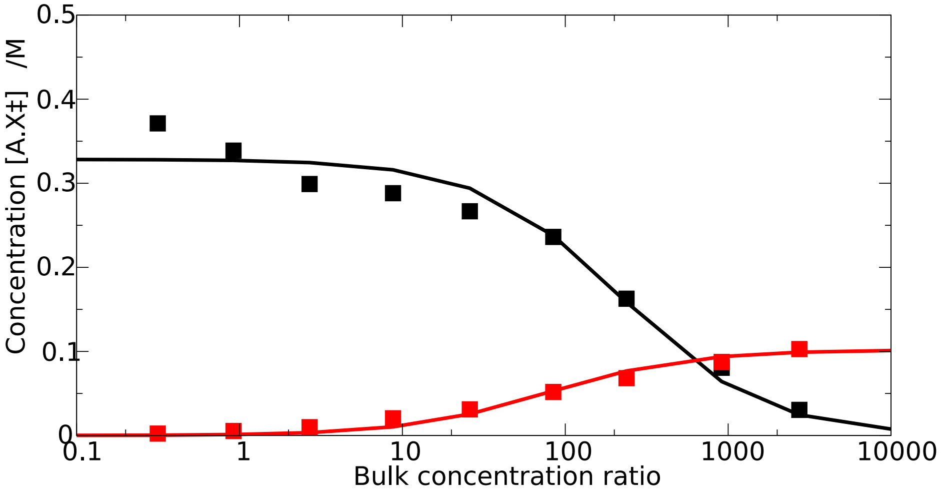

Figure e. Simulated (squares) and fitted (lines) values for $[A.X^{\ddagger}]$ within the CavAb channel. Black is [Ca^{2+}.$X^{\ddagger}$] and red is [Na^{+}.$X^{\ddagger}$] ($\alpha = 590$). Concentration ratio is bulk [Na^{+}]/[Ca^{2}+].

[1] B. Hille, *Ionic channels of excitable membranes*, 2nd ed. (Sinauer Associates, Inc, Sunderland, Massachusetts, 1992).

[2] D. E. Goldman, The Journal of General Physiology **27**, 37 (1943), http://jgp.rupress.org/content/27/1/37.full.pdf+html.

[3] A. L. Hodgkin and B. Katz, J Physiol **108**, 37 (1949).

[4] P. Debye, Annalen der Physik **348**, 49 (1913).

[5] D. Boda, M. Valiskó, B. Eisenberg, W. Nonner, D. Henderson, and D. Gillespie, J Chem Phys **125**, 34901 (2006).

[6] J. J. Finnerty, R. Eisenberg, and P. Carloni, Journal of Chemical Theory and Computation **9**, 766 (2013), http://pubs.acs.org/doi/pdf/10.1021/ct300768j.

E. DENSITY PROFILES

The density profiles are computed as population histograms in intervals along the z-axis of the simulation. The density is then estimated by dividing the population in a histogram bin by the calculated volume of the bin. Note that the program used has an error in the volume calculations for the histogram bins around the transition from bulk to the beginning of the membrane region and at the ends of the cell. This has no impact on our results as the density at these points is never used when calculating results.

The volume of the histogram bin is determined to be the volume accessible by the centre point of a sphere with the radius of the corresponding cation. For the cylindrical portions of the simulation cell this is $V = \pi(R - r)^2 l$ where R is the radius of the cylinder, r is the radius of the cation and l is the width of the histogram bin.

Different simulations have different midpoint and volume ranges. The program calculates the total volume from the target number of solute particles and target concentration from user input and then calculates the length and outer radius of the simulation cell. Differences in the axial length of the model channel will also result in changes, particularly to the midpoint ranges.

E.1. KCSA channel: Potassium and Sodium

Table of Na^+ population data for simulation with K^+ and Na^+ across bulk density ratios (#JJF-GRS1459)

	Population in histogram bin at Na^+/K^+ ratio:							
Midpoint	8.6	2.8	0.97	0.34	0.11	0.041	0.009	Volume
-58.625	4.547	3.908	2.656	1.395	0.581	0.250	0.031	1260.533
-58.375	98.364	83.223	57.648	29.266	11.293	4.402	1.042	1260.533
-58.125	98.507	82.227	56.887	29.541	11.376	4.477	0.978	1260.533
-57.875	99.056	83.032	57.315	29.100	11.112	4.443	1.019	1260.533
-57.625	98.859	83.791	56.682	30.026	11.081	4.327	0.965	1260.533
-57.375	98.689	82.697	57.081	29.950	11.036	4.346	1.037	1260.533
-57.125	98.996	82.735	57.675	29.070	10.923	4.391	0.996	1260.533
-56.875	99.214	82.353	57.330	29.173	11.013	4.492	1.074	1260.533
-56.625	99.219	83.238	57.418	29.403	11.262	4.426	0.969	1260.533
-56.375	100.243	82.921	57.302	29.341	11.164	4.408	1.042	1260.533
-56.125	100.411	83.391	57.489	29.727	11.836	4.437	1.062	1260.533
-55.875	100.429	82.227	57.658	29.740	11.149	4.411	1.011	1260.533
-55.625	98.609	82.901	57.209	30.083	11.723	4.350	1.034	1260.533
-55.375	99.871	83.105	57.532	29.391	11.428	4.544	1.046	1260.533
-55.125	99.754	83.257	58.000	29.388	11.278	4.422	1.044	1260.533
-54.875	99.901	84.999	57.922	29.732	11.006	4.510	1.120	1260.533
-54.625	99.934	83.446	56.748	28.999	11.429	4.477	1.054	1260.533
-54.375	99.871	83.677	57.463	30.013	11.708	4.471	1.056	1260.533
-54.125	99.629	84.109	57.317	29.639	11.217	4.484	1.008	1260.533
-53.875	100.121	84.524	57.456	29.847	11.723	4.531	1.046	1260.533
-53.625	99.593	83.938	57.456	29.692	11.542	4.400	1.013	1260.533
-53.375	100.119	83.582	57.776	30.246	11.308	4.525	1.014	1260.533
-53.125	100.086	85.841	57.214	29.471	11.829	4.383	1.016	1260.533
-52.875	100.349	84.783	58.311	30.204	11.715	4.540	0.951	1260.533
-52.625	100.819	84.122	57.491	29.933	11.300	4.508	1.047	1260.533
-52.375	101.038	84.813	57.663	29.885	11.474	4.320	1.034	1260.533
-52.125	101.407	84.889	57.458	29.790	11.444	4.630	1.140	1260.533
-51.875	100.446	85.545	58.111	29.697	11.874	4.501	1.031	1260.533
-51.625	100.068	84.836	58.674	30.299	11.617	4.531	1.034	1260.533
-51.375	101.886	84.761	59.084	29.775	11.708	4.505	1.049	1260.533
-51.125	102.804	85.813	58.585	30.146	11.534	4.755	1.057	1260.533
-50.875	102.136	86.168	58.958	30.006	12.115	4.744	1.023	1260.533
-50.625	101.437	85.328	59.099	30.369	12.047	4.665	1.052	1260.533
-50.375	102.306	85.937	58.611	30.427	11.466	4.697	1.085	1260.533
-50.125	101.656	86.497	59.150	31.147	11.587	4.691	1.077	1260.533
-49.875	102.640	87.215	59.707	30.181	11.980	4.650	1.059	1260.533
-49.625	103.243	86.725	59.807	30.741	12.395	4.656	1.105	1260.533
-49.375	103.898	87.334	59.843	31.052	11.934	4.531	1.087	1260.533
-49.125	103.404	87.947	59.326	31.006	12.055	4.559	1.188	1260.533
-48.875	104.373	87.575	60.014	31.290	12.153	4.841	1.066	1260.533
-48.625	104.470	87.678	60.438	30.853	12.115	4.626	1.097	1260.533
-48.375	105.135	88.816	60.369	31.170	11.889	4.594	1.061	1260.533
-48.125	105.923	89.455	59.961	31.338	11.670	4.841	1.046	1260.533

Midpoint	Ratio: 8.6	2.8	0.97	0.34	0.11	0.041	0.009	Volume
-47.875	106.178	89.585	60.841	31.200	11.746	4.576	1.081	1260.533
-47.625	105.283	89.159	60.947	31.310	11.670	4.753	1.110	1260.533
-47.375	106.068	88.925	60.828	31.370	12.749	4.970	1.095	1260.533
-47.125	105.418	89.227	60.763	31.817	12.123	4.622	1.094	1260.533
-46.875	107.337	89.342	61.380	31.215	12.470	4.884	1.153	1260.533
-46.625	107.068	89.927	61.819	31.804	12.342	4.805	1.122	1260.533
-46.375	106.963	90.528	61.489	31.942	11.678	4.878	1.087	1260.533
-46.125	106.590	89.980	61.310	31.812	11.904	4.761	1.099	1260.533
-45.875	107.500	90.872	61.882	31.874	12.402	4.916	1.163	1260.533
-45.625	106.645	90.422	61.531	31.694	12.176	4.837	1.115	1260.533
-45.375	106.917	90.430	62.043	31.857	12.425	4.770	1.170	1260.533
-45.125	108.193	91.782	62.277	32.053	12.470	4.817	1.072	1260.533
-44.875	107.835	91.513	62.123	32.377	12.153	4.869	1.052	1260.533
-44.625	107.865	90.644	62.320	32.146	12.712	4.839	1.056	1260.533
-44.375	108.101	90.415	63.144	32.080	12.274	4.923	1.147	1260.533
-44.125	107.787	90.802	62.441	32.231	12.395	4.921	1.076	1260.533
-43.875	107.008	90.945	62.665	31.990	12.214	4.764	1.117	1260.533
-43.625	108.697	91.211	62.718	32.226	12.342	4.891	1.132	1260.533
-43.375	108.315	91.362	62.381	32.597	12.440	4.839	1.122	1260.533
-43.125	109.185	91.420	63.218	32.264	12.236	5.063	1.090	1260.533
-42.875	108.905	91.569	61.849	32.216	12.281	4.712	1.163	1260.533
-42.625	108.088	92.084	62.600	32.351	12.176	5.031	1.084	1260.533
-42.375	109.004	91.805	62.684	32.223	12.357	4.906	1.067	1260.533
-42.125	109.615	91.015	62.688	32.627	12.863	4.828	1.069	1260.533
-41.875	108.670	91.885	62.189	32.441	12.508	4.901	1.147	1260.533
-41.625	108.432	92.358	63.038	32.459	12.674	5.000	1.145	1260.533
-41.375	109.340	92.722	63.278	32.492	12.810	4.946	1.082	1260.533
-41.125	109.840	92.496	63.520	32.547	12.478	5.065	1.152	1260.533
-40.875	108.782	92.284	62.925	32.499	12.636	5.050	1.077	1260.533
-40.625	110.135	92.207	63.107	33.074	12.583	4.930	1.085	1260.533
-40.375	109.417	92.667	63.192	32.248	12.417	4.876	1.079	1260.533
-40.125	110.008	92.915	63.104	32.289	12.493	4.919	1.167	1260.533
-39.875	109.302	92.101	62.671	32.738	12.327	4.807	1.165	1260.533
-39.625	109.260	91.912	63.376	32.785	12.840	4.919	1.102	1260.533
-39.375	108.298	91.279	63.525	32.768	12.931	4.955	1.104	1260.533
-39.125	109.647	92.608	63.702	32.863	12.848	5.003	1.147	1260.533
-38.875	108.882	91.719	62.098	32.898	11.972	4.863	1.122	1260.533
-38.625	109.432	92.368	63.286	33.044	12.364	4.942	1.175	1260.533
-38.375	109.864	92.882	62.570	32.708	13.036	4.933	1.193	1260.533
-38.125	110.016	92.448	64.105	32.524	11.957	4.817	1.145	1260.533
-37.875	109.285	92.299	63.510	33.312	12.863	4.861	1.130	1260.533
-37.625	110.029	93.117	62.958	33.036	12.402	4.893	1.129	1260.533
-37.375	108.754	92.398	63.533	32.549	12.855	4.919	1.104	1260.533
-37.125	110.197	93.023	62.859	32.617	12.749	4.873	1.041	1260.533
-36.875	108.448	92.486	63.268	33.121	12.999	4.886	1.105	1260.533

Midpoint	Ratio: 8.6	2.8	0.97	0.34	0.11	0.041	0.009	Volume
-36.625	110.435	92.262	63.510	32.888	12.372	5.009	1.115	1260.533
-36.375	109.190	93.334	63.124	32.793	12.251	4.876	1.104	1260.533
-36.125	111.355	92.807	63.994	32.991	12.999	4.910	1.140	1260.533
-35.875	109.887	92.498	63.555	32.866	12.704	4.981	1.172	1260.533
-35.625	109.108	92.880	63.356	32.763	12.629	4.878	1.145	1260.533
-35.375	109.512	92.099	63.522	32.692	12.236	4.929	1.137	1260.533
-35.125	108.955	92.697	63.533	33.084	12.417	5.013	1.072	1260.533
-34.875	109.930	92.830	63.121	33.264	12.931	5.044	1.162	1260.533
-34.625	109.353	92.325	63.658	32.823	12.395	4.972	1.102	1260.533
-34.375	109.560	93.119	63.316	32.815	12.470	4.895	1.134	1260.533
-34.125	110.254	92.541	63.316	32.823	12.349	4.988	1.150	1260.533
-33.875	110.451	91.935	63.391	32.993	13.029	5.071	1.031	1260.533
-33.625	109.605	91.687	63.220	32.692	13.104	4.940	1.081	1260.533
-33.375	109.970	91.767	64.074	32.657	12.727	4.848	1.119	1260.533
-33.125	108.662	92.493	62.698	32.509	12.078	4.890	1.132	1260.533
-32.875	109.712	92.380	62.872	32.524	12.251	4.886	1.084	1260.533
-32.625	110.017	92.795	63.099	32.785	12.463	4.841	1.157	1260.533
-32.375	108.585	92.074	62.476	32.798	12.380	4.863	1.071	1260.533
-32.125	108.953	92.111	63.373	31.912	13.202	4.906	1.104	1260.533
-31.875	108.955	91.654	63.739	32.499	12.583	4.979	1.062	1260.533
-31.625	108.995	91.556	63.056	32.442	12.666	4.899	1.064	1260.533
-31.375	109.038	91.591	63.528	32.895	13.104	4.938	1.021	1260.533
-31.125	109.080	91.878	62.673	32.790	12.961	4.929	1.158	1260.533
-30.875	108.665	92.174	63.386	32.123	11.934	5.248	1.163	1260.533
-30.625	108.322	90.860	62.459	32.680	11.957	4.811	1.095	1260.533
-30.375	107.782	90.943	63.172	32.728	12.123	5.016	1.125	1260.533
-30.125	107.867	90.526	62.509	32.657	13.044	4.908	1.139	1260.533
-29.875	108.255	92.252	62.141	32.156	12.644	4.979	1.059	1260.533
-29.625	107.800	90.948	62.819	32.454	12.493	4.867	1.158	1260.533
-29.375	107.143	90.433	61.955	32.434	12.757	4.715	1.064	1260.533
-29.125	108.590	89.781	62.257	32.399	12.100	4.878	1.115	1260.533
-28.875	108.774	90.923	62.689	32.243	12.214	4.820	1.061	1260.533
-28.625	107.730	90.540	62.287	32.289	12.108	4.873	1.092	1260.533
-28.375	107.908	90.553	62.219	32.447	12.515	4.856	1.105	1260.533
-28.125	107.063	90.686	62.577	32.308	12.402	4.721	1.049	1260.533
-27.875	106.892	90.805	62.718	32.128	12.138	4.690	1.069	1260.533
-27.625	106.338	90.671	61.955	32.411	11.995	4.744	1.056	1260.533
-27.375	106.640	90.473	61.783	31.917	12.289	4.796	1.092	1260.533
-27.125	106.740	90.583	61.761	32.095	12.357	4.604	1.026	1260.533
-26.875	105.698	89.713	61.257	31.822	12.387	4.768	1.074	1260.533
-26.625	106.678	89.864	62.343	31.626	12.070	4.796	0.998	1260.533
-26.375	106.915	90.287	61.970	31.671	12.183	4.635	1.071	1260.533
-26.125	107.180	89.188	62.411	32.100	12.047	4.871	1.052	1260.533
-25.875	106.773	89.957	62.600	31.832	12.342	4.809	0.976	1260.533
-25.625	106.515	90.118	62.076	32.118	12.297	4.790	1.112	1260.533

Midpoint	Ratio: 8.6	2.8	0.97	0.34	0.11	0.041	0.009	Volume
-25.375	105.968	90.161	61.988	32.198	12.153	4.761	1.041	1260.533
-25.125	107.745	90.435	62.328	32.070	11.580	4.678	1.071	1260.533
-24.875	107.025	90.671	62.655	31.787	12.138	4.748	0.971	1260.533
-24.625	108.410	90.621	62.749	32.203	11.972	4.716	1.013	1260.533
-24.375	106.538	90.942	63.127	32.161	12.659	4.983	1.041	1260.533
-24.125	108.016	90.513	63.033	32.758	12.108	4.686	0.946	1260.533
-23.875	107.845	90.972	63.374	32.532	12.183	4.860	1.056	1260.533
-23.625	107.790	91.833	63.462	33.011	12.802	4.669	0.989	1260.533
-23.375	108.540	91.408	63.918	32.948	12.297	4.800	1.019	1260.533
-23.125	109.089	92.340	62.996	32.590	12.478	4.663	1.021	1260.533
-22.875	108.382	92.491	64.412	33.089	12.100	4.850	0.983	1260.533
-22.625	110.137	93.270	64.306	33.194	12.251	4.757	0.986	1260.533
-22.375	109.542	92.662	64.909	33.310	12.342	4.792	0.991	1260.533
-22.125	109.302	94.612	64.795	33.493	12.848	4.785	0.998	1260.533
-21.875	110.722	93.260	64.808	33.734	12.727	4.729	0.974	1260.533
-21.625	112.859	94.669	66.627	34.265	12.599	4.901	1.018	1260.533
-21.375	112.058	94.964	65.639	34.549	12.968	4.850	0.945	1260.533
-21.125	113.377	95.954	66.544	34.800	13.059	4.796	0.971	1260.533
-20.875	113.518	97.833	67.058	34.760	13.127	4.837	0.895	1260.533
-20.625	114.846	97.705	67.658	35.751	12.833	4.706	0.921	1260.533
-20.375	115.099	98.894	69.236	35.663	12.953	4.813	0.921	1260.533
-20.125	115.946	98.534	69.569	34.787	12.568	4.671	0.877	1260.533
-19.875	115.204	98.628	68.858	35.176	12.704	4.746	0.809	1260.533
-19.625	115.226	99.545	69.108	34.599	12.115	4.703	0.850	1260.533
-19.375	46.797	40.317	28.472	14.265	4.997	1.825	0.351	1260.533
-19.125	13.425	11.492	8.058	4.074	1.638	0.499	0.068	5020.223
-18.875	11.661	9.967	7.047	3.412	1.306	0.452	0.088	126.617
-18.625	10.726	8.866	5.930	3.033	1.132	0.351	0.073	109.272
-18.375	20.687	15.674	8.254	2.817	0.777	0.291	0.073	96.873
-18.125	20.192	15.043	7.669	2.489	0.649	0.205	0.051	87.108
-17.875	17.922	13.767	6.916	2.336	0.627	0.198	0.046	79.052
-17.625	16.965	12.837	6.674	2.208	0.513	0.205	0.033	72.219
-17.375	16.297	12.603	6.462	2.188	0.566	0.233	0.031	66.314
-17.125	15.343	11.826	6.341	2.087	0.619	0.181	0.025	61.144
-16.875	14.983	11.741	5.794	2.025	0.521	0.189	0.036	56.571
-16.625	14.357	10.819	5.754	1.919	0.551	0.144	0.035	52.497
-16.375	13.445	10.019	5.247	1.844	0.536	0.155	0.043	48.845
-16.125	12.666	9.437	4.804	1.533	0.347	0.127	0.033	45.554
-15.875	11.923	8.811	4.461	1.548	0.415	0.131	0.031	42.579
-15.625	10.903	8.012	3.972	1.392	0.279	0.120	0.025	39.878
-15.375	9.883	7.650	3.876	1.149	0.325	0.105	0.023	37.421
-15.125	9.026	6.903	3.491	1.056	0.287	0.071	0.015	35.181
-14.875	8.488	6.325	3.203	1.071	0.189	0.082	0.018	33.134
-14.625	8.231	6.373	3.085	0.966	0.264	0.069	0.015	31.262
-14.375	8.066	5.971	3.092	0.981	0.249	0.077	0.010	29.548

Midpoint	Ratio: 8.6	2.8	0.97	0.34	0.11	0.041	0.009	Volume
-14.125	8.401	6.378	3.249	0.896	0.279	0.073	0.012	27.976
-13.875	8.404	6.323	3.082	1.006	0.257	0.065	0.013	26.535
-13.625	8.431	6.471	3.375	1.024	0.226	0.099	0.020	25.212
-13.375	8.204	6.122	3.513	1.049	0.189	0.084	0.020	23.999
-13.125	8.176	6.167	3.332	0.951	0.287	0.090	0.020	22.886
-12.875	8.009	6.021	3.221	1.069	0.279	0.067	0.013	21.866
-12.625	7.841	6.132	3.176	1.049	0.272	0.086	0.010	20.932
-12.375	7.609	5.838	3.120	0.926	0.226	0.082	0.010	20.077
-12.125	7.534	5.926	3.153	0.918	0.219	0.071	0.025	19.295
-11.875	7.384	5.853	2.805	0.830	0.128	0.056	0.012	18.583
-11.625	7.464	5.411	2.672	0.815	0.143	0.060	0.015	17.935
-11.375	6.936	5.182	2.631	0.795	0.181	0.062	0.007	17.348
-11.125	6.596	5.167	2.475	0.708	0.219	0.052	0.008	16.818
-10.875	6.574	5.013	2.624	0.820	0.181	0.065	0.010	16.342
-10.625	6.959	5.250	2.636	0.733	0.189	0.054	0.012	15.916
-10.375	7.184	5.202	2.926	0.878	0.181	0.039	0.012	15.539
-10.125	7.711	5.876	3.110	0.918	0.242	0.063	0.017	15.209
-9.875	8.544	6.592	3.317	0.845	0.272	0.075	0.015	14.923
-9.625	9.548	7.318	3.982	1.157	0.257	0.095	0.022	14.679
-9.375	9.816	7.652	4.096	1.405	0.362	0.086	0.015	14.477
-9.125	10.373	8.117	4.489	1.528	0.332	0.082	0.022	14.315
-8.875	10.023	7.790	4.509	1.603	0.325	0.110	0.022	14.193
-8.625	9.834	7.728	4.300	1.603	0.347	0.108	0.012	14.109
-8.375	3.944	3.124	2.014	0.825	0.272	0.103	0.025	14.064
-8.125	2.392	1.900	1.235	0.637	0.204	0.088	0.020	14.053
-7.875	2.102	1.721	1.059	0.595	0.181	0.069	0.027	14.053
-7.625	1.900	1.551	1.036	0.524	0.166	0.067	0.015	14.053
-7.375	1.482	1.319	0.844	0.414	0.098	0.045	0.022	14.053
-7.125	1.355	1.118	0.864	0.371	0.143	0.050	0.013	14.053
-6.875	1.202	1.020	0.615	0.321	0.091	0.047	0.008	14.053
-6.625	1.047	0.844	0.534	0.261	0.068	0.049	0.012	14.053
-6.375	0.750	0.699	0.428	0.213	0.106	0.034	0.002	14.053
-6.125	0.835	0.581	0.411	0.208	0.045	0.030	0.005	14.053
-5.875	0.685	0.603	0.353	0.178	0.060	0.019	0.000	14.053
-5.625	0.557	0.523	0.292	0.161	0.060	0.026	0.005	14.053
-5.375	0.600	0.485	0.287	0.135	0.060	0.024	0.008	14.053
-5.125	0.577	0.500	0.353	0.125	0.030	0.017	0.003	14.053
-4.875	0.562	0.493	0.355	0.146	0.060	0.032	0.008	14.053
-4.625	0.605	0.510	0.345	0.188	0.083	0.026	0.000	14.053
-4.375	0.700	0.608	0.345	0.135	0.053	0.041	0.007	14.053
-4.125	0.790	0.626	0.444	0.213	0.083	0.026	0.005	14.053
-3.875	0.950	0.759	0.479	0.266	0.106	0.034	0.005	14.053
-3.625	1.015	0.832	0.587	0.263	0.091	0.032	0.007	14.053
-3.375	1.157	0.960	0.612	0.314	0.136	0.049	0.015	14.053
-3.125	1.367	1.151	0.746	0.334	0.128	0.050	0.010	14.053

Midpoint	Ratio: 8.6	2.8	0.97	0.34	0.11	0.041	0.009	Volume
-2.875	1.527	1.234	0.839	0.399	0.106	0.062	0.018	14.053
-2.625	1.747	1.440	0.907	0.409	0.181	0.075	0.013	14.053
-2.375	1.962	1.669	1.021	0.522	0.219	0.075	0.012	14.053
-2.125	2.167	1.701	1.202	0.504	0.325	0.058	0.010	14.053
-1.875	2.147	1.809	1.167	0.559	0.219	0.093	0.017	14.053
-1.625	2.217	1.799	1.149	0.587	0.204	0.080	0.017	14.053
-1.375	2.237	1.832	1.013	0.622	0.219	0.095	0.013	14.053
-1.125	2.147	1.714	0.955	0.602	0.204	0.065	0.017	14.053
-0.875	1.930	1.571	1.046	0.492	0.302	0.071	0.012	14.053
-0.625	1.842	1.410	0.900	0.504	0.257	0.090	0.012	14.053
-0.125	1.727	1.317	0.927	0.487	0.211	0.082	0.018	14.053
0.125	1.537	1.342	0.875	0.409	0.128	0.049	0.013	14.053
0.625	1.497	1.279	0.824	0.421	0.113	0.054	0.012	14.053
0.875	1.472	1.096	0.749	0.359	0.174	0.045	0.003	14.053
1.125	1.597	1.211	0.741	0.421	0.091	0.065	0.012	14.053
1.375	1.525	1.199	0.766	0.404	0.136	0.049	0.012	14.053
1.625	1.435	1.153	0.829	0.389	0.166	0.052	0.008	14.053
1.875	1.545	1.239	0.718	0.366	0.181	0.037	0.015	14.053
2.125	1.535	1.204	0.743	0.424	0.113	0.054	0.010	14.053
2.375	1.537	1.098	0.675	0.329	0.174	0.071	0.003	14.053
2.625	1.322	1.086	0.686	0.356	0.113	0.039	0.015	14.053
2.875	1.392	1.146	0.655	0.324	0.136	0.039	0.012	14.053
3.125	1.350	1.216	0.645	0.354	0.106	0.050	0.013	14.053
3.375	1.360	1.096	0.736	0.346	0.098	0.052	0.020	14.053
3.625	1.445	1.292	0.771	0.381	0.143	0.052	0.010	14.053
3.875	1.487	1.136	0.796	0.404	0.098	0.047	0.022	14.053
4.125	1.447	1.146	0.807	0.406	0.219	0.052	0.013	14.053
4.375	1.490	1.226	0.688	0.406	0.143	0.058	0.013	14.053
4.625	1.342	1.121	0.653	0.404	0.113	0.052	0.013	14.053
4.875	1.177	0.988	0.623	0.369	0.159	0.049	0.005	14.053
5.125	1.052	0.844	0.514	0.246	0.068	0.032	0.002	14.053
5.375	0.877	0.744	0.499	0.276	0.091	0.024	0.010	14.053
5.625	0.845	0.575	0.489	0.251	0.106	0.026	0.007	14.053
5.875	0.760	0.596	0.378	0.173	0.083	0.030	0.008	14.053
6.125	0.732	0.621	0.386	0.216	0.083	0.028	0.000	14.053
6.375	0.740	0.636	0.434	0.188	0.045	0.019	0.000	14.053
6.625	0.765	0.653	0.441	0.218	0.166	0.024	0.002	14.053
6.875	0.862	0.668	0.527	0.263	0.075	0.035	0.005	14.053
7.125	1.075	0.862	0.502	0.311	0.098	0.021	0.010	14.053
7.375	1.190	0.882	0.633	0.336	0.143	0.043	0.015	14.053
7.625	1.412	1.179	0.761	0.429	0.174	0.034	0.013	14.053
7.875	1.732	1.347	0.978	0.472	0.136	0.069	0.022	14.053
8.125	2.075	1.598	1.011	0.502	0.174	0.050	0.007	14.053
8.375	3.452	2.719	1.774	0.718	0.279	0.080	0.027	14.064
8.625	8.901	7.150	3.992	1.355	0.347	0.080	0.017	14.109

Midpoint	Ratio: 8.6	2.8	0.97	0.34	0.11	0.041	0.009	Volume
8.875	9.841	7.680	4.579	1.563	0.332	0.120	0.028	14.193
9.125	9.821	7.743	4.408	1.428	0.325	0.088	0.022	14.315
9.375	9.918	7.796	4.012	1.450	0.332	0.090	0.017	14.477
9.625	9.411	6.984	3.796	1.182	0.181	0.095	0.015	14.679
9.875	8.478	6.587	3.355	0.973	0.219	0.069	0.018	14.923
10.125	7.864	5.963	3.047	0.898	0.136	0.045	0.008	15.209
10.375	7.219	5.358	2.742	0.843	0.151	0.069	0.008	15.539
10.625	6.731	5.011	2.636	0.810	0.159	0.080	0.008	15.916
10.875	6.524	4.787	2.568	0.750	0.159	0.037	0.015	16.342
11.125	6.656	4.913	2.470	0.750	0.189	0.062	0.017	16.818
11.375	6.906	5.215	2.659	0.773	0.159	0.062	0.012	17.348
11.625	7.314	5.448	2.798	0.808	0.219	0.054	0.013	17.935
11.875	7.371	5.511	2.767	0.830	0.166	0.065	0.013	18.583
12.125	7.396	5.790	3.264	0.863	0.219	0.071	0.007	19.295
12.375	7.611	5.825	3.014	1.034	0.226	0.078	0.017	20.077
12.625	7.789	6.122	2.961	1.059	0.219	0.080	0.022	20.932
12.875	7.826	6.029	3.314	1.011	0.249	0.086	0.015	21.866
13.125	7.804	6.190	3.201	1.081	0.242	0.078	0.017	22.886
13.375	8.121	6.295	3.266	1.054	0.189	0.095	0.018	23.999
13.625	8.461	6.386	3.380	1.084	0.287	0.052	0.023	25.212
13.875	8.341	6.388	3.181	1.016	0.234	0.092	0.020	26.535
14.125	8.104	6.094	3.244	1.011	0.204	0.075	0.013	27.976
14.375	8.201	6.147	3.244	0.936	0.219	0.077	0.012	29.548
14.625	8.126	6.215	3.110	0.908	0.272	0.075	0.017	31.262
14.875	8.381	6.172	3.312	0.891	0.302	0.075	0.018	33.134
15.125	8.946	6.846	3.294	0.993	0.294	0.084	0.023	35.181
15.375	9.693	7.562	3.821	1.239	0.279	0.101	0.030	37.421
15.625	10.776	8.281	4.252	1.162	0.257	0.090	0.038	39.878
15.875	12.046	9.130	4.665	1.560	0.309	0.157	0.028	42.579
16.125	12.588	9.575	4.932	1.535	0.370	0.138	0.033	45.554
16.375	13.475	10.356	5.419	1.698	0.460	0.148	0.035	48.845
16.625	14.163	11.191	5.620	2.042	0.438	0.155	0.018	52.497
16.875	14.825	11.439	5.968	2.100	0.483	0.196	0.043	56.571
17.125	15.862	12.166	6.288	1.974	0.513	0.174	0.035	61.144
17.375	16.282	12.128	6.324	2.145	0.642	0.168	0.035	66.314
17.625	16.785	12.603	6.583	2.175	0.634	0.181	0.027	72.219
17.875	17.622	13.545	6.989	2.288	0.694	0.172	0.045	79.052
18.125	19.997	15.156	7.758	2.481	0.589	0.237	0.033	87.108
18.375	20.099	15.591	8.393	2.825	0.838	0.250	0.056	96.873
18.625	10.263	8.891	6.021	2.863	1.117	0.435	0.071	109.272
18.875	11.493	10.055	6.785	3.465	1.389	0.502	0.085	126.617
19.125	13.535	11.364	8.068	4.172	1.495	0.583	0.093	5020.223
19.375	47.317	41.023	27.930	14.177	5.397	1.997	0.371	1260.533
19.625	115.891	99.263	68.225	34.855	12.583	4.511	0.903	1260.533
19.875	115.281	98.105	68.344	34.963	12.395	4.731	0.900	1260.533

Midpoint	Ratio: 8.6	2.8	0.97	0.34	0.11	0.041	0.009	Volume
20.125	115.816	99.625	68.414	35.665	12.923	4.686	0.883	1260.533
20.375	115.656	98.133	67.847	35.577	12.780	4.886	0.955	1260.533
20.625	113.871	97.182	67.565	35.156	13.051	4.856	1.027	1260.533
20.875	113.829	98.972	68.251	34.654	13.436	4.929	0.956	1260.533
21.125	113.291	96.791	66.663	34.524	12.591	4.850	0.923	1260.533
21.375	111.496	95.626	66.078	34.215	12.863	4.708	1.071	1260.533
21.625	111.197	93.825	65.886	33.934	12.636	4.904	0.940	1260.533
21.875	110.855	94.207	65.987	33.400	12.644	4.680	1.021	1260.533
22.125	110.664	94.067	64.586	33.834	12.991	4.678	0.961	1260.533
22.375	109.024	93.222	65.058	33.249	12.410	4.869	0.973	1260.533
22.625	108.729	92.875	64.246	33.397	12.923	4.678	0.976	1260.533
22.875	109.629	92.362	64.077	33.091	12.493	4.710	0.960	1260.533
23.125	108.478	92.194	63.923	33.169	12.493	4.733	0.973	1260.533
23.375	107.798	91.262	63.344	33.046	12.742	5.011	1.069	1260.533
23.625	107.977	90.875	62.504	32.760	12.221	4.744	1.016	1260.533
23.875	107.815	91.689	63.598	33.014	11.995	4.910	0.993	1260.533
24.125	107.520	91.021	62.235	32.562	11.942	4.729	1.037	1260.533
24.375	106.728	89.953	63.389	32.171	12.365	4.738	0.979	1260.533
24.625	107.142	89.880	62.857	32.552	12.614	4.815	1.013	1260.533
24.875	106.952	89.654	62.748	32.519	12.402	4.802	0.998	1260.533
25.125	106.930	89.802	62.164	32.424	12.055	4.665	0.988	1260.533
25.375	106.393	90.113	62.020	32.424	12.025	4.779	1.024	1260.533
25.625	106.530	89.630	61.730	32.522	12.085	4.824	1.090	1260.533
25.875	106.847	90.392	62.300	32.103	12.425	4.835	1.072	1260.533
26.125	105.857	90.462	62.580	31.756	11.949	4.790	1.046	1260.533
26.375	106.408	89.598	62.020	31.536	12.591	4.682	1.037	1260.533
26.625	105.848	89.164	62.769	32.025	12.410	4.648	1.054	1260.533
26.875	105.720	89.324	62.247	31.970	11.919	4.781	1.087	1260.533
27.125	108.113	90.309	62.275	31.767	12.289	4.747	1.023	1260.533
27.375	106.385	90.013	62.222	31.812	11.210	4.858	1.107	1260.533
27.625	106.950	90.349	62.242	32.344	12.327	4.903	1.105	1260.533
27.875	106.248	89.943	61.804	32.331	12.176	4.738	1.077	1260.533
28.125	107.667	89.726	62.224	32.261	12.070	4.757	1.107	1260.533
28.375	106.345	90.129	62.693	32.414	11.821	4.787	1.067	1260.533
28.625	107.440	91.045	62.537	32.813	12.251	4.835	1.076	1260.533
28.875	106.943	90.626	62.361	32.058	11.980	4.604	1.110	1260.533
29.125	107.052	90.400	62.618	32.155	12.229	4.817	1.085	1260.533
29.375	108.557	91.435	62.388	32.185	12.508	4.822	1.122	1260.533
29.625	107.488	91.194	62.497	32.663	12.561	4.704	1.143	1260.533
29.875	108.497	90.855	62.880	32.030	12.576	5.022	1.097	1260.533
30.125	107.992	91.423	62.539	31.987	12.191	4.938	1.145	1260.533
30.375	108.770	91.463	63.144	32.532	12.470	5.026	1.009	1260.533
30.625	107.785	92.136	63.240	32.908	12.161	4.813	1.112	1260.533
30.875	108.360	91.334	63.099	32.572	12.553	4.966	1.127	1260.533
31.125	107.277	91.925	63.059	32.301	12.900	4.998	1.119	1260.533

Midpoint	Ratio: 8.6	2.8	0.97	0.34	0.11	0.041	0.009	Volume
31.375	108.322	91.415	62.446	32.941	12.516	4.942	1.160	1260.533
31.625	109.172	93.069	62.892	32.692	13.021	4.889	1.049	1260.533
31.875	110.123	92.041	63.273	32.825	12.734	4.959	1.095	1260.533
32.125	108.312	91.840	63.618	33.034	12.621	4.729	1.099	1260.533
32.375	108.688	92.026	63.313	32.702	12.765	4.845	1.092	1260.533
32.625	109.323	92.101	63.215	32.690	12.621	4.981	1.175	1260.533
32.875	108.863	92.312	63.525	32.670	12.757	4.889	1.061	1260.533
33.125	109.010	91.950	63.401	32.958	12.531	4.815	1.094	1260.533
33.375	109.715	91.968	63.432	32.519	12.546	4.942	1.069	1260.533
33.625	110.355	93.229	63.427	33.330	12.508	4.861	1.112	1260.533
33.875	109.748	91.299	63.427	32.687	12.229	4.945	1.085	1260.533
34.125	109.152	92.568	63.147	32.286	12.417	5.110	1.127	1260.533
34.375	109.063	92.832	63.495	32.700	12.380	4.822	1.148	1260.533
34.625	110.802	92.586	64.349	32.512	13.036	4.889	1.177	1260.533
34.875	110.039	92.186	63.237	32.474	12.251	4.824	1.109	1260.533
35.125	109.612	93.325	63.467	32.991	12.576	5.184	1.119	1260.533
35.375	109.337	92.726	63.563	33.049	13.127	4.944	1.182	1260.533
35.625	110.005	92.111	62.935	32.692	12.825	4.750	1.143	1260.533
35.875	110.542	92.330	63.742	32.850	12.719	4.940	1.115	1260.533
36.125	109.308	92.382	63.092	32.522	12.644	4.800	1.037	1260.533
36.375	109.720	93.431	63.449	33.061	12.810	4.873	1.124	1260.533
36.625	110.295	91.870	63.306	33.059	12.644	5.130	1.115	1260.533
36.875	109.737	91.591	63.316	33.104	12.689	4.972	1.130	1260.533
37.125	110.574	92.159	63.081	32.349	12.795	5.084	1.140	1260.533
37.375	109.812	92.657	63.739	32.542	12.712	4.636	1.109	1260.533
37.625	110.400	92.556	63.598	32.590	12.787	4.871	1.090	1260.533
37.875	109.732	93.038	63.555	32.384	12.900	4.776	1.190	1260.533
38.125	109.646	91.591	62.875	32.861	12.244	5.024	1.089	1260.533
38.375	109.145	93.237	64.205	32.642	12.387	4.968	1.119	1260.533
38.625	109.199	91.558	63.288	33.234	12.546	5.032	1.117	1260.533
38.875	110.007	92.687	63.094	33.141	12.093	4.981	1.157	1260.533
39.125	110.015	92.269	63.177	32.941	12.515	5.022	1.152	1260.533
39.375	109.192	92.845	63.540	32.785	12.183	4.957	1.112	1260.533
39.625	108.512	92.556	63.729	32.740	12.425	4.757	1.102	1260.533
39.875	109.365	92.588	62.854	32.411	12.772	5.076	1.067	1260.533
40.125	108.955	92.679	63.505	32.717	12.229	4.929	1.172	1260.533
40.375	109.684	92.671	63.548	32.201	12.523	4.733	1.122	1260.533
40.625	109.507	92.096	63.311	32.351	12.274	5.007	1.124	1260.533
40.875	108.745	92.579	62.857	32.790	12.297	4.876	1.157	1260.533
41.125	108.347	91.988	62.504	33.006	12.395	4.654	1.200	1260.533
41.375	109.912	91.920	63.079	32.288	12.470	5.039	1.188	1260.533
41.625	109.819	91.848	62.285	32.146	13.051	4.943	1.090	1260.533
41.875	109.260	91.845	62.592	32.645	12.410	4.889	1.170	1260.533
42.125	109.359	91.814	62.157	32.537	12.410	4.945	1.197	1260.533
42.375	109.590	92.076	63.167	32.502	12.719	4.899	1.192	1260.533

Midpoint	Ratio: 8.6	2.8	0.97	0.34	0.11	0.041	0.009	Volume
42.625	109.067	91.564	62.663	32.148	11.776	5.003	1.148	1260.533
42.875	107.752	91.239	62.734	32.025	12.666	4.753	1.180	1260.533
43.125	108.835	92.142	62.865	32.519	12.878	4.805	1.180	1260.533
43.375	108.631	91.654	63.084	32.339	13.036	4.891	1.119	1260.533
43.625	108.534	91.770	63.230	32.359	12.485	4.988	1.162	1260.533
43.875	108.760	90.267	62.928	32.512	12.123	4.776	1.105	1260.533
44.125	108.154	90.975	62.096	32.156	12.576	4.974	1.160	1260.533
44.375	107.862	91.845	62.401	31.935	12.221	4.768	1.158	1260.533
44.625	108.215	91.106	62.474	32.228	12.697	4.930	1.127	1260.533
44.875	107.455	90.259	61.907	32.514	11.912	4.828	1.090	1260.533
45.125	108.274	90.952	62.247	32.735	11.866	4.930	1.129	1260.533
45.375	107.222	90.425	61.390	32.063	12.093	4.882	1.130	1260.533
45.625	107.410	90.819	61.494	31.844	12.349	4.654	1.125	1260.533
45.875	106.702	90.149	61.930	31.870	12.055	4.893	1.090	1260.533
46.125	107.263	90.968	61.859	31.754	12.251	4.860	1.104	1260.533
46.375	106.897	90.868	61.252	31.581	12.516	4.847	1.084	1260.533
46.625	107.448	89.864	61.358	31.744	11.821	4.957	1.037	1260.533
46.875	106.122	89.701	61.073	31.912	11.972	4.789	1.072	1260.533
47.125	105.995	89.425	61.307	31.895	11.806	4.807	1.120	1260.533
47.375	105.793	88.980	60.722	31.676	12.236	4.727	1.090	1260.533
47.625	105.678	88.286	61.430	31.792	12.312	4.930	1.087	1260.533
47.875	105.047	88.827	60.815	31.631	11.972	4.807	1.127	1260.533
48.125	105.157	88.567	60.980	31.528	11.859	4.734	1.135	1260.533
48.375	105.366	88.082	61.126	31.323	12.191	4.777	1.072	1260.533
48.625	103.980	88.010	59.840	31.107	12.206	4.761	1.152	1260.533
48.875	103.383	87.919	60.410	30.558	12.380	4.636	1.059	1260.533
49.125	103.907	88.138	60.377	30.964	11.987	4.632	1.089	1260.533
49.375	103.240	87.010	59.760	31.155	12.017	4.738	1.094	1260.533
49.625	103.547	87.186	59.268	30.284	11.776	4.714	1.122	1260.533
49.875	102.431	87.070	58.779	30.317	11.595	4.548	1.024	1260.533
50.125	102.186	86.816	59.795	30.868	11.413	4.523	1.104	1260.533
50.375	102.071	86.263	58.567	30.214	11.700	4.611	1.069	1260.533
50.625	102.051	85.263	58.492	30.783	11.813	4.568	1.059	1260.533
50.875	102.149	85.979	57.935	30.745	11.753	4.630	1.049	1260.533
51.125	101.697	84.944	58.144	30.246	11.851	4.593	1.037	1260.533
51.375	100.878	85.499	58.275	30.003	11.361	4.546	1.032	1260.533
51.625	101.436	86.610	57.960	30.254	11.542	4.453	1.042	1260.533
51.875	101.771	85.532	58.285	29.363	11.761	4.458	1.081	1260.533
52.125	100.734	84.929	58.008	30.339	11.851	4.378	1.077	1260.533
52.375	100.909	84.534	57.794	30.179	10.719	4.512	1.011	1260.533
52.625	100.141	84.041	57.443	30.289	11.285	4.619	1.031	1260.533
52.875	101.431	84.491	58.162	29.840	11.776	4.468	1.056	1260.533
53.125	99.394	84.946	58.036	30.206	11.557	4.553	1.042	1260.533
53.375	99.689	84.471	57.753	29.589	11.421	4.342	1.082	1260.533
53.625	99.654	84.024	58.330	29.554	10.847	4.609	1.014	1260.533

Midpoint	Ratio: 8.6	2.8	0.97	0.34	0.11	0.041	0.009	Volume
53.875	100.561	83.836	57.428	30.128	11.338	4.443	1.094	1260.533
54.125	99.219	83.735	57.587	29.439	11.527	4.387	1.076	1260.533
54.375	100.933	83.389	58.600	29.185	10.885	4.441	1.011	1260.533
54.625	100.049	84.308	57.131	29.780	11.059	4.410	0.994	1260.533
54.875	99.408	84.276	57.020	29.933	11.345	4.525	1.016	1260.533
55.125	99.526	84.104	57.816	29.872	11.708	4.359	1.029	1260.533
55.375	99.322	84.017	57.721	29.253	11.617	4.639	1.021	1260.533
55.625	98.597	83.418	56.801	29.770	11.225	4.551	0.983	1260.533
55.875	99.266	83.305	57.123	29.862	11.368	4.460	1.037	1260.533
56.125	99.107	83.358	58.275	29.469	11.247	4.340	1.021	1260.533
56.375	99.534	82.880	57.055	29.416	12.032	4.415	1.049	1260.533
56.625	99.883	83.404	57.441	29.807	11.723	4.516	1.009	1260.533
56.875	99.311	83.004	57.441	30.015	11.512	4.493	1.019	1260.533
57.125	99.032	83.277	57.035	29.338	10.749	4.400	1.061	1260.533
57.375	98.591	82.768	57.292	29.566	10.953	4.518	1.021	1260.533
57.625	98.911	83.011	56.942	29.027	11.164	4.442	0.993	1260.533
57.875	98.593	82.813	56.539	29.785	10.749	4.551	1.023	1260.533
58.125	98.556	83.517	57.111	29.742	11.232	4.464	0.973	1260.533
58.375	97.614	82.345	56.776	29.042	11.353	4.292	0.974	1260.533
58.625	4.492	3.978	2.752	1.495	0.551	0.256	0.058	1260.533

Table of K^+ population data for simulation with K^+ and Na^+ across bulk density ratios (#JJF-GRS1459)

	Population in histogram bin at Na^+/K^+ ratio:							
Midpoint	8.6	2.8	0.97	0.34	0.11	0.041	0.009	Volume
-58.625	0.000	0.000	0.000	0.000	0.000	0.000	0.000	1232.996
-58.375	0.000	0.000	0.000	0.000	0.000	0.000	0.000	1232.996
-58.125	3.115	8.208	16.632	24.526	30.330	32.100	32.954	1232.996
-57.875	11.508	29.521	59.110	87.425	104.480	111.741	112.602	1232.996
-57.625	10.976	30.207	58.916	87.465	104.185	110.241	113.759	1232.996
-57.375	11.413	29.589	59.563	87.686	104.291	110.720	114.272	1232.996
-57.125	11.263	29.790	58.961	87.244	104.736	110.447	113.343	1232.996
-56.875	11.328	29.873	59.205	87.505	105.068	110.791	114.138	1232.996
-56.625	11.288	30.006	59.228	88.343	105.378	110.888	114.294	1232.996
-56.375	11.088	30.094	58.782	86.958	104.404	110.722	113.338	1232.996
-56.125	11.770	29.702	58.623	86.263	104.532	110.634	113.553	1232.996
-55.875	11.198	30.237	58.794	87.389	103.204	110.978	113.371	1232.996
-55.625	11.433	30.069	58.865	87.345	104.072	110.529	113.326	1232.996
-55.375	11.383	30.172	59.556	87.164	104.781	110.555	113.372	1232.996
-55.125	11.098	30.338	59.485	86.868	103.422	110.331	112.957	1232.996
-54.875	11.411	30.506	58.898	87.269	104.562	111.300	113.401	1232.996
-54.625	11.473	29.634	59.084	88.052	107.303	110.563	112.371	1232.996
-54.375	11.571	30.215	59.240	87.299	104.268	110.433	112.433	1232.996
-54.125	11.083	30.094	59.230	86.542	104.585	110.874	113.048	1232.996
-53.875	11.231	30.353	58.905	86.705	104.442	110.305	114.148	1232.996

Midpoint	Ratio: 8.6	2.8	0.97	0.34	0.11	0.041	0.009	Volume
-53.625	11.586	30.619	59.546	87.252	103.959	110.781	113.101	1232.996
-53.375	11.291	29.993	59.921	87.962	103.317	110.180	113.624	1232.996
-53.125	11.451	30.350	59.152	86.652	103.747	109.883	112.782	1232.996
-52.875	11.406	30.516	58.757	87.555	103.430	110.542	113.137	1232.996
-52.625	11.278	30.230	58.789	87.084	105.438	110.962	113.150	1232.996
-52.375	11.163	29.772	59.802	88.140	104.691	110.813	113.258	1232.996
-52.125	11.463	30.549	60.115	87.593	103.060	111.204	113.697	1232.996
-51.875	11.371	30.237	59.386	87.018	104.359	111.267	114.171	1232.996
-51.625	11.611	30.222	59.508	87.061	103.619	111.633	113.883	1232.996
-51.375	11.238	30.408	59.535	86.672	103.385	111.250	113.666	1232.996
-51.125	11.653	30.388	60.450	87.074	104.948	111.172	114.292	1232.996
-50.875	11.566	30.410	59.019	88.403	104.193	111.865	114.759	1232.996
-50.625	11.473	31.056	59.714	88.619	104.887	112.326	113.914	1232.996
-50.375	11.643	30.684	59.369	88.401	105.476	111.710	114.794	1232.996
-50.125	11.743	30.745	59.949	88.542	104.932	112.479	114.290	1232.996
-49.875	11.526	30.390	60.322	88.468	107.008	112.710	114.597	1232.996
-49.625	11.403	30.976	60.115	88.464	106.782	113.284	115.456	1232.996
-49.375	11.823	31.059	60.430	89.204	107.235	112.904	115.948	1232.996
-49.125	11.688	30.893	60.334	89.853	107.672	113.461	115.079	1232.996
-48.875	12.005	31.511	61.584	90.245	107.990	113.134	116.771	1232.996
-48.625	11.801	31.765	61.201	89.362	107.771	115.038	116.712	1232.996
-48.375	11.798	31.082	61.022	89.700	107.061	114.100	117.999	1232.996
-48.125	11.723	31.089	61.309	91.068	108.707	114.397	117.796	1232.996
-47.875	12.008	31.019	61.715	91.228	109.409	115.948	117.842	1232.996
-47.625	11.728	31.408	62.175	90.955	108.012	115.479	117.723	1232.996
-47.375	11.878	31.591	62.754	91.105	110.360	115.400	118.309	1232.996
-47.125	12.063	31.825	62.497	90.932	108.450	115.809	119.642	1232.996
-46.875	12.066	31.571	62.108	91.712	109.258	116.104	118.568	1232.996
-46.625	12.143	31.715	62.542	92.435	109.137	116.061	119.140	1232.996
-46.375	12.076	32.348	63.049	92.801	109.839	116.945	118.632	1232.996
-46.125	12.155	31.893	62.824	92.605	109.839	116.822	119.643	1232.996
-45.875	11.936	32.114	61.912	93.193	111.975	117.574	119.683	1232.996
-45.625	11.878	31.881	62.370	93.358	110.707	116.898	120.334	1232.996
-45.375	12.000	31.782	63.573	92.756	110.782	117.421	120.721	1232.996
-45.125	12.238	32.059	62.658	92.984	110.209	116.880	120.298	1232.996
-44.875	12.121	32.310	63.028	92.493	111.228	117.230	120.054	1232.996
-44.625	12.043	32.235	62.983	92.932	110.503	117.743	120.242	1232.996
-44.375	12.183	32.154	63.381	92.427	111.092	118.058	120.316	1232.996
-44.125	12.413	32.127	63.825	93.115	111.688	118.502	120.653	1232.996
-43.875	12.255	32.561	63.424	93.727	111.892	118.228	120.180	1232.996
-43.625	12.093	32.504	63.651	93.383	111.394	117.197	120.518	1232.996
-43.375	12.155	32.685	63.732	92.886	111.749	118.827	120.389	1232.996
-43.125	12.170	31.818	63.293	93.827	109.635	118.382	120.779	1232.996
-42.875	12.250	32.605	63.913	93.702	111.794	118.496	121.591	1232.996
-42.625	12.435	32.451	63.840	93.970	112.232	118.476	121.776	1232.996

Midpoint	Ratio: 8.6	2.8	0.97	0.34	0.11	0.041	0.009	Volume
-42.375	12.256	32.443	63.147	93.893	112.254	119.048	121.253	1232.996
-42.125	12.443	32.529	63.800	93.386	111.213	118.550	121.557	1232.996
-41.875	12.360	32.592	64.488	93.765	113.160	118.157	121.508	1232.996
-41.625	12.308	32.414	63.961	93.777	112.911	118.631	121.990	1232.996
-41.375	12.358	32.820	64.354	94.417	113.432	119.662	122.363	1232.996
-41.125	12.013	32.918	64.566	94.701	110.058	119.216	122.535	1232.996
-40.875	12.643	32.875	64.145	93.975	114.451	119.586	120.890	1232.996
-40.625	12.161	32.632	64.263	93.795	112.949	119.446	121.332	1232.996
-40.375	12.286	32.939	63.414	93.764	113.311	119.232	121.549	1232.996
-40.125	12.340	33.436	63.467	94.422	111.303	119.613	121.635	1232.996
-39.875	12.353	32.710	63.500	94.480	113.017	119.214	121.758	1232.996
-39.625	12.415	32.740	64.672	94.432	113.402	118.382	121.839	1232.996
-39.375	12.595	32.348	65.140	94.048	113.953	118.945	122.515	1232.996
-39.125	12.445	32.768	65.032	94.196	112.707	119.873	122.882	1232.996
-38.875	12.495	33.044	64.747	94.086	112.722	120.297	122.260	1232.996
-38.625	12.123	32.888	64.493	94.289	113.545	119.810	122.428	1232.996
-38.375	12.338	32.838	64.044	94.741	112.383	119.989	121.882	1232.996
-38.125	13.030	33.054	64.853	94.638	114.089	119.909	122.369	1232.996
-37.875	12.578	32.836	63.618	95.654	114.157	119.813	122.815	1232.996
-37.625	12.256	32.657	64.861	94.861	113.666	119.553	122.674	1232.996
-37.375	12.751	32.657	64.450	94.688	113.508	119.499	121.949	1232.996
-37.125	12.723	33.089	63.876	94.858	112.941	119.921	122.084	1232.996
-36.875	12.681	33.203	65.513	94.996	114.617	119.891	122.885	1232.996
-36.625	12.410	32.235	64.455	94.455	112.783	119.641	122.179	1232.996
-36.375	12.435	32.680	64.122	95.205	112.111	120.167	122.736	1232.996
-36.125	12.255	32.778	64.455	94.432	112.300	120.732	122.041	1232.996
-35.875	12.698	33.220	64.382	95.174	112.624	120.108	121.521	1232.996
-35.625	12.696	32.755	65.279	94.043	113.175	119.546	122.497	1232.996
-35.375	12.216	32.707	64.735	94.620	112.541	119.070	122.436	1232.996
-35.125	12.588	33.024	64.291	95.104	112.753	119.471	122.739	1232.996
-34.875	12.520	32.341	64.389	94.653	113.877	119.987	121.894	1232.996
-34.625	12.588	33.177	65.113	95.160	114.323	120.217	122.056	1232.996
-34.375	12.573	32.318	64.223	94.803	114.489	120.149	121.831	1232.996
-34.125	12.456	32.793	64.450	94.226	112.670	119.879	121.857	1232.996
-33.875	12.496	32.778	64.264	95.453	112.654	118.983	122.240	1232.996
-33.625	12.133	32.536	65.045	94.352	112.813	119.088	122.053	1232.996
-33.375	12.318	32.388	64.238	95.097	112.224	119.433	121.940	1232.996
-33.125	12.211	32.630	64.415	95.012	112.436	119.859	122.263	1232.996
-32.875	12.243	32.624	63.782	95.061	114.504	119.398	122.192	1232.996
-32.625	12.250	32.647	64.218	94.176	113.508	119.758	123.124	1232.996
-32.375	12.858	33.230	64.203	93.782	114.345	119.136	122.046	1232.996
-32.125	12.533	32.818	64.213	94.700	113.477	119.674	121.335	1232.996
-31.875	12.551	32.690	64.087	93.509	111.190	119.046	122.061	1232.996
-31.625	12.413	33.150	64.541	93.489	114.149	118.510	121.852	1232.996
-31.375	12.218	32.710	64.246	94.156	111.552	119.366	121.375	1232.996

Midpoint	Ratio: 8.6	2.8	0.97	0.34	0.11	0.041	0.009	Volume
-31.125	12.598	33.049	64.395	93.775	111.062	120.065	121.557	1232.996
-30.875	12.185	33.150	63.651	94.314	113.002	119.226	122.623	1232.996
-30.625	12.270	32.572	63.974	93.662	113.598	119.308	122.099	1232.996
-30.375	12.365	32.964	63.840	92.774	111.258	119.061	121.272	1232.996
-30.125	12.278	32.054	63.447	93.978	112.926	118.900	121.408	1232.996
-29.875	12.646	32.881	63.792	93.712	111.575	119.147	120.709	1232.996
-29.625	12.373	32.270	64.079	93.097	110.888	119.646	120.676	1232.996
-29.375	11.973	32.280	63.744	93.348	111.009	118.739	120.837	1232.996
-29.125	12.383	32.722	64.092	92.909	112.670	117.915	120.621	1232.996
-28.875	12.203	31.818	63.618	93.607	112.609	118.460	120.639	1232.996
-28.625	11.960	32.529	63.558	93.764	110.549	117.854	120.691	1232.996
-28.375	12.391	32.532	63.568	92.392	110.775	117.996	120.868	1232.996
-28.125	11.933	32.082	62.782	93.564	111.379	117.670	120.747	1232.996
-27.875	12.185	32.531	63.639	92.977	112.368	117.266	120.359	1232.996
-27.625	12.211	32.233	63.134	93.720	110.873	117.135	120.815	1232.996
-27.375	12.323	32.356	62.766	92.874	111.402	117.025	119.933	1232.996
-27.125	12.236	32.549	63.528	93.501	110.163	117.726	119.774	1232.996
-26.875	12.246	31.782	63.339	92.222	111.009	117.598	120.147	1232.996
-26.625	12.098	32.735	63.303	93.100	111.711	118.313	121.085	1232.996
-26.375	12.100	31.770	63.039	93.607	112.851	117.946	120.800	1232.996
-26.125	11.806	32.630	63.339	93.200	111.402	118.959	120.565	1232.996
-25.875	12.275	32.200	63.319	94.312	112.639	118.031	121.746	1232.996
-25.625	12.463	32.519	63.381	93.481	112.443	119.457	121.544	1232.996
-25.375	12.128	31.981	63.613	93.750	112.005	119.774	121.286	1232.996
-25.125	12.248	32.451	63.399	93.737	112.821	118.996	122.018	1232.996
-24.875	12.008	32.386	64.200	94.289	112.058	119.581	122.605	1232.996
-24.625	12.183	32.944	63.926	94.911	113.787	119.907	122.621	1232.996
-24.375	12.466	33.373	64.347	95.124	114.043	120.878	123.105	1232.996
-24.125	12.413	32.783	65.158	95.513	114.089	120.559	123.243	1232.996
-23.875	12.398	33.026	64.417	95.365	114.081	121.669	123.377	1232.996
-23.625	12.440	33.140	65.231	96.271	114.542	122.402	123.798	1232.996
-23.375	12.176	33.883	65.294	97.001	114.829	122.123	125.074	1232.996
-23.125	12.123	33.514	66.025	96.389	115.757	123.434	125.758	1232.996
-22.875	12.226	33.502	66.214	97.676	115.757	123.405	126.640	1232.996
-22.625	12.436	33.755	66.799	97.862	117.923	124.532	126.463	1232.996
-22.375	12.206	33.517	67.343	98.905	118.716	124.975	128.582	1232.996
-22.125	12.298	35.160	67.542	98.880	120.150	125.106	128.541	1232.996
-21.875	12.330	34.557	68.911	100.820	119.441	126.640	129.703	1232.996
-21.625	12.345	35.276	69.337	101.063	120.444	127.285	131.496	1232.996
-21.375	12.533	34.906	69.521	102.729	120.867	129.003	131.191	1232.996
-21.125	12.583	35.492	69.113	102.448	121.728	129.036	133.572	1232.996
-20.875	12.568	35.449	70.741	104.497	124.513	131.832	134.222	1232.996
-20.625	12.801	35.824	69.977	105.095	125.419	131.843	135.150	1232.996
-20.375	12.733	36.002	71.721	105.466	127.948	133.110	136.199	1232.996
-20.125	12.486	36.017	71.522	106.650	126.559	134.190	138.206	1232.996

Midpoint	Ratio: 8.6	2.8	0.97	0.34	0.11	0.041	0.009	Volume
-19.875	7.831	22.497	45.477	66.256	78.822	83.739	85.079	1232.996
-19.625	1.620	4.245	8.872	12.675	15.278	16.215	16.501	5642.311
-19.375	1.405	4.114	7.730	11.235	13.452	14.235	14.615	131.318
-19.125	1.115	3.091	6.898	9.536	11.723	12.391	12.459	111.609
-18.875	1.010	2.918	5.570	8.249	10.477	10.337	10.620	98.132
-18.625	0.912	2.460	5.169	7.755	9.572	10.003	10.437	87.699
-18.375	0.627	2.252	5.983	11.531	13.769	15.796	16.083	79.176
-18.125	0.540	1.980	5.991	10.868	14.840	15.925	16.335	71.991
-17.875	0.517	1.809	5.527	10.507	14.516	14.474	15.155	65.812
-17.625	0.467	1.822	5.144	9.802	13.157	13.981	14.333	60.420
-17.375	0.432	1.666	4.900	9.376	12.659	13.337	13.548	55.666
-17.125	0.392	1.478	4.615	8.911	11.338	12.515	12.824	51.439
-16.875	0.400	1.523	4.552	8.410	11.044	11.658	11.955	47.657
-16.625	0.360	1.392	4.282	8.066	9.911	11.107	11.153	44.256
-16.375	0.345	1.350	3.844	7.612	9.677	10.119	10.487	41.184
-16.125	0.282	1.073	3.506	6.811	9.043	9.667	9.807	38.400
-15.875	0.295	1.143	3.334	6.398	8.084	9.209	9.115	35.869
-15.625	0.250	0.990	3.090	5.853	7.866	8.614	8.639	33.564
-15.375	0.247	0.854	2.845	5.580	7.307	8.061	8.276	31.460
-15.125	0.247	0.882	2.697	5.344	7.262	7.444	7.767	29.537
-14.875	0.215	0.699	2.482	4.832	6.635	7.244	7.310	27.776
-14.625	0.227	0.789	2.485	5.023	6.507	7.067	7.023	26.162
-14.375	0.200	0.741	2.593	4.882	6.462	6.858	7.008	24.682
-14.125	0.210	0.839	2.437	4.762	6.092	6.774	6.869	23.324
-13.875	0.180	0.764	2.377	4.759	6.341	6.436	6.705	22.078
-13.625	0.160	0.714	2.417	4.594	5.835	6.544	6.569	20.933
-13.375	0.215	0.784	2.397	4.458	5.676	6.445	6.440	19.883
-13.125	0.207	0.787	2.175	4.280	5.699	6.094	6.085	18.920
-12.875	0.187	0.744	2.238	4.290	5.284	5.980	6.109	18.036
-12.625	0.172	0.686	2.311	4.200	5.548	5.872	5.915	17.227
-12.375	0.155	0.731	2.248	4.222	5.465	5.861	5.878	16.487
-12.125	0.172	0.691	2.034	4.145	5.201	5.842	5.721	15.810
-11.875	0.150	0.616	2.024	4.042	5.178	5.616	5.676	15.194
-11.625	0.135	0.598	2.054	4.130	5.186	5.566	5.779	14.634
-11.375	0.135	0.628	1.789	3.911	5.382	5.336	5.749	14.126
-11.125	0.140	0.596	2.031	3.823	4.861	5.601	5.630	13.668
-10.875	0.125	0.568	1.986	3.914	5.209	5.444	5.664	13.256
-10.625	0.140	0.638	2.132	3.994	5.420	5.866	5.726	12.889
-10.375	0.180	0.686	2.311	4.273	6.031	5.954	5.908	12.563
-10.125	0.192	0.751	2.344	4.421	5.850	6.404	6.448	12.278
-9.875	0.180	0.696	2.722	4.804	6.137	6.822	6.909	12.031
-9.625	0.175	0.812	2.702	4.902	6.680	6.815	6.993	11.821
-9.375	0.220	0.847	2.818	4.950	6.726	6.729	7.186	11.647
-9.125	0.180	0.980	2.803	4.947	5.971	6.766	6.698	11.508
-8.875	0.167	0.849	2.568	4.451	5.578	5.965	6.225	11.402

Midpoint	Ratio: 8.6	2.8	0.97	0.34	0.11	0.041	0.009	Volume
-8.625	0.195	0.759	2.324	3.608	4.612	5.110	5.399	11.330
-8.375	0.125	0.385	0.943	1.653	1.834	2.019	2.111	11.291
-8.125	0.080	0.281	0.628	0.926	1.042	1.143	1.177	11.282
-7.875	0.075	0.239	0.539	0.735	0.928	0.896	0.906	11.282
-7.625	0.050	0.141	0.350	0.635	0.717	0.631	0.689	11.282
-7.375	0.022	0.121	0.275	0.314	0.513	0.583	0.462	11.282
-7.125	0.035	0.078	0.202	0.283	0.294	0.362	0.383	11.282
-6.875	0.013	0.075	0.118	0.146	0.189	0.258	0.235	11.282
-6.625	0.015	0.055	0.111	0.153	0.143	0.172	0.202	11.282
-6.375	0.010	0.030	0.078	0.065	0.159	0.153	0.123	11.282
-6.125	0.010	0.030	0.038	0.063	0.060	0.105	0.075	11.282
-5.875	0.013	0.025	0.035	0.048	0.068	0.073	0.093	11.282
-5.625	0.020	0.020	0.035	0.050	0.030	0.060	0.063	11.282
-5.375	0.002	0.008	0.028	0.028	0.045	0.073	0.048	11.282
-5.125	0.000	0.008	0.018	0.030	0.053	0.062	0.056	11.282
-4.875	0.010	0.010	0.023	0.030	0.045	0.054	0.046	11.282
-4.625	0.008	0.018	0.033	0.045	0.045	0.045	0.051	11.282
-4.375	0.005	0.013	0.040	0.058	0.075	0.064	0.061	11.282
-4.125	0.007	0.010	0.028	0.065	0.091	0.065	0.055	11.282
-3.875	0.005	0.035	0.040	0.070	0.075	0.086	0.101	11.282
-3.625	0.000	0.028	0.040	0.075	0.174	0.106	0.136	11.282
-3.375	0.010	0.040	0.066	0.153	0.121	0.151	0.124	11.282
-3.125	0.015	0.038	0.106	0.148	0.242	0.192	0.177	11.282
-2.875	0.015	0.045	0.111	0.171	0.189	0.207	0.229	11.282
-2.625	0.025	0.113	0.149	0.226	0.272	0.282	0.290	11.282
-2.375	0.043	0.085	0.174	0.294	0.302	0.342	0.384	11.282
-2.125	0.042	0.095	0.156	0.294	0.423	0.402	0.381	11.282
-1.875	0.030	0.103	0.171	0.341	0.370	0.403	0.418	11.282
-1.625	0.050	0.121	0.242	0.339	0.483	0.407	0.476	11.282
-1.375	0.047	0.113	0.204	0.349	0.347	0.411	0.491	11.282
-1.125	0.037	0.128	0.199	0.361	0.483	0.407	0.428	11.282
-0.875	0.040	0.106	0.207	0.301	0.483	0.435	0.404	11.282
-0.625	0.025	0.085	0.161	0.253	0.362	0.372	0.376	11.282
-0.125	0.035	0.075	0.184	0.276	0.370	0.347	0.353	11.282
0.125	0.042	0.085	0.124	0.213	0.264	0.304	0.315	11.282
0.625	0.022	0.090	0.129	0.218	0.302	0.338	0.297	11.282
0.875	0.017	0.080	0.139	0.201	0.279	0.265	0.272	11.282
1.125	0.025	0.060	0.139	0.228	0.196	0.241	0.245	11.282
1.375	0.017	0.055	0.111	0.161	0.189	0.218	0.229	11.282
1.625	0.010	0.068	0.096	0.171	0.196	0.247	0.225	11.282
1.875	0.020	0.043	0.083	0.163	0.196	0.217	0.242	11.282
2.125	0.015	0.048	0.098	0.151	0.257	0.228	0.219	11.282
2.375	0.017	0.050	0.101	0.143	0.211	0.202	0.217	11.282
2.625	0.020	0.043	0.118	0.146	0.211	0.226	0.212	11.282
2.875	0.027	0.065	0.108	0.188	0.385	0.239	0.229	11.282

Midpoint	Ratio: 8.6	2.8	0.97	0.34	0.11	0.041	0.009	Volume
3.125	0.020	0.040	0.136	0.178	0.287	0.261	0.244	11.282
3.375	0.010	0.065	0.139	0.218	0.294	0.218	0.282	11.282
3.625	0.037	0.063	0.123	0.193	0.272	0.293	0.215	11.282
3.875	0.017	0.080	0.139	0.216	0.226	0.261	0.255	11.282
4.125	0.022	0.038	0.091	0.191	0.226	0.228	0.237	11.282
4.375	0.020	0.060	0.071	0.141	0.196	0.213	0.239	11.282
4.625	0.015	0.053	0.088	0.130	0.181	0.161	0.156	11.282
4.875	0.015	0.043	0.058	0.113	0.174	0.142	0.152	11.282
5.125	0.000	0.023	0.063	0.090	0.091	0.164	0.134	11.282
5.375	0.020	0.010	0.068	0.065	0.091	0.101	0.123	11.282
5.625	0.015	0.028	0.055	0.073	0.121	0.116	0.099	11.282
5.875	0.007	0.033	0.048	0.070	0.159	0.106	0.096	11.282
6.125	0.013	0.018	0.050	0.083	0.091	0.106	0.118	11.282
6.375	0.013	0.033	0.076	0.093	0.068	0.120	0.108	11.282
6.625	0.002	0.038	0.083	0.108	0.121	0.121	0.119	11.282
6.875	0.007	0.063	0.086	0.110	0.128	0.174	0.149	11.282
7.125	0.030	0.055	0.113	0.163	0.181	0.218	0.222	11.282
7.375	0.032	0.078	0.176	0.236	0.234	0.308	0.287	11.282
7.625	0.020	0.116	0.202	0.326	0.438	0.407	0.423	11.282
7.875	0.047	0.148	0.318	0.442	0.559	0.628	0.608	11.282
8.125	0.085	0.194	0.423	0.640	0.793	0.811	0.847	11.282
8.375	0.095	0.354	0.728	1.222	1.396	1.464	1.483	11.291
8.625	0.142	0.578	1.800	2.853	3.729	4.241	4.077	11.330
8.875	0.157	0.764	2.190	3.849	4.891	5.071	5.310	11.402
9.125	0.190	0.854	2.483	4.431	5.956	5.982	6.133	11.508
9.375	0.198	0.862	2.586	4.847	6.303	6.557	6.684	11.647
9.625	0.220	0.819	2.656	4.890	6.175	6.793	6.924	11.821
9.875	0.142	0.726	2.561	4.579	6.235	6.677	6.894	12.031
10.125	0.175	0.724	2.450	4.634	5.790	6.189	6.196	12.278
10.375	0.122	0.658	2.064	4.132	5.163	6.068	6.021	12.563
10.625	0.160	0.621	2.125	4.207	5.307	5.728	5.872	12.889
10.875	0.165	0.500	2.036	3.826	5.510	5.620	5.679	13.256
11.125	0.152	0.550	1.953	3.977	5.050	5.295	5.590	13.668
11.375	0.170	0.548	2.162	4.059	5.141	5.549	5.633	14.126
11.625	0.162	0.591	2.029	3.941	5.216	5.511	5.689	14.634
11.875	0.190	0.696	1.951	3.783	5.624	5.609	5.827	15.194
12.125	0.165	0.653	2.173	4.167	5.639	5.859	5.802	15.810
12.375	0.167	0.653	2.185	4.177	5.133	5.562	5.789	16.487
12.625	0.207	0.666	2.188	4.232	5.307	5.844	5.958	17.227
12.875	0.215	0.777	2.309	4.493	5.480	6.049	6.142	18.036
13.125	0.150	0.726	2.120	4.308	5.646	6.159	6.266	18.920
13.375	0.210	0.681	2.316	4.330	5.850	6.281	6.357	19.883
13.625	0.200	0.832	2.561	4.481	6.160	6.339	6.501	20.933
13.875	0.203	0.782	2.503	4.674	6.326	6.438	6.702	22.078
14.125	0.200	0.726	2.505	4.629	6.371	7.015	6.871	23.324

Midpoint	Ratio: 8.6	2.8	0.97	0.34	0.11	0.041	0.009	Volume
14.375	0.200	0.789	2.490	4.975	6.341	6.669	7.080	24.682
14.625	0.202	0.761	2.480	4.905	6.763	6.964	6.834	26.162
14.875	0.197	0.862	2.528	5.176	6.960	7.127	7.300	27.776
15.125	0.235	0.854	2.578	5.246	6.613	7.614	7.448	29.537
15.375	0.190	0.915	2.719	5.386	7.835	8.143	7.865	31.460
15.625	0.287	1.005	3.092	6.024	8.175	8.507	8.541	33.564
15.875	0.257	0.935	3.125	6.285	8.537	9.308	9.478	35.869
16.125	0.312	1.148	3.513	6.874	9.073	9.519	9.872	38.400
16.375	0.337	1.214	3.675	7.396	9.556	10.221	10.562	41.184
16.625	0.340	1.435	4.063	7.792	9.972	10.952	11.393	44.256
16.875	0.360	1.510	4.290	8.106	10.440	11.658	11.904	47.657
17.125	0.447	1.656	4.731	8.773	11.723	12.321	12.660	51.439
17.375	0.505	1.659	4.907	9.571	12.606	13.208	13.496	55.666
17.625	0.470	1.588	5.139	9.717	13.368	14.059	14.398	60.420
17.875	0.510	1.975	5.537	10.341	13.852	14.790	15.024	65.812
18.125	0.555	1.988	5.673	10.858	14.795	15.680	16.150	71.991
18.375	0.655	2.335	6.029	11.174	14.455	15.640	16.183	79.176
18.625	0.907	2.425	5.051	8.026	9.209	9.915	10.227	87.699
18.875	0.915	2.759	5.479	7.996	10.198	10.233	10.434	98.132
19.125	1.067	3.345	6.717	9.712	11.194	12.134	12.684	111.609
19.375	1.312	3.843	7.501	10.891	12.606	13.962	14.226	131.318
19.625	1.592	4.129	8.655	12.677	15.633	16.129	16.486	5642.311
19.875	8.049	22.004	44.469	65.550	78.165	83.958	85.253	1232.996
20.125	12.883	36.271	71.948	106.075	126.295	133.850	136.453	1232.996
20.375	12.820	35.530	71.865	106.462	126.031	134.376	136.960	1232.996
20.625	12.650	35.416	71.582	105.295	123.592	132.142	135.082	1232.996
20.875	12.908	35.653	70.647	104.287	125.102	131.590	134.456	1232.996
21.125	12.843	35.328	69.352	102.872	123.238	131.059	132.732	1232.996
21.375	12.536	34.755	69.214	101.532	123.019	128.322	130.939	1232.996
21.625	12.420	34.851	69.251	100.438	120.263	127.567	130.038	1232.996
21.875	12.250	34.160	69.317	100.553	119.033	126.277	129.297	1232.996
22.125	12.523	34.524	67.409	100.210	117.856	125.151	128.824	1232.996
22.375	12.531	33.803	66.338	98.559	117.561	125.431	126.847	1232.996
22.625	12.441	33.137	66.754	97.616	117.380	123.894	125.785	1232.996
22.875	12.663	33.383	65.418	97.375	114.980	123.196	125.493	1232.996
23.125	12.411	33.677	65.456	97.415	115.991	122.428	124.700	1232.996
23.375	12.378	33.276	66.242	96.868	115.191	122.499	124.673	1232.996
23.625	12.178	33.084	65.680	95.978	113.372	121.259	123.944	1232.996
23.875	12.325	32.675	64.647	96.178	114.527	121.622	123.999	1232.996
24.125	12.248	33.029	65.075	95.386	113.364	121.190	123.024	1232.996
24.375	12.248	32.708	64.420	94.836	112.715	120.222	122.833	1232.996
24.625	12.238	32.841	64.402	94.585	112.964	120.729	122.477	1232.996
24.875	12.320	32.461	64.261	94.543	111.009	119.731	122.316	1232.996
25.125	12.303	32.745	64.031	94.063	111.870	118.683	121.819	1232.996
25.375	12.241	32.836	63.742	94.209	111.688	119.740	121.261	1232.996

Midpoint	Ratio: 8.6	2.8	0.97	0.34	0.11	0.041	0.009	Volume
25.625	12.341	32.838	63.583	93.659	111.975	118.411	121.016	1232.996
25.875	12.006	32.547	63.759	93.185	111.454	118.239	120.293	1232.996
26.125	12.111	32.280	63.003	92.666	113.319	119.105	120.290	1232.996
26.375	12.078	32.290	63.321	92.560	111.975	118.111	120.860	1232.996
26.625	12.038	31.964	63.843	93.132	111.349	118.232	120.807	1232.996
26.875	12.210	32.104	62.159	92.891	111.907	117.652	120.636	1232.996
27.125	12.046	32.069	62.973	92.493	111.832	117.610	119.996	1232.996
27.375	11.993	31.841	63.255	93.376	113.379	117.745	119.766	1232.996
27.625	12.293	32.290	63.938	93.409	109.756	117.642	120.545	1232.996
27.875	12.223	32.383	63.402	92.749	110.299	117.347	120.689	1232.996
28.125	12.078	32.353	62.809	92.430	111.802	117.826	120.722	1232.996
28.375	12.178	32.200	63.737	92.380	110.911	117.867	120.817	1232.996
28.625	12.305	32.250	63.616	92.766	111.756	118.145	120.152	1232.996
28.875	12.013	32.720	63.386	93.426	109.635	118.037	120.628	1232.996
29.125	12.158	32.315	63.586	94.377	110.964	118.531	120.793	1232.996
29.375	12.193	32.519	63.397	93.614	110.858	118.034	120.606	1232.996
29.625	11.946	32.627	63.873	93.697	112.088	117.546	121.541	1232.996
29.875	12.408	32.363	63.954	92.924	111.416	117.846	121.432	1232.996
30.125	12.148	32.579	64.437	94.116	111.975	118.860	121.748	1232.996
30.375	11.848	32.667	64.301	93.835	112.866	119.124	121.084	1232.996
30.625	12.665	32.700	64.059	93.406	112.202	119.202	121.693	1232.996
30.875	12.460	32.501	63.767	94.407	113.122	119.085	121.393	1232.996
31.125	12.228	32.790	64.122	93.647	113.545	118.954	121.117	1232.996
31.375	12.470	32.740	63.810	94.139	112.790	119.146	121.811	1232.996
31.625	12.180	32.662	64.712	94.392	111.658	120.260	121.513	1232.996
31.875	12.178	33.514	64.165	94.685	111.870	119.313	121.995	1232.996
32.125	12.323	32.836	64.050	95.049	112.662	118.958	121.790	1232.996
32.375	12.435	32.899	64.715	93.669	112.224	119.408	121.537	1232.996
32.625	12.361	32.562	64.873	95.506	113.145	119.254	122.476	1232.996
32.875	12.188	33.124	64.127	94.843	112.247	120.003	122.509	1232.996
33.125	12.575	32.682	64.223	94.946	113.892	119.152	122.452	1232.996
33.375	12.336	33.007	63.757	93.900	114.142	120.397	122.562	1232.996
33.625	12.571	33.190	64.042	94.277	112.239	118.839	122.316	1232.996
33.875	12.413	33.170	64.248	95.024	113.462	119.868	122.431	1232.996
34.125	11.965	33.027	64.279	94.462	113.794	120.338	122.048	1232.996
34.375	12.358	33.155	64.838	95.127	111.560	120.024	121.432	1232.996
34.625	12.308	32.677	64.072	94.801	112.088	119.636	122.481	1232.996
34.875	12.371	32.642	63.515	94.605	112.488	120.354	121.658	1232.996
35.125	12.541	32.710	64.216	94.979	111.515	120.330	122.882	1232.996
35.375	12.513	32.790	63.606	94.010	112.768	120.226	122.655	1232.996
35.625	12.421	33.409	64.178	94.899	112.300	119.737	122.195	1232.996
35.875	12.545	33.215	64.397	94.001	113.258	119.111	121.857	1232.996
36.125	12.361	32.713	64.059	94.309	112.383	120.171	123.102	1232.996
36.375	12.545	33.075	65.083	94.068	112.941	120.743	121.415	1232.996
36.625	12.410	33.441	64.175	94.364	111.703	119.535	123.100	1232.996

Midpoint	Ratio: 8.6	2.8	0.97	0.34	0.11	0.041	0.009	Volume
36.875	12.465	32.976	64.399	95.172	112.783	119.675	122.444	1232.996
37.125	12.205	33.049	64.750	95.373	113.500	119.789	121.402	1232.996
37.375	12.488	33.064	65.153	94.876	113.394	120.678	121.519	1232.996
37.625	12.135	32.418	64.080	94.459	114.330	119.148	122.388	1232.996
37.875	12.193	32.886	64.866	94.367	112.224	120.165	122.114	1232.996
38.125	12.223	32.544	64.102	93.975	114.323	119.615	121.763	1232.996
38.375	12.763	33.188	64.833	94.593	113.870	120.009	122.235	1232.996
38.625	12.473	33.562	63.447	94.395	112.473	119.223	121.538	1232.996
38.875	12.243	32.280	65.130	94.083	115.621	119.111	122.915	1232.996
39.125	12.243	32.805	64.077	94.555	112.028	119.576	122.539	1232.996
39.375	12.293	32.991	64.367	94.829	112.511	119.124	123.198	1232.996
39.625	12.573	33.084	64.213	94.906	112.956	119.759	122.486	1232.996
39.875	12.611	32.607	64.034	94.888	113.122	120.038	122.187	1232.996
40.125	12.470	33.004	64.654	93.519	112.488	120.077	121.279	1232.996
40.375	12.425	32.582	64.715	94.560	113.470	118.694	122.714	1232.996
40.625	12.238	32.180	63.928	93.426	113.024	119.391	121.544	1232.996
40.875	12.505	32.831	64.228	94.314	113.500	118.794	122.028	1232.996
41.125	12.458	32.546	63.706	93.900	112.322	119.176	120.911	1232.996
41.375	12.515	33.215	63.926	94.625	111.303	119.441	122.835	1232.996
41.625	12.443	33.114	64.397	93.752	110.835	119.378	120.911	1232.996
41.875	12.538	32.431	63.195	93.609	113.183	118.344	121.118	1232.996
42.125	12.088	32.710	63.580	93.436	113.825	118.934	122.573	1232.996
42.375	12.465	33.032	64.163	93.491	110.926	118.172	121.491	1232.996
42.625	12.288	32.534	63.827	94.632	111.017	118.086	121.403	1232.996
42.875	12.135	32.496	63.724	92.703	111.802	119.009	121.796	1232.996
43.125	12.206	31.916	63.646	94.001	112.073	119.439	122.084	1232.996
43.375	12.133	32.667	63.669	93.534	111.545	118.330	121.281	1232.996
43.625	12.293	32.130	63.797	94.369	112.549	118.162	121.016	1232.996
43.875	12.265	32.424	63.334	93.162	113.115	118.359	120.203	1232.996
44.125	12.331	32.298	63.613	93.436	111.258	117.458	120.575	1232.996
44.375	12.255	32.401	63.394	93.283	111.620	117.891	120.815	1232.996
44.625	11.938	32.092	63.291	92.789	111.364	117.509	120.131	1232.996
44.875	11.996	31.775	63.021	93.072	109.733	117.731	120.288	1232.996
45.125	12.275	32.142	62.852	92.106	110.277	118.027	119.393	1232.996
45.375	11.888	31.750	63.195	93.637	110.458	116.694	119.945	1232.996
45.625	12.175	32.242	63.011	93.037	109.205	116.660	119.584	1232.996
45.875	11.790	32.288	62.797	92.746	109.605	116.968	118.866	1232.996
46.125	11.990	31.881	62.005	91.186	108.926	116.581	118.947	1232.996
46.375	11.976	32.129	63.003	92.548	110.699	116.129	119.416	1232.996
46.625	12.215	31.886	61.635	91.612	110.103	116.568	118.624	1232.996
46.875	11.891	31.936	62.151	92.159	108.035	116.007	118.594	1232.996
47.125	11.880	31.976	62.459	91.565	108.933	115.532	118.664	1232.996
47.375	11.931	31.099	62.086	91.085	109.801	115.643	118.546	1232.996
47.625	11.893	31.567	61.584	90.716	108.661	115.239	117.055	1232.996
47.875	12.001	31.263	61.779	91.158	107.891	114.805	117.310	1232.996

Midpoint	Ratio: 8.6	2.8	0.97	0.34	0.11	0.041	0.009	Volume
48.125	11.891	31.308	61.768	90.870	106.449	115.122	116.831	1232.996
48.375	11.908	31.059	61.801	90.722	109.175	114.802	117.494	1232.996
48.625	11.595	31.323	61.025	89.954	108.178	113.706	117.015	1232.996
48.875	11.703	31.200	61.622	89.598	106.933	112.884	116.432	1232.996
49.125	11.663	30.795	60.707	89.146	107.016	113.679	116.486	1232.996
49.375	11.853	30.649	59.732	90.277	105.966	113.224	115.363	1232.996
49.625	11.765	31.373	60.468	89.166	107.408	112.596	116.529	1232.996
49.875	11.738	30.531	60.385	89.650	106.661	112.619	114.390	1232.996
50.125	11.673	30.843	59.916	87.879	106.578	111.252	115.328	1232.996
50.375	11.305	31.051	60.357	88.431	104.970	112.096	114.992	1232.996
50.625	11.578	30.596	60.337	87.049	105.906	111.840	113.624	1232.996
50.875	11.661	30.561	60.034	88.890	105.000	111.347	114.610	1232.996
51.125	11.433	30.177	59.785	87.721	104.948	111.849	113.192	1232.996
51.375	11.463	30.476	59.611	88.466	104.925	111.263	113.798	1232.996
51.625	11.511	29.943	59.326	87.656	106.042	111.428	113.770	1232.996
51.875	11.273	30.484	59.664	87.808	105.695	110.124	114.304	1232.996
52.125	11.496	29.714	59.316	87.134	105.121	109.790	113.131	1232.996
52.375	11.373	29.697	59.132	87.242	104.661	111.209	113.488	1232.996
52.625	11.391	29.727	58.381	87.809	104.827	111.275	113.483	1232.996
52.875	11.308	30.041	58.636	87.601	102.124	110.723	112.575	1232.996
53.125	11.338	30.056	58.316	87.262	104.713	110.665	113.437	1232.996
53.375	11.456	30.144	60.138	87.001	105.272	110.389	113.505	1232.996
53.625	11.295	30.355	59.016	86.497	103.302	110.636	113.318	1232.996
53.875	11.053	29.400	59.152	86.582	105.936	110.567	113.676	1232.996
54.125	11.301	30.101	59.135	87.352	105.453	110.595	112.428	1232.996
54.375	11.223	30.340	58.542	86.639	103.468	110.434	113.435	1232.996
54.625	11.721	29.586	58.431	87.349	104.955	110.534	113.517	1232.996
54.875	11.473	30.561	58.963	86.695	104.170	110.636	113.142	1232.996
55.125	11.291	29.845	59.485	85.764	104.291	111.090	113.102	1232.996
55.375	11.168	29.903	59.218	86.981	104.449	111.123	113.578	1232.996
55.625	11.396	29.812	58.777	87.006	103.075	111.064	113.112	1232.996
55.875	11.323	30.056	58.956	87.879	103.491	110.016	113.618	1232.996
56.125	11.448	30.066	58.590	86.587	105.672	111.295	114.151	1232.996
56.375	11.466	29.549	59.384	87.969	103.611	110.871	113.611	1232.996
56.625	11.443	30.207	58.389	86.775	103.128	110.929	113.908	1232.996
56.875	11.321	29.740	59.112	86.148	104.495	110.427	113.709	1232.996
57.125	11.633	29.755	59.218	87.721	104.524	110.016	113.629	1232.996
57.375	11.188	29.780	59.150	87.886	104.729	110.722	114.402	1232.996
57.625	11.173	30.403	58.553	87.641	103.989	110.733	113.155	1232.996
57.875	11.293	30.099	58.900	86.848	105.393	110.641	113.210	1232.996
58.125	3.302	8.625	17.002	25.324	30.511	31.342	32.810	1232.996
58.375	0.000	0.000	0.000	0.000	0.000	0.000	0.000	1232.996
58.625	0.000	0.000	0.000	0.000	0.000	0.000	0.000	1232.996

Midpoint	Ratio: 110	25	9.1	2.9	1	0.35	0.11	Volume

E.2. KCSA channel: Potassium and Ammonium

Table of NH_4^+ population data for simulation with K^+ and NH_4^+ across bulk density ratios (#JJF-GRS1459)

	Population in histogram bin at NH_4^+/K^+ ratio:							
Midpoint	110	25	9.1	2.9	1	0.35	0.11	Volume
-58.375	0.000	0.000	0.000	0.000	0.000	0.000	0.000	1234.242
-58.125	41.010	41.949	39.052	33.399	22.258	11.178	4.406	1234.242
-57.875	115.785	114.562	107.656	88.733	60.746	30.245	11.734	1234.242
-57.625	115.231	113.818	105.936	88.614	59.829	31.747	11.100	1234.242
-57.375	114.987	112.143	107.527	87.931	59.686	30.266	11.447	1234.242
-57.125	115.116	113.112	107.043	88.674	60.470	30.054	11.188	1234.242
-56.875	114.556	113.390	107.312	88.986	59.417	30.473	10.967	1234.242
-56.625	114.211	112.932	105.796	87.224	59.647	30.404	11.395	1234.242
-56.375	115.073	113.202	106.226	89.233	60.022	30.399	11.270	1234.242
-56.125	115.886	111.549	106.506	88.431	59.507	30.510	11.425	1234.242
-55.875	114.642	110.941	106.731	90.284	59.175	30.017	11.484	1234.242
-55.625	116.885	113.743	107.328	89.197	59.746	30.728	11.092	1234.242
-55.375	114.807	112.323	106.189	88.632	60.085	30.091	11.771	1234.242
-55.125	115.526	113.443	105.753	87.628	59.524	30.675	11.255	1234.242
-54.875	115.339	111.850	104.345	88.435	59.676	30.235	11.749	1234.242
-54.625	115.519	111.572	104.936	88.288	59.301	30.441	10.960	1234.242
-54.375	113.053	113.653	107.021	89.160	60.155	30.617	11.579	1234.242
-54.125	113.722	113.518	104.910	90.018	60.749	30.898	11.100	1234.242
-53.875	113.614	110.490	104.206	87.307	59.361	30.495	11.159	1234.242
-53.625	115.770	110.324	105.952	88.811	59.593	30.357	11.461	1234.242
-53.375	113.937	112.526	105.839	88.554	59.829	31.232	11.498	1234.242
-53.125	115.174	111.474	104.657	88.853	59.178	29.534	11.853	1234.242
-52.875	114.592	111.136	107.107	88.623	59.962	30.288	11.373	1234.242
-52.625	113.017	111.729	107.064	87.692	59.813	30.972	11.749	1234.242
-52.375	113.923	112.917	106.543	87.921	60.361	30.914	11.535	1234.242
-52.125	113.743	113.781	104.668	88.839	59.587	29.879	11.151	1234.242
-51.875	114.052	112.676	106.425	88.940	60.002	30.410	11.675	1234.242
-51.625	114.261	112.203	107.382	89.527	60.623	30.171	11.550	1234.242
-51.375	114.606	113.150	107.548	89.513	60.540	30.473	11.734	1234.242
-51.125	115.440	111.113	105.237	87.550	60.121	31.062	11.292	1234.242
-50.875	114.167	113.991	107.145	90.054	59.906	31.057	11.675	1234.242
-50.625	115.677	112.714	106.017	89.311	60.620	31.174	11.875	1234.242
-50.375	114.836	111.947	106.817	89.431	59.583	30.802	11.520	1234.242
-50.125	116.238	112.894	108.354	89.843	61.005	30.585	11.838	1234.242
-49.875	116.461	112.143	107.554	90.041	60.361	31.709	12.303	1234.242
-49.625	117.776	112.789	107.124	91.435	60.696	30.388	11.491	1234.242
-49.375	116.763	114.247	108.650	90.229	61.742	30.701	11.587	1234.242
-49.125	116.259	114.900	107.328	90.573	60.955	31.874	11.609	1234.242

Midpoint	Ratio: 110	25	9.1	2.9	1	0.35	0.11	Volume
-48.875	117.941	116.148	107.623	90.513	61.476	32.320	11.070	1234.242
-48.625	116.195	116.185	109.144	91.385	61.413	31.365	11.808	1234.242
-48.375	116.899	116.659	110.267	91.775	61.935	32.028	11.395	1234.242
-48.125	119.904	116.245	108.790	91.431	61.995	31.216	12.273	1234.242
-47.875	119.408	115.832	108.800	91.687	61.726	31.253	12.192	1234.242
-47.625	117.934	115.652	109.467	92.087	62.174	31.922	12.214	1234.242
-47.375	118.064	115.659	109.891	91.490	62.679	31.412	11.565	1234.242
-47.125	119.013	116.381	111.116	92.137	62.386	31.752	12.140	1234.242
-46.875	117.812	116.741	111.487	92.353	62.543	31.996	11.934	1234.242
-46.625	119.825	117.297	111.079	92.119	62.649	32.516	11.794	1234.242
-46.375	120.810	118.342	111.251	94.642	62.928	32.282	12.554	1234.242
-46.125	120.350	117.410	110.751	93.889	63.260	32.335	12.074	1234.242
-45.875	120.040	118.154	111.133	93.637	63.313	32.330	12.347	1234.242
-45.625	121.169	118.214	111.751	92.857	64.077	31.853	11.572	1234.242
-45.375	120.127	119.236	111.133	93.495	62.941	32.526	11.971	1234.242
-45.125	120.903	117.560	112.170	93.334	63.403	32.028	11.963	1234.242
-44.875	122.607	117.380	112.455	93.554	63.655	32.457	12.118	1234.242
-44.625	121.967	118.327	111.982	93.499	63.758	32.065	11.816	1234.242
-44.375	122.096	117.966	111.697	93.908	63.675	32.606	11.949	1234.242
-44.125	120.723	117.756	112.868	94.380	63.137	32.611	12.517	1234.242
-43.875	121.140	119.799	114.357	93.788	64.193	32.489	11.912	1234.242
-43.625	121.457	119.348	112.793	94.330	63.801	32.261	12.502	1234.242
-43.375	123.031	120.581	112.094	95.293	64.007	32.564	12.140	1234.242
-43.125	120.184	118.943	113.390	94.215	63.187	33.402	12.081	1234.242
-42.875	122.513	118.890	112.514	94.325	64.721	33.094	12.679	1234.242
-42.625	122.873	119.935	113.341	94.201	64.127	32.447	12.502	1234.242
-42.375	121.349	120.137	114.163	95.100	64.392	32.548	12.266	1234.242
-42.125	122.312	120.288	111.718	94.710	63.818	32.139	12.037	1234.242
-41.875	121.773	119.852	114.765	96.128	64.499	33.073	12.148	1234.242
-41.625	122.183	119.161	114.158	95.334	64.744	32.622	12.635	1234.242
-41.375	121.794	122.211	114.368	95.201	64.074	32.479	12.133	1234.242
-41.125	124.699	120.258	113.513	94.646	64.658	33.099	12.657	1234.242
-40.875	123.599	119.311	113.363	94.949	63.705	33.163	12.258	1234.242
-40.625	122.147	120.979	113.298	95.770	64.505	32.044	12.775	1234.242
-40.375	122.751	119.401	113.148	96.109	63.937	33.312	12.642	1234.242
-40.125	123.491	120.190	114.303	95.834	64.744	33.731	12.495	1234.242
-39.875	122.736	119.904	113.148	95.132	64.957	32.611	12.428	1234.242
-39.625	123.764	121.122	114.266	96.109	65.286	32.426	12.229	1234.242
-39.375	121.636	119.844	114.550	94.866	64.622	32.537	12.642	1234.242
-39.125	123.052	119.243	115.002	95.871	65.548	32.579	12.871	1234.242
-38.875	122.786	121.400	114.384	95.353	65.179	32.622	12.413	1234.242
-38.625	121.751	120.340	114.927	94.068	64.821	33.184	12.539	1234.242
-38.375	122.822	121.272	114.282	95.348	64.990	32.935	12.495	1234.242
-38.125	120.702	120.460	114.765	95.114	65.067	32.373	12.436	1234.242
-37.875	123.160	120.521	113.637	94.990	64.040	33.418	12.318	1234.242

Midpoint	Ratio: 110	25	9.1	2.9	1	0.35	0.11	Volume
-37.625	124.073	120.393	114.550	95.876	65.455	33.725	12.650	1234.242
-37.375	123.232	119.754	115.690	96.284	65.179	32.930	12.532	1234.242
-37.125	121.708	120.130	114.389	95.220	65.256	33.900	12.074	1234.242
-36.875	122.398	120.528	115.066	94.880	64.748	33.221	12.480	1234.242
-36.625	123.728	120.926	113.583	94.811	65.033	33.014	12.694	1234.242
-36.375	121.766	119.641	115.093	95.820	65.963	33.343	12.532	1234.242
-36.125	122.887	122.159	113.734	96.366	64.881	32.808	12.605	1234.242
-35.875	124.174	120.213	113.959	96.710	65.292	32.744	12.236	1234.242
-35.625	122.183	122.256	113.685	95.981	64.781	33.853	12.000	1234.242
-35.375	124.203	119.348	113.244	95.976	64.847	33.163	12.436	1234.242
-35.125	124.023	122.038	114.008	95.655	64.940	32.442	12.694	1234.242
-34.875	123.239	121.903	114.497	95.302	64.568	33.105	12.627	1234.242
-34.625	122.053	121.047	114.878	95.270	65.140	32.415	12.605	1234.242
-34.375	121.823	119.889	116.469	94.917	66.136	32.877	13.004	1234.242
-34.125	121.845	122.076	114.674	95.270	64.844	32.510	12.052	1234.242
-33.875	123.548	119.867	113.486	96.476	64.688	32.633	12.436	1234.242
-33.625	119.976	120.859	114.352	95.541	64.974	33.603	12.369	1234.242
-33.375	123.347	120.513	114.787	96.123	65.595	32.834	12.487	1234.242
-33.125	124.483	121.249	113.556	95.536	64.555	32.505	13.033	1234.242
-32.875	123.261	120.303	115.270	96.600	65.263	32.144	11.734	1234.242
-32.625	122.520	121.227	114.400	95.724	64.353	32.935	12.369	1234.242
-32.375	123.980	119.288	114.018	96.912	64.539	33.349	12.561	1234.242
-32.125	120.745	119.409	113.803	95.885	64.064	33.768	12.450	1234.242
-31.875	123.369	121.625	113.648	95.747	64.622	32.728	12.797	1234.242
-31.625	122.449	119.950	114.174	96.045	64.917	32.627	12.022	1234.242
-31.375	123.520	120.648	112.530	95.105	64.147	32.739	12.089	1234.242
-31.125	123.218	120.663	113.583	95.192	64.442	33.030	12.746	1234.242
-30.875	122.427	121.107	113.524	95.229	64.907	32.808	12.864	1234.242
-30.625	121.399	119.769	114.518	95.206	64.183	32.362	12.472	1234.242
-30.375	121.557	120.092	114.513	95.105	64.648	32.765	12.288	1234.242
-30.125	122.873	118.875	113.191	94.220	63.446	32.792	12.399	1234.242
-29.875	122.319	120.378	113.540	94.224	64.688	32.924	12.207	1234.242
-29.625	123.469	119.521	113.153	93.660	64.279	32.946	12.310	1234.242
-29.375	121.730	120.182	112.954	94.820	63.731	32.272	12.576	1234.242
-29.125	120.414	120.340	112.390	94.843	64.117	32.755	12.044	1234.242
-28.875	120.795	119.206	113.030	94.431	64.578	32.712	11.299	1234.242
-28.625	120.637	119.762	112.712	94.802	64.150	33.365	11.934	1234.242
-28.375	120.493	120.513	111.718	95.119	63.748	33.142	11.801	1234.242
-28.125	122.190	118.221	113.594	94.142	63.629	32.723	12.347	1234.242
-27.875	121.787	119.333	112.261	94.320	63.492	32.866	12.377	1234.242
-27.625	120.680	118.898	112.868	95.018	63.934	32.648	12.532	1234.242
-27.375	122.118	118.394	111.401	94.353	63.821	32.930	12.325	1234.242
-27.125	120.565	120.250	112.879	94.843	63.765	32.351	12.465	1234.242
-26.875	122.060	120.235	112.481	95.169	64.200	32.664	11.882	1234.242
-26.625	122.722	119.927	112.729	93.986	64.638	32.102	12.096	1234.242

Midpoint	Ratio: 110	25	9.1	2.9	1	0.35	0.11	Volume
-26.375	121.356	120.934	112.987	94.628	64.319	32.542	12.318	1234.242
-26.125	121.406	119.747	113.975	95.357	63.639	32.548	12.030	1234.242
-25.875	122.456	119.469	113.997	95.825	65.106	32.802	11.993	1234.242
-25.625	123.239	119.589	112.729	95.128	65.233	33.492	12.192	1234.242
-25.375	121.097	121.918	113.927	95.706	64.744	32.351	12.664	1234.242
-25.125	123.081	119.950	115.335	95.307	65.027	33.518	12.332	1234.242
-24.875	123.096	121.896	115.222	95.155	65.053	33.285	12.502	1234.242
-24.625	123.124	121.978	114.964	95.880	66.432	32.999	12.229	1234.242
-24.375	125.568	122.993	114.996	96.435	65.900	33.778	12.687	1234.242
-24.125	123.469	123.436	115.485	95.568	65.017	33.646	12.605	1234.242
-23.875	123.721	122.902	116.200	97.775	65.784	34.007	12.436	1234.242
-23.625	124.217	125.134	116.781	99.137	66.312	34.145	12.340	1234.242
-23.375	126.014	123.984	117.920	99.554	66.790	34.118	12.974	1234.242
-23.125	125.597	123.226	116.979	98.848	66.465	34.251	12.281	1234.242
-22.875	126.122	124.849	120.241	100.151	67.654	34.399	12.576	1234.242
-22.625	128.595	125.547	117.984	100.275	69.480	35.206	12.650	1234.242
-22.375	129.019	126.441	120.021	101.054	69.205	34.824	13.004	1234.242
-22.125	128.961	128.252	120.413	100.403	69.553	34.712	13.151	1234.242
-21.875	131.405	129.898	121.123	100.972	69.942	35.036	12.930	1234.242
-21.625	134.044	129.913	123.455	103.917	69.736	35.980	12.709	1234.242
-21.375	133.447	130.461	124.583	104.109	71.563	35.412	13.550	1234.242
-21.125	134.935	132.513	125.707	104.426	71.805	36.288	12.746	1234.242
-20.875	135.855	133.377	125.148	106.224	72.552	36.680	12.915	1234.242
-20.625	136.294	135.526	128.748	107.210	73.074	36.887	13.137	1234.242
-20.375	138.709	134.646	128.694	107.967	74.120	36.797	13.019	1234.242
-20.125	114.894	113.337	108.628	90.449	60.995	31.502	10.635	1234.242
-19.875	17.691	17.206	16.143	13.362	9.419	4.828	1.535	6066.954
-19.625	15.311	13.991	13.660	11.486	7.987	3.963	1.306	137.850
-19.375	13.061	12.698	11.828	10.289	6.815	4.016	1.328	115.056
-19.125	11.516	10.805	10.517	8.858	6.197	2.897	1.181	100.438
-18.875	8.914	9.099	8.786	7.298	5.008	2.801	0.775	89.348
-18.625	9.784	9.903	9.609	7.734	4.982	2.308	0.871	80.382
-18.375	16.900	16.913	15.864	11.362	6.131	2.345	0.539	72.874
-18.125	16.936	17.184	15.762	12.344	6.257	2.048	0.539	66.445
-17.875	16.232	15.899	15.493	11.119	5.848	1.873	0.524	60.855
-17.625	14.765	14.381	13.575	10.729	5.407	1.809	0.546	55.939
-17.375	14.262	13.532	13.016	9.771	5.144	1.613	0.428	51.577
-17.125	13.119	13.187	12.075	9.349	4.653	1.809	0.443	47.681
-16.875	12.105	12.338	11.253	8.651	4.520	1.422	0.472	44.182
-16.625	11.566	11.721	10.361	8.261	4.151	1.390	0.428	41.026
-16.375	10.675	10.091	10.296	7.716	4.168	1.310	0.546	38.169
-16.125	10.244	10.331	9.168	6.899	3.749	1.125	0.295	35.574
-15.875	9.532	9.557	9.028	6.743	3.444	1.050	0.244	33.213
-15.625	8.770	8.716	8.346	6.303	3.105	0.923	0.288	31.058
-15.375	8.468	8.814	7.540	5.931	3.016	0.950	0.266	29.090

Midpoint	Ratio: 110	25	9.1	2.9	1	0.35	0.11	Volume
-15.125	8.267	7.784	7.593	5.463	2.979	0.902	0.185	27.289
-14.875	7.958	7.626	7.357	5.518	2.690	0.812	0.192	25.639
-14.625	7.864	7.611	6.868	5.289	2.680	0.801	0.185	24.127
-14.375	7.081	6.950	6.395	5.225	2.670	0.822	0.236	22.739
-14.125	7.081	7.288	6.465	5.018	2.537	0.711	0.251	21.465
-13.875	6.664	7.085	5.944	4.995	2.481	0.859	0.185	20.296
-13.625	6.807	6.860	6.304	4.743	2.683	0.859	0.162	19.223
-13.375	6.757	6.402	5.960	4.739	2.461	0.785	0.221	18.237
-13.125	6.369	6.056	5.777	4.573	2.278	0.844	0.266	17.333
-12.875	5.808	6.334	5.616	3.963	2.361	0.780	0.162	16.505
-12.625	6.117	6.161	5.616	4.298	2.225	0.679	0.170	15.746
-12.375	5.916	6.176	5.938	4.413	2.255	0.668	0.140	15.052
-12.125	6.196	5.891	5.648	4.239	2.238	0.674	0.192	14.418
-11.875	5.470	5.673	5.578	4.096	2.222	0.653	0.140	13.840
-11.625	5.377	6.094	5.266	4.174	2.042	0.536	0.199	13.315
-11.375	6.247	5.831	5.369	4.032	2.169	0.653	0.125	12.840
-11.125	6.038	5.936	5.481	4.436	2.066	0.599	0.111	12.411
-10.875	6.441	5.741	5.809	4.124	1.916	0.642	0.133	12.025
-10.625	6.153	5.455	5.519	4.523	2.205	0.674	0.155	11.681
-10.375	6.283	6.747	6.341	4.798	2.507	0.732	0.118	11.377
-10.125	6.657	6.590	6.411	4.950	2.557	0.727	0.199	11.110
-9.875	7.045	7.251	6.567	5.142	2.634	0.790	0.148	10.879
-9.625	7.289	6.755	6.287	5.073	2.753	0.769	0.273	10.683
-9.375	6.807	6.537	6.228	4.931	2.673	0.875	0.162	10.520
-9.125	6.427	5.816	5.637	4.537	2.481	0.722	0.125	10.390
-8.875	5.154	5.072	5.105	3.885	2.255	0.817	0.185	10.292
-8.625	4.371	4.185	4.025	2.950	1.807	0.626	0.177	10.224
-8.375	1.574	1.465	1.403	1.124	0.804	0.334	0.074	10.187
-8.125	0.748	0.781	0.725	0.619	0.408	0.180	0.089	10.179
-7.875	0.604	0.518	0.494	0.394	0.299	0.133	0.044	10.179
-7.625	0.273	0.361	0.403	0.289	0.186	0.074	0.007	10.179
-7.375	0.273	0.233	0.301	0.156	0.136	0.101	0.000	10.179
-7.125	0.187	0.150	0.188	0.165	0.066	0.053	0.007	10.179
-6.875	0.086	0.128	0.081	0.096	0.056	0.021	0.015	10.179
-6.625	0.050	0.090	0.091	0.050	0.027	0.011	0.000	10.179
-6.375	0.058	0.045	0.032	0.050	0.037	0.011	0.000	10.179
-6.125	0.014	0.030	0.027	0.023	0.020	0.000	0.007	10.179
-5.875	0.014	0.000	0.032	0.018	0.007	0.005	0.000	10.179
-5.625	0.007	0.023	0.016	0.009	0.007	0.000	0.000	10.179
-5.375	0.014	0.015	0.011	0.009	0.007	0.011	0.000	10.179
-5.125	0.029	0.008	0.032	0.018	0.003	0.000	0.015	10.179
-4.875	0.014	0.008	0.005	0.018	0.003	0.005	0.000	10.179
-4.625	0.014	0.015	0.005	0.014	0.003	0.000	0.000	10.179
-4.375	0.007	0.015	0.005	0.009	0.010	0.005	0.000	10.179
-4.125	0.022	0.000	0.016	0.018	0.007	0.011	0.000	10.179

Midpoint	Ratio: 110	25	9.1	2.9	1	0.35	0.11	Volume
-3.875	0.022	0.038	0.016	0.005	0.013	0.011	0.000	10.179
-3.625	0.007	0.008	0.011	0.023	0.020	0.021	0.000	10.179
-3.375	0.058	0.023	0.043	0.028	0.010	0.016	0.000	10.179
-3.125	0.022	0.023	0.043	0.055	0.027	0.000	0.007	10.179
-2.875	0.065	0.090	0.075	0.069	0.030	0.037	0.000	10.179
-2.625	0.079	0.105	0.102	0.060	0.037	0.011	0.015	10.179
-2.375	0.115	0.075	0.097	0.087	0.056	0.037	0.007	10.179
-2.125	0.158	0.158	0.113	0.050	0.083	0.037	0.022	10.179
-1.875	0.158	0.188	0.172	0.106	0.073	0.016	0.022	10.179
-1.625	0.165	0.188	0.134	0.115	0.083	0.069	0.022	10.179
-1.375	0.158	0.150	0.156	0.142	0.056	0.032	0.015	10.179
-1.125	0.180	0.143	0.210	0.124	0.080	0.032	0.015	10.179
-0.875	0.122	0.180	0.118	0.110	0.060	0.064	0.022	10.179
-0.625	0.180	0.188	0.134	0.133	0.076	0.021	0.000	10.179
-0.125	0.122	0.173	0.124	0.087	0.063	0.037	0.007	10.179
0.125	0.093	0.165	0.118	0.073	0.060	0.027	0.007	10.179
0.625	0.122	0.105	0.043	0.073	0.063	0.037	0.015	10.179
0.875	0.108	0.120	0.102	0.064	0.056	0.032	0.015	10.179
1.125	0.050	0.083	0.054	0.018	0.050	0.032	0.015	10.179
1.375	0.093	0.113	0.064	0.046	0.043	0.027	0.007	10.179
1.625	0.093	0.053	0.070	0.060	0.033	0.011	0.007	10.179
1.875	0.065	0.090	0.064	0.060	0.017	0.011	0.000	10.179
2.125	0.086	0.083	0.054	0.046	0.027	0.027	0.015	10.179
2.375	0.043	0.045	0.129	0.064	0.037	0.011	0.022	10.179
2.625	0.050	0.113	0.097	0.046	0.030	0.000	0.000	10.179
2.875	0.086	0.105	0.054	0.060	0.050	0.042	0.000	10.179
3.125	0.065	0.068	0.097	0.060	0.046	0.027	0.015	10.179
3.375	0.065	0.090	0.059	0.041	0.040	0.016	0.007	10.179
3.625	0.115	0.083	0.075	0.055	0.043	0.005	0.015	10.179
3.875	0.093	0.060	0.043	0.055	0.020	0.021	0.007	10.179
4.125	0.093	0.083	0.043	0.050	0.033	0.016	0.000	10.179
4.375	0.050	0.038	0.048	0.046	0.037	0.011	0.015	10.179
4.625	0.022	0.023	0.032	0.032	0.020	0.016	0.000	10.179
4.875	0.029	0.023	0.016	0.028	0.033	0.016	0.000	10.179
5.125	0.036	0.008	0.016	0.037	0.023	0.000	0.000	10.179
5.375	0.029	0.015	0.016	0.018	0.010	0.011	0.000	10.179
5.625	0.022	0.030	0.021	0.014	0.003	0.011	0.000	10.179
5.875	0.036	0.023	0.011	0.018	0.010	0.016	0.000	10.179
6.125	0.014	0.008	0.027	0.000	0.030	0.000	0.000	10.179
6.375	0.029	0.015	0.021	0.037	0.027	0.000	0.000	10.179
6.625	0.036	0.015	0.054	0.037	0.037	0.000	0.000	10.179
6.875	0.036	0.105	0.091	0.050	0.020	0.027	0.007	10.179
7.125	0.079	0.090	0.043	0.087	0.033	0.048	0.007	10.179
7.375	0.137	0.180	0.129	0.060	0.083	0.027	0.000	10.179
7.625	0.244	0.218	0.220	0.133	0.110	0.058	0.030	10.179

Midpoint	Ratio: 110	25	9.1	2.9	1	0.35	0.11	Volume
7.875	0.316	0.346	0.317	0.257	0.199	0.143	0.044	10.179
8.125	0.561	0.533	0.382	0.381	0.256	0.058	0.030	10.179
8.375	0.978	0.954	1.010	0.794	0.452	0.196	0.096	10.187
8.625	3.177	3.171	2.945	2.326	1.299	0.509	0.111	10.224
8.875	4.227	4.208	3.601	3.046	1.780	0.605	0.140	10.292
9.125	5.025	4.831	4.863	3.872	1.986	0.695	0.185	10.390
9.375	6.117	5.741	5.815	4.862	2.444	0.769	0.221	10.520
9.625	6.570	6.695	6.185	4.872	2.474	0.934	0.170	10.683
9.875	7.354	6.454	6.325	4.812	2.627	0.812	0.199	10.879
10.125	6.685	6.980	6.438	4.661	2.404	0.780	0.199	11.110
10.375	6.520	6.297	5.874	4.541	2.385	0.780	0.096	11.377
10.625	6.196	6.199	5.874	4.537	2.212	0.642	0.185	11.681
10.875	6.053	6.064	5.895	4.303	2.159	0.626	0.096	12.025
11.125	6.067	5.996	5.320	4.427	2.086	0.525	0.140	12.411
11.375	6.139	5.748	5.605	4.156	2.142	0.626	0.133	12.840
11.625	5.873	5.801	5.374	3.991	2.009	0.642	0.155	13.315
11.875	5.959	5.387	5.487	4.385	2.149	0.631	0.133	13.840
12.125	5.859	6.064	5.723	4.216	2.199	0.615	0.170	14.418
12.375	6.211	5.808	5.412	4.399	2.079	0.716	0.162	15.052
12.625	6.283	5.823	5.675	4.248	2.242	0.668	0.133	15.746
12.875	6.211	5.928	6.013	4.491	2.448	0.737	0.207	16.505
13.125	5.981	6.274	5.858	4.761	2.561	0.690	0.207	17.333
13.375	7.038	6.725	6.056	4.601	2.487	0.822	0.192	18.237
13.625	6.556	6.755	6.481	4.422	2.634	0.716	0.177	19.223
13.875	7.188	6.725	6.250	4.702	2.673	0.822	0.177	20.296
14.125	7.440	7.258	6.562	4.908	2.627	0.790	0.236	21.465
14.375	7.268	7.559	6.954	5.018	2.727	0.785	0.170	22.739
14.625	7.706	7.619	6.809	5.060	2.697	0.769	0.185	24.127
14.875	7.670	8.047	6.932	5.619	2.959	0.849	0.229	25.639
15.125	8.195	7.942	7.282	5.505	2.909	0.801	0.192	27.289
15.375	8.691	8.566	8.190	5.922	3.072	0.897	0.221	29.090
15.625	9.043	8.881	8.217	6.229	3.258	1.088	0.273	31.058
15.875	9.597	9.911	8.878	6.605	3.484	0.971	0.266	33.213
16.125	9.776	10.346	9.254	6.913	3.603	1.061	0.280	35.574
16.375	10.768	10.850	10.060	7.670	3.935	1.215	0.325	38.169
16.625	11.085	10.933	10.522	8.101	4.171	1.618	0.384	41.026
16.875	12.177	12.202	11.371	8.541	4.473	1.496	0.421	44.182
17.125	13.241	12.706	12.129	9.128	5.214	1.501	0.546	47.681
17.375	13.565	13.660	12.849	9.904	5.184	1.623	0.494	51.577
17.625	15.153	14.336	13.870	10.417	5.656	1.692	0.399	55.939
17.875	16.454	15.847	14.939	11.078	5.749	2.069	0.465	60.855
18.125	17.295	16.395	15.815	12.261	6.074	2.180	0.480	66.445
18.375	17.001	16.748	15.337	12.105	6.220	2.175	0.664	72.874
18.625	9.647	9.978	9.109	7.872	4.975	2.276	0.708	80.382
18.875	8.799	9.452	9.001	7.541	5.151	2.382	0.923	89.348

Midpoint	Ratio: 110	25	9.1	2.9	1	0.35	0.11	Volume
19.125	11.437	11.000	10.286	9.050	5.905	2.897	1.137	100.438
19.375	12.400	12.450	11.774	10.385	6.808	3.448	1.247	115.056
19.625	15.125	14.381	13.687	11.633	7.891	4.106	1.439	137.850
19.875	17.252	17.349	15.676	13.431	8.907	4.329	1.786	6066.954
20.125	113.880	112.511	107.376	89.697	61.699	31.020	11.056	1234.242
20.375	137.559	134.767	128.356	108.275	73.695	37.206	13.484	1234.242
20.625	135.992	134.511	127.319	107.738	73.721	35.789	13.225	1234.242
20.875	135.905	132.776	126.325	105.655	72.536	36.474	12.871	1234.242
21.125	135.187	131.085	123.761	105.417	71.695	35.895	12.436	1234.242
21.375	133.267	131.934	122.600	104.288	69.567	35.312	12.687	1234.242
21.625	133.181	127.929	122.993	103.453	70.161	35.683	12.753	1234.242
21.875	131.118	129.447	121.821	102.453	70.071	35.259	13.070	1234.242
22.125	131.556	127.674	120.784	101.596	69.945	34.346	12.576	1234.242
22.375	127.114	126.036	120.601	100.164	68.790	35.179	12.620	1234.242
22.625	127.984	125.457	119.124	100.596	67.856	33.980	12.340	1234.242
22.875	127.042	122.835	119.134	99.619	67.252	33.752	12.458	1234.242
23.125	126.496	125.044	118.296	98.784	67.066	33.662	12.657	1234.242
23.375	126.115	123.744	117.694	98.343	65.937	34.118	12.325	1234.242
23.625	125.906	124.022	117.135	97.050	66.079	33.858	12.377	1234.242
23.875	125.245	122.241	115.684	97.467	66.618	33.900	12.509	1234.242
24.125	124.936	122.429	113.250	97.183	66.614	33.428	11.867	1234.242
24.375	126.302	120.956	116.071	96.408	65.392	33.110	12.222	1234.242
24.625	122.930	120.430	115.969	95.059	65.448	33.662	12.782	1234.242
24.875	122.794	121.249	113.755	96.151	65.186	33.142	12.517	1234.242
25.125	121.974	120.363	113.352	96.522	66.272	33.561	12.576	1234.242
25.375	122.039	120.100	112.863	94.531	64.289	33.142	12.192	1234.242
25.625	122.894	120.498	113.744	96.201	65.206	33.534	12.295	1234.242
25.875	123.211	118.740	113.153	94.655	64.034	31.667	11.971	1234.242
26.125	123.052	121.475	114.443	95.256	64.622	32.473	12.251	1234.242
26.375	120.048	119.672	112.347	95.022	64.326	32.251	11.956	1234.242
26.625	121.442	117.883	111.858	95.848	64.293	32.436	12.266	1234.242
26.875	123.843	118.920	112.358	94.807	63.645	32.744	12.140	1234.242
27.125	120.098	120.205	114.454	95.435	64.452	32.564	11.889	1234.242
27.375	122.729	119.769	111.589	94.577	63.698	32.590	12.524	1234.242
27.625	120.788	118.161	112.917	95.330	63.954	32.770	11.675	1234.242
27.875	121.543	118.883	111.939	94.632	65.276	31.969	12.318	1234.242
28.125	120.759	120.716	112.755	94.779	64.117	32.388	12.133	1234.242
28.375	119.961	119.551	112.976	93.449	64.250	32.532	12.258	1234.242
28.625	121.219	119.484	113.776	94.151	63.901	32.887	12.458	1234.242
28.875	121.802	120.122	113.234	94.087	64.173	33.412	12.377	1234.242
29.125	121.938	120.280	114.497	95.128	64.715	33.343	11.624	1234.242
29.375	121.536	119.318	113.239	95.623	64.382	32.569	11.889	1234.242
29.625	120.961	120.228	113.599	94.082	63.947	33.259	12.266	1234.242
29.875	123.469	117.613	114.314	94.894	64.479	33.004	12.377	1234.242
30.125	122.154	118.702	114.045	94.894	64.044	32.415	12.428	1234.242

Midpoint	Ratio: 110	25	9.1	2.9	1	0.35	0.11	Volume
30.375	122.362	118.725	113.755	95.926	64.831	32.877	12.008	1234.242
30.625	121.838	120.333	113.631	94.518	64.293	32.309	12.222	1234.242
30.875	121.349	119.619	114.846	94.499	64.970	32.553	12.207	1234.242
31.125	121.550	119.566	112.358	95.880	65.199	32.749	12.222	1234.242
31.375	122.657	120.738	113.637	95.004	64.861	32.930	12.244	1234.242
31.625	120.860	120.017	113.637	95.247	65.233	32.739	12.568	1234.242
31.875	123.060	119.206	112.412	95.536	65.156	33.280	11.690	1234.242
32.125	123.448	119.183	114.088	94.761	64.333	32.940	12.214	1234.242
32.375	123.060	120.708	114.636	96.114	64.781	32.643	12.502	1234.242
32.625	122.290	120.145	113.674	95.169	65.286	32.760	12.620	1234.242
32.875	122.204	121.137	114.427	96.247	64.934	33.041	12.591	1234.242
33.125	122.664	120.663	113.502	95.591	64.774	33.190	12.502	1234.242
33.375	121.909	121.024	114.061	95.174	64.987	32.537	12.222	1234.242
33.625	122.657	119.724	113.158	96.050	64.914	33.672	12.945	1234.242
33.875	121.550	121.903	112.524	95.242	64.333	32.266	12.465	1234.242
34.125	122.413	121.783	114.867	96.298	64.080	33.274	12.472	1234.242
34.375	121.874	121.685	114.061	95.729	65.322	32.516	12.022	1234.242
34.625	123.635	119.236	113.750	96.302	64.695	33.460	12.458	1234.242
34.875	123.081	120.385	113.347	95.169	64.615	32.818	12.406	1234.242
35.125	123.771	119.950	114.292	94.784	64.349	32.638	12.413	1234.242
35.375	123.822	122.219	113.610	96.600	64.326	33.057	12.598	1234.242
35.625	121.895	119.919	114.072	95.596	64.678	34.033	12.199	1234.242
35.875	123.304	121.437	112.863	96.366	65.478	33.455	11.919	1234.242
36.125	121.320	120.152	113.707	95.554	64.256	32.744	12.576	1234.242
36.375	121.385	120.122	115.254	95.091	64.910	33.243	12.805	1234.242
36.625	120.910	120.445	114.884	95.683	64.685	33.556	12.458	1234.242
36.875	123.160	121.370	113.970	95.857	64.455	33.168	12.509	1234.242
37.125	122.477	121.715	113.556	95.559	65.312	32.983	12.908	1234.242
37.375	122.628	122.316	112.981	95.683	65.063	32.972	12.480	1234.242
37.625	121.787	119.995	115.840	95.600	65.286	32.866	12.679	1234.242
37.875	122.096	121.122	114.174	96.128	64.834	32.924	12.539	1234.242
38.125	123.613	121.257	114.846	96.453	64.449	33.020	12.362	1234.242
38.375	122.010	118.890	114.120	95.068	64.675	33.280	12.059	1234.242
38.625	121.787	119.874	113.916	94.678	64.313	32.956	12.635	1234.242
38.875	123.088	120.160	114.357	96.307	65.206	33.057	11.904	1234.242
39.125	121.938	120.235	116.582	96.509	65.661	32.558	12.199	1234.242
39.375	122.909	120.355	114.986	95.187	64.216	32.701	12.517	1234.242
39.625	123.232	122.857	115.518	96.298	64.625	32.946	12.495	1234.242
39.875	121.428	121.197	114.722	96.357	63.619	32.887	12.229	1234.242
40.125	122.664	120.881	113.798	95.674	65.595	32.728	12.746	1234.242
40.375	122.219	121.482	114.180	95.981	65.017	32.855	11.712	1234.242
40.625	122.319	120.784	114.695	93.775	64.688	32.967	12.443	1234.242
40.875	123.455	121.588	113.782	95.187	64.117	32.892	12.199	1234.242
41.125	121.449	120.107	113.282	95.059	64.821	32.924	11.845	1234.242
41.375	123.031	119.679	113.889	95.242	64.160	32.510	12.627	1234.242

Midpoint	Ratio: 110	25	9.1	2.9	1	0.35	0.11	Volume
41.625	121.428	119.116	113.798	96.339	64.861	33.237	12.222	1234.242
41.875	121.730	119.694	113.137	94.697	65.425	32.654	12.303	1234.242
42.125	122.355	118.838	114.894	95.944	64.157	33.359	12.015	1234.242
42.375	122.039	119.446	113.610	94.343	64.548	32.686	12.244	1234.242
42.625	121.600	120.160	112.524	94.710	64.595	32.877	12.081	1234.242
42.875	121.579	120.002	112.960	94.614	64.266	33.009	11.904	1234.242
43.125	121.507	119.980	113.121	93.669	64.276	32.733	12.738	1234.242
43.375	121.471	118.807	112.406	94.903	64.103	32.818	11.853	1234.242
43.625	121.428	118.980	113.578	92.692	64.691	33.550	12.546	1234.242
43.875	121.047	121.685	114.126	95.114	63.439	33.142	12.450	1234.242
44.125	120.184	120.182	113.250	94.265	64.067	32.670	12.620	1234.242
44.375	120.400	119.409	111.519	94.146	63.396	32.855	11.934	1234.242
44.625	120.882	119.491	113.293	93.738	64.103	33.386	12.244	1234.242
44.875	120.393	117.943	111.837	94.064	64.279	31.635	13.070	1234.242
45.125	119.307	116.824	112.395	94.481	63.599	32.542	11.720	1234.242
45.375	120.817	118.026	111.799	93.169	62.858	32.001	12.709	1234.242
45.625	120.666	118.327	112.390	94.137	62.865	32.447	11.919	1234.242
45.875	119.832	116.749	112.003	93.481	62.393	32.341	12.155	1234.242
46.125	119.897	118.094	111.342	91.807	63.433	31.699	12.281	1234.242
46.375	121.579	117.756	110.138	93.077	63.336	31.375	11.764	1234.242
46.625	118.502	118.154	112.922	93.385	63.257	31.487	12.000	1234.242
46.875	121.363	115.231	111.390	92.775	63.519	31.911	12.679	1234.242
47.125	119.681	116.193	110.493	91.499	62.463	32.452	11.705	1234.242
47.375	120.867	115.546	109.569	91.146	62.978	32.357	12.103	1234.242
47.625	117.848	116.155	110.026	91.513	63.094	32.012	11.454	1234.242
47.875	117.654	115.817	109.225	91.362	62.672	31.312	11.550	1234.242
48.125	119.616	116.816	109.246	91.642	61.586	31.646	11.491	1234.242
48.375	118.401	115.193	109.477	90.298	61.702	31.099	11.845	1234.242
48.625	117.237	113.803	111.944	91.885	61.181	30.781	11.853	1234.242
48.875	116.468	114.119	109.424	90.816	61.636	31.646	12.340	1234.242
49.125	117.165	114.292	108.902	90.412	60.762	31.380	11.690	1234.242
49.375	116.252	114.660	109.096	90.389	60.812	30.988	11.779	1234.242
49.625	118.524	114.630	108.591	90.665	60.849	30.850	11.587	1234.242
49.875	116.238	114.367	107.290	90.302	61.267	30.882	11.063	1234.242
50.125	116.712	114.547	106.909	89.403	60.417	31.189	10.886	1234.242
50.375	116.554	112.616	107.957	89.398	59.879	30.744	11.734	1234.242
50.625	114.570	112.781	107.554	89.077	60.826	31.338	11.624	1234.242
50.875	113.506	113.202	107.586	88.775	60.663	30.107	11.838	1234.242
51.125	116.525	113.285	106.328	88.233	60.786	30.558	11.432	1234.242
51.375	114.570	111.219	106.049	88.128	60.603	29.736	11.410	1234.242
51.625	114.527	111.271	105.044	87.976	59.922	30.935	11.284	1234.242
51.875	112.679	112.556	105.479	88.357	60.248	31.248	11.520	1234.242
52.125	114.017	112.038	106.511	87.912	60.782	30.176	11.306	1234.242
52.375	116.921	111.098	106.694	88.018	60.068	31.354	11.506	1234.242
52.625	114.412	112.849	107.704	87.908	59.331	30.818	11.439	1234.242

Midpoint	Ratio: 110	25	9.1	2.9	1	0.35	0.11	Volume
52.875	113.952	111.557	105.941	89.683	59.826	30.102	11.845	1234.242
53.125	113.995	111.925	106.226	89.265	59.889	30.563	11.439	1234.242
53.375	114.735	111.459	104.630	88.876	60.065	30.277	11.749	1234.242
53.625	112.895	112.398	106.699	87.678	60.899	30.887	11.705	1234.242
53.875	114.088	110.670	105.807	88.087	58.786	30.792	11.218	1234.242
54.125	114.728	111.970	105.119	88.715	59.736	30.951	11.698	1234.242
54.375	114.002	110.880	105.329	89.715	59.696	30.845	11.698	1234.242
54.625	115.267	113.495	106.269	87.403	59.819	30.606	11.107	1234.242
54.875	114.031	110.445	106.446	89.032	60.121	30.314	10.753	1234.242
55.125	115.116	112.316	106.264	87.554	59.922	30.097	11.270	1234.242
55.375	115.720	112.691	105.662	88.481	60.500	30.744	11.336	1234.242
55.625	114.958	110.505	106.189	87.834	59.955	30.707	11.255	1234.242
55.875	115.993	113.878	106.495	89.004	59.673	30.426	11.270	1234.242
56.125	115.519	111.977	106.667	89.045	59.872	30.441	11.329	1234.242
56.375	114.045	112.864	107.000	88.256	61.251	30.229	11.358	1234.242
56.625	116.166	113.600	106.393	89.178	60.397	30.558	11.786	1234.242
56.875	114.764	112.488	106.307	89.802	60.341	30.325	10.819	1234.242
57.125	116.072	112.887	105.834	88.513	60.224	30.696	11.351	1234.242
57.375	114.304	113.082	106.608	88.545	59.344	29.736	11.557	1234.242
57.625	115.044	111.542	107.188	87.655	60.351	31.083	11.292	1234.242
57.875	114.764	112.586	107.011	88.876	60.092	30.786	11.247	1234.242
58.125	41.628	41.266	38.934	32.656	21.786	11.300	4.155	1234.242
58.375	0.000	0.000	0.000	0.000	0.000	0.000	0.000	1234.242

Table of K^+ population data for simulation with K^+ and NH_4^+ across bulk density ratios (#JJF-GRS1459)

	Population in histogram bin at NH_4^+/K^+ ratio:							
Midpoint	110	25	9.1	2.9	1	0.35	0.11	Volume
-58.375	0.129	0.503	1.446	3.867	7.632	11.290	13.646	1246.101
-58.125	1.100	4.275	11.183	30.344	58.943	87.717	106.031	1246.101
-57.875	1.121	4.719	11.753	30.027	60.251	87.414	104.924	1246.101
-57.625	1.078	4.478	11.527	30.192	59.696	89.372	105.101	1246.101
-57.375	1.021	4.456	11.022	30.940	60.261	87.330	106.031	1246.101
-57.125	1.042	4.846	11.559	29.977	59.122	89.346	105.345	1246.101
-56.875	1.064	4.260	11.704	30.459	59.188	88.788	104.614	1246.101
-56.625	1.136	4.411	11.334	30.243	60.772	88.269	106.393	1246.101
-56.375	0.906	4.501	11.737	29.642	59.826	87.579	104.378	1246.101
-56.125	1.050	4.598	11.995	30.321	59.603	89.155	104.658	1246.101
-55.875	1.042	4.245	11.667	30.238	59.617	87.897	103.529	1246.101
-55.625	1.071	4.328	11.790	31.160	59.686	87.096	105.278	1246.101
-55.375	1.064	4.643	11.640	30.738	60.158	87.658	104.518	1246.101
-55.125	1.085	4.568	11.995	30.431	60.145	88.210	106.939	1246.101
-54.875	1.014	4.418	11.565	30.087	59.490	87.696	105.994	1246.101
-54.625	0.999	4.493	11.758	30.459	59.012	88.953	104.931	1246.101
-54.375	1.100	4.711	11.291	29.793	60.580	88.550	105.625	1246.101

Midpoint	Ratio: 110	25	9.1	2.9	1	0.35	0.11	Volume
-54.125	1.021	4.553	11.092	30.807	59.301	87.515	104.821	1246.101
-53.875	1.021	4.463	11.468	30.491	60.527	88.873	103.898	1246.101
-53.625	1.050	4.441	11.624	30.592	60.616	87.101	106.208	1246.101
-53.375	0.970	4.411	12.231	30.954	59.447	88.438	105.581	1246.101
-53.125	0.999	4.155	11.839	30.647	59.959	88.226	104.474	1246.101
-52.875	0.992	4.396	11.473	29.926	59.789	88.046	104.688	1246.101
-52.625	1.078	4.561	11.291	30.041	59.517	87.945	105.529	1246.101
-52.375	0.999	4.824	11.371	31.266	60.311	88.189	104.865	1246.101
-52.125	1.006	4.606	11.635	30.830	59.799	88.438	104.474	1246.101
-51.875	1.193	4.779	11.995	30.449	60.483	89.918	105.721	1246.101
-51.625	1.028	4.283	11.516	30.225	61.151	88.937	105.544	1246.101
-51.375	1.064	4.846	11.672	30.009	59.879	88.253	104.260	1246.101
-51.125	1.157	4.771	11.731	30.674	60.480	88.895	107.182	1246.101
-50.875	1.100	4.463	11.151	30.633	60.381	89.526	106.363	1246.101
-50.625	1.028	4.230	11.618	30.660	60.968	88.677	106.297	1246.101
-50.375	1.222	4.794	11.350	31.247	60.925	90.433	105.802	1246.101
-50.125	1.215	4.388	11.215	30.954	60.932	90.513	107.005	1246.101
-49.875	1.078	4.891	11.301	31.197	61.493	89.940	106.614	1246.101
-49.625	1.078	4.756	11.876	31.119	60.955	90.470	108.378	1246.101
-49.375	1.064	4.643	11.758	31.390	61.364	91.064	108.031	1246.101
-49.125	1.050	4.914	12.102	30.738	61.476	90.773	109.168	1246.101
-48.875	1.093	4.764	12.424	31.495	61.566	91.160	108.562	1246.101
-48.625	1.100	4.779	12.102	30.981	62.237	91.892	106.990	1246.101
-48.375	1.107	4.719	11.645	31.821	62.543	90.826	109.588	1246.101
-48.125	1.129	4.411	12.188	32.147	62.214	91.102	109.780	1246.101
-47.875	1.229	5.042	12.381	32.312	62.586	92.677	109.035	1246.101
-47.625	1.121	4.598	11.763	31.945	62.632	92.619	110.009	1246.101
-47.375	0.920	4.906	12.279	32.046	62.642	92.003	108.872	1246.101
-47.125	1.402	4.756	12.537	31.867	63.267	93.712	111.057	1246.101
-46.875	1.165	4.734	12.263	31.692	63.625	93.197	111.404	1246.101
-46.625	1.280	4.952	12.038	31.720	63.243	93.611	110.245	1246.101
-46.375	1.143	4.801	12.424	32.463	63.360	92.592	110.680	1246.101
-46.125	0.906	4.613	12.188	32.367	63.642	94.444	112.068	1246.101
-45.875	1.042	4.674	12.064	32.426	63.821	92.942	112.909	1246.101
-45.625	1.165	4.794	11.790	32.550	63.991	92.889	111.625	1246.101
-45.375	0.906	4.974	12.167	32.229	63.602	94.253	112.038	1246.101
-45.125	0.956	5.297	12.790	32.266	63.712	94.964	111.396	1246.101
-44.875	1.100	4.689	12.183	31.807	63.555	94.232	112.016	1246.101
-44.625	1.114	4.869	12.279	32.486	63.984	94.025	111.721	1246.101
-44.375	0.942	4.906	12.027	33.348	64.240	94.147	112.157	1246.101
-44.125	1.085	5.072	11.769	32.628	64.409	94.126	112.703	1246.101
-43.875	1.165	4.997	12.795	33.371	64.940	93.500	111.500	1246.101
-43.625	1.165	5.147	12.408	32.275	63.974	95.065	113.337	1246.101
-43.375	1.200	5.154	12.210	33.027	64.123	96.577	112.592	1246.101
-43.125	1.208	4.914	12.650	32.936	64.339	95.261	111.765	1246.101

Midpoint	Ratio: 110	25	9.1	2.9	1	0.35	0.11	Volume
-42.875	1.021	5.064	12.489	32.500	64.319	95.006	112.924	1246.101
-42.625	1.057	4.914	12.306	33.270	64.250	95.860	112.880	1246.101
-42.375	1.193	4.666	11.790	33.225	64.283	94.985	113.382	1246.101
-42.125	1.093	4.937	12.897	33.981	64.449	95.526	114.061	1246.101
-41.875	1.136	4.749	12.435	33.170	64.233	94.895	113.714	1246.101
-41.625	1.100	4.666	12.414	32.651	64.980	94.799	112.917	1246.101
-41.375	1.143	5.252	12.629	32.835	64.492	94.895	113.662	1246.101
-41.125	1.165	4.891	12.806	33.335	64.937	96.014	110.917	1246.101
-40.875	1.172	4.824	12.317	33.096	65.502	96.545	113.027	1246.101
-40.625	0.992	5.335	12.107	33.647	65.515	96.327	112.164	1246.101
-40.375	0.927	4.779	13.021	32.945	65.223	95.001	114.348	1246.101
-40.125	1.114	5.102	12.398	33.605	64.980	94.141	115.101	1246.101
-39.875	1.100	5.019	12.570	33.247	65.256	96.099	113.079	1246.101
-39.625	0.963	5.064	12.247	32.945	65.920	95.691	114.562	1246.101
-39.375	1.121	4.906	12.339	33.458	64.788	95.017	114.326	1246.101
-39.125	1.208	4.659	12.215	33.399	64.462	95.706	114.053	1246.101
-38.875	1.107	4.929	12.344	34.307	64.628	96.460	116.075	1246.101
-38.625	1.093	4.944	12.623	32.729	65.415	96.189	115.544	1246.101
-38.375	1.129	4.734	12.446	33.720	65.013	96.051	113.773	1246.101
-38.125	1.057	4.854	12.586	33.592	64.984	95.457	112.961	1246.101
-37.875	1.172	5.004	12.231	33.981	64.944	97.606	114.378	1246.101
-37.625	1.272	5.049	12.817	33.238	65.485	96.497	114.481	1246.101
-37.375	1.157	4.891	12.548	32.936	65.701	95.611	113.913	1246.101
-37.125	1.150	5.094	12.688	33.422	65.183	97.415	113.847	1246.101
-36.875	1.100	4.621	12.613	33.101	65.538	96.720	113.182	1246.101
-36.625	1.085	5.004	12.000	32.940	64.984	95.346	115.522	1246.101
-36.375	1.165	4.801	12.446	33.293	64.794	95.102	115.101	1246.101
-36.125	1.100	4.711	12.613	33.426	65.020	96.173	113.544	1246.101
-35.875	1.028	5.184	12.489	33.344	65.319	96.619	114.843	1246.101
-35.625	1.093	4.989	12.494	33.101	65.402	96.248	113.455	1246.101
-35.375	1.172	4.922	12.070	33.660	65.233	96.226	112.769	1246.101
-35.125	1.093	4.831	12.430	33.321	65.329	97.277	111.817	1246.101
-34.875	1.157	4.501	12.510	33.376	65.246	96.598	114.961	1246.101
-34.625	1.114	4.734	12.532	33.303	63.977	97.431	113.765	1246.101
-34.375	1.244	4.982	12.795	33.399	65.156	96.518	114.555	1246.101
-34.125	0.992	4.553	12.387	33.009	65.236	96.646	114.186	1246.101
-33.875	1.315	4.922	12.828	33.972	65.199	94.990	112.791	1246.101
-33.625	1.042	5.192	12.811	33.078	64.734	96.226	112.776	1246.101
-33.375	1.093	4.681	12.763	32.844	64.854	95.834	114.954	1246.101
-33.125	1.071	4.779	12.258	33.270	65.196	95.330	113.197	1246.101
-32.875	1.172	5.072	12.553	33.293	65.585	95.171	113.463	1246.101
-32.625	1.006	4.982	13.059	32.917	65.216	96.274	113.315	1246.101
-32.375	1.172	4.508	12.494	33.624	64.960	95.770	114.252	1246.101
-32.125	1.050	4.583	12.602	33.018	65.379	95.956	113.352	1246.101
-31.875	0.920	4.982	12.462	32.958	64.708	95.425	114.924	1246.101

Midpoint	Ratio: 110	25	9.1	2.9	1	0.35	0.11	Volume
-31.625	1.100	4.696	12.505	33.179	66.398	94.396	114.312	1246.101
-31.375	1.165	4.959	12.387	33.284	64.542	95.834	113.891	1246.101
-31.125	1.085	4.719	12.220	32.803	64.429	96.444	112.437	1246.101
-30.875	1.186	4.643	12.258	32.803	65.223	93.579	115.064	1246.101
-30.625	0.970	4.922	12.564	32.325	65.973	94.523	113.522	1246.101
-30.375	1.179	5.042	11.989	32.766	65.196	95.436	113.500	1246.101
-30.125	1.157	4.861	12.408	33.885	64.771	94.640	113.219	1246.101
-29.875	1.172	4.846	12.167	33.729	65.332	94.752	112.371	1246.101
-29.625	1.107	4.989	11.887	33.339	64.778	95.404	112.164	1246.101
-29.375	1.050	4.974	12.247	32.436	64.615	95.659	111.883	1246.101
-29.125	0.978	4.478	12.763	33.376	64.837	93.500	111.647	1246.101
-28.875	1.093	4.929	12.279	33.156	64.648	95.515	113.168	1246.101
-28.625	1.244	4.914	12.344	32.858	64.572	94.571	112.201	1246.101
-28.375	1.050	4.681	12.032	33.179	63.861	95.340	112.186	1246.101
-28.125	1.193	4.478	12.150	32.803	65.239	94.476	111.153	1246.101
-27.875	0.978	4.764	12.645	32.431	63.768	94.290	112.311	1246.101
-27.625	1.107	4.831	12.054	32.931	64.449	94.810	112.430	1246.101
-27.375	1.157	4.749	12.339	32.862	64.482	95.664	111.906	1246.101
-27.125	0.992	4.884	12.349	32.702	63.878	95.303	113.367	1246.101
-26.875	0.942	4.711	12.215	32.670	64.515	94.401	110.503	1246.101
-26.625	0.834	4.839	12.548	33.064	65.010	94.279	113.478	1246.101
-26.375	1.114	4.854	12.167	33.243	64.160	94.189	113.721	1246.101
-26.125	0.927	4.621	12.424	33.385	64.459	94.126	113.389	1246.101
-25.875	1.287	4.659	12.263	33.032	64.157	94.645	111.500	1246.101
-25.625	1.215	4.591	12.371	32.899	64.167	93.542	112.496	1246.101
-25.375	0.935	4.403	12.339	32.903	64.675	94.518	114.046	1246.101
-25.125	1.136	4.606	12.365	33.917	64.525	95.659	112.717	1246.101
-24.875	0.992	4.922	11.817	32.692	64.990	96.375	113.241	1246.101
-24.625	1.021	4.516	12.559	33.665	65.442	95.537	113.773	1246.101
-24.375	1.078	4.516	12.677	33.780	65.957	96.126	114.090	1246.101
-24.125	0.963	4.448	12.666	34.234	65.535	96.264	114.562	1246.101
-23.875	1.129	4.861	13.150	33.417	65.581	96.433	113.810	1246.101
-23.625	0.970	4.508	12.505	34.174	66.262	96.014	117.330	1246.101
-23.375	0.985	4.801	12.387	34.059	66.647	96.147	115.957	1246.101
-23.125	0.963	4.891	12.940	33.789	67.205	99.468	116.754	1246.101
-22.875	0.855	4.568	12.290	34.362	66.943	99.203	116.880	1246.101
-22.625	1.006	4.734	12.758	34.949	67.850	99.622	117.706	1246.101
-22.375	0.819	4.989	12.871	34.958	68.291	101.229	116.555	1246.101
-22.125	1.021	4.937	12.521	35.339	69.308	100.216	119.131	1246.101
-21.875	0.985	4.982	12.537	35.353	69.610	101.293	120.577	1246.101
-21.625	1.136	4.824	12.795	35.436	69.935	101.861	121.662	1246.101
-21.375	0.956	5.079	13.021	36.275	70.689	103.001	121.544	1246.101
-21.125	1.064	4.756	13.021	35.426	71.908	105.590	123.684	1246.101
-20.875	0.963	4.952	12.914	37.289	72.223	104.036	127.168	1246.101
-20.625	1.014	5.034	13.467	37.050	72.483	107.001	124.939	1246.101

Midpoint	Ratio: 110	25	9.1	2.9	1	0.35	0.11	Volume
-20.375	0.927	4.621	13.182	36.991	73.838	106.927	127.249	1246.101
-20.125	0.927	4.997	13.757	37.738	74.412	106.715	127.219	1246.101
-19.875	0.604	2.953	8.093	23.027	46.382	67.270	78.532	1246.101
-19.625	0.072	0.564	1.725	4.372	9.312	13.003	14.782	5649.650
-19.375	0.108	0.609	1.403	3.748	7.788	11.168	13.159	131.318
-19.125	0.093	0.353	1.188	3.289	6.636	9.719	10.923	111.609
-18.875	0.050	0.301	1.026	2.986	5.603	8.329	9.447	98.132
-18.625	0.050	0.316	0.881	2.495	5.121	8.064	9.550	87.699
-18.375	0.065	0.233	0.704	2.197	6.772	12.085	15.912	79.176
-18.125	0.050	0.270	0.650	2.055	6.204	12.441	17.129	71.991
-17.875	0.065	0.180	0.516	1.954	6.137	11.491	15.439	65.812
-17.625	0.029	0.225	0.484	1.959	5.563	11.120	14.583	60.420
-17.375	0.036	0.143	0.419	1.679	5.563	10.356	13.454	55.666
-17.125	0.065	0.098	0.430	1.716	5.002	10.165	12.458	51.439
-16.875	0.036	0.120	0.468	1.605	4.633	8.945	11.823	47.657
-16.625	0.007	0.128	0.387	1.555	4.420	8.722	12.030	44.256
-16.375	0.050	0.165	0.328	1.459	4.400	8.791	10.502	41.184
-16.125	0.050	0.090	0.392	1.298	3.972	7.533	10.406	38.400
-15.875	0.000	0.075	0.263	1.156	3.766	7.104	9.336	35.869
-15.625	0.014	0.083	0.269	1.096	3.454	6.632	8.428	33.564
-15.375	0.029	0.105	0.231	0.982	3.255	6.250	8.022	31.460
-15.125	0.014	0.045	0.236	1.096	3.152	5.899	7.889	29.537
-14.875	0.029	0.120	0.215	0.835	2.962	5.401	7.233	27.776
-14.625	0.014	0.038	0.231	0.894	2.926	5.374	7.395	26.162
-14.375	0.007	0.120	0.215	0.853	2.730	5.348	6.849	24.682
-14.125	0.000	0.098	0.210	0.881	2.664	5.178	6.996	23.324
-13.875	0.007	0.090	0.161	0.839	2.733	5.056	7.070	22.078
-13.625	0.014	0.098	0.161	0.950	2.697	5.093	6.996	20.933
-13.375	0.007	0.090	0.242	0.784	2.637	4.865	6.236	19.883
-13.125	0.007	0.083	0.167	0.780	2.650	4.923	6.199	18.920
-12.875	0.022	0.038	0.220	0.872	2.341	4.865	6.044	18.036
-12.625	0.022	0.068	0.210	0.766	2.421	4.860	5.912	17.227
-12.375	0.022	0.045	0.231	0.812	2.418	4.791	5.565	16.487
-12.125	0.014	0.053	0.210	0.716	2.308	4.509	5.683	15.810
-11.875	0.014	0.053	0.215	0.706	2.242	4.472	5.971	15.194
-11.625	0.014	0.075	0.145	0.734	2.258	4.228	5.513	14.634
-11.375	0.000	0.083	0.193	0.798	2.438	3.931	5.690	14.126
-11.125	0.014	0.045	0.156	0.688	2.268	4.313	5.926	13.668
-10.875	0.007	0.053	0.172	0.610	2.152	4.377	5.852	13.256
-10.625	0.014	0.075	0.134	0.624	2.564	4.642	5.705	12.889
-10.375	0.022	0.030	0.161	0.798	2.511	4.907	5.978	12.563
-10.125	0.014	0.053	0.161	0.817	2.501	5.353	6.421	12.278
-9.875	0.007	0.083	0.204	0.885	3.026	5.735	7.277	12.031
-9.625	0.014	0.060	0.247	1.023	3.006	5.549	7.188	11.821
-9.375	0.014	0.060	0.204	0.982	3.115	5.645	7.218	11.647

Midpoint	Ratio: 110	25	9.1	2.9	1	0.35	0.11	Volume
-9.125	0.036	0.098	0.210	0.927	3.069	5.533	6.768	11.508
-8.875	0.014	0.090	0.177	1.023	2.896	4.504	5.985	11.402
-8.625	0.007	0.060	0.193	0.817	2.471	4.340	5.447	11.330
-8.375	0.007	0.068	0.183	0.472	1.076	1.671	1.934	11.291
-8.125	0.007	0.053	0.086	0.339	0.604	0.817	1.387	11.282
-7.875	0.000	0.053	0.070	0.284	0.508	0.812	1.063	11.282
-7.625	0.014	0.053	0.086	0.197	0.349	0.557	0.657	11.282
-7.375	0.007	0.023	0.064	0.138	0.329	0.414	0.480	11.282
-7.125	0.000	0.000	0.038	0.096	0.236	0.329	0.362	11.282
-6.875	0.000	0.000	0.011	0.078	0.146	0.202	0.280	11.282
-6.625	0.000	0.015	0.048	0.083	0.139	0.138	0.170	11.282
-6.375	0.000	0.008	0.027	0.023	0.076	0.090	0.111	11.282
-6.125	0.000	0.008	0.000	0.005	0.053	0.074	0.096	11.282
-5.875	0.000	0.008	0.000	0.023	0.027	0.042	0.059	11.282
-5.625	0.000	0.015	0.000	0.023	0.040	0.053	0.059	11.282
-5.375	0.000	0.000	0.000	0.023	0.020	0.037	0.030	11.282
-5.125	0.000	0.000	0.000	0.009	0.020	0.037	0.030	11.282
-4.875	0.000	0.000	0.000	0.014	0.023	0.032	0.059	11.282
-4.625	0.000	0.000	0.000	0.014	0.027	0.042	0.037	11.282
-4.375	0.000	0.008	0.005	0.028	0.060	0.037	0.059	11.282
-4.125	0.014	0.000	0.011	0.014	0.033	0.053	0.052	11.282
-3.875	0.000	0.008	0.000	0.041	0.050	0.080	0.081	11.282
-3.625	0.000	0.000	0.005	0.018	0.066	0.101	0.089	11.282
-3.375	0.000	0.000	0.021	0.041	0.090	0.117	0.140	11.282
-3.125	0.000	0.015	0.000	0.028	0.080	0.164	0.185	11.282
-2.875	0.000	0.023	0.032	0.055	0.106	0.218	0.244	11.282
-2.625	0.000	0.008	0.032	0.069	0.133	0.249	0.229	11.282
-2.375	0.014	0.008	0.048	0.096	0.203	0.223	0.288	11.282
-2.125	0.000	0.008	0.043	0.092	0.216	0.276	0.362	11.282
-1.875	0.000	0.015	0.038	0.115	0.216	0.340	0.494	11.282
-1.625	0.000	0.023	0.048	0.119	0.232	0.403	0.376	11.282
-1.375	0.000	0.008	0.027	0.115	0.209	0.382	0.421	11.282
-1.125	0.000	0.008	0.048	0.055	0.216	0.302	0.354	11.282
-0.875	0.000	0.000	0.032	0.073	0.203	0.265	0.406	11.282
-0.625	0.007	0.008	0.032	0.096	0.173	0.371	0.376	11.282
-0.125	0.000	0.000	0.059	0.087	0.163	0.286	0.288	11.282
0.125	0.000	0.008	0.021	0.083	0.139	0.218	0.251	11.282
0.625	0.000	0.008	0.048	0.069	0.186	0.196	0.244	11.282
0.875	0.000	0.008	0.032	0.041	0.156	0.186	0.258	11.282
1.125	0.000	0.015	0.005	0.060	0.096	0.207	0.199	11.282
1.375	0.000	0.008	0.005	0.041	0.110	0.223	0.273	11.282
1.625	0.000	0.008	0.032	0.046	0.133	0.170	0.221	11.282
1.875	0.007	0.008	0.016	0.037	0.116	0.191	0.258	11.282
2.125	0.000	0.015	0.038	0.064	0.110	0.149	0.229	11.282
2.375	0.000	0.008	0.011	0.060	0.139	0.143	0.251	11.282

Midpoint	Ratio: 110	25	9.1	2.9	1	0.35	0.11	Volume
2.625	0.000	0.008	0.005	0.037	0.133	0.149	0.325	11.282
2.875	0.007	0.000	0.011	0.055	0.120	0.239	0.140	11.282
3.125	0.000	0.000	0.043	0.032	0.120	0.149	0.251	11.282
3.375	0.000	0.023	0.011	0.073	0.136	0.191	0.347	11.282
3.625	0.007	0.000	0.021	0.096	0.139	0.159	0.221	11.282
3.875	0.000	0.008	0.011	0.083	0.116	0.149	0.244	11.282
4.125	0.000	0.000	0.021	0.073	0.106	0.149	0.229	11.282
4.375	0.000	0.015	0.011	0.060	0.113	0.138	0.192	11.282
4.625	0.007	0.000	0.027	0.041	0.086	0.191	0.177	11.282
4.875	0.000	0.015	0.021	0.032	0.090	0.037	0.103	11.282
5.125	0.000	0.008	0.021	0.037	0.070	0.074	0.140	11.282
5.375	0.000	0.000	0.005	0.037	0.063	0.106	0.118	11.282
5.625	0.000	0.000	0.011	0.046	0.043	0.032	0.118	11.282
5.875	0.000	0.008	0.005	0.032	0.046	0.064	0.089	11.282
6.125	0.000	0.000	0.021	0.041	0.046	0.085	0.103	11.282
6.375	0.000	0.008	0.016	0.028	0.053	0.101	0.081	11.282
6.625	0.000	0.008	0.000	0.028	0.063	0.053	0.133	11.282
6.875	0.000	0.000	0.016	0.037	0.080	0.133	0.111	11.282
7.125	0.007	0.008	0.038	0.046	0.103	0.207	0.170	11.282
7.375	0.000	0.023	0.054	0.083	0.176	0.281	0.295	11.282
7.625	0.007	0.008	0.054	0.115	0.223	0.350	0.450	11.282
7.875	0.000	0.015	0.059	0.151	0.272	0.462	0.686	11.282
8.125	0.000	0.038	0.086	0.197	0.412	0.647	0.701	11.282
8.375	0.007	0.045	0.107	0.362	0.794	1.188	1.432	11.291
8.625	0.014	0.030	0.161	0.670	1.953	3.358	4.317	11.330
8.875	0.000	0.060	0.193	0.812	2.245	4.186	5.395	11.402
9.125	0.007	0.075	0.188	0.821	2.664	4.860	5.941	11.508
9.375	0.022	0.105	0.161	0.945	3.145	5.555	6.982	11.647
9.625	0.029	0.060	0.156	1.018	3.012	5.311	7.085	11.821
9.875	0.000	0.053	0.150	0.862	2.849	5.358	6.930	12.031
10.125	0.007	0.068	0.172	0.798	2.743	5.422	6.819	12.278
10.375	0.007	0.060	0.150	0.821	2.192	4.812	6.089	12.563
10.625	0.014	0.075	0.167	0.633	2.252	4.366	6.118	12.889
10.875	0.000	0.030	0.113	0.697	2.152	4.387	5.683	13.256
11.125	0.000	0.038	0.129	0.578	2.385	4.462	5.483	13.668
11.375	0.007	0.053	0.177	0.697	2.265	4.287	5.454	14.126
11.625	0.000	0.038	0.177	0.615	2.232	4.127	5.646	14.634
11.875	0.036	0.090	0.102	0.693	2.225	4.271	5.491	15.194
12.125	0.022	0.015	0.263	0.775	2.607	4.621	5.506	15.810
12.375	0.036	0.068	0.210	0.775	2.414	4.462	5.934	16.487
12.625	0.043	0.038	0.172	0.830	2.501	4.265	6.133	17.227
12.875	0.014	0.038	0.188	0.839	2.481	4.748	6.000	18.036
13.125	0.000	0.105	0.220	0.817	2.524	4.722	5.779	18.920
13.375	0.007	0.068	0.226	0.771	2.667	4.876	6.303	19.883
13.625	0.007	0.083	0.220	0.812	2.614	5.151	6.790	20.933

Midpoint	Ratio: 110	25	9.1	2.9	1	0.35	0.11	Volume
13.875	0.043	0.113	0.253	0.789	2.717	5.173	6.908	22.078
14.125	0.014	0.068	0.167	0.872	2.521	5.146	6.613	23.324
14.375	0.007	0.075	0.269	0.881	2.740	4.913	6.923	24.682
14.625	0.036	0.038	0.199	0.867	2.511	5.385	7.070	26.162
14.875	0.014	0.038	0.236	0.835	2.869	5.592	7.432	27.776
15.125	0.007	0.068	0.199	1.023	2.989	5.878	7.417	29.537
15.375	0.014	0.113	0.258	0.986	3.075	5.974	8.096	31.460
15.625	0.036	0.075	0.322	1.060	3.338	6.255	8.583	33.564
15.875	0.036	0.105	0.306	1.174	3.647	6.748	9.225	35.869
16.125	0.022	0.120	0.301	1.261	4.068	7.231	9.624	38.400
16.375	0.022	0.105	0.339	1.312	4.188	7.926	9.845	41.184
16.625	0.058	0.075	0.312	1.495	4.464	8.451	11.380	44.256
16.875	0.043	0.158	0.446	1.546	4.889	9.486	12.273	47.657
17.125	0.036	0.195	0.414	1.771	4.889	9.979	12.583	51.439
17.375	0.036	0.188	0.575	1.628	5.540	10.473	13.520	55.666
17.625	0.036	0.188	0.543	1.986	6.041	11.210	13.048	60.420
17.875	0.029	0.210	0.602	1.982	5.931	11.608	15.218	65.812
18.125	0.043	0.173	0.607	2.183	6.423	12.552	16.369	71.991
18.375	0.058	0.195	0.704	2.413	6.260	12.091	15.683	79.176
18.625	0.058	0.308	0.924	2.546	5.374	8.144	9.882	87.699
18.875	0.079	0.421	1.010	2.913	5.672	8.133	10.163	98.132
19.125	0.058	0.458	1.279	3.404	6.805	9.682	11.528	111.609
19.375	0.093	0.391	1.435	4.037	7.911	11.565	12.812	131.318
19.625	0.129	0.564	1.623	4.555	9.000	12.918	14.731	5649.650
19.875	0.618	2.855	8.120	23.637	45.226	67.233	79.278	1246.101
20.125	0.920	4.816	13.338	37.637	73.532	107.383	127.758	1246.101
20.375	0.870	4.846	13.225	37.234	73.140	107.447	124.378	1246.101
20.625	1.014	5.109	13.279	36.298	72.180	105.558	124.585	1246.101
20.875	0.942	4.929	12.736	36.234	72.572	104.497	123.470	1246.101
21.125	0.899	5.027	13.128	36.160	71.818	103.081	122.504	1246.101
21.375	1.064	4.824	13.005	36.261	70.477	103.744	122.954	1246.101
21.625	0.906	4.396	12.860	34.669	69.723	102.943	119.323	1246.101
21.875	1.042	4.523	13.311	35.527	69.039	101.213	119.079	1246.101
22.125	0.877	4.899	12.914	35.174	68.999	100.831	119.382	1246.101
22.375	0.935	4.426	12.607	34.890	67.982	100.227	118.961	1246.101
22.625	0.985	5.004	12.758	34.348	67.537	98.417	117.286	1246.101
22.875	1.121	4.854	12.570	34.614	67.288	99.155	115.891	1246.101
23.125	0.970	4.809	12.999	33.963	67.212	98.752	116.895	1246.101
23.375	1.172	4.756	12.258	33.963	66.800	98.741	115.824	1246.101
23.625	1.057	5.034	13.037	33.559	67.063	97.218	116.245	1246.101
23.875	0.992	4.989	12.494	33.440	66.754	97.033	114.282	1246.101
24.125	0.963	4.884	12.940	33.803	65.176	97.521	113.374	1246.101
24.375	1.172	4.831	12.371	32.766	64.987	95.664	112.924	1246.101
24.625	1.006	4.523	12.301	33.449	65.266	97.139	114.496	1246.101
24.875	1.093	4.561	12.070	33.147	65.176	95.717	114.164	1246.101

Midpoint	Ratio: 110	25	9.1	2.9	1	0.35	0.11	Volume
25.125	1.107	4.764	12.333	33.449	64.854	95.378	112.562	1246.101
25.375	1.035	4.674	12.510	32.729	65.701	96.237	112.813	1246.101
25.625	1.085	4.681	12.269	33.303	63.639	94.465	112.570	1246.101
25.875	1.006	4.651	12.725	32.931	64.708	94.083	113.204	1246.101
26.125	1.050	4.576	12.204	33.903	64.648	94.836	109.869	1246.101
26.375	0.927	4.831	12.365	32.463	63.815	94.523	113.935	1246.101
26.625	1.042	4.719	12.059	33.137	64.064	94.072	111.433	1246.101
26.875	1.071	4.809	12.408	32.711	64.090	93.457	111.588	1246.101
27.125	1.014	4.982	12.236	33.399	64.236	92.741	111.559	1246.101
27.375	1.071	4.824	12.210	32.679	64.163	92.990	113.330	1246.101
27.625	0.935	4.561	12.021	32.665	64.123	94.152	112.939	1246.101
27.875	1.157	4.388	12.441	32.926	63.632	94.147	112.872	1246.101
28.125	1.085	4.719	12.210	32.348	64.270	91.977	111.699	1246.101
28.375	1.014	4.809	12.457	32.188	63.934	96.094	112.466	1246.101
28.625	1.078	5.027	12.387	32.486	64.532	95.484	111.249	1246.101
28.875	1.028	4.876	12.618	32.899	64.363	95.309	112.946	1246.101
29.125	0.970	5.275	12.376	33.477	64.698	94.767	112.518	1246.101
29.375	1.050	4.538	12.392	32.670	64.708	95.494	113.374	1246.101
29.625	1.287	4.831	12.801	33.385	64.595	95.287	112.644	1246.101
29.875	1.157	4.711	12.478	32.770	64.436	95.728	113.050	1246.101
30.125	1.107	4.659	12.408	32.761	65.787	94.481	113.382	1246.101
30.375	1.222	4.681	12.527	32.578	64.824	95.187	113.323	1246.101
30.625	1.071	4.801	13.016	33.229	65.429	94.905	112.865	1246.101
30.875	1.035	4.704	12.709	32.816	65.455	94.969	115.101	1246.101
31.125	1.200	4.659	12.462	33.385	65.163	94.518	113.913	1246.101
31.375	1.121	5.252	12.247	33.692	64.618	95.515	113.300	1246.101
31.625	0.999	4.719	12.494	33.807	63.921	95.998	113.227	1246.101
31.875	1.150	4.689	12.527	33.128	64.877	95.935	111.751	1246.101
32.125	1.042	4.786	12.494	32.747	64.638	93.850	112.725	1246.101
32.375	1.071	4.831	12.183	32.825	64.698	95.622	114.186	1246.101
32.625	1.121	4.922	12.392	33.702	65.017	94.789	115.241	1246.101
32.875	0.999	4.816	12.339	32.835	65.628	95.383	114.223	1246.101
33.125	1.143	4.876	12.747	32.546	65.213	97.272	115.013	1246.101
33.375	0.970	4.771	12.854	33.266	64.638	95.839	113.529	1246.101
33.625	1.093	5.012	12.973	32.821	65.150	95.966	112.872	1246.101
33.875	1.157	4.944	12.946	33.669	65.176	95.409	114.363	1246.101
34.125	1.006	4.628	12.865	33.238	65.691	95.197	115.596	1246.101
34.375	1.014	4.876	12.822	33.628	65.179	95.256	114.112	1246.101
34.625	1.114	4.794	12.231	33.284	66.010	96.518	113.345	1246.101
34.875	1.193	4.876	12.510	33.018	65.405	95.807	113.847	1246.101
35.125	1.165	4.884	12.779	33.133	65.987	95.728	116.378	1246.101
35.375	1.323	5.019	12.688	33.225	64.426	95.871	113.382	1246.101
35.625	1.050	4.779	12.344	33.137	65.206	96.502	112.518	1246.101
35.875	1.014	4.891	12.532	33.592	64.794	96.088	113.736	1246.101
36.125	1.172	4.794	13.311	33.174	65.884	95.102	114.732	1246.101

Midpoint	Ratio: 110	25	9.1	2.9	1	0.35	0.11	Volume
36.375	1.071	5.245	12.983	33.766	65.329	97.038	113.965	1246.101
36.625	1.085	4.982	12.322	33.110	65.654	95.213	115.360	1246.101
36.875	1.021	5.139	12.376	32.413	64.671	96.131	116.134	1246.101
37.125	1.222	4.764	12.967	33.674	65.432	95.473	112.813	1246.101
37.375	1.100	5.064	12.349	33.211	64.758	95.744	115.618	1246.101
37.625	1.150	4.741	12.940	32.899	65.658	95.595	113.544	1246.101
37.875	1.100	4.914	12.537	32.991	64.432	95.510	115.323	1246.101
38.125	1.121	4.824	12.226	33.234	64.482	95.505	112.939	1246.101
38.375	1.050	5.184	12.414	33.036	64.532	95.998	113.190	1246.101
38.625	1.028	5.004	12.193	33.596	65.196	95.006	114.481	1246.101
38.875	1.071	5.252	12.838	33.660	64.658	95.378	113.603	1246.101
39.125	1.114	5.072	12.575	33.298	64.545	95.537	112.939	1246.101
39.375	1.143	4.944	12.446	32.958	64.954	95.919	114.467	1246.101
39.625	1.093	4.643	12.188	33.137	64.661	95.754	114.267	1246.101
39.875	1.215	5.237	12.408	33.353	64.299	95.797	114.216	1246.101
40.125	0.992	4.997	12.408	32.665	64.874	96.051	112.732	1246.101
40.375	1.129	4.824	12.849	33.197	64.103	95.537	114.592	1246.101
40.625	1.129	4.929	12.306	32.848	64.894	96.126	112.260	1246.101
40.875	1.078	4.756	12.097	33.110	64.047	94.539	112.407	1246.101
41.125	1.071	4.929	12.204	33.170	64.396	94.640	114.304	1246.101
41.375	1.258	4.922	12.279	32.312	64.837	96.401	113.109	1246.101
41.625	1.064	4.997	12.322	33.170	64.582	94.439	114.289	1246.101
41.875	1.107	4.546	12.145	33.339	64.768	95.197	115.064	1246.101
42.125	1.136	4.801	12.242	32.784	64.130	94.799	112.629	1246.101
42.375	1.236	5.124	12.553	32.858	64.608	94.444	112.489	1246.101
42.625	1.071	5.064	11.995	33.059	64.123	95.765	113.470	1246.101
42.875	1.121	4.734	12.140	32.885	64.406	94.226	111.714	1246.101
43.125	0.999	4.861	12.177	32.903	64.648	94.921	112.599	1246.101
43.375	1.035	4.899	12.312	33.211	63.931	94.502	112.127	1246.101
43.625	1.093	4.861	12.306	33.261	64.675	96.009	112.658	1246.101
43.875	1.107	4.711	12.709	32.679	64.177	93.473	112.422	1246.101
44.125	1.078	4.974	12.199	33.321	64.635	95.187	111.669	1246.101
44.375	1.014	4.876	12.296	33.046	64.040	93.526	111.906	1246.101
44.625	1.093	4.591	12.392	32.858	63.346	94.688	111.758	1246.101
44.875	1.121	5.162	12.602	33.036	62.835	93.600	112.695	1246.101
45.125	1.165	4.794	12.430	33.082	63.399	92.815	111.655	1246.101
45.375	0.985	4.884	12.183	32.312	63.605	92.820	110.666	1246.101
45.625	1.028	4.794	12.457	31.899	63.655	94.449	112.024	1246.101
45.875	1.035	4.628	12.650	32.683	64.349	93.648	112.134	1246.101
46.125	1.129	5.072	12.424	32.018	63.360	93.887	111.581	1246.101
46.375	1.042	4.546	12.355	32.642	63.316	93.791	111.817	1246.101
46.625	1.006	5.019	12.102	32.504	63.386	92.513	110.710	1246.101
46.875	1.107	4.779	12.086	32.147	63.486	92.157	110.311	1246.101
47.125	1.042	4.982	12.339	31.867	63.360	93.383	109.315	1246.101
47.375	0.992	4.538	12.253	32.124	63.805	92.619	109.773	1246.101

Midpoint	Ratio: 110	25	9.1	2.9	1	0.35	0.11	Volume
47.625	1.006	5.094	12.360	31.816	62.257	91.897	110.976	1246.101
47.875	0.927	4.583	12.048	31.059	62.682	91.308	109.861	1246.101
48.125	0.978	4.531	12.086	31.459	62.589	90.730	108.813	1246.101
48.375	1.114	4.869	12.355	32.266	62.383	91.319	109.581	1246.101
48.625	1.093	4.884	11.968	31.660	62.546	91.484	108.732	1246.101
48.875	1.064	4.786	11.828	31.257	61.377	90.990	109.256	1246.101
49.125	1.200	4.411	12.231	31.757	61.486	90.502	107.832	1246.101
49.375	1.229	4.516	11.801	31.500	61.629	91.070	106.164	1246.101
49.625	0.913	4.516	11.801	31.592	60.992	91.659	106.245	1246.101
49.875	1.100	4.553	12.000	31.362	60.743	90.030	108.031	1246.101
50.125	1.129	4.756	11.817	31.082	60.513	90.094	106.496	1246.101
50.375	1.042	4.561	11.962	30.926	60.978	87.998	108.001	1246.101
50.625	1.078	4.659	11.522	30.734	60.925	89.961	106.858	1246.101
50.875	1.157	4.553	11.586	30.903	60.188	89.632	105.197	1246.101
51.125	1.042	4.719	11.790	30.839	60.640	89.855	106.739	1246.101
51.375	1.172	4.531	11.425	30.449	60.417	89.489	105.987	1246.101
51.625	1.014	4.659	11.586	30.825	60.248	89.680	104.931	1246.101
51.875	0.949	4.486	11.549	30.596	60.022	88.576	105.514	1246.101
52.125	0.920	4.358	11.113	30.335	59.962	87.977	105.049	1246.101
52.375	0.985	4.628	11.667	30.660	59.610	89.430	104.540	1246.101
52.625	0.884	4.426	11.575	30.183	60.201	89.632	106.835	1246.101
52.875	0.978	4.659	11.334	30.848	59.693	87.812	105.241	1246.101
53.125	0.841	4.441	11.269	31.358	60.241	89.515	104.548	1246.101
53.375	1.129	4.418	11.253	30.459	60.490	87.520	107.101	1246.101
53.625	0.913	4.591	11.645	30.101	59.995	87.611	105.345	1246.101
53.875	0.992	4.643	11.441	30.922	59.773	89.001	103.227	1246.101
54.125	1.035	4.636	11.377	30.954	59.969	87.658	104.400	1246.101
54.375	0.978	4.381	11.796	30.252	58.790	87.966	105.477	1246.101
54.625	1.050	4.260	11.360	30.413	58.524	87.531	105.677	1246.101
54.875	0.942	4.869	11.543	30.688	59.427	87.690	105.330	1246.101
55.125	0.935	4.516	11.387	30.683	59.424	89.987	105.190	1246.101
55.375	0.978	4.696	11.678	30.734	59.358	88.481	105.898	1246.101
55.625	1.021	4.178	11.608	30.601	59.630	88.168	105.839	1246.101
55.875	1.035	4.448	11.398	30.188	59.916	87.780	105.072	1246.101
56.125	1.050	4.433	11.317	30.284	59.368	87.542	106.769	1246.101
56.375	0.963	4.238	11.382	30.495	59.454	89.271	104.858	1246.101
56.625	1.028	4.230	11.667	30.248	60.520	87.101	106.186	1246.101
56.875	0.935	4.531	11.285	31.046	59.740	88.391	105.965	1246.101
57.125	0.970	4.553	11.747	29.784	59.693	88.168	105.832	1246.101
57.375	0.899	4.163	11.479	30.280	59.896	88.109	105.079	1246.101
57.625	0.906	4.576	11.812	30.757	59.567	88.184	105.382	1246.101
57.875	1.028	4.508	11.715	30.381	60.201	88.528	104.843	1246.101
58.125	1.014	4.501	11.258	30.179	59.773	88.168	104.739	1246.101
58.375	0.122	0.511	1.424	3.853	7.735	11.459	13.181	1246.101

Midpoint	Ratio: 110	24.5	9.1	2.9	2.9	0.35	0.11	Volume

E.3. KCSA channel: Potassium and Rubidium

Table of Rb^{+} population data for simulation with K^{+} and Rb^{+} across bulk density ratios (#JJF-GRS1459)

	Population in histogram bin at Rb^{+}/K^{+} ratio:							
Midpoint	110	24.5	9.1	2.9	2.9	0.35	0.11	Volume
-58.375	0.000	0.000	0.000	0.000	0.000	0.000	0.000	1238.396
-58.125	72.656	70.731	66.621	55.495	55.657	19.606	7.154	1238.396
-57.875	113.978	112.189	104.788	88.578	87.393	30.568	11.415	1238.396
-57.625	113.242	111.275	106.199	88.125	88.057	30.548	11.506	1238.396
-57.375	114.993	112.172	104.871	89.052	87.788	29.958	11.116	1238.396
-57.125	114.085	112.366	105.945	88.235	88.830	30.283	11.521	1238.396
-56.875	113.787	111.816	105.208	88.162	87.905	29.984	11.598	1238.396
-56.625	114.194	112.205	104.118	88.128	88.001	30.009	11.626	1238.396
-56.375	114.073	112.042	106.763	88.855	87.818	30.534	11.236	1238.396
-56.125	113.886	112.197	105.782	87.758	88.333	30.210	11.144	1238.396
-55.875	114.562	110.793	105.207	88.527	87.586	30.218	11.509	1238.396
-55.625	114.477	111.298	106.319	87.595	87.909	30.685	11.301	1238.396
-55.375	114.450	111.932	105.173	87.797	87.464	30.520	11.161	1238.396
-55.125	113.634	111.203	105.188	88.303	88.177	30.305	11.318	1238.396
-54.875	113.453	112.030	104.457	87.734	87.833	30.585	11.583	1238.396
-54.625	114.545	112.467	105.288	87.787	87.704	30.707	11.341	1238.396
-54.375	113.714	111.830	105.616	87.474	87.521	30.254	11.313	1238.396
-54.125	114.182	111.330	105.212	89.110	87.273	29.746	11.079	1238.396
-53.875	113.307	113.469	105.730	87.673	87.566	29.657	11.678	1238.396
-53.625	114.186	112.182	104.946	87.938	87.768	29.947	11.384	1238.396
-53.375	114.010	111.788	105.361	88.461	87.734	30.445	11.564	1238.396
-53.125	112.991	112.110	104.709	88.347	87.747	30.146	11.515	1238.396
-52.875	113.855	112.964	104.371	88.211	87.699	30.193	11.236	1238.396
-52.625	113.662	111.343	104.525	88.857	88.255	30.445	11.538	1238.396
-52.375	114.778	112.253	105.539	87.586	88.504	30.548	11.558	1238.396
-52.125	113.689	111.940	105.449	87.931	88.441	30.198	11.515	1238.396
-51.875	114.978	111.159	105.240	88.393	88.234	30.339	11.261	1238.396
-51.625	114.147	112.900	106.008	87.440	89.102	30.216	11.718	1238.396
-51.375	114.303	112.535	106.274	88.867	88.486	29.995	11.352	1238.396
-51.125	114.503	113.212	105.670	88.052	88.128	30.431	11.706	1238.396
-50.875	115.426	112.106	106.298	88.748	89.377	30.677	11.407	1238.396
-50.625	113.389	112.337	105.976	89.310	88.285	30.358	11.298	1238.396
-50.375	114.862	112.577	107.000	89.079	89.082	30.543	11.655	1238.396
-50.125	115.100	112.901	106.816	89.888	88.744	30.864	11.692	1238.396
-49.875	116.207	112.758	107.449	89.168	89.394	31.160	11.312	1238.396
-49.625	115.762	113.547	106.432	89.686	90.040	31.233	11.800	1238.396
-49.375	116.537	113.818	106.977	89.869	89.794	30.906	11.564	1238.396
-49.125	117.425	114.075	107.324	89.728	90.729	31.182	11.937	1238.396

Midpoint	Ratio: 110	24.5	9.1	2.9	2.9	0.35	0.11	Volume
-48.875	117.112	114.501	107.529	91.379	91.667	31.068	11.603	1238.396
-48.625	117.608	115.648	108.989	90.520	91.319	31.320	11.900	1238.396
-48.375	117.659	116.073	109.686	90.462	91.019	31.778	12.082	1238.396
-48.125	117.838	116.111	108.940	91.145	91.576	31.627	11.991	1238.396
-47.875	117.694	116.533	109.914	90.630	91.339	31.507	11.746	1238.396
-47.625	118.829	116.675	109.919	91.984	92.160	31.582	12.040	1238.396
-47.375	118.339	116.335	109.530	91.848	91.502	31.915	11.806	1238.396
-47.125	119.391	116.461	109.862	91.972	92.727	32.317	11.886	1238.396
-46.875	119.075	116.495	110.613	92.604	92.521	31.884	11.869	1238.396
-46.625	120.019	117.463	110.295	91.707	93.273	32.541	12.203	1238.396
-46.375	120.335	117.993	110.004	92.132	92.989	31.957	12.105	1238.396
-46.125	119.247	117.836	110.763	93.190	93.143	32.074	12.231	1238.396
-45.875	120.740	117.608	111.111	93.117	94.317	31.753	12.596	1238.396
-45.625	119.838	117.707	111.381	93.564	93.573	32.214	12.425	1238.396
-45.375	119.989	118.149	111.169	93.000	93.882	32.244	12.071	1238.396
-45.125	120.574	118.647	112.354	93.187	92.625	32.099	12.154	1238.396
-44.875	120.596	119.584	111.859	93.557	93.454	32.611	11.931	1238.396
-44.625	120.086	118.570	112.672	93.897	93.859	32.488	12.151	1238.396
-44.375	121.555	119.170	113.127	94.000	94.023	32.912	11.960	1238.396
-44.125	121.388	118.932	112.402	93.523	94.559	32.468	12.242	1238.396
-43.875	121.548	118.703	112.411	93.640	93.768	32.133	12.034	1238.396
-43.625	121.438	119.069	113.001	94.663	93.815	32.731	12.376	1238.396
-43.375	121.421	118.735	111.644	94.406	94.033	32.161	11.829	1238.396
-43.125	121.127	119.136	113.103	94.136	94.736	32.806	12.311	1238.396
-42.875	121.843	119.648	113.347	94.836	93.981	32.558	12.316	1238.396
-42.625	121.016	118.458	113.116	93.939	94.745	32.583	12.305	1238.396
-42.375	121.818	119.706	113.313	93.657	95.057	32.831	12.496	1238.396
-42.125	121.982	120.599	113.459	94.814	95.136	32.507	12.479	1238.396
-41.875	121.117	119.251	113.244	94.355	94.666	32.332	12.228	1238.396
-41.625	121.762	120.350	113.715	95.067	95.101	33.178	11.948	1238.396
-41.375	122.149	119.503	112.882	94.478	95.522	33.077	12.274	1238.396
-41.125	122.308	119.954	113.027	94.712	94.978	33.295	12.339	1238.396
-40.875	122.164	120.731	112.922	95.609	95.352	33.052	12.285	1238.396
-40.625	122.651	119.644	113.929	95.553	94.696	33.038	12.482	1238.396
-40.375	122.734	119.961	113.835	94.549	95.463	32.812	12.505	1238.396
-40.125	121.829	120.230	113.015	95.167	96.047	33.231	12.613	1238.396
-39.875	122.062	120.759	113.799	95.094	94.495	32.742	12.063	1238.396
-39.625	124.017	120.659	114.149	94.853	94.669	32.194	12.542	1238.396
-39.375	121.741	121.459	113.815	94.435	94.792	33.201	12.602	1238.396
-39.125	121.609	120.188	113.671	96.047	95.185	32.720	12.157	1238.396
-38.875	122.974	121.523	113.866	95.181	95.680	33.650	12.362	1238.396
-38.625	122.581	121.352	113.448	94.855	94.849	33.382	12.642	1238.396
-38.375	122.383	120.832	114.628	94.836	95.800	32.876	12.559	1238.396
-38.125	122.461	121.385	113.413	95.563	95.089	32.988	12.291	1238.396
-37.875	122.604	120.447	113.618	95.033	94.510	33.047	12.168	1238.396

Midpoint	Ratio: 110	24.5	9.1	2.9	2.9	0.35	0.11	Volume
-37.625	121.637	120.848	113.593	95.932	96.002	33.192	12.673	1238.396
-37.375	123.421	120.284	114.642	94.863	95.458	32.781	12.767	1238.396
-37.125	122.136	121.097	113.613	94.880	95.149	33.044	12.414	1238.396
-36.875	122.644	121.827	114.181	96.066	95.347	33.195	12.759	1238.396
-36.625	122.650	120.234	114.308	95.062	95.601	33.203	12.562	1238.396
-36.375	123.191	120.228	113.981	95.983	96.324	33.427	12.705	1238.396
-36.125	122.444	120.079	115.084	95.390	94.611	33.111	12.513	1238.396
-35.875	121.679	121.294	114.362	95.232	95.312	33.159	12.533	1238.396
-35.625	122.453	121.364	113.473	94.887	95.475	33.508	12.291	1238.396
-35.375	123.001	120.787	113.671	94.960	95.079	32.689	12.696	1238.396
-35.125	122.855	121.390	114.078	95.794	95.688	32.784	12.408	1238.396
-34.875	122.792	120.084	114.151	94.836	95.562	33.069	12.282	1238.396
-34.625	122.563	120.320	114.040	95.546	95.735	33.273	12.145	1238.396
-34.375	122.928	120.131	115.022	95.526	96.220	33.773	12.111	1238.396
-34.125	123.337	120.167	113.634	96.112	94.567	33.360	12.305	1238.396
-33.875	121.418	121.107	112.474	95.925	95.023	33.103	12.528	1238.396
-33.625	122.636	120.265	112.785	95.453	94.847	33.164	12.531	1238.396
-33.375	122.991	121.128	114.550	95.628	95.532	33.463	12.194	1238.396
-33.125	122.843	120.537	113.678	95.266	95.421	32.965	12.422	1238.396
-32.875	121.674	120.328	113.799	95.004	95.253	33.566	12.502	1238.396
-32.625	121.818	120.542	113.318	95.487	95.723	32.823	12.447	1238.396
-32.375	122.779	120.941	111.996	95.098	94.921	32.667	12.522	1238.396
-32.125	121.277	119.791	113.456	95.342	95.114	33.055	12.057	1238.396
-31.875	122.755	119.707	114.065	95.541	95.987	32.912	12.393	1238.396
-31.625	122.827	121.187	113.318	95.227	94.881	33.035	12.154	1238.396
-31.375	121.409	120.093	113.487	95.006	94.938	33.081	12.265	1238.396
-31.125	121.764	119.056	113.421	94.938	95.614	32.773	12.476	1238.396
-30.875	121.793	119.067	112.995	95.113	94.557	33.190	12.163	1238.396
-30.625	121.615	120.034	113.017	95.490	94.684	32.231	12.185	1238.396
-30.375	121.202	119.690	112.968	93.708	94.382	32.412	12.174	1238.396
-30.125	121.016	120.067	112.975	93.817	95.131	33.133	12.029	1238.396
-29.875	121.140	119.280	112.939	94.330	94.956	32.826	12.223	1238.396
-29.625	121.594	119.901	111.907	94.405	94.367	32.675	12.456	1238.396
-29.375	121.850	119.302	112.949	94.036	94.114	32.871	12.299	1238.396
-29.125	121.207	118.734	112.827	94.114	94.552	32.795	12.228	1238.396
-28.875	120.872	118.605	111.122	94.413	94.013	32.368	12.080	1238.396
-28.625	120.342	119.197	111.714	93.683	95.166	32.686	12.319	1238.396
-28.375	120.265	118.055	112.107	93.781	94.174	32.689	12.020	1238.396
-28.125	120.183	118.108	112.378	94.420	93.535	32.865	12.687	1238.396
-27.875	120.216	119.321	112.839	94.216	94.584	32.885	12.431	1238.396
-27.625	120.397	118.421	112.523	94.189	93.731	32.457	12.003	1238.396
-27.375	121.793	118.718	112.137	93.170	93.889	32.569	12.342	1238.396
-27.125	122.054	119.253	111.734	93.978	93.645	32.270	12.023	1238.396
-26.875	119.953	119.212	112.461	92.670	94.018	32.711	12.203	1238.396
-26.625	121.028	117.827	111.676	93.837	94.409	32.709	12.146	1238.396

Midpoint	Ratio: 110	24.5	9.1	2.9	2.9	0.35	0.11	Volume
-26.375	119.907	118.886	112.228	93.805	94.438	32.893	12.274	1238.396
-26.125	120.141	119.471	112.499	94.255	94.755	32.694	12.086	1238.396
-25.875	121.458	119.849	112.369	94.364	94.345	32.935	12.097	1238.396
-25.625	121.704	119.202	112.762	95.215	94.498	32.834	12.371	1238.396
-25.375	122.037	120.155	113.606	95.060	94.545	32.356	12.342	1238.396
-25.125	122.175	119.442	113.386	95.398	95.579	32.410	12.394	1238.396
-24.875	122.417	120.588	114.068	95.434	95.602	32.857	12.414	1238.396
-24.625	122.761	121.156	114.231	95.563	95.660	33.184	12.074	1238.396
-24.375	123.444	120.859	114.629	96.321	95.698	32.940	12.622	1238.396
-24.125	124.588	121.238	114.667	95.602	96.116	33.734	12.650	1238.396
-23.875	124.470	122.317	115.285	96.759	96.428	33.080	12.642	1238.396
-23.625	124.535	122.570	116.475	97.488	97.155	34.341	12.542	1238.396
-23.375	125.250	123.482	116.014	97.384	96.529	34.022	12.807	1238.396
-23.125	125.623	123.771	116.221	97.865	98.137	33.251	12.530	1238.396
-22.875	126.264	125.928	117.726	98.534	99.751	33.712	12.565	1238.396
-22.625	127.132	126.154	117.507	99.144	99.293	34.296	12.750	1238.396
-22.375	128.622	126.621	118.259	99.832	99.909	34.486	12.770	1238.396
-22.125	129.348	126.579	120.674	100.566	99.807	34.709	12.808	1238.396
-21.875	130.590	128.458	120.187	102.020	101.248	35.417	12.556	1238.396
-21.625	130.963	129.565	121.798	101.870	102.083	34.782	12.958	1238.396
-21.375	132.840	130.083	123.026	104.107	104.197	35.631	12.742	1238.396
-21.125	133.368	131.027	124.494	104.238	105.048	36.023	12.967	1238.396
-20.875	135.009	132.020	125.466	104.233	105.423	35.967	13.230	1238.396
-20.625	136.090	133.686	125.736	105.167	105.131	36.462	13.312	1238.396
-20.375	137.636	134.358	125.831	107.134	107.564	37.208	13.358	1238.396
-20.125	137.201	135.765	128.152	107.744	106.626	36.319	12.819	1238.396
-19.875	42.133	41.602	39.042	32.981	33.578	11.333	4.128	1238.396
-19.625	15.587	15.055	14.422	12.302	12.361	4.237	1.386	5327.013
-19.375	13.410	13.089	12.468	10.656	10.505	3.631	1.269	122.850
-19.125	11.523	11.508	10.989	9.258	9.434	3.214	1.192	106.025
-18.875	9.854	9.434	9.081	7.758	7.771	2.652	0.804	93.806
-18.625	9.757	9.804	9.247	7.710	7.717	2.356	0.921	84.123
-18.375	17.060	16.820	15.686	11.935	12.192	2.264	0.596	76.111
-18.125	17.714	17.033	16.362	12.006	12.170	2.124	0.570	69.303
-17.875	16.645	16.384	15.053	11.208	11.475	2.037	0.465	63.415
-17.625	15.500	15.199	14.245	10.673	10.705	1.803	0.533	58.257
-17.375	14.836	14.126	13.400	10.129	10.074	1.797	0.431	53.695
-17.125	13.367	13.417	12.530	9.730	9.399	1.551	0.459	49.629
-16.875	12.776	12.549	11.612	8.694	8.922	1.476	0.445	45.985
-16.625	12.023	12.320	11.107	8.266	8.325	1.383	0.425	42.703
-16.375	11.362	11.100	10.248	7.894	7.944	1.294	0.331	39.735
-16.125	10.363	10.534	9.974	7.233	7.254	1.182	0.302	37.043
-15.875	9.670	9.761	9.063	7.107	7.086	1.040	0.260	34.594
-15.625	9.307	9.162	8.636	6.509	6.440	0.947	0.271	32.362
-15.375	8.872	8.738	8.184	5.920	6.034	0.978	0.223	30.323

Midpoint	Ratio: 110	24.5	9.1	2.9	2.9	0.35	0.11	Volume
-15.125	8.169	7.959	7.451	5.796	5.822	0.892	0.225	28.458
-14.875	7.694	7.993	7.278	5.354	5.344	0.878	0.211	26.751
-14.625	7.766	7.605	7.102	5.225	5.260	0.917	0.194	25.186
-14.375	7.366	7.278	6.965	5.334	5.401	0.875	0.177	23.750
-14.125	7.388	7.247	6.820	5.176	5.052	0.822	0.214	22.433
-13.875	7.314	7.021	6.701	5.145	5.141	0.777	0.205	21.223
-13.625	6.953	6.917	6.504	4.996	4.884	0.880	0.245	20.113
-13.375	6.777	6.824	6.196	4.751	4.730	0.838	0.174	19.094
-13.125	6.995	6.694	5.855	4.797	4.763	0.780	0.185	18.159
-12.875	6.250	6.280	5.948	4.634	4.597	0.771	0.222	17.302
-12.625	6.161	6.179	5.775	4.571	4.488	0.718	0.191	16.517
-12.375	6.280	6.265	5.831	4.403	4.332	0.805	0.185	15.798
-12.125	6.417	5.981	5.708	4.265	4.312	0.654	0.171	15.142
-11.875	5.989	6.156	5.573	4.240	4.305	0.766	0.165	14.545
-11.625	6.071	6.144	5.428	4.435	4.547	0.693	0.145	14.001
-11.375	6.011	5.890	5.452	4.211	4.154	0.646	0.126	13.509
-11.125	5.974	5.862	5.521	4.301	4.258	0.707	0.183	13.064
-10.875	6.280	6.118	5.643	4.440	4.298	0.752	0.174	12.665
-10.625	6.342	6.341	5.953	4.393	4.342	0.643	0.137	12.309
-10.375	6.777	6.628	6.044	4.619	4.780	0.771	0.174	11.994
-10.125	7.102	6.968	6.393	4.948	4.901	0.763	0.168	11.717
-9.875	7.209	7.217	6.963	5.077	5.262	0.956	0.137	11.478
-9.625	7.468	7.455	6.758	5.373	5.381	0.917	0.183	11.275
-9.375	7.152	7.252	6.879	5.113	5.213	0.992	0.191	11.106
-9.125	6.767	6.912	6.388	5.050	4.889	0.920	0.237	10.971
-8.875	5.914	5.968	5.501	4.201	4.439	0.855	0.154	10.869
-8.625	5.000	4.786	4.794	3.586	3.855	0.822	0.177	10.799
-8.375	1.936	1.798	1.705	1.342	1.482	0.450	0.120	10.761
-8.125	0.962	0.966	0.937	0.776	0.779	0.226	0.060	10.752
-7.875	0.683	0.781	0.678	0.571	0.584	0.176	0.054	10.752
-7.625	0.539	0.555	0.574	0.406	0.398	0.131	0.051	10.752
-7.375	0.417	0.378	0.323	0.311	0.322	0.095	0.029	10.752
-7.125	0.244	0.266	0.212	0.207	0.178	0.050	0.037	10.752
-6.875	0.211	0.165	0.132	0.129	0.148	0.050	0.014	10.752
-6.625	0.127	0.117	0.109	0.097	0.089	0.050	0.020	10.752
-6.375	0.070	0.117	0.065	0.056	0.059	0.020	0.023	10.752
-6.125	0.052	0.058	0.062	0.058	0.049	0.014	0.011	10.752
-5.875	0.052	0.038	0.039	0.024	0.015	0.006	0.009	10.752
-5.625	0.030	0.048	0.026	0.027	0.042	0.011	0.009	10.752
-5.375	0.027	0.038	0.023	0.027	0.035	0.008	0.006	10.752
-5.125	0.027	0.030	0.031	0.022	0.017	0.011	0.011	10.752
-4.875	0.030	0.036	0.018	0.024	0.010	0.006	0.000	10.752
-4.625	0.037	0.023	0.023	0.029	0.025	0.011	0.000	10.752
-4.375	0.042	0.028	0.041	0.017	0.032	0.003	0.003	10.752
-4.125	0.042	0.041	0.044	0.041	0.045	0.011	0.003	10.752

Midpoint	Ratio: 110	24.5	9.1	2.9	2.9	0.35	0.11	Volume
-3.875	0.037	0.051	0.031	0.027	0.022	0.020	0.006	10.752
-3.625	0.065	0.058	0.067	0.068	0.045	0.008	0.011	10.752
-3.375	0.075	0.096	0.096	0.063	0.054	0.008	0.011	10.752
-3.125	0.084	0.094	0.103	0.061	0.054	0.025	0.020	10.752
-2.875	0.114	0.167	0.114	0.124	0.089	0.017	0.009	10.752
-2.625	0.167	0.167	0.137	0.107	0.126	0.045	0.014	10.752
-2.375	0.204	0.208	0.212	0.170	0.171	0.048	0.017	10.752
-2.125	0.253	0.226	0.238	0.197	0.213	0.053	0.026	10.752
-1.875	0.268	0.244	0.259	0.185	0.198	0.092	0.020	10.752
-1.625	0.316	0.320	0.251	0.224	0.218	0.070	0.031	10.752
-1.375	0.278	0.261	0.248	0.195	0.218	0.078	0.020	10.752
-1.125	0.318	0.249	0.308	0.197	0.223	0.081	0.031	10.752
-0.875	0.263	0.251	0.267	0.214	0.235	0.042	0.026	10.752
-0.625	0.288	0.241	0.223	0.197	0.200	0.078	0.017	10.752
-0.125	0.239	0.210	0.215	0.170	0.153	0.059	0.006	10.752
0.125	0.189	0.200	0.181	0.134	0.188	0.036	0.020	10.752
0.625	0.219	0.134	0.191	0.134	0.171	0.039	0.029	10.752
0.875	0.157	0.178	0.166	0.122	0.119	0.017	0.017	10.752
1.125	0.124	0.147	0.137	0.109	0.119	0.042	0.026	10.752
1.375	0.152	0.147	0.119	0.102	0.096	0.039	0.006	10.752
1.625	0.144	0.114	0.147	0.100	0.106	0.028	0.006	10.752
1.875	0.157	0.129	0.122	0.085	0.089	0.045	0.009	10.752
2.125	0.099	0.107	0.132	0.122	0.114	0.028	0.006	10.752
2.375	0.147	0.150	0.111	0.097	0.104	0.034	0.026	10.752
2.625	0.139	0.137	0.124	0.095	0.119	0.025	0.014	10.752
2.875	0.154	0.122	0.116	0.102	0.114	0.061	0.011	10.752
3.125	0.152	0.150	0.158	0.136	0.139	0.048	0.003	10.752
3.375	0.174	0.183	0.137	0.124	0.158	0.034	0.014	10.752
3.625	0.184	0.185	0.155	0.146	0.109	0.045	0.020	10.752
3.875	0.179	0.162	0.155	0.129	0.109	0.034	0.011	10.752
4.125	0.152	0.162	0.142	0.095	0.109	0.031	0.014	10.752
4.375	0.114	0.122	0.111	0.095	0.101	0.034	0.003	10.752
4.625	0.082	0.104	0.093	0.068	0.101	0.039	0.014	10.752
4.875	0.065	0.074	0.080	0.053	0.062	0.014	0.003	10.752
5.125	0.082	0.056	0.057	0.046	0.054	0.014	0.009	10.752
5.375	0.067	0.063	0.062	0.058	0.047	0.011	0.006	10.752
5.625	0.062	0.066	0.062	0.039	0.049	0.020	0.011	10.752
5.875	0.067	0.069	0.049	0.066	0.035	0.006	0.003	10.752
6.125	0.070	0.035	0.044	0.034	0.040	0.011	0.003	10.752
6.375	0.060	0.056	0.075	0.058	0.047	0.011	0.003	10.752
6.625	0.089	0.081	0.075	0.083	0.049	0.017	0.014	10.752
6.875	0.084	0.101	0.106	0.078	0.079	0.028	0.003	10.752
7.125	0.164	0.134	0.140	0.105	0.116	0.034	0.009	10.752
7.375	0.181	0.223	0.173	0.168	0.151	0.050	0.006	10.752
7.625	0.323	0.302	0.316	0.209	0.203	0.092	0.020	10.752

Midpoint	Ratio: 110	24.5	9.1	2.9	2.9	0.35	0.11	Volume
7.875	0.477	0.477	0.411	0.350	0.334	0.117	0.046	10.752
8.125	0.661	0.616	0.618	0.491	0.455	0.190	0.043	10.752
8.375	1.250	1.375	1.136	0.980	1.079	0.291	0.071	10.761
8.625	3.976	3.942	3.483	2.881	2.830	0.671	0.114	10.799
8.875	4.828	4.858	4.567	3.506	3.731	0.721	0.126	10.869
9.125	6.200	5.806	5.557	4.469	4.421	0.886	0.208	10.971
9.375	6.521	6.765	6.134	4.970	4.874	0.950	0.231	11.106
9.625	7.130	7.262	6.833	5.118	5.210	0.959	0.222	11.275
9.875	7.167	6.928	6.830	5.208	5.220	0.844	0.191	11.478
10.125	7.105	6.582	6.360	5.006	4.698	0.718	0.146	11.717
10.375	6.606	6.775	5.977	4.595	4.614	0.671	0.143	11.994
10.625	6.210	6.369	5.886	4.299	4.421	0.749	0.151	12.309
10.875	5.939	5.961	5.508	4.301	4.152	0.662	0.171	12.665
11.125	6.091	6.085	5.609	4.248	4.377	0.710	0.154	13.064
11.375	6.044	5.923	5.532	4.437	4.253	0.634	0.151	13.509
11.625	6.225	5.915	5.625	4.425	4.211	0.604	0.183	14.001
11.875	6.084	5.816	5.591	4.316	4.062	0.648	0.108	14.545
12.125	6.136	6.275	5.705	4.277	4.310	0.721	0.165	15.142
12.375	6.188	6.364	5.837	4.423	4.401	0.785	0.180	15.798
12.625	6.312	6.240	5.889	4.610	4.545	0.749	0.203	16.517
12.875	6.613	6.311	6.062	4.566	4.295	0.738	0.163	17.302
13.125	6.526	6.562	6.290	4.641	4.621	0.769	0.205	18.159
13.375	6.790	6.826	6.194	4.615	4.656	0.785	0.183	19.094
13.625	6.852	6.920	6.328	4.724	4.889	0.693	0.208	20.113
13.875	7.055	7.019	6.712	5.016	4.899	0.897	0.228	21.223
14.125	7.214	7.039	6.859	5.108	5.099	0.704	0.205	22.433
14.375	7.664	7.599	6.831	5.179	5.270	0.880	0.160	23.750
14.625	7.764	7.660	7.051	5.444	5.381	0.822	0.200	25.186
14.875	8.084	7.501	7.314	5.752	5.327	0.880	0.222	26.751
15.125	8.109	8.254	7.827	5.461	5.710	0.866	0.223	28.458
15.375	8.855	8.558	8.404	5.908	6.052	0.922	0.234	30.323
15.625	9.163	9.195	8.455	6.419	6.339	1.068	0.237	32.362
15.875	10.050	9.550	8.836	6.808	6.695	1.244	0.243	34.594
16.125	10.574	10.207	9.637	7.311	7.229	1.252	0.285	37.043
16.375	10.922	10.895	10.264	7.946	7.900	1.272	0.357	39.735
16.625	12.080	11.706	10.991	8.089	8.254	1.512	0.362	42.703
16.875	12.607	12.835	11.640	8.779	8.875	1.453	0.391	45.985
17.125	13.586	13.330	12.442	9.582	9.451	1.615	0.465	49.629
17.375	14.337	14.364	13.118	10.061	10.020	1.789	0.431	53.695
17.625	15.296	15.027	14.310	10.659	10.527	1.881	0.496	58.257
17.875	16.327	15.892	14.695	11.366	11.304	1.833	0.579	63.415
18.125	17.368	17.340	16.008	11.986	11.618	1.982	0.531	69.303
18.375	17.182	16.871	15.938	11.943	12.106	2.222	0.627	76.111
18.625	9.968	10.055	9.276	7.595	7.840	2.459	0.830	84.123
18.875	9.878	9.647	8.905	7.564	7.432	2.697	0.921	93.806

Midpoint	Ratio: 110	24.5	9.1	2.9	2.9	0.35	0.11	Volume
19.125	11.725	11.475	10.988	9.322	9.382	3.200	1.101	106.025
19.375	13.504	13.109	12.570	10.678	10.394	3.667	1.284	122.850
19.625	15.567	14.890	14.766	12.115	12.254	4.229	1.506	5327.013
19.875	41.872	40.966	39.351	33.297	33.099	11.241	3.990	1238.396
20.125	136.897	134.756	126.720	106.618	107.709	37.060	13.104	1238.396
20.375	137.156	134.269	126.173	107.248	107.729	37.346	13.423	1238.396
20.625	135.954	132.760	125.950	106.458	106.188	36.460	13.332	1238.396
20.875	135.362	133.549	124.932	105.446	105.747	36.009	13.187	1238.396
21.125	133.128	130.654	124.502	104.204	102.996	35.733	12.887	1238.396
21.375	131.667	129.329	122.117	103.421	104.310	35.909	12.833	1238.396
21.625	129.933	129.357	120.694	101.549	102.235	35.185	12.613	1238.396
21.875	130.031	127.726	119.095	101.080	102.064	35.593	12.956	1238.396
22.125	128.670	126.606	119.467	100.408	100.367	34.707	12.639	1238.396
22.375	127.753	126.306	118.084	99.964	100.172	34.497	12.919	1238.396
22.625	127.156	125.210	117.868	100.100	98.907	34.657	12.576	1238.396
22.875	126.535	124.575	117.259	99.166	98.990	34.805	12.499	1238.396
23.125	125.062	124.503	117.660	97.678	98.677	34.067	12.436	1238.396
23.375	124.865	123.442	115.988	97.440	97.412	33.885	12.554	1238.396
23.625	123.464	121.845	115.161	97.498	97.227	33.254	12.445	1238.396
23.875	124.448	121.528	114.928	96.708	96.848	34.551	12.408	1238.396
24.125	123.406	121.348	114.306	96.467	96.695	32.991	12.322	1238.396
24.375	123.304	120.997	113.702	96.095	96.373	33.413	12.265	1238.396
24.625	122.301	120.803	114.343	95.237	95.678	32.686	12.613	1238.396
24.875	122.209	121.931	114.147	95.599	96.027	32.927	12.177	1238.396
25.125	121.773	120.439	113.749	94.255	95.443	33.058	12.587	1238.396
25.375	121.185	119.752	113.427	93.897	95.008	32.530	12.066	1238.396
25.625	122.179	119.945	112.745	94.177	94.924	32.639	12.339	1238.396
25.875	120.840	120.703	112.661	93.725	93.298	32.943	11.780	1238.396
26.125	121.110	118.724	112.762	93.603	94.570	32.622	12.323	1238.396
26.375	119.686	118.906	111.641	93.508	93.649	32.703	12.200	1238.396
26.625	121.485	118.389	111.545	93.564	94.647	32.876	12.174	1238.396
26.875	120.124	118.325	111.535	93.732	93.813	32.239	11.834	1238.396
27.125	120.562	118.651	112.227	92.767	94.755	32.563	12.017	1238.396
27.375	120.417	120.059	112.701	93.754	92.781	32.801	12.456	1238.396
27.625	119.935	119.057	111.899	94.902	93.669	32.608	12.171	1238.396
27.875	121.402	118.572	111.419	94.145	94.259	32.507	12.362	1238.396
28.125	119.851	117.891	111.829	93.917	93.540	32.124	12.026	1238.396
28.375	120.526	119.775	112.409	93.832	94.790	32.795	12.040	1238.396
28.625	120.267	119.176	112.461	93.982	93.941	32.284	12.245	1238.396
28.875	121.026	118.554	111.227	93.873	94.347	32.614	12.062	1238.396
29.125	121.147	118.840	112.667	94.123	94.278	32.569	12.519	1238.396
29.375	121.312	119.501	112.372	93.844	93.995	33.069	12.088	1238.396
29.625	121.383	118.991	113.005	94.320	94.008	32.736	12.337	1238.396
29.875	121.670	119.495	114.065	95.111	94.743	32.560	12.442	1238.396
30.125	120.763	119.555	112.405	94.982	94.857	33.203	12.354	1238.396

Midpoint	Ratio: 110	24.5	9.1	2.9	2.9	0.35	0.11	Volume
30.375	121.659	119.911	112.545	94.658	94.703	33.281	11.934	1238.396
30.625	122.047	119.138	112.936	94.724	94.683	32.929	12.496	1238.396
30.875	122.077	120.176	114.228	94.792	94.179	32.969	12.308	1238.396
31.125	123.016	120.539	112.832	95.050	95.151	33.320	12.277	1238.396
31.375	122.370	119.539	113.186	95.098	94.367	32.927	12.348	1238.396
31.625	121.990	120.172	113.995	95.023	94.753	32.832	12.342	1238.396
31.875	121.272	119.661	113.738	95.213	95.596	32.230	12.140	1238.396
32.125	122.623	120.236	112.890	95.067	95.089	32.734	12.576	1238.396
32.375	123.146	120.978	113.295	96.258	94.186	33.195	12.664	1238.396
32.625	122.469	120.872	113.196	94.824	95.743	32.898	12.665	1238.396
32.875	122.101	119.858	114.445	95.823	96.316	32.910	12.451	1238.396
33.125	122.723	120.537	113.027	96.312	95.790	33.234	12.028	1238.396
33.375	122.089	120.908	113.565	95.573	94.993	32.857	12.562	1238.396
33.625	122.820	120.674	113.878	95.006	94.943	33.759	12.471	1238.396
33.875	122.327	120.548	113.724	95.594	96.153	32.600	12.411	1238.396
34.125	121.938	120.320	115.053	95.050	96.138	32.879	12.188	1238.396
34.375	122.193	120.571	114.158	94.624	96.455	33.572	12.394	1238.396
34.625	123.180	120.126	114.868	95.371	96.148	33.298	12.513	1238.396
34.875	122.875	121.321	113.595	95.565	95.500	33.150	12.804	1238.396
35.125	121.123	120.975	113.598	95.782	96.391	33.089	12.682	1238.396
35.375	122.940	121.649	114.336	95.162	95.327	32.586	12.719	1238.396
35.625	122.333	120.554	114.205	94.792	95.525	33.181	12.174	1238.396
35.875	123.577	120.563	113.738	95.590	95.497	33.458	12.610	1238.396
36.125	123.009	120.984	114.356	96.348	95.267	32.740	12.565	1238.396
36.375	123.272	120.174	113.841	94.644	95.923	32.731	12.439	1238.396
36.625	122.698	120.870	114.286	95.159	95.675	32.910	12.502	1238.396
36.875	123.615	120.524	115.166	95.808	95.060	32.955	12.585	1238.396
37.125	121.975	121.236	114.195	95.337	96.304	33.282	12.282	1238.396
37.375	123.374	120.665	113.342	94.836	95.478	32.876	12.664	1238.396
37.625	121.687	121.936	113.284	95.796	95.663	33.111	12.428	1238.396
37.875	121.584	121.246	114.022	95.171	96.223	33.360	12.488	1238.396
38.125	122.499	120.927	113.376	95.716	96.534	32.795	12.248	1238.396
38.375	121.611	121.066	113.889	95.453	95.869	32.821	12.753	1238.396
38.625	122.082	120.546	113.422	95.906	96.138	33.357	12.802	1238.396
38.875	122.107	121.183	113.653	95.291	94.530	33.231	12.787	1238.396
39.125	123.252	120.739	113.313	95.473	95.213	32.831	12.548	1238.396
39.375	122.747	120.906	113.409	94.194	95.755	32.863	12.590	1238.396
39.625	123.679	121.127	114.486	95.789	95.510	32.748	12.454	1238.396
39.875	122.989	120.433	113.564	94.712	94.768	32.851	12.525	1238.396
40.125	121.888	119.815	113.453	95.602	95.322	33.469	12.230	1238.396
40.375	122.849	119.808	112.703	94.770	96.024	32.737	12.516	1238.396
40.625	122.789	119.827	113.727	94.736	94.147	32.893	12.368	1238.396
40.875	122.055	119.605	114.294	95.322	94.511	33.170	12.034	1238.396
41.125	121.709	121.475	111.967	94.880	95.442	32.622	12.325	1238.396
41.375	121.662	119.391	112.824	94.850	94.834	32.547	12.493	1238.396

Midpoint	Ratio: 110	24.5	9.1	2.9	2.9	0.35	0.11	Volume
41.625	122.989	119.942	113.279	95.264	93.612	32.633	12.399	1238.396
41.875	121.485	120.050	111.661	95.658	94.510	32.870	12.117	1238.396
42.125	122.327	119.563	113.194	94.782	94.404	33.041	12.422	1238.396
42.375	121.003	119.319	112.783	94.668	95.572	32.561	12.326	1238.396
42.625	121.617	119.136	113.035	93.941	94.347	32.219	12.234	1238.396
42.875	121.657	118.502	112.457	94.814	94.686	33.117	12.516	1238.396
43.125	122.003	119.109	112.368	93.343	94.864	32.588	12.205	1238.396
43.375	120.370	119.468	112.157	94.542	95.218	32.664	12.425	1238.396
43.625	121.767	119.707	113.024	94.838	94.273	33.178	12.251	1238.396
43.875	120.601	119.361	112.163	94.031	95.406	32.683	12.225	1238.396
44.125	121.124	119.499	111.788	93.362	93.810	32.616	12.162	1238.396
44.375	119.918	119.062	112.377	93.404	92.457	32.323	11.908	1238.396
44.625	119.992	118.839	112.864	93.824	93.707	32.977	12.205	1238.396
44.875	120.665	119.683	112.442	94.150	93.068	32.382	12.516	1238.396
45.125	120.125	118.062	110.590	92.570	93.054	32.493	12.025	1238.396
45.375	120.478	118.749	111.401	93.469	92.821	32.195	12.254	1238.396
45.625	121.259	117.200	111.542	92.837	93.528	31.582	12.040	1238.396
45.875	120.755	118.591	110.810	92.606	92.499	32.088	12.279	1238.396
46.125	119.098	117.418	110.067	92.003	92.956	32.144	12.003	1238.396
46.375	119.596	116.557	111.089	93.083	92.650	32.242	11.880	1238.396
46.625	119.222	117.460	110.600	93.012	91.912	32.561	12.005	1238.396
46.875	119.460	117.477	110.828	91.935	92.390	31.837	11.815	1238.396
47.125	118.567	116.546	110.790	92.538	91.967	31.691	12.223	1238.396
47.375	118.466	117.431	110.665	91.972	92.278	31.473	11.906	1238.396
47.625	118.570	117.618	109.129	91.889	91.670	32.113	12.009	1238.396
47.875	118.138	116.479	109.841	91.879	91.709	31.915	11.957	1238.396
48.125	117.693	116.140	109.217	91.427	92.096	31.641	11.917	1238.396
48.375	118.053	114.592	109.399	91.843	90.522	31.549	11.743	1238.396
48.625	117.665	114.684	109.316	90.664	91.148	31.507	11.797	1238.396
48.875	116.396	113.798	107.952	90.377	91.403	31.283	11.989	1238.396
49.125	116.958	115.005	107.132	90.141	90.473	30.831	11.629	1238.396
49.375	116.324	114.133	107.378	89.978	90.707	30.903	11.771	1238.396
49.625	115.799	113.801	107.090	89.059	89.904	30.937	11.789	1238.396
49.875	115.507	113.913	106.595	89.455	90.007	30.492	11.669	1238.396
50.125	115.944	113.069	107.754	89.324	89.313	30.741	11.421	1238.396
50.375	115.066	112.509	106.442	89.032	88.667	30.573	11.350	1238.396
50.625	114.152	113.056	106.126	88.709	88.380	30.484	11.846	1238.396
50.875	114.107	112.952	106.205	88.291	89.315	29.877	11.618	1238.396
51.125	114.887	113.116	107.113	88.950	89.381	30.431	11.207	1238.396
51.375	114.758	112.513	107.297	88.602	88.222	30.481	11.655	1238.396
51.625	114.954	111.163	105.352	88.950	88.540	30.380	11.441	1238.396
51.875	113.968	112.276	104.737	88.043	88.429	30.274	11.618	1238.396
52.125	113.956	111.555	105.714	87.736	88.459	30.274	11.444	1238.396
52.375	113.948	111.411	105.221	88.901	88.276	30.377	11.458	1238.396
52.625	112.872	111.486	105.842	87.814	87.820	30.517	11.621	1238.396

Midpoint	Ratio: 110	24.5	9.1	2.9	2.9	0.35	0.11	Volume
52.875	113.669	111.828	105.093	87.479	88.033	30.026	11.121	1238.396
53.125	113.856	112.105	104.504	87.294	88.486	30.040	11.792	1238.396
53.375	113.538	110.785	105.367	87.282	87.662	30.372	11.307	1238.396
53.625	114.181	112.035	105.215	87.459	87.756	29.914	11.096	1238.396
53.875	114.102	112.667	104.923	86.932	88.162	30.612	11.293	1238.396
54.125	114.208	111.641	106.255	88.432	87.452	30.162	11.370	1238.396
54.375	113.197	112.393	105.423	87.518	88.355	30.559	11.515	1238.396
54.625	114.135	112.486	104.944	87.612	87.813	30.378	11.238	1238.396
54.875	114.319	111.468	105.715	87.615	88.453	30.268	11.501	1238.396
55.125	114.679	111.545	104.737	87.775	86.870	31.012	11.418	1238.396
55.375	113.743	112.338	104.949	88.398	87.924	30.285	11.295	1238.396
55.625	114.492	111.692	105.573	88.181	88.459	30.291	11.384	1238.396
55.875	114.124	111.952	104.874	89.081	88.399	30.272	11.732	1238.396
56.125	114.239	111.530	104.679	88.087	88.206	30.310	11.098	1238.396
56.375	114.070	112.038	106.033	88.194	88.827	30.604	11.429	1238.396
56.625	113.930	111.999	105.389	87.924	89.070	30.372	11.595	1238.396
56.875	114.758	111.064	105.841	88.592	87.867	30.316	11.204	1238.396
57.125	114.266	111.416	105.278	87.535	88.389	30.143	11.501	1238.396
57.375	114.530	111.579	105.784	87.289	88.144	30.350	11.746	1238.396
57.625	114.515	111.563	104.810	87.666	88.662	30.534	11.849	1238.396
57.875	113.772	111.754	105.539	88.371	88.384	30.227	11.489	1238.396
58.125	72.180	71.089	66.925	55.485	55.912	19.514	7.274	1238.396
58.375	0.000	0.000	0.000	0.000	0.000	0.000	0.000	1238.396

Table of K^+ population data for simulation with K^+ and Rb^+ across bulk density ratios (#JJF-GRS1459)

	Population in histogram bin at Rb^+/K^+ ratio:							
Midpoint	110	24.5	9.1	2.9	2.9	0.35	0.11	Volume
-58.375	0.000	0.000	0.000	0.000	0.000	0.000	0.000	1244.016
-58.125	1.061	4.568	11.705	30.277	29.966	87.888	104.418	1244.016
-57.875	1.036	4.601	11.198	29.652	30.258	88.383	105.270	1244.016
-57.625	0.972	4.553	11.558	30.202	30.308	87.902	104.985	1244.016
-57.375	1.036	4.475	11.439	30.287	30.320	87.664	104.902	1244.016
-57.125	1.054	4.330	11.330	30.374	30.471	87.153	105.764	1244.016
-56.875	1.086	4.492	11.617	30.562	30.513	88.497	105.994	1244.016
-56.625	0.969	4.622	11.710	30.175	30.625	86.994	104.544	1244.016
-56.375	0.992	4.561	11.412	30.231	30.842	87.511	104.506	1244.016
-56.125	1.091	4.566	11.467	30.523	30.382	87.338	104.714	1244.016
-55.875	1.004	4.564	11.412	30.391	30.256	87.941	104.269	1244.016
-55.625	1.026	4.670	11.317	30.727	30.548	87.709	105.262	1244.016
-55.375	1.086	4.543	11.729	30.601	29.832	88.165	104.972	1244.016
-55.125	1.084	4.710	11.751	30.411	30.434	88.805	104.565	1244.016
-54.875	1.034	4.426	11.599	29.616	30.681	87.844	105.600	1244.016
-54.625	0.969	4.398	11.458	30.156	30.614	87.877	104.932	1244.016
-54.375	1.081	4.604	11.418	30.615	30.639	87.871	104.399	1244.016

Midpoint	Ratio: 110	24.5	9.1	2.9	2.9	0.35	0.11	Volume
-54.125	1.066	4.609	11.630	30.117	30.342	86.854	104.817	1244.016
-53.875	1.089	4.538	11.596	29.954	29.964	87.405	104.476	1244.016
-53.625	0.979	4.469	11.498	30.494	30.350	87.329	103.840	1244.016
-53.375	0.982	4.662	11.498	30.486	30.738	88.640	104.827	1244.016
-53.125	0.979	4.322	11.555	30.788	30.622	87.603	105.435	1244.016
-52.875	0.964	4.457	11.356	30.005	30.634	87.751	103.433	1244.016
-52.625	1.136	4.518	11.545	30.652	30.161	87.223	106.199	1244.016
-52.375	1.064	4.512	11.514	29.998	29.969	87.997	104.996	1244.016
-52.125	1.103	4.632	11.749	30.639	30.224	87.798	105.041	1244.016
-51.875	0.992	4.556	11.604	30.666	30.083	87.782	105.172	1244.016
-51.625	1.083	4.611	11.806	30.797	30.778	88.732	105.812	1244.016
-51.375	1.019	4.515	11.610	30.756	31.027	88.394	105.381	1244.016
-51.125	0.989	4.665	11.656	30.579	30.953	87.925	106.465	1244.016
-50.875	1.143	4.525	11.491	30.404	30.523	88.536	105.510	1244.016
-50.625	1.016	4.715	11.599	30.477	30.686	88.544	106.288	1244.016
-50.375	0.992	4.558	11.281	31.371	30.711	88.509	106.351	1244.016
-50.125	1.168	4.640	11.846	30.797	31.194	88.505	105.781	1244.016
-49.875	1.076	4.637	11.844	30.999	30.958	89.861	107.055	1244.016
-49.625	1.126	4.929	11.767	30.861	30.874	90.135	107.649	1244.016
-49.375	0.964	4.484	11.697	31.364	31.287	91.077	107.731	1244.016
-49.125	1.004	4.675	11.992	31.383	31.141	91.122	108.208	1244.016
-48.875	1.158	4.680	11.583	31.665	31.961	91.703	107.446	1244.016
-48.625	1.044	4.533	12.165	31.116	31.624	91.957	108.949	1244.016
-48.375	0.984	4.804	12.097	32.290	31.809	90.985	108.136	1244.016
-48.125	1.098	4.805	11.832	31.814	31.475	91.340	108.709	1244.016
-47.875	1.034	4.599	11.951	31.447	32.089	92.142	108.515	1244.016
-47.625	1.128	4.561	11.914	31.084	31.418	91.994	109.502	1244.016
-47.375	1.086	4.736	12.305	31.731	31.540	92.821	109.562	1244.016
-47.125	1.118	4.431	12.355	32.203	31.837	91.952	109.959	1244.016
-46.875	1.019	4.647	12.365	31.602	32.272	93.081	110.395	1244.016
-46.625	1.101	4.855	12.225	32.011	31.997	92.796	111.325	1244.016
-46.375	1.081	4.751	12.321	31.651	31.973	92.052	110.963	1244.016
-46.125	1.069	4.670	12.059	31.994	32.299	92.927	112.104	1244.016
-45.875	1.059	4.668	12.121	32.336	32.596	92.915	110.844	1244.016
-45.625	1.059	4.936	12.463	32.380	32.650	92.421	111.315	1244.016
-45.375	1.116	4.807	12.468	32.417	32.646	93.338	111.442	1244.016
-45.125	1.180	4.865	12.360	31.904	32.012	93.519	111.627	1244.016
-44.875	1.163	4.530	12.078	32.186	32.623	93.813	111.493	1244.016
-44.625	1.133	4.632	12.370	32.735	31.856	93.073	111.656	1244.016
-44.375	1.138	4.987	12.453	33.090	32.386	94.045	112.038	1244.016
-44.125	1.103	5.010	12.308	32.419	32.740	93.788	112.361	1244.016
-43.875	1.086	4.766	12.217	32.504	32.712	94.275	112.734	1244.016
-43.625	1.166	4.847	12.569	32.565	33.240	94.858	112.262	1244.016
-43.375	1.103	5.030	12.489	33.307	32.599	94.269	112.238	1244.016
-43.125	1.123	5.040	12.339	32.743	32.824	94.205	113.216	1244.016

Midpoint	Ratio: 110	24.5	9.1	2.9	2.9	0.35	0.11	Volume
-42.875	1.059	5.022	12.466	33.042	32.967	94.227	112.611	1244.016
-42.625	1.041	5.182	12.256	32.665	32.668	95.255	112.745	1244.016
-42.375	1.213	4.804	12.308	33.068	33.296	94.120	113.849	1244.016
-42.125	1.108	4.911	12.114	32.930	32.856	94.238	113.793	1244.016
-41.875	1.086	4.787	12.592	32.927	32.977	95.759	113.062	1244.016
-41.625	1.108	5.129	12.400	32.876	33.274	95.297	112.855	1244.016
-41.375	1.170	5.010	12.453	33.375	33.559	94.588	113.895	1244.016
-41.125	1.203	4.913	12.258	32.952	33.358	95.302	113.480	1244.016
-40.875	1.049	4.807	12.763	32.786	33.029	94.831	113.408	1244.016
-40.625	1.133	5.015	12.523	33.010	33.155	95.666	113.918	1244.016
-40.375	1.148	4.901	12.310	32.888	32.737	95.258	113.410	1244.016
-40.125	1.101	4.928	12.688	32.789	33.603	96.127	113.689	1244.016
-39.875	1.171	4.827	12.225	33.282	33.145	95.479	114.024	1244.016
-39.625	1.188	4.763	12.394	33.185	33.232	94.246	114.415	1244.016
-39.375	1.175	4.954	12.251	32.867	33.089	95.909	112.911	1244.016
-39.125	1.161	4.992	12.512	33.110	33.361	94.705	113.422	1244.016
-38.875	1.111	4.934	12.328	33.146	33.346	94.785	113.883	1244.016
-38.625	1.195	4.825	12.293	32.983	33.687	95.820	113.787	1244.016
-38.375	1.156	4.946	12.432	33.044	32.950	95.102	112.994	1244.016
-38.125	1.200	5.099	12.621	33.525	33.395	95.154	113.893	1244.016
-37.875	1.098	4.972	12.579	33.423	33.742	94.431	114.149	1244.016
-37.625	1.158	4.954	12.326	33.243	32.754	95.342	113.633	1244.016
-37.375	1.143	4.710	12.381	32.867	33.153	96.301	114.500	1244.016
-37.125	1.183	4.680	12.445	33.290	33.069	94.523	113.491	1244.016
-36.875	1.136	5.018	12.634	33.056	32.987	94.528	113.796	1244.016
-36.625	1.168	4.911	12.471	33.280	33.022	95.443	114.925	1244.016
-36.375	1.118	5.091	12.357	33.102	33.215	95.870	114.389	1244.016
-36.125	1.064	4.769	12.577	32.832	33.529	95.633	114.059	1244.016
-35.875	1.081	4.967	12.336	33.987	33.093	94.928	114.899	1244.016
-35.625	1.180	4.807	12.712	33.457	33.625	97.089	113.430	1244.016
-35.375	1.141	4.977	12.414	33.518	33.094	95.272	113.805	1244.016
-35.125	1.205	4.931	12.572	33.003	32.935	95.882	114.766	1244.016
-34.875	1.131	4.794	12.805	32.728	32.994	96.172	114.220	1244.016
-34.625	1.074	4.774	12.848	33.243	33.381	95.554	114.344	1244.016
-34.375	1.146	4.995	12.890	33.226	33.346	95.588	113.955	1244.016
-34.125	1.133	4.845	12.585	33.231	33.200	95.918	114.095	1244.016
-33.875	1.086	4.781	12.606	33.518	32.621	95.865	113.778	1244.016
-33.625	1.111	4.797	12.150	33.406	32.750	94.663	113.809	1244.016
-33.375	1.121	4.632	12.455	33.282	33.400	95.163	113.864	1244.016
-33.125	1.029	4.583	12.590	32.478	33.573	95.082	113.932	1244.016
-32.875	1.002	4.878	12.458	33.156	33.536	95.484	113.445	1244.016
-32.625	1.074	4.941	12.779	33.418	32.997	95.149	113.742	1244.016
-32.375	1.051	4.934	12.445	32.850	33.163	95.286	113.830	1244.016
-32.125	1.061	4.738	12.489	32.730	33.217	94.881	114.138	1244.016
-31.875	1.203	4.817	12.316	33.382	32.866	95.431	113.242	1244.016

Midpoint	Ratio: 110	24.5	9.1	2.9	2.9	0.35	0.11	Volume
-31.625	1.116	4.744	12.360	33.469	32.653	96.203	113.045	1244.016
-31.375	1.106	4.865	12.600	33.027	33.551	94.872	111.348	1244.016
-31.125	0.997	4.731	12.551	33.034	33.502	94.345	113.416	1244.016
-30.875	1.228	4.834	12.262	33.025	33.259	94.897	113.302	1244.016
-30.625	1.113	4.687	12.194	33.375	32.646	95.568	112.768	1244.016
-30.375	1.131	4.895	12.442	32.186	32.631	94.272	112.888	1244.016
-30.125	0.977	4.738	12.150	32.976	33.207	94.355	113.080	1244.016
-29.875	1.014	4.822	12.266	32.738	33.366	94.065	113.097	1244.016
-29.625	1.198	4.936	12.019	32.344	32.799	94.372	113.433	1244.016
-29.375	1.084	4.792	12.329	32.504	33.255	94.788	112.569	1244.016
-29.125	1.071	4.883	12.595	32.682	32.153	94.425	112.869	1244.016
-28.875	1.046	4.799	12.028	32.833	33.041	94.950	111.679	1244.016
-28.625	1.141	4.609	12.497	32.922	32.176	94.371	112.970	1244.016
-28.375	0.982	4.853	12.212	32.312	32.621	94.375	111.309	1244.016
-28.125	1.041	4.875	12.471	32.409	32.784	93.903	112.221	1244.016
-27.875	1.148	4.893	12.176	32.840	32.972	93.995	111.976	1244.016
-27.625	1.121	4.683	11.982	32.064	32.789	93.179	111.006	1244.016
-27.375	1.151	4.885	12.274	32.713	32.322	94.255	111.747	1244.016
-27.125	1.111	4.951	12.018	32.614	32.804	93.388	110.986	1244.016
-26.875	1.158	4.612	12.315	32.550	32.398	94.710	111.583	1244.016
-26.625	1.081	4.784	12.314	32.796	33.064	94.004	112.275	1244.016
-26.375	1.031	4.776	12.072	32.677	32.678	94.319	112.132	1244.016
-26.125	1.089	4.804	12.406	32.677	32.693	94.014	111.926	1244.016
-25.875	1.039	4.860	12.274	32.935	32.819	94.855	112.318	1244.016
-25.625	1.059	4.705	12.311	32.738	33.017	94.445	111.809	1244.016
-25.375	1.011	4.733	12.417	32.446	33.145	95.303	112.315	1244.016
-25.125	1.004	4.733	12.207	32.691	33.034	93.997	112.899	1244.016
-24.875	1.126	4.814	12.561	33.416	32.022	95.365	113.562	1244.016
-24.625	1.039	4.675	12.233	33.752	32.816	95.876	112.969	1244.016
-24.375	0.969	4.670	12.561	33.353	33.999	95.527	113.710	1244.016
-24.125	0.979	4.685	12.417	32.820	33.784	95.674	113.609	1244.016
-23.875	0.939	4.787	12.564	33.910	33.561	96.468	114.731	1244.016
-23.625	1.024	4.695	12.812	34.055	33.764	96.256	114.942	1244.016
-23.375	0.912	4.916	12.657	33.824	34.016	97.852	115.412	1244.016
-23.125	1.054	4.880	12.675	34.136	34.375	96.566	116.311	1244.016
-22.875	1.069	4.908	12.727	34.316	34.377	98.176	117.413	1244.016
-22.625	1.034	4.824	12.983	34.571	34.397	99.059	118.433	1244.016
-22.375	1.004	4.878	12.986	34.257	34.630	98.371	118.122	1244.016
-22.125	1.021	4.779	12.888	34.588	34.482	100.557	118.279	1244.016
-21.875	0.957	4.934	12.874	34.982	35.320	100.865	119.458	1244.016
-21.625	0.880	4.906	12.887	35.407	34.989	101.705	118.884	1244.016
-21.375	0.924	4.886	12.760	35.539	35.612	103.341	121.977	1244.016
-21.125	0.927	4.649	13.097	35.582	36.208	102.570	122.915	1244.016
-20.875	0.999	4.718	13.345	36.353	36.557	104.031	123.808	1244.016
-20.625	0.964	4.749	13.172	36.941	36.792	105.719	125.207	1244.016

Midpoint	Ratio: 110	24.5	9.1	2.9	2.9	0.35	0.11	Volume
-20.375	0.907	4.946	12.978	36.035	36.508	105.965	126.121	1244.016
-20.125	0.937	4.797	12.981	37.014	36.859	107.969	127.132	1244.016
-19.875	0.527	3.018	8.245	23.083	23.163	67.077	78.489	1244.016
-19.625	0.104	0.502	1.537	4.386	4.448	13.072	15.266	5648.482
-19.375	0.087	0.434	1.343	3.812	3.988	11.087	13.055	131.318
-19.125	0.082	0.444	1.286	3.564	3.343	9.360	11.438	111.609
-18.875	0.055	0.289	0.960	2.701	2.754	8.435	9.538	98.132
-18.625	0.047	0.320	0.838	2.580	2.454	7.870	9.778	87.699
-18.375	0.055	0.241	0.699	2.300	2.437	12.174	15.377	79.176
-18.125	0.030	0.216	0.574	2.305	2.019	12.493	16.173	71.991
-17.875	0.035	0.162	0.647	2.013	1.878	11.671	15.234	65.812
-17.625	0.055	0.218	0.510	2.069	1.885	10.992	14.521	60.420
-17.375	0.025	0.155	0.528	1.734	1.762	10.453	14.014	55.666
-17.125	0.032	0.152	0.504	1.804	1.724	9.827	12.827	51.439
-16.875	0.025	0.127	0.435	1.673	1.564	8.812	12.114	47.657
-16.625	0.037	0.142	0.398	1.430	1.445	8.625	11.270	44.256
-16.375	0.032	0.137	0.352	1.427	1.418	8.052	10.371	41.184
-16.125	0.032	0.160	0.336	1.267	1.309	7.594	10.117	38.400
-15.875	0.017	0.114	0.316	1.194	1.195	7.079	9.148	35.869
-15.625	0.020	0.089	0.251	1.002	1.047	6.406	8.686	33.564
-15.375	0.017	0.101	0.243	0.934	0.970	6.291	7.847	31.460
-15.125	0.022	0.099	0.256	0.907	0.896	5.956	7.679	29.537
-14.875	0.017	0.096	0.194	0.873	0.901	5.769	7.402	27.776
-14.625	0.017	0.096	0.210	0.895	0.898	5.445	7.336	26.162
-14.375	0.012	0.089	0.210	0.800	0.846	5.347	6.943	24.682
-14.125	0.012	0.066	0.220	0.890	0.920	5.305	6.911	23.324
-13.875	0.015	0.068	0.191	0.849	0.886	5.126	6.951	22.078
-13.625	0.032	0.074	0.212	0.849	0.858	5.067	6.595	20.933
-13.375	0.017	0.063	0.243	0.829	0.844	4.958	6.446	19.883
-13.125	0.012	0.056	0.228	0.817	0.789	4.894	6.021	18.920
-12.875	0.015	0.074	0.191	0.771	0.807	4.723	6.287	18.036
-12.625	0.007	0.056	0.204	0.790	0.752	4.307	5.833	17.227
-12.375	0.012	0.051	0.171	0.703	0.760	4.458	6.061	16.487
-12.125	0.017	0.056	0.178	0.712	0.658	4.589	5.736	15.810
-11.875	0.007	0.061	0.137	0.664	0.713	4.517	5.805	15.194
-11.625	0.010	0.071	0.158	0.744	0.532	4.380	5.904	14.634
-11.375	0.012	0.063	0.166	0.615	0.675	4.203	5.859	14.126
-11.125	0.017	0.035	0.129	0.652	0.720	4.262	5.839	13.668
-10.875	0.005	0.043	0.147	0.639	0.698	4.268	5.773	13.256
-10.625	0.005	0.058	0.150	0.725	0.784	4.371	5.978	12.889
-10.375	0.010	0.048	0.124	0.715	0.737	4.721	6.158	12.563
-10.125	0.007	0.041	0.178	0.749	0.760	5.042	6.572	12.278
-9.875	0.007	0.074	0.197	0.921	0.777	5.489	6.840	12.031
-9.625	0.025	0.074	0.202	0.977	0.975	5.573	7.276	11.821
-9.375	0.010	0.066	0.241	1.058	1.074	5.509	7.048	11.647

Midpoint	Ratio: 110	24.5	9.1	2.9	2.9	0.35	0.11	Volume
-9.125	0.017	0.086	0.210	1.053	1.007	5.319	6.791	11.508
-8.875	0.010	0.068	0.217	0.934	0.943	5.009	6.093	11.402
-8.625	0.005	0.066	0.163	0.861	0.920	4.368	5.251	11.330
-8.375	0.010	0.041	0.142	0.464	0.428	1.610	1.985	11.291
-8.125	0.007	0.053	0.093	0.309	0.282	0.939	1.110	11.282
-7.875	0.005	0.033	0.072	0.236	0.240	0.685	0.947	11.282
-7.625	0.002	0.013	0.057	0.209	0.161	0.604	0.665	11.282
-7.375	0.002	0.018	0.065	0.158	0.134	0.428	0.513	11.282
-7.125	0.005	0.015	0.031	0.112	0.119	0.302	0.357	11.282
-6.875	0.010	0.010	0.041	0.066	0.092	0.201	0.217	11.282
-6.625	0.002	0.010	0.013	0.053	0.069	0.126	0.148	11.282
-6.375	0.000	0.000	0.008	0.019	0.047	0.095	0.128	11.282
-6.125	0.002	0.000	0.010	0.029	0.022	0.115	0.074	11.282
-5.875	0.002	0.003	0.003	0.019	0.012	0.039	0.068	11.282
-5.625	0.005	0.003	0.008	0.005	0.007	0.039	0.091	11.282
-5.375	0.000	0.003	0.005	0.010	0.005	0.042	0.046	11.282
-5.125	0.000	0.000	0.003	0.005	0.010	0.025	0.057	11.282
-4.875	0.000	0.005	0.005	0.017	0.010	0.028	0.066	11.282
-4.625	0.000	0.000	0.005	0.017	0.012	0.039	0.054	11.282
-4.375	0.000	0.003	0.003	0.024	0.017	0.059	0.043	11.282
-4.125	0.000	0.003	0.010	0.017	0.017	0.056	0.083	11.282
-3.875	0.002	0.010	0.010	0.022	0.012	0.078	0.091	11.282
-3.625	0.002	0.010	0.016	0.032	0.035	0.098	0.123	11.282
-3.375	0.000	0.000	0.005	0.039	0.030	0.092	0.168	11.282
-3.125	0.000	0.005	0.026	0.029	0.054	0.145	0.174	11.282
-2.875	0.000	0.013	0.018	0.073	0.067	0.159	0.220	11.282
-2.625	0.000	0.015	0.028	0.068	0.077	0.246	0.305	11.282
-2.375	0.007	0.018	0.039	0.097	0.082	0.310	0.371	11.282
-2.125	0.002	0.013	0.036	0.129	0.116	0.369	0.385	11.282
-1.875	0.007	0.008	0.026	0.095	0.099	0.310	0.442	11.282
-1.625	0.005	0.018	0.044	0.105	0.087	0.347	0.405	11.282
-1.375	0.005	0.020	0.034	0.107	0.096	0.389	0.428	11.282
-1.125	0.005	0.018	0.041	0.105	0.136	0.313	0.396	11.282
-0.875	0.000	0.013	0.034	0.107	0.124	0.271	0.388	11.282
-0.625	0.000	0.010	0.036	0.100	0.109	0.333	0.359	11.282
-0.125	0.000	0.015	0.054	0.066	0.082	0.229	0.288	11.282
0.125	0.005	0.008	0.034	0.066	0.094	0.226	0.254	11.282
0.625	0.000	0.013	0.018	0.097	0.092	0.198	0.274	11.282
0.875	0.000	0.010	0.034	0.075	0.067	0.182	0.260	11.282
1.125	0.000	0.008	0.010	0.053	0.074	0.170	0.242	11.282
1.375	0.000	0.010	0.021	0.071	0.057	0.176	0.242	11.282
1.625	0.000	0.008	0.021	0.046	0.077	0.170	0.154	11.282
1.875	0.002	0.003	0.021	0.056	0.040	0.182	0.214	11.282
2.125	0.000	0.013	0.023	0.066	0.049	0.154	0.220	11.282
2.375	0.002	0.005	0.013	0.068	0.064	0.134	0.168	11.282

Midpoint	Ratio: 110	24.5	9.1	2.9	2.9	0.35	0.11	Volume
2.625	0.000	0.008	0.026	0.041	0.059	0.170	0.211	11.282
2.875	0.000	0.003	0.026	0.066	0.062	0.207	0.220	11.282
3.125	0.000	0.010	0.013	0.051	0.067	0.173	0.211	11.282
3.375	0.000	0.000	0.034	0.058	0.069	0.218	0.197	11.282
3.625	0.002	0.000	0.023	0.083	0.047	0.201	0.260	11.282
3.875	0.000	0.010	0.028	0.044	0.082	0.243	0.211	11.282
4.125	0.002	0.003	0.018	0.053	0.042	0.198	0.188	11.282
4.375	0.000	0.008	0.021	0.061	0.049	0.151	0.205	11.282
4.625	0.005	0.015	0.023	0.044	0.032	0.140	0.143	11.282
4.875	0.002	0.000	0.016	0.039	0.037	0.120	0.163	11.282
5.125	0.000	0.005	0.008	0.029	0.020	0.084	0.137	11.282
5.375	0.000	0.003	0.023	0.034	0.020	0.087	0.103	11.282
5.625	0.000	0.000	0.000	0.032	0.025	0.070	0.117	11.282
5.875	0.000	0.003	0.013	0.041	0.027	0.073	0.080	11.282
6.125	0.005	0.008	0.008	0.032	0.037	0.056	0.117	11.282
6.375	0.000	0.003	0.008	0.044	0.027	0.078	0.123	11.282
6.625	0.000	0.013	0.005	0.034	0.045	0.087	0.163	11.282
6.875	0.000	0.005	0.023	0.053	0.057	0.140	0.183	11.282
7.125	0.002	0.003	0.018	0.061	0.054	0.207	0.191	11.282
7.375	0.005	0.013	0.034	0.071	0.092	0.201	0.288	11.282
7.625	0.002	0.013	0.047	0.114	0.116	0.330	0.405	11.282
7.875	0.005	0.015	0.047	0.187	0.156	0.486	0.596	11.282
8.125	0.010	0.015	0.065	0.267	0.168	0.604	0.693	11.282
8.375	0.010	0.030	0.122	0.306	0.336	1.258	1.492	11.291
8.625	0.015	0.051	0.168	0.739	0.735	3.536	4.182	11.330
8.875	0.007	0.079	0.197	0.802	0.948	4.156	5.206	11.402
9.125	0.010	0.069	0.210	0.990	0.854	4.665	6.261	11.508
9.375	0.012	0.071	0.233	1.038	0.925	5.226	6.814	11.647
9.625	0.007	0.086	0.220	0.960	1.004	5.447	7.219	11.821
9.875	0.005	0.071	0.199	0.941	0.849	5.601	6.948	12.031
10.125	0.010	0.074	0.150	0.812	0.829	4.905	6.523	12.278
10.375	0.020	0.043	0.140	0.693	0.725	4.774	6.215	12.563
10.625	0.020	0.041	0.215	0.649	0.735	4.505	5.762	12.889
10.875	0.012	0.048	0.145	0.605	0.601	4.304	6.021	13.256
11.125	0.010	0.061	0.158	0.588	0.626	4.159	5.716	13.668
11.375	0.017	0.046	0.142	0.661	0.705	4.229	5.691	14.126
11.625	0.012	0.048	0.176	0.630	0.648	4.380	5.668	14.634
11.875	0.012	0.048	0.135	0.705	0.675	4.357	5.882	15.194
12.125	0.012	0.046	0.186	0.759	0.762	4.525	5.856	15.810
12.375	0.017	0.051	0.194	0.819	0.732	4.559	5.631	16.487
12.625	0.007	0.074	0.223	0.763	0.802	4.830	6.198	17.227
12.875	0.012	0.086	0.215	0.846	0.824	4.913	6.335	18.036
13.125	0.022	0.099	0.212	0.812	0.846	4.972	6.389	18.920
13.375	0.010	0.048	0.202	0.800	0.888	5.131	6.406	19.883
13.625	0.017	0.079	0.186	0.912	0.863	4.989	6.846	20.933

Midpoint	Ratio: 110	24.5	9.1	2.9	2.9	0.35	0.11	Volume
13.875	0.020	0.063	0.228	0.853	0.861	5.140	6.646	22.078
14.125	0.017	0.081	0.192	0.795	0.757	5.321	6.746	23.324
14.375	0.012	0.096	0.241	0.890	0.804	5.369	6.846	24.682
14.625	0.015	0.063	0.236	0.909	0.876	5.525	7.068	26.162
14.875	0.027	0.071	0.251	0.941	0.901	5.464	7.145	27.776
15.125	0.010	0.084	0.207	0.897	0.923	5.662	7.753	29.537
15.375	0.030	0.091	0.282	0.956	0.955	6.112	8.095	31.460
15.625	0.025	0.079	0.274	1.109	1.094	6.459	8.720	33.564
15.875	0.020	0.094	0.292	1.135	1.202	6.970	9.330	35.869
16.125	0.015	0.122	0.347	1.296	1.220	7.538	9.929	38.400
16.375	0.030	0.099	0.321	1.410	1.408	7.812	10.594	41.184
16.625	0.032	0.145	0.367	1.469	1.383	8.427	10.902	44.256
16.875	0.025	0.142	0.445	1.675	1.608	9.265	11.891	47.657
17.125	0.030	0.152	0.471	1.726	1.769	9.542	12.859	51.439
17.375	0.030	0.165	0.461	1.916	1.826	10.481	13.494	55.666
17.625	0.042	0.178	0.502	1.845	1.873	10.760	14.125	60.420
17.875	0.037	0.216	0.572	1.843	2.068	11.649	15.289	65.812
18.125	0.027	0.206	0.590	2.251	2.076	12.406	15.993	71.991
18.375	0.037	0.233	0.562	2.288	2.293	12.314	15.666	79.176
18.625	0.077	0.243	0.898	2.458	2.392	7.597	9.606	87.699
18.875	0.062	0.380	0.887	2.789	2.746	8.172	9.444	98.132
19.125	0.082	0.403	1.097	3.307	3.360	9.852	11.463	111.609
19.375	0.117	0.500	1.392	3.980	3.743	11.093	13.104	131.318
19.625	0.089	0.558	1.573	4.581	4.453	12.541	14.904	5648.482
19.875	0.517	2.871	8.370	22.750	23.088	67.382	78.313	1244.016
20.125	0.892	4.754	13.058	36.898	36.794	107.041	126.200	1244.016
20.375	0.892	4.746	13.255	36.776	36.936	106.018	125.518	1244.016
20.625	0.929	4.751	13.399	36.224	36.656	104.928	124.667	1244.016
20.875	0.957	4.875	13.444	36.146	36.357	104.967	123.705	1244.016
21.125	0.967	4.873	12.779	35.711	35.355	103.986	122.347	1244.016
21.375	0.979	4.787	12.761	35.453	36.104	102.863	121.602	1244.016
21.625	0.989	5.028	12.525	35.436	34.840	101.029	120.005	1244.016
21.875	1.016	4.744	12.701	34.765	35.147	101.125	119.823	1244.016
22.125	0.962	4.906	12.838	34.989	34.479	99.814	117.954	1244.016
22.375	0.964	4.738	12.691	34.379	34.788	98.799	118.181	1244.016
22.625	0.917	4.817	12.324	34.522	34.425	99.649	117.087	1244.016
22.875	0.944	4.771	12.658	34.488	34.687	98.064	117.185	1244.016
23.125	1.004	4.903	12.396	33.756	33.573	97.766	115.681	1244.016
23.375	0.947	4.622	12.492	33.983	33.888	97.804	114.911	1244.016
23.625	0.982	4.898	12.554	33.581	33.601	96.497	115.235	1244.016
23.875	1.044	4.794	12.277	33.309	33.274	95.643	114.885	1244.016
24.125	0.967	4.761	12.694	33.268	32.777	96.337	114.271	1244.016
24.375	1.039	4.911	12.762	32.913	33.079	95.839	114.854	1244.016
24.625	1.086	4.880	12.116	33.146	33.262	94.518	114.366	1244.016
24.875	0.939	4.748	12.536	33.311	33.457	94.626	113.790	1244.016

Midpoint	Ratio: 110	24.5	9.1	2.9	2.9	0.35	0.11	Volume
25.125	1.084	4.890	12.362	32.881	33.108	95.876	112.287	1244.016
25.375	1.131	4.708	12.228	33.100	33.222	94.233	112.761	1244.016
25.625	1.041	4.505	12.360	32.871	32.411	94.338	112.413	1244.016
25.875	1.061	4.746	12.005	33.020	33.148	95.121	111.633	1244.016
26.125	1.024	4.662	12.528	32.485	32.290	94.205	112.061	1244.016
26.375	1.061	4.812	12.323	32.560	32.695	94.581	110.972	1244.016
26.625	1.049	4.690	11.975	32.509	32.715	93.939	111.850	1244.016
26.875	1.034	4.900	12.119	33.066	32.388	93.268	111.562	1244.016
27.125	1.113	4.703	12.489	32.409	32.379	93.534	112.164	1244.016
27.375	1.034	4.738	12.393	32.743	32.697	93.332	111.570	1244.016
27.625	1.138	4.675	12.067	31.911	32.240	93.928	113.000	1244.016
27.875	1.091	4.774	12.235	32.096	32.871	93.693	112.278	1244.016
28.125	1.126	4.609	11.761	32.750	32.792	93.601	111.100	1244.016
28.375	1.049	4.977	12.186	33.229	33.331	93.531	111.912	1244.016
28.625	0.997	4.563	12.456	32.903	32.329	93.679	110.949	1244.016
28.875	1.158	4.713	12.251	33.168	32.544	94.003	112.619	1244.016
29.125	1.121	4.896	12.310	32.954	32.532	94.562	112.269	1244.016
29.375	1.071	4.885	12.509	32.708	32.769	94.786	113.417	1244.016
29.625	1.026	4.809	12.386	32.706	33.430	94.727	112.598	1244.016
29.875	1.213	5.012	12.235	32.857	32.905	95.233	112.672	1244.016
30.125	1.084	4.835	12.137	32.961	32.836	95.230	113.296	1244.016
30.375	1.024	5.012	12.223	33.122	33.333	94.537	112.760	1244.016
30.625	1.098	4.776	12.287	33.204	32.814	94.470	113.353	1244.016
30.875	1.076	4.885	12.218	32.942	33.363	95.138	113.243	1244.016
31.125	1.019	4.688	12.574	32.713	32.871	96.089	113.482	1244.016
31.375	1.108	4.969	12.339	33.153	33.027	95.825	112.922	1244.016
31.625	1.185	4.758	11.958	33.341	33.138	94.895	112.617	1244.016
31.875	1.143	4.883	12.453	32.769	33.242	95.529	112.988	1244.016
32.125	1.111	4.868	12.520	32.910	32.611	95.917	113.214	1244.016
32.375	1.200	4.698	12.600	33.122	32.754	94.878	113.263	1244.016
32.625	1.188	5.005	12.554	32.638	33.163	94.721	113.606	1244.016
32.875	1.054	4.967	12.166	33.282	33.118	94.269	113.305	1244.016
33.125	1.076	4.835	12.590	32.903	32.678	95.426	114.485	1244.016
33.375	1.165	5.045	12.321	33.676	33.131	95.278	114.355	1244.016
33.625	1.093	4.909	12.556	33.076	33.432	95.960	114.298	1244.016
33.875	1.066	4.893	12.960	33.469	33.610	95.147	113.609	1244.016
34.125	1.121	4.774	12.802	33.406	33.022	95.957	114.034	1244.016
34.375	1.158	4.969	12.528	33.175	33.559	94.883	114.285	1244.016
34.625	1.153	4.660	12.287	33.586	32.938	95.493	113.159	1244.016
34.875	1.153	4.972	12.367	33.270	33.178	95.398	112.797	1244.016
35.125	1.088	5.126	12.461	33.234	33.123	95.839	113.428	1244.016
35.375	1.153	4.944	12.439	33.277	33.492	95.300	113.966	1244.016
35.625	1.121	5.197	12.706	32.954	33.269	95.113	114.294	1244.016
35.875	1.098	4.989	12.662	33.377	32.789	96.390	113.627	1244.016
36.125	1.096	4.944	12.404	32.869	33.603	95.734	113.735	1244.016

Midpoint	Ratio: 110	24.5	9.1	2.9	2.9	0.35	0.11	Volume
36.375	1.046	5.017	12.572	33.815	33.712	94.900	113.956	1244.016
36.625	1.151	4.987	12.401	33.355	32.925	95.727	114.055	1244.016
36.875	1.061	4.867	12.502	33.034	33.203	96.063	113.435	1244.016
37.125	1.138	4.840	12.549	33.511	33.168	95.957	114.204	1244.016
37.375	1.163	5.068	12.375	33.338	32.571	95.286	114.210	1244.016
37.625	1.151	5.028	12.168	33.056	33.281	96.077	114.177	1244.016
37.875	1.034	4.951	12.541	33.336	33.932	94.579	113.610	1244.016
38.125	1.160	4.835	12.535	33.078	32.571	95.582	114.221	1244.016
38.375	1.146	4.789	12.691	33.175	33.007	96.638	113.758	1244.016
38.625	1.171	4.977	12.231	33.102	33.126	95.172	113.657	1244.016
38.875	1.113	4.994	12.489	32.864	33.281	95.331	113.524	1244.016
39.125	1.086	4.939	12.694	32.582	32.955	95.261	113.296	1244.016
39.375	1.193	4.868	12.538	33.484	33.506	94.325	113.887	1244.016
39.625	1.121	4.994	12.350	32.300	33.083	95.809	114.415	1244.016
39.875	1.098	4.840	12.580	33.178	33.514	96.013	112.485	1244.016
40.125	1.021	4.931	12.313	33.136	33.220	95.795	113.077	1244.016
40.375	1.123	4.817	12.331	32.716	32.522	95.283	113.683	1244.016
40.625	1.091	4.726	12.665	32.660	33.034	95.345	113.625	1244.016
40.875	1.073	4.916	12.805	33.584	33.076	95.339	113.513	1244.016
41.125	1.180	4.830	12.155	32.961	33.385	95.367	112.871	1244.016
41.375	1.146	5.010	12.427	33.129	32.720	95.096	112.783	1244.016
41.625	1.081	4.972	12.430	32.910	33.343	94.912	113.456	1244.016
41.875	1.123	4.926	12.155	33.100	32.992	94.255	113.434	1244.016
42.125	1.079	4.685	12.269	32.730	32.806	95.250	113.097	1244.016
42.375	1.086	4.848	12.750	32.626	32.369	94.045	112.438	1244.016
42.625	1.056	4.883	12.481	32.443	33.039	93.768	112.635	1244.016
42.875	1.151	4.873	12.313	32.623	32.908	93.922	112.591	1244.016
43.125	1.175	4.979	12.619	32.550	33.390	94.884	112.799	1244.016
43.375	1.101	4.888	12.201	32.794	32.586	94.696	112.626	1244.016
43.625	1.136	4.974	12.751	32.473	32.910	94.411	111.918	1244.016
43.875	1.098	4.792	12.202	32.840	32.718	93.279	112.495	1244.016
44.125	1.118	4.858	12.109	32.380	32.183	94.165	111.799	1244.016
44.375	1.128	4.847	12.166	32.905	32.515	93.640	111.066	1244.016
44.625	1.175	4.962	12.440	32.453	32.913	94.514	112.233	1244.016
44.875	1.059	4.926	12.393	32.922	32.138	94.448	112.170	1244.016
45.125	1.069	4.705	12.494	32.504	32.022	92.672	112.157	1244.016
45.375	1.163	4.710	12.171	32.091	32.121	92.989	111.388	1244.016
45.625	1.116	4.761	11.920	32.319	32.472	91.908	110.656	1244.016
45.875	1.156	4.789	12.326	32.446	32.062	92.771	111.822	1244.016
46.125	1.076	4.807	12.367	32.655	32.769	92.731	110.141	1244.016
46.375	1.153	4.863	12.199	31.867	32.265	91.949	111.025	1244.016
46.625	1.074	5.040	12.091	32.045	31.508	92.933	110.678	1244.016
46.875	1.096	4.726	11.891	32.183	32.030	92.608	109.536	1244.016
47.125	1.128	4.614	12.168	32.123	31.745	92.357	110.935	1244.016
47.375	1.151	4.703	11.927	31.634	31.572	93.212	109.763	1244.016

Midpoint	Ratio: 110	24.5	9.1	2.9	2.9	0.35	0.11	Volume
47.625	1.098	4.870	12.044	31.561	32.050	92.892	110.056	1244.016
47.875	1.031	4.591	12.034	32.040	31.386	91.767	109.426	1244.016
48.125	1.131	4.599	11.979	31.923	31.552	92.639	109.168	1244.016
48.375	1.079	4.721	11.948	31.349	32.047	91.113	108.644	1244.016
48.625	1.074	4.710	11.979	31.131	31.218	91.295	108.004	1244.016
48.875	1.059	4.814	11.915	31.595	31.347	91.857	108.054	1244.016
49.125	1.086	4.685	11.938	31.403	30.558	90.601	107.554	1244.016
49.375	1.138	4.771	12.197	31.033	30.872	90.549	106.580	1244.016
49.625	1.143	4.520	11.829	30.759	31.045	89.481	106.850	1244.016
49.875	1.044	4.806	11.689	30.887	31.035	88.950	107.046	1244.016
50.125	1.108	4.388	11.759	31.094	30.966	90.311	106.474	1244.016
50.375	1.049	4.513	11.345	31.264	31.102	89.718	107.107	1244.016
50.625	1.106	4.518	11.762	30.948	30.642	89.031	105.980	1244.016
50.875	1.066	4.477	11.529	30.503	30.661	88.595	105.871	1244.016
51.125	1.116	4.533	11.677	30.579	31.065	88.532	104.737	1244.016
51.375	1.021	4.675	11.544	30.306	30.538	88.679	105.541	1244.016
51.625	1.056	4.533	11.356	30.151	30.353	89.297	105.906	1244.016
51.875	1.081	4.376	11.627	30.603	30.587	87.975	105.721	1244.016
52.125	0.999	4.589	11.534	30.943	29.941	88.123	106.009	1244.016
52.375	1.093	4.543	11.633	30.156	30.820	88.313	104.528	1244.016
52.625	1.101	4.441	11.576	30.953	30.454	88.198	105.241	1244.016
52.875	1.126	4.472	11.622	30.501	30.312	88.173	104.987	1244.016
53.125	1.014	4.568	11.733	30.350	30.753	88.218	105.512	1244.016
53.375	1.044	4.652	11.563	30.102	30.231	87.961	104.624	1244.016
53.625	1.133	4.553	11.374	30.192	30.481	87.816	104.603	1244.016
53.875	1.039	4.558	11.645	30.630	30.728	88.572	105.458	1244.016
54.125	1.094	4.624	11.522	30.481	30.006	87.930	105.711	1244.016
54.375	1.054	4.513	11.296	30.469	30.666	88.617	105.243	1244.016
54.625	0.922	4.434	11.475	29.915	29.949	88.383	104.143	1244.016
54.875	1.024	4.383	11.268	29.954	29.682	86.739	104.340	1244.016
55.125	0.937	4.505	11.560	30.080	30.016	87.480	106.171	1244.016
55.375	1.046	4.535	11.837	30.394	29.870	87.466	105.576	1244.016
55.625	1.106	4.373	11.630	30.260	30.399	86.949	105.129	1244.016
55.875	1.086	4.599	11.196	30.802	30.172	88.291	104.747	1244.016
56.125	1.086	4.459	11.278	30.287	30.797	88.184	104.605	1244.016
56.375	0.962	4.360	11.472	30.557	30.800	87.544	104.719	1244.016
56.625	1.019	4.574	11.351	30.681	30.441	88.377	105.412	1244.016
56.875	1.056	4.627	11.521	30.690	30.575	88.335	104.151	1244.016
57.125	1.026	4.586	11.201	30.610	30.511	87.757	105.340	1244.016
57.375	1.101	4.683	11.250	30.053	30.226	88.178	104.941	1244.016
57.625	1.061	4.429	11.452	30.221	30.171	88.321	104.990	1244.016
57.875	0.967	4.546	11.369	30.075	30.295	87.936	105.289	1244.016
58.125	1.061	4.571	11.309	29.694	30.169	87.695	103.290	1244.016
58.375	0.000	0.000	0.000	0.000	0.000	0.000	0.000	1244.016

Midpoint	Ratio: 1860	657	189	62	19	6.3	2.1	0.65	0.13	Volume

E.4. NavMs channel: Sodium and Potassium

Table of Na^{+} population data for simulation with K^{+} and Na^{+} across bulk density ratios

	Population in histogram bin at K^{+}/Na^{+} ratio:									
Midpoint	1860	657	189	62	19	6.3	2.1	0.65	0.13	Volume
-99.7	0.000	0.000	0.000	2.906	0.000	0.001	0.002	0.003	0.006	4443.486
-99.5	0.226	0.705	2.522	7.860	24.714	66.312	154.278	293.365	412.763	4443.486
-99.3	0.259	0.774	2.547	7.823	25.199	68.682	158.786	298.665	422.477	4443.486
-99.1	0.234	0.796	2.668	7.738	25.166	68.540	155.874	300.374	424.102	4443.486
-98.9	0.263	0.820	2.622	7.877	25.179	67.756	158.706	302.881	419.616	4443.486
-98.7	0.249	0.731	2.745	7.757	25.060	68.412	158.523	300.835	422.733	4443.486
-98.5	0.227	0.687	2.602	7.865	25.032	68.184	158.725	295.158	421.644	4443.486
-98.3	0.262	0.834	2.634	7.833	25.237	67.653	156.945	299.912	420.874	4443.486
-98.1	0.275	0.791	2.630	7.902	25.195	67.816	158.310	298.636	420.068	4443.486
-97.9	0.269	0.813	2.670	7.950	25.262	67.944	156.835	298.498	422.651	4443.486
-97.7	0.254	0.749	2.655	7.828	25.290	68.777	158.510	302.941	420.240	4443.486
-97.5	0.271	0.749	2.610	7.761	25.332	68.440	157.227	300.520	422.950	4443.486
-97.3	0.248	0.819	2.725	7.738	25.356	67.814	156.855	297.569	421.063	4443.486
-97.1	0.236	0.754	2.606	7.743	25.204	68.333	158.378	300.409	420.964	4443.486
-96.9	0.261	0.840	2.678	7.923	25.417	68.382	159.314	297.680	422.720	4443.486
-96.7	0.244	0.792	2.654	7.966	25.232	68.183	158.075	299.585	423.394	4443.486
-96.5	0.250	0.782	2.649	7.767	25.457	68.731	158.807	295.959	423.164	4443.486
-96.3	0.289	0.743	2.706	7.862	25.511	68.726	158.789	298.541	423.888	4443.486
-96.1	0.242	0.803	2.680	7.841	25.367	67.440	156.777	296.069	427.225	4443.486
-95.9	0.243	0.819	2.666	7.744	25.374	68.452	158.109	302.209	423.871	4443.486
-95.7	0.253	0.701	2.695	7.738	25.457	68.380	157.262	300.071	428.384	4443.486
-95.5	0.292	0.833	2.657	7.904	25.229	68.656	160.107	300.638	425.866	4443.486
-95.3	0.264	0.745	2.731	7.823	25.664	68.778	157.809	303.595	424.997	4443.486
-95.1	0.273	0.777	2.655	7.808	25.617	69.397	158.837	298.837	428.047	4443.486
-94.9	0.220	0.798	2.639	7.844	25.628	68.617	160.242	303.190	426.741	4443.486
-94.7	0.251	0.766	2.720	7.892	25.504	68.858	157.825	304.581	425.275	4443.486
-94.5	0.231	0.763	2.623	7.881	25.500	69.284	158.269	302.156	428.403	4443.486
-94.3	0.265	0.764	2.616	7.891	25.281	69.589	159.229	303.372	427.842	4443.486
-94.1	0.272	0.714	2.709	7.891	25.515	69.483	159.742	302.164	427.397	4443.486
-93.9	0.256	0.766	2.585	8.025	25.291	69.100	159.349	302.436	427.113	4443.486
-93.7	0.254	0.772	2.651	7.911	25.701	68.629	160.799	304.626	428.732	4443.486
-93.5	0.250	0.755	2.746	7.940	25.601	68.605	159.658	302.230	424.748	4443.486
-93.3	0.295	0.766	2.707	8.043	25.529	68.942	161.971	299.408	428.153	4443.486
-93.1	0.238	0.758	2.599	7.744	25.358	69.760	161.225	304.962	430.665	4443.486
-92.9	0.239	0.810	2.645	7.928	25.429	68.981	159.649	305.480	425.343	4443.486
-92.7	0.262	0.799	2.679	7.940	25.373	69.124	160.807	304.045	429.183	4443.486
-92.5	0.229	0.794	2.708	7.905	25.391	68.817	159.839	300.528	429.845	4443.486

Midpoint	Ratio: 1860	657	189	62	19	6.3	2.1	0.65	0.13	Volume
-92.3	0.242	0.766	2.570	7.926	25.249	69.343	160.748	300.788	431.050	4443.486
-92.1	0.264	0.721	2.717	8.049	25.546	68.827	160.064	304.465	427.527	4443.486
-91.9	0.281	0.817	2.615	7.889	25.527	68.916	160.102	308.364	428.569	4443.486
-91.7	0.264	0.806	2.740	7.927	25.442	69.104	159.828	301.598	429.199	4443.486
-91.5	0.254	0.762	2.698	7.924	25.712	69.231	161.391	305.363	429.345	4443.486
-91.3	0.274	0.807	2.671	8.049	25.243	69.155	159.603	301.710	428.832	4443.486
-91.1	0.281	0.832	2.739	7.814	25.183	69.007	159.892	304.705	431.423	4443.486
-90.9	0.261	0.761	2.603	7.858	25.470	68.433	159.126	305.736	430.455	4443.486
-90.7	0.263	0.771	2.668	7.912	25.497	69.244	159.873	303.184	431.006	4443.486
-90.5	0.254	0.824	2.745	7.925	25.429	69.573	160.919	303.314	428.552	4443.486
-90.3	0.214	0.765	2.693	7.954	25.625	69.002	160.698	308.355	429.660	4443.486
-90.1	0.245	0.744	2.729	7.976	25.574	69.100	161.243	304.040	429.420	4443.486
-89.9	0.264	0.795	2.636	8.000	25.578	68.874	158.740	301.431	430.572	4443.486
-89.7	0.243	0.783	2.583	7.939	25.376	68.709	160.164	302.438	427.087	4443.486
-89.5	0.250	0.774	2.681	7.868	25.347	69.005	158.831	305.739	431.365	4443.486
-89.3	0.248	0.764	2.632	7.835	25.451	69.267	160.429	305.373	430.213	4443.486
-89.1	0.270	0.814	2.696	7.856	25.394	68.803	160.722	302.141	428.710	4443.486
-88.9	0.247	0.743	2.772	8.032	25.420	69.112	159.762	300.133	430.031	4443.486
-88.7	0.251	0.778	2.685	7.894	25.416	69.446	160.651	306.280	429.249	4443.486
-88.5	0.244	0.808	2.656	7.885	25.619	68.372	158.284	306.832	428.594	4443.486
-88.3	0.246	0.793	2.792	8.042	25.629	69.671	160.610	305.011	428.220	4443.486
-88.1	0.220	0.811	2.782	8.022	25.463	68.873	159.639	303.953	432.276	4443.486
-87.9	0.289	0.794	2.788	7.919	25.403	68.473	158.835	306.408	428.303	4443.486
-87.7	0.208	0.801	2.645	7.990	25.595	69.168	159.654	306.995	430.626	4443.486
-87.5	0.267	0.834	2.673	8.053	25.465	69.112	158.931	298.313	430.779	4443.486
-87.3	0.252	0.776	2.580	7.875	25.704	69.407	160.077	305.011	433.280	4443.486
-87.1	0.270	0.809	2.661	8.047	25.625	68.352	160.564	306.202	428.712	4443.486
-86.9	0.266	0.838	2.769	7.986	25.490	68.762	161.175	305.263	430.227	4443.486
-86.7	0.277	0.842	2.631	7.813	25.535	69.175	160.107	304.738	431.046	4443.486
-86.5	0.262	0.747	2.708	7.814	25.390	68.765	159.769	306.251	429.963	4443.486
-86.3	0.267	0.814	2.660	7.732	25.814	69.418	159.948	304.826	427.350	4443.486
-86.1	0.264	0.754	2.722	7.857	25.383	69.505	159.991	302.217	430.752	4443.486
-85.9	0.271	0.804	2.727	7.956	25.469	69.099	159.965	302.264	430.845	4443.486
-85.7	0.239	0.714	2.629	7.976	25.296	68.899	160.542	303.954	428.201	4443.486
-85.5	0.270	0.844	2.739	8.003	25.167	69.208	159.291	303.748	429.414	4443.486
-85.3	0.289	0.775	2.653	8.038	25.644	69.066	159.363	305.572	427.044	4443.486
-85.1	0.270	0.729	2.743	7.795	25.415	68.952	158.219	302.117	427.924	4443.486
-84.9	0.252	0.812	2.592	8.034	25.379	69.563	159.129	305.743	427.114	4443.486
-84.7	0.237	0.786	2.665	7.752	25.249	69.348	159.212	304.885	425.817	4443.486
-84.5	0.288	0.799	2.688	7.820	25.566	68.951	159.648	304.162	431.245	4443.486
-84.3	0.275	0.764	2.613	7.802	25.705	68.838	160.097	304.549	428.933	4443.486
-84.1	0.250	0.765	2.584	7.750	25.574	68.769	159.296	304.100	428.218	4443.486
-83.9	0.263	0.842	2.572	7.893	25.511	69.135	159.847	301.984	428.354	4443.486
-83.7	0.266	0.823	2.678	7.875	25.516	68.508	160.193	302.417	430.154	4443.486

Midpoint	Ratio: 1860	657	189	62	19	6.3	2.1	0.65	0.13	Volume
-83.5	0.258	0.763	2.704	7.951	25.485	68.897	160.455	304.644	428.300	4443.486
-83.3	0.245	0.738	2.680	7.957	25.565	69.326	158.422	304.390	427.154	4443.486
-83.1	0.245	0.807	2.647	7.912	25.279	69.571	159.337	302.250	429.478	4443.486
-82.9	0.287	0.789	2.683	7.913	25.252	68.937	160.006	306.197	429.122	4443.486
-82.7	0.217	0.810	2.611	7.782	25.509	68.833	158.497	302.819	428.556	4443.486
-82.5	0.247	0.790	2.721	7.876	25.447	68.827	160.567	305.040	429.824	4443.486
-82.3	0.231	0.777	2.601	7.966	25.606	68.394	158.533	303.517	431.757	4443.486
-82.1	0.268	0.755	2.624	7.852	25.328	68.987	158.883	302.422	430.533	4443.486
-81.9	0.268	0.870	2.474	7.797	25.429	69.443	159.616	305.261	427.924	4443.486
-81.7	0.245	0.806	2.528	7.707	25.300	68.740	158.461	304.907	431.465	4443.486
-81.5	0.251	0.829	2.616	7.854	25.411	69.293	159.698	303.661	429.339	4443.486
-81.3	0.283	0.758	2.745	7.960	25.365	68.516	159.937	306.572	428.360	4443.486
-81.1	0.244	0.863	2.681	7.922	25.267	68.404	159.321	302.094	429.457	4443.486
-80.9	0.259	0.804	2.587	7.941	25.504	69.006	159.126	300.825	430.689	4443.486
-80.7	0.248	0.833	2.697	7.939	25.276	68.917	159.168	302.811	431.091	4443.486
-80.5	0.303	0.781	2.677	7.870	25.266	68.462	159.442	306.830	427.865	4443.486
-80.3	0.253	0.827	2.589	7.856	25.359	68.809	159.273	301.973	427.820	4443.486
-80.1	0.257	0.747	2.666	7.832	25.354	68.384	158.824	304.319	427.962	4443.486
-79.9	0.264	0.854	2.599	7.974	25.377	69.159	156.790	303.501	428.215	4443.486
-79.7	0.235	0.758	2.555	7.902	25.629	68.654	159.146	304.697	427.257	4443.486
-79.5	0.267	0.815	2.579	7.937	25.404	68.956	159.051	306.189	429.337	4443.486
-79.3	0.217	0.791	2.713	7.852	25.427	68.907	160.453	303.453	425.940	4443.486
-79.1	0.269	0.792	2.636	7.670	25.309	68.821	157.978	303.269	428.887	4443.486
-78.9	0.257	0.843	2.675	7.891	25.237	68.894	157.351	306.767	429.397	4443.486
-78.7	0.245	0.817	2.644	7.915	25.490	68.993	158.056	302.452	431.019	4443.486
-78.5	0.257	0.768	2.679	7.995	25.365	68.826	160.772	304.653	427.439	4443.486
-78.3	0.250	0.761	2.701	7.893	25.562	68.402	160.042	305.768	430.265	4443.486
-78.1	0.257	0.834	2.722	7.810	25.511	68.507	158.502	302.794	429.404	4443.486
-77.9	0.259	0.761	2.693	7.878	25.336	68.517	159.314	303.130	429.610	4443.486
-77.7	0.258	0.844	2.676	7.838	25.415	68.643	161.427	302.082	428.050	4443.486
-77.5	0.236	0.791	2.712	7.858	25.266	68.476	159.492	302.113	427.192	4443.486
-77.3	0.266	0.776	2.688	7.778	25.535	69.011	160.243	301.291	429.158	4443.486
-77.1	0.277	0.836	2.630	8.016	25.368	69.143	157.975	301.760	428.404	4443.486
-76.9	0.246	0.795	2.743	7.927	25.299	68.585	159.844	301.718	429.203	4443.486
-76.7	0.270	0.765	2.662	7.847	25.384	68.885	158.672	299.010	428.170	4443.486
-76.5	0.246	0.746	2.702	7.821	25.315	68.651	158.675	304.844	427.034	4443.486
-76.3	0.259	0.725	2.655	7.874	25.672	68.528	158.817	302.427	430.280	4443.486
-76.1	0.232	0.801	2.674	7.798	25.360	68.536	159.395	299.910	430.366	4443.486
-75.9	0.228	0.769	2.567	7.785	25.208	68.229	159.273	303.253	428.334	4443.486
-75.7	0.295	0.761	2.614	7.796	25.405	68.392	159.001	305.970	425.769	4443.486
-75.5	0.251	0.770	2.663	7.896	25.381	68.673	159.383	299.132	429.767	4443.486
-75.3	0.232	0.734	2.594	7.730	25.242	68.325	157.878	301.464	427.275	4443.486
-75.1	0.236	0.849	2.616	7.870	25.166	68.377	158.443	304.804	427.002	4443.486
-74.9	0.252	0.788	2.667	7.844	25.404	68.818	158.441	297.360	427.001	4443.486

Midpoint	Ratio: 1860	657	189	62	19	6.3	2.1	0.65	0.13	Volume
-74.7	0.274	0.785	2.663	7.916	25.195	68.823	159.430	304.049	428.604	4443.486
-74.5	0.241	0.798	2.567	7.904	25.170	68.537	160.062	301.078	428.297	4443.486
-74.3	0.268	0.780	2.725	7.817	25.577	68.368	157.760	304.278	429.689	4443.486
-74.1	0.281	0.758	2.674	7.870	25.381	68.948	158.067	301.996	429.181	4443.486
-73.9	0.242	0.748	2.652	7.914	25.237	68.655	158.610	303.890	428.333	4443.486
-73.7	0.256	0.713	2.671	7.902	25.185	68.786	159.288	300.953	427.946	4443.486
-73.5	0.258	0.785	2.587	7.937	25.409	68.344	159.262	304.262	429.384	4443.486
-73.3	0.233	0.787	2.619	7.943	25.197	68.615	158.428	303.395	427.691	4443.486
-73.1	0.250	0.732	2.634	8.007	25.300	68.592	158.669	303.728	426.682	4443.486
-72.9	0.265	0.770	2.735	7.944	25.448	68.320	160.613	301.533	426.474	4443.486
-72.7	0.267	0.793	2.714	7.938	25.346	69.126	158.691	299.731	427.922	4443.486
-72.5	0.257	0.758	2.681	7.841	25.504	68.897	159.726	303.372	427.625	4443.486
-72.3	0.248	0.796	2.592	7.711	25.408	68.775	158.620	304.761	430.214	4443.486
-72.1	0.260	0.802	2.665	7.825	25.210	68.656	159.611	306.912	427.564	4443.486
-71.9	0.264	0.715	2.729	7.764	25.429	68.640	158.476	300.011	429.287	4443.486
-71.7	0.248	0.766	2.593	7.933	25.344	68.737	158.852	303.156	426.802	4443.486
-71.5	0.251	0.770	2.647	7.899	25.282	68.380	159.960	298.084	430.348	4443.486
-71.3	0.241	0.830	2.621	7.844	25.530	68.935	159.142	302.032	429.936	4443.486
-71.1	0.249	0.768	2.678	7.814	25.299	68.217	158.671	301.243	426.984	4443.486
-70.9	0.242	0.748	2.562	7.835	25.152	68.359	159.618	301.467	428.003	4443.486
-70.7	0.269	0.770	2.635	7.860	25.216	68.992	157.980	302.209	428.444	4443.486
-70.5	0.251	0.767	2.707	7.935	25.506	68.488	157.903	301.844	430.174	4443.486
-70.3	0.248	0.745	2.636	7.683	25.302	68.316	158.743	301.720	429.678	4443.486
-70.1	0.244	0.751	2.628	7.966	25.204	68.694	158.591	304.598	426.145	4443.486
-69.9	0.270	0.797	2.648	7.852	25.434	68.943	159.351	301.662	428.747	4443.486
-69.7	0.274	0.691	2.631	7.845	25.200	68.403	159.281	302.831	427.424	4443.486
-69.5	0.271	0.764	2.590	7.786	25.333	68.971	157.759	300.805	428.611	4443.486
-69.3	0.245	0.735	2.637	7.957	25.358	68.832	158.549	305.024	427.578	4443.486
-69.1	0.251	0.749	2.662	7.822	25.252	68.298	156.939	307.399	428.482	4443.486
-68.9	0.241	0.780	2.738	8.004	25.218	68.748	158.975	301.641	425.280	4443.486
-68.7	0.245	0.791	2.642	7.906	25.612	67.986	158.668	303.175	428.551	4443.486
-68.5	0.238	0.801	2.692	7.803	25.363	68.541	160.025	303.856	426.370	4443.486
-68.3	0.244	0.850	2.562	7.789	25.337	68.708	157.968	302.859	431.236	4443.486
-68.1	0.269	0.729	2.695	7.911	25.348	68.607	159.634	304.453	429.235	4443.486
-67.9	0.270	0.762	2.619	7.882	25.157	68.423	158.585	302.486	429.097	4443.486
-67.7	0.253	0.758	2.639	7.753	25.469	68.158	158.744	306.569	424.684	4443.486
-67.5	0.260	0.767	2.656	7.882	25.456	68.456	158.717	301.207	427.225	4443.486
-67.3	0.228	0.755	2.625	7.682	25.294	68.501	160.140	301.153	429.017	4443.486
-67.1	0.297	0.767	2.617	7.966	25.348	68.546	160.161	301.252	429.412	4443.486
-66.9	0.268	0.730	2.687	7.841	25.207	68.543	158.386	299.995	428.508	4443.486
-66.7	0.269	0.808	2.569	7.830	25.298	68.337	158.208	304.511	428.633	4443.486
-66.5	0.270	0.833	2.645	7.780	25.653	67.970	158.233	304.339	427.080	4443.486
-66.3	0.253	0.732	2.772	7.765	25.366	68.665	157.878	299.764	428.763	4443.486
-66.1	0.219	0.805	2.625	7.769	25.330	68.602	156.847	303.071	428.360	4443.486

Midpoint	Ratio: 1860	657	189	62	19	6.3	2.1	0.65	0.13	Volume
-65.9	0.256	0.800	2.637	7.806	25.508	68.154	157.604	304.465	428.010	4443.486
-65.7	0.282	0.805	2.561	7.943	25.235	68.352	159.647	301.070	428.524	4443.486
-65.5	0.262	0.791	2.664	7.839	25.525	68.648	159.915	302.882	429.576	4443.486
-65.3	0.255	0.763	2.629	7.811	25.285	68.956	159.058	300.693	427.771	4443.486
-65.1	0.260	0.703	2.620	7.831	25.110	68.663	158.988	298.753	428.900	4443.486
-64.9	0.211	0.780	2.702	7.903	25.256	68.394	159.144	302.200	428.110	4443.486
-64.7	0.257	0.759	2.635	7.821	25.262	68.527	157.437	300.175	428.344	4443.486
-64.5	0.251	0.748	2.548	7.885	25.405	68.177	159.231	304.097	427.752	4443.486
-64.3	0.265	0.793	2.670	7.879	25.221	69.222	160.357	300.399	429.197	4443.486
-64.1	0.230	0.819	2.675	7.743	25.405	68.338	159.924	303.115	430.312	4443.486
-63.9	0.255	0.760	2.631	7.991	25.146	68.881	158.380	303.055	427.975	4443.486
-63.7	0.239	0.762	2.685	7.792	25.225	68.613	158.392	306.000	426.270	4443.486
-63.5	0.279	0.820	2.663	7.747	25.395	68.363	157.922	302.121	429.338	4443.486
-63.3	0.252	0.803	2.672	7.847	25.349	68.535	159.386	302.410	427.329	4443.486
-63.1	0.231	0.792	2.731	7.998	25.400	68.194	159.967	306.870	432.351	4443.486
-62.9	0.262	0.867	2.587	7.883	25.476	69.068	158.199	302.963	427.094	4443.486
-62.7	0.245	0.864	2.692	7.813	25.226	68.330	157.060	303.654	426.871	4443.486
-62.5	0.239	0.757	2.737	7.864	25.447	68.491	159.072	303.338	427.656	4443.486
-62.3	0.256	0.793	2.672	7.824	25.395	68.535	159.750	303.385	425.610	4443.486
-62.1	0.299	0.788	2.656	8.002	25.419	68.396	158.559	299.726	431.134	4443.486
-61.9	0.247	0.720	2.703	7.871	25.489	68.985	157.541	303.671	427.743	4443.486
-61.7	0.237	0.819	2.585	7.783	25.196	68.347	159.087	302.312	430.685	4443.486
-61.5	0.282	0.800	2.648	7.912	25.214	68.320	157.752	301.477	427.287	4443.486
-61.3	0.258	0.757	2.692	7.819	25.340	68.753	158.057	303.765	429.839	4443.486
-61.1	0.265	0.739	2.672	7.888	25.367	68.214	160.348	299.590	430.778	4443.486
-60.9	0.270	0.779	2.659	7.859	25.424	68.542	159.228	297.908	427.661	4443.486
-60.7	0.237	0.749	2.649	7.758	25.152	68.578	158.701	302.285	428.827	4443.486
-60.5	0.236	0.810	2.570	7.768	25.243	68.719	159.241	304.620	427.016	4443.486
-60.3	0.258	0.840	2.711	7.799	25.259	68.548	158.021	303.426	424.696	4443.486
-60.1	0.223	0.768	2.592	7.799	25.438	69.400	160.438	301.207	429.138	4443.486
-59.9	0.274	0.729	2.579	7.835	25.581	68.544	157.911	301.033	427.328	4443.486
-59.7	0.228	0.755	2.648	7.920	25.084	68.655	157.309	303.498	428.688	4443.486
-59.5	0.254	0.714	2.676	7.834	25.364	68.821	159.407	303.092	428.469	4443.486
-59.3	0.286	0.775	2.572	7.746	25.162	68.424	157.213	300.126	428.114	4443.486
-59.1	0.237	0.756	2.723	7.794	25.152	69.031	158.138	302.069	426.147	4443.486
-58.9	0.251	0.781	2.728	7.807	25.150	68.744	160.523	302.470	431.454	4443.486
-58.7	0.219	0.738	2.794	7.783	25.416	68.871	158.671	302.259	429.386	4443.486
-58.5	0.249	0.789	2.599	7.857	25.504	68.481	158.314	307.416	425.870	4443.486
-58.3	0.255	0.843	2.637	7.882	25.287	69.032	159.503	301.961	427.128	4443.486
-58.1	0.256	0.780	2.598	7.888	25.077	68.815	158.784	302.295	428.873	4443.486
-57.9	0.242	0.798	2.689	7.814	25.304	68.683	157.414	301.867	427.788	4443.486
-57.7	0.256	0.789	2.745	7.870	25.292	68.591	159.661	302.670	429.091	4443.486
-57.5	0.226	0.783	2.664	7.833	25.129	68.886	159.702	304.488	429.561	4443.486
-57.3	0.288	0.784	2.621	7.885	25.046	69.062	157.954	304.439	431.092	4443.486

Midpoint	Ratio: 1860	657	189	62	19	6.3	2.1	0.65	0.13	Volume
-57.1	0.246	0.745	2.631	7.772	25.233	68.594	159.732	299.510	429.182	4443.486
-56.9	0.277	0.770	2.649	7.757	25.247	68.536	158.587	302.198	428.225	4443.486
-56.7	0.268	0.746	2.562	7.832	25.261	69.023	158.510	301.512	427.334	4443.486
-56.5	0.276	0.805	2.674	7.868	25.244	68.659	158.907	304.626	430.567	4443.486
-56.3	0.261	0.850	2.658	7.770	25.194	68.556	158.230	304.986	427.502	4443.486
-56.1	0.261	0.736	2.708	8.031	25.276	68.347	158.253	300.983	426.726	4443.486
-55.9	0.255	0.778	2.574	7.711	25.472	68.541	159.340	303.060	429.889	4443.486
-55.7	0.243	0.770	2.653	7.917	25.134	68.708	157.990	304.941	429.039	4443.486
-55.5	0.308	0.795	2.621	7.811	25.298	69.281	158.817	303.976	431.744	4443.486
-55.3	0.256	0.768	2.735	7.888	25.441	68.798	158.654	302.114	427.196	4443.486
-55.1	0.265	0.810	2.649	7.811	25.240	68.284	161.182	305.666	430.119	4443.486
-54.9	0.211	0.855	2.625	7.836	25.341	68.274	157.596	299.947	430.698	4443.486
-54.7	0.246	0.777	2.712	7.810	25.414	68.782	158.049	303.706	427.273	4443.486
-54.5	0.270	0.743	2.664	7.879	25.438	68.680	159.423	304.297	427.681	4443.486
-54.3	0.278	0.761	2.763	7.758	25.317	69.219	159.627	305.218	427.293	4443.486
-54.1	0.245	0.757	2.731	7.851	25.302	68.350	158.601	302.776	426.928	4443.486
-53.9	0.232	0.894	2.560	7.758	25.138	68.768	159.291	301.890	431.575	4443.486
-53.7	0.253	0.864	2.655	7.735	25.428	68.661	159.851	301.320	429.611	4443.486
-53.5	0.302	0.733	2.720	7.820	25.271	68.691	158.600	303.978	428.698	4443.486
-53.3	0.279	0.791	2.567	7.852	25.538	68.669	157.900	305.164	429.073	4443.486
-53.1	0.263	0.796	2.654	7.871	25.298	68.482	159.203	301.907	425.954	4443.486
-52.9	0.253	0.739	2.699	7.822	25.489	68.220	159.251	305.038	430.288	4443.486
-52.7	0.250	0.796	2.676	7.684	25.366	69.694	157.188	301.308	429.099	4443.486
-52.5	0.253	0.742	2.555	7.806	25.508	68.567	161.295	301.950	431.857	4443.486
-52.3	0.234	0.827	2.645	7.763	25.352	69.012	158.604	304.166	430.353	4443.486
-52.1	0.244	0.790	2.643	7.948	25.226	68.412	156.902	301.828	432.023	4443.486
-51.9	0.239	0.805	2.694	7.898	25.338	68.932	158.260	303.641	427.479	4443.486
-51.7	0.262	0.808	2.642	7.842	25.384	68.855	159.401	300.837	428.259	4443.486
-51.5	0.246	0.725	2.639	7.729	25.437	69.562	158.229	303.939	429.828	4443.486
-51.3	0.255	0.737	2.604	7.830	25.216	69.053	159.964	302.180	427.786	4443.486
-51.1	0.246	0.883	2.531	7.973	25.366	69.210	159.373	302.006	426.716	4443.486
-50.9	0.221	0.825	2.606	7.817	25.241	68.791	159.339	305.253	429.933	4443.486
-50.7	0.265	0.720	2.564	7.856	25.535	68.405	159.934	305.277	430.324	4443.486
-50.5	0.284	0.783	2.727	7.946	25.259	68.878	157.758	303.256	431.370	4443.486
-50.3	0.232	0.762	2.734	7.929	25.444	69.441	159.700	304.202	429.699	4443.486
-50.1	0.270	0.719	2.604	7.782	25.488	68.841	158.187	305.886	430.506	4443.486
-49.9	0.276	0.755	2.593	7.881	25.478	68.442	158.747	300.778	428.628	4443.486
-49.7	0.232	0.774	2.615	7.825	25.412	68.947	160.010	299.277	428.992	4443.486
-49.5	0.272	0.770	2.657	7.962	25.608	68.680	160.344	302.295	428.804	4443.486
-49.3	0.257	0.814	2.721	7.873	25.449	68.689	159.280	300.804	427.672	4443.486
-49.1	0.256	0.729	2.644	7.744	25.375	68.688	158.713	306.914	428.804	4443.486
-48.9	0.248	0.806	2.666	7.771	25.014	69.064	159.577	304.407	429.449	4443.486
-48.7	0.288	0.768	2.707	7.839	25.339	68.995	159.548	303.279	426.664	4443.486
-48.5	0.288	0.775	2.616	7.758	25.696	68.674	159.841	304.444	429.464	4443.486

Midpoint	Ratio: 1860	657	189	62	19	6.3	2.1	0.65	0.13	Volume
-48.3	0.258	0.833	2.526	7.862	25.634	68.934	159.707	307.959	430.683	4443.486
-48.1	0.254	0.746	2.707	7.871	25.477	68.848	159.760	302.826	427.945	4443.486
-47.9	0.249	0.789	2.691	7.855	25.636	68.755	160.981	303.336	430.359	4443.486
-47.7	0.265	0.760	2.685	7.841	25.316	68.892	158.917	302.884	430.514	4443.486
-47.5	0.266	0.795	2.653	7.982	25.507	68.970	158.685	308.057	429.145	4443.486
-47.3	0.271	0.780	2.629	7.886	25.541	68.782	158.608	302.656	429.224	4443.486
-47.1	0.287	0.817	2.619	7.951	25.486	68.837	159.810	303.101	429.250	4443.486
-46.9	0.232	0.763	2.629	8.008	25.668	68.605	159.362	304.476	430.933	4443.486
-46.7	0.254	0.747	2.656	7.723	25.628	68.782	157.969	302.234	429.485	4443.486
-46.5	0.235	0.777	2.666	7.955	25.508	68.685	159.425	304.257	431.079	4443.486
-46.3	0.258	0.776	2.546	7.889	25.409	69.651	159.122	305.401	431.347	4443.486
-46.1	0.223	0.819	2.607	8.107	25.406	68.803	160.467	306.422	431.326	4443.486
-45.9	0.259	0.875	2.701	7.778	25.485	69.165	160.076	300.139	431.917	4443.486
-45.7	0.239	0.732	2.724	7.859	25.273	69.320	160.783	302.716	430.740	4443.486
-45.5	0.265	0.770	2.664	7.836	25.392	68.804	159.169	304.248	430.898	4443.486
-45.3	0.263	0.787	2.651	7.946	25.436	68.915	161.014	305.552	428.682	4443.486
-45.1	0.233	0.825	2.601	7.828	25.445	68.725	158.288	306.057	424.846	4443.486
-44.9	0.253	0.709	2.612	7.982	25.421	69.259	158.970	303.543	433.030	4443.486
-44.7	0.275	0.876	2.626	7.894	25.397	69.223	159.053	305.479	431.575	4443.486
-44.5	0.254	0.775	2.630	7.899	25.576	68.334	159.823	303.716	431.979	4443.486
-44.3	0.245	0.822	2.609	8.014	25.782	68.616	159.967	304.593	428.599	4443.486
-44.1	0.266	0.817	2.702	7.769	25.609	68.791	160.751	305.191	432.496	4443.486
-43.9	0.216	0.762	2.680	7.921	25.315	69.061	158.567	301.673	432.624	4443.486
-43.7	0.256	0.771	2.595	7.801	25.342	69.114	162.532	305.290	429.610	4443.486
-43.5	0.262	0.797	2.683	7.873	25.505	68.961	160.627	305.076	430.981	4443.486
-43.3	0.258	0.721	2.583	7.889	25.436	68.383	160.811	305.417	428.947	4443.486
-43.1	0.260	0.863	2.689	7.885	25.502	69.435	159.009	303.314	434.207	4443.486
-42.9	0.264	0.793	2.606	7.945	25.783	69.419	158.992	307.248	431.628	4443.486
-42.7	0.233	0.767	2.673	7.818	25.139	68.916	160.450	304.534	429.808	4443.486
-42.5	0.269	0.791	2.727	7.972	25.431	68.842	159.463	303.024	429.963	4443.486
-42.3	0.227	0.772	2.662	7.853	25.627	68.618	161.491	302.692	429.156	4443.486
-42.1	0.265	0.827	2.664	7.886	25.576	69.413	162.104	304.376	435.238	4443.486
-41.9	0.259	0.755	2.616	7.864	25.484	69.122	160.682	303.287	428.388	4443.486
-41.7	0.250	0.830	2.720	7.963	25.550	69.494	159.741	305.176	434.240	4443.486
-41.5	0.248	0.758	2.723	7.823	25.456	69.305	161.288	303.183	429.736	4443.486
-41.3	0.277	0.804	2.725	7.975	25.441	69.273	159.074	304.121	428.201	4443.486
-41.1	0.278	0.760	2.673	7.874	25.595	69.339	160.898	303.785	431.630	4443.486
-40.9	0.254	0.725	2.722	7.942	25.492	68.753	161.732	305.436	433.353	4443.486
-40.7	0.235	0.852	2.558	8.008	25.481	69.060	158.678	306.052	429.816	4443.486
-40.5	0.208	0.805	2.605	7.907	25.515	68.537	160.569	304.045	432.034	4443.486
-40.3	0.265	0.822	2.736	7.864	25.445	69.109	160.308	305.535	430.635	4443.486
-40.1	0.214	0.794	2.705	7.779	25.692	69.130	161.364	305.087	429.764	4443.486
-39.9	0.280	0.827	2.628	7.929	25.460	68.914	159.799	307.863	433.819	4443.486
-39.7	0.278	0.813	2.700	7.893	25.813	69.258	160.279	305.968	430.994	4443.486

Midpoint	Ratio: 1860	657	189	62	19	6.3	2.1	0.65	0.13	Volume
-39.5	0.266	0.846	2.635	7.695	25.483	69.688	161.666	306.452	433.698	4443.486
-39.3	0.265	0.834	2.628	8.007	25.620	69.238	160.867	310.970	431.558	4443.486
-39.1	0.234	0.824	2.727	7.865	25.676	69.078	160.531	303.170	432.511	4443.486
-38.9	0.265	0.829	2.642	7.887	25.589	69.609	160.945	304.192	431.582	4443.486
-38.7	0.256	0.785	2.638	7.939	25.492	69.463	160.636	305.725	431.456	4443.486
-38.5	0.244	0.735	2.707	7.872	25.631	69.600	160.355	306.712	431.552	4443.486
-38.3	0.240	0.820	2.644	7.994	25.504	69.451	159.569	305.785	434.088	4443.486
-38.1	0.246	0.690	2.669	7.958	25.747	69.096	160.940	304.491	433.687	4443.486
-37.9	0.248	0.779	2.701	7.920	25.672	69.350	160.185	305.807	430.029	4443.486
-37.7	0.267	0.807	2.671	7.986	25.731	69.451	159.775	303.414	430.596	4443.486
-37.5	0.255	0.780	2.679	7.978	25.843	69.419	162.001	306.228	432.169	4443.486
-37.3	0.248	0.755	2.692	7.909	25.927	69.626	160.849	307.679	432.825	4443.486
-37.1	0.221	0.806	2.629	8.014	25.577	70.303	163.969	303.151	436.192	4443.486
-36.9	0.265	0.831	2.630	8.111	25.612	69.636	159.842	309.856	431.245	4443.486
-36.7	0.277	0.818	2.608	7.879	25.601	69.454	161.049	304.816	434.154	4443.486
-36.5	0.239	0.779	2.722	8.022	25.998	69.973	161.724	308.076	433.516	4443.486
-36.3	0.251	0.800	2.734	7.923	25.832	70.115	160.187	310.438	433.928	4443.486
-36.1	0.241	0.800	2.662	7.842	25.746	69.861	160.501	304.750	434.639	4443.486
-35.9	0.247	0.894	2.659	7.865	25.602	70.003	161.661	309.221	436.750	4443.486
-35.7	0.254	0.836	2.626	8.003	25.490	69.450	161.068	308.806	435.720	4443.486
-35.5	0.277	0.801	2.656	8.007	25.586	69.648	159.011	308.978	438.024	4443.486
-35.3	0.266	0.762	2.518	7.853	25.799	69.880	162.062	306.811	435.369	4443.486
-35.1	0.249	0.832	2.734	8.052	25.647	69.866	161.532	309.721	433.959	4443.486
-34.9	0.262	0.797	2.747	8.023	25.775	70.021	160.791	311.921	433.918	4443.486
-34.7	0.271	0.866	2.816	7.943	25.634	70.032	161.833	306.379	437.256	4443.486
-34.5	0.266	0.781	2.646	8.114	25.833	69.978	161.335	307.708	438.953	4443.486
-34.3	0.257	0.797	2.755	8.043	25.909	70.650	161.081	310.076	437.283	4443.486
-34.1	0.264	0.757	2.704	8.122	25.916	70.211	164.252	309.696	438.413	4443.486
-33.9	0.249	0.854	2.778	7.988	26.157	69.498	161.930	308.828	437.868	4443.486
-33.7	0.255	0.758	2.765	8.122	25.733	70.170	161.724	309.740	436.776	4443.486
-33.5	0.280	0.794	2.696	8.072	26.041	70.111	162.427	309.736	434.583	4443.486
-33.3	0.251	0.733	2.665	7.918	25.740	70.185	163.584	308.414	436.182	4443.486
-33.1	0.247	0.784	2.722	8.086	25.904	70.794	161.946	308.636	436.390	4443.486
-32.9	0.264	0.786	2.678	8.015	26.062	69.690	163.013	308.610	437.779	4443.486
-32.7	0.250	0.834	2.776	7.801	26.016	70.532	162.558	309.266	438.455	4443.486
-32.5	0.256	0.775	2.696	7.924	26.004	69.826	163.774	307.779	436.478	4443.486
-32.3	0.270	0.759	2.699	7.968	25.884	69.962	162.759	307.995	434.648	4443.486
-32.1	0.273	0.747	2.699	7.960	25.838	70.031	162.147	310.613	443.356	4443.486
-31.9	0.217	0.775	2.640	8.141	25.974	69.758	164.959	311.144	436.993	4443.486
-31.7	0.242	0.787	2.743	8.023	26.027	70.586	164.713	314.110	439.221	4443.486
-31.5	0.249	0.785	2.609	8.143	26.015	70.555	164.085	310.644	436.619	4443.486
-31.3	0.252	0.811	2.750	8.097	25.985	70.278	165.422	308.473	437.328	4443.486
-31.1	0.238	0.755	2.693	8.061	25.908	70.602	162.436	312.166	439.370	4443.486
-30.9	0.263	0.767	2.831	7.999	25.956	70.674	164.510	313.475	437.956	4443.486

Midpoint	Ratio: 1860	657	189	62	19	6.3	2.1	0.65	0.13	Volume
-30.7	0.261	0.845	2.679	8.099	26.130	70.900	163.158	312.722	436.480	4443.486
-30.5	0.243	0.828	2.637	8.180	26.337	70.631	164.451	311.393	439.002	4443.486
-30.3	0.241	0.779	2.645	8.082	26.030	71.425	162.348	313.662	437.379	4443.486
-30.1	0.280	0.793	2.736	8.073	26.227	70.773	164.036	309.174	440.066	4443.486
-29.9	0.276	0.821	2.699	8.022	26.210	70.289	163.355	313.306	441.443	4443.486
-29.7	0.295	0.836	2.746	8.178	26.048	70.818	163.135	308.724	442.107	4443.486
-29.5	0.243	0.794	2.746	8.208	26.011	70.866	164.315	308.936	441.353	4443.486
-29.3	0.279	0.743	2.661	8.119	26.293	70.997	166.198	315.579	443.716	4443.486
-29.1	0.269	0.825	2.787	8.126	26.290	71.096	163.657	312.205	443.022	4443.486
-28.9	0.242	0.781	2.778	8.197	26.238	70.256	164.501	314.086	441.933	4443.486
-28.7	0.258	0.861	2.689	8.125	26.159	71.738	164.671	311.941	444.069	4443.486
-28.5	0.302	0.809	2.830	8.336	26.360	70.945	164.957	315.103	442.825	4443.486
-28.3	0.262	0.839	2.769	8.132	26.445	71.742	164.578	314.771	442.564	4443.486
-28.1	0.263	0.814	2.690	8.104	26.280	70.981	165.396	316.203	442.979	4443.486
-27.9	0.277	0.799	2.706	8.149	26.467	71.961	165.018	307.759	445.171	4443.486
-27.7	0.243	0.793	2.726	8.202	26.138	71.509	164.107	313.633	444.304	4443.486
-27.5	0.229	0.819	2.768	8.074	26.314	71.704	166.253	312.365	445.226	4443.486
-27.3	0.279	0.833	2.808	8.042	26.583	70.983	166.662	315.040	447.076	4443.486
-27.1	0.249	0.802	2.742	8.157	26.180	72.078	165.750	318.613	445.623	4443.486
-26.9	0.284	0.826	2.770	8.148	26.586	71.538	167.580	313.147	447.974	4443.486
-26.7	0.302	0.843	2.694	8.108	26.412	72.498	166.543	313.406	443.845	4443.486
-26.5	0.260	0.874	2.726	8.141	26.489	71.978	165.137	314.856	447.417	4443.486
-26.3	0.271	0.777	2.845	8.132	26.471	71.496	166.906	315.443	444.830	4443.486
-26.1	0.256	0.866	2.780	8.187	26.562	71.548	165.971	315.428	445.943	4443.486
-25.9	0.276	0.838	2.713	8.324	26.488	71.938	165.999	318.636	446.372	4443.486
-25.7	0.271	0.805	2.808	8.259	26.363	72.086	166.901	316.478	450.726	4443.486
-25.5	0.251	0.766	2.772	8.198	26.662	71.910	166.403	316.719	445.979	4443.486
-25.3	0.269	0.851	2.807	8.383	26.454	72.278	166.322	317.196	447.750	4443.486
-25.1	0.246	0.768	2.778	8.261	26.717	71.928	168.790	318.135	446.922	4443.486
-24.9	0.280	0.818	2.803	8.267	26.443	72.400	165.967	320.003	449.383	4443.486
-24.7	0.283	0.821	2.807	8.441	26.726	72.606	167.975	317.052	450.858	4443.486
-24.5	0.276	0.803	2.766	8.216	26.653	72.619	168.316	320.068	448.836	4443.486
-24.3	0.272	0.850	2.818	8.296	26.835	72.285	167.671	320.219	449.122	4443.486
-24.1	0.244	0.842	2.745	8.272	26.902	72.584	168.559	319.680	451.320	4443.486
-23.9	0.276	0.832	2.786	8.227	26.922	72.797	168.470	317.895	451.195	4443.486
-23.7	0.258	0.836	2.748	8.253	26.671	72.681	169.212	317.492	453.723	4443.486
-23.5	0.284	0.837	2.782	8.447	26.812	73.088	167.174	318.798	453.784	4443.486
-23.3	0.292	0.819	2.815	8.335	26.878	72.361	168.106	317.321	452.597	4443.486
-23.1	0.239	0.862	2.729	8.421	27.232	72.991	168.443	324.026	452.367	4443.486
-22.9	0.255	0.784	2.747	8.378	27.215	73.304	168.508	322.899	452.436	4443.486
-22.7	0.240	0.834	2.731	8.357	26.865	73.526	168.546	323.011	455.848	4443.486
-22.5	0.286	0.877	2.779	8.408	26.786	73.702	169.154	319.042	453.106	4443.486
-22.3	0.295	0.777	2.839	8.466	26.960	73.680	170.702	320.490	452.031	4443.486
-22.1	0.280	0.826	2.783	8.276	27.176	73.180	168.695	321.337	457.256	4443.486

Midpoint	Ratio: 1860	657	189	62	19	6.3	2.1	0.65	0.13	Volume
-21.9	0.272	0.875	2.844	8.541	27.004	73.243	169.194	322.926	454.785	4443.486
-21.7	0.274	0.871	2.885	8.447	27.055	73.451	170.901	323.720	453.533	4443.486
-21.5	0.275	0.801	2.910	8.437	27.199	74.017	169.065	323.066	455.295	4443.486
-21.3	0.258	0.794	2.816	8.472	27.190	74.585	170.957	321.512	455.041	4443.486
-21.1	0.275	0.817	2.788	8.308	26.993	74.040	171.081	322.183	456.007	4443.486
-20.9	0.282	0.808	2.828	8.498	27.154	74.235	170.478	326.389	455.056	4443.486
-20.7	0.269	0.727	2.822	8.402	27.095	74.420	171.300	325.411	460.178	4443.486
-20.5	0.286	0.848	2.790	8.494	27.393	74.353	171.685	328.504	457.688	4443.486
-20.3	0.291	0.875	2.837	8.608	27.728	74.301	172.506	328.040	460.250	4443.486
-20.1	0.286	0.837	2.833	8.576	27.579	75.131	172.282	328.113	456.161	4443.486
-19.9	0.281	0.805	2.788	8.663	27.800	75.046	171.922	331.594	462.936	4443.486
-19.7	0.254	0.884	2.852	8.597	27.919	75.080	172.748	327.445	459.860	4443.486
-19.5	0.239	0.844	2.888	8.647	27.837	74.977	172.510	330.907	467.250	4443.486
-19.3	0.255	0.831	2.871	8.733	27.981	75.550	174.292	327.769	464.520	4443.486
-19.1	0.252	0.814	2.884	8.586	27.436	75.991	174.635	332.138	468.788	4443.486
-18.9	0.249	0.813	2.804	8.753	28.226	75.430	175.623	334.836	465.617	4443.486
-18.7	0.257	0.836	2.926	8.666	28.296	76.524	178.018	334.655	467.837	4443.486
-18.5	0.267	0.824	2.957	8.646	28.249	76.177	176.857	332.923	468.459	4443.486
-18.3	0.259	0.751	2.611	7.856	25.651	69.779	158.944	302.989	428.877	4443.486
-18.1	0.015	0.050	0.159	0.520	1.719	4.334	10.525	19.503	26.846	11525.603
-17.9	0.015	0.061	0.173	0.429	1.434	3.942	9.559	17.466	24.135	184.223
-17.7	0.010	0.028	0.136	0.429	1.301	3.649	8.163	15.998	22.384	160.425
-17.5	0.011	0.038	0.145	0.361	1.227	3.463	7.741	14.804	21.518	145.306
-17.3	0.010	0.038	0.111	0.366	1.196	3.178	7.256	14.011	19.562	133.670
-17.1	0.011	0.025	0.115	0.335	1.115	2.975	7.062	13.885	19.293	124.097
-16.9	0.003	0.030	0.084	0.292	1.033	2.849	6.578	12.376	17.237	115.931
-16.7	0.007	0.033	0.092	0.310	1.012	2.789	6.312	11.971	16.965	108.807
-16.5	0.006	0.011	0.114	0.307	0.983	2.706	5.930	11.162	16.215	102.492
-16.3	0.008	0.022	0.100	0.282	0.935	2.451	6.129	10.278	15.876	96.830
-16.1	0.002	0.020	0.088	0.255	0.943	2.338	5.815	10.268	14.781	91.707
-15.9	0.006	0.028	0.087	0.277	0.908	2.403	5.520	10.227	14.364	87.041
-15.7	0.008	0.025	0.064	0.261	0.893	2.427	5.456	10.848	14.325	82.765
-15.5	0.008	0.020	0.079	0.201	0.713	2.004	5.518	10.864	15.337	78.830
-15.3	0.005	0.011	0.055	0.183	0.567	1.670	4.644	9.826	14.216	75.194
-15.1	0.006	0.013	0.063	0.180	0.565	1.645	4.689	9.669	13.907	71.823
-14.9	0.007	0.017	0.060	0.174	0.505	1.640	4.505	9.405	13.545	68.689
-14.7	0.006	0.009	0.047	0.156	0.542	1.601	4.501	9.015	13.517	65.768
-14.5	0.003	0.007	0.050	0.143	0.558	1.565	4.269	9.083	13.008	63.041
-14.3	0.004	0.015	0.046	0.154	0.522	1.509	4.192	8.764	12.864	60.488
-14.1	0.008	0.009	0.055	0.147	0.465	1.398	4.036	8.762	12.452	58.097
-13.9	0.007	0.024	0.054	0.143	0.506	1.431	4.120	8.325	12.145	55.853
-13.7	0.003	0.013	0.043	0.136	0.479	1.431	4.038	8.289	12.041	53.744
-13.5	0.006	0.011	0.051	0.156	0.475	1.426	4.021	8.327	12.052	51.761
-13.3	0.011	0.011	0.040	0.140	0.431	1.434	3.934	8.065	11.646	49.893

Midpoint	Ratio: 1860	657	189	62	19	6.3	2.1	0.65	0.13	Volume
-13.1	0.007	0.007	0.040	0.129	0.441	1.333	3.949	8.051	11.750	48.134
-12.9	0.002	0.013	0.036	0.136	0.468	1.381	3.963	8.114	11.862	46.475
-12.7	0.008	0.005	0.054	0.131	0.401	1.378	3.926	7.954	11.716	44.910
-12.5	0.004	0.005	0.051	0.139	0.446	1.381	3.830	7.962	11.591	43.433
-12.3	0.005	0.007	0.033	0.123	0.454	1.439	4.043	8.149	11.614	42.039
-12.1	0.007	0.007	0.041	0.134	0.467	1.409	3.961	8.095	11.487	40.722
-11.9	0.003	0.014	0.041	0.119	0.447	1.406	3.874	8.110	11.555	39.477
-11.7	0.004	0.011	0.026	0.140	0.479	1.535	4.031	8.168	11.577	38.302
-11.5	0.007	0.002	0.036	0.124	0.484	1.446	3.928	7.901	11.576	37.191
-11.3	0.006	0.017	0.053	0.119	0.421	1.426	3.814	7.765	10.940	36.142
-11.1	0.006	0.015	0.039	0.123	0.397	1.378	3.490	7.236	10.624	35.150
-10.9	0.001	0.009	0.046	0.107	0.388	1.246	3.355	6.723	9.987	34.214
-10.7	0.003	0.018	0.039	0.122	0.402	1.196	3.165	6.278	9.076	33.331
-10.5	0.003	0.021	0.020	0.093	0.360	1.162	3.156	5.948	8.690	32.497
-10.3	0.004	0.013	0.036	0.081	0.324	1.082	2.827	5.756	7.948	31.710
-10.1	0.003	0.009	0.027	0.088	0.301	0.999	2.518	5.173	7.256	30.969
-9.9	0.005	0.009	0.014	0.074	0.284	0.958	2.221	4.734	6.633	30.272
-9.7	0.002	0.002	0.020	0.068	0.261	0.824	1.990	4.071	5.836	29.615
-9.5	0.002	0.011	0.023	0.059	0.233	0.719	1.911	3.665	5.236	28.999
-9.3	0.000	0.008	0.016	0.052	0.177	0.561	1.562	3.136	4.560	28.420
-9.1	0.000	0.004	0.019	0.045	0.181	0.544	1.422	2.729	3.806	27.878
-8.9	0.001	0.002	0.008	0.054	0.141	0.478	1.212	2.317	3.315	27.372
-8.7	0.002	0.002	0.015	0.029	0.125	0.390	0.984	2.044	2.683	26.899
-8.5	0.000	0.002	0.008	0.033	0.107	0.345	0.783	1.452	2.348	26.459
-8.3	0.001	0.002	0.012	0.020	0.100	0.283	0.702	1.351	1.975	26.050
-8.1	0.001	0.004	0.007	0.017	0.079	0.235	0.556	1.122	1.623	25.673
-7.9	0.000	0.000	0.000	0.016	0.055	0.189	0.475	0.935	1.369	25.325
-7.7	0.000	0.002	0.007	0.016	0.037	0.143	0.381	0.790	1.056	25.006
-7.5	0.000	0.000	0.002	0.009	0.044	0.128	0.318	0.652	0.890	24.716
-7.3	0.000	0.000	0.003	0.017	0.034	0.124	0.288	0.505	0.711	24.452
-7.1	0.000	0.002	0.003	0.009	0.025	0.097	0.284	0.474	0.665	24.216
-6.9	0.000	0.002	0.002	0.004	0.027	0.088	0.221	0.392	0.572	24.006
-6.7	0.000	0.000	0.002	0.007	0.027	0.081	0.172	0.401	0.502	23.822
-6.5	0.000	0.000	0.001	0.006	0.023	0.070	0.179	0.354	0.479	23.663
-6.3	0.000	0.000	0.002	0.006	0.029	0.075	0.138	0.283	0.397	23.529
-6.1	0.000	0.000	0.001	0.006	0.019	0.057	0.118	0.233	0.359	23.420
-5.9	0.001	0.002	0.000	0.005	0.014	0.061	0.158	0.241	0.372	23.336
-5.7	0.001	0.002	0.001	0.002	0.019	0.059	0.132	0.222	0.325	23.276
-5.5	0.000	0.000	0.003	0.001	0.013	0.030	0.100	0.234	0.272	23.240
-5.3	0.000	0.002	0.001	0.005	0.017	0.034	0.089	0.177	0.238	23.227
-5.1	0.000	0.000	0.001	0.004	0.012	0.030	0.081	0.187	0.237	23.227
-4.9	0.000	0.000	0.001	0.004	0.017	0.043	0.087	0.178	0.271	23.227
-4.7	0.000	0.000	0.000	0.005	0.013	0.029	0.104	0.196	0.274	23.227
-4.5	0.000	0.000	0.001	0.005	0.015	0.046	0.101	0.191	0.273	23.227

Midpoint	Ratio: 1860	657	189	62	19	6.3	2.1	0.65	0.13	Volume
-4.3	0.001	0.002	0.001	0.005	0.025	0.055	0.117	0.163	0.290	23.227
-4.1	0.000	0.000	0.005	0.005	0.017	0.058	0.126	0.213	0.352	23.227
-3.9	0.000	0.002	0.001	0.005	0.019	0.056	0.145	0.239	0.339	23.227
-3.7	0.000	0.000	0.003	0.003	0.022	0.065	0.138	0.264	0.390	23.227
-3.5	0.000	0.000	0.001	0.007	0.017	0.061	0.164	0.326	0.390	23.227
-3.3	0.000	0.004	0.000	0.006	0.027	0.069	0.169	0.348	0.408	23.227
-3.1	0.000	0.000	0.006	0.008	0.030	0.077	0.160	0.338	0.469	23.227
-2.9	0.000	0.000	0.003	0.006	0.023	0.074	0.166	0.391	0.506	23.227
-2.7	0.000	0.000	0.001	0.008	0.027	0.060	0.176	0.425	0.560	23.227
-2.5	0.001	0.002	0.003	0.009	0.029	0.073	0.199	0.437	0.566	23.227
-2.3	0.000	0.002	0.002	0.014	0.025	0.100	0.225	0.443	0.636	23.227
-2.1	0.000	0.002	0.005	0.010	0.039	0.096	0.259	0.474	0.628	23.227
-1.9	0.000	0.000	0.003	0.015	0.032	0.109	0.238	0.484	0.618	23.227
-1.7	0.000	0.004	0.007	0.013	0.035	0.113	0.256	0.559	0.672	23.227
-1.5	0.001	0.002	0.005	0.011	0.035	0.122	0.239	0.494	0.652	23.227
-1.3	0.000	0.000	0.006	0.010	0.042	0.098	0.257	0.482	0.691	23.227
-1.1	0.000	0.002	0.002	0.009	0.030	0.095	0.249	0.443	0.704	23.227
-0.9	0.000	0.000	0.005	0.010	0.035	0.105	0.241	0.404	0.682	23.227
-0.7	0.000	0.000	0.002	0.007	0.034	0.097	0.222	0.392	0.614	23.227
-0.5	0.000	0.002	0.007	0.011	0.033	0.061	0.217	0.357	0.476	23.227
-0.1	0.000	0.000	0.001	0.011	0.020	0.063	0.156	0.265	0.414	23.227
0.1	0.001	0.000	0.003	0.003	0.019	0.037	0.110	0.212	0.293	23.227
0.5	0.000	0.000	0.002	0.002	0.012	0.028	0.069	0.179	0.179	23.227
0.7	0.000	0.000	0.000	0.004	0.011	0.026	0.049	0.109	0.159	23.227
0.9	0.000	0.000	0.001	0.002	0.004	0.020	0.041	0.044	0.108	23.227
1.1	0.000	0.000	0.001	0.001	0.003	0.011	0.031	0.049	0.071	23.227
1.3	0.000	0.000	0.000	0.001	0.004	0.008	0.026	0.034	0.055	23.227
1.5	0.000	0.000	0.001	0.001	0.003	0.004	0.022	0.021	0.045	23.227
1.7	0.000	0.000	0.000	0.000	0.004	0.004	0.012	0.018	0.032	23.227
1.9	0.000	0.000	0.001	0.003	0.003	0.001	0.012	0.021	0.028	23.227
2.1	0.000	0.000	0.000	0.000	0.000	0.003	0.002	0.013	0.024	23.227
2.3	0.000	0.000	0.000	0.001	0.001	0.001	0.000	0.010	0.020	23.227
2.5	0.000	0.000	0.000	0.001	0.001	0.009	0.012	0.013	0.023	23.227
2.7	0.000	0.000	0.000	0.000	0.001	0.001	0.012	0.013	0.014	23.227
2.9	0.000	0.000	0.000	0.000	0.001	0.002	0.004	0.013	0.015	23.227
3.1	0.000	0.000	0.000	0.001	0.001	0.002	0.006	0.023	0.022	23.227
3.3	0.000	0.000	0.000	0.000	0.003	0.005	0.006	0.023	0.020	23.227
3.5	0.000	0.000	0.000	0.000	0.001	0.002	0.010	0.013	0.034	23.227
3.7	0.000	0.000	0.000	0.000	0.002	0.003	0.018	0.026	0.020	23.227
3.9	0.000	0.000	0.000	0.000	0.002	0.003	0.014	0.041	0.048	23.227
4.1	0.000	0.000	0.000	0.000	0.002	0.007	0.020	0.016	0.058	23.227
4.3	0.000	0.000	0.000	0.002	0.001	0.008	0.031	0.044	0.059	23.227
4.5	0.000	0.000	0.000	0.001	0.004	0.011	0.030	0.081	0.088	23.227
4.7	0.000	0.000	0.002	0.002	0.010	0.016	0.032	0.052	0.108	23.227

Midpoint	Ratio: 1860	657	189	62	19	6.3	2.1	0.65	0.13	Volume
4.9	0.000	0.000	0.000	0.005	0.009	0.015	0.069	0.114	0.174	23.227
5.1	0.001	0.000	0.000	0.005	0.014	0.028	0.092	0.130	0.206	23.227
5.3	0.001	0.000	0.000	0.003	0.017	0.048	0.104	0.203	0.304	23.227
5.5	0.000	0.000	0.001	0.009	0.026	0.074	0.183	0.280	0.413	23.240
5.7	0.000	0.002	0.001	0.014	0.045	0.123	0.229	0.503	0.661	23.276
5.9	0.001	0.007	0.003	0.012	0.063	0.134	0.359	0.668	0.918	23.336
6.1	0.001	0.004	0.007	0.017	0.088	0.212	0.442	0.801	1.160	23.420
6.3	0.000	0.004	0.003	0.022	0.098	0.262	0.619	1.129	1.573	23.529
6.5	0.000	0.000	0.008	0.035	0.123	0.308	0.738	1.365	2.082	23.663
6.7	0.001	0.004	0.007	0.045	0.141	0.391	0.996	1.731	2.435	23.822
6.9	0.001	0.007	0.014	0.047	0.174	0.509	1.110	2.093	2.872	24.006
7.1	0.003	0.000	0.016	0.052	0.183	0.571	1.291	2.477	3.384	24.216
7.3	0.002	0.006	0.016	0.059	0.209	0.632	1.548	2.732	3.888	24.452
7.5	0.002	0.004	0.012	0.079	0.242	0.646	1.610	3.126	4.472	24.716
7.7	0.001	0.012	0.018	0.077	0.247	0.743	1.846	3.496	4.721	25.006
7.9	0.002	0.004	0.017	0.070	0.250	0.788	1.892	3.543	4.996	25.325
8.1	0.001	0.003	0.024	0.081	0.267	0.802	1.912	3.729	5.067	25.673
8.3	0.004	0.006	0.025	0.074	0.271	0.786	1.886	3.661	5.263	26.050
8.5	0.003	0.006	0.018	0.063	0.272	0.788	1.968	3.952	5.246	26.459
8.7	0.002	0.011	0.023	0.074	0.267	0.823	1.994	3.843	5.541	26.899
8.9	0.001	0.011	0.031	0.061	0.277	0.843	2.129	4.084	5.662	27.372
9.1	0.002	0.008	0.016	0.065	0.294	0.902	2.218	4.395	6.038	27.878
9.3	0.002	0.009	0.033	0.095	0.313	0.992	2.303	4.866	6.470	28.420
9.5	0.002	0.013	0.024	0.099	0.333	1.031	2.727	5.054	7.005	28.999
9.7	0.000	0.008	0.028	0.099	0.333	1.094	2.691	5.343	7.726	29.615
9.9	0.002	0.006	0.033	0.090	0.414	1.212	2.965	5.969	8.264	30.272
10.1	0.002	0.009	0.038	0.114	0.396	1.273	3.443	6.805	9.044	30.969
10.3	0.001	0.014	0.040	0.113	0.454	1.421	3.602	6.940	9.858	31.710
10.5	0.004	0.011	0.036	0.131	0.478	1.555	3.965	7.826	10.829	32.497
10.7	0.003	0.009	0.036	0.135	0.503	1.596	4.090	8.292	11.914	33.331
10.9	0.006	0.024	0.051	0.152	0.542	1.760	4.407	8.547	12.398	34.214
11.1	0.009	0.011	0.036	0.162	0.576	1.851	4.637	8.818	13.488	35.150
11.3	0.008	0.019	0.068	0.162	0.608	1.904	4.683	9.883	13.773	36.142
11.5	0.003	0.013	0.065	0.162	0.616	1.892	5.079	10.303	14.417	37.191
11.7	0.003	0.025	0.058	0.166	0.591	2.000	5.283	10.161	14.951	38.302
11.9	0.006	0.020	0.051	0.168	0.607	2.121	5.210	10.393	15.176	39.477
12.1	0.004	0.018	0.050	0.167	0.604	2.068	5.202	10.687	15.205	40.722
12.3	0.005	0.006	0.042	0.165	0.574	1.957	5.310	10.990	15.424	42.039
12.5	0.003	0.020	0.063	0.183	0.581	2.011	5.208	10.796	15.206	43.433
12.7	0.003	0.011	0.059	0.153	0.603	1.984	5.289	10.630	15.483	44.910
12.9	0.002	0.011	0.059	0.169	0.645	1.915	5.306	10.800	15.151	46.475
13.1	0.007	0.015	0.067	0.165	0.594	1.836	5.282	10.373	15.338	48.134
13.3	0.004	0.009	0.067	0.183	0.615	1.919	5.222	10.372	15.249	49.893
13.5	0.007	0.014	0.057	0.164	0.636	1.951	5.291	10.306	15.170	51.761

Midpoint	Ratio: 1860	657	189	62	19	6.3	2.1	0.65	0.13	Volume
13.7	0.006	0.011	0.073	0.173	0.600	1.918	5.263	10.473	15.454	53.744
13.9	0.003	0.006	0.066	0.173	0.604	2.024	5.307	10.923	15.379	55.853
14.1	0.007	0.026	0.054	0.173	0.597	1.936	5.164	10.469	15.345	58.097
14.3	0.001	0.015	0.051	0.166	0.615	1.918	5.352	10.993	15.399	60.488
14.5	0.007	0.015	0.064	0.192	0.674	1.876	5.338	11.141	15.736	63.041
14.7	0.008	0.013	0.072	0.183	0.614	1.983	5.441	11.066	15.981	65.768
14.9	0.007	0.013	0.071	0.197	0.655	2.002	5.466	11.325	16.594	68.689
15.1	0.008	0.032	0.074	0.223	0.662	2.061	5.534	11.548	16.876	71.823
15.3	0.011	0.015	0.067	0.199	0.638	1.972	5.540	11.791	17.002	75.194
15.5	0.008	0.021	0.089	0.265	0.882	2.555	6.332	12.150	16.918	78.830
15.7	0.003	0.026	0.087	0.295	0.974	2.727	6.369	11.853	16.371	82.765
15.9	0.012	0.029	0.097	0.328	1.021	2.803	6.333	12.011	16.742	87.041
16.1	0.009	0.022	0.107	0.320	1.038	2.740	6.430	12.384	17.317	91.707
16.3	0.002	0.034	0.099	0.321	1.061	2.829	6.534	11.686	17.618	96.830
16.5	0.013	0.029	0.114	0.318	1.112	3.078	6.675	13.286	18.218	102.492
16.7	0.007	0.030	0.100	0.339	1.136	3.158	6.831	13.328	18.243	108.807
16.9	0.007	0.027	0.115	0.384	1.262	3.212	7.457	13.991	19.571	115.931
17.1	0.008	0.020	0.133	0.363	1.242	3.543	7.783	14.753	20.443	124.097
17.3	0.013	0.040	0.116	0.378	1.278	3.546	7.785	15.835	20.000	133.670
17.5	0.016	0.024	0.138	0.426	1.353	3.783	8.941	15.862	22.943	145.306
17.7	0.009	0.040	0.165	0.431	1.437	3.998	9.016	17.257	24.598	160.425
17.9	0.009	0.048	0.171	0.449	1.587	4.427	10.320	18.870	26.250	184.223
18.1	0.010	0.043	0.157	0.578	1.781	4.993	11.640	21.126	30.141	11525.603
18.3	0.277	0.755	2.773	7.938	25.984	70.098	164.347	311.002	435.252	4443.486
18.5	0.245	0.837	2.992	8.989	28.743	77.192	178.759	338.644	479.640	4443.486
18.7	0.273	0.812	3.054	8.754	28.272	77.333	177.384	336.137	472.075	4443.486
18.9	0.243	0.776	3.019	8.745	28.153	77.057	177.029	338.497	472.437	4443.486
19.1	0.253	0.825	2.846	8.650	28.157	76.209	176.999	334.184	470.611	4443.486
19.3	0.244	0.785	2.837	8.718	28.039	76.283	173.543	331.460	469.619	4443.486
19.5	0.242	0.793	3.036	8.700	28.222	76.209	176.387	329.962	469.245	4443.486
19.7	0.272	0.856	2.919	8.541	28.177	75.251	173.751	326.191	466.957	4443.486
19.9	0.284	0.914	2.903	8.519	27.812	75.480	173.722	330.494	461.228	4443.486
20.1	0.285	0.844	2.884	8.562	27.673	75.326	174.171	331.244	465.323	4443.486
20.3	0.238	0.827	2.895	8.524	27.769	74.656	174.063	328.982	461.713	4443.486
20.5	0.271	0.849	2.862	8.657	27.855	74.933	173.189	325.701	461.522	4443.486
20.7	0.253	0.801	2.812	8.669	27.479	74.974	171.737	329.548	459.575	4443.486
20.9	0.283	0.791	2.858	8.296	27.647	74.341	173.788	324.085	460.393	4443.486
21.1	0.257	0.771	2.849	8.494	27.407	74.584	172.740	325.453	457.724	4443.486
21.3	0.293	0.825	2.838	8.436	27.630	74.220	171.262	323.902	458.252	4443.486
21.5	0.261	0.800	2.894	8.403	27.134	74.033	169.870	321.946	455.616	4443.486
21.7	0.284	0.879	2.886	8.420	27.306	73.902	171.038	322.909	458.447	4443.486
21.9	0.248	0.863	2.838	8.400	27.451	74.132	171.620	323.414	456.519	4443.486
22.1	0.276	0.843	2.736	8.393	27.397	73.746	171.873	323.271	454.716	4443.486
22.3	0.266	0.872	2.861	8.524	27.370	73.706	169.368	322.359	453.783	4443.486

Midpoint	Ratio: 1860	657	189	62	19	6.3	2.1	0.65	0.13	Volume
22.5	0.267	0.857	2.753	8.300	27.017	73.669	170.175	320.914	456.748	4443.486
22.7	0.250	0.811	2.880	8.303	27.433	73.637	169.666	319.373	455.092	4443.486
22.9	0.281	0.840	2.891	8.338	27.202	73.159	169.940	323.510	458.236	4443.486
23.1	0.254	0.829	2.816	8.276	27.088	72.941	168.388	323.116	456.158	4443.486
23.3	0.275	0.799	2.869	8.350	27.142	73.011	170.521	323.126	456.471	4443.486
23.5	0.243	0.798	2.890	8.202	26.639	72.743	170.120	322.542	455.636	4443.486
23.7	0.274	0.857	2.785	8.210	27.010	73.168	169.653	323.217	453.229	4443.486
23.9	0.264	0.832	2.820	8.372	26.917	73.070	169.925	318.946	453.816	4443.486
24.1	0.275	0.835	2.845	8.374	27.141	73.233	169.502	320.981	451.481	4443.486
24.3	0.253	0.847	2.807	8.346	26.800	72.219	168.930	319.841	449.769	4443.486
24.5	0.293	0.796	2.845	8.355	27.072	72.879	168.050	319.314	450.335	4443.486
24.7	0.225	0.784	2.860	8.199	27.131	72.875	168.126	319.756	450.024	4443.486
24.9	0.266	0.815	2.713	8.277	26.845	72.689	167.364	317.025	452.331	4443.486
25.1	0.280	0.855	2.738	8.342	26.824	72.404	168.576	316.463	452.995	4443.486
25.3	0.283	0.811	2.760	8.356	26.539	72.506	168.870	318.599	449.658	4443.486
25.5	0.287	0.767	2.671	8.259	26.705	72.355	168.037	318.480	450.706	4443.486
25.7	0.255	0.755	2.707	8.177	26.690	72.318	167.586	311.435	448.417	4443.486
25.9	0.264	0.801	2.746	8.222	26.572	72.671	166.631	316.859	448.941	4443.486
26.1	0.258	0.820	2.791	8.223	26.634	72.482	168.072	317.485	447.563	4443.486
26.3	0.247	0.858	2.854	8.308	26.438	72.249	167.061	316.315	449.042	4443.486
26.5	0.272	0.871	2.799	8.191	26.715	71.944	167.017	317.714	445.835	4443.486
26.7	0.254	0.801	2.700	8.161	26.559	71.569	165.084	313.380	451.498	4443.486
26.9	0.246	0.867	2.758	8.243	26.753	71.664	166.290	317.359	443.497	4443.486
27.1	0.258	0.871	2.793	8.193	26.531	72.253	167.105	315.653	443.845	4443.486
27.3	0.232	0.825	2.688	8.091	26.353	71.788	166.428	315.685	447.454	4443.486
27.5	0.260	0.760	2.828	8.201	26.282	71.660	166.062	317.459	442.299	4443.486
27.7	0.268	0.799	2.772	8.121	26.388	71.406	166.094	313.827	445.032	4443.486
27.9	0.270	0.811	2.730	8.257	26.189	71.433	165.613	313.523	442.813	4443.486
28.1	0.265	0.787	2.797	8.213	26.162	71.712	164.498	314.982	442.289	4443.486
28.3	0.299	0.827	2.848	8.048	26.600	71.650	165.368	316.761	442.878	4443.486
28.5	0.276	0.801	2.712	8.109	26.271	71.297	164.461	314.626	443.066	4443.486
28.7	0.272	0.885	2.736	8.068	26.436	71.610	163.954	314.325	444.083	4443.486
28.9	0.265	0.817	2.765	8.089	26.394	71.015	164.689	314.399	442.449	4443.486
29.1	0.271	0.761	2.754	8.156	26.416	71.110	164.064	316.904	440.981	4443.486
29.3	0.264	0.824	2.873	8.157	26.388	71.164	165.430	315.679	443.369	4443.486
29.5	0.258	0.830	2.719	8.044	26.134	71.539	164.608	313.519	440.460	4443.486
29.7	0.275	0.836	2.697	8.135	26.395	71.033	164.689	313.259	437.908	4443.486
29.9	0.261	0.819	2.807	8.247	26.262	71.410	162.998	314.053	437.289	4443.486
30.1	0.277	0.775	2.712	8.106	26.190	71.158	165.372	316.897	442.780	4443.486
30.3	0.264	0.824	2.664	7.953	26.133	70.435	163.662	309.316	442.349	4443.486
30.5	0.235	0.765	2.671	8.066	26.040	71.036	164.907	308.667	442.670	4443.486
30.7	0.266	0.762	2.685	8.232	26.050	71.202	163.429	308.982	440.807	4443.486
30.9	0.259	0.815	2.663	8.106	25.914	70.799	163.926	309.781	440.328	4443.486
31.1	0.283	0.743	2.786	8.126	26.074	70.285	163.265	308.647	441.320	4443.486

Midpoint	Ratio: 1860	657	189	62	19	6.3	2.1	0.65	0.13	Volume
31.3	0.238	0.874	2.755	7.912	26.160	71.080	163.076	310.627	439.535	4443.486
31.5	0.240	0.739	2.669	8.081	25.944	70.442	166.035	312.118	442.809	4443.486
31.7	0.260	0.800	2.880	7.982	25.984	70.547	161.498	313.238	439.943	4443.486
31.9	0.229	0.738	2.675	7.871	25.903	70.175	162.284	313.781	437.877	4443.486
32.1	0.257	0.803	2.730	8.211	25.975	70.534	162.114	309.705	435.658	4443.486
32.3	0.238	0.868	2.686	7.955	25.732	70.572	164.599	309.321	437.611	4443.486
32.5	0.244	0.908	2.736	8.114	25.824	70.194	162.920	311.461	437.529	4443.486
32.7	0.275	0.775	2.651	7.939	25.724	69.840	161.619	307.910	439.537	4443.486
32.9	0.269	0.746	2.718	8.101	25.934	70.262	162.629	310.579	439.540	4443.486
33.1	0.245	0.740	2.686	8.005	26.007	70.771	162.726	311.031	435.809	4443.486
33.3	0.265	0.793	2.677	8.044	25.852	70.617	162.639	309.906	438.555	4443.486
33.5	0.235	0.741	2.630	8.183	26.002	70.870	162.385	306.929	437.650	4443.486
33.7	0.262	0.808	2.600	8.127	26.028	70.371	161.250	309.150	436.052	4443.486
33.9	0.268	0.738	2.723	8.053	25.732	69.902	161.965	308.660	439.238	4443.486
34.1	0.250	0.771	2.608	7.981	25.888	69.781	162.572	305.731	435.814	4443.486
34.3	0.282	0.753	2.621	7.926	25.918	70.110	161.796	307.121	436.419	4443.486
34.5	0.280	0.778	2.733	7.936	25.598	69.896	163.661	305.452	437.543	4443.486
34.7	0.258	0.793	2.617	7.900	25.658	70.112	162.080	305.818	437.059	4443.486
34.9	0.261	0.800	2.677	8.071	25.885	70.010	161.426	308.604	438.109	4443.486
35.1	0.261	0.857	2.623	8.016	25.972	69.631	161.975	308.770	435.433	4443.486
35.3	0.257	0.862	2.652	8.065	25.945	70.144	161.182	307.494	433.639	4443.486
35.5	0.282	0.743	2.644	8.007	25.807	69.387	161.548	306.476	433.749	4443.486
35.7	0.253	0.734	2.648	8.012	25.625	69.549	160.419	305.606	433.948	4443.486
35.9	0.268	0.803	2.600	8.037	25.676	69.752	161.551	306.360	433.274	4443.486
36.1	0.264	0.780	2.657	8.097	25.815	69.727	163.033	308.687	434.286	4443.486
36.3	0.258	0.781	2.618	8.023	25.623	69.347	160.695	308.064	431.804	4443.486
36.5	0.246	0.841	2.627	7.983	25.841	69.660	161.581	306.955	433.396	4443.486
36.7	0.205	0.748	2.645	7.928	25.863	70.213	160.035	309.927	433.977	4443.486
36.9	0.279	0.791	2.616	8.059	25.619	69.351	159.662	307.686	433.121	4443.486
37.1	0.241	0.703	2.659	7.935	25.598	69.980	159.873	310.234	431.811	4443.486
37.3	0.271	0.749	2.732	8.069	25.757	69.461	160.724	308.065	433.958	4443.486
37.5	0.259	0.791	2.650	8.024	25.566	69.281	161.409	304.519	430.143	4443.486
37.7	0.239	0.797	2.760	7.938	25.543	69.761	162.364	306.220	430.807	4443.486
37.9	0.229	0.748	2.653	7.980	25.614	69.398	160.873	306.279	434.561	4443.486
38.1	0.278	0.819	2.713	7.930	25.710	69.427	159.420	308.377	434.722	4443.486
38.3	0.263	0.738	2.715	7.964	25.754	69.120	160.033	310.523	434.613	4443.486
38.5	0.224	0.793	2.714	7.914	25.909	69.734	160.721	306.281	435.403	4443.486
38.7	0.254	0.781	2.630	7.971	25.639	69.733	160.237	309.964	434.297	4443.486
38.9	0.230	0.757	2.542	8.038	25.752	69.860	160.998	305.771	431.851	4443.486
39.1	0.260	0.746	2.742	7.911	25.611	69.453	161.350	304.286	434.830	4443.486
39.3	0.233	0.813	2.687	7.964	25.696	69.140	160.393	305.140	428.464	4443.486
39.5	0.243	0.815	2.673	8.001	25.663	68.817	159.682	302.007	433.635	4443.486
39.7	0.236	0.781	2.629	8.087	25.468	69.406	160.955	305.513	433.521	4443.486
39.9	0.249	0.760	2.763	7.875	25.536	69.398	161.451	306.105	436.000	4443.486

Midpoint	Ratio: 1860	657	189	62	19	6.3	2.1	0.65	0.13	Volume
40.1	0.249	0.719	2.723	7.952	25.464	68.635	160.799	304.403	433.420	4443.486
40.3	0.253	0.721	2.623	7.936	25.465	68.987	160.144	306.460	433.925	4443.486
40.5	0.240	0.738	2.690	7.875	25.460	69.587	162.302	308.500	431.534	4443.486
40.7	0.239	0.761	2.791	7.781	25.770	69.266	160.396	306.780	430.408	4443.486
40.9	0.295	0.790	2.686	7.949	25.544	69.385	161.103	304.025	432.496	4443.486
41.1	0.249	0.830	2.635	7.971	25.346	68.996	159.788	303.910	431.745	4443.486
41.3	0.267	0.850	2.645	7.794	25.642	68.714	160.694	304.691	429.284	4443.486
41.5	0.241	0.760	2.627	7.927	25.752	69.672	159.956	305.648	431.934	4443.486
41.7	0.285	0.798	2.686	7.879	25.218	69.637	160.594	305.107	433.045	4443.486
41.9	0.283	0.751	2.680	7.914	25.436	69.534	159.546	308.455	432.121	4443.486
42.1	0.275	0.748	2.722	8.006	25.429	69.172	161.248	303.027	432.357	4443.486
42.3	0.234	0.746	2.734	7.837	25.678	68.866	160.259	303.089	432.291	4443.486
42.5	0.246	0.751	2.642	8.101	25.711	69.492	159.664	304.134	433.511	4443.486
42.7	0.234	0.800	2.630	7.836	25.625	68.459	159.034	304.571	432.305	4443.486
42.9	0.253	0.712	2.651	7.871	25.560	68.954	160.426	304.242	427.820	4443.486
43.1	0.264	0.825	2.629	7.864	25.467	68.786	161.258	307.380	429.303	4443.486
43.3	0.263	0.813	2.596	7.837	25.393	69.053	160.104	305.285	434.642	4443.486
43.5	0.240	0.760	2.673	7.962	25.539	68.487	160.282	307.027	430.048	4443.486
43.7	0.243	0.775	2.703	7.919	25.581	69.177	160.961	304.531	430.382	4443.486
43.9	0.273	0.806	2.632	7.984	25.423	69.143	160.514	301.986	430.990	4443.486
44.1	0.249	0.755	2.607	7.858	25.152	68.881	160.022	303.370	430.231	4443.486
44.3	0.239	0.796	2.620	7.921	25.377	68.638	159.962	303.646	431.677	4443.486
44.5	0.244	0.803	2.754	7.897	25.193	69.045	159.791	306.467	429.832	4443.486
44.7	0.256	0.798	2.714	7.782	25.548	68.331	160.366	304.727	433.102	4443.486
44.9	0.254	0.705	2.809	7.925	25.511	68.710	160.635	303.085	429.836	4443.486
45.1	0.229	0.771	2.624	7.872	25.363	69.046	159.488	305.143	430.027	4443.486
45.3	0.272	0.795	2.662	7.849	25.437	68.970	159.617	302.640	429.538	4443.486
45.5	0.242	0.731	2.635	7.833	25.614	69.447	159.841	296.031	431.397	4443.486
45.7	0.270	0.815	2.752	7.806	25.511	69.814	159.074	305.468	427.698	4443.486
45.9	0.250	0.801	2.715	7.795	25.466	69.061	159.807	304.961	429.546	4443.486
46.1	0.265	0.750	2.658	7.915	25.389	68.836	159.101	300.564	430.893	4443.486
46.3	0.237	0.770	2.587	7.891	25.213	68.932	159.209	302.271	428.639	4443.486
46.5	0.236	0.805	2.588	7.937	25.221	68.122	160.079	304.008	432.210	4443.486
46.7	0.266	0.772	2.755	7.891	25.576	69.107	160.641	301.842	432.126	4443.486
46.9	0.266	0.813	2.701	7.890	25.430	68.324	159.892	304.351	431.320	4443.486
47.1	0.238	0.815	2.692	7.926	25.385	68.774	159.670	303.885	431.397	4443.486
47.3	0.290	0.760	2.711	7.907	25.507	69.124	160.560	305.776	429.953	4443.486
47.5	0.255	0.844	2.654	7.950	25.459	68.891	158.991	302.773	428.640	4443.486
47.7	0.254	0.825	2.788	7.896	25.491	69.454	158.642	305.168	430.293	4443.486
47.9	0.223	0.754	2.687	7.910	25.254	68.393	160.412	305.153	429.356	4443.486
48.1	0.267	0.823	2.682	7.887	25.420	68.788	159.318	303.471	430.184	4443.486
48.3	0.288	0.847	2.717	7.858	25.234	68.649	159.949	301.137	429.766	4443.486
48.5	0.238	0.778	2.633	7.937	25.461	69.157	160.122	300.564	429.939	4443.486
48.7	0.278	0.781	2.717	7.926	25.382	69.176	159.186	300.552	429.087	4443.486

Midpoint	Ratio: 1860	657	189	62	19	6.3	2.1	0.65	0.13	Volume
48.9	0.262	0.835	2.709	7.938	25.713	68.536	159.338	301.259	428.056	4443.486
49.1	0.246	0.722	2.685	7.869	25.628	68.904	159.630	305.784	430.986	4443.486
49.3	0.235	0.773	2.598	7.787	25.470	69.388	159.022	301.952	430.912	4443.486
49.5	0.256	0.758	2.630	7.805	25.275	68.625	160.006	300.856	430.120	4443.486
49.7	0.234	0.802	2.686	8.010	25.311	69.171	160.236	303.879	430.350	4443.486
49.9	0.232	0.802	2.703	7.853	25.250	69.552	160.529	300.520	430.195	4443.486
50.1	0.253	0.754	2.676	7.997	25.347	68.375	160.710	304.001	428.812	4443.486
50.3	0.257	0.855	2.677	7.986	25.420	68.998	160.507	305.269	427.915	4443.486
50.5	0.242	0.764	2.607	7.765	25.277	68.943	160.063	303.523	431.327	4443.486
50.7	0.257	0.734	2.707	7.899	25.326	68.695	160.022	305.347	427.092	4443.486
50.9	0.255	0.830	2.633	7.929	25.210	68.891	159.459	303.543	429.964	4443.486
51.1	0.239	0.714	2.678	7.980	25.350	68.836	159.671	303.383	428.920	4443.486
51.3	0.248	0.707	2.595	7.690	25.277	69.003	157.382	300.955	430.383	4443.486
51.5	0.281	0.737	2.638	7.864	25.174	67.811	158.639	301.121	429.152	4443.486
51.7	0.248	0.712	2.596	7.939	25.313	68.242	159.246	305.629	428.107	4443.486
51.9	0.241	0.813	2.646	7.900	25.552	68.769	158.540	298.309	429.804	4443.486
52.1	0.236	0.840	2.660	7.899	25.175	68.878	158.344	302.951	427.892	4443.486
52.3	0.249	0.705	2.634	7.900	25.315	68.904	160.803	301.900	431.026	4443.486
52.5	0.260	0.779	2.685	7.838	25.532	68.963	158.574	303.395	428.399	4443.486
52.7	0.263	0.835	2.601	7.817	25.293	69.006	157.955	306.198	429.683	4443.486
52.9	0.257	0.741	2.671	7.926	25.530	68.422	158.469	301.034	428.446	4443.486
53.1	0.244	0.658	2.753	7.847	25.303	68.633	159.738	301.002	426.819	4443.486
53.3	0.254	0.765	2.560	7.818	25.137	68.733	159.016	303.098	431.294	4443.486
53.5	0.256	0.760	2.707	7.948	25.317	68.522	158.877	305.798	432.466	4443.486
53.7	0.270	0.824	2.634	7.775	25.605	68.750	159.046	304.322	429.073	4443.486
53.9	0.259	0.700	2.626	7.784	25.526	68.704	158.445	301.692	430.919	4443.486
54.1	0.268	0.828	2.668	7.890	25.305	69.156	160.522	299.826	431.130	4443.486
54.3	0.225	0.778	2.657	7.867	25.727	68.844	158.397	303.031	430.680	4443.486
54.5	0.276	0.777	2.695	7.899	25.353	68.387	160.805	303.852	429.620	4443.486
54.7	0.286	0.796	2.683	7.856	25.466	67.796	160.623	303.415	428.568	4443.486
54.9	0.240	0.763	2.700	7.889	25.576	68.835	160.307	299.892	426.910	4443.486
55.1	0.284	0.748	2.603	7.728	25.108	67.716	158.405	300.966	428.506	4443.486
55.3	0.231	0.844	2.647	8.072	25.514	68.020	159.630	305.254	427.706	4443.486
55.5	0.258	0.740	2.657	7.964	25.596	68.496	159.085	300.659	430.983	4443.486
55.7	0.231	0.776	2.625	7.861	25.335	68.677	158.716	305.435	427.173	4443.486
55.9	0.236	0.756	2.677	7.899	25.534	69.224	158.179	306.446	428.273	4443.486
56.1	0.246	0.789	2.672	7.930	25.476	68.972	157.635	303.082	428.779	4443.486
56.3	0.279	0.756	2.708	7.823	25.307	68.615	159.269	305.519	426.098	4443.486
56.5	0.255	0.796	2.648	7.874	25.383	68.549	159.391	304.668	429.065	4443.486
56.7	0.262	0.783	2.790	7.745	25.317	68.478	160.189	304.205	427.706	4443.486
56.9	0.266	0.766	2.604	7.719	25.388	68.423	158.298	306.241	430.798	4443.486
57.1	0.270	0.761	2.669	7.859	25.619	68.371	160.560	305.173	428.962	4443.486
57.3	0.254	0.772	2.675	7.895	25.445	68.646	159.696	301.406	429.185	4443.486
57.5	0.276	0.851	2.633	7.887	25.463	68.975	159.433	305.005	428.927	4443.486

Midpoint	Ratio: 1860	657	189	62	19	6.3	2.1	0.65	0.13	Volume
57.7	0.266	0.711	2.676	7.968	25.453	68.431	157.095	301.196	430.745	4443.486
57.9	0.259	0.749	2.707	7.927	25.171	69.276	159.378	306.417	430.538	4443.486
58.1	0.255	0.771	2.626	7.890	25.284	68.723	157.100	300.399	428.940	4443.486
58.3	0.262	0.750	2.648	7.956	25.435	68.796	160.490	301.317	429.027	4443.486
58.5	0.260	0.758	2.671	7.822	25.315	68.338	158.797	300.216	427.017	4443.486
58.7	0.246	0.844	2.645	7.864	25.400	68.974	159.738	302.189	431.178	4443.486
58.9	0.237	0.778	2.706	7.911	25.516	69.418	158.893	301.299	430.929	4443.486
59.1	0.267	0.750	2.644	7.859	25.345	68.629	158.233	302.617	427.258	4443.486
59.3	0.242	0.779	2.609	7.844	25.222	69.002	158.945	302.738	427.822	4443.486
59.5	0.277	0.717	2.598	7.706	25.317	68.413	160.046	303.563	429.218	4443.486
59.7	0.262	0.744	2.669	7.934	25.311	68.712	157.912	302.384	426.294	4443.486
59.9	0.252	0.806	2.616	7.827	25.328	68.723	158.412	302.583	429.507	4443.486
60.1	0.249	0.805	2.606	7.920	25.304	68.903	157.989	304.325	427.111	4443.486
60.3	0.206	0.812	2.712	7.805	25.505	68.475	157.635	304.931	427.595	4443.486
60.5	0.247	0.787	2.701	7.877	25.226	68.957	157.105	305.441	429.228	4443.486
60.7	0.265	0.819	2.755	7.907	25.333	69.084	158.621	299.487	428.327	4443.486
60.9	0.269	0.797	2.730	7.836	25.263	68.638	159.137	303.152	428.277	4443.486
61.1	0.247	0.694	2.621	7.831	25.322	69.131	158.047	304.232	427.057	4443.486
61.3	0.271	0.754	2.570	7.904	25.249	68.360	162.002	303.194	427.007	4443.486
61.5	0.261	0.744	2.689	7.876	25.219	68.376	157.875	302.928	427.354	4443.486
61.7	0.267	0.847	2.575	7.818	25.511	68.699	157.957	304.166	428.050	4443.486
61.9	0.242	0.834	2.763	7.792	25.196	68.677	159.832	302.658	427.460	4443.486
62.1	0.264	0.798	2.573	7.845	25.405	68.630	158.855	303.197	429.078	4443.486
62.3	0.203	0.757	2.583	7.899	25.140	68.619	158.555	306.088	429.028	4443.486
62.5	0.238	0.775	2.656	7.816	25.382	68.607	159.391	302.033	430.305	4443.486
62.7	0.279	0.770	2.694	7.952	25.319	68.290	159.954	298.524	430.365	4443.486
62.9	0.241	0.812	2.647	7.917	25.318	69.047	158.847	301.761	430.928	4443.486
63.1	0.223	0.715	2.626	7.910	25.474	68.615	158.307	301.235	423.730	4443.486
63.3	0.231	0.779	2.668	7.850	25.394	67.827	157.636	302.848	426.737	4443.486
63.5	0.256	0.783	2.604	7.784	25.087	68.985	159.584	304.268	426.720	4443.486
63.7	0.297	0.816	2.708	7.893	25.431	68.845	159.944	306.281	427.930	4443.486
63.9	0.249	0.773	2.621	7.876	25.341	68.703	159.419	301.938	429.343	4443.486
64.1	0.273	0.741	2.657	7.706	25.429	69.128	158.135	301.938	425.898	4443.486
64.3	0.249	0.780	2.575	7.939	25.413	68.347	158.653	304.121	427.604	4443.486
64.5	0.251	0.760	2.711	7.849	25.421	68.415	158.520	300.980	427.848	4443.486
64.7	0.241	0.798	2.618	7.915	25.696	68.669	158.422	301.727	430.647	4443.486
64.9	0.242	0.739	2.588	7.734	25.245	68.511	159.581	300.937	427.570	4443.486
65.1	0.261	0.761	2.652	7.873	25.218	68.241	157.232	304.594	430.218	4443.486
65.3	0.247	0.750	2.732	7.765	25.419	68.239	159.417	302.992	425.829	4443.486
65.5	0.228	0.777	2.641	7.847	25.556	68.592	159.112	302.013	428.388	4443.486
65.7	0.253	0.782	2.682	7.944	25.426	68.782	158.099	301.143	427.869	4443.486
65.9	0.244	0.752	2.556	7.861	25.156	68.436	159.666	301.159	426.624	4443.486
66.1	0.258	0.796	2.728	7.878	25.308	68.095	158.943	306.391	425.967	4443.486
66.3	0.240	0.729	2.701	7.861	25.203	68.772	160.295	301.998	426.041	4443.486

Midpoint	Ratio: 1860	657	189	62	19	6.3	2.1	0.65	0.13	Volume
66.5	0.257	0.804	2.649	7.823	25.264	68.450	159.317	303.830	424.382	4443.486
66.7	0.257	0.814	2.629	7.751	25.112	68.709	159.844	302.112	427.610	4443.486
66.9	0.265	0.774	2.631	7.784	25.124	68.471	158.171	300.262	430.995	4443.486
67.1	0.244	0.731	2.683	7.872	25.249	68.964	159.772	300.706	426.313	4443.486
67.3	0.263	0.741	2.612	7.794	25.461	68.533	157.229	302.443	430.228	4443.486
67.5	0.292	0.777	2.625	7.750	25.340	68.664	159.655	304.389	427.509	4443.486
67.7	0.272	0.851	2.677	7.686	25.352	69.285	159.638	304.378	426.823	4443.486
67.9	0.250	0.797	2.678	7.719	25.330	68.746	158.254	301.069	428.318	4443.486
68.1	0.281	0.744	2.594	7.837	25.329	69.136	160.279	304.051	428.229	4443.486
68.3	0.273	0.722	2.664	7.873	25.094	68.029	158.474	302.066	428.791	4443.486
68.5	0.250	0.765	2.721	7.770	25.204	68.672	158.413	305.849	428.428	4443.486
68.7	0.246	0.817	2.691	7.854	25.447	69.054	158.917	303.372	427.976	4443.486
68.9	0.240	0.817	2.667	7.831	25.407	68.070	158.869	304.480	427.673	4443.486
69.1	0.247	0.820	2.618	7.941	25.274	68.572	159.527	304.916	426.907	4443.486
69.3	0.271	0.722	2.609	8.013	25.096	68.951	160.928	303.345	430.826	4443.486
69.5	0.212	0.755	2.786	7.762	25.325	68.386	159.419	304.012	427.954	4443.486
69.7	0.238	0.806	2.555	8.017	25.424	69.011	158.755	301.610	427.809	4443.486
69.9	0.258	0.719	2.662	7.815	25.241	67.880	160.006	300.435	426.432	4443.486
70.1	0.241	0.762	2.600	8.008	25.543	68.672	159.034	302.045	429.666	4443.486
70.3	0.269	0.781	2.756	7.853	25.293	69.146	159.892	299.003	430.648	4443.486
70.5	0.230	0.772	2.651	7.775	25.197	68.213	159.864	299.858	427.616	4443.486
70.7	0.254	0.767	2.605	7.854	25.493	68.142	159.661	302.662	426.005	4443.486
70.9	0.254	0.776	2.607	7.950	25.493	68.501	158.513	303.047	429.722	4443.486
71.1	0.225	0.679	2.592	7.881	25.087	68.653	158.657	303.827	428.630	4443.486
71.3	0.230	0.800	2.603	7.865	25.449	68.574	158.067	303.612	428.196	4443.486
71.5	0.232	0.793	2.621	7.923	25.405	68.286	159.237	303.578	429.541	4443.486
71.7	0.269	0.761	2.641	7.881	25.273	68.141	158.800	305.470	429.845	4443.486
71.9	0.245	0.809	2.696	7.824	25.313	68.127	158.895	302.938	431.131	4443.486
72.1	0.267	0.754	2.556	7.919	25.355	68.943	157.656	299.513	428.596	4443.486
72.3	0.245	0.814	2.675	7.824	25.319	67.997	159.167	302.387	429.414	4443.486
72.5	0.246	0.768	2.657	7.913	25.140	68.193	159.803	301.336	429.029	4443.486
72.7	0.245	0.786	2.597	7.807	25.251	68.843	159.048	300.521	427.541	4443.486
72.9	0.253	0.725	2.653	7.853	25.435	68.573	158.046	302.886	429.403	4443.486
73.1	0.276	0.779	2.636	7.744	25.260	68.361	158.867	301.442	424.907	4443.486
73.3	0.275	0.836	2.654	7.919	25.413	68.679	159.159	303.145	428.445	4443.486
73.5	0.263	0.830	2.665	7.881	25.674	68.798	157.330	302.168	426.399	4443.486
73.7	0.254	0.763	2.694	7.914	25.468	68.333	160.555	302.718	427.616	4443.486
73.9	0.271	0.745	2.635	7.763	25.343	69.249	159.471	304.557	428.385	4443.486
74.1	0.261	0.774	2.519	7.888	25.563	68.688	158.478	304.986	428.103	4443.486
74.3	0.258	0.736	2.622	7.851	25.332	68.434	159.765	305.421	428.053	4443.486
74.5	0.283	0.795	2.686	7.820	25.305	68.425	158.332	305.369	426.583	4443.486
74.7	0.281	0.765	2.622	7.763	25.329	68.125	158.597	305.364	429.545	4443.486
74.9	0.282	0.819	2.586	7.818	25.469	69.550	158.922	305.622	425.632	4443.486
75.1	0.258	0.786	2.618	7.880	25.259	68.117	159.593	300.787	425.940	4443.486

Midpoint	Ratio: 1860	657	189	62	19	6.3	2.1	0.65	0.13	Volume
75.3	0.247	0.763	2.702	7.731	25.446	68.974	159.220	303.177	428.612	4443.486
75.5	0.254	0.747	2.643	7.856	25.301	68.353	158.938	301.122	428.667	4443.486
75.7	0.231	0.783	2.566	7.913	25.297	68.776	159.217	304.337	428.435	4443.486
75.9	0.277	0.842	2.519	7.908	25.646	68.917	158.018	303.677	426.926	4443.486
76.1	0.254	0.852	2.621	7.886	25.526	68.873	159.940	302.665	427.627	4443.486
76.3	0.262	0.762	2.661	7.783	25.432	69.262	159.840	304.163	428.675	4443.486
76.5	0.268	0.734	2.647	7.762	25.317	68.786	158.059	301.073	431.360	4443.486
76.7	0.246	0.732	2.676	7.919	25.263	68.677	157.192	301.904	428.858	4443.486
76.9	0.257	0.810	2.727	7.996	25.221	68.966	158.639	300.696	426.669	4443.486
77.1	0.260	0.763	2.679	7.932	25.258	68.772	158.854	304.949	428.580	4443.486
77.3	0.252	0.800	2.673	7.723	25.400	68.065	160.137	302.558	431.357	4443.486
77.5	0.249	0.852	2.693	7.850	25.356	69.208	160.210	305.160	426.599	4443.486
77.7	0.273	0.694	2.625	7.869	25.418	68.426	158.996	302.879	429.380	4443.486
77.9	0.232	0.771	2.679	7.849	25.577	68.881	160.833	304.837	431.057	4443.486
78.1	0.238	0.751	2.760	7.835	25.343	68.639	158.639	301.930	429.130	4443.486
78.3	0.220	0.785	2.671	7.816	25.313	68.607	158.114	301.062	425.137	4443.486
78.5	0.283	0.729	2.667	7.836	25.388	68.336	159.753	302.069	431.289	4443.486
78.7	0.270	0.776	2.719	7.953	25.357	68.089	158.792	304.908	428.519	4443.486
78.9	0.254	0.838	2.652	7.836	25.137	68.866	159.301	305.509	429.818	4443.486
79.1	0.254	0.717	2.615	7.841	25.450	69.348	159.498	304.397	427.439	4443.486
79.3	0.252	0.789	2.619	7.826	25.471	68.556	158.180	300.114	428.637	4443.486
79.5	0.232	0.842	2.721	7.938	25.311	68.833	160.262	305.160	427.266	4443.486
79.7	0.266	0.717	2.677	7.878	25.427	69.134	160.270	300.826	429.196	4443.486
79.9	0.244	0.838	2.583	7.976	25.193	69.192	160.138	303.543	427.815	4443.486
80.1	0.270	0.787	2.670	7.831	25.607	68.801	160.215	306.833	429.576	4443.486
80.3	0.226	0.806	2.711	7.785	25.601	68.847	159.798	305.098	426.901	4443.486
80.5	0.255	0.819	2.717	7.949	25.497	68.665	159.220	304.718	428.011	4443.486
80.7	0.226	0.806	2.618	7.840	25.624	68.698	159.815	303.614	429.798	4443.486
80.9	0.244	0.730	2.685	7.958	25.619	68.709	158.891	301.343	431.377	4443.486
81.1	0.247	0.758	2.698	7.903	25.171	68.348	160.259	303.906	428.857	4443.486
81.3	0.247	0.808	2.680	7.867	25.524	68.983	158.423	305.431	426.573	4443.486
81.5	0.240	0.765	2.653	7.913	25.448	68.926	159.833	299.570	429.351	4443.486
81.7	0.251	0.785	2.704	7.921	25.549	68.723	158.065	301.468	427.467	4443.486
81.9	0.229	0.768	2.583	7.723	25.702	69.018	157.867	304.047	430.595	4443.486
82.1	0.250	0.832	2.599	7.868	25.502	68.578	157.671	300.045	427.511	4443.486
82.3	0.262	0.823	2.740	7.933	25.263	68.519	159.126	302.832	428.009	4443.486
82.5	0.270	0.802	2.687	7.959	25.219	69.011	158.823	300.404	427.118	4443.486
82.7	0.296	0.772	2.673	7.820	25.490	68.703	160.114	301.606	427.819	4443.486
82.9	0.282	0.805	2.672	7.892	25.359	68.777	158.515	303.909	431.292	4443.486
83.1	0.264	0.709	2.680	7.834	25.351	68.709	158.349	303.043	427.862	4443.486
83.3	0.271	0.802	2.783	7.959	25.620	69.010	157.858	303.172	426.158	4443.486
83.5	0.246	0.686	2.649	7.856	25.466	68.859	159.590	303.900	429.023	4443.486
83.7	0.283	0.744	2.643	7.808	25.221	68.934	159.060	302.140	432.181	4443.486
83.9	0.305	0.861	2.641	7.960	25.493	68.949	158.768	302.515	428.882	4443.486

Midpoint	Ratio: 1860	657	189	62	19	6.3	2.1	0.65	0.13	Volume
84.1	0.236	0.846	2.634	7.971	25.576	68.587	158.247	306.940	429.393	4443.486
84.3	0.255	0.839	2.648	7.924	25.545	68.879	160.338	301.239	426.656	4443.486
84.5	0.258	0.777	2.651	7.950	25.627	68.777	159.531	303.958	429.795	4443.486
84.7	0.256	0.773	2.723	7.748	25.360	69.177	161.272	310.149	428.163	4443.486
84.9	0.257	0.793	2.699	7.953	25.404	69.277	161.141	303.923	429.479	4443.486
85.1	0.271	0.735	2.626	7.806	25.317	69.048	160.381	306.771	429.266	4443.486
85.3	0.224	0.842	2.695	7.882	25.437	68.753	159.013	301.900	432.698	4443.486
85.5	0.261	0.829	2.656	7.919	25.620	68.870	159.230	303.556	430.891	4443.486
85.7	0.223	0.774	2.648	8.111	25.508	69.186	159.730	305.132	429.480	4443.486
85.9	0.245	0.738	2.620	7.889	25.354	69.061	158.761	303.601	429.977	4443.486
86.1	0.254	0.838	2.661	7.952	25.465	68.954	157.867	301.587	430.137	4443.486
86.3	0.257	0.834	2.702	7.857	25.510	68.608	159.654	304.999	430.058	4443.486
86.5	0.256	0.773	2.600	7.866	25.345	69.148	160.026	302.101	428.416	4443.486
86.7	0.235	0.733	2.653	8.050	25.657	69.037	161.839	305.188	430.517	4443.486
86.9	0.252	0.841	2.642	7.908	25.514	68.840	159.527	304.095	429.512	4443.486
87.1	0.267	0.751	2.633	7.884	25.558	68.827	160.412	305.384	429.901	4443.486
87.3	0.246	0.821	2.664	7.908	25.368	69.157	159.645	302.679	429.230	4443.486
87.5	0.280	0.729	2.791	7.998	25.553	68.799	158.922	305.181	431.280	4443.486
87.7	0.222	0.793	2.708	7.904	25.537	68.904	157.806	304.705	430.872	4443.486
87.9	0.256	0.798	2.530	7.923	25.554	68.565	159.794	303.632	427.865	4443.486
88.1	0.257	0.824	2.713	7.966	25.647	68.370	159.881	303.587	430.867	4443.486
88.3	0.269	0.785	2.721	7.969	25.621	68.672	158.972	302.300	431.290	4443.486
88.5	0.230	0.815	2.701	7.858	25.529	68.887	161.188	303.169	427.897	4443.486
88.7	0.269	0.758	2.702	7.964	25.564	68.912	159.619	304.493	430.768	4443.486
88.9	0.272	0.739	2.698	7.860	25.371	69.300	160.218	305.638	433.322	4443.486
89.1	0.281	0.752	2.710	7.914	25.495	69.228	159.403	303.968	427.929	4443.486
89.3	0.246	0.770	2.675	7.910	25.751	69.787	159.668	303.884	429.953	4443.486
89.5	0.277	0.720	2.712	7.922	25.440	69.597	160.443	304.509	430.363	4443.486
89.7	0.230	0.774	2.680	7.790	25.682	68.861	159.521	306.025	431.139	4443.486
89.9	0.259	0.791	2.673	7.930	25.706	69.296	159.057	301.983	429.285	4443.486
90.1	0.266	0.754	2.696	7.940	25.570	69.571	159.404	303.931	428.869	4443.486
90.3	0.279	0.810	2.796	7.932	25.718	68.412	159.554	306.254	427.053	4443.486
90.5	0.252	0.724	2.681	8.026	25.593	69.393	159.428	304.444	427.340	4443.486
90.7	0.265	0.716	2.603	7.935	25.401	69.121	160.114	299.328	430.050	4443.486
90.9	0.273	0.760	2.692	7.940	25.655	69.672	161.453	303.843	429.663	4443.486
91.1	0.245	0.817	2.615	8.001	25.718	69.380	160.544	304.507	429.162	4443.486
91.3	0.264	0.808	2.708	7.941	25.402	69.387	160.255	300.143	431.964	4443.486
91.5	0.273	0.819	2.749	8.074	25.621	69.107	160.059	301.414	431.209	4443.486
91.7	0.241	0.765	2.727	7.794	25.658	69.346	158.259	303.615	428.531	4443.486
91.9	0.240	0.798	2.820	7.984	25.586	69.371	159.946	303.491	430.144	4443.486
92.1	0.251	0.845	2.692	7.822	25.532	69.158	158.069	301.937	427.875	4443.486
92.3	0.265	0.784	2.669	8.036	25.352	68.577	159.789	304.183	429.813	4443.486
92.5	0.262	0.780	2.665	8.109	25.551	68.899	160.802	304.122	432.082	4443.486
92.7	0.254	0.763	2.635	7.887	25.765	68.701	160.183	301.925	427.200	4443.486

Midpoint	Ratio: 1860	657	189	62	19	6.3	2.1	0.65	0.13	Volume
92.9	0.254	0.761	2.688	7.888	25.583	69.245	160.529	302.576	429.662	4443.486
93.1	0.256	0.798	2.689	7.831	25.337	69.445	158.720	301.133	429.209	4443.486
93.3	0.279	0.799	2.715	7.899	25.472	68.795	159.824	302.070	427.109	4443.486
93.5	0.256	0.846	2.706	7.882	25.490	69.418	160.186	305.206	429.416	4443.486
93.7	0.272	0.786	2.610	7.903	25.382	68.987	158.443	306.029	430.768	4443.486
93.9	0.283	0.819	2.634	8.005	25.579	69.160	160.270	307.731	425.340	4443.486
94.1	0.275	0.736	2.760	7.916	25.458	68.560	158.095	304.161	427.694	4443.486
94.3	0.263	0.743	2.681	7.868	25.238	68.744	159.767	304.187	427.986	4443.486
94.5	0.236	0.801	2.607	7.954	25.353	69.368	158.012	302.564	428.781	4443.486
94.7	0.293	0.792	2.620	7.864	25.673	69.394	158.306	304.669	428.148	4443.486
94.9	0.236	0.753	2.718	7.879	25.366	68.960	158.640	302.943	428.110	4443.486
95.1	0.253	0.824	2.652	7.925	25.612	68.616	160.192	299.875	427.435	4443.486
95.3	0.257	0.776	2.719	7.914	25.278	68.697	160.159	305.231	425.254	4443.486
95.5	0.239	0.743	2.644	7.892	25.470	69.115	158.906	301.940	426.766	4443.486
95.7	0.268	0.789	2.505	7.896	25.376	68.250	157.612	301.202	423.913	4443.486
95.9	0.289	0.766	2.671	7.905	25.399	68.273	157.229	302.857	423.072	4443.486
96.1	0.258	0.740	2.624	7.877	25.549	68.104	158.222	299.509	426.509	4443.486
96.3	0.228	0.732	2.570	7.757	25.196	68.164	157.817	301.075	423.646	4443.486
96.5	0.235	0.803	2.743	7.896	25.250	68.516	159.073	303.367	420.944	4443.486
96.7	0.282	0.847	2.673	7.837	25.406	68.156	156.847	300.998	423.745	4443.486
96.9	0.255	0.788	2.666	7.844	25.114	68.373	158.701	301.417	425.279	4443.486
97.1	0.217	0.813	2.613	7.869	25.402	68.296	157.559	302.634	424.745	4443.486
97.3	0.249	0.770	2.659	7.992	25.371	67.826	158.326	299.873	424.687	4443.486
97.5	0.302	0.794	2.579	7.793	24.950	68.123	157.849	298.993	421.266	4443.486
97.7	0.264	0.847	2.584	7.831	25.018	68.811	157.548	299.439	423.024	4443.486
97.9	0.248	0.775	2.544	7.906	25.206	68.384	157.333	297.777	423.377	4443.486
98.1	0.261	0.806	2.664	7.746	25.378	68.113	156.904	297.056	421.270	4443.486
98.3	0.282	0.761	2.636	7.731	25.075	68.461	158.834	294.446	424.194	4443.486
98.5	0.243	0.753	2.646	7.886	25.249	68.144	155.018	297.568	421.102	4443.486
98.7	0.270	0.787	2.630	7.916	25.312	68.231	158.032	300.875	423.412	4443.486
98.9	0.254	0.784	2.643	7.754	25.274	67.596	158.473	298.615	423.490	4443.486
99.1	0.228	0.805	2.659	7.837	25.137	68.559	158.136	298.537	422.080	4443.486
99.3	0.244	0.775	2.695	7.837	25.007	68.185	158.016	297.838	422.692	4443.486
99.5	0.218	0.763	2.510	7.862	24.647	66.468	153.833	294.152	413.763	4443.486
99.7	0.000	0.000	0.000	2.777	0.000	0.001	0.008	0.000	0.004	4443.486

Table of K^+ population data for simulation with K^+ and Na^+ across bulk density ratios

	Population in histogram bin at K^+/Na^+ ratio:									
Midpoint	1860	657	189	62	19	6.3	2.1	0.65	0.13	Volume
-99.7	0.000	0.000	0.000	0.143	0.000	0.000	0.000	0.000	0.000	4402.894
-99.5	0.945	0.950	0.912	1.282	0.896	0.886	0.615	0.327	0.117	4402.894
-99.3	27.208	26.161	27.116	215.688	25.746	23.338	18.120	10.979	3.596	4402.894
-99.1	499.051	499.323	498.668	490.725	475.832	431.370	341.763	191.814	68.215	4402.894

Midpoint	Ratio: 1860	657	189	62	19	6.3	2.1	0.65	0.13	Volume
-98.9	499.445	500.439	496.368	489.849	471.466	429.255	338.651	192.314	67.534	4402.894
-98.7	500.931	497.133	497.944	490.377	472.586	430.691	337.947	194.134	68.201	4402.894
-98.5	497.959	501.230	496.552	489.454	472.038	431.337	339.577	192.959	67.488	4402.894
-98.3	497.219	502.182	494.271	486.553	471.747	427.506	336.124	191.439	67.767	4402.894
-98.1	499.395	500.232	495.990	489.358	472.651	427.386	336.824	192.714	67.276	4402.894
-97.9	495.958	488.461	495.642	487.385	471.827	427.075	339.468	193.275	67.934	4402.894
-97.7	493.579	496.356	493.765	488.897	470.358	430.388	339.128	193.045	67.398	4402.894
-97.5	495.631	493.630	496.523	488.748	471.963	427.604	336.484	195.755	66.953	4402.894
-97.3	498.729	494.885	493.955	487.570	470.435	426.712	338.629	191.490	67.379	4402.894
-97.1	497.450	495.946	493.554	488.775	471.932	427.424	340.122	194.328	67.359	4402.894
-96.9	495.484	497.587	495.175	490.476	474.023	427.604	338.800	195.010	67.910	4402.894
-96.7	498.090	501.251	493.756	487.240	471.098	428.281	333.053	194.623	67.547	4402.894
-96.5	493.820	493.549	492.968	486.428	472.213	428.644	337.309	194.006	68.250	4402.894
-96.3	498.019	492.607	491.151	490.277	470.642	431.467	338.705	192.391	67.023	4402.894
-96.1	496.289	492.834	494.193	490.712	470.568	429.783	338.409	193.618	67.956	4402.894
-95.9	497.192	493.440	494.407	490.348	470.393	431.143	340.197	192.176	67.946	4402.894
-95.7	495.416	500.768	491.386	490.120	472.378	429.322	334.855	195.228	67.329	4402.894
-95.5	497.239	497.549	494.709	490.956	469.812	428.705	336.867	192.617	67.943	4402.894
-95.3	495.996	495.473	494.787	485.850	472.888	429.637	338.206	193.906	67.518	4402.894
-95.1	493.547	494.789	496.940	490.980	472.470	430.326	339.275	194.868	68.156	4402.894
-94.9	498.950	491.882	496.809	492.040	473.642	431.002	338.012	193.472	68.205	4402.894
-94.7	498.395	498.336	494.263	488.062	472.434	428.437	339.122	193.485	67.895	4402.894
-94.5	498.200	496.822	495.951	490.589	471.543	429.564	336.703	194.564	67.823	4402.894
-94.3	496.667	498.029	495.650	491.871	471.279	428.097	337.994	193.324	68.227	4402.894
-94.1	496.307	493.388	496.347	490.509	472.586	428.984	339.467	194.281	68.311	4402.894
-93.9	502.111	496.059	491.367	486.227	472.546	431.522	339.869	193.293	67.734	4402.894
-93.7	498.241	493.445	494.017	485.281	475.289	427.243	336.738	194.571	68.496	4402.894
-93.5	500.537	494.172	498.687	491.929	476.252	432.777	341.308	193.999	68.099	4402.894
-93.3	495.862	502.889	497.923	490.588	474.376	431.358	339.344	196.247	67.825	4402.894
-93.1	502.350	499.526	494.364	489.055	475.846	432.014	341.520	194.786	68.313	4402.894
-92.9	499.116	501.613	495.677	489.876	473.009	431.021	340.710	195.369	68.612	4402.894
-92.7	498.702	493.574	498.217	488.013	475.356	429.139	339.052	194.645	68.855	4402.894
-92.5	498.334	492.627	496.328	488.792	473.508	426.931	343.043	193.929	68.718	4402.894
-92.3	497.676	502.080	497.554	491.454	472.279	430.115	340.079	194.476	69.106	4402.894
-92.1	495.761	495.994	495.026	491.047	471.435	428.566	340.461	195.417	68.455	4402.894
-91.9	495.293	504.391	500.946	489.909	471.307	431.914	338.945	196.590	68.337	4402.894
-91.7	498.510	499.785	496.985	489.902	472.264	430.553	337.326	194.201	68.064	4402.894
-91.5	499.409	497.896	496.863	489.560	473.851	428.485	339.006	197.576	68.241	4402.894
-91.3	498.451	497.083	496.698	489.828	475.229	431.218	339.699	193.560	67.709	4402.894
-91.1	496.025	498.645	496.748	493.183	476.247	431.843	338.106	195.612	68.523	4402.894
-90.9	492.129	499.194	494.998	491.331	471.628	430.929	339.941	194.565	69.156	4402.894
-90.7	499.641	496.403	496.408	491.254	474.724	433.887	343.129	195.408	68.356	4402.894
-90.5	497.041	497.770	493.662	489.734	474.522	432.049	338.841	196.706	68.596	4402.894
-90.3	497.794	498.841	497.509	492.037	473.326	432.274	339.991	196.027	68.544	4402.894

Midpoint	Ratio: 1860	657	189	62	19	6.3	2.1	0.65	0.13	Volume
-90.1	495.970	495.537	497.656	490.247	473.384	432.144	340.557	193.217	68.172	4402.894
-89.9	499.785	502.550	495.539	489.030	472.040	434.550	343.581	194.174	67.906	4402.894
-89.7	496.858	500.689	495.196	491.939	473.804	434.478	339.543	195.327	68.731	4402.894
-89.5	497.355	498.272	495.262	494.434	474.305	428.596	339.148	195.615	68.961	4402.894
-89.3	498.786	497.932	495.124	491.458	474.415	431.811	340.025	193.390	68.159	4402.894
-89.1	498.878	491.834	497.216	488.828	471.418	431.552	339.707	196.897	68.841	4402.894
-88.9	497.847	494.846	495.818	489.062	475.214	434.639	339.106	195.940	68.549	4402.894
-88.7	496.086	494.321	497.095	490.884	472.534	428.527	341.471	195.177	68.573	4402.894
-88.5	497.968	498.324	496.808	489.465	474.121	431.859	338.276	195.294	68.227	4402.894
-88.3	499.230	497.169	495.033	489.711	474.231	429.128	337.422	197.421	68.697	4402.894
-88.1	497.674	499.816	493.674	487.529	472.106	429.985	340.036	195.838	68.684	4402.894
-87.9	496.194	495.723	491.755	486.881	472.900	430.465	338.606	195.802	68.104	4402.894
-87.7	497.515	499.171	495.026	491.126	470.334	430.672	337.871	194.078	68.616	4402.894
-87.5	494.686	495.195	492.357	487.627	472.906	433.613	340.236	193.301	68.241	4402.894
-87.3	495.632	498.032	492.889	488.057	473.583	429.282	337.801	194.366	67.856	4402.894
-87.1	493.718	501.506	496.726	489.579	472.811	430.481	342.165	195.071	68.706	4402.894
-86.9	499.785	496.356	494.728	489.057	471.907	431.023	340.535	193.541	68.369	4402.894
-86.7	497.350	498.227	499.415	486.397	474.074	430.373	339.771	193.800	68.298	4402.894
-86.5	495.881	491.334	495.371	489.020	475.043	429.056	340.421	195.359	67.711	4402.894
-86.3	500.014	500.917	496.330	491.114	475.198	432.279	337.050	194.796	67.686	4402.894
-86.1	495.705	499.818	496.435	489.709	473.381	428.391	338.356	195.535	68.287	4402.894
-85.9	496.083	498.855	495.310	491.313	473.440	433.114	345.043	194.445	68.326	4402.894
-85.7	497.149	495.654	496.317	487.552	473.461	427.727	337.902	194.057	68.678	4402.894
-85.5	494.988	492.247	489.147	487.962	473.600	431.770	337.371	194.250	68.995	4402.894
-85.3	498.486	497.687	493.902	491.861	470.798	432.045	336.318	194.520	68.315	4402.894
-85.1	499.614	496.322	494.129	490.242	473.721	428.514	338.625	194.892	68.706	4402.894
-84.9	493.519	495.608	494.777	488.929	470.698	428.804	339.282	196.357	68.386	4402.894
-84.7	497.068	493.606	495.589	487.031	472.344	429.163	338.543	197.475	68.314	4402.894
-84.5	494.001	495.869	494.289	487.051	470.552	427.596	341.312	194.896	68.633	4402.894
-84.3	495.630	498.024	497.014	486.222	473.811	429.685	337.684	194.961	68.599	4402.894
-84.1	493.859	497.140	494.022	488.772	474.225	428.296	339.703	194.530	68.483	4402.894
-83.9	499.549	497.823	495.079	490.955	473.203	427.191	339.044	193.207	68.499	4402.894
-83.7	496.815	495.192	494.321	487.906	472.867	428.530	338.206	194.228	68.701	4402.894
-83.5	495.937	495.431	489.366	484.134	474.547	427.451	342.005	193.438	68.781	4402.894
-83.3	496.053	495.813	489.373	485.457	473.959	426.828	339.358	195.636	68.564	4402.894
-83.1	496.714	490.852	492.795	487.987	470.076	427.700	337.913	195.469	68.405	4402.894
-82.9	493.693	495.627	491.412	490.137	473.102	427.890	334.609	194.822	68.461	4402.894
-82.7	497.906	497.268	490.567	489.446	469.721	432.361	339.080	194.889	68.487	4402.894
-82.5	494.512	496.332	493.434	485.246	475.371	429.327	339.952	193.831	68.639	4402.894
-82.3	493.644	494.462	492.257	486.237	471.914	428.136	341.187	193.813	68.307	4402.894
-82.1	495.882	491.619	494.449	488.209	470.479	425.332	337.760	193.985	67.981	4402.894
-81.9	498.806	497.877	492.728	490.362	471.092	428.747	341.100	193.059	68.340	4402.894
-81.7	493.120	493.866	491.763	486.163	470.598	429.955	340.154	194.742	68.201	4402.894
-81.5	496.255	498.378	493.958	485.355	471.065	429.009	339.675	196.355	68.603	4402.894

Midpoint	Ratio: 1860	657	189	62	19	6.3	2.1	0.65	0.13	Volume
-81.3	497.269	494.185	492.683	489.008	472.889	431.665	341.147	194.548	68.473	4402.894
-81.1	498.129	499.161	495.180	488.575	470.692	427.172	333.227	194.065	68.268	4402.894
-80.9	492.694	491.214	490.869	488.995	473.016	427.598	338.780	196.673	68.303	4402.894
-80.7	492.025	491.867	496.819	489.307	472.442	427.706	336.099	195.531	68.090	4402.894
-80.5	490.716	497.888	492.951	486.910	472.690	430.172	336.640	194.526	68.730	4402.894
-80.3	493.312	495.174	495.257	488.790	472.898	426.847	338.436	193.282	68.162	4402.894
-80.1	494.696	491.934	493.451	488.344	469.585	430.754	337.756	192.913	68.286	4402.894
-79.9	497.145	490.251	492.603	489.060	469.928	427.061	341.726	194.506	68.532	4402.894
-79.7	493.522	493.829	491.735	484.395	471.788	425.924	340.259	194.415	67.909	4402.894
-79.5	492.828	495.121	488.662	487.923	470.650	427.483	337.893	194.622	67.692	4402.894
-79.3	497.554	498.393	492.882	484.792	472.073	427.790	340.719	194.298	69.030	4402.894
-79.1	498.350	496.938	493.807	487.669	469.550	429.707	335.599	193.490	68.194	4402.894
-78.9	494.381	491.366	491.491	485.939	470.357	424.339	337.527	195.372	67.865	4402.894
-78.7	493.240	494.820	494.352	488.416	472.065	430.586	337.297	195.833	68.549	4402.894
-78.5	491.642	499.995	497.587	485.891	471.711	427.604	340.641	193.981	68.416	4402.894
-78.3	492.180	489.725	495.077	485.915	469.832	428.197	337.237	193.457	68.823	4402.894
-78.1	493.543	489.868	491.014	488.417	468.802	429.060	338.306	194.669	67.699	4402.894
-77.9	494.674	492.999	493.485	486.969	472.601	424.514	340.448	194.813	68.508	4402.894
-77.7	493.946	490.439	494.234	488.683	469.124	428.335	340.858	190.572	68.598	4402.894
-77.5	491.624	497.171	490.390	486.477	472.123	427.403	335.710	193.668	68.039	4402.894
-77.3	493.152	497.517	492.553	487.080	470.904	430.044	338.582	195.083	67.734	4402.894
-77.1	493.871	495.182	495.567	487.920	473.641	427.174	336.395	193.072	68.064	4402.894
-76.9	495.685	497.875	491.867	487.598	470.220	429.564	339.840	195.075	68.635	4402.894
-76.7	493.114	492.755	491.916	488.135	470.700	427.575	338.886	193.329	68.158	4402.894
-76.5	493.976	491.597	489.510	485.831	473.618	428.770	337.517	193.103	67.871	4402.894
-76.3	492.698	488.987	495.078	484.158	470.260	426.521	336.437	192.969	68.219	4402.894
-76.1	494.249	499.302	491.248	484.982	470.989	425.435	335.785	193.698	68.140	4402.894
-75.9	496.032	494.210	490.725	485.900	472.231	428.464	341.346	193.301	68.447	4402.894
-75.7	492.829	496.708	491.858	485.991	470.644	425.643	338.076	193.850	68.400	4402.894
-75.5	491.343	492.025	494.270	486.750	470.639	429.901	339.163	194.575	68.735	4402.894
-75.3	494.391	494.834	489.758	485.174	469.887	427.520	339.416	195.531	68.329	4402.894
-75.1	491.696	492.931	492.718	482.998	471.841	428.875	340.190	192.741	68.154	4402.894
-74.9	498.201	495.134	492.913	488.165	470.507	429.420	335.924	194.914	68.024	4402.894
-74.7	491.931	496.663	487.775	484.654	470.104	426.369	334.936	192.432	67.958	4402.894
-74.5	495.328	491.802	491.873	487.431	470.455	428.397	336.101	194.952	67.772	4402.894
-74.3	492.780	498.639	491.421	487.396	471.190	428.035	337.590	193.668	67.834	4402.894
-74.1	494.063	501.619	496.519	486.435	471.910	428.962	338.329	193.506	68.553	4402.894
-73.9	495.783	500.080	493.012	485.856	467.696	426.419	341.093	196.191	68.516	4402.894
-73.7	492.081	494.538	491.856	485.457	469.756	429.261	338.132	194.916	68.941	4402.894
-73.5	496.995	496.634	487.943	488.535	467.798	430.276	338.317	197.446	68.282	4402.894
-73.3	493.999	495.200	490.634	482.275	467.524	427.215	342.314	195.017	68.142	4402.894
-73.1	492.439	491.014	493.841	485.381	471.571	429.618	339.634	191.011	68.014	4402.894
-72.9	493.075	492.896	488.811	486.053	468.891	428.231	335.622	194.050	68.372	4402.894
-72.7	490.806	492.282	492.035	485.718	472.203	427.560	342.422	194.185	68.283	4402.894

Midpoint	Ratio: 1860	657	189	62	19	6.3	2.1	0.65	0.13	Volume
-72.5	496.020	496.428	495.496	484.132	467.866	426.597	338.481	193.264	67.585	4402.894
-72.3	492.818	495.845	492.178	482.374	471.264	426.646	335.928	194.131	68.678	4402.894
-72.1	494.635	495.913	491.915	484.439	472.542	425.556	337.066	192.801	67.592	4402.894
-71.9	496.560	494.070	489.921	485.459	470.989	427.363	342.058	195.766	68.302	4402.894
-71.7	495.078	495.826	492.409	488.484	472.467	429.262	338.137	193.648	68.210	4402.894
-71.5	497.851	493.361	491.192	485.936	474.635	427.253	337.853	191.567	68.055	4402.894
-71.3	495.207	493.749	492.263	486.047	471.027	427.562	336.354	190.074	67.396	4402.894
-71.1	496.721	488.769	492.113	486.763	472.857	426.356	334.813	194.242	67.831	4402.894
-70.9	494.355	490.590	492.858	485.722	469.526	429.585	337.546	193.856	68.376	4402.894
-70.7	494.457	493.848	490.125	486.866	472.663	425.196	336.736	194.101	68.163	4402.894
-70.5	490.594	493.175	491.277	487.138	464.612	428.024	339.047	193.249	68.090	4402.894
-70.3	495.907	492.042	492.921	484.941	471.302	427.853	337.383	192.935	68.846	4402.894
-70.1	494.108	492.998	493.914	488.689	471.252	429.343	334.771	194.937	68.525	4402.894
-69.9	490.285	489.909	491.956	485.337	469.310	427.780	335.841	192.565	67.734	4402.894
-69.7	492.331	491.202	489.619	484.810	472.709	428.503	336.141	194.722	67.977	4402.894
-69.5	492.469	494.003	491.079	486.980	469.994	424.894	334.784	193.859	68.099	4402.894
-69.3	489.847	495.686	490.767	485.288	469.423	428.681	334.587	193.220	68.540	4402.894
-69.1	496.748	495.041	492.468	486.998	468.860	426.632	340.780	195.042	68.173	4402.894
-68.9	493.244	490.727	492.274	484.768	469.320	429.206	340.220	192.566	67.722	4402.894
-68.7	492.307	493.861	491.899	485.669	472.420	427.069	337.460	195.393	68.390	4402.894
-68.5	498.264	490.374	491.105	486.785	470.118	430.596	340.605	196.043	67.949	4402.894
-68.3	491.818	495.839	494.618	484.150	470.122	428.306	340.367	194.327	67.873	4402.894
-68.1	494.208	489.942	491.064	483.875	468.490	429.435	338.538	193.449	67.632	4402.894
-67.9	492.329	493.383	490.339	486.172	470.551	427.334	338.053	193.544	68.864	4402.894
-67.7	491.896	493.978	488.601	484.313	470.888	429.796	332.617	194.284	68.180	4402.894
-67.5	491.579	491.638	494.123	485.449	469.443	429.254	335.680	193.807	68.056	4402.894
-67.3	493.930	484.294	493.378	487.269	471.134	430.182	337.055	191.173	68.515	4402.894
-67.1	492.178	486.924	490.356	485.591	470.358	423.957	335.177	191.852	68.232	4402.894
-66.9	494.345	489.196	494.696	484.572	470.051	425.662	338.399	193.610	68.574	4402.894
-66.7	491.761	493.248	488.419	482.172	469.330	426.287	340.267	194.229	67.902	4402.894
-66.5	494.597	493.190	494.137	485.837	468.506	426.329	339.410	194.405	68.307	4402.894
-66.3	491.684	494.752	489.920	486.913	467.629	427.102	340.470	196.465	68.045	4402.894
-66.1	495.908	494.442	490.524	485.550	470.749	427.216	341.447	194.143	67.632	4402.894
-65.9	492.516	493.738	495.328	487.365	467.622	426.670	337.674	192.299	68.060	4402.894
-65.7	495.263	492.790	490.725	487.543	468.910	429.680	341.113	194.181	67.698	4402.894
-65.5	490.775	494.638	491.129	485.317	470.318	425.761	337.375	194.486	68.678	4402.894
-65.3	491.819	492.744	494.374	485.836	467.758	424.638	334.724	193.755	68.760	4402.894
-65.1	491.709	495.423	494.550	488.731	469.863	428.420	338.809	193.433	68.375	4402.894
-64.9	491.600	491.532	491.886	485.685	468.314	423.001	336.416	192.881	68.216	4402.894
-64.7	494.246	491.084	492.520	485.435	471.123	428.086	339.717	195.702	68.201	4402.894
-64.5	492.877	493.653	496.352	484.419	467.842	427.600	333.891	193.244	68.111	4402.894
-64.3	494.796	494.014	490.291	485.792	471.326	429.654	340.597	192.584	68.290	4402.894
-64.1	492.899	492.184	491.697	488.301	471.550	431.632	336.708	193.670	67.844	4402.894
-63.9	494.294	493.028	491.731	489.247	469.540	429.265	339.377	197.126	67.886	4402.894

Midpoint	Ratio: 1860	657	189	62	19	6.3	2.1	0.65	0.13	Volume
-63.7	496.676	494.596	490.526	484.501	469.184	427.585	338.116	194.137	68.277	4402.894
-63.5	494.843	495.196	491.526	483.859	470.956	427.287	340.939	193.975	68.081	4402.894
-63.3	495.425	490.122	493.159	486.694	472.124	429.195	338.482	193.593	68.506	4402.894
-63.1	494.400	493.341	492.167	486.876	469.929	425.942	338.139	192.182	67.899	4402.894
-62.9	492.684	492.225	492.777	483.414	470.573	427.946	337.029	193.614	68.413	4402.894
-62.7	497.475	493.344	491.280	484.203	468.046	430.232	334.856	194.085	68.844	4402.894
-62.5	494.891	494.671	491.347	486.743	470.402	426.943	335.105	192.521	68.597	4402.894
-62.3	490.177	494.234	491.042	487.040	471.413	427.634	338.486	194.508	68.398	4402.894
-62.1	494.531	492.168	488.969	490.292	471.224	427.841	335.768	196.049	68.139	4402.894
-61.9	496.324	493.846	493.946	486.022	470.230	424.566	338.923	195.870	68.474	4402.894
-61.7	494.856	493.052	491.674	483.709	469.793	428.957	338.333	195.016	68.294	4402.894
-61.5	492.409	496.450	494.535	484.662	471.232	431.084	340.761	194.277	68.359	4402.894
-61.3	497.269	494.925	491.953	486.373	469.660	424.939	338.634	194.870	68.203	4402.894
-61.1	495.298	491.931	490.616	490.372	469.697	430.711	342.827	192.998	68.541	4402.894
-60.9	495.335	498.306	493.373	486.853	471.411	426.838	340.340	193.918	67.956	4402.894
-60.7	492.685	498.568	489.388	490.624	466.750	426.804	339.351	194.705	68.150	4402.894
-60.5	489.798	494.051	495.354	484.642	472.468	429.406	336.642	193.608	67.696	4402.894
-60.3	489.278	494.622	492.924	488.414	470.777	431.145	337.267	195.343	67.903	4402.894
-60.1	494.231	490.220	490.311	487.086	471.935	428.310	340.454	194.223	68.670	4402.894
-59.9	496.906	493.888	490.502	487.139	470.821	426.390	339.303	193.495	68.929	4402.894
-59.7	491.728	492.481	489.449	487.433	470.788	428.573	339.007	194.804	68.062	4402.894
-59.5	494.079	491.077	491.519	486.749	470.495	429.276	339.887	193.468	67.706	4402.894
-59.3	493.903	491.620	492.993	487.683	469.661	426.470	340.139	193.320	68.403	4402.894
-59.1	494.118	494.658	488.011	486.361	466.774	426.408	339.478	194.198	68.527	4402.894
-58.9	490.861	493.784	492.575	488.579	467.973	429.461	335.647	194.509	68.214	4402.894
-58.7	496.249	497.742	494.891	485.412	469.303	428.624	337.880	194.046	68.285	4402.894
-58.5	494.134	498.700	492.289	484.022	472.024	427.121	337.232	194.753	67.900	4402.894
-58.3	499.725	494.358	493.026	484.700	470.844	428.612	337.930	193.303	68.677	4402.894
-58.1	496.084	494.713	493.289	488.409	470.883	428.383	337.217	194.871	68.233	4402.894
-57.9	497.291	496.528	492.622	490.038	471.770	428.104	336.769	195.259	67.808	4402.894
-57.7	494.479	489.073	493.579	485.731	468.574	427.291	336.890	193.104	68.619	4402.894
-57.5	494.995	488.359	493.861	486.617	471.865	426.249	339.260	195.087	68.178	4402.894
-57.3	490.536	494.488	490.578	484.896	468.635	428.741	335.659	192.591	68.098	4402.894
-57.1	493.112	495.183	492.172	489.375	470.078	425.996	339.397	194.208	68.663	4402.894
-56.9	492.154	494.272	491.104	484.885	469.841	428.065	341.876	194.759	68.360	4402.894
-56.7	497.298	490.139	493.589	485.899	472.138	424.848	334.917	194.299	68.261	4402.894
-56.5	491.355	494.090	490.745	488.616	468.343	426.419	336.474	194.676	68.572	4402.894
-56.3	493.873	496.614	488.698	486.641	470.101	427.500	340.158	192.179	68.566	4402.894
-56.1	494.183	493.291	493.561	484.438	470.184	428.726	339.225	191.857	68.169	4402.894
-55.9	495.627	492.855	491.074	486.858	470.354	428.003	340.451	193.967	68.755	4402.894
-55.7	494.298	494.960	491.296	486.566	471.071	428.193	338.195	192.508	67.982	4402.894
-55.5	494.854	495.583	493.591	484.297	469.276	430.485	337.757	194.933	68.495	4402.894
-55.3	495.802	494.298	492.504	487.779	471.215	427.035	337.818	195.295	67.620	4402.894
-55.1	490.642	489.845	493.397	489.293	472.711	428.542	339.074	194.756	68.385	4402.894

Midpoint	Ratio: 1860	657	189	62	19	6.3	2.1	0.65	0.13	Volume
-54.9	495.734	497.291	489.969	486.083	472.498	428.918	338.598	194.374	68.640	4402.894
-54.7	497.417	492.934	493.265	485.124	472.527	426.709	337.104	195.020	68.593	4402.894
-54.5	498.375	490.684	494.567	483.915	471.546	429.767	337.971	195.144	68.549	4402.894
-54.3	493.828	496.325	492.905	488.490	471.014	425.811	338.943	195.812	67.901	4402.894
-54.1	494.175	493.028	493.259	486.527	473.400	426.821	336.462	193.992	68.683	4402.894
-53.9	495.835	491.865	493.840	485.893	469.269	426.039	340.842	197.147	68.264	4402.894
-53.7	495.607	492.338	496.010	487.163	474.697	429.294	336.834	195.074	67.835	4402.894
-53.5	497.201	492.171	493.079	486.421	471.219	428.746	339.744	195.602	67.972	4402.894
-53.3	495.371	495.047	492.881	489.059	473.239	428.430	338.617	191.710	68.398	4402.894
-53.1	494.243	491.834	488.958	488.294	468.095	426.325	336.908	195.130	68.634	4402.894
-52.9	494.692	492.247	492.430	490.821	473.140	434.439	338.493	193.367	68.455	4402.894
-52.7	490.935	492.473	493.513	487.250	471.008	428.592	339.435	196.393	68.197	4402.894
-52.5	493.146	493.274	490.690	486.113	472.191	430.253	336.416	193.690	68.005	4402.894
-52.3	494.369	494.895	494.066	487.453	467.918	427.625	337.598	192.188	68.573	4402.894
-52.1	495.010	495.394	494.839	487.372	467.326	428.731	338.058	194.871	68.272	4402.894
-51.9	491.740	492.101	491.183	485.209	472.710	426.708	339.777	192.724	67.754	4402.894
-51.7	496.580	499.347	489.610	487.897	468.218	428.110	341.882	194.956	68.641	4402.894
-51.5	496.890	494.387	489.349	488.211	472.314	428.011	341.369	195.379	68.136	4402.894
-51.3	491.394	494.820	497.658	490.713	470.244	428.597	337.421	194.491	68.525	4402.894
-51.1	494.505	496.014	490.190	487.868	469.894	429.169	337.419	193.001	67.944	4402.894
-50.9	494.536	493.383	493.230	484.654	472.256	431.047	337.742	193.239	68.430	4402.894
-50.7	493.176	496.349	493.731	485.898	471.750	425.914	337.961	194.563	68.658	4402.894
-50.5	495.793	488.366	493.012	488.363	470.894	429.907	335.832	193.814	69.071	4402.894
-50.3	492.950	492.554	489.226	485.683	475.556	429.035	338.329	195.999	68.180	4402.894
-50.1	493.817	492.756	492.329	486.092	471.375	429.668	334.051	193.680	68.587	4402.894
-49.9	495.189	490.945	489.915	485.828	470.419	430.473	340.519	195.082	68.008	4402.894
-49.7	495.266	494.206	494.647	486.863	473.386	426.868	337.912	195.730	68.379	4402.894
-49.5	493.212	493.673	494.209	487.136	470.080	427.349	339.597	195.805	69.023	4402.894
-49.3	492.784	495.852	493.831	488.385	474.305	430.437	338.544	194.382	68.503	4402.894
-49.1	494.465	495.547	493.386	490.766	472.126	431.350	335.948	193.734	68.726	4402.894
-48.9	494.614	490.270	494.289	489.170	470.276	427.751	337.149	194.253	68.339	4402.894
-48.7	495.559	496.452	493.998	485.855	468.883	426.382	336.916	196.322	68.285	4402.894
-48.5	493.644	492.448	491.785	489.176	470.878	429.442	341.906	197.439	68.106	4402.894
-48.3	490.985	493.240	495.767	487.148	472.275	432.319	334.440	192.798	68.450	4402.894
-48.1	494.651	500.491	492.674	487.690	473.136	432.046	340.312	197.610	68.434	4402.894
-47.9	497.274	496.332	493.925	489.761	469.338	427.685	339.335	193.470	68.401	4402.894
-47.7	494.937	496.434	500.282	488.277	473.396	427.824	336.831	194.474	68.076	4402.894
-47.5	493.002	494.518	490.232	486.453	471.851	427.590	342.144	194.128	68.411	4402.894
-47.3	497.008	503.118	495.552	489.462	471.454	430.568	338.924	196.250	68.624	4402.894
-47.1	495.733	496.893	492.158	487.154	471.900	429.344	341.829	196.166	68.810	4402.894
-46.9	497.379	496.021	495.537	489.661	475.622	428.607	340.130	192.555	68.932	4402.894
-46.7	492.717	497.836	491.006	486.300	472.582	423.840	336.839	195.779	68.071	4402.894
-46.5	496.434	497.092	495.322	490.498	471.642	429.202	338.963	196.554	68.329	4402.894
-46.3	496.879	499.099	495.221	489.596	476.619	427.763	340.225	194.209	68.277	4402.894

Midpoint	Ratio: 1860	657	189	62	19	6.3	2.1	0.65	0.13	Volume
-46.1	494.775	493.887	491.005	488.400	470.876	429.137	336.460	193.255	68.264	4402.894
-45.9	497.152	493.191	496.324	487.856	468.839	428.796	336.859	194.161	68.624	4402.894
-45.7	495.720	492.360	498.151	488.854	472.826	431.597	339.722	195.691	68.655	4402.894
-45.5	498.271	501.357	494.432	489.823	469.481	428.899	337.567	194.961	68.622	4402.894
-45.3	499.933	494.626	493.571	489.407	473.764	428.639	345.656	192.559	68.577	4402.894
-45.1	496.074	494.835	494.778	489.969	472.235	434.376	339.757	195.218	68.057	4402.894
-44.9	493.478	502.089	494.604	487.882	473.171	431.472	339.889	194.921	68.513	4402.894
-44.7	492.872	496.689	496.232	487.968	470.319	430.562	340.116	194.625	68.345	4402.894
-44.5	499.639	493.847	492.442	488.350	472.209	428.975	341.104	195.177	68.099	4402.894
-44.3	495.730	499.728	497.367	489.754	472.303	431.297	339.367	194.272	69.103	4402.894
-44.1	494.390	491.302	497.850	488.954	473.039	430.579	343.184	194.315	68.144	4402.894
-43.9	496.002	495.132	497.514	487.483	470.670	429.395	340.689	197.931	68.445	4402.894
-43.7	496.254	491.218	496.127	490.557	473.980	429.427	339.262	194.570	68.556	4402.894
-43.5	498.277	500.203	495.366	489.426	470.684	431.046	343.884	197.825	68.454	4402.894
-43.3	495.301	495.895	496.637	491.153	470.464	429.746	341.277	193.662	69.016	4402.894
-43.1	494.149	494.545	497.033	489.052	472.651	431.985	339.896	196.998	69.048	4402.894
-42.9	498.681	493.479	498.005	489.536	473.457	429.716	340.685	193.692	68.830	4402.894
-42.7	496.680	496.950	497.186	489.569	477.249	430.301	341.517	194.354	68.796	4402.894
-42.5	500.938	501.071	495.224	488.993	469.395	431.479	339.627	194.990	68.655	4402.894
-42.3	497.666	495.548	493.523	489.549	475.351	433.519	339.547	196.767	68.118	4402.894
-42.1	496.282	498.537	498.110	491.724	474.378	430.897	341.075	195.379	68.573	4402.894
-41.9	498.636	496.353	497.774	489.339	473.384	433.462	339.722	196.124	69.285	4402.894
-41.7	500.300	494.131	494.010	491.065	471.895	431.420	340.282	196.479	68.682	4402.894
-41.5	501.217	492.280	493.781	488.686	473.899	431.971	342.286	195.524	68.319	4402.894
-41.3	497.406	496.166	498.939	490.417	473.465	429.036	338.911	195.967	68.551	4402.894
-41.1	499.337	499.274	494.348	487.641	476.095	430.978	340.492	195.021	68.972	4402.894
-40.9	501.838	497.735	498.333	490.730	471.711	432.917	341.566	196.675	69.173	4402.894
-40.7	495.902	499.995	495.522	489.227	473.811	431.217	340.802	195.488	69.046	4402.894
-40.5	497.937	499.015	498.182	488.703	476.123	429.715	342.769	196.042	68.360	4402.894
-40.3	500.733	497.357	497.525	487.421	473.346	428.661	339.476	195.163	68.877	4402.894
-40.1	496.288	497.814	498.251	493.144	476.234	429.502	340.390	194.401	68.574	4402.894
-39.9	493.406	499.242	494.542	495.735	475.446	432.509	343.095	196.004	69.024	4402.894
-39.7	503.142	503.056	498.507	490.799	475.625	433.663	343.401	196.349	69.256	4402.894
-39.5	496.042	497.539	497.528	490.255	475.308	433.589	344.246	195.725	68.882	4402.894
-39.3	494.920	497.346	495.553	489.944	477.286	434.157	339.865	194.502	68.630	4402.894
-39.1	498.288	498.260	498.592	492.183	475.053	430.235	340.280	196.729	69.065	4402.894
-38.9	500.924	504.717	494.609	493.738	475.878	431.570	338.707	194.765	68.546	4402.894
-38.7	499.442	502.087	501.203	496.461	474.305	430.460	340.682	196.120	69.361	4402.894
-38.5	500.247	495.419	500.091	494.651	477.550	436.163	341.029	199.542	69.114	4402.894
-38.3	503.566	503.155	501.146	491.449	474.896	432.791	340.796	195.356	69.354	4402.894
-38.1	501.735	501.469	497.089	491.454	476.940	430.849	336.739	198.783	68.685	4402.894
-37.9	500.771	496.455	499.186	493.638	474.331	430.806	343.139	197.597	69.094	4402.894
-37.7	497.882	497.355	499.822	491.601	474.830	434.138	348.258	198.912	69.311	4402.894
-37.5	499.899	500.159	500.195	493.244	475.570	431.385	343.573	196.188	69.298	4402.894

Midpoint	Ratio: 1860	657	189	62	19	6.3	2.1	0.65	0.13	Volume
-37.3	502.950	503.027	499.838	495.705	476.732	435.278	340.578	196.866	69.444	4402.894
-37.1	499.707	502.080	500.164	494.135	478.581	435.534	337.308	196.532	69.565	4402.894
-36.9	502.076	502.698	500.271	494.611	477.888	433.737	343.206	195.935	69.456	4402.894
-36.7	501.456	498.009	496.855	495.187	476.368	435.499	346.978	197.752	69.642	4402.894
-36.5	502.408	505.125	496.259	495.052	477.384	432.400	342.902	196.646	69.209	4402.894
-36.3	499.430	502.065	497.300	495.113	478.518	437.465	343.065	198.608	68.991	4402.894
-36.1	496.844	500.483	501.541	494.859	480.008	432.794	342.946	197.370	68.523	4402.894
-35.9	498.991	500.457	498.549	495.089	476.592	434.813	344.209	197.385	69.156	4402.894
-35.7	503.237	500.708	502.838	497.169	477.685	432.607	344.553	198.540	69.569	4402.894
-35.5	501.258	503.602	497.717	495.697	479.358	435.077	341.386	196.429	68.998	4402.894
-35.3	502.788	496.927	499.258	495.105	479.537	437.148	344.113	197.514	69.513	4402.894
-35.1	503.872	502.923	496.401	492.217	478.220	433.050	344.217	196.385	69.301	4402.894
-34.9	503.532	502.912	503.722	494.264	477.660	437.153	341.580	195.315	69.247	4402.894
-34.7	502.713	502.969	503.653	495.439	480.324	432.657	346.640	197.483	69.445	4402.894
-34.5	503.430	504.911	500.440	494.851	478.256	435.409	347.913	198.137	69.652	4402.894
-34.3	499.158	507.476	505.158	497.180	481.582	436.253	345.470	199.170	68.932	4402.894
-34.1	497.580	503.371	500.063	498.656	479.657	435.674	344.328	196.976	69.889	4402.894
-33.9	504.332	502.975	505.830	498.004	478.777	435.500	344.739	197.255	69.897	4402.894
-33.7	502.023	503.076	499.797	497.308	482.178	437.269	344.379	198.293	69.680	4402.894
-33.5	504.381	500.643	500.073	498.364	478.165	433.256	343.880	196.997	69.934	4402.894
-33.3	507.878	509.148	501.535	498.175	480.292	440.071	348.191	198.279	69.585	4402.894
-33.1	504.378	507.075	501.293	495.037	479.602	438.923	346.709	197.316	69.895	4402.894
-32.9	503.720	504.964	505.692	497.542	480.147	439.226	346.146	198.163	69.305	4402.894
-32.7	506.257	505.903	509.184	499.141	482.368	439.982	345.096	198.083	70.145	4402.894
-32.5	508.011	510.003	503.220	499.388	483.060	437.020	345.177	197.977	69.885	4402.894
-32.3	509.890	502.602	503.902	498.063	480.678	436.442	345.915	199.759	69.734	4402.894
-32.1	506.230	510.086	505.221	498.615	484.054	439.941	346.311	198.751	70.114	4402.894
-31.9	508.462	504.159	504.854	497.249	482.591	437.032	344.821	199.160	69.944	4402.894
-31.7	504.261	506.534	508.211	499.660	480.271	438.060	344.751	199.674	69.972	4402.894
-31.5	510.595	503.705	505.719	497.465	484.214	442.112	346.813	198.777	70.181	4402.894
-31.3	506.988	505.862	508.502	504.450	483.930	437.947	347.314	199.395	70.158	4402.894
-31.1	505.080	509.224	504.321	502.497	482.799	440.920	347.710	199.283	70.600	4402.894
-30.9	505.633	505.587	506.813	500.731	485.408	437.546	348.443	200.404	70.209	4402.894
-30.7	513.125	509.743	509.973	501.644	484.866	438.378	346.049	198.172	70.460	4402.894
-30.5	507.519	508.054	506.576	503.596	485.117	439.963	346.301	198.195	70.260	4402.894
-30.3	511.677	511.305	507.874	501.852	484.762	438.688	345.913	199.830	70.636	4402.894
-30.1	510.522	510.530	502.979	501.876	484.845	444.506	350.366	198.391	70.372	4402.894
-29.9	508.972	508.456	508.381	501.829	487.410	441.529	348.271	200.500	70.252	4402.894
-29.7	511.211	510.743	507.669	503.847	485.722	444.018	345.559	199.322	70.258	4402.894
-29.5	505.784	512.311	510.382	506.733	487.645	444.773	351.216	200.260	70.213	4402.894
-29.3	512.006	510.317	509.538	503.253	488.970	441.040	350.861	200.766	70.130	4402.894
-29.1	513.571	513.282	512.121	508.080	485.949	440.446	349.520	200.201	70.776	4402.894
-28.9	511.942	508.752	512.390	505.293	489.177	441.634	347.278	200.189	70.993	4402.894
-28.7	513.568	511.330	510.760	505.329	488.158	444.019	347.252	201.327	71.467	4402.894

Midpoint	Ratio: 1860	657	189	62	19	6.3	2.1	0.65	0.13	Volume
-28.5	513.658	507.569	511.611	504.513	489.777	443.995	352.368	198.659	70.759	4402.894
-28.3	514.578	516.263	509.198	506.585	491.852	444.956	351.690	200.788	70.528	4402.894
-28.1	514.590	512.160	513.993	506.502	488.393	443.008	349.130	204.290	70.639	4402.894
-27.9	516.440	514.084	510.854	507.551	489.344	445.414	352.510	202.498	70.752	4402.894
-27.7	511.956	517.098	515.897	507.924	491.299	444.537	349.906	200.969	70.957	4402.894
-27.5	516.488	515.748	515.586	509.968	490.055	445.054	354.213	203.848	71.171	4402.894
-27.3	513.478	512.302	516.115	504.449	493.485	443.285	350.339	201.583	71.101	4402.894
-27.1	517.283	514.652	512.017	508.643	491.798	445.474	352.454	199.878	71.355	4402.894
-26.9	517.120	515.216	515.646	508.609	492.565	443.022	355.249	199.627	70.629	4402.894
-26.7	520.788	519.272	516.480	509.654	492.779	446.365	349.976	200.547	70.795	4402.894
-26.5	518.960	517.157	516.958	508.824	494.781	451.098	353.020	203.080	71.375	4402.894
-26.3	517.850	521.460	516.738	509.376	493.497	448.536	352.951	201.573	71.637	4402.894
-26.1	517.708	515.438	517.214	511.316	493.057	449.180	354.524	204.073	70.919	4402.894
-25.9	517.375	519.712	517.889	510.818	491.275	449.055	353.147	203.095	71.408	4402.894
-25.7	519.055	519.840	519.767	512.875	494.509	451.315	355.170	201.806	71.265	4402.894
-25.5	521.506	518.783	519.244	512.146	496.299	449.643	352.745	203.269	71.157	4402.894
-25.3	524.610	520.326	519.557	511.621	495.942	450.322	358.070	204.518	71.393	4402.894
-25.1	518.580	520.020	517.585	512.379	495.798	452.538	354.274	204.691	71.496	4402.894
-24.9	523.465	521.801	517.465	514.730	497.198	450.797	355.579	203.252	71.628	4402.894
-24.7	521.434	518.452	518.176	515.042	497.835	452.803	358.585	202.876	72.083	4402.894
-24.5	526.939	520.562	521.620	514.593	498.631	451.720	358.241	206.027	72.065	4402.894
-24.3	521.462	522.701	522.697	516.450	499.386	455.054	355.389	204.516	72.063	4402.894
-24.1	524.173	523.387	521.349	516.339	497.894	454.577	358.640	203.641	71.829	4402.894
-23.9	524.854	520.361	523.545	516.264	497.229	455.209	355.489	205.143	71.174	4402.894
-23.7	523.976	525.112	522.892	517.252	500.482	454.109	360.114	203.901	71.802	4402.894
-23.5	525.499	526.272	523.613	518.024	501.403	453.215	358.482	205.455	71.932	4402.894
-23.3	525.414	527.900	525.366	518.896	500.008	457.829	356.626	204.064	72.478	4402.894
-23.1	528.336	525.751	524.460	519.076	503.240	455.047	357.798	209.778	72.400	4402.894
-22.9	531.639	526.066	527.460	519.847	503.884	455.828	361.636	205.518	71.975	4402.894
-22.7	532.290	527.520	526.999	521.362	503.935	457.119	361.156	206.388	72.392	4402.894
-22.5	530.464	527.766	529.620	521.425	506.564	457.770	356.452	204.066	72.008	4402.894
-22.3	530.156	525.495	531.131	521.642	504.517	458.848	362.609	207.518	73.017	4402.894
-22.1	528.345	531.771	528.591	522.432	506.077	460.016	362.806	205.169	72.562	4402.894
-21.9	530.342	530.097	532.716	523.549	503.246	458.944	363.618	208.449	72.531	4402.894
-21.7	532.485	532.212	529.902	524.584	506.174	459.861	364.799	207.552	72.487	4402.894
-21.5	533.604	537.128	529.716	526.460	505.991	461.847	364.112	208.196	72.604	4402.894
-21.3	534.121	534.461	534.888	526.011	510.729	463.748	368.005	210.827	72.742	4402.894
-21.1	534.690	531.164	535.638	526.684	511.531	463.652	365.620	207.409	72.666	4402.894
-20.9	535.489	540.728	536.116	530.078	512.757	467.415	365.609	208.846	73.617	4402.894
-20.7	538.106	539.766	536.795	527.010	513.163	467.777	367.713	209.987	73.176	4402.894
-20.5	541.088	535.747	534.297	530.193	512.632	467.221	368.687	211.279	73.581	4402.894
-20.3	542.260	538.068	541.424	535.416	516.716	468.531	368.297	207.774	74.054	4402.894
-20.1	541.728	540.979	541.691	532.306	514.699	467.124	370.367	211.179	73.894	4402.894
-19.9	544.752	540.574	543.154	536.461	515.179	473.279	366.211	211.347	73.928	4402.894

Midpoint	Ratio: 1860	657	189	62	19	6.3	2.1	0.65	0.13	Volume
-19.7	545.646	544.241	543.050	538.871	518.995	471.276	371.672	211.061	74.556	4402.894
-19.5	546.737	551.864	547.959	541.571	522.659	471.038	373.157	215.264	73.977	4402.894
-19.3	550.729	551.122	545.232	537.959	522.799	476.245	374.695	211.157	74.814	4402.894
-19.1	552.169	555.945	547.086	545.327	526.087	478.448	377.445	213.998	75.555	4402.894
-18.9	553.758	555.895	554.684	545.644	527.244	480.108	375.784	214.563	75.637	4402.894
-18.7	545.258	545.645	544.652	536.888	516.645	468.395	368.430	213.535	74.280	4402.894
-18.5	34.268	34.455	34.126	33.425	32.868	29.934	22.571	12.889	4.489	12264.807
-18.3	28.875	28.949	29.007	28.613	27.816	24.462	19.421	11.264	3.874	186.603
-18.1	26.005	25.489	26.415	25.498	25.490	22.647	18.076	9.993	3.497	160.940
-17.9	23.913	23.561	25.460	23.761	23.080	21.159	15.687	9.366	3.208	145.255
-17.7	23.156	22.880	23.499	22.702	21.863	19.171	15.273	9.034	3.049	133.279
-17.5	21.407	21.866	21.747	21.008	19.890	18.110	14.481	8.337	2.859	123.465
-17.3	20.543	20.148	19.661	19.369	18.944	17.637	13.473	8.352	2.732	115.116
-17.1	19.645	19.271	19.142	18.289	18.313	15.873	12.734	7.517	2.543	107.844
-16.9	18.589	17.931	18.542	18.207	17.507	15.760	13.225	7.051	2.712	101.407
-16.7	17.402	17.480	17.762	17.214	16.959	15.672	11.998	8.005	2.439	95.641
-16.5	16.677	17.218	16.644	17.131	15.854	14.107	11.729	7.338	2.367	90.430
-16.3	16.400	16.549	16.463	16.313	15.671	14.508	10.974	6.362	2.177	85.685
-16.1	15.369	16.456	16.084	15.987	15.643	14.228	11.876	6.376	2.202	81.341
-15.9	15.300	14.165	15.049	15.064	15.123	13.893	10.089	6.377	2.068	77.345
-15.7	15.388	15.490	15.039	15.795	15.037	13.625	10.473	5.882	2.064	73.655
-15.5	15.998	16.169	16.181	16.155	14.906	14.269	11.173	5.778	1.920	70.235
-15.3	15.450	15.258	15.378	15.288	14.959	13.240	10.198	5.199	1.532	67.056
-15.1	14.946	15.153	14.886	14.638	14.355	13.071	10.032	5.188	1.519	64.095
-14.9	14.646	14.697	14.765	14.430	13.871	12.780	9.568	5.218	1.514	61.330
-14.7	14.400	14.475	14.269	14.119	13.495	12.192	9.338	5.220	1.490	58.744
-14.5	14.062	14.126	14.173	14.003	13.400	12.188	9.377	4.954	1.526	56.321
-14.3	13.838	13.593	13.856	13.561	13.128	11.815	9.043	4.948	1.394	54.048
-14.1	13.622	13.384	13.419	13.354	12.865	11.722	8.910	4.701	1.395	51.912
-13.9	13.286	13.366	13.343	13.150	12.698	11.557	8.814	4.990	1.393	49.904
-13.7	12.938	13.284	13.291	13.087	12.564	11.285	8.531	4.552	1.330	48.013
-13.5	13.035	13.146	13.042	12.683	12.450	11.425	8.799	4.530	1.337	46.231
-13.3	12.660	12.954	13.037	12.823	12.263	11.101	8.517	4.466	1.265	44.551
-13.1	12.704	13.034	12.803	12.759	12.262	11.107	8.567	4.624	1.306	42.966
-12.9	12.646	12.811	12.735	12.459	11.999	10.835	8.486	4.406	1.336	41.470
-12.7	12.786	12.500	12.608	12.585	12.174	11.060	8.407	4.497	1.251	40.057
-12.5	12.580	12.874	12.459	12.528	12.124	10.934	8.392	4.430	1.301	38.722
-12.3	12.358	12.603	12.553	12.374	11.800	10.885	8.220	4.571	1.300	37.461
-12.1	12.436	12.388	12.623	12.308	11.944	10.630	8.369	4.307	1.286	36.268
-11.9	12.269	12.211	12.123	12.065	11.745	10.691	8.328	4.366	1.258	35.141
-11.7	11.820	11.789	11.907	11.864	11.174	10.054	7.731	4.268	1.242	34.076
-11.5	10.984	11.225	11.055	11.013	10.648	9.611	7.482	3.996	1.122	33.068
-11.3	10.554	10.479	10.511	10.294	10.091	9.029	7.075	3.743	1.090	32.116
-11.1	9.759	9.775	9.771	9.545	9.415	8.316	6.516	3.424	1.080	31.217

Midpoint	Ratio: 1860	657	189	62	19	6.3	2.1	0.65	0.13	Volume
-10.9	9.003	8.896	8.831	9.005	8.533	7.886	5.962	3.287	0.999	30.367
-10.7	8.107	8.222	8.157	8.091	7.961	6.948	5.496	3.042	0.946	29.564
-10.5	7.420	7.375	7.307	7.378	6.985	6.463	4.839	2.574	0.845	28.806
-10.3	6.541	6.612	6.427	6.450	6.125	5.739	4.204	2.381	0.695	28.092
-10.1	5.804	5.696	5.788	5.569	5.488	4.938	3.930	2.097	0.624	27.418
-9.9	4.872	4.790	4.843	4.812	4.679	4.334	3.292	1.821	0.553	26.784
-9.7	4.082	4.104	4.155	3.995	4.036	3.637	2.671	1.527	0.480	26.188
-9.5	3.477	3.386	3.373	3.391	3.300	3.079	2.250	1.300	0.367	25.627
-9.3	2.875	2.843	2.779	2.743	2.666	2.400	1.927	1.104	0.304	25.101
-9.1	2.267	2.342	2.257	2.242	2.186	1.878	1.589	0.923	0.260	24.608
-8.9	1.854	1.843	1.751	1.814	1.668	1.636	1.215	0.709	0.202	24.147
-8.7	1.438	1.407	1.475	1.494	1.391	1.262	0.970	0.509	0.161	23.717
-8.5	1.186	1.174	1.178	1.100	1.120	1.003	0.759	0.484	0.148	23.317
-8.3	0.926	0.905	0.901	0.871	0.862	0.704	0.586	0.340	0.097	22.946
-8.1	0.675	0.747	0.692	0.670	0.666	0.621	0.476	0.245	0.077	22.603
-7.9	0.557	0.522	0.543	0.532	0.509	0.492	0.380	0.223	0.071	22.286
-7.7	0.421	0.452	0.429	0.407	0.379	0.345	0.288	0.160	0.044	21.996
-7.5	0.332	0.326	0.324	0.357	0.350	0.296	0.218	0.109	0.046	21.732
-7.3	0.276	0.278	0.283	0.261	0.260	0.258	0.197	0.109	0.033	21.493
-7.1	0.203	0.194	0.225	0.213	0.213	0.187	0.130	0.073	0.034	21.278
-6.9	0.182	0.147	0.184	0.165	0.157	0.143	0.121	0.072	0.013	21.087
-6.7	0.137	0.145	0.129	0.124	0.125	0.099	0.093	0.052	0.018	20.919
-6.5	0.104	0.122	0.111	0.108	0.102	0.101	0.110	0.049	0.018	20.775
-6.3	0.108	0.101	0.103	0.109	0.104	0.088	0.087	0.036	0.008	20.654
-6.1	0.080	0.089	0.087	0.092	0.099	0.076	0.053	0.031	0.011	20.554
-5.9	0.089	0.083	0.086	0.067	0.070	0.064	0.047	0.028	0.003	20.478
-5.7	0.076	0.079	0.066	0.076	0.067	0.039	0.061	0.021	0.010	20.423
-5.5	0.056	0.049	0.059	0.056	0.056	0.042	0.052	0.021	0.003	20.390
-5.3	0.044	0.044	0.044	0.047	0.038	0.056	0.030	0.016	0.005	20.379
-5.1	0.049	0.043	0.044	0.050	0.048	0.044	0.039	0.013	0.006	20.378
-4.9	0.045	0.039	0.029	0.043	0.049	0.049	0.040	0.028	0.003	20.378
-4.7	0.044	0.038	0.055	0.055	0.052	0.044	0.035	0.029	0.007	20.378
-4.5	0.028	0.037	0.042	0.052	0.047	0.060	0.043	0.013	0.006	20.378
-4.3	0.044	0.047	0.048	0.041	0.054	0.041	0.018	0.015	0.005	20.378
-4.1	0.054	0.054	0.054	0.055	0.056	0.045	0.039	0.015	0.009	20.378
-3.9	0.056	0.051	0.049	0.050	0.040	0.040	0.045	0.023	0.004	20.378
-3.7	0.054	0.084	0.062	0.057	0.057	0.053	0.031	0.039	0.005	20.378
-3.5	0.081	0.057	0.067	0.060	0.067	0.063	0.047	0.021	0.011	20.378
-3.3	0.068	0.043	0.073	0.065	0.069	0.065	0.036	0.039	0.011	20.378
-3.1	0.080	0.059	0.083	0.073	0.068	0.075	0.054	0.013	0.005	20.378
-2.9	0.079	0.058	0.062	0.067	0.090	0.075	0.059	0.018	0.009	20.378
-2.7	0.091	0.083	0.077	0.074	0.071	0.083	0.063	0.036	0.011	20.378
-2.5	0.085	0.084	0.073	0.085	0.082	0.066	0.075	0.034	0.013	20.378
-2.3	0.096	0.071	0.093	0.083	0.094	0.070	0.032	0.036	0.012	20.378

Midpoint	Ratio: 1860	657	189	62	19	6.3	2.1	0.65	0.13	Volume
-2.1	0.077	0.088	0.092	0.090	0.087	0.067	0.055	0.026	0.015	20.378
-1.9	0.088	0.077	0.096	0.093	0.079	0.085	0.058	0.031	0.012	20.378
-1.7	0.079	0.071	0.078	0.070	0.089	0.072	0.043	0.031	0.005	20.378
-1.5	0.074	0.080	0.081	0.082	0.075	0.067	0.067	0.018	0.006	20.378
-1.3	0.076	0.075	0.062	0.082	0.087	0.059	0.045	0.023	0.007	20.378
-1.1	0.081	0.056	0.075	0.071	0.066	0.061	0.053	0.026	0.010	20.378
-0.9	0.050	0.050	0.063	0.058	0.045	0.041	0.026	0.016	0.007	20.378
-0.7	0.044	0.035	0.051	0.037	0.043	0.030	0.030	0.021	0.002	20.378
-0.5	0.025	0.020	0.031	0.028	0.029	0.020	0.022	0.008	0.002	20.378
-0.1	0.014	0.017	0.020	0.023	0.016	0.013	0.014	0.008	0.001	20.378
0.1	0.013	0.015	0.019	0.011	0.011	0.008	0.008	0.005	0.003	20.378
0.5	0.008	0.006	0.010	0.008	0.006	0.006	0.004	0.003	0.002	20.378
0.7	0.008	0.000	0.008	0.003	0.005	0.004	0.002	0.000	0.000	20.378
0.9	0.006	0.004	0.000	0.003	0.004	0.001	0.000	0.005	0.001	20.378
1.1	0.003	0.000	0.001	0.004	0.002	0.002	0.002	0.003	0.000	20.378
1.3	0.001	0.000	0.001	0.001	0.001	0.000	0.000	0.000	0.001	20.378
1.5	0.001	0.000	0.002	0.000	0.001	0.002	0.000	0.000	0.000	20.378
1.7	0.001	0.000	0.003	0.000	0.001	0.001	0.002	0.003	0.000	20.378
1.9	0.001	0.000	0.000	0.000	0.002	0.000	0.000	0.000	0.000	20.378
2.1	0.001	0.000	0.001	0.000	0.002	0.002	0.002	0.000	0.000	20.378
2.3	0.000	0.002	0.000	0.000	0.001	0.000	0.000	0.003	0.000	20.378
2.5	0.000	0.000	0.000	0.000	0.000	0.001	0.000	0.000	0.000	20.378
2.7	0.001	0.000	0.000	0.000	0.000	0.000	0.000	0.000	0.000	20.378
2.9	0.000	0.000	0.000	0.001	0.003	0.000	0.000	0.000	0.000	20.378
3.1	0.001	0.000	0.000	0.001	0.002	0.001	0.000	0.000	0.000	20.378
3.3	0.000	0.000	0.001	0.001	0.001	0.001	0.002	0.000	0.000	20.378
3.5	0.000	0.000	0.000	0.001	0.001	0.000	0.000	0.000	0.000	20.378
3.7	0.003	0.000	0.000	0.001	0.005	0.000	0.000	0.000	0.000	20.378
3.9	0.002	0.002	0.003	0.001	0.001	0.002	0.002	0.000	0.001	20.378
4.1	0.005	0.000	0.000	0.003	0.002	0.001	0.002	0.000	0.000	20.378
4.3	0.002	0.000	0.003	0.001	0.004	0.006	0.002	0.003	0.000	20.378
4.5	0.004	0.004	0.006	0.002	0.003	0.005	0.002	0.003	0.000	20.378
4.7	0.007	0.013	0.003	0.006	0.003	0.009	0.004	0.003	0.000	20.378
4.9	0.008	0.006	0.010	0.010	0.014	0.010	0.006	0.005	0.001	20.378
5.1	0.023	0.007	0.022	0.014	0.018	0.014	0.010	0.010	0.006	20.378
5.3	0.039	0.025	0.032	0.031	0.026	0.028	0.018	0.011	0.004	20.379
5.5	0.048	0.065	0.046	0.053	0.059	0.058	0.034	0.021	0.007	20.390
5.7	0.103	0.081	0.071	0.106	0.095	0.080	0.071	0.018	0.011	20.423
5.9	0.111	0.159	0.122	0.136	0.115	0.121	0.079	0.055	0.011	20.478
6.1	0.207	0.223	0.190	0.194	0.190	0.158	0.144	0.077	0.029	20.554
6.3	0.279	0.218	0.309	0.302	0.291	0.252	0.199	0.103	0.043	20.654
6.5	0.448	0.439	0.429	0.408	0.384	0.342	0.258	0.187	0.053	20.775
6.7	0.600	0.621	0.580	0.601	0.561	0.536	0.391	0.207	0.072	20.919
6.9	0.840	0.874	0.821	0.824	0.811	0.710	0.560	0.264	0.114	21.087

Midpoint	Ratio: 1860	657	189	62	19	6.3	2.1	0.65	0.13	Volume
7.1	1.080	1.082	1.138	1.040	1.062	0.919	0.766	0.458	0.132	21.278
7.3	1.491	1.473	1.405	1.425	1.403	1.231	1.031	0.549	0.189	21.493
7.5	1.902	1.724	1.787	1.775	1.731	1.558	1.161	0.754	0.206	21.732
7.7	2.179	2.248	2.192	2.170	2.144	1.940	1.477	0.892	0.280	21.996
7.9	2.582	2.684	2.577	2.513	2.547	2.266	1.773	0.933	0.318	22.286
8.1	2.963	2.939	2.930	2.925	2.871	2.714	1.998	1.194	0.335	22.603
8.3	3.285	3.253	3.158	3.241	3.057	2.845	2.313	1.193	0.390	22.946
8.5	3.503	3.600	3.473	3.504	3.327	2.958	2.316	1.489	0.452	23.317
8.7	3.810	3.643	3.662	3.648	3.534	3.228	2.495	1.312	0.460	23.717
8.9	3.913	3.877	3.899	3.857	3.665	3.408	2.854	1.438	0.436	24.147
9.1	4.224	4.270	4.241	4.194	4.085	3.713	2.972	1.524	0.478	24.608
9.3	4.536	4.476	4.657	4.425	4.345	4.148	3.151	1.795	0.552	25.101
9.5	5.021	4.953	5.102	4.866	4.678	4.371	3.513	1.923	0.617	25.627
9.7	5.548	5.342	5.447	5.538	5.249	4.736	3.651	2.037	0.648	26.188
9.9	6.151	5.952	6.121	6.048	6.011	5.270	4.079	2.309	0.717	26.784
10.1	6.859	6.792	6.914	6.879	6.564	6.023	4.744	2.521	0.800	27.418
10.3	7.741	7.715	7.755	7.554	7.420	6.650	5.192	2.853	0.877	28.092
10.5	8.652	8.674	8.616	8.499	8.259	7.341	5.653	3.143	0.972	28.806
10.7	9.653	9.319	9.717	9.386	9.149	8.277	6.599	3.495	1.112	29.564
10.9	10.514	10.841	10.650	10.576	10.214	9.280	6.948	3.786	1.276	30.367
11.1	11.654	11.781	11.697	11.509	11.185	10.047	7.833	4.356	1.298	31.217
11.3	12.526	12.564	12.592	12.593	12.078	11.118	8.615	4.773	1.437	32.116
11.5	13.438	13.378	13.381	13.463	13.018	11.761	8.885	5.242	1.544	33.068
11.7	14.264	14.378	14.412	14.205	13.724	12.243	9.666	5.281	1.564	34.076
11.9	14.961	14.948	15.117	14.881	14.183	13.001	9.996	5.287	1.656	35.141
12.1	15.391	15.517	15.417	15.460	14.726	13.465	10.547	5.624	1.668	36.268
12.3	16.047	15.897	15.853	15.514	15.199	13.671	10.556	5.943	1.708	37.461
12.5	16.489	16.038	16.294	16.009	15.437	13.945	10.615	5.777	1.706	38.722
12.7	16.506	16.360	16.476	16.109	15.501	13.996	10.557	5.839	1.747	40.057
12.9	16.442	16.468	16.485	16.116	15.740	14.249	10.975	5.964	1.758	41.470
13.1	16.564	16.711	16.532	16.433	15.592	14.205	10.836	5.650	1.769	42.966
13.3	16.510	16.505	16.375	16.375	15.760	14.409	10.811	5.977	1.685	44.551
13.5	16.423	16.820	16.622	16.020	15.830	14.250	11.497	6.181	1.751	46.231
13.7	16.643	16.818	16.589	16.516	15.827	14.482	11.129	5.797	1.733	48.013
13.9	16.691	16.469	16.778	16.514	16.012	14.387	11.130	6.047	1.750	49.904
14.1	16.644	16.721	16.908	16.572	16.139	14.433	11.212	5.926	1.725	51.912
14.3	16.995	17.006	16.803	16.741	16.105	14.587	11.164	6.058	1.683	54.048
14.5	17.107	17.130	17.355	17.035	16.370	14.772	11.426	5.896	1.823	56.321
14.7	17.268	17.075	17.323	17.242	16.691	15.151	11.658	6.230	1.814	58.744
14.9	17.899	17.485	17.470	17.359	16.745	15.231	11.491	6.221	1.771	61.330
15.1	18.138	18.168	17.906	17.721	16.963	15.405	11.990	6.388	1.908	64.095
15.3	18.059	18.121	18.381	18.074	17.279	15.898	11.903	6.303	1.907	67.056
15.5	18.972	18.896	19.448	19.086	18.424	16.629	12.397	7.253	2.198	70.235
15.7	18.385	18.879	17.964	18.484	17.898	15.692	12.491	6.633	2.564	73.655

Midpoint	Ratio: 1860	657	189	62	19	6.3	2.1	0.65	0.13	Volume
15.9	18.123	17.895	18.060	17.669	17.081	16.426	12.495	6.722	2.505	77.345
16.1	18.707	19.007	18.051	19.065	17.218	16.534	11.538	7.195	2.433	81.341
16.3	18.421	18.788	18.620	18.383	18.097	16.904	12.641	7.194	2.496	85.685
16.5	19.139	18.552	19.783	19.431	18.994	16.508	13.309	7.394	2.685	90.430
16.7	20.859	19.692	20.207	19.334	19.651	16.612	14.667	7.555	2.844	95.641
16.9	21.416	21.511	20.986	20.943	20.099	17.993	15.129	8.486	2.772	101.407
17.1	21.852	20.971	21.648	20.751	21.252	18.892	14.956	8.594	3.126	107.844
17.3	23.395	22.436	23.221	22.263	20.937	19.041	16.052	9.001	3.084	115.116
17.5	23.676	22.304	22.330	23.729	22.434	19.884	16.783	8.780	3.174	123.465
17.7	25.772	24.799	25.472	25.489	23.804	21.445	17.764	9.389	3.412	133.279
17.9	26.216	26.952	28.770	26.160	24.882	22.869	18.659	10.538	3.668	145.255
18.1	29.295	28.224	28.521	28.563	26.632	24.966	20.288	10.878	3.996	160.940
18.3	33.107	30.306	32.303	30.876	30.283	27.706	21.809	12.034	4.396	186.603
18.5	36.562	37.765	36.623	36.673	34.481	30.883	24.068	14.791	4.898	12264.807
18.7	550.689	546.977	550.025	541.494	522.451	474.608	373.969	213.508	75.168	4402.894
18.9	561.988	560.241	560.713	548.692	533.693	482.200	383.823	218.304	76.117	4402.894
19.1	562.225	559.075	552.927	551.501	528.336	481.608	380.553	215.400	75.923	4402.894
19.3	557.317	555.977	553.061	545.563	528.510	478.452	374.206	214.838	75.271	4402.894
19.5	550.336	550.696	555.076	544.362	525.047	479.011	376.368	212.961	75.068	4402.894
19.7	552.757	548.548	552.754	540.731	524.043	478.106	378.004	214.592	75.196	4402.894
19.9	552.709	555.879	543.397	540.085	522.484	474.989	373.413	212.898	74.314	4402.894
20.1	544.800	538.181	547.848	541.118	520.667	474.879	371.844	213.519	74.582	4402.894
20.3	545.601	545.555	543.680	538.181	518.811	473.254	370.952	213.856	74.152	4402.894
20.5	547.469	546.967	542.401	534.588	518.757	470.408	370.299	212.765	74.137	4402.894
20.7	545.934	539.327	541.936	532.220	517.487	470.668	371.384	212.291	73.440	4402.894
20.9	543.658	540.619	539.382	533.186	513.583	465.404	372.281	210.362	73.159	4402.894
21.1	544.334	537.460	534.760	531.080	511.598	469.072	365.546	210.816	73.422	4402.894
21.3	536.224	540.535	532.592	533.396	511.783	463.124	367.371	209.980	72.977	4402.894
21.5	538.303	535.858	536.539	531.131	510.736	464.417	368.213	211.180	73.301	4402.894
21.7	538.220	529.758	535.700	527.054	510.892	462.767	363.825	210.478	72.905	4402.894
21.9	533.050	539.375	538.282	525.128	513.721	462.178	364.480	209.850	72.961	4402.894
22.1	533.446	535.195	532.022	525.362	508.943	463.896	363.669	208.477	73.637	4402.894
22.3	533.021	529.098	533.886	526.306	508.277	458.684	360.458	208.351	72.338	4402.894
22.5	533.783	533.528	529.948	523.588	508.115	459.919	363.703	208.150	72.923	4402.894
22.7	531.575	527.666	526.719	526.592	506.530	460.626	364.055	206.838	72.882	4402.894
22.9	531.829	531.571	526.889	523.267	506.510	459.046	359.293	209.428	72.678	4402.894
23.1	526.735	533.071	526.594	521.156	502.318	457.816	363.321	205.926	72.670	4402.894
23.3	528.728	526.250	526.806	521.709	501.942	459.138	362.429	207.631	72.615	4402.894
23.5	528.897	527.689	526.909	521.643	502.894	454.452	357.728	206.148	72.563	4402.894
23.7	524.188	525.381	523.409	519.569	502.422	456.969	359.938	205.429	72.582	4402.894
23.9	526.569	532.538	522.345	519.497	500.568	458.232	359.248	207.522	72.405	4402.894
24.1	527.488	523.669	526.281	519.801	499.942	455.697	362.696	208.215	72.438	4402.894
24.3	520.362	524.764	525.827	518.473	498.249	455.630	357.327	206.686	71.848	4402.894
24.5	523.136	523.197	523.858	516.211	497.982	453.718	361.019	206.576	71.497	4402.894

Midpoint	Ratio: 1860	657	189	62	19	6.3	2.1	0.65	0.13	Volume
24.7	522.922	524.289	522.852	518.275	502.218	453.298	360.371	205.893	72.328	4402.894
24.9	520.613	524.322	518.221	517.929	496.099	454.327	356.368	204.659	72.396	4402.894
25.1	525.936	520.980	526.494	514.938	493.583	454.990	357.633	204.665	71.848	4402.894
25.3	523.099	521.457	518.826	514.533	499.579	450.547	357.379	204.806	71.606	4402.894
25.5	522.688	521.270	523.869	511.396	496.661	452.107	353.390	202.857	71.195	4402.894
25.7	522.620	525.063	520.799	512.972	498.142	452.458	353.526	205.826	71.929	4402.894
25.9	525.424	522.258	520.667	513.067	494.369	452.870	351.661	202.813	71.555	4402.894
26.1	520.259	522.076	519.327	509.579	496.023	449.726	356.274	201.028	70.622	4402.894
26.3	521.616	521.485	519.055	511.540	493.555	448.339	353.473	204.711	71.929	4402.894
26.5	523.267	520.554	515.619	514.104	494.244	449.685	355.637	202.296	70.902	4402.894
26.7	518.861	516.652	515.382	512.237	491.890	447.142	354.264	202.460	71.339	4402.894
26.9	516.663	523.231	514.682	512.189	493.540	451.766	350.312	203.051	71.654	4402.894
27.1	520.642	516.328	517.344	511.730	490.892	449.928	357.138	203.907	71.326	4402.894
27.3	516.796	517.509	519.055	509.938	491.375	445.928	352.234	203.175	71.099	4402.894
27.5	519.845	511.488	515.200	509.244	489.949	445.475	353.747	202.577	71.110	4402.894
27.7	515.067	514.553	513.634	509.290	492.770	445.392	348.602	203.333	71.004	4402.894
27.9	516.196	519.474	514.647	508.740	492.432	447.821	351.557	203.818	70.753	4402.894
28.1	516.628	513.480	511.280	509.536	489.942	446.086	352.645	199.474	70.592	4402.894
28.3	517.068	509.721	512.435	508.923	489.351	444.286	351.899	201.295	69.953	4402.894
28.5	516.072	512.116	509.292	507.373	491.423	446.444	355.645	200.704	70.291	4402.894
28.7	510.495	508.922	508.519	504.603	487.371	444.235	348.027	198.407	71.355	4402.894
28.9	511.751	512.505	507.766	507.781	489.008	444.682	348.500	202.805	70.427	4402.894
29.1	510.717	512.510	510.354	505.865	491.284	443.918	348.435	200.998	70.577	4402.894
29.3	516.563	510.304	512.826	504.518	487.181	445.735	351.962	201.744	70.707	4402.894
29.5	512.752	514.077	509.430	504.080	487.488	444.727	348.494	203.577	70.559	4402.894
29.7	513.373	512.359	512.105	502.000	485.043	445.326	349.097	199.623	70.236	4402.894
29.9	512.211	512.670	508.983	505.854	485.652	443.665	348.125	201.368	70.000	4402.894
30.1	510.850	503.850	514.200	503.431	487.449	442.522	350.954	199.720	70.771	4402.894
30.3	508.722	512.441	508.244	501.567	485.893	441.873	349.767	200.254	69.958	4402.894
30.5	512.644	508.979	511.317	502.862	489.393	441.833	354.421	199.753	70.212	4402.894
30.7	507.686	508.477	508.745	502.125	486.915	444.427	348.665	198.372	69.651	4402.894
30.9	512.200	514.355	504.585	504.495	487.727	440.375	349.669	199.756	70.444	4402.894
31.1	511.322	512.897	504.000	500.914	488.575	440.050	347.133	200.254	70.101	4402.894
31.3	506.984	506.944	502.203	501.282	485.218	440.671	348.250	200.400	70.651	4402.894
31.5	508.079	509.786	509.052	501.595	483.638	440.310	348.024	198.537	70.086	4402.894
31.7	507.279	511.665	510.879	499.560	486.040	441.940	348.073	200.449	69.979	4402.894
31.9	510.845	506.425	505.643	501.828	485.849	441.100	351.167	199.112	70.209	4402.894
32.1	509.178	509.693	502.489	500.894	481.774	438.960	348.210	200.085	70.403	4402.894
32.3	510.059	505.694	505.896	500.013	483.371	438.628	349.224	201.166	70.259	4402.894
32.5	509.490	503.849	504.940	499.290	482.957	437.460	345.376	199.679	70.471	4402.894
32.7	506.817	507.287	503.388	496.569	483.469	441.646	349.579	198.318	70.035	4402.894
32.9	505.843	503.764	506.829	496.088	481.433	435.939	346.704	196.400	69.217	4402.894
33.1	505.932	505.085	504.165	498.968	483.424	438.226	346.099	199.122	70.103	4402.894
33.3	508.529	503.471	501.021	497.710	482.766	438.491	344.086	200.202	70.009	4402.894

Midpoint	Ratio: 1860	657	189	62	19	6.3	2.1	0.65	0.13	Volume
33.5	506.846	509.884	504.006	495.772	482.262	438.213	344.137	199.448	69.956	4402.894
33.7	506.362	502.006	501.063	497.907	479.016	437.415	344.311	198.803	69.674	4402.894
33.9	502.759	507.030	499.246	498.720	483.109	437.239	345.050	200.070	69.900	4402.894
34.1	504.900	504.191	504.581	497.504	481.441	438.484	346.709	198.469	69.216	4402.894
34.3	509.130	504.657	502.421	495.147	481.262	437.695	343.830	197.017	69.121	4402.894
34.5	507.437	505.393	499.235	498.238	478.957	434.387	344.745	198.086	68.948	4402.894
34.7	502.500	502.058	502.562	496.004	478.286	435.705	344.945	196.149	68.477	4402.894
34.9	507.569	502.065	506.224	496.576	480.868	439.827	343.930	198.130	69.159	4402.894
35.1	501.926	502.371	503.460	493.236	477.683	437.304	344.336	199.419	69.005	4402.894
35.3	504.912	503.628	500.122	493.488	478.813	435.626	346.353	200.677	68.930	4402.894
35.5	500.561	505.276	500.869	493.415	477.509	436.395	345.153	197.586	69.118	4402.894
35.7	502.910	503.834	501.249	494.932	479.188	437.238	340.912	196.394	69.826	4402.894
35.9	504.061	501.968	500.112	495.060	481.164	435.235	345.122	196.492	69.715	4402.894
36.1	503.135	498.958	504.226	494.942	477.287	434.876	343.946	197.127	69.802	4402.894
36.3	499.156	501.145	498.775	493.609	479.953	435.009	341.129	197.162	69.357	4402.894
36.5	499.304	504.752	500.071	497.813	476.283	432.930	342.932	197.591	69.497	4402.894
36.7	503.753	499.423	496.947	495.984	475.771	435.639	342.442	195.384	69.121	4402.894
36.9	499.336	503.805	494.415	492.823	476.502	436.113	340.944	197.772	68.933	4402.894
37.1	499.669	498.883	500.424	493.321	479.949	434.522	341.029	198.382	68.842	4402.894
37.3	496.026	499.629	499.404	492.187	474.070	431.684	342.247	197.065	69.527	4402.894
37.5	501.233	499.987	498.630	493.783	476.186	434.488	342.973	195.484	68.797	4402.894
37.7	500.662	499.620	502.526	490.668	475.160	433.669	344.556	197.090	69.159	4402.894
37.9	505.299	497.411	500.154	491.667	475.760	432.262	343.301	195.544	69.376	4402.894
38.1	500.214	504.583	495.621	494.227	475.721	431.696	341.620	195.846	69.352	4402.894
38.3	498.520	498.342	499.301	493.069	477.564	435.336	340.217	196.055	69.438	4402.894
38.5	499.295	497.361	497.038	493.808	475.608	432.753	343.302	197.989	68.638	4402.894
38.7	500.294	500.970	498.987	494.016	474.589	431.713	344.167	198.187	69.269	4402.894
38.9	500.564	497.309	495.547	491.754	474.249	432.055	340.869	196.050	69.282	4402.894
39.1	499.680	501.935	497.419	491.673	476.888	433.334	341.280	197.073	69.202	4402.894
39.3	501.518	500.781	497.032	492.817	476.978	431.718	342.961	195.915	68.789	4402.894
39.5	501.003	502.764	492.094	494.843	477.721	430.888	342.258	199.062	68.873	4402.894
39.7	493.625	494.033	496.065	490.634	476.457	434.556	340.709	198.064	68.988	4402.894
39.9	497.251	499.531	496.931	490.100	476.068	433.450	341.146	195.016	68.956	4402.894
40.1	500.016	496.647	490.883	492.099	475.970	431.074	339.868	194.036	68.989	4402.894
40.3	500.130	492.767	497.427	492.265	475.451	432.793	343.118	197.904	69.126	4402.894
40.5	497.980	502.533	498.384	492.512	475.971	433.564	342.287	195.632	69.249	4402.894
40.7	498.002	496.214	500.322	492.458	472.849	431.820	342.747	195.988	69.129	4402.894
40.9	499.095	496.680	496.421	488.665	475.531	431.360	344.093	195.611	69.067	4402.894
41.1	495.000	502.834	496.202	491.154	474.166	434.024	341.610	194.294	68.901	4402.894
41.3	496.201	498.850	496.469	490.184	475.131	433.038	339.164	194.680	69.008	4402.894
41.5	495.286	499.397	495.434	491.489	476.518	430.052	339.136	195.476	69.019	4402.894
41.7	498.368	491.165	495.587	488.350	474.293	429.712	339.615	195.748	68.956	4402.894
41.9	495.290	496.831	497.330	490.746	475.273	432.720	341.455	194.412	68.367	4402.894
42.1	497.070	495.770	494.335	490.252	474.040	435.193	343.512	196.771	68.863	4402.894

Midpoint	Ratio: 1860	657	189	62	19	6.3	2.1	0.65	0.13	Volume
42.3	499.332	502.637	501.694	492.195	474.808	434.835	339.737	194.670	68.465	4402.894
42.5	496.534	503.604	492.347	488.742	472.929	430.689	342.328	196.837	69.218	4402.894
42.7	498.368	497.772	499.005	490.949	472.912	433.395	345.454	195.112	68.922	4402.894
42.9	497.671	495.150	499.515	492.274	472.580	430.416	338.113	196.205	69.532	4402.894
43.1	495.209	495.762	497.761	490.818	475.959	430.755	340.399	195.951	68.253	4402.894
43.3	495.756	497.789	493.448	492.474	472.019	430.171	340.425	192.457	68.532	4402.894
43.5	496.341	499.559	495.064	489.256	473.623	432.772	340.954	194.170	68.946	4402.894
43.7	495.879	495.734	493.278	488.049	474.718	429.837	336.553	196.866	68.528	4402.894
43.9	497.074	498.915	494.510	489.506	472.825	430.020	339.894	193.798	68.909	4402.894
44.1	500.931	493.032	496.840	487.836	475.683	429.087	341.555	195.634	68.398	4402.894
44.3	493.345	496.633	491.514	492.286	475.172	428.936	341.000	194.573	69.075	4402.894
44.5	495.713	496.698	494.985	489.259	474.287	427.743	341.152	195.050	68.284	4402.894
44.7	496.525	496.791	495.941	487.985	468.231	428.687	341.255	195.412	68.355	4402.894
44.9	496.194	497.042	496.302	489.428	474.862	428.849	340.875	195.120	67.863	4402.894
45.1	496.510	493.895	496.563	490.828	471.098	430.180	340.350	193.170	68.326	4402.894
45.3	498.806	497.464	496.574	487.547	472.315	429.283	341.699	194.016	68.173	4402.894
45.5	493.294	495.160	492.998	487.544	474.133	430.678	335.482	192.895	68.611	4402.894
45.7	496.612	497.960	492.632	488.411	476.610	430.423	342.769	194.075	68.089	4402.894
45.9	491.090	494.911	492.117	488.875	470.927	431.742	340.463	195.687	68.695	4402.894
46.1	498.606	500.811	492.525	487.577	472.857	431.373	337.560	194.817	68.542	4402.894
46.3	494.808	492.992	494.289	488.860	470.616	427.117	339.618	196.081	68.034	4402.894
46.5	493.972	496.732	495.919	487.619	472.763	428.950	336.495	196.928	68.945	4402.894
46.7	494.433	496.678	495.033	489.824	473.805	429.670	340.755	195.271	68.676	4402.894
46.9	498.085	495.617	493.883	488.603	472.594	431.153	338.057	195.281	68.487	4402.894
47.1	494.564	499.223	487.490	487.162	470.516	427.676	339.937	195.227	68.346	4402.894
47.3	494.251	496.998	499.926	486.807	472.762	431.213	341.442	194.370	68.593	4402.894
47.5	495.466	498.216	492.881	486.814	469.985	431.826	340.126	194.242	68.312	4402.894
47.7	490.447	496.700	495.931	486.753	471.370	429.687	339.538	195.682	68.309	4402.894
47.9	497.894	499.108	491.210	486.861	471.727	426.406	339.624	195.074	68.142	4402.894
48.1	493.256	495.097	496.134	486.026	473.275	429.471	339.282	193.513	68.240	4402.894
48.3	495.964	495.272	494.103	489.978	475.449	428.142	337.190	194.285	68.208	4402.894
48.5	493.023	493.886	492.159	487.106	471.414	430.268	339.920	195.124	68.134	4402.894
48.7	498.549	493.391	492.119	485.789	474.596	429.191	340.490	194.503	68.072	4402.894
48.9	494.097	497.021	491.200	486.320	472.414	428.207	340.230	194.549	68.535	4402.894
49.1	496.038	494.593	493.401	487.424	473.012	427.599	337.305	194.219	68.975	4402.894
49.3	497.414	493.280	494.720	484.696	470.097	425.580	338.709	195.675	68.312	4402.894
49.5	498.662	496.595	491.954	485.162	471.120	423.911	341.868	195.692	69.247	4402.894
49.7	493.217	497.734	491.784	488.249	471.280	429.975	337.774	194.652	68.713	4402.894
49.9	493.080	493.992	493.212	487.839	471.208	428.150	338.692	194.191	68.922	4402.894
50.1	493.455	491.708	493.027	488.018	471.666	430.524	339.986	195.840	68.493	4402.894
50.3	494.227	496.089	492.930	484.848	470.773	429.958	340.782	194.097	68.024	4402.894
50.5	493.250	494.432	491.277	488.332	470.381	428.582	338.129	193.070	68.836	4402.894
50.7	494.483	498.454	490.835	488.549	473.803	428.862	339.266	196.196	68.276	4402.894
50.9	493.957	494.159	491.485	485.644	469.799	431.986	340.811	194.069	68.896	4402.894

Midpoint	Ratio: 1860	657	189	62	19	6.3	2.1	0.65	0.13	Volume
51.1	497.514	490.250	495.223	486.778	473.042	429.227	339.813	193.986	68.909	4402.894
51.3	496.046	491.953	492.356	488.509	471.816	427.420	338.301	195.209	68.344	4402.894
51.5	497.900	490.705	492.877	489.254	471.966	428.699	336.474	195.754	68.403	4402.894
51.7	498.121	490.440	491.650	485.922	469.478	429.659	336.337	194.348	68.362	4402.894
51.9	494.787	489.806	494.022	488.395	469.409	427.239	339.445	193.915	68.409	4402.894
52.1	493.722	491.687	494.799	491.599	472.373	431.197	338.274	196.339	69.229	4402.894
52.3	495.834	492.033	491.723	487.257	472.254	431.451	337.129	195.690	68.729	4402.894
52.5	496.962	490.789	490.014	484.396	469.190	430.138	336.701	195.108	68.567	4402.894
52.7	495.094	494.329	495.516	487.894	470.181	430.489	337.908	196.393	68.322	4402.894
52.9	494.658	494.395	490.564	488.557	470.357	427.160	338.826	195.542	68.474	4402.894
53.1	497.661	492.836	491.621	486.962	471.163	425.688	338.507	195.617	68.485	4402.894
53.3	493.194	493.147	492.416	486.066	473.981	427.455	340.905	195.006	68.836	4402.894
53.5	496.025	489.868	493.545	486.647	472.755	431.348	338.567	195.001	68.220	4402.894
53.7	496.344	496.743	493.345	485.186	474.147	427.172	337.549	193.615	68.983	4402.894
53.9	495.184	496.449	494.011	484.712	471.782	427.919	338.754	192.028	68.323	4402.894
54.1	493.296	493.880	490.333	487.060	470.744	429.976	337.692	194.188	68.581	4402.894
54.3	494.578	491.770	493.211	486.596	472.897	426.522	336.865	194.682	68.003	4402.894
54.5	493.044	498.663	494.910	487.152	469.845	427.828	336.654	193.967	68.620	4402.894
54.7	495.320	489.595	490.035	485.696	471.066	428.611	336.913	194.154	68.389	4402.894
54.9	492.438	492.286	490.465	488.001	471.975	428.644	338.554	195.187	68.421	4402.894
55.1	494.774	492.530	493.488	487.902	469.438	428.470	338.742	191.428	68.542	4402.894
55.3	495.039	495.504	495.265	487.893	473.214	426.932	339.731	193.144	68.030	4402.894
55.5	494.216	491.079	492.187	486.910	466.775	427.996	336.397	196.541	68.614	4402.894
55.7	491.967	492.829	490.553	486.764	474.248	428.605	336.340	195.723	68.393	4402.894
55.9	493.546	491.071	491.211	487.358	468.583	428.426	338.133	193.630	68.192	4402.894
56.1	492.496	491.810	490.904	488.560	470.299	429.080	336.311	195.303	67.743	4402.894
56.3	492.916	495.365	492.243	482.747	468.935	426.346	337.731	193.250	67.995	4402.894
56.5	493.706	490.593	490.021	485.420	469.340	428.408	335.014	194.385	68.167	4402.894
56.7	496.821	491.422	493.735	485.963	470.678	426.019	338.110	196.057	68.068	4402.894
56.9	493.163	493.155	492.433	486.467	472.201	425.970	337.149	194.975	68.919	4402.894
57.1	493.361	495.537	495.175	487.153	470.165	424.321	338.256	198.181	68.220	4402.894
57.3	495.148	491.797	492.443	487.082	470.751	428.114	338.508	193.781	68.871	4402.894
57.5	498.636	491.073	493.426	486.582	469.085	424.941	335.599	192.210	67.939	4402.894
57.7	492.734	493.890	495.624	484.014	473.406	427.856	335.954	194.149	68.537	4402.894
57.9	494.377	493.344	490.598	487.140	470.020	427.971	336.387	192.130	68.044	4402.894
58.1	492.885	491.270	492.321	485.033	468.950	429.178	339.035	192.875	68.155	4402.894
58.3	493.880	492.933	494.255	486.142	472.172	428.436	332.379	194.858	68.729	4402.894
58.5	494.659	492.957	493.312	486.037	473.578	426.806	336.228	195.052	68.067	4402.894
58.7	493.342	492.013	490.063	487.158	472.102	427.282	336.528	193.831	68.301	4402.894
58.9	494.324	494.280	495.264	486.133	468.079	423.265	333.895	194.854	67.988	4402.894
59.1	494.624	495.270	494.303	486.817	471.564	430.910	339.473	193.128	68.390	4402.894
59.3	496.064	494.837	488.749	490.748	470.509	427.563	339.260	194.645	68.306	4402.894
59.5	491.843	492.035	492.806	485.594	468.694	430.039	338.766	194.058	67.547	4402.894
59.7	494.482	494.542	490.397	485.420	469.203	430.064	336.697	193.353	68.650	4402.894

Midpoint	Ratio: 1860	657	189	62	19	6.3	2.1	0.65	0.13	Volume
59.9	491.618	493.120	490.995	484.659	471.202	428.641	335.144	193.685	68.426	4402.894
60.1	492.677	490.769	491.186	487.970	466.254	427.518	339.072	192.608	67.622	4402.894
60.3	495.722	490.917	490.753	490.078	470.741	426.209	341.805	192.730	68.200	4402.894
60.5	492.547	496.906	487.768	486.294	468.751	427.272	337.180	194.513	68.282	4402.894
60.7	492.708	491.651	492.982	485.530	468.580	431.039	337.459	196.079	68.639	4402.894
60.9	492.712	494.197	487.372	488.604	469.587	426.865	336.169	193.234	68.454	4402.894
61.1	492.780	493.402	494.593	487.227	471.790	431.640	336.952	192.768	68.342	4402.894
61.3	493.492	492.914	495.281	486.481	470.568	431.694	334.136	195.168	68.294	4402.894
61.5	497.243	495.590	489.033	488.359	469.563	427.140	337.537	192.687	68.410	4402.894
61.7	492.290	491.086	493.574	485.309	470.335	430.065	339.303	194.926	68.066	4402.894
61.9	493.862	496.816	491.602	485.938	470.559	427.560	338.585	193.165	68.104	4402.894
62.1	493.292	491.622	492.626	485.942	471.343	427.940	338.816	194.185	68.031	4402.894
62.3	491.210	491.342	494.443	484.746	472.582	426.689	337.051	193.001	68.849	4402.894
62.5	493.278	492.831	494.645	487.794	469.832	427.220	335.897	193.934	68.383	4402.894
62.7	491.807	495.057	489.498	485.534	469.416	425.860	335.582	193.148	68.869	4402.894
62.9	491.499	492.542	491.561	486.706	471.985	425.812	335.740	194.627	67.604	4402.894
63.1	492.399	492.011	491.326	485.218	472.023	429.188	336.833	194.135	68.524	4402.894
63.3	490.623	492.893	493.431	486.470	467.933	428.078	338.657	194.419	68.293	4402.894
63.5	496.808	494.912	493.787	485.513	469.072	428.183	338.153	194.187	68.074	4402.894
63.7	493.335	495.789	489.458	484.772	470.775	428.026	338.255	195.705	67.755	4402.894
63.9	493.006	493.435	488.559	483.843	467.806	427.039	336.779	194.495	68.522	4402.894
64.1	493.098	492.219	492.465	484.492	469.181	427.360	336.944	192.850	67.715	4402.894
64.3	494.027	491.633	494.125	487.217	469.639	430.381	338.474	193.380	68.656	4402.894
64.5	494.248	493.771	492.003	484.439	471.907	426.342	339.190	195.451	67.737	4402.894
64.7	494.644	490.222	493.161	488.617	469.516	426.593	338.116	192.412	68.257	4402.894
64.9	494.542	494.825	492.314	486.035	469.745	430.188	335.899	195.608	68.069	4402.894
65.1	493.234	498.860	490.210	487.225	470.310	429.500	336.916	193.855	68.244	4402.894
65.3	494.756	491.169	494.536	483.552	467.739	427.974	340.312	194.354	67.942	4402.894
65.5	490.618	490.592	489.869	488.228	469.680	426.221	340.131	193.773	68.178	4402.894
65.7	492.815	490.681	494.454	482.875	467.442	425.281	337.657	194.641	67.932	4402.894
65.9	491.664	498.160	493.893	489.400	470.350	425.850	338.816	193.493	67.945	4402.894
66.1	491.562	494.681	488.612	487.243	470.130	426.915	338.020	194.785	67.815	4402.894
66.3	492.487	491.512	491.735	486.723	467.109	426.928	336.721	193.916	68.190	4402.894
66.5	496.629	496.188	487.353	485.692	471.789	427.343	336.728	192.103	67.698	4402.894
66.7	490.767	490.954	493.400	483.158	471.996	429.146	340.943	194.540	67.780	4402.894
66.9	492.095	493.698	495.396	484.656	468.783	426.394	338.957	196.158	68.346	4402.894
67.1	492.164	494.739	490.594	485.177	468.083	429.027	336.248	194.697	68.446	4402.894
67.3	490.027	496.672	492.582	485.416	469.006	428.716	339.707	193.099	68.472	4402.894
67.5	495.205	497.919	489.872	487.102	471.567	427.150	336.524	194.212	68.174	4402.894
67.7	495.271	494.577	489.142	484.392	471.414	428.851	337.079	194.995	67.548	4402.894
67.9	492.806	492.781	491.286	485.523	470.208	427.621	336.410	194.201	68.073	4402.894
68.1	490.110	492.713	492.824	486.236	472.711	426.006	334.561	195.665	67.987	4402.894
68.3	496.342	495.227	489.712	485.255	466.868	430.686	336.270	194.945	67.970	4402.894
68.5	491.007	490.976	493.840	487.536	468.244	430.350	338.885	190.432	68.145	4402.894

Midpoint	Ratio: 1860	657	189	62	19	6.3	2.1	0.65	0.13	Volume
68.7	494.778	497.169	493.782	484.851	472.609	427.241	332.662	195.879	67.755	4402.894
68.9	496.109	487.040	493.879	484.660	469.901	426.277	336.335	193.215	68.412	4402.894
69.1	493.755	492.042	491.092	488.469	471.736	429.585	338.915	193.480	69.062	4402.894
69.3	494.533	495.693	490.018	487.968	470.968	425.279	338.767	195.165	68.188	4402.894
69.5	494.287	496.495	493.057	486.968	469.469	429.350	333.168	193.229	68.085	4402.894
69.7	491.659	492.893	493.303	487.911	468.664	428.272	339.123	193.737	68.224	4402.894
69.9	496.010	493.260	494.405	487.135	468.988	425.541	337.523	194.885	68.405	4402.894
70.1	492.559	494.283	491.988	484.189	468.002	427.084	339.291	193.455	68.788	4402.894
70.3	497.412	495.363	492.805	485.040	469.376	429.341	337.128	193.587	68.063	4402.894
70.5	494.269	491.806	490.948	486.230	469.552	430.903	338.650	195.112	68.336	4402.894
70.7	493.693	493.669	490.560	486.923	471.362	429.313	340.336	192.416	68.151	4402.894
70.9	492.009	497.116	497.256	487.120	469.333	425.070	340.712	194.024	68.383	4402.894
71.1	493.989	496.620	490.688	487.947	470.745	426.060	338.968	192.654	68.783	4402.894
71.3	497.840	495.102	493.682	484.749	470.653	428.888	336.282	193.137	68.210	4402.894
71.5	494.117	493.202	493.987	488.330	469.135	430.171	338.156	194.057	68.267	4402.894
71.7	492.366	497.587	492.927	488.903	471.732	426.363	335.364	193.224	68.141	4402.894
71.9	494.909	488.120	494.494	486.278	468.850	428.197	336.528	193.479	67.953	4402.894
72.1	496.774	493.370	491.218	487.714	473.371	428.556	337.838	194.691	68.762	4402.894
72.3	492.085	492.152	492.678	487.865	469.506	428.887	336.795	193.517	68.697	4402.894
72.5	492.772	492.672	495.312	485.529	470.982	427.027	338.324	193.273	67.919	4402.894
72.7	495.073	493.344	489.204	488.294	471.538	427.841	336.026	192.732	68.521	4402.894
72.9	492.569	488.795	489.898	486.229	470.297	427.255	339.177	192.613	68.496	4402.894
73.1	493.891	495.692	495.175	487.528	470.294	429.381	342.541	193.536	67.871	4402.894
73.3	493.233	495.397	490.706	484.950	471.982	429.553	341.642	195.684	68.502	4402.894
73.5	496.749	493.725	490.894	486.833	469.121	424.328	338.943	194.529	68.038	4402.894
73.7	488.889	493.399	494.072	483.732	469.523	428.296	336.894	193.597	67.710	4402.894
73.9	491.690	496.582	493.832	487.941	467.370	428.821	338.666	192.790	68.088	4402.894
74.1	495.783	490.170	490.686	488.014	468.157	428.988	337.481	194.566	68.187	4402.894
74.3	495.767	491.030	490.080	485.080	470.007	425.997	340.518	194.245	68.571	4402.894
74.5	491.983	494.987	493.434	488.748	469.507	427.319	341.625	193.084	68.557	4402.894
74.7	494.829	494.423	489.293	484.426	469.063	426.897	337.237	193.347	68.451	4402.894
74.9	496.362	493.829	492.363	488.389	468.119	430.360	335.851	193.773	67.689	4402.894
75.1	492.711	493.354	493.164	487.201	469.379	425.367	339.813	193.694	68.393	4402.894
75.3	492.977	493.997	493.904	485.834	474.141	426.469	336.398	196.046	68.442	4402.894
75.5	493.591	498.394	491.696	487.670	468.280	425.360	335.498	193.287	68.457	4402.894
75.7	497.710	491.618	491.812	485.427	469.392	427.257	337.806	191.055	68.458	4402.894
75.9	494.438	491.391	491.210	485.662	471.839	428.277	335.996	193.254	68.545	4402.894
76.1	497.392	494.053	493.098	486.224	469.198	427.879	338.615	194.256	68.165	4402.894
76.3	494.275	495.570	490.378	487.483	472.977	428.752	336.879	193.023	67.937	4402.894
76.5	495.359	492.798	491.516	485.822	470.685	427.845	340.146	194.426	68.509	4402.894
76.7	490.071	492.643	491.138	487.232	469.230	428.742	336.708	193.225	68.375	4402.894
76.9	496.229	493.736	495.135	487.206	470.413	426.435	341.495	194.137	68.046	4402.894
77.1	494.285	493.075	494.594	488.604	469.695	426.843	338.662	195.832	67.883	4402.894
77.3	490.849	494.779	493.174	488.131	470.940	428.433	340.012	195.100	68.523	4402.894

Midpoint	Ratio: 1860	657	189	62	19	6.3	2.1	0.65	0.13	Volume
77.5	492.648	494.835	495.271	487.205	470.803	426.035	341.871	192.801	68.169	4402.894
77.7	495.395	496.918	497.650	484.676	469.423	426.562	341.752	195.597	67.593	4402.894
77.9	495.515	490.730	491.202	487.098	473.720	425.557	338.885	192.568	68.565	4402.894
78.1	498.128	489.632	490.485	487.469	471.316	432.044	339.746	195.859	68.243	4402.894
78.3	494.242	492.823	494.961	485.483	472.220	428.973	336.106	194.461	68.356	4402.894
78.5	495.188	496.703	492.890	487.542	470.966	425.846	336.796	192.665	68.062	4402.894
78.7	495.666	496.838	494.348	488.697	470.406	427.491	339.389	193.811	68.370	4402.894
78.9	496.597	493.003	494.801	486.661	470.650	425.997	335.549	195.088	67.979	4402.894
79.1	497.515	494.378	492.165	486.121	471.262	432.420	339.237	193.496	68.288	4402.894
79.3	494.662	495.311	490.221	488.010	473.349	428.042	339.223	194.923	67.992	4402.894
79.5	497.293	492.745	490.442	485.558	472.488	428.380	337.538	195.607	68.091	4402.894
79.7	494.531	499.826	488.783	485.032	470.346	428.860	340.414	192.581	68.749	4402.894
79.9	490.544	491.922	494.050	488.049	472.808	429.049	338.970	193.712	67.886	4402.894
80.1	497.453	490.784	494.594	488.735	472.004	431.950	340.020	194.805	67.627	4402.894
80.3	493.759	487.997	491.617	489.449	471.043	428.930	331.953	192.965	68.254	4402.894
80.5	493.654	496.655	493.446	487.572	471.798	428.696	336.065	192.812	68.289	4402.894
80.7	493.475	499.501	497.105	487.104	470.512	426.833	336.181	194.058	68.758	4402.894
80.9	495.641	494.379	494.091	489.212	472.113	428.202	336.043	194.996	68.634	4402.894
81.1	497.291	492.349	495.226	488.329	472.235	429.268	342.063	195.037	68.146	4402.894
81.3	494.756	494.541	490.871	490.070	470.942	428.529	336.868	191.884	68.249	4402.894
81.5	495.772	498.711	491.154	488.084	474.471	430.110	336.214	196.420	68.222	4402.894
81.7	496.274	492.054	493.920	488.049	472.416	427.148	339.118	195.490	68.302	4402.894
81.9	495.233	499.647	493.238	488.761	472.457	429.810	339.591	195.502	68.557	4402.894
82.1	493.892	496.721	493.505	488.669	471.950	428.276	338.495	196.192	68.963	4402.894
82.3	497.943	495.205	491.858	488.131	472.666	430.663	338.927	194.457	67.953	4402.894
82.5	494.859	489.402	496.064	489.576	471.499	429.363	335.811	192.649	68.117	4402.894
82.7	496.914	491.217	493.048	488.019	470.331	429.554	336.210	194.452	68.346	4402.894
82.9	494.667	495.763	491.642	487.230	474.495	431.014	341.797	194.407	68.626	4402.894
83.1	493.360	499.334	494.545	488.036	473.100	431.186	340.630	193.718	68.787	4402.894
83.3	494.010	494.549	492.808	489.292	473.123	429.949	340.138	193.425	68.015	4402.894
83.5	497.077	500.216	490.481	489.176	470.604	430.285	338.081	196.371	68.009	4402.894
83.7	493.988	495.752	492.788	485.077	472.981	430.759	343.562	194.332	68.529	4402.894
83.9	498.167	494.550	491.066	488.176	472.287	428.192	338.231	194.105	68.082	4402.894
84.1	493.342	494.944	496.395	488.102	472.626	425.477	338.580	194.760	68.663	4402.894
84.3	494.310	497.190	491.279	489.159	471.939	430.086	336.929	193.707	67.908	4402.894
84.5	493.532	494.137	492.251	489.046	471.262	426.840	339.160	194.137	68.217	4402.894
84.7	493.114	495.754	494.504	487.663	473.423	426.796	337.498	194.196	68.543	4402.894
84.9	497.560	495.630	494.796	489.116	471.506	426.711	341.886	193.895	68.613	4402.894
85.1	496.610	492.726	491.204	489.067	472.585	427.794	338.425	194.254	68.326	4402.894
85.3	499.720	497.849	492.157	490.249	471.994	429.142	339.572	195.622	67.709	4402.894
85.5	496.126	494.362	492.187	487.994	472.262	428.771	338.417	193.138	67.826	4402.894
85.7	494.505	499.301	494.775	488.661	476.471	434.457	338.971	193.351	67.911	4402.894
85.9	498.070	497.819	496.301	486.913	473.903	426.342	339.280	193.466	68.538	4402.894
86.1	497.623	496.575	495.958	487.384	473.744	430.733	340.320	194.181	68.600	4402.894

Midpoint	Ratio: 1860	657	189	62	19	6.3	2.1	0.65	0.13	Volume
86.3	494.128	494.210	495.420	488.397	472.103	430.322	340.110	194.781	67.779	4402.894
86.5	502.569	492.652	493.713	489.022	473.215	432.671	339.641	194.523	68.091	4402.894
86.7	498.878	493.844	496.528	487.848	472.853	428.927	337.239	195.148	68.604	4402.894
86.9	493.807	494.837	496.572	491.356	471.650	429.193	340.719	194.576	68.332	4402.894
87.1	495.614	498.249	496.609	488.677	472.115	431.723	341.069	192.784	68.356	4402.894
87.3	497.371	495.441	494.519	490.069	475.168	428.134	341.685	195.273	68.174	4402.894
87.5	492.632	496.973	494.513	489.422	472.766	427.705	336.720	194.198	68.422	4402.894
87.7	496.784	497.002	495.420	489.847	470.605	430.564	339.979	196.122	68.050	4402.894
87.9	498.199	489.064	494.249	487.701	473.283	429.308	339.674	195.113	67.569	4402.894
88.1	493.240	495.473	499.398	490.808	476.239	431.762	335.801	191.378	67.969	4402.894
88.3	492.813	495.378	495.357	489.997	471.797	428.388	340.243	194.647	68.322	4402.894
88.5	497.953	498.413	495.779	487.960	476.513	431.238	337.798	193.608	68.381	4402.894
88.7	495.546	498.816	495.727	488.903	474.012	429.597	340.646	192.764	68.369	4402.894
88.9	497.816	501.710	496.532	489.103	473.619	431.105	344.246	193.901	68.667	4402.894
89.1	496.783	496.614	495.770	490.051	471.960	431.002	336.324	194.032	68.869	4402.894
89.3	498.754	494.607	494.311	489.149	475.833	432.735	339.696	196.906	68.790	4402.894
89.5	495.765	500.870	493.906	490.554	474.982	430.678	335.911	196.455	68.209	4402.894
89.7	498.449	499.315	495.583	489.509	477.281	433.077	339.308	193.326	68.588	4402.894
89.9	499.125	499.437	493.130	489.843	475.451	430.738	339.592	193.976	68.397	4402.894
90.1	492.271	501.082	497.975	490.856	474.620	432.330	343.141	195.003	68.309	4402.894
90.3	494.426	495.289	496.354	488.751	473.619	428.890	341.710	194.549	68.565	4402.894
90.5	496.591	498.375	495.285	489.428	476.987	431.642	339.276	195.568	68.651	4402.894
90.7	502.951	496.486	494.044	488.679	474.693	432.110	341.083	196.077	68.550	4402.894
90.9	501.136	493.887	495.721	489.013	472.535	431.019	340.259	196.387	68.514	4402.894
91.1	499.808	498.232	495.811	490.224	474.798	429.395	336.817	194.319	68.589	4402.894
91.3	494.171	500.771	496.478	490.742	476.121	430.092	337.724	194.021	68.500	4402.894
91.5	497.527	499.506	495.019	492.481	476.491	433.354	340.063	193.880	68.165	4402.894
91.7	501.489	499.538	494.289	494.055	472.875	429.758	339.857	196.508	68.867	4402.894
91.9	495.367	499.299	497.895	490.355	473.486	430.208	337.017	197.048	69.077	4402.894
92.1	494.522	499.629	494.667	490.919	473.607	430.076	340.184	193.217	68.325	4402.894
92.3	500.046	498.380	494.060	489.615	475.417	431.927	338.803	195.944	68.141	4402.894
92.5	497.373	496.908	496.844	490.080	475.900	430.760	340.311	194.934	68.233	4402.894
92.7	498.683	497.554	496.972	489.912	473.346	430.622	336.776	195.288	67.993	4402.894
92.9	503.968	502.155	496.118	490.299	474.385	430.708	340.189	193.766	68.550	4402.894
93.1	496.814	495.909	500.187	493.854	472.439	430.727	340.558	195.212	67.932	4402.894
93.3	504.793	499.035	494.950	487.330	474.722	427.786	339.909	194.655	68.309	4402.894
93.5	497.330	498.723	498.503	487.625	476.356	432.152	338.279	192.677	67.683	4402.894
93.7	497.469	499.675	492.092	488.244	472.958	432.756	343.270	194.323	68.214	4402.894
93.9	498.799	495.952	498.527	490.485	474.171	429.841	339.932	194.691	68.390	4402.894
94.1	498.761	499.529	500.063	490.376	473.258	429.902	337.121	195.137	67.396	4402.894
94.3	502.462	495.970	495.943	488.562	472.642	429.142	341.280	195.636	68.309	4402.894
94.5	497.739	499.928	496.924	487.848	470.825	432.469	341.959	195.390	68.106	4402.894
94.7	496.234	498.084	492.069	488.721	469.961	427.477	341.322	195.144	68.191	4402.894
94.9	499.724	496.823	496.656	488.343	474.489	432.206	339.073	193.193	67.941	4402.894

Midpoint	Ratio: 1860	657	189	62	19	6.3	2.1	0.65	0.13	Volume
95.1	494.640	496.691	493.395	486.770	473.841	428.851	343.019	194.640	67.950	4402.894
95.3	491.698	496.006	493.970	487.419	469.034	428.506	336.178	194.446	67.536	4402.894
95.5	497.916	498.300	497.432	489.853	473.208	428.812	338.072	192.484	67.858	4402.894
95.7	494.261	492.433	493.163	490.227	471.197	429.693	336.971	195.101	67.966	4402.894
95.9	496.414	498.151	492.948	487.158	468.256	431.163	335.120	193.606	68.220	4402.894
96.1	496.833	495.461	495.487	487.806	471.905	429.644	343.011	193.908	67.893	4402.894
96.3	497.745	491.543	494.986	485.241	470.378	428.486	340.312	194.579	68.187	4402.894
96.5	497.673	491.908	493.738	489.685	470.671	426.039	343.412	191.956	67.801	4402.894
96.7	494.828	495.603	494.919	488.563	470.366	427.712	336.340	194.086	68.282	4402.894
96.9	495.552	494.473	492.544	490.367	473.609	427.580	336.015	194.537	67.202	4402.894
97.1	495.682	496.103	492.367	488.297	471.226	431.258	335.240	193.816	67.754	4402.894
97.3	494.239	493.569	494.615	487.710	470.495	427.670	338.823	195.852	67.152	4402.894
97.5	493.420	499.893	494.591	487.697	471.381	427.446	340.955	194.466	67.563	4402.894
97.7	499.026	496.436	495.608	487.627	471.209	431.716	339.462	193.741	67.598	4402.894
97.9	492.146	496.393	494.941	489.698	471.567	433.836	336.817	193.870	67.798	4402.894
98.1	494.518	493.391	494.135	489.386	471.069	429.167	338.098	192.528	67.622	4402.894
98.3	499.581	499.184	492.405	490.024	472.864	431.035	338.160	192.435	67.941	4402.894
98.5	496.890	499.801	493.384	490.004	474.960	430.540	338.993	194.547	67.776	4402.894
98.7	499.316	491.094	496.079	488.185	474.072	429.913	337.535	195.474	67.644	4402.894
98.9	501.122	499.800	497.196	488.510	471.320	428.752	340.129	193.081	67.224	4402.894
99.1	499.303	499.381	493.743	490.159	473.938	429.053	341.110	193.977	67.498	4402.894
99.3	27.357	27.641	27.159	215.197	25.329	23.415	17.751	10.635	3.732	4402.894
99.5	0.946	0.965	0.965	1.213	0.929	0.846	0.680	0.356	0.142	4402.894
99.7	0.000	0.000	0.000	0.152	0.000	0.000	0.000	0.000	0.000	4402.894

E.5. NavAb channel: Sodium and Potassium

Table of Na^+ population data for simulation with K^+ and Na^+ across bulk density ratios (#JJF-GRS12F1)

	Population in histogram bin at K^+/Na^+ ratio:					
Midpoint	62	19	6.3	2.1	0.65	Volume
-100.1	7.395	18.486	47.863	113.815	215.048	4510.005
-99.9	7.731	25.616	66.763	157.625	297.220	4510.005
-99.7	8.312	24.271	70.133	156.187	295.079	4510.005
-99.5	7.359	25.231	65.054	155.149	296.461	4510.005
-99.3	7.268	24.869	66.031	156.091	293.992	4510.005
-99.1	8.040	24.397	67.398	154.258	296.746	4510.005
-98.9	7.622	25.420	64.810	154.772	293.899	4510.005
-98.7	7.804	25.372	68.741	153.273	292.590	4510.005
-98.5	7.831	24.845	64.175	152.988	290.399	4510.005
-98.3	7.295	24.444	67.398	154.924	293.011	4510.005
-98.1	7.894	24.223	65.127	152.886	289.567	4510.005
-97.9	7.559	24.160	66.495	152.359	290.621	4510.005
-97.7	7.568	24.562	65.249	151.189	287.061	4510.005

Midpoint	Ratio: 62	19	6.3	2.1	0.65	Volume
-97.5	7.613	24.381	64.151	154.245	287.906	4510.005
-97.3	7.540	24.042	66.153	152.375	290.001	4510.005
-97.1	7.205	24.538	65.738	151.702	288.977	4510.005
-96.9	7.214	23.515	64.712	151.030	284.456	4510.005
-96.7	7.686	23.122	66.446	151.046	285.812	4510.005
-96.5	7.586	24.326	66.715	149.664	286.398	4510.005
-96.3	7.413	23.610	67.350	150.887	285.719	4510.005
-96.1	7.459	24.271	67.227	149.966	283.313	4510.005
-95.9	7.658	23.602	64.517	150.052	285.805	4510.005
-95.7	7.332	24.546	64.639	149.700	283.564	4510.005
-95.5	7.459	23.759	62.807	147.954	283.462	4510.005
-95.3	7.758	23.232	64.028	149.240	281.526	4510.005
-95.1	7.495	23.995	66.593	149.289	280.943	4510.005
-94.9	7.386	23.027	63.198	148.941	281.838	4510.005
-94.7	7.395	23.924	63.784	148.385	279.372	4510.005
-94.5	7.649	23.877	66.666	148.948	281.201	4510.005
-94.3	6.651	24.334	65.225	147.977	279.776	4510.005
-94.1	7.187	23.798	65.249	148.010	281.877	4510.005
-93.9	7.867	24.208	64.541	149.853	282.573	4510.005
-93.7	7.432	23.822	64.175	147.858	285.248	4510.005
-93.5	7.468	23.240	63.760	146.843	283.369	4510.005
-93.3	7.060	23.311	62.514	150.781	278.135	4510.005
-93.1	7.386	23.570	60.952	148.342	279.614	4510.005
-92.9	7.804	23.704	63.980	149.366	280.664	4510.005
-92.7	7.559	23.019	61.977	148.938	283.568	4510.005
-92.5	7.305	23.924	64.248	149.061	281.725	4510.005
-92.3	7.540	23.806	64.175	149.200	280.678	4510.005
-92.1	7.168	23.948	65.787	148.660	281.181	4510.005
-91.9	7.822	24.428	66.812	149.541	283.379	4510.005
-91.7	7.323	23.783	66.055	148.885	288.039	4510.005
-91.5	7.649	23.720	65.249	150.138	281.728	4510.005
-91.3	7.386	24.578	64.566	150.423	287.386	4510.005
-91.1	7.931	23.673	62.588	148.739	285.407	4510.005
-90.9	7.168	22.948	64.908	149.508	286.962	4510.005
-90.7	7.649	23.122	66.153	150.950	288.195	4510.005
-90.5	7.450	23.712	66.470	150.317	287.847	4510.005
-90.3	7.332	24.334	66.763	150.430	288.768	4510.005
-90.1	7.395	24.153	65.787	151.914	288.201	4510.005
-89.9	7.395	23.137	65.274	150.224	289.491	4510.005
-89.7	7.658	24.554	64.663	152.014	288.427	4510.005
-89.5	7.295	24.869	65.616	151.958	290.545	4510.005
-89.3	7.640	23.610	65.909	151.517	288.868	4510.005
-89.1	7.459	22.712	66.031	152.829	285.513	4510.005
-88.9	7.604	24.806	68.302	149.926	290.853	4510.005
-88.7	7.577	23.909	65.884	152.909	292.338	4510.005

Midpoint	Ratio: 62	19	6.3	2.1	0.65	Volume
-88.5	7.940	24.326	68.839	150.917	291.701	4510.005
-88.3	7.568	24.641	65.982	153.138	291.754	4510.005
-88.1	7.813	25.309	68.204	153.204	294.983	4510.005
-87.9	7.958	24.971	66.788	153.655	292.795	4510.005
-87.7	7.668	24.947	65.103	154.351	292.692	4510.005
-87.5	8.003	25.073	67.789	152.829	292.991	4510.005
-87.3	7.885	24.483	66.324	154.639	297.028	4510.005
-87.1	7.477	24.247	65.860	153.943	293.813	4510.005
-86.9	7.740	24.877	66.470	154.354	293.438	4510.005
-86.7	7.695	25.042	67.520	155.995	292.232	4510.005
-86.5	7.595	24.609	66.763	153.665	292.513	4510.005
-86.3	7.758	24.766	69.279	155.166	297.880	4510.005
-86.1	7.531	24.365	64.981	154.546	294.018	4510.005
-85.9	7.940	24.310	66.031	154.019	296.070	4510.005
-85.7	7.894	25.207	67.936	154.785	294.605	4510.005
-85.5	8.285	25.357	64.517	153.197	290.266	4510.005
-85.3	7.386	23.861	68.375	155.968	291.851	4510.005
-85.1	7.459	25.254	67.936	155.103	296.232	4510.005
-84.9	7.323	24.664	66.983	154.175	294.409	4510.005
-84.7	7.686	24.664	66.470	155.113	291.274	4510.005
-84.5	8.130	24.467	67.252	156.452	296.362	4510.005
-84.3	7.649	25.396	69.401	153.413	294.568	4510.005
-84.1	7.931	25.160	66.324	154.682	293.673	4510.005
-83.9	7.831	24.688	67.569	156.893	294.999	4510.005
-83.7	7.613	24.900	65.567	155.613	296.693	4510.005
-83.5	7.922	25.018	69.034	155.527	293.571	4510.005
-83.3	7.894	24.751	67.423	156.730	296.776	4510.005
-83.1	7.477	24.601	67.325	157.291	293.190	4510.005
-82.9	7.795	25.514	67.008	155.932	295.414	4510.005
-82.7	7.795	25.136	67.545	156.717	293.657	4510.005
-82.5	7.386	23.578	67.227	155.663	298.407	4510.005
-82.3	7.831	25.065	66.544	153.545	299.908	4510.005
-82.1	7.622	24.987	68.180	156.800	302.331	4510.005
-81.9	7.658	25.081	67.301	158.374	301.479	4510.005
-81.7	8.167	24.318	67.056	156.462	296.756	4510.005
-81.5	7.858	25.042	66.226	156.485	296.299	4510.005
-81.3	7.477	24.160	67.130	157.344	297.455	4510.005
-81.1	7.704	24.955	69.132	156.717	295.599	4510.005
-80.9	7.096	24.617	67.472	156.522	294.098	4510.005
-80.7	8.085	24.806	68.058	157.244	298.241	4510.005
-80.5	7.250	24.853	67.887	155.345	293.852	4510.005
-80.3	7.976	24.955	68.375	154.954	300.326	4510.005
-80.1	7.241	25.042	67.447	157.218	295.712	4510.005
-79.9	7.867	25.160	69.059	157.566	297.084	4510.005
-79.7	7.450	25.089	69.645	155.246	297.349	4510.005

Midpoint	Ratio: 62	19	6.3	2.1	0.65	Volume
-79.5	8.221	24.648	67.691	157.135	303.266	4510.005
-79.3	7.740	25.121	66.177	155.262	297.422	4510.005
-79.1	7.649	24.806	65.762	158.547	299.434	4510.005
-78.9	8.021	25.010	66.739	156.561	298.178	4510.005
-78.7	7.631	24.987	69.572	157.224	298.499	4510.005
-78.5	7.849	25.168	68.277	158.371	297.001	4510.005
-78.3	7.795	25.404	69.132	155.093	299.328	4510.005
-78.1	7.695	24.837	66.202	155.345	302.977	4510.005
-77.9	7.232	24.995	69.498	155.932	297.873	4510.005
-77.7	7.840	25.144	69.816	155.431	298.135	4510.005
-77.5	8.167	25.286	67.374	156.518	300.773	4510.005
-77.3	7.876	25.239	70.939	155.729	298.234	4510.005
-77.1	7.740	24.806	64.492	156.150	300.886	4510.005
-76.9	7.922	24.861	70.109	158.000	299.577	4510.005
-76.7	6.842	25.695	67.691	157.864	300.041	4510.005
-76.5	8.094	24.782	66.422	156.462	296.226	4510.005
-76.3	8.112	25.191	69.230	157.755	299.818	4510.005
-76.1	8.130	24.971	67.984	155.816	301.986	4510.005
-75.9	7.423	24.884	64.639	159.064	298.894	4510.005
-75.7	7.876	24.759	68.302	156.933	300.163	4510.005
-75.5	7.804	25.333	68.668	155.756	298.963	4510.005
-75.3	7.985	25.184	68.522	156.190	297.671	4510.005
-75.1	7.522	25.506	67.374	155.978	298.864	4510.005
-74.9	7.994	25.325	66.763	158.845	298.523	4510.005
-74.7	8.103	24.971	69.816	156.767	300.501	4510.005
-74.5	7.776	25.050	68.693	158.828	298.400	4510.005
-74.3	7.513	25.687	70.353	157.771	299.152	4510.005
-74.1	7.622	25.081	68.302	155.663	298.960	4510.005
-73.9	7.949	24.530	66.983	157.542	301.439	4510.005
-73.7	8.067	24.766	66.666	159.041	302.390	4510.005
-73.5	7.450	25.302	69.425	158.451	300.929	4510.005
-73.3	8.121	24.467	68.717	158.146	299.411	4510.005
-73.1	7.704	25.254	67.643	158.152	300.160	4510.005
-72.9	7.776	25.286	69.718	156.034	302.629	4510.005
-72.7	7.595	25.034	70.573	158.268	300.624	4510.005
-72.5	8.076	25.191	68.546	158.600	297.140	4510.005
-72.3	7.758	25.640	66.690	157.811	299.640	4510.005
-72.1	7.495	25.671	65.689	157.682	296.143	4510.005
-71.9	8.112	25.136	66.886	158.905	302.291	4510.005
-71.7	8.321	25.010	68.766	159.869	301.492	4510.005
-71.5	8.158	24.113	66.397	156.518	301.360	4510.005
-71.3	8.257	26.222	68.424	157.340	299.398	4510.005
-71.1	7.767	24.499	67.056	158.958	301.532	4510.005
-70.9	7.795	25.365	67.545	156.452	298.778	4510.005
-70.7	7.368	25.483	69.328	155.981	296.779	4510.005

Midpoint	Ratio: 62	19	6.3	2.1	0.65	Volume
-70.5	8.357	25.742	68.375	158.407	302.298	4510.005
-70.3	8.167	25.546	66.080	158.023	301.655	4510.005
-70.1	8.003	24.719	67.569	157.665	299.023	4510.005
-69.9	8.312	25.286	65.323	156.462	303.855	4510.005
-69.7	7.740	25.443	68.375	159.266	299.348	4510.005
-69.5	7.658	25.601	68.326	158.063	303.156	4510.005
-69.3	7.867	25.262	67.862	157.145	305.463	4510.005
-69.1	7.922	25.829	70.842	155.703	303.425	4510.005
-68.9	7.586	24.688	67.618	158.109	305.970	4510.005
-68.7	7.649	25.357	69.987	159.077	298.764	4510.005
-68.5	7.758	25.467	65.616	158.500	301.161	4510.005
-68.3	7.785	25.585	67.496	156.150	303.643	4510.005
-68.1	8.167	24.845	68.277	158.994	299.142	4510.005
-67.9	8.003	25.223	71.232	158.944	298.310	4510.005
-67.7	7.704	24.664	66.886	158.050	299.020	4510.005
-67.5	7.804	25.522	65.616	156.926	304.624	4510.005
-67.3	7.649	25.514	66.299	158.209	299.759	4510.005
-67.1	8.366	24.751	69.987	157.228	297.634	4510.005
-66.9	8.421	25.349	68.741	156.956	298.867	4510.005
-66.7	7.704	24.822	69.010	157.476	302.520	4510.005
-66.5	7.286	24.121	67.472	158.888	304.465	4510.005
-66.3	8.185	25.443	68.204	156.740	303.259	4510.005
-66.1	8.012	26.380	67.350	159.004	302.235	4510.005
-65.9	8.348	25.058	66.861	158.835	300.839	4510.005
-65.7	7.958	24.168	68.204	157.609	299.679	4510.005
-65.5	7.958	24.924	67.716	158.298	300.889	4510.005
-65.3	8.003	26.128	68.961	158.586	305.993	4510.005
-65.1	7.513	24.625	67.716	159.206	305.625	4510.005
-64.9	7.531	25.341	71.306	158.490	304.005	4510.005
-64.7	8.012	25.081	70.842	156.472	303.163	4510.005
-64.5	7.967	24.145	66.788	156.303	300.206	4510.005
-64.3	8.303	25.065	68.375	158.242	299.596	4510.005
-64.1	7.531	24.861	68.619	158.593	301.158	4510.005
-63.9	7.894	25.616	66.910	157.188	302.278	4510.005
-63.7	7.550	25.121	67.301	157.900	300.137	4510.005
-63.5	8.239	24.947	67.813	157.261	300.123	4510.005
-63.3	7.677	25.333	68.277	158.845	304.419	4510.005
-63.1	7.486	24.822	65.201	159.501	300.866	4510.005
-62.9	7.586	25.396	69.474	158.315	298.831	4510.005
-62.7	7.985	25.010	68.229	158.059	302.036	4510.005
-62.5	7.450	24.208	68.619	158.543	296.955	4510.005
-62.3	8.312	25.294	66.495	158.318	299.069	4510.005
-62.1	8.112	23.814	65.518	159.127	299.517	4510.005
-61.9	7.985	25.868	66.422	158.411	298.599	4510.005
-61.7	8.103	24.656	68.253	160.214	299.444	4510.005

Midpoint	Ratio: 62	19	6.3	2.1	0.65	Volume
-61.5	7.876	24.869	65.738	158.232	300.760	4510.005
-61.3	8.067	24.719	68.009	158.132	300.326	4510.005
-61.1	8.457	25.435	66.324	158.649	304.356	4510.005
-60.9	8.030	25.734	67.227	160.446	301.313	4510.005
-60.7	7.740	24.483	69.523	158.345	302.775	4510.005
-60.5	7.686	24.837	69.083	159.249	303.514	4510.005
-60.3	7.631	25.955	66.031	157.344	300.929	4510.005
-60.1	7.804	24.940	69.816	157.748	303.431	4510.005
-59.9	7.976	24.436	69.498	157.930	298.257	4510.005
-59.7	7.713	25.341	69.083	160.373	300.863	4510.005
-59.5	7.432	24.955	65.396	157.397	300.995	4510.005
-59.3	7.785	24.617	67.569	156.889	299.116	4510.005
-59.1	7.785	24.452	68.644	157.731	303.186	4510.005
-58.9	8.040	26.089	70.402	156.999	302.371	4510.005
-58.7	7.758	25.105	68.375	158.968	300.113	4510.005
-58.5	7.785	25.097	67.838	157.529	305.151	4510.005
-58.3	7.522	25.073	68.424	157.907	301.350	4510.005
-58.1	7.586	25.010	65.762	158.069	300.435	4510.005
-57.9	8.103	25.750	67.813	158.948	302.042	4510.005
-57.7	7.668	25.616	66.739	157.367	301.774	4510.005
-57.5	7.413	25.632	67.423	158.209	299.199	4510.005
-57.3	8.230	24.578	66.446	158.941	298.864	4510.005
-57.1	7.640	25.427	67.032	156.962	301.419	4510.005
-56.9	7.876	25.105	67.252	161.394	302.195	4510.005
-56.7	7.931	25.506	69.328	157.808	295.493	4510.005
-56.5	8.076	25.144	71.037	159.511	299.553	4510.005
-56.3	7.441	25.302	64.395	157.479	295.609	4510.005
-56.1	7.740	25.089	67.765	159.650	299.573	4510.005
-55.9	7.468	25.852	67.740	159.800	301.857	4510.005
-55.7	8.285	24.011	68.595	159.210	300.014	4510.005
-55.5	7.695	24.554	70.964	157.552	301.091	4510.005
-55.3	8.076	25.727	68.424	158.437	300.640	4510.005
-55.1	7.795	25.160	69.010	156.870	301.615	4510.005
-54.9	8.040	25.058	68.668	159.074	298.085	4510.005
-54.7	7.776	24.467	67.203	158.696	301.525	4510.005
-54.5	8.058	25.687	66.470	158.865	304.972	4510.005
-54.3	8.121	24.412	67.081	157.798	301.565	4510.005
-54.1	7.513	24.719	66.055	156.528	300.909	4510.005
-53.9	7.513	25.286	69.059	159.690	300.581	4510.005
-53.7	8.049	25.302	69.914	157.387	302.904	4510.005
-53.5	8.321	24.916	66.886	159.747	301.834	4510.005
-53.3	7.695	25.215	69.474	160.834	301.648	4510.005
-53.1	8.248	24.987	68.961	158.361	300.455	4510.005
-52.9	7.822	25.624	66.080	158.291	300.402	4510.005
-52.7	7.876	24.680	67.447	161.112	302.102	4510.005

Midpoint	Ratio: 62	19	6.3	2.1	0.65	Volume
-52.5	8.176	25.199	67.691	158.103	302.656	4510.005
-52.3	7.767	25.065	70.231	157.307	303.448	4510.005
-52.1	7.295	25.223	69.230	160.025	303.895	4510.005
-51.9	8.575	25.105	69.132	158.219	304.174	4510.005
-51.7	7.894	25.538	66.348	158.881	299.053	4510.005
-51.5	8.566	25.160	68.717	158.772	302.546	4510.005
-51.3	7.386	25.286	69.498	155.981	298.764	4510.005
-51.1	7.423	24.341	67.643	158.195	300.568	4510.005
-50.9	8.185	25.601	69.865	158.636	298.357	4510.005
-50.7	7.795	25.577	67.643	158.076	304.495	4510.005
-50.5	8.021	25.239	67.838	157.963	299.842	4510.005
-50.3	7.885	25.239	70.549	159.130	301.353	4510.005
-50.1	8.421	25.018	68.937	160.307	305.741	4510.005
-49.9	7.404	26.285	69.328	156.508	303.193	4510.005
-49.7	7.749	24.822	68.790	158.391	302.162	4510.005
-49.5	7.903	25.467	68.351	158.106	305.314	4510.005
-49.3	7.813	25.199	69.547	158.305	304.767	4510.005
-49.1	7.550	24.782	63.931	155.723	303.126	4510.005
-48.9	7.677	25.593	67.643	158.872	299.984	4510.005
-48.7	7.804	25.278	68.986	158.295	303.759	4510.005
-48.5	7.441	25.632	68.864	157.257	300.730	4510.005
-48.3	7.595	24.546	67.789	158.527	301.098	4510.005
-48.1	7.813	25.365	71.843	159.110	300.551	4510.005
-47.9	7.858	25.813	67.545	157.281	300.916	4510.005
-47.7	7.913	25.152	69.523	160.393	304.005	4510.005
-47.5	7.876	24.892	69.694	158.885	305.357	4510.005
-47.3	8.030	25.191	71.379	158.514	299.974	4510.005
-47.1	8.421	25.892	68.790	156.707	300.094	4510.005
-46.9	7.858	25.805	67.081	159.206	302.268	4510.005
-46.7	7.486	24.656	71.306	157.536	303.468	4510.005
-46.5	8.321	24.940	69.157	159.011	303.935	4510.005
-46.3	7.413	25.695	68.229	158.537	302.864	4510.005
-46.1	7.931	25.042	68.351	154.145	300.912	4510.005
-45.9	8.403	25.160	65.909	157.615	299.517	4510.005
-45.7	7.559	25.892	69.718	159.670	300.816	4510.005
-45.5	8.003	25.522	68.741	156.571	295.347	4510.005
-45.3	7.277	25.372	67.618	161.235	304.880	4510.005
-45.1	7.668	25.081	66.886	157.012	302.709	4510.005
-44.9	8.067	25.546	70.304	158.341	302.387	4510.005
-44.7	7.604	26.049	67.789	156.740	302.752	4510.005
-44.5	7.223	25.498	67.130	156.419	302.980	4510.005
-44.3	7.858	26.002	66.593	157.416	301.065	4510.005
-44.1	7.922	25.616	68.522	159.816	301.088	4510.005
-43.9	7.550	25.727	69.498	156.260	303.362	4510.005
-43.7	7.949	25.128	68.326	157.791	303.186	4510.005

Midpoint	Ratio: 62	19	6.3	2.1	0.65	Volume
-43.5	7.522	25.286	67.227	159.322	303.252	4510.005
-43.3	7.468	25.561	67.716	159.428	302.941	4510.005
-43.1	7.931	25.341	69.889	158.765	303.302	4510.005
-42.9	8.230	25.593	70.085	158.951	297.087	4510.005
-42.7	8.357	25.262	66.641	158.159	302.052	4510.005
-42.5	7.668	25.105	68.717	159.117	299.364	4510.005
-42.3	7.822	25.609	69.547	156.691	301.399	4510.005
-42.1	7.967	25.435	70.353	158.179	301.217	4510.005
-41.9	7.913	25.105	69.034	157.791	299.673	4510.005
-41.7	8.266	25.506	70.671	158.351	301.880	4510.005
-41.5	7.831	25.372	69.108	159.392	302.364	4510.005
-41.3	7.323	26.309	67.643	160.784	299.633	4510.005
-41.1	7.713	25.254	69.962	157.440	302.231	4510.005
-40.9	7.677	25.443	69.914	157.728	299.500	4510.005
-40.7	7.903	25.302	71.403	156.823	296.700	4510.005
-40.5	7.522	24.774	68.937	159.263	299.398	4510.005
-40.3	7.931	25.553	69.401	158.050	299.895	4510.005
-40.1	7.640	24.483	70.158	157.353	301.515	4510.005
-39.9	7.849	25.341	66.715	157.516	305.367	4510.005
-39.7	7.913	25.365	65.274	158.255	299.040	4510.005
-39.5	8.257	25.325	66.788	157.039	303.766	4510.005
-39.3	7.722	25.530	67.130	158.321	301.482	4510.005
-39.1	7.885	25.420	69.596	158.119	301.572	4510.005
-38.9	8.103	25.435	68.668	159.226	302.457	4510.005
-38.7	7.522	25.144	70.671	159.531	304.943	4510.005
-38.5	7.849	25.640	68.351	158.600	302.904	4510.005
-38.3	7.686	25.215	69.132	156.230	301.453	4510.005
-38.1	8.221	24.719	67.398	159.703	305.668	4510.005
-37.9	8.130	24.814	69.743	157.847	301.154	4510.005
-37.7	7.867	26.081	67.838	156.190	302.722	4510.005
-37.5	7.849	25.459	69.059	161.318	300.700	4510.005
-37.3	7.495	25.561	71.183	157.934	298.426	4510.005
-37.1	8.185	26.065	69.059	160.409	301.757	4510.005
-36.9	7.840	25.254	67.276	156.694	299.974	4510.005
-36.7	7.713	25.443	69.474	158.815	299.192	4510.005
-36.5	7.640	25.128	69.572	159.369	297.754	4510.005
-36.3	7.595	25.915	69.157	158.268	298.788	4510.005
-36.1	8.049	25.278	68.546	157.857	296.862	4510.005
-35.9	8.239	26.207	66.031	158.288	302.835	4510.005
-35.7	7.767	24.877	69.474	159.763	301.330	4510.005
-35.5	7.804	25.734	71.916	158.265	300.229	4510.005
-35.3	7.758	25.490	67.130	157.526	297.886	4510.005
-35.1	8.094	25.302	69.791	156.210	300.452	4510.005
-34.9	8.285	25.435	70.060	158.881	301.290	4510.005
-34.7	8.221	24.562	69.425	157.612	300.684	4510.005

Midpoint	Ratio: 62	19	6.3	2.1	0.65	Volume
-34.5	7.404	25.073	68.058	157.546	298.380	4510.005
-34.3	8.430	25.742	68.302	157.967	303.541	4510.005
-34.1	7.522	25.459	68.009	159.849	302.954	4510.005
-33.9	7.413	24.522	68.033	156.979	301.224	4510.005
-33.7	7.686	25.601	67.496	159.279	302.901	4510.005
-33.5	7.867	25.018	68.986	159.876	301.824	4510.005
-33.3	7.604	25.286	66.959	158.398	299.361	4510.005
-33.1	7.785	24.963	66.177	157.622	302.440	4510.005
-32.9	7.640	25.420	66.983	157.711	304.701	4510.005
-32.7	8.384	24.688	69.010	157.363	305.009	4510.005
-32.5	8.575	25.404	66.422	159.273	300.226	4510.005
-32.3	7.686	25.333	68.351	158.898	305.446	4510.005
-32.1	7.740	25.687	65.689	157.403	301.320	4510.005
-31.9	7.776	25.089	67.887	157.871	302.182	4510.005
-31.7	7.994	25.435	69.840	156.760	300.226	4510.005
-31.5	7.522	24.735	70.378	159.014	301.549	4510.005
-31.3	7.559	25.435	65.103	158.795	299.792	4510.005
-31.1	7.631	24.782	67.960	156.797	300.014	4510.005
-30.9	7.486	24.822	66.739	160.002	299.978	4510.005
-30.7	7.885	26.348	67.838	159.007	298.834	4510.005
-30.5	8.003	25.215	67.936	157.804	301.045	4510.005
-30.3	7.849	26.057	68.522	158.971	298.224	4510.005
-30.1	7.668	25.010	69.034	158.066	301.980	4510.005
-29.9	8.003	25.892	67.472	157.380	300.631	4510.005
-29.7	7.994	24.940	65.762	158.467	296.385	4510.005
-29.5	8.221	25.475	65.005	157.943	298.102	4510.005
-29.3	7.305	25.459	68.473	158.272	303.660	4510.005
-29.1	7.831	25.514	70.671	157.281	296.540	4510.005
-28.9	7.840	24.271	69.328	158.606	303.136	4510.005
-28.7	8.085	24.515	70.988	157.880	300.296	4510.005
-28.5	7.595	25.585	65.835	157.009	303.710	4510.005
-28.3	7.913	24.444	67.789	157.426	302.261	4510.005
-28.1	7.831	25.412	67.032	158.268	296.464	4510.005
-27.9	7.341	25.388	69.523	156.240	298.135	4510.005
-27.7	8.139	25.498	66.031	157.244	301.121	4510.005
-27.5	7.967	25.081	68.302	158.427	300.571	4510.005
-27.3	7.776	24.648	67.398	155.613	298.824	4510.005
-27.1	7.595	25.207	70.085	155.517	296.395	4510.005
-26.9	7.432	24.884	67.936	155.441	294.883	4510.005
-26.7	7.804	25.010	68.815	156.773	299.580	4510.005
-26.5	7.940	25.585	67.398	157.350	298.536	4510.005
-26.3	7.540	25.498	66.739	157.274	296.845	4510.005
-26.1	7.159	25.058	68.107	157.934	300.945	4510.005
-25.9	7.813	25.908	69.328	155.338	296.554	4510.005
-25.7	7.767	25.467	69.328	157.542	295.324	4510.005

Midpoint	Ratio: 62	19	6.3	2.1	0.65	Volume
-25.5	7.513	25.065	69.010	156.313	299.079	4510.005
-25.3	7.967	25.325	67.325	155.985	300.382	4510.005
-25.1	7.913	24.884	65.933	157.960	300.021	4510.005
-24.9	7.985	25.782	70.695	156.591	296.534	4510.005
-24.7	7.785	25.388	67.984	155.842	300.829	4510.005
-24.5	8.330	25.978	67.130	155.852	301.337	4510.005
-24.3	8.348	25.246	66.153	156.972	298.291	4510.005
-24.1	8.421	25.152	65.591	158.169	298.987	4510.005
-23.9	7.749	24.648	67.765	158.659	301.920	4510.005
-23.7	8.203	25.750	67.227	157.237	296.580	4510.005
-23.5	8.203	25.065	71.403	160.883	302.493	4510.005
-23.3	7.785	26.018	67.032	159.259	300.982	4510.005
-23.1	7.940	25.231	70.500	159.107	304.392	4510.005
-22.9	7.967	25.246	71.012	163.054	306.835	4510.005
-22.7	7.849	25.915	70.890	161.400	303.865	4510.005
-22.5	8.130	26.010	68.546	159.137	301.565	4510.005
-22.3	7.713	27.434	70.085	162.836	306.726	4510.005
-22.1	8.294	25.483	70.280	163.091	310.653	4510.005
-21.9	8.221	26.852	71.452	164.609	312.251	4510.005
-21.7	8.112	25.837	70.524	166.133	316.129	4510.005
-21.5	8.321	26.742	70.524	165.623	314.651	4510.005
-21.3	8.103	26.411	70.036	166.230	315.678	4510.005
-21.1	8.139	25.860	70.915	163.959	314.757	4510.005
-20.9	8.303	26.065	71.623	168.507	320.047	4510.005
-20.7	8.657	26.325	70.744	167.681	318.280	4510.005
-20.5	8.112	27.057	72.356	167.950	322.343	4510.005
-20.3	8.557	27.190	70.964	169.010	318.237	4510.005
-20.1	8.357	27.458	73.650	171.321	323.726	4510.005
-19.9	8.294	27.718	72.820	170.956	324.591	4510.005
-19.7	8.566	27.167	74.895	169.942	325.635	4510.005
-19.5	8.430	27.615	73.967	171.377	325.075	4510.005
-19.3	8.384	27.261	75.921	173.180	326.821	4510.005
-19.1	8.584	27.781	72.722	176.203	331.949	4510.005
-18.9	8.675	27.733	76.141	176.435	331.896	4510.005
-18.7	8.729	29.221	76.653	176.335	334.925	4510.005
-18.5	8.611	29.654	76.067	176.302	340.086	4510.005
-18.3	8.239	26.553	70.426	162.136	308.970	4510.005
-18.1	0.590	2.023	5.446	12.247	21.249	11585.470
-17.9	0.572	1.598	3.907	10.984	19.426	184.223
-17.7	0.381	1.314	3.980	9.267	16.781	160.425
-17.5	0.454	1.377	3.223	8.270	16.353	145.306
-17.3	0.299	1.299	3.736	7.961	15.621	133.670
-17.1	0.318	1.385	2.833	8.094	15.482	124.097
-16.9	0.381	1.133	2.515	6.897	13.861	115.931
-16.7	0.281	1.236	2.882	6.997	12.906	108.807

Midpoint	Ratio: 62	19	6.3	2.1	0.65	Volume
-16.5	0.372	1.149	2.466	7.093	12.287	102.492
-16.3	0.372	0.968	2.613	6.062	12.389	96.830
-16.1	0.327	0.984	3.345	6.364	11.074	91.707
-15.9	0.281	0.992	2.759	6.225	11.163	87.041
-15.7	0.354	0.866	2.759	6.029	11.262	82.765
-15.5	0.245	0.905	2.027	5.419	11.581	78.830
-15.3	0.127	0.669	1.661	5.064	10.619	75.194
-15.1	0.209	0.645	1.807	4.799	10.265	71.823
-14.9	0.163	0.574	1.929	4.743	9.857	68.689
-14.7	0.191	0.496	1.465	4.813	10.036	65.768
-14.5	0.218	0.661	1.758	4.534	9.559	63.041
-14.3	0.127	0.598	1.538	4.392	9.387	60.488
-14.1	0.172	0.543	1.416	4.524	9.002	58.097
-13.9	0.145	0.456	1.831	4.332	8.903	55.853
-13.7	0.172	0.425	1.636	4.302	8.972	53.744
-13.5	0.145	0.480	1.245	4.176	8.744	51.761
-13.3	0.127	0.551	1.587	4.040	8.896	49.893
-13.1	0.181	0.472	1.294	4.461	9.005	48.134
-12.9	0.181	0.441	1.270	4.229	8.780	46.475
-12.7	0.091	0.512	1.758	4.077	8.707	44.910
-12.5	0.145	0.386	1.343	4.130	8.565	43.433
-12.3	0.109	0.433	1.563	4.405	8.647	42.039
-12.1	0.154	0.464	1.294	4.153	8.717	40.722
-11.9	0.127	0.527	1.709	4.176	9.085	39.477
-11.7	0.127	0.425	1.587	4.249	9.125	38.302
-11.5	0.118	0.496	1.319	4.130	8.896	37.191
-11.3	0.118	0.504	2.051	4.359	9.158	36.142
-11.1	0.118	0.519	1.661	4.571	9.022	35.150
-10.9	0.118	0.614	1.636	4.571	9.370	34.214
-10.7	0.218	0.590	1.563	4.630	9.456	33.331
-10.5	0.091	0.637	2.222	4.839	9.648	32.497
-10.3	0.172	0.527	1.587	4.895	9.904	31.710
-10.1	0.227	0.567	2.149	5.197	9.847	30.969
-9.9	0.145	0.630	1.758	5.316	10.610	30.272
-9.7	0.145	0.740	2.662	5.118	11.064	29.615
-9.5	0.299	0.645	2.222	5.671	11.286	28.999
-9.3	0.227	0.708	2.222	5.989	11.316	28.420
-9.1	0.209	0.795	2.735	6.138	12.273	27.878
-8.9	0.172	0.874	2.222	6.549	12.820	27.372
-8.7	0.227	0.850	2.369	6.924	13.337	26.899
-8.5	0.281	0.724	2.784	7.371	14.269	26.459
-8.3	0.254	0.929	2.711	7.743	15.061	26.050
-8.1	0.218	0.803	3.150	8.001	15.667	25.673
-7.9	0.318	1.031	3.370	8.508	16.764	25.325
-7.7	0.318	1.086	3.565	9.194	17.736	25.006

Midpoint	Ratio: 62	19	6.3	2.1	0.65	Volume
-7.5	0.426	1.157	4.127	9.267	17.756	24.716
-7.3	0.218	1.369	3.834	9.181	18.173	24.452
-7.1	0.290	1.180	3.516	9.148	17.683	24.216
-6.9	0.336	1.180	2.808	9.009	16.953	24.006
-6.7	0.281	1.007	3.712	8.154	15.442	23.822
-6.5	0.272	1.039	3.223	7.199	13.897	23.663
-6.3	0.290	0.834	2.320	6.427	12.370	23.529
-6.1	0.163	0.606	2.198	5.492	10.225	23.420
-5.9	0.127	0.559	2.002	4.408	8.316	23.336
-5.7	0.136	0.512	1.343	3.563	6.612	23.276
-5.5	0.118	0.275	1.026	2.098	4.259	23.240
-5.3	0.064	0.197	0.513	1.379	2.768	23.227
-5.1	0.054	0.118	0.464	0.984	1.909	23.227
-4.9	0.018	0.126	0.244	0.686	1.309	23.227
-4.7	0.036	0.063	0.195	0.411	0.885	23.227
-4.5	0.009	0.039	0.171	0.365	0.660	23.227
-4.3	0.009	0.047	0.122	0.252	0.431	23.227
-4.1	0.009	0.039	0.024	0.192	0.305	23.227
-3.9	0.000	0.008	0.024	0.103	0.219	23.227
-3.7	0.009	0.024	0.122	0.056	0.169	23.227
-3.5	0.000	0.008	0.049	0.046	0.116	23.227
-3.3	0.000	0.031	0.000	0.053	0.126	23.227
-3.1	0.000	0.000	0.049	0.060	0.083	23.227
-2.9	0.000	0.000	0.000	0.030	0.126	23.227
-2.7	0.000	0.000	0.000	0.046	0.129	23.227
-2.5	0.018	0.000	0.049	0.050	0.099	23.227
-2.3	0.000	0.000	0.024	0.080	0.176	23.227
-2.1	0.000	0.008	0.000	0.070	0.169	23.227
-1.9	0.009	0.031	0.049	0.080	0.242	23.227
-1.7	0.000	0.079	0.049	0.182	0.447	23.227
-1.5	0.000	0.063	0.147	0.239	0.527	23.227
-1.3	0.045	0.016	0.147	0.424	0.696	23.227
-1.1	0.009	0.087	0.220	0.494	0.991	23.227
-0.9	0.027	0.063	0.317	0.679	1.475	23.227
-0.7	0.045	0.165	0.391	0.998	1.863	23.227
-0.5	0.036	0.212	0.635	1.283	2.383	23.227
-0.1	0.100	0.236	0.733	1.700	3.235	23.227
0.1	0.091	0.220	0.977	1.869	3.682	23.227
0.5	0.127	0.291	0.904	2.367	4.246	23.227
0.7	0.109	0.386	0.806	2.622	4.465	23.227
0.9	0.109	0.425	1.074	2.579	4.793	23.227
1.1	0.136	0.338	0.684	2.492	4.756	23.227
1.3	0.127	0.323	0.830	2.320	4.170	23.227
1.5	0.082	0.244	0.879	2.048	4.027	23.227
1.7	0.045	0.299	0.806	2.055	3.653	23.227

Midpoint	Ratio: 62	19	6.3	2.1	0.65	Volume
1.9	0.036	0.315	0.733	1.624	3.198	23.227
2.1	0.073	0.212	0.684	1.624	2.628	23.227
2.3	0.064	0.244	0.537	1.299	2.496	23.227
2.5	0.064	0.244	0.562	1.200	2.383	23.227
2.7	0.027	0.197	0.537	1.077	2.085	23.227
2.9	0.027	0.165	0.415	1.044	1.965	23.227
3.1	0.045	0.126	0.342	0.938	1.846	23.227
3.3	0.045	0.165	0.440	1.027	1.896	23.227
3.5	0.082	0.165	0.269	1.071	1.919	23.227
3.7	0.073	0.165	0.440	0.945	1.926	23.227
3.9	0.036	0.165	0.464	1.167	2.171	23.227
4.1	0.045	0.181	0.317	1.160	2.138	23.227
4.3	0.127	0.197	0.635	1.240	2.360	23.227
4.5	0.136	0.228	0.537	1.342	2.602	23.227
4.7	0.045	0.220	0.610	1.415	2.834	23.227
4.9	0.054	0.236	0.879	1.733	3.228	23.227
5.1	0.200	0.268	0.684	1.932	3.407	23.227
5.3	0.073	0.252	0.952	1.989	3.891	23.227
5.5	0.118	0.441	1.343	2.748	5.280	23.240
5.7	0.154	0.504	1.465	3.497	7.086	23.276
5.9	0.100	0.433	1.514	4.004	7.607	23.336
6.1	0.118	0.370	1.734	4.395	8.147	23.420
6.3	0.145	0.488	1.758	4.690	9.055	23.529
6.5	0.145	0.630	1.880	4.985	9.831	23.663
6.7	0.145	0.637	2.735	5.492	10.407	23.822
6.9	0.191	0.700	2.393	5.631	10.812	24.006
7.1	0.172	0.716	2.100	5.707	11.355	24.216
7.3	0.163	0.693	2.076	5.535	11.176	24.452
7.5	0.154	0.614	2.100	5.764	11.276	24.716
7.7	0.118	0.740	2.295	5.701	11.107	25.006
7.9	0.127	0.543	2.051	5.074	10.716	25.325
8.1	0.172	0.614	2.149	5.336	10.265	25.673
8.3	0.136	0.590	1.954	4.985	9.801	26.050
8.5	0.163	0.582	1.807	4.494	9.672	26.459
8.7	0.227	0.582	1.978	4.372	9.029	26.899
8.9	0.209	0.472	1.954	4.312	9.377	27.372
9.1	0.127	0.567	1.563	4.064	8.793	27.878
9.3	0.118	0.456	1.587	4.252	8.568	28.420
9.5	0.136	0.488	1.831	4.110	8.270	28.999
9.7	0.091	0.456	1.538	3.967	8.074	29.615
9.9	0.100	0.417	1.538	3.808	7.646	30.272
10.1	0.100	0.512	1.612	3.888	8.021	30.969
10.3	0.109	0.441	1.514	3.679	7.474	31.710
10.5	0.127	0.488	1.270	3.682	7.342	32.497
10.7	0.154	0.386	1.123	3.788	7.600	33.331

Midpoint	Ratio: 62	19	6.3	2.1	0.65	Volume
10.9	0.145	0.346	1.514	3.500	7.477	34.214
11.1	0.136	0.299	1.538	3.523	7.646	35.150
11.3	0.172	0.409	1.099	3.497	7.766	36.142
11.5	0.082	0.472	1.758	3.394	7.574	37.191
11.7	0.118	0.441	1.368	3.537	7.322	38.302
11.9	0.100	0.401	1.099	3.643	7.401	39.477
12.1	0.127	0.464	1.490	3.606	7.560	40.722
12.3	0.136	0.393	1.294	3.341	7.520	42.039
12.5	0.154	0.393	1.538	3.553	7.262	43.433
12.7	0.091	0.449	1.148	3.629	7.597	44.910
12.9	0.054	0.417	1.709	3.537	7.520	46.475
13.1	0.191	0.417	1.148	3.752	7.786	48.134
13.3	0.191	0.401	1.490	3.798	7.752	49.893
13.5	0.136	0.409	1.563	3.762	8.150	51.761
13.7	0.100	0.472	1.807	3.822	8.177	53.744
13.9	0.127	0.559	1.172	3.941	7.779	55.853
14.1	0.145	0.449	1.294	3.918	8.352	58.097
14.3	0.191	0.606	1.587	3.911	8.624	60.488
14.5	0.082	0.386	1.392	4.150	8.903	63.041
14.7	0.145	0.496	1.465	4.385	8.753	65.768
14.9	0.100	0.614	1.929	4.395	8.972	68.689
15.1	0.163	0.567	1.514	4.246	9.410	71.823
15.3	0.181	0.527	1.612	4.488	9.688	75.194
15.5	0.236	0.763	2.051	5.449	10.209	78.830
15.7	0.299	0.818	2.759	5.886	10.182	82.765
15.9	0.272	0.826	2.515	5.741	9.360	87.041
16.1	0.299	0.889	2.759	5.585	10.991	91.707
16.3	0.327	0.921	2.759	6.347	11.229	96.830
16.5	0.290	1.015	2.735	6.337	12.104	102.492
16.7	0.299	0.960	2.393	6.466	13.811	108.807
16.9	0.345	1.180	2.735	6.400	12.744	115.931
17.1	0.336	1.306	3.541	6.891	13.085	124.097
17.3	0.354	1.125	2.930	7.852	14.192	133.670
17.5	0.363	1.236	3.639	8.806	15.532	145.306
17.7	0.554	1.377	3.907	8.787	15.604	160.425
17.9	0.399	1.566	3.980	9.367	18.269	184.223
18.1	0.544	1.747	4.347	11.402	20.556	11585.470
18.3	8.239	25.065	70.158	160.631	306.616	4510.005
18.5	8.883	28.379	79.511	176.199	332.227	4510.005
18.7	8.693	27.387	75.115	176.816	331.462	4510.005
18.9	8.811	27.710	76.141	174.960	330.809	4510.005
19.1	8.902	28.190	75.481	174.287	327.322	4510.005
19.3	8.294	27.395	76.507	173.518	327.938	4510.005
19.5	8.076	27.198	71.550	174.522	324.408	4510.005
19.7	8.893	28.182	74.627	170.737	320.524	4510.005

Midpoint	Ratio: 62	19	6.3	2.1	0.65	Volume
19.9	8.648	28.001	75.017	171.334	323.460	4510.005
20.1	8.067	28.882	72.649	168.782	318.187	4510.005
20.3	8.357	26.277	72.746	167.168	322.029	4510.005
20.5	8.030	27.332	72.966	166.657	318.161	4510.005
20.7	7.867	27.253	72.575	165.076	316.991	4510.005
20.9	8.357	26.773	71.916	165.792	312.841	4510.005
21.1	8.067	27.198	73.454	164.891	310.968	4510.005
21.3	7.940	26.994	72.868	165.295	313.007	4510.005
21.5	7.722	26.592	71.354	164.204	308.990	4510.005
21.7	8.167	26.128	68.253	162.952	311.714	4510.005
21.9	8.167	25.506	71.965	160.810	310.534	4510.005
22.1	8.121	26.183	71.989	163.585	307.369	4510.005
22.3	8.094	26.734	71.135	161.026	305.546	4510.005
22.5	7.559	26.569	67.765	160.512	305.218	4510.005
22.7	8.176	25.829	70.744	160.890	304.721	4510.005
22.9	8.030	25.050	68.619	158.918	305.874	4510.005
23.1	7.668	25.215	69.230	160.747	309.530	4510.005
23.3	7.686	25.711	68.546	158.726	299.994	4510.005
23.5	8.148	25.585	67.301	159.926	300.790	4510.005
23.7	8.185	24.971	69.303	161.271	301.651	4510.005
23.9	7.749	25.758	67.740	158.371	299.951	4510.005
24.1	7.740	25.105	66.812	158.252	302.029	4510.005
24.3	7.477	25.687	68.155	156.565	299.961	4510.005
24.5	7.622	25.309	70.915	159.170	303.150	4510.005
24.7	7.903	25.199	67.667	157.390	296.895	4510.005
24.9	8.030	24.861	66.837	155.342	297.323	4510.005
25.1	7.613	25.215	69.474	157.642	298.897	4510.005
25.3	7.214	25.601	65.933	155.610	296.965	4510.005
25.5	7.749	25.215	69.034	156.097	288.374	4510.005
25.7	7.477	25.239	67.350	157.075	295.831	4510.005
25.9	7.858	24.766	66.983	154.815	299.020	4510.005
26.1	7.686	25.097	71.086	157.897	293.113	4510.005
26.3	8.339	25.671	66.299	157.025	298.397	4510.005
26.5	7.159	24.892	66.470	156.648	295.715	4510.005
26.7	7.795	24.491	64.712	157.324	295.944	4510.005
26.9	7.332	24.963	67.960	157.032	297.260	4510.005
27.1	7.876	25.333	68.546	154.208	296.531	4510.005
27.3	7.686	24.861	66.031	156.137	299.742	4510.005
27.5	8.112	24.365	66.690	154.626	300.584	4510.005
27.7	8.112	24.578	68.473	158.129	298.900	4510.005
27.9	7.985	24.735	67.813	156.976	299.016	4510.005
28.1	7.286	25.396	68.302	157.609	300.859	4510.005
28.3	8.257	25.097	69.572	156.439	299.961	4510.005
28.5	7.894	25.231	70.182	156.296	296.093	4510.005
28.7	7.386	24.680	69.303	157.194	298.118	4510.005

Midpoint	Ratio: 62	19	6.3	2.1	0.65	Volume
28.9	8.584	24.932	66.422	156.992	296.889	4510.005
29.1	8.212	24.373	66.153	156.671	304.532	4510.005
29.3	7.922	25.231	65.835	157.281	298.741	4510.005
29.5	7.640	25.475	68.204	158.093	299.762	4510.005
29.7	7.622	24.719	66.446	157.413	298.105	4510.005
29.9	7.940	25.860	68.717	156.571	298.271	4510.005
30.1	8.085	24.892	68.766	158.040	304.409	4510.005
30.3	7.940	25.624	66.617	155.259	299.752	4510.005
30.5	8.058	25.609	70.988	155.600	301.045	4510.005
30.7	7.332	25.553	67.618	157.072	299.799	4510.005
30.9	7.196	25.349	70.085	156.329	297.889	4510.005
31.1	7.622	24.751	68.082	156.359	298.566	4510.005
31.3	7.713	24.908	66.812	156.565	299.683	4510.005
31.5	7.840	24.444	67.667	156.704	299.928	4510.005
31.7	7.867	25.246	68.204	158.166	298.917	4510.005
31.9	7.831	25.003	69.474	158.928	301.171	4510.005
32.1	8.148	25.656	67.691	158.192	301.479	4510.005
32.3	7.441	25.679	67.472	159.064	300.475	4510.005
32.5	7.377	25.727	68.595	158.659	297.260	4510.005
32.7	8.294	25.349	67.032	158.623	302.987	4510.005
32.9	7.486	25.003	70.378	156.442	296.411	4510.005
33.1	7.640	25.215	68.717	159.455	298.884	4510.005
33.3	7.894	24.790	70.915	159.283	303.020	4510.005
33.5	7.595	25.105	66.910	157.158	301.042	4510.005
33.7	7.894	25.199	67.838	157.059	302.947	4510.005
33.9	7.858	25.443	65.469	157.184	299.268	4510.005
34.1	7.386	25.144	68.595	159.647	297.740	4510.005
34.3	7.713	25.380	66.763	157.645	300.902	4510.005
34.5	7.713	25.042	65.542	156.929	300.568	4510.005
34.7	7.631	26.159	69.376	157.241	302.808	4510.005
34.9	8.321	25.546	69.059	157.924	301.787	4510.005
35.1	8.003	25.081	70.622	158.371	301.423	4510.005
35.3	7.903	25.624	67.105	157.542	299.550	4510.005
35.5	8.357	24.971	71.403	158.272	299.663	4510.005
35.7	7.704	26.411	67.838	157.993	300.130	4510.005
35.9	8.584	25.695	68.375	156.541	299.689	4510.005
36.1	7.577	25.105	66.886	157.151	300.985	4510.005
36.3	7.804	25.073	68.155	158.010	302.371	4510.005
36.5	7.704	25.333	68.912	156.929	305.755	4510.005
36.7	8.158	24.995	66.959	157.032	301.496	4510.005
36.9	7.577	25.632	65.958	158.563	301.022	4510.005
37.1	7.931	25.191	72.404	160.897	301.592	4510.005
37.3	8.566	24.168	65.445	159.186	302.798	4510.005
37.5	7.795	24.916	69.938	156.793	302.596	4510.005
37.7	7.658	25.900	70.842	157.208	300.568	4510.005

Midpoint	Ratio: 62	19	6.3	2.1	0.65	Volume
37.9	7.649	25.357	70.402	157.211	302.861	4510.005
38.1	7.949	25.341	66.837	156.356	299.845	4510.005
38.3	7.903	24.404	67.716	158.709	300.365	4510.005
38.5	7.658	25.176	70.426	159.693	298.672	4510.005
38.7	7.867	25.349	68.741	159.624	305.546	4510.005
38.9	7.586	25.333	66.690	160.413	301.917	4510.005
39.1	7.903	24.837	69.987	160.366	300.332	4510.005
39.3	7.840	24.365	68.400	156.770	300.783	4510.005
39.5	7.649	25.223	66.519	159.465	301.217	4510.005
39.7	7.867	24.727	68.058	157.930	303.014	4510.005
39.9	7.704	25.065	66.446	159.863	301.784	4510.005
40.1	7.613	25.931	68.864	158.470	302.755	4510.005
40.3	7.867	26.049	69.962	157.791	298.695	4510.005
40.5	8.575	24.719	69.938	158.845	300.568	4510.005
40.7	7.958	24.460	67.984	156.734	302.662	4510.005
40.9	8.030	25.443	69.645	158.901	299.175	4510.005
41.1	7.668	25.427	70.036	159.113	301.323	4510.005
41.3	7.867	25.459	69.987	157.529	303.279	4510.005
41.5	8.167	25.294	67.105	159.418	300.362	4510.005
41.7	7.767	25.514	70.036	156.465	301.658	4510.005
41.9	7.949	25.223	66.886	157.489	304.591	4510.005
42.1	8.012	25.191	66.544	158.699	299.567	4510.005
42.3	7.903	24.664	69.157	157.910	301.559	4510.005
42.5	7.831	25.656	67.862	160.022	300.379	4510.005
42.7	7.813	25.003	67.838	158.500	300.654	4510.005
42.9	7.831	26.096	68.277	158.046	300.859	4510.005
43.1	7.758	25.742	69.669	158.925	301.562	4510.005
43.3	7.677	25.813	69.572	158.255	302.251	4510.005
43.5	7.686	25.160	70.085	156.996	299.606	4510.005
43.7	7.114	25.058	68.595	158.686	300.810	4510.005
43.9	7.731	25.034	68.937	159.571	301.950	4510.005
44.1	7.649	25.719	67.350	160.320	303.444	4510.005
44.3	7.840	25.239	66.739	157.029	300.849	4510.005
44.5	7.876	25.939	68.229	159.445	298.241	4510.005
44.7	7.268	25.837	68.058	159.853	305.652	4510.005
44.9	7.613	25.624	67.447	160.197	303.411	4510.005
45.1	7.740	25.845	67.520	158.338	301.283	4510.005
45.3	7.776	25.239	69.157	156.538	301.622	4510.005
45.5	7.813	26.435	69.865	158.288	303.236	4510.005
45.7	7.903	25.427	67.862	160.413	303.630	4510.005
45.9	7.649	25.805	67.594	159.495	299.391	4510.005
46.1	8.012	25.034	68.277	158.898	303.756	4510.005
46.3	7.740	25.845	67.154	158.944	303.070	4510.005
46.5	8.021	25.341	67.789	158.891	300.796	4510.005
46.7	7.740	25.341	67.716	156.512	300.604	4510.005

Midpoint	Ratio: 62	19	6.3	2.1	0.65	Volume
46.9	7.776	25.372	71.012	156.167	302.215	4510.005
47.1	7.468	25.199	72.600	156.144	302.158	4510.005
47.3	7.931	25.254	67.936	158.487	303.024	4510.005
47.5	7.813	25.546	71.232	159.428	302.493	4510.005
47.7	7.976	26.167	67.862	158.590	300.408	4510.005
47.9	8.021	24.837	67.789	159.050	302.954	4510.005
48.1	7.513	26.348	67.203	160.333	298.281	4510.005
48.3	8.176	25.254	66.495	158.736	300.196	4510.005
48.5	7.731	24.680	71.916	158.159	302.964	4510.005
48.7	7.776	25.435	68.766	158.898	299.673	4510.005
48.9	7.395	25.018	68.693	158.878	302.530	4510.005
49.1	7.568	24.743	67.374	157.615	302.039	4510.005
49.3	7.885	25.239	65.909	159.693	299.630	4510.005
49.5	7.858	25.058	71.135	158.759	299.676	4510.005
49.7	7.795	25.372	68.741	155.736	301.738	4510.005
49.9	7.658	25.845	67.765	160.042	301.933	4510.005
50.1	8.021	25.782	69.157	156.707	304.767	4510.005
50.3	7.976	24.121	66.348	159.882	302.626	4510.005
50.5	7.849	24.727	68.619	160.419	300.289	4510.005
50.7	7.867	25.483	68.986	160.406	305.132	4510.005
50.9	7.931	25.081	65.787	157.198	302.434	4510.005
51.1	7.677	24.932	65.469	158.507	304.813	4510.005
51.3	7.840	25.490	70.255	158.354	302.149	4510.005
51.5	7.677	25.333	66.275	156.223	298.145	4510.005
51.7	7.749	24.908	66.446	160.078	303.663	4510.005
51.9	8.130	25.774	69.108	158.361	299.016	4510.005
52.1	7.931	25.876	67.520	158.984	301.406	4510.005
52.3	7.840	25.915	69.181	155.902	302.278	4510.005
52.5	7.613	25.624	69.425	156.488	301.283	4510.005
52.7	8.085	25.601	68.961	159.594	301.509	4510.005
52.9	7.522	24.735	69.279	159.843	303.899	4510.005
53.1	7.559	25.467	67.862	159.538	300.869	4510.005
53.3	7.913	25.065	68.912	159.617	303.454	4510.005
53.5	7.631	24.680	68.302	158.560	301.983	4510.005
53.7	7.804	25.750	69.743	157.798	303.163	4510.005
53.9	7.731	25.797	69.987	158.215	300.100	4510.005
54.1	7.949	25.388	66.129	158.331	303.385	4510.005
54.3	7.776	24.853	67.154	158.891	303.872	4510.005
54.5	8.076	25.215	66.763	158.785	302.579	4510.005
54.7	8.040	25.467	67.130	155.776	299.944	4510.005
54.9	7.658	25.317	70.280	158.600	299.577	4510.005
55.1	7.704	25.168	69.645	159.299	303.421	4510.005
55.3	7.831	25.664	67.813	159.707	301.042	4510.005
55.5	7.459	26.049	69.034	158.166	298.993	4510.005
55.7	7.613	24.845	66.544	158.931	301.018	4510.005

Midpoint	Ratio: 62	19	6.3	2.1	0.65	Volume
55.9	8.012	25.396	66.617	155.050	302.135	4510.005
56.1	7.967	25.593	69.450	157.526	300.998	4510.005
56.3	7.677	24.892	69.132	156.319	305.509	4510.005
56.5	8.239	24.672	69.157	158.232	303.362	4510.005
56.7	7.931	25.884	66.299	158.732	306.411	4510.005
56.9	7.994	24.884	68.131	158.533	296.352	4510.005
57.1	8.720	25.530	68.400	159.601	301.966	4510.005
57.3	7.776	24.979	68.253	160.738	300.783	4510.005
57.5	7.831	24.845	67.520	157.410	301.436	4510.005
57.7	7.785	25.286	68.790	155.981	299.364	4510.005
57.9	8.221	24.074	67.179	157.526	301.532	4510.005
58.1	7.931	25.121	68.277	159.253	300.982	4510.005
58.3	7.586	24.703	68.180	157.145	300.438	4510.005
58.5	7.795	26.041	68.302	157.509	301.598	4510.005
58.7	7.795	25.325	67.960	158.229	300.694	4510.005
58.9	7.677	25.380	70.207	159.302	300.929	4510.005
59.1	8.130	25.380	66.031	156.558	299.219	4510.005
59.3	7.994	25.553	68.058	159.256	301.012	4510.005
59.5	7.595	25.302	71.476	157.990	298.410	4510.005
59.7	7.631	24.947	69.596	159.816	298.509	4510.005
59.9	7.377	24.680	70.817	156.535	299.195	4510.005
60.1	7.849	24.688	65.762	160.247	301.864	4510.005
60.3	7.903	24.892	66.715	158.364	298.516	4510.005
60.5	7.622	25.128	68.571	159.147	299.610	4510.005
60.7	7.858	24.884	68.741	159.786	306.782	4510.005
60.9	8.040	25.711	67.960	157.297	305.291	4510.005
61.1	7.785	24.593	68.717	161.188	302.347	4510.005
61.3	7.913	24.940	67.887	157.668	301.217	4510.005
61.5	7.332	24.971	65.909	157.890	302.801	4510.005
61.7	7.722	25.050	66.593	157.287	304.101	4510.005
61.9	8.357	24.766	69.352	158.577	300.017	4510.005
62.1	7.477	24.963	72.820	158.659	300.399	4510.005
62.3	8.185	25.923	67.081	160.834	301.890	4510.005
62.5	7.804	25.034	66.275	158.146	298.032	4510.005
62.7	8.158	25.671	68.522	157.423	304.131	4510.005
62.9	8.067	25.270	72.233	157.337	302.782	4510.005
63.1	7.813	25.955	69.108	155.222	301.515	4510.005
63.3	8.230	24.979	67.203	157.526	303.938	4510.005
63.5	7.985	25.624	65.884	157.317	300.584	4510.005
63.7	7.495	24.908	69.205	158.799	300.935	4510.005
63.9	7.586	24.853	67.569	158.315	300.412	4510.005
64.1	7.577	24.798	69.840	157.513	303.325	4510.005
64.3	7.704	24.884	64.151	158.623	299.073	4510.005
64.5	8.076	24.680	68.424	157.884	304.320	4510.005
64.7	7.713	24.861	69.523	159.226	300.332	4510.005

Midpoint	Ratio: 62	19	6.3	2.1	0.65	Volume
64.9	7.785	24.373	67.325	159.216	299.185	4510.005
65.1	8.811	25.695	67.301	157.983	300.011	4510.005
65.3	7.550	25.065	69.865	157.135	297.326	4510.005
65.5	7.949	25.349	67.252	159.475	299.059	4510.005
65.7	7.894	24.916	70.011	158.457	303.226	4510.005
65.9	7.785	26.073	66.739	157.340	302.917	4510.005
66.1	8.130	25.569	69.987	159.011	300.853	4510.005
66.3	7.141	25.215	70.451	158.567	299.318	4510.005
66.5	7.931	25.711	67.472	158.709	299.775	4510.005
66.7	7.577	25.632	67.862	159.495	298.914	4510.005
66.9	7.894	25.380	66.470	155.544	299.560	4510.005
67.1	7.931	24.609	69.303	157.403	301.870	4510.005
67.3	7.903	25.003	67.887	159.475	296.517	4510.005
67.5	8.148	25.199	69.328	159.206	302.450	4510.005
67.7	7.404	25.538	70.719	157.095	299.716	4510.005
67.9	7.949	26.899	69.865	157.314	300.289	4510.005
68.1	7.658	25.081	69.132	158.401	300.889	4510.005
68.3	7.849	24.310	67.569	157.234	301.903	4510.005
68.5	7.885	25.199	67.350	156.379	301.376	4510.005
68.7	8.030	25.829	66.983	158.079	298.983	4510.005
68.9	7.568	25.727	67.887	156.333	300.425	4510.005
69.1	7.885	25.435	66.763	158.872	300.969	4510.005
69.3	7.486	25.136	69.743	157.857	297.008	4510.005
69.5	7.831	24.790	68.302	156.150	299.878	4510.005
69.7	8.003	24.932	68.912	157.861	298.934	4510.005
69.9	7.749	25.380	68.986	159.027	304.449	4510.005
70.1	7.586	24.160	65.152	157.420	300.906	4510.005
70.3	7.949	24.861	66.470	157.383	298.407	4510.005
70.5	7.577	26.073	67.130	157.310	298.648	4510.005
70.7	7.994	24.869	67.008	158.795	298.529	4510.005
70.9	7.922	24.837	71.599	159.511	298.890	4510.005
71.1	8.003	25.829	66.397	158.838	296.842	4510.005
71.3	7.931	25.113	67.813	157.834	301.817	4510.005
71.5	7.713	25.113	67.520	157.105	302.205	4510.005
71.7	8.266	25.302	68.155	156.836	298.257	4510.005
71.9	7.976	24.845	68.180	158.457	296.418	4510.005
72.1	8.312	24.955	67.740	158.345	298.967	4510.005
72.3	7.668	24.782	66.153	157.711	301.065	4510.005
72.5	7.776	25.671	66.763	157.731	299.746	4510.005
72.7	7.776	24.688	66.495	156.316	296.713	4510.005
72.9	7.758	25.986	65.591	157.539	298.211	4510.005
73.1	7.958	25.443	66.373	157.890	297.160	4510.005
73.3	8.030	24.916	69.401	157.300	300.200	4510.005
73.5	7.903	24.947	69.743	158.076	301.602	4510.005
73.7	7.913	25.262	65.274	156.548	296.766	4510.005

Midpoint	Ratio: 62	19	6.3	2.1	0.65	Volume
73.9	7.713	25.270	68.473	155.229	301.854	4510.005
74.1	7.931	24.751	69.059	158.427	299.113	4510.005
74.3	7.395	25.073	69.205	158.818	301.864	4510.005
74.5	7.903	25.286	66.544	157.705	301.144	4510.005
74.7	7.903	25.538	69.474	155.673	295.606	4510.005
74.9	7.495	24.641	69.010	157.718	301.572	4510.005
75.1	7.668	25.357	67.203	159.823	298.483	4510.005
75.3	7.677	25.294	68.693	158.162	299.626	4510.005
75.5	7.958	25.215	69.181	158.056	301.708	4510.005
75.7	8.121	25.711	67.496	156.492	301.429	4510.005
75.9	7.785	24.641	64.199	158.586	296.918	4510.005
76.1	7.858	25.081	68.522	155.249	297.800	4510.005
76.3	8.076	24.790	65.933	156.714	299.096	4510.005
76.5	8.393	25.498	68.473	157.095	299.606	4510.005
76.7	7.468	24.648	68.400	158.848	296.020	4510.005
76.9	7.813	25.498	69.083	157.068	297.389	4510.005
77.1	7.631	24.774	67.838	155.139	300.425	4510.005
77.3	7.250	24.617	68.937	158.020	298.847	4510.005
77.5	7.985	25.254	67.765	155.766	298.609	4510.005
77.7	7.940	25.530	69.987	156.959	299.951	4510.005
77.9	7.677	24.766	67.130	156.913	299.331	4510.005
78.1	7.577	24.444	67.716	158.059	299.706	4510.005
78.3	7.822	25.790	68.180	155.255	295.261	4510.005
78.5	7.404	24.404	66.055	157.221	301.615	4510.005
78.7	8.285	24.554	70.402	156.512	298.924	4510.005
78.9	8.021	25.435	67.081	154.162	301.370	4510.005
79.1	7.477	25.978	65.298	157.165	299.083	4510.005
79.3	7.722	24.916	67.765	158.229	300.829	4510.005
79.5	7.586	25.097	66.715	157.324	296.388	4510.005
79.7	8.257	24.774	68.741	157.413	301.350	4510.005
79.9	8.312	24.633	66.080	157.029	295.609	4510.005
80.1	7.568	25.561	68.668	157.032	294.310	4510.005
80.3	7.949	25.490	67.105	155.925	298.963	4510.005
80.5	7.876	24.648	67.838	157.125	298.934	4510.005
80.7	7.559	25.199	64.785	156.412	297.952	4510.005
80.9	7.631	25.640	67.496	157.387	299.132	4510.005
81.1	7.668	24.271	69.401	156.621	300.869	4510.005
81.3	7.559	24.153	65.738	156.183	297.485	4510.005
81.5	7.704	25.498	69.181	156.651	295.493	4510.005
81.7	7.731	24.483	66.617	153.824	298.549	4510.005
81.9	7.985	24.861	65.542	154.258	298.960	4510.005
82.1	7.749	24.452	65.958	157.052	300.839	4510.005
82.3	8.457	24.892	67.350	157.871	296.902	4510.005
82.5	7.368	25.538	69.279	157.241	295.579	4510.005
82.7	7.513	24.326	63.198	154.738	295.758	4510.005

Midpoint	Ratio: 62	19	6.3	2.1	0.65	Volume
82.9	7.668	24.578	66.275	157.755	298.045	4510.005
83.1	7.668	25.380	66.934	156.091	298.178	4510.005
83.3	7.731	24.609	65.298	155.159	295.327	4510.005
83.5	7.522	26.136	67.789	157.105	294.224	4510.005
83.7	7.577	24.900	69.328	156.336	295.417	4510.005
83.9	7.795	25.231	67.154	157.920	295.871	4510.005
84.1	7.178	24.688	66.886	155.869	298.151	4510.005
84.3	7.785	24.208	65.909	154.085	295.513	4510.005
84.5	7.658	25.616	67.350	155.816	298.708	4510.005
84.7	7.595	24.286	65.396	154.891	294.224	4510.005
84.9	8.040	25.160	67.691	156.836	299.238	4510.005
85.1	7.432	24.656	65.274	155.212	295.530	4510.005
85.3	7.441	24.412	67.301	154.821	291.589	4510.005
85.5	7.749	25.160	64.859	155.236	292.722	4510.005
85.7	7.323	24.617	66.641	154.748	291.874	4510.005
85.9	7.976	25.089	68.400	153.237	294.983	4510.005
86.1	7.368	25.097	67.716	153.890	290.886	4510.005
86.3	7.713	25.081	64.395	154.079	293.518	4510.005
86.5	7.976	24.467	68.204	154.291	292.944	4510.005
86.7	7.595	24.995	66.397	154.450	291.201	4510.005
86.9	7.622	23.972	66.080	154.162	291.569	4510.005
87.1	7.568	25.105	68.009	153.293	294.068	4510.005
87.3	7.649	25.097	66.763	154.586	290.502	4510.005
87.5	7.767	25.065	68.619	154.941	299.467	4510.005
87.7	7.922	24.357	65.396	151.656	293.882	4510.005
87.9	7.686	24.633	65.860	152.607	291.675	4510.005
88.1	7.504	24.042	69.181	153.655	292.769	4510.005
88.3	7.695	24.892	67.325	151.991	292.275	4510.005
88.5	7.686	24.460	66.251	155.656	288.258	4510.005
88.7	7.495	24.900	66.299	153.154	289.547	4510.005
88.9	7.314	24.223	64.053	150.277	291.310	4510.005
89.1	7.713	24.326	67.594	157.370	288.291	4510.005
89.3	7.577	24.759	65.738	152.518	287.210	4510.005
89.5	7.459	24.625	67.374	150.015	289.302	4510.005
89.7	7.377	24.247	63.076	150.529	291.642	4510.005
89.9	7.867	24.483	64.468	151.805	288.795	4510.005
90.1	7.831	24.263	64.199	152.640	286.153	4510.005
90.3	7.540	24.578	66.226	150.327	285.861	4510.005
90.5	7.286	24.105	66.544	151.020	287.167	4510.005
90.7	7.604	23.657	64.615	150.095	284.118	4510.005
90.9	7.305	24.467	66.446	149.949	286.730	4510.005
91.1	7.559	24.334	65.469	147.738	284.854	4510.005
91.3	8.067	24.255	64.321	148.918	285.918	4510.005
91.5	7.550	23.311	63.247	149.044	281.851	4510.005
91.7	7.450	24.027	63.687	148.361	285.891	4510.005

Midpoint	Ratio: 62	19	6.3	2.1	0.65	Volume
91.9	7.395	23.987	62.295	149.319	280.992	4510.005
92.1	7.631	22.350	64.028	147.887	280.979	4510.005
92.3	7.341	23.531	66.886	148.212	282.361	4510.005
92.5	7.722	23.468	66.812	149.127	280.870	4510.005
92.7	7.649	23.948	63.931	149.800	285.185	4510.005
92.9	7.468	23.657	64.688	149.217	279.458	4510.005
93.1	7.640	23.004	65.884	148.282	282.527	4510.005
93.3	7.205	24.192	61.562	148.888	282.812	4510.005
93.5	7.187	22.854	62.026	150.271	280.051	4510.005
93.7	7.550	23.854	64.395	150.164	281.582	4510.005
93.9	7.477	24.129	66.275	148.179	280.144	4510.005
94.1	6.969	22.720	62.417	148.985	281.214	4510.005
94.3	7.250	23.303	65.591	149.137	279.276	4510.005
94.5	7.314	22.319	64.248	148.696	279.948	4510.005
94.7	7.658	23.318	64.932	148.153	284.095	4510.005
94.9	7.060	24.412	64.370	149.515	283.137	4510.005
95.1	7.259	23.649	64.883	148.666	283.455	4510.005
95.3	7.640	23.460	65.225	148.766	282.938	4510.005
95.5	8.040	23.767	64.639	151.278	280.035	4510.005
95.7	7.795	23.673	63.100	147.029	283.452	4510.005
95.9	7.776	23.641	62.612	148.345	286.339	4510.005
96.1	7.577	23.901	61.416	149.104	283.336	4510.005
96.3	8.012	24.475	67.911	150.711	286.736	4510.005
96.5	7.423	24.341	66.153	148.663	283.704	4510.005
96.7	7.586	23.594	64.517	151.315	286.243	4510.005
96.9	7.767	24.279	63.198	149.760	287.727	4510.005
97.1	7.468	23.751	65.274	151.861	288.201	4510.005
97.3	7.849	24.884	67.179	151.318	289.242	4510.005
97.5	7.305	24.562	64.492	152.060	288.430	4510.005
97.7	7.840	24.593	64.517	152.044	289.746	4510.005
97.9	7.550	24.460	65.127	153.698	289.769	4510.005
98.1	7.813	25.176	64.932	152.985	294.887	4510.005
98.3	7.513	26.128	65.225	152.915	291.874	4510.005
98.5	7.568	24.940	67.350	153.240	294.336	4510.005
98.7	7.804	24.412	65.640	153.138	295.427	4510.005
98.9	7.985	24.562	64.053	153.413	294.104	4510.005
99.1	7.341	25.215	64.566	155.763	293.650	4510.005
99.3	7.368	25.081	66.080	155.898	299.583	4510.005
99.5	7.677	24.900	67.374	156.810	290.863	4510.005
99.7	7.776	25.034	66.177	155.328	298.960	4510.005
99.9	7.677	24.751	65.152	156.548	297.217	4510.005
100.1	7.849	18.817	47.179	113.019	217.537	4510.005

Table of K^+ population data for simulation with K^+ and Na^+ across bulk density ratios (#JJF-GRS12F1)

Population in histogram bin at K^+/Na^+ ratio:

Midpoint	62	19	6.3	2.1	0.65	Volume
-100.1	1.225	0.614	0.537	0.414	0.215	4469.109
-99.9	94.624	1.314	1.245	0.852	0.461	4469.109
-99.7	488.144	376.510	341.363	274.407	157.095	4469.109
-99.5	486.220	473.183	430.666	342.429	192.430	4469.109
-99.3	495.322	460.945	432.400	335.664	194.946	4469.109
-99.1	482.999	463.566	438.724	339.933	192.825	4469.109
-98.9	481.511	467.721	425.049	333.609	193.252	4469.109
-98.7	481.184	466.116	440.238	332.966	191.860	4469.109
-98.5	469.334	463.991	421.191	332.386	190.110	4469.109
-98.3	479.633	454.224	426.539	333.454	188.155	4469.109
-98.1	476.185	461.488	423.315	331.495	189.295	4469.109
-97.9	479.243	457.915	426.392	328.081	189.239	4469.109
-97.7	472.410	463.700	408.126	329.145	189.815	4469.109
-97.5	486.983	460.072	411.277	326.158	188.993	4469.109
-97.3	469.996	453.839	408.786	325.668	185.692	4469.109
-97.1	478.979	459.568	423.071	328.711	187.283	4469.109
-96.9	469.660	451.462	428.908	323.915	187.263	4469.109
-96.7	467.719	440.720	406.710	329.824	186.166	4469.109
-96.5	467.129	457.963	399.995	324.538	184.744	4469.109
-96.3	460.323	451.061	412.644	322.317	186.070	4469.109
-96.1	463.545	450.990	416.869	320.448	184.353	4469.109
-95.9	460.323	441.585	411.521	323.762	184.094	4469.109
-95.7	464.633	441.821	412.009	321.356	182.852	4469.109
-95.5	456.431	444.749	403.389	322.655	185.294	4469.109
-95.3	462.401	449.085	396.893	321.730	183.359	4469.109
-95.1	461.430	448.117	398.456	318.747	179.673	4469.109
-94.9	459.271	441.790	394.818	318.744	182.487	4469.109
-94.7	453.445	441.837	398.310	317.793	181.834	4469.109
-94.5	457.057	442.530	397.919	320.481	181.592	4469.109
-94.3	453.119	449.487	403.804	318.306	181.529	4469.109
-94.1	455.496	442.302	420.458	312.871	180.134	4469.109
-93.9	459.298	449.550	405.733	316.444	181.768	4469.109
-93.7	458.871	447.614	409.030	317.849	183.471	4469.109
-93.5	447.719	441.947	397.308	315.506	180.309	4469.109
-93.3	463.889	445.174	394.696	317.435	182.527	4469.109
-93.1	465.260	439.476	393.987	317.511	182.394	4469.109
-92.9	454.144	435.848	402.656	318.181	182.490	4469.109
-92.7	448.482	450.888	409.616	314.014	181.088	4469.109
-92.5	455.859	445.662	379.043	315.715	181.118	4469.109
-92.3	451.630	448.165	401.338	317.345	178.788	4469.109
-92.1	442.992	445.135	392.766	313.872	182.020	4469.109
-91.9	454.797	437.131	391.057	312.592	181.824	4469.109
-91.7	458.418	439.720	392.449	320.497	180.329	4469.109

Midpoint	Ratio: 62	19	6.3	2.1	0.65	Volume
-91.5	451.930	443.600	407.955	317.352	183.060	4469.109
-91.3	458.980	440.366	404.122	319.387	180.866	4469.109
-91.1	460.296	443.018	395.379	317.806	184.466	4469.109
-90.9	455.950	437.414	402.827	316.523	182.934	4469.109
-90.7	454.670	441.082	398.871	320.083	183.259	4469.109
-90.5	463.018	440.830	400.776	318.432	184.708	4469.109
-90.3	459.017	442.246	415.159	322.595	182.931	4469.109
-90.1	462.283	456.892	393.011	320.090	184.065	4469.109
-89.9	463.871	442.128	402.827	322.844	181.953	4469.109
-89.7	456.303	453.123	407.907	322.466	186.372	4469.109
-89.5	471.403	458.789	406.466	318.704	183.461	4469.109
-89.3	465.396	449.219	403.462	323.265	185.119	4469.109
-89.1	463.254	453.217	408.786	326.967	187.114	4469.109
-88.9	463.200	451.250	411.179	321.644	187.691	4469.109
-88.7	460.605	458.191	404.927	325.631	185.049	4469.109
-88.5	468.862	456.097	420.458	324.243	187.591	4469.109
-88.3	464.416	448.306	411.472	325.691	186.000	4469.109
-88.1	466.412	441.467	403.438	321.995	185.434	4469.109
-87.9	467.782	448.755	416.942	325.810	187.797	4469.109
-87.7	481.393	451.903	402.510	330.964	186.451	4469.109
-87.5	472.600	455.602	402.778	325.075	186.179	4469.109
-87.3	470.559	452.336	406.344	327.484	185.579	4469.109
-87.1	466.485	466.069	417.821	326.457	188.141	4469.109
-86.9	472.310	453.784	423.071	330.454	188.947	4469.109
-86.7	459.180	449.109	416.380	325.184	186.375	4469.109
-86.5	477.065	446.213	412.107	323.089	187.764	4469.109
-86.3	470.731	457.231	407.565	326.609	186.955	4469.109
-86.1	479.669	443.962	407.540	329.370	189.149	4469.109
-85.9	467.818	462.795	430.104	327.279	191.685	4469.109
-85.7	470.423	463.031	421.704	330.192	188.937	4469.109
-85.5	475.323	455.444	411.008	327.739	190.266	4469.109
-85.3	478.889	449.558	410.349	327.040	190.339	4469.109
-85.1	471.720	460.512	430.006	331.339	191.022	4469.109
-84.9	471.566	455.232	422.998	331.034	191.240	4469.109
-84.7	472.864	455.830	408.468	328.737	187.694	4469.109
-84.5	465.659	468.248	412.034	325.012	191.522	4469.109
-84.3	473.707	465.156	425.000	333.377	189.364	4469.109
-84.1	476.303	463.369	411.521	329.045	188.231	4469.109
-83.9	474.179	463.125	410.666	334.153	186.415	4469.109
-83.7	465.940	460.922	424.610	328.280	190.631	4469.109
-83.5	481.674	461.048	426.392	332.224	190.418	4469.109
-83.3	483.144	453.666	438.578	329.851	190.766	4469.109
-83.1	480.939	464.156	430.275	326.274	190.425	4469.109
-82.9	478.925	453.516	415.990	332.774	192.841	4469.109
-82.7	468.481	463.448	413.303	331.909	192.218	4469.109

Midpoint	Ratio: 62	19	6.3	2.1	0.65	Volume
-82.5	480.005	458.230	422.681	326.695	187.101	4469.109
-82.3	477.382	466.336	412.327	330.901	191.476	4469.109
-82.1	473.299	464.282	418.407	333.079	189.587	4469.109
-81.9	481.647	466.800	411.154	334.159	191.529	4469.109
-81.7	474.742	457.797	412.180	332.572	189.825	4469.109
-81.5	478.326	457.160	415.184	332.638	190.902	4469.109
-81.3	478.408	460.190	428.468	329.463	192.129	4469.109
-81.1	480.767	464.321	423.047	332.194	191.804	4469.109
-80.9	477.028	466.092	411.838	333.321	192.059	4469.109
-80.7	474.107	466.596	421.679	334.355	191.038	4469.109
-80.5	491.084	459.230	416.844	331.843	190.283	4469.109
-80.3	473.009	460.953	417.040	331.027	192.553	4469.109
-80.1	476.847	468.729	428.859	333.450	190.700	4469.109
-79.9	478.889	460.355	422.998	336.920	188.261	4469.109
-79.7	476.212	457.852	428.541	333.205	191.386	4469.109
-79.5	479.152	462.629	430.617	332.476	189.292	4469.109
-79.3	479.397	462.448	419.652	332.343	190.578	4469.109
-79.1	469.579	463.361	431.032	334.862	190.770	4469.109
-78.9	477.192	479.557	423.755	334.677	190.839	4469.109
-78.7	470.994	467.855	427.809	332.144	190.829	4469.109
-78.5	475.831	461.386	422.900	331.604	192.795	4469.109
-78.3	486.710	473.568	425.928	336.440	190.558	4469.109
-78.1	481.965	460.748	410.104	332.565	189.921	4469.109
-77.9	486.892	459.261	412.668	333.798	191.668	4469.109
-77.7	477.528	462.008	429.298	333.589	191.668	4469.109
-77.5	486.239	464.967	413.377	332.277	191.042	4469.109
-77.3	479.506	464.770	428.859	335.780	191.837	4469.109
-77.1	480.858	461.591	424.243	333.990	190.763	4469.109
-76.9	477.945	475.906	425.318	333.526	192.536	4469.109
-76.7	489.278	467.548	425.220	331.710	192.583	4469.109
-76.5	482.972	464.676	423.193	335.542	190.283	4469.109
-76.3	466.022	461.693	428.639	331.641	191.118	4469.109
-76.1	483.181	461.661	416.429	333.871	194.164	4469.109
-75.9	478.281	467.288	427.784	332.605	193.862	4469.109
-75.7	476.992	463.511	426.392	333.563	194.091	4469.109
-75.5	485.994	462.732	425.855	332.721	191.741	4469.109
-75.3	480.722	462.134	428.273	338.598	194.943	4469.109
-75.1	476.067	467.682	422.094	333.490	192.129	4469.109
-74.9	483.825	458.655	433.791	334.769	191.817	4469.109
-74.7	484.206	455.964	436.404	334.173	191.098	4469.109
-74.5	493.452	465.557	419.579	332.055	190.909	4469.109
-74.3	489.687	470.365	414.988	332.980	190.853	4469.109
-74.1	487.500	470.570	440.287	334.130	192.039	4469.109
-73.9	473.154	460.024	449.225	334.998	192.298	4469.109
-73.7	477.736	456.625	414.769	333.679	191.618	4469.109

Midpoint	Ratio: 62	19	6.3	2.1	0.65	Volume
-73.5	477.019	463.708	416.820	340.079	192.106	4469.109
-73.3	478.880	468.186	409.176	338.823	192.013	4469.109
-73.1	479.805	467.296	441.239	335.031	191.426	4469.109
-72.9	481.311	473.073	415.452	335.091	193.219	4469.109
-72.7	474.696	462.000	428.541	333.596	195.082	4469.109
-72.5	488.616	462.409	428.151	337.636	191.211	4469.109
-72.3	473.326	471.963	428.248	338.061	192.112	4469.109
-72.1	479.460	459.733	431.398	335.943	193.991	4469.109
-71.9	475.631	459.230	422.387	331.475	191.777	4469.109
-71.7	482.300	471.711	421.411	333.556	193.677	4469.109
-71.5	495.186	469.083	418.309	333.573	193.312	4469.109
-71.3	472.174	458.931	418.969	339.516	192.033	4469.109
-71.1	485.122	467.666	428.321	333.391	196.209	4469.109
-70.9	479.488	463.810	423.755	334.100	193.723	4469.109
-70.7	478.653	463.841	419.872	333.434	191.824	4469.109
-70.5	478.226	464.518	424.170	331.336	193.484	4469.109
-70.3	465.205	462.197	437.943	333.284	193.620	4469.109
-70.1	470.187	462.566	420.458	337.073	190.578	4469.109
-69.9	492.364	461.764	421.069	335.058	192.845	4469.109
-69.7	480.322	472.923	420.068	337.060	193.865	4469.109
-69.5	488.643	459.056	420.385	334.222	194.641	4469.109
-69.3	487.264	485.869	415.355	334.050	191.794	4469.109
-69.1	477.700	465.305	412.327	334.846	189.596	4469.109
-68.9	488.008	460.764	423.975	337.481	191.927	4469.109
-68.7	484.696	464.613	434.622	335.654	194.190	4469.109
-68.5	475.177	463.896	418.187	336.632	190.385	4469.109
-68.3	484.578	464.361	433.669	337.421	194.190	4469.109
-68.1	475.649	472.357	428.785	334.014	191.728	4469.109
-67.9	484.161	468.335	428.614	333.115	190.607	4469.109
-67.7	476.257	472.852	423.706	333.381	193.484	4469.109
-67.5	480.313	467.587	415.770	332.966	193.995	4469.109
-67.3	479.315	464.369	423.486	336.692	193.285	4469.109
-67.1	480.794	460.174	409.054	333.924	192.427	4469.109
-66.9	484.505	463.212	424.170	336.904	194.505	4469.109
-66.7	482.192	466.564	423.438	336.410	191.446	4469.109
-66.5	476.375	459.914	430.739	332.469	191.453	4469.109
-66.3	481.838	472.679	420.019	330.640	193.070	4469.109
-66.1	478.762	462.385	414.305	342.267	190.916	4469.109
-65.9	479.433	462.252	420.507	340.245	190.773	4469.109
-65.7	482.391	464.864	427.076	335.518	195.490	4469.109
-65.5	480.903	469.704	428.199	335.512	194.608	4469.109
-65.3	481.284	466.730	422.729	334.285	195.347	4469.109
-65.1	481.602	468.492	414.329	337.663	192.483	4469.109
-64.9	482.328	467.265	421.826	336.234	192.944	4469.109
-64.7	495.049	466.454	427.125	336.549	194.906	4469.109

Midpoint	Ratio: 62	19	6.3	2.1	0.65	Volume
-64.5	495.095	467.879	424.561	334.348	192.858	4469.109
-64.3	478.353	458.474	423.682	335.886	194.489	4469.109
-64.1	479.978	467.776	421.899	337.991	191.734	4469.109
-63.9	483.008	473.206	421.777	335.316	191.151	4469.109
-63.7	480.513	460.725	425.171	335.880	195.065	4469.109
-63.5	480.005	468.272	432.595	341.769	190.336	4469.109
-63.3	473.354	473.238	410.910	336.732	192.192	4469.109
-63.1	470.287	464.164	418.676	338.581	194.820	4469.109
-62.9	487.990	460.859	435.672	336.851	193.352	4469.109
-62.7	488.525	470.751	422.412	336.851	191.794	4469.109
-62.5	483.752	465.809	426.905	336.301	195.012	4469.109
-62.3	477.301	467.241	417.748	338.399	195.307	4469.109
-62.1	486.847	472.231	420.336	335.913	191.784	4469.109
-61.9	474.451	471.467	425.611	337.872	193.309	4469.109
-61.7	488.498	459.615	424.878	332.641	194.671	4469.109
-61.5	483.834	463.361	424.903	334.030	191.970	4469.109
-61.3	481.829	465.895	430.129	338.962	192.984	4469.109
-61.1	482.028	471.845	446.856	334.266	193.385	4469.109
-60.9	484.805	470.003	439.164	335.518	193.958	4469.109
-60.7	482.201	475.741	420.605	332.927	191.787	4469.109
-60.5	484.505	455.114	430.910	334.415	194.866	4469.109
-60.3	481.502	462.244	441.215	337.003	190.700	4469.109
-60.1	482.790	460.127	411.130	338.574	193.975	4469.109
-59.9	482.310	474.702	434.744	336.301	198.115	4469.109
-59.7	489.387	464.376	419.018	337.428	192.444	4469.109
-59.5	487.963	471.711	426.246	338.574	193.686	4469.109
-59.3	483.652	470.468	426.685	340.957	192.384	4469.109
-59.1	472.237	471.034	423.682	335.051	195.327	4469.109
-58.9	481.193	459.371	426.759	335.011	191.449	4469.109
-58.7	480.994	463.165	412.424	335.008	192.785	4469.109
-58.5	479.841	460.804	452.424	330.855	195.092	4469.109
-58.3	477.646	467.336	424.414	332.983	193.183	4469.109
-58.1	482.119	467.721	421.411	333.281	192.884	4469.109
-57.9	494.904	468.933	425.074	335.081	192.758	4469.109
-57.7	478.226	462.338	419.726	337.865	192.739	4469.109
-57.5	479.451	475.198	414.158	335.996	195.264	4469.109
-57.3	482.854	465.297	426.783	336.778	194.681	4469.109
-57.1	490.022	468.673	418.114	333.589	190.843	4469.109
-56.9	475.940	464.581	425.953	336.118	191.834	4469.109
-56.7	480.812	460.709	419.970	333.454	191.595	4469.109
-56.5	477.582	467.517	432.302	337.172	194.452	4469.109
-56.3	486.910	459.828	442.753	338.216	193.521	4469.109
-56.1	481.248	470.295	435.379	340.043	197.263	4469.109
-55.9	478.226	456.633	414.231	339.661	194.021	4469.109
-55.7	477.401	462.283	418.725	339.864	193.276	4469.109

Midpoint	Ratio: 62	19	6.3	2.1	0.65	Volume
-55.5	484.469	469.067	427.589	334.786	195.506	4469.109
-55.3	491.692	472.246	429.372	335.346	194.074	4469.109
-55.1	487.173	464.959	411.960	339.764	195.042	4469.109
-54.9	471.992	459.175	422.705	336.092	192.987	4469.109
-54.7	472.501	472.026	419.970	340.089	194.303	4469.109
-54.5	477.038	465.919	418.651	337.176	193.789	4469.109
-54.3	490.404	475.449	433.059	336.350	195.178	4469.109
-54.1	485.295	470.877	437.357	337.650	195.175	4469.109
-53.9	489.115	468.351	420.019	336.377	195.519	4469.109
-53.7	489.741	464.180	426.270	330.295	191.661	4469.109
-53.5	488.416	463.086	429.030	335.300	192.437	4469.109
-53.3	487.082	460.324	432.204	337.113	194.638	4469.109
-53.1	476.330	478.440	423.901	339.055	195.622	4469.109
-52.9	484.034	462.204	428.932	337.686	192.145	4469.109
-52.7	493.353	471.971	437.454	336.834	191.509	4469.109
-52.5	468.662	457.372	429.176	336.947	194.025	4469.109
-52.3	484.142	460.937	442.143	336.828	194.131	4469.109
-52.1	477.473	470.617	429.591	334.193	192.294	4469.109
-51.9	484.052	465.148	423.633	339.158	190.445	4469.109
-51.7	492.100	469.421	426.807	335.326	194.389	4469.109
-51.5	487.627	466.533	436.722	334.358	193.935	4469.109
-51.3	481.130	474.143	430.373	337.053	194.661	4469.109
-51.1	479.605	467.336	426.881	336.466	192.268	4469.109
-50.9	479.896	469.405	428.737	335.412	194.485	4469.109
-50.7	472.464	475.953	419.652	335.412	194.303	4469.109
-50.5	466.811	478.298	428.151	337.938	190.839	4469.109
-50.3	475.186	464.927	426.343	336.596	193.000	4469.109
-50.1	481.647	459.702	421.191	337.219	195.195	4469.109
-49.9	486.493	462.086	417.894	335.018	194.515	4469.109
-49.7	487.128	476.945	425.464	332.973	195.035	4469.109
-49.5	481.711	463.102	430.495	336.039	193.723	4469.109
-49.3	489.024	478.857	418.041	336.682	193.368	4469.109
-49.1	482.437	466.517	419.311	343.891	191.887	4469.109
-48.9	490.576	466.415	419.213	335.740	193.080	4469.109
-48.7	480.649	468.681	426.612	335.830	191.877	4469.109
-48.5	490.603	463.794	422.339	339.317	194.797	4469.109
-48.3	489.351	472.097	402.437	333.268	195.851	4469.109
-48.1	468.091	469.681	434.231	336.450	191.207	4469.109
-47.9	478.780	463.172	435.061	335.366	191.936	4469.109
-47.7	474.397	466.470	422.412	333.755	193.024	4469.109
-47.5	475.967	467.548	424.952	337.620	192.026	4469.109
-47.3	478.308	459.584	431.154	337.703	193.435	4469.109
-47.1	490.776	461.142	415.306	336.367	195.586	4469.109
-46.9	493.498	466.793	423.926	336.039	194.111	4469.109
-46.7	481.910	463.369	425.489	333.861	194.339	4469.109

Midpoint	Ratio: 62	19	6.3	2.1	0.65	Volume
-46.5	486.665	480.628	418.651	334.948	194.220	4469.109
-46.3	480.958	475.693	439.970	335.442	194.866	4469.109
-46.1	498.688	471.436	444.170	338.849	194.502	4469.109
-45.9	479.896	461.874	422.754	338.044	193.116	4469.109
-45.7	479.342	472.553	434.866	335.376	190.909	4469.109
-45.5	486.901	463.700	417.577	336.330	193.113	4469.109
-45.3	491.429	467.060	436.478	333.735	193.458	4469.109
-45.1	472.718	462.850	430.080	334.882	193.289	4469.109
-44.9	483.190	463.188	431.105	335.379	193.348	4469.109
-44.7	477.119	470.271	439.530	342.456	192.589	4469.109
-44.5	490.231	463.621	421.264	337.994	193.083	4469.109
-44.3	484.397	467.611	433.743	334.637	195.519	4469.109
-44.1	488.407	474.119	417.894	334.643	191.820	4469.109
-43.9	485.948	469.901	428.688	333.023	196.925	4469.109
-43.7	488.208	462.503	435.696	336.864	192.520	4469.109
-43.5	484.932	469.940	431.496	340.314	194.091	4469.109
-43.3	482.591	469.626	430.519	336.715	192.347	4469.109
-43.1	480.921	473.057	424.610	337.020	192.493	4469.109
-42.9	475.686	461.071	421.533	340.778	192.642	4469.109
-42.7	480.958	467.800	416.307	335.734	193.488	4469.109
-42.5	488.725	469.508	430.202	334.899	193.408	4469.109
-42.3	475.803	468.083	412.546	333.284	193.932	4469.109
-42.1	487.173	463.566	431.520	337.109	192.291	4469.109
-41.9	482.772	470.554	436.966	338.313	195.231	4469.109
-41.7	482.682	470.413	421.411	332.655	195.791	4469.109
-41.5	482.881	465.344	423.950	335.654	196.875	4469.109
-41.3	488.843	462.661	414.598	335.986	192.543	4469.109
-41.1	482.908	476.291	430.861	335.124	191.502	4469.109
-40.9	484.995	472.931	412.473	335.071	194.704	4469.109
-40.7	479.079	475.087	422.021	337.616	192.235	4469.109
-40.5	483.144	473.443	426.099	334.624	192.241	4469.109
-40.3	495.857	474.883	408.590	336.453	195.417	4469.109
-40.1	486.411	460.819	431.130	338.339	192.437	4469.109
-39.9	485.939	458.395	439.799	337.895	194.598	4469.109
-39.7	486.302	462.204	432.277	336.791	191.158	4469.109
-39.5	490.385	474.749	431.203	336.513	193.030	4469.109
-39.3	480.413	472.750	445.122	337.729	192.410	4469.109
-39.1	480.994	459.411	428.761	339.555	192.384	4469.109
-38.9	480.304	469.697	422.778	335.949	192.782	4469.109
-38.7	481.221	465.533	425.416	335.989	192.430	4469.109
-38.5	485.658	470.515	419.872	339.897	191.963	4469.109
-38.3	482.727	463.645	414.769	336.496	194.134	4469.109
-38.1	478.362	466.698	426.026	338.352	194.774	4469.109
-37.9	484.406	468.351	421.240	336.559	194.217	4469.109
-37.7	493.625	465.793	418.480	338.979	194.263	4469.109

Midpoint	Ratio: 62	19	6.3	2.1	0.65	Volume
-37.5	476.974	468.264	433.327	339.648	193.673	4469.109
-37.3	487.808	468.382	426.173	336.092	194.794	4469.109
-37.1	487.082	471.633	424.927	337.766	192.029	4469.109
-36.9	489.460	472.577	430.348	338.999	193.322	4469.109
-36.7	479.460	464.093	433.157	333.321	193.862	4469.109
-36.5	472.945	470.539	420.214	336.383	194.144	4469.109
-36.3	490.059	472.679	417.406	338.322	193.600	4469.109
-36.1	488.943	465.360	428.175	333.062	194.326	4469.109
-35.9	481.121	467.839	429.909	336.327	191.817	4469.109
-35.7	490.993	480.903	426.490	338.319	192.918	4469.109
-35.5	485.204	475.866	424.463	338.356	192.659	4469.109
-35.3	486.620	477.110	427.003	338.041	193.759	4469.109
-35.1	483.426	469.185	440.116	337.123	193.547	4469.109
-34.9	479.370	465.581	415.184	334.534	195.811	4469.109
-34.7	476.012	461.693	427.491	333.009	193.488	4469.109
-34.5	481.602	463.723	422.949	335.936	192.487	4469.109
-34.3	484.660	465.179	422.851	338.657	192.410	4469.109
-34.1	495.004	464.550	432.155	339.370	195.496	4469.109
-33.9	490.794	456.522	433.230	340.215	193.020	4469.109
-33.7	482.237	469.523	436.429	335.329	190.942	4469.109
-33.5	494.369	465.541	425.025	335.817	194.359	4469.109
-33.3	493.688	468.634	426.930	338.813	193.567	4469.109
-33.1	483.262	467.776	425.513	337.040	193.143	4469.109
-32.9	482.418	470.586	415.379	335.276	192.772	4469.109
-32.7	481.021	463.007	440.922	338.717	192.043	4469.109
-32.5	473.417	463.818	423.144	337.159	194.847	4469.109
-32.3	485.839	473.537	409.250	333.891	194.422	4469.109
-32.1	477.283	469.098	429.030	334.759	192.758	4469.109
-31.9	471.802	468.658	427.271	337.494	189.709	4469.109
-31.7	467.655	465.014	420.361	334.365	187.996	4469.109
-31.5	479.841	476.410	418.773	336.500	192.838	4469.109
-31.3	482.155	462.991	406.759	338.276	192.583	4469.109
-31.1	482.609	463.046	426.221	336.592	191.303	4469.109
-30.9	484.524	471.829	425.611	332.393	193.126	4469.109
-30.7	485.095	469.106	429.078	333.752	192.573	4469.109
-30.5	483.970	459.135	437.357	338.094	194.081	4469.109
-30.3	480.404	465.108	411.911	335.525	192.666	4469.109
-30.1	481.039	460.945	418.431	335.532	192.493	4469.109
-29.9	480.713	456.066	426.832	332.111	193.484	4469.109
-29.7	470.840	461.473	431.643	335.668	193.554	4469.109
-29.5	479.360	468.406	411.545	336.917	190.147	4469.109
-29.3	487.382	467.477	419.482	333.314	191.509	4469.109
-29.1	483.045	463.637	422.827	334.358	190.717	4469.109
-28.9	479.569	472.199	432.375	332.002	190.849	4469.109
-28.7	493.815	462.378	415.379	335.532	189.520	4469.109

Midpoint	Ratio: 62	19	6.3	2.1	0.65	Volume
-28.5	480.395	464.778	423.462	335.154	193.000	4469.109
-28.3	483.907	473.828	413.816	329.963	192.613	4469.109
-28.1	479.805	470.098	420.385	337.653	191.744	4469.109
-27.9	472.011	454.169	431.301	331.564	192.238	4469.109
-27.7	482.963	464.479	425.538	330.136	190.866	4469.109
-27.5	474.615	458.411	438.529	334.285	191.648	4469.109
-27.3	474.143	469.154	417.845	335.213	191.953	4469.109
-27.1	481.066	477.645	416.576	331.435	190.180	4469.109
-26.9	476.783	461.221	421.069	332.549	190.299	4469.109
-26.7	482.654	459.371	424.048	330.451	190.395	4469.109
-26.5	488.843	472.388	426.881	335.502	191.907	4469.109
-26.3	477.074	464.274	412.742	330.437	192.328	4469.109
-26.1	479.624	462.315	415.452	333.742	191.695	4469.109
-25.9	485.358	456.436	432.155	336.321	193.179	4469.109
-25.7	479.660	466.202	426.710	333.417	190.070	4469.109
-25.5	490.975	460.741	435.794	331.193	191.128	4469.109
-25.3	483.262	467.997	419.140	336.983	193.899	4469.109
-25.1	481.239	469.500	415.526	339.721	190.965	4469.109
-24.9	483.761	470.255	440.141	334.024	191.313	4469.109
-24.7	482.582	475.748	442.363	334.458	191.479	4469.109
-24.5	476.911	484.209	432.253	336.297	191.068	4469.109
-24.3	488.434	470.129	427.418	340.016	193.909	4469.109
-24.1	493.761	476.488	436.966	343.483	191.512	4469.109
-23.9	488.208	475.111	413.425	342.993	193.067	4469.109
-23.7	480.812	472.018	426.392	344.680	193.077	4469.109
-23.5	496.737	481.722	429.860	341.402	196.603	4469.109
-23.3	495.830	479.243	435.965	344.756	197.498	4469.109
-23.1	505.457	475.504	432.522	343.520	199.135	4469.109
-22.9	495.666	480.163	434.207	347.994	195.612	4469.109
-22.7	497.617	487.970	429.396	346.602	201.187	4469.109
-22.5	504.985	484.295	442.729	344.620	199.304	4469.109
-22.3	496.465	486.192	439.530	349.025	197.883	4469.109
-22.1	499.559	490.182	446.880	355.256	199.536	4469.109
-21.9	508.787	482.847	430.763	357.480	200.885	4469.109
-21.7	506.664	488.797	454.621	352.886	202.838	4469.109
-21.5	508.170	495.730	456.233	357.135	202.102	4469.109
-21.3	508.497	492.771	443.340	359.757	205.347	4469.109
-21.1	506.138	497.076	440.434	358.411	202.645	4469.109
-20.9	518.959	504.442	456.306	359.946	206.666	4469.109
-20.7	529.131	510.431	460.287	361.775	209.175	4469.109
-20.5	527.634	508.487	468.199	362.856	210.358	4469.109
-20.3	527.044	502.380	473.693	365.806	207.886	4469.109
-20.1	538.577	512.650	471.837	366.681	209.755	4469.109
-19.9	535.102	516.601	471.227	369.624	210.464	4469.109
-19.7	533.487	529.862	479.774	365.822	211.422	4469.109

Midpoint	Ratio: 62	19	6.3	2.1	0.65	Volume
-19.5	542.915	527.894	473.425	368.736	214.352	4469.109
-19.3	546.263	532.923	491.227	377.330	213.789	4469.109
-19.1	552.969	525.108	485.366	382.255	217.232	4469.109
-18.9	553.504	533.615	497.014	384.228	216.135	4469.109
-18.7	552.696	522.275	474.011	376.677	215.658	4469.109
-18.5	35.262	33.376	35.213	25.127	14.282	12329.366
-18.3	31.450	31.826	28.205	21.998	12.850	186.603
-18.1	27.095	27.316	28.644	19.525	11.657	160.940
-17.9	24.899	23.554	19.462	17.891	9.582	145.255
-17.7	23.366	22.909	22.320	17.351	9.566	133.279
-17.5	21.995	21.910	20.293	15.478	8.250	123.465
-17.3	21.151	23.248	20.342	16.198	8.588	115.116
-17.1	19.936	22.343	20.903	15.034	7.958	107.844
-16.9	19.400	21.548	15.116	14.948	7.206	101.407
-16.7	19.799	17.463	16.215	13.884	7.212	95.641
-16.5	17.685	16.826	15.384	13.076	7.216	90.430
-16.3	17.331	15.881	12.698	11.872	6.712	85.685
-16.1	16.742	17.589	16.337	11.915	6.692	81.341
-15.9	16.614	16.558	17.631	12.326	6.576	77.345
-15.7	17.077	17.306	12.259	11.402	6.480	73.655
-15.5	18.475	17.156	12.942	11.965	6.984	70.235
-15.3	16.524	16.826	15.213	11.160	5.601	67.056
-15.1	16.161	15.811	13.382	10.928	5.399	64.095
-14.9	15.517	15.378	13.333	10.679	5.625	61.330
-14.7	15.108	14.701	13.113	10.699	5.134	58.744
-14.5	15.199	14.103	13.187	10.427	5.190	56.321
-14.3	14.028	14.473	12.552	10.036	5.137	54.048
-14.1	14.518	14.000	12.234	9.337	5.015	51.912
-13.9	14.981	13.528	12.698	9.698	5.141	49.904
-13.7	14.400	13.615	11.966	9.705	5.031	48.013
-13.5	13.620	13.552	12.478	9.320	4.915	46.231
-13.3	13.965	13.906	11.526	9.367	4.958	44.551
-13.1	13.802	13.701	12.527	9.516	4.657	42.966
-12.9	14.047	13.764	11.697	9.257	4.809	41.470
-12.7	13.965	12.922	11.721	9.029	4.859	40.057
-12.5	13.774	13.095	12.625	8.906	5.031	38.722
-12.3	13.665	13.245	11.697	9.125	4.932	37.461
-12.1	13.874	12.867	11.453	9.158	4.998	36.268
-11.9	13.375	13.292	12.039	9.340	4.975	35.141
-11.7	13.520	13.387	11.746	9.217	4.972	34.076
-11.5	13.919	13.497	12.039	9.158	4.819	33.068
-11.3	14.527	13.056	12.430	9.380	5.008	32.116
-11.1	13.811	13.269	12.283	9.347	5.293	31.217
-10.9	14.546	13.733	13.065	10.308	5.492	30.367
-10.7	15.272	14.205	12.942	10.262	5.555	29.564

Midpoint	Ratio: 62	19	6.3	2.1	0.65	Volume
-10.5	14.872	14.717	13.309	9.986	5.386	28.806
-10.3	15.199	14.740	13.406	10.371	5.326	28.092
-10.1	15.988	15.307	12.820	10.858	5.747	27.418
-9.9	16.297	15.677	15.311	10.861	5.780	26.784
-9.7	17.295	15.944	14.920	11.342	5.751	26.188
-9.5	16.977	17.078	13.895	11.717	6.602	25.627
-9.3	18.329	17.802	17.069	12.469	6.596	25.101
-9.1	19.346	18.644	16.654	13.138	6.834	24.608
-8.9	20.498	19.187	17.851	13.526	7.037	24.147
-8.7	20.562	20.249	18.559	14.166	7.623	23.717
-8.5	21.959	21.996	20.244	14.832	8.293	23.317
-8.3	22.540	22.358	20.293	15.465	8.369	22.946
-8.1	24.101	22.201	21.611	16.755	8.594	22.603
-7.9	24.064	23.916	21.611	16.440	8.922	22.286
-7.7	23.774	22.547	20.488	15.986	8.329	21.996
-7.5	23.166	21.548	19.756	15.296	8.502	21.732
-7.3	20.562	21.013	18.437	14.471	7.527	21.493
-7.1	18.529	18.455	16.825	12.956	6.911	21.278
-6.9	15.943	16.204	16.166	10.987	5.996	21.087
-6.7	13.094	13.450	11.673	9.241	4.985	20.919
-6.5	10.880	10.491	9.426	7.295	4.120	20.775
-6.3	7.831	8.271	6.886	5.522	3.076	20.654
-6.1	5.862	5.950	4.835	3.994	2.217	20.554
-5.9	3.911	3.974	3.883	2.691	1.551	20.478
-5.7	2.396	2.298	1.783	1.684	1.044	20.423
-5.5	1.506	1.330	1.099	0.875	0.494	20.390
-5.3	0.735	0.740	0.684	0.494	0.242	20.379
-5.1	0.508	0.441	0.122	0.239	0.123	20.378
-4.9	0.318	0.197	0.220	0.136	0.076	20.378
-4.7	0.209	0.181	0.147	0.109	0.060	20.378
-4.5	0.064	0.079	0.098	0.066	0.033	20.378
-4.3	0.064	0.016	0.049	0.013	0.030	20.378
-4.1	0.036	0.031	0.000	0.017	0.010	20.378
-3.9	0.009	0.039	0.024	0.013	0.007	20.378
-3.7	0.018	0.024	0.000	0.007	0.007	20.378
-3.5	0.018	0.008	0.000	0.010	0.007	20.378
-3.3	0.000	0.016	0.000	0.000	0.003	20.378
-3.1	0.018	0.000	0.024	0.007	0.000	20.378
-2.9	0.018	0.000	0.024	0.010	0.003	20.378
-2.7	0.000	0.000	0.000	0.017	0.013	20.378
-2.5	0.036	0.008	0.000	0.007	0.007	20.378
-2.3	0.009	0.016	0.024	0.023	0.013	20.378
-2.1	0.027	0.008	0.000	0.017	0.007	20.378
-1.9	0.027	0.031	0.049	0.020	0.007	20.378
-1.7	0.091	0.024	0.000	0.046	0.013	20.378

Midpoint	Ratio: 62	19	6.3	2.1	0.65	Volume
-1.5	0.100	0.087	0.049	0.030	0.033	20.378
-1.3	0.172	0.102	0.073	0.083	0.046	20.378
-1.1	0.345	0.205	0.415	0.169	0.080	20.378
-0.9	0.327	0.291	0.195	0.182	0.119	20.378
-0.7	0.472	0.504	0.635	0.345	0.172	20.378
-0.5	0.771	0.693	0.855	0.534	0.272	20.378
-0.1	1.143	0.826	0.830	0.653	0.401	20.378
0.1	1.388	1.259	1.319	0.862	0.504	20.378
0.5	1.751	1.668	1.758	1.157	0.603	20.378
0.7	1.978	1.889	1.514	1.515	0.699	20.378
0.9	2.042	1.983	1.636	1.425	0.832	20.378
1.1	2.332	2.306	2.051	1.617	0.895	20.378
1.3	2.396	2.440	2.198	1.594	0.822	20.378
1.5	2.014	2.219	1.661	1.309	0.782	20.378
1.7	2.132	1.983	1.905	1.319	0.689	20.378
1.9	1.906	1.692	1.612	1.167	0.620	20.378
2.1	1.597	1.629	1.538	1.104	0.577	20.378
2.3	1.524	1.401	1.343	1.001	0.471	20.378
2.5	1.452	1.275	1.074	0.875	0.461	20.378
2.7	1.125	1.118	0.733	0.772	0.361	20.378
2.9	1.053	0.929	0.855	0.686	0.358	20.378
3.1	1.025	0.913	0.806	0.613	0.305	20.378
3.3	1.062	0.866	0.659	0.636	0.361	20.378
3.5	1.016	0.984	0.635	0.762	0.302	20.378
3.7	0.926	1.133	0.977	0.636	0.378	20.378
3.9	1.034	1.110	0.879	0.749	0.421	20.378
4.1	1.107	1.220	0.952	0.792	0.375	20.378
4.3	1.570	1.291	1.441	0.905	0.474	20.378
4.5	1.633	1.550	1.612	1.004	0.577	20.378
4.7	1.942	1.558	1.563	1.303	0.607	20.378
4.9	2.096	2.141	1.905	1.521	0.786	20.378
5.1	2.713	2.432	2.418	1.634	0.868	20.378
5.3	3.031	3.006	2.540	1.949	1.110	20.379
5.5	4.337	4.273	3.052	2.724	1.601	20.390
5.7	5.807	5.407	5.397	3.928	2.350	20.423
5.9	7.050	6.674	6.032	4.644	2.602	20.478
6.1	8.212	7.665	6.886	5.383	3.053	20.554
6.3	8.992	9.066	7.619	6.201	3.550	20.654
6.5	10.535	10.648	8.693	7.116	3.901	20.775
6.7	11.479	11.183	9.743	8.054	4.398	20.919
6.9	13.402	12.332	11.453	8.747	4.730	21.087
7.1	14.246	13.347	12.552	9.430	5.157	21.278
7.3	14.827	14.142	12.967	9.844	5.399	21.493
7.5	15.671	14.347	13.138	10.175	5.595	21.732
7.7	15.834	15.149	15.091	10.712	5.353	21.996

Midpoint	Ratio: 62	19	6.3	2.1	0.65	Volume
7.9	15.634	16.141	13.016	10.490	5.731	22.286
8.1	15.063	14.795	13.968	10.716	5.810	22.603
8.3	15.535	14.843	13.675	10.361	5.552	22.946
8.5	15.462	14.481	11.770	10.248	5.419	23.317
8.7	14.464	13.316	11.868	9.930	5.190	23.717
8.9	14.228	13.040	11.404	9.327	4.988	24.147
9.1	14.037	13.269	12.503	9.267	4.985	24.608
9.3	12.940	13.206	11.526	8.866	4.663	25.101
9.5	12.540	12.836	10.940	8.833	4.617	25.627
9.7	12.740	11.946	10.476	8.601	4.541	26.188
9.9	12.377	12.222	10.940	8.150	4.504	26.784
10.1	12.595	11.262	10.427	8.296	4.342	27.418
10.3	11.742	11.640	9.866	8.018	4.269	28.092
10.5	12.159	11.640	11.184	7.849	4.180	28.806
10.7	12.059	11.640	9.279	7.782	4.126	29.564
10.9	10.744	11.026	10.281	7.822	3.954	30.367
11.1	10.898	11.388	9.768	7.769	4.242	31.217
11.3	10.771	10.656	10.110	7.832	4.040	32.116
11.5	11.606	10.876	10.183	7.782	3.928	33.068
11.7	10.771	11.136	9.743	7.570	3.901	34.076
11.9	11.696	11.521	9.963	7.583	4.160	35.141
12.1	11.542	10.797	9.841	7.782	4.123	36.268
12.3	11.442	11.010	10.110	7.613	4.047	37.461
12.5	11.633	10.837	9.914	7.825	3.964	38.722
12.7	12.123	11.325	10.671	7.948	4.272	40.057
12.9	11.288	11.498	10.671	7.789	4.010	41.470
13.1	12.059	11.923	10.256	7.799	4.100	42.966
13.3	12.558	11.372	11.697	8.409	4.322	44.551
13.5	11.742	12.663	11.380	8.458	4.166	46.231
13.7	12.223	11.946	11.819	8.604	4.282	48.013
13.9	12.540	12.820	10.769	8.674	4.392	49.904
14.1	12.577	12.198	11.624	8.651	4.375	51.912
14.3	13.520	12.458	11.111	9.029	4.909	54.048
14.5	13.611	13.379	12.747	9.161	4.730	56.321
14.7	14.409	13.875	12.649	9.483	4.528	58.744
14.9	14.491	14.292	12.772	9.347	4.958	61.330
15.1	14.564	13.953	13.748	9.963	4.995	64.095
15.3	15.798	14.032	12.772	10.039	5.194	67.056
15.5	15.752	15.024	16.996	10.716	5.638	70.235
15.7	15.163	15.488	13.382	11.796	5.946	73.655
15.9	14.428	12.891	10.305	9.953	5.893	77.345
16.1	14.419	15.795	13.089	10.361	6.682	81.341
16.3	18.230	13.623	17.704	11.780	6.649	85.685
16.5	17.095	15.692	16.288	12.290	7.159	90.430
16.7	18.094	19.659	18.510	12.423	7.617	95.641

Midpoint	Ratio: 62	19	6.3	2.1	0.65	Volume
16.9	20.435	15.134	15.604	12.883	7.792	101.407
17.1	19.827	18.219	15.824	13.970	7.580	107.844
17.3	21.251	20.650	18.510	14.607	8.488	115.116
17.5	20.852	19.509	17.240	14.212	8.601	123.465
17.7	21.251	22.665	22.271	15.571	9.453	133.279
17.9	26.505	24.743	22.661	18.236	10.298	145.255
18.1	26.614	29.740	24.469	19.144	10.358	160.940
18.3	31.124	29.638	27.716	20.765	12.048	186.603
18.5	38.619	35.406	31.794	23.456	13.861	12329.366
18.7	528.632	524.746	458.260	373.204	213.361	4469.109
18.9	550.945	536.645	487.466	385.242	217.647	4469.109
19.1	540.483	532.427	487.002	377.367	215.694	4469.109
19.3	548.804	516.743	493.034	374.549	214.405	4469.109
19.5	531.908	519.969	479.285	371.401	213.358	4469.109
19.7	544.521	505.929	471.911	374.954	212.201	4469.109
19.9	528.786	517.333	466.953	364.573	210.083	4469.109
20.1	531.754	513.020	454.353	366.429	212.542	4469.109
20.3	514.540	510.785	461.044	360.857	208.847	4469.109
20.5	523.442	503.403	451.667	364.165	206.036	4469.109
20.7	520.048	494.967	454.304	362.800	208.008	4469.109
20.9	518.043	492.661	465.000	357.390	206.083	4469.109
21.1	512.898	499.476	466.294	360.721	203.554	4469.109
21.3	520.230	483.248	469.786	359.558	203.007	4469.109
21.5	506.546	481.391	430.886	360.778	201.611	4469.109
21.7	510.874	491.952	441.606	353.499	199.739	4469.109
21.9	511.845	500.625	450.275	347.523	198.708	4469.109
22.1	498.507	484.004	450.861	351.007	199.215	4469.109
22.3	503.906	481.950	435.208	344.875	198.990	4469.109
22.5	500.911	477.614	439.579	350.437	197.491	4469.109
22.7	491.374	483.075	433.279	343.307	199.573	4469.109
22.9	498.688	481.336	447.149	346.887	198.903	4469.109
23.1	476.293	477.732	419.091	347.411	198.052	4469.109
23.3	497.663	480.533	427.882	339.658	195.529	4469.109
23.5	491.492	478.739	431.130	345.008	194.856	4469.109
23.7	481.402	477.149	430.226	341.160	193.899	4469.109
23.9	485.032	466.958	429.738	336.108	196.689	4469.109
24.1	480.631	471.617	425.122	335.482	193.150	4469.109
24.3	483.662	472.742	428.077	331.312	192.357	4469.109
24.5	475.831	473.742	420.312	339.357	193.319	4469.109
24.7	481.874	462.543	425.416	335.462	190.657	4469.109
24.9	488.244	469.350	423.608	336.526	191.250	4469.109
25.1	485.222	464.652	429.005	335.081	190.816	4469.109
25.3	481.003	460.001	440.116	334.140	191.337	4469.109
25.5	470.704	464.235	445.562	333.344	192.785	4469.109
25.7	475.894	462.645	405.587	333.364	190.180	4469.109

Midpoint	Ratio: 62	19	6.3	2.1	0.65	Volume
25.9	476.566	482.816	435.379	337.974	192.444	4469.109
26.1	477.945	464.841	419.872	336.586	192.255	4469.109
26.3	476.693	465.093	427.467	329.596	189.812	4469.109
26.5	477.237	457.797	408.957	330.865	191.270	4469.109
26.7	470.432	472.128	418.725	333.881	189.053	4469.109
26.9	479.642	469.476	421.875	329.576	192.291	4469.109
27.1	486.175	463.409	429.860	331.445	191.472	4469.109
27.3	472.827	464.959	417.577	330.703	187.820	4469.109
27.5	485.830	456.932	425.611	334.166	190.077	4469.109
27.7	480.912	466.848	424.683	332.254	190.541	4469.109
27.9	489.342	461.756	424.219	328.439	194.104	4469.109
28.1	477.809	456.475	427.271	334.683	191.293	4469.109
28.3	477.582	464.652	431.960	337.686	191.953	4469.109
28.5	475.413	473.537	420.214	332.307	190.952	4469.109
28.7	478.517	468.083	417.821	333.139	191.479	4469.109
28.9	468.635	469.043	405.977	334.163	192.331	4469.109
29.1	495.558	471.318	427.540	339.270	193.965	4469.109
29.3	484.515	469.390	423.731	339.274	192.984	4469.109
29.5	486.801	467.155	423.755	335.996	191.224	4469.109
29.7	477.736	471.302	422.534	335.220	191.936	4469.109
29.9	480.313	468.949	420.116	332.701	191.870	4469.109
30.1	481.910	464.935	429.249	333.758	193.803	4469.109
30.3	481.983	465.171	414.598	335.674	191.767	4469.109
30.5	481.375	465.038	422.314	336.035	190.843	4469.109
30.7	473.844	474.702	421.508	338.525	192.745	4469.109
30.9	489.342	471.971	417.479	334.063	194.803	4469.109
31.1	476.938	470.869	412.742	337.829	192.947	4469.109
31.3	483.045	455.413	423.608	334.398	194.697	4469.109
31.5	484.369	464.038	420.385	335.044	193.574	4469.109
31.7	485.857	469.397	414.475	336.973	193.527	4469.109
31.9	484.850	463.212	431.545	331.783	194.797	4469.109
32.1	475.014	465.337	421.850	337.036	195.741	4469.109
32.3	481.302	457.128	431.984	334.978	191.585	4469.109
32.5	490.748	467.721	441.557	335.104	195.271	4469.109
32.7	483.616	464.849	435.208	338.419	195.175	4469.109
32.9	472.519	474.151	433.108	339.748	193.700	4469.109
33.1	484.678	471.121	428.273	338.200	193.836	4469.109
33.3	482.437	465.226	428.859	338.883	191.698	4469.109
33.5	487.990	472.388	442.851	337.063	194.890	4469.109
33.7	479.624	463.708	431.667	332.748	193.063	4469.109
33.9	492.345	464.967	432.155	339.506	194.648	4469.109
34.1	475.686	470.200	418.431	335.949	193.063	4469.109
34.3	479.370	464.967	426.661	338.134	193.206	4469.109
34.5	484.378	461.134	420.116	337.388	193.259	4469.109
34.7	485.857	465.573	428.151	342.449	193.498	4469.109

Midpoint	Ratio: 62	19	6.3	2.1	0.65	Volume
34.9	478.734	467.886	427.003	337.229	193.040	4469.109
35.1	489.469	476.685	441.362	335.731	194.684	4469.109
35.3	486.656	475.371	439.115	339.294	194.373	4469.109
35.5	485.077	465.455	407.491	338.879	194.320	4469.109
35.7	477.782	467.430	430.422	336.466	193.726	4469.109
35.9	482.691	461.732	420.873	337.945	193.614	4469.109
36.1	474.388	467.981	435.281	339.715	193.319	4469.109
36.3	483.562	468.272	425.904	335.051	193.053	4469.109
36.5	479.243	464.502	422.949	340.450	193.266	4469.109
36.7	483.389	464.093	402.021	338.210	193.411	4469.109
36.9	483.907	467.225	429.738	334.557	194.750	4469.109
37.1	478.880	465.195	422.412	339.847	195.553	4469.109
37.3	479.878	461.229	441.020	340.255	192.709	4469.109
37.5	478.480	463.865	428.614	339.575	193.511	4469.109
37.7	475.386	462.047	424.341	336.251	194.502	4469.109
37.9	478.480	464.353	423.999	336.098	193.521	4469.109
38.1	479.578	469.657	426.954	336.788	194.276	4469.109
38.3	478.099	466.509	421.875	340.082	189.587	4469.109
38.5	487.890	470.247	423.242	335.220	193.991	4469.109
38.7	483.897	472.955	425.977	334.517	193.365	4469.109
38.9	487.464	464.927	434.671	334.981	193.607	4469.109
39.1	483.290	462.692	421.484	339.426	193.567	4469.109
39.3	492.699	474.332	438.895	335.028	192.699	4469.109
39.5	474.424	467.005	429.176	332.237	195.549	4469.109
39.7	498.443	466.745	429.494	338.412	192.497	4469.109
39.9	488.752	467.438	426.148	334.809	193.289	4469.109
40.1	482.790	467.886	435.965	334.799	190.070	4469.109
40.3	478.653	469.563	422.558	333.540	191.685	4469.109
40.5	486.030	459.812	413.132	338.730	193.942	4469.109
40.7	484.442	471.656	440.629	341.345	191.751	4469.109
40.9	471.049	476.913	421.582	331.783	194.035	4469.109
41.1	487.228	475.064	410.544	335.412	195.344	4469.109
41.3	484.306	467.265	432.986	338.359	195.291	4469.109
41.5	471.466	466.336	409.933	336.755	194.237	4469.109
41.7	494.850	471.585	428.614	336.619	196.590	4469.109
41.9	475.422	463.401	425.806	337.285	192.981	4469.109
42.1	486.130	465.738	427.149	338.402	195.085	4469.109
42.3	478.172	470.468	416.014	336.553	193.176	4469.109
42.5	484.170	464.597	432.693	339.141	195.185	4469.109
42.7	478.199	463.306	415.819	341.975	192.752	4469.109
42.9	478.299	459.962	433.718	334.093	194.585	4469.109
43.1	487.681	467.399	416.576	336.702	193.173	4469.109
43.3	493.144	460.638	436.526	333.281	194.691	4469.109
43.5	479.841	466.061	435.428	340.570	192.865	4469.109
43.7	483.244	465.022	441.166	338.027	196.636	4469.109

Midpoint	Ratio: 62	19	6.3	2.1	0.65	Volume
43.9	484.623	457.986	428.419	336.877	193.236	4469.109
44.1	487.591	466.297	425.879	339.204	192.066	4469.109
44.3	481.039	468.233	435.794	338.395	196.308	4469.109
44.5	476.511	462.204	423.193	341.233	192.069	4469.109
44.7	476.557	464.628	438.920	336.340	193.607	4469.109
44.9	481.965	463.629	427.296	337.487	191.668	4469.109
45.1	487.536	460.623	419.066	334.150	193.544	4469.109
45.3	481.393	475.253	411.350	337.510	192.871	4469.109
45.5	482.083	463.172	422.070	337.143	194.074	4469.109
45.7	487.727	473.655	418.822	336.874	194.611	4469.109
45.9	479.941	464.408	428.639	333.742	191.356	4469.109
46.1	482.192	476.197	424.317	336.602	192.338	4469.109
46.3	491.574	468.673	419.482	335.525	193.620	4469.109
46.5	479.596	466.572	432.741	334.998	194.329	4469.109
46.7	482.999	463.165	418.236	340.991	196.152	4469.109
46.9	485.431	467.855	421.069	335.532	193.819	4469.109
47.1	487.464	466.643	428.614	334.726	192.599	4469.109
47.3	485.104	467.005	419.970	338.558	194.522	4469.109
47.5	481.956	461.953	430.959	340.069	193.922	4469.109
47.7	484.351	463.802	430.886	332.950	193.882	4469.109
47.9	479.478	470.515	423.804	332.817	195.785	4469.109
48.1	478.244	466.832	410.446	337.285	195.006	4469.109
48.3	481.629	476.968	427.735	338.936	194.697	4469.109
48.5	486.130	472.931	435.623	335.300	191.651	4469.109
48.7	484.088	469.783	416.893	340.613	190.359	4469.109
48.9	475.250	469.358	414.622	333.520	193.123	4469.109
49.1	481.638	466.391	414.720	337.875	195.138	4469.109
49.3	483.770	466.588	429.005	341.325	192.444	4469.109
49.5	486.375	471.192	432.741	336.539	194.210	4469.109
49.7	476.629	477.590	412.717	334.865	194.210	4469.109
49.9	479.805	458.498	432.693	338.667	192.235	4469.109
50.1	481.348	468.091	424.878	336.801	196.033	4469.109
50.3	475.921	471.420	434.182	340.599	192.026	4469.109
50.5	478.970	460.355	430.666	334.504	192.868	4469.109
50.7	494.196	467.107	426.075	334.955	192.967	4469.109
50.9	491.256	470.295	420.312	336.523	192.619	4469.109
51.1	480.576	465.966	422.827	336.586	194.154	4469.109
51.3	488.943	459.080	419.188	336.549	194.581	4469.109
51.5	475.785	470.806	416.478	339.986	193.902	4469.109
51.7	485.068	470.499	425.831	337.739	197.153	4469.109
51.9	483.471	465.352	426.392	336.367	192.805	4469.109
52.1	474.279	464.888	436.380	336.324	195.354	4469.109
52.3	480.731	466.879	419.897	336.032	191.913	4469.109
52.5	482.282	467.910	436.917	336.168	191.983	4469.109
52.7	479.497	465.714	422.192	336.705	194.565	4469.109

Midpoint	Ratio: 62	19	6.3	2.1	0.65	Volume
52.9	489.324	470.916	419.091	332.877	194.568	4469.109
53.1	475.141	467.855	425.269	338.770	191.927	4469.109
53.3	480.032	473.120	430.495	339.386	195.794	4469.109
53.5	483.507	461.087	431.032	334.014	195.519	4469.109
53.7	483.344	475.811	437.406	339.055	191.333	4469.109
53.9	490.013	477.409	421.289	338.356	196.305	4469.109
54.1	493.325	461.213	420.727	335.489	191.943	4469.109
54.3	474.696	475.481	431.887	336.828	194.489	4469.109
54.5	501.365	467.808	422.851	337.537	192.354	4469.109
54.7	481.747	473.498	422.534	336.178	193.604	4469.109
54.9	487.591	461.882	419.628	333.944	194.794	4469.109
55.1	491.302	467.060	433.010	336.055	193.435	4469.109
55.3	489.224	469.563	434.817	334.746	194.177	4469.109
55.5	478.662	471.774	430.910	336.602	193.451	4469.109
55.7	486.438	459.962	418.895	335.068	193.832	4469.109
55.9	481.511	466.887	422.192	337.205	192.510	4469.109
56.1	481.538	462.511	437.625	339.913	194.482	4469.109
56.3	479.125	466.383	426.612	334.653	194.833	4469.109
56.5	486.656	469.051	426.588	334.564	193.156	4469.109
56.7	497.826	468.673	419.213	337.736	192.235	4469.109
56.9	480.658	470.775	420.825	334.355	193.408	4469.109
57.1	476.475	469.571	414.060	339.423	194.081	4469.109
57.3	483.516	468.823	413.157	336.682	195.543	4469.109
57.5	487.246	464.274	422.168	339.754	193.842	4469.109
57.7	486.602	465.746	426.881	339.592	194.180	4469.109
57.9	486.420	464.778	421.118	331.909	191.777	4469.109
58.1	475.023	477.291	425.000	334.730	194.757	4469.109
58.3	478.979	466.328	419.311	339.983	193.544	4469.109
58.5	473.943	457.388	426.099	339.131	192.056	4469.109
58.7	487.391	468.414	435.623	338.733	191.880	4469.109
58.9	473.245	475.481	429.274	334.577	193.236	4469.109
59.1	480.295	486.097	404.756	337.567	192.914	4469.109
59.3	480.322	463.243	422.461	334.527	193.955	4469.109
59.5	477.963	473.167	418.602	335.356	193.166	4469.109
59.7	469.987	461.583	429.103	335.452	193.150	4469.109
59.9	488.008	457.412	424.927	331.534	195.387	4469.109
60.1	473.299	466.462	449.615	336.440	193.213	4469.109
60.3	473.771	473.946	424.390	336.973	192.666	4469.109
60.5	483.398	454.681	418.944	335.946	191.708	4469.109
60.7	479.115	467.288	414.891	338.641	193.276	4469.109
60.9	473.834	454.445	411.716	334.942	192.275	4469.109
61.1	483.489	461.142	432.009	339.035	191.844	4469.109
61.3	486.003	474.977	417.674	335.097	192.944	4469.109
61.5	493.380	468.304	418.090	333.288	192.208	4469.109
61.7	483.625	463.495	421.997	334.945	192.762	4469.109

Midpoint	Ratio: 62	19	6.3	2.1	0.65	Volume
61.9	487.754	471.129	428.639	334.116	193.945	4469.109
62.1	486.774	474.332	452.204	333.550	193.130	4469.109
62.3	485.331	456.286	419.066	340.838	193.617	4469.109
62.5	489.877	469.311	409.762	338.216	195.831	4469.109
62.7	484.442	460.166	422.705	333.016	192.231	4469.109
62.9	480.495	471.546	420.678	336.732	193.710	4469.109
63.1	485.059	459.631	428.981	332.310	194.008	4469.109
63.3	482.672	463.700	438.236	335.866	192.679	4469.109
63.5	478.753	474.127	415.477	337.676	192.603	4469.109
63.7	490.313	468.516	436.966	337.355	191.085	4469.109
63.9	479.451	464.424	428.151	335.015	194.048	4469.109
64.1	481.484	467.249	419.726	334.050	192.642	4469.109
64.3	475.831	453.477	438.016	337.444	193.736	4469.109
64.5	478.734	464.833	427.442	334.428	194.280	4469.109
64.7	480.567	474.725	427.516	336.695	193.696	4469.109
64.9	486.992	455.798	437.064	337.739	193.073	4469.109
65.1	464.443	451.808	414.085	337.583	196.318	4469.109
65.3	483.153	466.667	415.452	333.284	192.172	4469.109
65.5	485.504	468.067	419.750	336.848	193.912	4469.109
65.7	487.309	468.311	431.740	337.176	193.093	4469.109
65.9	485.159	467.225	417.259	333.888	190.392	4469.109
66.1	483.798	465.242	435.794	336.652	193.116	4469.109
66.3	484.778	463.165	425.660	336.284	193.236	4469.109
66.5	478.063	476.410	425.733	334.159	192.440	4469.109
66.7	477.827	465.337	432.204	335.605	193.981	4469.109
66.9	472.836	460.190	414.256	338.240	193.279	4469.109
67.1	487.445	462.708	431.545	334.832	195.698	4469.109
67.3	478.725	458.325	417.015	335.843	192.851	4469.109
67.5	477.473	460.410	419.482	335.011	191.612	4469.109
67.7	477.582	471.633	424.903	335.376	192.507	4469.109
67.9	480.912	469.287	430.006	337.129	191.562	4469.109
68.1	478.008	473.616	416.502	335.373	191.164	4469.109
68.3	480.468	471.105	432.473	332.297	190.591	4469.109
68.5	481.792	469.846	409.958	338.687	193.216	4469.109
68.7	483.798	473.112	423.584	336.443	193.093	4469.109
68.9	481.330	462.401	426.636	337.033	193.730	4469.109
69.1	483.308	463.220	435.818	333.162	192.473	4469.109
69.3	476.684	470.444	439.921	334.776	193.183	4469.109
69.5	479.115	463.645	421.411	337.643	189.759	4469.109
69.7	484.678	455.554	423.608	334.213	193.169	4469.109
69.9	481.565	456.570	428.077	337.384	191.254	4469.109
70.1	480.377	472.648	431.154	340.182	194.124	4469.109
70.3	481.956	465.313	422.094	333.616	194.157	4469.109
70.5	477.718	467.249	431.618	335.618	192.268	4469.109
70.7	491.991	462.433	415.843	331.873	192.029	4469.109

Midpoint	Ratio: 62	19	6.3	2.1	0.65	Volume
70.9	479.714	471.381	433.474	339.045	193.464	4469.109
71.1	480.331	471.703	435.330	334.673	191.095	4469.109
71.3	481.484	468.807	416.478	335.959	193.378	4469.109
71.5	487.600	469.114	418.407	330.484	190.972	4469.109
71.7	485.567	462.118	430.153	334.451	191.340	4469.109
71.9	481.166	464.825	432.668	333.152	192.709	4469.109
72.1	481.438	472.892	433.987	333.377	190.849	4469.109
72.3	475.758	474.033	409.982	332.923	192.052	4469.109
72.5	476.484	461.693	411.179	333.351	194.833	4469.109
72.7	483.081	459.489	416.405	337.842	196.384	4469.109
72.9	471.847	472.451	422.436	332.824	196.050	4469.109
73.1	487.618	462.826	426.026	336.864	192.536	4469.109
73.3	471.457	472.223	423.047	332.519	191.999	4469.109
73.5	483.108	463.519	427.980	332.486	193.607	4469.109
73.7	487.064	464.620	418.138	331.160	192.967	4469.109
73.9	479.351	468.492	412.082	334.981	192.394	4469.109
74.1	476.829	452.721	433.840	336.324	193.501	4469.109
74.3	481.965	478.408	430.715	337.245	191.539	4469.109
74.5	486.974	463.558	420.947	335.896	192.818	4469.109
74.7	484.596	465.966	438.334	332.767	193.428	4469.109
74.9	481.720	468.957	423.218	333.735	193.272	4469.109
75.1	477.156	459.041	426.832	335.078	190.786	4469.109
75.3	478.743	463.778	416.942	336.224	192.026	4469.109
75.5	475.849	460.772	426.832	336.364	192.162	4469.109
75.7	468.898	461.103	430.202	334.358	191.158	4469.109
75.9	484.251	466.840	422.021	335.615	192.023	4469.109
76.1	479.007	455.491	423.169	333.530	192.868	4469.109
76.3	478.181	469.523	418.505	337.510	191.943	4469.109
76.5	483.217	467.304	426.514	332.575	191.061	4469.109
76.7	494.233	469.342	425.269	329.489	188.778	4469.109
76.9	473.000	464.329	432.497	338.833	195.055	4469.109
77.1	470.559	463.857	443.730	337.902	192.261	4469.109
77.3	480.994	466.242	400.971	333.414	192.374	4469.109
77.5	476.366	470.971	435.525	335.827	194.714	4469.109
77.7	466.911	459.655	423.120	337.066	188.410	4469.109
77.9	482.083	457.703	409.323	334.203	191.811	4469.109
78.1	479.342	464.101	423.706	335.300	189.166	4469.109
78.3	468.590	462.338	426.783	335.648	188.854	4469.109
78.5	468.898	473.167	417.357	332.900	190.902	4469.109
78.7	479.714	462.669	411.154	335.051	191.565	4469.109
78.9	470.495	468.241	411.936	331.014	192.719	4469.109
79.1	472.873	463.479	420.825	333.676	192.036	4469.109
79.3	488.371	464.046	429.787	332.661	192.351	4469.109
79.5	466.829	468.941	411.154	333.659	190.936	4469.109
79.7	482.727	461.386	422.851	332.118	188.128	4469.109

Midpoint	Ratio: 62	19	6.3	2.1	0.65	Volume
79.9	476.411	470.743	422.851	330.474	192.162	4469.109
80.1	480.159	468.854	423.584	333.516	189.845	4469.109
80.3	482.391	466.541	415.501	330.971	192.758	4469.109
80.5	472.310	455.004	435.696	329.658	190.737	4469.109
80.7	471.947	455.539	426.930	329.440	190.220	4469.109
80.9	474.569	449.471	417.162	327.063	189.467	4469.109
81.1	476.039	457.301	406.930	333.894	192.483	4469.109
81.3	475.486	460.434	419.018	329.854	190.843	4469.109
81.5	484.560	455.240	408.126	331.236	189.623	4469.109
81.7	472.437	459.474	426.392	336.413	190.031	4469.109
81.9	476.048	462.047	425.635	330.368	191.718	4469.109
82.1	481.057	464.227	413.816	333.208	188.632	4469.109
82.3	468.327	463.495	417.943	331.465	190.186	4469.109
82.5	483.398	463.149	429.249	333.536	188.161	4469.109
82.7	472.210	465.494	425.757	331.080	190.200	4469.109
82.9	482.028	456.829	400.239	333.172	191.293	4469.109
83.1	474.724	460.607	429.396	332.837	192.420	4469.109
83.3	483.444	456.105	416.893	336.045	190.607	4469.109
83.5	470.423	464.715	409.421	330.610	189.865	4469.109
83.7	478.462	454.697	425.733	329.828	187.641	4469.109
83.9	469.016	456.758	434.109	331.077	189.142	4469.109
84.1	475.713	456.522	425.171	330.981	189.948	4469.109
84.3	470.713	463.739	420.458	330.262	191.502	4469.109
84.5	462.655	467.603	429.127	327.388	186.372	4469.109
84.7	481.266	445.261	424.512	327.759	190.740	4469.109
84.9	474.642	453.437	408.346	328.233	188.718	4469.109
85.1	476.048	453.422	416.038	328.757	188.801	4469.109
85.3	470.740	451.509	415.452	331.717	188.824	4469.109
85.5	476.266	453.390	418.334	329.778	189.427	4469.109
85.7	475.041	452.422	403.340	324.640	189.500	4469.109
85.9	474.143	451.187	407.027	331.753	190.829	4469.109
86.1	462.320	465.895	426.124	330.603	189.003	4469.109
86.3	486.493	455.649	419.775	329.181	187.555	4469.109
86.5	468.045	458.710	412.742	326.284	186.978	4469.109
86.7	475.196	456.766	418.920	326.258	187.876	4469.109
86.9	462.519	451.635	401.631	325.688	188.950	4469.109
87.1	477.482	457.443	415.184	329.702	187.038	4469.109
87.3	481.547	458.742	420.922	327.295	187.250	4469.109
87.5	472.891	447.803	414.329	327.481	189.222	4469.109
87.7	469.842	456.711	410.568	327.491	186.531	4469.109
87.9	474.379	461.166	406.710	327.922	187.538	4469.109
88.1	480.150	454.634	403.853	322.904	187.382	4469.109
88.3	468.109	450.470	411.838	323.059	186.491	4469.109
88.5	469.461	455.240	404.634	330.232	188.589	4469.109
88.7	459.978	453.965	405.562	323.146	184.515	4469.109

Midpoint	Ratio: 62	19	6.3	2.1	0.65	Volume
88.9	464.252	436.730	397.650	321.488	186.259	4469.109
89.1	471.992	449.739	406.148	325.323	184.174	4469.109
89.3	460.605	449.487	404.879	322.566	185.831	4469.109
89.5	464.506	440.192	414.549	319.453	184.575	4469.109
89.7	456.812	453.130	406.344	323.775	187.260	4469.109
89.9	468.381	437.619	411.423	321.654	183.975	4469.109
90.1	468.671	445.544	412.717	324.196	183.737	4469.109
90.3	458.073	458.671	406.857	320.507	182.722	4469.109
90.5	465.541	444.489	400.532	317.776	183.127	4469.109
90.7	458.663	439.201	419.726	317.057	183.160	4469.109
90.9	468.072	445.953	403.951	319.367	182.215	4469.109
91.1	457.029	452.273	403.340	321.873	185.695	4469.109
91.3	447.828	443.372	391.790	316.838	182.046	4469.109
91.5	465.132	435.612	395.330	320.955	181.566	4469.109
91.7	457.919	441.979	415.721	319.609	182.865	4469.109
91.9	451.249	440.861	399.433	316.132	181.499	4469.109
92.1	460.686	444.820	405.782	310.846	183.160	4469.109
92.3	448.572	441.467	393.523	316.735	181.645	4469.109
92.5	453.599	436.722	419.701	314.929	182.865	4469.109
92.7	449.770	449.597	402.485	318.210	181.831	4469.109
92.9	451.177	438.500	405.049	317.458	182.351	4469.109
93.1	464.225	442.207	409.616	321.051	180.372	4469.109
93.3	451.703	440.255	398.554	317.514	180.833	4469.109
93.5	451.358	437.871	402.266	317.289	181.343	4469.109
93.7	465.541	444.261	400.336	317.037	182.010	4469.109
93.9	465.132	439.217	405.123	317.856	182.812	4469.109
94.1	452.973	447.378	413.401	317.697	179.935	4469.109
94.3	465.205	435.604	414.060	319.258	179.753	4469.109
94.5	462.247	440.122	405.513	322.214	181.463	4469.109
94.7	460.441	443.632	411.081	317.925	181.264	4469.109
94.9	459.071	442.624	394.647	315.957	181.045	4469.109
95.1	460.087	444.285	407.296	318.499	181.111	4469.109
95.3	454.398	445.316	402.290	319.178	181.403	4469.109
95.5	449.607	449.644	415.550	317.700	184.714	4469.109
95.7	462.183	454.287	408.517	317.180	184.472	4469.109
95.9	461.330	446.646	407.321	318.847	185.801	4469.109
96.1	466.965	454.279	420.287	325.131	183.475	4469.109
96.3	461.076	446.504	400.556	323.533	187.054	4469.109
96.5	460.931	448.039	411.765	324.743	185.109	4469.109
96.7	460.786	444.749	419.726	320.298	187.416	4469.109
96.9	472.718	456.223	426.832	326.215	184.761	4469.109
97.1	459.280	458.545	408.542	323.825	185.626	4469.109
97.3	463.608	447.913	407.858	327.686	187.916	4469.109
97.5	470.858	461.268	411.374	326.155	189.288	4469.109
97.7	466.439	465.644	419.164	330.792	189.142	4469.109

Midpoint	Ratio: 62	19	6.3	2.1	0.65	Volume
97.9	471.793	462.622	423.364	331.733	190.173	4469.109
98.1	481.738	467.698	409.860	330.964	188.953	4469.109
98.3	476.711	460.442	423.633	330.444	188.354	4469.109
98.5	489.460	464.558	419.750	331.806	189.550	4469.109
98.7	489.560	466.525	421.972	334.846	192.480	4469.109
98.9	489.188	465.494	404.854	329.887	191.801	4469.109
99.1	480.894	474.859	435.281	335.896	192.802	4469.109
99.3	476.829	463.212	421.435	338.982	193.345	4469.109
99.5	479.841	481.564	431.374	336.987	193.594	4469.109
99.7	484.052	385.387	347.810	271.039	156.691	4469.109
99.9	97.418	1.125	1.245	0.865	0.504	4469.109
100.1	1.016	0.582	0.366	0.388	0.209	4469.109

E.6. CavAb channel: Calcium and Sodium

Table of Na^+ population data for simulation with Ca^{2+} and Na^+ across bulk density ratios (#JJF-GRS12G8)

Population in histogram bin at Na^+/Ca^{2+} ratio:

Midpt.	11000	2700	910	240	84	26	8.8	2.7	0.92	0.31	Volume	
-99.85	67.06	59.19	34.69	0.00	38.31	36.46	5.42	91.63	28.83	13.58	0.28	2239.26
-99.75	261.74	261.92	262.90	193.61	259.51	246.47	226.22	188.55	103.26	48.93	14.11	2239.26
-99.65	261.51	260.74	259.47	259.84	253.68	248.31	224.66	181.90	104.89	54.03	14.45	2239.26
-99.55	256.38	256.03	258.78	258.72	253.07	248.58	221.03	177.38	103.07	46.73	13.06	2239.26
-99.45	255.68	259.04	260.01	255.75	256.41	246.21	226.83	180.39	105.22	48.63	13.95	2239.26
-99.35	260.55	259.89	258.48	260.32	256.14	248.13	223.20	181.30	105.04	49.64	14.17	2239.26
-99.25	257.87	258.57	257.71	258.06	256.19	246.96	228.52	189.15	103.65	47.86	14.56	2239.26
-99.15	258.34	258.28	256.96	257.10	252.59	247.19	222.41	168.92	104.34	53.32	13.95	2239.26
-99.05	256.30	255.67	256.90	258.62	251.83	245.38	223.43	176.32	102.77	48.16	13.28	2239.26
-98.95	255.85	255.12	257.42	256.53	251.08	241.94	224.94	173.75	102.86	46.79	13.89	2239.26
-98.85	256.73	254.36	254.44	256.45	254.08	241.33	218.81	177.38	102.11	48.81	14.00	2239.26
-98.75	253.73	250.34	253.45	252.25	252.67	246.47	220.88	157.00	103.65	46.61	14.39	2239.26
-98.65	254.33	254.28	257.30	253.70	250.20	242.69	220.29	176.32	104.04	47.56	13.12	2239.26
-98.55	251.69	253.38	253.36	256.36	255.39	243.60	219.68	186.58	96.43	44.66	13.73	2239.26
-98.45	254.37	254.78	255.49	251.31	248.39	242.72	224.20	164.39	101.69	45.25	12.45	2239.26
-98.35	251.15	250.42	253.37	253.73	249.38	239.94	222.64	166.81	103.62	48.75	13.78	2239.26
-98.25	252.07	252.99	252.86	253.70	247.07	243.51	218.27	164.24	101.35	45.96	12.79	2239.26
-98.15	248.87	249.58	252.99	254.97	248.21	236.37	218.78	167.71	103.92	46.91	14.50	2239.26
-98.05	252.08	251.46	250.76	248.39	248.54	243.85	223.25	178.28	101.96	49.28	12.95	2239.26
-97.95	248.16	248.91	250.00	249.07	243.75	242.17	215.59	165.00	99.66	48.93	12.45	2239.26
-97.85	250.45	251.59	249.46	248.85	245.16	238.56	220.19	167.56	99.78	44.30	13.06	2239.26
-97.75	247.23	248.85	247.77	249.03	247.30	238.12	216.53	171.79	100.39	46.02	14.61	2239.26
-97.65	251.47	253.74	250.28	244.92	245.71	235.19	220.80	169.98	104.80	49.22	12.23	2239.26
-97.55	251.12	247.49	247.11	248.22	245.52	237.06	217.94	163.79	99.99	46.55	12.45	2239.26
-97.45	247.85	248.18	248.10	249.57	249.47	234.49	216.02	158.36	98.00	46.44	13.95	2239.26
-97.35	250.37	248.03	244.87	245.69	245.14	236.81	217.63	153.22	102.47	45.66	12.62	2239.26

Midpt.	11000	2700	910	240	84	26	8.8	2.7	0.92	0.31	Volume	
-97.25	245.96	248.33	247.31	247.45	248.09	237.47	217.74	158.36	102.89	47.62	12.56	2239.26
-97.15	246.51	245.92	245.00	248.02	246.08	237.26	213.57	170.73	100.75	46.08	12.34	2239.26
-97.05	250.10	245.41	246.71	243.12	240.36	232.96	210.15	164.85	99.51	47.15	12.56	2239.26
-96.95	242.62	245.27	247.59	245.07	242.69	235.67	218.53	170.43	97.58	45.49	12.84	2239.26
-96.85	246.54	242.90	247.21	244.08	245.19	234.94	211.50	164.39	99.51	44.66	12.45	2239.26
-96.75	244.11	245.34	243.64	245.17	241.90	232.87	214.26	166.36	99.33	46.50	12.68	2239.26
-96.65	244.75	244.27	244.67	247.43	244.42	233.81	214.29	163.94	96.88	45.61	13.28	2239.26
-96.55	245.47	245.37	243.81	241.62	238.58	226.98	216.66	161.98	99.75	47.03	12.56	2239.26
-96.45	244.93	242.67	242.65	241.40	241.20	234.94	216.18	162.28	103.04	42.40	11.01	2239.26
-96.35	238.40	242.64	242.30	239.62	241.18	232.81	214.34	167.71	96.37	46.55	12.56	2239.26
-96.25	244.34	241.56	244.11	242.78	239.64	232.58	217.51	158.66	98.88	47.09	11.79	2239.26
-96.15	240.57	237.77	243.01	243.15	244.57	229.42	214.34	162.58	99.00	45.84	13.40	2239.26
-96.05	241.49	239.89	240.17	239.11	238.50	232.76	214.01	160.02	98.45	46.55	12.84	2239.26
-95.95	239.06	241.68	243.02	241.93	237.68	228.60	208.10	154.88	98.42	44.66	12.34	2239.26
-95.85	241.92	240.16	239.68	239.96	238.04	229.55	207.03	166.51	95.62	44.54	12.01	2239.26
-95.75	238.38	241.48	240.01	239.48	239.72	227.90	213.78	157.90	98.39	45.55	13.12	2239.26
-95.65	241.22	242.86	241.81	238.81	237.16	224.96	208.18	159.71	99.99	44.54	12.18	2239.26
-95.55	238.01	240.56	239.86	239.15	238.03	228.30	211.81	163.64	95.01	43.11	11.73	2239.26
-95.45	239.28	242.10	239.06	240.34	235.48	230.65	208.46	152.92	96.43	46.14	12.62	2239.26
-95.35	237.41	239.53	240.19	241.96	236.75	229.92	205.24	157.00	96.16	44.06	13.17	2239.26
-95.25	237.28	239.06	240.25	239.46	237.05	225.94	205.24	160.47	92.42	44.78	12.07	2239.26
-95.15	237.54	238.24	237.34	238.76	234.49	232.17	205.83	164.70	96.82	44.66	13.51	2239.26
-95.05	236.15	236.87	238.36	237.96	233.48	228.85	211.09	150.81	98.09	44.66	13.56	2239.26
-94.95	235.00	239.04	237.51	238.10	237.84	228.73	210.58	162.43	92.99	45.66	10.18	2239.26
-94.85	236.86	236.43	235.82	240.62	238.21	229.55	204.73	150.81	95.50	46.85	12.18	2239.26
-94.75	240.74	237.75	238.26	237.28	234.51	225.01	203.35	155.18	97.58	45.72	13.17	2239.26
-94.65	235.44	239.68	235.14	236.84	231.45	224.71	210.12	166.51	93.96	42.11	11.62	2239.26
-94.55	238.06	236.57	233.80	235.17	234.45	227.62	201.74	152.01	96.25	44.83	12.90	2239.26
-94.45	236.08	238.58	235.17	236.50	233.16	222.35	203.66	162.58	91.78	44.66	13.06	2239.26
-94.35	235.47	235.80	236.32	236.38	236.01	228.82	202.56	155.03	94.35	44.12	11.96	2239.26
-94.25	234.10	234.73	236.32	235.39	236.12	227.15	208.69	157.15	97.97	42.70	12.73	2239.26
-94.15	232.88	234.37	236.40	234.16	233.41	227.57	201.44	150.35	96.98	42.94	11.35	2239.26
-94.05	236.72	235.86	235.98	235.42	231.00	226.08	201.59	154.58	93.74	42.52	12.01	2239.26
-93.95	234.62	235.44	237.81	234.71	232.07	229.55	206.19	154.13	93.71	43.17	11.35	2239.26
-93.85	233.79	233.75	235.90	234.72	234.77	222.69	204.83	156.09	95.47	41.45	12.73	2239.26
-93.75	232.70	238.21	234.60	235.64	231.15	222.73	207.39	156.85	95.19	44.18	11.62	2239.26
-93.65	232.33	235.58	235.89	233.13	231.77	226.46	206.37	158.51	95.07	44.60	11.68	2239.26
-93.55	234.00	234.85	235.12	233.00	228.79	218.64	201.46	157.15	93.11	44.36	10.90	2239.26
-93.45	235.70	234.50	235.50	233.03	228.58	220.62	202.07	149.30	94.20	45.72	10.46	2239.26
-93.35	234.86	234.51	234.33	235.19	233.18	223.89	201.69	160.32	94.41	43.11	11.51	2239.26
-93.25	234.99	232.03	233.71	231.28	226.62	221.46	204.12	153.83	96.28	44.60	10.85	2239.26
-93.15	233.24	234.12	234.06	235.93	229.19	222.46	205.01	160.77	92.90	46.14	12.73	2239.26
-93.05	234.46	236.05	234.44	233.95	233.40	225.40	201.90	159.56	93.44	44.66	11.90	2239.26
-92.95	231.93	235.47	232.75	233.77	226.86	219.62	198.14	152.32	94.14	41.10	11.51	2239.26
-92.85	230.55	233.64	235.43	236.22	226.47	224.33	204.25	150.96	92.78	46.14	11.79	2239.26

Midpt.	11000	2700	910	240	84	26	8.8	2.7	0.92	0.31	Volume	
-92.75	230.99	234.36	234.84	232.36	228.90	223.60	203.30	154.13	92.11	43.06	11.85	2239.26
-92.65	236.74	232.13	231.57	229.56	235.95	224.83	201.67	150.05	93.93	42.28	10.07	2239.26
-92.55	234.84	233.66	232.28	231.64	232.37	218.89	204.37	149.15	92.96	41.69	11.07	2239.26
-92.45	234.49	234.54	234.54	232.01	232.89	223.64	206.78	166.05	93.90	46.97	10.35	2239.26
-92.35	232.46	232.04	233.72	232.66	226.96	224.28	202.56	149.75	93.50	45.72	10.96	2239.26
-92.25	235.35	234.36	231.70	233.53	227.16	223.37	205.98	166.81	93.62	43.11	9.96	2239.26
-92.15	232.75	233.38	234.80	232.32	228.40	222.85	204.20	155.18	93.53	42.17	11.62	2239.26
-92.05	234.59	235.17	231.11	231.51	230.97	225.87	203.43	156.69	91.21	44.12	10.57	2239.26
-91.95	231.67	234.15	233.04	233.64	231.00	222.41	206.78	157.60	91.24	43.65	11.46	2239.26
-91.85	233.44	234.13	229.19	235.04	231.67	217.98	203.20	156.39	92.60	42.46	12.18	2239.26
-91.75	234.37	233.39	231.67	232.61	229.39	220.89	201.03	151.26	93.68	41.69	12.90	2239.26
-91.65	233.10	234.68	233.36	234.46	230.55	225.01	206.03	146.58	94.05	44.60	12.29	2239.26
-91.55	235.75	234.48	234.57	234.62	231.22	223.10	204.50	160.02	96.85	45.78	11.35	2239.26
-91.45	232.19	232.04	233.34	233.62	229.46	222.33	203.66	154.28	95.31	41.16	11.51	2239.26
-91.35	232.70	234.12	234.86	234.42	230.28	225.26	202.97	143.71	94.41	42.58	10.74	2239.26
-91.25	236.02	234.48	232.51	232.56	229.34	222.19	201.44	161.53	95.62	41.34	12.01	2239.26
-91.15	233.68	234.32	236.39	232.29	232.46	221.35	203.48	160.77	96.19	44.36	11.13	2239.26
-91.05	235.19	234.10	234.32	231.30	227.93	222.57	203.10	166.36	93.08	44.78	11.85	2239.26
-90.95	231.78	233.39	236.08	235.34	229.22	223.51	206.70	154.58	94.92	42.34	11.40	2239.26
-90.85	235.78	234.71	234.90	234.56	231.42	225.12	203.61	156.09	94.56	43.94	10.90	2239.26
-90.75	232.84	235.09	235.04	235.68	231.18	226.48	207.08	160.32	92.33	41.93	11.85	2239.26
-90.65	236.15	233.62	234.11	233.60	231.18	221.30	202.99	161.83	94.26	42.05	10.90	2239.26
-90.55	236.67	233.42	235.93	233.54	234.19	223.32	203.15	152.17	93.87	43.77	11.96	2239.26
-90.45	232.13	236.07	233.04	233.47	233.10	223.96	201.46	159.71	93.93	40.15	11.24	2239.26
-90.35	231.99	234.02	234.64	236.68	231.18	222.64	202.84	164.39	93.65	42.58	11.90	2239.26
-90.25	236.55	233.89	236.14	235.43	232.74	228.73	201.26	160.62	93.62	43.35	10.52	2239.26
-90.15	240.56	236.30	237.15	235.16	232.53	219.42	205.68	158.81	93.41	44.72	10.74	2239.26
-90.05	234.50	235.84	236.65	235.57	232.24	225.74	207.29	154.28	95.68	43.35	11.79	2239.26
-89.95	235.16	235.20	237.34	236.42	233.65	224.01	210.61	160.92	94.74	42.52	10.52	2239.26
-89.85	239.61	237.02	235.79	234.64	235.36	227.01	208.33	159.41	97.97	44.66	10.13	2239.26
-89.75	236.40	238.12	234.66	236.98	233.62	230.74	205.68	166.81	96.22	46.44	10.46	2239.26
-89.65	237.97	239.51	235.95	235.94	234.19	224.85	207.44	151.41	95.56	45.90	10.30	2239.26
-89.55	237.96	236.07	235.74	237.07	235.33	220.66	207.18	163.64	95.56	43.77	11.90	2239.26
-89.45	238.38	242.66	235.04	234.89	238.06	224.35	204.94	156.69	95.77	42.11	11.68	2239.26
-89.35	236.35	235.14	236.11	233.09	233.13	224.55	207.62	162.28	93.68	43.17	12.18	2239.26
-89.25	235.11	237.69	238.39	236.53	233.45	222.23	204.40	155.79	97.82	46.08	11.35	2239.26
-89.15	236.04	237.44	239.57	238.50	234.09	225.57	203.76	165.30	94.29	43.35	9.47	2239.26
-89.05	235.35	239.39	238.12	236.65	233.77	223.55	208.08	149.90	96.73	43.77	10.46	2239.26
-88.95	236.62	236.57	237.95	236.20	232.83	226.26	198.91	147.03	97.70	42.28	10.07	2239.26
-88.85	238.28	237.21	241.00	236.58	235.06	227.60	210.76	156.69	93.68	42.58	11.01	2239.26
-88.75	237.75	236.34	240.76	235.62	237.09	227.98	207.29	156.69	93.41	42.46	10.41	2239.26
-88.65	237.36	240.53	238.96	237.93	232.58	223.03	208.97	158.51	94.74	44.54	10.74	2239.26
-88.55	238.87	236.46	241.07	237.08	232.14	228.53	210.97	161.07	97.19	42.82	11.07	2239.26
-88.45	236.82	237.47	238.38	239.31	232.48	225.23	211.88	151.86	94.74	44.54	10.52	2239.26
-88.35	236.68	237.56	239.95	237.86	239.00	227.74	207.57	155.49	94.38	43.89	11.13	2239.26

Midpt.	11000	2700	910	240	84	26	8.8	2.7	0.92	0.31	Volume	
-88.25	236.40	237.60	238.45	239.05	235.76	229.44	205.40	166.05	97.52	44.95	8.80	2239.26
-88.15	237.95	238.06	238.59	236.26	234.34	224.58	204.88	154.13	94.80	43.59	10.57	2239.26
-88.05	233.98	235.88	239.03	237.91	233.30	227.15	206.60	160.47	93.90	43.41	10.63	2239.26
-87.95	239.13	237.96	241.19	237.72	233.72	226.21	204.12	166.20	95.19	45.55	9.85	2239.26
-87.85	237.81	238.36	239.82	239.05	236.60	230.56	206.55	158.05	97.43	45.43	9.47	2239.26
-87.75	236.45	239.84	238.24	236.30	236.22	227.65	211.27	155.03	99.12	43.71	9.80	2239.26
-87.65	239.81	235.92	237.90	238.13	234.07	229.03	208.87	165.45	94.68	43.17	9.69	2239.26
-87.55	240.04	236.57	240.46	238.95	235.43	227.65	207.41	169.38	97.58	44.83	10.41	2239.26
-87.45	238.74	239.80	239.49	240.09	237.39	228.74	203.66	153.37	97.07	42.05	9.08	2239.26
-87.35	237.50	240.79	238.33	239.23	235.26	225.87	210.84	154.58	94.83	43.89	9.80	2239.26
-87.25	239.71	238.25	239.66	236.87	231.47	230.46	209.94	153.07	94.35	43.89	11.29	2239.26
-87.15	235.17	240.62	238.01	238.58	235.38	227.35	205.96	152.32	95.25	42.76	12.29	2239.26
-87.05	240.43	238.33	239.27	238.39	235.36	228.65	204.78	158.05	96.76	40.98	9.91	2239.26
-86.95	239.38	236.81	239.96	240.88	232.81	228.44	208.46	160.92	96.31	43.29	10.79	2239.26
-86.85	239.15	241.24	237.78	241.38	237.19	228.76	205.14	163.34	96.55	43.71	10.24	2239.26
-86.75	240.93	239.99	238.66	241.12	237.44	228.05	203.40	152.62	98.06	44.66	10.68	2239.26
-86.65	239.13	238.31	239.13	237.68	237.66	231.53	202.97	161.53	94.62	41.75	11.24	2239.26
-86.55	240.12	238.94	242.09	240.50	233.83	227.26	212.68	159.56	94.29	49.28	10.18	2239.26
-86.45	237.58	240.83	239.58	236.13	238.14	226.89	212.42	157.90	95.31	43.29	10.30	2239.26
-86.35	239.00	237.69	237.94	238.93	235.56	226.92	207.36	157.00	96.22	43.59	10.18	2239.26
-86.25	237.93	241.17	238.61	239.30	240.59	229.74	206.16	165.75	94.95	43.77	10.30	2239.26
-86.15	242.53	240.41	239.59	237.61	235.73	225.78	205.45	163.94	98.00	44.24	11.13	2239.26
-86.05	239.89	240.52	241.05	237.78	236.67	225.26	208.90	160.62	96.19	41.45	9.63	2239.26
-85.95	241.71	238.90	237.81	239.50	237.59	231.03	211.91	161.98	96.43	44.83	9.47	2239.26
-85.85	237.86	238.80	240.03	241.53	238.08	229.90	206.14	150.20	96.79	44.42	10.07	2239.26
-85.75	238.27	239.32	237.36	235.71	234.10	226.30	205.27	156.85	100.63	45.72	11.24	2239.26
-85.65	235.51	239.40	240.42	236.32	238.77	229.64	204.71	155.94	97.37	40.74	10.02	2239.26
-85.55	242.10	240.07	239.82	238.67	231.82	223.41	207.75	162.13	94.23	47.27	11.57	2239.26
-85.45	239.84	242.27	239.92	243.02	238.03	226.92	209.02	156.24	92.30	43.00	10.85	2239.26
-85.35	238.29	241.32	240.71	239.97	237.69	226.69	206.90	163.94	95.25	44.42	11.46	2239.26
-85.25	242.98	241.27	241.15	237.75	235.39	227.21	209.38	157.30	95.25	46.61	11.01	2239.26
-85.15	242.22	240.48	237.39	240.31	236.89	227.40	207.85	148.09	95.83	42.17	9.69	2239.26
-85.05	243.58	242.54	238.83	240.65	238.68	231.49	208.87	162.28	97.70	44.30	10.74	2239.26
-84.95	239.06	239.08	237.89	243.39	235.16	233.31	205.40	152.92	95.89	45.25	10.90	2239.26
-84.85	240.19	241.83	239.14	237.20	237.74	231.87	204.86	149.15	98.39	42.64	9.85	2239.26
-84.75	239.19	240.60	238.16	239.98	236.38	224.51	211.81	157.90	97.22	45.37	11.13	2239.26
-84.65	239.97	238.44	242.75	239.73	234.76	226.01	211.27	161.68	94.53	46.67	11.13	2239.26
-84.55	241.36	242.95	239.19	238.81	236.17	228.53	206.62	162.73	95.35	47.38	9.24	2239.26
-84.45	240.50	241.73	241.16	240.01	241.31	227.74	204.02	157.30	97.97	43.94	9.24	2239.26
-84.35	236.19	240.50	243.85	239.89	239.79	227.24	205.70	152.77	95.38	45.19	10.79	2239.26
-84.25	241.56	243.04	238.96	236.40	234.25	229.96	204.09	167.71	92.02	45.84	10.85	2239.26
-84.15	238.83	239.85	237.55	238.53	235.43	227.05	208.05	154.88	96.76	43.59	10.13	2239.26
-84.05	244.66	240.61	240.76	240.87	234.87	230.44	207.87	157.45	97.16	43.35	9.13	2239.26
-83.95	243.26	240.07	238.30	239.12	239.86	231.10	210.51	161.98	96.10	41.81	9.69	2239.26
-83.85	241.11	242.91	239.74	241.35	241.89	229.67	208.72	150.51	96.40	46.02	10.68	2239.26

Midpt.	11000	2700	910	240	84	26	8.8	2.7	0.92	0.31	Volume	
-83.75	241.88	239.40	239.72	237.68	237.93	229.90	210.94	158.05	95.38	44.00	10.35	2239.26
-83.65	241.23	242.63	244.87	240.24	238.83	230.40	211.83	160.62	95.35	41.39	10.35	2239.26
-83.55	241.60	238.13	241.83	240.93	238.93	231.03	206.90	159.41	96.95	46.79	8.36	2239.26
-83.45	239.88	242.99	238.43	240.30	236.52	228.40	208.59	157.90	97.07	44.95	9.19	2239.26
-83.35	239.15	237.98	241.22	240.40	240.28	229.19	204.63	168.77	95.77	44.78	10.18	2239.26
-83.25	240.91	238.47	241.28	241.55	235.14	222.39	211.04	151.11	98.30	42.17	10.85	2239.26
-83.15	239.35	240.70	240.68	238.23	240.07	234.55	210.02	163.79	96.52	43.29	10.79	2239.26
-83.05	240.04	240.40	238.07	240.43	235.98	233.17	207.08	155.79	98.36	46.61	10.41	2239.26
-82.95	236.53	240.53	239.45	239.79	237.74	229.64	208.23	152.01	94.05	43.53	11.24	2239.26
-82.85	239.16	240.20	241.82	242.68	238.51	229.24	215.28	169.07	96.31	45.07	10.68	2239.26
-82.75	240.04	241.87	240.41	240.88	237.69	230.08	207.39	159.71	96.25	45.66	10.18	2239.26
-82.65	241.18	237.96	241.29	238.95	239.97	233.14	211.30	161.37	95.65	42.58	10.41	2239.26
-82.55	240.58	241.27	241.46	238.07	235.61	229.85	212.73	160.17	97.37	44.83	10.02	2239.26
-82.45	242.49	239.45	242.24	235.65	238.38	228.67	208.33	161.83	97.73	43.17	9.74	2239.26
-82.35	241.61	240.90	241.97	243.99	239.64	229.40	212.63	162.43	100.51	43.65	8.97	2239.26
-82.25	239.32	241.39	239.29	236.51	238.93	227.21	209.82	157.75	95.22	45.55	9.13	2239.26
-82.15	243.42	242.34	241.74	239.64	239.00	227.48	210.40	150.51	97.61	41.81	10.24	2239.26
-82.05	240.83	240.70	239.96	242.38	240.51	226.67	211.35	160.17	95.95	39.62	9.85	2239.26
-81.95	240.63	240.84	239.69	241.79	240.02	231.05	207.54	155.18	96.55	45.19	11.85	2239.26
-81.85	241.60	239.79	243.76	238.47	236.99	232.44	205.93	160.17	96.49	43.29	10.52	2239.26
-81.75	240.90	241.52	238.34	240.40	242.35	228.98	212.11	152.62	95.47	43.71	10.52	2239.26
-81.65	242.11	241.51	242.74	237.55	240.33	230.99	209.38	162.43	96.88	43.89	9.80	2239.26
-81.55	241.91	239.45	242.28	241.49	238.25	230.44	209.92	162.13	96.34	45.90	10.35	2239.26
-81.45	241.29	240.43	240.45	240.47	238.43	230.14	209.13	155.64	96.82	47.21	8.91	2239.26
-81.35	243.35	241.96	242.60	241.61	236.87	232.56	214.18	161.98	94.11	40.62	10.85	2239.26
-81.25	243.90	243.95	241.98	241.31	239.57	228.21	209.28	163.79	94.14	43.94	9.30	2239.26
-81.15	237.72	242.23	243.31	238.99	238.06	228.85	207.01	158.51	95.13	43.77	8.63	2239.26
-81.05	242.06	241.71	239.69	240.36	238.80	229.17	210.74	161.37	97.07	44.60	9.96	2239.26
-80.95	239.67	241.94	242.83	242.33	237.68	231.10	205.75	164.70	98.49	47.86	10.79	2239.26
-80.85	240.81	238.88	242.94	240.17	238.33	228.74	211.27	160.47	96.52	42.76	10.74	2239.26
-80.75	242.14	240.79	242.44	240.20	236.70	230.65	211.94	152.17	98.49	44.42	9.63	2239.26
-80.65	239.86	240.69	240.63	241.24	237.24	231.17	205.80	165.75	95.35	40.68	9.74	2239.26
-80.55	241.44	242.92	239.67	241.37	241.18	232.28	208.10	161.53	94.77	45.01	10.07	2239.26
-80.45	240.79	241.12	243.85	240.10	240.19	227.51	211.71	161.68	94.86	44.18	10.07	2239.26
-80.35	241.59	243.54	245.05	242.25	233.72	230.62	209.25	164.70	95.77	42.28	9.91	2239.26
-80.25	241.65	240.16	243.08	241.49	239.44	230.99	211.32	163.79	95.07	46.32	9.13	2239.26
-80.15	242.56	240.14	241.16	242.87	236.15	229.65	208.54	163.34	96.70	44.36	10.35	2239.26
-80.05	238.19	242.73	243.08	239.27	237.05	233.53	209.07	166.20	96.10	40.80	9.24	2239.26
-79.95	238.95	242.87	241.97	239.91	238.23	230.32	209.30	160.92	95.44	44.95	11.29	2239.26
-79.85	240.86	243.48	242.78	238.45	241.33	228.98	202.76	159.11	96.04	47.92	9.74	2239.26
-79.75	240.08	242.85	240.44	240.61	235.13	232.30	209.76	163.64	95.50	42.58	10.41	2239.26
-79.65	241.53	237.98	243.92	241.64	238.08	226.23	211.20	161.37	96.43	41.81	8.58	2239.26
-79.55	243.85	239.33	241.81	245.10	235.43	228.35	206.06	153.83	96.58	42.28	9.35	2239.26
-79.45	241.92	240.01	243.10	240.30	238.98	231.53	208.74	160.77	96.76	44.36	9.47	2239.26
-79.35	242.57	239.53	242.50	241.90	239.03	229.53	206.14	157.15	95.53	44.89	9.24	2239.26

Midpt.	11000	2700	910	240	84	26	8.8	2.7	0.92	0.31	Volume	
-79.25	242.07	243.80	242.78	243.15	241.87	228.80	209.10	164.70	97.64	45.49	10.68	2239.26
-79.15	238.21	242.30	241.71	240.10	240.19	229.30	211.55	152.01	96.07	42.52	9.63	2239.26
-79.05	241.34	246.37	243.12	239.96	237.74	226.64	209.64	157.90	97.40	44.30	10.24	2239.26
-78.95	241.06	240.05	240.21	238.18	233.47	231.03	207.90	166.96	94.71	42.82	8.47	2239.26
-78.85	243.46	240.32	243.78	244.62	236.22	231.85	208.36	153.07	95.74	42.46	9.63	2239.26
-78.75	243.16	241.65	242.22	239.14	238.09	229.78	210.86	160.02	97.55	45.84	9.24	2239.26
-78.65	241.83	243.31	245.54	242.22	239.97	227.69	210.89	156.85	93.08	41.39	10.68	2239.26
-78.55	240.04	242.99	239.75	241.53	234.77	229.46	212.09	148.24	95.04	44.42	10.74	2239.26
-78.45	242.46	238.58	240.69	241.16	240.21	230.80	207.77	159.56	92.69	43.35	9.13	2239.26
-78.35	241.94	242.46	241.52	239.33	237.39	231.74	207.13	154.73	95.95	42.40	9.85	2239.26
-78.25	241.87	241.85	240.50	241.88	239.96	229.65	209.00	147.34	98.97	44.06	9.41	2239.26
-78.15	238.38	244.07	239.67	237.84	237.54	232.10	206.60	153.52	97.67	41.51	10.52	2239.26
-78.05	242.66	239.85	238.63	241.71	238.56	229.76	210.33	166.36	93.17	41.16	9.96	2239.26
-77.95	242.11	245.33	241.75	242.81	240.09	226.33	210.20	163.03	96.95	41.69	11.46	2239.26
-77.85	239.05	243.18	246.48	242.88	236.82	227.23	210.20	164.24	94.56	44.06	9.52	2239.26
-77.75	242.73	243.26	239.31	240.04	236.33	230.05	204.20	162.13	95.95	45.31	9.02	2239.26
-77.65	239.93	243.25	240.32	242.52	240.51	231.74	212.32	153.22	98.82	45.61	9.69	2239.26
-77.55	241.82	240.75	241.45	239.74	237.99	232.90	213.14	156.39	93.53	42.28	10.41	2239.26
-77.45	240.04	241.29	241.95	240.94	237.76	231.44	206.62	153.07	94.92	44.00	8.97	2239.26
-77.35	242.04	241.96	240.96	241.92	240.98	230.83	209.66	154.58	96.22	46.08	10.85	2239.26
-77.25	242.80	242.91	243.85	241.00	239.05	229.33	209.15	168.62	97.10	44.60	10.07	2239.26
-77.15	244.61	240.63	244.28	241.19	235.71	230.92	208.56	165.75	99.06	44.78	10.30	2239.26
-77.05	240.66	241.82	242.92	240.94	243.26	225.96	212.96	153.52	97.61	45.43	10.79	2239.26
-76.95	243.17	240.85	242.49	241.67	237.57	232.71	211.07	159.86	97.25	41.39	10.46	2239.26
-76.85	244.39	240.81	241.47	239.49	238.85	230.48	210.38	163.64	94.50	46.08	9.02	2239.26
-76.75	243.47	241.84	240.49	241.83	240.01	230.42	211.53	156.24	94.20	43.06	9.91	2239.26
-76.65	246.07	240.92	241.33	244.07	239.32	233.94	205.73	159.26	96.22	44.06	8.75	2239.26
-76.55	239.67	244.49	241.81	240.56	241.08	231.26	208.49	162.58	96.98	46.44	9.91	2239.26
-76.45	240.76	238.56	242.19	243.17	241.06	227.49	206.83	166.81	95.71	45.31	7.64	2239.26
-76.35	245.43	243.54	242.21	239.27	243.33	228.14	209.66	157.75	97.97	43.53	9.08	2239.26
-76.25	241.47	244.56	243.42	241.49	240.86	229.83	210.68	156.69	95.25	44.24	9.80	2239.26
-76.15	239.78	243.17	242.93	240.20	241.47	230.01	210.91	152.62	94.02	44.72	10.02	2239.26
-76.05	244.64	244.44	242.66	242.72	237.71	225.83	209.13	163.94	95.77	46.32	10.52	2239.26
-75.95	240.25	242.73	242.84	240.45	239.76	232.49	211.65	167.26	93.05	45.25	9.74	2239.26
-75.85	243.57	241.02	242.61	242.69	241.08	232.30	210.89	158.66	93.84	48.10	9.63	2239.26
-75.75	240.08	242.06	242.99	242.36	239.22	228.60	214.21	166.20	95.71	43.35	26.51	2239.26
-75.65	243.77	245.29	243.32	242.86	239.40	231.65	212.19	162.28	97.88	42.88	8.63	2239.26
-75.55	244.04	241.42	242.46	243.63	242.22	229.10	203.35	151.86	96.49	46.08	9.19	2239.26
-75.45	244.23	240.69	241.02	242.11	238.16	227.78	213.09	160.77	98.06	39.85	10.24	2239.26
-75.35	242.48	245.42	241.18	242.55	235.96	229.74	209.92	154.73	97.19	43.11	8.80	2239.26
-75.25	240.70	241.56	242.20	242.20	234.47	230.64	207.13	162.58	93.50	44.24	10.41	2239.26
-75.15	242.94	239.22	242.85	244.85	237.62	232.46	208.49	161.07	97.49	47.15	9.96	2239.26
-75.05	243.57	241.89	242.37	243.30	239.94	225.08	213.83	156.24	94.86	44.83	9.63	2239.26
-74.95	242.10	240.35	243.12	244.48	241.73	226.98	212.34	146.88	96.88	44.18	10.30	2239.26
-74.85	244.77	242.82	242.15	241.41	239.62	232.78	209.02	162.88	93.05	44.78	9.41	2239.26

Midpt.	11000	2700	910	240	84	26	8.8	2.7	0.92	0.31	Volume	
-74.75	241.97	245.32	241.67	243.45	237.21	228.62	211.55	156.39	93.90	46.79	10.13	2239.26
-74.65	242.19	242.19	242.52	240.25	238.31	231.31	209.46	170.73	95.89	44.72	8.80	2239.26
-74.55	242.33	243.24	242.01	243.02	237.71	231.83	211.32	163.03	95.56	44.72	10.13	2239.26
-74.45	242.09	242.55	244.17	240.95	243.45	230.15	206.44	164.85	93.08	43.94	10.57	2239.26
-74.35	241.33	240.69	246.39	244.45	236.55	231.28	210.81	165.60	93.02	44.12	10.41	2239.26
-74.25	241.69	241.73	243.97	240.87	240.73	226.40	208.90	159.26	94.92	41.93	9.74	2239.26
-74.15	243.40	243.13	240.55	242.45	238.72	230.39	208.00	165.60	94.68	42.23	10.07	2239.26
-74.05	240.91	243.15	242.55	241.41	239.17	232.17	210.45	154.58	95.80	41.16	9.91	2239.26
-73.95	241.66	242.31	240.79	239.78	237.16	232.98	208.84	152.01	96.82	46.38	9.30	2239.26
-73.85	244.07	241.21	242.68	241.79	237.41	230.30	206.06	163.64	95.71	44.24	10.90	2239.26
-73.75	241.80	243.47	241.55	242.13	239.35	232.94	212.96	168.77	96.76	45.31	10.90	2239.26
-73.65	240.26	241.36	243.95	240.09	237.04	229.69	211.91	161.37	98.39	43.29	8.30	2239.26
-73.55	239.02	245.71	242.94	244.37	240.36	233.15	209.92	157.60	95.19	43.53	10.07	2239.26
-73.45	240.52	243.19	243.36	239.65	241.26	232.12	211.07	157.30	97.07	44.24	9.74	2239.26
-73.35	242.62	243.73	240.89	242.32	240.98	231.26	205.68	156.39	96.95	43.83	8.86	2239.26
-73.25	242.34	239.81	243.02	241.31	239.49	227.67	211.63	156.85	96.64	45.96	9.63	2239.26
-73.15	244.15	242.23	240.32	245.22	240.53	230.14	212.19	160.92	94.17	43.41	10.18	2239.26
-73.05	242.32	244.06	244.06	241.84	237.91	227.53	214.67	161.98	95.19	41.69	9.91	2239.26
-72.95	240.15	238.42	243.16	242.94	239.96	232.78	211.27	162.43	95.89	46.38	10.41	2239.26
-72.85	245.45	243.31	242.45	238.82	237.16	229.21	209.74	162.28	97.25	44.48	10.24	2239.26
-72.75	241.92	239.57	243.45	241.45	237.88	231.44	210.38	162.13	96.67	44.66	9.69	2239.26
-72.65	240.89	240.33	244.41	242.81	242.99	231.01	207.75	160.32	94.20	44.06	9.74	2239.26
-72.55	240.35	237.65	242.55	243.56	238.43	233.44	212.86	159.71	96.85	43.89	10.68	2239.26
-72.45	242.47	242.10	239.94	241.72	236.67	232.87	212.17	161.98	94.53	44.24	9.30	2239.26
-72.35	241.55	244.26	241.79	239.80	234.17	234.19	208.72	157.60	96.22	43.29	9.96	2239.26
-72.25	243.59	243.66	240.79	242.54	241.37	233.03	211.40	165.45	97.22	43.83	9.35	2239.26
-72.15	242.83	242.50	243.42	240.03	239.77	231.31	214.34	163.03	94.77	44.12	10.41	2239.26
-72.05	244.86	239.67	240.95	242.01	240.56	231.56	209.38	160.17	93.90	44.00	10.57	2239.26
-71.95	242.15	241.04	245.18	241.89	238.55	233.03	205.22	159.56	93.14	42.94	7.86	2239.26
-71.85	241.86	242.99	242.04	242.79	238.85	231.65	210.89	167.26	99.24	46.55	8.69	2239.26
-71.75	240.79	242.61	242.85	245.33	241.26	231.12	212.83	164.39	98.06	43.47	9.91	2239.26
-71.65	239.99	241.82	239.79	242.86	241.58	226.53	210.07	166.51	97.97	43.65	10.96	2239.26
-71.55	241.71	241.90	243.15	242.03	243.83	229.03	209.79	164.70	95.62	44.78	9.91	2239.26
-71.45	239.72	240.17	243.12	242.78	238.78	230.39	213.80	166.05	94.80	45.07	9.74	2239.26
-71.35	244.68	242.83	242.27	240.92	239.47	227.60	210.17	161.37	97.16	43.59	10.68	2239.26
-71.25	240.43	243.58	242.11	240.95	243.51	231.62	214.31	167.56	94.89	44.95	9.24	2239.26
-71.15	243.21	244.17	243.71	243.24	237.19	229.32	207.95	163.94	94.38	44.24	10.57	2239.26
-71.05	241.96	244.79	242.96	241.47	241.82	233.12	210.17	158.20	94.86	42.58	9.08	2239.26
-70.95	241.34	241.15	242.32	243.00	238.58	233.23	211.40	158.36	96.13	43.23	10.90	2239.26
-70.85	242.88	243.12	241.49	244.74	241.85	230.92	210.61	156.54	93.44	45.01	9.13	2239.26
-70.75	238.79	243.36	240.31	243.43	237.84	230.64	210.45	160.62	94.35	46.32	10.46	2239.26
-70.65	243.36	242.60	240.82	244.31	240.46	228.71	210.45	158.96	97.22	46.20	9.47	2239.26
-70.55	243.53	243.13	244.30	241.18	244.10	232.19	210.30	162.28	96.34	44.00	10.30	2239.26
-70.45	243.30	241.36	245.79	241.77	240.63	233.78	207.64	165.45	96.76	46.08	10.07	2239.26
-70.35	240.44	241.61	244.21	241.94	238.23	227.89	209.20	168.62	96.58	43.65	9.96	2239.26

Midpt.	11000	2700	910	240	84	26	8.8	2.7	0.92	0.31	Volume	
-70.25	244.10	244.80	244.07	245.60	239.30	229.76	207.34	158.20	95.25	41.75	9.13	2239.26
-70.15	244.79	240.29	243.00	242.61	237.52	236.12	209.79	161.83	99.99	44.00	8.08	2239.26
-70.05	243.41	243.03	241.60	244.16	238.72	229.99	210.97	158.81	97.70	46.38	9.63	2239.26
-69.95	243.96	240.13	245.48	242.12	239.20	225.80	208.97	164.24	98.18	44.12	8.69	2239.26
-69.85	244.07	241.67	243.78	242.83	240.01	231.76	206.72	158.81	93.62	45.13	9.69	2239.26
-69.75	242.98	241.73	243.60	243.98	238.35	233.64	208.00	154.58	95.80	44.48	9.41	2239.26
-69.65	242.10	242.69	242.43	240.39	239.97	225.96	209.66	155.49	96.04	43.17	8.91	2239.26
-69.55	245.19	245.74	241.03	243.31	243.23	230.23	212.91	166.36	97.70	44.66	8.63	2239.26
-69.45	243.76	241.25	240.30	243.69	235.41	227.24	209.02	155.03	95.35	43.77	9.74	2239.26
-69.35	244.58	244.19	243.91	242.05	244.45	229.01	210.15	151.71	96.37	44.48	10.13	2239.26
-69.25	239.60	243.40	241.77	245.53	243.41	226.85	215.03	155.49	96.52	45.37	9.47	2239.26
-69.15	240.95	242.70	242.04	243.08	238.33	231.31	210.45	160.47	97.58	43.11	9.96	2239.26
-69.05	240.14	241.28	242.78	243.96	241.37	228.07	208.13	160.62	96.55	44.78	9.74	2239.26
-68.95	245.36	245.22	244.42	242.87	244.25	233.14	205.37	159.41	95.53	42.52	9.58	2239.26
-68.85	239.81	241.60	242.44	243.44	240.17	234.26	214.49	161.68	94.74	43.35	8.86	2239.26
-68.75	243.78	241.47	243.98	244.03	242.81	224.96	211.78	159.41	95.25	39.68	9.24	2239.26
-68.65	241.35	240.02	242.65	247.51	241.82	233.40	218.14	156.85	96.40	43.17	8.80	2239.26
-68.55	243.26	242.66	240.93	244.95	242.39	229.71	213.49	156.54	97.55	42.40	8.63	2239.26
-68.45	244.21	242.66	242.28	239.14	240.86	231.14	211.99	159.41	93.02	42.70	9.19	2239.26
-68.35	240.69	241.84	243.29	242.29	238.08	232.46	206.19	160.62	94.80	43.06	8.80	2239.26
-68.25	241.22	243.15	240.56	241.02	239.18	230.39	212.45	171.34	95.98	45.96	9.13	2239.26
-68.15	241.58	242.73	245.66	241.76	237.44	230.35	208.54	152.92	97.01	41.87	8.75	2239.26
-68.05	237.91	244.24	244.77	242.30	243.60	226.60	210.28	163.03	96.37	46.14	9.74	2239.26
-67.95	241.44	244.42	242.37	242.17	238.21	230.73	212.01	160.17	94.32	45.90	9.02	2239.26
-67.85	241.11	241.76	244.38	240.07	237.93	230.21	211.04	165.90	96.34	42.17	9.08	2239.26
-67.75	239.99	243.99	246.22	241.44	237.71	228.12	208.10	155.18	98.12	45.01	9.30	2239.26
-67.65	244.32	245.42	243.28	239.81	238.55	231.39	212.70	165.15	96.64	46.26	8.80	2239.26
-67.55	242.17	241.22	243.83	241.81	240.31	233.39	214.47	160.02	93.53	43.41	9.74	2239.26
-67.45	242.33	240.79	239.69	244.77	240.76	229.73	209.76	159.11	94.59	44.18	8.91	2239.26
-67.35	241.21	244.43	241.06	238.78	239.77	227.07	209.71	163.19	94.68	46.14	9.80	2239.26
-67.25	242.24	239.32	242.91	242.64	237.04	225.82	208.44	149.60	95.28	47.50	8.97	2239.26
-67.15	242.49	242.86	240.99	241.62	238.13	233.89	212.63	159.56	95.71	44.42	10.90	2239.26
-67.05	241.47	243.40	242.43	240.56	239.24	230.39	211.12	166.05	97.94	44.95	9.24	2239.26
-66.95	242.95	242.78	245.95	242.41	235.73	232.64	211.53	160.02	99.81	44.48	9.52	2239.26
-66.85	243.20	244.07	241.46	241.50	238.13	230.33	213.65	151.11	95.95	45.07	9.30	2239.26
-66.75	244.55	242.02	245.38	242.31	239.54	232.64	207.21	162.13	99.21	43.29	9.80	2239.26
-66.65	240.80	243.14	243.57	243.06	245.73	235.60	211.55	172.39	94.44	44.60	9.74	2239.26
-66.55	245.18	242.53	243.42	245.14	238.20	234.10	207.64	156.09	96.19	43.41	9.47	2239.26
-66.45	240.69	244.25	239.71	242.46	245.39	231.19	209.97	153.83	92.96	47.27	10.35	2239.26
-66.35	243.41	241.64	244.35	242.42	239.96	230.81	207.29	168.77	100.93	44.54	7.47	2239.26
-66.25	242.76	243.88	241.41	242.87	241.55	231.96	213.11	154.13	96.16	42.46	9.85	2239.26
-66.15	242.28	242.62	243.16	240.10	234.82	236.49	212.75	159.41	96.04	43.17	9.19	2239.26
-66.05	243.32	240.99	242.85	242.57	237.71	234.42	211.96	162.13	96.16	44.54	9.96	2239.26
-65.95	241.33	242.51	241.02	240.33	239.08	233.83	209.66	157.00	92.96	44.42	9.35	2239.26
-65.85	242.51	240.03	244.91	242.37	242.56	231.78	211.35	158.81	95.98	43.59	9.41	2239.26

Midpt.	11000	2700	910	240	84	26	8.8	2.7	0.92	0.31	Volume	
-65.75	242.50	241.11	242.69	243.17	238.98	233.87	208.61	151.86	92.90	44.24	9.19	2239.26
-65.65	242.06	242.62	238.86	241.65	241.53	229.65	205.01	168.77	94.20	46.55	9.19	2239.26
-65.55	242.99	239.42	243.25	242.96	238.45	233.17	206.70	163.64	97.61	44.06	10.02	2239.26
-65.45	240.74	245.26	242.72	241.67	240.56	233.62	210.74	158.51	96.70	45.61	9.85	2239.26
-65.35	243.14	243.85	241.85	244.72	236.28	232.15	207.77	161.53	95.47	43.23	8.97	2239.26
-65.25	241.11	245.45	243.95	243.24	239.99	232.37	211.02	162.43	95.16	42.52	8.80	2239.26
-65.15	240.26	243.22	242.65	243.97	239.37	231.74	208.36	159.26	96.46	46.73	9.96	2239.26
-65.05	238.73	245.69	243.81	242.46	238.20	232.37	206.83	171.79	96.92	43.77	10.24	2239.26
-64.95	242.27	242.29	241.08	244.83	236.52	228.89	209.00	160.77	95.56	46.38	10.74	2239.26
-64.85	243.69	241.61	242.21	241.99	244.94	230.62	210.33	161.98	96.55	42.40	10.13	2239.26
-64.75	240.73	240.79	240.02	241.19	240.36	229.46	209.53	157.90	94.59	42.11	9.58	2239.26
-64.65	243.53	244.91	242.38	239.50	234.96	227.57	201.79	168.02	97.07	46.73	9.41	2239.26
-64.55	244.71	243.48	244.40	242.85	240.22	229.01	209.05	157.15	95.41	45.01	9.80	2239.26
-64.45	241.86	244.07	242.50	243.70	243.28	234.65	210.51	156.54	96.07	44.54	9.35	2239.26
-64.35	242.51	240.29	247.69	243.43	239.07	229.67	211.40	156.39	96.76	43.11	9.63	2239.26
-64.25	242.08	243.83	239.89	243.18	241.55	226.62	211.91	162.43	96.43	44.18	8.69	2239.26
-64.15	244.23	242.90	243.85	242.89	237.37	229.40	207.26	156.24	96.10	43.59	10.68	2239.26
-64.05	242.73	243.31	245.22	242.80	237.64	235.06	209.07	156.09	97.43	44.12	10.52	2239.26
-63.95	244.14	245.07	243.49	241.89	241.78	230.80	211.22	157.15	94.59	43.17	9.52	2239.26
-63.85	242.95	242.16	243.15	245.09	238.21	234.98	212.14	157.75	96.28	42.76	9.69	2239.26
-63.75	239.71	243.62	242.12	240.46	238.82	232.60	208.74	152.47	92.36	42.82	9.52	2239.26
-63.65	240.08	243.13	243.05	241.68	244.23	229.62	210.97	166.05	95.25	43.23	9.47	2239.26
-63.55	241.19	243.42	241.95	241.79	242.30	231.15	213.19	164.09	96.16	43.89	10.79	2239.26
-63.45	241.50	243.67	242.21	241.99	239.70	230.30	206.49	152.62	97.37	42.17	10.07	2239.26
-63.35	243.96	246.55	243.95	241.00	241.25	227.32	214.49	151.11	94.71	44.72	9.19	2239.26
-63.25	241.04	243.17	243.93	241.96	243.81	227.49	211.83	153.98	92.48	43.17	9.69	2239.26
-63.15	241.49	243.51	242.51	244.45	242.04	232.35	204.37	159.26	99.45	42.52	11.24	2239.26
-63.05	242.22	243.77	243.59	244.72	240.51	229.37	211.88	162.88	96.46	44.24	8.47	2239.26
-62.95	242.12	245.60	245.94	240.86	239.13	233.65	211.78	161.07	99.42	45.55	10.57	2239.26
-62.85	241.39	243.24	244.81	242.17	241.08	229.92	211.53	166.96	96.16	43.06	9.91	2239.26
-62.75	243.72	243.98	242.56	243.10	241.72	229.19	206.83	156.54	93.93	43.17	9.47	2239.26
-62.65	242.48	244.55	244.99	243.63	238.92	233.21	208.67	155.94	95.53	41.57	10.35	2239.26
-62.55	242.51	243.74	241.86	241.26	237.51	230.32	213.29	172.85	99.36	46.61	7.97	2239.26
-62.45	243.50	239.30	240.71	242.36	239.30	236.80	205.14	163.03	95.04	47.03	10.46	2239.26
-62.35	247.53	241.73	244.58	242.18	238.53	231.37	209.56	158.20	94.59	45.25	9.96	2239.26
-62.25	244.76	241.60	242.52	243.55	239.25	236.12	207.98	162.28	94.92	45.31	9.96	2239.26
-62.15	245.61	241.92	247.84	243.60	241.89	232.14	209.30	155.34	94.47	46.02	10.07	2239.26
-62.05	243.85	242.80	244.55	242.73	245.06	230.81	212.73	171.79	93.84	44.18	8.63	2239.26
-61.95	241.80	242.14	243.29	243.55	239.50	229.71	209.51	154.43	97.10	46.08	10.07	2239.26
-61.85	238.04	241.91	241.93	244.15	238.38	226.01	209.79	161.22	94.26	41.28	9.85	2239.26
-61.75	240.62	243.54	240.65	243.74	239.20	230.58	208.23	164.85	94.71	46.67	9.96	2239.26
-61.65	243.10	243.33	244.47	240.84	238.75	231.23	213.70	166.36	92.60	43.00	10.96	2239.26
-61.55	245.02	240.76	241.73	243.49	236.74	230.67	205.91	164.85	94.65	41.63	9.13	2239.26
-61.45	243.51	244.06	243.79	243.96	237.32	232.48	213.42	163.79	94.17	42.64	8.58	2239.26
-61.35	244.28	243.95	241.45	245.05	234.72	228.74	211.94	156.54	95.89	42.70	10.63	2239.26

Midpt.	11000	2700	910	240	84	26	8.8	2.7	0.92	0.31	Volume	
-61.25	240.31	244.87	243.05	240.97	240.63	231.80	215.28	153.98	92.24	43.59	10.02	2239.26
-61.15	240.87	240.97	241.55	242.95	237.21	233.14	210.30	158.81	93.90	47.44	10.30	2239.26
-61.05	241.98	243.94	240.95	241.45	239.15	230.40	212.91	160.77	94.53	46.14	8.69	2239.26
-60.95	242.44	242.66	242.87	245.28	239.81	233.37	213.44	163.03	90.97	44.00	9.52	2239.26
-60.85	241.33	243.93	243.66	242.74	238.36	233.69	207.49	159.56	93.71	45.19	9.30	2239.26
-60.75	240.11	243.41	243.24	240.90	241.75	229.78	209.20	162.73	95.62	43.35	9.58	2239.26
-60.65	240.90	244.84	240.70	241.01	242.52	235.37	205.55	159.71	92.69	45.96	9.35	2239.26
-60.55	239.36	241.37	243.05	242.73	242.10	231.87	213.98	165.15	92.72	45.01	10.35	2239.26
-60.45	242.84	241.57	243.62	244.94	238.46	232.55	210.25	163.94	95.07	43.83	9.58	2239.26
-60.35	244.38	241.46	244.89	240.37	237.26	230.37	211.71	158.51	94.92	43.83	10.57	2239.26
-60.25	244.01	242.31	244.55	241.22	238.53	230.30	208.79	156.85	94.65	46.08	11.07	2239.26
-60.15	242.30	245.00	243.05	242.36	237.02	233.80	211.30	156.85	97.73	45.07	9.19	2239.26
-60.05	243.94	242.74	242.75	244.35	242.41	233.33	210.17	162.88	91.93	45.31	10.79	2239.26
-59.95	243.04	240.93	243.97	244.02	240.91	234.67	210.35	166.81	93.84	45.37	10.35	2239.26
-59.85	243.25	243.04	243.22	243.72	239.08	228.46	206.83	162.28	92.93	43.94	9.02	2239.26
-59.75	242.02	243.84	242.45	245.21	239.67	231.24	210.68	159.41	93.81	44.89	10.63	2239.26
-59.65	242.82	245.92	243.57	244.68	243.24	234.42	215.69	145.37	99.30	41.75	9.74	2239.26
-59.55	242.52	242.58	242.62	240.24	241.47	235.28	208.69	160.02	94.20	40.74	10.52	2239.26
-59.45	243.23	244.50	243.26	246.11	240.29	232.06	209.89	163.19	94.77	41.22	9.96	2239.26
-59.35	244.28	243.67	241.72	244.75	242.86	234.40	209.48	168.62	95.95	46.08	10.74	2239.26
-59.25	245.69	243.85	244.71	243.43	241.99	232.53	212.55	160.62	98.42	43.23	9.08	2239.26
-59.15	243.74	241.69	245.91	242.83	241.26	231.33	213.42	152.77	97.37	45.84	10.30	2239.26
-59.05	244.06	241.83	242.20	244.51	242.89	230.73	209.71	159.71	97.40	45.31	10.41	2239.26
-58.95	240.53	242.63	244.48	243.83	241.21	228.48	208.00	162.13	94.95	47.62	9.69	2239.26
-58.85	244.94	243.40	244.35	241.80	238.72	229.35	208.82	163.03	95.25	47.62	10.57	2239.26
-58.75	242.83	244.69	241.85	244.26	242.00	226.46	207.06	157.45	97.55	44.18	9.58	2239.26
-58.65	244.05	243.89	244.56	242.49	238.61	233.83	209.79	164.70	100.39	43.06	9.58	2239.26
-58.55	244.34	245.11	242.22	241.81	236.45	228.19	206.67	172.09	95.62	43.23	10.02	2239.26
-58.45	244.86	240.12	244.20	244.80	237.61	231.55	208.38	153.68	98.03	42.28	11.07	2239.26
-58.35	241.41	240.32	243.05	244.48	242.35	233.83	212.45	149.30	92.93	44.78	9.85	2239.26
-58.25	246.30	241.77	244.58	242.99	242.49	229.58	209.23	158.36	98.49	41.87	10.02	2239.26
-58.15	243.83	241.84	243.64	243.22	235.28	232.83	216.79	150.51	96.82	44.24	10.07	2239.26
-58.05	241.77	245.06	241.69	242.36	243.08	232.69	213.49	162.28	94.83	46.91	10.30	2239.26
-57.95	243.21	243.95	243.64	241.01	240.29	231.48	211.32	161.68	97.88	41.93	10.02	2239.26
-57.85	245.67	243.03	243.05	244.89	241.89	227.80	206.57	161.83	96.55	46.38	11.46	2239.26
-57.75	242.39	241.90	245.17	243.17	243.39	227.94	212.57	161.22	95.10	44.48	11.13	2239.26
-57.65	247.43	243.91	244.77	243.93	240.31	231.65	211.45	159.86	96.49	43.94	9.69	2239.26
-57.55	242.12	241.91	244.84	242.52	241.65	232.56	211.45	163.79	95.47	43.17	9.96	2239.26
-57.45	241.58	241.77	243.68	239.65	237.74	230.92	214.62	163.79	96.92	45.66	9.24	2239.26
-57.35	243.92	243.86	243.71	241.25	239.24	232.26	208.74	160.47	95.35	44.89	10.79	2239.26
-57.25	240.37	241.04	240.18	244.12	241.70	236.08	208.41	160.47	95.44	43.83	10.41	2239.26
-57.15	243.91	243.40	242.81	243.43	241.08	230.14	211.32	156.24	95.53	43.00	10.07	2239.26
-57.05	242.36	241.22	247.11	241.70	237.64	228.48	206.93	158.81	93.44	43.77	10.41	2239.26
-56.95	242.34	244.40	244.91	241.12	241.73	230.53	210.45	171.94	94.74	43.77	10.63	2239.26
-56.85	242.30	242.83	242.05	242.70	244.55	231.83	214.69	156.54	95.80	44.60	10.52	2239.26

Midpt.	11000	2700	910	240	84	26	8.8	2.7	0.92	0.31	Volume	
-56.75	245.79	245.69	243.34	239.27	240.54	229.21	208.77	160.62	96.70	42.52	11.46	2239.26
-56.65	241.18	244.75	243.35	244.71	240.66	236.40	212.68	158.66	94.71	47.62	10.57	2239.26
-56.55	240.25	241.81	245.57	241.85	240.24	235.89	209.76	165.75	97.16	41.34	12.73	2239.26
-56.45	241.90	240.61	244.01	242.09	238.97	232.35	211.78	156.54	97.28	43.65	10.46	2239.26
-56.35	245.52	243.11	241.13	243.44	238.92	232.46	215.23	168.02	97.55	44.06	10.24	2239.26
-56.25	243.69	245.87	243.80	241.32	236.84	232.44	205.40	160.32	98.45	46.20	8.91	2239.26
-56.15	241.10	243.31	243.10	242.29	239.25	232.10	213.14	157.60	94.44	43.41	10.18	2239.26
-56.05	243.58	243.40	243.81	243.70	236.48	231.85	210.79	162.13	92.72	43.11	10.85	2239.26
-55.95	247.25	243.03	244.67	242.21	240.26	233.17	213.03	159.11	92.69	45.84	9.96	2239.26
-55.85	241.76	243.79	241.73	242.90	244.17	230.87	207.59	154.13	95.59	43.94	10.68	2239.26
-55.75	240.06	245.36	243.39	243.95	237.34	235.03	209.92	168.32	96.01	44.60	11.40	2239.26
-55.65	239.45	244.67	245.68	243.13	237.78	232.05	208.46	161.53	96.40	42.64	11.62	2239.26
-55.55	242.88	244.76	240.56	242.25	242.15	232.40	213.62	164.39	94.50	44.48	10.90	2239.26
-55.45	240.80	242.66	245.05	241.90	243.61	234.49	210.89	159.56	95.41	44.06	11.24	2239.26
-55.35	241.58	241.34	243.54	242.93	240.51	232.23	213.01	157.30	95.16	45.49	10.13	2239.26
-55.25	240.84	242.90	241.36	243.53	241.08	233.81	208.59	162.88	95.10	40.21	10.79	2239.26
-55.15	239.92	243.86	245.11	243.61	238.77	231.74	208.92	154.28	95.98	43.71	10.30	2239.26
-55.05	242.63	240.40	245.66	245.23	239.18	230.10	210.53	162.13	96.25	44.06	9.30	2239.26
-54.95	243.29	244.39	243.85	242.80	240.86	226.37	218.25	160.47	94.41	46.08	10.30	2239.26
-54.85	242.52	243.20	243.50	244.44	242.93	233.80	209.79	162.88	98.91	43.77	11.29	2239.26
-54.75	242.50	241.92	244.99	245.61	244.23	233.10	212.32	162.13	94.02	45.13	10.24	2239.26
-54.65	241.79	242.85	243.94	241.51	239.44	235.53	210.38	161.98	96.85	42.76	9.85	2239.26
-54.55	242.71	242.28	243.95	240.19	239.59	235.51	212.80	162.43	96.82	44.66	10.68	2239.26
-54.45	244.01	245.43	245.43	243.51	242.29	231.06	209.00	159.56	94.08	42.23	10.63	2239.26
-54.35	243.37	244.52	243.94	243.74	241.31	231.55	210.43	164.09	93.71	42.40	10.57	2239.26
-54.25	243.66	243.53	243.89	243.55	243.18	235.46	212.11	157.45	94.95	45.49	10.57	2239.26
-54.15	243.57	245.56	242.82	243.79	237.61	229.17	213.06	157.60	93.59	45.72	10.30	2239.26
-54.05	241.79	243.98	242.27	244.76	238.98	231.17	203.02	153.68	91.69	45.13	10.07	2239.26
-53.95	239.81	242.30	242.49	242.94	239.22	227.99	211.07	160.47	100.18	43.47	10.18	2239.26
-53.85	240.37	243.76	243.36	244.51	242.17	236.62	210.17	157.90	95.25	47.15	9.91	2239.26
-53.75	242.82	245.11	241.26	245.23	238.82	233.98	210.30	153.83	95.35	45.07	10.68	2239.26
-53.65	241.51	245.75	244.72	242.46	242.59	228.92	209.92	161.37	98.09	43.11	9.02	2239.26
-53.55	244.90	243.00	242.53	239.73	239.57	230.15	210.99	169.07	96.22	42.28	9.52	2239.26
-53.45	239.45	241.40	243.19	242.36	238.97	228.85	207.87	160.02	98.36	44.60	10.46	2239.26
-53.35	240.43	243.99	241.59	244.25	236.62	229.58	209.38	160.17	96.85	41.81	10.35	2239.26
-53.25	244.29	245.90	244.92	241.17	240.11	228.21	213.90	161.98	97.88	44.36	11.24	2239.26
-53.15	241.37	241.96	245.57	242.96	239.84	231.08	209.82	164.85	95.38	43.65	11.07	2239.26
-53.05	246.77	240.74	245.58	243.08	241.42	231.78	207.31	157.60	94.41	42.23	9.80	2239.26
-52.95	242.18	245.42	242.55	244.21	244.15	233.12	213.37	160.17	95.38	45.25	11.18	2239.26
-52.85	244.70	244.70	244.75	244.95	240.33	233.21	206.24	150.66	95.28	42.88	9.74	2239.26
-52.75	243.50	240.82	243.91	243.61	240.16	232.64	213.03	156.54	98.15	43.17	11.07	2239.26
-52.65	243.09	244.31	245.65	244.67	238.01	234.33	211.45	170.58	95.31	42.52	10.41	2239.26
-52.55	245.89	243.29	244.55	243.69	240.61	234.08	205.88	154.88	94.20	44.48	11.01	2239.26
-52.45	244.35	241.72	242.75	242.83	242.44	233.06	212.63	155.49	96.13	49.22	10.30	2239.26
-52.35	243.10	243.14	245.64	243.93	238.33	229.57	211.65	166.05	95.74	46.73	9.80	2239.26

Midpt.	11000	2700	910	240	84	26	8.8	2.7	0.92	0.31	Volume	
-52.25	244.27	248.37	244.72	242.32	241.65	234.03	211.83	157.15	96.76	44.83	11.07	2239.26
-52.15	241.71	244.03	240.41	240.00	239.54	234.40	206.26	156.09	98.82	43.59	10.30	2239.26
-52.05	241.71	242.99	243.67	244.76	238.78	233.01	211.20	160.62	95.19	45.84	10.24	2239.26
-51.95	242.31	245.48	245.95	244.57	239.96	233.42	207.80	166.05	97.79	42.28	10.35	2239.26
-51.85	241.65	243.79	242.87	242.61	239.30	234.28	210.10	163.03	94.83	43.94	12.12	2239.26
-51.75	244.00	243.69	242.60	243.70	242.41	232.83	211.04	157.45	97.61	43.41	9.91	2239.26
-51.65	241.82	244.70	245.68	242.72	239.50	230.81	208.10	156.85	95.50	44.89	10.74	2239.26
-51.55	244.18	249.12	242.25	241.10	242.17	231.14	205.42	159.41	96.04	44.00	11.79	2239.26
-51.45	244.96	246.45	244.25	244.40	241.60	232.24	209.13	166.66	97.40	43.83	11.01	2239.26
-51.35	242.85	240.81	245.01	241.43	237.17	232.83	209.89	158.20	95.41	43.06	11.57	2239.26
-51.25	243.91	244.34	244.89	243.06	242.20	227.42	214.01	161.53	97.01	40.86	8.41	2239.26
-51.15	242.23	242.97	242.21	243.68	243.14	229.74	211.73	157.45	94.89	46.02	9.52	2239.26
-51.05	246.27	245.86	241.89	244.82	242.00	230.26	206.75	154.58	97.64	44.06	10.30	2239.26
-50.95	242.83	242.05	243.57	243.78	242.32	227.30	210.53	161.07	98.82	43.00	10.74	2239.26
-50.85	244.10	246.32	244.86	242.51	239.39	237.10	213.55	152.17	96.58	44.54	8.91	2239.26
-50.75	241.86	242.05	241.65	243.68	239.76	225.78	206.83	164.70	96.52	45.96	9.91	2239.26
-50.65	242.90	243.10	240.84	245.32	239.79	230.96	207.13	160.47	93.65	44.89	9.80	2239.26
-50.55	241.23	241.69	242.32	243.89	241.06	229.53	210.22	157.90	100.27	44.48	9.80	2239.26
-50.45	242.42	242.08	245.03	245.90	243.39	232.69	211.32	157.45	98.33	44.48	9.19	2239.26
-50.35	243.00	246.32	244.30	242.69	240.86	228.80	209.64	163.03	93.81	46.02	11.01	2239.26
-50.25	243.92	246.33	248.65	240.84	239.79	234.14	209.71	162.13	94.71	43.53	9.69	2239.26
-50.15	243.05	246.41	243.55	241.40	241.31	225.80	208.74	158.20	93.53	45.90	10.30	2239.26
-50.05	243.34	242.75	243.25	242.31	238.53	229.23	214.01	159.56	95.01	42.11	10.85	2239.26
-49.95	243.37	242.78	244.62	243.02	243.93	232.71	210.28	171.49	94.41	42.11	9.80	2239.26
-49.85	243.32	246.20	244.31	245.60	240.12	234.01	213.49	165.60	95.62	43.53	9.96	2239.26
-49.75	242.46	243.52	244.60	244.30	241.01	230.10	211.83	152.92	97.34	44.78	10.57	2239.26
-49.65	244.64	244.84	243.67	240.30	238.25	227.78	210.43	161.83	94.50	44.36	10.24	2239.26
-49.55	241.42	245.24	242.07	246.15	236.67	236.10	214.52	165.30	95.62	44.83	9.85	2239.26
-49.45	243.58	242.78	244.64	242.25	244.08	230.49	210.12	155.34	95.98	41.93	11.01	2239.26
-49.35	243.00	244.25	245.24	243.43	240.66	232.10	213.03	158.20	96.22	43.29	10.46	2239.26
-49.25	243.45	244.96	246.94	246.75	241.58	231.03	205.29	161.98	92.42	43.29	10.30	2239.26
-49.15	241.50	244.97	243.68	242.71	240.19	233.49	213.80	164.39	96.01	42.70	10.74	2239.26
-49.05	243.17	244.79	242.69	243.15	239.94	234.08	211.35	163.79	96.64	43.65	10.79	2239.26
-48.95	244.00	242.87	245.19	242.84	240.98	234.06	214.47	160.77	91.66	43.29	10.30	2239.26
-48.85	242.48	245.06	241.33	242.93	237.39	235.01	207.36	154.28	93.90	44.00	9.74	2239.26
-48.75	246.27	244.91	243.74	240.70	240.11	234.55	208.84	159.11	94.74	43.94	10.24	2239.26
-48.65	244.04	242.12	246.65	243.66	236.12	230.89	204.86	164.54	94.98	43.59	10.52	2239.26
-48.55	243.19	242.52	244.38	246.29	241.97	233.03	211.81	158.81	97.34	42.64	9.63	2239.26
-48.45	242.87	247.60	244.65	246.10	238.83	233.37	212.70	159.41	95.77	40.80	10.46	2239.26
-48.35	242.57	242.15	242.79	240.15	242.37	228.67	212.93	158.96	98.67	44.48	9.74	2239.26
-48.25	244.68	246.37	246.67	242.80	238.11	232.98	211.76	163.03	96.07	43.17	10.46	2239.26
-48.15	240.74	243.66	244.28	241.97	243.11	232.48	207.67	161.68	95.10	45.19	10.85	2239.26
-48.05	241.82	240.53	242.06	245.74	238.43	234.60	210.63	174.51	95.53	43.35	10.85	2239.26
-47.95	244.08	242.77	242.76	243.09	242.41	234.60	211.73	158.20	95.68	40.51	10.74	2239.26
-47.85	243.86	240.76	245.68	242.13	242.12	230.23	208.84	158.05	95.16	43.35	9.74	2239.26

Midpt.	11000	2700	910	240	84	26	8.8	2.7	0.92	0.31	Volume	
-47.75	243.06	245.76	242.04	244.53	240.90	230.48	207.01	161.83	94.14	43.06	9.02	2239.26
-47.65	243.80	245.50	247.39	244.23	239.13	230.07	207.34	163.34	97.07	44.24	9.19	2239.26
-47.55	243.50	245.04	245.87	248.69	241.43	237.28	215.84	157.45	97.79	43.71	10.07	2239.26
-47.45	243.19	248.07	243.85	241.46	240.86	227.64	214.44	157.90	96.76	41.93	11.07	2239.26
-47.35	242.30	243.21	246.06	243.52	240.91	231.35	211.27	158.05	96.43	45.61	10.57	2239.26
-47.25	243.22	240.31	245.24	244.79	241.15	232.08	216.59	157.60	96.28	46.55	9.85	2239.26
-47.15	244.78	244.55	243.55	243.92	242.35	236.58	208.05	160.92	97.67	46.44	8.36	2239.26
-47.05	243.02	242.63	243.91	241.39	241.25	231.69	211.88	156.24	95.35	44.54	10.35	2239.26
-46.95	243.01	238.37	243.90	242.98	241.55	233.06	209.66	157.15	95.65	42.58	10.07	2239.26
-46.85	243.48	244.17	242.92	246.14	241.21	233.56	210.05	149.75	94.71	46.61	11.62	2239.26
-46.75	243.47	245.28	244.37	242.34	238.93	231.83	213.19	158.96	95.71	43.47	9.24	2239.26
-46.65	244.50	240.52	243.65	241.86	242.71	235.24	211.65	160.32	97.07	45.43	9.47	2239.26
-46.55	242.30	245.74	244.78	245.87	248.28	229.23	210.84	163.34	97.64	41.63	11.13	2239.26
-46.45	242.94	243.67	245.51	244.34	242.61	231.35	213.83	157.30	97.67	43.29	9.52	2239.26
-46.35	244.12	246.12	246.37	245.23	239.65	233.67	210.79	160.32	93.62	46.38	10.35	2239.26
-46.25	245.44	241.61	245.57	244.31	240.96	230.49	210.05	171.19	91.66	44.54	9.63	2239.26
-46.15	245.28	244.20	245.35	243.96	237.99	229.12	208.90	156.69	94.26	44.18	9.63	2239.26
-46.05	241.02	244.57	244.75	245.69	240.39	232.15	210.10	158.66	97.01	46.20	9.24	2239.26
-45.95	241.86	244.61	245.16	241.83	240.64	231.76	207.52	162.73	97.31	46.08	10.63	2239.26
-45.85	242.04	241.96	244.00	246.13	239.97	230.87	207.18	162.28	98.12	44.24	9.96	2239.26
-45.75	245.39	246.27	241.78	245.20	236.60	230.12	211.42	163.34	98.42	44.95	9.24	2239.26
-45.65	241.92	245.30	244.07	244.78	238.45	233.08	209.20	154.43	97.37	42.23	9.58	2239.26
-45.55	244.99	243.98	245.49	244.86	247.29	229.37	211.12	160.92	97.64	46.14	9.30	2239.26
-45.45	245.52	246.62	243.17	245.13	238.18	231.69	212.14	163.03	94.98	42.28	10.74	2239.26
-45.35	241.03	244.89	245.11	244.02	241.16	231.73	210.10	153.22	96.16	43.47	9.35	2239.26
-45.25	243.97	246.40	244.32	246.66	243.14	231.89	209.25	158.51	98.27	42.94	9.58	2239.26
-45.15	245.71	246.46	246.08	243.08	237.71	231.69	210.35	156.54	97.10	43.35	10.63	2239.26
-45.05	242.81	245.83	244.01	245.55	243.71	231.74	213.44	164.24	93.81	45.01	10.63	2239.26
-44.95	244.74	242.17	243.35	242.25	243.65	237.72	207.67	151.86	96.13	44.72	9.52	2239.26
-44.85	240.73	245.34	243.61	243.37	241.80	231.65	208.10	148.24	96.52	41.57	9.35	2239.26
-44.75	247.30	248.42	244.85	244.26	242.52	231.14	208.92	164.39	97.04	41.93	9.47	2239.26
-44.65	240.80	246.39	244.95	245.19	242.61	234.06	210.81	158.66	94.62	41.04	9.58	2239.26
-44.55	243.22	242.99	246.75	242.55	240.16	233.30	211.71	155.18	96.34	43.11	8.97	2239.26
-44.45	242.96	244.57	243.48	242.92	241.21	228.01	209.82	160.92	100.06	45.84	10.96	2239.26
-44.35	242.94	245.29	244.81	243.81	242.49	234.67	215.87	153.68	95.07	40.68	9.63	2239.26
-44.25	243.73	245.37	244.79	241.03	245.93	229.90	210.33	171.04	96.16	44.42	9.80	2239.26
-44.15	242.15	243.97	245.14	246.98	242.47	229.08	213.06	171.34	94.14	44.12	9.74	2239.26
-44.05	244.27	241.47	244.51	244.39	240.85	234.05	210.74	157.15	100.42	43.11	10.02	2239.26
-43.95	239.42	242.32	243.45	242.18	237.79	229.80	212.47	155.94	99.51	44.00	10.46	2239.26
-43.85	242.35	243.85	243.22	243.54	237.47	234.76	207.39	160.92	99.93	46.38	10.57	2239.26
-43.75	245.57	245.09	245.55	241.62	241.80	230.24	212.22	164.39	96.34	45.72	9.30	2239.26
-43.65	248.38	244.81	243.15	242.32	240.48	232.05	210.53	159.41	93.50	45.90	9.24	2239.26
-43.55	242.21	244.64	243.69	245.64	239.77	227.60	215.69	163.79	98.94	47.44	9.41	2239.26
-43.45	245.04	245.29	245.38	243.10	245.73	231.23	210.76	159.56	94.92	42.52	9.80	2239.26
-43.35	242.29	242.74	242.74	242.93	243.90	230.12	208.28	161.07	98.39	41.28	8.08	2239.26

Midpt.	11000	2700	910	240	84	26	8.8	2.7	0.92	0.31	Volume	
-43.25	245.36	248.53	243.85	245.06	238.03	231.31	211.88	158.81	93.71	42.64	10.02	2239.26
-43.15	244.87	244.03	248.26	244.33	242.74	234.12	211.25	166.05	95.44	45.07	9.35	2239.26
-43.05	241.90	244.23	244.41	243.40	238.78	231.90	213.70	151.86	93.93	41.93	9.96	2239.26
-42.95	243.17	242.67	246.14	245.37	238.65	231.78	213.78	162.43	93.23	46.14	10.02	2239.26
-42.85	244.58	244.59	244.40	246.51	238.20	233.80	211.55	164.70	94.14	42.64	10.57	2239.26
-42.75	243.42	244.46	244.86	245.32	241.00	231.78	208.41	159.71	93.87	43.71	9.35	2239.26
-42.65	246.75	246.74	246.44	246.00	241.80	231.53	210.48	162.28	95.38	45.43	9.63	2239.26
-42.55	246.43	244.82	245.79	240.90	244.57	231.24	214.98	160.62	96.98	46.85	9.85	2239.26
-42.45	243.78	244.79	244.95	244.49	242.19	233.83	212.50	162.58	96.58	47.62	10.35	2239.26
-42.35	243.70	243.90	246.79	242.42	242.22	232.92	211.55	171.64	95.92	46.20	11.13	2239.26
-42.25	244.85	248.02	244.63	240.53	239.60	233.67	219.68	150.35	97.76	45.66	8.03	2239.26
-42.15	247.83	243.35	246.21	247.03	240.91	231.35	215.13	150.51	95.98	46.44	10.30	2239.26
-42.05	245.49	246.82	246.31	247.94	242.19	234.53	210.53	156.69	95.68	42.05	8.08	2239.26
-41.95	243.90	245.30	243.68	246.98	242.20	237.78	211.27	154.13	94.23	47.38	9.13	2239.26
-41.85	244.66	242.89	246.96	244.78	239.89	227.96	217.20	164.39	98.24	43.41	9.02	2239.26
-41.75	244.08	245.76	244.46	244.49	238.78	234.78	215.90	162.58	95.98	43.41	11.35	2239.26
-41.65	245.75	248.17	247.65	244.40	242.34	235.30	213.14	155.94	96.31	44.18	8.97	2239.26
-41.55	245.21	244.50	244.65	244.72	244.45	230.19	211.40	166.96	94.20	44.42	9.02	2239.26
-41.45	246.28	248.65	244.58	242.50	237.78	234.44	208.74	158.36	99.63	44.18	9.63	2239.26
-41.35	246.98	246.10	245.07	247.27	244.22	232.26	213.44	166.51	97.13	44.00	11.07	2239.26
-41.25	241.32	244.17	243.12	244.03	242.07	232.03	211.53	160.02	94.08	41.39	10.30	2239.26
-41.15	244.81	246.37	242.18	244.47	249.94	233.03	211.96	168.02	94.17	43.35	8.69	2239.26
-41.05	243.95	245.47	244.88	241.81	241.99	232.58	213.14	158.96	97.85	42.28	8.97	2239.26
-40.95	246.54	246.38	243.38	242.13	242.94	233.35	211.94	158.36	96.49	43.35	10.02	2239.26
-40.85	245.77	241.08	246.94	243.06	242.67	227.78	208.95	155.18	97.40	44.24	9.30	2239.26
-40.75	243.51	246.37	244.72	242.64	235.54	232.40	208.84	157.90	93.47	48.04	9.13	2239.26
-40.65	245.06	245.51	245.78	242.27	242.99	232.65	206.34	166.81	97.46	45.78	10.24	2239.26
-40.55	245.30	243.36	246.53	245.11	240.29	234.39	210.99	172.24	94.77	42.28	9.35	2239.26
-40.45	245.78	248.68	243.25	242.46	240.86	232.08	207.21	165.15	96.28	43.59	8.30	2239.26
-40.35	246.48	246.15	244.85	243.22	239.96	236.53	216.84	161.83	93.87	46.20	10.85	2239.26
-40.25	246.76	244.72	246.43	244.24	236.77	229.87	212.42	164.09	95.98	43.00	8.91	2239.26
-40.15	243.59	246.64	246.02	247.28	244.05	231.78	208.26	166.05	96.61	43.89	10.85	2239.26
-40.05	243.15	245.63	248.80	243.15	239.17	233.40	204.43	152.01	94.98	43.00	8.47	2239.26
-39.95	246.26	247.10	243.28	244.28	240.88	238.53	208.67	156.85	93.23	44.36	8.91	2239.26
-39.85	245.51	246.08	244.41	245.74	242.12	231.74	213.70	154.58	95.10	45.49	9.35	2239.26
-39.75	242.86	246.55	243.63	246.26	241.85	234.01	209.23	162.73	94.41	45.31	9.80	2239.26
-39.65	243.20	247.04	245.51	242.37	241.83	229.26	210.86	161.68	96.34	41.39	8.41	2239.26
-39.55	244.78	244.17	246.67	244.18	242.64	234.30	214.85	148.84	95.89	40.62	11.40	2239.26
-39.45	242.42	245.81	245.11	246.29	241.87	234.05	213.72	160.62	94.41	46.67	10.35	2239.26
-39.35	244.36	244.36	245.58	244.21	243.93	233.48	207.54	166.51	101.20	44.24	9.35	2239.26
-39.25	244.10	247.62	246.39	244.49	242.51	232.60	210.66	163.79	95.35	43.94	9.02	2239.26
-39.15	244.97	245.19	246.71	247.68	240.83	230.32	213.83	163.64	96.64	46.08	8.91	2239.26
-39.05	242.94	242.20	246.62	242.65	241.00	232.12	210.94	156.54	94.83	44.36	8.36	2239.26
-38.95	245.20	243.86	245.36	243.27	244.82	235.83	210.33	164.24	96.31	45.66	10.57	2239.26
-38.85	244.67	248.26	245.27	244.99	241.89	237.64	214.62	157.60	95.98	43.53	10.24	2239.26

Midpt.	11000	2700	910	240	84	26	8.8	2.7	0.92	0.31	Volume	
-38.75	246.11	245.97	246.05	245.85	242.24	232.71	210.15	154.88	95.04	45.43	9.13	2239.26
-38.65	243.22	245.60	244.78	244.81	242.82	230.96	212.22	165.45	97.04	46.08	10.41	2239.26
-38.55	245.92	246.27	243.93	242.79	245.89	232.67	209.36	157.90	96.13	45.37	8.80	2239.26
-38.45	243.89	245.10	246.31	243.65	243.93	232.19	211.55	166.51	97.34	47.44	9.58	2239.26
-38.35	246.42	247.12	244.59	247.72	242.00	233.03	209.99	162.43	97.64	46.02	9.13	2239.26
-38.25	245.87	247.38	243.90	246.98	241.13	231.87	208.69	163.79	98.21	42.64	9.19	2239.26
-38.15	244.31	246.69	245.81	241.58	244.92	235.21	211.53	166.36	96.76	46.14	8.58	2239.26
-38.05	245.73	245.94	245.39	243.89	239.18	235.15	203.53	168.17	95.41	44.54	10.07	2239.26
-37.95	245.95	247.45	244.19	244.20	243.81	231.35	208.92	163.19	95.80	43.59	10.63	2239.26
-37.85	246.21	244.53	244.24	245.09	246.43	230.60	214.08	164.54	93.08	44.24	9.30	2239.26
-37.75	247.34	246.90	244.41	245.28	240.63	233.23	208.84	162.88	96.34	43.83	10.13	2239.26
-37.65	245.46	244.53	245.81	246.75	243.01	232.49	209.18	161.07	96.95	46.38	9.58	2239.26
-37.55	243.90	244.89	245.91	244.11	241.08	229.62	210.25	156.09	98.00	47.09	9.35	2239.26
-37.45	244.81	245.12	245.01	245.27	246.45	232.96	208.84	164.09	96.95	44.30	9.02	2239.26
-37.35	245.31	243.94	242.64	246.43	245.76	232.71	212.22	165.30	93.23	45.25	8.41	2239.26
-37.25	245.76	243.68	246.00	244.13	241.37	231.98	210.05	164.85	97.28	43.83	10.85	2239.26
-37.15	243.27	245.69	244.79	245.32	240.96	236.98	210.33	160.17	95.44	44.48	9.58	2239.26
-37.05	247.63	245.08	243.02	246.94	241.92	233.89	213.75	160.62	99.42	45.55	10.52	2239.26
-36.95	245.48	246.16	244.85	244.15	245.17	233.40	209.25	163.34	96.49	44.00	9.74	2239.26
-36.85	245.02	244.39	246.50	244.09	239.55	231.98	213.44	161.53	95.86	43.06	9.52	2239.26
-36.75	246.88	245.30	246.26	247.99	239.84	233.90	206.52	168.17	97.07	45.37	10.85	2239.26
-36.65	246.01	245.23	245.31	243.29	244.33	230.78	211.04	160.92	100.42	43.71	9.91	2239.26
-36.55	245.34	249.47	247.61	245.84	241.31	234.26	208.82	159.56	99.42	44.06	8.63	2239.26
-36.45	242.06	242.32	247.09	242.51	242.44	228.82	211.42	160.77	95.92	44.48	8.58	2239.26
-36.35	246.68	247.53	245.02	245.81	244.52	232.60	207.54	161.83	96.73	42.64	8.58	2239.26
-36.25	245.17	247.92	247.30	246.92	243.18	236.01	212.70	164.70	95.62	46.02	9.35	2239.26
-36.15	246.27	247.82	245.06	245.30	242.82	233.05	211.76	158.66	100.36	44.78	9.19	2239.26
-36.05	244.67	246.95	244.46	245.52	247.02	233.99	211.81	170.88	97.73	44.42	10.68	2239.26
-35.95	250.43	247.51	244.69	242.94	243.13	237.40	211.73	165.15	98.42	46.26	7.47	2239.26
-35.85	245.71	247.42	246.45	246.11	241.03	228.96	213.72	153.83	94.92	45.84	9.24	2239.26
-35.75	244.10	247.86	247.41	246.90	244.47	236.89	210.53	158.96	96.43	46.32	8.58	2239.26
-35.65	245.74	248.15	245.25	247.64	242.34	235.24	211.17	155.18	98.12	47.80	10.63	2239.26
-35.55	243.44	245.32	244.75	248.26	245.44	235.53	210.38	163.03	96.01	44.48	9.69	2239.26
-35.45	248.43	245.60	247.54	245.86	244.60	236.39	216.25	161.53	94.86	45.49	8.97	2239.26
-35.35	245.72	248.42	245.24	245.04	239.55	236.05	211.25	167.26	95.68	45.01	9.69	2239.26
-35.25	248.67	247.06	246.29	244.48	244.42	229.26	215.92	169.53	98.79	44.54	10.02	2239.26
-35.15	245.12	245.27	246.09	246.21	244.50	236.35	215.36	161.98	98.97	45.66	9.02	2239.26
-35.05	248.30	243.00	248.12	245.46	246.45	231.81	212.55	164.85	95.68	45.19	9.02	2239.26
-34.95	248.90	248.06	250.26	247.40	242.17	232.12	211.78	151.86	97.70	43.06	10.02	2239.26
-34.85	250.02	248.79	247.00	247.45	240.81	231.48	211.94	153.98	94.38	46.26	8.58	2239.26
-34.75	252.00	248.51	247.77	249.93	239.60	234.14	210.05	160.17	98.09	44.60	9.58	2239.26
-34.65	248.76	248.61	247.24	247.60	243.53	235.06	207.01	163.49	92.84	41.45	9.13	2239.26
-34.55	248.79	246.67	245.74	244.21	242.61	234.67	210.12	160.02	95.25	47.03	8.80	2239.26
-34.45	247.88	247.11	249.44	246.38	241.21	229.37	210.12	158.96	97.61	45.37	8.30	2239.26
-34.35	245.92	246.91	246.87	247.27	240.98	233.92	210.86	162.28	96.73	45.37	9.41	2239.26

Midpt.	11000	2700	910	240	84	26	8.8	2.7	0.92	0.31	Volume	
-34.25	248.87	246.88	249.75	246.38	245.88	232.44	215.72	163.94	96.25	44.83	8.30	2239.26
-34.15	247.89	246.52	246.32	243.15	243.73	234.76	212.19	158.05	96.49	44.12	8.19	2239.26
-34.05	248.47	246.54	248.04	250.10	241.75	235.69	213.34	149.90	98.15	44.36	9.58	2239.26
-33.95	249.86	243.85	244.80	248.15	239.02	232.39	216.94	152.92	96.19	43.71	8.63	2239.26
-33.85	246.93	247.44	246.90	248.05	242.82	233.17	209.41	162.43	99.66	44.48	9.63	2239.26
-33.75	246.83	250.56	248.21	244.70	242.96	240.22	212.83	163.64	95.07	43.77	9.41	2239.26
-33.65	246.80	245.33	247.31	245.27	241.43	236.67	213.06	165.00	98.12	44.12	11.01	2239.26
-33.55	247.74	247.61	249.38	244.82	245.61	234.96	213.21	148.09	98.58	44.60	9.52	2239.26
-33.45	248.28	250.11	249.52	249.64	245.39	237.03	212.01	161.98	95.95	41.87	8.97	2239.26
-33.35	249.73	245.25	245.04	247.23	242.47	237.05	215.74	166.66	96.58	45.55	9.24	2239.26
-33.25	249.17	246.52	248.85	248.36	245.31	233.51	211.02	162.73	96.88	45.49	9.96	2239.26
-33.15	248.67	246.36	246.62	247.87	243.58	235.71	213.83	164.70	97.52	47.38	9.35	2239.26
-33.05	247.14	247.06	248.97	246.65	244.12	235.76	211.83	163.49	98.21	44.54	9.19	2239.26
-32.95	249.42	247.82	248.06	245.00	242.51	236.51	210.25	160.62	93.44	44.48	9.52	2239.26
-32.85	251.49	249.30	245.70	248.44	243.66	231.12	208.23	152.01	96.40	44.00	9.41	2239.26
-32.75	246.90	248.22	246.38	245.03	242.64	233.71	209.59	155.03	94.35	44.60	9.30	2239.26
-32.65	248.40	250.68	248.17	247.75	241.53	232.71	209.28	171.94	96.98	45.61	9.58	2239.26
-32.55	248.19	248.58	251.07	245.55	245.36	235.76	213.80	155.49	98.82	41.10	9.35	2239.26
-32.45	249.75	249.85	249.24	246.44	248.64	235.65	208.33	161.68	97.94	43.71	8.91	2239.26
-32.35	248.21	248.83	248.96	246.14	244.35	236.23	208.21	154.28	95.68	46.08	7.86	2239.26
-32.25	248.74	246.91	249.88	245.31	243.90	236.14	209.48	162.88	99.60	43.06	8.08	2239.26
-32.15	248.03	245.78	249.68	247.35	241.97	232.90	212.50	160.47	98.64	44.18	9.85	2239.26
-32.05	247.88	246.89	248.50	247.99	244.22	233.12	212.17	160.77	94.35	44.78	8.80	2239.26
-31.95	247.58	248.65	247.76	247.99	242.84	235.26	208.21	166.96	97.19	44.36	10.85	2239.26
-31.85	246.90	246.84	247.83	249.30	242.67	231.35	213.42	166.66	96.92	44.36	9.96	2239.26
-31.75	249.11	249.60	249.70	250.78	243.80	235.08	215.08	160.02	96.28	44.89	8.63	2239.26
-31.65	248.62	248.47	249.70	250.13	246.48	238.67	210.22	163.34	99.21	45.78	10.85	2239.26
-31.55	247.20	249.56	247.57	249.29	248.81	234.17	210.68	161.37	98.45	44.60	10.46	2239.26
-31.45	250.93	248.65	247.78	251.43	242.69	238.39	211.65	161.68	98.30	45.84	8.25	2239.26
-31.35	251.09	250.55	250.49	247.59	246.56	235.21	211.35	170.43	96.16	43.00	8.80	2239.26
-31.25	249.00	247.04	249.80	247.60	248.58	236.60	216.36	160.62	97.58	42.94	8.86	2239.26
-31.15	248.24	247.94	247.90	249.34	246.08	234.10	215.87	161.07	97.22	43.89	10.24	2239.26
-31.05	249.12	247.65	249.04	246.15	247.67	236.62	212.60	163.34	98.36	46.61	10.24	2239.26
-30.95	248.63	250.66	249.46	246.52	245.83	236.40	215.56	167.56	98.30	43.11	9.69	2239.26
-30.85	247.76	250.55	249.43	248.16	244.59	235.56	212.14	159.56	94.47	43.17	9.13	2239.26
-30.75	249.58	250.46	246.82	252.18	245.34	230.87	206.14	170.58	99.21	44.83	9.63	2239.26
-30.65	248.70	251.28	249.87	248.76	247.12	238.90	211.48	151.71	97.13	45.66	9.02	2239.26
-30.55	252.33	252.70	250.32	247.63	243.19	239.14	210.74	159.71	95.07	48.93	9.35	2239.26
-30.45	251.82	250.26	247.93	249.64	247.86	236.30	209.69	166.05	98.15	44.30	9.74	2239.26
-30.35	248.07	250.19	251.86	249.62	243.75	231.87	208.56	161.22	95.25	43.65	9.24	2239.26
-30.25	251.58	244.80	249.36	252.50	246.38	236.21	211.04	156.39	95.16	41.93	8.97	2239.26
-30.15	250.96	250.27	249.37	251.14	245.24	235.69	212.32	159.41	95.77	44.24	10.18	2239.26
-30.05	248.12	250.53	247.89	248.13	252.00	232.60	214.26	165.45	97.28	44.72	8.80	2239.26
-29.95	250.61	250.54	250.63	247.17	245.46	236.85	217.48	174.51	95.19	46.08	9.69	2239.26
-29.85	251.03	245.52	250.29	249.95	240.66	234.99	212.50	161.83	95.44	46.20	10.24	2239.26

Midpt.	11000	2700	910	240	84	26	8.8	2.7	0.92	0.31	Volume	
-29.75	250.97	250.68	247.47	249.53	242.14	238.39	210.22	157.90	95.65	44.36	10.68	2239.26
-29.65	251.15	248.89	251.23	249.60	245.54	233.81	211.45	160.92	94.41	43.35	9.58	2239.26
-29.55	250.24	248.03	251.00	249.78	247.92	236.14	214.64	164.70	97.46	43.89	8.36	2239.26
-29.45	251.59	250.65	247.88	248.46	243.36	234.64	206.70	158.20	93.90	43.00	9.69	2239.26
-29.35	248.10	248.32	250.01	250.61	245.11	235.55	215.54	154.58	95.56	45.84	9.85	2239.26
-29.25	249.92	251.74	248.89	251.80	246.92	236.37	216.00	150.81	95.50	44.60	9.41	2239.26
-29.15	250.11	251.80	249.74	249.23	244.99	237.10	212.63	168.17	96.40	44.36	8.91	2239.26
-29.05	248.04	249.45	250.97	250.55	246.10	236.01	210.15	157.75	97.76	44.72	8.25	2239.26
-28.95	252.31	251.25	250.01	248.13	246.58	234.23	210.89	168.32	98.45	44.00	8.91	2239.26
-28.85	250.94	251.52	249.80	251.56	244.97	234.80	212.73	155.18	98.03	44.48	10.41	2239.26
-28.75	248.56	251.27	251.59	246.98	245.58	235.69	213.98	164.54	94.41	43.06	9.96	2239.26
-28.65	247.22	252.14	251.97	249.31	243.23	238.05	215.13	152.47	96.34	44.78	8.47	2239.26
-28.55	250.76	251.08	247.96	249.91	246.73	233.89	208.82	170.43	95.44	43.77	8.97	2239.26
-28.45	250.78	252.97	249.80	246.70	243.28	233.49	213.01	163.19	99.48	45.07	9.35	2239.26
-28.35	250.56	248.75	250.50	248.69	241.28	230.33	207.44	167.56	98.64	44.48	11.35	2239.26
-28.25	247.26	251.18	253.58	248.88	245.86	232.90	213.52	161.22	96.34	47.21	10.41	2239.26
-28.15	249.65	252.95	247.10	248.94	243.91	232.53	210.28	153.22	96.07	41.99	8.41	2239.26
-28.05	252.16	249.56	252.09	248.72	244.02	238.99	212.19	168.17	94.95	42.40	10.18	2239.26
-27.95	250.36	250.05	250.53	248.69	249.00	234.73	213.21	163.34	94.41	43.23	9.47	2239.26
-27.85	251.74	250.06	249.40	249.42	246.03	233.01	218.17	157.00	95.13	42.76	8.97	2239.26
-27.75	250.15	249.76	250.34	250.32	244.07	233.65	214.59	167.11	96.95	42.52	8.97	2239.26
-27.65	250.11	250.55	249.62	248.84	246.50	233.01	211.65	162.88	92.42	44.48	10.24	2239.26
-27.55	249.05	247.13	250.83	247.83	244.45	234.80	211.14	166.66	94.65	44.83	8.69	2239.26
-27.45	247.28	250.93	249.93	247.48	246.20	233.28	210.63	166.36	97.79	42.76	10.35	2239.26
-27.35	250.19	252.21	251.49	249.97	246.95	229.80	212.75	160.02	97.55	42.64	8.75	2239.26
-27.25	253.24	250.24	252.61	251.14	243.21	236.73	212.40	161.83	97.88	43.53	8.30	2239.26
-27.15	251.42	252.39	252.18	249.55	249.35	237.49	211.40	168.32	96.55	44.18	9.74	2239.26
-27.05	252.69	252.18	252.39	248.65	243.97	236.83	213.06	161.98	95.56	45.96	9.19	2239.26
-26.95	252.76	254.92	253.09	248.14	248.91	231.05	210.51	164.24	99.42	47.21	10.41	2239.26
-26.85	252.17	252.91	251.10	249.83	248.53	232.87	212.14	168.92	97.88	42.40	9.96	2239.26
-26.75	256.05	249.92	251.60	248.20	242.49	233.83	210.61	151.41	97.70	46.79	10.02	2239.26
-26.65	250.80	253.08	254.46	252.50	247.20	237.44	212.27	162.43	98.09	44.12	9.24	2239.26
-26.55	253.63	252.80	252.02	250.78	248.07	238.89	217.35	166.81	97.25	42.82	10.02	2239.26
-26.45	254.00	252.69	254.29	252.09	242.69	236.06	208.31	161.37	96.95	45.01	9.13	2239.26
-26.35	255.78	255.41	253.46	250.68	247.97	235.06	206.11	167.41	96.64	44.89	10.30	2239.26
-26.25	255.11	251.62	249.90	252.06	251.88	239.08	211.30	155.18	95.28	41.81	11.01	2239.26
-26.15	253.35	250.97	252.98	251.34	247.05	237.74	217.66	153.07	97.28	43.83	10.79	2239.26
-26.05	251.40	252.35	253.59	250.68	248.85	235.23	214.59	157.60	94.89	46.32	10.18	2239.26
-25.95	253.94	257.06	252.34	252.61	251.35	236.08	213.03	166.20	95.77	42.05	9.63	2239.26
-25.85	254.75	252.34	252.82	251.78	249.62	237.51	216.84	157.45	96.64	47.86	10.30	2239.26
-25.75	255.34	255.11	255.38	253.00	244.27	240.26	216.97	159.26	98.21	46.38	10.13	2239.26
-25.65	256.54	254.20	255.22	254.10	249.90	237.71	215.33	158.36	96.25	44.42	10.18	2239.26
-25.55	256.14	258.65	253.56	254.42	250.71	238.05	214.62	174.36	97.16	44.18	9.74	2239.26
-25.45	255.86	255.04	257.43	256.76	248.26	240.12	214.34	166.66	92.51	45.25	10.02	2239.26
-25.35	258.50	255.98	256.24	253.73	248.18	240.49	217.05	156.69	97.31	45.96	10.41	2239.26

Midpt.	11000	2700	910	240	84	26	8.8	2.7	0.92	0.31	Volume	
-25.25	256.51	257.77	258.97	253.92	252.40	240.81	210.99	155.79	97.61	43.23	9.35	2239.26
-25.15	259.71	256.93	257.75	254.44	249.13	236.23	210.89	155.03	98.00	47.03	9.24	2239.26
-25.05	255.82	258.80	259.44	256.19	250.69	236.23	215.28	174.66	97.46	45.66	9.58	2239.26
-24.95	258.91	260.05	256.45	256.35	250.26	236.35	214.16	158.36	95.80	46.44	9.80	2239.26
-24.85	258.81	258.48	256.09	254.24	251.93	241.87	217.51	169.68	98.52	46.08	10.35	2239.26
-24.75	260.05	257.94	260.87	257.02	253.16	242.69	219.78	165.15	97.07	46.97	10.63	2239.26
-24.65	261.24	258.65	262.13	258.65	251.66	244.44	216.79	168.17	97.37	45.01	11.24	2239.26
-24.55	261.99	264.07	262.30	255.82	252.27	239.47	219.93	170.58	99.72	46.67	8.86	2239.26
-24.45	263.23	258.23	262.59	258.28	253.29	244.19	218.50	167.87	97.28	44.89	10.52	2239.26
-24.35	263.65	259.60	259.07	255.75	250.32	241.44	222.95	165.30	101.75	47.03	10.07	2239.26
-24.25	263.60	262.62	263.53	261.42	257.48	247.56	224.48	163.79	100.72	47.80	9.63	2239.26
-24.15	264.71	263.58	263.29	261.89	254.65	244.37	219.63	163.34	99.99	44.89	10.79	2239.26
-24.05	263.83	262.94	262.54	259.50	253.73	242.97	215.97	175.72	99.72	46.50	11.01	2239.26
-23.95	264.46	263.88	263.66	263.04	257.77	248.03	218.22	160.17	97.25	45.31	9.69	2239.26
-23.85	265.01	264.97	266.23	265.10	259.51	242.28	220.57	173.75	102.26	45.01	11.18	2239.26
-23.75	264.45	267.71	265.07	265.29	255.87	246.60	222.97	163.94	98.18	49.28	10.85	2239.26
-23.65	264.69	266.70	266.12	263.58	257.32	242.74	218.09	168.02	100.36	46.14	10.74	2239.26
-23.55	265.07	270.26	268.19	263.15	256.46	250.05	219.47	172.70	98.88	47.09	11.62	2239.26
-23.45	267.85	268.16	265.49	268.23	260.32	242.74	225.27	169.22	103.13	46.32	9.47	2239.26
-23.35	266.07	270.91	269.51	264.76	260.49	250.40	221.41	160.47	102.65	47.80	10.90	2239.26
-23.25	265.71	269.40	266.96	264.87	264.26	250.51	227.67	161.07	101.75	46.02	11.01	2239.26
-23.15	267.14	270.68	269.18	267.17	263.29	247.12	223.38	169.53	100.54	46.08	10.13	2239.26
-23.05	270.04	271.81	272.54	264.92	262.75	250.81	228.57	170.73	98.73	49.28	11.13	2239.26
-22.95	271.39	270.84	269.44	268.97	261.64	252.35	226.65	157.75	99.72	47.15	9.63	2239.26
-22.85	269.71	272.39	272.91	269.99	259.88	250.33	225.88	170.28	98.49	44.89	9.91	2239.26
-22.75	273.53	275.77	269.75	270.50	264.48	249.51	227.47	160.17	100.84	48.10	10.68	2239.26
-22.65	275.24	270.88	273.32	270.47	269.24	252.12	224.94	166.66	103.74	47.80	11.01	2239.26
-22.55	277.12	272.66	273.69	272.04	267.88	255.03	229.26	165.15	102.29	48.87	9.85	2239.26
-22.45	276.04	276.64	276.13	272.99	264.38	250.05	233.17	171.79	102.74	44.36	11.46	2239.26
-22.35	275.68	272.32	274.23	272.44	266.68	254.08	224.68	170.28	102.80	45.49	11.24	2239.26
-22.25	277.70	279.80	276.67	273.91	266.12	254.08	229.03	174.36	103.62	47.44	10.63	2239.26
-22.15	276.33	276.04	276.29	274.88	264.04	254.26	228.82	173.75	99.57	48.10	10.85	2239.26
-22.05	278.44	277.66	278.56	275.53	267.93	253.71	227.83	163.79	103.16	45.90	11.79	2239.26
-21.95	279.27	282.87	279.89	276.86	269.56	255.28	231.45	169.38	104.86	48.16	11.57	2239.26
-21.85	280.75	282.28	279.26	278.92	272.53	260.56	230.81	178.28	103.56	49.82	9.91	2239.26
-21.75	281.27	283.27	282.05	279.32	273.99	258.24	229.44	181.00	104.67	49.10	11.07	2239.26
-21.65	282.82	283.31	282.71	284.64	270.15	257.10	227.34	183.11	108.66	44.72	12.51	2239.26
-21.55	284.10	285.92	281.69	275.25	274.09	257.37	230.15	174.36	104.52	47.92	11.29	2239.26
-21.45	284.05	288.49	284.48	282.59	275.68	261.78	236.15	174.66	103.89	49.99	10.90	2239.26
-21.35	287.18	290.69	283.66	280.52	281.65	266.33	239.37	169.07	106.37	50.29	11.40	2239.26
-21.25	286.16	284.41	288.99	281.32	279.56	261.45	238.33	171.04	104.64	48.21	11.96	2239.26
-21.15	286.00	291.50	288.03	281.91	275.03	268.54	236.97	174.81	105.64	48.69	11.68	2239.26
-21.05	287.99	284.87	287.17	285.40	274.68	269.13	238.79	180.85	107.45	48.75	12.40	2239.26
-20.95	286.51	290.67	289.26	286.98	281.42	265.65	239.35	183.57	108.54	49.58	10.18	2239.26
-20.85	294.99	289.89	288.25	286.55	280.03	262.58	238.35	186.13	108.27	47.15	12.40	2239.26

Midpt.	11000	2700	910	240	84	26	8.8	2.7	0.92	0.31	Volume	
-20.75	289.36	290.03	291.42	289.95	281.94	264.72	239.94	175.56	108.39	50.94	11.62	2239.26
-20.65	289.30	292.12	293.35	289.75	281.87	269.10	240.42	181.45	107.12	48.21	13.34	2239.26
-20.55	296.49	294.28	293.93	291.36	278.67	271.01	239.17	169.22	107.21	48.45	11.40	2239.26
-20.45	293.63	295.14	296.36	289.62	287.76	266.69	235.69	181.15	112.64	53.14	12.73	2239.26
-20.35	294.95	297.84	295.31	292.18	284.19	270.60	244.48	179.34	110.50	48.99	11.90	2239.26
-20.25	297.33	297.62	298.53	292.51	284.32	274.85	242.06	185.68	107.09	49.64	12.79	2239.26
-20.15	299.05	298.85	297.07	295.77	283.85	273.77	249.18	181.60	107.63	48.99	10.79	2239.26
-20.05	299.71	299.18	300.88	296.55	286.35	277.67	246.55	180.55	110.20	46.91	13.12	2239.26
-19.95	301.38	302.66	300.93	299.09	290.12	276.95	242.23	183.11	108.99	47.80	12.84	2239.26
-19.85	301.31	301.90	300.93	297.94	291.43	279.43	243.72	183.87	110.59	52.72	12.62	2239.26
-19.75	298.55	303.90	298.48	299.27	296.43	277.61	245.63	177.07	110.05	51.06	12.95	2239.26
-19.65	304.40	302.93	305.57	298.28	288.80	283.18	248.88	182.66	107.60	50.17	13.62	2239.26
-19.55	307.79	308.25	308.26	302.37	289.37	280.54	241.67	189.75	108.30	50.88	12.95	2239.26
-19.45	306.12	308.67	304.88	299.46	292.89	286.18	246.35	181.15	112.13	51.89	11.85	2239.26
-19.35	306.14	312.35	310.77	304.06	296.35	281.38	252.30	182.66	111.59	51.12	11.68	2239.26
-19.25	313.20	310.55	311.24	304.11	297.15	283.15	250.23	192.77	112.49	50.29	11.57	2239.26
-19.15	255.55	258.55	251.89	253.65	242.24	230.89	198.24	153.07	91.81	41.22	11.35	2239.26
-19.05	28.37	29.50	28.83	29.37	26.60	23.73	19.39	12.98	8.12	3.85	0.89	12225.45
-18.95	28.62	27.96	27.68	26.82	24.02	21.68	18.09	10.87	7.00	3.02	0.77	139.22
-18.85	27.53	27.44	26.39	25.52	24.35	21.10	18.65	12.98	6.55	2.61	0.89	128.90
-18.75	25.98	25.19	25.41	23.70	22.09	20.91	17.27	9.66	6.49	2.97	0.72	121.87
-18.65	24.78	24.53	25.15	23.93	22.01	19.98	15.63	11.62	6.34	3.20	0.33	116.27
-18.55	24.85	24.78	23.79	21.69	21.96	19.64	14.26	11.17	6.46	2.73	0.22	111.53
-18.45	23.45	25.11	22.94	22.54	21.10	17.53	15.46	11.62	5.74	2.85	0.33	107.38
-18.35	23.05	23.30	21.49	21.18	19.22	17.64	15.53	9.81	6.10	2.31	0.44	103.68
-18.25	23.52	21.10	21.52	20.07	19.04	17.98	13.95	9.21	5.34	2.61	0.89	100.32
-18.15	22.30	21.87	21.69	20.67	19.37	17.64	14.13	11.17	5.77	2.08	0.77	97.24
-18.05	20.93	22.16	19.75	20.35	18.67	16.09	13.62	9.36	5.01	2.08	0.66	94.39
-17.95	21.46	20.63	20.40	18.28	17.36	14.91	13.21	11.02	4.56	2.02	0.44	91.74
-17.85	20.15	20.13	19.37	19.04	17.48	17.00	11.32	6.64	5.71	1.78	0.39	89.27
-17.75	19.77	18.72	19.82	17.88	17.39	16.14	12.57	9.21	4.95	2.13	0.72	86.94
-17.65	19.87	18.87	19.49	18.05	17.29	15.48	12.90	7.70	3.83	1.84	0.39	84.75
-17.55	20.74	19.17	18.90	17.07	17.09	14.27	10.96	9.36	4.47	1.54	0.44	82.67
-17.45	18.01	18.67	18.91	17.47	17.75	13.91	12.06	8.30	4.41	1.78	0.50	80.70
-17.35	19.82	17.97	18.05	17.04	18.40	15.41	11.01	7.85	4.02	1.60	0.33	78.83
-17.25	17.75	18.03	17.44	17.26	15.55	13.12	11.39	7.10	4.35	1.36	0.44	77.04
-17.15	18.41	17.85	16.96	17.59	15.15	13.64	11.57	6.64	3.83	1.54	0.33	75.33
-17.05	18.39	18.09	18.17	16.13	15.10	12.16	10.60	8.45	3.32	1.84	0.28	73.70
-16.95	17.28	17.86	16.93	15.59	14.78	12.36	10.09	7.55	4.05	1.90	0.39	72.14
-16.85	16.82	17.47	15.24	15.21	15.85	14.18	10.55	10.11	4.14	1.84	0.22	70.64
-16.75	16.90	16.76	16.52	14.76	14.74	11.48	9.73	6.34	3.20	1.36	0.55	69.20
-16.65	16.48	16.75	16.34	14.53	13.89	12.78	10.30	6.49	3.74	1.78	0.33	67.81
-16.55	16.69	17.30	16.52	16.47	14.49	12.89	9.96	5.43	3.56	1.84	0.39	66.48
-16.45	16.23	15.79	15.95	13.91	13.74	11.82	10.04	5.13	3.74	1.60	0.33	65.19
-16.35	14.99	15.62	16.31	13.32	14.27	11.82	10.27	6.04	3.80	1.78	0.28	63.95

Midpt.	11000	2700	910	240	84	26	8.8	2.7	0.92	0.31	Volume	
-16.25	16.07	15.90	15.39	15.08	14.42	11.34	9.30	8.76	3.71	1.36	0.39	62.76
-16.15	16.59	15.28	15.14	13.79	12.36	11.78	8.89	5.28	3.05	1.01	0.44	61.60
-16.05	15.94	16.15	15.06	13.76	12.88	11.30	8.40	5.59	3.65	1.54	0.61	60.48
-15.95	16.46	14.97	15.42	14.04	13.07	10.91	7.54	5.89	3.08	1.66	0.17	59.40
-15.85	15.65	14.73	14.42	13.79	12.88	10.30	8.53	5.43	2.81	1.13	0.33	58.35
-15.75	14.19	15.03	13.81	13.93	11.72	10.87	8.76	6.49	3.50	1.36	0.33	57.34
-15.65	14.73	14.39	13.58	12.37	11.81	10.98	9.68	6.94	3.17	1.48	0.28	56.36
-15.55	13.55	14.08	13.51	13.31	10.73	10.59	7.31	6.19	3.44	1.48	0.17	55.41
-15.45	19.94	20.72	20.46	18.33	15.65	14.34	10.24	11.02	3.65	1.84	0.28	54.48
-15.35	32.12	30.50	31.56	29.89	27.09	21.64	18.16	11.02	5.89	2.19	0.50	53.59
-15.25	31.79	31.05	30.24	29.45	27.07	23.10	18.73	10.27	6.40	2.67	0.44	52.72
-15.15	32.11	31.73	31.06	29.46	27.21	23.05	16.32	10.42	5.92	2.02	0.44	51.87
-15.05	31.45	31.08	29.57	28.48	26.89	23.23	17.53	9.36	5.83	1.90	0.39	51.05
-14.95	31.17	30.77	30.24	29.26	26.07	21.96	15.74	9.66	5.31	2.08	0.28	50.25
-14.85	30.48	30.54	29.63	27.43	26.52	22.62	17.12	11.77	5.16	1.90	0.44	49.48
-14.75	30.64	31.12	29.21	29.36	26.08	22.19	15.99	8.76	4.68	2.02	0.39	48.72
-14.65	30.25	29.78	29.11	28.48	25.88	22.64	17.58	10.72	5.16	1.66	0.39	47.99
-14.55	30.13	30.46	30.12	28.50	25.78	22.10	16.89	10.72	4.26	1.36	0.28	47.28
-14.45	30.76	29.82	29.07	29.22	25.61	21.78	14.74	11.77	4.59	1.25	0.39	46.58
-14.35	30.74	29.73	28.47	28.02	26.33	20.87	15.51	10.27	5.10	1.84	0.39	45.91
-14.25	31.37	30.89	29.35	27.13	25.85	21.37	17.04	8.76	4.83	1.19	0.50	45.25
-14.15	29.98	30.45	29.69	27.82	25.83	21.82	15.12	9.36	4.29	1.36	0.39	44.61
-14.05	30.57	30.14	29.20	27.81	25.11	21.80	14.69	9.66	4.23	1.13	0.55	43.98
-13.95	30.51	29.87	29.34	27.48	25.51	21.12	16.20	12.23	4.62	1.90	0.55	43.38
-13.85	30.34	29.83	28.73	27.65	25.76	21.23	15.97	8.00	4.65	1.72	0.22	42.78
-13.75	30.49	29.43	29.47	27.52	25.65	20.14	16.07	9.96	3.80	1.96	0.22	42.21
-13.65	30.61	28.84	29.44	27.60	26.25	20.50	15.84	9.06	4.56	1.36	0.33	41.65
-13.55	30.17	30.94	29.90	28.25	24.84	21.10	14.71	10.87	4.65	1.54	0.17	41.10
-13.45	30.01	30.38	29.74	27.14	23.65	20.80	15.02	8.15	4.38	1.96	0.44	40.56
-13.35	30.73	29.93	29.50	27.59	26.12	20.39	15.20	8.60	4.23	1.25	0.55	40.04
-13.25	30.76	30.07	29.50	27.78	25.16	20.25	15.43	8.91	4.14	1.13	0.55	39.54
-13.15	30.11	30.47	29.47	27.97	25.16	19.32	16.22	7.70	3.50	1.19	0.33	39.04
-13.05	30.75	31.05	29.16	28.14	26.23	19.66	15.10	8.30	4.68	1.30	0.50	38.56
-12.95	30.74	31.73	30.00	28.06	26.07	21.00	13.69	6.94	3.71	1.30	0.44	38.09
-12.85	30.45	30.47	30.89	28.96	25.36	20.66	14.10	8.45	3.99	1.42	0.28	37.63
-12.75	31.20	31.17	29.94	29.32	25.65	20.52	15.61	9.36	3.50	0.95	0.28	37.18
-12.65	31.74	32.01	31.39	29.31	27.06	21.52	13.87	6.34	4.11	0.89	0.50	36.74
-12.55	32.49	31.40	31.07	29.74	25.51	19.43	14.10	6.34	3.59	1.01	0.55	36.32
-12.45	32.04	32.62	30.71	29.17	26.47	21.78	14.13	8.60	4.05	1.60	0.28	35.90
-12.35	32.89	31.59	31.98	29.34	27.26	20.96	14.51	6.34	3.89	1.72	0.28	35.50
-12.25	33.21	32.61	32.63	29.41	26.02	21.19	14.92	6.79	3.96	1.54	0.50	35.10
-12.15	33.27	34.65	32.29	29.60	26.23	22.10	14.10	6.94	3.53	1.84	0.17	34.72
-12.05	34.85	33.68	33.72	31.13	28.33	20.57	14.36	8.45	3.68	2.02	0.22	34.34
-11.95	34.89	34.58	32.86	31.64	28.20	20.66	15.79	8.45	3.92	1.13	0.44	33.98
-11.85	36.32	35.46	34.56	30.97	28.33	21.10	15.20	8.30	3.29	1.30	0.55	33.62

Midpt.	11000	2700	910	240	84	26	8.8	2.7	0.92	0.31	Volume	
-11.75	36.51	35.62	35.46	31.87	28.87	21.44	14.33	9.81	4.14	0.95	0.28	33.27
-11.65	38.61	37.68	35.51	33.49	28.16	21.21	15.51	8.30	3.86	0.95	0.22	32.93
-11.55	38.23	37.85	36.73	33.04	28.73	21.85	13.95	6.79	3.86	1.72	0.11	32.60
-11.45	38.23	38.23	37.55	34.02	30.14	22.73	15.92	8.45	3.56	1.36	0.33	32.27
-11.35	39.83	40.77	38.19	34.20	29.39	22.77	13.77	8.00	3.68	1.01	0.44	31.96
-11.25	40.25	40.90	38.92	35.27	30.36	22.60	15.92	9.06	3.99	1.84	0.33	31.65
-11.15	42.08	41.14	39.75	36.00	31.42	24.16	14.69	6.49	3.83	1.54	0.22	31.35
-11.05	43.81	43.13	40.58	37.15	32.14	23.53	14.82	6.19	4.44	1.66	0.39	31.06
-10.95	45.58	44.28	41.57	37.74	32.46	24.03	15.89	7.10	4.17	0.95	0.28	30.77
-10.85	47.15	46.18	43.20	39.68	34.20	23.00	17.17	8.00	3.92	1.13	0.50	30.50
-10.75	47.88	45.24	44.37	41.16	35.29	24.60	16.96	9.66	3.32	1.30	0.33	30.23
-10.65	48.43	48.01	45.88	40.17	35.78	25.43	16.94	7.10	4.17	1.25	0.77	29.96
-10.55	50.14	49.66	46.70	41.04	35.37	25.89	16.94	7.25	3.89	1.66	0.17	29.71
-10.45	51.55	50.50	49.42	41.75	35.31	25.41	15.58	10.42	4.35	1.66	0.72	29.46
-10.35	53.08	51.69	50.78	45.05	37.09	26.27	17.73	7.10	4.38	1.13	0.50	29.21
-10.25	55.23	53.42	50.78	44.23	36.58	27.39	16.86	9.96	4.65	1.13	0.33	28.98
-10.15	55.14	56.43	51.62	45.43	37.91	26.62	17.70	6.79	4.29	1.78	0.72	28.75
-10.05	58.00	56.12	52.96	46.93	37.14	27.51	16.48	9.81	3.47	1.01	0.28	28.52
-9.95	61.25	57.64	54.75	47.84	37.34	26.78	16.63	6.94	3.83	2.19	0.55	28.31
-9.85	62.53	57.92	55.49	47.97	37.12	26.69	17.58	9.21	4.17	1.42	0.50	28.09
-9.75	63.13	60.54	57.51	47.87	38.33	27.68	17.35	8.76	4.32	1.78	0.44	27.89
-9.65	63.51	61.24	58.24	48.19	37.40	27.32	16.58	7.85	3.96	1.19	0.17	27.69
-9.55	65.81	61.43	57.43	49.37	38.88	26.23	17.58	7.55	4.17	1.54	0.55	27.49
-9.45	67.85	64.27	59.93	49.96	39.17	27.87	16.09	8.00	3.56	1.60	0.66	27.30
-9.35	68.64	66.27	61.89	51.09	39.69	26.68	16.17	9.66	3.56	1.48	0.50	27.12
-9.25	69.41	68.46	62.20	51.53	40.88	26.66	16.78	8.76	4.41	1.48	0.33	26.94
-9.15	73.53	69.87	64.15	52.18	41.13	26.78	16.12	6.64	3.92	1.54	0.39	26.77
-9.05	73.89	70.47	66.43	51.81	40.44	26.16	16.94	6.49	4.20	1.36	0.50	26.60
-8.95	75.17	73.39	65.74	55.21	39.77	25.82	15.58	7.85	3.56	1.13	0.72	26.44
-8.85	78.26	74.50	67.08	54.65	38.63	25.46	17.45	7.40	3.02	1.19	0.50	26.29
-8.75	78.22	73.63	67.17	55.12	39.72	24.84	15.20	6.34	3.68	1.42	0.44	26.14
-8.65	79.43	74.20	66.29	53.06	38.28	24.84	14.69	7.70	3.20	1.30	0.50	25.99
-8.55	78.04	73.28	65.31	52.57	37.30	23.82	15.12	6.94	2.99	0.83	0.55	25.85
-8.45	75.47	73.51	63.71	51.04	37.69	24.53	15.20	5.89	3.62	1.48	0.17	25.71
-8.35	74.57	72.21	62.74	49.60	36.10	23.32	12.65	6.79	3.14	1.54	0.22	25.58
-8.25	73.07	67.93	60.46	46.80	34.84	21.23	12.54	5.13	2.48	1.36	0.33	25.45
-8.15	69.43	66.45	59.86	43.73	32.41	20.39	11.27	6.34	2.60	0.77	0.11	25.33
-8.05	70.30	63.52	58.06	43.82	30.93	18.82	11.14	3.92	2.35	0.95	0.17	25.21
-7.95	68.40	60.65	56.60	41.95	29.81	18.89	11.06	5.74	2.45	0.83	0.33	25.10
-7.85	65.87	56.82	53.11	37.95	27.32	17.32	10.53	4.08	2.05	0.83	0.39	24.99
-7.75	58.52	56.85	47.81	36.68	24.29	16.66	9.15	6.79	1.84	0.65	0.11	24.89
-7.65	58.26	53.69	46.63	33.92	24.17	14.32	8.53	4.23	1.75	0.65	0.39	24.79
-7.55	52.96	46.89	40.98	30.71	22.98	13.86	6.85	4.68	2.02	0.77	0.17	24.69
-7.45	48.09	44.58	39.95	28.99	21.54	13.41	6.97	3.32	1.54	0.53	0.11	24.60
-7.35	44.97	42.94	37.95	26.76	17.26	11.05	7.36	5.43	1.57	0.36	0.17	24.52

Midpt.	11000	2700	910	240	84	26	8.8	2.7	0.92	0.31	Volume	
-7.25	39.55	42.31	33.53	23.58	16.19	10.84	5.90	3.32	1.45	0.53	0.33	24.44
-7.15	38.48	39.40	34.29	23.05	15.68	9.66	6.21	1.81	1.06	0.24	0.17	24.36
-7.05	34.36	35.20	30.47	20.01	14.93	10.07	5.26	1.96	1.09	0.53	0.11	24.28
-6.95	33.51	31.21	26.67	18.09	14.54	7.12	4.96	3.32	1.06	0.53	0.17	24.22
-6.85	30.08	28.85	22.97	16.34	11.86	7.09	4.37	1.51	0.97	0.53	0.00	24.15
-6.75	29.21	25.83	20.77	14.57	11.69	9.05	3.99	1.51	0.78	0.30	0.17	24.09
-6.65	23.82	25.62	18.80	12.82	9.49	6.50	3.22	0.91	0.72	0.24	0.11	24.03
-6.55	21.31	21.06	16.96	11.63	8.74	5.70	3.27	1.51	0.54	0.36	0.06	23.98
-6.45	20.43	17.89	16.65	10.87	7.65	4.05	3.14	1.51	0.69	0.36	0.06	23.93
-6.35	19.07	17.33	14.93	10.59	7.31	3.95	3.32	1.36	0.51	0.06	0.00	23.89
-6.25	15.16	15.25	13.42	9.24	7.50	3.27	2.09	0.75	0.75	0.18	0.00	23.85
-6.15	14.03	13.63	12.70	7.63	6.19	3.07	2.12	1.36	0.42	0.18	0.11	23.81
-6.05	11.72	11.91	10.18	7.10	5.40	3.02	2.32	1.21	0.51	0.12	0.11	23.78
-5.95	10.80	10.05	9.23	6.22	4.71	2.75	1.51	0.30	0.24	0.30	0.11	23.75
-5.85	11.43	9.52	8.48	6.62	3.49	2.43	1.71	0.60	0.45	0.30	0.00	23.73
-5.75	10.56	8.73	7.49	5.61	3.30	2.27	1.33	0.75	0.21	0.18	0.06	23.71
-5.65	7.99	7.27	6.47	5.37	3.74	2.05	1.58	0.45	0.30	0.24	0.06	23.69
-5.55	7.59	5.46	6.84	4.32	3.69	2.37	1.12	0.45	0.24	0.18	0.06	23.68
-5.45	5.98	5.45	5.62	4.43	2.90	1.70	1.35	0.45	0.24	0.06	0.11	23.67
-5.35	3.75	3.64	3.14	2.43	1.61	0.73	0.61	0.30	0.21	0.00	0.06	23.67
-5.25	3.30	3.35	2.79	2.06	1.38	0.93	0.77	0.45	0.15	0.06	0.00	23.67
-5.15	3.16	3.26	2.58	2.08	1.53	0.95	0.46	0.45	0.12	0.12	0.00	23.67
-5.05	3.22	2.77	2.63	1.86	1.24	0.66	0.66	0.15	0.06	0.06	0.06	23.67
-4.95	3.09	2.64	2.11	1.81	1.43	0.82	0.38	0.30	0.09	0.06	0.06	23.67
-4.85	2.70	2.41	2.21	1.80	1.38	0.84	0.61	0.30	0.06	0.00	0.11	23.67
-4.75	2.66	2.55	2.13	1.72	1.04	0.75	0.56	0.00	0.18	0.00	0.00	23.67
-4.65	2.39	2.39	2.11	1.59	1.09	0.71	0.56	0.15	0.21	0.06	0.06	23.67
-4.55	2.55	2.59	1.91	1.56	1.19	0.50	0.61	0.30	0.12	0.00	0.00	23.67
-4.45	2.35	2.48	1.82	1.48	1.27	0.73	0.33	0.45	0.21	0.06	0.00	23.67
-4.35	2.48	2.31	1.99	1.52	1.09	0.73	0.36	0.30	0.12	0.06	0.00	23.67
-4.25	2.37	2.26	1.81	1.37	1.07	0.73	0.31	0.15	0.06	0.06	0.00	23.67
-4.15	2.46	2.18	1.98	1.29	1.07	0.55	0.38	0.30	0.09	0.00	0.00	23.67
-4.05	2.45	2.37	1.73	1.40	1.26	0.71	0.36	0.15	0.18	0.00	0.06	23.67
-3.95	2.55	2.16	1.88	1.50	1.16	0.64	0.61	0.15	0.12	0.18	0.00	23.67
-3.85	2.31	2.09	2.18	1.46	1.14	0.75	0.51	0.15	0.09	0.06	0.00	23.67
-3.75	2.32	2.30	1.82	1.59	1.12	0.71	0.46	0.15	0.18	0.12	0.00	23.67
-3.65	2.44	2.17	1.80	1.32	1.09	0.95	0.28	0.45	0.15	0.12	0.00	23.67
-3.55	2.58	2.44	2.14	1.56	1.14	0.66	0.46	0.45	0.12	0.00	0.06	23.67
-3.45	2.69	2.30	2.25	1.79	1.04	0.71	0.49	0.15	0.12	0.00	0.06	23.67
-3.35	2.65	2.53	2.39	1.69	1.17	0.89	0.56	0.30	0.12	0.00	0.11	23.67
-3.25	2.60	2.77	2.18	2.07	1.46	0.75	0.74	0.15	0.06	0.12	0.00	23.67
-3.15	2.51	2.57	2.38	1.64	1.73	0.75	0.56	0.15	0.21	0.12	0.00	23.67
-3.05	3.00	3.15	2.61	2.10	1.56	0.82	0.54	0.15	0.03	0.06	0.06	23.67
-2.95	3.06	3.14	2.16	2.08	1.63	0.95	0.64	0.30	0.24	0.00	0.00	23.67
-2.85	3.45	3.35	2.72	2.19	1.54	0.96	0.66	0.15	0.09	0.00	0.06	23.67

Midpt.	11000	2700	910	240	84	26	8.8	2.7	0.92	0.31	Volume	
-2.75	3.51	3.21	2.90	2.47	1.69	0.89	0.49	0.45	0.18	0.06	0.06	23.67
-2.65	3.55	3.48	2.68	2.28	1.85	1.27	0.66	0.30	0.18	0.00	0.00	23.67
-2.55	3.81	3.98	3.27	2.54	1.74	1.18	0.64	0.45	0.24	0.12	0.06	23.67
-2.45	4.14	3.68	3.15	2.51	1.83	1.27	0.74	0.91	0.39	0.00	0.00	23.67
-2.35	4.31	4.22	3.20	2.70	2.37	1.50	0.95	0.60	0.18	0.06	0.00	23.67
-2.25	4.65	4.03	3.71	2.89	2.01	1.48	0.89	0.30	0.18	0.00	0.00	23.67
-2.15	4.76	4.66	4.17	3.25	2.00	1.46	0.69	0.60	0.24	0.00	0.00	23.67
-2.05	5.52	4.85	4.31	3.30	2.73	1.32	0.87	0.91	0.36	0.12	0.06	23.67
-1.95	5.54	5.29	4.57	3.66	2.37	2.11	0.92	0.30	0.18	0.12	0.06	23.67
-1.85	5.98	5.78	4.98	3.89	2.80	2.12	1.25	0.45	0.15	0.00	0.00	23.67
-1.75	6.35	6.23	4.90	4.13	3.17	2.05	1.15	0.60	0.33	0.18	0.00	23.67
-1.65	6.80	6.53	5.87	4.55	3.20	2.34	1.23	1.06	0.39	0.00	0.00	23.67
-1.55	7.76	7.16	6.24	4.71	3.87	2.54	1.30	0.75	0.39	0.30	0.06	23.67
-1.45	8.14	7.36	6.83	5.10	4.11	2.37	1.15	0.30	0.39	0.06	0.11	23.67
-1.35	8.88	7.62	7.53	5.57	4.08	2.75	1.61	1.06	0.30	0.24	0.00	23.67
-1.25	9.29	8.74	7.63	6.17	5.13	2.91	1.94	1.36	0.63	0.24	0.00	23.67
-1.15	10.40	9.18	8.10	6.86	5.25	3.21	1.86	0.60	0.54	0.06	0.17	23.67
-1.05	10.73	9.79	9.04	7.17	5.40	3.18	2.12	1.06	0.54	0.12	0.11	23.67
-0.95	11.46	11.10	9.69	7.96	6.11	4.02	2.30	2.11	0.60	0.30	0.17	23.67
-0.85	12.68	11.59	10.95	8.77	6.47	3.96	2.66	0.60	0.39	0.24	0.00	23.67
-0.75	13.60	12.84	11.75	9.32	6.91	4.57	2.63	0.75	0.69	0.18	0.00	23.67
-0.65	14.46	13.47	12.15	9.90	7.21	3.98	2.66	0.91	0.78	0.30	0.17	23.67
-0.55	15.27	14.19	12.55	10.35	7.13	4.46	2.45	1.66	0.63	0.36	0.00	23.67
-0.45	15.89	14.71	13.25	11.15	7.18	5.84	3.07	1.06	0.88	0.30	0.00	23.67
-0.35	16.38	15.74	14.09	11.60	8.15	5.20	3.47	1.66	0.91	0.06	0.11	23.67
-0.25	17.59	16.34	14.18	11.92	8.54	6.04	3.32	2.26	0.78	0.30	0.22	23.67
-0.05	17.65	15.94	15.70	12.29	7.95	5.50	3.76	2.11	0.94	0.18	0.17	23.67
0.05	16.58	17.10	15.51	12.06	9.09	6.34	3.88	2.11	0.54	0.06	0.06	23.67
0.25	17.54	16.61	15.18	12.17	9.24	6.16	3.60	1.21	1.12	0.30	0.00	23.67
0.35	17.34	16.74	15.31	12.19	9.61	6.70	3.86	1.36	0.60	0.18	0.06	23.67
0.45	16.99	16.43	15.30	12.23	9.53	6.12	4.01	1.36	0.54	0.12	0.00	23.67
0.55	16.89	15.91	14.68	11.80	8.47	5.80	3.50	2.42	1.06	0.36	0.11	23.67
0.65	16.32	15.59	14.71	11.94	8.84	5.95	3.68	3.02	0.85	0.36	0.11	23.67
0.75	15.89	15.25	14.48	11.44	8.54	5.89	3.88	1.51	0.82	0.53	0.33	23.67
0.85	14.86	15.55	13.64	10.90	8.39	5.55	3.24	2.26	0.66	0.24	0.06	23.67
0.95	14.25	14.15	13.09	10.52	8.29	5.66	3.42	1.36	0.72	0.42	0.17	23.67
1.05	13.39	12.85	12.50	10.00	8.07	4.87	3.12	1.51	0.75	0.36	0.06	23.67
1.15	13.23	12.52	11.75	9.70	7.78	4.96	2.78	0.91	0.69	0.24	0.11	23.67
1.25	12.18	11.77	11.19	9.16	6.69	4.87	2.55	1.36	0.75	0.18	0.17	23.67
1.35	11.60	11.75	10.39	8.80	6.36	3.89	2.94	1.36	0.57	0.24	0.06	23.67
1.45	10.91	10.57	10.27	8.48	6.09	3.93	2.32	1.21	0.57	0.18	0.00	23.67
1.55	10.46	10.31	8.89	7.80	6.11	3.73	2.30	1.21	0.54	0.24	0.00	23.67
1.65	9.34	9.56	8.84	7.36	5.89	3.57	2.61	0.75	0.54	0.30	0.39	23.67
1.75	8.99	8.70	8.39	7.16	5.18	3.48	2.02	0.30	0.60	0.18	0.00	23.67
1.85	8.74	8.23	8.15	6.77	5.45	3.55	2.12	1.06	0.36	0.06	0.11	23.67

Midpt.	11000	2700	910	240	84	26	8.8	2.7	0.92	0.31	Volume	
1.95	8.15	8.26	7.91	6.07	4.81	3.32	2.15	0.91	0.66	0.12	0.00	23.67
2.05	7.51	7.50	7.56	6.23	4.56	3.20	2.07	0.60	0.75	0.24	0.00	23.67
2.15	7.49	7.48	6.63	5.94	4.80	3.32	1.92	0.75	0.48	0.12	0.11	23.67
2.25	7.66	6.86	6.71	5.66	4.88	3.39	1.97	0.60	0.36	0.24	0.17	23.67
2.35	7.00	6.72	6.64	5.19	3.91	3.11	1.61	1.06	0.21	0.18	0.00	23.67
2.45	6.73	6.83	6.46	5.05	3.96	2.57	1.97	0.45	0.45	0.24	0.00	23.67
2.55	6.68	6.93	6.10	5.10	4.23	2.73	1.53	1.51	0.18	0.00	0.06	23.67
2.65	6.59	6.15	6.07	4.79	4.09	3.32	2.04	0.60	0.33	0.06	0.00	23.67
2.75	6.96	6.31	5.83	5.00	4.34	2.73	1.48	0.45	0.33	0.36	0.00	23.67
2.85	6.61	6.32	6.37	5.20	4.28	3.00	1.63	1.21	0.51	0.18	0.11	23.67
2.95	6.54	6.31	6.00	5.15	4.23	3.21	1.74	0.75	0.27	0.30	0.11	23.67
3.05	6.43	6.24	6.23	4.82	4.13	2.25	1.81	0.91	0.27	0.24	0.00	23.67
3.15	6.85	6.18	5.71	4.83	4.38	2.98	1.69	0.45	0.30	0.12	0.00	23.67
3.25	6.38	6.54	6.25	5.52	4.13	2.55	1.66	0.75	0.30	0.18	0.00	23.67
3.35	7.32	6.85	6.25	5.28	3.91	3.36	1.81	0.60	0.33	0.06	0.06	23.67
3.45	6.55	6.64	6.70	4.97	3.87	2.95	1.66	0.60	0.39	0.18	0.17	23.67
3.55	7.26	6.82	6.57	5.43	4.29	2.86	1.86	1.06	0.42	0.18	0.00	23.67
3.65	6.86	6.87	6.82	5.21	4.88	3.12	1.94	0.91	0.54	0.24	0.06	23.67
3.75	6.94	7.20	6.90	5.52	4.66	3.18	1.86	0.75	0.48	0.24	0.06	23.67
3.85	7.38	7.47	7.12	5.66	5.52	3.18	1.66	1.06	0.30	0.24	0.00	23.67
3.95	7.92	7.60	6.91	5.54	5.35	3.09	2.35	1.21	0.57	0.24	0.06	23.67
4.05	8.13	7.65	7.50	6.00	5.03	3.61	2.48	0.91	0.63	0.00	0.11	23.67
4.15	8.11	7.64	7.33	6.08	5.10	3.57	2.58	1.06	0.75	0.24	0.00	23.67
4.25	8.35	7.80	7.60	6.56	5.54	3.89	2.40	1.06	0.39	0.42	0.06	23.67
4.35	8.63	8.35	7.93	7.21	5.65	3.96	2.68	1.36	0.48	0.36	0.00	23.67
4.45	8.67	8.36	8.35	7.11	5.67	4.00	2.35	0.60	0.69	0.06	0.17	23.67
4.55	9.52	8.83	8.24	7.35	6.09	4.05	2.53	2.57	0.69	0.36	0.22	23.67
4.65	10.01	9.26	8.46	7.37	6.64	4.45	2.91	0.60	0.72	0.12	0.06	23.67
4.75	9.90	8.99	8.96	7.63	6.66	4.29	2.53	2.11	0.54	0.36	0.06	23.67
4.85	9.69	9.47	9.36	8.13	6.41	4.30	2.15	1.81	0.66	0.30	0.11	23.67
4.95	10.19	9.64	9.78	8.16	7.15	5.04	2.61	1.06	0.60	0.24	0.11	23.67
5.05	10.85	10.40	9.72	9.19	6.61	4.91	3.37	0.60	0.69	0.30	0.11	23.67
5.15	10.84	10.36	9.93	8.80	6.99	4.70	2.94	1.51	0.57	0.24	0.06	23.67
5.25	11.62	10.76	10.26	8.93	7.56	5.86	3.07	1.21	0.63	0.47	0.11	23.67
5.35	10.86	11.02	11.02	9.08	7.98	5.54	3.30	2.11	0.69	0.12	0.11	23.67
5.45	20.70	19.90	19.33	17.18	14.59	9.77	6.54	1.66	1.51	0.77	0.33	23.67
5.55	22.48	21.55	20.63	17.18	14.48	10.84	6.82	4.08	1.72	0.65	0.22	23.68
5.65	22.15	22.23	20.31	17.65	15.43	11.71	6.59	3.17	1.54	0.30	0.17	23.69
5.75	21.74	22.59	20.85	18.45	16.05	11.27	7.03	4.83	1.72	0.65	0.17	23.71
5.85	23.21	22.87	21.16	20.01	17.36	11.84	8.02	3.32	2.02	0.83	0.17	23.73
5.95	25.63	23.71	23.16	19.43	17.73	13.02	8.71	5.13	2.08	1.30	0.22	23.75
6.05	25.35	24.97	23.22	21.09	18.18	12.77	7.82	4.68	2.45	0.89	0.33	23.78
6.15	25.41	25.41	23.99	22.29	19.07	13.55	9.66	3.77	2.11	0.65	0.17	23.81
6.25	26.52	25.86	24.22	21.92	18.53	13.71	9.25	4.38	2.17	0.42	0.22	23.85
6.35	27.86	26.38	26.22	23.49	21.07	14.48	9.43	4.23	2.39	0.89	0.22	23.89

Midpt.	11000	2700	910	240	84	26	8.8	2.7	0.92	0.31	Volume	
6.45	28.43	28.32	26.21	24.30	20.23	14.62	9.35	3.92	2.48	0.89	0.39	23.93
6.55	30.72	27.98	27.76	25.73	20.45	15.09	10.14	5.43	2.96	1.48	0.33	23.98
6.65	29.81	30.14	29.46	25.73	21.82	17.16	11.88	7.70	2.81	1.13	0.33	24.03
6.75	32.01	30.94	30.26	26.30	22.06	17.11	11.42	5.13	2.66	1.01	0.44	24.09
6.85	33.43	32.24	31.37	27.02	24.07	18.66	11.37	6.34	2.66	0.83	0.39	24.15
6.95	34.10	33.40	31.79	28.76	25.06	18.87	13.49	7.25	2.72	1.01	0.28	24.22
7.05	35.53	35.27	32.92	30.66	25.44	19.43	13.03	5.59	3.02	0.95	0.39	24.28
7.15	36.97	36.00	34.09	30.82	27.93	20.00	14.10	6.34	3.14	1.42	0.28	24.36
7.25	38.69	36.61	36.33	32.19	29.10	22.18	14.54	6.94	3.71	1.36	0.61	24.44
7.35	39.25	39.14	37.23	34.39	29.10	22.34	15.38	6.94	4.11	1.19	0.39	24.52
7.45	41.12	40.50	37.53	35.68	31.97	23.93	15.28	9.36	3.65	1.07	0.55	24.60
7.55	42.24	41.08	39.33	36.83	32.77	24.84	16.71	7.55	3.86	1.54	0.72	24.69
7.65	43.27	42.52	40.91	37.42	32.51	26.77	17.73	9.81	4.44	1.66	1.00	24.79
7.75	43.97	42.86	42.74	39.28	33.65	25.41	17.14	9.21	4.62	1.54	0.39	24.89
7.85	47.05	45.33	43.47	39.17	35.44	25.00	17.63	11.17	4.59	1.78	0.61	24.99
7.95	45.80	45.54	43.95	40.31	36.45	27.73	18.29	10.42	4.68	1.66	0.50	25.10
8.05	48.32	46.24	45.63	42.06	36.55	29.30	19.59	12.98	5.49	1.84	0.61	25.21
8.15	47.85	47.06	45.79	42.20	38.01	28.01	20.82	10.72	4.89	1.84	0.50	25.33
8.25	48.16	46.64	45.93	42.59	38.18	29.30	21.08	7.40	5.89	2.31	0.72	25.45
8.35	48.45	48.06	46.94	43.32	38.76	28.69	20.36	12.38	4.92	2.37	0.61	25.58
8.45	47.59	46.27	45.98	41.73	38.56	30.01	19.77	14.94	6.10	2.08	0.50	25.71
8.55	47.33	47.24	45.51	42.27	37.74	29.00	20.39	13.13	5.83	2.02	0.66	25.85
8.65	48.07	47.86	44.48	41.92	37.66	30.39	19.49	10.42	6.22	1.72	0.83	25.99
8.75	46.41	45.55	44.67	42.15	35.06	29.87	19.44	11.47	5.37	2.13	0.77	26.14
8.85	44.89	44.77	42.45	40.67	36.85	28.55	20.67	10.87	4.89	1.72	0.22	26.29
8.95	43.54	44.10	41.87	39.84	35.56	28.00	19.49	9.51	6.31	1.66	0.72	26.44
9.05	42.90	41.42	41.69	38.26	33.76	29.09	20.44	9.66	5.04	1.72	1.00	26.60
9.15	41.34	41.14	40.30	37.32	34.25	26.03	20.39	13.44	4.92	1.78	0.28	26.77
9.25	40.45	39.30	38.72	35.69	32.19	26.91	19.57	11.17	5.46	2.13	0.22	26.94
9.35	38.54	37.18	37.26	35.93	31.38	26.80	18.44	11.62	4.44	2.13	0.39	27.12
9.45	38.51	36.61	35.76	33.93	31.27	24.41	18.90	8.00	4.74	2.43	0.66	27.30
9.55	36.33	36.73	35.57	33.19	30.58	24.75	17.68	9.96	4.71	1.48	0.77	27.49
9.65	35.23	34.21	34.05	32.23	30.34	22.68	17.37	10.27	4.62	1.30	0.44	27.69
9.75	34.28	34.16	33.43	30.93	27.54	23.98	16.73	9.36	4.71	1.84	0.39	27.89
9.85	33.93	32.84	32.27	30.68	29.67	23.71	16.45	7.85	4.62	1.25	0.61	28.09
9.95	31.96	32.28	31.50	30.09	26.05	22.07	17.47	9.36	4.98	1.78	0.77	28.31
10.05	32.23	31.40	30.79	28.83	26.43	21.18	14.89	9.21	3.96	1.78	0.61	28.52
10.15	30.16	30.83	30.54	28.97	26.15	21.78	16.45	9.21	4.80	1.13	0.44	28.75
10.25	30.10	29.44	29.51	27.45	25.91	20.71	14.51	9.06	4.20	1.19	0.50	28.98
10.35	29.76	28.89	28.30	27.42	25.46	21.32	15.81	8.60	4.23	1.84	0.44	29.21
10.45	29.28	29.14	27.41	26.73	24.56	20.39	14.87	8.76	5.07	1.42	0.28	29.46
10.55	28.32	27.15	27.37	26.53	22.21	20.75	15.81	9.06	4.62	1.25	0.44	29.71
10.65	27.27	27.39	26.95	25.82	23.10	20.14	13.77	11.32	4.38	1.48	0.28	29.96
10.75	26.59	26.85	26.55	24.64	23.82	20.52	14.94	8.76	3.80	1.19	0.33	30.23
10.85	26.51	26.43	26.04	24.74	23.85	18.27	13.97	9.06	4.17	1.13	0.39	30.50

Midpt.	11000	2700	910	240	84	26	8.8	2.7	0.92	0.31	Volume	
10.95	25.89	25.50	25.78	25.31	24.51	18.48	14.03	6.79	4.05	1.66	0.39	30.77
11.05	25.58	26.45	25.27	23.63	21.29	18.37	14.00	8.76	4.08	0.89	0.72	31.06
11.15	25.10	25.73	24.28	23.54	21.99	18.86	14.26	7.10	3.80	1.36	0.33	31.35
11.25	25.38	24.86	24.73	23.13	21.20	17.89	13.46	6.64	4.35	1.36	0.22	31.65
11.35	24.40	25.14	23.61	23.02	20.46	17.66	14.71	8.00	4.20	1.30	0.66	31.96
11.45	24.54	23.98	23.54	23.22	20.90	18.98	12.77	8.15	4.02	1.54	0.28	32.27
11.55	24.61	23.84	23.26	22.72	21.13	17.50	13.87	6.49	4.08	1.19	0.11	32.60
11.65	23.88	24.21	22.72	22.12	20.92	17.78	12.82	8.76	3.92	1.78	0.33	32.93
11.75	23.49	23.55	22.44	21.68	19.81	18.25	13.05	8.60	4.35	1.54	0.28	33.27
11.85	23.03	22.58	22.25	21.64	20.77	17.61	12.90	8.91	3.74	1.54	0.83	33.62
11.95	22.72	23.17	22.98	21.78	19.62	17.50	13.54	6.64	4.17	1.42	0.61	33.98
12.05	22.72	22.36	22.72	21.17	20.43	18.37	12.59	8.45	3.74	1.25	0.33	34.34
12.15	21.89	22.99	21.75	21.64	20.43	17.11	12.72	7.85	3.65	1.72	0.11	34.72
12.25	22.20	22.39	22.38	20.43	19.59	16.77	13.26	8.60	3.71	1.66	0.61	35.10
12.35	22.22	21.64	22.26	20.66	20.31	17.53	13.74	6.64	3.89	1.19	0.06	35.50
12.45	23.18	21.38	21.59	20.79	19.34	17.55	12.93	10.11	4.32	1.19	0.55	35.90
12.55	22.22	22.80	21.94	20.57	19.78	15.80	11.83	9.06	3.99	1.30	0.33	36.32
12.65	21.42	21.44	20.62	20.85	18.45	17.14	11.85	9.21	4.08	1.78	0.39	36.74
12.75	21.54	21.68	22.29	20.51	19.00	16.57	13.23	7.40	4.32	1.54	0.28	37.18
12.85	21.76	22.19	22.06	20.10	20.13	17.18	12.34	8.00	3.99	1.36	0.22	37.63
12.95	21.36	21.84	21.43	20.34	19.94	16.91	12.42	8.76	4.26	1.36	0.39	38.09
13.05	22.07	21.84	20.41	20.62	19.74	16.39	11.75	8.91	4.89	1.72	0.39	38.56
13.15	21.96	21.72	21.36	21.43	19.26	16.78	12.31	6.94	3.65	0.83	0.33	39.04
13.25	22.13	21.44	21.79	20.67	19.67	17.19	13.41	8.45	4.08	1.42	0.17	39.54
13.35	21.27	20.72	20.71	20.41	19.59	17.59	12.93	9.21	3.74	1.84	0.44	40.04
13.45	22.89	22.01	20.78	20.96	19.27	16.59	13.41	5.74	4.35	1.25	0.39	40.56
13.55	21.92	22.02	21.32	20.90	20.09	16.91	14.08	7.70	4.53	1.19	0.28	41.10
13.65	21.49	21.44	21.96	20.90	20.40	17.50	11.90	8.30	4.38	1.36	0.50	41.65
13.75	21.89	21.69	21.26	21.00	19.56	16.78	13.23	8.91	4.62	1.84	0.50	42.21
13.85	21.28	22.06	21.97	20.67	19.89	18.07	13.57	8.60	3.92	1.36	0.44	42.78
13.95	21.94	21.37	21.84	21.10	20.03	18.00	15.00	8.76	5.13	1.42	0.61	43.38
14.05	21.99	21.68	21.43	21.76	19.42	16.48	13.44	9.06	4.29	1.07	0.61	43.98
14.15	22.19	21.14	21.92	20.56	20.01	17.69	13.85	11.62	5.04	1.42	0.50	44.61
14.25	22.11	21.66	22.40	21.28	19.47	17.55	14.03	8.76	5.31	2.31	0.33	45.25
14.35	21.86	22.42	22.32	21.07	19.86	17.62	13.85	10.42	4.14	1.30	0.28	45.91
14.45	22.62	22.38	22.62	21.50	20.35	17.84	13.69	8.15	4.65	1.78	0.61	46.58
14.55	22.46	22.64	22.99	21.73	20.75	18.64	14.23	11.47	4.41	1.96	0.50	47.28
14.65	23.70	22.93	22.49	22.53	21.39	17.71	15.35	9.51	5.28	1.96	0.33	47.99
14.75	23.18	22.98	22.39	22.51	20.66	18.69	14.97	8.45	5.62	2.19	0.28	48.72
14.85	23.76	22.76	22.17	21.62	21.74	18.09	14.87	9.36	5.37	1.84	0.22	49.48
14.95	24.06	23.89	22.80	21.80	20.45	18.98	14.64	8.45	4.95	1.66	0.33	50.25
15.05	23.18	24.11	22.82	22.60	20.83	19.44	15.48	12.08	5.56	2.25	0.33	51.05
15.15	22.86	23.60	22.96	23.12	22.07	19.82	15.12	10.27	5.22	1.78	0.55	51.87
15.25	22.98	23.75	23.22	23.20	21.72	19.34	15.71	10.27	5.13	1.84	0.50	52.72
15.35	24.24	22.72	23.84	22.12	21.72	19.30	14.49	10.27	5.40	1.66	0.77	53.59

Midpt.	11000	2700	910	240	84	26	8.8	2.7	0.92	0.31	Volume	
15.45	14.77	15.44	14.91	13.33	11.52	10.82	8.76	5.89	2.78	1.96	1.05	54.48
15.55	11.89	10.79	11.48	10.97	9.98	8.70	6.49	5.28	3.17	1.54	0.55	55.41
15.65	11.36	11.48	11.02	11.25	10.87	9.62	6.59	4.38	3.23	0.89	0.22	56.36
15.75	11.78	11.12	11.42	11.11	10.10	8.64	7.77	4.08	2.87	1.48	0.50	57.34
15.85	11.46	12.17	11.86	11.68	9.91	9.09	7.87	5.13	3.26	1.60	0.61	58.35
15.95	12.07	12.24	11.58	11.54	10.68	9.14	7.46	4.68	2.87	1.54	0.72	59.40
16.05	12.19	12.83	12.34	11.37	10.62	10.16	8.94	4.68	2.72	0.95	0.83	60.48
16.15	13.39	12.88	12.38	11.58	10.70	9.62	8.20	7.85	3.26	1.25	0.39	61.60
16.25	13.01	12.83	12.27	11.94	11.05	9.50	7.89	7.40	3.32	1.25	0.66	62.76
16.35	12.37	12.39	12.78	12.64	11.88	10.82	7.72	5.74	3.74	1.72	0.61	63.95
16.45	12.55	13.28	12.67	11.62	12.01	10.53	9.50	6.19	2.87	1.96	0.44	65.19
16.55	12.91	13.95	12.74	13.19	12.21	10.68	9.15	6.94	3.38	1.42	0.50	66.48
16.65	13.64	12.76	13.75	13.34	11.99	12.45	10.17	6.04	2.81	1.42	0.22	67.81
16.75	13.84	14.23	13.63	11.99	12.85	11.03	9.32	6.19	3.59	1.84	0.33	69.20
16.85	13.49	14.12	13.74	13.72	12.66	10.96	9.55	7.10	3.29	1.60	0.72	70.64
16.95	14.22	14.05	13.71	13.98	14.12	11.53	8.79	7.10	3.17	1.30	0.22	72.14
17.05	14.09	14.72	14.48	13.42	13.54	11.70	9.58	6.49	3.41	2.08	0.44	73.70
17.15	15.38	14.69	15.09	13.54	13.28	12.59	10.53	6.64	3.44	1.78	0.61	75.33
17.25	14.78	14.90	16.25	14.82	14.58	11.52	9.09	5.89	4.08	1.30	0.66	77.04
17.35	16.18	15.08	14.83	13.88	14.74	12.12	10.96	6.04	3.47	1.66	0.50	78.83
17.45	15.33	15.38	14.72	15.24	15.20	12.16	10.30	6.64	4.56	2.61	0.55	80.70
17.55	15.75	15.88	15.72	16.22	14.34	12.93	10.50	5.13	4.14	1.54	0.50	82.67
17.65	15.72	15.87	16.13	15.91	14.53	13.93	11.01	6.49	4.14	1.78	0.77	84.75
17.75	17.18	16.00	17.09	16.19	15.45	12.43	10.68	6.94	4.47	2.55	1.22	86.94
17.85	17.05	17.52	16.31	15.27	15.75	14.43	10.93	6.64	4.53	1.90	0.44	89.27
17.95	18.49	18.08	18.57	16.93	16.59	13.87	12.31	9.81	4.77	1.60	0.44	91.74
18.05	17.93	18.52	17.49	17.95	17.23	15.36	13.39	9.21	4.80	2.31	0.50	94.39
18.15	18.36	18.19	18.42	18.21	16.79	15.11	12.42	9.51	4.44	1.84	0.55	97.24
18.25	19.07	19.12	19.11	17.64	17.66	14.93	12.88	8.91	5.31	1.96	0.50	100.32
18.35	20.21	20.02	19.24	18.59	18.82	16.87	12.65	8.76	5.28	2.37	0.66	103.68
18.45	20.40	19.68	21.35	19.06	19.94	15.25	12.82	8.76	5.80	3.26	0.50	107.38
18.55	21.21	21.24	20.60	19.01	19.73	16.86	14.61	9.36	5.56	2.25	0.61	111.53
18.65	21.41	21.51	22.21	21.32	19.81	17.96	15.58	9.51	5.49	2.73	1.00	116.27
18.75	22.50	22.37	23.63	20.84	20.35	18.77	16.71	10.11	7.10	2.25	0.89	121.87
18.85	23.02	24.22	22.84	22.17	22.17	19.16	16.45	11.93	6.67	4.15	0.89	128.90
18.95	25.03	24.96	24.26	24.34	21.70	19.61	17.73	12.38	7.88	3.08	0.83	139.22
19.05	26.28	27.29	27.24	24.78	23.70	22.39	21.25	14.04	7.64	3.50	0.89	12225.45
19.15	251.84	253.80	247.84	248.80	246.04	226.19	204.88	147.34	88.19	39.44	9.91	2239.26
19.25	302.57	305.25	303.53	301.84	295.41	277.47	253.86	183.41	110.59	54.09	12.01	2239.26
19.35	304.70	308.13	299.24	301.53	292.72	277.67	244.02	179.64	108.93	48.04	12.07	2239.26
19.45	304.01	302.79	302.27	300.47	291.15	277.35	246.45	182.51	111.41	52.78	11.85	2239.26
19.55	302.34	303.45	302.64	295.95	290.39	274.47	246.99	175.11	112.10	51.12	11.57	2239.26
19.65	300.46	299.71	302.06	294.25	290.48	273.67	244.07	188.24	109.63	51.30	11.18	2239.26
19.75	300.75	296.12	298.59	293.93	287.61	274.27	246.99	187.94	109.29	49.28	12.18	2239.26
19.85	294.68	298.29	299.54	295.54	289.37	272.45	242.13	178.43	109.14	50.59	11.46	2239.26

Midpt.	11000	2700	910	240	84	26	8.8	2.7	0.92	0.31	Volume	
19.95	296.22	298.35	296.55	293.09	294.39	274.77	240.88	178.89	105.82	52.37	11.96	2239.26
20.05	299.26	296.80	294.73	291.40	284.74	271.63	246.17	175.87	110.38	46.44	12.73	2239.26
20.15	292.36	295.61	291.56	291.18	286.22	277.83	248.16	174.36	107.39	49.46	12.29	2239.26
20.25	295.19	291.00	291.19	289.11	284.62	274.29	243.10	181.15	105.22	51.30	11.85	2239.26
20.35	289.75	292.49	292.90	289.31	283.68	268.28	240.62	180.24	107.66	49.52	12.79	2239.26
20.45	289.94	288.00	288.62	285.56	284.29	270.17	238.86	178.58	107.75	50.35	11.96	2239.26
20.55	290.99	286.37	289.34	290.95	281.86	264.76	239.42	180.39	108.81	47.38	11.01	2239.26
20.65	288.18	290.56	287.28	284.26	274.34	266.04	234.70	178.28	108.90	51.12	11.62	2239.26
20.75	289.21	287.37	287.00	282.08	280.25	265.70	239.65	174.21	107.87	49.10	12.12	2239.26
20.85	284.46	288.77	286.51	285.79	277.90	263.67	236.64	171.19	105.34	49.46	11.35	2239.26
20.95	283.03	288.07	286.57	284.09	279.62	260.47	232.86	182.81	106.61	48.04	11.18	2239.26
21.05	286.14	285.43	283.08	285.52	274.37	263.56	230.43	182.51	108.84	49.16	11.29	2239.26
21.15	285.62	286.50	284.11	283.79	275.73	261.13	231.91	170.58	105.40	51.60	12.01	2239.26
21.25	285.46	283.44	282.93	280.22	275.63	256.81	235.03	179.04	105.91	44.83	12.23	2239.26
21.35	279.45	281.91	281.25	278.21	273.05	265.11	235.21	175.72	109.11	48.04	12.29	2239.26
21.45	279.83	280.96	282.53	281.75	272.83	257.65	227.09	177.22	104.13	48.51	11.46	2239.26
21.55	281.31	281.57	280.58	274.66	268.35	255.60	232.35	162.73	104.34	47.38	12.79	2239.26
21.65	278.56	276.36	278.47	276.95	269.34	256.49	226.98	176.32	105.55	48.16	11.51	2239.26
21.75	278.82	276.65	278.62	275.12	273.55	258.53	227.39	174.66	106.12	46.26	11.24	2239.26
21.85	277.71	275.45	275.27	276.32	271.27	255.24	227.24	178.28	101.93	47.98	10.68	2239.26
21.95	275.98	277.59	276.64	274.70	266.58	254.37	232.86	166.96	105.07	47.74	11.18	2239.26
22.05	274.14	274.44	273.04	273.82	268.66	254.51	227.24	182.96	103.01	47.33	10.68	2239.26
22.15	277.32	273.83	272.91	272.16	268.47	250.94	229.87	177.53	99.72	48.16	10.79	2239.26
22.25	270.56	276.69	271.36	271.74	266.58	252.10	225.88	171.94	101.75	48.21	9.96	2239.26
22.35	270.78	273.78	269.65	269.80	262.63	250.46	227.16	164.85	102.80	47.56	11.40	2239.26
22.45	270.96	272.84	272.18	271.00	266.11	252.90	224.94	174.96	103.35	43.89	9.91	2239.26
22.55	272.99	272.25	267.54	267.24	265.45	249.15	224.86	168.32	102.26	47.50	10.46	2239.26
22.65	270.04	268.23	269.58	269.76	262.83	257.01	225.27	171.49	100.69	45.55	11.40	2239.26
22.75	270.84	268.88	271.26	269.26	260.64	248.15	225.81	170.58	95.38	47.50	11.96	2239.26
22.85	271.97	267.28	268.15	268.12	265.22	252.10	225.99	174.21	102.38	48.27	10.63	2239.26
22.95	270.10	271.55	269.86	265.83	266.34	249.35	219.80	171.49	97.43	44.54	10.52	2239.26
23.05	268.31	270.53	270.19	265.86	264.88	250.60	221.77	170.43	100.72	44.95	10.41	2239.26
23.15	266.50	266.94	270.44	266.95	261.44	246.72	220.95	174.96	101.90	44.72	10.24	2239.26
23.25	269.82	265.78	266.71	265.15	263.35	247.80	225.07	168.62	103.47	44.89	12.29	2239.26
23.35	265.12	267.19	263.66	259.74	256.73	248.65	221.57	160.47	100.78	47.86	11.62	2239.26
23.45	266.89	263.97	263.24	263.03	256.63	246.03	219.01	174.51	100.51	46.26	9.96	2239.26
23.55	264.17	267.46	261.04	263.38	259.60	241.94	220.98	177.83	101.93	47.62	10.24	2239.26
23.65	263.72	263.13	262.42	261.93	259.58	247.53	221.39	168.02	99.09	47.62	10.63	2239.26
23.75	261.44	264.65	262.98	263.80	255.59	249.06	217.91	168.17	99.69	44.06	10.07	2239.26
23.85	261.19	263.27	260.49	262.05	253.63	247.24	216.66	174.05	99.24	48.04	12.45	2239.26
23.95	262.21	261.17	265.41	259.08	254.45	242.96	220.26	168.17	99.90	43.29	12.51	2239.26
24.05	258.29	260.09	263.25	258.30	257.55	245.46	219.86	167.41	101.20	43.94	10.35	2239.26
24.15	265.67	261.88	261.40	257.66	254.35	241.47	214.16	160.32	100.24	46.91	12.62	2239.26
24.25	261.75	260.67	260.27	256.55	255.32	241.26	217.25	161.83	99.06	45.55	10.57	2239.26
24.35	261.83	261.40	259.27	258.96	250.41	240.31	214.13	161.98	99.21	46.32	8.41	2239.26

Midpt.	11000	2700	910	240	84	26	8.8	2.7	0.92	0.31	Volume	
24.45	257.88	260.88	259.49	256.09	253.34	239.97	221.72	156.09	99.03	48.57	12.18	2239.26
24.55	256.33	256.68	263.53	255.44	250.29	242.64	212.96	164.09	95.83	42.94	10.57	2239.26
24.65	255.69	257.91	257.01	254.14	249.45	239.51	213.21	155.18	96.92	45.84	11.51	2239.26
24.75	257.63	259.09	257.38	255.83	248.21	242.46	214.98	165.75	99.09	45.07	8.75	2239.26
24.85	259.53	259.90	259.29	253.98	253.12	241.55	215.21	165.60	97.40	43.35	11.13	2239.26
24.95	258.57	259.26	258.04	256.97	249.72	235.65	219.32	157.60	95.56	45.96	10.90	2239.26
25.05	257.59	258.19	257.74	254.70	248.68	240.33	215.61	166.36	98.79	44.60	9.91	2239.26
25.15	255.61	255.49	260.32	254.33	251.81	239.42	218.53	163.34	97.10	43.65	10.18	2239.26
25.25	255.55	254.24	254.89	255.21	251.29	237.99	214.64	162.13	96.34	42.46	10.52	2239.26
25.35	255.76	254.14	254.62	253.11	246.15	235.65	212.78	170.13	96.52	44.95	10.13	2239.26
25.45	255.82	256.05	254.52	250.54	244.15	236.83	213.65	163.49	97.13	43.59	11.07	2239.26
25.55	254.16	252.04	250.80	252.53	252.85	234.74	213.16	156.24	98.70	43.23	10.24	2239.26
25.65	250.73	255.02	251.86	254.75	246.82	232.78	210.35	165.15	95.77	45.61	11.18	2239.26
25.75	253.08	251.76	254.78	249.34	247.82	235.31	212.52	158.51	95.65	43.29	9.41	2239.26
25.85	251.09	250.36	253.65	254.27	247.24	238.78	213.21	159.41	97.85	44.00	11.13	2239.26
25.95	255.15	253.25	249.76	250.59	246.04	235.23	211.53	161.68	93.87	45.01	10.74	2239.26
26.05	251.52	248.89	251.12	253.14	242.94	233.37	214.08	154.28	98.36	43.83	9.80	2239.26
26.15	251.00	253.96	254.42	251.88	244.50	230.42	213.44	153.83	96.19	43.83	10.79	2239.26
26.25	252.18	249.06	251.51	247.15	247.37	235.24	214.47	162.88	99.39	47.50	10.41	2239.26
26.35	249.55	252.03	249.03	250.12	253.24	234.03	213.80	165.15	96.85	43.41	10.68	2239.26
26.45	250.73	254.95	251.78	250.30	250.24	236.87	208.44	172.85	97.97	42.40	10.96	2239.26
26.55	254.60	252.75	249.90	251.48	247.64	233.51	208.90	167.26	96.31	47.33	10.35	2239.26
26.65	251.01	249.25	250.84	249.25	241.58	231.74	210.56	166.20	96.55	44.12	9.80	2239.26
26.75	250.97	248.70	252.18	247.72	246.36	235.87	209.23	158.81	95.19	42.64	9.47	2239.26
26.85	249.22	249.91	250.87	246.62	243.51	234.42	210.66	155.34	94.32	44.83	10.30	2239.26
26.95	248.20	251.17	248.23	248.74	239.50	235.55	210.81	164.85	95.04	43.29	10.30	2239.26
27.05	250.02	245.55	248.07	253.00	242.09	231.51	209.66	163.19	95.38	45.66	10.24	2239.26
27.15	253.70	254.05	250.29	248.74	244.60	233.17	215.08	162.43	96.61	45.25	12.34	2239.26
27.25	250.83	249.38	250.34	250.00	242.51	237.26	212.86	164.39	96.55	46.26	11.29	2239.26
27.35	247.60	249.43	248.33	245.86	244.25	237.60	216.79	167.41	97.31	43.47	10.96	2239.26
27.45	250.19	246.88	248.91	247.66	239.72	232.31	215.56	153.83	97.25	45.31	11.79	2239.26
27.55	251.52	250.28	248.88	248.60	245.19	236.10	209.38	158.66	96.55	47.27	10.35	2239.26
27.65	253.71	248.91	249.14	245.49	247.02	231.83	213.39	163.79	97.10	45.25	10.68	2239.26
27.75	250.17	253.46	251.73	247.50	244.57	237.21	209.59	161.22	98.03	46.14	10.41	2239.26
27.85	248.02	250.41	249.24	249.75	245.89	231.71	212.45	152.01	95.74	46.26	10.85	2239.26
27.95	249.20	252.90	250.94	246.15	241.94	230.99	210.53	166.96	96.34	44.66	11.57	2239.26
28.05	248.05	250.23	251.38	247.67	248.14	235.85	217.22	149.90	96.46	44.72	10.24	2239.26
28.15	248.66	249.73	248.40	249.94	248.24	235.90	210.17	161.37	95.16	42.64	10.96	2239.26
28.25	251.24	248.33	244.01	245.78	243.03	232.99	209.10	155.03	97.16	43.06	10.18	2239.26
28.35	249.96	247.86	249.23	247.12	245.17	231.42	215.26	148.84	96.04	44.36	11.79	2239.26
28.45	251.16	249.65	247.67	249.50	242.62	236.90	213.55	152.62	97.52	44.83	10.30	2239.26
28.55	248.71	244.61	249.81	249.86	244.17	231.03	216.76	152.17	98.00	46.44	11.07	2239.26
28.65	250.83	249.72	247.15	247.14	242.52	234.64	212.42	158.20	96.07	43.06	10.24	2239.26
28.75	249.11	251.14	248.64	247.17	243.58	234.99	206.44	161.68	95.59	41.75	10.57	2239.26
28.85	249.19	248.66	251.63	246.20	244.13	233.42	208.67	159.41	97.10	43.59	9.30	2239.26

Midpt.	11000	2700	910	240	84	26	8.8	2.7	0.92	0.31	Volume	
28.95	248.06	247.68	248.69	250.62	247.42	231.12	209.56	164.70	96.82	44.48	10.24	2239.26
29.05	249.86	250.98	245.88	248.78	239.91	234.30	214.13	163.19	93.74	46.26	10.79	2239.26
29.15	249.66	246.68	249.18	249.66	241.23	239.21	209.64	155.94	95.71	43.71	10.13	2239.26
29.25	247.93	249.31	248.18	245.00	247.10	230.76	209.02	154.28	97.31	46.08	11.96	2239.26
29.35	251.35	249.43	249.07	251.09	244.23	234.74	215.23	160.02	96.58	42.34	11.29	2239.26
29.45	250.35	248.48	248.50	245.93	242.44	238.87	213.14	160.02	97.94	44.18	9.91	2239.26
29.55	250.95	250.01	246.34	251.16	243.31	233.94	210.89	161.07	96.82	45.49	11.35	2239.26
29.65	249.87	249.46	249.13	244.39	242.41	237.56	211.68	164.54	95.28	45.78	10.90	2239.26
29.75	248.32	248.74	250.50	245.17	246.01	234.05	210.28	166.96	95.31	44.36	11.13	2239.26
29.85	248.53	251.18	249.11	247.47	245.79	235.78	212.98	152.62	93.47	44.48	11.29	2239.26
29.95	247.34	249.04	248.79	245.59	244.37	237.15	213.21	164.09	92.57	45.49	10.30	2239.26
30.05	254.38	247.42	248.13	246.37	246.82	235.40	212.86	163.34	99.06	45.78	11.24	2239.26
30.15	249.25	247.80	250.38	248.09	241.73	234.99	212.34	164.24	93.90	45.61	11.46	2239.26
30.25	245.74	247.96	246.23	245.47	246.01	236.94	216.36	164.39	97.07	43.41	11.68	2239.26
30.35	250.57	248.38	249.07	249.62	245.29	236.56	211.48	164.70	94.74	43.83	10.74	2239.26
30.45	247.65	250.03	248.80	250.14	244.12	233.42	208.87	170.43	95.53	45.37	10.85	2239.26
30.55	247.51	247.67	247.14	246.82	246.45	231.81	215.84	150.05	95.95	44.24	11.13	2239.26
30.65	246.13	248.43	249.27	247.18	240.86	237.65	210.58	155.94	93.47	42.23	10.79	2239.26
30.75	249.31	245.99	250.21	245.96	243.86	237.74	212.04	168.47	99.39	46.14	10.96	2239.26
30.85	251.31	249.68	249.28	249.86	243.55	235.17	212.34	164.70	97.43	43.35	11.46	2239.26
30.95	249.91	247.36	248.57	245.32	247.30	229.65	209.61	163.64	97.67	45.78	10.74	2239.26
31.05	249.87	248.65	249.80	247.08	242.25	237.80	215.31	159.56	96.10	46.38	10.52	2239.26
31.15	247.26	247.54	250.32	248.83	246.18	233.98	209.53	154.28	96.67	41.22	11.18	2239.26
31.25	248.45	246.32	250.91	246.53	242.42	238.72	212.55	156.39	98.76	46.85	11.46	2239.26
31.35	249.13	249.17	247.93	247.52	239.55	229.03	207.54	157.30	94.71	45.84	11.46	2239.26
31.45	247.61	248.52	247.59	246.66	245.86	235.67	210.12	170.58	97.88	43.77	11.07	2239.26
31.55	243.70	247.74	248.19	248.16	241.94	239.15	216.74	161.83	98.18	43.83	10.90	2239.26
31.65	248.67	246.60	249.62	249.50	241.08	232.81	211.30	160.17	95.59	43.89	11.46	2239.26
31.75	245.68	247.40	247.74	244.25	242.94	230.73	212.11	160.17	97.88	43.23	12.79	2239.26
31.85	247.11	247.88	251.61	244.36	245.01	236.67	209.71	159.71	98.09	44.72	10.30	2239.26
31.95	246.89	249.76	249.67	248.37	241.70	235.89	210.28	152.92	98.39	45.43	10.46	2239.26
32.05	248.08	249.11	244.86	247.04	243.88	231.23	214.62	159.11	96.52	42.76	12.34	2239.26
32.15	247.18	244.65	248.06	246.34	244.37	234.48	207.95	163.94	96.95	45.19	11.24	2239.26
32.25	245.07	251.13	247.57	248.15	247.15	237.81	210.84	167.26	96.10	46.32	13.06	2239.26
32.35	246.40	248.15	250.60	249.29	250.49	233.87	210.22	158.51	98.97	46.50	11.68	2239.26
32.45	248.07	245.04	246.45	246.92	241.40	234.46	210.71	163.19	98.52	43.29	10.57	2239.26
32.55	245.42	246.14	251.86	247.98	244.99	233.05	211.76	168.92	98.49	46.50	12.45	2239.26
32.65	247.61	245.31	249.93	247.68	244.38	233.21	216.76	167.11	96.28	43.94	11.46	2239.26
32.75	246.07	247.47	249.60	248.24	246.68	232.51	208.97	160.92	97.07	42.94	10.85	2239.26
32.85	249.93	247.44	245.11	248.62	239.42	239.19	218.25	167.26	99.54	45.07	11.57	2239.26
32.95	247.07	247.98	246.01	245.05	243.16	234.92	212.06	151.56	98.58	44.42	10.63	2239.26
33.05	248.29	246.28	248.42	245.11	243.33	234.62	211.42	169.38	95.86	45.49	12.07	2239.26
33.15	247.28	244.39	247.64	248.88	243.55	235.23	211.81	163.94	96.01	45.31	11.24	2239.26
33.25	246.54	245.92	244.30	250.02	240.81	231.08	211.83	159.11	96.98	44.48	11.85	2239.26
33.35	248.15	246.28	246.27	247.04	240.74	229.92	209.87	163.94	96.76	44.83	12.07	2239.26

Midpt.	11000	2700	910	240	84	26	8.8	2.7	0.92	0.31	Volume	
33.45	246.58	244.38	245.88	243.61	243.48	232.44	214.72	160.47	98.30	43.89	12.40	2239.26
33.55	245.91	246.97	247.43	246.11	242.93	237.83	206.29	162.13	97.52	44.00	11.35	2239.26
33.65	243.75	247.39	247.07	246.71	244.72	234.76	212.88	155.64	97.76	41.93	10.30	2239.26
33.75	246.46	245.90	249.70	247.10	243.23	232.10	210.71	158.36	96.37	46.61	10.07	2239.26
33.85	246.97	244.66	247.44	245.91	242.20	233.85	213.80	163.19	94.35	45.07	11.35	2239.26
33.95	244.71	249.00	246.14	246.50	239.30	230.49	217.79	152.92	98.18	44.60	11.07	2239.26
34.05	244.76	245.87	247.11	247.81	243.18	228.64	213.32	160.17	95.38	44.48	9.63	2239.26
34.15	247.36	247.84	248.78	244.52	241.99	231.94	208.46	167.87	94.26	44.83	11.18	2239.26
34.25	247.47	247.84	246.87	242.56	238.46	230.44	210.10	160.92	97.91	45.49	10.02	2239.26
34.35	243.66	246.18	247.34	244.59	244.07	234.44	215.36	157.15	94.38	44.72	11.35	2239.26
34.45	245.20	247.26	246.70	248.01	242.42	234.96	211.04	153.52	98.18	45.01	12.07	2239.26
34.55	244.02	247.35	245.58	245.81	242.44	236.30	213.95	153.07	94.44	46.38	11.51	2239.26
34.65	244.01	246.52	247.38	245.90	246.10	235.49	212.14	155.64	97.64	42.94	11.51	2239.26
34.75	242.64	245.74	246.04	247.18	242.66	234.15	212.37	158.96	97.73	42.28	12.18	2239.26
34.85	246.38	246.02	246.44	247.03	244.47	235.64	210.81	163.64	96.55	44.60	12.29	2239.26
34.95	247.63	247.05	243.68	249.85	242.66	235.65	209.41	165.90	96.37	45.78	10.63	2239.26
35.05	245.42	248.21	247.64	247.19	241.70	233.80	211.58	160.92	96.98	40.80	10.79	2239.26
35.15	247.25	244.76	244.61	244.77	239.22	234.19	209.05	159.71	95.10	45.49	9.96	2239.26
35.25	247.19	246.59	246.20	242.46	241.33	237.28	216.69	155.64	96.01	44.95	9.91	2239.26
35.35	245.09	246.20	247.59	246.60	244.80	231.90	212.09	176.62	96.37	46.20	11.62	2239.26
35.45	246.79	247.03	245.37	246.93	245.47	230.19	211.02	163.49	96.98	49.40	11.13	2239.26
35.55	243.19	249.62	245.56	246.09	241.62	232.85	212.04	163.03	97.61	42.88	13.06	2239.26
35.65	241.91	245.45	245.84	244.73	240.31	231.03	210.17	159.41	96.92	43.23	10.46	2239.26
35.75	243.69	248.01	245.01	245.74	244.43	236.03	213.06	163.64	98.73	44.83	11.40	2239.26
35.85	245.40	244.88	246.31	244.87	242.35	230.64	215.23	154.88	96.98	44.36	12.62	2239.26
35.95	245.71	243.96	246.88	247.99	244.17	234.31	213.16	162.88	96.95	44.95	11.24	2239.26
36.05	244.50	248.00	244.35	248.95	242.56	234.62	208.84	159.26	94.80	45.43	11.07	2239.26
36.15	246.75	247.02	248.26	243.87	238.58	234.49	209.64	162.58	99.81	46.50	11.96	2239.26
36.25	245.40	246.09	248.19	247.52	245.19	228.62	206.62	167.56	94.98	43.35	10.24	2239.26
36.35	247.15	243.08	245.98	244.04	243.53	237.49	209.00	163.19	96.37	43.41	11.29	2239.26
36.45	244.08	245.76	249.08	246.06	242.02	229.82	208.33	158.51	95.80	42.76	11.62	2239.26
36.55	244.92	244.46	244.58	242.73	240.16	231.90	213.98	163.49	98.61	46.79	9.85	2239.26
36.65	249.43	247.04	245.00	244.79	245.94	229.55	212.65	165.60	95.98	44.60	11.35	2239.26
36.75	248.04	247.01	245.94	244.13	243.11	234.89	211.58	166.51	97.07	44.30	10.74	2239.26
36.85	247.36	244.86	244.93	246.98	244.43	234.92	214.36	157.00	98.15	43.47	9.74	2239.26
36.95	245.96	244.18	248.29	246.71	240.14	235.23	211.12	160.47	96.31	43.83	11.29	2239.26
37.05	243.77	246.95	246.35	242.62	241.55	229.08	214.36	166.05	97.31	43.23	11.73	2239.26
37.15	244.86	247.60	246.07	246.85	244.65	231.15	209.56	157.90	93.65	42.34	11.51	2239.26
37.25	246.97	248.56	247.27	244.61	247.22	229.98	211.86	154.13	99.30	42.17	10.46	2239.26
37.35	247.39	248.08	242.97	246.86	242.24	226.94	211.78	166.51	94.95	42.58	13.06	2239.26
37.45	243.88	247.09	246.55	245.19	242.89	235.96	207.29	160.62	93.65	45.13	10.30	2239.26
37.55	245.33	244.16	245.19	243.60	240.68	233.35	207.93	160.92	94.26	45.78	10.74	2239.26
37.65	246.11	244.36	247.23	246.06	240.86	231.98	210.17	159.86	93.59	43.94	11.01	2239.26
37.75	246.58	242.63	244.11	243.70	243.75	233.56	211.50	171.19	95.47	45.78	10.57	2239.26
37.85	244.98	247.49	244.24	247.05	243.58	232.65	212.83	160.32	96.13	44.95	11.62	2239.26

Midpt.	11000	2700	910	240	84	26	8.8	2.7	0.92	0.31	Volume	
37.95	242.35	246.95	243.95	244.19	244.02	237.06	209.28	154.88	95.74	43.94	11.90	2239.26
38.05	242.49	247.04	244.41	243.72	241.30	228.74	212.63	157.75	96.82	45.37	12.40	2239.26
38.15	245.14	244.36	244.45	243.03	241.63	233.98	212.47	157.90	95.53	43.23	11.29	2239.26
38.25	247.76	246.02	247.18	240.86	247.87	234.30	209.94	160.77	95.80	45.13	12.07	2239.26
38.35	246.88	244.94	246.59	244.84	241.58	232.01	211.81	157.90	97.31	43.47	11.35	2239.26
38.45	246.75	248.36	244.67	246.85	241.23	229.40	213.67	173.00	97.85	42.23	11.35	2239.26
38.55	245.73	244.24	243.84	245.21	241.10	231.85	217.28	168.32	97.31	44.00	11.07	2239.26
38.65	245.31	243.98	244.68	243.72	238.09	230.14	217.20	165.90	97.76	44.72	12.01	2239.26
38.75	242.94	243.62	249.02	245.97	242.59	233.48	212.57	160.47	95.71	45.84	12.84	2239.26
38.85	244.44	249.25	243.96	245.96	242.59	232.44	212.86	161.98	94.53	45.61	10.85	2239.26
38.95	245.57	243.95	244.84	244.12	243.75	232.53	213.65	160.17	96.16	45.25	10.63	2239.26
39.05	243.96	245.42	245.92	245.34	236.25	230.53	211.68	159.86	97.25	42.34	11.29	2239.26
39.15	244.61	246.25	246.62	244.56	240.19	233.64	208.41	162.58	96.31	45.25	10.30	2239.26
39.25	247.01	244.99	245.84	242.56	243.63	232.92	213.75	155.49	94.14	43.23	11.90	2239.26
39.35	245.86	248.16	245.94	244.54	241.94	234.67	213.67	162.43	99.51	42.76	10.13	2239.26
39.45	243.13	244.22	249.12	244.17	242.62	231.83	212.68	161.22	100.30	43.29	10.02	2239.26
39.55	245.80	244.61	246.17	244.26	238.56	238.17	212.29	165.90	95.53	46.44	12.40	2239.26
39.65	242.12	247.95	245.06	245.69	246.40	232.37	212.14	166.36	96.31	41.99	12.18	2239.26
39.75	244.87	245.16	244.35	243.34	238.51	233.46	208.64	166.20	97.31	42.23	11.46	2239.26
39.85	242.95	246.80	244.05	245.68	241.68	233.58	211.30	159.11	97.76	44.78	11.35	2239.26
39.95	245.10	248.41	242.55	244.12	242.07	234.33	211.78	165.45	90.73	43.53	11.46	2239.26
40.05	245.30	244.91	245.54	247.41	242.14	230.21	207.90	164.85	99.27	42.05	11.96	2239.26
40.15	245.13	244.10	244.66	246.49	240.98	232.64	211.09	158.96	94.35	43.94	11.35	2239.26
40.25	241.00	246.96	244.98	246.71	241.60	232.56	212.93	163.03	97.94	44.30	12.23	2239.26
40.35	244.40	246.69	244.11	243.66	242.51	231.03	217.02	160.47	94.65	42.23	11.07	2239.26
40.45	244.89	244.34	246.44	244.55	243.09	230.48	216.20	152.17	97.52	44.30	12.95	2239.26
40.55	243.64	244.08	246.59	245.87	241.48	234.81	209.56	150.81	100.02	43.17	12.01	2239.26
40.65	246.32	245.78	243.66	242.91	239.29	232.48	212.47	164.85	99.30	42.23	11.57	2239.26
40.75	244.66	244.52	244.98	242.19	240.49	228.49	208.21	163.03	96.22	44.95	11.29	2239.26
40.85	244.21	246.47	243.24	242.54	239.45	234.01	208.87	164.39	94.98	40.68	12.23	2239.26
40.95	242.03	243.51	245.04	241.47	240.28	226.99	210.45	168.02	98.76	45.01	11.96	2239.26
41.05	243.79	244.79	240.27	246.69	241.38	233.48	212.50	159.86	98.09	45.84	11.24	2239.26
41.15	246.52	248.70	243.88	248.45	242.10	230.30	207.41	161.07	96.61	45.31	10.90	2239.26
41.25	245.81	243.67	245.18	243.01	243.23	229.87	211.50	164.09	95.80	43.94	10.02	2239.26
41.35	244.58	246.77	246.08	242.07	240.14	236.60	213.06	152.92	95.13	46.14	11.85	2239.26
41.45	243.53	244.78	247.40	246.71	243.18	233.06	217.33	163.19	95.28	44.36	12.23	2239.26
41.55	245.72	245.80	242.88	244.34	244.74	233.99	215.69	158.36	98.00	40.33	11.13	2239.26
41.65	246.22	244.83	244.28	243.38	234.79	233.35	211.78	166.36	92.54	41.87	10.79	2239.26
41.75	246.96	248.12	244.28	244.22	241.00	232.64	208.26	161.37	100.06	44.60	12.29	2239.26
41.85	247.29	244.94	245.23	244.32	242.51	231.12	212.22	169.83	95.16	44.48	10.68	2239.26
41.95	245.88	243.75	244.31	244.49	241.53	231.76	211.83	153.98	93.99	44.06	10.90	2239.26
42.05	245.09	244.89	244.85	240.56	238.61	231.48	206.16	161.68	94.80	40.80	11.79	2239.26
42.15	243.08	242.64	243.14	244.48	242.99	235.96	212.37	149.00	97.52	45.90	12.95	2239.26
42.25	246.19	244.36	245.60	245.27	241.65	232.71	210.35	160.02	96.92	44.95	11.18	2239.26
42.35	242.74	245.67	245.65	243.44	241.33	235.74	211.76	154.73	95.80	44.48	11.79	2239.26

Midpt.	11000	2700	910	240	84	26	8.8	2.7	0.92	0.31	Volume	
42.45	247.58	243.09	242.99	245.60	240.44	232.28	214.26	151.11	97.01	44.89	12.73	2239.26
42.55	242.81	244.45	245.61	244.58	242.30	230.69	208.38	161.98	94.38	44.42	11.24	2239.26
42.65	244.93	244.00	244.61	242.48	239.67	234.73	209.13	157.90	96.43	44.78	11.07	2239.26
42.75	244.82	244.45	244.58	242.69	241.06	232.55	209.76	166.96	96.88	46.50	11.07	2239.26
42.85	243.79	244.84	244.53	246.19	244.03	232.90	209.76	156.39	94.02	45.55	11.46	2239.26
42.95	245.03	240.83	244.36	245.27	241.83	231.48	211.02	154.88	93.99	44.60	11.62	2239.26
43.05	244.68	245.79	242.38	243.54	238.53	230.48	211.12	164.39	95.53	43.17	11.13	2239.26
43.15	242.05	245.15	246.04	244.84	239.10	234.69	210.45	174.21	94.41	39.14	10.90	2239.26
43.25	242.19	245.37	242.42	242.83	242.49	229.07	205.11	163.94	96.73	43.65	12.34	2239.26
43.35	246.32	245.00	244.93	246.05	238.51	234.51	211.58	156.54	98.06	42.52	11.85	2239.26
43.45	246.46	243.90	245.96	242.13	244.35	231.85	214.80	155.18	98.49	45.19	12.40	2239.26
43.55	244.88	246.50	243.22	244.29	240.14	232.65	209.89	160.77	97.49	43.77	10.96	2239.26
43.65	241.18	244.20	242.28	243.33	237.27	235.53	213.24	162.73	95.16	45.61	11.18	2239.26
43.75	243.42	248.34	246.43	243.33	244.35	233.30	208.28	166.66	98.33	43.59	11.07	2239.26
43.85	243.32	247.21	243.82	245.80	240.16	231.15	211.30	155.64	95.31	42.46	12.62	2239.26
43.95	245.79	244.20	244.04	243.44	241.77	236.65	211.65	163.03	95.41	43.17	13.06	2239.26
44.05	245.36	248.41	246.67	245.08	237.81	234.69	214.01	162.58	97.22	41.69	12.84	2239.26
44.15	244.26	240.96	243.42	242.70	244.87	231.64	212.57	161.07	98.30	45.90	10.74	2239.26
44.25	247.70	246.66	247.55	243.73	235.19	230.46	208.21	161.68	97.55	42.05	10.85	2239.26
44.35	241.78	244.54	245.89	245.97	246.68	232.85	213.62	158.36	96.16	40.39	12.18	2239.26
44.45	243.61	243.07	242.41	246.11	243.43	229.99	210.68	162.43	95.44	44.06	13.23	2239.26
44.55	243.86	245.19	244.45	244.51	243.18	232.69	209.99	163.19	94.38	45.37	10.63	2239.26
44.65	243.85	240.62	245.25	239.54	239.99	233.39	216.48	160.62	97.70	43.35	12.01	2239.26
44.75	242.30	244.52	244.18	243.04	239.29	230.92	213.72	175.26	96.82	45.96	12.07	2239.26
44.85	245.34	241.78	246.06	242.96	238.23	232.03	213.98	163.03	100.24	44.00	12.45	2239.26
44.95	243.38	245.13	242.82	244.88	241.53	230.71	210.89	162.58	99.30	46.73	11.73	2239.26
45.05	244.75	244.16	244.55	241.93	245.27	230.21	213.19	162.43	96.58	43.35	10.85	2239.26
45.15	245.69	244.48	243.61	243.29	241.77	234.40	208.77	160.92	96.22	46.97	11.57	2239.26
45.25	243.43	243.11	244.77	244.94	240.78	229.24	210.58	163.03	96.67	45.72	10.85	2239.26
45.35	239.70	245.28	244.51	244.05	243.65	233.01	208.82	155.64	94.98	45.55	10.13	2239.26
45.45	242.63	243.10	243.02	243.40	239.55	230.26	213.52	159.71	93.23	47.33	12.90	2239.26
45.55	245.87	247.21	244.45	245.67	240.98	234.15	206.60	177.98	97.34	47.33	11.18	2239.26
45.65	243.14	245.54	245.91	241.63	240.90	231.05	211.09	162.73	96.16	47.92	11.85	2239.26
45.75	244.99	244.64	242.10	244.81	239.18	231.42	210.51	160.47	95.19	44.60	11.18	2239.26
45.85	243.11	241.56	244.25	244.90	242.82	231.35	206.85	159.56	97.79	42.82	10.79	2239.26
45.95	244.33	243.15	241.07	246.62	237.46	233.01	207.26	153.83	94.23	44.42	11.24	2239.26
46.05	245.20	244.43	245.24	242.32	242.37	234.51	211.78	165.15	93.65	47.09	11.79	2239.26
46.15	242.04	243.08	243.95	244.81	241.38	234.55	210.68	160.32	94.05	45.90	10.52	2239.26
46.25	243.52	244.77	243.38	243.26	242.19	231.30	208.21	150.51	95.83	40.62	12.68	2239.26
46.35	242.65	245.08	243.02	245.27	241.31	232.74	212.17	167.71	96.46	44.72	11.79	2239.26
46.45	241.38	244.88	242.13	243.60	241.23	228.73	212.29	156.09	96.31	42.05	10.35	2239.26
46.55	244.77	244.93	244.75	242.73	240.78	229.30	210.58	163.64	97.97	45.37	12.45	2239.26
46.65	244.70	244.53	242.16	242.04	242.51	232.33	211.91	159.71	96.52	45.19	11.96	2239.26
46.75	245.45	241.22	247.89	239.41	240.49	232.23	209.64	159.26	95.71	46.02	14.17	2239.26
46.85	243.91	243.62	245.81	242.14	244.17	230.39	214.41	161.83	97.34	46.97	10.79	2239.26

Midpt.	11000	2700	910	240	84	26	8.8	2.7	0.92	0.31	Volume	
46.95	244.12	247.14	244.45	243.80	241.33	231.48	207.29	155.94	97.13	47.98	11.62	2239.26
47.05	243.80	244.08	246.14	244.31	242.46	228.28	211.88	167.11	94.20	43.59	12.01	2239.26
47.15	245.68	244.44	242.67	241.40	243.18	231.12	208.15	168.02	99.12	45.96	12.51	2239.26
47.25	244.75	243.74	244.04	245.61	243.21	233.42	218.04	164.70	95.77	45.49	11.79	2239.26
47.35	243.32	243.58	248.72	244.55	237.04	234.83	212.50	153.52	94.47	43.00	11.73	2239.26
47.45	245.95	245.49	246.14	243.83	237.16	232.37	206.09	165.60	97.46	44.48	11.62	2239.26
47.55	243.18	241.83	246.94	243.36	241.11	230.15	208.10	160.62	96.92	46.14	10.35	2239.26
47.65	244.76	243.85	243.19	242.68	242.76	231.73	213.39	159.11	94.17	44.12	12.23	2239.26
47.75	245.13	245.54	242.61	242.04	241.16	236.56	211.99	161.37	93.35	47.62	10.46	2239.26
47.85	244.96	242.70	246.51	247.26	239.86	233.05	210.33	158.81	94.68	42.34	13.06	2239.26
47.95	242.95	247.47	245.38	241.87	240.95	234.28	211.81	155.79	93.62	45.96	12.23	2239.26
48.05	242.05	242.17	241.35	245.50	236.65	230.81	209.05	158.05	95.86	43.94	11.79	2239.26
48.15	243.58	241.92	243.86	243.84	241.90	229.74	205.75	167.71	92.93	45.55	10.46	2239.26
48.25	241.65	244.97	239.30	245.11	242.81	233.26	213.49	157.90	99.42	46.14	12.12	2239.26
48.35	245.44	244.22	242.87	242.13	241.06	234.31	210.89	157.75	98.45	41.39	10.63	2239.26
48.45	244.22	246.34	244.68	245.00	237.91	232.35	211.09	158.20	97.61	46.50	11.90	2239.26
48.55	245.01	244.12	245.41	244.30	239.00	236.12	209.66	172.70	95.35	44.12	12.29	2239.26
48.65	242.97	243.26	242.22	241.48	244.27	234.55	210.48	160.92	96.98	42.88	11.73	2239.26
48.75	241.56	245.65	244.86	241.76	236.65	229.32	213.67	164.39	96.22	41.93	12.56	2239.26
48.85	242.38	241.37	246.19	241.49	242.52	228.98	214.64	160.17	98.00	44.24	12.40	2239.26
48.95	243.75	242.66	242.45	243.19	240.64	231.33	207.67	174.21	98.52	45.25	12.40	2239.26
49.05	243.26	244.24	246.64	243.20	240.93	225.51	213.09	158.05	95.71	41.81	10.18	2239.26
49.15	240.81	245.86	242.11	241.72	237.52	233.10	211.30	158.96	92.66	43.41	11.18	2239.26
49.25	240.96	241.37	244.19	241.84	238.98	232.10	211.02	165.75	96.13	47.15	11.79	2239.26
49.35	241.24	242.96	243.20	244.53	240.34	234.08	210.89	158.36	95.07	43.83	11.35	2239.26
49.45	244.93	243.58	246.23	243.96	241.72	233.10	213.16	163.19	98.21	46.14	13.45	2239.26
49.55	244.17	246.96	246.84	245.18	238.82	234.33	210.20	150.05	94.29	42.94	11.85	2239.26
49.65	241.44	243.83	245.82	241.77	239.50	231.28	210.97	154.58	94.44	42.34	11.85	2239.26
49.75	243.45	245.47	246.18	241.30	243.18	229.94	203.22	164.09	96.04	42.82	11.68	2239.26
49.85	244.24	240.75	242.45	244.25	238.66	229.92	213.24	165.00	96.07	44.95	11.73	2239.26
49.95	246.75	242.83	240.89	243.60	243.31	232.44	209.53	158.66	99.57	44.30	12.18	2239.26
50.05	244.25	246.68	241.52	243.38	243.01	233.31	213.42	161.83	96.04	44.06	11.57	2239.26
50.15	241.41	242.82	244.19	244.64	238.92	229.62	214.36	160.47	93.93	44.83	12.34	2239.26
50.25	242.46	243.94	245.01	242.25	242.56	233.10	209.51	161.37	93.08	44.18	12.18	2239.26
50.35	245.12	243.52	244.12	243.81	243.18	229.10	212.06	156.24	93.50	44.18	11.62	2239.26
50.45	242.77	246.57	245.21	243.49	239.59	236.17	214.26	161.68	96.16	44.95	11.13	2239.26
50.55	241.55	244.37	245.11	243.92	240.29	229.28	211.12	161.68	97.13	43.65	12.56	2239.26
50.65	243.26	245.70	243.25	244.50	241.77	233.67	207.41	161.22	98.94	42.82	11.73	2239.26
50.75	242.14	242.23	245.04	241.96	240.56	233.64	209.23	149.75	96.67	45.55	12.68	2239.26
50.85	242.39	242.81	245.88	244.35	238.04	231.23	205.98	164.54	99.93	46.20	9.96	2239.26
50.95	245.52	243.95	244.39	243.75	238.65	231.58	211.53	158.51	96.55	45.31	11.51	2239.26
51.05	241.97	242.73	242.55	239.32	241.38	232.60	206.52	156.85	97.70	42.88	12.18	2239.26
51.15	246.06	242.82	242.24	241.65	241.67	233.99	211.12	165.45	96.95	46.61	11.68	2239.26
51.25	244.12	244.72	246.01	241.51	237.83	235.08	206.16	158.81	97.49	44.18	11.68	2239.26
51.35	243.79	242.95	245.79	242.21	234.45	230.51	207.75	156.39	96.58	45.13	13.23	2239.26

Midpt.	11000	2700	910	240	84	26	8.8	2.7	0.92	0.31	Volume	
51.45	245.86	239.85	243.44	240.93	244.32	233.24	209.30	152.62	94.71	43.77	12.34	2239.26
51.55	245.80	241.78	244.42	241.83	240.48	231.85	211.37	160.92	96.28	42.70	11.57	2239.26
51.65	247.31	243.38	242.16	244.37	243.71	230.46	209.76	161.37	97.73	42.28	11.96	2239.26
51.75	240.19	242.81	242.55	241.93	241.63	230.96	211.40	157.00	96.88	46.38	11.57	2239.26
51.85	240.42	239.43	244.10	244.74	239.22	231.92	209.13	158.05	94.29	44.12	13.34	2239.26
51.95	243.06	241.65	244.45	243.24	237.93	229.69	209.02	154.73	98.97	41.51	9.63	2239.26
52.05	243.86	241.79	244.88	243.51	240.44	234.96	213.75	158.81	93.11	40.74	11.62	2239.26
52.15	240.77	242.23	245.54	241.52	242.22	232.87	210.89	168.77	95.10	46.85	12.23	2239.26
52.25	241.71	243.75	242.51	243.08	245.22	229.32	206.75	161.07	94.98	44.18	12.23	2239.26
52.35	244.51	244.30	241.99	242.74	238.25	232.71	209.51	165.15	94.59	43.35	11.96	2239.26
52.45	242.30	239.37	245.10	244.30	239.92	235.81	208.44	168.17	95.53	44.00	12.95	2239.26
52.55	241.28	243.13	244.09	244.49	238.56	229.21	209.69	157.00	95.50	43.77	12.68	2239.26
52.65	244.32	244.52	243.72	245.69	240.11	228.82	208.44	160.92	94.23	47.21	11.96	2239.26
52.75	241.12	239.80	246.78	242.13	236.57	234.62	210.99	162.58	96.25	47.50	13.51	2239.26
52.85	241.83	243.25	243.03	243.48	245.99	232.51	213.29	169.68	99.33	44.30	12.56	2239.26
52.95	241.83	242.11	244.16	242.01	245.22	235.69	210.33	157.15	99.69	44.60	12.62	2239.26
53.05	244.47	245.07	245.76	243.44	238.28	231.83	210.66	159.56	100.27	47.09	12.62	2239.26
53.15	243.57	246.23	245.24	244.49	243.13	234.39	212.55	147.03	96.22	41.87	11.57	2239.26
53.25	243.45	243.89	242.03	244.31	241.77	234.10	211.09	148.39	97.55	41.39	12.01	2239.26
53.35	241.63	243.87	242.19	243.43	242.25	235.48	213.49	156.09	94.02	43.77	11.68	2239.26
53.45	244.61	243.40	245.77	243.07	243.14	232.55	210.40	159.11	95.47	44.54	14.11	2239.26
53.55	240.95	243.15	243.95	243.77	238.83	234.35	204.94	166.05	95.38	44.00	12.79	2239.26
53.65	242.35	243.41	242.93	241.72	242.87	232.42	208.79	159.56	94.26	43.47	12.12	2239.26
53.75	246.32	243.64	242.66	244.40	237.31	233.01	212.11	157.75	95.07	46.26	11.96	2239.26
53.85	241.08	244.43	244.60	245.40	241.95	230.32	209.53	160.32	96.58	45.07	12.73	2239.26
53.95	242.79	244.22	243.75	244.18	236.32	231.35	216.43	158.66	92.36	47.15	12.73	2239.26
54.05	244.68	242.32	243.81	241.36	237.51	232.94	210.91	166.05	96.79	41.39	13.56	2239.26
54.15	242.17	242.95	242.97	240.88	241.95	231.85	208.13	162.88	95.92	42.70	12.45	2239.26
54.25	243.71	245.75	246.31	241.61	238.53	231.56	210.38	165.60	97.43	46.97	11.62	2239.26
54.35	245.71	244.77	240.03	242.67	240.71	230.73	211.63	161.68	96.43	41.81	11.29	2239.26
54.45	241.67	241.91	243.18	243.60	239.97	230.03	209.64	167.11	96.31	45.55	15.28	2239.26
54.55	241.15	243.76	242.85	245.40	239.74	233.73	207.41	156.54	98.06	47.15	11.62	2239.26
54.65	242.68	242.45	241.33	242.87	241.53	228.10	209.89	159.86	97.22	44.42	12.84	2239.26
54.75	245.56	239.35	241.29	244.12	239.29	231.58	202.10	150.51	94.29	44.30	11.24	2239.26
54.85	244.74	243.71	243.79	240.81	238.72	229.23	212.55	151.41	95.25	44.72	12.40	2239.26
54.95	245.07	239.05	242.12	242.65	239.24	233.06	210.40	172.24	93.71	43.23	11.79	2239.26
55.05	240.89	240.67	242.82	243.71	245.86	232.87	209.05	163.79	97.61	42.88	14.17	2239.26
55.15	243.26	244.13	244.95	242.92	244.57	231.78	207.54	156.69	97.04	45.66	11.46	2239.26
55.25	242.78	243.63	243.45	243.17	241.63	234.21	212.83	149.00	94.05	46.97	12.34	2239.26
55.35	244.36	242.62	243.32	243.14	243.58	229.46	211.76	153.98	93.81	45.31	13.01	2239.26
55.45	245.45	242.73	245.79	242.54	239.35	231.98	212.32	151.86	97.79	45.25	11.35	2239.26
55.55	242.88	241.59	244.98	245.10	237.32	234.26	206.88	159.86	99.51	42.94	11.90	2239.26
55.65	243.17	243.47	241.52	243.91	243.65	228.42	212.68	160.32	94.08	43.59	10.90	2239.26
55.75	242.39	244.20	243.35	242.26	241.40	230.69	208.08	163.79	91.75	46.26	12.68	2239.26
55.85	243.71	245.29	244.56	245.03	240.36	234.08	211.53	167.11	95.13	44.66	13.17	2239.26

Midpt.	11000	2700	910	240	84	26	8.8	2.7	0.92	0.31	Volume	
55.95	245.17	245.09	244.61	242.95	237.88	234.08	213.60	156.09	96.88	44.60	12.84	2239.26
56.05	240.89	241.58	243.26	244.34	240.29	226.80	213.55	166.05	95.68	45.78	13.34	2239.26
56.15	242.06	244.76	242.25	243.51	236.62	231.15	210.79	165.00	96.19	44.72	12.07	2239.26
56.25	243.95	244.68	242.77	242.32	242.69	230.62	209.89	157.15	98.09	44.48	11.68	2239.26
56.35	244.91	245.41	242.85	244.57	240.74	233.44	204.96	158.05	98.09	42.40	12.07	2239.26
56.45	241.55	240.43	243.01	240.30	244.84	232.85	207.34	162.43	96.95	43.41	14.11	2239.26
56.55	243.10	243.62	242.84	244.00	234.96	232.96	212.55	162.88	95.50	39.79	13.01	2239.26
56.65	240.85	244.51	241.83	245.82	239.08	233.10	208.03	153.68	96.22	44.72	12.45	2239.26
56.75	243.25	241.36	243.88	242.60	236.32	229.35	210.02	162.88	94.08	43.71	12.84	2239.26
56.85	240.95	242.37	242.38	239.68	239.37	230.49	210.25	159.26	95.56	44.48	12.34	2239.26
56.95	241.22	242.28	244.07	242.04	238.60	233.21	210.84	158.51	96.92	43.47	10.90	2239.26
57.05	241.76	244.01	243.99	242.00	243.65	231.83	212.75	148.09	95.19	42.70	12.18	2239.26
57.15	240.73	242.79	242.61	242.71	239.99	233.01	209.82	162.58	92.66	45.13	12.56	2239.26
57.25	241.28	244.61	244.88	245.32	238.68	232.96	212.57	160.47	95.47	45.01	13.06	2239.26
57.35	240.45	243.18	244.17	242.72	243.13	232.23	210.61	156.69	97.40	45.25	12.56	2239.26
57.45	240.44	242.34	244.48	242.75	241.30	232.96	210.33	157.75	96.31	42.76	12.18	2239.26
57.55	246.48	246.01	245.00	241.19	238.06	234.19	205.01	157.45	94.80	42.40	11.90	2239.26
57.65	241.93	245.51	243.84	244.23	240.76	227.71	205.68	158.51	96.16	47.38	13.12	2239.26
57.75	240.94	243.82	241.83	239.25	237.74	233.94	207.93	163.64	94.56	42.76	13.62	2239.26
57.85	240.90	246.98	243.39	242.07	243.46	230.56	211.09	158.20	96.82	44.83	13.17	2239.26
57.95	242.63	243.67	243.58	241.48	239.62	231.08	209.94	159.56	96.55	44.78	12.68	2239.26
58.05	242.69	240.51	242.88	243.25	241.77	236.19	210.17	161.98	95.89	42.58	11.79	2239.26
58.15	241.99	242.50	246.66	241.44	238.11	230.58	209.66	159.41	98.61	46.91	12.07	2239.26
58.25	243.85	243.57	246.23	242.43	241.21	231.12	208.36	161.22	98.18	40.80	11.51	2239.26
58.35	245.81	241.29	243.51	240.91	239.35	231.96	210.12	164.39	95.98	44.78	13.67	2239.26
58.45	239.09	244.63	243.04	244.51	239.67	231.35	207.70	169.07	97.22	46.26	10.57	2239.26
58.55	241.56	242.39	242.10	239.92	241.52	232.89	207.31	157.60	96.16	41.87	13.12	2239.26
58.65	241.65	243.74	242.58	243.94	237.09	232.83	204.68	162.73	97.49	43.35	12.95	2239.26
58.75	243.15	242.55	245.66	242.95	242.59	229.99	207.70	163.94	96.07	43.17	11.85	2239.26
58.85	243.65	243.47	244.69	244.53	243.26	229.92	209.79	147.49	93.96	42.70	11.40	2239.26
58.95	242.23	244.50	241.45	241.53	241.16	231.46	211.40	158.96	94.44	44.12	13.01	2239.26
59.05	243.74	241.01	242.13	245.53	239.70	228.94	209.07	168.32	95.92	43.41	10.35	2239.26
59.15	243.37	243.13	246.46	239.68	236.89	233.90	211.83	172.39	95.28	44.36	12.95	2239.26
59.25	247.64	243.22	243.41	244.53	239.47	232.40	211.53	153.68	98.45	45.31	12.62	2239.26
59.35	240.20	242.05	244.55	241.25	238.72	230.23	209.92	163.19	95.98	43.65	12.34	2239.26
59.45	241.23	238.89	242.81	241.12	243.76	234.12	208.82	165.30	96.01	45.96	13.40	2239.26
59.55	241.22	242.64	246.06	241.74	239.49	229.92	209.05	155.49	95.31	46.38	13.06	2239.26
59.65	241.83	243.13	244.30	244.05	243.78	235.78	208.72	153.68	95.38	44.48	13.28	2239.26
59.75	244.36	244.99	243.35	244.55	240.86	230.53	210.61	151.41	92.93	44.48	12.34	2239.26
59.85	245.49	238.86	244.30	243.16	242.91	226.51	211.09	149.90	96.82	41.87	13.78	2239.26
59.95	244.14	241.50	242.18	240.58	240.91	229.32	211.48	146.73	95.80	44.48	13.28	2239.26
60.05	243.26	239.20	241.45	240.59	238.58	231.65	205.98	174.51	95.71	43.23	11.90	2239.26
60.15	244.16	246.38	244.07	244.40	241.83	237.37	210.97	160.17	96.46	43.77	13.84	2239.26
60.25	240.46	244.70	243.52	242.82	243.81	226.85	208.10	148.09	96.92	46.08	12.68	2239.26
60.35	244.67	244.10	244.60	245.00	241.57	237.58	206.62	157.00	96.07	46.91	13.78	2239.26

Midpt.	11000	2700	910	240	84	26	8.8	2.7	0.92	0.31	Volume	
60.45	246.28	242.73	241.23	239.82	238.73	233.74	210.61	162.43	96.25	43.71	12.90	2239.26
60.55	240.20	243.22	242.62	238.36	243.28	225.48	208.84	157.15	98.97	42.82	11.01	2239.26
60.65	240.09	241.64	241.30	244.67	237.41	232.06	208.61	158.05	96.67	47.15	12.23	2239.26
60.75	245.05	242.95	240.97	243.88	238.16	226.14	211.99	158.96	95.44	45.96	13.12	2239.26
60.85	241.18	242.73	242.15	242.30	239.24	228.03	209.74	156.69	96.16	42.34	13.12	2239.26
60.95	245.13	245.60	243.60	243.87	237.59	227.58	212.11	159.26	96.82	46.91	13.12	2239.26
61.05	244.62	243.03	244.61	247.77	241.31	231.17	209.46	151.56	94.02	44.60	12.73	2239.26
61.15	244.17	242.41	242.53	241.23	239.12	233.15	214.72	164.09	93.68	44.78	12.95	2239.26
61.25	244.65	242.51	243.78	244.59	238.35	225.87	209.69	164.70	94.23	44.12	13.56	2239.26
61.35	241.33	244.19	244.25	240.81	243.09	229.55	213.70	163.49	94.20	44.12	11.57	2239.26
61.45	244.87	243.90	243.68	244.54	239.30	229.14	208.82	166.05	93.93	45.31	13.12	2239.26
61.55	242.49	242.29	243.86	242.61	238.60	231.17	210.76	162.58	95.65	42.94	12.07	2239.26
61.65	245.60	245.18	241.42	243.32	239.74	231.14	209.79	160.17	94.20	42.17	13.73	2239.26
61.75	242.67	244.03	242.29	238.90	241.10	230.58	208.74	162.88	99.03	46.20	12.62	2239.26
61.85	243.44	242.95	244.17	242.30	238.72	230.39	209.64	165.45	94.86	42.11	12.56	2239.26
61.95	241.19	242.08	245.27	239.04	238.80	233.94	212.83	158.96	91.00	42.82	12.34	2239.26
62.05	242.41	241.39	240.32	242.92	236.60	233.87	210.30	168.02	93.17	45.25	14.39	2239.26
62.15	244.77	241.73	240.89	243.21	241.63	228.26	208.87	158.36	99.90	40.33	11.62	2239.26
62.25	241.28	241.62	241.72	241.76	240.81	230.32	210.43	155.79	94.47	43.17	11.01	2239.26
62.35	243.71	245.26	243.19	241.64	242.57	227.85	209.43	158.96	97.34	46.97	13.95	2239.26
62.45	241.15	247.29	243.90	243.25	242.66	236.37	212.11	165.90	95.62	44.12	12.84	2239.26
62.55	243.99	242.22	244.15	240.70	244.30	238.46	210.38	163.64	97.13	44.60	14.34	2239.26
62.65	244.94	241.69	240.53	242.66	238.33	232.10	209.02	157.90	94.74	43.94	13.51	2239.26
62.75	243.92	244.03	243.80	243.16	243.04	229.33	209.36	151.41	95.19	44.95	13.06	2239.26
62.85	243.56	244.43	239.78	244.87	237.34	229.90	209.02	153.22	97.22	43.35	13.56	2239.26
62.95	241.51	243.73	246.37	242.90	243.36	230.69	208.69	153.68	94.71	43.77	12.29	2239.26
63.05	244.26	241.96	243.45	240.17	236.08	232.19	215.15	157.90	96.55	44.48	11.90	2239.26
63.15	243.50	242.28	244.84	242.18	243.56	228.67	213.78	157.60	93.50	43.89	12.51	2239.26
63.25	245.57	238.28	241.42	242.23	238.13	232.08	212.17	162.73	96.61	43.77	12.62	2239.26
63.35	241.00	243.05	242.32	241.24	239.37	231.89	211.76	158.66	94.74	45.72	12.51	2239.26
63.45	242.84	241.55	241.66	243.05	239.55	235.62	210.56	168.92	100.45	44.06	13.51	2239.26
63.55	242.86	245.08	243.58	239.47	238.97	231.60	207.85	162.58	96.88	45.25	12.12	2239.26
63.65	242.50	240.87	244.05	241.62	235.63	230.78	210.99	163.19	97.52	44.83	10.46	2239.26
63.75	244.45	241.01	245.24	240.12	237.52	234.19	208.97	159.86	93.38	42.46	12.12	2239.26
63.85	242.51	242.30	247.81	239.41	240.43	234.44	212.45	161.98	97.88	44.48	12.12	2239.26
63.95	241.55	241.38	241.40	242.25	239.50	227.26	210.45	164.09	95.74	44.89	13.40	2239.26
64.05	244.12	244.57	244.61	243.32	239.62	232.12	209.00	164.70	97.49	44.48	12.29	2239.26
64.15	242.65	244.94	242.76	243.42	241.35	230.10	211.81	163.34	92.30	42.52	12.95	2239.26
64.25	245.49	245.79	243.62	242.04	236.40	230.87	211.81	158.05	97.34	43.47	12.79	2239.26
64.35	241.56	243.91	242.68	245.88	243.75	232.21	209.59	154.88	95.95	42.11	11.62	2239.26
64.45	240.85	244.52	243.26	240.60	241.99	231.69	208.51	168.62	94.77	47.09	13.17	2239.26
64.55	240.83	246.78	243.54	242.98	241.77	228.90	209.43	156.85	96.85	45.78	12.73	2239.26
64.65	245.02	242.53	245.70	242.60	239.10	228.74	213.47	149.60	95.50	46.67	13.67	2239.26
64.75	243.00	244.30	244.23	241.53	240.09	231.46	204.96	168.47	92.99	41.28	12.45	2239.26
64.85	244.48	243.33	242.20	240.73	239.96	233.10	208.74	157.30	94.56	42.52	14.50	2239.26

Midpt.	11000	2700	910	240	84	26	8.8	2.7	0.92	0.31	Volume	
64.95	241.67	242.92	243.45	240.80	240.14	231.78	209.20	158.20	98.00	41.63	13.40	2239.26
65.05	242.30	242.32	241.54	242.29	239.39	232.48	202.30	162.28	93.81	45.07	14.06	2239.26
65.15	242.81	245.00	243.43	242.68	241.65	230.53	207.93	161.07	94.83	44.18	13.06	2239.26
65.25	241.08	240.18	242.48	243.65	242.09	232.62	213.29	159.11	98.36	46.32	13.28	2239.26
65.35	239.58	242.96	243.76	243.94	241.87	229.99	216.15	155.79	92.84	46.38	13.23	2239.26
65.45	242.31	243.66	241.01	243.84	237.91	234.03	211.88	165.90	96.16	46.61	14.56	2239.26
65.55	243.90	244.44	242.98	242.96	237.89	237.03	209.10	157.30	99.36	43.41	11.90	2239.26
65.65	242.64	240.75	243.95	241.97	236.74	236.30	209.74	141.60	97.37	45.31	12.62	2239.26
65.75	242.90	241.81	244.51	240.98	236.97	233.08	206.93	166.96	93.96	45.37	12.07	2239.26
65.85	242.90	240.01	241.82	242.59	236.57	236.92	206.75	169.68	95.35	44.89	12.95	2239.26
65.95	243.63	242.78	242.52	241.21	241.85	229.39	210.20	156.69	92.69	45.84	12.51	2239.26
66.05	245.01	243.27	242.42	243.38	236.70	231.24	213.55	160.47	96.13	43.59	14.11	2239.26
66.15	245.19	240.58	243.13	240.85	237.93	228.03	205.06	155.34	94.20	42.70	12.84	2239.26
66.25	239.95	243.72	241.64	243.98	240.04	232.37	209.41	158.51	94.92	41.57	12.95	2239.26
66.35	245.35	243.58	239.65	242.32	236.80	232.15	208.05	153.68	95.04	44.06	12.45	2239.26
66.45	244.28	241.48	240.88	240.45	242.51	229.85	212.45	163.49	96.61	42.58	13.56	2239.26
66.55	246.42	243.94	243.52	246.21	240.01	230.69	209.02	156.09	97.22	42.76	13.67	2239.26
66.65	242.73	242.13	246.27	240.39	235.14	231.44	210.97	160.77	94.50	45.31	11.90	2239.26
66.75	243.78	239.64	241.91	242.71	242.96	234.99	208.41	161.22	99.03	43.17	13.45	2239.26
66.85	242.65	243.26	243.60	243.06	236.12	231.80	212.78	165.60	96.28	44.00	10.63	2239.26
66.95	242.24	245.62	243.93	243.14	236.77	230.96	208.90	161.68	93.26	46.85	12.07	2239.26
67.05	243.42	243.91	243.89	241.57	239.91	229.40	211.12	162.28	96.28	48.04	14.11	2239.26
67.15	245.30	242.55	241.09	242.35	238.40	230.64	207.16	150.35	97.19	46.08	14.00	2239.26
67.25	246.87	241.11	243.99	240.04	239.32	228.33	207.36	155.49	96.85	43.00	12.68	2239.26
67.35	244.44	241.15	242.27	244.13	241.08	229.87	208.13	153.37	98.79	43.65	12.07	2239.26
67.45	242.19	246.59	243.64	241.21	236.15	232.06	213.52	153.52	97.70	44.48	13.01	2239.26
67.55	240.75	241.95	242.68	241.95	241.15	227.67	210.66	158.20	96.28	43.89	14.06	2239.26
67.65	239.48	240.73	242.03	242.86	240.28	230.55	212.32	159.71	98.09	42.70	12.62	2239.26
67.75	245.44	243.87	243.92	240.48	238.31	229.53	210.17	150.20	97.13	44.66	13.45	2239.26
67.85	241.75	243.94	243.08	240.79	238.60	231.17	212.57	155.18	97.55	45.01	13.17	2239.26
67.95	242.41	241.14	241.58	241.77	239.08	231.83	211.07	161.98	98.61	43.77	14.45	2239.26
68.05	244.98	242.33	244.93	241.12	238.35	227.96	212.29	153.37	94.86	41.22	13.84	2239.26
68.15	244.72	240.21	246.81	244.44	233.18	233.51	213.29	158.05	100.84	43.00	12.01	2239.26
68.25	242.64	242.91	240.47	241.47	242.56	229.39	212.47	158.96	97.76	44.66	12.51	2239.26
68.35	241.54	239.00	242.09	239.29	240.33	228.23	211.53	155.34	93.99	46.55	13.95	2239.26
68.45	242.73	241.20	247.09	242.62	236.58	231.89	207.70	168.02	96.07	44.00	14.34	2239.26
68.55	246.21	243.67	239.95	244.32	241.82	231.98	211.53	152.17	97.04	43.94	14.17	2239.26
68.65	241.82	242.45	240.66	243.36	241.94	229.35	208.00	157.00	96.52	41.81	12.73	2239.26
68.75	241.27	242.21	246.51	242.81	238.95	231.35	207.26	167.71	98.27	43.35	13.17	2239.26
68.85	241.26	242.41	245.25	241.79	239.82	232.14	205.01	160.77	96.22	47.68	11.73	2239.26
68.95	242.69	241.74	241.03	241.39	241.10	232.83	213.65	160.32	96.04	42.64	11.96	2239.26
69.05	241.57	242.16	242.55	240.91	241.35	230.24	207.62	157.30	95.31	44.60	14.50	2239.26
69.15	241.71	242.08	243.60	243.48	242.30	234.06	210.28	164.24	98.18	46.97	13.28	2239.26
69.25	243.53	241.81	243.91	240.85	244.55	232.83	206.80	165.30	94.71	46.08	12.18	2239.26
69.35	245.36	245.25	243.39	243.44	240.46	232.15	213.55	160.62	94.71	46.32	13.12	2239.26

Midpt.	11000	2700	910	240	84	26	8.8	2.7	0.92	0.31	Volume	
69.45	242.83	242.77	242.22	242.97	233.95	228.19	212.86	166.20	96.76	43.23	12.79	2239.26
69.55	243.18	243.26	243.97	244.62	239.55	229.94	208.95	156.24	96.58	42.70	13.12	2239.26
69.65	242.78	242.58	241.20	245.75	237.41	230.58	204.99	160.47	92.78	43.71	13.23	2239.26
69.75	244.00	244.09	243.20	243.27	239.94	227.60	210.30	153.68	93.35	43.71	14.11	2239.26
69.85	242.24	242.08	243.71	242.06	241.57	233.49	213.62	157.00	96.22	45.25	12.51	2239.26
69.95	242.68	239.69	243.63	245.24	234.89	228.32	210.66	166.36	96.55	43.77	12.79	2239.26
70.05	242.46	240.52	241.35	241.35	237.51	229.74	208.08	168.47	95.25	44.78	12.56	2239.26
70.15	241.46	244.48	243.86	241.60	241.10	228.07	208.41	159.26	94.86	43.41	10.90	2239.26
70.25	241.98	244.25	242.85	241.35	240.41	231.10	210.48	157.45	95.68	45.19	12.62	2239.26
70.35	239.48	240.17	240.89	243.02	238.36	230.08	207.36	149.00	97.55	47.68	12.23	2239.26
70.45	243.28	243.43	241.35	243.58	238.40	230.78	211.99	165.60	94.86	42.52	13.45	2239.26
70.55	243.37	244.54	243.42	242.02	236.79	226.64	213.60	158.05	94.65	45.01	12.95	2239.26
70.65	241.22	242.67	244.79	243.01	242.69	231.62	211.35	163.03	97.07	43.94	12.51	2239.26
70.75	243.14	244.34	241.33	241.84	240.66	230.73	206.57	171.34	97.43	46.20	12.62	2239.26
70.85	241.14	242.26	240.89	244.19	242.15	230.07	210.15	162.13	96.85	47.56	12.18	2239.26
70.95	239.45	244.31	244.38	242.74	238.92	231.40	209.51	164.39	96.34	43.89	13.45	2239.26
71.05	241.06	242.73	243.45	239.86	239.32	228.49	209.13	154.13	97.61	42.58	12.68	2239.26
71.15	240.46	241.62	244.41	241.34	240.49	233.01	213.57	157.30	96.49	44.60	13.23	2239.26
71.25	242.67	240.56	242.78	243.24	237.37	231.49	211.27	155.94	95.83	43.47	11.57	2239.26
71.35	241.92	241.87	242.77	240.98	237.51	229.94	208.92	158.81	97.52	46.38	12.90	2239.26
71.45	242.35	245.68	242.44	241.26	242.69	231.76	208.82	152.77	94.92	42.70	13.34	2239.26
71.55	241.65	242.52	244.57	240.91	239.02	233.33	211.63	157.60	94.02	44.24	13.95	2239.26
71.65	243.37	243.80	244.84	239.55	240.88	227.23	209.02	154.58	96.95	45.01	12.73	2239.26
71.75	242.41	241.66	241.64	242.83	233.99	228.23	211.83	163.19	96.01	43.83	13.89	2239.26
71.85	241.97	245.19	241.02	242.65	239.35	232.46	210.40	157.75	97.73	45.96	11.85	2239.26
71.95	243.49	240.18	242.15	242.82	241.18	227.64	209.56	160.17	97.49	44.54	11.07	2239.26
72.05	240.37	241.24	243.88	241.46	238.88	234.49	210.38	153.07	94.50	44.24	11.73	2239.26
72.15	245.28	241.94	243.85	239.11	241.58	230.55	207.67	162.28	99.57	46.32	14.17	2239.26
72.25	243.50	241.31	240.59	242.87	239.37	233.65	206.57	161.07	96.95	44.95	12.40	2239.26
72.35	242.18	241.24	241.25	246.43	238.60	230.24	210.22	167.71	96.52	44.48	13.28	2239.26
72.45	241.64	241.06	243.43	242.78	239.67	230.98	210.89	154.58	98.12	46.26	11.73	2239.26
72.55	239.19	243.15	241.16	242.87	238.40	228.03	210.66	156.09	94.71	43.83	12.62	2239.26
72.65	244.37	239.95	241.91	240.15	237.69	233.58	207.44	166.05	97.37	44.42	13.28	2239.26
72.75	242.57	244.40	243.15	243.70	243.53	230.94	215.00	156.39	97.31	45.07	13.73	2239.26
72.85	239.72	242.65	244.04	241.76	237.71	230.71	208.28	155.79	96.52	42.70	12.34	2239.26
72.95	244.95	243.85	241.74	242.23	236.60	234.99	214.95	161.37	97.25	43.29	11.29	2239.26
73.05	242.88	242.46	240.45	240.93	238.73	229.94	207.54	154.88	95.44	43.47	13.45	2239.26
73.15	244.87	243.57	242.94	238.53	235.13	230.90	211.45	163.79	97.46	44.48	12.34	2239.26
73.25	240.06	239.46	243.29	244.89	235.38	231.87	206.55	164.85	96.61	43.11	12.51	2239.26
73.35	242.07	241.46	243.35	242.27	240.83	229.98	210.43	149.30	98.73	47.21	12.62	2239.26
73.45	241.98	242.97	241.76	241.78	240.43	232.30	206.83	157.90	94.47	46.67	12.62	2239.26
73.55	240.69	246.51	241.85	241.72	237.84	231.33	211.68	159.71	95.35	46.55	13.45	2239.26
73.65	244.99	243.07	241.32	241.74	241.01	228.55	212.11	158.51	94.65	43.06	13.01	2239.26
73.75	243.19	241.87	242.99	242.37	235.86	230.71	213.42	169.38	96.16	42.40	11.01	2239.26
73.85	242.82	244.36	242.12	242.57	241.99	232.56	210.22	159.26	96.01	45.72	12.56	2239.26

Midpt.	11000	2700	910	240	84	26	8.8	2.7	0.92	0.31	Volume	
73.95	241.70	244.79	239.21	243.34	237.56	231.19	206.16	154.58	96.58	42.76	12.79	2239.26
74.05	239.69	241.92	243.92	238.20	239.97	231.19	208.84	150.05	97.25	46.73	12.45	2239.26
74.15	244.46	241.38	243.17	240.83	243.13	231.46	209.33	162.58	97.10	45.37	11.46	2239.26
74.25	238.35	242.68	242.46	240.71	240.04	229.26	210.15	158.81	95.53	45.55	12.45	2239.26
74.35	239.81	243.04	243.04	239.50	241.77	229.37	215.05	169.22	93.05	43.00	12.01	2239.26
74.45	240.66	242.44	241.75	240.64	236.74	229.33	206.85	157.90	99.39	44.89	12.51	2239.26
74.55	241.92	242.57	243.04	243.17	240.71	229.90	210.66	154.43	95.89	43.89	12.90	2239.26
74.65	242.62	243.51	244.38	240.42	243.21	230.80	210.51	154.13	96.01	43.29	12.23	2239.26
74.75	241.76	241.82	245.39	242.77	239.34	231.42	210.91	157.75	97.16	42.88	11.51	2239.26
74.85	241.47	241.47	242.02	242.83	233.15	228.98	211.35	169.38	94.86	42.64	11.73	2239.26
74.95	239.88	243.94	243.26	241.82	237.02	231.73	211.76	159.26	97.04	42.40	11.68	2239.26
75.05	247.08	240.46	245.32	241.37	240.78	228.89	211.20	161.22	97.67	44.06	12.18	2239.26
75.15	239.51	243.78	241.05	241.94	237.74	228.92	213.11	156.54	95.31	49.46	11.85	2239.26
75.25	244.95	243.40	242.01	243.63	239.82	227.89	206.78	159.56	95.62	43.71	12.01	2239.26
75.35	241.31	241.53	242.62	241.46	240.36	229.80	212.52	157.90	94.53	43.59	12.56	2239.26
75.45	242.77	242.81	242.18	242.23	241.47	226.58	209.15	158.05	96.04	45.90	13.73	2239.26
75.55	244.72	241.06	244.11	241.72	240.39	234.90	214.67	158.81	95.07	47.03	13.51	2239.26
75.65	244.40	240.52	241.09	244.09	238.60	228.44	212.86	164.85	94.47	45.31	11.85	2239.26
75.75	240.92	242.67	244.21	237.39	242.44	233.64	211.17	152.17	98.39	45.66	12.40	2239.26
75.85	241.67	241.16	241.50	245.22	239.10	230.07	210.84	169.38	94.56	45.78	13.34	2239.26
75.95	239.69	245.26	242.75	244.35	239.49	231.23	208.56	160.32	94.68	42.76	11.96	2239.26
76.05	241.42	241.42	242.05	241.87	236.38	230.58	206.75	149.45	96.10	44.60	11.01	2239.26
76.15	241.94	241.84	244.32	242.83	243.41	229.71	205.75	167.41	97.55	45.25	12.51	2239.26
76.25	242.94	242.85	243.41	242.12	238.92	228.99	209.28	161.98	96.52	42.94	12.51	2239.26
76.35	241.65	242.96	242.71	242.01	238.46	229.03	207.21	160.47	97.52	42.05	11.68	2239.26
76.45	241.19	242.47	240.81	245.10	240.64	232.40	212.42	161.22	94.47	43.94	14.28	2239.26
76.55	242.33	240.59	241.21	241.01	240.74	225.57	210.15	160.17	94.74	45.31	12.23	2239.26
76.65	240.66	239.70	243.25	242.06	240.16	229.71	209.10	163.64	95.83	44.78	12.18	2239.26
76.75	241.21	241.32	241.35	242.40	236.32	226.23	210.63	152.77	96.92	45.07	11.51	2239.26
76.85	243.45	243.09	240.12	242.61	236.99	231.39	209.13	165.30	96.37	44.60	12.51	2239.26
76.95	241.00	241.24	239.99	239.92	241.77	228.82	209.84	162.73	94.68	42.94	12.40	2239.26
77.05	239.48	241.87	238.83	241.16	239.57	229.15	208.36	156.39	97.64	44.83	10.85	2239.26
77.15	240.62	243.30	243.20	242.54	242.52	228.64	208.61	150.35	97.25	41.93	11.57	2239.26
77.25	240.09	241.67	242.19	240.33	240.41	232.17	208.36	159.86	93.59	42.17	12.51	2239.26
77.35	240.21	242.82	243.65	242.23	237.81	231.67	208.28	169.53	97.46	43.06	11.46	2239.26
77.45	242.82	241.96	241.97	240.12	238.21	231.49	209.30	159.71	98.85	47.44	13.23	2239.26
77.55	238.78	244.56	240.58	243.21	239.74	227.62	212.45	153.52	95.98	44.06	12.07	2239.26
77.65	243.50	239.98	239.74	240.76	236.99	230.62	209.64	161.83	97.34	43.11	11.13	2239.26
77.75	238.56	241.79	242.52	242.73	239.79	227.58	209.53	161.68	94.23	45.66	12.51	2239.26
77.85	239.14	241.70	240.85	238.98	238.66	232.74	209.15	153.52	98.18	45.55	11.35	2239.26
77.95	242.16	241.92	241.02	240.77	241.40	229.74	206.39	164.24	96.01	44.66	14.00	2239.26
78.05	239.37	241.32	243.02	241.01	239.59	230.51	207.59	156.39	96.55	46.97	11.79	2239.26
78.15	238.44	241.92	244.29	240.24	237.47	233.08	207.11	156.24	95.92	43.94	13.51	2239.26
78.25	240.66	238.41	241.83	241.49	239.77	227.96	213.32	157.30	94.38	43.17	12.23	2239.26
78.35	242.18	239.62	242.41	242.47	240.61	231.44	206.95	155.64	91.54	46.73	13.01	2239.26

Midpt.	11000	2700	910	240	84	26	8.8	2.7	0.92	0.31	Volume	
78.45	240.85	242.58	239.89	242.55	239.39	231.01	209.89	156.69	94.56	47.74	12.23	2239.26
78.55	241.74	239.89	241.87	238.72	234.30	228.82	208.36	169.98	96.31	43.29	11.73	2239.26
78.65	241.95	240.58	240.89	241.32	240.07	231.87	212.34	149.75	94.83	43.65	11.51	2239.26
78.75	242.33	240.67	241.75	239.87	236.05	231.89	207.70	158.20	96.52	44.72	12.90	2239.26
78.85	244.19	241.47	241.65	241.77	234.45	228.65	211.78	154.58	94.62	47.38	13.73	2239.26
78.95	242.89	239.94	242.15	240.41	240.54	228.94	207.18	158.05	95.92	43.83	11.40	2239.26
79.05	242.24	241.56	242.24	241.67	235.78	229.35	211.27	154.73	95.22	44.36	12.68	2239.26
79.15	242.56	240.73	239.78	242.60	238.06	232.44	208.15	167.71	95.95	45.01	11.79	2239.26
79.25	239.82	239.93	246.50	243.03	240.88	229.44	210.63	153.68	96.73	45.96	12.34	2239.26
79.35	241.50	239.65	242.13	239.79	237.93	232.33	210.84	170.43	96.64	44.72	12.07	2239.26
79.45	238.78	240.66	242.35	243.89	236.80	229.87	207.62	160.47	96.58	42.40	10.18	2239.26
79.55	238.68	241.82	241.56	240.28	238.08	229.92	209.00	158.05	94.53	47.44	12.40	2239.26
79.65	242.32	240.75	243.55	240.71	237.98	229.83	206.21	163.34	92.14	44.72	11.13	2239.26
79.75	240.63	244.66	240.95	239.71	243.11	227.60	213.21	160.32	94.50	46.38	12.45	2239.26
79.85	241.92	240.56	243.87	244.52	240.71	230.76	211.53	159.71	96.61	42.28	10.46	2239.26
79.95	239.14	240.88	243.47	241.77	234.44	228.21	208.92	162.13	94.95	42.88	9.85	2239.26
80.05	240.55	238.05	239.96	241.35	240.78	232.64	208.72	152.92	94.14	41.63	10.68	2239.26
80.15	238.43	243.73	242.35	242.78	243.26	226.10	205.98	172.55	94.98	45.55	12.45	2239.26
80.25	242.11	241.29	242.08	239.79	235.70	228.69	205.73	161.53	93.56	46.32	14.06	2239.26
80.35	238.87	240.20	243.02	242.09	242.20	231.03	205.86	160.32	98.42	45.37	12.90	2239.26
80.45	239.85	242.32	239.24	242.76	233.92	231.06	204.83	161.83	95.04	43.94	10.63	2239.26
80.55	241.77	242.00	240.24	245.52	239.15	224.12	211.12	156.09	96.07	46.79	10.85	2239.26
80.65	238.61	240.77	243.10	242.59	236.15	228.48	209.51	166.51	97.22	46.67	12.45	2239.26
80.75	243.03	243.31	239.42	240.86	236.79	231.23	205.91	161.07	96.34	43.00	11.35	2239.26
80.85	239.96	240.83	243.29	242.41	237.10	232.89	206.60	161.07	97.61	44.66	10.74	2239.26
80.95	238.59	241.81	245.59	242.65	233.57	230.60	204.22	162.58	95.50	44.12	11.62	2239.26
81.05	241.38	239.29	242.82	242.48	234.47	230.49	208.36	154.88	97.76	45.72	12.95	2239.26
81.15	240.67	241.02	240.84	239.48	239.64	229.07	210.20	157.15	95.35	43.06	11.40	2239.26
81.25	244.61	240.61	244.93	238.99	240.46	227.17	204.14	150.05	96.16	44.24	11.85	2239.26
81.35	239.88	239.55	238.86	243.80	242.10	233.33	204.94	164.54	96.31	43.35	11.73	2239.26
81.45	242.19	241.75	241.63	240.52	240.41	227.73	207.39	161.98	94.02	44.95	13.51	2239.26
81.55	240.89	238.05	240.92	239.99	239.07	228.82	212.91	162.13	98.24	40.98	12.12	2239.26
81.65	243.03	238.99	238.22	241.62	239.40	230.12	207.80	166.51	98.36	45.37	12.29	2239.26
81.75	239.40	240.95	242.67	239.48	237.99	228.94	208.95	164.70	98.06	42.58	13.12	2239.26
81.85	243.07	241.47	242.57	238.97	240.38	230.44	207.44	164.85	97.25	43.71	12.45	2239.26
81.95	240.15	241.63	242.09	240.54	236.20	232.30	209.23	165.45	95.04	44.12	12.07	2239.26
82.05	241.56	241.88	241.20	242.37	239.55	231.39	211.25	175.26	97.34	43.29	10.85	2239.26
82.15	242.42	241.17	241.92	240.71	240.91	223.85	208.38	159.26	98.06	46.97	12.12	2239.26
82.25	240.61	238.53	244.19	239.64	239.17	230.58	208.54	166.96	94.35	45.90	11.51	2239.26
82.35	239.94	243.24	241.62	240.21	237.36	231.49	209.38	155.18	96.76	47.50	10.35	2239.26
82.45	240.21	240.58	238.43	240.72	238.09	229.53	210.30	156.85	95.86	44.48	10.90	2239.26
82.55	240.26	241.43	239.54	242.46	237.21	232.46	210.58	155.49	94.29	42.52	11.90	2239.26
82.65	241.22	239.86	242.75	238.64	238.41	231.92	213.62	167.41	93.53	43.77	10.74	2239.26
82.75	238.27	238.94	241.45	237.27	237.36	229.05	209.00	164.24	94.44	44.24	10.90	2239.26
82.85	242.50	239.83	239.83	239.99	238.25	230.17	208.23	167.71	96.13	43.47	11.24	2239.26

Midpt.	11000	2700	910	240	84	26	8.8	2.7	0.92	0.31	Volume	
82.95	235.99	238.32	239.42	239.43	239.30	229.10	206.11	159.56	95.13	42.64	11.01	2239.26
83.05	241.91	242.16	241.72	240.36	238.46	229.19	207.16	153.98	93.41	42.52	12.29	2239.26
83.15	237.88	242.10	238.88	240.42	240.64	226.65	206.67	155.94	95.13	47.21	12.23	2239.26
83.25	241.60	240.05	242.38	242.57	238.68	232.90	208.92	156.39	95.44	41.81	11.29	2239.26
83.35	244.12	238.39	243.19	241.87	237.34	227.71	206.01	158.20	98.76	44.72	12.73	2239.26
83.45	239.26	241.13	243.45	237.53	237.71	228.28	209.92	166.36	99.36	41.99	12.29	2239.26
83.55	239.54	239.23	237.71	241.08	237.42	225.96	209.25	160.17	95.50	43.94	12.29	2239.26
83.65	238.48	242.96	240.56	243.33	240.48	226.94	209.89	157.90	94.65	44.60	10.90	2239.26
83.75	240.25	239.40	243.03	239.17	238.63	226.37	207.08	150.66	91.72	42.46	10.90	2239.26
83.85	241.56	239.83	243.05	239.55	235.85	226.26	208.90	161.98	94.38	45.19	11.51	2239.26
83.95	241.12	239.24	241.35	239.86	238.95	227.46	209.99	152.47	98.97	46.50	11.46	2239.26
84.05	239.95	240.39	238.89	238.58	238.63	228.73	213.65	164.70	94.92	43.29	11.90	2239.26
84.15	239.98	240.76	237.70	241.30	237.17	222.89	203.91	163.64	93.32	44.83	11.57	2239.26
84.25	239.16	237.60	240.19	241.76	236.80	230.71	210.58	151.11	95.89	46.32	9.74	2239.26
84.35	240.47	241.78	240.36	236.87	236.52	229.78	206.95	158.81	97.61	46.26	12.62	2239.26
84.45	239.11	235.80	237.50	243.23	238.31	229.46	212.40	162.13	96.88	45.78	10.96	2239.26
84.55	241.42	241.87	241.11	237.72	237.88	230.23	209.23	161.83	95.80	41.69	11.73	2239.26
84.65	237.42	239.29	241.09	243.47	237.69	231.42	212.57	160.92	97.49	45.01	11.96	2239.26
84.75	238.81	237.54	241.09	238.89	236.57	229.14	209.28	158.20	96.07	44.78	11.73	2239.26
84.85	238.76	244.33	243.15	240.89	236.97	230.07	204.99	157.75	97.37	44.18	12.23	2239.26
84.95	239.24	241.16	241.04	241.39	240.90	225.80	207.57	153.68	97.52	45.37	10.79	2239.26
85.05	240.65	240.69	243.28	237.12	237.98	228.15	205.57	153.07	94.08	43.71	12.07	2239.26
85.15	239.01	240.39	240.79	244.32	239.76	228.35	206.47	148.84	96.31	43.00	11.57	2239.26
85.25	243.20	240.67	240.45	238.84	239.17	227.98	208.95	160.92	97.91	44.72	10.79	2239.26
85.35	238.20	235.28	239.22	241.06	241.08	230.56	210.71	155.18	95.50	42.82	11.46	2239.26
85.45	238.41	240.29	240.56	240.79	239.74	230.08	206.85	159.56	95.10	42.88	12.01	2239.26
85.55	239.95	241.09	242.28	240.05	240.86	229.42	206.90	158.05	97.55	44.24	12.29	2239.26
85.65	239.02	240.27	239.92	238.15	234.07	229.83	208.72	158.96	98.91	43.00	12.45	2239.26
85.75	239.78	239.99	239.63	241.12	235.58	227.98	207.39	157.30	92.33	44.89	11.18	2239.26
85.85	238.12	241.14	241.31	238.31	235.98	226.89	202.38	155.79	94.11	45.72	11.51	2239.26
85.95	239.32	237.49	237.85	239.55	236.30	229.21	209.10	150.20	93.71	46.91	12.68	2239.26
86.05	240.60	241.51	239.25	238.77	235.13	227.58	210.35	162.73	96.61	44.12	11.73	2239.26
86.15	240.27	237.76	241.51	237.52	235.21	233.76	206.49	159.56	99.63	42.40	11.85	2239.26
86.25	239.42	241.12	238.64	239.55	236.25	226.78	205.75	157.60	92.84	44.95	10.85	2239.26
86.35	237.50	241.30	240.66	241.89	235.71	229.28	210.12	157.00	96.49	42.34	11.40	2239.26
86.45	240.45	240.39	239.65	237.33	238.61	230.39	207.06	147.64	93.78	42.52	11.90	2239.26
86.55	240.26	240.00	238.83	237.61	239.13	227.44	201.00	163.19	96.98	44.00	11.68	2239.26
86.65	240.03	240.28	239.65	240.19	241.78	231.64	214.01	157.15	95.25	42.17	11.40	2239.26
86.75	235.85	241.34	241.99	237.14	235.60	226.05	208.61	161.98	94.62	45.01	10.79	2239.26
86.85	240.10	240.12	237.49	240.54	235.31	230.33	207.93	158.51	93.65	44.24	10.79	2239.26
86.95	236.64	238.55	238.08	237.90	233.01	227.96	213.65	156.69	95.22	45.84	12.29	2239.26
87.05	241.51	237.65	239.63	236.40	235.70	223.53	207.36	164.39	98.18	43.47	10.35	2239.26
87.15	240.83	237.15	239.20	237.30	235.85	231.42	209.05	170.28	94.83	44.78	10.96	2239.26
87.25	242.51	237.84	238.66	237.68	236.72	227.80	206.39	166.81	95.10	45.07	11.62	2239.26
87.35	238.12	239.85	239.96	238.84	233.67	228.42	206.14	164.85	95.10	43.77	10.90	2239.26

Midpt.	11000	2700	910	240	84	26	8.8	2.7	0.92	0.31	Volume	
87.45	237.41	239.94	239.05	238.69	237.27	227.08	208.15	147.18	101.05	43.17	10.90	2239.26
87.55	236.84	239.20	240.04	239.54	230.19	228.32	206.24	160.47	94.05	42.46	11.07	2239.26
87.65	236.92	237.69	236.99	236.18	233.70	227.96	208.31	171.19	96.73	42.64	11.35	2239.26
87.75	238.38	237.00	242.31	237.19	238.01	228.42	208.90	157.60	96.88	45.07	11.62	2239.26
87.85	237.84	234.59	237.63	237.19	238.85	228.32	210.45	158.96	97.22	45.43	12.01	2239.26
87.95	236.94	240.47	239.86	239.08	238.77	227.26	208.36	159.26	95.19	44.24	12.01	2239.26
88.05	237.25	240.10	236.86	237.51	241.06	227.28	203.53	144.77	96.95	42.70	11.01	2239.26
88.15	237.82	239.43	240.97	238.68	237.22	226.67	204.88	155.03	96.31	44.42	10.85	2239.26
88.25	237.36	241.19	237.52	240.84	235.66	228.07	207.77	154.88	96.01	42.70	11.73	2239.26
88.35	238.52	236.00	236.00	237.56	237.36	226.90	205.55	167.41	96.95	44.95	11.57	2239.26
88.45	240.12	237.49	234.90	239.41	235.38	229.05	208.23	150.81	96.46	48.10	11.96	2239.26
88.55	237.79	234.07	238.25	239.18	232.21	231.01	202.56	159.86	93.08	44.30	11.24	2239.26
88.65	237.97	236.58	237.69	239.05	236.08	225.40	206.72	155.03	93.87	45.61	11.07	2239.26
88.75	238.39	240.61	236.46	238.04	234.99	227.67	211.35	160.32	96.73	44.95	11.13	2239.26
88.85	238.91	237.86	237.83	239.55	234.72	228.32	203.91	155.64	99.33	43.47	11.07	2239.26
88.95	234.55	238.68	239.43	239.15	232.36	225.08	209.05	162.28	96.34	43.89	11.35	2239.26
89.05	239.60	236.24	235.93	237.26	235.93	223.99	207.62	161.83	98.76	43.23	10.35	2239.26
89.15	237.84	239.52	236.78	236.43	236.77	226.26	204.88	160.17	95.25	44.66	11.57	2239.26
89.25	236.41	239.39	237.42	239.34	237.71	229.05	203.79	154.58	96.07	44.72	12.51	2239.26
89.35	235.72	235.74	238.06	238.35	238.40	228.10	208.69	152.47	95.31	43.71	13.84	2239.26
89.45	236.50	236.26	237.74	235.72	233.63	227.40	205.19	155.49	94.38	46.02	11.07	2239.26
89.55	239.65	234.77	238.53	238.11	231.52	224.49	210.61	160.62	97.01	44.30	10.74	2239.26
89.65	234.77	236.57	236.81	234.49	234.76	220.83	204.78	156.85	94.44	43.94	11.13	2239.26
89.75	233.91	238.93	235.84	237.28	234.84	225.46	202.18	155.49	92.08	45.19	10.02	2239.26
89.85	236.91	236.17	237.16	235.86	234.32	223.08	204.53	161.68	92.87	44.12	11.90	2239.26
89.95	236.47	237.22	235.36	237.67	231.03	225.12	210.35	155.34	93.81	46.55	12.12	2239.26
90.05	237.81	236.90	236.40	234.01	232.24	226.35	207.39	155.79	95.59	47.38	11.51	2239.26
90.15	235.88	237.96	232.91	237.40	231.77	225.65	202.64	147.79	93.20	44.72	10.68	2239.26
90.25	235.79	236.15	238.20	232.61	231.94	224.17	205.45	165.00	94.20	46.20	10.52	2239.26
90.35	235.84	235.04	236.00	235.34	233.63	226.53	203.25	159.71	93.93	42.64	12.18	2239.26
90.45	236.18	235.71	235.54	234.61	227.83	225.48	206.85	143.86	95.98	45.07	11.68	2239.26
90.55	236.93	238.58	234.01	234.23	233.25	223.69	204.12	152.17	95.80	42.82	10.18	2239.26
90.65	233.57	234.79	234.65	233.24	233.60	225.32	201.46	163.34	95.59	42.05	10.90	2239.26
90.75	233.26	235.69	236.03	232.92	229.96	222.58	201.69	158.05	95.68	45.43	9.47	2239.26
90.85	235.87	234.35	232.11	234.33	232.58	226.73	205.22	151.26	96.76	45.90	10.68	2239.26
90.95	230.89	233.45	234.83	234.82	230.36	224.51	200.36	157.90	94.74	42.34	10.85	2239.26
91.05	232.48	234.01	234.86	233.77	227.44	221.66	199.34	162.43	92.72	44.00	11.85	2239.26
91.15	234.55	231.60	234.93	235.43	232.41	226.49	202.07	159.11	97.70	43.65	10.35	2239.26
91.25	234.59	230.52	235.78	231.74	233.88	226.78	204.78	160.92	94.32	43.53	11.51	2239.26
91.35	234.06	237.42	233.83	233.54	232.78	222.62	207.80	158.05	96.82	43.83	10.52	2239.26
91.45	232.04	237.64	234.69	232.13	230.08	224.57	201.00	152.17	93.68	44.78	10.96	2239.26
91.55	233.94	236.59	233.91	234.05	232.66	221.42	200.31	145.83	90.61	45.31	12.90	2239.26
91.65	230.64	234.25	235.33	235.50	230.95	226.26	203.10	156.24	94.26	42.34	11.68	2239.26
91.75	232.78	234.95	235.83	234.06	228.15	223.42	204.22	157.45	92.42	43.77	11.62	2239.26
91.85	233.54	231.17	234.95	233.80	229.89	222.07	202.56	150.96	95.28	42.64	11.29	2239.26

Midpt.	11000	2700	910	240	84	26	8.8	2.7	0.92	0.31	Volume	
91.95	232.96	232.90	234.15	232.30	228.94	223.53	200.06	159.26	93.99	44.89	12.18	2239.26
92.05	233.40	232.03	233.97	234.31	231.37	220.89	201.36	149.45	92.90	45.61	12.12	2239.26
92.15	233.07	231.88	233.64	236.68	229.99	222.71	206.78	153.68	92.78	44.95	10.96	2239.26
92.25	233.01	232.57	231.85	235.88	229.76	222.14	207.36	147.94	94.80	45.72	12.01	2239.26
92.35	233.07	233.87	234.34	234.03	237.10	223.12	207.08	154.43	95.62	44.06	10.79	2239.26
92.45	231.58	232.48	234.79	233.64	229.14	224.12	202.69	152.01	92.99	44.18	11.96	2239.26
92.55	233.25	232.79	233.58	233.06	228.30	225.99	197.71	161.98	91.81	43.89	11.18	2239.26
92.65	234.93	234.32	232.38	232.94	231.45	221.41	197.91	149.75	95.89	43.23	10.74	2239.26
92.75	232.42	233.06	234.41	231.56	230.70	221.80	205.04	157.00	94.08	43.11	11.51	2239.26
92.85	235.27	234.50	231.11	233.41	230.40	221.12	201.62	154.43	97.37	43.00	10.46	2239.26
92.95	233.14	235.39	232.68	233.40	230.16	223.01	203.48	165.15	92.57	45.01	10.90	2239.26
93.05	235.48	233.20	233.28	233.95	229.72	223.99	205.40	152.77	95.80	41.87	11.96	2239.26
93.15	233.73	235.56	234.10	236.19	226.60	222.42	201.82	157.15	94.89	40.98	11.13	2239.26
93.25	234.63	231.90	234.03	234.97	235.56	222.28	205.47	150.66	91.45	43.47	12.07	2239.26
93.35	236.86	232.93	234.33	231.29	230.83	227.74	206.42	152.32	94.29	45.31	11.62	2239.26
93.45	231.46	235.62	231.71	233.46	233.11	225.15	205.24	156.69	92.36	42.58	10.79	2239.26
93.55	236.90	235.83	235.86	234.99	227.51	228.96	198.55	162.13	92.66	45.72	11.62	2239.26
93.65	235.75	233.40	232.99	235.13	229.89	224.10	203.58	160.62	95.01	42.46	9.69	2239.26
93.75	234.02	236.97	233.47	233.95	230.14	223.87	201.90	153.68	96.73	44.18	11.40	2239.26
93.85	232.43	238.03	236.94	235.07	232.81	222.16	203.48	157.75	97.04	41.75	13.12	2239.26
93.95	231.81	235.20	235.84	232.56	233.57	221.14	202.30	154.13	96.46	40.45	12.07	2239.26
94.05	235.77	237.17	235.76	234.09	232.83	226.32	207.03	152.92	96.22	43.71	11.90	2239.26
94.15	234.64	238.19	237.03	234.67	229.02	227.40	202.97	164.24	95.31	46.61	11.24	2239.26
94.25	234.48	235.22	238.65	233.82	232.91	225.58	205.40	169.53	94.92	40.98	11.62	2239.26
94.35	234.57	235.34	238.23	235.11	233.31	223.73	205.55	158.66	98.64	44.95	11.68	2239.26
94.45	236.29	235.83	234.37	237.47	236.18	225.19	207.31	158.05	94.89	44.18	12.51	2239.26
94.55	236.84	238.36	237.11	232.90	236.85	231.17	204.91	162.58	98.30	44.00	11.73	2239.26
94.65	235.59	236.12	236.16	235.52	229.79	230.80	206.09	161.68	94.26	43.23	11.73	2239.26
94.75	234.36	234.90	233.89	235.54	235.33	229.26	206.16	158.51	94.95	43.59	12.62	2239.26
94.85	235.41	238.77	238.60	238.02	234.09	226.03	202.00	157.00	93.78	43.65	12.45	2239.26
94.95	238.63	237.74	238.59	238.88	235.26	223.92	206.11	163.94	97.70	42.46	12.29	2239.26
95.05	236.20	240.04	238.33	237.64	232.91	225.69	209.10	151.26	94.05	44.48	11.57	2239.26
95.15	240.13	238.59	237.00	238.70	236.97	227.74	207.01	160.47	95.56	43.94	11.57	2239.26
95.25	237.63	238.10	239.43	238.71	234.45	227.74	203.89	161.07	96.46	44.78	10.46	2239.26
95.35	236.48	238.44	238.63	237.51	236.20	228.08	207.08	158.96	98.21	44.83	11.85	2239.26
95.45	238.86	240.53	240.89	240.46	237.31	225.32	208.33	162.28	95.13	43.71	11.79	2239.26
95.55	237.20	239.69	238.82	238.99	236.28	229.62	213.67	162.73	98.91	44.48	11.73	2239.26
95.65	238.39	239.91	239.49	241.31	236.03	234.24	208.87	157.60	94.71	46.50	10.96	2239.26
95.75	240.74	242.10	242.38	241.25	233.05	231.69	210.22	161.37	98.12	47.09	12.62	2239.26
95.85	242.35	238.94	240.92	241.95	239.50	231.24	212.86	163.19	98.73	48.81	11.68	2239.26
95.95	239.56	238.73	239.65	242.23	239.30	231.78	210.63	162.58	97.64	46.67	12.51	2239.26
96.05	241.71	243.07	241.04	242.76	241.58	230.99	210.74	164.54	100.02	45.49	13.12	2239.26
96.15	241.82	243.06	241.92	239.44	242.20	230.37	210.81	165.45	97.94	47.15	11.96	2239.26
96.25	244.05	240.37	243.09	245.85	240.44	233.30	212.78	163.79	97.40	45.43	12.51	2239.26
96.35	243.62	242.46	242.77	241.49	242.04	227.14	208.90	170.73	97.91	47.92	11.01	2239.26

Midpt.	11000	2700	910	240	84	26	8.8	2.7	0.92	0.31	Volume	
96.45	241.67	244.81	244.50	242.81	241.45	233.12	212.88	169.07	95.35	44.60	11.57	2239.26
96.55	243.84	240.94	242.28	241.99	241.01	228.85	212.11	168.47	97.76	44.06	10.24	2239.26
96.65	246.64	246.30	246.08	244.52	238.75	233.30	208.10	172.09	98.79	47.03	13.34	2239.26
96.75	242.30	242.84	244.11	243.91	241.87	238.21	209.87	169.07	103.07	46.79	13.23	2239.26
96.85	246.91	247.38	245.31	244.54	245.59	230.87	212.40	168.47	101.59	45.19	12.40	2239.26
96.95	245.56	246.46	246.93	246.40	240.31	233.94	216.53	162.88	98.49	45.37	11.07	2239.26
97.05	245.18	247.23	244.23	244.25	241.08	232.96	213.16	166.05	98.67	45.37	11.68	2239.26
97.15	246.08	246.76	246.41	246.46	245.39	233.10	218.91	174.81	99.57	46.85	11.57	2239.26
97.25	241.48	245.42	248.77	249.89	243.06	236.23	213.26	165.60	100.12	46.26	12.12	2239.26
97.35	248.75	248.26	250.96	244.83	243.19	238.78	215.13	171.19	100.06	43.41	12.68	2239.26
97.45	249.18	245.82	246.73	247.52	244.77	236.83	216.84	163.79	104.83	48.63	12.18	2239.26
97.55	250.36	247.58	249.37	248.17	242.99	238.78	218.76	160.62	98.58	48.27	12.90	2239.26
97.65	248.31	251.58	250.36	247.08	243.83	236.78	224.73	165.60	100.60	45.01	13.40	2239.26
97.75	247.21	247.04	251.66	248.37	251.41	236.90	214.92	170.88	99.27	44.42	12.79	2239.26
97.85	250.26	248.03	249.11	250.57	247.05	234.30	216.33	160.92	100.87	46.85	12.84	2239.26
97.95	250.51	251.66	250.18	252.91	244.72	239.67	219.11	155.34	103.23	46.20	13.89	2239.26
98.05	251.61	250.70	253.05	248.99	247.69	237.65	219.73	168.62	104.67	47.86	12.18	2239.26
98.15	249.29	248.05	254.65	251.96	252.82	241.22	212.34	168.77	101.41	48.27	13.56	2239.26
98.25	253.46	252.99	252.25	249.63	248.96	235.81	217.20	161.07	101.47	45.66	12.84	2239.26
98.35	250.27	253.85	253.41	250.92	251.80	245.19	218.30	168.47	100.12	50.29	12.07	2239.26
98.45	250.46	254.92	252.86	253.03	249.79	238.97	217.38	172.55	104.98	48.04	13.40	2239.26
98.55	253.48	253.74	254.46	253.77	251.38	241.03	221.57	163.49	101.90	47.62	12.07	2239.26
98.65	254.74	254.88	253.72	253.17	250.66	240.40	219.11	182.96	101.66	49.88	13.40	2239.26
98.75	252.16	253.68	254.81	252.94	252.92	241.81	220.88	176.62	102.23	49.99	14.61	2239.26
98.85	253.35	252.88	256.06	254.11	257.05	243.78	221.85	171.04	104.80	46.79	12.23	2239.26
98.95	253.57	258.70	259.67	256.79	252.52	247.03	221.98	170.58	103.59	46.79	12.23	2239.26
99.05	256.49	257.04	257.13	255.35	254.08	244.24	220.60	171.94	103.35	46.97	10.57	2239.26
99.15	255.34	253.70	256.05	255.62	253.07	243.14	225.02	166.66	103.74	49.22	12.34	2239.26
99.25	254.66	259.24	259.04	256.51	255.10	243.44	223.79	167.87	102.86	46.20	13.23	2239.26
99.35	257.08	255.69	255.63	260.43	253.98	251.29	230.33	167.26	106.82	48.87	12.90	2239.26
99.45	261.41	258.78	256.63	256.81	254.60	246.17	224.79	178.28	104.43	50.53	14.83	2239.26
99.55	261.09	258.75	256.53	261.21	257.77	248.62	224.27	187.34	104.89	48.99	11.85	2239.26
99.65	260.32	259.04	261.23	259.70	259.25	249.28	225.53	170.73	109.02	48.27	13.12	2239.26
99.75	262.24	261.80	259.13	197.16	257.67	246.58	226.09	166.96	102.29	50.88	13.51	2239.26
99.85	67.90	60.20	34.31	0.00	39.11	36.80	4.27	91.33	29.71	13.88	0.33	2239.26

Table of Ca^{2+} population data for simulation with Ca^{2+} and Na^{+} across bulk density ratios (#JJF-GRS12G8)

	Population in histogram bin at Na^{+}/Ca^{2+} ratio:											
Midpt.	11000	2700	910	240	84	26	8.8	2.7	0.92	0.31	0.05	Volume
-99.85	0.02	0.04	0.01	0.00	0.32	1.09	0.41	31.85	26.30	35.29	1.55	2239.26
-99.75	0.00	0.06	0.22	0.59	2.37	7.20	19.31	50.87	91.45	123.35	93.93	2239.26
-99.65	0.05	0.10	0.14	0.79	2.90	7.50	20.59	52.68	90.82	122.64	91.94	2239.26
-99.55	0.03	0.05	0.25	0.84	2.45	7.27	20.77	48.46	91.66	123.06	90.06	2239.26

Midpt.	11000	2700	910	240	84	26	8.8	2.7	0.92	0.31	0.05	Volume
-99.45	0.03	0.04	0.22	0.95	2.68	7.71	20.97	55.55	91.39	121.69	94.15	2239.26
-99.35	0.03	0.07	0.25	0.81	2.72	7.36	19.59	52.53	93.14	123.53	95.76	2239.26
-99.25	0.04	0.07	0.30	0.76	2.33	7.07	21.43	55.70	92.18	122.05	91.22	2239.26
-99.15	0.03	0.09	0.26	0.90	2.06	7.41	20.11	51.93	91.27	124.36	88.45	2239.26
-99.05	0.01	0.09	0.24	0.84	2.30	7.18	19.65	54.95	91.45	122.41	96.59	2239.26
-98.95	0.01	0.07	0.31	0.91	2.37	7.25	19.34	56.46	95.95	126.91	90.55	2239.26
-98.85	0.03	0.07	0.19	0.90	2.16	7.57	20.69	50.72	93.02	126.08	89.78	2239.26
-98.75	0.02	0.04	0.18	0.77	2.47	7.30	21.18	47.85	92.08	123.95	90.83	2239.26
-98.65	0.03	0.08	0.22	0.81	2.10	7.61	19.42	53.59	93.96	122.23	91.61	2239.26
-98.55	0.03	0.09	0.19	0.80	2.20	7.23	20.31	52.68	94.17	123.06	90.67	2239.26
-98.45	0.03	0.11	0.22	0.79	2.68	8.00	21.02	51.93	88.64	128.34	91.27	2239.26
-98.35	0.03	0.11	0.23	0.75	2.47	7.46	20.49	47.25	90.42	124.24	90.17	2239.26
-98.25	0.03	0.10	0.29	0.84	2.20	7.30	21.25	53.74	94.32	117.19	91.39	2239.26
-98.15	0.01	0.12	0.21	0.88	2.15	7.05	20.87	51.78	89.73	124.18	91.22	2239.26
-98.05	0.07	0.07	0.22	0.85	2.47	7.37	20.34	52.23	90.00	114.93	90.61	2239.26
-97.95	0.03	0.02	0.30	0.96	2.67	7.02	20.54	52.08	89.31	129.52	94.21	2239.26
-97.85	0.02	0.09	0.25	1.01	2.31	7.77	18.01	51.17	91.09	123.71	89.12	2239.26
-97.75	0.00	0.07	0.25	0.83	2.40	7.18	20.31	50.72	90.73	124.01	92.55	2239.26
-97.65	0.02	0.05	0.34	0.76	2.72	7.73	21.20	50.12	90.48	121.22	85.13	2239.26
-97.55	0.01	0.05	0.38	0.95	2.35	7.20	20.51	50.42	91.93	122.17	86.79	2239.26
-97.45	0.02	0.09	0.26	0.73	2.37	6.84	20.41	51.17	91.75	121.46	1749.43	2239.26
-97.35	0.00	0.10	0.24	0.92	2.30	7.32	20.69	52.23	90.15	125.43	90.00	2239.26
-97.25	0.03	0.07	0.18	0.81	2.00	7.25	21.08	46.95	91.78	121.34	89.72	2239.26
-97.15	0.03	0.07	0.17	0.74	2.63	7.89	18.98	50.12	92.30	126.26	87.84	2239.26
-97.05	0.01	0.08	0.21	0.79	2.28	6.93	19.49	45.29	86.71	123.71	83.36	2239.26
-96.95	0.03	0.09	0.18	0.84	2.33	7.59	20.41	49.21	89.16	123.35	90.17	2239.26
-96.85	0.02	0.05	0.22	0.89	2.60	7.68	21.69	46.95	89.13	122.46	89.72	2239.26
-96.75	0.01	0.05	0.20	0.88	2.21	7.34	18.75	47.70	86.98	128.93	90.33	2239.26
-96.65	0.00	0.07	0.24	0.69	2.57	7.61	20.57	52.53	89.55	120.63	85.52	2239.26
-96.55	0.02	0.05	0.21	0.85	2.53	7.30	19.95	54.04	91.00	126.08	87.90	2239.26
-96.45	0.02	0.07	0.22	0.85	2.40	8.25	20.36	56.76	87.59	122.29	83.03	2239.26
-96.35	0.03	0.15	0.22	0.72	2.18	7.27	20.49	56.01	90.06	122.82	89.00	2239.26
-96.25	0.03	0.04	0.15	0.90	2.13	7.75	20.18	50.27	93.62	125.49	85.41	2239.26
-96.15	0.01	0.07	0.22	0.93	2.21	7.41	20.00	52.84	94.14	124.54	82.25	2239.26
-96.05	0.02	0.07	0.30	0.94	2.13	7.07	20.00	48.16	90.45	120.80	87.95	2239.26
-95.95	0.04	0.09	0.29	0.84	2.25	7.28	19.62	48.91	91.00	123.65	89.67	2239.26
-95.85	0.01	0.05	0.19	0.96	2.65	7.53	20.21	52.23	92.24	125.85	89.61	2239.26
-95.75	0.03	0.09	0.18	0.80	2.47	7.62	20.05	48.76	89.85	124.42	86.74	2239.26
-95.65	0.00	0.09	0.22	0.81	2.47	7.30	19.31	49.21	90.24	121.87	86.96	2239.26
-95.55	0.03	0.07	0.29	0.85	2.65	6.98	19.98	46.04	91.75	119.74	86.07	2239.26
-95.45	0.02	0.06	0.24	0.90	2.25	7.36	20.44	48.76	93.87	128.69	83.80	2239.26
-95.35	0.02	0.11	0.17	0.76	2.57	7.39	20.16	52.68	90.09	130.17	82.58	2239.26
-95.25	0.03	0.07	0.18	0.72	2.42	7.89	20.54	50.42	89.85	120.74	84.24	2239.26
-95.15	0.05	0.09	0.28	0.85	2.42	6.86	19.72	46.04	91.66	123.77	80.59	2239.26
-95.05	0.03	0.07	0.29	0.81	2.52	6.87	18.85	51.02	90.85	129.70	86.90	2239.26

Midpt.	11000	2700	910	240	84	26	8.8	2.7	0.92	0.31	0.05	Volume
-94.95	0.00	0.06	0.21	0.94	2.52	7.57	19.75	49.51	90.09	119.20	84.24	2239.26
-94.85	0.05	0.07	0.20	0.81	2.26	7.43	19.62	53.89	90.09	122.52	81.98	2239.26
-94.75	0.04	0.07	0.24	0.86	2.21	7.98	21.20	48.91	85.59	124.07	84.02	2239.26
-94.65	0.01	0.11	0.22	0.70	2.50	7.86	19.80	48.61	91.69	125.07	84.36	2239.26
-94.55	0.03	0.15	0.18	0.81	2.75	7.77	20.92	46.80	88.85	122.35	82.69	2239.26
-94.45	0.02	0.10	0.22	0.85	2.37	7.78	19.57	53.29	90.03	123.83	88.51	2239.26
-94.35	0.02	0.12	0.29	0.91	2.25	7.14	20.51	51.78	91.72	127.15	81.86	2239.26
-94.25	0.00	0.07	0.20	0.92	2.38	7.77	21.36	47.25	88.67	126.56	80.87	2239.26
-94.15	0.03	0.07	0.22	0.96	2.18	7.37	19.06	54.80	93.11	121.04	86.68	2239.26
-94.05	0.03	0.08	0.28	0.87	2.05	7.71	21.31	48.76	91.75	122.88	84.08	2239.26
-93.95	0.03	0.13	0.24	0.84	2.13	7.39	20.31	48.16	89.64	127.51	79.65	2239.26
-93.85	0.05	0.07	0.28	0.72	2.33	6.54	20.92	44.68	92.66	127.62	80.87	2239.26
-93.75	0.03	0.10	0.22	0.94	2.52	8.07	19.65	52.84	93.05	125.67	83.41	2239.26
-93.65	0.03	0.08	0.25	0.81	2.16	7.02	20.05	57.82	88.73	124.48	88.56	2239.26
-93.55	0.03	0.09	0.14	0.72	1.91	8.16	20.54	45.89	88.73	123.83	84.24	2239.26
-93.45	0.03	0.10	0.28	0.90	2.57	7.20	20.49	50.57	87.62	123.41	82.03	2239.26
-93.35	0.02	0.09	0.25	0.83	2.40	7.45	20.11	53.29	91.48	124.42	88.17	2239.26
-93.25	0.02	0.07	0.22	0.90	2.21	8.12	18.96	50.57	87.77	121.34	80.87	2239.26
-93.15	0.02	0.08	0.20	0.95	1.93	7.77	19.39	49.82	94.77	122.41	83.25	2239.26
-93.05	0.02	0.07	0.21	0.73	2.73	7.61	20.59	49.21	93.62	130.95	81.70	2239.26
-92.95	0.02	0.07	0.21	0.86	2.16	7.78	20.41	55.85	92.63	125.79	85.24	2239.26
-92.85	0.02	0.13	0.18	0.84	2.25	7.03	20.64	51.02	93.62	126.26	83.47	2239.26
-92.75	0.03	0.12	0.18	0.81	2.10	7.80	22.58	50.72	92.63	122.64	78.82	2239.26
-92.65	0.01	0.04	0.22	0.92	2.25	7.52	20.92	51.78	90.70	123.47	81.92	2239.26
-92.55	0.05	0.10	0.26	0.84	2.55	7.16	21.20	57.21	95.80	124.18	82.69	2239.26
-92.45	0.01	0.07	0.24	0.88	1.90	7.21	20.41	49.97	92.66	128.99	78.16	2239.26
-92.35	0.03	0.07	0.22	1.02	2.40	7.55	21.48	50.27	91.30	129.64	77.22	2239.26
-92.25	0.03	0.09	0.20	1.04	2.60	8.05	20.69	50.27	90.57	125.55	82.69	2239.26
-92.15	0.01	0.05	0.28	0.80	2.58	7.68	21.61	52.38	92.24	125.37	83.47	2239.26
-92.05	0.04	0.08	0.18	0.95	2.28	7.48	20.59	53.44	96.13	128.16	79.82	2239.26
-91.95	0.03	0.06	0.32	0.85	2.28	8.32	20.67	51.33	90.42	125.25	86.02	2239.26
-91.85	0.02	0.08	0.18	0.81	2.67	7.46	20.87	54.50	91.90	129.23	80.26	2239.26
-91.75	0.02	0.09	0.22	1.01	2.42	7.23	21.48	51.17	89.79	128.75	83.58	2239.26
-91.65	0.03	0.06	0.23	1.05	2.70	7.39	20.16	54.80	92.81	128.16	83.03	2239.26
-91.55	0.03	0.05	0.23	0.83	2.53	7.87	19.42	53.14	94.92	126.91	84.96	2239.26
-91.45	0.04	0.08	0.25	0.93	2.62	7.70	21.31	49.21	95.59	127.98	83.14	2239.26
-91.35	0.04	0.07	0.28	0.91	2.30	7.71	19.93	49.06	92.96	127.39	86.68	2239.26
-91.25	0.00	0.09	0.29	0.95	2.55	7.89	21.25	49.36	94.68	129.52	1742.29	2239.26
-91.15	0.02	0.08	0.22	1.04	2.50	7.66	20.74	50.42	95.35	126.73	82.64	2239.26
-91.05	0.03	0.11	0.24	0.94	2.77	7.73	21.38	55.40	94.89	129.70	77.38	2239.26
-90.95	0.00	0.15	0.25	0.78	2.63	8.02	21.23	49.06	94.95	127.86	80.92	2239.26
-90.85	0.01	0.08	0.25	0.81	2.62	7.73	21.08	56.31	94.68	132.72	83.80	2239.26
-90.75	0.06	0.08	0.28	0.81	2.26	7.66	22.63	57.21	93.62	135.04	87.23	2239.26
-90.65	0.02	0.11	0.28	0.90	2.50	7.89	20.82	54.95	94.89	122.58	83.69	2239.26
-90.55	0.03	0.10	0.24	0.98	2.50	8.68	21.36	49.67	98.85	133.79	84.85	2239.26

Midpt.	11000	2700	910	240	84	26	8.8	2.7	0.92	0.31	0.05	Volume
-90.45	0.03	0.07	0.25	0.93	2.55	7.98	21.43	55.10	97.55	132.43	78.54	2239.26
-90.35	0.03	0.11	0.19	1.00	2.48	7.59	21.87	48.61	95.95	129.82	1741.46	2239.26
-90.25	0.03	0.09	0.20	0.90	2.65	8.14	20.28	53.44	99.84	128.34	88.78	2239.26
-90.15	0.02	0.11	0.28	0.91	2.62	7.48	20.67	57.52	96.13	123.89	87.07	2239.26
-90.05	0.02	0.13	0.27	0.82	2.21	8.45	21.46	50.57	94.50	129.76	79.43	2239.26
-89.95	0.01	0.11	0.21	0.88	2.70	7.77	22.02	55.70	93.59	135.39	80.15	2239.26
-89.85	0.03	0.08	0.28	1.04	2.33	7.98	21.31	53.59	96.49	129.64	82.20	2239.26
-89.75	0.00	0.06	0.24	0.73	2.58	8.45	22.17	56.46	96.52	135.04	81.31	2239.26
-89.65	0.03	0.09	0.25	0.87	2.38	8.36	22.00	57.67	97.13	133.79	86.40	2239.26
-89.55	0.04	0.10	0.29	0.83	2.45	8.32	21.54	52.38	98.27	131.54	83.08	2239.26
-89.45	0.02	0.05	0.27	0.92	2.42	7.62	23.78	50.72	97.40	129.23	79.43	2239.26
-89.35	0.01	0.10	0.26	1.01	2.62	8.57	21.71	57.21	101.41	131.83	82.25	2239.26
-89.25	0.03	0.11	0.25	0.89	2.47	7.89	20.64	53.89	98.12	129.76	86.29	2239.26
-89.15	0.03	0.05	0.25	0.99	2.50	7.70	20.97	57.67	97.58	131.83	85.30	2239.26
-89.05	0.03	0.16	0.30	0.92	2.85	8.25	22.05	57.67	99.15	132.78	86.13	2239.26
-88.95	0.03	0.15	0.28	0.90	2.43	7.53	21.97	53.74	98.82	135.45	79.48	2239.26
-88.85	0.02	0.08	0.26	0.95	2.68	7.70	22.97	56.31	101.20	130.41	82.36	2239.26
-88.75	0.02	0.07	0.12	1.02	2.47	7.53	21.97	57.67	99.45	131.18	80.43	2239.26
-88.65	0.03	0.06	0.27	0.95	2.20	8.09	22.15	59.78	97.70	131.83	80.54	2239.26
-88.55	0.04	0.07	0.21	0.93	3.07	8.16	22.51	46.34	100.99	135.27	82.25	2239.26
-88.45	0.02	0.04	0.26	0.85	2.68	7.70	20.95	55.10	98.24	130.83	77.77	2239.26
-88.35	0.02	0.07	0.25	0.99	2.67	8.62	21.87	57.06	95.95	133.61	87.90	2239.26
-88.25	0.03	0.08	0.16	0.98	2.55	7.78	22.38	52.68	100.21	142.57	81.64	2239.26
-88.15	0.02	0.10	0.25	0.91	2.47	7.64	22.20	54.04	97.01	130.77	87.23	2239.26
-88.05	0.01	0.13	0.32	0.99	2.26	8.12	21.97	53.29	102.14	132.37	1742.40	2239.26
-87.95	0.02	0.08	0.28	0.87	2.43	8.39	22.23	56.16	95.35	139.90	81.20	2239.26
-87.85	0.03	0.10	0.28	0.95	2.38	8.21	21.66	56.91	98.27	132.55	81.09	2239.26
-87.75	0.04	0.05	0.24	0.81	2.33	8.02	23.02	53.29	102.98	135.16	82.58	2239.26
-87.65	0.01	0.12	0.23	0.95	2.62	8.82	22.79	53.89	99.24	132.67	82.14	2239.26
-87.55	0.01	0.13	0.25	1.09	2.85	8.46	21.02	53.44	99.09	139.07	85.57	2239.26
-87.45	0.03	0.07	0.22	0.88	2.33	8.41	22.51	53.59	102.47	137.59	78.21	2239.26
-87.35	0.04	0.10	0.25	0.91	2.87	8.46	22.25	59.18	99.87	133.91	81.59	2239.26
-87.25	0.02	0.09	0.22	0.90	2.60	8.43	22.12	59.02	102.83	137.88	80.65	2239.26
-87.15	0.01	0.07	0.22	0.85	2.30	8.03	23.38	58.12	98.85	135.45	82.20	2239.26
-87.05	0.01	0.16	0.36	0.95	2.62	8.18	22.23	58.27	99.15	140.20	1741.68	2239.26
-86.95	0.03	0.11	0.25	0.77	2.60	8.32	23.55	59.02	98.03	135.75	75.72	2239.26
-86.85	0.00	0.12	0.28	0.97	2.55	8.11	21.71	58.87	102.35	130.29	83.69	2239.26
-86.75	0.03	0.05	0.23	0.95	2.77	8.36	21.89	53.44	97.97	135.27	76.94	2239.26
-86.65	0.03	0.09	0.28	0.99	2.40	8.95	22.02	60.08	99.00	136.88	84.85	2239.26
-86.55	0.06	0.10	0.26	0.97	2.58	8.59	22.79	57.97	101.93	132.61	1741.96	2239.26
-86.45	0.04	0.12	0.30	0.95	2.75	8.32	22.86	57.36	100.81	132.49	74.67	2239.26
-86.35	0.03	0.07	0.24	0.94	2.57	8.11	21.87	56.31	101.35	135.51	77.10	2239.26
-86.25	0.02	0.09	0.27	0.86	2.77	7.91	22.84	57.67	102.38	142.81	75.67	2239.26
-86.15	0.03	0.08	0.25	0.94	2.82	8.36	22.43	55.55	99.54	139.78	77.60	2239.26
-86.05	0.03	0.10	0.25	0.96	2.65	8.23	22.02	57.21	98.79	136.99	78.27	2239.26

Midpt.	11000	2700	910	240	84	26	8.8	2.7	0.92	0.31	0.05	Volume
-85.95	0.03	0.07	0.27	0.90	2.67	8.21	22.51	56.01	100.54	139.49	77.38	2239.26
-85.85	0.03	0.05	0.21	0.96	2.40	8.11	22.07	52.68	99.69	133.85	77.33	2239.26
-85.75	0.02	0.07	0.29	1.13	2.33	8.45	23.45	54.95	100.96	134.56	73.89	2239.26
-85.65	0.03	0.05	0.22	1.08	2.73	7.52	22.61	55.70	101.47	132.61	80.59	2239.26
-85.55	0.00	0.11	0.30	1.01	2.47	8.91	23.40	60.69	101.02	131.00	76.66	2239.26
-85.45	0.03	0.12	0.27	0.93	2.38	8.07	23.32	47.40	102.08	138.60	76.94	2239.26
-85.35	0.04	0.10	0.28	0.73	2.55	8.80	23.58	56.76	102.35	138.30	79.93	2239.26
-85.25	0.04	0.06	0.25	0.95	2.65	9.20	21.18	55.10	100.90	136.99	74.17	2239.26
-85.15	0.03	0.06	0.26	0.95	3.10	8.20	23.27	52.08	100.69	138.24	79.48	2239.26
-85.05	0.03	0.15	0.22	0.99	2.55	8.48	22.23	56.16	100.66	137.94	81.86	2239.26
-84.95	0.03	0.11	0.28	0.95	2.06	7.98	23.12	51.17	100.18	141.50	80.48	2239.26
-84.85	0.06	0.13	0.26	0.69	2.73	8.03	22.43	62.19	104.64	135.57	1737.09	2239.26
-84.75	0.05	0.11	0.15	0.91	3.04	8.09	22.58	57.06	105.43	138.83	75.17	2239.26
-84.65	0.01	0.11	0.35	0.94	2.53	8.50	23.09	57.97	101.38	138.24	81.92	2239.26
-84.55	0.03	0.02	0.22	0.90	2.60	9.75	22.61	58.72	100.96	140.73	77.33	2239.26
-84.45	0.05	0.08	0.31	0.99	2.57	8.68	22.51	54.50	100.21	132.67	1735.15	2239.26
-84.35	0.03	0.13	0.25	1.02	2.70	8.39	23.43	56.91	101.11	139.49	74.67	2239.26
-84.25	0.04	0.11	0.17	0.97	2.58	8.45	22.81	56.76	100.48	135.10	75.67	2239.26
-84.15	0.05	0.10	0.22	1.09	2.38	8.09	21.92	59.48	101.53	140.61	78.16	2239.26
-84.05	0.04	0.10	0.28	1.02	2.67	8.55	21.59	59.48	101.17	138.30	82.09	2239.26
-83.95	0.02	0.14	0.31	0.99	2.87	8.73	23.55	57.36	101.05	136.82	74.06	2239.26
-83.85	0.03	0.12	0.25	0.86	2.75	9.00	23.22	52.84	99.06	136.88	79.76	2239.26
-83.75	0.03	0.04	0.28	1.06	2.63	8.61	22.66	58.72	100.54	139.60	76.77	2239.26
-83.65	0.03	0.09	0.23	0.83	2.73	8.37	23.02	57.82	102.50	140.79	74.39	2239.26
-83.55	0.04	0.12	0.25	0.99	2.78	8.82	23.76	56.46	104.01	141.44	76.33	2239.26
-83.45	0.02	0.09	0.27	0.90	2.82	8.12	22.38	59.02	101.26	137.53	75.17	2239.26
-83.35	0.07	0.11	0.29	1.05	2.87	7.93	22.61	57.97	101.29	138.30	77.44	2239.26
-83.25	0.01	0.07	0.34	0.96	2.67	8.82	22.48	59.02	101.84	135.99	75.83	2239.26
-83.15	0.02	0.09	0.23	1.07	2.67	8.02	22.00	56.31	100.57	135.63	73.67	2239.26
-83.05	0.01	0.05	0.28	0.99	2.88	9.16	22.28	58.72	103.01	141.92	76.27	2239.26
-82.95	0.03	0.11	0.36	1.13	2.73	8.30	23.40	58.12	100.02	135.99	75.28	2239.26
-82.85	0.00	0.05	0.24	1.09	2.47	8.28	23.43	64.91	98.09	135.87	73.51	2239.26
-82.75	0.02	0.08	0.25	1.11	2.33	8.80	23.43	52.53	101.35	130.17	73.62	2239.26
-82.65	0.06	0.09	0.18	0.95	2.57	8.75	21.77	56.01	100.30	137.94	1736.54	2239.26
-82.55	0.00	0.09	0.28	0.99	2.83	8.55	24.27	59.33	100.24	132.55	77.27	2239.26
-82.45	0.03	0.13	0.34	0.95	2.62	8.36	22.76	60.23	102.14	143.40	78.88	2239.26
-82.35	0.00	0.11	0.32	1.04	2.68	7.82	22.74	55.10	102.26	137.05	74.83	2239.26
-82.25	0.03	0.07	0.22	1.00	2.99	8.46	23.15	57.67	97.76	139.96	71.29	2239.26
-82.15	0.01	0.07	0.32	0.86	2.70	8.48	23.91	55.10	103.65	142.98	76.22	2239.26
-82.05	0.00	0.07	0.24	0.86	2.82	7.95	23.32	59.78	101.93	136.94	70.19	2239.26
-81.95	0.05	0.07	0.25	0.79	2.85	8.62	20.97	53.29	100.72	139.54	1735.10	2239.26
-81.85	0.05	0.09	0.27	1.09	2.53	9.02	21.23	56.76	99.54	135.51	73.67	2239.26
-81.75	0.03	0.07	0.27	1.03	2.77	8.84	22.12	53.74	99.33	134.27	1737.87	2239.26
-81.65	0.01	0.11	0.26	0.91	2.63	8.68	21.82	63.55	99.63	136.94	78.93	2239.26
-81.55	0.01	0.10	0.25	1.07	2.78	8.28	23.76	57.67	99.48	136.88	75.39	2239.26

Midpt.	11000	2700	910	240	84	26	8.8	2.7	0.92	0.31	0.05	Volume
-81.45	0.02	0.07	0.25	1.00	2.50	8.84	23.78	63.10	100.36	140.14	1734.10	2239.26
-81.35	0.02	0.10	0.26	1.02	2.94	8.71	22.15	52.53	102.41	135.99	1731.61	2239.26
-81.25	0.02	0.11	0.26	0.90	2.62	8.91	22.10	62.19	98.36	137.77	75.89	2239.26
-81.15	0.03	0.08	0.28	1.00	2.70	8.50	22.89	65.82	102.89	134.15	73.01	2239.26
-81.05	0.03	0.07	0.31	1.09	2.52	8.52	22.86	53.44	99.63	134.50	71.90	2239.26
-80.95	0.01	0.12	0.28	1.08	2.80	8.09	23.20	56.46	98.33	133.67	71.90	2239.26
-80.85	0.03	0.11	0.31	0.94	2.60	8.27	22.84	54.19	100.42	136.58	1732.94	2239.26
-80.75	0.05	0.11	0.23	0.92	2.75	8.64	23.15	56.31	101.35	133.85	74.39	2239.26
-80.65	0.03	0.14	0.23	0.97	2.57	8.77	23.17	50.42	102.41	137.35	64.48	2239.26
-80.55	0.02	0.07	0.17	0.95	2.58	8.70	22.17	54.50	97.79	142.63	78.10	2239.26
-80.45	0.03	0.07	0.28	0.99	3.09	8.02	22.02	59.02	103.77	133.91	73.78	2239.26
-80.35	0.03	0.05	0.30	1.14	3.14	8.25	21.41	59.18	98.42	135.63	76.27	2239.26
-80.25	0.02	0.10	0.25	0.89	3.00	8.46	22.58	58.57	105.70	133.67	75.17	2239.26
-80.15	0.06	0.11	0.32	0.97	3.07	8.21	23.38	58.27	102.14	130.47	71.46	2239.26
-80.05	0.01	0.12	0.27	1.12	2.70	8.23	22.58	59.93	100.84	137.88	72.29	2239.26
-79.95	0.01	0.05	0.29	0.81	2.42	8.45	24.07	54.80	101.23	135.81	70.35	2239.26
-79.85	0.04	0.10	0.30	0.99	2.62	8.27	23.73	60.38	101.69	135.16	71.13	2239.26
-79.75	0.04	0.08	0.25	0.93	2.83	9.32	21.46	56.16	101.14	133.97	71.79	2239.26
-79.65	0.03	0.10	0.19	0.93	2.58	8.28	22.89	62.04	103.41	133.97	74.12	2239.26
-79.55	0.03	0.05	0.29	0.88	2.33	8.66	23.27	58.12	103.62	135.69	1734.77	2239.26
-79.45	0.02	0.09	0.29	1.04	2.75	8.09	22.40	52.23	100.72	137.47	72.34	2239.26
-79.35	0.03	0.08	0.22	1.04	2.90	8.18	23.55	57.06	100.96	135.39	76.99	2239.26
-79.25	0.03	0.07	0.32	1.00	2.48	9.28	23.22	59.02	96.98	136.58	70.07	2239.26
-79.15	0.02	0.07	0.28	0.90	3.04	8.16	21.59	56.31	100.63	136.88	73.23	2239.26
-79.05	0.03	0.13	0.38	0.88	2.62	8.68	23.30	55.85	102.35	135.22	77.60	2239.26
-78.95	0.02	0.12	0.28	0.89	2.75	8.02	22.15	52.23	102.86	134.80	76.27	2239.26
-78.85	0.03	0.13	0.25	0.88	2.80	8.57	22.76	60.84	99.39	138.66	70.85	2239.26
-78.75	0.00	0.14	0.22	0.99	2.58	8.89	22.97	56.16	99.60	139.25	75.89	2239.26
-78.65	0.04	0.16	0.25	1.07	2.62	8.09	23.32	61.29	98.88	125.67	72.57	2239.26
-78.55	0.02	0.07	0.15	1.13	2.92	8.21	22.69	55.85	102.86	135.33	73.17	2239.26
-78.45	0.02	0.08	0.30	1.04	2.72	7.95	21.89	51.33	102.05	138.30	72.79	2239.26
-78.35	0.03	0.10	0.23	0.99	2.85	8.68	23.61	52.38	100.12	136.05	72.07	2239.26
-78.25	0.02	0.11	0.32	0.99	2.57	8.73	22.28	56.16	99.12	134.21	68.30	2239.26
-78.15	0.03	0.05	0.24	0.85	2.52	7.89	22.56	54.19	101.56	135.99	71.90	2239.26
-78.05	0.03	0.11	0.27	1.09	2.94	8.21	23.30	54.65	104.28	142.21	70.02	2239.26
-77.95	0.02	0.09	0.22	0.90	2.50	8.68	23.73	54.04	100.99	133.50	1731.89	2239.26
-77.85	0.01	0.11	0.28	1.04	2.63	8.50	23.61	54.65	102.35	132.13	73.51	2239.26
-77.75	0.03	0.10	0.27	0.95	2.83	9.14	23.22	57.97	100.63	137.53	71.02	2239.26
-77.65	0.01	0.03	0.30	0.93	2.57	8.07	22.56	54.95	100.93	129.46	69.80	2239.26
-77.55	0.03	0.11	0.28	0.95	2.33	8.43	23.66	55.40	102.71	137.53	71.79	2239.26
-77.45	0.03	0.10	0.27	1.07	2.62	9.05	22.48	62.04	103.74	138.60	70.35	2239.26
-77.35	0.03	0.08	0.31	0.95	2.70	8.61	21.77	56.46	102.50	136.82	72.23	2239.26
-77.25	0.01	0.14	0.27	1.04	2.83	7.68	23.07	56.46	101.08	135.22	69.02	2239.26
-77.15	0.01	0.08	0.26	0.93	2.62	8.95	24.73	56.61	103.77	136.10	70.96	2239.26
-77.05	0.02	0.12	0.24	0.97	2.94	7.87	22.94	54.34	102.83	143.04	71.79	2239.26

Midpt.	11000	2700	910	240	84	26	8.8	2.7	0.92	0.31	0.05	Volume
-76.95	0.03	0.11	0.26	0.92	2.63	8.86	21.43	55.55	100.90	138.12	74.17	2239.26
-76.85	0.03	0.05	0.25	0.93	2.94	8.34	22.81	56.91	99.30	131.60	66.03	2239.26
-76.75	0.01	0.08	0.22	0.91	2.92	7.98	23.38	56.31	99.69	140.08	67.25	2239.26
-76.65	0.02	0.06	0.23	1.01	2.42	8.14	22.15	57.52	102.86	141.80	69.13	2239.26
-76.55	0.03	0.08	0.27	0.90	2.92	8.27	22.92	56.76	99.96	136.22	72.68	2239.26
-76.45	0.03	0.09	0.25	0.91	3.10	8.39	22.92	61.59	101.23	137.35	68.91	2239.26
-76.35	0.00	0.10	0.28	1.10	2.82	8.66	23.58	55.70	100.54	137.05	68.52	2239.26
-76.25	0.04	0.05	0.26	1.02	2.62	8.96	22.56	59.33	102.44	135.57	70.19	2239.26
-76.15	0.03	0.10	0.24	0.99	2.72	7.77	22.92	62.95	99.66	132.72	66.31	2239.26
-76.05	0.01	0.10	0.34	1.06	2.90	9.30	23.25	56.46	100.84	132.78	1728.90	2239.26
-75.95	0.06	0.11	0.31	1.09	2.60	8.43	23.66	57.21	102.44	136.76	71.46	2239.26
-75.85	0.01	0.08	0.30	0.90	2.68	8.98	22.35	54.80	103.74	136.99	71.29	2239.26
-75.75	0.02	0.11	0.38	0.99	2.68	8.36	23.40	56.46	99.60	142.15	71.74	2239.26
-75.65	0.01	0.07	0.22	0.88	2.50	8.36	22.81	56.31	99.72	136.52	71.29	2239.26
-75.55	0.00	0.09	0.22	0.99	2.78	8.82	24.07	51.63	98.79	138.00	71.46	2239.26
-75.45	0.01	0.07	0.28	0.87	2.62	8.46	23.12	61.44	101.05	136.34	70.30	2239.26
-75.35	0.03	0.06	0.23	0.93	2.90	8.53	22.79	57.97	100.99	137.94	1729.84	2239.26
-75.25	0.01	0.11	0.27	0.78	2.82	8.70	23.50	53.74	99.93	135.69	75.33	2239.26
-75.15	0.03	0.07	0.25	1.00	2.85	7.84	23.73	54.50	102.14	143.04	68.25	2239.26
-75.05	0.04	0.09	0.27	0.92	2.10	8.87	22.97	58.12	97.40	134.21	74.45	2239.26
-74.95	0.03	0.11	0.26	0.85	2.77	8.43	22.69	58.27	100.90	135.69	69.80	2239.26
-74.85	0.02	0.09	0.26	0.94	2.90	8.55	22.58	60.69	100.84	138.24	71.79	2239.26
-74.75	0.01	0.07	0.23	1.01	2.68	8.48	22.12	62.04	100.96	139.60	68.47	2239.26
-74.65	0.04	0.10	0.23	1.06	2.75	7.87	22.46	53.59	103.50	132.67	68.75	2239.26
-74.55	0.03	0.04	0.27	1.06	2.45	7.64	22.61	60.69	101.63	141.44	68.75	2239.26
-74.45	0.03	0.05	0.25	0.98	2.57	8.32	23.20	59.63	103.01	130.29	71.46	2239.26
-74.35	0.04	0.11	0.18	1.07	2.77	8.59	23.17	55.40	100.18	141.38	72.07	2239.26
-74.25	0.03	0.09	0.21	0.83	2.57	8.00	21.51	60.84	102.59	141.74	73.45	2239.26
-74.15	0.02	0.14	0.21	0.84	2.92	8.75	22.99	52.99	103.92	127.33	67.25	2239.26
-74.05	0.03	0.09	0.27	0.85	2.83	8.02	24.07	58.87	99.93	136.70	67.09	2239.26
-73.95	0.06	0.07	0.22	1.00	2.58	8.89	22.71	56.16	102.26	136.28	69.69	2239.26
-73.85	0.03	0.11	0.25	1.09	2.73	8.27	22.30	59.02	102.65	135.99	72.90	2239.26
-73.75	0.02	0.11	0.26	1.08	2.55	8.20	22.58	54.50	101.11	140.97	71.02	2239.26
-73.65	0.03	0.08	0.22	1.09	2.58	8.57	23.12	55.85	96.55	134.86	67.14	2239.26
-73.55	0.04	0.08	0.33	1.11	2.58	8.37	22.63	60.38	101.20	139.19	67.09	2239.26
-73.45	0.03	0.15	0.24	1.04	2.50	9.25	22.94	55.10	98.91	139.07	69.63	2239.26
-73.35	0.03	0.11	0.25	1.11	2.67	8.53	24.07	55.25	100.93	137.23	68.91	2239.26
-73.25	0.00	0.07	0.18	1.04	2.94	8.50	22.33	57.21	102.05	140.02	70.79	2239.26
-73.15	0.03	0.07	0.27	0.95	2.23	9.02	24.29	55.55	102.20	131.00	71.74	2239.26
-73.05	0.03	0.13	0.26	1.08	2.75	8.12	24.29	59.78	102.23	135.93	70.46	2239.26
-72.95	0.02	0.07	0.22	0.88	2.75	8.80	22.28	56.91	100.99	138.71	72.45	2239.26
-72.85	0.04	0.15	0.23	0.90	2.67	8.59	22.51	61.29	101.78	139.96	68.14	2239.26
-72.75	0.03	0.09	0.32	0.92	2.70	8.46	22.00	57.52	102.14	143.28	71.79	2239.26
-72.65	0.01	0.11	0.22	0.99	2.60	8.50	22.86	57.06	101.84	140.43	66.48	2239.26
-72.55	0.00	0.07	0.32	0.95	2.58	8.20	22.71	56.46	97.01	139.90	68.30	2239.26

Midpt.	11000	2700	910	240	84	26	8.8	2.7	0.92	0.31	0.05	Volume
-72.45	0.01	0.11	0.31	1.04	2.55	9.23	24.75	59.02	103.86	136.76	73.56	2239.26
-72.35	0.01	0.10	0.22	0.92	2.47	8.59	23.68	53.44	102.08	138.00	65.65	2239.26
-72.25	0.02	0.10	0.25	0.96	2.52	8.68	24.42	54.65	108.27	131.12	72.90	2239.26
-72.15	0.03	0.10	0.25	1.14	3.07	9.52	22.28	54.50	101.11	139.31	71.68	2239.26
-72.05	0.03	0.14	0.21	0.90	3.02	8.41	22.74	58.87	100.81	138.77	1731.39	2239.26
-71.95	0.02	0.13	0.22	0.95	2.73	9.05	22.48	55.25	103.47	136.16	70.85	2239.26
-71.85	0.02	0.07	0.24	0.98	2.20	8.43	23.50	58.72	101.26	138.89	66.31	2239.26
-71.75	0.02	0.10	0.26	0.85	2.83	9.39	23.22	53.89	100.02	130.12	68.03	2239.26
-71.65	0.02	0.12	0.22	1.03	2.42	8.91	23.04	57.82	102.29	133.91	66.70	2239.26
-71.55	0.01	0.07	0.25	1.00	2.65	8.75	22.92	59.93	98.97	140.61	73.40	2239.26
-71.45	0.00	0.15	0.32	1.09	2.97	8.84	22.97	56.01	104.43	140.73	64.76	2239.26
-71.35	0.04	0.07	0.31	0.97	2.33	9.07	24.65	54.50	102.56	140.97	67.42	2239.26
-71.25	0.03	0.04	0.35	1.00	2.92	9.37	23.50	53.14	102.71	133.61	73.56	2239.26
-71.15	0.04	0.06	0.28	1.07	3.17	8.34	22.38	58.72	104.40	133.85	65.54	2239.26
-71.05	0.01	0.10	0.26	0.97	2.65	8.57	22.46	64.76	105.52	142.15	69.69	2239.26
-70.95	0.01	0.14	0.22	0.99	2.68	8.71	23.30	55.10	97.43	139.90	70.19	2239.26
-70.85	0.03	0.11	0.34	0.99	2.63	8.37	22.10	58.42	102.08	135.63	73.34	2239.26
-70.75	0.06	0.07	0.30	1.04	2.58	8.82	23.40	58.72	100.12	134.50	72.29	2239.26
-70.65	0.03	0.12	0.15	1.00	2.83	8.80	23.48	57.36	100.09	135.51	68.03	2239.26
-70.55	0.02	0.12	0.27	1.09	2.94	8.14	23.32	58.27	101.59	133.55	69.96	2239.26
-70.45	0.01	0.10	0.27	1.06	2.88	7.98	22.17	56.46	101.96	137.94	72.01	2239.26
-70.35	0.02	0.10	0.35	1.01	2.72	8.80	23.91	60.53	100.84	144.88	72.07	2239.26
-70.25	0.03	0.12	0.25	1.05	2.75	8.61	22.89	55.55	99.24	136.99	71.29	2239.26
-70.15	0.03	0.10	0.28	1.08	2.35	8.09	22.97	57.82	104.13	135.04	69.58	2239.26
-70.05	0.01	0.10	0.23	0.95	2.63	8.59	22.38	57.36	102.74	133.44	72.84	2239.26
-69.95	0.03	0.07	0.24	1.02	2.97	8.43	22.99	58.87	100.51	137.11	68.97	2239.26
-69.85	0.02	0.07	0.23	1.02	2.63	8.59	22.35	56.91	99.69	142.63	69.91	2239.26
-69.75	0.01	0.13	0.31	0.81	2.94	8.02	23.43	61.44	99.18	142.63	1730.89	2239.26
-69.65	0.03	0.06	0.38	1.06	2.60	8.32	21.18	62.19	101.50	130.89	73.01	2239.26
-69.55	0.03	0.09	0.30	1.02	2.78	8.09	22.58	59.78	101.38	138.54	69.08	2239.26
-69.45	0.02	0.10	0.28	0.96	2.95	8.75	23.86	56.01	100.42	143.28	69.47	2239.26
-69.35	0.01	0.06	0.27	1.14	2.38	8.80	22.71	55.85	102.05	135.87	69.24	2239.26
-69.25	0.00	0.10	0.26	1.02	2.50	7.80	22.43	60.99	98.06	131.66	72.34	2239.26
-69.15	0.03	0.06	0.25	0.83	2.67	8.18	22.66	61.44	101.56	137.11	1730.78	2239.26
-69.05	0.01	0.08	0.17	0.92	2.82	8.03	22.97	59.02	99.45	136.64	68.80	2239.26
-68.95	0.03	0.11	0.20	0.93	2.99	8.98	22.40	59.78	101.29	136.64	68.14	2239.26
-68.85	0.05	0.10	0.20	1.09	2.90	9.20	22.66	58.12	99.78	127.62	70.63	2239.26
-68.75	0.01	0.12	0.23	1.26	2.68	8.52	24.14	54.19	100.96	137.71	65.20	2239.26
-68.65	0.02	0.11	0.26	1.02	2.57	9.05	23.68	61.44	99.39	136.46	70.91	2239.26
-68.55	0.01	0.07	0.23	1.11	2.72	9.09	24.24	60.23	101.93	150.28	69.69	2239.26
-68.45	0.02	0.10	0.22	0.95	2.90	8.75	22.23	51.48	100.72	138.54	69.74	2239.26
-68.35	0.03	0.15	0.29	1.03	2.60	8.50	22.35	61.44	101.02	137.23	67.47	2239.26
-68.25	0.01	0.10	0.22	1.30	2.94	8.64	23.99	56.31	100.51	138.60	69.19	2239.26
-68.15	0.03	0.05	0.35	1.04	2.92	8.86	24.24	56.46	103.01	134.92	72.95	2239.26
-68.05	0.04	0.14	0.27	1.10	2.97	8.45	22.56	55.40	101.29	138.95	69.69	2239.26

Midpt.	11000	2700	910	240	84	26	8.8	2.7	0.92	0.31	0.05	Volume
-67.95	0.05	0.04	0.31	0.97	3.14	8.41	23.81	60.08	99.93	143.04	67.53	2239.26
-67.85	0.03	0.10	0.28	1.04	2.77	8.70	23.78	59.02	99.24	132.78	76.77	2239.26
-67.75	0.02	0.09	0.25	0.93	3.20	8.36	24.91	60.08	101.78	132.25	72.62	2239.26
-67.65	0.03	0.09	0.32	0.76	2.55	8.75	24.04	59.78	99.24	140.26	69.02	2239.26
-67.55	0.03	0.11	0.37	0.77	3.00	8.64	22.38	58.12	100.81	130.35	1730.12	2239.26
-67.45	0.06	0.16	0.25	0.97	2.55	8.77	23.15	61.89	101.90	135.51	68.14	2239.26
-67.35	0.03	0.10	0.25	0.90	2.43	8.66	23.43	55.40	101.17	133.85	70.19	2239.26
-67.25	0.02	0.11	0.32	1.11	3.12	8.14	22.58	56.76	101.63	137.71	69.36	2239.26
-67.15	0.01	0.10	0.25	1.08	2.83	9.05	23.68	54.19	103.10	137.59	68.25	2239.26
-67.05	0.03	0.08	0.27	0.86	2.08	8.02	23.22	57.67	100.06	139.37	1730.95	2239.26
-66.95	0.01	0.07	0.22	1.05	2.83	8.98	23.68	59.48	98.36	131.18	71.02	2239.26
-66.85	0.00	0.10	0.32	0.95	2.97	8.41	25.06	51.48	100.18	134.33	65.87	2239.26
-66.75	0.03	0.10	0.19	0.86	2.77	8.59	22.20	57.52	100.72	137.82	68.80	2239.26
-66.65	0.03	0.12	0.25	0.85	2.72	8.57	22.38	57.21	102.74	140.49	69.63	2239.26
-66.55	0.03	0.07	0.31	0.92	2.67	8.64	21.94	54.50	101.63	138.60	71.51	2239.26
-66.45	0.06	0.07	0.25	1.07	2.82	8.46	22.69	56.01	103.83	129.28	65.09	2239.26
-66.35	0.03	0.11	0.19	1.02	2.68	8.34	22.00	59.18	102.26	140.97	71.46	2239.26
-66.25	0.02	0.08	0.22	0.99	2.77	8.46	22.46	59.02	99.69	139.31	1732.39	2239.26
-66.15	0.03	0.06	0.27	0.84	3.17	8.41	23.76	56.61	102.83	130.65	71.02	2239.26
-66.05	0.00	0.06	0.29	1.04	2.94	8.16	22.02	56.46	100.24	132.13	1731.89	2239.26
-65.95	0.01	0.07	0.30	0.99	2.90	9.09	22.76	55.55	99.21	134.68	68.80	2239.26
-65.85	0.01	0.11	0.29	0.77	2.67	8.05	23.50	59.33	101.50	136.40	72.90	2239.26
-65.75	0.03	0.07	0.22	1.06	2.92	8.68	24.45	57.06	98.15	137.94	72.57	2239.26
-65.65	0.04	0.11	0.28	0.95	2.11	8.89	22.99	60.23	104.58	140.97	67.58	2239.26
-65.55	0.02	0.07	0.27	0.98	2.77	8.39	21.89	61.74	102.68	136.52	72.51	2239.26
-65.45	0.02	0.08	0.22	0.97	2.97	8.18	23.04	58.72	104.25	129.58	1730.72	2239.26
-65.35	0.03	0.10	0.22	1.00	2.94	8.36	22.74	59.63	102.05	137.71	70.30	2239.26
-65.25	0.03	0.07	0.25	0.85	3.25	8.96	23.22	59.48	99.30	136.76	69.30	2239.26
-65.15	0.03	0.07	0.23	0.98	2.15	9.34	22.02	58.57	98.91	130.35	67.09	2239.26
-65.05	0.00	0.07	0.33	0.98	2.75	8.86	21.59	51.33	102.47	133.14	72.95	2239.26
-64.95	0.02	0.08	0.20	1.00	2.73	8.25	21.79	61.29	101.81	142.98	72.84	2239.26
-64.85	0.03	0.10	0.18	0.92	2.77	8.96	22.89	58.42	104.89	136.40	72.62	2239.26
-64.75	0.06	0.09	0.25	1.09	3.07	8.28	24.19	55.55	99.18	133.08	73.17	2239.26
-64.65	0.04	0.09	0.32	0.95	2.47	8.25	23.32	57.52	98.82	144.53	72.23	2239.26
-64.55	0.03	0.11	0.23	0.92	2.50	8.75	23.76	55.70	99.87	139.72	69.80	2239.26
-64.45	0.03	0.12	0.23	1.02	2.35	9.21	22.84	55.25	101.78	129.05	70.85	2239.26
-64.35	0.03	0.11	0.19	0.98	3.02	8.78	23.99	59.63	101.69	135.45	67.86	2239.26
-64.25	0.03	0.07	0.28	1.14	2.87	8.55	21.18	62.50	101.59	138.24	73.51	2239.26
-64.15	0.04	0.11	0.32	0.90	2.62	8.37	22.63	59.78	101.72	138.48	74.17	2239.26
-64.05	0.03	0.06	0.26	1.03	2.62	8.43	22.56	59.78	102.23	135.99	71.57	2239.26
-63.95	0.01	0.06	0.34	0.94	2.68	8.27	22.86	56.46	105.73	133.26	73.56	2239.26
-63.85	0.05	0.11	0.22	1.08	2.68	8.64	25.04	55.10	104.13	133.08	73.29	2239.26
-63.75	0.03	0.11	0.21	1.04	2.97	9.28	23.78	55.25	101.32	135.99	72.57	2239.26
-63.65	0.03	0.10	0.23	1.03	2.75	8.02	23.55	56.16	101.84	136.64	74.28	2239.26
-63.55	0.01	0.08	0.30	0.99	2.82	9.20	22.79	54.65	102.35	129.40	1737.37	2239.26

Midpt.	11000	2700	910	240	84	26	8.8	2.7	0.92	0.31	0.05	Volume
-63.45	0.02	0.09	0.25	0.92	2.88	9.28	21.74	59.78	100.69	131.72	70.57	2239.26
-63.35	0.01	0.09	0.26	1.02	2.47	8.78	23.07	51.93	100.30	129.88	69.30	2239.26
-63.25	0.03	0.07	0.32	0.92	2.92	8.62	24.32	56.16	100.30	136.99	74.23	2239.26
-63.15	0.03	0.10	0.35	0.93	2.68	8.61	23.61	56.76	102.02	134.33	73.01	2239.26
-63.05	0.03	0.05	0.31	0.94	2.68	8.50	21.79	62.19	99.63	140.91	73.40	2239.26
-62.95	0.02	0.05	0.28	0.89	2.78	8.48	22.81	54.65	102.86	135.45	1738.53	2239.26
-62.85	0.03	0.11	0.23	0.93	2.88	9.36	23.63	55.85	101.78	131.72	72.45	2239.26
-62.75	0.01	0.07	0.28	0.85	2.68	8.80	22.53	57.82	98.76	133.50	71.51	2239.26
-62.65	0.03	0.09	0.29	0.82	2.88	10.02	22.92	54.19	101.81	138.00	1736.20	2239.26
-62.55	0.02	0.07	0.20	1.01	2.80	8.75	22.94	51.48	105.73	137.47	75.67	2239.26
-62.45	0.05	0.11	0.27	0.91	2.30	8.75	23.15	56.46	100.12	136.58	1734.71	2239.26
-62.35	0.02	0.08	0.26	1.01	2.77	8.43	23.48	58.42	99.39	131.95	70.57	2239.26
-62.25	0.03	0.10	0.25	0.92	2.48	8.57	24.60	61.29	102.14	142.81	70.46	2239.26
-62.15	0.04	0.07	0.27	0.99	2.47	8.96	22.89	56.46	101.32	139.60	1732.50	2239.26
-62.05	0.02	0.07	0.23	0.90	2.67	8.68	24.83	57.82	101.78	131.36	75.44	2239.26
-61.95	0.03	0.04	0.27	1.04	2.63	8.61	25.06	59.93	102.35	131.06	70.41	2239.26
-61.85	0.05	0.11	0.25	0.96	2.40	8.84	22.76	56.31	104.10	139.84	72.84	2239.26
-61.75	0.03	0.11	0.25	1.09	2.72	8.32	23.48	52.68	100.27	136.58	75.78	2239.26
-61.65	0.01	0.09	0.25	0.93	2.58	8.53	23.55	52.68	101.41	135.51	73.12	2239.26
-61.55	0.03	0.11	0.35	0.84	2.50	8.71	23.61	55.70	100.93	136.70	72.51	2239.26
-61.45	0.04	0.11	0.31	1.17	2.63	8.73	22.56	61.89	105.25	135.93	71.68	2239.26
-61.35	0.04	0.11	0.31	1.02	2.85	8.14	23.22	64.01	103.74	134.39	76.77	2239.26
-61.25	0.03	0.09	0.34	0.92	2.30	9.23	22.84	59.48	101.93	139.49	1728.57	2239.26
-61.15	0.06	0.08	0.32	1.08	2.78	8.64	24.35	56.46	101.02	141.92	76.44	2239.26
-61.05	0.02	0.10	0.25	1.16	2.55	8.86	22.84	56.31	100.78	140.20	77.27	2239.26
-60.95	0.02	0.14	0.22	1.12	2.82	9.11	22.81	54.65	102.74	138.12	73.73	2239.26
-60.85	0.02	0.10	0.28	0.95	2.87	8.16	22.84	55.10	99.48	139.72	72.90	2239.26
-60.75	0.05	0.06	0.22	0.98	2.50	8.82	22.99	59.02	101.99	141.56	73.67	2239.26
-60.65	0.02	0.10	0.26	1.04	2.88	8.68	22.61	54.19	101.96	134.15	78.88	2239.26
-60.55	0.01	0.09	0.22	0.95	2.58	8.75	22.30	54.04	103.44	136.40	74.95	2239.26
-60.45	0.01	0.17	0.36	1.00	2.25	8.64	23.17	63.25	100.36	141.26	70.57	2239.26
-60.35	0.02	0.07	0.25	0.79	3.02	8.27	23.09	56.61	105.49	137.17	77.10	2239.26
-60.25	0.01	0.07	0.32	0.84	2.52	8.59	23.02	57.97	104.22	135.27	1735.37	2239.26
-60.15	0.01	0.15	0.31	0.95	2.26	7.11	23.30	60.53	104.07	136.58	1731.89	2239.26
-60.05	0.05	0.08	0.22	1.16	2.87	8.12	22.56	53.74	101.66	135.04	75.44	2239.26
-59.95	0.03	0.11	0.25	0.99	2.67	8.87	23.81	54.65	98.79	136.58	72.34	2239.26
-59.85	0.03	0.10	0.24	0.98	2.25	8.28	24.04	55.55	101.50	136.52	70.96	2239.26
-59.75	0.03	0.07	0.19	1.05	2.68	8.82	22.51	57.36	100.75	137.94	76.44	2239.26
-59.65	0.02	0.13	0.22	0.89	2.47	8.37	23.86	60.08	100.69	139.78	74.72	2239.26
-59.55	0.02	0.12	0.21	0.92	2.21	8.64	23.12	59.78	100.51	140.55	77.55	2239.26
-59.45	0.03	0.11	0.22	0.95	2.83	8.80	22.63	58.72	102.08	138.36	68.86	2239.26
-59.35	0.02	0.14	0.24	0.89	2.83	8.61	23.96	60.99	100.87	137.47	75.00	2239.26
-59.25	0.03	0.10	0.35	0.95	2.87	9.00	25.04	54.34	104.61	139.19	73.78	2239.26
-59.15	0.04	0.13	0.33	0.89	2.33	8.46	22.46	51.78	100.02	133.97	78.93	2239.26
-59.05	0.03	0.10	0.30	0.90	3.20	8.59	23.25	57.67	99.78	134.33	81.92	2239.26

Midpt.	11000	2700	910	240	84	26	8.8	2.7	0.92	0.31	0.05	Volume
-58.95	0.02	0.12	0.22	1.01	2.88	8.66	24.35	60.38	102.23	139.19	77.05	2239.26
-58.85	0.01	0.10	0.28	0.98	3.19	8.55	22.71	51.78	102.17	137.11	76.61	2239.26
-58.75	0.04	0.11	0.25	0.86	2.67	7.87	21.59	54.80	101.26	140.61	78.76	2239.26
-58.65	0.03	0.10	0.35	0.83	2.95	8.28	23.63	53.59	102.38	131.83	75.67	2239.26
-58.55	0.01	0.08	0.37	0.90	2.50	9.23	23.02	54.95	99.99	133.26	77.38	2239.26
-58.45	0.01	0.11	0.30	0.91	3.00	8.70	23.40	63.55	100.96	141.32	77.38	2239.26
-58.35	0.03	0.08	0.19	1.23	3.10	8.45	24.19	62.80	100.81	133.73	71.46	2239.26
-58.25	0.02	0.06	0.21	1.02	3.04	9.02	23.15	55.10	102.56	134.62	75.67	2239.26
-58.15	0.01	0.11	0.28	1.04	3.37	8.53	22.38	58.57	101.44	140.20	78.76	2239.26
-58.05	0.02	0.10	0.22	1.04	2.63	8.28	22.05	55.25	100.02	130.65	75.06	2239.26
-57.95	0.03	0.10	0.35	0.89	2.50	8.66	22.30	60.23	98.70	137.35	1737.70	2239.26
-57.85	0.03	0.03	0.28	0.87	2.83	9.48	24.60	51.93	101.35	136.46	80.31	2239.26
-57.75	0.03	0.14	0.28	0.78	2.85	8.53	22.66	57.36	101.63	132.19	74.72	2239.26
-57.65	0.06	0.11	0.18	0.76	2.83	8.86	23.07	60.99	101.38	133.61	73.56	2239.26
-57.55	0.03	0.07	0.25	1.05	2.83	9.37	22.63	58.72	103.95	141.03	72.29	2239.26
-57.45	0.01	0.10	0.26	0.94	2.67	8.78	23.25	60.53	99.48	138.71	75.39	2239.26
-57.35	0.03	0.09	0.34	0.87	2.80	8.98	22.94	58.42	100.60	133.50	74.17	2239.26
-57.25	0.03	0.10	0.23	0.94	2.63	8.53	24.40	56.91	99.18	133.73	75.17	2239.26
-57.15	0.02	0.10	0.22	0.92	2.60	8.80	24.70	58.87	102.50	137.47	72.23	2239.26
-57.05	0.03	0.11	0.25	1.02	2.35	8.59	23.27	56.16	102.47	136.34	74.12	2239.26
-56.95	0.05	0.03	0.25	0.90	2.67	8.45	22.38	57.97	102.56	133.61	77.99	2239.26
-56.85	0.01	0.11	0.32	1.09	2.53	8.46	24.12	55.70	103.38	133.20	75.72	2239.26
-56.75	0.03	0.09	0.27	1.19	3.00	8.89	22.92	61.89	104.28	140.55	72.01	2239.26
-56.65	0.01	0.10	0.26	0.85	2.83	8.57	23.55	50.27	99.54	138.30	71.74	2239.26
-56.55	0.03	0.14	0.18	0.97	2.78	9.20	24.37	56.76	100.63	137.82	77.71	2239.26
-56.45	0.03	0.07	0.25	0.80	2.58	9.23	24.24	55.25	103.38	130.06	77.71	2239.26
-56.35	0.04	0.07	0.29	0.99	2.95	8.57	22.00	57.67	99.24	136.88	76.16	2239.26
-56.25	0.03	0.15	0.30	0.99	2.83	8.48	22.89	51.17	99.48	139.96	80.04	2239.26
-56.15	0.03	0.13	0.27	1.11	2.83	8.64	23.45	58.72	102.59	140.67	75.61	2239.26
-56.05	0.03	0.06	0.31	0.98	2.92	8.66	22.56	54.04	100.81	132.37	79.37	2239.26
-55.95	0.03	0.11	0.22	0.86	2.92	8.68	22.92	56.01	100.84	139.37	76.77	2239.26
-55.85	0.02	0.07	0.35	0.96	2.33	8.46	23.07	61.74	99.42	137.17	80.54	2239.26
-55.75	0.03	0.12	0.22	0.91	2.43	8.43	22.86	57.67	101.90	135.69	77.99	2239.26
-55.65	0.03	0.05	0.31	0.95	2.82	8.87	22.63	56.91	103.80	131.54	79.71	2239.26
-55.55	0.03	0.13	0.28	0.97	2.65	8.50	22.35	59.63	105.94	137.94	74.50	2239.26
-55.45	0.04	0.06	0.31	1.04	3.32	8.87	23.27	59.18	98.88	141.56	72.29	2239.26
-55.35	0.03	0.09	0.17	1.03	2.68	8.36	23.20	57.36	105.19	137.05	76.38	2239.26
-55.25	0.02	0.09	0.35	0.88	2.88	8.18	23.96	59.78	101.69	139.96	79.21	2239.26
-55.15	0.02	0.07	0.28	1.17	2.75	8.86	24.32	56.31	99.78	131.06	74.23	2239.26
-55.05	0.01	0.11	0.24	1.09	2.68	8.28	23.89	56.16	102.80	138.42	76.72	2239.26
-54.95	0.05	0.09	0.20	1.04	2.57	8.28	22.66	58.72	102.11	137.88	77.77	2239.26
-54.85	0.02	0.07	0.30	0.89	2.63	8.87	22.07	55.40	101.87	136.58	71.96	2239.26
-54.75	0.03	0.07	0.28	0.83	2.48	8.55	22.33	61.89	104.31	139.25	79.87	2239.26
-54.65	0.01	0.11	0.24	0.95	2.55	8.53	24.17	55.25	100.21	128.16	76.05	2239.26
-54.55	0.03	0.11	0.22	0.99	2.87	8.20	23.07	56.16	102.14	135.22	79.82	2239.26

Midpt.	11000	2700	910	240	84	26	8.8	2.7	0.92	0.31	0.05	Volume
-54.45	0.00	0.11	0.30	0.92	2.80	8.78	23.25	56.01	101.32	135.27	76.27	2239.26
-54.35	0.00	0.11	0.27	0.99	2.95	8.27	23.17	58.12	105.97	132.55	79.48	2239.26
-54.25	0.03	0.07	0.32	0.89	2.73	9.23	24.81	60.23	105.49	135.57	81.86	2239.26
-54.15	0.03	0.13	0.29	0.74	2.73	9.16	23.71	59.63	97.88	137.77	78.76	2239.26
-54.05	0.03	0.10	0.26	0.88	2.88	8.18	22.89	62.50	102.05	133.79	72.79	2239.26
-53.95	0.03	0.08	0.28	1.09	2.63	9.21	21.94	58.57	99.96	138.95	77.38	2239.26
-53.85	0.07	0.13	0.28	0.95	2.52	8.71	23.32	57.97	101.69	135.51	79.93	2239.26
-53.75	0.01	0.11	0.33	0.98	2.85	8.82	23.09	56.76	102.23	133.44	79.04	2239.26
-53.65	0.03	0.11	0.18	0.99	2.73	8.45	23.81	59.18	100.72	137.53	78.32	2239.26
-53.55	0.05	0.11	0.17	0.90	3.05	8.50	23.55	63.25	98.64	133.44	78.49	2239.26
-53.45	0.01	0.07	0.29	0.93	2.75	7.82	22.92	59.33	102.11	139.54	78.88	2239.26
-53.35	0.04	0.07	0.30	1.04	2.87	8.21	23.84	66.27	100.15	137.59	73.62	2239.26
-53.25	0.03	0.14	0.24	0.85	2.21	8.71	23.27	55.70	102.08	136.52	75.50	2239.26
-53.15	0.03	0.18	0.31	1.09	2.82	8.57	23.84	56.76	102.38	137.47	75.50	2239.26
-53.05	0.01	0.13	0.28	1.12	2.58	8.48	21.92	58.42	102.11	137.17	78.21	2239.26
-52.95	0.02	0.10	0.26	1.04	2.75	8.28	22.63	55.25	101.44	140.02	1739.25	2239.26
-52.85	0.01	0.06	0.21	1.03	3.12	8.57	22.66	60.99	102.47	141.50	79.60	2239.26
-52.75	0.03	0.07	0.18	0.98	2.45	8.07	22.48	53.74	100.54	137.77	79.87	2239.26
-52.65	0.01	0.06	0.25	1.02	2.43	8.11	23.66	62.50	102.86	140.26	81.86	2239.26
-52.55	0.03	0.07	0.32	0.82	2.73	8.30	23.73	60.84	101.81	131.42	77.88	2239.26
-52.45	0.00	0.11	0.22	1.03	2.70	8.87	22.58	54.80	97.82	137.59	78.10	2239.26
-52.35	0.00	0.11	0.23	0.99	2.55	8.84	23.76	56.76	103.32	139.49	76.27	2239.26
-52.25	0.05	0.14	0.30	1.14	2.67	8.32	23.58	54.65	101.17	144.05	77.16	2239.26
-52.15	0.02	0.09	0.34	0.90	2.43	8.55	24.50	60.69	103.95	135.33	76.61	2239.26
-52.05	0.03	0.11	0.29	0.84	2.94	8.23	23.71	62.95	106.52	134.98	78.49	2239.26
-51.95	0.04	0.09	0.20	0.99	3.00	8.50	22.63	53.59	102.62	139.72	75.67	2239.26
-51.85	0.01	0.07	0.30	1.01	3.00	8.62	23.76	66.12	97.88	139.31	79.37	2239.26
-51.75	0.02	0.08	0.22	1.07	2.77	8.75	22.23	58.27	100.78	136.64	73.62	2239.26
-51.65	0.03	0.11	0.28	1.09	2.83	8.57	22.81	61.59	99.18	139.60	78.10	2239.26
-51.55	0.03	0.07	0.19	0.95	3.05	8.36	23.94	55.10	101.72	138.60	79.10	2239.26
-51.45	0.04	0.10	0.33	1.01	2.58	8.37	23.45	51.93	103.80	135.04	78.54	2239.26
-51.35	0.02	0.11	0.25	1.13	2.65	8.95	22.02	56.61	99.75	138.66	75.61	2239.26
-51.25	0.03	0.11	0.25	0.95	2.42	8.48	23.02	57.21	98.94	135.87	81.53	2239.26
-51.15	0.03	0.07	0.26	0.91	2.90	8.95	23.50	56.31	103.04	138.60	80.15	2239.26
-51.05	0.02	0.11	0.24	1.12	2.53	9.07	22.38	58.57	96.19	137.59	78.10	2239.26
-50.95	0.05	0.11	0.30	0.90	3.04	8.55	21.89	50.57	100.81	141.74	81.09	2239.26
-50.85	0.04	0.09	0.32	0.96	2.83	9.05	24.14	57.36	105.04	137.77	76.77	2239.26
-50.75	0.02	0.09	0.22	1.07	2.78	9.02	22.02	56.16	104.10	141.62	1733.16	2239.26
-50.65	0.03	0.13	0.31	1.03	3.00	8.55	23.17	56.91	103.98	138.24	79.15	2239.26
-50.55	0.03	0.11	0.28	0.94	2.58	8.64	23.89	51.17	101.93	135.45	80.20	2239.26
-50.45	0.03	0.09	0.22	0.94	2.73	8.55	23.58	54.34	99.87	141.98	76.05	2239.26
-50.35	0.03	0.11	0.27	0.88	2.77	8.66	22.94	58.27	100.96	137.17	76.61	2239.26
-50.25	0.03	0.09	0.28	1.18	2.62	8.41	23.02	56.31	101.75	134.98	1738.92	2239.26
-50.15	0.05	0.10	0.22	1.05	2.87	8.41	24.65	58.42	97.55	136.76	74.00	2239.26
-50.05	0.03	0.14	0.41	1.01	2.63	8.66	23.27	57.06	99.57	134.33	75.44	2239.26

Midpt.	11000	2700	910	240	84	26	8.8	2.7	0.92	0.31	0.05	Volume
-49.95	0.03	0.11	0.25	1.04	2.77	8.52	23.40	59.78	98.00	139.31	76.66	2239.26
-49.85	0.01	0.08	0.28	0.85	3.07	8.55	23.32	56.76	103.19	136.88	81.53	2239.26
-49.75	0.05	0.08	0.28	0.93	2.77	8.14	23.73	61.59	100.72	133.08	76.05	2239.26
-49.65	0.02	0.05	0.30	1.05	3.05	8.89	24.07	58.42	103.59	138.95	79.15	2239.26
-49.55	0.05	0.10	0.28	1.13	2.68	8.36	23.32	59.63	102.53	136.28	77.44	2239.26
-49.45	0.00	0.08	0.32	0.90	2.75	8.55	22.12	56.61	105.64	132.61	75.72	2239.26
-49.35	0.02	0.09	0.22	0.95	2.99	8.05	23.43	56.16	100.51	139.84	70.07	2239.26
-49.25	0.03	0.06	0.24	0.89	2.60	8.25	22.46	55.85	102.80	138.00	75.06	2239.26
-49.15	0.04	0.06	0.28	1.12	2.90	9.18	22.05	60.38	102.50	136.22	74.12	2239.26
-49.05	0.03	0.09	0.23	0.92	3.00	8.91	21.87	60.69	105.67	130.41	71.40	2239.26
-48.95	0.02	0.07	0.22	0.94	2.88	9.28	22.66	60.53	100.69	135.69	74.17	2239.26
-48.85	0.02	0.13	0.24	0.92	3.10	8.62	24.12	54.80	102.29	133.97	77.22	2239.26
-48.75	0.04	0.11	0.32	0.97	2.57	8.89	23.50	59.18	99.66	134.92	74.17	2239.26
-48.65	0.04	0.10	0.23	0.93	2.62	8.86	25.19	60.08	101.93	134.86	70.91	2239.26
-48.55	0.02	0.07	0.25	0.86	2.73	8.21	22.74	57.06	102.32	135.75	73.23	2239.26
-48.45	0.04	0.08	0.25	1.07	2.65	8.96	24.55	58.72	106.21	136.99	77.66	2239.26
-48.35	0.04	0.10	0.24	1.14	2.85	9.03	21.92	54.04	100.51	136.46	70.91	2239.26
-48.25	0.02	0.08	0.34	0.95	2.95	8.14	21.71	55.25	99.15	140.08	71.62	2239.26
-48.15	0.02	0.12	0.33	0.85	2.88	9.53	23.35	59.93	100.63	137.94	73.62	2239.26
-48.05	0.03	0.11	0.24	0.94	2.90	7.95	23.45	50.72	99.93	140.67	76.55	2239.26
-47.95	0.04	0.11	0.20	0.83	2.97	8.80	22.43	59.18	100.75	135.69	1739.30	2239.26
-47.85	0.03	0.07	0.27	1.03	2.92	7.73	23.63	57.06	99.27	137.29	71.62	2239.26
-47.75	0.02	0.12	0.22	0.85	3.12	8.09	25.29	54.65	105.34	139.07	69.36	2239.26
-47.65	0.03	0.06	0.31	0.94	2.68	9.34	24.78	60.38	100.87	138.66	74.34	2239.26
-47.55	0.00	0.09	0.26	0.96	2.48	8.71	22.20	57.52	105.28	145.30	74.00	2239.26
-47.45	0.03	0.11	0.26	0.98	2.57	9.46	22.79	58.27	105.01	136.40	73.29	2239.26
-47.35	0.05	0.07	0.25	1.10	2.63	9.50	24.32	52.68	102.26	133.14	75.22	2239.26
-47.25	0.04	0.16	0.23	0.88	2.75	8.48	22.71	55.70	100.51	139.90	71.85	2239.26
-47.15	0.01	0.12	0.32	0.86	2.97	8.23	22.63	56.46	105.10	133.14	73.67	2239.26
-47.05	0.03	0.07	0.31	1.09	2.73	8.14	22.40	62.95	100.75	135.87	1731.06	2239.26
-46.95	0.03	0.11	0.28	0.98	3.15	8.70	23.71	51.48	101.35	142.09	74.28	2239.26
-46.85	0.04	0.12	0.32	0.93	2.67	8.23	23.45	51.63	101.84	131.06	69.08	2239.26
-46.75	0.00	0.10	0.33	0.93	2.47	8.93	21.46	57.97	99.21	141.98	75.39	2239.26
-46.65	0.02	0.07	0.22	1.08	3.07	8.21	22.58	57.21	101.20	136.16	77.88	2239.26
-46.55	0.03	0.08	0.23	0.99	3.02	9.96	23.12	56.16	101.78	136.46	74.06	2239.26
-46.45	0.03	0.11	0.39	0.99	2.88	8.87	22.25	60.38	101.63	136.46	70.35	2239.26
-46.35	0.03	0.07	0.22	0.91	2.63	8.23	22.53	51.02	100.66	136.52	72.62	2239.26
-46.25	0.03	0.12	0.35	1.18	2.30	8.07	21.79	60.38	100.24	135.87	75.11	2239.26
-46.15	0.03	0.07	0.24	0.96	2.77	8.82	23.53	52.99	102.62	138.30	78.32	2239.26
-46.05	0.03	0.12	0.23	1.09	2.62	8.89	22.38	58.72	100.39	138.30	74.28	2239.26
-45.95	0.02	0.05	0.20	1.13	2.75	8.86	23.20	61.14	98.82	135.81	73.06	2239.26
-45.85	0.04	0.16	0.25	0.97	2.85	8.68	23.09	61.89	100.39	141.50	69.96	2239.26
-45.75	0.01	0.15	0.27	0.88	2.85	8.78	22.38	57.52	98.03	138.71	71.79	2239.26
-45.65	0.03	0.09	0.31	0.89	3.02	8.03	23.76	55.70	101.11	137.35	74.95	2239.26
-45.55	0.01	0.08	0.25	1.18	2.67	8.66	22.71	57.21	105.52	131.36	68.03	2239.26

Midpt.	11000	2700	910	240	84	26	8.8	2.7	0.92	0.31	0.05	Volume
-45.45	0.03	0.06	0.24	0.99	2.90	9.05	24.93	60.08	100.42	138.71	69.08	2239.26
-45.35	0.03	0.08	0.22	0.91	2.60	8.18	24.45	60.69	99.21	137.94	73.12	2239.26
-45.25	0.02	0.15	0.25	0.87	2.63	9.34	24.42	58.12	101.14	137.41	67.69	2239.26
-45.15	0.04	0.09	0.26	0.94	2.65	8.64	23.66	59.63	102.11	140.32	1728.51	2239.26
-45.05	0.02	0.16	0.35	1.21	2.78	8.62	22.69	54.34	104.16	132.96	71.74	2239.26
-44.95	0.01	0.12	0.18	1.02	2.99	8.50	22.61	56.31	101.29	136.34	70.19	2239.26
-44.85	0.02	0.08	0.28	0.88	2.70	8.34	23.76	55.25	100.72	140.20	72.73	2239.26
-44.75	0.05	0.06	0.16	1.09	2.57	8.52	22.63	56.01	102.26	136.05	69.80	2239.26
-44.65	0.03	0.08	0.22	1.02	3.09	8.20	21.84	53.29	98.91	139.84	69.69	2239.26
-44.55	0.05	0.09	0.29	0.86	2.42	8.11	23.17	60.69	103.44	137.65	69.85	2239.26
-44.45	0.02	0.15	0.20	0.87	2.58	8.57	23.40	60.99	104.76	135.27	74.67	2239.26
-44.35	0.03	0.07	0.22	0.93	2.53	8.57	22.40	62.35	103.44	131.42	73.23	2239.26
-44.25	0.02	0.10	0.26	1.07	2.92	8.12	22.02	59.33	100.39	139.72	72.51	2239.26
-44.15	0.00	0.16	0.34	0.87	2.68	8.82	22.89	55.25	102.53	140.32	72.18	2239.26
-44.05	0.03	0.10	0.19	1.17	2.90	8.93	23.22	58.42	98.09	134.62	70.63	2239.26
-43.95	0.03	0.11	0.25	0.95	2.45	8.95	23.48	61.59	99.51	133.97	72.73	2239.26
-43.85	0.03	0.11	0.29	0.99	2.82	8.89	23.84	56.76	104.34	138.83	70.63	2239.26
-43.75	0.03	0.10	0.31	0.99	2.38	8.50	21.61	56.46	100.39	144.94	73.78	2239.26
-43.65	0.03	0.14	0.22	0.91	2.53	8.68	23.53	59.33	100.12	137.47	71.57	2239.26
-43.55	0.05	0.11	0.35	0.93	2.73	8.50	23.78	51.48	104.80	140.85	1733.60	2239.26
-43.45	0.03	0.07	0.25	0.95	2.53	8.87	23.27	57.82	103.80	140.79	1734.43	2239.26
-43.35	0.02	0.08	0.38	1.18	2.90	8.59	24.19	59.63	101.87	140.85	72.12	2239.26
-43.25	0.03	0.09	0.25	1.04	2.97	8.75	23.45	55.25	102.95	136.82	71.02	2239.26
-43.15	0.03	0.11	0.28	1.01	2.48	7.71	23.94	61.29	102.92	135.45	66.20	2239.26
-43.05	0.03	0.10	0.23	0.75	2.85	8.86	21.51	56.31	100.90	130.23	67.81	2239.26
-42.95	0.02	0.07	0.23	0.99	2.80	7.95	22.94	60.69	100.09	131.83	69.80	2239.26
-42.85	0.02	0.10	0.19	0.97	2.62	8.66	22.76	57.36	104.34	138.95	67.14	2239.26
-42.75	0.03	0.06	0.32	1.00	2.85	7.62	22.99	59.48	99.84	130.47	71.79	2239.26
-42.65	0.02	0.07	0.34	0.99	2.88	8.57	24.91	57.67	99.24	132.72	70.02	2239.26
-42.55	0.03	0.12	0.30	0.95	2.43	8.98	23.30	58.72	103.01	139.37	71.62	2239.26
-42.45	0.03	0.08	0.28	1.01	2.94	8.70	22.76	60.38	102.26	127.68	71.18	2239.26
-42.35	0.03	0.09	0.29	1.04	2.97	8.57	23.94	59.93	106.46	138.24	67.58	2239.26
-42.25	0.04	0.07	0.24	1.13	3.15	7.48	24.07	58.87	100.33	135.81	1729.06	2239.26
-42.15	0.03	0.15	0.26	0.90	3.24	8.50	23.43	53.14	104.28	139.25	68.08	2239.26
-42.05	0.04	0.09	0.25	1.04	2.80	8.77	22.81	55.10	100.69	133.20	72.62	2239.26
-41.95	0.02	0.04	0.22	1.02	2.78	8.89	22.61	57.82	100.09	135.51	69.69	2239.26
-41.85	0.03	0.07	0.26	1.00	2.68	8.25	24.04	64.01	101.44	137.41	64.82	2239.26
-41.75	0.03	0.10	0.35	0.77	2.65	7.78	23.61	60.38	100.72	133.61	67.36	2239.26
-41.65	0.01	0.07	0.38	1.25	2.85	7.95	24.17	55.40	100.84	129.82	74.72	2239.26
-41.55	0.02	0.09	0.31	1.00	2.53	8.64	21.97	53.74	102.56	138.06	70.52	2239.26
-41.45	0.03	0.13	0.33	0.92	2.95	8.53	23.58	59.63	98.06	138.36	1729.95	2239.26
-41.35	0.03	0.11	0.20	0.99	2.58	7.86	22.05	54.95	97.58	132.72	68.69	2239.26
-41.25	0.01	0.10	0.25	1.05	2.45	7.98	23.81	58.27	99.96	135.04	66.81	2239.26
-41.15	0.03	0.08	0.26	1.19	2.57	9.20	24.07	52.99	100.09	138.95	70.96	2239.26
-41.05	0.02	0.09	0.22	1.12	2.83	9.32	22.94	59.78	100.72	142.39	69.24	2239.26

Midpt.	11000	2700	910	240	84	26	8.8	2.7	0.92	0.31	0.05	Volume
-40.95	0.03	0.11	0.31	0.93	3.09	8.28	23.43	53.14	101.26	135.51	65.70	2239.26
-40.85	0.06	0.11	0.22	0.95	3.04	9.07	23.25	54.34	101.87	139.37	69.96	2239.26
-40.75	0.05	0.05	0.28	0.94	2.75	9.09	23.30	58.57	102.26	135.69	3388.66	2239.26
-40.65	0.01	0.07	0.26	1.08	2.35	8.75	24.35	59.33	101.53	136.34	64.76	2239.26
-40.55	0.03	0.07	0.28	0.94	2.87	8.82	23.25	58.42	101.32	138.71	67.69	2239.26
-40.45	0.02	0.05	0.16	1.00	3.04	8.75	24.24	59.93	102.38	133.02	69.58	2239.26
-40.35	0.02	0.11	0.25	0.94	2.92	8.45	24.68	59.78	102.56	138.12	69.30	2239.26
-40.25	0.03	0.08	0.24	1.21	2.94	8.70	22.30	60.99	103.59	138.48	72.12	2239.26
-40.15	0.02	0.08	0.23	0.94	2.67	9.03	23.99	57.06	104.55	136.88	72.90	2239.26
-40.05	0.05	0.08	0.28	1.09	2.78	8.66	22.69	61.59	102.17	141.50	67.20	2239.26
-39.95	0.01	0.08	0.25	1.16	2.60	8.52	22.97	65.36	103.83	137.77	66.70	2239.26
-39.85	0.02	0.11	0.41	1.04	2.82	9.02	22.66	52.99	98.33	140.91	67.36	2239.26
-39.75	0.01	0.11	0.22	0.81	2.48	8.37	22.94	55.10	99.57	130.89	64.21	2239.26
-39.65	0.03	0.12	0.33	1.05	2.63	8.89	23.30	54.19	98.70	142.15	67.47	2239.26
-39.55	0.03	0.14	0.27	1.06	3.04	8.71	23.86	54.34	99.81	136.28	69.58	2239.26
-39.45	0.03	0.13	0.25	0.95	3.19	8.50	22.10	57.97	100.99	139.84	66.31	2239.26
-39.35	0.01	0.14	0.30	0.95	2.99	7.93	24.45	58.27	104.25	133.79	72.40	2239.26
-39.25	0.03	0.08	0.30	0.97	2.87	8.86	22.56	58.87	102.95	136.99	66.75	2239.26
-39.15	0.03	0.07	0.28	0.87	2.99	8.78	23.99	59.93	101.66	137.82	66.20	2239.26
-39.05	0.02	0.08	0.28	1.05	2.60	8.52	22.94	60.84	106.76	139.07	69.96	2239.26
-38.95	0.01	0.15	0.36	1.17	2.42	8.75	24.01	62.80	101.72	137.17	70.91	2239.26
-38.85	0.01	0.12	0.22	1.13	2.85	8.41	23.30	54.95	100.90	131.89	64.21	2239.26
-38.75	0.04	0.14	0.29	1.02	2.83	9.09	23.04	57.97	104.28	139.96	66.70	2239.26
-38.65	0.05	0.07	0.29	0.93	2.99	8.55	24.12	54.19	102.29	133.02	66.09	2239.26
-38.55	0.03	0.06	0.22	1.03	2.92	8.46	24.12	60.69	96.98	138.30	68.03	2239.26
-38.45	0.02	0.11	0.22	0.86	2.82	8.61	22.23	58.12	99.81	140.67	68.75	2239.26
-38.35	0.03	0.12	0.27	1.08	2.47	8.23	24.42	59.78	101.11	130.35	64.71	2239.26
-38.25	0.02	0.05	0.25	0.95	2.50	9.02	23.58	58.12	103.92	138.18	1724.53	2239.26
-38.15	0.03	0.07	0.33	1.12	2.75	8.93	24.40	59.18	97.19	139.43	70.52	2239.26
-38.05	0.01	0.02	0.21	0.88	2.58	9.00	25.09	57.82	100.45	142.21	70.24	2239.26
-37.95	0.01	0.13	0.28	1.04	2.97	8.57	23.43	55.85	100.96	134.39	61.38	2239.26
-37.85	0.04	0.07	0.30	1.07	2.33	8.23	23.55	58.87	103.68	141.09	67.58	2239.26
-37.75	0.02	0.10	0.25	1.04	2.90	8.87	24.96	57.82	102.56	137.11	66.48	2239.26
-37.65	0.03	0.14	0.27	0.89	2.65	8.80	23.96	57.52	102.62	140.97	68.75	2239.26
-37.55	0.01	0.11	0.22	1.04	2.68	8.50	24.01	62.65	100.69	133.85	67.36	2239.26
-37.45	0.02	0.07	0.32	1.14	2.58	9.00	22.92	62.50	99.57	142.45	65.54	2239.26
-37.35	0.00	0.07	0.25	1.09	2.97	8.50	24.01	59.63	101.63	140.97	1727.68	2239.26
-37.25	0.04	0.08	0.33	1.07	2.62	8.93	23.35	57.06	101.50	136.05	70.19	2239.26
-37.15	0.03	0.06	0.34	1.04	2.85	8.11	23.66	56.91	104.07	136.16	70.79	2239.26
-37.05	0.02	0.07	0.21	0.91	2.62	8.34	22.86	57.52	101.99	136.58	64.76	2239.26
-36.95	0.04	0.07	0.24	0.90	2.33	9.11	22.33	51.78	102.44	140.79	70.07	2239.26
-36.85	0.03	0.11	0.27	0.99	3.02	9.18	22.97	60.08	103.62	141.92	65.15	2239.26
-36.75	0.02	0.07	0.29	1.01	2.67	8.28	24.47	56.76	102.14	139.84	1725.85	2239.26
-36.65	0.04	0.07	0.26	1.25	2.60	8.64	22.74	59.33	99.42	137.65	66.81	2239.26
-36.55	0.03	0.07	0.35	1.13	3.17	8.89	23.50	60.38	103.47	134.09	68.25	2239.26

Midpt.	11000	2700	910	240	84	26	8.8	2.7	0.92	0.31	0.05	Volume
-36.45	0.02	0.09	0.25	1.06	2.70	9.52	22.61	54.19	101.26	140.49	68.19	2239.26
-36.35	0.01	0.13	0.24	1.01	3.10	8.55	23.81	52.38	103.07	139.66	65.81	2239.26
-36.25	0.03	0.11	0.27	0.85	3.00	8.05	24.73	56.01	103.95	137.59	62.33	2239.26
-36.15	0.03	0.15	0.32	0.87	2.67	8.32	23.96	51.93	103.35	141.15	65.09	2239.26
-36.05	0.03	0.08	0.22	1.12	2.70	8.73	21.89	53.14	101.41	137.05	67.31	2239.26
-35.95	0.06	0.06	0.29	1.13	2.80	8.75	23.20	57.52	99.75	139.78	65.59	2239.26
-35.85	0.03	0.15	0.26	0.98	2.88	8.80	23.50	55.85	102.98	136.64	64.87	2239.26
-35.75	0.01	0.11	0.27	1.09	2.78	7.96	23.02	56.16	101.99	139.43	68.80	2239.26
-35.65	0.04	0.11	0.24	0.86	2.57	9.23	23.73	55.85	103.01	143.40	66.26	2239.26
-35.55	0.03	0.08	0.30	1.09	2.85	8.32	23.15	58.42	100.12	134.50	66.03	2239.26
-35.45	0.02	0.14	0.27	0.82	2.67	8.36	22.53	54.95	101.50	140.91	64.76	2239.26
-35.35	0.02	0.07	0.24	1.03	2.73	8.70	21.66	57.21	99.54	138.77	65.59	2239.26
-35.25	0.02	0.07	0.13	1.06	2.57	8.86	23.15	59.48	103.35	136.34	64.04	2239.26
-35.15	0.03	0.09	0.28	0.90	2.85	8.61	22.12	59.63	105.37	136.88	65.09	2239.26
-35.05	0.02	0.07	0.22	0.99	2.85	8.41	23.35	56.01	101.90	143.28	62.93	2239.26
-34.95	0.00	0.12	0.32	1.14	2.48	9.03	23.84	58.72	101.93	142.98	67.92	2239.26
-34.85	0.04	0.11	0.25	1.00	2.92	9.05	24.32	56.46	105.52	145.30	65.26	2239.26
-34.75	0.06	0.17	0.33	0.99	2.53	8.61	23.76	57.36	100.75	142.39	67.42	2239.26
-34.65	0.03	0.13	0.24	1.06	2.80	8.89	23.27	53.14	100.18	138.06	64.87	2239.26
-34.55	0.02	0.10	0.28	1.00	2.72	8.89	22.86	56.61	102.80	132.37	62.16	2239.26
-34.45	0.04	0.15	0.33	1.09	2.85	9.05	24.73	57.52	102.41	138.30	65.09	2239.26
-34.35	0.01	0.07	0.28	0.97	2.75	9.34	25.27	60.84	105.28	126.73	65.81	2239.26
-34.25	0.05	0.08	0.23	0.89	2.83	8.62	22.84	59.48	100.27	138.06	1723.97	2239.26
-34.15	0.03	0.07	0.35	1.09	2.92	9.36	23.22	55.55	102.65	131.42	64.76	2239.26
-34.05	0.01	0.07	0.22	0.95	2.90	9.43	23.22	59.78	102.02	133.08	1725.74	2239.26
-33.95	0.03	0.13	0.24	0.99	2.35	8.25	23.76	61.74	100.42	139.90	68.30	2239.26
-33.85	0.04	0.08	0.16	0.91	2.97	9.05	22.43	56.76	100.87	137.05	1726.74	2239.26
-33.75	0.03	0.09	0.33	1.10	2.68	9.21	23.94	64.61	105.79	137.94	62.44	2239.26
-33.65	0.02	0.16	0.30	0.97	2.35	8.89	23.86	54.80	101.99	132.13	68.91	2239.26
-33.55	0.02	0.16	0.29	1.04	2.87	8.77	24.32	56.31	104.13	130.95	69.58	2239.26
-33.45	0.02	0.07	0.35	0.91	2.48	8.77	23.91	64.91	104.19	139.07	68.75	2239.26
-33.35	0.03	0.11	0.22	1.04	2.63	8.91	23.63	53.14	102.23	132.55	66.31	2239.26
-33.25	0.03	0.12	0.28	0.91	2.80	8.36	21.25	56.91	101.96	137.77	66.98	2239.26
-33.15	0.03	0.11	0.31	0.91	2.85	8.80	24.78	59.48	102.32	140.79	67.14	2239.26
-33.05	0.02	0.07	0.25	1.09	2.99	9.14	23.81	59.93	103.71	141.74	67.47	2239.26
-32.95	0.03	0.07	0.24	1.04	2.83	8.46	23.35	56.46	103.35	132.72	69.24	2239.26
-32.85	0.05	0.11	0.30	1.01	2.77	8.41	22.99	58.57	100.15	133.85	63.05	2239.26
-32.75	0.06	0.12	0.24	1.06	2.85	9.61	23.99	58.72	102.62	132.55	70.74	2239.26
-32.65	0.04	0.17	0.28	0.88	2.94	8.62	23.12	54.65	105.61	131.24	66.75	2239.26
-32.55	0.03	0.11	0.26	1.04	2.65	8.16	23.25	61.29	102.95	133.50	1729.29	2239.26
-32.45	0.05	0.13	0.28	1.05	2.75	9.30	22.02	55.85	99.03	139.19	66.03	2239.26
-32.35	0.02	0.07	0.26	1.09	2.92	8.61	23.91	63.70	102.53	137.59	67.36	2239.26
-32.25	0.03	0.08	0.25	0.99	2.94	8.21	22.84	49.82	102.32	141.03	64.54	2239.26
-32.15	0.03	0.11	0.29	1.07	2.62	8.34	23.76	56.91	103.01	144.41	66.42	2239.26
-32.05	0.02	0.07	0.21	1.18	2.97	8.87	22.84	54.19	105.22	139.13	62.44	2239.26

Midpt.	11000	2700	910	240	84	26	8.8	2.7	0.92	0.31	0.05	Volume
-31.95	0.03	0.10	0.23	1.12	2.33	8.59	23.94	56.46	99.54	137.35	1726.57	2239.26
-31.85	0.01	0.14	0.25	1.17	2.78	8.59	23.76	57.52	98.67	138.83	66.92	2239.26
-31.75	0.01	0.07	0.28	0.98	2.85	9.20	22.35	52.23	102.26	129.76	62.77	2239.26
-31.65	0.04	0.10	0.19	1.13	2.60	8.48	23.30	59.48	99.96	131.48	62.93	2239.26
-31.55	0.01	0.10	0.20	1.02	2.77	8.95	23.89	57.97	100.78	134.03	64.71	2239.26
-31.45	0.06	0.13	0.29	0.90	3.32	8.70	23.89	60.84	99.24	140.49	70.85	2239.26
-31.35	0.03	0.16	0.25	0.96	3.04	8.89	23.81	60.38	99.48	140.79	64.98	2239.26
-31.25	0.04	0.10	0.27	1.15	2.50	8.23	23.17	57.52	103.38	134.68	66.53	2239.26
-31.15	0.02	0.13	0.31	0.99	2.70	8.80	22.69	60.53	100.69	143.40	70.19	2239.26
-31.05	0.02	0.08	0.28	0.90	2.57	8.59	22.02	59.78	99.09	137.29	64.43	2239.26
-30.95	0.01	0.10	0.30	0.96	2.58	8.37	21.54	53.74	101.75	134.56	63.82	2239.26
-30.85	0.00	0.05	0.28	0.85	2.26	9.02	23.30	59.48	100.60	133.26	62.05	2239.26
-30.75	0.03	0.09	0.20	1.14	2.95	9.18	21.87	54.19	102.59	138.71	68.69	2239.26
-30.65	0.03	0.07	0.26	1.04	3.12	8.34	23.32	56.76	101.11	133.85	1724.58	2239.26
-30.55	0.03	0.14	0.15	0.87	2.73	8.11	23.40	57.82	104.46	130.53	64.59	2239.26
-30.45	0.00	0.15	0.24	1.07	2.53	8.61	22.48	58.42	100.93	138.00	66.37	2239.26
-30.35	0.02	0.09	0.24	0.90	2.75	8.52	23.91	60.53	101.78	138.24	64.26	2239.26
-30.25	0.03	0.09	0.24	0.93	3.17	9.12	22.81	58.27	103.44	133.67	67.47	2239.26
-30.15	0.00	0.08	0.23	1.03	2.82	9.30	23.53	58.42	104.70	136.34	1724.58	2239.26
-30.05	0.03	0.08	0.26	0.95	2.47	8.89	23.17	52.68	104.01	141.15	65.65	2239.26
-29.95	0.05	0.11	0.24	1.12	2.42	8.59	21.94	56.01	102.62	136.94	66.53	2239.26
-29.85	0.03	0.07	0.26	0.97	2.55	9.09	23.12	60.84	103.50	136.58	59.34	2239.26
-29.75	0.02	0.12	0.28	0.83	2.95	9.28	22.48	57.97	103.01	133.55	68.97	2239.26
-29.65	0.03	0.13	0.30	1.14	2.68	8.21	24.63	54.34	100.96	142.92	70.19	2239.26
-29.55	0.02	0.11	0.24	1.08	2.48	8.91	23.07	58.12	99.09	137.65	67.53	2239.26
-29.45	0.04	0.10	0.28	0.97	2.83	9.39	22.48	57.82	98.42	140.32	65.15	2239.26
-29.35	0.02	0.06	0.23	1.12	3.15	9.21	22.74	56.76	99.96	138.30	67.75	2239.26
-29.25	0.00	0.08	0.24	1.06	3.22	8.66	23.35	57.67	101.56	131.83	67.69	2239.26
-29.15	0.03	0.08	0.25	0.89	2.60	7.71	22.61	58.57	99.93	137.35	1726.63	2239.26
-29.05	0.03	0.09	0.24	0.92	2.87	8.78	22.25	53.14	101.11	139.25	65.87	2239.26
-28.95	0.03	0.11	0.28	1.03	3.04	8.96	22.40	56.91	101.44	134.50	71.62	2239.26
-28.85	0.02	0.09	0.30	0.95	2.99	9.25	23.55	54.34	101.63	140.91	65.70	2239.26
-28.75	0.03	0.08	0.20	0.89	2.38	9.12	23.76	54.65	100.45	141.62	66.03	2239.26
-28.65	0.02	0.11	0.28	1.10	2.72	9.07	23.53	55.40	101.96	135.51	67.92	2239.26
-28.55	0.00	0.07	0.32	0.99	3.07	8.62	22.84	56.31	99.18	132.96	1725.08	2239.26
-28.45	0.00	0.11	0.30	0.92	2.97	8.86	22.51	52.68	101.35	134.98	62.55	2239.26
-28.35	0.04	0.09	0.25	1.05	3.02	8.25	23.78	59.78	96.82	135.10	68.30	2239.26
-28.25	0.03	0.13	0.27	1.05	2.42	9.14	22.94	49.82	99.00	136.82	64.26	2239.26
-28.15	0.01	0.11	0.28	1.03	2.85	8.84	23.43	55.10	97.70	135.27	64.65	2239.26
-28.05	0.03	0.07	0.22	1.08	2.78	8.11	22.30	57.82	98.12	133.61	65.54	2239.26
-27.95	0.03	0.07	0.24	0.84	2.65	8.84	21.28	56.16	97.55	129.76	64.21	2239.26
-27.85	0.03	0.11	0.25	1.01	2.60	9.14	22.10	52.84	98.42	125.90	62.05	2239.26
-27.75	0.02	0.07	0.25	0.99	2.90	8.11	23.66	49.51	98.91	130.59	63.99	2239.26
-27.65	0.03	0.09	0.16	0.86	2.63	9.20	23.58	57.67	98.88	130.95	63.27	2239.26
-27.55	0.01	0.05	0.31	1.02	2.78	8.53	23.02	54.34	100.63	131.54	67.47	2239.26

Midpt.	11000	2700	910	240	84	26	8.8	2.7	0.92	0.31	0.05	Volume
-27.45	0.02	0.11	0.17	1.00	2.85	8.07	22.38	53.29	98.33	141.80	66.98	2239.26
-27.35	0.02	0.07	0.34	1.12	2.45	8.53	23.78	55.40	99.18	136.34	1724.58	2239.26
-27.25	0.02	0.11	0.28	1.10	2.77	8.73	24.24	52.53	99.06	136.82	60.00	2239.26
-27.15	0.02	0.07	0.30	1.14	3.17	9.20	23.38	58.87	101.29	131.42	60.17	2239.26
-27.05	0.03	0.05	0.35	0.91	2.63	8.12	23.84	50.12	95.62	133.79	65.92	2239.26
-26.95	0.02	0.08	0.22	1.10	2.63	8.80	22.17	54.50	94.53	124.90	63.88	2239.26
-26.85	0.02	0.10	0.32	0.96	2.50	9.18	21.38	53.59	97.64	131.06	61.11	2239.26
-26.75	0.03	0.07	0.38	0.92	2.78	9.09	23.04	44.68	95.86	131.60	65.54	2239.26
-26.65	0.00	0.15	0.28	0.99	3.05	8.75	22.30	55.25	96.07	138.30	64.93	2239.26
-26.55	0.02	0.09	0.22	1.22	2.97	8.70	21.94	51.93	94.35	134.50	1724.08	2239.26
-26.45	0.03	0.10	0.25	1.05	2.73	8.36	23.04	59.78	97.85	128.28	68.25	2239.26
-26.35	0.02	0.11	0.25	0.89	2.78	8.84	21.74	62.04	98.45	125.79	60.39	2239.26
-26.25	0.02	0.10	0.27	1.12	2.82	8.71	23.68	53.74	100.60	131.42	62.33	2239.26
-26.15	0.04	0.12	0.25	0.93	2.94	7.87	23.91	58.27	97.07	129.05	61.94	2239.26
-26.05	0.03	0.11	0.24	0.89	3.05	8.03	23.55	58.42	98.42	136.10	68.69	2239.26
-25.95	0.03	0.18	0.24	0.96	2.58	8.23	23.61	52.53	98.00	129.52	65.65	2239.26
-25.85	0.02	0.09	0.25	0.98	2.97	8.78	23.48	53.14	96.46	134.68	64.93	2239.26
-25.75	0.04	0.11	0.28	0.96	2.33	9.02	22.51	52.08	95.53	130.53	67.03	2239.26
-25.65	0.07	0.15	0.26	1.04	2.63	8.20	20.59	53.59	96.82	130.06	62.93	2239.26
-25.55	0.03	0.08	0.35	0.99	2.53	9.80	22.10	55.10	95.65	133.91	68.08	2239.26
-25.45	0.02	0.13	0.24	0.85	2.42	8.41	22.10	54.34	94.62	128.34	67.42	2239.26
-25.35	0.03	0.07	0.27	1.04	2.83	8.16	22.89	54.34	99.84	129.70	67.69	2239.26
-25.25	0.02	0.08	0.35	0.98	2.92	8.39	22.35	54.95	98.30	132.37	64.32	2239.26
-25.15	0.00	0.04	0.35	1.04	3.10	9.20	23.30	59.33	95.62	134.68	66.75	2239.26
-25.05	0.02	0.07	0.25	1.09	3.86	8.45	23.17	51.02	98.03	133.20	66.14	2239.26
-24.95	0.03	0.09	0.31	1.04	3.27	8.46	22.79	50.87	96.88	131.48	62.66	2239.26
-24.85	0.01	0.08	0.22	1.11	2.75	8.77	22.92	50.12	96.16	127.98	1723.58	2239.26
-24.75	0.03	0.12	0.40	1.22	3.10	9.05	22.94	49.06	98.24	128.63	59.17	2239.26
-24.65	0.00	0.07	0.27	0.95	2.60	9.64	22.10	53.14	97.52	127.86	68.52	2239.26
-24.55	0.03	0.13	0.26	1.00	2.90	9.14	22.69	56.46	98.61	135.57	64.21	2239.26
-24.45	0.03	0.14	0.28	1.06	2.99	9.62	23.20	51.63	96.34	122.82	70.19	2239.26
-24.35	0.02	0.10	0.18	1.20	2.94	8.36	23.38	57.36	98.73	129.76	66.64	2239.26
-24.25	0.05	0.12	0.22	0.91	2.52	8.95	22.81	54.34	99.69	126.73	65.92	2239.26
-24.15	0.03	0.11	0.25	1.11	2.83	8.71	23.15	57.52	94.98	127.09	66.03	2239.26
-24.05	0.02	0.11	0.21	1.01	3.19	9.03	22.05	55.40	95.62	128.69	65.37	2239.26
-23.95	0.03	0.08	0.30	0.95	2.57	8.61	22.43	54.80	99.48	126.97	72.84	2239.26
-23.85	0.02	0.12	0.22	1.09	2.88	9.12	22.02	58.72	96.95	131.12	66.26	2239.26
-23.75	0.03	0.11	0.27	1.03	2.47	9.37	24.47	56.01	97.97	129.82	63.93	2239.26
-23.65	0.03	0.09	0.22	1.22	3.34	9.03	22.02	54.80	97.70	134.03	66.98	2239.26
-23.55	0.03	0.07	0.29	0.96	2.70	8.95	22.97	58.12	98.55	133.14	66.86	2239.26
-23.45	0.03	0.12	0.30	1.13	2.70	8.53	23.25	56.01	98.21	129.82	66.59	2239.26
-23.35	0.03	0.08	0.24	1.14	2.70	9.03	22.92	59.78	98.27	133.73	61.83	2239.26
-23.25	0.03	0.15	0.35	1.11	3.15	8.98	22.43	52.08	94.77	135.45	70.57	2239.26
-23.15	0.02	0.10	0.28	1.27	3.27	9.11	24.50	52.53	99.87	132.01	64.71	2239.26
-23.05	0.08	0.07	0.26	1.09	2.73	8.71	23.50	56.31	98.88	127.74	66.03	2239.26

Midpt.	11000	2700	910	240	84	26	8.8	2.7	0.92	0.31	0.05	Volume
-22.95	0.03	0.10	0.21	0.91	2.78	9.41	23.58	56.01	98.58	134.98	66.75	2239.26
-22.85	0.02	0.16	0.24	1.05	2.88	9.71	22.71	56.31	94.62	126.56	68.52	2239.26
-22.75	0.04	0.15	0.28	1.05	3.02	9.23	23.55	55.85	99.42	129.82	70.30	2239.26
-22.65	0.03	0.11	0.32	1.18	3.10	8.78	24.27	56.46	97.07	130.65	67.53	2239.26
-22.55	0.03	0.07	0.27	1.10	3.04	9.45	22.99	59.63	100.99	131.78	67.97	2239.26
-22.45	0.03	0.11	0.37	1.15	2.85	9.30	24.12	57.67	98.24	132.37	68.30	2239.26
-22.35	0.03	0.10	0.27	1.05	3.00	8.59	25.09	54.65	97.58	134.27	64.21	2239.26
-22.25	0.00	0.07	0.22	1.00	2.85	8.87	23.12	54.34	98.94	132.61	70.96	2239.26
-22.15	0.03	0.11	0.34	1.18	3.22	8.87	24.09	58.72	103.59	134.03	1730.01	2239.26
-22.05	0.01	0.11	0.22	1.26	2.72	8.95	24.83	59.63	101.53	129.70	65.92	2239.26
-21.95	0.03	0.12	0.32	1.21	3.17	9.25	23.53	55.85	100.87	131.30	1728.73	2239.26
-21.85	0.03	0.07	0.38	0.99	3.05	9.57	25.04	59.48	101.99	130.47	65.09	2239.26
-21.75	0.03	0.12	0.35	1.21	3.46	8.75	24.29	57.82	99.30	133.73	70.13	2239.26
-21.65	0.03	0.13	0.28	1.10	2.87	9.66	24.37	59.48	102.17	131.18	70.02	2239.26
-21.55	0.03	0.10	0.25	1.12	3.00	9.03	23.55	56.16	99.21	140.55	67.09	2239.26
-21.45	0.02	0.15	0.30	1.16	2.95	9.91	24.60	51.48	97.40	140.43	69.36	2239.26
-21.35	0.02	0.14	0.37	1.18	3.35	9.73	25.16	56.61	102.11	136.70	72.45	2239.26
-21.25	0.04	0.13	0.25	1.27	3.27	8.21	24.35	52.68	99.72	138.24	74.95	2239.26
-21.15	0.06	0.12	0.31	1.10	3.61	9.55	25.27	52.23	103.26	131.89	71.07	2239.26
-21.05	0.03	0.07	0.30	0.99	3.29	9.05	24.50	64.31	102.02	130.12	67.42	2239.26
-20.95	0.06	0.10	0.25	1.20	3.00	10.05	25.32	54.65	102.98	134.33	70.63	2239.26
-20.85	0.03	0.11	0.25	1.39	3.19	9.43	23.45	61.89	99.78	131.60	73.95	2239.26
-20.75	0.06	0.06	0.25	1.13	3.59	9.43	25.04	56.61	104.58	132.67	70.85	2239.26
-20.65	0.03	0.12	0.37	1.10	2.88	9.62	26.26	62.04	102.17	143.76	69.47	2239.26
-20.55	0.03	0.13	0.37	1.27	3.44	9.37	23.38	56.31	99.03	134.33	72.07	2239.26
-20.45	0.01	0.10	0.24	1.19	3.54	9.41	25.14	56.16	102.14	136.05	71.62	2239.26
-20.35	0.03	0.09	0.33	1.41	3.12	9.96	26.03	64.31	100.87	133.97	72.34	2239.26
-20.25	0.00	0.09	0.35	1.31	3.15	9.46	25.57	60.69	101.17	138.60	72.18	2239.26
-20.15	0.05	0.11	0.26	1.09	3.24	10.03	25.04	57.06	103.10	141.68	73.23	2239.26
-20.05	0.02	0.11	0.32	1.16	3.17	10.30	25.04	59.78	103.16	136.40	71.02	2239.26
-19.95	0.07	0.16	0.35	1.25	3.67	10.11	26.98	63.86	101.99	140.26	74.17	2239.26
-19.85	0.02	0.11	0.24	1.08	3.56	9.61	25.04	59.18	103.10	140.02	70.35	2239.26
-19.75	0.04	0.11	0.26	1.16	3.27	10.46	26.82	59.02	104.22	137.17	69.96	2239.26
-19.65	0.02	0.10	0.38	1.27	3.32	10.23	25.04	63.70	101.66	133.61	77.71	2239.26
-19.55	0.02	0.08	0.26	1.27	3.39	9.62	25.16	61.14	103.10	136.70	72.73	2239.26
-19.45	0.03	0.18	0.28	1.28	3.25	10.45	26.26	59.48	105.22	142.81	73.84	2239.26
-19.35	0.01	0.11	0.26	1.32	3.25	10.78	26.70	62.65	102.92	144.17	70.96	2239.26
-19.25	0.03	0.07	0.41	1.12	3.04	10.18	26.19	55.10	102.32	143.40	1731.67	2239.26
-19.15	0.02	0.07	0.31	0.98	2.67	8.66	21.23	53.29	85.74	112.80	59.23	2239.26
-19.05	0.00	0.00	0.06	0.17	0.50	1.25	2.76	6.94	8.36	11.98	3.65	12225.45
-18.95	0.00	0.00	0.03	0.16	0.49	1.27	2.86	4.83	7.37	10.56	3.93	139.22
-18.85	0.00	0.01	0.06	0.19	0.50	1.34	2.76	5.13	7.88	10.38	3.93	128.90
-18.75	0.00	0.02	0.05	0.16	0.45	1.18	2.43	4.08	8.09	8.66	3.82	121.87
-18.65	0.00	0.00	0.05	0.13	0.34	1.09	1.92	3.62	6.31	8.24	2.93	116.27
-18.55	0.00	0.02	0.05	0.15	0.42	1.16	2.55	3.62	6.61	8.07	3.65	111.53

Midpt.	11000	2700	910	240	84	26	8.8	2.7	0.92	0.31	0.05	Volume
-18.45	0.01	0.02	0.04	0.20	0.50	0.98	1.84	5.89	6.85	8.24	3.10	107.38
-18.35	0.01	0.02	0.03	0.15	0.45	1.16	2.22	3.77	6.82	7.59	4.10	103.68
-18.25	0.00	0.03	0.01	0.12	0.39	1.23	2.55	6.34	6.61	9.55	3.04	100.32
-18.15	0.00	0.01	0.07	0.12	0.47	1.02	2.66	5.43	6.46	7.29	3.04	97.24
-18.05	0.01	0.01	0.05	0.13	0.39	1.32	1.86	4.98	5.83	7.65	2.82	94.39
-17.95	0.00	0.01	0.04	0.21	0.52	0.96	1.79	3.17	5.49	8.18	2.60	91.74
-17.85	0.02	0.01	0.04	0.16	0.27	0.87	1.86	4.98	4.98	8.72	3.60	89.27
-17.75	0.00	0.01	0.04	0.16	0.44	0.95	1.86	4.23	5.25	6.82	2.44	86.94
-17.65	0.00	0.02	0.03	0.15	0.35	0.75	1.94	2.26	5.80	7.53	3.65	84.75
-17.55	0.00	0.02	0.05	0.13	0.42	0.93	1.89	3.32	6.37	5.87	2.38	82.67
-17.45	0.00	0.02	0.04	0.12	0.35	0.93	2.53	2.42	4.80	5.04	1.94	80.70
-17.35	0.00	0.02	0.03	0.14	0.29	0.95	2.02	2.42	4.68	5.69	2.49	78.83
-17.25	0.00	0.00	0.03	0.13	0.35	0.84	2.20	3.92	5.19	5.57	2.60	77.04
-17.15	0.00	0.01	0.05	0.13	0.39	0.87	1.81	4.23	5.31	7.18	3.32	75.33
-17.05	0.02	0.02	0.01	0.15	0.25	0.82	1.69	3.32	4.80	5.52	2.82	73.70
-16.95	0.01	0.01	0.02	0.15	0.44	0.75	1.48	3.17	4.23	5.63	3.16	72.14
-16.85	0.00	0.01	0.03	0.16	0.39	0.77	2.09	4.08	5.19	4.39	3.21	70.64
-16.75	0.00	0.01	0.05	0.16	0.34	0.93	1.71	3.47	4.53	5.10	2.60	69.20
-16.65	0.00	0.02	0.04	0.16	0.35	0.75	2.15	3.32	4.68	5.81	2.32	67.81
-16.55	0.02	0.02	0.05	0.13	0.39	0.93	1.86	4.38	5.01	5.34	2.49	66.48
-16.45	0.00	0.01	0.05	0.13	0.44	0.82	1.76	3.32	3.32	5.57	3.38	65.19
-16.35	0.00	0.01	0.05	0.14	0.35	0.82	1.86	1.81	4.23	4.63	2.27	63.95
-16.25	0.00	0.03	0.05	0.12	0.40	0.87	1.89	4.08	4.65	4.51	2.60	62.76
-16.15	0.01	0.02	0.07	0.19	0.23	0.91	1.76	2.42	4.71	5.22	2.82	61.60
-16.05	0.02	0.01	0.07	0.17	0.55	0.70	1.76	3.47	3.38	5.34	2.16	60.48
-15.95	0.00	0.03	0.02	0.13	0.35	0.91	2.04	4.83	4.23	5.87	3.99	59.40
-15.85	0.00	0.02	0.05	0.14	0.22	0.96	1.76	4.08	4.74	3.85	2.55	58.35
-15.75	0.00	0.02	0.04	0.16	0.45	1.12	1.81	2.26	3.68	5.69	2.44	57.34
-15.65	0.00	0.02	0.02	0.13	0.25	1.11	1.84	3.47	4.56	5.28	3.60	56.36
-15.55	0.00	0.01	0.06	0.15	0.29	0.73	2.07	2.42	4.32	3.97	3.54	55.41
-15.45	0.00	0.04	0.05	0.18	0.44	0.98	2.15	5.13	6.40	8.54	3.71	54.48
-15.35	0.00	0.02	0.02	0.04	0.25	0.77	2.45	4.08	6.43	8.54	3.99	53.59
-15.25	0.00	0.02	0.05	0.12	0.18	0.91	2.15	4.23	6.19	9.13	4.87	52.72
-15.15	0.01	0.01	0.01	0.09	0.25	0.95	1.97	4.23	6.13	8.72	5.31	51.87
-15.05	0.00	0.01	0.05	0.15	0.20	0.86	2.38	3.62	6.04	7.35	3.87	51.05
-14.95	0.02	0.01	0.02	0.12	0.37	0.89	1.46	3.62	5.74	9.13	3.93	50.25
-14.85	0.00	0.01	0.06	0.18	0.25	0.89	2.15	3.92	6.19	7.29	4.82	49.48
-14.75	0.00	0.01	0.03	0.13	0.30	0.95	2.22	3.17	6.52	10.02	4.93	48.72
-14.65	0.00	0.00	0.05	0.16	0.23	1.07	1.92	4.68	7.52	7.65	4.98	47.99
-14.55	0.01	0.01	0.02	0.16	0.35	1.20	2.55	4.53	6.25	7.47	5.04	47.28
-14.45	0.00	0.02	0.04	0.15	0.39	1.07	2.71	5.43	7.25	7.35	5.37	46.58
-14.35	0.00	0.02	0.05	0.10	0.45	1.02	2.17	4.98	6.07	7.89	5.37	45.91
-14.25	0.01	0.02	0.05	0.14	0.49	1.50	2.63	4.68	7.61	8.07	4.87	45.25
-14.15	0.00	0.02	0.05	0.19	0.70	1.20	2.48	4.53	7.31	8.30	5.98	44.61
-14.05	0.00	0.01	0.02	0.12	0.45	1.30	2.40	4.38	7.85	8.30	5.15	43.98

Midpt.	11000	2700	910	240	84	26	8.8	2.7	0.92	0.31	0.05	Volume
-13.95	0.00	0.01	0.06	0.16	0.45	1.12	3.04	5.28	7.82	9.19	5.65	43.38
-13.85	0.01	0.02	0.05	0.20	0.44	1.05	2.43	4.53	7.06	9.13	5.20	42.78
-13.75	0.01	0.01	0.05	0.21	0.54	1.54	3.42	6.34	7.37	9.19	6.03	42.21
-13.65	0.01	0.00	0.04	0.18	0.82	1.41	2.71	5.13	7.28	9.79	6.86	41.65
-13.55	0.00	0.01	0.08	0.23	0.54	1.25	2.94	5.74	7.10	8.96	5.54	41.10
-13.45	0.00	0.03	0.07	0.23	0.52	1.45	3.47	6.64	7.97	10.62	7.80	40.56
-13.35	0.00	0.01	0.06	0.25	0.62	1.46	3.24	5.89	8.51	8.66	7.03	40.04
-13.25	0.00	0.02	0.09	0.23	0.77	1.52	3.45	6.49	8.36	10.62	7.47	39.54
-13.15	0.00	0.02	0.08	0.22	0.62	1.86	3.96	6.34	8.06	8.54	7.53	39.04
-13.05	0.00	0.02	0.09	0.26	0.55	1.87	3.35	5.89	8.88	10.79	8.36	38.56
-12.95	0.01	0.04	0.05	0.26	0.79	1.59	3.65	6.49	9.15	10.85	8.03	38.09
-12.85	0.00	0.02	0.07	0.23	0.77	2.11	3.99	6.49	7.91	12.04	10.07	37.63
-12.75	0.00	0.02	0.09	0.30	0.81	2.00	3.83	7.55	8.85	9.79	9.19	37.18
-12.65	0.02	0.04	0.10	0.34	0.81	2.09	4.75	6.79	9.24	10.38	9.41	36.74
-12.55	0.01	0.09	0.14	0.30	0.82	2.41	4.11	8.91	9.63	12.75	10.24	36.32
-12.45	0.01	0.04	0.09	0.39	0.79	2.30	4.45	6.34	10.93	11.86	10.07	35.90
-12.35	0.01	0.03	0.09	0.41	1.04	2.62	5.70	8.30	10.96	13.88	11.13	35.50
-12.25	0.00	0.07	0.11	0.49	1.04	2.95	5.54	9.06	12.05	14.29	10.13	35.10
-12.15	0.00	0.07	0.13	0.47	1.21	2.87	5.39	8.91	12.26	12.16	10.57	34.72
-12.05	0.00	0.03	0.18	0.48	1.24	3.27	6.11	14.49	11.74	13.70	12.62	34.34
-11.95	0.02	0.04	0.23	0.59	1.26	3.27	6.36	10.27	14.16	13.70	12.07	33.98
-11.85	0.01	0.06	0.15	0.53	1.41	3.25	7.23	12.23	13.59	15.24	13.45	33.62
-11.75	0.03	0.08	0.18	0.72	1.44	4.04	6.62	9.66	15.34	15.24	15.39	33.27
-11.65	0.03	0.07	0.18	0.65	1.81	3.89	7.89	13.13	14.67	20.52	14.06	32.93
-11.55	0.01	0.08	0.24	0.68	1.78	4.36	7.97	12.38	15.70	18.98	15.72	32.60
-11.45	0.00	0.09	0.25	0.97	1.81	4.48	8.61	12.23	16.79	20.22	16.72	32.27
-11.35	0.05	0.10	0.32	0.98	2.06	4.79	9.86	12.98	17.06	19.69	17.82	31.96
-11.25	0.03	0.10	0.30	0.85	2.01	5.46	9.43	13.89	18.96	19.81	18.76	31.65
-11.15	0.04	0.11	0.37	1.03	2.58	5.87	9.81	16.76	19.99	20.70	18.65	31.35
-11.05	0.03	0.10	0.33	1.28	2.52	5.84	11.09	14.04	21.53	24.26	21.92	31.06
-10.95	0.03	0.16	0.36	1.23	2.95	6.82	12.19	19.47	24.39	26.33	23.58	30.77
-10.85	0.07	0.18	0.58	1.55	2.78	7.62	12.47	20.98	24.21	25.38	22.80	30.50
-10.75	0.03	0.19	0.52	1.48	3.44	8.53	14.87	22.49	25.57	29.83	23.69	30.23
-10.65	0.03	0.25	0.68	1.67	3.62	8.80	16.02	21.13	27.96	28.82	24.63	29.96
-10.55	0.08	0.30	0.70	1.75	4.09	10.11	15.79	22.64	28.08	31.79	26.18	29.71
-10.45	0.05	0.25	0.69	2.15	4.33	10.28	17.53	24.46	30.67	38.25	28.89	29.46
-10.35	0.08	0.20	0.85	2.29	4.60	11.39	19.59	27.93	32.73	35.76	29.72	29.21
-10.25	0.11	0.37	1.01	2.75	5.85	11.77	20.54	25.36	35.02	38.07	33.65	28.98
-10.15	0.14	0.53	1.10	2.77	6.32	13.07	20.74	31.70	35.72	43.65	34.76	28.75
-10.05	0.08	0.56	1.18	3.20	6.63	14.43	22.92	33.51	39.31	44.24	38.25	28.52
-9.95	0.13	0.56	1.29	3.72	7.04	15.93	25.70	38.65	44.86	49.58	41.24	28.31
-9.85	0.17	0.56	1.44	4.12	8.03	15.98	27.41	43.78	45.71	49.76	44.06	28.09
-9.75	0.17	0.68	1.66	4.68	8.92	17.25	28.89	46.19	45.74	48.27	47.10	27.89
-9.65	0.20	0.83	2.03	4.99	9.17	19.11	29.48	47.70	53.59	54.86	47.38	27.69
-9.55	0.21	0.79	2.11	5.61	10.43	21.19	32.57	55.70	54.62	58.30	47.27	27.49

Midpt.	11000	2700	910	240	84	26	8.8	2.7	0.92	0.31	0.05	Volume
-9.45	0.30	0.98	2.28	6.03	12.14	24.07	34.46	57.67	58.24	61.97	50.70	27.30
-9.35	0.27	1.28	2.74	7.19	12.68	26.18	38.04	58.42	58.00	68.56	51.09	27.12
-9.25	0.44	1.29	3.21	7.41	14.39	28.00	41.82	61.14	62.13	74.78	60.33	26.94
-9.15	0.42	1.48	3.43	8.50	14.61	31.10	44.32	60.99	69.59	75.67	60.50	26.77
-9.05	0.40	1.55	3.94	9.55	17.66	34.60	48.44	81.06	75.51	85.34	68.30	26.60
-8.95	0.45	1.81	4.86	10.84	19.05	39.66	54.06	79.86	74.60	90.38	72.18	26.44
-8.85	0.72	2.02	4.96	11.97	21.23	39.66	58.09	77.74	83.45	88.13	79.26	26.29
-8.75	0.67	2.20	5.76	13.28	23.88	42.32	61.80	80.91	87.47	92.22	84.24	26.14
-8.65	0.66	2.35	5.94	13.78	24.05	43.44	64.38	83.93	97.28	93.58	75.17	25.99
-8.55	0.89	2.90	6.37	15.41	25.19	44.94	66.19	83.78	96.19	98.15	81.75	25.85
-8.45	0.83	2.94	6.97	16.73	26.72	49.10	70.69	95.41	91.84	101.23	94.26	25.71
-8.35	1.13	3.29	7.41	18.17	28.43	50.24	71.53	87.71	103.50	95.13	91.00	25.58
-8.25	0.92	3.75	8.15	18.27	30.24	51.24	71.35	97.37	96.92	105.33	89.39	25.45
-8.15	1.13	3.77	8.46	19.10	32.74	52.51	73.45	87.10	95.31	104.85	74.17	25.33
-8.05	1.16	3.96	9.03	20.44	33.45	52.87	74.11	91.93	99.18	101.65	88.45	25.21
-7.95	1.20	3.85	9.75	22.31	33.56	52.99	76.28	95.10	103.80	104.73	91.77	25.10
-7.85	1.27	4.10	10.05	22.14	34.10	55.23	72.35	80.91	92.48	94.47	98.03	24.99
-7.75	1.33	4.11	10.21	22.24	34.99	54.40	71.66	70.04	87.98	87.53	85.74	24.89
-7.65	1.31	4.30	10.26	22.96	37.27	52.62	72.32	73.82	84.66	96.55	85.07	24.79
-7.55	1.45	4.79	10.54	23.17	34.84	53.80	69.18	78.35	89.00	91.33	69.63	24.69
-7.45	1.44	4.60	11.31	23.06	35.11	51.71	64.53	69.74	82.06	82.91	66.09	24.60
-7.35	1.43	4.43	11.16	24.41	35.54	52.28	61.87	63.70	78.14	88.42	58.45	24.52
-7.25	1.47	4.53	11.61	25.03	35.14	48.35	59.91	60.38	75.54	75.67	57.57	24.44
-7.15	1.45	4.71	11.62	24.77	35.42	48.42	57.43	53.74	66.45	75.32	59.83	24.36
-7.05	1.31	4.44	11.43	24.18	35.98	44.96	49.79	55.40	61.65	67.19	67.86	24.28
-6.95	1.54	4.45	11.08	22.34	33.45	43.14	52.68	60.23	65.67	58.12	74.78	24.22
-6.85	1.55	4.51	10.84	21.72	33.55	39.42	50.02	60.23	61.35	53.14	78.71	24.15
-6.75	1.52	3.95	10.40	20.52	31.38	36.51	45.91	62.80	62.01	60.55	67.81	24.09
-6.65	1.08	3.98	9.94	21.03	31.53	34.51	42.56	57.67	57.39	59.19	61.16	24.03
-6.55	1.10	4.22	9.89	19.49	28.31	32.37	40.57	51.33	52.35	47.33	59.00	23.98
-6.45	1.15	3.70	9.39	18.27	27.24	28.91	36.53	47.25	45.92	46.26	54.91	23.93
-6.35	1.08	3.60	8.48	16.85	26.54	27.82	33.57	38.95	38.92	40.98	47.38	23.89
-6.25	1.01	3.57	7.63	15.93	24.05	25.68	28.69	33.06	33.33	30.90	48.88	23.85
-6.15	0.83	3.15	7.70	14.52	23.48	24.32	26.01	27.93	31.94	28.70	48.82	23.81
-6.05	0.95	2.66	6.42	13.54	20.93	22.80	24.17	25.51	27.96	28.29	35.15	23.78
-5.95	0.72	2.65	6.57	12.00	19.71	20.93	19.36	28.53	23.07	27.93	27.07	23.75
-5.85	0.76	2.32	6.09	10.97	18.10	19.59	18.96	19.78	22.52	28.82	29.95	23.73
-5.75	0.74	2.26	5.50	9.92	16.69	17.82	16.78	15.85	20.89	31.85	26.68	23.71
-5.65	0.51	2.15	4.97	8.68	14.83	15.89	16.50	16.15	19.17	25.20	25.68	23.69
-5.55	0.51	1.82	4.70	7.75	13.08	15.82	17.12	15.10	20.20	21.65	27.40	23.68
-5.45	0.56	1.59	4.11	6.93	11.99	13.27	13.72	11.32	16.82	17.38	22.36	23.67
-5.35	0.35	1.70	3.76	6.42	11.14	11.46	11.39	6.19	14.13	10.14	11.85	23.67
-5.25	0.35	1.56	3.54	5.66	9.36	10.09	10.58	7.70	12.83	9.55	8.80	23.67
-5.15	0.41	1.23	3.30	5.81	9.44	9.03	10.37	8.30	13.34	10.20	6.64	23.67
-5.05	0.36	1.19	3.09	5.18	8.62	8.71	8.74	5.74	10.20	10.44	5.70	23.67

Midpt.	11000	2700	910	240	84	26	8.8	2.7	0.92	0.31	0.05	Volume
-4.95	0.30	0.93	2.65	5.27	7.93	7.62	8.23	8.00	10.20	7.12	7.58	23.67
-4.85	0.19	0.99	2.61	4.73	7.85	7.78	6.28	6.79	10.81	7.18	8.58	23.67
-4.75	0.17	0.82	2.45	4.33	6.91	6.98	5.77	6.19	8.21	8.60	7.14	23.67
-4.65	0.19	0.77	2.06	4.33	6.73	6.80	5.98	6.04	7.55	9.73	8.03	23.67
-4.55	0.22	0.70	1.88	4.33	6.04	6.30	5.13	3.32	7.61	8.90	6.70	23.67
-4.45	0.18	0.65	1.94	3.86	6.11	6.61	5.82	4.38	6.64	8.12	8.30	23.67
-4.35	0.20	0.78	1.97	4.00	5.77	6.45	5.42	6.79	6.61	6.46	8.41	23.67
-4.25	0.20	0.61	1.78	3.82	5.80	6.21	5.65	4.98	5.65	6.64	9.58	23.67
-4.15	0.22	0.71	1.71	3.78	5.23	5.32	5.90	6.19	7.10	5.81	9.58	23.67
-4.05	0.25	0.65	1.61	3.63	5.37	5.29	5.19	5.89	6.94	6.70	7.58	23.67
-3.95	0.26	0.70	1.58	3.48	4.31	6.04	6.62	6.94	6.97	7.29	7.03	23.67
-3.85	0.16	0.62	1.65	3.44	4.53	5.70	7.08	5.13	6.91	8.72	6.81	23.67
-3.75	0.18	0.51	1.78	3.19	4.63	5.64	6.46	6.64	6.91	10.91	6.97	23.67
-3.65	0.21	0.63	1.60	3.30	4.60	5.87	6.77	8.15	6.94	9.31	7.75	23.67
-3.55	0.24	0.52	1.79	3.18	4.86	5.48	6.85	9.81	8.76	11.86	9.13	23.67
-3.45	0.28	0.67	1.72	3.50	5.13	5.96	8.17	8.45	8.45	9.90	9.02	23.67
-3.35	0.17	0.82	1.75	3.45	4.80	5.95	8.20	10.42	9.42	9.73	8.47	23.67
-3.25	0.35	0.78	1.85	3.86	5.50	6.70	8.86	8.60	8.79	10.97	10.57	23.67
-3.15	0.30	0.76	1.96	3.72	5.13	6.68	9.35	8.91	9.18	10.67	7.75	23.67
-3.05	0.30	0.83	1.80	3.81	4.60	6.89	8.71	9.96	10.75	11.09	8.58	23.67
-2.95	0.22	1.01	1.92	4.50	5.40	6.39	10.58	11.32	10.69	11.27	10.90	23.67
-2.85	0.30	1.01	2.08	4.41	5.55	7.68	10.63	10.11	11.80	11.68	9.96	23.67
-2.75	0.35	0.97	1.96	5.05	5.22	8.43	11.27	8.15	12.80	11.62	12.23	23.67
-2.65	0.24	1.15	2.25	5.14	6.36	8.45	11.27	11.47	11.96	13.64	12.68	23.67
-2.55	0.30	1.14	2.10	5.29	5.80	9.14	11.44	13.13	12.86	12.45	11.73	23.67
-2.45	0.33	1.27	2.34	5.55	6.91	9.18	13.85	13.89	13.77	13.58	13.23	23.67
-2.35	0.35	1.32	2.46	6.13	7.88	11.00	13.92	15.55	14.61	15.72	14.00	23.67
-2.25	0.40	1.19	2.57	6.15	8.07	11.21	14.13	16.00	17.24	15.18	13.84	23.67
-2.15	0.40	1.27	3.04	7.03	9.17	11.96	15.48	15.85	18.30	18.44	15.11	23.67
-2.05	0.35	1.54	2.99	7.17	9.54	13.68	14.71	19.02	17.09	18.50	19.15	23.67
-1.95	0.49	1.70	3.21	7.47	10.13	13.89	16.86	17.81	19.35	18.74	19.87	23.67
-1.85	0.47	1.62	3.57	7.94	11.00	15.23	17.12	18.42	21.04	20.88	18.87	23.67
-1.75	0.49	1.86	4.09	9.23	12.09	16.39	19.11	21.44	23.91	23.66	23.19	23.67
-1.65	0.41	1.70	4.27	9.13	11.07	17.32	20.92	27.17	25.75	27.52	20.81	23.67
-1.55	0.61	1.99	4.48	10.26	12.76	19.80	23.73	22.79	25.93	29.06	24.58	23.67
-1.45	0.54	1.92	4.79	10.83	13.65	21.96	24.63	28.53	26.81	28.82	28.51	23.67
-1.35	0.78	2.61	5.21	12.14	14.81	24.18	27.54	31.25	30.83	29.18	25.68	23.67
-1.25	0.72	2.40	5.70	12.53	16.10	27.10	28.48	31.40	32.06	30.01	28.17	23.67
-1.15	0.68	2.63	5.45	13.83	17.81	26.25	32.09	34.72	36.62	33.51	30.22	23.67
-1.05	0.75	2.63	6.51	14.36	18.92	28.85	33.59	35.63	40.55	37.07	34.21	23.67
-0.95	0.83	2.65	6.59	15.05	20.50	31.18	36.25	40.00	41.51	42.46	37.64	23.67
-0.85	0.91	2.95	6.80	15.77	20.40	34.60	38.50	53.14	46.68	44.06	41.40	23.67
-0.75	0.80	2.63	6.88	16.01	22.48	33.92	39.98	60.99	46.50	50.94	45.39	23.67
-0.65	0.82	2.85	7.10	16.38	22.88	36.78	42.41	56.61	51.69	57.11	48.43	23.67
-0.55	0.82	2.98	7.13	17.29	23.73	36.41	43.46	67.93	54.95	59.66	55.85	23.67

Midpt.	11000	2700	910	240	84	26	8.8	2.7	0.92	0.31	0.05	Volume
-0.45	0.83	2.99	7.00	16.31	24.67	37.84	41.87	59.48	58.39	58.71	58.67	23.67
-0.35	0.83	2.52	6.68	16.53	24.71	38.87	43.46	55.25	58.93	57.82	58.23	23.67
-0.25	0.84	2.76	6.55	15.74	24.74	38.01	44.66	54.95	59.72	63.63	62.44	23.67
-0.05	0.86	2.36	6.11	15.54	24.10	38.71	47.24	56.76	60.87	62.98	58.95	23.67
0.05	0.83	2.25	5.80	14.57	25.14	39.48	47.29	53.89	64.37	60.85	59.89	23.67
0.25	0.66	1.96	5.79	13.33	24.47	39.16	47.67	57.97	61.44	61.14	64.48	23.67
0.35	0.78	2.08	5.20	12.86	24.25	37.50	48.82	58.27	63.55	66.48	60.67	23.67
0.45	0.53	2.03	4.76	11.99	21.65	34.01	47.31	48.91	59.09	56.93	59.12	23.67
0.55	0.51	1.88	4.31	10.98	19.99	34.35	44.07	49.06	60.53	64.11	55.68	23.67
0.65	0.47	1.83	3.91	10.20	18.32	32.55	42.02	48.46	56.16	56.28	53.41	23.67
0.75	0.37	1.65	3.71	9.59	18.01	29.34	39.44	40.31	50.66	56.34	51.14	23.67
0.85	0.46	1.42	3.51	9.31	16.67	26.32	37.48	42.42	50.48	51.77	49.82	23.67
0.95	0.39	1.36	2.92	8.05	15.36	25.64	35.59	38.04	46.83	45.25	44.72	23.67
1.05	0.30	1.28	3.13	7.58	13.75	24.07	30.94	36.23	43.63	43.77	38.30	23.67
1.15	0.36	0.80	2.55	6.57	13.03	21.55	29.38	34.12	41.39	40.15	38.80	23.67
1.25	0.29	0.88	2.23	5.68	11.52	20.80	26.80	28.53	36.86	36.95	39.30	23.67
1.35	0.25	0.84	1.94	5.73	10.63	18.12	24.37	28.83	33.88	38.01	33.38	23.67
1.45	0.19	0.73	1.79	5.10	10.13	16.37	22.10	25.21	30.64	33.21	30.50	23.67
1.55	0.19	0.72	1.90	4.40	8.67	14.53	21.02	25.51	29.29	29.53	28.23	23.67
1.65	0.22	0.66	1.38	4.33	8.29	14.21	18.78	22.19	26.84	26.63	27.34	23.67
1.75	0.12	0.56	1.41	4.05	7.58	12.62	19.03	24.15	23.40	25.98	23.97	23.67
1.85	0.20	0.48	1.29	3.35	6.88	11.43	15.61	20.08	22.31	24.37	21.86	23.67
1.95	0.12	0.48	1.15	2.99	6.42	10.66	15.69	17.81	19.93	20.64	19.93	23.67
2.05	0.07	0.41	1.15	2.93	5.48	9.70	15.28	18.87	19.99	19.21	19.87	23.67
2.15	0.11	0.37	1.21	2.63	5.57	9.11	12.93	16.91	18.18	20.28	18.49	23.67
2.25	0.08	0.36	0.99	2.59	5.37	9.14	13.03	11.93	18.48	16.90	18.76	23.67
2.35	0.11	0.28	0.80	2.39	4.58	8.52	11.47	14.49	15.82	16.31	14.56	23.67
2.45	0.04	0.29	0.73	2.29	4.73	7.36	11.16	12.68	15.67	15.18	13.06	23.67
2.55	0.10	0.30	0.71	2.11	3.99	7.09	9.71	15.10	15.01	16.43	16.38	23.67
2.65	0.04	0.18	0.75	1.79	3.87	6.59	10.04	12.08	13.07	14.11	14.34	23.67
2.75	0.08	0.20	0.82	1.73	3.44	6.20	10.07	9.51	13.77	15.78	12.95	23.67
2.85	0.07	0.23	0.60	1.76	3.29	7.12	9.40	11.93	13.89	12.28	13.73	23.67
2.95	0.03	0.23	0.68	1.79	2.88	6.59	8.48	9.21	13.04	13.58	11.90	23.67
3.05	0.03	0.27	0.53	1.69	3.17	5.73	9.20	11.32	12.32	13.64	12.56	23.67
3.15	0.08	0.15	0.60	1.53	3.17	6.12	8.12	11.32	12.17	12.39	12.68	23.67
3.25	0.08	0.18	0.65	1.61	3.15	5.09	8.38	12.38	12.20	11.33	11.79	23.67
3.35	0.04	0.24	0.48	1.49	3.17	5.98	7.89	11.77	13.13	10.67	12.12	23.67
3.45	0.02	0.19	0.57	1.50	3.32	5.73	8.71	10.72	11.87	11.03	11.29	23.67
3.55	0.06	0.16	0.49	1.43	2.82	5.64	8.00	10.42	11.74	12.22	11.40	23.67
3.65	0.02	0.12	0.38	1.37	3.04	6.02	8.46	9.51	11.56	12.93	12.12	23.67
3.75	0.08	0.16	0.46	1.22	2.85	5.27	8.28	10.11	11.44	13.88	11.40	23.67
3.85	0.04	0.16	0.46	1.55	3.35	5.62	8.07	10.87	10.48	11.51	11.24	23.67
3.95	0.04	0.16	0.44	1.32	3.02	5.52	7.41	9.36	11.32	11.56	13.28	23.67
4.05	0.04	0.12	0.36	1.20	2.50	5.02	7.61	9.36	11.50	12.75	12.79	23.67
4.15	0.03	0.19	0.45	1.32	3.40	5.52	7.89	10.27	11.59	11.15	11.85	23.67

Midpt.	11000	2700	910	240	84	26	8.8	2.7	0.92	0.31	0.05	Volume
4.25	0.05	0.11	0.48	1.30	3.05	5.73	8.58	11.02	11.93	12.16	11.62	23.67
4.35	0.02	0.08	0.42	1.20	2.78	5.16	7.72	10.87	11.17	13.52	12.40	23.67
4.45	0.07	0.13	0.30	1.32	2.45	5.39	7.51	12.08	11.20	13.46	13.17	23.67
4.55	0.03	0.11	0.42	1.32	2.83	5.14	7.89	12.83	11.47	12.04	12.45	23.67
4.65	0.03	0.17	0.43	1.18	2.58	5.05	8.46	7.85	11.90	13.70	14.50	23.67
4.75	0.04	0.15	0.35	1.21	2.92	5.52	8.86	9.66	11.68	13.40	14.50	23.67
4.85	0.02	0.11	0.33	1.27	2.80	5.41	8.25	13.13	11.47	14.94	14.00	23.67
4.95	0.03	0.11	0.37	1.32	2.70	5.12	7.92	12.68	11.84	12.69	13.23	23.67
5.05	0.02	0.12	0.38	1.32	2.33	5.20	7.51	10.11	11.59	13.28	14.39	23.67
5.15	0.06	0.13	0.28	1.26	2.55	5.00	8.35	11.17	13.89	13.76	14.72	23.67
5.25	0.03	0.12	0.40	1.22	2.67	5.52	7.74	10.72	12.98	13.76	14.39	23.67
5.35	0.00	0.13	0.32	1.06	2.72	5.54	8.23	11.62	12.83	14.94	14.67	23.67
5.45	0.04	0.11	0.45	1.35	2.94	7.00	12.47	18.72	24.46	23.72	23.14	23.67
5.55	0.03	0.15	0.52	1.48	3.09	7.84	14.46	20.83	26.51	27.58	22.47	23.68
5.65	0.03	0.14	0.45	1.52	3.32	8.52	15.51	19.17	27.56	28.11	20.48	23.69
5.75	0.08	0.20	0.53	1.35	3.29	7.80	16.91	19.62	27.29	29.12	24.91	23.71
5.85	0.05	0.17	0.58	1.41	3.44	8.61	15.43	23.10	29.71	32.50	23.64	23.73
5.95	0.06	0.20	0.53	1.37	3.25	8.18	17.35	20.98	30.52	30.90	24.19	23.75
6.05	0.03	0.19	0.48	1.43	3.39	8.59	16.76	24.61	30.74	32.80	25.18	23.78
6.15	0.04	0.15	0.51	1.59	3.56	7.87	16.78	28.83	28.83	32.97	26.85	23.81
6.25	0.03	0.21	0.46	1.49	3.14	8.73	16.40	24.30	27.44	35.23	26.73	23.85
6.35	0.03	0.16	0.45	1.34	3.76	8.61	17.12	27.78	28.44	33.15	29.61	23.89
6.45	0.07	0.17	0.55	1.56	3.42	8.95	16.68	23.10	30.01	35.23	28.78	23.93
6.55	0.02	0.13	0.48	1.57	3.87	8.68	17.60	27.63	30.43	32.14	26.57	23.98
6.65	0.02	0.16	0.49	1.68	3.76	9.73	18.09	22.04	32.55	35.17	26.02	24.03
6.75	0.04	0.18	0.56	1.62	4.26	9.36	16.50	28.23	34.09	34.28	29.83	24.09
6.85	0.06	0.17	0.53	1.47	4.11	9.68	17.30	27.78	33.75	37.30	31.66	24.15
6.95	0.04	0.17	0.48	1.63	3.77	9.71	18.88	28.38	35.29	37.30	34.21	24.22
7.05	0.08	0.17	0.44	1.59	3.46	10.03	19.29	32.31	35.75	42.40	32.82	24.28
7.15	0.03	0.14	0.47	1.76	3.62	10.41	20.46	25.36	37.41	38.84	32.66	24.36
7.25	0.07	0.15	0.51	1.60	4.19	10.84	19.90	30.19	35.81	38.49	34.54	24.44
7.35	0.03	0.17	0.61	1.72	3.49	11.25	19.67	27.63	35.90	39.32	32.16	24.52
7.45	0.03	0.24	0.42	1.67	3.99	10.78	20.34	28.53	38.04	41.75	39.74	24.60
7.55	0.03	0.19	0.52	1.61	3.99	10.25	19.62	29.89	35.44	39.68	37.03	24.69
7.65	0.04	0.25	0.53	1.53	3.51	10.59	20.67	33.66	36.74	41.99	38.36	24.79
7.75	0.03	0.20	0.43	1.54	3.81	11.50	20.34	31.55	37.01	43.71	38.30	24.89
7.85	0.05	0.24	0.40	1.50	3.86	11.25	19.95	27.47	38.07	40.62	39.80	24.99
7.95	0.05	0.16	0.54	1.76	4.39	10.14	18.88	27.93	38.40	42.11	35.31	25.10
8.05	0.03	0.16	0.49	1.56	3.71	10.30	19.44	27.63	38.62	40.92	38.86	25.21
8.15	0.03	0.18	0.49	1.43	4.01	9.02	19.34	29.13	38.80	38.55	39.74	25.33
8.25	0.03	0.11	0.47	1.64	3.47	9.66	20.34	27.63	37.35	39.79	37.31	25.45
8.35	0.04	0.20	0.48	1.59	3.64	8.80	18.73	27.78	34.24	38.31	39.91	25.58
8.45	0.04	0.12	0.40	1.38	3.14	9.66	17.70	27.47	34.15	37.78	36.81	25.71
8.55	0.03	0.15	0.28	1.04	3.15	8.05	17.24	24.61	33.12	38.37	33.93	25.85
8.65	0.03	0.15	0.40	1.27	2.68	8.28	15.56	26.57	33.45	36.59	34.26	25.99

Midpt.	11000	2700	910	240	84	26	8.8	2.7	0.92	0.31	0.05	Volume
8.75	0.03	0.10	0.38	1.01	3.05	7.62	15.23	27.02	30.25	36.35	32.38	26.14
8.85	0.02	0.13	0.25	1.03	2.53	7.14	13.82	24.15	28.11	36.06	30.00	26.29
8.95	0.03	0.04	0.27	0.81	2.48	6.61	13.67	21.74	28.38	31.61	29.17	26.44
9.05	0.01	0.09	0.34	0.86	2.52	6.20	12.72	19.47	26.48	29.89	26.35	26.60
9.15	0.02	0.08	0.31	0.82	2.42	5.79	12.42	17.81	25.48	27.64	25.52	26.77
9.25	0.01	0.07	0.23	0.62	1.85	5.12	11.24	19.78	23.01	25.26	25.13	26.94
9.35	0.00	0.04	0.18	0.58	1.96	5.30	10.14	18.72	22.25	23.37	22.53	27.12
9.45	0.02	0.03	0.12	0.64	1.95	4.64	9.07	16.76	20.71	23.07	19.48	27.30
9.55	0.03	0.08	0.18	0.57	1.34	4.16	9.58	14.49	18.27	20.93	18.99	27.49
9.65	0.00	0.03	0.20	0.55	1.29	3.98	7.82	13.74	17.72	21.71	19.37	27.69
9.75	0.00	0.02	0.12	0.49	1.43	3.59	7.97	13.28	17.18	18.15	16.77	27.89
9.85	0.00	0.02	0.12	0.41	1.29	3.27	7.41	9.66	16.06	18.86	18.16	28.09
9.95	0.02	0.03	0.10	0.40	1.17	3.30	6.92	13.28	15.49	17.91	15.66	28.31
10.05	0.01	0.03	0.08	0.30	1.12	2.71	7.46	11.02	15.04	16.37	14.83	28.52
10.15	0.00	0.02	0.12	0.31	1.01	2.84	5.90	10.57	14.61	15.18	15.06	28.75
10.25	0.00	0.00	0.09	0.25	0.77	2.50	6.16	10.72	13.44	14.94	14.11	28.98
10.35	0.01	0.02	0.05	0.30	0.77	2.48	6.77	10.42	11.99	13.05	12.84	29.21
10.45	0.01	0.01	0.07	0.25	0.87	1.95	5.03	6.79	11.41	14.94	14.78	29.46
10.55	0.00	0.03	0.08	0.23	0.60	2.16	5.03	9.66	10.93	12.69	12.62	29.71
10.65	0.00	0.02	0.03	0.29	0.55	2.14	4.39	9.51	10.72	13.28	12.18	29.96
10.75	0.01	0.00	0.07	0.22	0.89	2.02	4.09	5.43	9.87	13.11	12.40	30.23
10.85	0.00	0.03	0.05	0.29	0.70	1.89	4.39	5.89	10.17	12.57	11.35	30.50
10.95	0.00	0.01	0.05	0.21	0.72	1.59	4.04	6.94	10.27	11.74	10.63	30.77
11.05	0.00	0.02	0.08	0.19	0.42	1.50	4.06	6.34	8.88	10.50	10.90	31.06
11.15	0.00	0.02	0.04	0.17	0.37	1.59	3.93	5.74	9.09	10.56	9.19	31.35
11.25	0.00	0.02	0.06	0.17	0.40	1.61	3.07	7.10	8.73	10.62	8.25	31.65
11.35	0.01	0.00	0.07	0.12	0.45	1.62	3.65	7.40	7.79	10.02	8.36	31.96
11.45	0.01	0.00	0.04	0.20	0.44	1.30	2.86	6.64	7.94	9.19	9.63	32.27
11.55	0.00	0.02	0.02	0.14	0.39	1.32	2.78	6.79	7.94	9.79	8.75	32.60
11.65	0.00	0.01	0.02	0.16	0.52	1.29	2.73	5.13	7.70	8.84	8.69	32.93
11.75	0.01	0.02	0.01	0.14	0.40	1.34	2.25	4.53	6.40	8.90	8.58	33.27
11.85	0.00	0.01	0.02	0.11	0.29	1.07	2.45	4.08	7.28	9.07	7.08	33.62
11.95	0.00	0.02	0.02	0.11	0.17	1.00	2.50	4.83	6.61	8.36	7.47	33.98
12.05	0.00	0.01	0.05	0.08	0.32	1.11	2.25	4.83	5.86	7.47	6.92	34.34
12.15	0.00	0.00	0.02	0.12	0.22	1.18	2.30	4.38	6.10	8.24	7.53	34.72
12.25	0.00	0.00	0.02	0.12	0.29	0.84	1.97	4.38	6.79	6.88	7.58	35.10
12.35	0.01	0.01	0.05	0.08	0.30	1.11	1.92	4.08	5.80	7.77	7.53	35.50
12.45	0.02	0.01	0.01	0.09	0.18	0.89	1.92	3.02	5.86	7.59	7.08	35.90
12.55	0.00	0.02	0.03	0.07	0.29	0.96	2.02	3.02	5.40	6.46	6.70	36.32
12.65	0.00	0.00	0.02	0.10	0.20	0.84	2.27	3.92	4.77	7.29	6.20	36.74
12.75	0.00	0.01	0.03	0.12	0.18	0.79	1.84	3.47	4.53	8.18	6.53	37.18
12.85	0.01	0.01	0.02	0.09	0.35	0.70	2.15	3.32	5.74	7.47	6.37	37.63
12.95	0.00	0.01	0.02	0.07	0.30	0.68	1.53	3.47	5.71	6.58	7.03	38.09
13.05	0.00	0.00	0.02	0.09	0.17	0.66	2.20	3.47	4.65	6.17	5.87	38.56
13.15	0.01	0.02	0.02	0.05	0.15	0.75	1.41	3.62	5.07	7.29	6.42	39.04

Midpt.	11000	2700	910	240	84	26	8.8	2.7	0.92	0.31	0.05	Volume
13.25	0.00	0.01	0.03	0.07	0.15	0.46	1.94	3.92	5.10	7.47	5.98	39.54
13.35	0.00	0.01	0.02	0.09	0.29	0.57	1.43	2.57	4.47	6.88	6.81	40.04
13.45	0.00	0.02	0.02	0.06	0.18	0.62	1.35	2.42	5.56	6.11	5.09	40.56
13.55	0.00	0.00	0.02	0.09	0.13	0.62	1.51	3.62	5.65	6.70	6.20	41.10
13.65	0.00	0.00	0.02	0.09	0.20	0.66	1.51	3.02	4.98	6.29	5.31	41.65
13.75	0.00	0.01	0.02	0.03	0.23	0.66	1.63	3.17	5.22	6.46	5.04	42.21
13.85	0.01	0.01	0.02	0.05	0.27	0.70	1.46	2.57	4.71	4.74	6.53	42.78
13.95	0.00	0.01	0.02	0.04	0.18	0.55	1.18	3.77	4.41	5.87	6.03	43.38
14.05	0.00	0.02	0.04	0.07	0.17	0.70	1.53	2.72	4.83	6.82	5.09	43.98
14.15	0.00	0.01	0.02	0.06	0.15	0.52	1.48	3.02	4.68	6.23	6.31	44.61
14.25	0.01	0.01	0.02	0.04	0.12	0.39	1.12	3.02	3.89	6.52	4.87	45.25
14.35	0.00	0.00	0.02	0.10	0.29	0.59	1.33	3.32	4.44	5.87	5.76	45.91
14.45	0.00	0.00	0.02	0.06	0.18	0.66	1.35	3.02	5.56	5.57	4.54	46.58
14.55	0.00	0.01	0.02	0.07	0.25	0.62	1.28	2.87	4.77	6.70	5.76	47.28
14.65	0.01	0.01	0.05	0.06	0.17	0.70	1.48	4.08	4.11	5.52	5.54	47.99
14.75	0.00	0.01	0.01	0.08	0.20	0.70	1.51	3.17	4.59	6.17	5.31	48.72
14.85	0.00	0.00	0.00	0.07	0.10	0.68	1.35	3.62	4.77	6.52	4.76	49.48
14.95	0.00	0.02	0.02	0.07	0.17	0.45	1.56	2.11	4.35	5.69	5.26	50.25
15.05	0.01	0.00	0.02	0.08	0.18	0.50	1.10	3.02	4.71	5.63	5.26	51.05
15.15	0.00	0.02	0.02	0.07	0.20	0.54	1.74	1.21	5.04	6.64	4.93	51.87
15.25	0.01	0.02	0.00	0.08	0.20	0.34	1.15	3.02	4.35	6.35	4.98	52.72
15.35	0.00	0.01	0.01	0.05	0.12	0.43	1.15	2.26	4.59	4.92	4.26	53.59
15.45	0.00	0.01	0.01	0.12	0.35	0.54	1.58	3.02	3.53	3.85	4.37	54.48
15.55	0.00	0.02	0.07	0.09	0.22	0.61	1.41	2.42	4.23	4.57	2.93	55.41
15.65	0.00	0.01	0.01	0.11	0.20	0.55	1.23	3.32	3.89	4.09	3.76	56.36
15.75	0.00	0.01	0.03	0.11	0.22	0.54	1.28	2.26	3.62	3.62	4.21	57.34
15.85	0.00	0.01	0.02	0.12	0.18	0.86	1.41	1.96	2.93	3.80	3.32	58.35
15.95	0.00	0.02	0.02	0.06	0.25	0.62	1.02	2.42	3.11	4.33	3.60	59.40
16.05	0.00	0.00	0.01	0.05	0.15	0.64	1.30	2.11	4.05	4.63	3.43	60.48
16.15	0.01	0.00	0.03	0.15	0.20	0.68	1.05	2.11	2.84	4.15	2.93	61.60
16.25	0.00	0.00	0.04	0.08	0.20	0.54	1.00	3.62	3.74	5.28	4.10	62.76
16.35	0.00	0.01	0.02	0.12	0.23	0.68	1.35	1.66	3.41	5.63	4.98	63.95
16.45	0.00	0.01	0.02	0.08	0.29	0.62	1.38	1.51	3.80	5.22	4.59	65.19
16.55	0.00	0.02	0.00	0.07	0.29	0.52	1.18	2.57	4.23	5.81	2.27	66.48
16.65	0.00	0.00	0.02	0.09	0.20	0.66	1.41	1.06	3.47	4.03	2.38	67.81
16.75	0.00	0.01	0.01	0.10	0.42	0.61	1.20	1.21	3.53	4.27	4.59	69.20
16.85	0.01	0.00	0.02	0.07	0.18	0.73	1.35	3.32	4.32	5.57	4.82	70.64
16.95	0.00	0.01	0.01	0.12	0.08	0.55	1.41	2.42	4.77	5.04	3.99	72.14
17.05	0.00	0.02	0.03	0.12	0.39	0.80	1.38	2.57	3.74	5.40	4.54	73.70
17.15	0.00	0.01	0.05	0.09	0.20	0.64	1.28	3.62	4.14	5.87	4.37	75.33
17.25	0.00	0.02	0.01	0.05	0.25	0.66	1.48	3.32	4.38	5.87	4.26	77.04
17.35	0.00	0.01	0.02	0.10	0.17	0.80	1.48	3.47	4.65	5.81	4.93	78.83
17.45	0.00	0.00	0.04	0.10	0.34	0.79	1.61	1.96	4.62	6.23	4.98	80.70
17.55	0.01	0.00	0.04	0.11	0.22	0.89	1.58	4.53	4.47	5.69	5.65	82.67
17.65	0.00	0.00	0.02	0.11	0.30	0.80	1.97	3.17	4.68	4.86	3.87	84.75

Midpt.	11000	2700	910	240	84	26	8.8	2.7	0.92	0.31	0.05	Volume
17.75	0.00	0.00	0.05	0.06	0.22	0.86	1.79	4.38	5.10	7.35	5.59	86.94
17.85	0.00	0.00	0.02	0.08	0.34	0.66	1.66	4.23	5.01	4.63	3.87	89.27
17.95	0.00	0.01	0.02	0.10	0.32	0.80	1.61	3.17	5.68	7.35	5.54	91.74
18.05	0.01	0.00	0.02	0.10	0.32	0.75	1.48	3.47	5.07	7.12	5.42	94.39
18.15	0.00	0.00	0.03	0.12	0.22	0.75	1.53	3.17	5.71	5.75	7.03	97.24
18.25	0.01	0.00	0.02	0.17	0.37	0.77	1.97	3.62	6.16	7.47	6.97	100.32
18.35	0.01	0.01	0.03	0.14	0.29	1.02	1.63	5.43	5.68	7.12	6.25	103.68
18.45	0.00	0.01	0.04	0.14	0.25	0.75	2.09	2.87	6.22	7.00	7.08	107.38
18.55	0.00	0.00	0.01	0.12	0.29	0.71	1.84	3.47	5.86	6.58	6.64	111.53
18.65	0.00	0.00	0.03	0.19	0.42	0.93	1.84	4.83	6.55	8.36	6.92	116.27
18.75	0.00	0.02	0.02	0.12	0.44	0.80	2.22	3.92	6.22	8.07	6.81	121.87
18.85	0.00	0.00	0.02	0.13	0.25	0.86	2.40	5.13	6.34	8.90	6.37	128.90
18.95	0.00	0.02	0.04	0.13	0.27	1.04	2.53	4.68	7.37	8.12	7.92	139.22
19.05	0.00	0.01	0.04	0.17	0.29	1.05	2.53	6.04	8.24	11.39	6.31	12225.45
19.15	0.03	0.08	0.23	1.06	2.80	8.36	20.08	47.25	81.97	113.15	62.55	2239.26
19.25	0.05	0.07	0.24	1.13	3.15	9.64	24.93	56.76	102.95	131.95	75.33	2239.26
19.35	0.00	0.11	0.40	1.17	3.19	9.57	25.14	59.48	103.65	140.97	1737.64	2239.26
19.45	0.05	0.07	0.26	1.00	3.42	9.37	25.37	59.18	103.07	133.38	1739.47	2239.26
19.55	0.04	0.08	0.29	1.15	3.27	9.68	25.50	61.44	101.81	134.50	78.32	2239.26
19.65	0.04	0.10	0.28	1.19	3.19	9.27	24.58	61.74	100.57	137.11	80.04	2239.26
19.75	0.07	0.12	0.23	1.25	3.00	10.53	24.29	57.67	104.07	139.72	79.98	2239.26
19.85	0.05	0.10	0.26	1.01	3.22	9.75	24.17	59.93	102.26	136.10	78.32	2239.26
19.95	0.02	0.15	0.31	1.04	3.15	9.82	24.24	52.23	97.82	135.57	72.01	2239.26
20.05	0.03	0.10	0.31	1.09	3.05	10.03	25.04	56.31	100.87	131.18	75.72	2239.26
20.15	0.03	0.11	0.22	1.18	3.05	9.48	24.88	59.02	103.01	133.44	73.73	2239.26
20.25	0.02	0.09	0.28	1.17	3.20	9.23	25.65	65.67	102.56	132.37	73.84	2239.26
20.35	0.03	0.05	0.27	1.17	2.90	9.66	25.14	57.06	102.80	133.38	74.83	2239.26
20.45	0.00	0.06	0.33	1.11	2.60	9.25	24.14	58.57	101.14	134.50	75.61	2239.26
20.55	0.02	0.08	0.16	1.13	3.07	9.05	23.81	56.46	96.43	132.37	73.95	2239.26
20.65	0.05	0.08	0.25	0.99	3.57	9.55	22.84	54.50	97.40	130.89	1740.30	2239.26
20.75	0.03	0.11	0.22	1.32	2.80	8.96	24.55	55.25	100.15	138.30	79.15	2239.26
20.85	0.01	0.10	0.24	1.04	3.52	9.11	23.94	55.10	97.58	131.48	74.61	2239.26
20.95	0.03	0.07	0.31	1.22	2.83	8.70	24.12	60.38	99.66	129.05	77.66	2239.26
21.05	0.03	0.11	0.25	1.13	3.02	9.57	24.55	59.18	101.90	130.89	75.72	2239.26
21.15	0.02	0.15	0.21	1.00	3.30	9.68	23.50	53.59	99.66	134.09	76.88	2239.26
21.25	0.03	0.06	0.35	1.07	2.75	9.84	25.27	58.12	100.60	132.61	77.27	2239.26
21.35	0.01	0.10	0.34	1.17	3.12	9.37	22.81	56.31	103.53	131.89	74.50	2239.26
21.45	0.04	0.11	0.27	1.11	2.58	9.68	24.04	58.87	97.10	134.74	75.11	2239.26
21.55	0.03	0.09	0.28	0.94	2.97	9.86	23.84	59.48	98.82	135.69	79.43	2239.26
21.65	0.04	0.07	0.25	0.96	3.19	9.23	24.78	57.67	98.27	134.03	1735.26	2239.26
21.75	0.03	0.13	0.38	1.00	3.34	9.32	24.91	63.40	98.00	130.35	1731.83	2239.26
21.85	0.03	0.16	0.28	1.04	3.39	8.18	24.01	57.67	101.44	141.26	79.60	2239.26
21.95	0.03	0.07	0.29	1.12	3.49	8.78	24.09	59.33	97.64	132.78	74.23	2239.26
22.05	0.04	0.08	0.26	1.14	2.83	9.20	24.22	56.31	96.19	138.12	75.67	2239.26
22.15	0.05	0.09	0.31	1.02	2.97	8.62	23.22	57.21	96.43	136.64	69.96	2239.26

Midpt.	11000	2700	910	240	84	26	8.8	2.7	0.92	0.31	0.05	Volume
22.25	0.02	0.08	0.22	0.96	2.94	9.36	23.89	59.78	102.77	134.56	74.61	2239.26
22.35	0.03	0.12	0.28	1.22	2.57	8.96	24.55	53.59	98.97	135.22	74.83	2239.26
22.45	0.00	0.08	0.24	1.05	2.68	8.96	23.89	58.72	99.21	131.42	79.76	2239.26
22.55	0.01	0.05	0.32	1.13	2.94	8.78	24.19	55.70	97.25	135.57	75.00	2239.26
22.65	0.05	0.06	0.32	0.95	2.85	8.96	23.73	53.74	97.49	125.73	75.61	2239.26
22.75	0.04	0.14	0.27	1.32	3.02	8.62	22.12	57.36	96.79	129.23	73.01	2239.26
22.85	0.03	0.11	0.34	1.15	2.63	8.84	22.79	53.89	96.25	127.80	73.56	2239.26
22.95	0.03	0.08	0.25	1.13	2.85	8.28	23.99	59.93	98.24	129.94	76.11	2239.26
23.05	0.03	0.11	0.21	1.04	3.09	8.77	23.68	58.72	94.62	132.61	77.49	2239.26
23.15	0.02	0.08	0.29	1.03	2.83	8.77	23.43	53.59	100.42	129.52	70.30	2239.26
23.25	0.03	0.11	0.28	1.04	3.07	8.62	23.35	54.80	101.56	126.62	73.06	2239.26
23.35	0.03	0.10	0.31	0.97	2.77	7.84	23.30	58.27	97.34	131.54	75.06	2239.26
23.45	0.03	0.11	0.38	0.91	2.88	8.78	22.61	60.08	100.81	133.91	76.50	2239.26
23.55	0.01	0.12	0.28	1.08	2.57	8.55	21.77	51.93	98.52	134.44	76.94	2239.26
23.65	0.03	0.10	0.31	1.04	2.99	8.62	22.10	53.59	94.68	131.95	73.17	2239.26
23.75	0.02	0.09	0.35	0.97	2.63	9.12	23.04	52.68	97.91	131.48	72.73	2239.26
23.85	0.05	0.08	0.27	0.89	2.88	9.57	22.38	60.99	97.19	126.62	72.07	2239.26
23.95	0.02	0.07	0.24	1.18	2.62	8.02	22.74	52.38	96.82	128.75	1737.64	2239.26
24.05	0.05	0.06	0.26	1.05	2.99	8.66	21.92	52.68	96.31	131.48	73.17	2239.26
24.15	0.03	0.12	0.28	1.04	2.94	8.55	22.33	53.29	96.95	134.33	73.73	2239.26
24.25	0.03	0.10	0.33	1.10	2.97	8.89	21.46	56.31	94.41	126.97	73.45	2239.26
24.35	0.03	0.07	0.30	1.05	2.73	8.82	22.69	53.14	98.55	130.12	1736.98	2239.26
24.45	0.03	0.10	0.30	0.91	2.88	8.37	23.04	55.40	98.18	125.31	1739.91	2239.26
24.55	0.03	0.08	0.30	1.15	2.87	9.18	21.84	55.85	94.26	130.95	75.67	2239.26
24.65	0.03	0.07	0.28	1.19	2.62	8.11	22.81	56.46	96.64	126.44	73.62	2239.26
24.75	0.04	0.06	0.24	1.03	2.85	9.25	22.17	52.53	96.64	132.90	74.61	2239.26
24.85	0.01	0.11	0.28	1.11	2.50	9.02	23.38	54.19	94.23	131.30	77.16	2239.26
24.95	0.02	0.12	0.27	1.01	2.67	8.75	21.13	58.57	99.57	134.39	73.95	2239.26
25.05	0.01	0.06	0.21	0.91	2.38	9.77	21.18	52.68	97.94	133.61	76.11	2239.26
25.15	0.02	0.09	0.24	1.04	2.62	9.14	22.05	53.29	94.80	127.68	76.55	2239.26
25.25	0.02	0.07	0.29	1.18	2.88	8.77	22.40	51.02	94.44	131.00	79.54	2239.26
25.35	0.03	0.07	0.28	0.97	3.04	9.09	20.80	58.12	98.70	125.07	77.77	2239.26
25.45	0.05	0.08	0.31	1.04	2.68	8.27	22.69	56.16	96.79	126.56	75.11	2239.26
25.55	0.04	0.07	0.25	1.00	2.94	8.02	21.89	54.95	94.98	128.93	72.73	2239.26
25.65	0.01	0.10	0.25	0.84	2.60	8.28	21.77	57.36	96.34	131.30	73.29	2239.26
25.75	0.01	0.11	0.24	0.99	2.88	8.34	23.50	59.48	93.96	130.06	75.11	2239.26
25.85	0.02	0.11	0.32	0.80	2.63	8.48	22.79	61.14	95.25	127.27	76.05	2239.26
25.95	0.03	0.11	0.27	1.11	2.80	8.14	22.71	57.97	96.43	127.68	75.78	2239.26
26.05	0.03	0.11	0.28	1.10	2.60	8.73	22.92	51.48	95.68	128.75	76.22	2239.26
26.15	0.04	0.11	0.26	0.99	2.78	9.36	22.89	56.76	96.88	137.59	74.39	2239.26
26.25	0.05	0.09	0.27	1.04	3.04	8.41	22.56	54.65	95.68	129.88	75.06	2239.26
26.35	0.03	0.11	0.24	1.03	2.48	8.48	22.12	57.67	95.74	133.38	1735.82	2239.26
26.45	0.01	0.10	0.24	1.00	2.70	8.37	21.94	59.48	97.37	131.83	76.50	2239.26
26.55	0.03	0.11	0.26	0.95	2.63	8.34	22.30	54.04	93.53	135.87	72.95	2239.26
26.65	0.03	0.07	0.24	1.04	2.72	8.59	22.40	52.68	95.59	133.26	73.29	2239.26

Midpt.	11000	2700	910	240	84	26	8.8	2.7	0.92	0.31	0.05	Volume
26.75	0.03	0.11	0.24	1.10	3.02	8.18	22.51	56.76	97.97	131.95	76.50	2239.26
26.85	0.03	0.05	0.33	1.00	2.65	8.53	23.40	50.42	97.19	131.42	72.57	2239.26
26.95	0.01	0.11	0.19	1.28	2.92	8.62	21.71	55.25	98.06	138.00	77.33	2239.26
27.05	0.02	0.07	0.25	0.94	2.97	8.32	23.07	56.01	98.79	132.13	78.49	2239.26
27.15	0.05	0.02	0.30	1.05	2.83	7.77	21.94	54.65	100.90	130.83	78.54	2239.26
27.25	0.02	0.08	0.28	1.10	3.07	7.82	21.33	55.40	99.18	129.94	81.59	2239.26
27.35	0.01	0.08	0.20	1.04	2.75	8.93	23.09	55.25	99.30	128.87	77.55	2239.26
27.45	0.03	0.10	0.32	1.07	2.53	8.77	21.79	55.70	98.55	127.09	75.33	2239.26
27.55	0.03	0.11	0.28	0.97	2.78	8.52	22.79	53.89	96.88	134.21	75.22	2239.26
27.65	0.03	0.05	0.24	0.96	2.78	8.43	22.69	52.53	94.26	130.12	75.17	2239.26
27.75	0.04	0.07	0.30	1.01	2.75	9.20	22.56	58.12	96.76	129.40	76.27	2239.26
27.85	0.08	0.12	0.29	0.96	2.58	8.61	24.19	53.29	99.96	136.94	80.87	2239.26
27.95	0.01	0.09	0.24	0.91	2.55	8.52	21.87	50.72	98.73	135.45	75.78	2239.26
28.05	0.02	0.11	0.31	0.95	2.73	9.30	22.74	59.33	96.95	133.32	79.37	2239.26
28.15	0.03	0.03	0.37	1.07	2.65	8.87	23.78	54.04	98.52	131.42	77.99	2239.26
28.25	0.03	0.07	0.32	0.99	2.35	8.27	23.76	59.63	98.33	135.33	78.27	2239.26
28.35	0.01	0.05	0.32	0.90	2.87	8.46	23.27	52.84	100.36	134.27	79.60	2239.26
28.45	0.03	0.11	0.22	0.95	2.42	9.07	22.51	54.95	101.05	132.25	79.71	2239.26
28.55	0.04	0.07	0.21	0.86	2.48	8.75	22.25	60.53	99.03	131.54	1742.24	2239.26
28.65	0.04	0.11	0.24	0.94	2.38	8.62	22.17	57.97	98.27	134.15	82.09	2239.26
28.75	0.02	0.06	0.23	0.99	2.99	8.91	23.45	54.95	98.91	134.98	82.31	2239.26
28.85	0.04	0.04	0.27	1.07	2.60	7.82	24.14	54.80	100.66	133.02	78.43	2239.26
28.95	0.02	0.07	0.25	1.08	2.82	8.32	24.14	56.61	102.08	135.27	78.88	2239.26
29.05	0.02	0.09	0.24	1.21	2.57	8.91	21.87	60.08	101.23	131.00	75.61	2239.26
29.15	0.03	0.07	0.25	0.96	2.94	8.11	22.61	51.17	103.80	134.98	1739.97	2239.26
29.25	0.01	0.08	0.22	0.99	2.87	9.00	23.63	59.48	101.20	134.86	80.26	2239.26
29.35	0.03	0.12	0.22	1.00	2.38	8.73	23.12	55.70	101.81	133.20	80.20	2239.26
29.45	0.01	0.11	0.25	1.05	2.92	8.71	22.20	55.70	101.17	137.23	81.03	2239.26
29.55	0.06	0.11	0.18	1.07	2.30	8.75	23.12	53.14	101.20	133.08	1739.97	2239.26
29.65	0.04	0.09	0.27	1.13	2.52	8.55	22.20	54.19	98.42	138.66	82.92	2239.26
29.75	0.02	0.12	0.28	0.97	2.94	8.87	21.77	55.25	99.87	136.16	78.99	2239.26
29.85	0.02	0.11	0.26	0.99	3.20	8.32	23.32	56.01	101.35	131.95	85.19	2239.26
29.95	0.03	0.14	0.32	1.32	2.47	9.46	22.12	54.19	100.21	136.46	83.97	2239.26
30.05	0.04	0.11	0.32	1.22	2.85	8.11	23.32	62.04	103.38	131.89	81.98	2239.26
30.15	0.03	0.08	0.29	0.79	2.82	8.41	24.55	58.57	101.08	136.64	84.02	2239.26
30.25	0.02	0.11	0.28	1.10	3.10	8.86	24.22	57.52	101.38	130.71	87.29	2239.26
30.35	0.01	0.09	0.32	0.97	2.63	9.12	23.17	52.38	99.63	135.45	81.59	2239.26
30.45	0.01	0.13	0.26	0.90	2.55	9.18	22.92	58.27	101.11	133.26	78.99	2239.26
30.55	0.03	0.11	0.24	0.85	2.68	8.14	24.58	57.06	102.38	132.43	82.42	2239.26
30.65	0.02	0.12	0.20	0.85	2.53	8.37	22.23	55.40	103.35	132.72	83.58	2239.26
30.75	0.01	0.11	0.28	0.93	2.77	8.78	23.61	60.53	99.57	134.09	83.91	2239.26
30.85	0.03	0.07	0.25	1.13	3.22	9.18	24.04	57.52	101.44	140.49	83.08	2239.26
30.95	0.03	0.04	0.33	1.06	2.70	9.00	22.81	60.23	97.76	138.71	89.39	2239.26
31.05	0.04	0.10	0.22	0.99	2.87	8.91	24.09	59.93	100.21	142.04	83.91	2239.26
31.15	0.04	0.12	0.23	1.09	2.57	9.20	23.30	58.87	102.35	132.31	88.56	2239.26

Midpt.	11000	2700	910	240	84	26	8.8	2.7	0.92	0.31	0.05	Volume
31.25	0.03	0.15	0.27	0.88	2.70	8.75	22.10	61.74	102.65	142.27	80.31	2239.26
31.35	0.00	0.06	0.30	1.15	2.35	8.62	24.91	55.10	97.79	140.20	87.12	2239.26
31.45	0.03	0.09	0.23	1.02	2.52	8.66	23.22	55.25	101.20	137.41	84.63	2239.26
31.55	0.01	0.08	0.28	1.09	2.50	8.73	24.12	59.78	101.23	135.27	84.47	2239.26
31.65	0.05	0.13	0.21	1.01	2.68	7.87	22.89	56.16	101.90	143.10	88.95	2239.26
31.75	0.00	0.11	0.25	0.88	2.97	8.66	23.12	57.67	99.63	134.44	81.03	2239.26
31.85	0.03	0.09	0.29	1.00	2.88	9.05	22.53	60.69	99.75	136.05	83.36	2239.26
31.95	0.04	0.07	0.33	0.94	2.50	8.59	24.65	55.10	104.16	135.33	82.42	2239.26
32.05	0.02	0.11	0.33	1.01	2.37	9.02	22.07	57.97	100.63	139.84	84.41	2239.26
32.15	0.01	0.09	0.27	0.79	2.80	9.05	22.84	57.06	100.63	135.22	83.30	2239.26
32.25	0.01	0.11	0.27	0.97	2.68	8.89	23.76	58.12	101.35	135.81	82.58	2239.26
32.35	0.02	0.10	0.20	1.18	2.68	8.93	22.53	57.36	103.41	139.54	81.48	2239.26
32.45	0.02	0.11	0.26	0.99	2.83	8.91	23.84	57.82	100.69	134.27	87.40	2239.26
32.55	0.02	0.11	0.25	1.22	3.19	8.02	22.71	56.31	100.81	140.32	84.85	2239.26
32.65	0.07	0.13	0.24	0.99	2.83	8.61	22.53	66.72	103.86	132.67	83.58	2239.26
32.75	0.03	0.11	0.30	0.94	2.85	8.78	21.20	57.21	102.44	137.05	89.39	2239.26
32.85	0.01	0.11	0.26	0.86	2.68	8.39	22.79	57.21	102.68	138.12	82.36	2239.26
32.95	0.03	0.06	0.36	0.98	2.75	8.66	22.79	57.06	105.01	139.07	87.46	2239.26
33.05	0.04	0.11	0.22	0.99	2.67	8.80	22.07	56.31	104.19	132.13	1745.39	2239.26
33.15	0.02	0.09	0.34	0.92	2.70	8.70	23.27	55.10	102.44	141.32	84.24	2239.26
33.25	0.02	0.11	0.26	0.87	2.94	8.53	22.92	57.06	100.63	138.30	83.03	2239.26
33.35	0.03	0.07	0.28	1.02	2.85	8.23	22.12	53.89	98.64	139.43	88.67	2239.26
33.45	0.05	0.09	0.24	1.04	2.99	8.80	23.09	58.42	102.11	139.43	88.73	2239.26
33.55	0.02	0.11	0.35	0.95	2.92	8.77	22.48	58.27	103.50	147.73	82.53	2239.26
33.65	0.00	0.13	0.28	0.85	2.85	8.50	23.63	55.70	99.33	132.96	85.46	2239.26
33.75	0.03	0.09	0.22	0.95	2.47	8.43	22.92	61.59	99.27	137.59	85.68	2239.26
33.85	0.01	0.09	0.31	1.04	3.15	8.86	23.73	58.87	105.31	142.57	86.07	2239.26
33.95	0.02	0.11	0.31	1.21	2.62	9.27	24.32	60.53	100.18	138.95	85.41	2239.26
34.05	0.02	0.15	0.28	1.10	2.90	8.73	23.15	54.65	104.25	141.15	91.16	2239.26
34.15	0.03	0.16	0.32	1.11	2.77	8.46	23.04	58.87	99.66	140.73	83.03	2239.26
34.25	0.03	0.08	0.28	1.06	2.72	8.46	23.66	57.36	100.69	140.20	1749.82	2239.26
34.35	0.03	0.11	0.25	1.03	2.68	8.62	22.86	58.72	103.10	133.14	87.51	2239.26
34.45	0.03	0.08	0.21	1.03	2.92	8.59	23.40	65.21	103.41	136.46	82.86	2239.26
34.55	0.03	0.09	0.31	1.09	3.04	8.98	22.51	62.19	102.89	139.01	83.25	2239.26
34.65	0.01	0.12	0.25	1.02	2.62	7.98	23.27	53.59	105.73	141.15	85.52	2239.26
34.75	0.03	0.10	0.31	0.93	2.72	8.37	22.97	56.46	103.71	133.50	84.08	2239.26
34.85	0.01	0.09	0.29	0.80	2.99	8.52	23.27	55.10	105.16	136.22	91.22	2239.26
34.95	0.03	0.06	0.23	1.01	2.60	8.59	22.30	60.69	101.56	136.76	85.07	2239.26
35.05	0.01	0.14	0.29	1.09	2.78	8.68	24.63	53.74	104.80	139.01	87.84	2239.26
35.15	0.03	0.11	0.28	1.15	2.58	8.86	22.79	57.82	102.02	138.60	91.94	2239.26
35.25	0.03	0.08	0.29	0.96	3.12	8.27	23.94	58.27	101.32	137.59	91.44	2239.26
35.35	0.03	0.08	0.32	0.99	2.75	8.89	24.88	56.31	101.38	134.27	89.00	2239.26
35.45	0.03	0.08	0.33	1.13	2.78	8.62	24.07	54.80	99.06	136.88	86.40	2239.26
35.55	0.03	0.11	0.35	0.95	2.97	8.43	22.74	59.18	100.33	137.47	89.56	2239.26
35.65	0.02	0.10	0.20	0.90	2.73	8.03	24.88	55.70	101.72	142.87	87.01	2239.26

Midpt.	11000	2700	910	240	84	26	8.8	2.7	0.92	0.31	0.05	Volume
35.75	0.02	0.09	0.21	0.95	2.88	8.37	21.79	59.02	100.30	139.31	85.35	2239.26
35.85	0.03	0.06	0.25	0.94	3.09	8.55	24.24	57.82	100.33	136.94	87.40	2239.26
35.95	0.03	0.05	0.30	1.04	2.97	9.14	24.40	60.23	104.46	132.78	89.17	2239.26
36.05	0.04	0.07	0.31	0.85	2.57	8.89	23.20	54.34	100.27	142.33	88.29	2239.26
36.15	0.03	0.13	0.31	0.95	2.80	9.02	23.20	59.33	100.21	131.24	89.89	2239.26
36.25	0.03	0.13	0.29	1.18	2.45	8.45	23.25	64.31	102.02	137.17	88.89	2239.26
36.35	0.03	0.10	0.22	1.14	2.82	9.09	24.86	55.85	101.81	137.11	90.39	2239.26
36.45	0.03	0.05	0.21	1.11	3.07	8.84	24.45	60.23	103.38	138.00	90.67	2239.26
36.55	0.03	0.07	0.31	1.02	2.80	8.53	23.86	61.89	100.42	142.04	83.53	2239.26
36.65	0.03	0.13	0.25	0.98	2.63	8.78	22.56	54.95	101.50	137.47	87.68	2239.26
36.75	0.05	0.10	0.23	1.07	3.09	9.07	23.71	63.55	101.72	137.41	88.40	2239.26
36.85	0.01	0.07	0.19	0.95	2.65	8.84	24.60	56.46	99.09	137.94	85.68	2239.26
36.95	0.01	0.07	0.22	0.90	2.68	8.52	23.68	59.48	99.99	135.04	92.10	2239.26
37.05	0.01	0.07	0.22	1.14	2.99	8.71	22.89	59.78	103.80	136.28	89.17	2239.26
37.15	0.02	0.07	0.31	1.09	2.43	9.03	22.89	58.72	100.69	137.94	95.37	2239.26
37.25	0.03	0.12	0.30	0.83	3.24	8.23	23.15	62.35	99.93	146.01	86.18	2239.26
37.35	0.03	0.06	0.31	0.92	2.88	9.16	23.84	56.61	101.14	142.51	90.39	2239.26
37.45	0.03	0.07	0.27	0.98	2.57	8.77	23.53	56.01	103.89	143.46	84.47	2239.26
37.55	0.01	0.08	0.21	0.88	2.55	8.84	23.38	60.53	99.63	137.59	89.00	2239.26
37.65	0.01	0.09	0.35	0.93	2.55	8.07	24.78	58.27	106.24	139.60	89.00	2239.26
37.75	0.03	0.07	0.32	1.06	3.09	8.55	24.17	57.21	106.61	140.85	88.45	2239.26
37.85	0.04	0.08	0.24	1.09	2.73	8.59	23.66	54.80	99.57	138.48	92.16	2239.26
37.95	0.01	0.13	0.25	1.09	2.53	8.43	22.56	54.50	99.15	139.31	90.72	2239.26
38.05	0.02	0.11	0.29	0.94	2.31	8.43	23.94	57.52	103.19	139.13	87.29	2239.26
38.15	0.03	0.08	0.30	0.84	2.65	8.95	23.07	60.84	101.66	139.43	88.51	2239.26
38.25	0.03	0.11	0.28	0.96	2.70	7.98	23.35	56.76	104.25	135.81	92.82	2239.26
38.35	0.03	0.06	0.26	0.97	2.77	8.43	22.38	60.99	101.87	132.67	1752.53	2239.26
38.45	0.03	0.10	0.26	1.03	3.29	8.57	23.50	59.63	102.02	139.31	88.73	2239.26
38.55	0.03	0.07	0.30	0.96	2.60	8.71	23.27	56.31	99.84	135.51	84.80	2239.26
38.65	0.01	0.15	0.29	0.96	2.97	8.84	23.78	59.78	98.76	144.94	93.60	2239.26
38.75	0.03	0.07	0.23	0.85	2.97	8.18	23.30	55.40	100.06	135.22	89.84	2239.26
38.85	0.04	0.05	0.24	1.02	2.92	8.46	22.97	63.40	99.06	135.16	85.96	2239.26
38.95	0.04	0.11	0.30	0.99	2.95	8.37	24.19	55.10	98.82	138.83	90.61	2239.26
39.05	0.03	0.07	0.30	0.86	2.72	8.50	22.69	60.84	98.52	135.39	90.22	2239.26
39.15	0.03	0.11	0.23	1.07	2.72	8.37	24.04	61.44	101.38	138.12	88.45	2239.26
39.25	0.03	0.11	0.33	1.03	2.50	8.16	23.20	53.89	101.08	137.59	91.66	2239.26
39.35	0.02	0.09	0.25	1.18	2.83	8.70	23.02	56.91	100.87	140.20	86.68	2239.26
39.45	0.00	0.07	0.30	0.99	3.10	8.89	23.68	56.31	99.96	140.79	90.06	2239.26
39.55	0.03	0.08	0.22	0.96	2.87	8.57	23.02	58.72	101.56	137.29	1749.16	2239.26
39.65	0.06	0.06	0.32	0.87	2.60	8.53	24.37	56.31	99.69	134.27	90.72	2239.26
39.75	0.01	0.04	0.21	1.14	2.75	8.53	22.25	59.02	103.16	129.94	90.67	2239.26
39.85	0.03	0.14	0.25	0.97	2.62	9.05	23.40	57.82	102.98	138.36	87.34	2239.26
39.95	0.01	0.10	0.30	0.94	3.00	9.53	23.73	58.27	99.81	149.09	91.22	2239.26
40.05	0.03	0.11	0.28	1.23	2.67	8.95	24.60	57.21	102.44	135.87	90.22	2239.26
40.15	0.03	0.10	0.23	0.91	2.47	8.00	23.61	46.65	100.51	136.88	86.29	2239.26

Midpt.	11000	2700	910	240	84	26	8.8	2.7	0.92	0.31	0.05	Volume
40.25	0.03	0.09	0.28	0.99	2.65	8.71	23.58	56.91	99.15	143.81	89.72	2239.26
40.35	0.02	0.08	0.27	0.90	3.02	8.86	23.12	53.44	101.11	138.18	90.55	2239.26
40.45	0.02	0.07	0.25	0.99	2.70	8.05	21.43	56.01	104.92	135.39	87.07	2239.26
40.55	0.03	0.08	0.23	1.07	2.97	9.02	22.33	54.50	100.87	137.05	88.12	2239.26
40.65	0.04	0.08	0.27	1.07	2.87	9.03	22.35	58.72	101.90	133.97	93.77	2239.26
40.75	0.02	0.11	0.30	0.92	2.53	9.09	22.40	52.08	101.38	140.02	90.00	2239.26
40.85	0.02	0.05	0.34	0.99	2.68	8.61	23.91	57.36	100.63	133.50	89.23	2239.26
40.95	0.02	0.10	0.33	1.09	2.82	8.07	23.17	52.68	100.30	137.59	88.17	2239.26
41.05	0.01	0.11	0.25	0.96	2.70	8.98	23.40	55.10	101.47	142.39	89.56	2239.26
41.15	0.02	0.11	0.21	1.02	2.97	8.66	23.91	56.46	107.48	138.89	88.45	2239.26
41.25	0.03	0.11	0.25	0.98	2.78	8.61	24.17	51.33	102.05	131.30	95.15	2239.26
41.35	0.04	0.07	0.22	0.95	2.83	8.64	23.76	57.21	104.10	139.01	92.77	2239.26
41.45	0.03	0.09	0.32	1.02	2.78	7.93	23.17	53.29	100.21	138.89	91.00	2239.26
41.55	0.02	0.14	0.15	1.10	2.73	7.95	24.12	52.68	102.59	136.16	94.87	2239.26
41.65	0.00	0.12	0.28	1.09	3.17	7.84	24.07	55.10	104.25	142.63	91.88	2239.26
41.75	0.02	0.04	0.33	0.99	2.72	8.12	22.74	55.10	98.39	134.74	92.66	2239.26
41.85	0.04	0.08	0.34	0.83	2.68	8.78	23.15	59.63	102.29	140.49	86.74	2239.26
41.95	0.02	0.08	0.25	1.05	2.45	9.09	22.48	59.02	100.48	140.49	92.88	2239.26
42.05	0.04	0.07	0.34	1.07	2.90	8.77	23.32	57.67	103.83	132.90	87.73	2239.26
42.15	0.03	0.11	0.23	0.95	2.80	9.21	22.81	59.63	97.55	136.99	96.75	2239.26
42.25	0.02	0.13	0.24	1.13	2.97	8.53	23.35	60.69	99.96	136.10	93.49	2239.26
42.35	0.00	0.06	0.25	1.07	2.52	8.28	23.66	57.97	100.18	135.81	92.55	2239.26
42.45	0.02	0.11	0.34	0.98	2.67	8.73	23.27	60.08	102.86	137.47	89.95	2239.26
42.55	0.03	0.09	0.26	0.89	2.97	8.77	22.97	60.84	102.62	139.90	87.07	2239.26
42.65	0.03	0.13	0.18	0.94	2.67	8.84	23.40	60.99	102.56	137.47	91.33	2239.26
42.75	0.03	0.10	0.25	0.94	2.58	8.64	23.99	59.48	104.31	134.74	95.59	2239.26
42.85	0.03	0.08	0.29	0.99	2.65	9.32	23.25	56.46	98.70	140.08	91.55	2239.26
42.95	0.03	0.09	0.29	0.96	2.63	8.66	23.12	60.53	100.18	143.10	92.10	2239.26
43.05	0.02	0.07	0.27	1.12	2.87	8.59	22.30	55.25	101.84	140.97	93.49	2239.26
43.15	0.02	0.11	0.37	1.15	2.87	8.34	22.86	56.46	103.62	139.43	92.27	2239.26
43.25	0.03	0.07	0.22	1.04	2.85	8.43	23.66	55.10	102.35	135.10	95.92	2239.26
43.35	0.02	0.09	0.24	0.90	2.57	8.11	22.97	62.19	101.17	142.15	92.99	2239.26
43.45	0.00	0.10	0.25	0.93	2.78	9.00	22.00	59.63	104.07	134.09	90.06	2239.26
43.55	0.03	0.13	0.30	1.11	2.67	8.39	23.48	59.48	98.70	137.23	89.67	2239.26
43.65	0.07	0.08	0.37	1.07	3.12	9.00	24.04	55.85	100.02	136.05	94.76	2239.26
43.75	0.02	0.07	0.32	0.86	2.31	8.07	23.30	56.61	104.61	137.29	1753.70	2239.26
43.85	0.02	0.05	0.32	1.13	3.05	9.55	22.66	59.63	99.87	130.06	92.44	2239.26
43.95	0.01	0.14	0.23	1.03	3.29	9.00	23.55	61.44	103.53	136.16	93.05	2239.26
44.05	0.04	0.07	0.29	1.08	2.90	8.80	24.19	58.87	102.74	132.31	92.16	2239.26
44.15	0.02	0.07	0.31	1.10	2.88	8.45	23.07	54.65	103.35	139.66	90.44	2239.26
44.25	0.01	0.10	0.25	0.91	2.52	9.11	24.07	58.27	100.99	138.36	91.72	2239.26
44.35	0.04	0.07	0.28	0.81	2.88	9.28	23.32	59.33	99.42	135.51	94.71	2239.26
44.45	0.04	0.11	0.18	1.04	2.82	8.16	24.09	54.65	98.79	138.24	91.44	2239.26
44.55	0.04	0.07	0.22	0.92	2.78	7.93	23.94	56.76	105.31	131.83	90.44	2239.26
44.65	0.02	0.09	0.22	0.94	3.00	8.11	23.68	62.04	99.99	136.22	97.36	2239.26

Midpt.	11000	2700	910	240	84	26	8.8	2.7	0.92	0.31	0.05	Volume
44.75	0.03	0.07	0.31	0.90	2.73	9.05	23.84	52.84	105.43	137.17	95.92	2239.26
44.85	0.01	0.11	0.25	0.99	2.75	8.78	24.19	58.87	100.54	139.01	96.31	2239.26
44.95	0.02	0.09	0.33	0.89	2.97	8.37	23.94	57.36	103.26	133.02	93.99	2239.26
45.05	0.00	0.10	0.28	1.03	2.72	8.37	22.94	51.78	101.05	135.22	94.76	2239.26
45.15	0.03	0.14	0.28	0.97	2.50	8.75	23.81	59.48	99.96	139.54	1754.30	2239.26
45.25	0.03	0.07	0.18	0.95	2.77	8.86	22.35	57.06	98.36	141.44	98.08	2239.26
45.35	0.02	0.12	0.27	0.92	3.09	8.70	24.29	54.04	103.35	124.54	98.08	2239.26
45.45	0.03	0.07	0.35	0.84	2.83	8.87	23.07	54.04	99.60	137.88	93.77	2239.26
45.55	0.04	0.11	0.32	0.99	2.38	7.93	23.76	60.23	102.11	132.43	95.43	2239.26
45.65	0.03	0.10	0.26	0.94	2.70	8.48	24.70	59.63	97.64	138.83	1756.85	2239.26
45.75	0.02	0.08	0.31	0.99	2.78	8.55	23.71	54.19	100.24	131.24	99.08	2239.26
45.85	0.03	0.07	0.29	0.90	2.47	8.50	23.73	57.52	97.67	137.88	94.43	2239.26
45.95	0.03	0.10	0.28	0.93	2.90	8.30	23.66	55.70	99.33	134.74	89.50	2239.26
46.05	0.03	0.09	0.26	0.91	3.00	8.25	24.88	59.18	99.18	137.29	96.59	2239.26
46.15	0.02	0.11	0.24	1.09	2.50	8.52	24.04	62.50	100.60	138.77	95.31	2239.26
46.25	0.03	0.07	0.20	0.95	2.88	8.39	23.20	56.01	103.38	134.92	1757.79	2239.26
46.35	0.00	0.07	0.30	0.98	2.38	8.34	22.86	58.57	103.26	138.00	98.86	2239.26
46.45	0.00	0.08	0.25	0.99	2.90	8.62	22.63	60.38	100.69	137.94	96.15	2239.26
46.55	0.03	0.13	0.32	0.99	2.68	9.12	22.97	57.82	103.62	133.91	88.78	2239.26
46.65	0.05	0.07	0.19	1.02	3.44	9.11	21.23	60.53	101.11	135.33	98.08	2239.26
46.75	0.02	0.16	0.26	1.04	2.70	8.59	22.63	58.12	100.75	134.15	1760.78	2239.26
46.85	0.03	0.11	0.22	0.97	2.90	9.23	23.09	60.53	102.44	139.13	96.09	2239.26
46.95	0.01	0.06	0.25	1.12	2.43	8.98	23.86	55.70	99.78	132.78	99.69	2239.26
47.05	0.02	0.13	0.28	0.95	2.70	8.39	23.07	55.25	102.23	141.62	92.16	2239.26
47.15	0.03	0.09	0.36	1.06	2.68	8.27	23.91	54.65	103.35	135.33	92.66	2239.26
47.25	0.03	0.06	0.38	0.92	2.88	8.41	23.43	54.65	104.31	132.96	99.91	2239.26
47.35	0.01	0.08	0.30	1.04	2.82	8.50	22.89	52.99	102.14	134.62	1761.78	2239.26
47.45	0.03	0.12	0.22	0.95	2.65	8.61	22.15	54.04	103.10	133.91	97.03	2239.26
47.55	0.03	0.15	0.28	0.83	2.80	8.98	22.38	60.53	100.24	133.38	97.47	2239.26
47.65	0.00	0.10	0.27	0.95	3.22	8.03	24.42	61.74	101.23	140.49	99.30	2239.26
47.75	0.03	0.07	0.25	0.95	2.57	8.45	25.88	59.18	101.05	140.85	98.30	2239.26
47.85	0.03	0.08	0.20	0.95	2.82	8.34	23.96	54.95	101.93	134.86	99.02	2239.26
47.95	0.03	0.10	0.30	0.84	2.94	8.37	21.97	62.50	102.53	135.57	95.15	2239.26
48.05	0.03	0.05	0.25	1.13	3.02	8.89	22.63	56.61	102.62	133.97	98.97	2239.26
48.15	0.00	0.16	0.31	1.15	2.65	8.61	24.35	56.61	105.04	131.12	94.43	2239.26
48.25	0.04	0.09	0.32	1.02	2.80	8.82	23.84	56.46	100.63	143.28	97.03	2239.26
48.35	0.01	0.10	0.28	0.82	2.78	8.28	23.22	57.06	102.59	138.54	102.18	2239.26
48.45	0.03	0.09	0.24	0.99	2.82	8.86	23.73	55.85	102.38	142.27	97.97	2239.26
48.55	0.03	0.10	0.30	0.99	2.73	8.98	23.94	53.44	99.45	133.67	102.34	2239.26
48.65	0.02	0.11	0.25	0.93	2.72	8.59	23.96	55.55	99.96	133.44	93.82	2239.26
48.75	0.04	0.11	0.31	1.05	2.72	9.36	22.89	58.42	103.26	137.29	100.57	2239.26
48.85	0.03	0.07	0.23	0.95	2.68	8.75	23.43	61.29	100.96	134.15	101.13	2239.26
48.95	0.01	0.12	0.32	0.95	2.72	8.66	22.81	58.57	102.68	131.36	99.19	2239.26
49.05	0.02	0.14	0.22	1.09	2.75	7.87	22.89	60.23	103.13	134.50	98.08	2239.26
49.15	0.02	0.11	0.25	0.97	2.78	8.86	23.61	55.40	104.73	141.44	95.98	2239.26

Midpt.	11000	2700	910	240	84	26	8.8	2.7	0.92	0.31	0.05	Volume
49.25	0.03	0.08	0.23	0.87	2.78	8.21	22.63	59.93	100.99	134.98	99.30	2239.26
49.35	0.00	0.09	0.22	1.18	3.20	8.14	23.91	58.72	103.65	138.12	106.11	2239.26
49.45	0.01	0.05	0.28	1.04	2.43	7.91	24.09	57.97	102.11	141.32	97.25	2239.26
49.55	0.03	0.08	0.23	1.17	2.62	8.30	23.17	61.59	100.96	139.13	95.31	2239.26
49.65	0.02	0.09	0.28	0.92	2.75	8.62	22.66	57.06	100.96	136.16	94.93	2239.26
49.75	0.02	0.12	0.31	1.12	2.87	8.82	22.17	60.23	100.36	137.35	96.31	2239.26
49.85	0.03	0.04	0.35	1.13	2.63	8.34	24.01	57.52	100.57	130.29	100.41	2239.26
49.95	0.03	0.10	0.28	1.09	3.12	9.09	24.81	55.55	103.80	137.35	101.02	2239.26
50.05	0.00	0.12	0.24	1.12	2.73	8.57	22.17	62.04	99.27	138.83	97.64	2239.26
50.15	0.03	0.07	0.26	1.00	2.70	8.68	22.58	57.21	100.63	136.10	98.19	2239.26
50.25	0.03	0.11	0.32	0.86	2.70	9.50	24.86	58.12	96.13	139.19	96.37	2239.26
50.35	0.04	0.13	0.16	0.85	2.63	8.45	23.43	53.44	102.05	137.35	100.57	2239.26
50.45	0.03	0.14	0.26	1.01	2.65	9.05	24.04	59.63	102.26	140.08	101.90	2239.26
50.55	0.03	0.08	0.28	0.96	2.82	8.14	22.81	56.61	100.15	133.79	102.34	2239.26
50.65	0.03	0.07	0.33	1.04	2.83	8.98	23.53	59.33	103.53	133.61	99.80	2239.26
50.75	0.05	0.13	0.18	1.01	3.10	8.05	22.53	55.40	103.68	135.51	98.03	2239.26
50.85	0.01	0.16	0.24	1.00	2.72	8.43	21.87	60.23	99.18	135.33	97.14	2239.26
50.95	0.01	0.07	0.24	1.00	2.50	9.57	23.40	60.69	101.87	134.39	97.25	2239.26
51.05	0.03	0.11	0.25	1.05	2.57	8.45	24.68	59.33	101.93	133.50	101.63	2239.26
51.15	0.02	0.11	0.20	0.97	2.78	8.78	22.25	55.85	104.52	142.33	99.13	2239.26
51.25	0.03	0.05	0.31	1.05	2.87	9.41	22.33	57.82	103.19	136.99	100.85	2239.26
51.35	0.03	0.05	0.23	0.98	2.58	8.86	22.74	62.19	104.13	136.64	98.41	2239.26
51.45	0.04	0.13	0.28	1.25	2.57	8.41	24.19	60.08	100.09	134.39	100.96	2239.26
51.55	0.03	0.11	0.28	1.07	2.38	8.52	22.79	55.70	102.53	133.67	94.71	2239.26
51.65	0.02	0.09	0.30	1.11	2.97	9.11	23.09	57.21	99.96	131.06	101.68	2239.26
51.75	0.04	0.07	0.28	1.10	2.50	8.70	23.71	58.57	99.72	136.88	1762.33	2239.26
51.85	0.03	0.15	0.20	0.94	2.55	9.46	23.27	55.40	100.09	141.68	102.84	2239.26
51.95	0.03	0.11	0.23	1.15	2.31	8.73	22.76	64.16	103.16	134.74	98.30	2239.26
52.05	0.03	0.03	0.25	0.95	3.10	8.71	23.09	57.52	100.93	139.49	1769.30	2239.26
52.15	0.05	0.10	0.25	1.09	2.33	8.02	22.84	56.61	103.95	138.18	99.19	2239.26
52.25	0.01	0.07	0.26	1.05	2.35	8.77	22.89	55.10	98.30	134.27	98.03	2239.26
52.35	0.03	0.10	0.20	0.99	3.04	8.75	24.04	59.02	104.22	130.89	102.01	2239.26
52.45	0.01	0.07	0.32	1.20	2.88	7.89	22.56	57.36	99.84	142.57	100.13	2239.26
52.55	0.04	0.11	0.31	1.04	2.92	9.21	23.66	55.85	101.56	134.39	98.47	2239.26
52.65	0.03	0.08	0.21	1.19	3.39	8.75	22.00	52.53	100.63	137.23	100.85	2239.26
52.75	0.03	0.03	0.25	0.95	3.15	8.46	23.86	59.02	101.14	136.82	1761.06	2239.26
52.85	0.00	0.09	0.35	0.91	2.70	7.71	23.17	55.55	105.58	136.82	93.82	2239.26
52.95	0.01	0.10	0.35	1.05	2.82	8.05	24.86	58.57	100.96	135.75	101.29	2239.26
53.05	0.03	0.15	0.25	0.99	2.83	8.43	22.48	58.42	100.81	136.99	102.18	2239.26
53.15	0.01	0.07	0.32	1.14	2.73	8.43	24.83	61.44	99.93	143.34	103.45	2239.26
53.25	0.00	0.11	0.37	1.09	2.70	9.48	23.17	49.06	100.84	138.60	104.84	2239.26
53.35	0.02	0.08	0.28	0.89	2.63	8.57	23.20	53.74	103.68	139.60	103.01	2239.26
53.45	0.03	0.09	0.32	0.91	3.05	9.21	23.91	55.85	101.14	134.74	104.61	2239.26
53.55	0.01	0.07	0.28	0.99	2.47	8.75	23.71	55.25	102.41	139.84	105.06	2239.26
53.65	0.03	0.09	0.31	0.89	2.63	8.71	22.56	61.44	100.02	136.28	101.13	2239.26

Midpt.	11000	2700	910	240	84	26	8.8	2.7	0.92	0.31	0.05	Volume
53.75	0.04	0.10	0.32	1.04	2.97	8.80	21.74	64.16	101.99	139.54	1765.37	2239.26
53.85	0.03	0.11	0.23	0.98	2.72	8.14	22.51	59.33	96.31	135.93	101.79	2239.26
53.95	0.02	0.11	0.21	1.08	3.00	8.34	22.74	60.38	104.76	137.82	106.99	2239.26
54.05	0.02	0.07	0.24	1.06	3.34	9.14	22.99	57.36	103.89	132.01	104.95	2239.26
54.15	0.02	0.11	0.24	1.02	2.72	8.50	23.20	52.84	100.93	137.41	107.55	2239.26
54.25	0.03	0.12	0.22	0.89	2.50	8.39	23.38	61.89	99.90	135.27	108.82	2239.26
54.35	0.01	0.13	0.18	1.05	2.92	8.09	24.37	62.35	100.02	133.55	100.24	2239.26
54.45	0.03	0.10	0.24	1.01	2.62	8.93	23.30	58.12	102.59	134.80	104.06	2239.26
54.55	0.04	0.11	0.28	0.98	2.63	8.82	24.37	56.91	101.47	133.79	113.58	2239.26
54.65	0.01	0.08	0.18	0.88	2.65	9.18	23.86	54.50	100.78	141.32	101.57	2239.26
54.75	0.03	0.09	0.29	0.96	2.78	8.23	24.24	59.48	102.77	133.02	102.29	2239.26
54.85	0.00	0.14	0.26	0.96	3.19	8.84	23.20	51.78	99.99	136.05	96.75	2239.26
54.95	0.03	0.16	0.14	0.95	2.72	8.34	23.73	52.53	103.29	134.03	109.21	2239.26
55.05	0.01	0.13	0.22	1.11	3.09	8.68	23.12	57.82	104.16	141.44	103.84	2239.26
55.15	0.03	0.10	0.35	0.96	2.67	7.86	22.05	58.27	102.17	143.87	101.46	2239.26
55.25	0.03	0.07	0.19	0.99	3.09	8.32	23.91	55.10	98.33	128.81	103.89	2239.26
55.35	0.03	0.05	0.28	1.19	3.00	9.62	22.92	59.33	101.93	134.09	107.10	2239.26
55.45	0.02	0.09	0.25	1.06	2.50	8.59	22.76	57.21	97.13	140.20	98.58	2239.26
55.55	0.02	0.09	0.24	0.87	2.72	9.34	23.32	59.48	102.50	135.81	102.40	2239.26
55.65	0.03	0.10	0.32	0.90	2.60	8.28	24.75	54.95	103.07	131.54	98.14	2239.26
55.75	0.03	0.15	0.20	0.90	2.62	8.91	21.84	61.59	103.16	136.99	103.73	2239.26
55.85	0.03	0.13	0.25	1.00	2.77	8.41	22.89	59.18	101.38	140.02	100.68	2239.26
55.95	0.03	0.12	0.23	0.99	2.95	9.07	23.48	60.99	101.99	138.54	99.13	2239.26
56.05	0.03	0.10	0.26	1.03	2.68	9.28	22.10	54.95	102.05	137.59	102.23	2239.26
56.15	0.03	0.11	0.29	0.97	2.65	8.61	22.86	56.31	100.02	140.20	102.68	2239.26
56.25	0.01	0.09	0.25	1.10	2.62	8.64	23.07	58.87	101.93	139.31	105.22	2239.26
56.35	0.02	0.06	0.27	1.19	2.83	8.71	24.17	56.46	100.99	136.76	107.33	2239.26
56.45	0.03	0.04	0.28	0.96	2.60	8.68	22.94	57.06	98.82	136.88	105.06	2239.26
56.55	0.03	0.12	0.27	0.99	2.78	9.00	23.09	53.59	103.16	138.54	111.59	2239.26
56.65	0.02	0.10	0.31	1.01	2.31	8.48	23.61	57.21	103.38	139.43	106.55	2239.26
56.75	0.00	0.08	0.25	1.03	2.77	8.71	23.89	55.70	101.90	142.63	105.11	2239.26
56.85	0.03	0.09	0.25	1.15	2.83	9.12	22.17	61.14	99.66	137.17	107.44	2239.26
56.95	0.01	0.14	0.22	0.92	2.53	8.86	23.45	53.14	102.08	139.07	104.72	2239.26
57.05	0.04	0.10	0.28	1.09	2.68	9.07	22.61	55.70	99.48	134.98	103.95	2239.26
57.15	0.03	0.07	0.23	0.97	2.95	8.32	24.35	62.65	101.20	134.50	104.61	2239.26
57.25	0.04	0.10	0.35	0.79	2.88	8.16	23.86	55.70	105.43	140.08	106.66	2239.26
57.35	0.01	0.10	0.24	0.87	2.50	8.93	22.48	58.42	99.12	136.16	103.73	2239.26
57.45	0.03	0.11	0.20	1.11	3.02	8.73	23.76	59.48	100.06	132.01	103.95	2239.26
57.55	0.03	0.10	0.32	0.97	2.77	8.84	24.45	59.93	103.26	136.34	104.01	2239.26
57.65	0.03	0.05	0.22	1.16	2.11	8.75	22.74	57.06	100.39	135.16	100.19	2239.26
57.75	0.03	0.10	0.26	1.03	2.80	9.09	24.04	56.31	98.30	140.97	108.54	2239.26
57.85	0.00	0.07	0.29	0.86	2.47	8.68	22.71	56.46	99.57	134.15	104.78	2239.26
57.95	0.02	0.14	0.23	0.93	2.99	8.32	23.96	56.91	100.42	139.96	106.33	2239.26
58.05	0.03	0.11	0.25	0.92	2.42	8.71	22.74	56.61	103.71	142.87	114.19	2239.26
58.15	0.01	0.08	0.33	0.97	2.82	8.02	22.97	59.78	102.26	139.01	106.72	2239.26

Midpt.	11000	2700	910	240	84	26	8.8	2.7	0.92	0.31	0.05	Volume
58.25	0.01	0.13	0.18	0.86	3.09	8.36	23.25	56.76	99.69	131.83	107.82	2239.26
58.35	0.02	0.11	0.36	1.18	2.82	8.55	22.33	56.91	102.83	134.92	107.22	2239.26
58.45	0.00	0.11	0.28	1.02	2.95	8.64	23.35	60.23	100.42	132.37	110.32	2239.26
58.55	0.03	0.08	0.34	1.03	2.88	8.02	24.19	55.25	102.86	138.60	110.98	2239.26
58.65	0.02	0.08	0.25	0.93	2.82	8.23	23.32	55.55	101.81	139.43	107.99	2239.26
58.75	0.02	0.11	0.26	1.24	3.05	8.48	23.43	55.70	100.60	135.51	1773.29	2239.26
58.85	0.03	0.12	0.30	1.11	2.70	8.18	24.01	62.19	103.77	136.76	110.26	2239.26
58.95	0.03	0.07	0.28	0.99	2.83	8.53	23.84	56.91	102.32	134.15	111.37	2239.26
59.05	0.03	0.11	0.23	0.99	2.77	8.70	24.73	54.34	104.10	136.70	110.59	2239.26
59.15	0.03	0.12	0.17	1.01	2.68	9.05	22.99	54.50	100.42	133.55	106.94	2239.26
59.25	0.01	0.07	0.32	0.89	2.68	8.25	23.99	52.38	101.78	132.61	106.77	2239.26
59.35	0.02	0.08	0.30	0.95	2.90	9.09	23.20	50.57	99.06	135.33	107.27	2239.26
59.45	0.01	0.12	0.27	0.84	3.04	8.53	22.99	56.31	102.83	140.02	112.92	2239.26
59.55	0.03	0.07	0.28	0.97	2.52	8.41	22.92	56.46	101.50	139.37	110.54	2239.26
59.65	0.04	0.08	0.30	0.96	2.88	8.52	21.48	54.19	100.39	139.37	109.37	2239.26
59.75	0.03	0.07	0.28	0.98	3.00	8.55	22.17	57.21	100.99	141.86	108.82	2239.26
59.85	0.05	0.13	0.26	1.05	2.83	8.73	21.56	52.38	105.61	138.12	108.88	2239.26
59.95	0.03	0.14	0.22	0.99	3.05	8.73	23.43	59.48	101.26	139.01	108.16	2239.26
60.05	0.02	0.07	0.22	1.11	2.85	8.66	22.89	62.35	100.99	134.86	107.16	2239.26
60.15	0.02	0.09	0.36	0.96	2.62	9.18	22.71	57.97	101.69	132.07	109.10	2239.26
60.25	0.03	0.13	0.28	0.93	2.77	9.46	23.68	55.55	99.93	137.59	108.71	2239.26
60.35	0.03	0.10	0.29	1.09	2.68	8.16	23.89	60.08	103.29	133.08	100.85	2239.26
60.45	0.01	0.07	0.24	0.93	2.58	9.05	23.15	57.36	105.67	139.60	108.93	2239.26
60.55	0.02	0.06	0.27	0.97	2.52	8.91	22.94	54.19	100.72	134.68	110.81	2239.26
60.65	0.03	0.12	0.29	1.00	2.40	8.20	23.43	62.19	102.20	134.56	107.60	2239.26
60.75	0.01	0.12	0.27	0.83	2.72	8.48	23.17	60.08	101.53	141.92	109.32	2239.26
60.85	0.01	0.14	0.27	0.93	2.63	8.27	23.53	60.69	101.41	140.85	103.45	2239.26
60.95	0.02	0.09	0.23	1.15	2.47	7.50	23.78	59.18	104.86	135.99	107.99	2239.26
61.05	0.01	0.08	0.32	0.90	2.70	8.57	22.79	57.21	101.47	140.97	105.50	2239.26
61.15	0.02	0.05	0.22	0.99	2.77	9.07	22.61	57.06	101.56	141.50	1770.80	2239.26
61.25	0.03	0.12	0.30	1.04	2.35	8.09	23.12	57.52	102.41	133.67	115.74	2239.26
61.35	0.00	0.11	0.28	0.88	2.94	9.03	22.74	55.10	98.76	137.71	103.40	2239.26
61.45	0.03	0.09	0.22	0.96	3.09	8.57	23.45	58.87	101.75	131.89	106.00	2239.26
61.55	0.03	0.15	0.30	1.06	2.97	9.25	23.02	64.46	102.98	138.66	103.01	2239.26
61.65	0.01	0.08	0.29	0.97	2.65	8.98	24.58	50.12	97.34	133.97	111.42	2239.26
61.75	0.03	0.08	0.26	0.99	2.30	9.02	23.20	54.50	104.22	136.10	108.32	2239.26
61.85	0.03	0.07	0.22	1.11	2.62	8.53	23.22	56.91	101.02	135.93	109.43	2239.26
61.95	0.03	0.13	0.24	0.95	2.88	9.37	23.04	55.85	99.66	137.65	107.16	2239.26
62.05	0.03	0.09	0.24	0.93	2.60	8.78	23.61	61.14	102.20	136.28	109.71	2239.26
62.15	0.02	0.03	0.27	0.79	2.72	9.27	23.50	61.89	100.30	141.74	115.19	2239.26
62.25	0.03	0.07	0.25	0.98	2.57	8.87	24.17	54.80	99.42	137.47	108.27	2239.26
62.35	0.03	0.11	0.32	0.98	2.73	8.82	22.33	59.93	101.63	136.76	108.27	2239.26
62.45	0.03	0.14	0.32	0.90	2.87	8.55	23.02	61.59	102.11	133.32	110.20	2239.26
62.55	0.04	0.12	0.24	1.00	2.60	9.34	23.45	59.18	102.47	136.46	1768.14	2239.26
62.65	0.03	0.07	0.29	0.98	2.77	8.55	22.35	59.93	101.05	139.90	108.21	2239.26

Midpt.	11000	2700	910	240	84	26	8.8	2.7	0.92	0.31	0.05	Volume
62.75	0.03	0.09	0.25	1.05	2.52	8.78	22.74	59.18	99.84	138.95	116.79	2239.26
62.85	0.03	0.09	0.25	0.99	2.58	8.61	22.71	53.74	100.18	143.16	113.53	2239.26
62.95	0.00	0.11	0.25	1.04	2.53	8.59	23.66	59.48	99.69	135.57	109.98	2239.26
63.05	0.03	0.07	0.22	1.03	2.78	8.68	23.66	60.84	100.81	137.59	107.27	2239.26
63.15	0.03	0.04	0.29	1.05	2.83	8.62	23.25	55.55	100.18	136.28	109.65	2239.26
63.25	0.03	0.11	0.22	0.93	2.90	8.39	23.40	52.38	97.85	134.86	112.70	2239.26
63.35	0.02	0.10	0.31	1.03	2.80	9.00	24.14	55.85	95.74	137.65	108.88	2239.26
63.45	0.02	0.07	0.22	1.10	2.72	8.11	22.17	60.53	99.90	133.97	114.19	2239.26
63.55	0.05	0.07	0.30	1.02	2.37	8.07	23.76	59.33	100.78	142.69	112.64	2239.26
63.65	0.03	0.09	0.21	0.93	2.48	8.91	22.48	59.33	106.37	136.99	105.39	2239.26
63.75	0.01	0.05	0.25	0.87	2.92	9.03	24.12	57.67	99.93	138.24	109.87	2239.26
63.85	0.03	0.11	0.19	0.90	2.90	9.32	23.71	53.44	101.78	138.30	110.81	2239.26
63.95	0.02	0.11	0.33	1.08	2.90	9.11	21.87	51.48	104.07	137.88	113.19	2239.26
64.05	0.05	0.05	0.31	1.12	2.53	9.09	23.53	66.72	101.26	135.81	107.10	2239.26
64.15	0.01	0.09	0.19	1.00	2.67	8.30	22.46	55.40	102.86	133.14	113.30	2239.26
64.25	0.03	0.07	0.28	0.92	2.92	8.52	22.94	57.82	101.87	134.80	105.89	2239.26
64.35	0.05	0.09	0.29	0.95	2.45	8.87	21.97	64.76	101.75	131.06	110.20	2239.26
64.45	0.03	0.12	0.30	0.72	2.65	9.02	22.61	56.31	99.78	133.38	115.35	2239.26
64.55	0.01	0.12	0.32	0.93	2.70	8.71	23.63	57.82	100.69	133.67	111.53	2239.26
64.65	0.01	0.15	0.35	1.04	2.68	9.00	23.63	56.91	100.84	137.71	106.05	2239.26
64.75	0.03	0.04	0.27	1.09	2.75	8.68	22.17	54.80	101.93	145.12	111.03	2239.26
64.85	0.03	0.06	0.22	0.95	2.94	7.98	23.86	59.93	99.00	139.90	105.78	2239.26
64.95	0.00	0.11	0.27	0.98	2.83	8.05	23.35	56.91	101.93	135.45	112.53	2239.26
65.05	0.05	0.11	0.23	1.07	2.60	8.34	21.79	57.82	101.47	134.74	110.20	2239.26
65.15	0.04	0.10	0.28	1.03	3.04	8.57	23.12	60.99	102.02	137.53	113.69	2239.26
65.25	0.03	0.11	0.27	1.04	2.95	8.61	23.99	56.46	105.37	136.46	110.37	2239.26
65.35	0.03	0.09	0.19	0.92	2.73	8.78	24.32	64.46	101.75	141.86	110.26	2239.26
65.45	0.03	0.13	0.30	0.99	2.53	8.25	23.30	56.76	101.90	137.17	115.63	2239.26
65.55	0.01	0.08	0.24	1.12	2.45	8.64	24.29	55.55	99.48	133.55	114.41	2239.26
65.65	0.02	0.11	0.23	0.96	3.05	8.96	23.73	58.57	98.33	132.37	110.37	2239.26
65.75	0.04	0.07	0.21	1.00	2.75	8.32	23.55	54.50	102.65	138.36	117.07	2239.26
65.85	0.01	0.07	0.23	1.03	3.05	9.02	21.87	58.72	99.48	134.80	120.67	2239.26
65.95	0.00	0.11	0.31	1.01	3.10	8.16	23.66	62.35	101.11	141.09	112.86	2239.26
66.05	0.03	0.10	0.23	1.03	2.95	8.91	22.94	53.89	98.18	132.84	103.62	2239.26
66.15	0.03	0.12	0.30	0.96	2.65	8.50	23.81	59.78	99.63	130.35	110.15	2239.26
66.25	0.01	0.09	0.25	0.98	2.50	8.50	22.97	55.85	99.12	141.09	110.54	2239.26
66.35	0.03	0.07	0.29	1.05	2.58	7.93	23.48	53.59	100.51	129.94	108.05	2239.26
66.45	0.06	0.11	0.26	0.90	2.78	8.87	23.68	62.19	101.84	137.11	108.27	2239.26
66.55	0.02	0.05	0.25	1.01	2.73	8.61	23.40	64.01	98.97	133.85	112.64	2239.26
66.65	0.02	0.10	0.30	0.81	2.43	8.98	23.07	57.06	101.20	136.46	111.20	2239.26
66.75	0.02	0.07	0.42	0.86	3.14	8.39	23.50	56.16	101.87	132.31	111.37	2239.26
66.85	0.02	0.07	0.22	1.16	2.68	8.61	23.27	54.80	104.01	141.68	110.87	2239.26
66.95	0.03	0.07	0.26	0.89	3.07	8.71	23.27	53.89	102.20	134.98	108.32	2239.26
67.05	0.04	0.09	0.33	0.98	2.52	8.66	23.99	57.21	101.63	126.73	109.60	2239.26
67.15	0.01	0.08	0.27	1.16	2.80	9.07	23.50	60.69	101.32	137.82	113.91	2239.26

Midpt.	11000	2700	910	240	84	26	8.8	2.7	0.92	0.31	0.05	Volume
67.25	0.03	0.09	0.24	0.86	2.48	9.05	21.46	61.44	102.83	135.87	103.78	2239.26
67.35	0.06	0.07	0.30	1.07	2.94	8.86	23.71	52.23	101.69	142.09	112.47	2239.26
67.45	0.03	0.08	0.28	0.95	2.62	8.87	23.89	59.18	103.50	135.81	109.98	2239.26
67.55	0.00	0.06	0.23	0.95	2.68	8.53	23.43	60.23	104.64	133.61	108.49	2239.26
67.65	0.02	0.06	0.24	1.08	2.48	8.21	22.43	54.34	103.56	136.10	110.09	2239.26
67.75	0.04	0.11	0.31	1.03	2.85	8.55	23.43	56.91	98.15	138.06	111.75	2239.26
67.85	0.03	0.09	0.20	0.99	2.95	8.02	23.58	56.31	104.07	136.88	117.18	2239.26
67.95	0.02	0.11	0.27	0.91	2.68	8.84	24.01	60.38	98.94	138.48	106.88	2239.26
68.05	0.03	0.07	0.21	1.05	2.57	8.48	22.79	59.63	102.05	139.54	111.31	2239.26
68.15	0.03	0.11	0.28	0.99	2.90	7.41	23.20	55.10	99.09	133.55	112.31	2239.26
68.25	0.01	0.13	0.27	0.92	2.73	8.50	22.38	56.61	100.18	142.33	110.32	2239.26
68.35	0.03	0.11	0.23	0.95	2.60	8.14	25.44	56.76	99.72	139.31	109.98	2239.26
68.45	0.07	0.11	0.31	0.82	2.67	8.46	23.78	57.67	102.26	135.10	107.33	2239.26
68.55	0.02	0.14	0.35	1.04	2.68	7.98	22.51	55.55	100.39	136.94	110.59	2239.26
68.65	0.01	0.09	0.28	0.90	2.83	8.75	24.63	55.40	99.30	137.23	110.04	2239.26
68.75	0.03	0.07	0.27	0.90	2.80	8.34	23.48	52.08	101.20	133.97	117.95	2239.26
68.85	0.03	0.09	0.27	1.07	2.63	9.00	22.79	57.82	102.05	137.88	112.03	2239.26
68.95	0.04	0.10	0.28	1.03	2.78	9.09	23.04	54.34	101.26	137.35	112.75	2239.26
69.05	0.03	0.13	0.27	1.11	2.70	8.98	23.55	55.40	101.26	132.55	110.26	2239.26
69.15	0.02	0.08	0.28	1.01	2.92	8.62	22.66	54.50	100.27	132.67	108.27	2239.26
69.25	0.03	0.12	0.33	0.90	2.67	8.55	23.15	51.48	103.86	142.04	106.94	2239.26
69.35	0.03	0.11	0.32	0.99	2.73	8.57	22.53	56.16	101.81	135.63	106.99	2239.26
69.45	0.03	0.04	0.22	1.04	2.31	8.53	22.28	52.08	103.74	143.04	110.70	2239.26
69.55	0.03	0.07	0.20	1.10	2.57	8.18	23.22	59.48	100.51	136.58	103.78	2239.26
69.65	0.04	0.07	0.22	0.84	2.48	8.07	23.91	59.63	102.08	136.82	113.80	2239.26
69.75	0.03	0.04	0.23	0.85	2.80	8.61	22.51	60.84	100.96	135.99	111.20	2239.26
69.85	0.04	0.06	0.22	1.04	2.63	8.59	23.84	61.59	97.88	134.86	106.39	2239.26
69.95	0.02	0.11	0.22	0.85	2.83	8.45	22.89	59.18	103.32	133.91	113.14	2239.26
70.05	0.01	0.12	0.30	0.90	2.52	9.16	23.20	58.72	104.10	139.60	110.32	2239.26
70.15	0.03	0.11	0.22	1.00	2.85	8.78	22.97	59.78	100.27	139.01	113.80	2239.26
70.25	0.00	0.11	0.22	1.01	2.67	8.64	23.50	58.87	100.18	129.46	107.82	2239.26
70.35	0.04	0.07	0.32	0.99	2.28	8.50	22.97	63.40	101.11	131.00	107.44	2239.26
70.45	0.03	0.10	0.21	0.85	3.22	9.16	22.71	58.42	101.53	135.04	105.00	2239.26
70.55	0.02	0.11	0.27	0.94	2.77	8.34	22.97	58.72	98.30	132.72	109.87	2239.26
70.65	0.01	0.07	0.23	0.93	2.55	9.52	22.33	59.18	99.39	137.47	1776.56	2239.26
70.75	0.02	0.12	0.30	1.02	2.95	8.55	23.55	55.85	101.08	138.24	107.49	2239.26
70.85	0.02	0.08	0.30	1.09	2.67	8.68	22.33	50.12	101.20	137.71	105.89	2239.26
70.95	0.05	0.08	0.30	1.01	3.05	8.41	22.58	54.50	102.05	130.35	107.55	2239.26
71.05	0.03	0.11	0.28	0.95	2.47	8.82	24.45	59.18	100.15	130.35	107.71	2239.26
71.15	0.02	0.13	0.25	0.95	2.72	8.21	22.51	58.57	103.07	133.79	108.88	2239.26
71.25	0.03	0.16	0.23	1.02	2.42	8.77	23.91	58.42	105.01	131.54	107.22	2239.26
71.35	0.03	0.12	0.22	0.85	2.92	9.37	22.23	61.44	103.47	133.55	112.20	2239.26
71.45	0.03	0.12	0.26	0.98	2.90	9.07	22.58	56.61	101.02	134.03	108.60	2239.26
71.55	0.05	0.04	0.39	0.84	2.92	8.20	22.25	56.16	100.81	130.83	108.43	2239.26
71.65	0.04	0.11	0.30	1.00	2.62	9.09	23.38	58.87	100.54	129.23	107.99	2239.26

Midpt.	11000	2700	910	240	84	26	8.8	2.7	0.92	0.31	0.05	Volume
71.75	0.03	0.10	0.23	0.99	2.58	8.78	23.09	56.91	101.20	139.60	1769.80	2239.26
71.85	0.03	0.09	0.28	1.11	2.78	8.57	22.66	61.74	104.13	137.23	114.02	2239.26
71.95	0.03	0.06	0.34	0.94	2.92	8.55	23.07	53.74	98.94	137.53	113.08	2239.26
72.05	0.02	0.09	0.27	1.00	2.60	7.68	24.09	57.21	100.21	137.23	108.43	2239.26
72.15	0.00	0.07	0.25	0.83	2.70	8.96	23.91	59.18	103.56	135.51	105.55	2239.26
72.25	0.08	0.14	0.28	0.96	2.53	9.16	23.81	60.23	101.59	139.84	1767.20	2239.26
72.35	0.05	0.08	0.28	0.86	2.47	8.53	23.22	55.85	100.78	137.23	105.83	2239.26
72.45	0.01	0.08	0.24	1.29	2.52	8.57	22.61	56.76	101.69	131.83	112.86	2239.26
72.55	0.03	0.12	0.29	0.92	2.82	8.45	23.58	60.23	100.51	138.77	106.88	2239.26
72.65	0.02	0.11	0.18	1.05	2.40	8.37	22.97	54.65	99.63	135.99	110.81	2239.26
72.75	0.03	0.07	0.29	1.06	2.48	7.89	22.86	58.87	99.09	135.27	103.73	2239.26
72.85	0.01	0.11	0.27	1.01	2.50	8.70	22.81	55.10	105.64	140.97	103.95	2239.26
72.95	0.01	0.10	0.22	0.83	2.47	8.68	24.09	55.55	101.02	142.15	109.87	2239.26
73.05	0.04	0.02	0.27	0.93	2.65	8.59	21.84	54.04	100.90	135.45	103.45	2239.26
73.15	0.04	0.09	0.25	0.97	2.48	8.61	23.89	57.06	99.60	137.47	109.98	2239.26
73.25	0.03	0.06	0.28	0.99	2.62	9.39	23.22	57.36	101.87	132.61	109.76	2239.26
73.35	0.05	0.07	0.33	0.87	2.85	8.75	22.81	57.82	101.87	135.69	107.55	2239.26
73.45	0.03	0.10	0.28	1.09	2.72	8.46	22.53	55.10	103.19	130.83	108.60	2239.26
73.55	0.02	0.08	0.28	0.98	2.53	8.48	21.23	57.67	101.59	135.81	108.88	2239.26
73.65	0.02	0.11	0.28	0.90	2.73	8.61	23.89	59.48	99.78	132.43	105.11	2239.26
73.75	0.02	0.12	0.21	0.95	2.63	8.59	22.12	51.63	97.22	132.01	103.06	2239.26
73.85	0.05	0.05	0.23	0.96	2.92	8.59	23.20	54.34	100.90	145.30	104.28	2239.26
73.95	0.03	0.07	0.32	0.86	2.77	9.18	22.38	55.55	101.26	131.89	105.78	2239.26
74.05	0.02	0.09	0.27	1.04	2.45	7.89	24.96	56.76	98.91	134.92	103.56	2239.26
74.15	0.03	0.11	0.23	1.01	2.55	7.61	23.48	59.33	98.88	134.80	107.05	2239.26
74.25	0.03	0.11	0.22	1.12	2.70	8.59	22.79	57.21	106.24	135.27	108.21	2239.26
74.35	0.01	0.12	0.25	1.12	2.80	8.39	24.14	57.21	99.06	135.33	106.77	2239.26
74.45	0.01	0.10	0.23	0.90	2.45	8.87	22.56	59.78	101.72	137.65	100.08	2239.26
74.55	0.04	0.12	0.28	0.91	2.47	7.87	22.35	59.48	101.66	134.92	107.16	2239.26
74.65	0.02	0.10	0.26	1.15	2.60	8.59	23.20	55.10	100.93	141.32	105.39	2239.26
74.75	0.03	0.11	0.25	1.10	2.35	8.59	22.66	56.91	104.43	135.69	102.62	2239.26
74.85	0.01	0.09	0.24	1.12	2.62	8.80	22.48	57.67	100.99	136.28	109.10	2239.26
74.95	0.01	0.08	0.31	0.75	2.75	8.03	22.40	59.93	99.75	138.95	106.11	2239.26
75.05	0.02	0.13	0.28	0.90	2.60	8.32	23.30	56.46	95.38	134.03	98.30	2239.26
75.15	0.02	0.07	0.37	1.02	2.94	8.48	23.35	59.18	99.03	133.14	103.95	2239.26
75.25	0.03	0.09	0.28	1.15	2.85	9.18	24.45	52.08	99.63	137.41	105.72	2239.26
75.35	0.02	0.10	0.22	0.99	2.33	9.05	21.87	62.50	100.42	140.20	111.86	2239.26
75.45	0.01	0.10	0.24	0.90	3.00	8.95	23.89	60.38	104.10	135.45	98.53	2239.26
75.55	0.03	0.07	0.25	1.06	2.37	8.98	21.59	52.84	100.09	139.07	105.33	2239.26
75.65	0.03	0.06	0.34	0.89	2.77	8.87	23.91	57.97	98.64	140.26	106.16	2239.26
75.75	0.03	0.12	0.28	1.01	2.60	8.78	23.22	64.61	102.44	135.81	106.77	2239.26
75.85	0.03	0.08	0.32	1.01	2.77	8.71	23.58	57.97	100.87	135.39	106.83	2239.26
75.95	0.03	0.11	0.22	1.08	2.85	8.62	23.12	56.16	99.30	136.64	101.68	2239.26
76.05	0.03	0.10	0.25	0.96	2.38	7.89	22.71	59.33	100.78	139.37	103.73	2239.26
76.15	0.03	0.09	0.21	0.96	2.50	8.57	22.23	49.06	101.96	135.57	100.24	2239.26

Midpt.	11000	2700	910	240	84	26	8.8	2.7	0.92	0.31	0.05	Volume
76.25	0.03	0.12	0.28	1.13	2.75	9.02	24.55	58.12	102.32	138.95	102.29	2239.26
76.35	0.03	0.07	0.29	0.98	2.83	8.68	21.87	53.74	101.69	131.95	107.38	2239.26
76.45	0.02	0.11	0.22	1.04	2.77	8.95	22.46	54.50	101.69	137.41	105.06	2239.26
76.55	0.04	0.11	0.34	0.97	2.50	8.71	23.68	54.80	99.78	138.71	105.22	2239.26
76.65	0.05	0.06	0.22	0.90	2.65	8.52	22.43	61.89	100.42	136.76	103.56	2239.26
76.75	0.01	0.13	0.22	0.95	2.87	8.55	22.28	63.25	100.93	131.95	108.16	2239.26
76.85	0.02	0.09	0.20	0.78	2.48	8.09	24.40	49.67	103.04	138.18	104.95	2239.26
76.95	0.02	0.11	0.24	0.76	2.88	8.77	22.61	56.61	99.45	140.32	104.67	2239.26
77.05	0.00	0.04	0.45	1.01	2.82	8.09	22.33	59.78	101.96	142.15	102.29	2239.26
77.15	0.04	0.08	0.32	0.95	2.68	8.66	23.30	57.52	101.38	135.27	104.67	2239.26
77.25	0.03	0.11	0.21	0.90	2.25	8.68	22.51	61.74	102.44	135.93	101.96	2239.26
77.35	0.01	0.06	0.27	1.13	2.97	8.00	23.07	62.80	101.84	135.39	102.12	2239.26
77.45	0.01	0.06	0.24	1.04	3.09	8.41	24.83	56.91	101.81	139.54	101.24	2239.26
77.55	0.00	0.09	0.29	0.90	2.62	9.02	23.43	57.06	99.84	142.39	102.68	2239.26
77.65	0.04	0.08	0.25	1.03	2.73	8.16	23.68	52.53	100.54	136.16	100.63	2239.26
77.75	0.04	0.11	0.28	0.89	2.85	8.53	23.66	55.25	100.78	138.30	100.08	2239.26
77.85	0.00	0.09	0.26	1.01	2.95	8.43	21.92	56.76	106.12	137.88	100.85	2239.26
77.95	0.03	0.16	0.26	1.10	3.05	9.00	22.63	56.46	101.44	138.48	107.22	2239.26
78.05	0.02	0.11	0.29	1.09	2.52	8.70	23.68	61.14	102.08	139.13	104.17	2239.26
78.15	0.04	0.07	0.31	1.08	2.25	8.59	23.35	52.99	103.53	142.98	105.00	2239.26
78.25	0.03	0.10	0.30	1.13	3.29	8.41	22.00	61.59	99.54	136.10	105.61	2239.26
78.35	0.02	0.12	0.28	1.13	2.78	8.28	22.92	54.50	102.14	140.43	96.42	2239.26
78.45	0.02	0.06	0.22	1.00	3.04	8.16	23.02	57.21	98.24	135.99	101.63	2239.26
78.55	0.04	0.09	0.30	0.82	2.63	8.30	22.33	53.44	104.64	139.43	99.24	2239.26
78.65	0.02	0.11	0.26	0.96	2.35	8.57	23.35	54.19	100.54	138.95	101.46	2239.26
78.75	0.00	0.13	0.32	1.11	2.73	8.16	22.81	60.53	104.83	134.15	100.08	2239.26
78.85	0.01	0.11	0.30	0.93	2.47	8.05	21.94	59.33	105.52	138.12	100.91	2239.26
78.95	0.00	0.10	0.19	1.14	2.78	8.86	23.04	58.42	98.64	133.08	101.29	2239.26
79.05	0.04	0.04	0.25	0.93	2.37	9.07	21.48	59.93	98.88	139.13	102.01	2239.26
79.15	0.01	0.12	0.22	0.97	2.85	8.93	24.01	54.34	100.33	131.12	103.73	2239.26
79.25	0.02	0.11	0.24	0.94	2.78	8.53	22.92	59.48	99.99	136.34	98.30	2239.26
79.35	0.03	0.11	0.26	0.90	2.87	8.95	23.30	55.10	102.11	132.13	97.97	2239.26
79.45	0.03	0.11	0.32	0.94	2.77	8.68	21.66	59.02	102.56	134.92	102.29	2239.26
79.55	0.03	0.08	0.24	0.97	2.75	8.66	21.38	57.21	103.68	132.61	99.47	2239.26
79.65	0.03	0.07	0.28	1.12	2.70	7.78	24.40	58.12	102.17	133.91	99.02	2239.26
79.75	0.02	0.07	0.28	0.91	2.52	8.34	22.38	58.87	98.36	132.67	95.70	2239.26
79.85	0.02	0.07	0.31	0.92	2.57	8.30	23.45	56.61	101.53	131.48	99.69	2239.26
79.95	0.02	0.11	0.33	0.91	3.10	8.32	23.81	55.55	103.23	136.52	96.92	2239.26
80.05	0.03	0.02	0.23	1.04	2.75	8.02	22.94	54.34	103.41	141.38	97.81	2239.26
80.15	0.02	0.09	0.29	0.92	2.73	8.00	22.63	55.40	100.27	135.81	99.13	2239.26
80.25	0.02	0.05	0.29	0.88	2.82	8.52	23.04	54.04	101.96	129.64	98.86	2239.26
80.35	0.06	0.15	0.23	0.94	2.65	8.80	23.40	59.93	106.27	136.16	99.80	2239.26
80.45	0.03	0.13	0.27	0.94	2.87	8.80	22.40	55.25	102.80	135.51	99.02	2239.26
80.55	0.01	0.10	0.22	0.95	2.90	8.57	22.25	55.25	101.69	138.48	100.91	2239.26
80.65	0.03	0.08	0.20	0.95	3.04	9.18	22.79	58.27	101.23	138.42	101.29	2239.26

Midpt.	11000	2700	910	240	84	26	8.8	2.7	0.92	0.31	0.05	Volume
80.75	0.03	0.11	0.25	1.04	2.78	8.21	23.50	58.12	102.32	138.66	100.41	2239.26
80.85	0.02	0.09	0.31	0.85	2.88	8.62	22.10	54.80	99.48	136.46	95.81	2239.26
80.95	0.06	0.07	0.22	1.07	2.72	8.53	22.48	52.99	102.14	140.49	100.79	2239.26
81.05	0.00	0.11	0.28	1.04	2.75	8.82	23.43	57.21	103.35	136.82	98.91	2239.26
81.15	0.01	0.16	0.18	0.83	2.60	8.84	22.84	62.95	101.20	136.40	100.02	2239.26
81.25	0.04	0.06	0.26	0.92	2.57	8.71	24.37	54.80	102.47	136.58	97.53	2239.26
81.35	0.03	0.14	0.35	1.08	2.42	8.23	21.56	57.82	103.32	130.77	99.30	2239.26
81.45	0.03	0.08	0.33	0.95	2.77	8.28	23.50	57.21	101.96	133.14	3420.66	2239.26
81.55	0.03	0.05	0.20	0.95	2.35	8.23	23.15	61.74	98.76	133.97	95.65	2239.26
81.65	0.02	0.19	0.23	1.15	2.48	8.57	23.68	61.74	99.60	145.42	93.27	2239.26
81.75	0.02	0.06	0.27	1.11	2.70	8.70	22.61	53.59	101.08	143.28	97.03	2239.26
81.85	0.02	0.10	0.20	0.94	2.67	8.36	22.99	59.18	101.56	132.19	94.43	2239.26
81.95	0.04	0.11	0.24	0.89	2.40	8.36	23.22	56.16	100.96	133.55	1757.40	2239.26
82.05	0.04	0.08	0.32	0.95	2.58	8.28	23.58	58.87	100.18	137.41	93.65	2239.26
82.15	0.03	0.14	0.20	1.05	2.58	8.68	23.07	58.57	103.59	134.50	94.60	2239.26
82.25	0.03	0.11	0.29	0.99	2.85	9.37	23.20	59.48	102.32	135.69	97.03	2239.26
82.35	0.03	0.10	0.27	1.01	3.20	8.12	22.07	59.33	103.19	139.43	96.37	2239.26
82.45	0.01	0.07	0.27	1.02	2.70	8.27	21.87	57.06	99.36	139.13	101.68	2239.26
82.55	0.03	0.09	0.27	0.89	2.97	8.45	23.61	57.36	101.29	135.69	93.88	2239.26
82.65	0.02	0.07	0.32	0.84	2.57	9.80	23.25	54.34	102.14	135.45	98.41	2239.26
82.75	0.03	0.14	0.25	0.87	3.05	8.53	22.79	52.99	98.76	141.44	97.36	2239.26
82.85	0.00	0.09	0.29	1.02	2.25	8.14	22.63	58.57	97.19	135.75	94.04	2239.26
82.95	0.03	0.10	0.32	0.99	2.45	8.64	24.91	59.18	96.52	141.21	97.53	2239.26
83.05	0.03	0.11	0.31	1.03	2.63	8.16	23.12	60.84	102.98	134.80	95.65	2239.26
83.15	0.01	0.10	0.34	0.78	2.62	8.37	22.53	57.67	101.69	134.62	95.09	2239.26
83.25	0.03	0.06	0.25	1.03	2.77	7.41	22.86	56.31	104.89	133.08	98.08	2239.26
83.35	0.02	0.07	0.32	0.77	2.38	8.70	23.53	57.52	101.26	139.31	97.64	2239.26
83.45	0.02	0.11	0.28	0.85	2.53	8.66	22.43	58.27	98.97	139.19	96.48	2239.26
83.55	0.02	0.11	0.37	0.88	2.94	8.05	23.68	53.14	102.14	134.39	96.92	2239.26
83.65	0.04	0.08	0.27	0.87	2.62	8.82	21.77	58.27	98.00	135.75	96.31	2239.26
83.75	0.02	0.14	0.28	0.92	2.52	8.57	22.97	56.61	104.70	136.40	98.47	2239.26
83.85	0.04	0.13	0.18	1.02	2.37	8.30	22.43	53.74	101.47	132.67	98.69	2239.26
83.95	0.02	0.07	0.21	0.95	2.60	8.20	22.58	59.48	103.53	143.16	96.26	2239.26
84.05	0.02	0.11	0.28	1.04	2.72	8.23	22.92	55.70	101.11	135.22	99.41	2239.26
84.15	0.02	0.07	0.32	0.98	2.30	7.80	22.35	51.93	102.32	137.41	92.66	2239.26
84.25	0.03	0.08	0.29	0.94	2.77	7.70	20.46	61.89	99.87	135.22	96.48	2239.26
84.35	0.02	0.07	0.31	0.95	2.83	8.23	22.33	60.69	105.37	138.60	97.03	2239.26
84.45	0.04	0.10	0.22	0.88	3.15	7.75	22.63	64.61	98.27	137.59	97.42	2239.26
84.55	0.03	0.13	0.21	1.03	2.58	8.98	22.92	59.18	103.01	133.61	100.41	2239.26
84.65	0.03	0.09	0.28	1.00	2.35	8.66	21.69	55.40	98.91	134.03	102.23	2239.26
84.75	0.03	0.12	0.19	0.89	2.58	8.37	22.66	56.91	100.75	138.89	100.74	2239.26
84.85	0.03	0.07	0.31	1.05	2.62	8.57	21.08	51.63	101.75	133.79	1757.68	2239.26
84.95	0.02	0.06	0.29	0.94	2.94	8.39	22.51	54.19	102.35	134.80	99.02	2239.26
85.05	0.00	0.06	0.25	0.81	2.68	8.30	21.43	62.19	100.57	134.80	93.54	2239.26
85.15	0.01	0.06	0.16	0.85	2.47	8.34	22.12	57.06	100.57	133.97	93.54	2239.26

Midpt.	11000	2700	910	240	84	26	8.8	2.7	0.92	0.31	0.05	Volume
85.25	0.02	0.08	0.26	1.05	2.65	7.71	22.92	50.72	100.57	140.61	96.75	2239.26
85.35	0.05	0.06	0.15	0.92	2.73	8.71	20.74	55.25	97.67	134.98	98.47	2239.26
85.45	0.03	0.06	0.31	0.90	2.92	8.36	22.74	55.55	95.92	134.80	93.65	2239.26
85.55	0.03	0.07	0.24	0.85	2.48	8.61	22.76	59.48	99.66	133.79	1752.37	2239.26
85.65	0.03	0.07	0.27	0.87	2.60	7.78	22.43	55.40	97.58	138.77	96.42	2239.26
85.75	0.03	0.11	0.29	0.90	2.45	8.34	21.28	54.95	98.45	136.70	96.53	2239.26
85.85	0.02	0.09	0.25	1.10	2.62	8.32	21.41	57.06	99.78	133.44	96.42	2239.26
85.95	0.03	0.06	0.27	0.86	3.00	7.62	22.35	52.68	101.41	138.48	96.31	2239.26
86.05	0.00	0.11	0.28	1.01	2.80	8.84	21.46	54.50	99.30	142.87	1755.58	2239.26
86.15	0.02	0.10	0.30	1.00	2.67	8.50	23.35	59.33	97.22	135.51	1760.17	2239.26
86.25	0.00	0.13	0.22	0.78	2.63	7.89	22.74	56.46	97.28	129.17	98.41	2239.26
86.35	0.03	0.11	0.22	0.93	2.63	9.37	23.12	61.89	101.11	133.32	94.65	2239.26
86.45	0.03	0.07	0.35	1.09	2.31	8.43	22.00	54.80	95.74	131.78	102.84	2239.26
86.55	0.01	0.09	0.32	1.03	2.63	9.09	24.42	57.21	94.56	135.16	98.41	2239.26
86.65	0.00	0.04	0.32	0.89	2.60	8.73	23.04	56.46	99.39	131.24	95.87	2239.26
86.75	0.01	0.11	0.27	0.96	2.72	8.27	21.71	59.78	99.93	130.00	93.05	2239.26
86.85	0.01	0.07	0.26	0.92	2.58	8.48	22.20	56.46	100.75	135.22	96.09	2239.26
86.95	0.03	0.09	0.28	0.85	2.80	8.70	22.46	54.04	99.72	138.18	96.03	2239.26
87.05	0.03	0.10	0.35	0.75	2.42	8.16	22.53	54.50	98.91	137.71	93.71	2239.26
87.15	0.03	0.11	0.26	0.92	2.23	8.64	23.45	58.72	102.32	136.76	98.30	2239.26
87.25	0.00	0.09	0.25	0.92	2.31	7.80	23.25	57.36	100.21	136.46	99.91	2239.26
87.35	0.03	0.11	0.27	0.95	2.33	8.23	22.43	56.31	104.73	133.85	94.54	2239.26
87.45	0.03	0.15	0.27	0.91	2.63	8.46	22.23	56.46	99.81	136.58	97.20	2239.26
87.55	0.03	0.09	0.31	0.96	2.80	7.98	22.51	52.53	100.54	139.66	97.81	2239.26
87.65	0.03	0.11	0.25	0.89	2.31	7.87	22.76	53.14	100.12	131.54	92.71	2239.26
87.75	0.05	0.08	0.39	1.05	2.28	8.20	20.82	58.72	97.70	133.85	1756.63	2239.26
87.85	0.01	0.08	0.33	0.99	2.52	8.64	21.25	56.61	99.54	134.33	95.70	2239.26
87.95	0.04	0.07	0.31	0.76	2.37	8.02	22.07	60.53	97.61	132.31	97.47	2239.26
88.05	0.01	0.07	0.32	0.96	2.85	7.95	22.07	51.02	97.61	135.63	96.03	2239.26
88.15	0.03	0.04	0.23	0.85	2.78	8.39	22.56	60.23	98.70	129.82	93.43	2239.26
88.25	0.01	0.07	0.24	0.95	2.65	8.36	22.02	56.76	99.69	138.06	100.19	2239.26
88.35	0.02	0.10	0.28	0.92	2.67	7.55	21.89	57.36	97.58	131.18	92.93	2239.26
88.45	0.03	0.07	0.37	1.09	2.65	8.28	22.17	54.95	98.18	137.77	97.03	2239.26
88.55	0.02	0.09	0.25	0.76	2.95	8.23	22.48	56.31	98.76	137.29	92.99	2239.26
88.65	0.02	0.11	0.26	0.84	2.88	7.98	21.64	50.27	101.99	131.12	95.87	2239.26
88.75	0.02	0.07	0.21	0.79	2.67	7.53	21.48	51.48	98.18	142.75	3409.25	2239.26
88.85	0.03	0.08	0.21	0.82	2.47	8.18	22.00	55.10	98.49	134.62	97.53	2239.26
88.95	0.02	0.09	0.33	1.06	2.53	7.95	22.56	50.12	98.06	137.41	96.86	2239.26
89.05	0.00	0.07	0.33	0.92	2.40	7.91	21.23	52.38	98.12	136.10	88.62	2239.26
89.15	0.04	0.11	0.25	0.87	2.62	8.77	22.17	54.80	98.09	131.12	95.54	2239.26
89.25	0.02	0.10	0.22	0.81	2.83	8.16	21.36	53.74	99.84	130.23	101.68	2239.26
89.35	0.00	0.11	0.31	0.99	2.50	7.75	21.08	58.42	96.76	135.51	91.77	2239.26
89.45	0.02	0.09	0.30	0.81	2.45	8.36	22.89	54.19	99.96	129.82	93.60	2239.26
89.55	0.03	0.07	0.35	0.76	2.55	7.55	22.17	53.74	99.36	125.85	95.31	2239.26
89.65	0.03	0.08	0.24	0.92	2.45	7.89	22.33	56.16	97.61	132.01	89.28	2239.26

Midpt.	11000	2700	910	240	84	26	8.8	2.7	0.92	0.31	0.05	Volume
89.75	0.02	0.06	0.28	0.81	2.47	8.14	20.28	51.02	96.67	128.93	96.20	2239.26
89.85	0.00	0.07	0.32	1.16	2.26	7.93	21.59	52.68	95.77	134.62	92.77	2239.26
89.95	0.02	0.11	0.20	0.95	2.77	7.82	21.36	54.65	96.58	130.89	89.50	2239.26
90.05	0.01	0.07	0.27	0.68	2.52	8.84	20.92	55.85	93.38	135.87	94.76	2239.26
90.15	0.03	0.07	0.22	0.85	2.42	8.45	20.72	60.53	96.19	130.29	92.99	2239.26
90.25	0.03	0.07	0.28	0.98	2.70	7.98	21.71	50.12	92.84	134.98	1009.61	2239.26
90.35	0.01	0.10	0.23	0.97	2.35	8.11	19.98	52.23	95.13	130.12	91.05	2239.26
90.45	0.03	0.08	0.29	0.81	2.47	8.00	21.69	54.19	94.47	136.28	94.98	2239.26
90.55	0.02	0.06	0.18	0.91	2.80	7.43	21.13	52.53	95.77	123.71	87.84	2239.26
90.65	0.03	0.07	0.25	0.87	2.43	7.91	22.12	55.70	98.91	131.18	90.06	2239.26
90.75	0.00	0.09	0.15	1.04	2.62	7.70	20.46	57.06	94.98	131.95	90.78	2239.26
90.85	0.03	0.07	0.20	1.01	2.92	7.86	21.64	48.76	95.77	133.73	1752.59	2239.26
90.95	0.01	0.05	0.24	0.73	2.35	8.25	20.28	58.12	93.20	129.76	89.34	2239.26
91.05	0.02	0.12	0.22	0.87	2.65	7.34	21.33	53.59	94.38	129.94	90.72	2239.26
91.15	0.03	0.07	0.25	0.92	2.55	7.41	21.84	55.85	95.59	125.61	91.83	2239.26
91.25	0.01	0.10	0.23	0.99	2.45	7.95	20.26	50.27	93.14	127.68	91.33	2239.26
91.35	0.03	0.11	0.32	0.85	2.38	8.25	21.38	53.29	92.48	129.34	90.39	2239.26
91.45	0.03	0.05	0.21	0.83	2.53	8.07	21.48	55.55	94.56	127.86	94.32	2239.26
91.55	0.01	0.04	0.26	0.82	2.58	7.64	20.28	52.99	93.35	131.00	89.61	2239.26
91.65	0.00	0.12	0.25	0.87	2.18	7.70	20.44	56.91	91.90	126.32	92.27	2239.26
91.75	0.00	0.12	0.23	0.90	2.55	7.93	21.18	48.76	93.26	127.92	85.74	2239.26
91.85	0.03	0.07	0.22	0.83	2.08	7.87	21.41	50.57	94.68	128.63	90.89	2239.26
91.95	0.04	0.07	0.22	1.02	2.30	8.12	18.75	51.33	93.08	125.25	88.56	2239.26
92.05	0.01	0.07	0.25	0.81	2.18	7.20	21.69	53.89	92.75	126.56	90.00	2239.26
92.15	0.02	0.07	0.12	0.87	2.65	7.41	19.75	49.82	93.78	125.19	90.61	2239.26
92.25	0.04	0.09	0.31	0.91	2.26	8.07	20.36	45.74	90.64	125.96	84.85	2239.26
92.35	0.02	0.08	0.15	0.84	2.30	8.14	20.28	52.08	90.51	124.84	89.45	2239.26
92.45	0.03	0.11	0.23	0.80	2.25	7.02	20.64	48.61	89.82	124.42	87.18	2239.26
92.55	0.01	0.10	0.32	0.87	2.55	7.75	20.21	51.33	93.65	127.27	81.09	2239.26
92.65	0.01	0.09	0.18	0.73	2.25	7.43	20.41	49.82	90.91	125.13	82.42	2239.26
92.75	0.01	0.07	0.20	0.75	2.82	7.86	20.97	52.68	90.88	126.56	87.01	2239.26
92.85	0.03	0.12	0.18	0.80	2.52	7.86	21.38	50.72	93.68	122.29	92.77	2239.26
92.95	0.03	0.12	0.24	0.81	2.68	7.84	21.46	46.34	91.93	126.32	85.74	2239.26
93.05	0.03	0.06	0.27	0.75	2.58	7.50	19.80	52.08	92.99	128.34	86.74	2239.26
93.15	0.01	0.07	0.30	0.90	2.63	7.53	19.82	51.48	87.71	121.04	88.45	2239.26
93.25	0.00	0.04	0.17	1.02	2.31	7.20	21.25	53.44	92.87	123.30	1749.05	2239.26
93.35	0.03	0.11	0.30	0.87	2.35	7.73	20.31	49.21	92.90	124.01	88.12	2239.26
93.45	0.05	0.11	0.19	0.96	2.28	7.55	20.34	47.85	90.67	129.46	84.69	2239.26
93.55	0.01	0.07	0.23	0.82	2.13	6.70	18.83	50.57	86.20	123.83	96.26	2239.26
93.65	0.02	0.08	0.17	0.85	2.52	8.14	20.08	46.50	90.91	125.19	87.68	2239.26
93.75	0.02	0.10	0.22	0.79	2.57	7.61	21.43	55.10	93.23	123.24	1741.41	2239.26
93.85	0.02	0.08	0.25	0.88	2.30	7.86	18.98	50.27	91.84	125.73	87.40	2239.26
93.95	0.03	0.12	0.23	0.93	2.25	7.43	20.18	49.82	91.99	127.27	86.96	2239.26
94.05	0.01	0.08	0.22	0.94	2.23	7.32	21.51	49.67	88.79	123.83	91.50	2239.26
94.15	0.03	0.09	0.22	0.81	2.42	7.61	20.16	47.25	90.73	126.38	87.34	2239.26

Midpt.	11000	2700	910	240	84	26	8.8	2.7	0.92	0.31	0.05	Volume
94.25	0.04	0.05	0.16	0.80	2.35	6.82	19.77	52.99	93.26	127.15	1751.20	2239.26
94.35	0.03	0.08	0.20	0.85	2.15	7.73	19.70	46.95	93.44	123.18	86.85	2239.26
94.45	0.05	0.05	0.21	0.83	2.57	7.66	20.64	45.59	90.15	126.68	87.01	2239.26
94.55	0.02	0.10	0.22	0.88	2.31	7.23	19.39	42.72	91.60	125.73	91.33	2239.26
94.65	0.00	0.07	0.22	0.66	2.40	7.78	20.87	48.76	91.33	123.65	89.56	2239.26
94.75	0.04	0.07	0.18	0.85	2.35	7.28	20.05	48.46	92.05	130.59	88.62	2239.26
94.85	0.05	0.06	0.18	0.88	2.30	7.28	20.82	52.38	93.29	128.34	85.19	2239.26
94.95	0.02	0.04	0.27	0.90	2.33	7.75	20.16	53.14	93.17	127.03	91.66	2239.26
95.05	0.03	0.06	0.18	0.87	2.33	7.91	20.21	52.08	92.18	125.07	84.24	2239.26
95.15	0.04	0.09	0.25	0.76	2.33	7.20	20.23	52.38	90.61	119.44	83.97	2239.26
95.25	0.03	0.09	0.23	0.76	2.23	7.36	19.90	49.67	86.17	120.33	83.53	2239.26
95.35	0.02	0.08	0.25	0.74	2.13	6.70	19.54	45.44	91.30	121.40	85.79	2239.26
95.45	0.02	0.04	0.23	0.69	2.42	7.66	19.49	50.87	87.68	124.96	91.33	2239.26
95.55	0.01	0.07	0.21	0.97	2.23	7.70	19.47	50.42	89.85	125.07	84.47	2239.26
95.65	0.03	0.07	0.25	0.88	2.30	7.84	20.34	47.55	88.61	120.86	84.96	2239.26
95.75	0.02	0.14	0.24	0.85	2.47	6.57	20.51	54.34	90.15	125.55	86.62	2239.26
95.85	0.01	0.11	0.18	0.89	2.80	6.89	21.33	54.19	88.49	125.85	92.38	2239.26
95.95	0.02	0.05	0.25	0.90	2.28	7.75	19.11	50.27	91.99	122.46	85.19	2239.26
96.05	0.02	0.07	0.22	0.96	2.67	7.75	19.72	51.02	92.87	125.55	90.72	2239.26
96.15	0.00	0.08	0.25	0.87	2.80	7.21	20.46	52.68	90.12	120.74	93.27	2239.26
96.25	0.00	0.07	0.27	0.74	2.35	6.66	20.31	48.91	93.71	124.54	93.32	2239.26
96.35	0.03	0.08	0.24	0.85	2.47	7.77	20.16	48.16	93.47	122.46	88.84	2239.26
96.45	0.02	0.11	0.25	0.79	2.21	7.23	19.19	51.48	89.13	119.20	85.13	2239.26
96.55	0.03	0.10	0.26	0.84	2.47	7.37	19.67	49.21	89.79	125.67	90.50	2239.26
96.65	0.02	0.04	0.23	0.79	1.88	7.05	20.46	49.36	91.54	121.81	95.54	2239.26
96.75	0.04	0.15	0.19	0.76	2.48	7.98	20.11	51.17	93.08	125.31	88.40	2239.26
96.85	0.01	0.07	0.14	0.99	2.43	8.23	19.95	53.89	89.91	125.96	88.56	2239.26
96.95	0.01	0.11	0.18	0.83	2.40	7.68	19.90	52.53	90.85	127.15	91.83	2239.26
97.05	0.03	0.10	0.15	0.97	2.31	7.27	19.82	52.38	90.82	124.72	85.79	2239.26
97.15	0.03	0.07	0.26	0.84	2.16	7.84	20.08	53.44	91.21	122.23	95.70	2239.26
97.25	0.03	0.08	0.26	0.75	2.50	7.14	20.44	53.14	93.17	126.91	91.27	2239.26
97.35	0.04	0.09	0.24	0.89	2.31	7.36	20.36	52.08	91.39	121.81	86.85	2239.26
97.45	0.02	0.09	0.20	0.80	2.47	7.39	19.80	52.53	91.63	127.86	91.16	2239.26
97.55	0.03	0.02	0.25	0.87	2.20	7.53	20.54	49.51	91.21	126.50	86.90	2239.26
97.65	0.03	0.16	0.20	0.95	2.28	7.45	21.08	54.19	92.18	122.41	86.74	2239.26
97.75	0.03	0.07	0.23	0.75	2.10	7.62	20.03	49.36	89.52	124.72	92.77	2239.26
97.85	0.03	0.09	0.25	0.89	1.90	7.57	20.57	48.16	91.15	120.33	93.38	2239.26
97.95	0.03	0.06	0.31	0.84	2.43	7.78	20.87	52.53	90.45	125.31	85.91	2239.26
98.05	0.00	0.14	0.25	0.73	2.40	7.55	19.95	51.93	92.11	118.73	84.91	2239.26
98.15	0.03	0.05	0.22	0.98	2.47	6.78	20.54	49.36	94.95	124.90	87.79	2239.26
98.25	0.03	0.07	0.22	0.95	2.37	6.98	20.03	52.68	90.67	122.11	89.12	2239.26
98.35	0.02	0.06	0.23	0.85	2.25	7.02	20.28	53.74	91.18	131.06	88.40	2239.26
98.45	0.02	0.06	0.18	0.81	2.01	7.87	21.20	53.59	87.25	117.54	89.28	2239.26
98.55	0.00	0.10	0.25	0.88	2.52	7.52	21.02	50.87	88.70	127.39	85.35	2239.26
98.65	0.00	0.09	0.25	0.85	2.15	7.11	20.77	53.59	90.76	123.95	89.28	2239.26

Midpt.	11000	2700	910	240	84	26	8.8	2.7	0.92	0.31	0.05	Volume
98.75	0.00	0.11	0.23	0.85	2.26	7.84	21.79	49.06	89.19	123.89	88.45	2239.26
98.85	0.02	0.11	0.24	0.84	2.45	6.91	20.90	52.53	89.97	125.49	92.60	2239.26
98.95	0.04	0.04	0.25	0.80	2.38	7.66	19.67	54.50	90.79	123.06	85.96	2239.26
99.05	0.01	0.08	0.25	0.75	2.43	7.46	20.57	55.85	92.78	126.73	89.34	2239.26
99.15	0.03	0.07	0.20	0.86	2.08	7.50	20.51	50.12	92.72	126.02	93.49	2239.26
99.25	0.01	0.07	0.18	0.85	2.77	7.82	19.98	53.14	91.06	126.02	87.40	2239.26
99.35	0.04	0.10	0.28	0.83	2.75	7.62	20.87	46.95	94.86	123.53	92.93	2239.26
99.45	0.03	0.09	0.25	0.96	2.23	7.55	19.98	55.70	91.30	119.44	88.84	2239.26
99.55	0.05	0.07	0.18	0.83	2.75	7.37	20.39	49.97	88.91	124.90	91.99	2239.26
99.65	0.03	0.04	0.26	0.84	1.93	7.46	20.46	48.91	91.27	126.20	87.46	2239.26
99.75	0.02	0.07	0.36	0.49	2.57	7.30	21.20	51.78	91.87	118.02	84.91	2239.26
99.85	0.00	0.01	0.02	0.00	0.29	1.29	0.31	24.46	25.03	36.18	1.94	2239.26

E.7. CavAb channel: Calcium and Barium

Table of Ba^{2+} population data for simulation with Ca^{2+} and Ba^{2+} across bulk density ratios (#JJF-GRS12G8)

	Population in histogram bin at Ba^{2+}/Ca^{2+} ratio:							
Midpoint	103	30	10	3	1	0.34	0.1	Volume
-99.95	0.000	0.000	0.000	0.000	0.000	0.000	0.000	2225.611
-99.85	0.000	0.000	0.000	0.000	0.000	0.000	0.000	2225.611
-99.75	0.000	0.000	0.000	0.000	0.000	0.000	0.000	2225.611
-99.65	0.000	0.000	0.000	9.034	0.000	2.502	0.000	2225.611
-99.55	98.641	76.835	67.154	127.277	59.417	34.828	9.216	2225.611
-99.45	143.141	109.176	99.228	126.253	85.483	34.096	13.166	2225.611
-99.35	142.115	110.806	97.239	127.632	88.430	33.910	14.434	2225.611
-99.25	143.209	111.007	97.930	127.569	86.746	33.713	13.314	2225.611
-99.15	142.687	111.353	97.009	126.901	85.272	34.533	13.668	2225.611
-99.05	140.222	109.912	97.846	126.692	85.763	34.981	14.041	2225.611
-98.95	141.066	109.139	99.123	127.047	85.261	33.353	13.628	2225.611
-98.85	142.806	110.319	96.897	126.734	84.863	32.402	14.218	2225.611
-98.75	142.563	110.228	97.483	125.209	84.793	33.976	13.186	2225.611
-98.65	141.769	111.840	97.302	125.731	82.372	33.812	13.677	2225.611
-98.55	142.495	110.958	97.065	126.807	84.161	33.855	13.491	2225.611
-98.45	141.679	111.061	95.021	123.841	83.962	32.217	13.726	2225.611
-98.35	142.245	108.635	96.130	126.431	85.097	32.949	14.552	2225.611
-98.25	139.842	110.027	99.025	125.470	85.974	33.866	14.011	2225.611
-98.15	141.798	109.735	96.660	124.373	85.284	32.949	13.677	2225.611
-98.05	140.092	109.413	96.653	124.718	85.986	32.173	13.766	2225.611
-97.95	140.896	110.800	95.439	123.830	83.167	34.620	14.837	2225.611
-97.85	141.242	109.620	96.737	122.963	85.728	33.768	12.940	2225.611
-97.75	142.585	110.581	97.693	125.815	84.313	33.451	14.080	2225.611
-97.65	141.327	110.654	97.337	124.206	84.021	33.757	13.992	2225.611
-97.55	140.103	108.568	96.862	124.321	85.296	34.194	14.414	2225.611

Midpoint	103	30	10	3	1	0.34	0.1	Volume
-97.45	138.771	109.760	96.235	125.178	82.781	32.381	13.599	2225.611
-97.35	140.426	109.267	96.618	125.000	83.904	32.752	13.284	2225.611
-97.25	140.296	108.975	97.358	121.898	83.962	34.074	13.520	2225.611
-97.15	140.551	109.079	96.660	120.415	83.904	32.927	13.687	2225.611
-97.05	139.525	107.856	96.953	123.402	82.162	33.233	13.766	2225.611
-96.95	137.751	108.756	96.493	123.214	83.483	33.331	13.333	2225.611
-96.85	140.460	108.379	95.976	123.601	84.056	33.025	14.630	2225.611
-96.75	140.568	109.979	96.988	123.966	83.834	32.883	14.611	2225.611
-96.65	138.652	109.444	97.337	122.013	83.986	33.451	13.383	2225.611
-96.55	138.471	109.529	95.844	121.637	82.512	33.473	13.707	2225.611
-96.45	139.077	108.245	96.102	120.687	80.945	33.156	13.088	2225.611
-96.35	138.845	108.860	95.362	121.689	82.606	32.643	13.736	2225.611
-96.25	137.853	109.821	95.502	123.193	83.436	33.047	12.970	2225.611
-96.15	139.718	108.866	95.774	120.770	81.717	33.265	13.687	2225.611
-96.05	138.811	109.085	95.509	121.188	84.454	32.686	13.422	2225.611
-95.95	137.842	108.817	95.383	121.501	82.442	33.528	14.041	2225.611
-95.85	139.400	109.845	96.507	122.201	82.840	33.058	13.373	2225.611
-95.75	139.774	107.722	96.883	122.170	83.319	32.981	14.277	2225.611
-95.65	138.369	108.434	96.221	120.676	81.261	34.008	14.129	2225.611
-95.55	137.269	108.787	95.453	121.376	82.758	32.588	14.110	2225.611
-95.45	138.221	108.142	97.972	120.081	83.541	31.965	13.343	2225.611
-95.35	138.488	107.236	95.537	122.170	84.196	32.566	13.559	2225.611
-95.25	138.000	109.352	96.046	122.556	82.583	31.004	13.805	2225.611
-95.15	135.535	109.784	95.621	123.392	81.565	32.992	13.481	2225.611
-95.05	139.514	107.680	93.967	122.567	81.647	33.855	13.530	2225.611
-94.95	138.312	107.722	95.055	119.590	82.068	33.691	13.894	2225.611
-94.85	137.638	108.501	96.130	123.277	83.366	32.228	13.186	2225.611
-94.75	136.703	108.038	96.681	120.791	83.331	31.976	13.137	2225.611
-94.65	138.170	107.899	97.016	122.097	81.647	31.998	13.412	2225.611
-94.55	134.651	108.276	95.537	122.138	82.477	32.326	13.609	2225.611
-94.45	138.743	107.248	96.981	118.922	83.004	32.872	13.147	2225.611
-94.35	137.825	106.384	94.169	120.363	79.846	32.271	13.569	2225.611
-94.25	137.105	108.446	95.809	118.901	80.571	32.632	13.333	2225.611
-94.15	138.289	109.018	96.681	120.478	81.939	32.359	13.107	2225.611
-94.05	138.550	108.933	94.483	121.783	81.799	32.457	13.363	2225.611
-93.95	137.048	108.045	94.560	121.407	81.612	32.927	13.451	2225.611
-93.85	138.930	107.990	95.704	122.389	82.805	33.058	13.451	2225.611
-93.75	135.002	106.980	95.781	121.658	81.156	31.878	13.274	2225.611
-93.65	136.742	109.431	94.602	120.530	82.360	31.889	13.569	2225.611
-93.55	137.796	107.041	97.142	122.577	81.928	32.337	13.216	2225.611
-93.45	136.567	108.847	96.437	119.381	78.957	31.649	13.668	2225.611
-93.35	135.099	108.002	93.974	121.752	80.092	31.823	13.029	2225.611
-93.25	136.856	108.245	96.262	118.755	82.910	32.555	13.559	2225.611
-93.15	136.045	108.464	94.839	119.016	81.051	33.746	13.589	2225.611
-93.05	136.482	107.552	95.279	121.888	80.981	32.741	13.353	2225.611

Midpoint	103	30	10	3	1	0.34	0.1	Volume
-92.95	137.519	108.689	95.655	120.906	81.893	32.326	13.589	2225.611
-92.85	137.088	109.565	95.202	121.773	81.296	31.933	12.999	2225.611
-92.75	139.412	106.877	95.348	120.185	81.238	32.282	13.078	2225.611
-92.65	134.702	108.641	96.688	121.971	82.501	32.446	13.324	2225.611
-92.55	137.932	108.191	96.737	120.342	80.536	33.615	13.422	2225.611
-92.45	138.267	107.917	95.432	121.689	80.864	32.381	13.864	2225.611
-92.35	136.442	108.695	95.132	120.394	81.670	32.228	13.147	2225.611
-92.25	136.125	108.282	95.055	121.428	81.834	32.817	13.697	2225.611
-92.15	137.439	108.720	96.828	118.838	82.021	33.451	13.628	2225.611
-92.05	138.771	106.998	95.558	118.431	81.869	32.479	13.717	2225.611
-91.95	137.037	108.744	96.255	121.919	82.828	33.265	12.714	2225.611
-91.85	136.640	108.774	97.239	123.005	80.712	33.626	13.373	2225.611
-91.75	136.442	107.990	96.018	117.961	82.442	32.413	13.491	2225.611
-91.65	137.830	109.723	95.962	120.248	84.056	32.970	13.933	2225.611
-91.55	137.269	111.049	96.737	119.475	81.542	31.517	13.196	2225.611
-91.45	138.386	109.510	96.060	120.154	81.004	32.949	12.783	2225.611
-91.35	137.371	107.601	96.744	121.648	81.437	33.735	13.520	2225.611
-91.25	139.667	110.064	96.660	118.776	80.197	32.544	13.461	2225.611
-91.15	138.567	109.529	95.795	120.467	83.705	32.260	13.481	2225.611
-91.05	139.882	108.659	97.393	121.804	84.325	31.670	13.471	2225.611
-90.95	139.179	109.395	97.783	122.086	83.577	31.965	13.933	2225.611
-90.85	139.678	108.270	96.911	121.042	83.273	33.637	13.628	2225.611
-90.75	138.539	108.549	97.609	122.253	83.121	32.752	13.500	2225.611
-90.65	141.441	108.762	96.311	121.867	85.073	32.555	13.962	2225.611
-90.55	138.766	111.530	97.086	121.815	81.261	32.796	13.274	2225.611
-90.45	141.305	109.687	99.228	121.021	82.758	32.544	13.383	2225.611
-90.35	139.984	109.839	97.511	122.358	84.372	33.768	14.316	2225.611
-90.25	140.794	109.437	98.000	124.781	83.729	33.134	13.992	2225.611
-90.15	141.730	111.007	98.767	123.099	82.968	33.331	13.992	2225.611
-90.05	141.747	110.617	98.823	124.185	84.898	33.702	13.884	2225.611
-89.95	140.324	108.908	97.756	122.838	83.553	33.528	13.206	2225.611
-89.85	141.469	110.611	97.735	125.282	84.150	32.752	13.746	2225.611
-89.75	141.650	111.189	99.381	124.290	83.635	34.085	13.717	2225.611
-89.65	140.358	112.473	98.286	121.178	83.530	33.659	13.903	2225.611
-89.55	139.264	110.867	99.290	124.091	84.302	34.019	13.874	2225.611
-89.45	141.888	112.120	97.435	125.919	83.354	32.850	13.962	2225.611
-89.35	140.364	112.175	98.963	124.802	83.962	34.205	13.884	2225.611
-89.25	142.115	111.633	98.293	125.804	84.489	33.364	14.365	2225.611
-89.15	140.273	111.451	100.246	123.997	82.875	33.517	13.825	2225.611
-89.05	141.486	111.238	98.300	126.149	84.021	34.281	13.589	2225.611
-88.95	141.860	111.293	96.876	125.021	83.191	34.183	13.333	2225.611
-88.85	142.427	113.866	99.109	124.875	84.980	33.528	14.591	2225.611
-88.75	141.174	112.290	98.572	125.115	85.132	32.817	13.491	2225.611
-88.65	141.214	110.624	97.818	123.423	83.506	32.861	14.287	2225.611
-88.55	140.681	111.633	99.067	125.909	86.804	34.030	13.677	2225.611

Midpoint	103	30	10	3	1	0.34	0.1	Volume
-88.45	143.050	110.617	95.997	124.269	84.477	34.150	14.375	2225.611
-88.35	141.089	111.694	97.811	126.681	84.161	33.222	14.287	2225.611
-88.25	141.877	110.982	98.816	125.595	84.898	34.019	13.432	2225.611
-88.15	141.118	112.089	98.649	125.741	84.220	33.407	14.188	2225.611
-88.05	142.750	113.513	98.865	126.817	84.956	33.648	13.314	2225.611
-87.95	144.422	114.091	98.439	125.125	86.746	33.986	14.581	2225.611
-87.85	141.866	113.026	99.835	124.426	83.202	34.434	13.844	2225.611
-87.75	143.220	111.183	100.058	125.595	86.266	33.440	13.766	2225.611
-87.65	142.438	112.053	99.102	124.530	87.225	33.702	13.530	2225.611
-87.55	144.507	112.339	100.149	125.919	82.629	33.451	13.559	2225.611
-87.45	143.169	111.347	99.053	125.992	86.734	34.402	13.265	2225.611
-87.35	143.401	112.448	100.093	125.272	85.401	32.850	14.061	2225.611
-87.25	142.733	112.606	98.397	123.674	87.623	33.735	14.011	2225.611
-87.15	142.954	113.312	98.544	125.198	84.594	34.139	13.569	2225.611
-87.05	142.631	112.527	99.939	123.809	86.921	33.440	14.090	2225.611
-86.95	142.778	111.061	100.511	126.232	85.892	32.741	13.805	2225.611
-86.85	141.038	112.911	98.830	126.598	85.448	33.997	14.365	2225.611
-86.75	142.891	111.524	98.272	124.436	86.254	34.456	13.854	2225.611
-86.65	142.534	111.153	98.704	123.663	86.126	33.331	14.041	2225.611
-86.55	143.923	110.843	97.595	122.619	86.582	33.080	14.159	2225.611
-86.45	143.345	112.473	100.770	125.825	86.769	35.002	14.021	2225.611
-86.35	142.597	113.093	98.607	124.457	84.571	34.729	14.739	2225.611
-86.25	142.829	112.314	98.753	126.076	85.553	32.774	13.668	2225.611
-86.15	144.087	113.148	99.779	124.300	84.723	34.981	14.532	2225.611
-86.05	145.124	111.791	98.376	125.209	85.108	33.025	13.874	2225.611
-85.95	144.036	110.806	99.891	124.405	84.641	34.074	13.579	2225.611
-85.85	144.246	111.031	99.751	124.060	85.822	33.899	13.481	2225.611
-85.75	142.597	111.627	97.128	124.530	84.302	34.063	13.569	2225.611
-85.65	142.166	113.525	98.502	126.264	86.500	34.172	15.014	2225.611
-85.55	141.254	112.990	99.123	121.836	85.389	35.516	13.952	2225.611
-85.45	141.662	111.469	99.897	124.300	84.547	35.276	13.736	2225.611
-85.35	142.897	111.335	100.553	126.661	85.272	34.107	14.699	2225.611
-85.25	143.617	112.016	99.256	124.081	85.237	33.375	14.021	2225.611
-85.15	144.138	112.454	100.937	126.546	88.219	33.943	14.424	2225.611
-85.05	141.764	112.941	100.316	124.248	86.535	35.057	13.992	2225.611
-84.95	141.826	110.982	99.458	122.932	86.418	34.008	14.758	2225.611
-84.85	141.939	109.900	99.932	128.457	85.272	33.976	14.237	2225.611
-84.75	145.238	112.242	99.130	124.969	86.699	33.986	14.591	2225.611
-84.65	144.410	110.697	98.739	125.522	88.278	34.576	14.188	2225.611
-84.55	145.306	111.913	99.842	126.723	84.547	34.249	14.385	2225.611
-84.45	144.212	111.147	98.244	125.940	85.261	33.855	13.854	2225.611
-84.35	144.501	111.171	99.242	125.324	87.038	33.855	13.618	2225.611
-84.25	141.803	112.400	101.223	125.982	85.787	33.331	13.884	2225.611
-84.15	142.676	111.183	99.835	126.107	85.249	34.074	13.186	2225.611
-84.05	142.041	112.631	97.560	126.003	84.360	32.817	14.080	2225.611

Midpoint	103	30	10	3	1	0.34	0.1	Volume
-83.95	142.925	110.496	97.909	126.065	86.266	33.244	14.051	2225.611
-83.85	143.855	112.442	98.551	125.825	84.454	34.817	13.972	2225.611
-83.75	144.631	112.345	98.635	123.684	84.161	33.440	13.913	2225.611
-83.65	143.090	111.238	99.430	126.379	85.939	32.905	14.208	2225.611
-83.55	142.551	113.288	98.907	127.256	85.518	34.096	14.228	2225.611
-83.45	141.225	113.203	97.574	125.835	85.611	34.085	14.355	2225.611
-83.35	142.098	112.102	98.293	125.230	85.354	34.423	14.316	2225.611
-83.25	143.719	114.468	97.979	125.533	83.904	33.877	13.648	2225.611
-83.15	142.602	111.566	98.404	124.373	87.377	32.675	14.178	2225.611
-83.05	143.362	112.181	99.584	122.932	84.150	34.303	13.766	2225.611
-82.95	143.452	112.740	100.637	124.551	84.676	34.391	13.825	2225.611
-82.85	142.336	111.901	98.690	125.209	84.383	33.659	13.147	2225.611
-82.75	141.968	112.673	98.425	124.332	84.758	34.216	14.129	2225.611
-82.65	143.061	112.996	98.900	126.399	85.342	33.670	13.402	2225.611
-82.55	142.132	112.479	98.481	124.227	85.494	32.938	13.894	2225.611
-82.45	143.379	112.795	98.893	124.634	85.494	34.729	14.336	2225.611
-82.35	141.401	112.041	98.963	125.470	86.196	34.380	14.061	2225.611
-82.25	143.424	112.071	97.756	123.653	85.237	34.860	13.972	2225.611
-82.15	141.900	112.722	99.179	122.661	86.325	32.817	14.237	2225.611
-82.05	143.588	112.874	98.418	124.885	84.325	34.423	13.756	2225.611
-81.95	143.243	110.861	99.800	124.739	83.471	32.730	13.206	2225.611
-81.85	142.274	111.439	97.881	122.316	82.583	33.713	14.277	2225.611
-81.75	142.568	112.023	99.311	124.321	86.126	33.407	14.110	2225.611
-81.65	140.675	111.828	100.204	123.433	85.775	33.746	13.795	2225.611
-81.55	144.240	112.515	98.697	124.467	86.208	33.659	13.952	2225.611
-81.45	141.809	111.195	98.090	125.084	85.529	33.801	13.884	2225.611
-81.35	142.245	111.463	97.874	122.514	84.500	33.790	14.041	2225.611
-81.25	142.376	112.874	99.737	125.209	86.313	34.369	13.707	2225.611
-81.15	142.217	111.889	99.542	126.514	84.208	34.653	13.933	2225.611
-81.05	142.325	112.649	98.858	122.640	84.641	34.675	14.169	2225.611
-80.95	140.653	112.971	97.316	124.875	86.804	34.281	14.326	2225.611
-80.85	142.585	110.289	97.902	122.191	85.108	33.779	13.579	2225.611
-80.75	141.378	111.530	96.876	125.501	85.050	33.582	14.473	2225.611
-80.65	141.526	111.992	97.093	125.209	85.915	34.325	14.463	2225.611
-80.55	141.401	111.305	98.760	123.319	85.565	33.604	13.923	2225.611
-80.45	140.386	110.988	98.111	122.671	83.998	33.986	14.178	2225.611
-80.35	142.404	111.572	100.149	123.966	85.869	34.817	14.365	2225.611
-80.25	140.653	111.877	98.342	126.692	83.097	34.260	14.355	2225.611
-80.15	141.463	110.386	98.118	122.661	84.302	34.161	13.766	2225.611
-80.05	142.750	111.901	97.846	123.423	84.395	34.139	13.864	2225.611
-79.95	143.299	112.977	98.488	122.556	83.881	34.238	14.188	2225.611
-79.85	142.308	111.822	96.779	124.384	85.062	33.484	13.894	2225.611
-79.75	143.135	110.660	98.237	122.264	84.465	33.965	13.903	2225.611
-79.65	142.194	111.317	98.028	126.044	86.091	33.768	13.756	2225.611
-79.55	141.220	111.597	97.588	123.078	85.646	33.091	14.051	2225.611

Midpoint	103	30	10	3	1	0.34	0.1	Volume
-79.45	142.886	111.262	98.160	125.324	86.675	35.035	13.569	2225.611
-79.35	140.624	112.321	99.709	124.144	84.992	33.254	14.316	2225.611
-79.25	141.242	112.613	97.895	125.658	85.822	35.112	13.982	2225.611
-79.15	142.160	111.262	97.818	124.551	86.605	33.670	13.628	2225.611
-79.05	142.506	111.451	98.223	121.512	85.144	33.549	14.002	2225.611
-78.95	143.197	110.569	98.656	125.345	85.085	33.943	13.854	2225.611
-78.85	140.862	111.086	99.039	125.386	85.342	34.096	13.677	2225.611
-78.75	138.335	110.113	96.765	122.295	83.436	33.757	13.255	2225.611
-78.65	142.058	111.737	98.097	126.744	82.781	32.599	13.166	2225.611
-78.55	142.364	111.797	97.686	125.219	86.570	33.429	14.247	2225.611
-78.45	140.913	112.771	98.544	124.843	85.097	33.254	13.785	2225.611
-78.35	141.701	112.010	96.925	124.248	83.623	33.375	13.677	2225.611
-78.25	141.282	111.846	97.316	123.423	86.161	33.965	14.178	2225.611
-78.15	140.171	110.587	98.544	123.789	84.582	33.069	13.923	2225.611
-78.05	140.959	111.937	97.958	123.747	85.565	33.003	14.237	2225.611
-77.95	139.361	110.319	98.753	125.595	84.372	34.511	13.992	2225.611
-77.85	141.588	112.010	97.079	124.164	84.851	33.473	14.119	2225.611
-77.75	143.180	111.384	98.425	124.718	85.962	33.615	14.287	2225.611
-77.65	140.137	110.903	98.279	123.872	84.033	33.921	13.805	2225.611
-77.55	141.446	111.080	98.900	123.705	85.892	33.495	14.169	2225.611
-77.45	141.225	112.892	97.504	124.833	83.284	33.254	13.697	2225.611
-77.35	143.815	110.173	99.577	122.483	84.652	34.303	13.844	2225.611
-77.25	141.820	111.816	97.972	123.527	84.781	34.369	13.510	2225.611
-77.15	141.231	111.250	97.476	122.316	82.115	34.139	14.002	2225.611
-77.05	142.104	109.638	97.979	125.010	84.068	33.506	14.906	2225.611
-76.95	142.444	111.810	96.821	124.833	85.600	33.473	13.333	2225.611
-76.85	141.378	110.788	97.351	123.433	85.108	32.883	14.119	2225.611
-76.75	139.043	110.003	97.239	122.974	86.009	32.960	14.454	2225.611
-76.65	141.356	112.138	99.507	126.431	84.302	32.741	14.758	2225.611
-76.55	141.832	109.158	98.949	124.008	84.980	34.096	14.110	2225.611
-76.45	141.497	111.280	98.349	123.914	83.881	32.839	13.707	2225.611
-76.35	142.880	110.210	98.607	124.300	83.530	33.976	13.628	2225.611
-76.25	141.384	110.678	95.579	122.420	85.062	33.309	14.061	2225.611
-76.15	141.322	110.441	98.579	125.292	83.577	33.943	13.707	2225.611
-76.05	139.814	110.471	97.616	124.102	83.635	33.244	14.051	2225.611
-75.95	142.444	110.788	97.958	122.577	85.307	33.790	13.343	2225.611
-75.85	141.786	111.201	98.049	125.846	84.512	33.331	13.677	2225.611
-75.75	140.177	109.973	99.674	122.640	83.331	32.621	13.864	2225.611
-75.65	141.775	109.547	97.979	124.123	85.073	33.582	13.864	2225.611
-75.55	142.087	111.116	97.149	123.402	84.746	33.691	14.051	2225.611
-75.45	141.061	109.979	97.888	125.209	83.752	33.407	13.677	2225.611
-75.35	140.228	113.342	98.830	126.661	84.547	34.314	13.648	2225.611
-75.25	142.070	110.630	96.667	125.209	85.939	33.178	13.894	2225.611
-75.15	139.502	112.114	96.995	123.402	85.857	33.080	13.186	2225.611
-75.05	140.460	109.340	97.721	122.995	81.624	33.473	13.972	2225.611

Midpoint	103	30	10	3	1	0.34	0.1	Volume
-74.95	141.871	110.867	97.351	124.718	84.840	34.270	13.825	2225.611
-74.85	142.670	111.092	98.760	124.603	85.284	33.386	14.689	2225.611
-74.75	142.138	110.100	97.735	126.358	84.980	33.123	14.129	2225.611
-74.65	139.791	111.360	98.649	124.144	86.407	33.986	13.776	2225.611
-74.55	143.339	111.165	97.986	122.796	83.740	33.473	13.500	2225.611
-74.45	142.517	111.743	96.046	122.849	84.243	33.790	13.874	2225.611
-74.35	141.707	110.259	98.090	122.619	83.460	34.303	13.746	2225.611
-74.25	142.602	110.916	98.446	122.723	86.032	32.457	14.149	2225.611
-74.15	141.106	110.100	97.979	124.488	84.676	33.418	13.864	2225.611
-74.05	142.030	111.074	95.900	123.172	84.348	33.134	14.051	2225.611
-73.95	141.441	110.289	98.118	123.778	83.354	34.478	14.571	2225.611
-73.85	141.832	110.812	99.018	123.339	84.103	33.691	14.552	2225.611
-73.75	140.630	110.198	97.107	123.872	84.781	32.905	14.021	2225.611
-73.65	141.775	111.141	100.051	123.580	85.296	34.609	13.442	2225.611
-73.55	142.070	111.037	96.472	124.300	84.068	33.462	13.609	2225.611
-73.45	143.152	109.699	98.907	123.601	82.852	33.626	13.363	2225.611
-73.35	139.746	110.843	98.223	122.650	84.044	33.965	13.736	2225.611
-73.25	140.335	110.885	99.570	122.880	85.424	33.681	13.884	2225.611
-73.15	139.270	109.942	98.153	123.245	84.243	34.402	13.736	2225.611
-73.05	138.851	110.325	97.065	122.629	85.144	32.861	13.648	2225.611
-72.95	140.239	110.903	99.311	123.789	86.149	33.746	14.503	2225.611
-72.85	141.718	110.167	99.067	123.433	85.284	33.145	13.324	2225.611
-72.75	140.188	110.319	97.958	123.695	86.032	33.528	13.658	2225.611
-72.65	138.902	110.660	97.365	124.039	86.781	33.888	13.756	2225.611
-72.55	141.089	109.881	96.102	121.762	83.764	34.478	14.552	2225.611
-72.45	139.763	108.860	99.779	125.439	86.138	33.560	14.129	2225.611
-72.35	140.930	111.019	98.076	122.650	85.986	32.599	13.815	2225.611
-72.25	140.398	109.991	97.853	124.008	84.325	34.686	13.972	2225.611
-72.15	139.400	110.861	97.239	125.512	84.816	32.741	13.825	2225.611
-72.05	141.135	107.856	98.300	123.642	82.711	34.456	14.002	2225.611
-71.95	142.132	110.289	98.544	122.828	81.834	33.691	13.677	2225.611
-71.85	140.483	110.119	97.986	122.869	83.425	33.091	13.599	2225.611
-71.75	141.197	108.756	97.225	123.997	85.190	33.276	13.363	2225.611
-71.65	141.628	111.749	98.209	123.402	85.728	33.866	14.454	2225.611
-71.55	142.138	110.471	96.772	124.123	84.348	32.938	13.923	2225.611
-71.45	142.716	109.322	97.595	123.433	84.641	34.991	13.844	2225.611
-71.35	138.794	111.153	96.123	124.979	84.044	33.102	14.267	2225.611
-71.25	139.270	110.149	98.104	123.078	82.840	32.599	13.039	2225.611
-71.15	141.259	108.537	96.793	122.473	84.617	33.156	14.129	2225.611
-71.05	139.395	110.143	96.528	122.212	83.869	33.560	13.392	2225.611
-70.95	140.466	110.514	98.069	124.530	84.419	33.418	13.707	2225.611
-70.85	142.665	109.480	98.439	121.501	85.623	33.713	13.707	2225.611
-70.75	141.509	110.830	99.081	125.313	84.968	34.205	13.383	2225.611
-70.65	140.216	111.420	95.662	123.256	84.033	32.675	14.473	2225.611
-70.55	140.732	110.964	98.090	122.546	84.688	33.254	14.287	2225.611

Midpoint	103	30	10	3	1	0.34	0.1	Volume
-70.45	140.267	109.547	96.039	125.094	84.699	32.916	13.962	2225.611
-70.35	140.284	110.958	96.102	123.841	85.319	33.812	13.491	2225.611
-70.25	141.537	110.715	97.149	123.569	84.395	33.943	14.090	2225.611
-70.15	141.645	111.846	98.014	123.078	85.459	33.954	13.559	2225.611
-70.05	142.036	111.165	97.525	122.295	84.091	33.779	14.434	2225.611
-69.95	138.590	109.681	96.221	122.285	84.407	32.960	14.002	2225.611
-69.85	139.729	110.471	97.693	124.175	83.869	32.621	13.186	2225.611
-69.75	141.667	112.047	98.725	124.687	83.916	32.774	14.110	2225.611
-69.65	142.948	110.739	96.214	127.590	84.255	33.844	14.061	2225.611
-69.55	140.483	110.490	97.469	123.820	85.483	33.593	14.237	2225.611
-69.45	138.562	109.364	97.288	123.559	82.431	33.397	13.805	2225.611
-69.35	140.749	107.382	96.890	124.091	85.050	33.866	13.697	2225.611
-69.25	141.231	108.987	97.414	121.950	82.922	32.468	14.424	2225.611
-69.15	141.968	111.074	97.302	122.514	85.810	33.156	13.668	2225.611
-69.05	139.004	108.866	96.730	120.896	82.746	33.713	14.070	2225.611
-68.95	140.760	110.009	98.188	122.734	85.038	32.949	14.110	2225.611
-68.85	140.568	108.750	96.025	121.836	85.319	32.173	13.825	2225.611
-68.75	140.375	110.569	97.609	125.700	83.483	33.440	13.550	2225.611
-68.65	139.950	112.254	98.356	122.681	83.799	32.839	14.011	2225.611
-68.55	139.508	111.372	98.265	120.917	84.617	33.921	14.208	2225.611
-68.45	140.828	111.134	97.476	123.997	85.775	32.872	13.785	2225.611
-68.35	140.902	110.575	97.497	121.856	84.898	34.008	13.923	2225.611
-68.25	139.922	109.602	96.800	125.042	85.611	34.041	13.913	2225.611
-68.15	142.381	109.243	99.095	122.932	82.431	33.560	13.933	2225.611
-68.05	140.024	110.946	96.646	119.830	85.588	32.632	13.884	2225.611
-67.95	139.735	110.429	97.937	123.527	83.810	33.440	13.658	2225.611
-67.85	139.621	110.873	96.500	122.859	82.524	33.582	13.648	2225.611
-67.75	139.344	110.021	96.862	124.958	85.483	33.407	13.599	2225.611
-67.65	140.862	111.323	98.502	122.587	83.121	34.172	14.237	2225.611
-67.55	141.424	110.356	97.225	121.804	83.600	33.047	13.461	2225.611
-67.45	140.126	111.019	96.911	125.261	84.676	34.063	13.972	2225.611
-67.35	141.860	109.395	97.818	121.731	84.372	33.528	13.894	2225.611
-67.25	140.981	109.815	97.839	123.225	84.068	33.244	13.795	2225.611
-67.15	140.749	109.389	97.518	125.324	85.377	31.703	14.139	2225.611
-67.05	139.848	111.025	97.344	121.867	84.442	32.708	14.041	2225.611
-66.95	139.508	109.748	95.446	122.159	85.389	34.052	14.178	2225.611
-66.85	140.743	109.778	97.790	123.308	83.635	32.555	13.687	2225.611
-66.75	139.712	109.571	97.923	124.822	83.647	33.407	13.343	2225.611
-66.65	139.757	110.569	96.674	121.376	84.360	33.615	13.736	2225.611
-66.55	141.446	110.855	97.462	122.483	85.880	32.173	14.444	2225.611
-66.45	140.908	111.925	96.876	122.306	83.460	32.763	14.277	2225.611
-66.35	141.197	110.471	97.783	123.496	83.471	33.691	14.444	2225.611
-66.25	141.100	110.508	96.367	122.504	83.331	34.303	13.500	2225.611
-66.15	140.131	109.796	98.935	125.386	85.331	33.233	14.218	2225.611
-66.05	140.409	109.541	96.925	126.117	84.559	32.839	14.562	2225.611

Midpoint	103	30	10	3	1	0.34	0.1	Volume
-65.95	141.469	110.885	98.342	122.567	84.875	33.014	14.355	2225.611
-65.85	140.709	110.526	98.125	121.689	84.442	33.418	13.835	2225.611
-65.75	141.100	111.706	96.339	123.433	84.115	33.681	13.599	2225.611
-65.65	140.653	110.295	98.181	123.329	85.179	33.724	13.913	2225.611
-65.55	139.905	110.423	97.658	124.467	85.050	32.555	14.198	2225.611
-65.45	140.653	109.997	96.946	123.715	83.050	33.233	14.178	2225.611
-65.35	139.565	111.889	96.807	124.781	82.512	33.768	14.287	2225.611
-65.25	141.435	110.909	96.667	123.642	85.062	35.330	13.992	2225.611
-65.15	141.571	110.307	96.548	121.930	84.407	33.418	13.265	2225.611
-65.05	141.401	111.195	98.328	124.812	85.108	33.866	14.552	2225.611
-64.95	140.619	110.283	97.469	122.640	82.828	33.003	13.776	2225.611
-64.85	139.735	111.207	97.407	122.285	83.530	32.719	13.923	2225.611
-64.75	141.424	110.824	96.053	123.778	86.114	34.762	13.530	2225.611
-64.65	141.072	110.745	98.153	121.992	84.477	33.549	13.432	2225.611
-64.55	140.290	110.319	96.297	120.509	84.851	34.664	14.739	2225.611
-64.45	141.220	111.311	97.114	125.303	82.337	32.588	14.621	2225.611
-64.35	142.177	109.863	97.225	124.102	84.056	33.539	14.296	2225.611
-64.25	138.879	109.072	96.046	123.820	85.974	34.052	13.717	2225.611
-64.15	139.990	110.684	96.709	122.619	85.857	33.353	13.412	2225.611
-64.05	140.987	109.675	96.786	122.567	84.676	34.150	13.884	2225.611
-63.95	140.760	110.502	97.386	123.956	85.214	33.593	13.952	2225.611
-63.85	139.100	109.869	98.404	123.465	84.407	33.462	13.422	2225.611
-63.75	139.865	109.912	96.953	123.486	84.594	34.762	14.257	2225.611
-63.65	140.624	109.425	96.786	124.593	86.290	33.069	13.825	2225.611
-63.55	141.792	109.091	97.107	122.974	82.933	33.768	14.119	2225.611
-63.45	139.888	110.709	96.946	123.204	84.629	33.364	13.668	2225.611
-63.35	140.035	109.748	97.267	123.413	83.436	32.599	13.677	2225.611
-63.25	139.621	108.829	96.842	123.371	84.980	33.495	13.559	2225.611
-63.15	139.780	111.487	96.500	125.136	84.664	32.916	13.795	2225.611
-63.05	139.814	110.763	97.002	124.969	82.127	34.642	14.434	2225.611
-62.95	140.454	109.620	97.169	122.368	82.805	32.796	14.031	2225.611
-62.85	141.424	110.490	96.486	123.987	84.348	32.905	13.176	2225.611
-62.75	138.664	109.875	96.918	123.601	83.413	33.473	14.041	2225.611
-62.65	141.945	109.225	95.788	122.640	84.582	32.468	14.306	2225.611
-62.55	141.973	110.228	96.332	122.379	86.184	33.549	14.070	2225.611
-62.45	140.851	109.906	97.190	123.903	83.694	32.228	14.513	2225.611
-62.35	141.106	110.380	97.476	125.324	85.003	33.615	13.618	2225.611
-62.25	141.118	110.897	96.604	124.091	82.887	33.823	14.355	2225.611
-62.15	139.553	109.085	97.749	123.475	85.845	33.320	13.972	2225.611
-62.05	140.324	109.243	95.293	122.869	84.255	33.473	13.992	2225.611
-61.95	141.690	110.167	98.670	125.146	84.781	32.621	13.481	2225.611
-61.85	139.933	110.551	96.800	123.945	84.500	33.506	13.412	2225.611
-61.75	139.361	109.930	98.237	124.154	82.758	33.637	13.972	2225.611
-61.65	141.135	110.964	98.049	125.762	83.834	33.888	14.031	2225.611
-61.55	139.871	110.465	98.509	123.538	83.273	34.270	14.051	2225.611

Midpoint	103	30	10	3	1	0.34	0.1	Volume
-61.45	141.509	110.739	97.574	121.491	84.500	33.440	13.894	2225.611
-61.35	139.848	111.378	97.462	124.227	83.694	33.549	13.933	2225.611
-61.25	141.129	110.873	96.381	126.097	82.524	31.944	14.090	2225.611
-61.15	140.160	108.330	97.239	124.822	84.875	32.730	13.451	2225.611
-61.05	140.959	109.967	97.511	126.023	85.705	33.855	13.569	2225.611
-60.95	142.149	110.873	97.330	122.869	83.413	33.014	13.628	2225.611
-60.85	139.752	110.180	97.149	122.076	82.489	33.910	13.422	2225.611
-60.75	138.363	108.774	95.935	123.862	85.167	33.134	13.923	2225.611
-60.65	140.517	111.122	96.876	120.447	85.085	34.008	14.149	2225.611
-60.55	139.701	111.183	97.804	120.687	84.243	33.615	12.823	2225.611
-60.45	140.913	110.307	97.672	125.825	83.553	32.938	13.304	2225.611
-60.35	139.661	110.283	97.609	122.076	85.401	33.965	13.569	2225.611
-60.25	141.112	110.046	97.002	125.136	85.787	34.489	14.051	2225.611
-60.15	139.315	111.025	97.679	123.162	85.413	33.670	13.442	2225.611
-60.05	138.890	110.976	98.181	124.060	83.962	33.495	13.520	2225.611
-59.95	142.353	111.572	97.176	126.034	85.272	33.713	13.933	2225.611
-59.85	139.774	110.265	95.893	121.919	83.810	33.560	13.500	2225.611
-59.75	139.599	110.496	97.065	124.279	85.693	32.796	13.894	2225.611
-59.65	141.713	110.964	96.842	123.632	81.787	32.872	13.913	2225.611
-59.55	139.650	108.701	97.442	125.804	83.740	32.916	14.503	2225.611
-59.45	138.953	110.459	96.772	124.676	84.945	33.954	13.923	2225.611
-59.35	142.098	109.249	97.211	124.582	84.044	33.462	13.048	2225.611
-59.25	140.851	110.684	97.428	123.538	81.939	33.670	14.444	2225.611
-59.15	140.647	109.881	96.388	122.107	85.307	33.233	13.491	2225.611
-59.05	140.284	109.748	96.793	123.381	84.840	33.965	13.795	2225.611
-58.95	140.460	111.737	96.814	123.789	86.056	35.046	14.178	2225.611
-58.85	141.928	109.924	98.216	124.123	85.237	33.549	13.913	2225.611
-58.75	140.109	109.517	96.360	123.037	85.190	33.473	13.569	2225.611
-58.65	141.446	109.583	97.351	122.493	82.700	32.064	14.021	2225.611
-58.55	139.077	110.180	97.902	124.739	84.571	31.747	14.463	2225.611
-58.45	140.556	109.632	96.479	123.935	85.132	33.331	13.962	2225.611
-58.35	141.707	109.249	97.930	124.352	84.758	33.997	14.051	2225.611
-58.25	139.916	111.134	97.176	125.428	84.582	33.244	13.736	2225.611
-58.15	141.441	110.946	96.932	123.329	82.910	35.079	13.726	2225.611
-58.05	140.777	110.009	97.630	124.969	85.740	32.643	14.650	2225.611
-57.95	138.715	110.362	97.435	121.219	84.711	34.194	13.589	2225.611
-57.85	140.749	110.721	96.493	121.512	82.968	32.883	14.031	2225.611
-57.75	139.230	110.532	96.283	122.817	84.185	33.637	14.581	2225.611
-57.65	140.449	109.748	97.400	123.172	83.202	34.281	14.178	2225.611
-57.55	142.948	111.019	95.034	125.021	82.957	33.244	13.383	2225.611
-57.45	139.128	110.380	96.409	122.389	84.793	33.058	13.854	2225.611
-57.35	141.503	109.267	97.086	122.650	85.296	33.604	13.766	2225.611
-57.25	141.752	109.346	96.751	121.930	85.822	33.899	14.208	2225.611
-57.15	140.913	109.778	96.297	126.911	85.459	33.233	13.825	2225.611
-57.05	140.715	111.238	97.658	123.621	85.179	33.615	13.461	2225.611

Midpoint	103	30	10	3	1	0.34	0.1	Volume
-56.95	140.596	110.605	96.883	123.663	83.717	33.265	13.884	2225.611
-56.85	140.375	110.313	97.379	123.131	84.641	33.091	13.540	2225.611
-56.75	142.064	109.407	97.204	124.373	85.003	33.954	13.785	2225.611
-56.65	140.749	109.316	97.253	123.507	84.442	33.309	13.992	2225.611
-56.55	140.619	111.201	97.749	125.146	84.758	33.986	14.119	2225.611
-56.45	141.271	110.763	96.074	123.527	84.489	34.456	13.648	2225.611
-56.35	139.746	109.906	96.325	124.321	84.816	34.194	14.375	2225.611
-56.25	139.553	110.338	97.749	123.527	83.577	34.063	13.658	2225.611
-56.15	139.230	111.159	96.611	124.864	84.828	31.900	13.992	2225.611
-56.05	140.602	110.891	96.451	123.507	85.027	32.785	13.785	2225.611
-55.95	140.120	110.617	98.244	125.094	84.898	33.681	13.471	2225.611
-55.85	139.497	109.334	97.072	122.577	84.173	32.850	13.756	2225.611
-55.75	139.179	110.520	96.995	123.830	83.974	33.681	14.090	2225.611
-55.65	139.825	110.015	97.128	122.420	84.348	33.091	13.599	2225.611
-55.55	140.046	108.683	96.737	123.569	83.752	33.473	13.697	2225.611
-55.45	140.080	108.482	96.967	121.052	85.611	34.270	13.206	2225.611
-55.35	140.307	109.072	96.632	122.786	85.553	32.883	13.864	2225.611
-55.25	140.573	109.912	99.025	121.188	85.494	32.588	13.844	2225.611
-55.15	139.848	110.040	96.465	123.882	85.763	34.467	13.795	2225.611
-55.05	141.917	109.729	97.030	120.927	81.647	34.205	13.933	2225.611
-54.95	142.455	109.437	97.937	124.300	82.816	32.763	13.451	2225.611
-54.85	141.129	111.512	99.737	123.193	83.986	32.599	14.414	2225.611
-54.75	142.064	112.156	97.051	123.789	84.945	33.626	13.127	2225.611
-54.65	139.933	111.426	97.295	122.796	85.459	32.970	13.933	2225.611
-54.55	140.749	110.800	96.521	123.089	86.149	34.183	14.149	2225.611
-54.45	141.526	110.222	96.548	122.024	83.916	33.659	14.365	2225.611
-54.35	139.837	110.094	96.297	122.661	83.658	33.593	13.785	2225.611
-54.25	140.891	110.113	98.418	123.841	83.050	34.052	14.532	2225.611
-54.15	140.692	111.451	98.286	122.901	85.108	34.620	13.442	2225.611
-54.05	141.446	110.131	95.551	125.741	85.529	33.593	14.444	2225.611
-53.95	140.919	109.766	95.865	121.606	84.150	33.615	13.726	2225.611
-53.85	140.828	110.210	96.528	123.726	83.530	33.069	14.434	2225.611
-53.75	140.449	110.289	96.730	123.799	84.688	33.626	14.031	2225.611
-53.65	139.514	111.688	96.221	123.392	84.419	33.997	14.365	2225.611
-53.55	139.548	111.025	97.679	124.008	84.150	33.440	13.952	2225.611
-53.45	141.220	109.304	98.683	125.585	85.483	33.659	13.373	2225.611
-53.35	138.845	108.178	96.193	121.449	85.822	33.560	13.668	2225.611
-53.25	140.046	109.924	98.035	124.164	84.419	33.506	14.218	2225.611
-53.15	139.655	110.581	97.721	124.843	85.494	32.927	12.999	2225.611
-53.05	140.284	110.757	96.855	123.308	85.237	32.981	13.481	2225.611
-52.95	140.143	109.577	97.086	122.003	83.050	33.910	14.139	2225.611
-52.85	140.670	111.651	96.402	123.235	82.395	33.866	13.579	2225.611
-52.75	140.777	109.504	96.583	123.214	87.120	32.654	14.090	2225.611
-52.65	141.152	109.942	96.786	124.185	84.302	33.560	13.648	2225.611
-52.55	140.647	110.958	98.251	123.266	85.798	32.654	13.726	2225.611

Midpoint	103	30	10	3	1	0.34	0.1	Volume
-52.45	140.307	109.802	96.311	122.713	87.295	33.615	14.611	2225.611
-52.35	138.579	110.733	97.016	124.321	83.284	34.161	13.776	2225.611
-52.25	139.746	111.530	97.197	124.102	83.998	33.582	13.805	2225.611
-52.15	139.627	109.900	96.828	123.663	82.045	34.085	13.628	2225.611
-52.05	141.100	110.873	98.446	120.300	85.471	34.260	13.952	2225.611
-51.95	140.562	110.630	96.981	122.817	83.121	32.697	14.169	2225.611
-51.85	139.378	109.687	99.116	120.917	85.459	32.927	14.385	2225.611
-51.75	139.899	109.675	97.532	124.499	82.933	33.462	13.461	2225.611
-51.65	141.100	111.907	97.923	123.736	84.886	32.938	13.795	2225.611
-51.55	139.434	109.748	96.653	126.191	84.711	33.364	13.844	2225.611
-51.45	142.240	109.547	97.944	123.057	84.980	34.522	14.051	2225.611
-51.35	138.681	110.253	97.553	121.877	83.810	33.724	14.591	2225.611
-51.25	139.956	109.760	96.381	123.538	83.752	33.102	13.756	2225.611
-51.15	140.222	110.964	98.195	124.175	82.325	33.145	13.756	2225.611
-51.05	141.424	108.562	97.993	123.611	86.956	33.276	14.866	2225.611
-50.95	139.094	111.195	97.393	122.922	84.886	34.369	14.061	2225.611
-50.85	141.418	109.529	97.972	122.410	86.056	32.512	13.756	2225.611
-50.75	140.556	111.189	97.469	124.927	84.033	33.102	13.854	2225.611
-50.65	142.551	110.940	96.444	123.360	83.296	33.003	14.188	2225.611
-50.55	142.019	112.533	96.981	124.582	83.495	33.254	13.815	2225.611
-50.45	138.998	108.057	97.846	122.191	86.453	33.626	14.051	2225.611
-50.35	140.636	109.237	98.628	124.332	84.115	33.844	13.972	2225.611
-50.25	140.398	110.916	98.523	122.149	83.752	33.549	14.198	2225.611
-50.15	140.976	110.119	97.916	124.614	84.115	33.888	14.503	2225.611
-50.05	139.967	109.748	96.890	119.214	84.348	32.741	14.336	2225.611
-49.95	141.203	110.648	96.430	124.467	84.325	33.254	13.550	2225.611
-49.85	141.679	111.481	97.686	124.614	83.226	33.320	14.444	2225.611
-49.75	140.692	111.846	98.195	124.551	82.805	32.970	13.422	2225.611
-49.65	142.461	109.906	97.567	122.295	82.793	33.058	13.815	2225.611
-49.55	140.324	109.936	98.342	122.065	84.758	33.265	13.687	2225.611
-49.45	140.154	110.593	96.800	123.423	85.623	33.440	14.011	2225.611
-49.35	140.721	110.654	97.016	122.890	85.646	33.298	14.345	2225.611
-49.25	139.230	111.280	97.979	124.394	82.688	33.517	14.100	2225.611
-49.15	139.400	110.204	95.907	126.159	84.021	33.768	14.090	2225.611
-49.05	140.534	111.883	98.069	126.023	84.816	33.014	13.048	2225.611
-48.95	139.769	110.192	98.097	121.501	83.822	33.375	13.442	2225.611
-48.85	140.273	110.192	95.948	123.569	85.073	34.303	14.296	2225.611
-48.75	140.375	109.626	96.528	121.084	82.384	33.844	13.294	2225.611
-48.65	139.695	112.071	97.023	123.695	84.044	33.287	14.424	2225.611
-48.55	140.273	110.265	99.290	123.277	85.144	33.342	14.080	2225.611
-48.45	141.066	110.544	97.295	122.828	84.547	34.118	13.903	2225.611
-48.35	141.004	110.861	97.051	123.193	84.454	34.249	13.324	2225.611
-48.25	141.049	111.560	96.814	124.227	85.354	33.211	13.559	2225.611
-48.15	140.908	111.171	98.342	123.851	83.553	34.096	13.500	2225.611
-48.05	140.216	109.669	97.902	124.018	84.278	33.375	13.746	2225.611

Midpoint	103	30	10	3	1	0.34	0.1	Volume
-47.95	139.553	112.825	96.786	125.773	83.881	34.194	14.188	2225.611
-47.85	140.749	112.351	97.623	124.572	84.758	32.555	13.628	2225.611
-47.75	142.064	110.703	97.190	126.754	85.717	33.276	13.785	2225.611
-47.65	141.520	111.512	97.672	124.175	85.845	32.839	14.404	2225.611
-47.55	141.667	112.351	95.886	123.319	84.980	34.260	13.599	2225.611
-47.45	140.834	110.070	98.014	121.762	85.436	34.139	13.864	2225.611
-47.35	140.590	111.724	98.258	123.632	85.319	33.986	13.913	2225.611
-47.25	141.095	111.372	97.972	122.744	85.050	34.063	14.178	2225.611
-47.15	141.735	110.100	96.618	126.295	84.863	34.205	13.383	2225.611
-47.05	140.086	111.445	96.423	123.976	83.448	33.724	13.795	2225.611
-46.95	139.961	111.299	97.651	122.065	85.354	32.304	12.901	2225.611
-46.85	139.701	110.368	96.932	125.439	82.395	32.960	13.540	2225.611
-46.75	143.753	109.231	97.700	122.337	85.190	32.970	14.051	2225.611
-46.65	139.956	109.954	98.942	125.157	83.834	34.194	13.540	2225.611
-46.55	140.154	111.439	98.125	123.601	83.612	34.238	14.011	2225.611
-46.45	140.001	109.735	98.188	123.235	83.004	34.664	13.540	2225.611
-46.35	141.276	111.536	96.423	123.360	83.027	33.779	14.640	2225.611
-46.25	141.373	111.256	97.700	124.070	85.097	33.528	13.668	2225.611
-46.15	140.409	111.025	96.590	122.389	84.395	32.916	14.237	2225.611
-46.05	141.633	111.524	98.516	123.392	84.044	32.435	14.277	2225.611
-45.95	140.024	109.158	97.637	121.585	84.115	33.637	13.658	2225.611
-45.85	138.386	111.317	97.162	123.903	84.056	33.233	13.658	2225.611
-45.75	140.432	109.371	98.446	126.295	83.951	33.418	14.031	2225.611
-45.65	141.095	111.128	97.197	121.762	83.974	33.615	14.188	2225.611
-45.55	140.766	111.870	98.509	122.953	84.150	32.774	13.609	2225.611
-45.45	140.732	108.537	97.630	125.125	84.547	34.860	13.815	2225.611
-45.35	141.123	110.508	97.058	122.399	84.115	32.850	13.363	2225.611
-45.25	140.981	109.875	98.125	125.261	83.565	34.478	14.237	2225.611
-45.15	140.432	110.636	99.604	121.543	85.202	33.025	13.284	2225.611
-45.05	141.361	110.836	98.425	121.846	83.296	33.407	14.473	2225.611
-44.95	140.279	111.506	96.702	123.329	83.553	33.298	13.726	2225.611
-44.85	140.477	110.167	96.960	125.115	85.097	33.888	13.707	2225.611
-44.75	140.556	110.976	96.883	124.979	85.845	32.883	14.267	2225.611
-44.65	142.053	111.664	97.721	122.462	83.694	33.888	13.009	2225.611
-44.55	140.415	110.727	98.167	122.118	84.044	32.828	13.422	2225.611
-44.45	141.225	111.700	96.744	124.561	83.916	34.107	13.333	2225.611
-44.35	140.908	109.243	98.049	123.131	84.079	34.270	13.854	2225.611
-44.25	139.536	109.285	98.830	122.598	84.840	33.615	13.913	2225.611
-44.15	140.449	112.406	97.337	125.564	84.115	33.134	13.392	2225.611
-44.05	142.506	111.755	96.946	123.423	85.518	33.091	13.422	2225.611
-43.95	143.209	110.879	97.769	122.232	84.442	33.189	13.687	2225.611
-43.85	141.344	110.593	97.051	120.436	85.073	33.713	13.245	2225.611
-43.75	139.644	111.323	97.128	123.172	84.992	33.047	13.481	2225.611
-43.65	141.679	111.664	99.025	122.567	86.617	33.167	13.972	2225.611
-43.55	140.267	111.341	98.600	124.530	84.559	33.604	13.697	2225.611

Midpoint	103	30	10	3	1	0.34	0.1	Volume
-43.45	141.843	110.234	98.111	124.029	85.038	33.724	13.559	2225.611
-43.35	140.211	109.675	97.756	125.104	84.208	34.260	13.500	2225.611
-43.25	141.616	110.234	97.539	124.164	83.974	33.331	13.579	2225.611
-43.15	139.706	109.492	98.056	123.559	84.746	34.402	13.717	2225.611
-43.05	142.262	111.712	99.584	125.606	84.512	34.576	13.972	2225.611
-42.95	142.268	112.333	96.660	123.047	85.997	33.102	13.589	2225.611
-42.85	140.573	110.885	96.723	123.057	85.728	32.992	13.776	2225.611
-42.75	142.240	109.772	99.877	121.846	85.108	33.069	13.559	2225.611
-42.65	140.709	113.184	98.244	120.353	82.138	34.227	13.402	2225.611
-42.55	142.075	112.278	98.432	124.634	86.699	34.139	13.284	2225.611
-42.45	142.852	112.205	97.351	126.232	84.571	33.888	14.296	2225.611
-42.35	143.022	111.019	97.462	125.397	85.108	33.003	13.894	2225.611
-42.25	143.957	110.350	98.914	124.405	85.120	33.560	13.805	2225.611
-42.15	140.358	111.457	98.886	123.548	85.097	33.659	13.736	2225.611
-42.05	141.027	111.791	99.639	125.334	86.781	33.855	13.510	2225.611
-41.95	142.092	110.544	99.339	122.922	85.834	33.790	14.326	2225.611
-41.85	141.679	110.763	99.639	123.611	82.407	32.872	14.159	2225.611
-41.75	140.896	111.049	97.169	123.141	85.272	33.812	13.599	2225.611
-41.65	142.693	110.770	98.237	124.321	81.612	33.407	13.186	2225.611
-41.55	142.257	111.797	98.935	124.802	85.225	33.582	14.562	2225.611
-41.45	140.874	111.299	97.623	123.715	84.688	33.429	13.992	2225.611
-41.35	141.945	111.463	96.709	123.245	83.962	34.030	14.424	2225.611
-41.25	141.650	111.986	97.602	123.057	85.448	34.270	13.363	2225.611
-41.15	142.659	112.272	99.214	124.321	84.933	33.233	13.972	2225.611
-41.05	141.214	112.588	98.586	126.107	84.255	32.916	13.736	2225.611
-40.95	141.401	111.627	97.923	123.695	84.933	33.080	13.766	2225.611
-40.85	142.795	112.156	99.597	126.170	85.144	34.762	14.444	2225.611
-40.75	142.319	111.682	98.139	125.063	84.840	33.386	13.992	2225.611
-40.65	141.554	110.246	97.183	121.627	84.535	33.539	14.483	2225.611
-40.55	141.951	111.779	98.551	124.091	86.184	34.926	13.707	2225.611
-40.45	141.214	111.043	98.342	125.219	84.547	32.850	14.228	2225.611
-40.35	141.044	111.019	97.365	123.809	85.588	33.855	14.591	2225.611
-40.25	141.951	110.240	97.190	122.514	86.173	34.074	14.257	2225.611
-40.15	140.596	110.849	98.851	124.018	85.880	33.997	13.697	2225.611
-40.05	141.509	111.475	98.711	124.426	84.910	33.517	14.581	2225.611
-39.95	140.879	110.392	98.921	123.402	84.793	33.145	14.002	2225.611
-39.85	142.132	112.120	98.028	123.047	85.436	33.921	14.522	2225.611
-39.75	140.993	111.925	98.858	124.185	86.488	34.565	13.284	2225.611
-39.65	141.225	111.743	98.788	123.402	84.173	34.063	13.668	2225.611
-39.55	141.633	111.287	97.735	124.332	83.892	33.768	14.375	2225.611
-39.45	141.622	111.463	98.363	123.141	85.974	33.517	14.031	2225.611
-39.35	140.658	112.716	98.523	124.770	84.933	34.118	14.198	2225.611
-39.25	140.930	110.137	98.314	123.381	84.535	34.380	13.874	2225.611
-39.15	144.025	110.678	99.751	124.812	84.348	33.691	13.471	2225.611
-39.05	141.849	111.688	97.958	124.958	84.641	33.014	14.228	2225.611

Midpoint	103	30	10	3	1	0.34	0.1	Volume
-38.95	141.565	112.460	97.832	125.397	86.477	33.833	14.247	2225.611
-38.85	143.350	110.593	96.521	123.068	83.927	32.807	14.159	2225.611
-38.75	140.715	109.425	98.956	125.606	85.997	33.844	14.080	2225.611
-38.65	142.602	112.527	98.614	123.005	85.541	33.844	14.758	2225.611
-38.55	142.608	111.499	98.628	124.499	84.044	34.303	14.149	2225.611
-38.45	142.047	111.962	99.486	127.172	82.524	33.702	13.265	2225.611
-38.35	140.426	112.035	98.216	124.969	85.822	32.992	14.070	2225.611
-38.25	141.594	110.788	98.802	125.167	84.758	34.620	14.267	2225.611
-38.15	142.840	111.037	99.800	123.747	84.196	33.659	13.520	2225.611
-38.05	144.082	111.366	100.560	123.663	86.418	34.019	14.463	2225.611
-37.95	141.809	112.217	100.093	126.744	85.413	33.812	14.503	2225.611
-37.85	139.984	111.366	99.709	125.898	86.699	33.757	14.257	2225.611
-37.75	143.736	111.378	97.469	127.162	86.570	33.823	14.886	2225.611
-37.65	144.150	111.767	99.842	125.198	85.167	33.997	14.090	2225.611
-37.55	141.973	113.014	98.258	125.397	83.577	33.528	13.982	2225.611
-37.45	143.985	112.223	100.163	125.449	86.921	34.555	14.090	2225.611
-37.35	142.733	112.807	99.625	124.802	84.910	35.188	14.041	2225.611
-37.25	143.141	113.111	98.614	125.710	85.155	33.058	14.257	2225.611
-37.15	142.597	112.795	100.609	123.757	84.910	34.456	14.061	2225.611
-37.05	142.092	113.531	98.753	122.243	85.120	33.331	14.345	2225.611
-36.95	141.174	110.903	99.995	125.000	87.786	33.211	13.599	2225.611
-36.85	143.872	111.019	99.402	125.752	86.021	33.833	14.483	2225.611
-36.75	143.543	112.412	98.083	124.728	87.050	33.648	14.119	2225.611
-36.65	142.200	112.302	98.830	126.838	84.746	34.238	14.011	2225.611
-36.55	139.582	112.686	99.807	123.882	85.670	34.718	14.110	2225.611
-36.45	142.353	111.956	100.295	126.337	86.453	34.183	14.768	2225.611
-36.35	143.690	111.524	99.681	124.530	86.032	33.528	13.697	2225.611
-36.25	143.260	112.108	100.518	124.018	87.061	35.101	14.444	2225.611
-36.15	143.146	113.111	98.963	126.034	85.027	33.189	14.650	2225.611
-36.05	142.172	113.093	97.916	126.995	84.313	33.265	14.404	2225.611
-35.95	142.302	111.335	98.788	123.078	86.395	33.112	14.080	2225.611
-35.85	143.090	112.546	99.186	125.010	85.646	33.921	14.051	2225.611
-35.75	141.100	113.628	99.018	124.760	84.617	34.358	14.404	2225.611
-35.65	142.444	112.619	100.177	125.773	85.529	33.484	14.218	2225.611
-35.55	144.416	112.296	99.458	125.543	85.822	34.183	13.815	2225.611
-35.45	142.223	113.014	98.349	126.661	85.494	34.751	13.677	2225.611
-35.35	142.007	109.498	100.121	125.553	85.892	33.320	13.785	2225.611
-35.25	141.650	110.964	99.018	124.916	84.863	34.205	14.198	2225.611
-35.15	143.707	111.341	97.867	123.360	84.688	33.713	14.247	2225.611
-35.05	144.467	111.360	97.274	125.146	84.828	34.216	14.178	2225.611
-34.95	144.286	114.547	99.681	125.365	85.997	33.691	13.746	2225.611
-34.85	144.580	112.679	100.337	125.752	83.401	33.189	13.923	2225.611
-34.75	144.461	113.142	99.709	126.807	86.196	33.593	14.680	2225.611
-34.65	143.231	113.026	99.109	123.611	84.500	33.954	13.835	2225.611
-34.55	143.067	113.507	101.251	127.486	85.144	34.402	14.375	2225.611

Midpoint	103	30	10	3	1	0.34	0.1	Volume
-34.45	142.852	112.917	100.560	123.830	86.757	33.790	13.530	2225.611
-34.35	143.413	112.436	100.693	125.543	84.676	33.320	13.982	2225.611
-34.25	143.662	111.086	99.521	124.238	85.413	34.030	14.061	2225.611
-34.15	143.549	113.482	100.239	124.854	83.343	34.019	14.011	2225.611
-34.05	143.379	113.123	98.872	125.125	86.477	34.052	13.894	2225.611
-33.95	143.112	113.568	99.004	126.337	88.359	33.593	14.493	2225.611
-33.85	142.121	114.267	99.786	125.773	84.992	33.801	14.051	2225.611
-33.75	143.724	114.936	100.707	126.723	84.910	34.336	14.198	2225.611
-33.65	142.897	111.883	98.725	124.781	83.717	34.030	13.894	2225.611
-33.55	143.356	113.379	99.318	125.679	84.138	34.161	13.943	2225.611
-33.45	143.736	114.206	98.732	126.452	87.295	33.375	14.090	2225.611
-33.35	143.067	112.759	99.758	127.016	85.939	34.707	14.483	2225.611
-33.25	142.704	113.902	100.121	127.047	85.179	33.997	14.002	2225.611
-33.15	143.107	112.540	100.295	125.104	87.143	34.325	14.621	2225.611
-33.05	143.645	111.834	99.263	124.854	86.594	34.205	13.864	2225.611
-32.95	144.376	113.963	98.160	125.762	86.898	33.877	13.137	2225.611
-32.85	140.840	113.093	98.746	126.504	86.757	33.648	13.992	2225.611
-32.75	143.515	114.583	100.260	124.979	83.822	33.757	14.621	2225.611
-32.65	142.421	111.621	99.186	125.668	86.687	34.139	13.550	2225.611
-32.55	143.458	112.576	99.828	124.133	86.675	33.047	14.129	2225.611
-32.45	144.994	112.235	100.030	124.050	86.699	34.216	14.129	2225.611
-32.35	145.113	111.864	100.979	125.794	85.144	34.030	14.336	2225.611
-32.25	144.733	112.108	100.428	125.595	85.611	33.724	13.854	2225.611
-32.15	142.523	113.555	98.258	124.123	84.419	33.539	14.031	2225.611
-32.05	142.330	113.586	100.428	127.068	85.810	34.412	13.687	2225.611
-31.95	141.809	112.971	101.105	124.958	85.085	33.648	14.680	2225.611
-31.85	143.095	113.610	98.949	124.018	83.857	33.604	14.395	2225.611
-31.75	143.719	113.379	101.279	126.337	86.652	34.489	14.061	2225.611
-31.65	142.914	113.002	100.414	126.890	85.880	35.068	14.041	2225.611
-31.55	142.194	113.367	101.502	123.747	85.167	33.976	14.208	2225.611
-31.45	143.010	112.977	98.851	123.266	85.261	33.025	14.031	2225.611
-31.35	144.762	111.767	99.325	127.935	85.237	34.194	14.021	2225.611
-31.25	143.254	111.797	99.011	126.671	84.793	33.080	14.021	2225.611
-31.15	141.905	113.793	100.288	124.645	85.459	32.927	14.139	2225.611
-31.05	142.330	113.075	98.307	125.533	86.290	33.724	14.021	2225.611
-30.95	143.458	112.302	98.963	124.060	86.266	34.030	14.002	2225.611
-30.85	142.274	113.063	99.563	126.086	86.559	34.227	14.011	2225.611
-30.75	144.252	114.237	100.086	125.689	85.389	34.041	13.785	2225.611
-30.65	144.495	114.072	100.107	125.982	85.073	33.265	14.385	2225.611
-30.55	141.854	112.965	101.139	126.305	85.132	33.713	14.119	2225.611
-30.45	142.676	114.297	102.060	126.347	83.413	32.544	14.169	2225.611
-30.35	143.379	113.914	99.353	126.055	83.085	32.894	14.139	2225.611
-30.25	144.223	113.750	98.014	125.397	85.740	33.287	13.579	2225.611
-30.15	143.362	112.832	99.891	123.976	84.629	33.069	14.395	2225.611
-30.05	143.594	113.148	98.851	126.326	84.383	34.565	13.923	2225.611

Midpoint	103	30	10	3	1	0.34	0.1	Volume
-29.95	142.546	113.336	98.335	125.386	83.764	34.128	13.835	2225.611
-29.85	142.347	111.499	100.958	126.901	84.956	33.724	14.070	2225.611
-29.75	144.150	114.547	100.225	123.611	85.062	33.932	13.648	2225.611
-29.65	144.790	112.278	99.402	124.436	85.658	32.479	13.933	2225.611
-29.55	142.897	112.540	99.486	126.368	87.260	33.626	13.707	2225.611
-29.45	143.492	113.713	99.011	122.922	83.495	33.812	13.746	2225.611
-29.35	143.186	113.330	102.102	127.308	84.255	34.172	13.952	2225.611
-29.25	142.330	113.099	99.584	123.862	82.781	33.145	13.736	2225.611
-29.15	142.710	111.913	100.609	124.624	85.798	33.375	13.717	2225.611
-29.05	142.268	111.518	98.586	124.582	82.968	34.609	13.530	2225.611
-28.95	142.421	113.914	99.981	124.039	83.857	33.462	14.011	2225.611
-28.85	142.818	112.613	99.681	126.013	84.255	32.905	14.090	2225.611
-28.75	141.985	112.047	99.011	123.308	83.881	33.910	13.618	2225.611
-28.65	141.100	111.299	98.997	123.131	84.594	34.445	13.825	2225.611
-28.55	141.633	112.880	100.114	122.420	83.810	33.626	13.658	2225.611
-28.45	143.158	113.434	99.946	124.833	82.337	33.866	14.287	2225.611
-28.35	142.058	112.053	98.676	121.303	84.056	32.621	13.726	2225.611
-28.25	140.602	112.023	97.686	123.862	82.922	33.833	13.225	2225.611
-28.15	140.160	111.870	100.435	121.470	86.021	32.763	13.284	2225.611
-28.05	139.565	111.493	100.316	122.201	83.413	33.866	13.579	2225.611
-27.95	143.356	112.619	99.695	120.102	83.471	33.877	14.011	2225.611
-27.85	140.755	111.998	99.563	123.026	82.664	32.523	14.080	2225.611
-27.75	140.619	111.682	98.704	123.413	83.822	33.986	13.589	2225.611
-27.65	141.588	112.667	99.946	120.948	81.893	33.058	13.373	2225.611
-27.55	142.019	111.968	97.790	122.901	82.816	32.501	13.579	2225.611
-27.45	140.358	113.282	98.481	122.452	82.676	32.872	13.383	2225.611
-27.35	140.148	111.414	99.807	120.217	85.155	31.944	13.648	2225.611
-27.25	139.735	112.765	98.739	120.008	82.664	33.768	12.773	2225.611
-27.15	140.862	111.998	98.851	122.911	81.869	33.287	13.717	2225.611
-27.05	139.043	110.003	99.514	124.154	82.138	32.927	13.284	2225.611
-26.95	140.137	112.138	98.042	120.875	82.314	32.796	14.100	2225.611
-26.85	140.092	111.956	97.400	122.159	83.471	32.621	13.933	2225.611
-26.75	140.936	112.138	100.037	123.371	82.162	31.998	13.343	2225.611
-26.65	141.877	113.391	100.197	119.674	82.243	33.331	13.776	2225.611
-26.55	140.454	110.520	98.383	122.849	81.975	32.621	13.726	2225.611
-26.45	139.854	112.716	99.402	121.856	82.548	32.938	13.186	2225.611
-26.35	141.146	111.834	100.072	122.587	82.512	33.397	13.815	2225.611
-26.25	141.526	112.217	100.484	122.755	83.436	32.238	13.471	2225.611
-26.15	141.163	111.585	98.572	122.890	83.553	33.320	13.304	2225.611
-26.05	140.670	113.422	99.967	121.219	83.331	32.697	13.638	2225.611
-25.95	142.585	111.439	99.737	121.303	83.015	32.381	13.206	2225.611
-25.85	141.735	112.594	99.856	122.462	83.191	31.911	14.119	2225.611
-25.75	140.398	112.698	97.567	123.183	81.928	32.228	14.119	2225.611
-25.65	143.073	112.965	99.074	122.107	84.033	34.063	13.343	2225.611
-25.55	139.321	111.524	99.744	121.522	84.056	32.086	13.726	2225.611

Midpoint	103	30	10	3	1	0.34	0.1	Volume
-25.45	141.259	112.004	99.514	122.086	82.185	33.571	13.363	2225.611
-25.35	142.109	112.442	100.916	122.462	84.383	33.517	13.225	2225.611
-25.25	141.293	113.634	100.400	123.308	81.706	33.801	13.461	2225.611
-25.15	141.622	113.166	100.191	122.838	82.080	33.254	14.110	2225.611
-25.05	141.888	113.075	99.165	122.963	81.635	32.512	13.569	2225.611
-24.95	142.648	111.512	98.990	122.389	82.676	33.156	13.550	2225.611
-24.85	143.146	114.291	99.611	122.556	83.775	32.883	13.225	2225.611
-24.75	141.180	113.878	100.204	123.214	83.460	32.391	14.503	2225.611
-24.65	144.801	113.136	100.588	122.681	83.892	34.150	13.903	2225.611
-24.55	143.543	114.735	100.756	122.807	82.992	32.850	12.999	2225.611
-24.45	144.773	112.375	99.793	122.567	83.448	33.036	13.383	2225.611
-24.35	143.396	113.653	100.707	124.384	83.682	33.593	13.903	2225.611
-24.25	142.682	113.963	100.295	125.136	83.389	33.091	13.736	2225.611
-24.15	141.837	114.145	100.358	124.227	84.395	33.342	14.169	2225.611
-24.05	143.067	114.431	101.160	124.029	83.191	32.970	13.078	2225.611
-23.95	144.677	113.914	100.658	122.441	84.196	34.609	13.668	2225.611
-23.85	144.558	114.717	100.246	124.238	83.600	33.954	13.500	2225.611
-23.75	142.121	115.404	100.595	120.885	84.711	34.740	14.090	2225.611
-23.65	143.435	114.595	101.984	123.496	83.916	33.637	14.355	2225.611
-23.55	142.948	115.094	101.314	122.943	82.524	33.528	13.520	2225.611
-23.45	144.257	117.144	102.305	126.880	85.272	32.927	13.785	2225.611
-23.35	146.252	114.261	101.084	126.608	84.337	33.943	13.844	2225.611
-23.25	146.348	114.267	101.914	125.188	85.436	33.877	13.579	2225.611
-23.15	146.666	117.065	100.288	124.279	85.494	34.718	14.267	2225.611
-23.05	146.144	113.525	102.305	126.379	85.307	34.260	13.363	2225.611
-22.95	144.671	116.469	103.595	127.089	85.272	33.626	13.835	2225.611
-22.85	144.711	116.268	103.805	125.021	85.413	33.331	14.493	2225.611
-22.75	147.244	116.140	102.528	123.841	86.278	33.833	14.247	2225.611
-22.65	147.471	116.870	103.163	125.595	86.044	34.598	13.294	2225.611
-22.55	147.357	116.615	101.586	125.324	84.571	33.429	14.385	2225.611
-22.45	148.831	118.038	103.009	126.650	84.933	33.823	14.218	2225.611
-22.35	147.794	117.320	102.263	127.569	84.968	34.161	14.228	2225.611
-22.25	146.025	117.996	103.902	128.728	85.179	34.270	14.316	2225.611
-22.15	148.723	117.996	105.019	128.613	86.523	34.576	14.110	2225.611
-22.05	148.377	118.294	104.321	129.710	84.559	33.615	13.952	2225.611
-21.95	149.307	117.685	103.609	128.175	86.909	34.598	14.296	2225.611
-21.85	151.177	119.249	106.819	127.914	87.038	34.631	13.746	2225.611
-21.75	149.222	119.541	102.835	128.760	87.997	34.795	14.277	2225.611
-21.65	148.870	119.401	103.791	131.026	88.219	35.134	13.599	2225.611
-21.55	149.029	117.296	103.958	128.624	87.588	35.265	14.070	2225.611
-21.45	150.242	118.348	105.842	130.650	88.090	34.118	14.002	2225.611
-21.35	152.016	119.583	105.751	128.206	87.436	35.024	14.404	2225.611
-21.25	151.596	120.125	104.830	131.245	89.470	33.910	14.483	2225.611
-21.15	151.676	119.340	106.735	132.342	87.810	33.812	14.355	2225.611
-21.05	154.940	120.982	105.926	130.159	87.833	35.396	14.552	2225.611

Midpoint	103	30	10	3	1	0.34	0.1	Volume
-20.95	151.319	121.390	106.358	130.963	88.488	35.581	14.542	2225.611
-20.85	151.047	120.027	105.116	133.261	87.482	34.270	14.817	2225.611
-20.75	153.433	120.502	105.688	133.814	87.494	35.177	13.952	2225.611
-20.65	153.365	122.661	107.768	131.360	89.272	35.516	13.943	2225.611
-20.55	154.844	121.086	106.463	133.073	90.874	35.887	14.552	2225.611
-20.45	155.190	122.405	106.833	130.932	88.558	34.238	14.296	2225.611
-20.35	155.059	123.014	109.414	130.681	89.576	35.144	14.748	2225.611
-20.25	153.688	122.521	108.019	136.457	91.809	36.292	14.817	2225.611
-20.15	155.490	121.536	108.626	131.287	91.178	35.013	14.257	2225.611
-20.05	155.683	121.876	108.096	133.271	91.154	35.254	14.856	2225.611
-19.95	155.507	124.480	108.563	134.357	92.253	36.532	15.190	2225.611
-19.85	156.629	125.939	109.296	136.363	90.839	36.455	14.689	2225.611
-19.75	154.685	124.510	111.417	136.843	90.710	35.658	15.141	2225.611
-19.65	158.907	123.275	111.124	137.814	91.645	35.920	15.623	2225.611
-19.55	158.040	126.682	112.282	138.075	92.674	35.352	14.817	2225.611
-19.45	58.544	47.730	42.379	47.675	33.375	13.787	5.316	2225.611
-19.35	14.582	12.883	11.107	11.237	6.759	2.797	1.110	11710.508
-19.25	13.613	11.453	10.298	10.423	6.607	3.146	1.071	133.076
-19.15	12.491	10.863	9.977	9.483	6.478	2.753	1.032	124.757
-19.05	12.293	10.499	9.377	9.765	5.800	2.840	1.268	118.480
-18.95	11.675	9.501	9.503	8.773	5.929	2.622	0.924	113.296
-18.85	11.550	10.255	9.007	8.459	5.765	2.360	0.943	108.827
-18.75	10.740	9.659	8.407	8.553	5.426	2.196	0.766	104.873
-18.65	10.303	9.367	8.568	8.501	5.765	2.425	0.884	101.312
-18.55	10.802	8.820	8.686	8.167	5.403	2.283	0.845	98.066
-18.45	10.587	9.173	8.317	7.384	5.543	2.196	0.904	95.078
-18.35	10.371	8.978	7.738	7.227	4.642	2.174	0.639	92.307
-18.25	9.963	8.017	7.465	7.530	4.245	2.141	0.816	89.722
-18.15	9.351	8.692	7.528	7.436	4.607	1.977	0.678	87.299
-18.05	9.079	7.749	7.465	6.872	4.502	1.857	0.786	85.018
-17.95	8.830	8.327	7.458	7.091	4.549	1.934	0.757	82.863
-17.85	9.130	8.236	7.277	7.060	4.444	1.966	0.678	80.822
-17.75	8.677	8.382	7.124	7.279	4.607	2.327	0.717	78.882
-17.65	8.830	8.047	7.172	6.141	4.222	2.065	0.884	77.036
-17.55	8.954	7.190	7.068	5.942	4.432	1.803	0.874	75.274
-17.45	8.099	7.013	6.649	6.350	4.116	1.682	0.757	73.590
-17.35	7.940	7.615	6.747	5.420	4.011	1.868	0.717	71.978
-17.25	8.495	7.950	6.579	5.734	3.894	1.846	0.570	70.433
-17.15	8.269	6.879	6.461	5.316	3.520	1.759	0.698	68.949
-17.05	7.917	7.287	6.091	5.848	3.660	1.606	0.599	67.523
-16.95	7.810	6.308	5.930	5.681	4.140	1.464	0.648	66.151
-16.85	8.206	7.457	6.328	6.548	3.625	1.508	0.639	64.829
-16.75	7.702	7.238	6.886	5.807	3.730	1.661	0.491	63.555
-16.65	7.577	6.983	6.077	5.640	3.403	1.562	0.599	62.325
-16.55	7.685	7.250	6.056	5.619	3.461	1.409	0.462	61.138

Midpoint	103	30	10	3	1	0.34	0.1	Volume
-16.45	7.407	7.244	6.084	5.086	3.403	1.322	0.540	59.990
-16.35	7.152	6.922	5.512	5.253	4.011	1.584	0.481	58.881
-16.25	7.509	6.429	5.505	5.138	3.508	1.311	0.580	57.807
-16.15	7.413	6.654	5.644	5.316	3.356	1.737	0.668	56.768
-16.05	6.869	6.770	6.028	5.086	3.286	1.420	0.717	55.760
-15.95	7.198	6.703	5.721	4.595	3.087	1.682	0.599	54.784
-15.85	7.271	6.222	5.512	5.598	2.713	1.486	0.491	53.838
-15.75	6.540	6.034	5.735	4.804	3.403	1.650	0.540	52.920
-15.65	6.478	5.833	6.049	6.110	2.947	1.748	0.423	52.028
-15.55	6.602	6.089	6.168	4.700	3.134	1.573	0.570	51.163
-15.45	10.439	9.379	8.728	7.133	4.678	2.349	0.816	50.323
-15.35	11.074	10.377	8.359	8.637	5.695	2.229	0.717	49.506
-15.25	11.459	10.590	9.077	9.128	5.332	2.021	0.442	48.712
-15.15	11.136	10.359	9.503	8.877	5.484	2.349	0.531	47.940
-15.05	11.669	10.353	9.510	9.243	6.163	1.912	0.570	47.190
-14.95	11.561	10.432	9.614	9.337	5.941	1.824	0.570	46.460
-14.85	11.431	10.949	9.531	9.733	6.151	2.261	0.570	45.749
-14.75	11.782	10.827	10.033	9.117	5.964	1.977	0.629	45.058
-14.65	11.816	10.936	9.817	9.159	5.660	2.261	0.609	44.385
-14.55	11.822	10.426	10.054	9.493	5.976	2.414	0.580	43.729
-14.45	12.032	11.666	9.935	9.243	6.022	2.261	0.599	43.091
-14.35	12.310	11.575	10.180	9.013	5.964	2.174	0.776	42.469
-14.25	12.576	11.356	10.166	10.130	6.443	2.185	0.796	41.864
-14.15	12.769	11.289	10.263	9.660	5.788	2.250	0.521	41.273
-14.05	12.604	11.970	10.912	9.504	5.613	2.327	0.629	40.698
-13.95	13.290	12.123	10.354	9.796	6.046	2.414	0.550	40.137
-13.85	13.165	12.262	11.114	10.360	6.221	2.480	0.766	39.591
-13.75	13.267	13.005	11.226	11.018	6.128	2.338	0.639	39.058
-13.65	13.307	13.230	11.826	9.984	6.174	2.403	0.560	38.538
-13.55	14.282	13.230	12.154	10.987	6.537	2.545	0.599	38.031
-13.45	14.299	13.157	12.063	10.934	6.245	2.764	0.688	37.537
-13.35	14.372	13.552	12.091	11.216	6.584	2.720	0.766	37.055
-13.25	14.780	14.276	12.817	11.060	6.911	3.004	0.825	36.585
-13.15	15.574	14.318	13.033	11.509	7.344	2.819	0.776	36.126
-13.05	16.282	15.006	13.340	12.000	6.595	2.720	0.816	35.679
-12.95	16.078	15.322	13.940	11.509	7.227	2.971	0.648	35.242
-12.85	17.166	15.748	14.038	12.365	7.531	2.829	0.924	34.816
-12.75	17.359	16.052	14.422	12.762	7.835	2.971	0.865	34.401
-12.65	18.453	16.843	15.203	13.055	7.180	3.223	0.717	33.996
-12.55	18.266	17.743	15.908	13.953	8.314	3.146	1.042	33.600
-12.45	18.997	18.680	16.208	13.869	8.443	3.212	0.973	33.214
-12.35	19.898	18.351	16.682	14.558	8.747	3.452	0.914	32.838
-12.25	20.828	19.884	17.847	15.279	9.379	3.714	0.992	32.470
-12.15	21.372	20.139	18.398	15.216	9.367	3.660	1.189	32.112
-12.05	22.488	21.271	19.264	16.522	9.589	4.184	1.100	31.762

Midpoint	103	30	10	3	1	0.34	0.1	Volume
-11.95	22.908	21.988	19.633	16.981	9.507	3.922	1.022	31.421
-11.85	24.460	23.321	20.889	16.856	10.431	4.293	1.189	31.088
-11.75	25.271	23.576	21.978	18.308	10.805	4.479	1.395	30.763
-11.65	26.506	25.504	23.122	19.707	11.367	4.370	1.199	30.446
-11.55	28.360	26.891	23.813	20.699	11.998	4.905	1.356	30.137
-11.45	30.371	29.026	25.494	21.274	12.922	4.599	1.346	29.836
-11.35	31.579	29.488	26.443	22.120	12.559	5.670	1.326	29.542
-11.25	33.318	31.599	29.164	23.530	13.787	5.714	1.395	29.256
-11.15	34.055	32.481	29.332	25.368	14.325	6.227	1.582	28.976
-11.05	36.781	35.036	30.608	25.441	15.424	6.019	1.847	28.704
-10.95	38.918	35.662	33.246	27.112	15.307	6.522	1.778	28.438
-10.85	41.581	37.596	34.334	28.950	17.330	6.588	2.044	28.180
-10.75	44.290	39.969	36.881	28.814	17.038	6.325	1.965	27.927
-10.65	46.144	42.760	37.983	31.237	18.593	7.942	2.093	27.682
-10.55	48.609	45.041	39.365	32.636	18.897	7.451	2.181	27.442
-10.45	51.046	46.848	42.141	34.913	20.277	8.445	2.230	27.209
-10.35	54.662	48.460	44.793	37.148	20.745	8.412	2.555	26.982
-10.25	56.079	50.868	47.772	38.192	22.406	9.122	2.820	26.761
-10.15	58.385	54.342	50.067	40.448	22.429	9.122	2.840	26.546
-10.05	62.800	56.756	51.735	41.252	24.546	10.072	2.840	26.337
-9.95	66.161	60.734	55.293	41.983	24.335	9.985	3.154	26.134
-9.85	69.964	62.754	58.105	44.657	25.060	10.488	2.899	25.936
-9.75	72.865	65.461	60.540	46.318	26.311	10.859	3.164	25.743
-9.65	75.954	70.904	64.294	49.158	27.633	11.613	3.449	25.557
-9.55	80.375	74.986	67.482	52.761	28.884	11.471	4.156	25.375
-9.45	87.317	77.820	69.771	54.526	31.527	12.225	3.930	25.199
-9.35	92.503	82.096	74.334	61.127	33.445	12.978	4.156	25.028
-9.25	97.071	86.920	77.201	61.409	33.328	13.568	4.284	24.861
-9.15	98.890	90.527	80.320	60.176	33.620	14.082	3.861	24.701
-9.05	105.062	91.251	84.715	62.965	36.099	13.929	4.441	24.544
-8.95	111.783	97.497	85.490	65.701	36.473	15.240	4.264	24.393
-8.85	110.094	100.879	87.695	64.218	35.643	15.021	4.225	24.247
-8.75	108.938	100.405	86.202	65.722	34.708	14.289	4.569	24.105
-8.65	106.513	101.810	86.857	66.129	35.012	14.115	4.087	23.969
-8.55	105.419	102.412	86.257	63.320	35.807	13.645	4.078	23.836
-8.45	103.566	101.913	83.962	64.082	33.468	12.388	4.146	23.709
-8.35	102.234	99.018	82.825	57.075	32.065	12.585	3.842	23.586
-8.25	100.556	96.676	80.438	54.735	30.100	12.159	3.861	23.467
-8.15	96.039	93.325	75.073	51.853	27.387	11.657	3.292	23.353
-8.05	94.180	85.156	73.559	50.140	26.124	11.482	3.213	23.243
-7.95	89.057	81.026	70.468	49.294	24.510	10.335	3.331	23.137
-7.85	83.339	76.446	65.124	45.868	22.932	8.991	3.115	23.036
-7.75	78.555	70.521	62.159	40.929	22.055	8.445	3.066	22.939
-7.65	72.197	66.063	60.198	40.929	19.529	8.445	2.849	22.846
-7.55	66.161	61.081	58.838	34.276	18.313	7.309	2.496	22.757

Midpoint	103	30	10	3	1	0.34	0.1	Volume
-7.45	62.619	57.121	54.742	33.942	16.173	6.762	2.250	22.673
-7.35	56.396	56.300	46.935	30.798	14.337	6.588	1.955	22.592
-7.25	52.344	51.385	42.309	29.305	14.115	5.910	1.523	22.516
-7.15	51.953	49.263	37.662	27.394	13.635	5.484	1.690	22.443
-7.05	46.070	44.865	32.332	24.417	10.770	5.091	1.385	22.375
-6.95	40.986	39.500	29.987	21.284	10.150	5.408	0.983	22.310
-6.85	36.345	34.135	27.231	19.551	9.156	4.064	0.845	22.250
-6.75	31.754	30.376	25.271	17.347	7.461	3.157	0.816	22.193
-6.65	28.280	27.262	20.827	14.182	5.800	2.283	0.501	22.140
-6.55	22.919	25.620	18.489	12.574	4.642	2.425	0.491	22.091
-6.45	19.751	21.417	16.326	11.707	3.801	1.628	0.314	22.046
-6.35	18.481	16.545	14.003	10.966	3.064	1.792	0.462	22.005
-6.25	17.535	14.355	11.938	9.128	2.690	1.650	0.255	21.968
-6.15	14.185	13.071	9.921	7.446	2.631	1.573	0.403	21.934
-6.05	12.820	10.322	8.414	6.141	1.906	1.442	0.334	21.904
-5.95	10.349	9.568	6.419	4.574	2.432	1.005	0.334	21.878
-5.85	8.218	8.893	4.884	3.321	1.742	0.787	0.197	21.856
-5.75	7.458	7.044	3.607	2.684	0.994	0.557	0.157	21.837
-5.65	6.676	5.906	2.763	2.141	1.158	0.470	0.138	21.822
-5.55	5.412	5.006	2.623	1.744	1.321	0.393	0.285	21.811
-5.45	4.251	3.735	2.170	1.285	0.877	0.240	0.069	21.804
-5.35	2.856	2.238	1.647	1.023	0.433	0.164	0.029	21.800
-5.25	2.556	1.843	1.723	0.721	0.316	0.131	0.088	21.799
-5.15	2.522	1.648	1.395	0.835	0.339	0.186	0.029	21.799
-5.05	2.199	1.612	1.361	0.825	0.444	0.098	0.049	21.799
-4.95	2.131	1.545	1.284	0.564	0.257	0.142	0.039	21.799
-4.85	2.091	1.454	1.200	0.689	0.327	0.098	0.029	21.799
-4.75	1.751	1.296	0.935	0.501	0.199	0.218	0.049	21.799
-4.65	1.530	1.253	1.054	0.668	0.409	0.175	0.020	21.799
-4.55	1.553	1.472	1.054	0.668	0.292	0.120	0.039	21.799
-4.45	1.502	1.411	0.802	0.585	0.269	0.098	0.010	21.799
-4.35	1.360	1.435	0.914	0.616	0.304	0.066	0.000	21.799
-4.25	1.570	1.350	0.823	0.648	0.374	0.109	0.020	21.799
-4.15	1.621	1.058	0.795	0.658	0.246	0.087	0.020	21.799
-4.05	1.581	1.253	0.795	0.543	0.175	0.087	0.039	21.799
-3.95	1.474	1.192	0.754	0.554	0.175	0.109	0.010	21.799
-3.85	1.190	1.217	0.844	0.721	0.210	0.142	0.029	21.799
-3.75	1.218	1.223	0.907	0.595	0.175	0.098	0.049	21.799
-3.65	1.167	0.973	0.886	0.470	0.292	0.087	0.039	21.799
-3.55	1.230	1.089	0.900	0.491	0.210	0.131	0.020	21.799
-3.45	1.485	1.241	0.900	0.533	0.292	0.098	0.020	21.799
-3.35	1.559	1.174	0.921	0.574	0.363	0.022	0.029	21.799
-3.25	1.530	1.381	0.914	0.574	0.316	0.142	0.029	21.799
-3.15	1.899	1.448	1.256	0.752	0.246	0.164	0.039	21.799
-3.05	1.632	1.484	1.074	0.846	0.433	0.142	0.020	21.799

Midpoint	103	30	10	3	1	0.34	0.1	Volume
-2.95	2.029	1.472	1.158	0.710	0.374	0.142	0.029	21.799
-2.85	1.893	1.594	1.207	0.721	0.246	0.120	0.059	21.799
-2.75	2.352	1.636	1.416	0.762	0.526	0.066	0.108	21.799
-2.65	2.171	1.679	1.542	0.804	0.433	0.087	0.049	21.799
-2.55	2.380	1.873	1.361	0.794	0.526	0.164	0.039	21.799
-2.45	2.278	1.685	1.514	0.950	0.620	0.240	0.039	21.799
-2.35	2.358	2.342	1.758	0.940	0.772	0.164	0.039	21.799
-2.25	3.015	2.165	1.898	1.170	0.678	0.142	0.039	21.799
-2.15	2.856	2.628	2.337	1.138	0.783	0.317	0.049	21.799
-2.05	3.242	2.634	2.205	1.379	1.169	0.229	0.079	21.799
-1.95	3.769	3.230	2.323	1.347	1.310	0.306	0.059	21.799
-1.85	3.995	3.145	2.672	1.326	1.345	0.361	0.147	21.799
-1.75	4.511	3.467	2.833	1.765	1.099	0.328	0.147	21.799
-1.65	4.375	3.753	2.923	1.995	0.947	0.251	0.108	21.799
-1.55	4.404	4.051	3.105	1.859	1.134	0.273	0.128	21.799
-1.45	5.305	3.941	3.412	2.256	1.099	0.404	0.177	21.799
-1.35	4.953	4.750	3.509	2.433	1.204	0.328	0.246	21.799
-1.25	5.446	4.799	3.802	2.527	1.427	0.437	0.265	21.799
-1.15	6.194	5.140	3.991	2.788	1.076	0.404	0.246	21.799
-1.05	6.665	5.864	4.591	2.935	1.356	0.546	0.206	21.799
-0.95	7.169	5.645	4.493	3.300	1.356	0.743	0.236	21.799
-0.85	7.895	6.253	5.547	3.415	1.380	0.546	0.236	21.799
-0.75	7.810	6.898	5.847	3.133	1.731	0.754	0.187	21.799
-0.65	9.192	6.831	5.958	3.415	2.046	0.808	0.138	21.799
-0.55	9.187	7.409	6.210	3.728	2.000	0.568	0.206	21.799
-0.45	9.385	8.047	6.810	4.261	2.093	0.830	0.197	21.799
-0.35	9.436	7.992	7.096	4.418	1.894	0.863	0.167	21.799
-0.25	10.609	8.607	7.417	4.804	2.608	0.885	0.197	21.799
-0.05	10.978	8.874	8.198	4.439	2.257	0.885	0.187	21.799
0.05	11.442	9.671	7.877	5.117	2.479	0.907	0.354	21.799
0.25	11.805	10.456	8.191	5.065	2.807	0.972	0.314	21.799
0.35	11.595	9.683	8.596	5.504	2.374	0.961	0.216	21.799
0.45	12.077	9.665	8.470	5.243	2.865	0.950	0.226	21.799
0.55	12.508	10.292	8.596	5.671	2.970	1.038	0.403	21.799
0.65	12.219	10.547	9.147	5.128	2.771	0.983	0.334	21.799
0.75	12.735	10.541	8.603	5.922	2.655	0.896	0.413	21.799
0.85	12.684	9.793	8.833	5.598	2.947	0.896	0.285	21.799
0.95	12.361	9.282	9.014	5.692	2.783	0.994	0.255	21.799
1.05	11.301	9.100	8.233	5.305	2.771	0.841	0.354	21.799
1.15	10.768	8.941	7.710	5.243	2.538	0.874	0.265	21.799
1.25	10.173	8.966	7.521	4.825	2.526	0.797	0.285	21.799
1.35	9.890	8.418	6.914	4.647	2.538	0.754	0.206	21.799
1.45	9.215	7.828	6.586	4.376	2.140	0.808	0.285	21.799
1.55	8.779	7.275	5.798	4.125	2.070	0.666	0.255	21.799
1.65	8.048	6.770	6.161	3.906	1.871	0.655	0.246	21.799

Midpoint	103	30	10	3	1	0.34	0.1	Volume
1.75	7.203	6.989	5.212	3.822	1.789	0.655	0.197	21.799
1.85	7.107	6.076	4.905	3.290	1.462	0.513	0.206	21.799
1.95	6.302	5.772	5.072	2.924	1.509	0.623	0.118	21.799
2.05	6.217	5.286	4.591	2.924	1.637	0.503	0.147	21.799
2.15	5.764	5.280	4.207	2.935	1.438	0.371	0.285	21.799
2.25	5.469	4.939	4.186	2.694	1.403	0.492	0.177	21.799
2.35	5.072	4.775	3.607	2.694	1.099	0.546	0.157	21.799
2.45	5.027	4.471	3.719	2.517	1.240	0.382	0.118	21.799
2.55	5.293	4.483	3.279	2.350	1.181	0.437	0.157	21.799
2.65	5.038	4.118	3.377	2.141	1.310	0.459	0.088	21.799
2.75	4.500	4.319	3.440	2.621	1.275	0.317	0.187	21.799
2.85	5.095	3.966	3.321	2.402	1.017	0.306	0.157	21.799
2.95	4.358	4.221	3.496	2.235	1.286	0.437	0.147	21.799
3.05	4.613	4.142	3.293	2.245	1.169	0.437	0.187	21.799
3.15	4.670	4.118	3.335	2.151	0.912	0.306	0.079	21.799
3.25	4.880	4.167	3.461	2.266	1.181	0.339	0.167	21.799
3.35	4.851	4.258	3.391	2.120	1.392	0.459	0.187	21.799
3.45	5.118	4.294	3.607	2.486	1.251	0.437	0.088	21.799
3.55	5.027	4.544	3.502	2.475	1.438	0.404	0.079	21.799
3.65	4.914	4.556	3.607	2.580	1.485	0.623	0.187	21.799
3.75	5.713	4.623	3.830	3.070	1.321	0.557	0.167	21.799
3.85	5.571	5.030	4.089	2.747	1.403	0.415	0.177	21.799
3.95	5.900	5.164	4.375	3.039	1.684	0.590	0.118	21.799
4.05	6.109	5.316	4.772	2.914	1.661	0.590	0.177	21.799
4.15	6.546	5.511	4.612	3.091	1.661	0.765	0.236	21.799
4.25	6.353	6.022	4.933	3.384	1.836	0.655	0.118	21.799
4.35	7.243	6.186	5.142	3.937	2.339	0.743	0.236	21.799
4.45	7.555	6.563	5.686	4.313	1.836	0.743	0.177	21.799
4.55	7.657	7.378	5.700	4.177	2.058	0.830	0.255	21.799
4.65	7.934	7.281	6.258	4.386	2.070	0.819	0.187	21.799
4.75	7.997	7.676	6.307	4.261	1.906	0.743	0.265	21.799
4.85	8.813	7.895	6.761	4.804	2.655	0.787	0.147	21.799
4.95	9.362	8.364	6.872	5.065	2.678	0.994	0.324	21.799
5.05	10.139	8.838	7.570	5.452	2.467	0.961	0.295	21.799
5.15	10.388	9.245	7.675	5.608	2.549	1.103	0.265	21.799
5.25	10.609	9.763	7.954	6.036	3.403	1.038	0.373	21.799
5.35	10.813	10.158	8.449	5.974	2.888	1.092	0.354	21.800
5.45	20.272	18.351	13.801	12.313	6.245	2.611	0.757	21.804
5.55	23.950	21.830	17.694	14.506	7.730	2.622	0.884	21.811
5.65	26.648	23.120	19.340	14.694	7.999	2.458	0.963	21.822
5.75	28.416	24.914	21.217	15.603	8.385	3.288	1.120	21.837
5.85	28.802	25.608	20.387	16.386	8.794	3.288	1.042	21.856
5.95	31.669	26.660	21.636	17.316	8.326	3.256	1.159	21.878
6.05	30.672	27.177	22.941	17.232	9.273	3.889	1.002	21.904
6.15	32.627	28.570	23.338	17.514	9.893	3.266	0.845	21.934

Midpoint	103	30	10	3	1	0.34	0.1	Volume
6.25	33.795	29.263	24.852	18.537	10.045	4.687	1.071	21.968
6.35	33.420	30.887	25.745	20.344	11.039	4.184	1.022	22.005
6.45	35.398	32.834	26.973	21.221	11.612	4.195	1.268	22.046
6.55	37.512	33.880	29.276	21.274	12.068	4.785	1.307	22.091
6.65	38.498	35.467	29.611	23.195	12.431	4.654	1.415	22.140
6.75	41.105	36.672	30.783	22.475	12.606	5.342	1.641	22.193
6.85	41.343	36.891	32.520	22.997	12.746	4.654	1.552	22.250
6.95	42.596	38.308	32.660	25.848	13.822	5.528	1.395	22.310
7.05	43.780	39.944	35.436	26.903	14.150	5.626	1.454	22.375
7.15	45.605	41.878	35.506	27.561	14.840	6.314	1.739	22.443
7.25	47.198	44.007	36.204	29.430	16.839	6.402	1.611	22.516
7.35	49.170	45.443	38.499	28.323	15.962	7.123	2.004	22.592
7.45	51.941	45.899	40.348	31.133	16.231	6.872	1.985	22.673
7.55	51.822	47.669	42.051	31.133	17.436	7.844	2.004	22.757
7.65	53.404	48.600	43.404	32.501	18.664	7.265	2.073	22.846
7.75	55.024	50.309	44.374	34.307	19.389	7.560	2.407	22.939
7.85	56.101	52.334	44.535	33.461	19.353	7.789	2.230	23.036
7.95	56.305	51.690	47.053	33.911	19.868	8.019	2.211	23.137
8.05	57.320	53.417	46.739	36.469	21.295	8.379	2.339	23.243
8.15	59.196	53.314	48.337	36.981	21.493	8.914	2.545	23.353
8.25	57.904	54.524	50.102	37.399	20.698	9.319	2.604	23.467
8.35	58.918	54.159	49.070	36.636	21.622	9.351	2.407	23.586
8.45	57.955	53.952	47.842	37.200	22.230	9.056	2.692	23.709
8.55	57.682	53.320	47.486	38.516	20.815	9.067	2.535	23.836
8.65	56.022	53.241	47.214	36.302	21.318	8.914	2.515	23.969
8.75	55.206	52.067	47.563	37.388	20.956	8.904	2.820	24.105
8.85	53.948	49.920	45.211	34.537	20.733	8.587	2.506	24.247
8.95	50.927	47.675	42.916	33.347	19.295	8.576	2.437	24.393
9.05	50.462	46.270	41.674	32.271	19.997	7.942	2.329	24.544
9.15	46.943	45.254	40.683	31.289	18.418	7.243	2.211	24.701
9.25	44.727	43.557	38.716	30.663	18.032	8.019	2.191	24.861
9.35	43.191	40.212	36.818	29.785	17.073	6.588	1.887	25.028
9.45	40.125	37.943	34.543	27.456	17.225	6.500	2.073	25.199
9.55	37.308	35.078	32.129	26.255	15.109	7.352	1.828	25.375
9.65	35.874	33.655	30.560	24.950	14.769	5.954	1.710	25.557
9.75	33.647	32.006	28.173	23.091	13.062	5.812	1.729	25.743
9.85	31.409	29.525	26.750	21.389	12.723	5.801	1.474	25.936
9.95	29.606	27.347	25.459	20.584	12.969	4.610	1.503	26.134
10.05	27.436	26.185	23.492	19.947	11.858	5.025	1.444	26.337
10.15	26.903	25.054	22.899	18.339	11.156	4.468	1.376	26.546
10.25	25.004	23.424	21.706	18.088	10.758	4.064	1.130	26.761
10.35	23.486	22.280	20.045	16.480	10.139	4.064	1.179	26.982
10.45	23.157	20.796	18.880	16.355	9.659	3.911	1.012	27.209
10.55	21.049	20.304	18.761	15.812	9.367	3.878	1.051	27.442
10.65	20.907	19.245	16.912	15.154	8.829	3.911	1.100	27.682

Midpoint	103	30	10	3	1	0.34	0.1	Volume
10.75	19.224	18.357	17.157	13.639	8.806	3.845	1.140	27.927
10.85	18.793	17.518	15.531	13.378	8.279	3.616	1.051	28.180
10.95	17.677	16.624	15.531	12.323	7.695	3.452	1.032	28.438
11.05	16.747	16.082	14.680	13.075	7.893	3.343	0.786	28.704
11.15	16.934	15.815	13.933	12.230	7.344	2.928	0.924	28.976
11.25	15.880	14.738	13.333	11.655	7.239	2.513	0.648	29.256
11.35	15.619	14.355	13.047	11.519	7.145	2.655	0.688	29.542
11.45	14.514	13.369	12.601	11.143	6.888	2.458	0.776	29.836
11.55	13.743	13.272	11.931	11.133	6.747	2.425	0.698	30.137
11.65	13.449	12.427	11.045	10.559	6.490	2.753	0.629	30.446
11.75	13.058	12.153	11.107	9.629	6.046	2.666	0.658	30.763
11.85	12.797	11.727	10.842	9.305	5.917	2.392	0.629	31.088
11.95	12.519	11.593	10.549	9.399	5.215	2.305	0.698	31.421
12.05	11.839	10.815	10.103	9.284	5.648	2.196	0.570	31.762
12.15	12.196	10.407	9.761	9.284	5.122	2.382	0.707	32.112
12.25	11.720	10.803	9.586	8.804	5.414	2.327	0.688	32.470
12.35	11.153	10.304	9.126	8.418	4.982	2.032	0.403	32.838
12.45	11.306	9.805	9.489	7.906	5.321	1.890	0.599	33.214
12.55	10.332	9.483	8.477	8.031	5.169	2.043	0.619	33.600
12.65	10.235	9.726	8.268	8.188	5.356	2.294	0.550	33.996
12.75	9.986	9.464	8.442	7.206	4.888	1.945	0.599	34.401
12.85	10.156	8.716	8.254	7.801	4.713	1.846	0.560	34.816
12.95	9.754	8.814	8.310	7.551	4.876	1.912	0.472	35.242
13.05	9.748	8.582	7.786	7.279	4.935	1.726	0.639	35.679
13.15	9.567	8.297	7.375	6.778	4.198	2.065	0.462	36.126
13.25	9.249	8.607	7.528	7.394	4.619	1.999	0.442	36.585
13.35	8.966	8.345	7.382	7.175	4.292	1.726	0.550	37.055
13.45	9.124	7.786	7.193	7.185	4.771	1.606	0.531	37.537
13.55	9.090	8.084	7.110	6.851	4.467	1.704	0.481	38.031
13.65	8.898	7.889	7.340	6.747	4.327	1.934	0.462	38.538
13.75	8.694	7.609	7.179	6.841	3.941	1.650	0.472	39.058
13.85	8.869	7.427	7.179	7.227	4.537	1.650	0.491	39.591
13.95	8.206	7.439	7.145	7.185	4.385	1.671	0.462	40.137
14.05	8.359	7.682	6.642	6.287	4.467	1.573	0.540	40.698
14.15	8.104	7.767	6.314	6.621	4.011	1.628	0.442	41.273
14.25	8.495	7.482	6.782	6.736	4.409	1.573	0.560	41.864
14.35	7.844	7.092	6.489	6.350	4.584	1.617	0.481	42.469
14.45	8.138	7.031	6.705	6.767	4.315	1.573	0.481	43.091
14.55	8.478	7.573	6.572	6.653	4.140	2.043	0.344	43.729
14.65	7.781	7.445	6.251	6.475	4.315	1.431	0.472	44.385
14.75	8.076	7.256	6.433	6.444	4.818	1.977	0.531	45.058
14.85	8.337	6.776	6.517	6.454	4.105	1.584	0.364	45.749
14.95	7.957	7.068	6.803	6.444	3.894	1.748	0.334	46.460
15.05	8.308	6.922	6.824	6.788	4.222	1.759	0.413	47.190
15.15	8.410	7.056	6.705	6.632	4.128	1.934	0.550	47.940

Midpoint	103	30	10	3	1	0.34	0.1	Volume
15.25	8.529	7.232	6.356	7.029	4.175	1.682	0.383	48.712
15.35	8.002	6.940	6.168	7.175	4.338	1.628	0.698	49.506
15.45	6.965	6.198	6.161	5.535	3.976	1.617	0.540	50.323
15.55	5.333	4.848	4.235	3.989	2.538	1.038	0.432	51.163
15.65	5.254	4.440	4.193	3.488	2.818	1.114	0.452	52.028
15.75	5.299	4.909	4.082	4.052	2.888	1.016	0.442	52.920
15.85	5.356	4.556	4.165	4.167	2.818	1.442	0.344	53.838
15.95	5.435	4.617	4.640	4.418	2.421	1.322	0.324	54.784
16.05	5.401	4.933	4.696	4.397	2.853	1.005	0.383	55.760
16.15	5.395	4.750	4.682	3.948	2.561	1.082	0.364	56.768
16.25	5.650	4.945	4.828	4.439	2.725	1.136	0.413	57.807
16.35	5.843	4.805	4.430	4.616	2.923	1.366	0.511	58.881
16.45	5.594	5.188	4.612	4.898	2.807	1.125	0.570	59.990
16.55	5.866	5.024	4.744	4.710	3.263	1.311	0.531	61.138
16.65	5.866	4.988	4.668	4.459	2.655	1.464	0.481	62.325
16.75	5.741	5.134	5.275	4.376	3.251	1.333	0.550	63.555
16.85	6.302	5.584	5.365	4.627	3.076	1.355	0.462	64.829
16.95	6.387	5.803	4.947	5.880	2.959	1.595	0.639	66.151
17.05	6.223	5.882	5.533	5.076	3.099	1.464	0.550	67.523
17.15	6.563	5.511	5.289	5.096	3.228	1.584	0.580	68.949
17.25	7.198	6.259	5.686	5.076	3.391	1.409	0.560	70.433
17.35	6.699	6.454	5.714	5.222	3.485	1.529	0.540	71.978
17.45	6.909	6.277	5.463	5.608	3.438	1.311	0.531	73.590
17.55	7.521	6.210	5.428	5.065	3.181	1.693	0.668	75.274
17.65	7.209	6.575	6.161	5.378	3.847	1.420	0.678	77.036
17.75	7.464	7.056	6.000	5.587	3.520	1.737	0.717	78.882
17.85	7.288	6.198	6.084	5.922	3.730	1.693	0.619	80.822
17.95	7.481	7.086	6.879	6.339	4.210	1.650	0.698	82.863
18.05	8.246	6.642	6.091	6.433	4.210	1.781	0.727	85.018
18.15	8.342	7.202	6.119	6.256	4.151	1.803	0.737	87.299
18.25	8.614	7.536	6.572	7.143	3.859	2.010	0.688	89.722
18.35	8.971	7.798	7.521	6.423	4.654	1.835	0.835	92.307
18.45	8.841	7.615	7.521	7.290	4.689	1.748	0.874	95.078
18.55	8.733	7.962	7.591	7.446	4.783	1.977	0.757	98.066
18.65	8.654	8.315	7.821	7.363	5.134	2.141	0.855	101.312
18.75	9.680	8.619	7.661	7.989	5.286	2.229	0.933	104.873
18.85	10.099	8.516	7.410	7.864	5.309	2.065	0.786	108.827
18.95	10.439	9.398	8.275	8.470	5.309	2.316	0.786	113.296
19.05	11.323	9.197	8.477	8.898	5.531	2.764	1.081	118.480
19.15	11.964	9.969	9.691	9.681	5.683	2.458	0.953	124.757
19.25	12.480	10.632	9.419	9.921	5.812	2.622	1.081	133.076
19.35	13.233	11.344	9.761	10.872	6.970	2.895	1.130	11710.508
19.45	55.999	45.145	39.804	47.675	32.357	13.186	5.158	2225.611
19.55	157.037	123.081	109.100	135.485	88.652	35.592	14.729	2225.611
19.65	153.314	121.232	107.293	134.462	93.809	36.259	14.994	2225.611

Midpoint	103	30	10	3	1	0.34	0.1	Volume
19.75	157.581	123.665	106.826	132.948	88.640	34.937	14.503	2225.611
19.85	154.549	123.573	106.135	134.514	90.441	34.631	13.825	2225.611
19.95	154.135	121.189	106.854	130.796	89.084	34.412	14.503	2225.611
20.05	154.124	122.393	107.614	131.350	91.014	34.740	14.149	2225.611
20.15	153.892	120.234	107.461	132.948	88.675	35.254	14.955	2225.611
20.25	151.466	120.398	107.314	133.752	88.219	34.478	14.493	2225.611
20.35	150.463	120.879	105.688	131.454	88.652	34.970	14.463	2225.611
20.45	152.492	121.286	105.333	132.634	87.868	34.828	14.571	2225.611
20.55	151.132	120.307	105.263	131.496	86.711	35.505	14.483	2225.611
20.65	150.372	119.206	104.153	132.227	87.319	35.123	14.129	2225.611
20.75	151.103	117.813	104.523	126.963	86.067	34.456	13.894	2225.611
20.85	149.981	119.206	107.300	129.668	89.143	34.926	14.178	2225.611
20.95	150.299	119.571	104.412	131.402	87.634	34.434	14.876	2225.611
21.05	148.763	119.559	105.333	130.399	86.395	35.592	13.756	2225.611
21.15	149.347	118.744	103.428	129.136	86.909	34.981	14.011	2225.611
21.25	149.613	118.598	103.798	127.580	85.997	35.079	14.110	2225.611
21.35	149.313	116.870	103.686	127.141	88.172	34.314	14.011	2225.611
21.45	148.083	117.539	102.388	129.167	86.383	35.527	14.051	2225.611
21.55	148.083	117.168	102.012	128.342	86.032	33.254	13.776	2225.611
21.65	148.978	117.983	103.833	129.407	87.096	34.205	13.825	2225.611
21.75	146.768	118.835	103.442	123.360	86.149	34.292	14.051	2225.611
21.85	149.545	117.150	103.707	130.807	85.915	33.746	14.581	2225.611
21.95	148.678	116.870	103.170	127.277	85.997	34.172	13.962	2225.611
22.05	146.144	115.739	103.574	128.144	85.869	33.888	13.815	2225.611
22.15	146.621	116.262	101.760	128.457	86.535	33.156	13.795	2225.611
22.25	148.264	114.827	104.279	127.005	86.570	33.768	13.392	2225.611
22.35	144.671	117.497	101.914	128.770	86.559	34.161	13.874	2225.611
22.45	145.521	116.098	103.623	126.107	84.758	33.091	14.316	2225.611
22.55	144.603	115.185	102.709	128.144	85.448	32.839	13.854	2225.611
22.65	145.328	115.952	101.928	123.423	85.377	34.205	13.687	2225.611
22.75	145.391	114.492	102.402	123.987	85.483	33.364	13.579	2225.611
22.85	145.119	114.979	100.414	125.209	83.647	33.407	14.119	2225.611
22.95	146.246	115.684	102.039	126.222	83.986	33.539	14.493	2225.611
23.05	144.263	114.078	101.425	124.802	85.705	33.615	12.823	2225.611
23.15	144.286	113.300	100.867	124.175	84.711	33.659	13.736	2225.611
23.25	142.925	113.166	101.907	125.407	83.857	34.194	14.119	2225.611
23.35	143.685	113.464	100.644	126.859	83.097	32.828	13.707	2225.611
23.45	144.291	112.917	99.409	124.812	83.600	32.850	13.559	2225.611
23.55	143.985	114.164	99.067	123.517	85.027	33.091	13.481	2225.611
23.65	144.031	112.716	100.595	123.601	85.003	32.916	14.365	2225.611
23.75	146.541	112.448	100.065	125.386	83.541	33.702	14.169	2225.611
23.85	143.707	114.814	99.563	125.240	82.395	34.041	14.119	2225.611
23.95	142.183	114.808	100.679	125.188	84.945	33.702	13.697	2225.611
24.05	143.016	112.321	100.428	123.548	83.577	33.593	13.766	2225.611
24.15	143.515	113.117	100.644	122.295	83.916	33.965	13.373	2225.611

Midpoint	103	30	10	3	1	0.34	0.1	Volume
24.25	142.455	112.272	99.325	122.044	85.038	33.233	13.530	2225.611
24.35	142.064	111.804	100.749	122.911	84.115	31.933	14.051	2225.611
24.45	143.243	113.282	99.067	123.976	82.150	33.670	13.392	2225.611
24.55	142.693	112.722	99.263	123.569	84.278	32.763	13.422	2225.611
24.65	143.622	113.713	99.304	123.266	84.641	32.796	13.609	2225.611
24.75	139.349	113.026	99.528	124.457	83.647	32.173	13.481	2225.611
24.85	139.910	110.982	100.888	121.940	83.050	33.265	13.510	2225.611
24.95	141.871	112.679	99.249	122.410	83.132	33.954	13.304	2225.611
25.05	141.871	110.897	98.949	122.097	82.629	33.244	13.854	2225.611
25.15	142.381	113.160	100.149	120.854	82.162	32.304	13.697	2225.611
25.25	142.155	112.929	100.204	122.243	82.735	33.112	13.304	2225.611
25.35	141.418	111.609	98.656	119.663	82.887	32.337	13.284	2225.611
25.45	141.310	113.038	99.186	122.514	83.039	32.632	14.139	2225.611
25.55	142.478	112.467	98.076	121.094	83.460	33.047	13.559	2225.611
25.65	141.271	112.485	99.395	120.906	81.963	33.320	13.668	2225.611
25.75	139.667	113.604	99.277	119.308	82.466	32.752	13.933	2225.611
25.85	140.545	112.558	98.286	120.969	81.893	32.315	13.569	2225.611
25.95	139.689	110.161	98.579	123.486	84.150	33.604	13.952	2225.611
26.05	142.092	112.698	98.216	120.572	80.700	32.927	13.353	2225.611
26.15	139.780	112.114	96.151	122.306	82.828	32.872	13.402	2225.611
26.25	138.851	111.238	98.453	122.065	82.641	32.479	14.257	2225.611
26.35	140.794	111.408	97.637	120.509	82.816	33.014	13.609	2225.611
26.45	138.964	111.773	98.014	119.789	80.630	33.058	13.314	2225.611
26.55	139.672	110.751	97.937	120.227	82.583	33.976	12.842	2225.611
26.65	139.939	112.053	99.800	119.830	83.273	33.014	13.402	2225.611
26.75	137.983	109.541	96.897	120.635	83.717	31.780	13.766	2225.611
26.85	139.899	111.262	97.728	121.679	82.887	31.965	13.324	2225.611
26.95	141.871	110.313	97.986	122.723	82.208	32.752	13.314	2225.611
27.05	140.743	111.767	97.260	123.663	83.413	33.123	13.481	2225.611
27.15	143.186	110.125	98.683	119.903	83.296	33.670	13.225	2225.611
27.25	139.213	111.305	97.832	122.138	83.144	33.386	13.216	2225.611
27.35	138.822	111.141	98.502	121.898	85.892	32.938	13.658	2225.611
27.45	141.673	112.880	99.870	123.413	83.132	33.112	13.294	2225.611
27.55	140.341	109.425	98.628	120.896	82.641	33.036	13.618	2225.611
27.65	139.933	112.716	98.286	122.849	82.852	32.970	13.451	2225.611
27.75	142.319	111.256	97.574	121.888	84.535	33.779	13.127	2225.611
27.85	141.004	111.214	98.746	123.830	84.208	32.271	13.677	2225.611
27.95	140.471	111.439	98.983	121.825	84.267	33.320	13.471	2225.611
28.05	142.087	111.785	99.088	123.433	82.150	33.254	13.373	2225.611
28.15	141.078	111.731	99.786	122.452	83.612	32.719	13.442	2225.611
28.25	141.934	111.250	100.909	121.355	84.161	33.648	13.304	2225.611
28.35	141.225	111.335	99.681	122.013	84.582	33.528	14.051	2225.611
28.45	141.492	110.709	98.558	122.922	85.331	33.451	14.041	2225.611
28.55	140.777	112.795	99.179	124.060	82.770	34.423	13.805	2225.611
28.65	141.849	111.731	99.751	123.277	85.494	33.200	14.080	2225.611

Midpoint	103	30	10	3	1	0.34	0.1	Volume
28.75	141.078	111.864	98.209	123.903	81.776	32.894	13.874	2225.611
28.85	144.076	112.941	100.811	122.368	86.313	33.233	13.048	2225.611
28.95	141.248	113.117	100.470	122.222	84.571	33.560	13.717	2225.611
29.05	141.588	112.418	98.928	123.559	87.424	32.916	14.365	2225.611
29.15	144.297	111.895	99.451	123.402	85.085	33.364	13.707	2225.611
29.25	141.860	112.430	99.911	123.256	85.073	33.571	13.815	2225.611
29.35	141.594	113.768	98.104	126.117	83.869	33.910	14.503	2225.611
29.45	141.435	112.217	102.019	123.924	85.132	34.347	13.884	2225.611
29.55	142.993	112.308	100.539	125.679	87.108	33.713	13.835	2225.611
29.65	143.498	113.726	99.242	126.963	84.559	33.145	13.333	2225.611
29.75	142.240	110.362	99.297	122.462	86.149	33.910	13.658	2225.611
29.85	144.286	111.676	100.281	125.815	86.301	33.604	13.481	2225.611
29.95	143.815	112.667	100.023	125.115	86.056	33.331	14.827	2225.611
30.05	142.217	113.653	101.056	123.350	85.284	33.954	14.287	2225.611
30.15	142.109	113.732	99.381	126.243	83.869	35.046	13.707	2225.611
30.25	143.605	112.302	100.225	127.381	86.547	34.423	13.844	2225.611
30.35	141.469	112.029	100.511	124.520	85.670	32.381	13.579	2225.611
30.45	144.008	112.497	99.423	124.112	84.711	34.653	14.041	2225.611
30.55	143.951	113.391	99.835	125.146	84.489	33.888	14.326	2225.611
30.65	143.367	112.686	99.353	127.496	85.214	33.134	14.925	2225.611
30.75	143.509	112.637	100.100	123.914	85.658	34.161	13.972	2225.611
30.85	142.092	114.170	98.781	123.653	85.705	33.659	14.188	2225.611
30.95	144.031	113.738	99.095	124.290	86.430	34.194	14.198	2225.611
31.05	142.772	113.276	100.274	127.339	87.178	33.932	14.345	2225.611
31.15	143.554	112.947	100.470	126.044	86.278	33.637	13.835	2225.611
31.25	144.206	111.323	100.309	126.211	86.465	33.539	13.461	2225.611
31.35	143.010	112.661	99.074	122.441	85.553	33.560	13.992	2225.611
31.45	142.217	113.999	99.793	123.653	86.442	33.528	14.532	2225.611
31.55	144.807	113.044	100.770	125.292	86.371	34.303	13.677	2225.611
31.65	143.798	112.977	100.728	123.976	85.108	34.423	13.766	2225.611
31.75	143.866	112.394	99.946	129.689	85.939	32.883	14.110	2225.611
31.85	144.983	113.361	101.300	124.446	86.559	34.380	13.795	2225.611
31.95	142.874	113.939	99.884	126.828	85.331	34.620	13.844	2225.611
32.05	145.294	113.555	99.270	123.872	85.693	33.965	13.766	2225.611
32.15	144.274	114.310	97.407	127.694	85.015	34.183	13.392	2225.611
32.25	143.118	112.156	101.488	126.211	86.348	34.118	14.139	2225.611
32.35	143.509	115.137	100.274	125.606	86.184	33.222	14.680	2225.611
32.45	143.844	111.603	100.058	125.982	86.874	33.615	14.257	2225.611
32.55	143.486	114.054	100.197	127.997	85.155	34.533	14.178	2225.611
32.65	144.541	114.936	101.049	124.979	85.880	34.412	13.913	2225.611
32.75	142.268	114.085	97.825	126.389	86.746	33.615	13.717	2225.611
32.85	141.356	111.439	99.458	124.144	85.775	34.391	14.129	2225.611
32.95	144.195	114.887	99.904	126.556	87.225	33.276	13.982	2225.611
33.05	142.891	112.485	99.102	125.418	82.548	33.681	14.257	2225.611
33.15	142.370	113.653	98.711	124.812	84.804	34.817	13.618	2225.611

Midpoint	103	30	10	3	1	0.34	0.1	Volume
33.25	141.135	112.734	99.193	126.107	84.840	34.270	13.432	2225.611
33.35	141.894	112.387	97.658	125.073	85.377	33.833	14.110	2225.611
33.45	142.245	113.032	100.428	124.843	85.225	33.407	14.444	2225.611
33.55	145.022	111.731	100.602	126.828	86.734	34.150	14.198	2225.611
33.65	142.959	114.352	99.158	125.647	85.518	33.539	14.218	2225.611
33.75	141.582	111.889	99.528	128.029	85.541	33.626	13.687	2225.611
33.85	142.478	111.439	99.423	127.976	84.582	34.085	14.228	2225.611
33.95	143.169	112.716	99.325	122.640	87.553	33.014	14.149	2225.611
34.05	144.155	112.120	99.807	126.264	87.973	34.434	14.513	2225.611
34.15	142.438	110.867	99.521	125.272	85.974	33.932	14.159	2225.611
34.25	143.022	111.901	99.577	126.754	85.529	34.314	14.355	2225.611
34.35	142.081	113.020	99.849	125.679	86.208	34.412	14.817	2225.611
34.45	142.421	110.593	100.086	124.373	84.699	34.718	14.503	2225.611
34.55	143.656	114.164	98.272	124.436	83.588	34.238	13.972	2225.611
34.65	142.047	112.156	99.793	125.188	86.488	32.992	13.825	2225.611
34.75	142.296	113.951	101.293	123.862	83.308	33.462	14.424	2225.611
34.85	141.180	115.003	99.493	125.031	87.096	34.358	13.913	2225.611
34.95	143.798	111.360	98.509	125.345	85.448	34.118	14.247	2225.611
35.05	142.750	113.154	100.909	124.603	87.073	33.473	13.599	2225.611
35.15	142.296	114.036	99.821	127.768	86.032	34.391	13.746	2225.611
35.25	143.549	111.676	98.328	125.721	86.056	34.631	13.825	2225.611
35.35	143.044	113.744	100.030	126.546	84.956	33.997	14.021	2225.611
35.45	144.087	112.418	98.279	127.089	86.582	33.386	14.129	2225.611
35.55	142.585	113.367	99.200	125.553	84.138	34.533	13.137	2225.611
35.65	143.515	111.086	99.507	124.133	86.243	34.325	13.923	2225.611
35.75	143.696	113.683	98.858	124.572	86.523	34.128	14.198	2225.611
35.85	143.787	111.183	100.079	127.266	84.571	34.063	13.530	2225.611
35.95	143.118	112.759	99.577	125.992	87.319	33.615	14.178	2225.611
36.05	143.554	112.296	99.542	126.556	85.728	33.025	14.434	2225.611
36.15	143.775	111.603	98.000	125.292	84.419	33.659	14.296	2225.611
36.25	143.407	111.609	99.695	123.799	85.576	34.423	13.628	2225.611
36.35	142.121	111.256	99.974	124.676	86.184	33.549	14.483	2225.611
36.45	142.574	110.836	99.514	122.504	85.436	33.746	14.090	2225.611
36.55	143.498	112.984	98.977	124.258	87.810	33.265	14.090	2225.611
36.65	143.135	112.424	98.460	128.384	85.144	33.604	14.149	2225.611
36.75	142.291	112.266	98.844	126.138	86.243	34.227	13.402	2225.611
36.85	143.209	113.951	99.695	123.893	85.635	33.582	13.363	2225.611
36.95	142.376	113.367	99.597	122.556	85.436	34.871	14.287	2225.611
37.05	141.741	112.235	100.337	125.689	86.769	34.697	13.658	2225.611
37.15	142.217	111.907	97.483	124.332	86.816	33.648	14.031	2225.611
37.25	141.225	113.263	98.474	124.457	84.734	34.139	13.265	2225.611
37.35	143.203	111.743	99.549	125.084	84.044	33.648	14.611	2225.611
37.45	140.749	111.956	98.997	124.969	85.424	33.691	13.884	2225.611
37.55	141.492	111.512	100.511	124.530	87.085	33.823	14.316	2225.611
37.65	142.160	112.126	99.235	126.003	85.108	33.211	13.579	2225.611

Midpoint	103	30	10	3	1	0.34	0.1	Volume
37.75	143.656	112.807	98.286	126.222	84.559	32.730	14.257	2225.611
37.85	141.191	112.229	99.988	124.248	85.658	34.314	14.630	2225.611
37.95	140.194	111.591	98.495	121.804	85.752	33.189	14.277	2225.611
38.05	141.560	113.288	98.328	124.948	84.582	34.402	13.952	2225.611
38.15	141.985	111.360	98.153	123.851	86.126	33.659	14.090	2225.611
38.25	143.260	111.566	99.346	124.488	84.980	33.593	13.687	2225.611
38.35	142.251	111.280	99.618	124.102	87.038	33.167	14.680	2225.611
38.45	141.922	112.759	98.293	127.339	85.319	34.019	13.422	2225.611
38.55	143.441	113.239	99.542	125.627	83.857	33.910	14.257	2225.611
38.65	141.639	111.816	98.104	125.533	84.886	32.249	13.903	2225.611
38.75	142.370	112.016	99.158	125.084	85.331	33.757	13.864	2225.611
38.85	141.934	111.287	98.788	124.624	82.968	33.615	14.031	2225.611
38.95	142.087	112.242	98.363	124.342	86.021	33.069	14.424	2225.611
39.05	142.041	111.572	98.000	124.822	84.138	32.271	13.952	2225.611
39.15	141.350	112.460	99.563	123.465	82.700	33.943	13.805	2225.611
39.25	143.067	111.384	97.497	125.261	83.787	33.506	13.815	2225.611
39.35	141.305	109.790	96.737	122.755	84.594	32.708	13.962	2225.611
39.45	142.829	111.810	98.586	126.713	84.688	33.812	14.847	2225.611
39.55	141.135	111.956	99.563	123.360	83.062	33.659	14.100	2225.611
39.65	140.794	111.147	97.114	124.697	85.436	34.576	13.825	2225.611
39.75	140.653	113.288	100.009	124.990	85.015	33.244	13.864	2225.611
39.85	142.682	110.654	96.779	125.073	85.319	34.074	13.481	2225.611
39.95	142.886	113.385	98.523	125.595	83.261	33.506	13.668	2225.611
40.05	141.384	112.351	97.456	123.433	84.933	33.353	13.668	2225.611
40.15	139.106	111.122	99.263	122.880	81.589	33.254	14.031	2225.611
40.25	142.829	112.108	96.981	126.807	83.191	32.337	13.677	2225.611
40.35	141.180	113.276	97.239	124.624	84.816	32.533	14.414	2225.611
40.45	141.628	112.546	98.042	126.713	85.483	33.659	14.178	2225.611
40.55	139.888	109.437	98.481	123.475	85.413	33.495	13.766	2225.611
40.65	139.735	112.029	97.560	122.932	85.202	34.631	13.638	2225.611
40.75	142.478	110.934	97.916	123.903	85.459	33.276	13.412	2225.611
40.85	141.509	110.362	98.286	124.478	87.389	33.025	13.903	2225.611
40.95	141.673	110.970	98.670	123.903	83.939	33.779	13.776	2225.611
41.05	142.291	108.093	97.469	123.736	86.067	33.036	13.088	2225.611
41.15	142.665	110.405	98.083	124.290	83.857	33.222	13.540	2225.611
41.25	142.251	112.430	99.060	121.721	85.284	33.724	14.198	2225.611
41.35	140.964	109.760	98.090	124.885	84.337	34.281	14.385	2225.611
41.45	139.593	111.420	96.625	122.922	82.968	33.375	14.454	2225.611
41.55	140.602	110.259	98.139	122.535	86.933	33.626	13.314	2225.611
41.65	142.381	111.694	98.523	124.269	84.021	32.566	13.550	2225.611
41.75	140.726	112.314	98.439	126.441	86.453	34.358	14.404	2225.611
41.85	140.675	112.108	97.016	123.402	84.688	33.025	13.491	2225.611
41.95	142.410	111.572	97.630	123.809	85.249	33.473	14.562	2225.611
42.05	142.155	109.833	98.858	121.648	85.085	33.069	13.726	2225.611
42.15	140.760	110.697	98.537	125.230	85.565	33.025	14.630	2225.611

Midpoint	103	30	10	3	1	0.34	0.1	Volume
42.25	141.917	110.916	98.160	124.164	84.044	33.244	13.294	2225.611
42.35	141.684	110.605	98.342	123.851	84.758	33.069	13.677	2225.611
42.45	139.848	111.822	98.125	124.039	85.038	33.517	13.933	2225.611
42.55	140.619	111.579	99.444	122.097	85.939	33.626	13.677	2225.611
42.65	142.687	112.339	97.588	123.601	84.617	33.844	14.385	2225.611
42.75	142.597	110.630	99.639	122.546	84.290	33.276	14.100	2225.611
42.85	143.141	110.563	98.418	123.632	83.974	34.412	14.119	2225.611
42.95	140.273	111.670	97.832	124.896	85.261	33.615	14.051	2225.611
43.05	141.191	109.462	96.632	123.966	86.395	33.943	13.815	2225.611
43.15	140.511	110.982	98.997	121.313	84.664	33.560	13.206	2225.611
43.25	140.381	110.922	96.911	120.436	85.389	32.763	14.345	2225.611
43.35	141.792	111.658	96.465	123.768	84.571	33.604	13.766	2225.611
43.45	142.772	109.602	97.944	122.493	85.120	33.954	13.687	2225.611
43.55	140.630	110.958	97.714	123.695	84.582	34.194	14.257	2225.611
43.65	141.089	111.007	97.588	122.368	85.834	34.456	13.442	2225.611
43.75	141.083	110.849	100.037	121.950	84.454	32.544	13.815	2225.611
43.85	139.961	111.457	97.651	124.864	83.775	33.954	14.129	2225.611
43.95	141.299	110.611	98.697	124.258	84.395	33.560	14.110	2225.611
44.05	140.772	111.341	97.218	121.376	84.056	33.003	13.982	2225.611
44.15	142.823	109.687	97.456	122.358	81.846	32.730	13.628	2225.611
44.25	139.202	111.828	98.349	125.741	84.594	33.681	14.002	2225.611
44.35	139.893	111.694	97.358	123.486	85.974	33.353	14.247	2225.611
44.45	143.503	108.257	98.209	124.217	84.816	33.943	13.196	2225.611
44.55	142.591	111.244	98.021	124.342	82.255	33.943	14.070	2225.611
44.65	139.162	110.763	97.665	123.193	82.068	33.484	13.933	2225.611
44.75	139.038	112.667	97.986	123.924	84.091	32.293	13.894	2225.611
44.85	142.115	109.553	99.046	125.178	83.635	32.960	13.864	2225.611
44.95	142.342	111.037	99.577	123.078	85.927	33.724	14.404	2225.611
45.05	141.072	109.930	96.953	122.556	83.027	32.938	13.972	2225.611
45.15	141.118	110.916	97.644	125.574	84.138	35.723	13.884	2225.611
45.25	139.009	109.833	97.609	121.585	84.419	33.812	14.031	2225.611
45.35	139.973	110.672	97.093	124.311	85.190	32.435	13.019	2225.611
45.45	141.186	112.333	98.823	122.713	85.681	34.008	14.021	2225.611
45.55	141.259	111.287	96.765	123.287	83.541	33.593	13.274	2225.611
45.65	139.508	111.463	96.625	123.642	83.752	33.331	14.061	2225.611
45.75	140.953	110.709	98.042	123.162	83.904	33.353	13.864	2225.611
45.85	142.557	111.518	96.569	121.428	83.998	33.342	13.815	2225.611
45.95	141.310	109.717	97.804	121.428	87.096	34.871	14.316	2225.611
46.05	142.381	111.487	97.525	122.838	85.377	33.954	14.139	2225.611
46.15	140.964	109.547	99.444	120.290	84.442	33.276	13.618	2225.611
46.25	139.786	109.808	98.942	125.052	84.582	33.233	14.110	2225.611
46.35	138.482	110.800	96.821	122.608	82.945	33.888	14.090	2225.611
46.45	140.715	109.961	97.651	123.580	83.623	33.386	13.756	2225.611
46.55	140.573	111.165	97.462	124.708	85.974	33.965	13.884	2225.611
46.65	139.185	112.102	97.511	127.047	84.980	33.779	13.854	2225.611

Midpoint	103	30	10	3	1	0.34	0.1	Volume
46.75	140.789	109.693	96.821	121.564	84.898	33.287	14.021	2225.611
46.85	141.656	111.323	97.253	122.149	85.834	32.697	13.432	2225.611
46.95	141.157	109.954	98.816	122.577	83.577	33.844	12.980	2225.611
47.05	141.480	109.553	98.704	122.577	84.723	33.604	13.903	2225.611
47.15	141.877	111.797	97.190	122.462	83.998	33.462	13.776	2225.611
47.25	141.860	110.052	98.809	122.901	85.225	32.413	13.864	2225.611
47.35	139.797	110.782	97.093	124.739	82.688	33.233	14.139	2225.611
47.45	139.446	110.654	98.495	123.402	85.190	33.506	14.444	2225.611
47.55	141.129	110.490	96.255	123.444	84.582	32.828	13.844	2225.611
47.65	140.936	110.605	97.107	121.240	84.454	33.943	14.552	2225.611
47.75	141.118	110.757	97.156	123.496	83.436	33.123	13.471	2225.611
47.85	142.342	110.027	97.923	123.882	86.605	33.691	13.894	2225.611
47.95	140.732	110.575	97.979	122.034	84.115	33.320	13.461	2225.611
48.05	139.882	112.527	96.569	124.457	85.237	34.391	14.080	2225.611
48.15	140.664	110.690	97.183	122.514	86.371	33.539	13.510	2225.611
48.25	140.636	112.108	96.465	126.629	84.898	34.336	13.471	2225.611
48.35	139.281	110.021	97.818	124.478	84.372	33.659	13.736	2225.611
48.45	140.341	111.299	98.683	121.836	85.752	34.336	13.609	2225.611
48.55	139.474	110.830	97.267	122.880	85.939	33.921	13.894	2225.611
48.65	141.061	109.170	97.902	124.415	84.535	34.642	14.277	2225.611
48.75	141.373	109.699	96.932	122.086	85.740	32.533	14.807	2225.611
48.85	141.633	111.134	98.809	122.943	82.957	32.512	13.972	2225.611
48.95	140.602	109.517	97.309	124.927	82.980	33.746	13.844	2225.611
49.05	140.381	111.189	97.735	122.389	86.102	33.812	13.500	2225.611
49.15	140.256	110.405	98.502	123.590	82.957	33.102	13.952	2225.611
49.25	141.979	111.700	97.107	124.478	84.816	33.036	14.650	2225.611
49.35	141.815	110.052	98.523	124.248	85.377	33.440	13.844	2225.611
49.45	139.514	110.587	97.393	122.598	85.541	34.697	14.188	2225.611
49.55	139.667	110.155	97.797	122.253	83.588	33.888	13.864	2225.611
49.65	140.681	109.468	96.465	121.125	85.588	33.976	13.933	2225.611
49.75	141.169	108.835	95.725	123.653	85.155	33.691	13.972	2225.611
49.85	140.709	111.536	98.949	124.457	85.623	33.156	13.726	2225.611
49.95	139.967	110.295	98.404	123.517	84.746	34.456	14.198	2225.611
50.05	140.596	110.478	97.407	122.734	84.138	32.402	14.159	2225.611
50.15	140.891	110.088	97.072	124.384	85.401	33.833	13.697	2225.611
50.25	142.155	112.606	96.555	125.867	83.612	34.718	14.444	2225.611
50.35	139.519	109.431	97.714	123.987	84.185	33.418	13.736	2225.611
50.45	139.304	110.575	96.960	125.251	84.571	33.047	13.559	2225.611
50.55	142.602	110.283	96.346	124.008	82.664	34.118	14.149	2225.611
50.65	138.890	110.660	96.535	123.945	84.699	33.954	13.962	2225.611
50.75	139.026	110.715	98.335	122.065	84.419	34.697	13.766	2225.611
50.85	141.486	109.158	97.616	121.658	82.489	33.997	13.245	2225.611
50.95	141.089	111.232	97.156	120.687	83.015	33.582	14.581	2225.611
51.05	140.177	110.398	96.528	125.063	86.021	32.927	14.552	2225.611
51.15	141.560	110.733	98.872	124.499	83.752	33.539	13.707	2225.611

Midpoint	103	30	10	3	1	0.34	0.1	Volume
51.25	140.862	110.703	97.393	126.180	85.331	33.823	13.550	2225.611
51.35	141.696	110.332	97.595	122.953	83.705	33.397	12.744	2225.611
51.45	141.730	108.884	97.442	124.666	85.144	33.615	14.375	2225.611
51.55	141.214	112.126	98.656	124.018	85.319	33.462	14.856	2225.611
51.65	141.803	111.280	99.179	123.172	83.121	33.877	14.345	2225.611
51.75	141.707	110.441	96.716	123.569	82.746	33.123	13.284	2225.611
51.85	140.517	110.666	96.793	124.050	85.190	33.702	14.630	2225.611
51.95	141.492	108.476	97.672	123.413	83.717	34.030	13.982	2225.611
52.05	140.188	109.699	99.074	124.248	82.091	32.719	14.395	2225.611
52.15	139.497	109.948	97.930	123.433	84.056	33.921	14.100	2225.611
52.25	140.732	110.544	97.756	124.540	86.488	33.801	14.316	2225.611
52.35	142.251	110.721	97.581	122.149	86.512	33.429	14.267	2225.611
52.45	141.633	109.626	96.786	123.997	83.846	32.839	14.306	2225.611
52.55	139.973	110.319	97.316	123.945	83.471	34.369	13.894	2225.611
52.65	139.055	110.654	97.462	121.668	84.992	33.386	13.952	2225.611
52.75	141.254	110.739	97.274	125.188	85.518	33.746	14.031	2225.611
52.85	138.312	110.417	97.769	123.225	84.512	33.812	13.795	2225.611
52.95	140.012	110.362	96.416	122.681	85.003	33.222	14.198	2225.611
53.05	138.839	110.192	96.283	124.990	81.483	33.353	14.483	2225.611
53.15	141.843	110.697	96.514	124.332	84.933	31.878	14.051	2225.611
53.25	140.432	110.964	97.386	124.269	86.102	33.254	14.306	2225.611
53.35	140.063	109.541	96.688	122.514	85.494	33.637	13.943	2225.611
53.45	139.264	109.973	97.916	123.089	86.430	33.309	13.726	2225.611
53.55	140.205	111.226	98.663	122.389	85.319	33.681	13.835	2225.611
53.65	140.658	110.909	97.763	122.326	84.945	34.019	13.638	2225.611
53.75	142.393	109.048	97.700	126.786	82.828	32.479	13.078	2225.611
53.85	140.641	110.076	95.223	121.731	83.319	33.746	13.500	2225.611
53.95	139.797	110.709	97.686	124.018	84.921	33.626	14.159	2225.611
54.05	139.655	108.665	96.890	122.817	82.512	33.702	13.952	2225.611
54.15	141.599	111.773	97.211	124.300	85.155	32.883	14.218	2225.611
54.25	141.390	110.076	96.890	122.713	83.097	32.774	14.080	2225.611
54.35	141.752	109.206	97.121	122.702	84.594	33.539	14.149	2225.611
54.45	140.494	110.362	97.442	123.287	85.085	33.866	13.854	2225.611
54.55	139.859	108.914	98.216	124.446	85.810	32.883	14.041	2225.611
54.65	140.035	108.896	97.267	122.295	82.512	32.850	14.306	2225.611
54.75	139.038	108.446	95.439	122.326	85.015	33.364	13.952	2225.611
54.85	140.681	109.997	97.016	122.953	85.354	33.604	13.697	2225.611
54.95	140.908	110.885	96.967	123.256	84.442	33.910	14.424	2225.611
55.05	141.095	110.113	96.514	126.431	84.723	32.905	13.726	2225.611
55.15	140.681	111.147	98.411	124.091	85.202	33.713	13.903	2225.611
55.25	139.854	110.107	96.625	125.835	83.471	31.998	14.011	2225.611
55.35	141.140	110.770	96.862	125.094	83.249	33.211	13.884	2225.611
55.45	141.322	110.435	97.469	122.024	84.851	33.626	13.039	2225.611
55.55	141.718	109.839	95.530	123.517	83.904	32.391	13.589	2225.611
55.65	140.284	109.316	97.672	125.553	85.413	33.265	13.274	2225.611

Midpoint	103	30	10	3	1	0.34	0.1	Volume
55.75	141.384	108.245	96.939	125.971	84.793	34.205	13.697	2225.611
55.85	140.471	109.133	97.351	121.930	84.465	33.539	14.139	2225.611
55.95	143.441	110.471	97.490	125.303	86.746	33.626	13.933	2225.611
56.05	139.151	109.352	96.590	123.068	86.453	33.123	13.658	2225.611
56.15	139.168	110.976	97.679	122.723	85.366	33.549	13.383	2225.611
56.25	139.208	110.198	96.353	123.997	85.179	33.506	14.591	2225.611
56.35	139.480	111.037	97.218	122.922	85.915	32.675	13.599	2225.611
56.45	140.862	108.227	98.411	122.379	83.635	33.254	14.031	2225.611
56.55	140.936	109.066	98.104	123.057	83.085	33.364	14.581	2225.611
56.65	140.239	110.131	96.716	123.935	84.430	32.817	14.208	2225.611
56.75	139.383	109.948	96.660	120.823	85.529	33.123	14.237	2225.611
56.85	141.990	107.984	96.458	121.313	86.687	33.440	14.395	2225.611
56.95	141.180	110.325	95.962	121.888	85.132	32.916	13.795	2225.611
57.05	141.582	109.760	97.476	122.619	84.196	33.036	13.726	2225.611
57.15	141.667	110.800	98.111	123.392	82.887	33.593	13.412	2225.611
57.25	138.227	109.304	97.379	125.042	85.658	33.331	14.169	2225.611
57.35	138.822	111.353	98.467	121.407	85.377	34.260	14.110	2225.611
57.45	140.483	109.638	97.776	124.133	83.214	32.949	12.872	2225.611
57.55	141.673	110.076	97.002	122.692	84.161	33.571	13.864	2225.611
57.65	139.701	111.323	98.007	123.120	83.273	34.150	13.235	2225.611
57.75	141.696	107.941	97.302	123.329	85.108	32.817	13.196	2225.611
57.85	139.633	110.453	96.451	121.209	83.389	32.949	14.434	2225.611
57.95	139.990	111.743	99.039	119.642	85.518	33.102	13.245	2225.611
58.05	141.628	109.024	96.981	123.329	83.635	33.823	14.031	2225.611
58.15	140.477	110.690	96.702	122.775	82.360	32.446	13.972	2225.611
58.25	143.492	109.596	96.583	124.467	84.196	33.495	13.520	2225.611
58.35	138.862	110.064	97.058	121.115	85.190	32.894	14.139	2225.611
58.45	140.443	109.815	97.190	123.507	84.500	33.833	13.776	2225.611
58.55	139.140	110.776	97.588	124.144	85.132	33.112	14.424	2225.611
58.65	138.426	110.034	98.453	123.997	84.512	34.544	13.304	2225.611
58.75	139.230	109.620	95.935	120.509	84.407	33.112	14.061	2225.611
58.85	141.237	110.027	97.400	121.063	84.103	32.905	13.353	2225.611
58.95	140.177	109.298	97.511	123.507	84.699	33.691	13.510	2225.611
59.05	141.843	110.478	97.435	124.781	82.606	34.205	13.943	2225.611
59.15	139.157	109.681	97.295	124.770	84.688	33.801	13.707	2225.611
59.25	140.687	110.569	96.137	123.642	84.535	34.707	13.933	2225.611
59.35	142.041	110.021	97.518	122.191	84.840	33.582	14.041	2225.611
59.45	140.551	110.216	97.518	124.540	84.267	34.085	14.503	2225.611
59.55	140.851	110.526	97.553	123.037	82.770	32.949	14.483	2225.611
59.65	140.194	108.951	98.718	124.405	85.869	32.981	14.395	2225.611
59.75	141.713	109.808	97.637	123.820	83.764	32.785	13.726	2225.611
59.85	139.162	110.733	97.114	122.274	84.535	33.768	13.864	2225.611
59.95	142.132	111.481	97.330	124.488	82.395	33.517	13.569	2225.611
60.05	140.993	111.743	97.093	125.031	84.185	32.621	14.070	2225.611
60.15	138.981	109.985	97.679	122.713	83.799	33.440	14.316	2225.611

Midpoint	103	30	10	3	1	0.34	0.1	Volume
60.25	141.027	109.821	96.137	121.773	84.290	33.823	13.333	2225.611
60.35	139.230	110.770	97.707	122.086	83.366	33.823	14.237	2225.611
60.45	140.199	109.954	97.581	124.405	85.869	32.916	14.051	2225.611
60.55	138.913	108.744	97.225	122.713	85.740	33.265	13.992	2225.611
60.65	139.338	110.873	95.683	123.642	84.968	33.549	13.943	2225.611
60.75	138.641	109.620	97.365	124.258	83.939	34.096	13.333	2225.611
60.85	139.593	110.818	98.446	125.365	82.805	33.167	13.756	2225.611
60.95	142.075	111.183	96.395	125.052	81.507	32.140	13.383	2225.611
61.05	140.987	110.021	97.937	123.026	85.261	33.145	13.697	2225.611
61.15	140.058	109.711	96.353	123.120	85.518	34.172	13.451	2225.611
61.25	140.324	111.031	97.469	123.392	85.108	34.041	13.304	2225.611
61.35	139.134	111.183	96.925	123.611	84.465	32.839	13.500	2225.611
61.45	139.400	108.586	97.749	123.611	83.296	33.080	14.159	2225.611
61.55	142.132	110.751	97.149	122.222	83.834	33.342	14.522	2225.611
61.65	139.463	110.417	97.086	123.736	84.617	33.790	13.392	2225.611
61.75	140.857	110.392	97.218	122.420	85.237	33.724	13.952	2225.611
61.85	140.636	110.715	98.076	124.070	86.407	33.320	14.640	2225.611
61.95	141.015	110.380	97.672	124.426	84.208	33.495	14.041	2225.611
62.05	139.814	109.875	98.544	123.371	84.629	33.571	14.090	2225.611
62.15	140.114	110.210	97.100	122.546	86.488	32.719	13.658	2225.611
62.25	140.177	108.677	97.588	123.475	83.752	33.812	14.463	2225.611
62.35	140.426	111.889	98.460	123.903	84.641	33.495	14.002	2225.611
62.45	142.013	111.840	97.379	122.347	84.383	32.468	13.677	2225.611
62.55	139.009	109.851	97.023	121.292	85.541	34.096	14.070	2225.611
62.65	140.613	110.642	96.276	122.943	84.220	33.244	14.159	2225.611
62.75	141.208	111.147	97.156	121.198	86.032	34.205	13.009	2225.611
62.85	138.754	109.991	97.337	123.590	82.138	32.359	13.894	2225.611
62.95	139.276	110.362	98.697	125.146	84.407	33.866	13.343	2225.611
63.05	140.194	110.417	97.449	123.621	83.647	33.997	13.383	2225.611
63.15	141.645	109.723	97.030	125.658	85.097	33.495	14.424	2225.611
63.25	138.907	111.055	97.232	123.517	86.746	34.555	13.874	2225.611
63.35	142.036	111.609	96.137	124.029	84.220	32.807	14.100	2225.611
63.45	141.996	109.815	95.314	122.462	84.945	33.801	13.353	2225.611
63.55	140.477	111.268	97.909	122.828	84.044	33.112	13.687	2225.611
63.65	139.939	110.855	98.523	123.987	83.378	33.549	13.412	2225.611
63.75	140.035	110.526	97.100	123.308	84.313	33.156	14.375	2225.611
63.85	142.047	110.453	97.825	124.008	83.401	33.921	14.021	2225.611
63.95	141.305	109.298	95.328	122.692	83.799	32.905	13.884	2225.611
64.05	138.477	109.419	98.063	121.366	82.524	33.309	13.894	2225.611
64.15	140.415	109.541	96.395	125.115	84.115	33.156	13.451	2225.611
64.25	140.199	111.536	97.016	124.154	82.489	33.691	13.491	2225.611
64.35	141.135	110.563	97.846	124.478	85.331	31.889	13.933	2225.611
64.45	140.998	109.778	98.600	126.431	85.179	33.495	14.041	2225.611
64.55	140.902	111.347	96.737	121.856	85.693	33.539	14.002	2225.611
64.65	142.937	110.228	97.888	125.815	84.115	32.970	14.129	2225.611

Midpoint	103	30	10	3	1	0.34	0.1	Volume
64.75	139.055	110.368	96.402	123.841	84.360	32.654	13.785	2225.611
64.85	140.420	111.445	95.132	125.533	83.436	34.314	13.736	2225.611
64.95	142.347	109.139	97.267	122.796	85.705	34.303	13.697	2225.611
65.05	139.395	110.557	98.300	124.279	83.916	33.397	14.159	2225.611
65.15	139.780	110.757	97.302	122.838	84.723	32.785	13.697	2225.611
65.25	140.528	110.107	97.644	122.608	83.401	33.681	13.962	2225.611
65.35	140.488	110.064	97.490	123.997	84.407	33.801	14.188	2225.611
65.45	140.347	110.855	96.381	123.914	84.290	33.091	13.107	2225.611
65.55	142.755	109.164	98.635	124.990	84.723	33.298	13.756	2225.611
65.65	140.205	111.183	96.388	122.922	84.535	32.861	14.198	2225.611
65.75	142.483	110.757	97.630	122.567	84.863	32.927	14.228	2225.611
65.85	142.903	109.462	97.693	124.572	83.331	34.314	13.707	2225.611
65.95	141.985	109.821	96.144	123.089	83.518	33.648	14.208	2225.611
66.05	141.220	110.836	98.425	122.295	84.875	33.528	13.550	2225.611
66.15	138.448	111.013	96.946	122.086	85.588	33.746	13.854	2225.611
66.25	140.823	111.232	96.472	122.869	84.512	34.150	13.442	2225.611
66.35	140.573	109.778	97.958	123.768	84.009	33.418	14.306	2225.611
66.45	140.528	110.575	97.442	123.183	83.892	33.670	13.707	2225.611
66.55	139.264	111.414	97.665	122.525	83.518	33.418	13.461	2225.611
66.65	139.140	111.165	98.160	122.817	86.254	32.774	13.127	2225.611
66.75	140.262	110.745	96.304	125.230	85.658	33.091	13.982	2225.611
66.85	141.735	110.563	96.883	125.439	85.307	32.774	14.080	2225.611
66.95	140.256	112.807	98.509	122.974	85.553	34.664	12.823	2225.611
67.05	141.622	110.508	97.058	124.238	86.559	33.429	13.992	2225.611
67.15	140.097	108.714	98.621	121.961	83.050	33.058	14.119	2225.611
67.25	141.072	111.049	98.195	124.457	85.834	32.817	13.451	2225.611
67.35	141.095	110.897	98.432	124.760	84.091	33.899	14.287	2225.611
67.45	142.172	111.433	97.316	123.110	84.699	32.544	13.333	2225.611
67.55	140.925	110.125	96.744	122.556	84.945	33.976	14.169	2225.611
67.65	140.568	109.255	96.911	125.794	84.500	33.648	13.766	2225.611
67.75	140.834	109.760	97.651	121.386	82.723	32.861	13.677	2225.611
67.85	138.732	111.493	97.218	124.175	84.021	34.631	13.756	2225.611
67.95	139.825	110.484	97.616	122.410	83.670	34.172	13.825	2225.611
68.05	140.692	108.604	96.569	124.697	86.792	33.691	13.962	2225.611
68.15	141.180	110.630	97.037	122.149	84.641	33.429	14.080	2225.611
68.25	142.228	111.506	96.779	122.755	81.904	34.074	13.589	2225.611
68.35	139.400	110.751	96.932	121.794	82.711	32.817	13.874	2225.611
68.45	140.029	110.715	95.683	123.882	85.261	34.467	13.913	2225.611
68.55	139.961	109.808	96.102	125.031	84.325	34.063	13.687	2225.611
68.65	138.403	110.027	97.232	122.337	85.331	33.965	13.638	2225.611
68.75	138.975	111.293	97.351	125.251	84.664	34.063	14.041	2225.611
68.85	140.103	109.991	96.883	123.799	83.869	33.276	13.638	2225.611
68.95	142.517	110.338	97.016	122.326	84.208	32.686	13.707	2225.611
69.05	140.256	111.280	98.397	122.159	84.816	33.014	13.412	2225.611
69.15	141.503	110.654	98.816	125.376	84.664	33.484	13.884	2225.611

Midpoint	103	30	10	3	1	0.34	0.1	Volume
69.25	139.191	110.070	98.069	124.311	82.501	33.757	13.668	2225.611
69.35	139.570	110.849	96.158	125.052	84.676	33.528	13.903	2225.611
69.45	141.429	111.639	97.965	122.577	83.366	33.932	14.218	2225.611
69.55	139.021	111.469	96.500	125.282	83.273	33.123	13.628	2225.611
69.65	141.985	109.225	96.151	123.277	85.436	33.757	14.237	2225.611
69.75	142.512	110.143	97.023	122.097	85.296	33.418	14.218	2225.611
69.85	139.905	109.188	98.676	122.640	82.875	32.796	14.316	2225.611
69.95	142.563	111.031	97.539	122.681	85.892	32.719	13.805	2225.611
70.05	139.077	109.182	97.609	124.822	86.219	32.577	13.884	2225.611
70.15	142.013	109.158	98.390	124.551	84.711	33.976	13.717	2225.611
70.25	141.248	109.194	98.167	124.520	87.108	34.216	13.903	2225.611
70.35	139.491	110.009	98.216	124.749	85.120	33.397	14.621	2225.611
70.45	141.429	109.699	98.014	122.681	84.278	33.932	13.510	2225.611
70.55	141.327	110.703	97.100	122.065	83.425	32.435	13.756	2225.611
70.65	138.936	109.656	98.711	122.901	85.342	33.844	13.903	2225.611
70.75	139.888	110.465	96.339	121.982	82.863	33.429	14.169	2225.611
70.85	141.305	111.439	97.225	122.034	84.138	33.549	13.677	2225.611
70.95	141.628	110.995	97.846	125.480	85.646	33.036	14.434	2225.611
71.05	140.568	110.362	98.670	124.112	84.231	33.200	14.375	2225.611
71.15	141.350	112.126	98.000	123.621	85.097	32.533	13.864	2225.611
71.25	140.228	109.577	98.530	122.441	84.699	33.451	13.609	2225.611
71.35	141.582	110.563	97.714	123.548	84.138	33.506	13.658	2225.611
71.45	140.845	109.857	97.895	124.499	85.296	33.801	14.345	2225.611
71.55	140.590	111.372	99.549	124.561	85.834	33.233	13.648	2225.611
71.65	141.339	110.429	97.930	123.183	84.781	32.828	14.277	2225.611
71.75	141.061	110.496	98.425	126.097	85.565	32.927	14.090	2225.611
71.85	142.614	110.094	97.323	123.099	85.541	33.353	14.483	2225.611
71.95	140.092	110.575	98.732	122.044	83.588	33.320	13.835	2225.611
72.05	139.270	109.547	97.162	122.347	85.506	34.402	13.785	2225.611
72.15	140.403	110.186	97.072	120.373	84.138	33.801	14.395	2225.611
72.25	141.911	110.167	97.965	123.569	83.881	34.970	14.562	2225.611
72.35	142.891	110.478	96.772	123.329	84.161	34.642	13.628	2225.611
72.45	140.789	109.687	99.549	122.253	84.769	33.833	13.294	2225.611
72.55	141.356	112.071	96.095	123.026	84.325	34.041	14.061	2225.611
72.65	139.480	110.593	96.967	124.164	85.822	33.604	13.726	2225.611
72.75	141.038	109.389	98.697	123.966	85.950	33.375	14.404	2225.611
72.85	140.137	110.806	98.858	123.131	84.723	34.489	13.923	2225.611
72.95	141.730	111.001	98.600	124.582	84.348	33.855	14.070	2225.611
73.05	141.254	110.995	98.132	122.723	86.021	33.276	13.412	2225.611
73.15	141.503	109.875	97.742	122.943	83.799	33.768	13.717	2225.611
73.25	139.825	110.058	98.216	124.645	83.752	34.161	14.100	2225.611
73.35	141.684	111.384	97.267	125.355	85.494	33.965	14.395	2225.611
73.45	143.401	111.919	96.960	123.277	85.997	32.621	14.473	2225.611
73.55	140.545	110.982	96.772	125.073	85.529	34.718	13.756	2225.611
73.65	142.772	111.816	96.402	123.245	84.360	32.457	13.500	2225.611

Midpoint	103	30	10	3	1	0.34	0.1	Volume
73.75	141.769	109.656	96.276	121.836	83.916	33.571	14.011	2225.611
73.85	142.104	110.015	98.879	125.689	83.846	34.216	14.110	2225.611
73.95	142.047	109.383	98.258	124.896	84.477	33.495	13.972	2225.611
74.05	140.687	110.520	97.735	123.319	84.115	33.866	14.100	2225.611
74.15	142.551	112.199	98.237	125.627	84.781	32.774	14.178	2225.611
74.25	140.534	110.143	97.142	124.269	82.922	33.549	13.785	2225.611
74.35	141.378	110.009	98.272	122.713	86.348	33.976	13.874	2225.611
74.45	139.417	111.372	97.735	124.822	85.062	34.686	13.402	2225.611
74.55	140.358	111.128	98.530	123.778	85.997	33.997	14.247	2225.611
74.65	140.392	109.577	96.255	123.360	83.343	33.462	13.658	2225.611
74.75	141.894	110.581	98.725	123.225	85.939	32.981	14.424	2225.611
74.85	142.109	111.773	97.156	122.493	83.413	32.402	14.581	2225.611
74.95	141.883	110.283	97.490	124.645	84.793	33.582	14.257	2225.611
75.05	140.562	111.134	97.190	124.572	84.629	32.916	14.208	2225.611
75.15	140.233	113.458	98.795	121.345	84.524	33.549	14.178	2225.611
75.25	140.369	111.232	96.667	123.099	84.243	33.779	13.952	2225.611
75.35	142.381	109.030	98.523	121.209	84.103	34.008	13.884	2225.611
75.45	141.032	113.111	97.372	123.235	84.688	32.675	14.306	2225.611
75.55	141.356	111.037	98.279	125.679	84.348	33.965	13.766	2225.611
75.65	142.234	112.229	98.321	124.728	84.103	34.107	13.618	2225.611
75.75	141.055	112.023	98.090	123.099	83.471	32.293	14.061	2225.611
75.85	140.709	111.025	98.432	123.695	83.904	33.702	14.444	2225.611
75.95	141.854	111.512	97.609	124.154	83.799	33.702	14.562	2225.611
76.05	142.880	113.239	97.462	123.914	82.781	34.839	14.021	2225.611
76.15	141.186	110.891	99.102	123.360	84.571	32.654	14.630	2225.611
76.25	140.148	110.867	98.335	123.016	84.337	33.549	14.345	2225.611
76.35	143.101	110.265	97.469	123.851	83.752	33.309	14.208	2225.611
76.45	141.730	111.408	96.346	122.044	82.781	33.244	14.365	2225.611
76.55	141.310	111.755	99.549	123.496	87.026	34.686	13.442	2225.611
76.65	141.418	110.459	97.449	125.230	85.015	33.189	14.129	2225.611
76.75	141.135	112.552	97.609	123.663	83.506	32.970	13.835	2225.611
76.85	142.325	110.642	99.297	123.914	84.021	33.670	14.336	2225.611
76.95	140.993	112.053	97.065	124.530	86.126	34.074	13.795	2225.611
77.05	140.143	111.469	98.021	123.141	85.810	33.604	14.129	2225.611
77.15	141.384	111.128	97.728	124.175	85.845	32.894	13.255	2225.611
77.25	140.477	109.997	98.830	124.102	82.887	33.091	13.579	2225.611
77.35	140.619	112.223	97.246	123.590	84.009	34.303	14.110	2225.611
77.45	140.647	111.566	98.704	123.956	85.120	32.796	13.825	2225.611
77.55	143.379	112.156	96.953	119.987	84.746	34.205	13.805	2225.611
77.65	140.409	112.558	99.702	123.882	86.652	33.167	13.785	2225.611
77.75	142.444	110.307	96.458	124.875	84.208	34.423	14.011	2225.611
77.85	141.299	111.019	99.004	123.621	84.454	33.484	14.228	2225.611
77.95	140.755	112.552	97.986	123.924	85.857	33.713	13.884	2225.611
78.05	142.755	112.716	99.284	123.757	83.998	34.107	13.982	2225.611
78.15	142.670	112.308	97.651	123.319	85.190	34.249	13.432	2225.611

Midpoint	103	30	10	3	1	0.34	0.1	Volume
78.25	141.123	112.485	98.195	122.880	83.939	33.309	13.599	2225.611
78.35	141.571	111.457	99.221	122.661	84.442	33.866	14.287	2225.611
78.45	140.834	111.001	97.776	124.060	84.571	33.418	13.756	2225.611
78.55	144.014	111.530	98.202	121.877	85.763	33.069	13.609	2225.611
78.65	139.916	111.822	98.258	124.133	84.290	33.451	14.670	2225.611
78.75	141.713	111.220	99.374	123.705	83.483	34.402	13.992	2225.611
78.85	141.605	111.092	98.035	126.316	84.419	33.779	13.962	2225.611
78.95	143.492	111.670	98.342	122.849	85.904	34.085	13.726	2225.611
79.05	141.565	111.834	97.379	126.065	84.723	33.833	13.952	2225.611
79.15	141.622	111.134	98.195	123.193	83.296	34.118	13.599	2225.611
79.25	143.430	110.751	98.537	124.802	85.307	33.593	14.149	2225.611
79.35	140.925	110.782	96.444	123.329	83.822	34.107	13.638	2225.611
79.45	139.780	112.698	97.316	123.799	82.898	33.691	14.856	2225.611
79.55	142.852	111.469	95.209	125.042	84.851	34.609	13.726	2225.611
79.65	141.622	112.375	97.804	123.381	84.793	34.565	13.923	2225.611
79.75	143.753	112.254	97.888	125.929	85.728	33.582	14.630	2225.611
79.85	141.100	110.903	98.921	126.159	84.746	33.637	13.589	2225.611
79.95	141.254	112.114	99.402	125.345	86.547	33.134	14.051	2225.611
80.05	141.611	111.268	98.621	124.290	84.208	33.560	14.277	2225.611
80.15	141.112	111.566	100.023	125.178	85.354	34.303	13.972	2225.611
80.25	141.917	110.897	99.270	124.896	84.992	33.517	14.021	2225.611
80.35	141.866	112.144	98.663	125.115	83.705	33.910	14.237	2225.611
80.45	142.551	111.572	97.860	123.413	85.529	33.560	13.098	2225.611
80.55	141.276	111.341	98.746	125.397	86.079	33.276	14.149	2225.611
80.65	141.786	112.308	98.097	123.193	84.372	34.260	13.894	2225.611
80.75	142.721	111.439	98.544	125.553	86.266	34.019	13.628	2225.611
80.85	142.840	111.682	98.035	125.439	85.132	33.375	14.414	2225.611
80.95	142.081	112.132	98.216	125.679	85.717	33.331	13.717	2225.611
81.05	142.166	111.445	98.970	123.559	85.740	33.528	13.609	2225.611
81.15	141.639	111.159	98.076	126.410	84.910	34.194	13.894	2225.611
81.25	144.886	111.220	97.783	126.922	82.898	34.839	14.866	2225.611
81.35	141.820	110.873	98.649	124.572	85.202	33.637	13.117	2225.611
81.45	142.132	112.442	97.951	125.094	85.342	33.298	13.962	2225.611
81.55	141.174	110.843	98.704	124.269	85.600	33.932	14.070	2225.611
81.65	143.084	113.361	99.151	125.365	84.828	34.008	14.228	2225.611
81.75	142.262	109.888	100.567	123.454	85.342	33.757	13.481	2225.611
81.85	141.673	111.499	98.376	124.311	85.798	34.292	13.854	2225.611
81.95	141.526	112.984	99.235	124.238	85.179	33.265	14.277	2225.611
82.05	141.996	112.929	97.421	125.052	85.471	33.484	13.825	2225.611
82.15	141.628	113.458	99.723	124.969	83.647	33.713	14.080	2225.611
82.25	143.486	113.908	98.983	125.971	84.337	33.866	13.707	2225.611
82.35	143.214	112.010	98.893	125.125	86.383	33.506	13.412	2225.611
82.45	142.727	112.114	99.507	124.906	83.670	33.910	13.648	2225.611
82.55	141.758	113.203	98.069	124.258	85.132	33.604	13.874	2225.611
82.65	144.501	111.919	99.130	126.796	83.986	33.309	14.375	2225.611

Midpoint	103	30	10	3	1	0.34	0.1	Volume
82.75	142.642	111.183	99.290	123.705	84.723	32.828	13.894	2225.611
82.85	141.083	112.807	99.918	125.439	86.208	33.888	14.483	2225.611
82.95	144.637	112.381	97.783	126.159	85.915	32.730	14.228	2225.611
83.05	143.124	111.937	99.507	124.185	84.430	33.244	14.061	2225.611
83.15	141.764	112.205	98.753	124.154	83.799	33.407	14.110	2225.611
83.25	141.922	111.214	100.184	123.266	85.635	34.402	14.169	2225.611
83.35	142.648	112.217	100.358	125.950	85.342	34.249	14.866	2225.611
83.45	143.129	110.697	99.549	125.992	87.471	32.381	14.129	2225.611
83.55	141.401	111.804	99.967	125.094	86.290	34.358	13.402	2225.611
83.65	142.104	112.284	98.251	126.483	86.769	33.473	13.550	2225.611
83.75	142.682	110.940	99.060	123.851	86.699	34.489	13.903	2225.611
83.85	142.563	112.114	100.498	125.418	84.278	34.128	14.237	2225.611
83.95	143.622	112.114	98.607	125.365	84.407	34.118	14.090	2225.611
84.05	142.251	112.613	99.004	125.783	86.395	33.670	13.844	2225.611
84.15	141.254	112.686	98.104	124.050	84.956	34.172	14.739	2225.611
84.25	142.172	112.242	99.639	125.679	86.149	33.440	14.483	2225.611
84.35	142.121	111.280	99.081	125.700	84.535	33.976	13.638	2225.611
84.45	142.285	112.977	98.181	122.807	86.617	33.539	13.628	2225.611
84.55	142.914	112.035	99.570	125.773	86.102	34.369	13.668	2225.611
84.65	146.502	112.716	98.893	124.697	83.530	34.183	13.903	2225.611
84.75	142.835	112.722	99.290	126.410	86.453	34.281	14.021	2225.611
84.85	145.357	112.759	98.858	125.125	85.646	34.697	14.257	2225.611
84.95	143.900	112.740	100.574	123.569	83.857	33.823	14.444	2225.611
85.05	143.441	111.001	98.656	125.073	85.670	33.364	14.345	2225.611
85.15	143.923	111.889	98.781	124.572	87.482	33.145	13.854	2225.611
85.25	144.178	113.002	100.630	124.802	85.190	32.992	13.746	2225.611
85.35	142.370	112.442	97.986	125.334	85.179	33.757	13.550	2225.611
85.45	143.793	111.980	100.149	126.681	88.114	33.855	13.923	2225.611
85.55	143.549	113.130	99.374	126.681	84.489	33.943	14.070	2225.611
85.65	143.044	112.765	100.630	125.553	86.219	33.670	14.100	2225.611
85.75	142.914	111.244	97.707	126.232	84.793	33.866	13.943	2225.611
85.85	143.532	112.838	100.777	125.741	84.500	33.069	14.837	2225.611
85.95	143.152	113.026	99.472	125.251	85.401	34.270	13.461	2225.611
86.05	143.639	113.203	98.244	125.512	87.412	33.397	13.697	2225.611
86.15	143.197	111.694	99.849	127.392	85.986	34.063	14.945	2225.611
86.25	141.639	112.266	99.842	125.240	85.261	33.921	13.638	2225.611
86.35	142.908	111.043	97.951	125.303	85.740	32.796	13.874	2225.611
86.45	141.231	111.603	100.811	126.149	85.319	34.314	13.589	2225.611
86.55	141.854	112.369	100.609	125.313	84.711	34.358	13.854	2225.611
86.65	143.844	111.664	99.409	124.896	86.535	34.260	13.815	2225.611
86.75	143.526	112.211	99.737	125.825	83.577	35.625	14.375	2225.611
86.85	141.769	112.308	100.044	125.146	87.096	35.549	14.404	2225.611
86.95	142.138	113.957	99.249	124.530	87.868	33.790	13.933	2225.611
87.05	146.263	110.666	99.563	124.697	85.483	33.604	13.795	2225.611
87.15	143.991	111.487	98.579	124.405	84.688	33.615	13.903	2225.611

Midpoint	103	30	10	3	1	0.34	0.1	Volume
87.25	141.480	112.935	99.814	126.410	86.360	33.254	13.933	2225.611
87.35	144.280	110.922	100.239	124.060	86.664	33.539	13.677	2225.611
87.45	142.551	112.832	100.581	125.919	84.629	32.916	13.844	2225.611
87.55	141.928	112.150	99.235	125.501	88.710	33.823	13.658	2225.611
87.65	142.251	112.673	98.363	125.491	86.921	33.560	13.943	2225.611
87.75	144.297	112.515	97.958	127.454	85.097	32.828	13.844	2225.611
87.85	141.854	110.405	99.221	126.128	85.962	34.904	13.884	2225.611
87.95	143.056	111.433	98.670	124.990	83.354	34.096	14.070	2225.611
88.05	142.931	111.469	98.237	126.577	86.734	34.238	13.157	2225.611
88.15	143.186	110.873	98.063	124.885	83.121	34.697	14.296	2225.611
88.25	142.925	112.248	100.239	124.990	84.267	34.544	14.424	2225.611
88.35	142.863	112.266	98.572	126.285	85.693	34.511	14.345	2225.611
88.45	143.719	112.759	98.942	123.287	85.296	34.631	14.188	2225.611
88.55	142.172	110.180	98.502	124.363	85.132	34.347	14.375	2225.611
88.65	143.458	112.898	97.916	123.308	83.541	33.637	13.746	2225.611
88.75	142.432	112.929	98.349	122.097	85.413	33.506	13.471	2225.611
88.85	143.231	111.621	98.000	124.206	84.688	33.681	13.599	2225.611
88.95	142.999	111.487	98.314	122.608	83.740	33.539	13.500	2225.611
89.05	142.143	111.141	99.563	123.026	86.605	33.517	14.119	2225.611
89.15	141.276	112.728	98.600	123.997	85.997	32.588	13.677	2225.611
89.25	143.509	111.043	98.774	122.849	84.933	34.325	14.365	2225.611
89.35	139.349	111.262	98.593	124.415	84.091	33.823	13.864	2225.611
89.45	140.171	110.958	97.518	125.846	81.296	31.266	13.638	2225.611
89.55	139.718	111.013	97.714	122.201	84.196	33.298	13.324	2225.611
89.65	141.032	109.060	98.056	124.603	84.056	33.604	13.550	2225.611
89.75	140.976	111.153	97.462	123.809	83.202	32.675	13.746	2225.611
89.85	140.794	110.496	97.993	124.321	83.156	33.156	14.100	2225.611
89.95	141.344	109.413	98.167	121.950	84.535	32.632	14.306	2225.611
90.05	138.913	110.928	98.195	124.133	84.910	33.200	12.950	2225.611
90.15	141.106	110.800	97.721	121.877	83.144	33.823	14.041	2225.611
90.25	137.740	110.697	99.151	125.063	83.810	32.949	13.844	2225.611
90.35	139.706	110.332	96.569	124.070	83.846	33.866	13.835	2225.611
90.45	138.448	110.046	96.311	123.183	84.021	33.473	13.825	2225.611
90.55	137.825	110.471	97.365	119.298	83.810	33.888	13.884	2225.611
90.65	139.151	109.091	96.681	123.225	83.167	33.659	14.365	2225.611
90.75	137.513	110.441	96.276	120.603	82.208	32.719	13.844	2225.611
90.85	140.840	109.170	97.602	123.339	83.319	32.992	13.550	2225.611
90.95	138.562	109.291	96.451	123.789	82.477	32.872	13.746	2225.611
91.05	137.439	111.633	96.904	121.836	81.939	33.429	13.353	2225.611
91.15	138.170	110.173	96.876	122.264	81.133	32.675	13.648	2225.611
91.25	139.837	108.890	96.507	121.689	84.126	33.254	13.638	2225.611
91.35	138.437	109.699	95.565	120.050	82.922	32.370	13.962	2225.611
91.45	137.955	109.772	95.732	122.473	82.337	32.763	13.039	2225.611
91.55	138.250	109.474	96.479	122.326	81.238	31.780	13.795	2225.611
91.65	138.630	109.444	96.032	122.159	82.641	32.960	14.041	2225.611

Midpoint	103	30	10	3	1	0.34	0.1	Volume
91.75	137.944	107.868	98.063	121.700	82.875	33.735	13.402	2225.611
91.85	138.420	109.304	98.014	120.551	82.536	33.407	12.636	2225.611
91.95	137.553	109.602	96.067	119.757	80.864	34.161	13.589	2225.611
92.05	136.884	109.681	96.297	120.499	81.764	31.441	13.618	2225.611
92.15	139.633	108.434	96.402	120.112	82.945	32.555	13.255	2225.611
92.25	137.847	107.655	95.648	120.802	82.840	32.424	13.638	2225.611
92.35	138.165	108.628	96.060	119.256	81.589	32.544	13.058	2225.611
92.45	136.578	109.164	95.669	118.807	82.454	33.691	13.343	2225.611
92.55	137.490	106.518	97.204	121.073	80.817	33.724	13.491	2225.611
92.65	137.864	109.109	96.814	121.501	81.998	32.675	13.324	2225.611
92.75	138.471	106.883	95.321	120.635	80.068	32.599	13.383	2225.611
92.85	138.255	107.868	95.167	118.535	82.466	32.533	13.353	2225.611
92.95	135.280	107.290	95.697	119.423	81.191	31.998	13.314	2225.611
93.05	137.343	107.941	95.314	121.606	80.735	31.463	13.726	2225.611
93.15	137.751	106.639	95.593	120.008	81.682	31.583	12.597	2225.611
93.25	135.853	107.965	95.000	118.901	80.676	32.140	13.206	2225.611
93.35	136.890	108.318	94.218	121.501	81.097	32.555	13.972	2225.611
93.45	134.736	108.434	96.688	120.039	81.179	32.588	13.697	2225.611
93.55	137.377	108.020	96.088	120.958	81.530	32.435	13.245	2225.611
93.65	137.128	106.804	94.825	121.606	80.618	33.189	13.009	2225.611
93.75	137.088	107.491	95.586	123.507	82.068	32.533	13.186	2225.611
93.85	137.094	107.187	95.146	120.499	81.612	32.435	13.825	2225.611
93.95	136.935	108.239	96.283	120.467	82.957	31.998	13.933	2225.611
94.05	138.023	107.290	96.346	119.475	80.010	32.326	13.579	2225.611
94.15	137.847	107.850	93.095	119.601	81.016	33.102	13.874	2225.611
94.25	137.003	108.403	94.086	121.334	81.834	32.654	12.999	2225.611
94.35	138.998	107.473	98.453	120.488	81.717	33.648	13.628	2225.611
94.45	137.094	108.008	95.537	121.992	82.220	31.856	13.579	2225.611
94.55	138.085	108.270	95.760	118.880	81.729	32.577	13.373	2225.611
94.65	137.014	107.819	96.646	119.820	82.711	32.533	13.078	2225.611
94.75	138.068	109.097	97.379	120.823	82.349	31.780	13.098	2225.611
94.85	139.338	109.188	96.137	120.478	83.962	32.763	13.540	2225.611
94.95	138.533	109.212	96.325	120.875	81.904	32.402	13.107	2225.611
95.05	137.870	106.597	96.332	120.217	83.658	32.293	13.520	2225.611
95.15	137.949	108.781	94.909	121.909	80.291	33.287	13.176	2225.611
95.25	136.907	107.509	94.965	123.486	84.968	33.855	12.990	2225.611
95.35	138.477	108.227	94.741	120.196	82.419	31.823	13.147	2225.611
95.45	137.921	107.083	96.974	122.890	83.425	33.287	12.803	2225.611
95.55	138.981	107.844	96.876	120.864	82.395	32.020	12.921	2225.611
95.65	138.006	108.835	95.251	122.337	83.717	31.725	13.707	2225.611
95.75	138.715	108.525	96.716	123.005	81.951	32.839	13.265	2225.611
95.85	136.822	109.097	94.623	119.131	81.694	32.828	13.284	2225.611
95.95	137.071	109.206	96.716	119.569	83.588	32.817	13.835	2225.611
96.05	138.170	108.866	96.918	123.903	83.553	32.981	13.992	2225.611
96.15	137.853	108.854	96.221	122.431	82.501	33.528	13.373	2225.611

Midpoint	103	30	10	3	1	0.34	0.1	Volume
96.25	138.029	109.492	95.467	121.804	81.378	32.555	13.265	2225.611
96.35	137.570	109.145	97.358	122.556	82.512	32.315	13.952	2225.611
96.45	139.451	108.781	97.344	119.548	81.869	32.708	13.874	2225.611
96.55	138.930	107.789	95.795	124.363	83.986	34.533	13.235	2225.611
96.65	139.735	109.693	96.283	121.292	84.606	33.200	14.336	2225.611
96.75	137.417	109.541	96.493	120.279	84.921	33.080	13.510	2225.611
96.85	140.075	109.310	96.562	123.277	85.798	32.512	14.021	2225.611
96.95	139.270	109.413	96.325	122.170	81.858	33.691	13.648	2225.611
97.05	139.831	107.704	96.109	123.841	83.682	34.292	13.648	2225.611
97.15	139.565	108.860	96.639	121.606	82.197	33.200	13.923	2225.611
97.25	141.435	110.982	96.165	123.360	83.635	33.254	13.952	2225.611
97.35	139.049	109.200	95.655	123.757	84.746	32.785	14.031	2225.611
97.45	141.571	109.669	97.707	124.081	84.337	33.735	13.274	2225.611
97.55	140.851	109.504	95.809	125.522	84.851	33.407	13.638	2225.611
97.65	141.027	109.498	95.202	123.882	84.840	34.194	13.766	2225.611
97.75	139.684	108.671	96.890	123.862	84.851	33.298	13.923	2225.611
97.85	140.777	110.751	97.386	123.684	85.787	33.473	13.029	2225.611
97.95	140.256	110.034	97.135	124.352	83.986	32.774	13.353	2225.611
98.05	142.659	108.501	96.974	123.768	84.863	32.686	13.638	2225.611
98.15	140.483	111.305	96.646	124.927	84.243	34.489	13.687	2225.611
98.25	139.378	111.037	96.353	127.371	85.775	34.041	13.854	2225.611
98.35	142.716	111.110	97.881	124.144	84.746	33.954	14.414	2225.611
98.45	143.520	108.981	97.260	125.449	85.646	33.178	14.159	2225.611
98.55	141.633	112.126	97.742	124.645	85.062	33.211	14.581	2225.611
98.65	142.172	109.389	97.149	126.848	86.430	33.833	13.795	2225.611
98.75	140.930	110.490	96.744	125.564	82.828	33.200	14.208	2225.611
98.85	143.690	111.524	98.042	124.614	85.401	33.451	14.002	2225.611
98.95	141.798	110.843	98.237	125.491	83.413	33.954	13.746	2225.611
99.05	141.514	111.506	97.142	125.668	86.336	34.860	14.021	2225.611
99.15	143.231	109.833	98.697	126.326	85.623	34.107	14.139	2225.611
99.25	144.167	110.581	98.314	128.927	86.360	33.025	13.048	2225.611
99.35	141.520	109.881	98.160	126.514	85.927	33.254	14.139	2225.611
99.45	142.534	110.988	98.216	128.342	84.348	33.670	13.746	2225.611
99.55	97.994	76.135	66.666	127.935	59.627	34.314	9.580	2225.611
99.65	0.000	0.000	0.000	8.700	0.000	2.316	0.000	2225.611
99.75	0.000	0.000	0.000	0.000	0.000	0.000	0.000	2225.611
99.85	0.000	0.000	0.000	0.000	0.000	0.000	0.000	2225.611
99.95	0.000	0.000	0.000	0.000	0.000	0.000	0.000	2225.611

Table of Ca^{2+} population data for simulation with Ca^{2+} and Ba^{2+} across bulk density ratios (#JJF-GRS12G8)

	Population in histogram bin at Ba^{2+}/Ca^{2+} ratio:							
Midpoint	103	30	10	3	1	0.34	0.1	Volume
-99.95	0.221	0.699	1.751	24.271	15.658	56.196	25.557	2244.159

Midpoint	103	30	10	3	1	0.34	0.1	Volume
-99.85	1.411	3.796	9.335	40.939	83.740	99.927	131.438	2244.159
-99.75	1.394	3.631	9.712	41.305	85.132	97.655	133.590	2244.159
-99.65	1.536	3.735	9.593	40.751	83.846	97.666	130.731	2244.159
-99.55	1.428	3.905	9.259	41.607	84.617	99.753	135.270	2244.159
-99.45	1.309	3.534	9.363	42.558	84.606	98.769	132.322	2244.159
-99.35	1.434	3.467	9.705	40.365	85.097	98.081	131.546	2244.159
-99.25	1.474	3.577	9.349	40.751	82.945	99.174	132.902	2244.159
-99.15	1.349	3.826	9.454	42.182	85.518	98.354	130.387	2244.159
-99.05	1.303	3.570	10.012	42.600	84.337	98.005	131.281	2244.159
-98.95	1.423	3.838	9.628	40.093	82.863	98.245	134.268	2244.159
-98.85	1.417	3.771	9.733	40.991	83.939	97.371	131.644	2244.159
-98.75	1.173	3.844	9.607	42.130	85.366	99.545	133.108	2244.159
-98.65	1.383	3.905	9.684	41.367	85.062	97.579	131.212	2244.159
-98.55	1.417	3.716	9.266	40.814	82.220	97.327	130.888	2244.159
-98.45	1.332	3.522	9.810	40.876	83.214	98.321	131.527	2244.159
-98.35	1.394	3.455	9.949	41.451	83.857	98.431	131.959	2244.159
-98.25	1.320	3.826	10.347	41.179	84.699	96.464	132.509	2244.159
-98.15	1.451	3.759	9.852	41.597	82.840	98.037	133.894	2244.159
-98.05	1.366	3.923	9.049	41.044	83.822	97.404	133.148	2244.159
-97.95	1.281	3.832	9.273	40.124	84.337	97.710	133.531	2244.159
-97.85	1.343	3.358	9.566	39.414	82.852	96.475	131.625	2244.159
-97.75	1.332	3.595	9.963	41.513	82.162	98.158	129.237	2244.159
-97.65	1.496	3.504	9.377	40.835	84.547	97.600	128.146	2244.159
-97.55	1.383	3.966	9.084	41.743	84.208	96.038	130.131	2244.159
-97.45	1.394	3.643	9.398	40.636	83.179	97.032	131.939	2244.159
-97.35	1.264	3.650	9.468	40.668	81.706	95.131	131.438	2244.159
-97.25	1.315	3.710	9.217	39.947	81.402	97.284	129.954	2244.159
-97.15	1.406	3.583	9.719	41.607	82.770	97.054	130.731	2244.159
-97.05	1.230	3.905	9.775	41.096	82.372	95.405	129.876	2244.159
-96.95	1.139	3.637	9.691	40.459	81.799	97.830	131.340	2244.159
-96.85	1.445	3.583	9.614	41.190	84.079	95.656	130.249	2244.159
-96.75	1.462	3.504	9.014	40.448	81.472	97.349	129.984	2244.159
-96.65	1.377	3.729	9.300	40.981	82.688	96.552	131.340	2244.159
-96.55	1.298	3.577	9.161	39.895	82.781	95.361	130.544	2244.159
-96.45	1.525	3.577	9.873	40.459	83.998	95.689	128.215	2244.159
-96.35	1.309	3.619	9.056	41.138	81.799	96.530	130.279	2244.159
-96.25	1.366	3.771	9.496	40.970	83.085	97.797	131.635	2244.159
-96.15	1.292	3.461	9.628	39.686	83.471	94.946	131.566	2244.159
-96.05	1.252	3.437	9.412	40.939	81.589	95.579	126.447	2244.159
-95.95	1.366	3.686	9.419	40.177	82.957	95.798	130.898	2244.159
-95.85	1.252	3.528	9.412	40.542	81.834	97.120	131.094	2244.159
-95.75	1.213	3.443	9.552	40.866	80.571	95.918	130.220	2244.159
-95.65	1.337	3.552	9.642	39.383	81.039	97.426	128.648	2244.159
-95.55	1.286	3.351	9.419	39.498	83.354	97.163	128.628	2244.159
-95.45	1.224	3.534	9.824	41.023	81.554	97.294	129.719	2244.159

Midpoint	103	30	10	3	1	0.34	0.1	Volume
-95.35	1.326	3.461	9.475	40.166	84.383	95.361	131.900	2244.159
-95.25	1.445	3.777	9.342	40.521	80.770	98.048	131.458	2244.159
-95.15	1.355	3.777	9.593	40.751	82.279	95.667	129.817	2244.159
-95.05	1.292	3.698	9.398	40.041	81.612	97.808	129.208	2244.159
-94.95	1.303	3.607	9.524	39.811	81.156	97.273	128.785	2244.159
-94.85	1.400	3.875	9.586	40.302	81.811	98.529	130.230	2244.159
-94.75	1.366	3.668	9.628	41.085	81.928	96.049	132.008	2244.159
-94.65	1.337	3.881	9.517	39.341	80.291	96.475	133.069	2244.159
-94.55	1.309	3.789	9.266	40.323	80.560	97.054	129.473	2244.159
-94.45	1.389	3.388	9.293	39.696	81.238	96.410	127.970	2244.159
-94.35	1.201	3.540	9.210	39.759	81.238	95.492	129.610	2244.159
-94.25	1.241	3.686	9.740	39.122	80.712	95.710	132.204	2244.159
-94.15	1.298	3.650	9.210	39.769	83.857	96.158	129.355	2244.159
-94.05	1.366	3.589	9.377	41.545	83.460	97.579	128.598	2244.159
-93.95	1.275	3.479	9.531	39.602	81.811	94.815	129.552	2244.159
-93.85	1.326	3.558	9.489	41.221	80.992	95.394	130.554	2244.159
-93.75	1.496	3.862	9.782	39.540	81.156	96.268	130.004	2244.159
-93.65	1.258	3.716	9.914	41.117	81.904	95.405	128.500	2244.159
-93.55	1.360	3.643	9.607	41.273	79.425	96.377	130.259	2244.159
-93.45	1.372	3.589	9.398	38.621	79.764	96.464	128.255	2244.159
-93.35	1.156	3.747	9.286	39.247	82.033	97.404	130.210	2244.159
-93.25	1.400	3.698	9.517	40.741	81.343	95.077	131.644	2244.159
-93.15	1.474	3.668	9.824	39.519	81.554	95.831	128.304	2244.159
-93.05	1.145	3.832	8.986	40.239	81.016	96.934	128.372	2244.159
-92.95	1.326	3.723	9.684	40.156	83.658	96.289	131.792	2244.159
-92.85	1.201	3.832	9.140	39.519	83.694	95.885	129.326	2244.159
-92.75	1.468	3.558	9.391	41.576	83.857	98.289	130.829	2244.159
-92.65	1.372	3.723	9.391	40.135	83.787	97.830	130.632	2244.159
-92.55	1.389	3.808	9.761	41.127	82.711	99.272	130.171	2244.159
-92.45	1.173	3.729	9.328	40.469	82.185	96.770	131.703	2244.159
-92.35	1.423	3.814	9.363	40.782	82.968	96.715	133.010	2244.159
-92.25	1.201	3.564	9.831	39.581	81.565	98.452	130.672	2244.159
-92.15	1.264	3.735	9.607	40.605	82.688	96.333	133.668	2244.159
-92.05	1.372	4.094	9.768	41.399	81.413	97.404	132.116	2244.159
-91.95	1.332	3.729	9.970	40.918	82.477	97.152	131.448	2244.159
-91.85	1.423	3.899	9.517	40.657	82.676	95.852	132.499	2244.159
-91.75	1.394	3.759	9.782	40.521	83.764	96.027	132.499	2244.159
-91.65	1.213	3.856	9.210	41.399	82.103	99.195	132.509	2244.159
-91.55	1.383	3.844	9.880	41.232	82.068	98.223	130.849	2244.159
-91.45	1.298	3.625	10.193	40.553	84.676	99.632	130.790	2244.159
-91.35	1.360	3.662	9.740	39.947	82.922	97.142	132.214	2244.159
-91.25	1.286	3.601	9.412	41.044	83.039	97.032	131.978	2244.159
-91.15	1.224	4.002	9.873	40.866	84.044	99.949	130.230	2244.159
-91.05	1.496	3.710	9.810	40.636	83.623	97.939	131.890	2244.159
-90.95	1.332	3.650	9.970	41.754	82.571	97.131	134.857	2244.159

Midpoint	103	30	10	3	1	0.34	0.1	Volume
-90.85	1.485	3.826	9.873	40.762	84.196	99.468	132.136	2244.159
-90.75	1.298	3.510	9.468	42.203	81.858	97.775	133.226	2244.159
-90.65	1.394	3.783	9.517	42.182	83.109	99.534	136.194	2244.159
-90.55	1.423	3.729	9.705	41.660	84.302	99.152	136.105	2244.159
-90.45	1.326	4.002	9.733	41.942	85.506	96.519	134.209	2244.159
-90.35	1.309	3.692	9.817	41.054	87.225	99.785	135.948	2244.159
-90.25	1.513	3.881	9.845	42.015	84.629	97.600	134.317	2244.159
-90.15	1.315	4.106	10.061	42.600	85.015	99.261	135.545	2244.159
-90.05	1.105	3.716	9.426	42.840	85.693	100.069	132.755	2244.159
-89.95	1.400	3.789	9.663	41.670	84.921	100.342	133.865	2244.159
-89.85	1.445	3.893	9.698	41.555	85.576	101.500	133.158	2244.159
-89.75	1.389	3.710	9.726	41.639	86.231	98.922	135.840	2244.159
-89.65	1.445	3.887	9.956	41.221	82.267	101.675	134.661	2244.159
-89.55	1.235	3.668	9.600	42.892	86.933	97.863	138.532	2244.159
-89.45	1.269	3.723	10.124	42.057	86.219	100.954	135.319	2244.159
-89.35	1.377	3.510	9.628	41.754	85.272	101.238	135.467	2244.159
-89.25	1.440	3.954	9.914	43.070	85.646	100.408	134.965	2244.159
-89.15	1.394	3.844	9.614	41.837	85.775	98.769	138.002	2244.159
-89.05	1.201	3.753	9.607	42.996	84.804	101.664	136.675	2244.159
-88.95	1.428	3.814	10.012	41.576	85.027	101.468	135.987	2244.159
-88.85	1.377	3.680	9.670	42.746	87.857	101.752	138.375	2244.159
-88.75	1.474	3.741	9.824	43.007	85.296	100.856	137.668	2244.159
-88.65	1.241	3.704	9.719	42.203	86.278	100.288	138.355	2244.159
-88.55	1.281	3.814	10.940	42.652	86.781	102.462	135.879	2244.159
-88.45	1.530	3.802	9.531	41.482	87.529	101.577	139.367	2244.159
-88.35	1.462	3.923	10.026	41.513	85.974	101.686	136.243	2244.159
-88.25	1.474	3.911	9.880	42.464	85.483	101.642	138.404	2244.159
-88.15	1.587	3.692	10.214	42.944	88.172	101.096	135.467	2244.159
-88.05	1.286	4.069	9.914	41.983	87.085	102.877	137.147	2244.159
-87.95	1.530	3.838	9.824	42.067	88.289	99.621	136.036	2244.159
-87.85	1.451	3.881	10.326	41.816	87.892	100.069	137.039	2244.159
-87.75	1.604	3.619	9.942	42.255	87.015	101.752	139.977	2244.159
-87.65	1.411	3.856	9.928	41.252	86.617	99.720	136.567	2244.159
-87.55	1.417	3.844	9.510	41.910	86.559	100.200	137.392	2244.159
-87.45	1.417	3.796	9.873	42.286	86.664	101.009	137.333	2244.159
-87.35	1.434	3.747	10.033	43.132	86.968	101.763	137.638	2244.159
-87.25	1.315	3.789	9.412	42.902	87.798	101.096	137.186	2244.159
-87.15	1.389	3.650	9.970	42.161	87.412	101.872	137.727	2244.159
-87.05	1.372	3.850	10.012	42.882	87.108	104.450	137.058	2244.159
-86.95	1.315	4.118	9.845	42.359	87.400	103.030	135.034	2244.159
-86.85	1.417	3.607	9.510	42.641	86.816	102.003	136.881	2244.159
-86.75	1.303	3.595	10.438	41.775	87.307	101.446	139.505	2244.159
-86.65	1.530	3.948	9.621	41.754	87.143	101.435	138.002	2244.159
-86.55	1.394	3.698	9.726	42.527	85.939	100.790	136.813	2244.159
-86.45	1.269	3.850	9.447	42.474	90.043	100.517	138.748	2244.159

Midpoint	103	30	10	3	1	0.34	0.1	Volume
-86.35	1.423	3.680	9.914	42.171	85.974	100.375	139.643	2244.159
-86.25	1.292	3.765	10.180	42.704	87.003	100.408	137.039	2244.159
-86.15	1.343	3.856	9.768	43.675	88.055	101.205	140.556	2244.159
-86.05	1.417	3.820	10.159	40.887	86.968	99.468	137.835	2244.159
-85.95	1.281	3.765	9.928	43.905	87.459	102.921	135.889	2244.159
-85.85	1.428	3.680	10.166	42.297	87.050	99.709	135.889	2244.159
-85.75	1.343	3.966	9.928	43.550	85.752	102.713	136.950	2244.159
-85.65	1.355	3.808	10.277	42.015	85.389	101.424	137.098	2244.159
-85.55	1.530	3.808	10.075	42.506	87.517	102.877	138.778	2244.159
-85.45	1.394	3.674	9.677	43.446	87.073	101.894	134.101	2244.159
-85.35	1.258	3.759	9.893	41.367	86.360	100.179	138.188	2244.159
-85.25	1.428	3.881	9.921	43.038	86.442	102.112	138.522	2244.159
-85.15	1.394	3.625	10.124	43.320	86.126	100.058	138.974	2244.159
-85.05	1.320	3.832	10.340	43.028	86.874	102.888	137.451	2244.159
-84.95	1.377	3.808	9.663	40.824	87.073	102.025	137.245	2244.159
-84.85	1.564	3.808	9.880	42.098	87.833	101.413	136.302	2244.159
-84.75	1.423	3.631	10.068	41.430	85.483	100.616	138.513	2244.159
-84.65	1.400	3.875	9.866	42.391	86.056	101.009	137.589	2244.159
-84.55	1.349	4.014	9.886	41.472	87.868	100.736	138.296	2244.159
-84.45	1.502	3.966	9.768	42.098	87.786	99.447	136.430	2244.159
-84.35	1.417	3.911	10.166	42.380	86.909	100.080	137.147	2244.159
-84.25	1.445	3.747	9.880	42.098	87.225	101.402	137.324	2244.159
-84.15	1.457	3.729	9.538	41.419	87.471	99.709	137.343	2244.159
-84.05	1.423	3.954	9.970	43.362	86.290	99.119	138.296	2244.159
-83.95	1.445	3.978	10.082	41.399	86.874	100.616	136.587	2244.159
-83.85	1.525	3.595	9.782	42.662	86.886	99.600	136.361	2244.159
-83.75	1.485	3.643	9.573	43.070	87.260	101.173	136.518	2244.159
-83.65	1.298	3.674	9.677	41.701	86.477	101.348	141.136	2244.159
-83.55	1.269	3.917	9.733	41.837	84.956	102.495	137.029	2244.159
-83.45	1.292	3.771	10.061	42.934	86.044	101.632	136.528	2244.159
-83.35	1.411	3.820	9.210	42.036	86.559	99.654	138.120	2244.159
-83.25	1.474	3.972	9.893	42.224	85.494	100.463	138.984	2244.159
-83.15	1.468	3.674	9.545	42.046	86.991	101.632	137.324	2244.159
-83.05	1.468	3.826	9.649	42.474	87.248	102.582	139.456	2244.159
-82.95	1.377	3.643	9.824	41.785	84.430	101.348	136.960	2244.159
-82.85	1.337	3.917	10.124	42.934	86.956	101.162	137.471	2244.159
-82.75	1.440	3.637	10.005	41.670	84.115	99.993	136.262	2244.159
-82.65	1.411	3.625	9.726	41.869	87.611	101.260	137.736	2244.159
-82.55	1.383	3.838	9.754	42.474	86.453	102.320	139.122	2244.159
-82.45	1.372	3.698	10.019	42.882	86.442	100.987	138.689	2244.159
-82.35	1.406	3.680	10.054	43.393	84.173	100.124	137.461	2244.159
-82.25	1.417	3.613	9.810	43.508	85.775	99.589	137.029	2244.159
-82.15	1.604	3.723	9.586	41.127	85.822	100.244	138.395	2244.159
-82.05	1.394	3.789	10.103	43.153	86.746	98.780	136.469	2244.159
-81.95	1.406	3.765	9.747	41.691	84.138	99.370	136.980	2244.159

Midpoint	103	30	10	3	1	0.34	0.1	Volume
-81.85	1.337	3.656	10.005	41.075	87.073	100.921	139.584	2244.159
-81.75	1.474	3.820	9.649	41.910	85.880	102.003	138.080	2244.159
-81.65	1.406	3.808	9.545	41.555	86.617	101.435	135.702	2244.159
-81.55	1.513	3.923	9.349	42.213	86.114	99.206	136.587	2244.159
-81.45	1.496	4.008	10.326	41.754	85.354	100.921	136.999	2244.159
-81.35	1.309	3.941	9.566	42.255	85.132	103.762	137.825	2244.159
-81.25	1.355	3.631	9.921	40.720	84.652	101.260	136.282	2244.159
-81.15	1.417	3.990	9.377	41.138	86.933	100.484	135.928	2244.159
-81.05	1.434	3.972	9.356	40.187	85.003	100.146	136.950	2244.159
-80.95	1.377	4.106	9.810	42.077	85.424	98.769	138.404	2244.159
-80.85	1.502	3.996	10.131	42.339	88.383	100.976	139.898	2244.159
-80.75	1.491	3.710	9.907	41.869	86.278	99.665	136.675	2244.159
-80.65	1.383	3.935	9.761	42.339	85.494	101.096	136.577	2244.159
-80.55	1.468	3.771	9.956	42.297	86.757	100.113	135.673	2244.159
-80.45	1.474	3.771	9.719	41.576	85.997	101.140	136.783	2244.159
-80.35	1.383	3.796	9.614	41.419	85.869	101.861	139.721	2244.159
-80.25	1.644	3.802	9.405	43.268	85.261	102.134	138.375	2244.159
-80.15	1.411	3.601	9.880	41.545	84.980	99.239	137.088	2244.159
-80.05	1.428	3.783	9.873	42.589	86.699	100.954	139.102	2244.159
-79.95	1.474	3.826	9.921	42.349	85.342	101.096	137.392	2244.159
-79.85	1.383	3.613	9.893	42.203	83.892	100.998	137.933	2244.159
-79.75	1.468	3.589	9.691	40.960	88.570	100.474	136.262	2244.159
-79.65	1.360	3.710	10.117	42.088	86.114	99.523	133.924	2244.159
-79.55	1.355	3.948	10.096	42.151	87.447	101.500	135.545	2244.159
-79.45	1.332	3.783	9.377	41.284	84.769	100.976	136.174	2244.159
-79.35	1.400	3.789	9.593	41.315	86.126	99.436	135.850	2244.159
-79.25	1.479	3.528	10.152	42.746	85.600	100.299	136.695	2244.159
-79.15	1.462	3.650	9.705	40.803	85.471	101.042	136.547	2244.159
-79.05	1.406	3.789	10.312	42.694	86.523	99.458	137.835	2244.159
-78.95	1.355	3.643	9.342	42.276	86.056	100.474	139.171	2244.159
-78.85	1.417	3.850	9.942	41.367	85.705	98.857	137.569	2244.159
-78.75	1.462	3.856	9.935	41.628	85.354	101.369	133.944	2244.159
-78.65	1.309	3.692	9.586	40.887	86.395	97.721	138.120	2244.159
-78.55	1.468	3.844	9.586	40.876	85.717	99.665	138.041	2244.159
-78.45	1.269	3.850	9.468	43.028	86.032	101.577	137.785	2244.159
-78.35	1.275	3.777	9.782	41.169	85.319	99.785	138.689	2244.159
-78.25	1.496	3.741	9.370	42.318	86.605	99.141	138.856	2244.159
-78.15	1.394	3.905	10.082	42.036	86.231	99.916	138.002	2244.159
-78.05	1.513	3.504	9.663	42.265	85.319	99.567	135.850	2244.159
-77.95	1.508	3.796	10.277	42.652	85.015	100.310	134.366	2244.159
-77.85	1.383	3.881	9.789	41.785	86.734	100.288	138.159	2244.159
-77.75	1.372	3.881	9.998	41.983	86.371	99.141	136.479	2244.159
-77.65	1.264	3.844	9.607	40.970	85.459	99.184	135.614	2244.159
-77.55	1.547	3.850	10.131	42.527	86.570	101.544	136.793	2244.159
-77.45	1.428	3.650	9.545	41.211	87.775	100.550	135.005	2244.159

Midpoint	103	30	10	3	1	0.34	0.1	Volume
-77.35	1.343	3.680	9.524	41.555	86.827	102.025	137.835	2244.159
-77.25	1.491	3.637	9.880	41.639	87.459	100.386	137.255	2244.159
-77.15	1.383	3.881	9.831	42.913	86.138	100.255	133.963	2244.159
-77.05	1.360	3.741	9.663	40.939	86.395	102.003	138.247	2244.159
-76.95	1.372	3.814	9.970	42.631	84.617	99.567	135.024	2244.159
-76.85	1.298	3.759	9.949	41.576	86.804	99.665	137.736	2244.159
-76.75	1.400	3.716	10.221	41.816	84.009	99.130	137.373	2244.159
-76.65	1.468	3.881	9.928	43.090	85.880	101.479	137.785	2244.159
-76.55	1.360	3.686	9.405	40.876	85.763	100.463	136.302	2244.159
-76.45	1.326	3.832	9.775	41.461	86.734	99.884	137.373	2244.159
-76.35	1.406	3.656	9.838	40.574	85.436	98.900	137.206	2244.159
-76.25	1.394	3.820	9.907	42.213	83.717	98.922	135.270	2244.159
-76.15	1.343	3.802	10.235	42.109	85.003	101.490	136.577	2244.159
-76.05	1.383	4.215	10.096	41.294	85.261	100.561	136.538	2244.159
-75.95	1.389	3.875	9.607	41.973	85.986	101.260	135.359	2244.159
-75.85	1.423	3.656	9.656	41.493	85.237	100.474	137.432	2244.159
-75.75	1.485	3.656	9.419	40.991	84.617	101.544	137.088	2244.159
-75.65	1.496	3.765	9.768	41.388	87.354	99.753	135.290	2244.159
-75.55	1.224	3.710	9.914	42.077	86.570	101.184	135.624	2244.159
-75.45	1.303	3.753	9.503	41.733	85.810	98.933	138.198	2244.159
-75.35	1.303	3.504	9.754	42.715	85.728	99.326	135.732	2244.159
-75.25	1.462	3.923	9.531	41.942	85.179	99.195	134.582	2244.159
-75.15	1.360	3.850	10.200	42.297	86.547	100.200	137.196	2244.159
-75.05	1.303	4.136	9.782	41.722	86.266	98.420	136.587	2244.159
-74.95	1.406	3.504	10.124	41.639	86.044	100.015	136.695	2244.159
-74.85	1.411	3.534	10.438	41.169	86.594	98.431	136.990	2244.159
-74.75	1.434	3.619	9.412	42.339	83.331	99.818	137.727	2244.159
-74.65	1.411	3.449	10.207	42.683	85.904	100.463	134.278	2244.159
-74.55	1.355	3.595	10.096	41.743	84.302	98.256	135.496	2244.159
-74.45	1.542	3.826	9.628	42.422	86.757	98.463	138.336	2244.159
-74.35	1.292	3.783	10.012	40.908	85.296	97.873	137.442	2244.159
-74.25	1.530	3.637	9.886	42.004	85.448	100.026	135.565	2244.159
-74.15	1.332	3.607	9.803	41.670	86.290	102.440	138.336	2244.159
-74.05	1.411	3.650	9.977	41.284	87.167	99.840	137.717	2244.159
-73.95	1.156	3.619	9.866	41.869	84.512	99.490	137.501	2244.159
-73.85	1.372	3.625	9.942	39.853	85.319	99.206	137.333	2244.159
-73.75	1.468	3.802	9.782	41.597	83.846	100.681	138.689	2244.159
-73.65	1.292	4.094	9.224	42.046	85.494	99.512	136.950	2244.159
-73.55	1.406	3.796	9.970	42.986	86.266	98.321	136.911	2244.159
-73.45	1.196	3.796	9.712	42.788	86.278	100.299	135.005	2244.159
-73.35	1.349	3.814	9.698	41.263	86.395	100.222	135.034	2244.159
-73.25	1.417	3.765	9.684	42.318	86.079	99.621	134.848	2244.159
-73.15	1.326	3.808	9.370	42.380	85.740	99.283	138.719	2244.159
-73.05	1.349	3.710	9.538	42.746	84.945	100.135	136.341	2244.159
-72.95	1.292	4.002	9.726	42.004	84.781	100.146	134.975	2244.159

Midpoint	103	30	10	3	1	0.34	0.1	Volume
-72.85	1.434	3.996	9.838	40.427	86.395	99.468	135.771	2244.159
-72.75	1.479	3.929	10.005	42.829	83.764	101.337	134.504	2244.159
-72.65	1.474	3.729	9.866	41.440	85.915	99.163	134.661	2244.159
-72.55	1.474	3.704	9.754	41.848	86.138	99.305	131.644	2244.159
-72.45	1.491	3.558	9.977	41.795	85.787	99.436	137.255	2244.159
-72.35	1.332	3.601	9.405	41.701	85.261	97.294	134.514	2244.159
-72.25	1.355	3.686	10.103	42.151	84.079	100.878	135.260	2244.159
-72.15	1.389	3.856	9.384	41.681	83.717	99.938	134.916	2244.159
-72.05	1.400	3.631	9.663	40.741	85.939	100.943	137.324	2244.159
-71.95	1.394	3.856	10.173	40.730	86.371	100.441	136.606	2244.159
-71.85	1.383	3.668	9.531	42.004	85.389	99.840	137.697	2244.159
-71.75	1.440	3.765	9.684	42.339	85.436	100.026	136.616	2244.159
-71.65	1.423	3.729	9.845	42.234	86.453	100.179	136.940	2244.159
-71.55	1.258	3.881	10.005	41.639	87.494	100.856	135.152	2244.159
-71.45	1.366	3.990	9.649	42.861	84.723	100.572	134.346	2244.159
-71.35	1.207	3.862	9.349	41.764	84.863	99.720	136.262	2244.159
-71.25	1.485	3.686	9.454	41.701	84.115	98.977	138.444	2244.159
-71.15	1.355	3.662	9.775	41.816	85.775	99.567	135.820	2244.159
-71.05	1.479	3.881	10.061	41.179	85.553	100.124	135.378	2244.159
-70.95	1.303	3.674	9.914	41.775	87.798	97.753	139.308	2244.159
-70.85	1.320	3.905	9.949	42.161	85.284	98.321	135.614	2244.159
-70.75	1.247	3.662	9.935	42.715	84.652	98.398	134.278	2244.159
-70.65	1.343	3.643	9.886	42.255	86.348	101.173	136.400	2244.159
-70.55	1.496	3.650	9.531	41.910	86.500	98.584	135.712	2244.159
-70.45	1.411	3.966	10.193	41.252	86.056	98.671	135.663	2244.159
-70.35	1.485	3.680	9.852	43.184	86.816	99.501	136.646	2244.159
-70.25	1.474	3.564	9.789	41.440	85.518	100.211	137.373	2244.159
-70.15	1.468	3.929	9.817	41.848	86.219	101.118	135.575	2244.159
-70.05	1.372	3.704	9.810	42.151	84.711	100.332	134.396	2244.159
-69.95	1.417	4.014	10.291	41.399	85.892	97.961	136.567	2244.159
-69.85	1.224	3.735	10.200	42.286	84.185	99.862	135.869	2244.159
-69.75	1.502	3.747	9.761	41.472	86.453	99.545	134.101	2244.159
-69.65	1.417	3.960	9.691	42.339	85.483	99.195	138.178	2244.159
-69.55	1.355	3.674	9.949	42.621	84.524	99.632	134.720	2244.159
-69.45	1.320	3.850	10.145	41.879	86.360	99.709	137.491	2244.159
-69.35	1.315	3.625	9.740	42.401	85.073	101.632	133.826	2244.159
-69.25	1.264	3.680	9.782	41.513	84.442	97.633	136.616	2244.159
-69.15	1.406	3.850	9.712	42.004	85.974	98.595	136.479	2244.159
-69.05	1.496	3.418	9.461	42.119	84.921	99.643	134.985	2244.159
-68.95	1.462	3.455	9.677	42.683	86.243	97.415	135.643	2244.159
-68.85	1.320	3.759	9.845	43.184	84.348	98.562	136.380	2244.159
-68.75	1.275	3.692	9.377	41.179	85.775	100.506	134.671	2244.159
-68.65	1.258	3.692	9.963	41.743	84.875	98.955	135.585	2244.159
-68.55	1.564	3.747	9.789	41.096	85.459	97.742	136.302	2244.159
-68.45	1.360	3.595	9.070	41.900	84.688	99.611	136.980	2244.159

Midpoint	103	30	10	3	1	0.34	0.1	Volume
-68.35	1.406	3.923	9.859	40.939	83.226	101.227	137.029	2244.159
-68.25	1.326	3.686	10.110	41.503	85.681	99.326	133.914	2244.159
-68.15	1.394	4.051	9.656	41.639	85.272	100.637	136.872	2244.159
-68.05	1.355	3.534	9.649	41.837	87.436	101.227	134.946	2244.159
-67.95	1.286	3.589	9.754	42.276	84.898	100.244	135.722	2244.159
-67.85	1.615	3.838	10.040	41.096	85.190	96.650	137.039	2244.159
-67.75	1.343	3.954	10.005	41.367	87.073	101.271	134.455	2244.159
-67.65	1.468	3.826	9.817	41.242	85.448	99.337	139.014	2244.159
-67.55	1.337	3.796	9.810	41.357	84.489	99.873	133.256	2244.159
-67.45	1.349	3.844	9.517	40.438	85.225	99.960	135.388	2244.159
-67.35	1.519	3.741	9.635	42.140	85.576	100.583	137.216	2244.159
-67.25	1.411	3.698	9.300	40.970	84.828	99.174	136.587	2244.159
-67.15	1.332	3.747	9.517	42.130	86.266	99.676	136.764	2244.159
-67.05	1.315	3.838	9.663	42.976	85.717	98.715	135.250	2244.159
-66.95	1.337	3.595	9.412	41.848	83.202	98.715	137.903	2244.159
-66.85	1.355	3.753	9.293	40.709	87.354	100.484	136.990	2244.159
-66.75	1.303	3.741	10.054	40.856	86.313	99.294	135.869	2244.159
-66.65	1.547	3.796	9.656	41.096	84.921	100.594	133.953	2244.159
-66.55	1.326	3.704	9.900	42.098	84.664	99.337	136.557	2244.159
-66.45	1.292	3.820	9.789	40.950	85.880	99.152	136.410	2244.159
-66.35	1.491	3.656	9.782	42.808	84.395	98.420	137.471	2244.159
-66.25	1.411	3.777	9.733	41.273	86.570	100.594	134.504	2244.159
-66.15	1.162	3.577	9.628	41.388	85.506	97.404	134.965	2244.159
-66.05	1.383	3.869	9.824	40.991	85.950	99.938	137.727	2244.159
-65.95	1.264	3.856	9.635	41.221	86.044	100.310	138.090	2244.159
-65.85	1.303	3.741	9.559	41.691	85.225	98.780	133.727	2244.159
-65.75	1.337	3.789	9.740	41.482	85.986	99.305	134.897	2244.159
-65.65	1.383	3.564	9.573	41.983	85.179	98.278	137.933	2244.159
-65.55	1.309	3.656	10.110	40.835	84.185	99.709	135.142	2244.159
-65.45	1.383	3.802	10.159	41.430	84.793	99.163	136.970	2244.159
-65.35	1.513	3.485	9.928	41.315	84.758	100.495	135.752	2244.159
-65.25	1.372	3.802	9.935	42.307	85.822	98.081	134.641	2244.159
-65.15	1.389	3.595	10.382	41.211	85.997	99.250	136.449	2244.159
-65.05	1.326	4.063	9.859	40.553	86.898	99.873	136.587	2244.159
-64.95	1.406	3.656	9.642	41.816	84.044	100.932	134.101	2244.159
-64.85	1.468	3.625	9.677	42.328	81.752	100.626	137.412	2244.159
-64.75	1.519	3.546	9.656	41.346	84.372	99.305	137.952	2244.159
-64.65	1.406	3.662	9.949	41.085	86.629	98.507	135.860	2244.159
-64.55	1.400	3.838	9.573	39.696	83.986	100.233	138.758	2244.159
-64.45	1.394	3.753	9.489	42.276	85.728	99.720	133.049	2244.159
-64.35	1.269	3.881	9.956	42.589	85.600	98.354	136.488	2244.159
-64.25	1.423	3.710	9.754	40.072	86.278	100.889	135.250	2244.159
-64.15	1.400	3.899	9.538	41.388	84.348	99.654	134.062	2244.159
-64.05	1.201	3.796	9.545	41.837	84.781	100.102	135.103	2244.159
-63.95	1.468	3.637	9.349	40.177	86.652	102.287	137.894	2244.159

Midpoint	103	30	10	3	1	0.34	0.1	Volume
-63.85	1.218	3.583	9.712	40.835	84.313	99.086	135.339	2244.159
-63.75	1.349	3.698	9.998	40.970	86.348	100.266	135.693	2244.159
-63.65	1.479	3.941	9.614	40.469	84.933	97.240	135.752	2244.159
-63.55	1.286	3.723	9.747	40.845	87.494	100.911	136.538	2244.159
-63.45	1.377	3.485	9.866	42.004	88.126	98.747	134.258	2244.159
-63.35	1.320	3.753	9.286	41.085	87.342	101.118	136.223	2244.159
-63.25	1.417	3.832	10.082	41.743	87.132	100.026	136.262	2244.159
-63.15	1.372	3.765	9.831	41.524	85.190	100.976	135.732	2244.159
-63.05	1.332	3.613	9.782	42.297	86.582	99.305	133.953	2244.159
-62.95	1.332	4.014	9.691	41.451	86.044	98.114	135.113	2244.159
-62.85	1.394	3.601	9.956	42.349	85.284	97.950	135.869	2244.159
-62.75	1.496	3.637	9.740	41.983	84.921	97.764	136.685	2244.159
-62.65	1.230	4.039	9.761	41.399	85.354	98.911	133.295	2244.159
-62.55	1.468	3.698	9.740	42.046	86.734	100.211	135.683	2244.159
-62.45	1.360	3.862	10.040	41.806	87.003	102.353	136.656	2244.159
-62.35	1.519	3.887	9.754	41.973	83.530	100.091	136.980	2244.159
-62.25	1.411	3.735	9.998	41.691	85.436	99.042	136.557	2244.159
-62.15	1.360	3.710	9.761	42.213	86.032	99.578	136.233	2244.159
-62.05	1.366	3.650	9.559	40.344	85.927	98.431	136.302	2244.159
-61.95	1.326	3.680	9.677	41.357	84.500	97.841	137.854	2244.159
-61.85	1.434	3.692	9.838	42.767	86.757	99.763	135.781	2244.159
-61.75	1.440	4.179	9.433	42.506	84.185	98.179	135.634	2244.159
-61.65	1.394	3.540	9.859	40.104	84.547	97.721	135.860	2244.159
-61.55	1.269	3.838	9.789	41.701	82.489	99.829	137.756	2244.159
-61.45	1.496	3.941	10.180	41.524	84.582	101.173	136.331	2244.159
-61.35	1.474	3.881	9.782	40.908	85.225	100.419	136.695	2244.159
-61.25	1.406	3.710	10.326	41.232	84.898	99.905	136.243	2244.159
-61.15	1.468	4.087	9.182	42.882	85.155	100.725	133.767	2244.159
-61.05	1.451	3.552	10.193	42.370	85.307	98.737	137.402	2244.159
-60.95	1.468	3.759	9.426	40.386	84.863	100.386	139.092	2244.159
-60.85	1.343	3.723	9.468	41.607	86.851	97.426	137.628	2244.159
-60.75	1.400	3.838	9.796	41.534	84.746	100.998	134.356	2244.159
-60.65	1.264	3.680	9.482	41.973	88.278	99.676	136.547	2244.159
-60.55	1.281	3.686	9.803	41.848	85.483	98.791	134.130	2244.159
-60.45	1.423	3.887	10.040	41.952	86.149	100.528	137.825	2244.159
-60.35	1.615	3.729	9.607	41.325	85.810	100.463	136.086	2244.159
-60.25	1.428	3.473	9.768	41.848	83.495	98.474	136.931	2244.159
-60.15	1.428	3.777	9.747	40.741	84.430	100.550	134.651	2244.159
-60.05	1.332	3.826	9.984	42.506	84.804	100.419	136.803	2244.159
-59.95	1.281	3.595	9.956	40.166	85.062	99.130	135.958	2244.159
-59.85	1.377	3.875	9.740	41.430	85.670	99.272	136.980	2244.159
-59.75	1.230	3.741	9.510	41.012	84.758	97.884	136.724	2244.159
-59.65	1.423	3.954	10.089	42.819	86.629	100.812	136.557	2244.159
-59.55	1.496	3.850	9.984	41.284	83.600	99.294	138.277	2244.159
-59.45	1.462	3.729	9.775	41.701	85.459	99.392	136.636	2244.159

Midpoint	103	30	10	3	1	0.34	0.1	Volume
-59.35	1.326	3.637	9.796	41.545	84.407	102.036	137.353	2244.159
-59.25	1.581	3.710	10.173	41.357	84.769	99.556	136.184	2244.159
-59.15	1.326	3.747	9.649	40.563	84.804	98.835	135.889	2244.159
-59.05	1.355	3.540	10.312	41.305	85.377	99.119	135.437	2244.159
-58.95	1.309	3.856	9.510	42.077	85.728	100.637	133.963	2244.159
-58.85	1.326	3.856	9.893	42.840	85.576	100.463	137.058	2244.159
-58.75	1.400	3.729	9.684	41.649	84.945	98.081	136.390	2244.159
-58.65	1.275	3.704	10.019	42.370	83.378	99.174	135.830	2244.159
-58.55	1.428	3.844	9.768	41.910	84.383	98.944	135.722	2244.159
-58.45	1.366	3.832	9.740	41.639	85.904	98.638	136.990	2244.159
-58.35	1.366	3.522	9.656	41.242	85.927	97.513	135.938	2244.159
-58.25	1.394	3.929	9.984	41.597	85.015	99.709	136.439	2244.159
-58.15	1.366	3.850	10.061	40.626	83.565	99.294	137.589	2244.159
-58.05	1.309	3.753	9.670	40.887	85.237	99.895	134.818	2244.159
-57.95	1.491	3.753	9.782	42.130	84.278	100.200	136.115	2244.159
-57.85	1.372	3.796	9.314	41.994	85.740	100.004	134.543	2244.159
-57.75	1.417	3.729	10.207	41.952	83.927	99.075	134.897	2244.159
-57.65	1.377	3.668	9.977	42.161	87.319	98.933	136.076	2244.159
-57.55	1.269	3.881	9.873	41.963	87.482	99.643	138.011	2244.159
-57.45	1.332	3.351	9.691	41.754	85.798	99.818	136.017	2244.159
-57.35	1.241	3.735	9.607	41.942	86.909	100.244	136.056	2244.159
-57.25	1.326	3.710	9.866	41.346	86.991	98.649	135.447	2244.159
-57.15	1.462	3.935	10.089	41.023	85.600	98.715	137.776	2244.159
-57.05	1.457	4.021	9.859	42.192	84.091	99.676	135.201	2244.159
-56.95	1.400	3.826	10.089	42.182	84.079	99.578	135.958	2244.159
-56.85	1.315	3.686	9.342	42.412	83.834	98.879	137.913	2244.159
-56.75	1.355	3.881	9.398	42.015	85.483	98.857	136.164	2244.159
-56.65	1.281	3.960	9.552	41.921	85.869	99.840	136.341	2244.159
-56.55	1.423	3.905	10.026	42.516	84.641	99.272	139.299	2244.159
-56.45	1.468	3.595	9.684	40.344	85.342	99.742	136.115	2244.159
-56.35	1.400	3.796	9.768	42.088	85.413	100.430	135.270	2244.159
-56.25	1.400	3.753	10.131	40.292	84.992	99.414	137.471	2244.159
-56.15	1.394	3.528	9.475	41.054	84.664	98.955	135.093	2244.159
-56.05	1.377	4.081	9.391	41.973	83.389	97.327	136.233	2244.159
-55.95	1.445	3.862	9.649	42.537	85.787	98.616	135.634	2244.159
-55.85	1.496	3.899	9.719	41.461	84.746	101.063	132.411	2244.159
-55.75	1.406	3.789	9.607	41.378	85.857	97.305	136.184	2244.159
-55.65	1.184	3.510	9.761	40.981	85.646	97.633	136.115	2244.159
-55.55	1.423	3.595	9.873	41.325	85.600	98.758	134.523	2244.159
-55.45	1.434	3.881	10.019	41.994	84.992	100.976	136.842	2244.159
-55.35	1.332	3.759	9.914	41.002	84.816	99.305	133.246	2244.159
-55.25	1.423	3.899	9.691	41.555	85.635	100.102	134.327	2244.159
-55.15	1.360	3.777	9.649	41.106	87.529	99.731	135.997	2244.159
-55.05	1.440	3.875	9.719	42.537	86.301	101.063	136.665	2244.159
-54.95	1.196	3.704	9.789	40.835	85.962	99.949	138.355	2244.159

Midpoint	103	30	10	3	1	0.34	0.1	Volume
-54.85	1.423	3.692	9.817	42.036	84.419	100.157	134.455	2244.159
-54.75	1.400	3.546	10.040	40.856	83.471	98.179	135.614	2244.159
-54.65	1.326	3.650	9.586	41.576	85.319	98.605	135.005	2244.159
-54.55	1.343	3.710	9.796	42.265	85.553	98.136	134.700	2244.159
-54.45	1.360	3.570	9.775	42.098	85.284	102.189	133.747	2244.159
-54.35	1.377	3.789	9.866	42.673	84.769	100.310	136.960	2244.159
-54.25	1.332	3.692	9.266	41.002	84.231	99.381	136.656	2244.159
-54.15	1.258	3.777	9.517	41.472	85.085	99.534	136.832	2244.159
-54.05	1.434	3.674	9.489	40.960	82.852	99.763	136.852	2244.159
-53.95	1.326	3.887	9.782	42.234	86.313	99.971	135.732	2244.159
-53.85	1.366	3.978	9.670	41.889	85.822	97.764	136.204	2244.159
-53.75	1.298	4.057	9.677	42.203	85.015	98.496	137.559	2244.159
-53.65	1.400	3.662	9.733	41.294	86.254	99.501	134.769	2244.159
-53.55	1.377	3.850	9.579	41.221	86.617	98.911	137.697	2244.159
-53.45	1.417	3.777	9.579	41.054	83.752	99.305	136.204	2244.159
-53.35	1.479	3.759	9.761	41.942	86.383	98.660	134.602	2244.159
-53.25	1.264	3.796	9.684	41.242	84.559	99.654	137.029	2244.159
-53.15	1.355	3.808	9.503	42.412	84.454	99.130	134.612	2244.159
-53.05	1.303	3.759	9.761	42.234	84.676	99.742	134.956	2244.159
-52.95	1.411	3.540	9.684	40.166	84.021	100.725	137.265	2244.159
-52.85	1.423	3.723	9.803	43.122	86.594	101.042	134.916	2244.159
-52.75	1.394	3.534	9.335	41.044	84.524	99.326	136.253	2244.159
-52.65	1.360	3.716	9.405	40.741	87.354	99.348	136.793	2244.159
-52.55	1.258	3.680	10.124	40.950	83.331	97.906	138.454	2244.159
-52.45	1.275	3.704	9.859	41.012	86.407	98.846	134.936	2244.159
-52.35	1.491	3.741	9.677	41.106	86.816	98.922	136.282	2244.159
-52.25	1.337	3.528	9.419	39.989	85.342	99.425	135.447	2244.159
-52.15	1.406	3.826	9.524	39.916	84.267	99.960	137.628	2244.159
-52.05	1.349	3.783	9.977	41.430	84.734	100.353	135.722	2244.159
-51.95	1.286	3.619	9.852	41.346	82.922	98.791	136.449	2244.159
-51.85	1.474	3.522	9.161	41.357	85.541	97.699	134.504	2244.159
-51.75	1.389	3.881	9.733	41.963	85.225	99.643	134.160	2244.159
-51.65	1.235	3.954	9.733	41.012	85.132	97.218	135.270	2244.159
-51.55	1.474	3.832	9.726	42.631	86.559	101.850	136.046	2244.159
-51.45	1.366	3.735	9.873	42.171	84.115	100.943	137.333	2244.159
-51.35	1.252	3.875	10.054	40.970	85.775	99.676	136.469	2244.159
-51.25	1.372	3.802	9.607	41.221	85.565	98.256	136.547	2244.159
-51.15	1.315	3.595	9.712	42.495	85.459	98.518	136.872	2244.159
-51.05	1.315	3.461	9.579	42.976	85.413	99.785	135.388	2244.159
-50.95	1.377	3.869	9.719	41.399	88.032	99.534	135.987	2244.159
-50.85	1.423	3.789	9.677	41.461	84.383	99.152	135.221	2244.159
-50.75	1.451	3.856	9.761	42.224	85.413	99.206	133.118	2244.159
-50.65	1.315	3.777	9.670	41.681	85.284	102.298	136.184	2244.159
-50.55	1.286	3.643	9.747	41.085	83.916	98.846	136.803	2244.159
-50.45	1.355	3.583	9.838	41.649	87.319	100.605	138.070	2244.159

Midpoint	103	30	10	3	1	0.34	0.1	Volume
-50.35	1.298	3.577	9.886	41.994	85.670	98.693	137.206	2244.159
-50.25	1.337	3.759	9.517	40.375	85.237	99.021	137.874	2244.159
-50.15	1.389	3.723	9.977	42.882	84.465	98.671	136.567	2244.159
-50.05	1.394	3.759	9.440	41.221	85.869	100.233	132.195	2244.159
-49.95	1.372	3.893	9.963	41.211	86.839	98.321	137.392	2244.159
-49.85	1.349	3.589	9.405	43.090	85.576	97.120	135.948	2244.159
-49.75	1.337	3.777	9.314	42.537	85.015	100.190	137.903	2244.159
-49.65	1.411	3.844	9.307	42.067	84.992	99.774	135.791	2244.159
-49.55	1.400	3.692	9.970	41.837	88.348	98.671	136.931	2244.159
-49.45	1.377	3.802	9.949	41.200	85.097	100.211	137.216	2244.159
-49.35	1.400	3.777	9.677	41.921	86.325	99.490	135.555	2244.159
-49.25	1.457	3.558	9.607	42.130	83.389	98.977	135.368	2244.159
-49.15	1.445	3.753	9.880	41.378	84.828	99.075	134.975	2244.159
-49.05	1.451	3.668	9.977	40.897	85.681	99.195	134.612	2244.159
-48.95	1.320	3.631	10.068	42.265	86.769	99.862	136.862	2244.159
-48.85	1.258	3.540	9.768	42.151	86.067	98.944	137.117	2244.159
-48.75	1.247	3.753	9.998	41.054	86.243	99.709	135.113	2244.159
-48.65	1.201	3.862	9.642	41.367	85.027	98.267	135.958	2244.159
-48.55	1.491	3.753	9.182	40.981	84.652	97.010	135.260	2244.159
-48.45	1.462	3.899	9.566	40.521	85.179	98.638	135.054	2244.159
-48.35	1.474	3.570	9.921	41.973	86.664	100.932	134.985	2244.159
-48.25	1.525	3.747	9.286	42.224	87.459	100.506	134.130	2244.159
-48.15	1.360	3.558	9.405	41.325	86.746	99.654	138.031	2244.159
-48.05	1.298	3.881	10.089	41.618	84.606	99.501	135.594	2244.159
-47.95	1.377	3.777	9.775	42.224	85.658	99.501	136.626	2244.159
-47.85	1.485	3.966	9.782	42.203	85.167	101.621	135.938	2244.159
-47.75	1.406	3.716	9.677	43.132	86.418	100.342	133.443	2244.159
-47.65	1.269	3.741	10.249	41.419	86.629	100.430	135.270	2244.159
-47.55	1.445	3.802	9.545	42.276	84.477	98.016	136.420	2244.159
-47.45	1.275	3.771	10.068	42.171	85.354	101.348	135.388	2244.159
-47.35	1.252	3.510	9.991	42.297	85.869	98.922	136.469	2244.159
-47.25	1.298	3.729	9.677	42.255	86.909	99.523	135.830	2244.159
-47.15	1.485	3.558	9.545	41.002	84.910	100.026	135.702	2244.159
-47.05	1.366	3.656	9.984	41.273	87.295	99.064	133.777	2244.159
-46.95	1.298	3.522	9.880	41.472	85.787	99.021	135.643	2244.159
-46.85	1.496	3.990	9.719	41.482	87.120	98.081	138.454	2244.159
-46.75	1.496	3.710	10.075	41.503	85.857	101.326	135.594	2244.159
-46.65	1.355	3.826	9.614	40.960	85.974	98.081	135.909	2244.159
-46.55	1.508	3.850	9.768	40.490	85.927	99.392	135.869	2244.159
-46.45	1.309	3.698	9.928	41.670	86.523	99.337	138.021	2244.159
-46.35	1.457	3.674	9.719	40.845	86.944	99.556	138.110	2244.159
-46.25	1.258	3.613	9.461	41.900	86.021	100.102	138.090	2244.159
-46.15	1.372	3.850	9.984	41.482	86.477	99.938	137.599	2244.159
-46.05	1.445	3.735	10.354	42.057	87.927	100.965	135.162	2244.159
-45.95	1.292	3.656	10.312	41.440	84.512	98.442	136.036	2244.159

Midpoint	103	30	10	3	1	0.34	0.1	Volume
-45.85	1.468	3.552	9.698	41.618	86.886	100.353	134.268	2244.159
-45.75	1.462	3.844	9.873	41.754	86.465	98.092	136.253	2244.159
-45.65	1.355	3.491	9.768	42.788	84.068	101.009	135.408	2244.159
-45.55	1.264	3.558	10.242	42.777	85.506	100.135	136.832	2244.159
-45.45	1.235	3.497	9.245	41.158	84.454	96.705	136.469	2244.159
-45.35	1.145	3.577	9.391	40.438	84.044	99.425	136.950	2244.159
-45.25	1.264	3.935	10.012	41.973	87.845	100.539	133.708	2244.159
-45.15	1.485	4.045	9.586	41.816	85.681	99.097	133.885	2244.159
-45.05	1.513	4.014	9.733	43.247	86.067	100.058	137.019	2244.159
-44.95	1.485	3.643	9.768	41.534	86.371	98.813	136.606	2244.159
-44.85	1.525	3.954	10.103	42.192	84.033	99.851	136.351	2244.159
-44.75	1.440	3.844	9.468	40.500	86.348	97.513	136.803	2244.159
-44.65	1.593	3.650	9.880	42.506	84.267	99.611	135.712	2244.159
-44.55	1.366	4.033	9.775	41.545	84.699	99.862	136.174	2244.159
-44.45	1.440	3.431	10.305	42.976	83.752	99.174	136.793	2244.159
-44.35	1.417	3.771	9.810	40.824	86.325	100.146	139.544	2244.159
-44.25	1.423	3.704	9.956	42.662	87.553	99.305	137.589	2244.159
-44.15	1.337	3.838	9.300	43.007	86.851	100.681	135.437	2244.159
-44.05	1.145	3.838	10.214	42.464	85.576	100.987	136.538	2244.159
-43.95	1.536	4.094	9.998	40.970	85.027	99.272	135.074	2244.159
-43.85	1.184	3.765	9.998	41.158	86.114	99.960	132.617	2244.159
-43.75	1.252	3.948	9.782	42.453	86.629	99.326	137.844	2244.159
-43.65	1.281	3.601	9.356	41.649	85.225	98.212	137.648	2244.159
-43.55	1.440	3.680	9.586	42.067	86.266	101.905	137.874	2244.159
-43.45	1.281	3.966	9.907	41.002	87.810	100.692	136.803	2244.159
-43.35	1.332	3.881	9.586	41.942	86.184	100.889	134.848	2244.159
-43.25	1.372	3.613	9.621	42.151	86.102	98.485	136.469	2244.159
-43.15	1.349	3.783	9.384	41.983	85.986	99.501	138.444	2244.159
-43.05	1.320	3.668	9.747	41.921	84.021	99.381	137.461	2244.159
-42.95	1.372	3.607	10.249	41.566	86.079	100.703	135.300	2244.159
-42.85	1.372	3.850	9.259	42.433	86.383	98.573	135.506	2244.159
-42.75	1.173	3.856	9.796	41.106	84.500	102.200	138.473	2244.159
-42.65	1.474	3.668	10.180	41.482	83.998	101.282	139.132	2244.159
-42.55	1.491	3.802	9.684	40.782	86.874	99.348	135.899	2244.159
-42.45	1.383	3.449	10.207	41.879	88.196	101.205	134.818	2244.159
-42.35	1.372	3.710	10.173	42.600	83.577	99.632	138.326	2244.159
-42.25	1.332	3.923	9.405	42.600	85.997	99.042	135.309	2244.159
-42.15	1.281	3.789	9.482	41.273	85.565	100.965	135.467	2244.159
-42.05	1.428	3.534	9.628	42.067	86.126	98.846	135.427	2244.159
-41.95	1.406	3.856	9.440	40.887	85.950	100.211	135.427	2244.159
-41.85	1.542	3.437	9.600	39.581	86.699	100.845	135.683	2244.159
-41.75	1.320	4.014	10.061	42.996	86.664	101.085	137.383	2244.159
-41.65	1.468	3.716	9.286	41.127	85.927	98.638	136.547	2244.159
-41.55	1.252	3.777	9.545	41.472	83.389	101.566	136.587	2244.159
-41.45	1.320	3.637	9.621	42.412	85.588	100.921	137.461	2244.159

Midpoint	103	30	10	3	1	0.34	0.1	Volume
-41.35	1.496	3.869	9.880	41.785	87.389	99.982	135.378	2244.159
-41.25	1.366	3.838	9.670	41.305	86.056	101.839	134.435	2244.159
-41.15	1.428	3.668	9.859	42.297	87.915	98.769	135.810	2244.159
-41.05	1.372	3.869	9.496	42.171	86.664	100.069	140.281	2244.159
-40.95	1.326	3.735	10.389	41.252	83.635	100.069	138.139	2244.159
-40.85	1.445	3.650	10.033	43.362	85.366	102.648	137.501	2244.159
-40.75	1.479	3.680	9.852	42.036	85.225	101.632	136.842	2244.159
-40.65	1.275	3.881	9.761	42.098	87.365	98.485	139.534	2244.159
-40.55	1.258	3.875	9.510	42.015	86.184	102.254	135.319	2244.159
-40.45	1.207	3.674	9.761	41.816	86.196	100.069	135.201	2244.159
-40.35	1.451	3.692	9.998	41.440	85.038	101.304	136.351	2244.159
-40.25	1.383	3.826	9.670	42.171	86.173	100.626	138.090	2244.159
-40.15	1.508	3.747	10.298	41.743	84.886	100.430	136.773	2244.159
-40.05	1.320	3.607	9.482	42.286	85.179	99.621	135.329	2244.159
-39.95	1.179	3.759	9.475	41.399	88.184	100.190	137.952	2244.159
-39.85	1.530	3.802	9.831	42.527	84.500	99.075	135.673	2244.159
-39.75	1.315	3.753	9.566	42.057	85.740	98.573	136.400	2244.159
-39.65	1.417	3.929	9.866	42.819	84.945	100.736	137.304	2244.159
-39.55	1.377	3.802	9.824	42.057	89.330	100.987	137.952	2244.159
-39.45	1.355	3.814	10.200	42.015	85.939	100.047	139.417	2244.159
-39.35	1.457	3.881	9.928	41.566	85.775	101.074	137.294	2244.159
-39.25	1.326	3.893	9.817	42.600	86.816	100.015	137.049	2244.159
-39.15	1.332	3.960	9.775	42.286	87.003	101.020	138.562	2244.159
-39.05	1.462	3.917	9.949	41.764	86.032	100.037	138.178	2244.159
-38.95	1.406	3.893	10.187	41.576	85.892	98.999	137.117	2244.159
-38.85	1.355	3.887	9.552	40.553	87.167	102.079	137.540	2244.159
-38.75	1.218	3.753	9.538	42.245	85.857	100.943	137.196	2244.159
-38.65	1.496	3.759	9.552	42.328	86.196	100.430	135.427	2244.159
-38.55	1.360	3.820	9.663	42.600	87.167	100.812	138.346	2244.159
-38.45	1.462	3.862	9.698	42.286	85.448	99.807	136.705	2244.159
-38.35	1.462	3.650	9.768	41.451	84.629	101.031	135.634	2244.159
-38.25	1.315	3.510	9.859	42.339	85.073	99.709	135.250	2244.159
-38.15	1.508	3.850	10.047	42.088	87.634	100.037	139.092	2244.159
-38.05	1.417	3.808	10.207	41.712	87.295	100.463	138.346	2244.159
-37.95	1.292	3.723	9.307	42.234	87.365	100.550	137.501	2244.159
-37.85	1.372	3.856	10.019	43.477	86.313	99.905	138.542	2244.159
-37.75	1.326	3.577	9.377	41.023	87.833	101.184	139.495	2244.159
-37.65	1.309	3.856	10.375	42.151	86.430	101.850	137.766	2244.159
-37.55	1.377	4.002	9.914	42.328	86.617	100.353	138.051	2244.159
-37.45	1.355	3.564	9.866	42.203	88.523	101.326	137.216	2244.159
-37.35	1.468	3.589	9.531	41.910	87.155	101.096	138.002	2244.159
-37.25	1.224	3.479	10.124	41.722	86.243	101.435	135.722	2244.159
-37.15	1.428	3.826	9.991	43.007	87.202	99.905	137.471	2244.159
-37.05	1.303	3.716	9.266	42.203	87.377	99.785	138.002	2244.159
-36.95	1.235	4.027	10.033	42.756	85.529	102.462	136.204	2244.159

Midpoint	103	30	10	3	1	0.34	0.1	Volume
-36.85	1.292	3.595	9.768	41.179	85.997	99.938	138.306	2244.159
-36.75	1.281	4.221	10.493	41.994	86.792	101.894	139.819	2244.159
-36.65	1.355	3.698	10.054	41.628	83.460	100.419	137.766	2244.159
-36.55	1.326	3.765	9.900	41.743	87.424	99.960	138.699	2244.159
-36.45	1.536	3.741	9.942	43.247	86.827	101.730	139.967	2244.159
-36.35	1.502	3.747	9.775	40.500	88.500	100.375	136.538	2244.159
-36.25	1.530	3.850	9.893	40.960	86.886	100.528	134.759	2244.159
-36.15	1.337	3.802	9.949	43.132	86.243	100.222	137.903	2244.159
-36.05	1.417	3.887	10.005	41.816	87.751	100.430	135.791	2244.159
-35.95	1.394	3.777	10.047	43.874	87.085	102.025	138.758	2244.159
-35.85	1.462	3.893	9.998	43.550	88.207	100.889	137.520	2244.159
-35.75	1.343	3.911	9.984	41.555	85.904	102.462	135.594	2244.159
-35.65	1.281	3.643	9.810	42.098	84.652	102.189	135.034	2244.159
-35.55	1.513	3.893	9.579	42.151	85.787	99.392	138.847	2244.159
-35.45	1.332	3.808	9.914	41.639	86.161	100.168	135.516	2244.159
-35.35	1.598	3.510	9.991	43.174	85.553	102.658	138.031	2244.159
-35.25	1.530	3.741	10.438	42.704	86.629	99.228	136.891	2244.159
-35.15	1.406	3.783	9.621	42.495	86.056	101.435	138.414	2244.159
-35.05	1.491	3.650	9.852	42.652	83.986	103.063	136.341	2244.159
-34.95	1.417	3.710	9.566	42.057	87.389	101.785	138.680	2244.159
-34.85	1.513	3.948	9.796	41.252	86.184	99.403	137.442	2244.159
-34.75	1.332	3.771	10.075	42.808	87.096	101.642	136.724	2244.159
-34.65	1.445	3.820	10.431	41.117	86.383	99.425	135.191	2244.159
-34.55	1.559	3.832	9.817	42.547	88.359	100.812	136.872	2244.159
-34.45	1.309	3.735	10.270	40.605	86.523	102.921	138.346	2244.159
-34.35	1.474	3.783	10.187	43.299	86.021	100.965	139.220	2244.159
-34.25	1.581	3.844	9.747	43.592	88.044	100.222	137.157	2244.159
-34.15	1.502	4.197	10.256	41.336	87.588	102.156	137.019	2244.159
-34.05	1.423	3.954	9.649	42.328	87.541	101.839	141.234	2244.159
-33.95	1.315	3.765	9.754	43.790	86.944	99.392	138.572	2244.159
-33.85	1.337	3.753	9.726	43.080	87.786	100.517	137.225	2244.159
-33.75	1.360	3.881	10.417	42.370	85.377	101.380	136.292	2244.159
-33.65	1.372	3.844	9.859	42.610	86.430	101.413	136.508	2244.159
-33.55	1.389	3.692	9.740	43.487	86.839	101.282	136.538	2244.159
-33.45	1.547	3.698	9.949	42.902	86.664	101.850	135.594	2244.159
-33.35	1.252	3.972	9.628	42.495	86.442	100.594	135.093	2244.159
-33.25	1.559	3.771	9.573	42.495	88.383	99.632	140.881	2244.159
-33.15	1.355	3.832	10.668	41.252	88.336	101.599	137.078	2244.159
-33.05	1.286	4.106	10.026	42.673	87.459	98.988	138.699	2244.159
-32.95	1.434	3.814	10.012	42.673	85.541	102.036	136.616	2244.159
-32.85	1.377	3.741	10.082	42.965	86.956	101.369	139.102	2244.159
-32.75	1.349	3.674	10.431	42.996	86.430	102.243	134.789	2244.159
-32.65	1.485	3.911	10.131	42.036	87.658	101.271	137.550	2244.159
-32.55	1.542	3.747	10.110	41.722	87.400	101.151	137.019	2244.159
-32.45	1.281	4.185	9.886	43.393	84.804	99.523	137.235	2244.159

Midpoint	103	30	10	3	1	0.34	0.1	Volume
-32.35	1.428	3.820	9.845	43.237	86.009	103.609	136.823	2244.159
-32.25	1.491	3.747	10.012	42.735	86.792	100.867	135.702	2244.159
-32.15	1.400	3.619	9.782	40.814	87.517	102.506	138.267	2244.159
-32.05	1.326	4.081	10.110	40.699	87.471	101.686	137.058	2244.159
-31.95	1.400	3.881	9.775	42.621	88.722	100.681	136.194	2244.159
-31.85	1.377	4.112	9.928	43.352	86.699	101.730	139.063	2244.159
-31.75	1.360	3.607	9.789	43.529	86.044	101.883	136.253	2244.159
-31.65	1.462	3.990	10.005	43.508	86.102	100.648	139.436	2244.159
-31.55	1.423	3.808	10.005	42.506	87.120	99.895	138.513	2244.159
-31.45	1.570	3.753	9.949	41.722	86.360	101.304	135.506	2244.159
-31.35	1.570	3.783	9.531	42.140	86.254	103.248	135.555	2244.159
-31.25	1.406	3.923	10.305	43.017	87.634	99.916	137.520	2244.159
-31.15	1.423	3.911	9.845	43.738	86.371	101.566	137.844	2244.159
-31.05	1.423	3.650	9.447	42.944	86.231	102.145	137.491	2244.159
-30.95	1.434	3.710	9.119	42.704	86.512	100.736	134.278	2244.159
-30.85	1.389	3.844	10.089	42.819	85.845	101.522	135.712	2244.159
-30.75	1.309	3.856	9.754	42.234	88.394	103.139	137.952	2244.159
-30.65	1.519	4.008	10.089	41.440	88.394	101.490	136.292	2244.159
-30.55	1.502	3.929	10.159	43.446	86.956	100.659	136.528	2244.159
-30.45	1.445	3.796	9.900	43.456	87.494	99.032	138.866	2244.159
-30.35	1.292	3.862	10.193	42.808	86.933	101.500	136.862	2244.159
-30.25	1.417	3.777	10.263	43.122	87.868	101.850	136.459	2244.159
-30.15	1.389	3.941	9.824	42.119	84.009	101.304	138.090	2244.159
-30.05	1.406	3.960	9.991	42.923	86.535	99.862	135.300	2244.159
-29.95	1.184	4.118	9.824	41.388	87.833	100.878	138.434	2244.159
-29.85	1.162	3.862	10.354	42.297	86.114	100.943	136.557	2244.159
-29.75	1.519	4.039	9.412	42.527	86.149	101.544	137.265	2244.159
-29.65	1.411	3.893	10.773	42.401	84.091	100.878	135.968	2244.159
-29.55	1.275	3.881	9.719	42.380	87.167	98.223	135.673	2244.159
-29.45	1.303	3.808	10.187	42.516	85.342	100.375	135.860	2244.159
-29.35	1.417	3.905	9.956	41.931	86.079	100.419	135.260	2244.159
-29.25	1.542	3.905	9.991	42.307	86.757	100.900	135.378	2244.159
-29.15	1.190	3.710	9.635	43.205	86.664	99.261	137.275	2244.159
-29.05	1.496	3.990	9.942	42.808	86.944	99.174	136.773	2244.159
-28.95	1.428	4.027	9.838	42.652	85.483	98.857	133.845	2244.159
-28.85	1.400	3.643	9.712	42.276	84.910	100.758	135.565	2244.159
-28.75	1.372	3.662	9.377	42.077	86.687	97.611	135.653	2244.159
-28.65	1.411	3.850	9.914	41.607	85.892	100.190	135.663	2244.159
-28.55	1.303	3.753	9.984	41.775	84.302	99.108	133.953	2244.159
-28.45	1.269	3.869	9.705	41.294	83.904	99.523	133.167	2244.159
-28.35	1.190	4.197	9.935	41.701	86.336	97.415	134.307	2244.159
-28.25	1.479	3.729	10.305	41.033	85.459	99.326	134.091	2244.159
-28.15	1.394	3.862	9.998	42.036	84.021	98.420	137.432	2244.159
-28.05	1.491	3.808	9.349	42.913	82.735	100.179	134.759	2244.159
-27.95	1.139	3.838	10.068	42.057	83.658	98.409	135.231	2244.159

Midpoint	103	30	10	3	1	0.34	0.1	Volume
-27.85	1.332	3.844	10.159	42.610	83.261	100.255	133.678	2244.159
-27.75	1.349	3.838	9.670	40.250	87.143	99.141	134.759	2244.159
-27.65	1.201	3.723	9.768	41.503	83.495	98.496	135.260	2244.159
-27.55	1.389	3.869	9.900	42.098	85.389	100.244	134.907	2244.159
-27.45	1.320	3.583	9.628	40.751	84.348	98.649	132.558	2244.159
-27.35	1.326	3.972	9.949	42.203	82.664	96.956	132.922	2244.159
-27.25	1.491	4.021	9.684	40.490	82.571	96.410	129.601	2244.159
-27.15	1.298	3.589	9.866	40.584	85.272	97.699	132.037	2244.159
-27.05	1.235	3.765	9.656	40.312	82.536	96.901	134.956	2244.159
-26.95	1.309	3.844	9.621	41.806	85.939	97.262	134.681	2244.159
-26.85	1.269	3.948	9.349	40.490	83.810	97.589	131.291	2244.159
-26.75	1.366	3.856	9.468	41.545	84.804	99.108	133.334	2244.159
-26.65	1.315	3.832	9.880	39.393	85.389	95.612	133.177	2244.159
-26.55	1.530	3.546	9.440	41.513	85.179	97.338	134.956	2244.159
-26.45	1.332	3.899	10.193	41.785	82.220	99.359	132.823	2244.159
-26.35	1.349	3.893	9.656	40.312	83.354	97.327	132.755	2244.159
-26.25	1.235	3.796	10.068	40.480	83.109	98.365	132.401	2244.159
-26.15	1.400	3.729	9.942	41.138	83.050	97.142	132.028	2244.159
-26.05	1.360	3.680	9.698	41.315	83.810	99.479	132.440	2244.159
-25.95	1.423	3.741	10.019	41.064	81.858	98.201	130.259	2244.159
-25.85	1.417	3.802	9.956	40.845	83.226	96.956	130.328	2244.159
-25.75	1.451	3.820	9.670	41.952	82.571	98.343	132.735	2244.159
-25.65	1.394	3.875	9.880	40.845	83.869	96.639	132.165	2244.159
-25.55	1.247	3.558	9.670	40.908	82.220	97.775	132.932	2244.159
-25.45	1.400	3.759	9.503	41.232	83.799	99.152	132.637	2244.159
-25.35	1.389	3.789	9.935	39.957	83.869	96.158	131.065	2244.159
-25.25	1.417	3.832	9.698	41.117	84.875	97.284	129.483	2244.159
-25.15	1.377	3.783	9.907	40.709	84.337	97.579	129.561	2244.159
-25.05	1.332	3.698	10.298	39.811	83.132	96.650	133.089	2244.159
-24.95	1.372	3.686	10.459	40.312	83.623	97.917	132.224	2244.159
-24.85	1.355	3.911	10.068	40.500	83.483	99.982	131.841	2244.159
-24.75	1.417	4.033	10.200	41.023	82.735	98.584	132.519	2244.159
-24.65	1.167	4.136	9.349	40.427	82.945	96.868	131.232	2244.159
-24.55	1.315	3.686	9.747	40.793	83.296	96.311	132.568	2244.159
-24.45	1.389	3.765	9.935	41.681	85.307	98.595	131.340	2244.159
-24.35	1.406	3.777	10.005	40.856	83.308	98.168	134.690	2244.159
-24.25	1.423	3.850	9.349	41.305	83.565	97.535	130.151	2244.159
-24.15	1.462	4.014	9.538	42.245	84.606	98.933	131.359	2244.159
-24.05	1.315	3.832	10.103	41.545	81.811	98.977	133.099	2244.159
-23.95	1.360	3.656	10.200	41.221	81.226	99.687	131.310	2244.159
-23.85	1.315	3.996	10.138	42.433	82.816	97.873	132.401	2244.159
-23.75	1.542	3.637	10.152	41.493	81.156	96.781	132.784	2244.159
-23.65	1.366	3.941	9.307	41.942	84.091	97.251	133.128	2244.159
-23.55	1.491	3.656	9.949	41.701	83.202	97.939	132.096	2244.159
-23.45	1.491	3.844	9.817	39.978	82.559	100.637	133.501	2244.159

Midpoint	103	30	10	3	1	0.34	0.1	Volume
-23.35	1.423	3.972	9.907	41.649	82.571	100.037	131.045	2244.159
-23.25	1.337	3.716	10.033	41.545	83.202	98.879	133.649	2244.159
-23.15	1.315	4.033	9.949	42.495	86.138	97.524	133.079	2244.159
-23.05	1.372	3.869	10.514	42.976	83.974	97.251	133.993	2244.159
-22.95	1.485	3.814	10.082	42.422	84.769	100.397	135.457	2244.159
-22.85	1.133	4.118	9.998	41.754	84.173	98.605	135.801	2244.159
-22.75	1.383	3.972	10.012	41.294	83.027	98.573	133.256	2244.159
-22.65	1.326	3.941	9.775	41.451	84.816	99.567	131.084	2244.159
-22.55	1.326	3.899	10.480	41.816	85.600	98.562	135.417	2244.159
-22.45	1.292	3.850	9.768	42.255	84.360	98.518	134.111	2244.159
-22.35	1.389	3.941	10.284	40.887	85.623	99.621	132.666	2244.159
-22.25	1.423	3.911	10.333	41.983	84.886	99.021	135.496	2244.159
-22.15	1.723	3.978	9.782	42.203	85.646	100.747	136.272	2244.159
-22.05	1.298	3.893	10.159	42.819	87.167	97.753	133.207	2244.159
-21.95	1.502	4.057	10.382	42.673	84.477	99.785	135.643	2244.159
-21.85	1.190	3.856	10.368	44.302	87.026	100.878	135.702	2244.159
-21.75	1.496	4.014	9.949	43.101	84.781	98.955	134.936	2244.159
-21.65	1.389	3.941	10.403	43.038	87.132	102.178	135.182	2244.159
-21.55	1.286	3.869	10.535	43.195	86.056	101.577	135.624	2244.159
-21.45	1.400	3.856	10.842	41.607	86.734	100.889	135.565	2244.159
-21.35	1.485	4.197	10.500	43.331	88.453	101.446	135.093	2244.159
-21.25	1.559	3.668	11.031	42.495	87.517	98.452	137.333	2244.159
-21.15	1.389	4.045	10.459	42.339	88.324	101.511	135.585	2244.159
-21.05	1.372	4.027	10.877	42.683	86.465	100.812	137.933	2244.159
-20.95	1.366	4.246	10.549	42.715	86.664	101.031	135.634	2244.159
-20.85	1.457	3.905	9.956	42.788	86.746	103.019	138.257	2244.159
-20.75	1.292	3.929	10.438	43.446	88.453	101.490	136.940	2244.159
-20.65	1.400	4.039	10.619	43.895	87.681	102.680	136.272	2244.159
-20.55	1.366	4.057	10.633	43.247	88.301	101.632	135.486	2244.159
-20.45	1.440	4.008	10.082	43.947	88.757	104.199	138.424	2244.159
-20.35	1.434	4.276	9.956	43.164	89.470	102.331	138.306	2244.159
-20.25	1.445	4.002	10.919	42.600	88.605	100.769	139.643	2244.159
-20.15	1.434	4.081	10.891	44.344	88.605	101.216	139.633	2244.159
-20.05	1.349	4.021	11.366	43.947	89.178	102.123	138.424	2244.159
-19.95	1.400	4.021	10.863	43.519	89.646	101.610	139.210	2244.159
-19.85	1.474	4.240	10.975	45.367	89.447	102.079	137.392	2244.159
-19.75	1.406	3.935	10.389	43.759	90.757	99.905	141.215	2244.159
-19.65	1.468	4.179	10.696	44.981	89.225	102.516	140.497	2244.159
-19.55	1.485	4.045	10.403	45.158	90.464	105.717	138.925	2244.159
-19.45	1.547	4.331	10.591	46.495	90.090	103.412	141.362	2244.159
-19.35	1.491	4.039	10.640	44.490	90.125	104.614	140.438	2244.159
-19.25	1.462	4.154	10.759	44.897	90.663	103.631	140.252	2244.159
-19.15	1.179	3.461	9.000	36.647	74.514	86.446	116.660	2244.159
-19.05	0.119	0.481	1.158	4.021	7.589	9.362	12.449	12229.377
-18.95	0.142	0.347	1.026	3.561	6.478	9.166	10.759	139.219

Midpoint	103	30	10	3	1	0.34	0.1	Volume
-18.85	0.102	0.426	1.019	3.499	6.151	8.215	10.631	128.897
-18.75	0.119	0.335	1.137	3.018	6.268	7.429	10.219	121.873
-18.65	0.215	0.292	1.061	3.457	6.081	8.303	9.668	116.271
-18.55	0.125	0.328	0.998	2.736	5.730	7.472	8.519	111.530
-18.45	0.074	0.316	0.942	3.039	5.356	7.527	9.305	107.382
-18.35	0.091	0.426	0.977	2.674	5.543	6.817	8.588	103.676
-18.25	0.102	0.365	0.809	2.799	5.613	6.730	8.548	100.316
-18.15	0.079	0.310	0.754	2.642	5.215	6.391	7.340	97.236
-18.05	0.102	0.255	0.991	2.527	4.865	6.588	7.300	94.390
-17.95	0.096	0.201	0.837	2.548	4.830	5.998	8.175	91.743
-17.85	0.096	0.292	0.754	2.559	4.561	5.856	7.752	89.267
-17.75	0.085	0.286	0.656	2.224	4.432	5.724	8.087	86.940
-17.65	0.074	0.231	0.788	2.444	4.771	6.271	7.202	84.746
-17.55	0.079	0.249	0.726	2.444	4.830	6.161	7.124	82.670
-17.45	0.062	0.225	0.677	2.339	4.093	5.910	7.232	80.700
-17.35	0.108	0.219	0.593	2.120	4.280	5.353	7.153	78.826
-17.25	0.074	0.365	0.677	2.266	4.140	5.375	6.406	77.040
-17.15	0.057	0.243	0.677	1.953	4.175	4.807	6.947	75.335
-17.05	0.057	0.225	0.677	1.817	3.871	4.894	6.190	73.703
-16.95	0.108	0.292	0.600	2.183	3.801	5.593	6.701	72.140
-16.85	0.074	0.189	0.607	2.016	3.988	4.905	6.387	70.640
-16.75	0.074	0.225	0.677	1.807	4.069	4.566	5.994	69.199
-16.65	0.034	0.262	0.593	2.110	4.011	5.801	5.935	67.813
-16.55	0.085	0.195	0.628	1.838	3.976	4.829	6.318	66.479
-16.45	0.074	0.280	0.649	2.162	3.824	4.610	5.473	65.194
-16.35	0.051	0.237	0.565	1.943	2.842	4.763	5.561	63.953
-16.25	0.062	0.243	0.579	1.734	3.941	5.320	5.797	62.756
-16.15	0.040	0.182	0.523	2.099	3.321	4.687	5.679	61.600
-16.05	0.079	0.158	0.481	1.504	3.485	4.490	5.797	60.482
-15.95	0.091	0.207	0.628	1.963	3.099	4.785	5.768	59.400
-15.85	0.034	0.243	0.551	2.099	3.473	4.621	5.601	58.354
-15.75	0.062	0.237	0.544	1.734	3.181	4.545	5.787	57.340
-15.65	0.079	0.213	0.649	1.650	3.040	4.709	5.542	56.358
-15.55	0.074	0.255	0.537	1.901	3.239	4.894	5.100	55.405
-15.45	0.079	0.268	0.572	2.454	4.257	6.107	7.684	54.481
-15.35	0.028	0.176	0.467	2.402	4.759	6.555	8.037	53.585
-15.25	0.051	0.152	0.635	2.454	4.806	6.282	8.273	52.715
-15.15	0.028	0.182	0.607	2.433	4.502	6.730	8.656	51.870
-15.05	0.034	0.152	0.516	2.371	4.958	6.183	7.959	51.049
-14.95	0.051	0.195	0.467	2.517	4.385	6.631	8.057	50.252
-14.85	0.062	0.128	0.488	2.256	5.414	7.123	8.470	49.476
-14.75	0.034	0.176	0.586	2.245	4.724	6.205	8.666	48.723
-14.65	0.051	0.189	0.621	2.120	4.444	6.861	8.666	47.989
-14.55	0.023	0.164	0.558	2.538	4.947	6.642	9.226	47.276
-14.45	0.040	0.219	0.558	2.496	4.654	6.839	8.597	46.582

Midpoint	103	30	10	3	1	0.34	0.1	Volume
-14.35	0.028	0.146	0.586	2.486	4.818	6.817	8.470	45.906
-14.25	0.057	0.176	0.474	2.538	5.344	7.614	8.440	45.248
-14.15	0.034	0.170	0.628	2.768	4.982	6.795	9.148	44.608
-14.05	0.045	0.201	0.788	2.611	5.122	7.167	9.000	43.984
-13.95	0.028	0.116	0.684	2.548	5.403	7.090	8.833	43.377
-13.85	0.017	0.201	0.712	2.600	5.192	7.931	8.676	42.785
-13.75	0.017	0.219	0.705	2.663	5.753	7.833	9.374	42.208
-13.65	0.045	0.164	0.809	2.747	5.578	8.139	9.334	41.646
-13.55	0.034	0.207	0.691	2.705	6.057	8.499	9.403	41.098
-13.45	0.023	0.201	0.823	2.956	6.081	8.707	10.091	40.564
-13.35	0.062	0.176	0.635	2.788	5.964	8.576	10.405	40.043
-13.25	0.028	0.237	0.747	3.112	5.999	8.390	10.484	39.536
-13.15	0.034	0.249	0.663	3.196	6.174	8.805	10.612	39.041
-13.05	0.045	0.255	0.942	3.457	6.782	8.587	11.604	38.558
-12.95	0.062	0.316	0.977	3.164	6.467	9.592	11.594	38.088
-12.85	0.040	0.225	0.872	3.279	6.724	9.166	11.535	37.629
-12.75	0.034	0.219	0.935	3.332	6.935	10.138	11.761	37.181
-12.65	0.057	0.249	0.921	3.446	7.472	10.739	11.693	36.745
-12.55	0.051	0.195	0.998	4.418	8.045	10.739	11.909	36.319
-12.45	0.091	0.298	0.886	3.426	7.812	11.241	13.314	35.904
-12.35	0.051	0.322	1.221	4.136	8.338	10.815	13.451	35.499
-12.25	0.034	0.328	1.172	4.386	8.922	12.083	14.434	35.103
-12.15	0.091	0.341	1.235	4.115	8.770	12.760	13.844	34.718
-12.05	0.074	0.304	1.381	4.585	8.618	12.815	15.338	34.342
-11.95	0.068	0.438	1.319	4.909	9.437	13.077	15.377	33.976
-11.85	0.062	0.389	1.465	5.232	10.057	14.322	16.713	33.618
-11.75	0.074	0.438	1.465	5.786	9.987	14.705	17.706	33.269
-11.65	0.085	0.401	1.605	6.068	10.361	15.578	17.942	32.929
-11.55	0.102	0.414	1.591	6.308	12.068	15.721	19.032	32.597
-11.45	0.085	0.444	1.723	6.287	12.372	16.300	20.221	32.274
-11.35	0.057	0.547	2.128	6.851	13.331	19.173	20.791	31.959
-11.25	0.102	0.681	2.163	6.569	13.460	18.965	21.990	31.651
-11.15	0.125	0.693	2.009	7.561	14.536	20.200	23.483	31.351
-11.05	0.159	0.645	2.407	8.773	15.576	21.095	24.957	31.059
-10.95	0.159	0.870	2.798	8.397	15.845	22.505	27.109	30.775
-10.85	0.176	0.772	2.658	9.013	17.202	24.569	27.748	30.497
-10.75	0.170	0.864	2.993	10.193	18.476	25.553	29.909	30.227
-10.65	0.193	0.870	3.356	10.882	20.418	26.929	31.265	29.964
-10.55	0.221	1.016	3.579	10.924	20.102	28.426	31.924	29.707
-10.45	0.238	1.162	3.949	11.707	22.031	30.250	35.677	29.457
-10.35	0.232	1.277	3.900	13.431	23.610	31.867	36.620	29.214
-10.25	0.215	1.283	4.207	13.545	25.773	34.434	39.077	28.978
-10.15	0.295	1.350	4.723	14.851	26.686	35.385	41.622	28.747
-10.05	0.295	1.332	4.779	14.924	28.276	37.985	43.557	28.523
-9.95	0.329	1.508	5.435	17.357	31.387	40.224	46.515	28.306

Midpoint	103	30	10	3	1	0.34	0.1	Volume
-9.85	0.312	1.740	5.770	18.423	31.457	43.917	50.327	28.094
-9.75	0.442	1.916	6.328	18.955	34.380	45.151	53.638	27.888
-9.65	0.436	1.898	6.482	19.770	37.421	47.019	52.862	27.688
-9.55	0.516	2.159	7.110	21.127	38.625	49.925	56.095	27.493
-9.45	0.640	2.220	7.661	23.759	43.443	51.280	60.958	27.305
-9.35	0.578	2.579	8.275	23.926	44.238	56.579	65.930	27.122
-9.25	0.663	2.871	8.568	25.681	47.781	61.058	71.118	26.944
-9.15	0.623	2.993	9.782	28.292	51.009	64.138	75.609	26.772
-9.05	0.759	3.199	10.270	30.391	54.599	68.049	80.905	26.605
-8.95	0.844	3.358	11.575	31.780	56.985	73.676	85.464	26.443
-8.85	0.850	3.796	12.252	34.339	61.592	77.171	84.707	26.286
-8.75	1.009	4.021	12.754	36.521	62.808	79.968	90.180	26.135
-8.65	1.037	4.227	13.829	37.597	63.241	83.235	92.519	25.989
-8.55	1.218	4.203	14.359	37.211	67.264	86.053	94.238	25.847
-8.45	1.116	4.331	14.882	38.704	69.696	85.430	96.891	25.711
-8.35	1.133	4.927	14.875	40.532	65.100	84.480	95.810	25.579
-8.25	1.173	4.805	15.426	39.487	68.620	82.579	99.141	25.452
-8.15	1.150	4.860	15.873	41.085	71.660	84.098	101.951	25.330
-8.05	1.320	5.164	16.389	40.887	73.204	85.551	104.103	25.213
-7.95	1.224	4.933	16.864	40.605	73.648	85.387	98.522	25.100
-7.85	1.241	4.690	16.047	40.970	69.661	84.119	94.621	24.991
-7.75	1.292	5.000	15.984	40.135	69.380	80.187	93.531	24.888
-7.65	1.269	5.310	15.322	39.049	64.539	77.128	90.888	24.788
-7.55	1.241	4.921	15.573	38.380	62.691	74.473	86.053	24.694
-7.45	1.082	5.322	15.852	37.023	60.329	75.205	83.371	24.603
-7.35	1.128	4.823	15.015	37.127	62.878	71.949	85.749	24.517
-7.25	1.252	4.659	15.015	36.448	60.177	71.545	80.836	24.435
-7.15	1.122	4.538	14.694	34.850	54.026	67.667	78.075	24.358
-7.05	1.116	4.574	13.298	32.553	51.746	65.176	68.819	24.285
-6.95	1.020	4.313	13.012	30.871	49.875	61.702	68.288	24.216
-6.85	1.303	3.978	11.868	28.532	46.846	59.233	60.929	24.151
-6.75	1.054	3.589	11.240	27.185	40.870	54.492	55.967	24.091
-6.65	0.969	3.777	10.452	26.099	37.701	50.745	51.487	24.034
-6.55	0.884	3.291	9.880	23.143	35.854	45.261	45.296	23.982
-6.45	0.873	3.151	9.761	21.984	32.883	45.337	41.484	23.934
-6.35	0.935	2.804	9.112	19.269	30.428	44.998	42.044	23.890
-6.25	0.867	2.798	8.484	18.736	27.750	42.038	36.886	23.850
-6.15	0.691	2.616	7.465	18.934	26.721	36.532	32.326	23.814
-6.05	0.822	2.543	7.138	18.684	25.668	31.528	28.367	23.782
-5.95	0.635	2.397	6.133	16.699	24.008	29.649	25.704	23.754
-5.85	0.470	1.971	5.624	13.754	21.540	24.559	25.576	23.730
-5.75	0.572	1.782	5.519	12.167	17.997	22.789	24.859	23.710
-5.65	0.516	1.703	5.163	11.258	16.909	20.549	23.031	23.694
-5.55	0.499	1.667	4.584	10.172	14.921	17.578	21.616	23.682
-5.45	0.499	1.332	3.872	9.201	11.530	15.109	18.374	23.674

Midpoint	103	30	10	3	1	0.34	0.1	Volume
-5.35	0.368	1.247	3.363	6.527	8.724	10.433	10.789	23.670
-5.25	0.295	1.077	3.202	6.057	7.987	8.969	9.266	23.670
-5.15	0.363	1.125	2.812	5.295	7.227	8.685	8.981	23.670
-5.05	0.170	1.010	2.693	5.170	6.794	8.150	7.831	23.670
-4.95	0.283	1.064	2.616	4.512	7.239	7.134	8.440	23.670
-4.85	0.340	0.888	2.547	5.170	6.057	6.970	8.077	23.670
-4.75	0.312	0.894	2.414	4.647	6.525	7.025	6.731	23.670
-4.65	0.272	0.827	2.456	4.668	5.625	6.598	6.465	23.670
-4.55	0.363	0.852	2.114	4.365	5.520	7.057	5.846	23.670
-4.45	0.255	0.748	2.261	4.553	4.923	6.140	6.131	23.670
-4.35	0.198	0.681	2.163	3.707	5.391	6.194	6.377	23.670
-4.25	0.261	0.949	1.863	3.499	5.566	6.074	6.780	23.670
-4.15	0.283	0.687	1.926	3.666	5.777	6.347	7.006	23.670
-4.05	0.244	0.918	2.086	4.000	5.707	5.943	6.790	23.670
-3.95	0.346	0.772	1.912	3.843	6.034	6.205	6.780	23.670
-3.85	0.300	0.955	2.191	4.125	5.882	5.965	7.094	23.670
-3.75	0.323	0.815	2.135	4.042	6.654	6.172	7.242	23.670
-3.65	0.266	0.985	2.016	4.365	7.589	6.303	6.888	23.670
-3.55	0.255	0.888	2.281	4.303	6.759	6.740	6.917	23.670
-3.45	0.306	0.912	2.135	4.533	6.724	6.151	8.126	23.670
-3.35	0.289	0.754	2.777	4.721	6.385	6.631	8.214	23.670
-3.25	0.272	0.967	2.672	4.940	7.028	7.123	8.106	23.670
-3.15	0.334	1.083	2.763	5.076	8.233	8.226	8.145	23.670
-3.05	0.346	1.077	2.889	5.211	8.829	8.335	8.637	23.670
-2.95	0.334	1.095	2.986	5.629	8.314	8.565	8.892	23.670
-2.85	0.312	1.241	2.993	5.890	9.472	8.827	8.981	23.670
-2.75	0.414	1.119	3.126	6.339	9.928	9.657	9.826	23.670
-2.65	0.436	1.344	3.691	7.258	11.343	10.236	12.213	23.670
-2.55	0.544	1.472	3.461	6.914	12.875	9.821	12.027	23.670
-2.45	0.510	1.697	3.775	7.331	13.471	10.826	13.343	23.670
-2.35	0.652	1.861	4.123	7.927	13.729	12.279	14.041	23.670
-2.25	0.589	1.764	4.284	7.885	13.588	14.016	14.965	23.670
-2.15	0.550	2.117	4.403	8.908	14.980	14.661	16.438	23.670
-2.05	0.771	2.129	4.898	10.329	15.962	16.300	18.993	23.670
-1.95	0.720	2.257	5.156	10.402	17.097	17.403	19.307	23.670
-1.85	0.873	2.658	5.791	11.425	17.728	18.452	19.936	23.670
-1.75	0.788	2.761	5.526	12.010	18.968	19.784	21.479	23.670
-1.65	0.873	3.035	6.663	13.692	21.377	21.030	23.395	23.670
-1.55	0.935	3.187	7.096	15.624	21.634	21.685	27.099	23.670
-1.45	0.946	3.668	7.982	17.096	24.522	24.274	27.443	23.670
-1.35	1.190	4.087	8.917	18.360	24.242	27.967	30.921	23.670
-1.25	1.269	4.379	9.545	19.258	26.709	28.327	33.555	23.670
-1.15	1.303	4.623	9.817	20.960	31.001	31.277	35.677	23.670
-1.05	1.292	5.115	11.163	22.443	31.644	33.211	39.067	23.670
-0.95	1.377	5.347	11.128	24.950	33.059	36.543	43.931	23.670

Midpoint	103	30	10	3	1	0.34	0.1	Volume
-0.85	1.394	5.334	12.084	25.556	35.760	40.552	50.199	23.670
-0.75	1.366	5.730	12.273	27.143	37.701	39.613	52.243	23.670
-0.65	1.542	5.882	13.940	28.292	40.063	42.726	50.170	23.670
-0.55	1.712	5.882	13.947	29.702	41.584	43.786	52.508	23.670
-0.45	1.723	6.308	13.912	30.015	42.589	47.052	53.992	23.670
-0.35	1.876	6.423	14.373	30.694	42.180	47.981	56.969	23.670
-0.25	1.848	6.393	14.261	29.764	43.794	50.657	54.582	23.670
-0.05	1.683	5.924	14.498	30.851	46.647	49.084	54.749	23.670
0.05	1.763	6.295	14.142	30.934	47.466	48.997	56.606	23.670
0.25	1.644	5.632	14.415	30.955	43.934	49.073	54.945	23.670
0.35	1.706	5.614	13.877	29.691	43.560	48.986	55.820	23.670
0.45	1.712	5.426	12.740	29.138	42.122	46.244	55.643	23.670
0.55	1.593	5.085	12.691	27.373	39.935	46.211	52.214	23.670
0.65	1.394	4.951	12.119	26.214	38.953	43.939	49.079	23.670
0.75	1.235	4.434	10.752	25.295	38.005	42.114	47.262	23.670
0.85	1.309	4.112	10.270	24.146	35.456	39.733	44.196	23.670
0.95	1.145	3.710	9.426	21.127	33.258	36.466	41.120	23.670
1.05	0.952	3.400	8.365	20.574	30.241	34.336	37.770	23.670
1.15	0.873	3.206	8.282	18.579	27.504	31.059	34.272	23.670
1.25	0.816	2.719	7.640	16.908	24.557	29.704	31.786	23.670
1.35	0.861	2.901	6.251	16.344	24.557	26.208	28.740	23.670
1.45	0.720	2.384	6.105	14.245	21.716	23.805	27.895	23.670
1.55	0.759	2.050	5.491	13.305	20.651	22.166	24.378	23.670
1.65	0.567	2.062	4.863	12.459	18.441	20.975	23.375	23.670
1.75	0.623	1.861	4.703	11.561	17.412	19.435	21.459	23.670
1.85	0.623	1.685	4.368	10.371	15.483	17.949	19.465	23.670
1.95	0.448	1.545	4.054	9.702	15.261	16.868	17.716	23.670
2.05	0.459	1.496	3.830	9.076	13.413	16.234	17.460	23.670
2.15	0.380	1.326	3.356	8.271	13.109	14.595	15.603	23.670
2.25	0.374	1.320	3.489	8.062	11.951	13.896	14.237	23.670
2.35	0.289	1.150	3.056	7.217	11.156	13.667	14.080	23.670
2.45	0.351	1.210	2.756	6.663	11.109	13.099	13.697	23.670
2.55	0.289	1.107	2.707	6.350	10.875	12.596	13.402	23.670
2.65	0.295	0.912	2.540	6.475	10.104	10.651	12.036	23.670
2.75	0.317	0.979	2.561	5.974	9.963	11.460	11.909	23.670
2.85	0.244	0.967	2.442	5.577	9.402	10.389	12.312	23.670
2.95	0.300	0.991	2.037	5.734	8.852	10.357	11.732	23.670
3.05	0.204	0.839	2.233	5.514	8.747	9.920	10.661	23.670
3.15	0.210	0.730	2.344	5.640	8.841	10.335	10.975	23.670
3.25	0.198	0.791	2.184	5.462	8.958	10.018	11.123	23.670
3.35	0.187	0.852	2.030	5.546	9.051	9.668	10.838	23.670
3.45	0.244	0.803	2.337	4.919	8.069	9.177	10.297	23.670
3.55	0.232	0.779	2.065	5.692	8.408	9.767	10.808	23.670
3.65	0.266	0.882	2.135	5.128	7.706	9.679	10.985	23.670
3.75	0.210	0.833	1.835	5.253	8.314	9.920	10.681	23.670

Midpoint	103	30	10	3	1	0.34	0.1	Volume
3.85	0.164	0.827	2.358	5.410	7.987	9.526	10.897	23.670
3.95	0.198	0.864	2.051	4.919	8.116	10.193	10.661	23.670
4.05	0.227	0.748	1.842	5.284	8.069	10.247	10.867	23.670
4.15	0.210	0.772	1.981	5.598	8.338	10.040	10.474	23.670
4.25	0.125	0.657	1.877	5.337	7.917	10.291	11.034	23.670
4.35	0.193	0.754	2.030	5.807	8.607	10.258	11.457	23.670
4.45	0.130	0.736	1.863	5.065	8.642	9.996	12.479	23.670
4.55	0.164	0.833	2.009	5.358	8.583	10.477	11.693	23.670
4.65	0.153	0.827	2.051	5.452	8.525	10.367	12.302	23.670
4.75	0.130	0.718	1.870	5.577	9.495	10.662	11.928	23.670
4.85	0.198	0.791	2.002	5.452	9.098	10.815	12.429	23.670
4.95	0.215	0.560	1.919	5.431	9.051	11.001	12.086	23.670
5.05	0.221	0.669	1.884	5.650	8.770	11.591	12.744	23.670
5.15	0.187	0.693	1.988	5.462	9.379	11.340	12.980	23.670
5.25	0.221	0.809	1.870	5.514	9.484	10.936	12.665	23.670
5.35	0.147	0.718	2.302	5.890	9.554	10.269	12.803	23.670
5.45	0.204	1.156	3.837	10.579	17.623	21.762	26.018	23.674
5.55	0.295	1.423	4.096	11.749	19.798	25.039	26.254	23.682
5.65	0.357	1.575	4.430	12.605	20.780	24.613	27.119	23.694
5.75	0.204	1.283	4.347	12.511	21.400	25.935	27.748	23.710
5.85	0.312	1.484	4.563	12.814	21.072	26.285	29.713	23.730
5.95	0.334	1.515	4.835	12.501	20.546	26.296	31.246	23.754
6.05	0.261	1.435	4.416	12.574	21.634	26.929	32.150	23.782
6.15	0.346	1.393	4.821	12.658	21.482	28.098	31.825	23.814
6.25	0.312	1.563	4.382	13.650	22.791	28.459	32.012	23.850
6.35	0.300	1.448	4.828	13.598	23.002	29.879	32.798	23.890
6.45	0.334	1.569	4.570	13.441	22.850	28.524	34.075	23.934
6.55	0.357	1.223	4.884	13.754	23.224	30.523	33.250	23.982
6.65	0.346	1.545	4.842	12.971	24.160	30.250	34.164	24.034
6.75	0.408	1.429	5.051	14.109	24.277	30.764	35.864	24.091
6.85	0.368	1.502	4.975	13.462	24.417	31.517	36.994	24.151
6.95	0.249	1.557	4.744	14.339	24.920	33.407	35.913	24.216
7.05	0.368	1.508	4.849	14.851	25.294	33.899	37.790	24.285
7.15	0.380	1.746	4.954	14.579	25.586	34.380	38.654	24.358
7.25	0.414	1.575	4.954	15.812	26.966	36.139	38.684	24.435
7.35	0.306	1.557	5.212	15.060	26.510	35.243	40.216	24.517
7.45	0.391	1.612	5.114	15.425	27.422	37.013	41.101	24.603
7.55	0.295	1.460	5.554	15.112	28.463	36.204	42.083	24.694
7.65	0.357	1.648	4.968	15.436	27.376	36.182	43.842	24.788
7.75	0.317	1.454	5.065	15.561	27.317	36.597	42.584	24.888
7.85	0.380	1.460	5.156	15.634	27.469	37.821	42.565	24.991
7.95	0.295	1.588	4.933	15.373	28.369	36.619	43.783	25.100
8.05	0.363	1.545	4.598	15.112	27.984	37.624	42.496	25.213
8.15	0.227	1.411	5.058	14.851	26.943	37.067	42.938	25.330
8.25	0.255	1.460	4.772	14.517	26.370	35.789	42.535	25.452

Midpoint	103	30	10	3	1	0.34	0.1	Volume
8.35	0.283	1.350	4.591	14.475	26.428	34.642	40.492	25.579
8.45	0.249	1.302	4.298	13.702	25.645	33.549	39.273	25.711
8.55	0.278	1.308	4.298	12.543	23.821	34.194	37.475	25.847
8.65	0.255	1.204	3.775	12.773	23.072	31.408	37.190	25.989
8.75	0.181	1.040	3.865	12.062	22.569	30.742	35.952	26.135
8.85	0.159	0.973	3.684	10.465	20.897	29.343	34.547	26.286
8.95	0.130	1.004	3.293	10.339	19.774	27.956	32.867	26.443
9.05	0.153	0.821	3.286	10.203	18.582	25.979	30.597	26.605
9.15	0.204	0.943	2.826	8.814	17.272	24.340	28.436	26.772
9.25	0.153	0.979	2.770	8.292	16.372	22.679	27.315	26.944
9.35	0.153	0.657	2.421	7.739	15.471	21.958	26.559	27.122
9.45	0.147	0.706	2.163	7.854	15.226	20.429	24.289	27.305
9.55	0.130	0.675	2.100	6.486	12.898	19.828	23.385	27.493
9.65	0.130	0.566	1.905	6.976	13.284	17.468	21.518	27.688
9.75	0.102	0.657	1.702	6.736	11.916	17.665	20.054	27.888
9.85	0.102	0.493	1.681	5.859	12.185	16.157	19.180	28.094
9.95	0.085	0.456	1.542	6.026	10.583	16.136	17.185	28.306
10.05	0.068	0.444	1.423	5.566	9.647	14.366	17.254	28.523
10.15	0.096	0.383	1.521	5.003	9.928	14.464	16.822	28.747
10.25	0.091	0.450	1.256	4.439	9.531	12.968	15.377	28.978
10.35	0.051	0.426	1.284	4.459	9.004	12.061	14.965	29.214
10.45	0.057	0.335	1.207	4.209	8.116	11.569	14.562	29.457
10.55	0.040	0.353	1.109	4.219	8.057	11.809	14.306	29.707
10.65	0.062	0.280	1.088	4.010	7.730	11.394	12.921	29.964
10.75	0.074	0.268	1.207	3.415	7.519	10.324	12.027	30.227
10.85	0.034	0.182	0.998	3.520	7.227	10.597	12.135	30.497
10.95	0.040	0.274	0.872	3.070	6.677	9.701	11.987	30.775
11.05	0.068	0.231	1.012	3.342	6.514	8.751	11.634	31.059
11.15	0.079	0.176	0.754	3.175	6.724	9.308	10.985	31.351
11.25	0.040	0.219	0.928	3.164	6.057	8.314	10.671	31.651
11.35	0.051	0.207	0.767	2.966	5.952	8.172	10.307	31.959
11.45	0.034	0.255	0.802	2.851	5.555	7.571	10.160	32.274
11.55	0.040	0.182	0.802	2.841	5.566	7.265	9.796	32.597
11.65	0.051	0.170	0.760	2.600	5.461	7.494	9.501	32.929
11.75	0.057	0.176	0.712	2.872	5.099	6.937	8.647	33.269
11.85	0.062	0.176	0.670	2.486	4.806	6.882	9.059	33.618
11.95	0.017	0.146	0.642	2.318	5.169	6.511	8.362	33.976
12.05	0.028	0.182	0.649	2.569	5.063	6.839	8.548	34.342
12.15	0.034	0.207	0.642	2.465	4.397	6.522	8.548	34.718
12.25	0.045	0.128	0.488	2.131	4.724	6.413	7.919	35.103
12.35	0.023	0.152	0.509	1.922	4.175	5.899	7.841	35.499
12.45	0.028	0.164	0.551	2.089	4.619	6.151	7.448	35.904
12.55	0.051	0.103	0.523	2.256	4.479	5.888	7.261	36.319
12.65	0.017	0.158	0.572	1.869	4.093	5.812	7.133	36.745
12.75	0.040	0.134	0.579	1.890	4.303	5.550	7.409	37.181

Midpoint	103	30	10	3	1	0.34	0.1	Volume
12.85	0.028	0.146	0.488	1.890	3.847	5.681	7.084	37.629
12.95	0.028	0.116	0.419	1.807	4.081	6.041	6.947	38.088
13.05	0.028	0.128	0.426	1.734	4.163	5.156	7.055	38.558
13.15	0.017	0.116	0.495	1.890	4.069	5.484	7.143	39.041
13.25	0.034	0.109	0.523	1.859	3.719	5.135	6.367	39.536
13.35	0.023	0.036	0.398	1.943	3.871	5.080	6.986	40.043
13.45	0.028	0.073	0.342	1.452	3.578	4.916	7.124	40.564
13.55	0.028	0.122	0.293	1.546	3.520	5.277	6.416	41.098
13.65	0.034	0.097	0.537	1.713	3.450	5.233	6.436	41.646
13.75	0.034	0.103	0.460	1.598	3.730	5.003	6.623	42.208
13.85	0.023	0.103	0.481	1.859	3.649	5.102	6.701	42.785
13.95	0.034	0.134	0.363	1.723	3.204	4.993	6.878	43.377
14.05	0.028	0.061	0.412	1.681	3.649	5.255	6.642	43.984
14.15	0.023	0.073	0.342	1.567	3.590	5.025	6.338	44.608
14.25	0.051	0.073	0.398	1.650	3.742	4.730	6.112	45.248
14.35	0.045	0.116	0.398	1.974	3.578	5.091	5.994	45.906
14.45	0.034	0.152	0.412	1.452	4.163	4.643	6.229	46.582
14.55	0.028	0.091	0.272	1.901	3.508	4.774	6.544	47.276
14.65	0.028	0.122	0.286	1.932	3.251	5.014	6.593	47.989
14.75	0.057	0.109	0.412	1.786	3.754	5.233	6.200	48.723
14.85	0.051	0.128	0.419	1.493	3.181	4.621	6.377	49.476
14.95	0.034	0.182	0.384	1.577	3.344	4.687	6.564	50.252
15.05	0.028	0.091	0.370	1.514	3.333	5.145	5.915	51.049
15.15	0.023	0.109	0.426	1.734	3.520	5.102	6.259	51.870
15.25	0.017	0.158	0.412	1.744	3.917	4.752	6.279	52.715
15.35	0.045	0.116	0.286	1.462	3.882	4.566	6.416	53.585
15.45	0.057	0.170	0.460	1.796	3.391	4.894	6.102	54.481
15.55	0.011	0.225	0.384	1.274	2.643	3.747	4.441	55.405
15.65	0.062	0.195	0.447	1.170	2.643	3.835	4.353	56.358
15.75	0.045	0.152	0.530	1.587	2.631	3.572	4.598	57.340
15.85	0.034	0.152	0.586	1.671	3.204	3.540	4.392	58.354
15.95	0.062	0.164	0.335	1.347	2.923	3.867	4.824	59.400
16.05	0.028	0.158	0.349	1.577	2.900	3.856	4.726	60.482
16.15	0.040	0.103	0.412	1.556	2.888	4.053	4.697	61.600
16.25	0.057	0.207	0.454	1.410	2.994	3.977	5.217	62.756
16.35	0.034	0.182	0.537	1.326	3.052	3.856	4.412	63.953
16.45	0.062	0.195	0.530	1.838	2.935	4.009	4.923	65.194
16.55	0.074	0.128	0.544	1.525	3.286	4.031	5.158	66.479
16.65	0.034	0.182	0.516	1.577	3.356	4.545	6.003	67.813
16.75	0.028	0.225	0.412	1.587	3.415	4.075	5.669	69.199
16.85	0.051	0.195	0.426	1.650	3.415	4.512	5.483	70.640
16.95	0.068	0.225	0.509	1.598	3.496	4.566	5.414	72.140
17.05	0.045	0.164	0.572	1.817	3.567	3.998	5.886	73.703
17.15	0.034	0.164	0.572	1.901	3.450	4.719	5.620	75.335
17.25	0.062	0.213	0.642	1.817	3.052	4.479	6.328	77.040

Midpoint	103	30	10	3	1	0.34	0.1	Volume
17.35	0.074	0.176	0.579	2.037	3.707	4.632	5.286	78.826
17.45	0.051	0.170	0.572	1.744	4.034	5.495	5.699	80.700
17.55	0.068	0.213	0.635	2.183	3.988	5.156	6.053	82.670
17.65	0.062	0.249	0.712	2.235	3.871	5.069	6.328	84.746
17.75	0.051	0.207	0.523	2.183	4.257	5.593	6.583	86.940
17.85	0.091	0.213	0.558	2.183	4.069	5.102	7.507	89.267
17.95	0.074	0.213	0.795	2.298	4.093	5.670	7.133	91.743
18.05	0.085	0.237	0.593	2.475	4.678	5.768	7.458	94.390
18.15	0.074	0.243	0.691	2.308	4.607	5.714	7.173	97.236
18.25	0.062	0.219	0.642	2.757	4.385	5.670	8.037	100.316
18.35	0.074	0.249	0.677	2.360	5.005	6.260	7.959	103.676
18.45	0.079	0.298	0.767	2.590	5.403	6.631	8.696	107.382
18.55	0.051	0.322	0.691	2.412	5.438	7.167	8.568	111.530
18.65	0.102	0.268	0.809	3.008	5.239	6.806	8.195	116.271
18.75	0.096	0.408	0.872	3.238	5.625	7.724	9.069	121.873
18.85	0.102	0.322	0.963	2.830	5.999	7.735	9.384	128.897
18.95	0.062	0.280	0.886	3.018	6.303	8.215	10.513	139.219
19.05	0.125	0.414	0.977	3.426	6.993	8.456	10.592	12229.377
19.15	1.162	3.412	8.728	36.689	73.567	83.213	114.725	2244.159
19.25	1.417	4.087	10.640	44.855	90.301	101.970	139.534	2244.159
19.35	1.462	4.021	10.807	45.315	89.447	101.621	136.714	2244.159
19.45	1.320	4.209	10.605	43.874	86.699	103.161	140.252	2244.159
19.55	1.485	3.881	10.152	42.986	87.915	102.440	138.827	2244.159
19.65	1.355	4.179	10.919	43.372	88.406	102.637	136.862	2244.159
19.75	1.632	4.069	10.577	44.709	87.026	102.615	138.316	2244.159
19.85	1.394	4.021	10.556	43.341	87.330	101.872	135.329	2244.159
19.95	1.496	4.008	10.180	44.124	87.821	103.653	137.785	2244.159
20.05	1.406	4.063	10.431	43.895	89.318	102.735	136.105	2244.159
20.15	1.485	3.881	10.270	43.320	88.196	101.413	138.817	2244.159
20.25	1.366	3.966	10.466	43.874	86.336	101.970	138.365	2244.159
20.35	1.355	4.008	10.745	42.819	88.242	101.577	135.044	2244.159
20.45	1.417	3.856	10.745	43.164	86.746	100.528	137.481	2244.159
20.55	1.377	4.051	10.731	42.850	87.775	101.424	136.862	2244.159
20.65	1.428	3.783	10.542	44.250	87.517	101.500	135.702	2244.159
20.75	1.496	4.045	10.228	43.049	87.903	101.369	136.606	2244.159
20.85	1.462	3.832	10.033	42.788	86.290	101.107	136.950	2244.159
20.95	1.349	3.984	10.047	43.446	86.266	100.867	135.968	2244.159
21.05	1.303	3.941	10.298	42.756	86.254	101.785	138.385	2244.159
21.15	1.349	3.935	10.724	42.464	84.477	100.419	137.275	2244.159
21.25	1.587	3.869	10.305	42.621	86.582	98.955	137.235	2244.159
21.35	1.292	3.929	10.096	43.299	86.687	99.895	135.398	2244.159
21.45	1.343	4.160	10.026	43.289	85.787	100.605	135.457	2244.159
21.55	1.445	3.960	9.796	42.213	87.096	99.130	132.706	2244.159
21.65	1.542	4.045	10.131	43.654	87.611	99.818	133.177	2244.159
21.75	1.394	3.698	10.187	41.576	84.430	100.900	136.380	2244.159

Midpoint	103	30	10	3	1	0.34	0.1	Volume
21.85	1.496	4.027	10.061	43.853	84.746	100.616	136.351	2244.159
21.95	1.423	3.747	10.375	41.921	85.740	101.577	134.926	2244.159
22.05	1.423	3.875	10.396	40.950	85.038	99.840	132.568	2244.159
22.15	1.389	3.789	9.698	41.973	86.629	97.491	136.793	2244.159
22.25	1.372	3.990	10.689	41.232	85.179	97.666	133.246	2244.159
22.35	1.462	3.704	9.273	41.670	83.881	97.229	135.516	2244.159
22.45	1.264	3.613	10.263	42.485	85.027	99.993	132.372	2244.159
22.55	1.184	4.051	10.347	41.942	84.956	100.288	134.415	2244.159
22.65	1.366	3.698	9.998	41.628	83.588	98.584	132.372	2244.159
22.75	1.468	3.668	10.026	41.983	84.489	99.621	131.821	2244.159
22.85	1.360	3.899	9.963	42.088	84.781	97.972	132.774	2244.159
22.95	1.309	3.796	9.886	41.493	84.921	99.523	132.902	2244.159
23.05	1.315	3.996	9.670	41.200	84.278	98.518	132.283	2244.159
23.15	1.451	4.221	9.907	41.733	84.044	99.239	132.430	2244.159
23.25	1.343	3.607	9.559	43.216	83.623	98.944	132.224	2244.159
23.35	1.269	3.941	10.445	41.284	82.816	97.611	131.890	2244.159
23.45	1.337	3.650	9.935	42.986	84.033	95.492	131.615	2244.159
23.55	1.440	3.723	10.214	41.587	86.138	98.245	131.114	2244.159
23.65	1.428	3.613	9.412	41.545	84.044	97.710	132.204	2244.159
23.75	1.434	3.978	9.866	41.461	83.986	97.611	131.124	2244.159
23.85	1.587	3.923	9.900	41.399	84.980	99.250	131.939	2244.159
23.95	1.298	3.491	9.866	41.263	85.588	97.163	133.354	2244.159
24.05	1.360	3.917	9.454	42.245	84.781	97.830	131.507	2244.159
24.15	1.428	3.777	9.503	41.085	82.243	99.643	132.460	2244.159
24.25	1.286	3.869	9.482	40.396	83.998	97.142	131.025	2244.159
24.35	1.360	3.948	9.893	41.775	84.302	97.458	131.684	2244.159
24.45	1.332	4.057	10.103	41.200	83.518	96.541	131.566	2244.159
24.55	1.258	3.887	9.893	40.866	82.922	99.381	131.772	2244.159
24.65	1.298	3.698	9.928	40.918	82.992	95.634	130.799	2244.159
24.75	1.377	3.850	9.684	41.837	83.881	98.693	133.944	2244.159
24.85	1.445	3.869	10.047	41.764	84.606	97.305	131.124	2244.159
24.95	1.252	3.856	9.782	41.576	84.746	96.956	133.354	2244.159
25.05	1.440	3.534	9.775	41.357	82.898	97.120	131.536	2244.159
25.15	1.372	3.716	9.489	40.239	83.296	97.939	131.880	2244.159
25.25	1.326	3.589	9.566	41.785	83.050	97.032	130.937	2244.159
25.35	1.542	3.887	9.726	40.135	83.249	96.858	131.035	2244.159
25.45	1.428	3.996	10.340	42.098	82.887	97.677	134.946	2244.159
25.55	1.451	3.558	10.180	40.521	83.319	98.387	131.055	2244.159
25.65	1.377	3.558	9.433	40.605	84.383	97.207	131.978	2244.159
25.75	1.298	3.777	9.928	42.339	84.208	99.217	133.354	2244.159
25.85	1.355	3.875	10.047	41.712	82.711	97.196	131.900	2244.159
25.95	1.377	3.771	10.131	41.075	83.565	97.655	131.831	2244.159
26.05	1.173	3.668	9.963	41.148	83.226	97.710	130.780	2244.159
26.15	1.337	3.954	9.935	40.459	82.103	98.310	131.762	2244.159
26.25	1.428	3.856	9.921	41.054	84.535	98.125	130.750	2244.159

Midpoint	103	30	10	3	1	0.34	0.1	Volume
26.35	1.315	3.966	10.438	40.417	83.647	99.359	132.195	2244.159
26.45	1.366	3.887	9.475	40.218	83.249	98.442	130.465	2244.159
26.55	1.445	3.783	10.012	40.072	83.109	97.163	132.863	2244.159
26.65	1.366	3.802	9.977	40.198	83.319	98.125	132.932	2244.159
26.75	1.292	3.850	9.614	41.493	84.302	97.939	133.148	2244.159
26.85	1.337	3.741	9.279	40.229	82.512	100.190	134.170	2244.159
26.95	1.264	3.741	9.698	41.357	82.711	98.715	129.080	2244.159
27.05	1.451	3.710	9.886	40.950	84.243	98.147	131.998	2244.159
27.15	1.343	3.753	10.166	42.683	83.857	97.994	132.234	2244.159
27.25	1.252	3.796	10.033	40.563	84.325	95.831	131.035	2244.159
27.35	1.496	4.014	9.977	40.845	83.401	99.152	131.359	2244.159
27.45	1.292	3.820	9.782	40.386	83.857	97.316	133.944	2244.159
27.55	1.394	3.972	10.382	40.678	84.126	99.425	134.278	2244.159
27.65	1.491	3.704	9.942	40.772	82.852	98.824	133.177	2244.159
27.75	1.440	3.698	10.389	41.190	84.758	97.546	134.514	2244.159
27.85	1.491	3.820	9.496	42.391	84.734	99.873	131.595	2244.159
27.95	1.286	3.747	9.859	40.093	83.635	100.135	134.170	2244.159
28.05	1.462	3.850	10.096	42.391	86.453	99.305	133.944	2244.159
28.15	1.440	3.753	9.566	41.200	84.196	97.458	134.533	2244.159
28.25	1.445	3.948	10.270	40.521	83.530	97.327	132.863	2244.159
28.35	1.411	3.564	10.200	40.960	85.167	98.245	135.329	2244.159
28.45	1.394	3.552	9.977	41.148	84.851	99.468	133.806	2244.159
28.55	1.394	3.759	9.419	40.772	85.272	97.994	134.720	2244.159
28.65	1.536	3.820	9.747	41.451	85.553	98.420	135.162	2244.159
28.75	1.337	3.808	9.677	41.325	87.599	101.479	136.223	2244.159
28.85	1.400	4.051	9.782	41.587	84.056	100.408	135.683	2244.159
28.95	1.360	4.021	10.047	41.096	84.676	99.578	137.363	2244.159
29.05	1.451	3.893	9.942	41.211	86.126	96.715	135.928	2244.159
29.15	1.400	3.552	9.984	41.879	85.050	98.048	135.712	2244.159
29.25	1.525	4.075	9.733	42.412	85.529	97.873	132.715	2244.159
29.35	1.235	3.814	9.586	42.161	87.085	99.589	135.879	2244.159
29.45	1.389	3.777	9.705	41.587	86.921	97.415	138.473	2244.159
29.55	1.417	3.887	9.705	41.325	84.325	99.960	134.877	2244.159
29.65	1.377	3.820	10.159	41.493	85.986	100.911	136.361	2244.159
29.75	1.508	4.124	9.991	43.571	84.758	101.326	137.216	2244.159
29.85	1.377	4.021	10.424	43.059	85.623	101.894	135.801	2244.159
29.95	1.615	3.978	9.782	41.357	87.681	102.047	137.530	2244.159
30.05	1.513	3.759	10.466	41.691	86.114	100.288	138.748	2244.159
30.15	1.485	3.899	9.852	42.443	86.009	99.785	137.589	2244.159
30.25	1.411	3.789	10.054	41.837	85.822	99.895	138.739	2244.159
30.35	1.235	3.589	9.314	43.466	86.956	101.675	137.510	2244.159
30.45	1.445	3.808	10.326	43.289	88.383	100.211	136.911	2244.159
30.55	1.366	3.783	10.096	40.981	87.962	101.500	136.282	2244.159
30.65	1.258	3.789	10.019	42.067	85.834	101.173	137.412	2244.159
30.75	1.349	3.941	10.145	43.926	85.857	99.731	134.602	2244.159

Midpoint	103	30	10	3	1	0.34	0.1	Volume
30.85	1.530	3.771	10.382	43.341	85.541	101.107	136.262	2244.159
30.95	1.360	3.838	10.221	42.840	86.138	99.665	135.732	2244.159
31.05	1.281	3.735	10.054	43.216	86.570	101.544	137.589	2244.159
31.15	1.406	4.014	9.963	42.589	87.190	103.609	136.017	2244.159
31.25	1.513	3.698	9.663	41.430	88.710	101.358	138.611	2244.159
31.35	1.389	3.875	10.417	41.795	87.330	100.747	140.596	2244.159
31.45	1.400	3.802	9.496	42.213	86.991	102.200	138.680	2244.159
31.55	1.394	3.741	10.291	42.297	87.763	100.037	134.376	2244.159
31.65	1.343	4.027	9.670	41.639	86.290	101.304	138.208	2244.159
31.75	1.258	3.777	9.684	42.923	86.067	101.708	137.117	2244.159
31.85	1.451	3.650	10.626	43.560	84.594	100.681	137.756	2244.159
31.95	1.315	3.899	9.831	42.955	84.921	101.817	135.437	2244.159
32.05	1.224	3.881	9.831	42.694	89.236	100.779	136.823	2244.159
32.15	1.337	4.154	10.019	42.433	85.600	100.430	137.245	2244.159
32.25	1.343	3.662	9.552	42.140	85.693	101.566	137.058	2244.159
32.35	1.468	3.899	9.740	42.224	86.407	100.889	138.798	2244.159
32.45	1.423	3.777	9.510	42.422	88.687	100.408	139.132	2244.159
32.55	1.479	3.850	9.733	43.393	86.664	102.910	136.597	2244.159
32.65	1.349	4.045	10.054	42.464	87.588	101.074	137.727	2244.159
32.75	1.411	4.154	9.705	41.983	86.746	100.441	139.534	2244.159
32.85	1.519	3.704	9.775	42.547	86.851	100.528	138.473	2244.159
32.95	1.428	3.601	9.845	43.895	86.079	100.441	138.454	2244.159
33.05	1.406	3.771	10.354	42.474	86.138	99.971	137.795	2244.159
33.15	1.264	3.856	9.907	40.866	86.313	101.861	136.950	2244.159
33.25	1.377	3.887	9.866	41.534	84.442	100.758	138.473	2244.159
33.35	1.377	3.783	10.256	42.067	86.266	101.238	137.265	2244.159
33.45	1.525	3.631	10.291	42.506	86.804	100.965	137.943	2244.159
33.55	1.389	3.911	10.131	42.265	88.301	101.337	137.579	2244.159
33.65	1.428	3.631	9.356	41.910	87.003	100.506	137.245	2244.159
33.75	1.462	3.777	10.012	42.516	87.471	101.992	139.849	2244.159
33.85	1.491	4.148	10.089	42.130	86.734	101.053	137.117	2244.159
33.95	1.292	3.631	10.166	42.788	86.792	100.736	137.825	2244.159
34.05	1.372	3.978	9.866	41.827	88.009	100.987	138.532	2244.159
34.15	1.479	3.972	9.754	42.641	86.792	101.315	137.609	2244.159
34.25	1.411	3.674	10.026	42.662	85.518	102.265	137.638	2244.159
34.35	1.337	4.100	9.893	42.474	88.394	101.992	138.002	2244.159
34.45	1.360	3.589	10.382	41.754	87.096	101.063	136.174	2244.159
34.55	1.360	3.753	10.326	43.320	87.248	102.724	137.658	2244.159
34.65	1.309	3.589	10.417	42.474	88.207	100.539	137.540	2244.159
34.75	1.508	3.905	9.628	42.746	87.108	102.495	135.693	2244.159
34.85	1.547	4.282	9.747	42.422	88.009	100.211	135.978	2244.159
34.95	1.320	3.601	9.649	43.592	87.178	102.200	138.149	2244.159
35.05	1.479	3.668	10.082	43.299	85.565	101.610	138.552	2244.159
35.15	1.252	3.747	9.524	41.315	86.453	100.692	137.343	2244.159
35.25	1.383	3.558	9.656	42.412	87.400	100.517	136.410	2244.159

Midpoint	103	30	10	3	1	0.34	0.1	Volume
35.35	1.406	3.662	9.991	42.046	86.477	100.441	138.463	2244.159
35.45	1.389	3.796	9.775	42.704	87.038	101.260	138.807	2244.159
35.55	1.360	3.893	10.103	42.349	87.073	100.430	138.542	2244.159
35.65	1.258	3.808	9.747	42.245	86.430	100.921	137.157	2244.159
35.75	1.457	3.875	10.075	42.579	85.623	100.495	136.312	2244.159
35.85	1.343	3.899	10.026	42.422	87.132	101.927	138.070	2244.159
35.95	1.394	3.777	9.538	42.986	86.863	102.189	137.481	2244.159
36.05	1.451	3.783	9.845	42.203	85.471	101.402	137.992	2244.159
36.15	1.383	3.704	10.354	42.171	87.003	101.042	137.884	2244.159
36.25	1.451	4.167	10.110	42.067	88.488	100.965	137.815	2244.159
36.35	1.457	4.051	10.187	41.085	87.447	102.211	135.693	2244.159
36.45	1.366	3.485	9.684	41.399	86.360	99.217	135.869	2244.159
36.55	1.309	3.978	9.649	42.673	88.406	99.927	135.545	2244.159
36.65	1.309	3.850	9.677	42.829	85.167	101.053	138.237	2244.159
36.75	1.326	4.021	9.754	42.245	86.418	100.987	137.638	2244.159
36.85	1.434	3.765	10.626	42.151	86.629	101.883	137.412	2244.159
36.95	1.451	4.063	9.712	42.109	86.640	99.960	137.992	2244.159
37.05	1.553	3.741	9.754	42.443	86.360	100.867	138.346	2244.159
37.15	1.394	4.027	9.635	42.057	85.635	101.664	137.501	2244.159
37.25	1.366	3.875	10.152	43.038	87.003	99.578	136.528	2244.159
37.35	1.326	3.881	9.384	41.002	85.518	102.232	140.026	2244.159
37.45	1.508	3.899	9.984	41.994	86.231	102.342	138.090	2244.159
37.55	1.275	4.021	9.824	41.722	85.822	101.402	134.857	2244.159
37.65	1.309	3.735	9.656	41.806	84.325	99.567	138.847	2244.159
37.75	1.349	3.881	9.747	42.673	87.716	97.873	138.326	2244.159
37.85	1.355	3.704	9.698	42.109	86.617	101.522	135.869	2244.159
37.95	1.440	3.802	9.405	41.795	85.588	99.895	137.894	2244.159
38.05	1.366	3.850	9.719	42.088	85.120	100.211	137.491	2244.159
38.15	1.264	3.741	9.259	43.268	88.336	97.426	136.076	2244.159
38.25	1.377	3.814	9.880	42.944	87.295	101.555	137.127	2244.159
38.35	1.292	4.094	9.824	41.983	86.675	100.779	136.705	2244.159
38.45	1.258	3.966	9.545	40.960	85.997	98.354	136.174	2244.159
38.55	1.411	3.826	9.893	43.654	87.798	99.534	137.137	2244.159
38.65	1.423	3.923	9.621	41.785	86.605	98.518	138.572	2244.159
38.75	1.355	3.607	9.977	42.850	88.886	99.840	138.188	2244.159
38.85	1.326	3.838	9.803	42.234	86.091	100.714	137.088	2244.159
38.95	1.604	4.167	9.775	42.077	87.073	100.255	136.547	2244.159
39.05	1.366	3.698	9.614	40.532	86.453	100.244	138.444	2244.159
39.15	1.423	3.504	9.447	41.973	86.757	100.637	137.638	2244.159
39.25	1.417	3.966	9.775	41.336	86.383	104.111	138.385	2244.159
39.35	1.428	3.789	9.831	41.816	86.886	101.238	137.599	2244.159
39.45	1.383	3.869	9.782	41.493	86.254	100.080	134.897	2244.159
39.55	1.536	3.796	9.391	42.318	85.108	98.737	139.043	2244.159
39.65	1.400	3.838	9.977	41.106	87.178	101.795	137.766	2244.159
39.75	1.400	3.607	9.719	42.641	85.904	100.375	133.924	2244.159

Midpoint	103	30	10	3	1	0.34	0.1	Volume
39.85	1.337	3.723	9.266	42.495	86.570	99.163	136.950	2244.159
39.95	1.303	3.394	10.040	42.234	86.687	99.589	134.867	2244.159
40.05	1.451	3.783	9.545	42.746	86.325	100.484	139.308	2244.159
40.15	1.269	3.668	9.677	42.088	86.909	101.391	134.563	2244.159
40.25	1.372	3.473	9.670	43.425	86.512	102.123	134.907	2244.159
40.35	1.519	3.856	9.614	41.733	85.436	100.452	135.673	2244.159
40.45	1.355	3.838	10.068	42.265	86.594	101.970	134.985	2244.159
40.55	1.309	3.875	9.880	41.900	87.424	101.282	137.687	2244.159
40.65	1.451	3.723	9.384	43.425	86.173	101.511	136.744	2244.159
40.75	1.389	3.686	9.621	41.409	87.365	98.911	137.029	2244.159
40.85	1.332	3.637	9.496	41.106	86.512	100.561	137.324	2244.159
40.95	1.337	3.662	9.524	42.589	85.857	99.785	134.435	2244.159
41.05	1.355	3.881	9.831	42.203	87.634	101.883	136.764	2244.159
41.15	1.423	3.656	9.893	41.597	86.173	98.846	134.907	2244.159
41.25	1.525	3.929	9.489	41.691	86.956	99.337	137.049	2244.159
41.35	1.355	3.777	10.110	43.038	85.483	101.959	135.201	2244.159
41.45	1.343	3.777	10.005	42.088	85.986	99.643	136.990	2244.159
41.55	1.547	4.069	10.047	41.587	84.465	99.578	135.191	2244.159
41.65	1.303	3.753	9.886	42.391	84.758	99.392	136.901	2244.159
41.75	1.343	4.027	9.942	43.080	84.173	102.899	136.606	2244.159
41.85	1.372	3.966	9.956	42.474	87.506	99.512	139.947	2244.159
41.95	1.394	3.978	10.166	42.182	87.494	102.254	136.921	2244.159
42.05	1.559	3.583	9.852	42.192	86.161	100.288	136.312	2244.159
42.15	1.355	3.783	9.768	42.485	85.810	99.534	136.970	2244.159
42.25	1.298	3.856	9.691	40.866	87.821	98.726	136.911	2244.159
42.35	1.281	3.692	10.117	41.117	86.102	99.698	135.643	2244.159
42.45	1.513	3.650	9.649	41.701	87.716	100.157	138.178	2244.159
42.55	1.423	3.692	10.110	41.367	85.424	98.769	138.404	2244.159
42.65	1.372	3.875	9.900	42.516	85.436	98.977	135.791	2244.159
42.75	1.394	3.601	9.866	42.339	85.635	98.879	136.351	2244.159
42.85	1.377	3.631	9.607	41.367	86.208	99.086	136.488	2244.159
42.95	1.496	3.869	9.412	41.378	84.933	101.315	139.299	2244.159
43.05	1.360	3.692	9.775	42.203	85.869	99.490	135.319	2244.159
43.15	1.615	3.595	10.361	42.297	84.676	100.484	136.724	2244.159
43.25	1.400	3.850	9.614	42.944	85.436	100.452	137.353	2244.159
43.35	1.309	3.601	9.893	40.480	86.231	99.458	136.557	2244.159
43.45	1.218	3.522	9.817	41.357	84.559	100.157	133.727	2244.159
43.55	1.303	3.832	9.719	40.720	85.529	98.900	134.533	2244.159
43.65	1.377	3.723	10.033	42.683	85.144	100.026	135.476	2244.159
43.75	1.224	3.796	10.012	41.148	84.910	100.506	137.727	2244.159
43.85	1.394	3.595	9.831	41.252	82.431	99.239	138.591	2244.159
43.95	1.525	3.735	9.866	41.524	85.190	100.190	135.034	2244.159
44.05	1.224	4.033	9.754	41.691	84.079	98.081	135.241	2244.159
44.15	1.337	3.856	9.956	41.879	87.903	100.626	137.776	2244.159
44.25	1.440	3.668	9.803	42.318	88.067	98.496	134.003	2244.159

Midpoint	103	30	10	3	1	0.34	0.1	Volume
44.35	1.547	3.941	9.503	42.245	86.652	101.358	139.436	2244.159
44.45	1.383	3.686	9.761	41.848	84.348	100.080	136.233	2244.159
44.55	1.383	3.978	10.235	41.451	84.383	98.562	133.865	2244.159
44.65	1.423	3.735	9.586	41.138	85.284	99.152	136.174	2244.159
44.75	1.360	3.467	9.949	41.273	85.413	99.206	135.280	2244.159
44.85	1.372	3.850	9.663	41.785	86.161	99.884	135.378	2244.159
44.95	1.479	3.887	10.131	41.921	85.892	99.774	135.427	2244.159
45.05	1.383	3.887	9.907	41.691	85.401	97.699	135.997	2244.159
45.15	1.423	3.923	10.166	41.879	87.096	99.010	134.356	2244.159
45.25	1.440	4.008	9.454	41.722	85.494	100.102	134.494	2244.159
45.35	1.383	3.789	9.238	41.075	85.518	98.933	137.225	2244.159
45.45	1.275	3.710	9.866	40.866	85.728	99.927	136.518	2244.159
45.55	1.400	3.723	9.900	41.879	85.915	98.256	137.599	2244.159
45.65	1.474	3.808	9.635	42.140	85.050	100.943	133.285	2244.159
45.75	1.394	3.680	9.838	41.148	85.506	97.731	136.714	2244.159
45.85	1.326	3.729	9.503	41.587	84.828	102.374	135.791	2244.159
45.95	1.394	3.595	9.880	41.963	85.974	101.107	138.621	2244.159
46.05	1.440	3.741	9.656	40.991	84.992	100.004	135.712	2244.159
46.15	1.355	4.148	9.482	42.506	84.559	101.402	137.206	2244.159
46.25	1.496	3.674	9.545	41.440	84.886	99.163	140.733	2244.159
46.35	1.519	4.057	9.866	41.848	86.325	99.949	136.577	2244.159
46.45	1.411	3.741	9.377	42.422	84.723	99.053	136.862	2244.159
46.55	1.332	3.875	10.124	41.158	86.032	99.993	136.312	2244.159
46.65	1.491	3.510	9.733	42.547	87.272	99.753	137.176	2244.159
46.75	1.457	3.455	9.621	42.109	85.904	99.774	136.164	2244.159
46.85	1.394	3.808	9.733	42.516	84.793	100.211	137.727	2244.159
46.95	1.275	3.656	9.852	40.375	85.389	99.490	136.557	2244.159
47.05	1.309	3.631	9.747	41.952	84.758	99.359	138.336	2244.159
47.15	1.564	3.850	9.649	43.205	86.675	99.763	134.622	2244.159
47.25	1.406	3.686	9.684	43.195	84.500	99.993	133.649	2244.159
47.35	1.372	3.674	9.433	42.015	85.167	97.371	135.142	2244.159
47.45	1.286	3.741	9.607	42.464	85.202	99.119	134.199	2244.159
47.55	1.400	3.796	9.824	41.555	84.652	101.063	134.229	2244.159
47.65	1.496	3.941	9.300	41.023	86.465	100.408	134.936	2244.159
47.75	1.400	3.917	9.845	42.422	84.477	98.846	134.975	2244.159
47.85	1.496	3.692	9.740	41.942	86.032	99.774	135.634	2244.159
47.95	1.355	3.607	9.998	40.991	84.793	101.151	135.015	2244.159
48.05	1.428	4.002	9.914	43.049	83.483	101.053	136.842	2244.159
48.15	1.423	3.716	9.984	42.067	84.278	97.513	132.932	2244.159
48.25	1.298	3.954	9.942	40.897	84.348	98.310	136.036	2244.159
48.35	1.349	3.838	9.670	41.378	85.986	99.862	135.398	2244.159
48.45	1.320	3.716	9.349	41.012	85.529	97.513	136.184	2244.159
48.55	1.451	3.516	9.893	41.576	84.582	99.086	138.041	2244.159
48.65	1.213	4.063	10.026	41.628	84.629	99.578	136.538	2244.159
48.75	1.474	3.747	9.921	42.203	84.688	99.687	136.587	2244.159

Midpoint	103	30	10	3	1	0.34	0.1	Volume
48.85	1.457	3.765	9.914	41.837	86.500	99.501	136.174	2244.159
48.95	1.457	3.716	9.663	41.827	84.875	99.217	135.545	2244.159
49.05	1.457	3.686	9.649	42.339	87.307	98.365	138.798	2244.159
49.15	1.235	4.112	9.991	40.991	85.389	98.420	137.363	2244.159
49.25	1.457	3.716	9.810	41.378	84.500	99.163	135.182	2244.159
49.35	1.547	3.735	9.775	41.691	86.652	100.233	138.257	2244.159
49.45	1.343	3.607	9.579	41.346	87.225	100.758	135.889	2244.159
49.55	1.417	3.747	9.712	42.683	84.804	99.097	135.319	2244.159
49.65	1.332	3.917	9.852	42.171	87.038	100.976	136.213	2244.159
49.75	1.383	3.698	10.228	41.816	85.202	98.944	135.506	2244.159
49.85	1.326	3.528	9.328	42.422	83.717	99.807	133.541	2244.159
49.95	1.428	3.668	9.300	41.325	86.243	99.960	137.559	2244.159
50.05	1.343	3.601	9.733	42.077	87.400	100.474	136.341	2244.159
50.15	1.372	3.729	9.893	41.200	83.846	99.545	138.788	2244.159
50.25	1.525	3.674	10.228	41.263	85.892	100.277	134.386	2244.159
50.35	1.235	3.796	9.454	42.245	85.120	100.943	135.565	2244.159
50.45	1.502	3.777	9.335	42.464	84.524	98.190	137.540	2244.159
50.55	1.355	3.656	9.838	41.764	84.781	100.004	137.137	2244.159
50.65	1.332	3.796	10.270	40.699	87.564	100.463	135.437	2244.159
50.75	1.332	3.552	9.538	41.273	85.272	98.212	134.455	2244.159
50.85	1.604	3.741	9.656	41.848	86.149	100.080	138.984	2244.159
50.95	1.451	3.686	9.475	41.430	85.483	100.397	135.486	2244.159
51.05	1.394	3.893	9.670	41.869	84.921	101.129	135.712	2244.159
51.15	1.508	3.765	9.475	41.754	85.003	100.758	136.793	2244.159
51.25	1.457	3.607	9.238	40.939	85.342	98.835	137.687	2244.159
51.35	1.292	3.723	9.475	42.265	85.529	99.348	136.616	2244.159
51.45	1.428	3.686	9.949	40.939	84.652	100.867	135.545	2244.159
51.55	1.355	3.777	9.810	41.200	85.962	100.561	135.427	2244.159
51.65	1.343	3.467	9.907	41.044	84.816	99.807	133.551	2244.159
51.75	1.468	3.808	9.838	41.712	88.336	100.484	136.999	2244.159
51.85	1.326	3.650	9.656	41.482	83.822	99.272	136.135	2244.159
51.95	1.360	3.680	9.579	40.709	86.804	95.623	137.147	2244.159
52.05	1.411	3.972	9.300	42.036	85.869	99.763	136.587	2244.159
52.15	1.337	3.850	9.775	41.388	86.149	102.014	136.852	2244.159
52.25	1.479	3.747	9.607	40.490	86.547	101.806	135.516	2244.159
52.35	1.479	3.741	9.503	42.224	85.050	99.468	136.380	2244.159
52.45	1.235	3.577	9.628	41.127	85.261	99.195	136.960	2244.159
52.55	1.360	3.917	9.335	41.607	85.261	98.966	136.557	2244.159
52.65	1.337	3.802	9.956	42.245	84.395	100.255	137.304	2244.159
52.75	1.258	3.589	10.061	41.252	86.863	100.954	137.058	2244.159
52.85	1.440	3.783	9.579	41.681	85.073	99.523	136.783	2244.159
52.95	1.355	3.735	9.391	41.263	87.248	99.348	137.265	2244.159
53.05	1.207	3.686	9.614	41.232	84.992	98.267	136.872	2244.159
53.15	1.411	3.753	9.998	41.879	83.904	100.342	138.002	2244.159
53.25	1.218	3.583	9.880	41.795	85.132	99.490	136.351	2244.159

Midpoint	103	30	10	3	1	0.34	0.1	Volume
53.35	1.343	3.613	9.824	42.109	84.769	98.442	136.351	2244.159
53.45	1.349	3.540	9.684	41.232	84.594	99.905	133.079	2244.159
53.55	1.496	3.619	9.510	41.482	83.565	99.578	135.309	2244.159
53.65	1.355	3.729	9.726	42.318	83.764	100.037	135.034	2244.159
53.75	1.349	3.948	9.545	41.858	84.898	100.299	134.965	2244.159
53.85	1.332	3.826	9.279	40.594	81.951	97.437	138.591	2244.159
53.95	1.389	3.716	9.231	42.349	85.307	100.900	137.776	2244.159
54.05	1.190	3.528	9.998	41.357	84.688	98.933	136.734	2244.159
54.15	1.298	3.723	9.524	42.119	85.857	98.114	135.152	2244.159
54.25	1.298	4.160	9.461	41.305	84.559	98.857	138.739	2244.159
54.35	1.224	3.777	9.377	40.678	85.822	100.452	136.204	2244.159
54.45	1.519	3.650	9.684	42.265	84.617	99.523	134.474	2244.159
54.55	1.451	3.710	9.705	42.756	83.705	98.168	134.965	2244.159
54.65	1.559	3.808	9.573	41.367	85.611	99.075	136.174	2244.159
54.75	1.440	3.631	9.761	41.085	84.886	100.233	136.262	2244.159
54.85	1.355	3.929	9.677	41.545	83.810	98.343	137.727	2244.159
54.95	1.383	3.662	10.089	41.430	85.681	101.402	137.903	2244.159
55.05	1.349	3.716	9.893	41.305	87.178	100.353	132.981	2244.159
55.15	1.372	3.996	10.012	41.399	86.231	100.135	136.223	2244.159
55.25	1.343	3.826	9.949	42.506	84.840	100.222	135.467	2244.159
55.35	1.485	3.826	9.517	40.887	85.073	97.294	134.946	2244.159
55.45	1.337	3.643	9.796	41.273	85.962	98.879	136.184	2244.159
55.55	1.383	3.668	9.405	42.527	82.758	101.151	138.385	2244.159
55.65	1.247	3.662	9.866	43.237	85.658	98.529	137.933	2244.159
55.75	1.428	3.753	9.517	42.777	86.290	98.649	135.211	2244.159
55.85	1.440	3.765	9.747	42.401	84.150	97.939	134.504	2244.159
55.95	1.264	3.388	9.384	40.970	85.506	100.747	137.432	2244.159
56.05	1.303	3.814	9.217	40.406	85.880	99.818	133.659	2244.159
56.15	1.474	3.856	9.503	40.814	86.032	99.873	136.911	2244.159
56.25	1.298	3.698	9.419	42.098	85.974	99.807	136.852	2244.159
56.35	1.139	3.589	9.810	42.046	84.290	99.348	134.622	2244.159
56.45	1.332	3.662	9.970	42.077	86.640	100.517	135.417	2244.159
56.55	1.343	3.595	9.866	41.785	85.342	98.070	136.881	2244.159
56.65	1.462	3.370	9.468	41.910	83.892	99.490	137.442	2244.159
56.75	1.332	3.540	9.489	42.088	83.612	100.233	135.860	2244.159
56.85	1.303	3.850	9.907	40.866	88.278	100.736	133.590	2244.159
56.95	1.247	4.252	9.831	41.879	85.471	99.643	137.766	2244.159
57.05	1.320	3.674	9.900	41.493	86.605	98.977	134.985	2244.159
57.15	1.468	3.595	9.740	42.077	87.015	98.223	134.494	2244.159
57.25	1.366	4.063	9.796	41.430	85.845	100.474	139.249	2244.159
57.35	1.275	3.917	9.321	41.889	85.436	100.692	135.437	2244.159
57.45	1.491	3.595	9.705	42.600	84.477	101.774	134.327	2244.159
57.55	1.315	3.613	9.845	41.848	85.003	100.572	136.950	2244.159
57.65	1.383	3.875	9.328	41.284	84.196	99.010	138.237	2244.159
57.75	1.355	3.887	9.635	41.346	86.874	97.469	136.508	2244.159

Midpoint	103	30	10	3	1	0.34	0.1	Volume
57.85	1.485	3.929	9.496	41.743	84.033	100.157	135.604	2244.159
57.95	1.252	3.869	10.214	41.775	85.073	99.250	136.940	2244.159
58.05	1.355	3.716	9.893	42.391	86.301	99.174	134.681	2244.159
58.15	1.394	3.935	9.363	41.952	86.500	98.737	134.651	2244.159
58.25	1.451	3.692	9.642	41.576	85.529	98.387	137.658	2244.159
58.35	1.496	3.528	10.033	41.117	86.336	98.507	136.528	2244.159
58.45	1.474	4.087	9.293	42.036	83.577	99.053	136.312	2244.159
58.55	1.513	3.546	9.859	41.983	86.079	98.016	137.107	2244.159
58.65	1.406	3.796	9.586	42.610	85.576	100.845	136.724	2244.159
58.75	1.173	3.972	9.252	41.764	85.366	99.217	137.176	2244.159
58.85	1.423	4.039	10.117	41.618	84.348	99.567	137.166	2244.159
58.95	1.343	3.729	9.928	41.848	85.717	101.085	134.690	2244.159
59.05	1.281	3.753	9.614	40.960	83.144	99.359	136.783	2244.159
59.15	1.360	3.869	10.138	42.474	83.483	100.867	134.464	2244.159
59.25	1.394	3.875	9.300	41.869	85.752	101.227	136.351	2244.159
59.35	1.434	3.966	9.573	41.670	87.225	100.539	135.300	2244.159
59.45	1.383	3.777	9.635	41.273	83.951	98.507	136.754	2244.159
59.55	1.286	3.814	9.531	41.618	84.302	99.163	135.673	2244.159
59.65	1.496	3.826	9.691	41.033	85.740	99.818	136.488	2244.159
59.75	1.377	3.577	9.761	42.339	84.255	99.698	136.960	2244.159
59.85	1.372	3.729	9.503	41.607	86.067	97.797	137.933	2244.159
59.95	1.372	3.558	9.754	41.221	85.237	99.239	137.766	2244.159
60.05	1.286	3.656	9.203	40.897	85.062	98.999	137.284	2244.159
60.15	1.372	3.844	9.761	41.545	84.091	98.158	134.641	2244.159
60.25	1.417	3.723	9.914	42.036	84.372	98.212	134.268	2244.159
60.35	1.167	3.716	9.984	41.461	86.243	99.174	134.848	2244.159
60.45	1.406	3.650	9.684	42.412	85.962	99.021	134.828	2244.159
60.55	1.417	3.552	9.726	40.532	85.529	99.774	135.614	2244.159
60.65	1.247	3.753	9.684	41.357	84.500	99.895	136.744	2244.159
60.75	1.241	3.631	9.712	42.255	85.062	98.649	134.907	2244.159
60.85	1.275	3.710	9.552	40.876	85.459	98.791	135.260	2244.159
60.95	1.264	3.765	9.949	43.466	86.032	98.966	134.572	2244.159
61.05	1.417	3.729	9.684	40.772	85.261	100.124	134.818	2244.159
61.15	1.355	3.966	9.524	42.673	85.962	100.965	134.052	2244.159
61.25	1.332	3.802	9.259	41.628	86.617	99.403	136.793	2244.159
61.35	1.320	3.656	10.005	41.952	86.863	99.239	135.211	2244.159
61.45	1.496	3.394	9.810	42.537	86.091	100.474	137.756	2244.159
61.55	1.326	3.643	10.445	41.722	86.746	100.397	135.781	2244.159
61.65	1.252	3.729	9.998	41.273	85.693	101.151	135.978	2244.159
61.75	1.445	3.826	10.117	41.649	84.489	97.163	135.830	2244.159
61.85	1.326	3.862	9.873	42.234	84.115	100.954	134.985	2244.159
61.95	1.224	3.960	9.489	41.889	83.974	97.579	135.388	2244.159
62.05	1.343	3.710	9.328	40.856	85.494	98.168	138.532	2244.159
62.15	1.298	3.710	9.677	42.913	81.787	97.775	136.125	2244.159
62.25	1.457	3.771	9.977	43.519	85.623	98.540	135.074	2244.159

Midpoint	103	30	10	3	1	0.34	0.1	Volume
62.35	1.281	3.455	9.600	41.722	86.453	96.912	136.626	2244.159
62.45	1.462	3.729	10.145	42.245	83.085	99.119	133.138	2244.159
62.55	1.360	3.534	9.684	42.213	83.004	100.102	133.678	2244.159
62.65	1.462	3.723	9.998	41.722	84.629	100.736	136.095	2244.159
62.75	1.343	3.887	9.754	41.336	84.430	101.326	135.388	2244.159
62.85	1.315	3.589	9.510	40.876	83.530	99.337	137.009	2244.159
62.95	1.207	3.796	9.614	41.691	85.693	96.748	138.621	2244.159
63.05	1.275	4.014	9.852	41.430	87.798	98.300	134.229	2244.159
63.15	1.332	3.686	9.447	41.461	84.711	99.447	134.602	2244.159
63.25	1.309	3.686	9.028	42.140	85.027	100.058	136.233	2244.159
63.35	1.411	3.796	9.112	41.127	87.634	101.883	135.840	2244.159
63.45	1.428	3.881	9.803	40.678	85.565	100.441	136.204	2244.159
63.55	1.241	3.662	9.642	41.994	83.822	98.605	134.798	2244.159
63.65	1.491	3.753	10.124	41.764	84.933	98.813	134.857	2244.159
63.75	1.513	3.802	9.796	42.119	85.518	101.085	136.459	2244.159
63.85	1.462	3.899	9.468	41.785	84.933	99.851	133.354	2244.159
63.95	1.536	3.820	9.845	42.025	86.991	99.829	135.388	2244.159
64.05	1.440	4.106	9.796	40.166	83.998	99.862	134.533	2244.159
64.15	1.417	3.899	9.293	41.618	86.184	99.130	134.661	2244.159
64.25	1.275	3.777	9.921	40.626	86.360	99.195	134.494	2244.159
64.35	1.264	3.723	9.691	41.795	84.886	100.047	135.359	2244.159
64.45	1.462	4.142	9.621	42.203	86.360	100.135	136.557	2244.159
64.55	1.241	3.838	9.489	41.910	85.015	97.371	133.659	2244.159
64.65	1.581	3.698	9.705	41.294	84.992	99.611	135.408	2244.159
64.75	1.326	3.789	10.298	40.835	85.167	97.294	135.083	2244.159
64.85	1.406	3.826	9.677	42.453	86.266	100.211	135.172	2244.159
64.95	1.252	3.723	8.966	42.077	84.606	98.747	136.990	2244.159
65.05	1.570	3.491	9.684	42.746	86.442	100.517	138.591	2244.159
65.15	1.224	3.570	9.140	40.772	84.430	99.600	136.380	2244.159
65.25	1.587	3.552	9.217	41.900	85.132	98.321	134.563	2244.159
65.35	1.394	3.783	9.691	41.545	85.927	99.534	134.592	2244.159
65.45	1.252	3.935	9.621	41.607	83.577	99.895	133.934	2244.159
65.55	1.570	3.862	9.796	40.824	84.945	99.545	135.231	2244.159
65.65	1.320	3.279	9.866	41.764	86.512	99.982	136.626	2244.159
65.75	1.320	3.850	8.938	41.503	84.629	99.938	134.887	2244.159
65.85	1.445	3.656	9.391	41.085	84.582	98.813	135.526	2244.159
65.95	1.252	3.723	10.159	40.260	87.330	97.830	135.408	2244.159
66.05	1.406	3.869	10.110	42.965	84.606	99.359	135.791	2244.159
66.15	1.349	3.662	9.642	42.506	86.371	99.097	138.178	2244.159
66.25	1.434	3.546	9.496	41.315	82.501	100.026	133.845	2244.159
66.35	1.383	3.656	9.803	42.902	85.717	97.699	133.836	2244.159
66.45	1.411	3.625	10.173	40.657	85.740	100.539	134.975	2244.159
66.55	1.247	3.789	9.859	41.816	84.992	101.380	135.899	2244.159
66.65	1.343	3.856	9.614	41.639	84.547	98.190	136.007	2244.159
66.75	1.462	3.613	9.698	42.422	86.523	98.201	136.410	2244.159

Midpoint	103	30	10	3	1	0.34	0.1	Volume
66.85	1.423	3.534	9.824	40.762	87.412	98.092	135.506	2244.159
66.95	1.309	3.625	9.656	42.495	87.541	100.441	136.154	2244.159
67.05	1.451	3.607	9.893	42.203	85.459	97.207	133.934	2244.159
67.15	1.320	3.613	9.796	41.597	85.050	98.234	136.331	2244.159
67.25	1.218	3.911	9.880	42.203	85.681	97.873	135.555	2244.159
67.35	1.281	3.875	9.852	42.850	83.156	98.387	136.793	2244.159
67.45	1.411	3.978	9.880	40.876	84.968	99.316	136.262	2244.159
67.55	1.286	3.686	9.740	40.542	85.693	99.348	137.049	2244.159
67.65	1.349	3.796	9.628	41.064	84.571	98.737	135.437	2244.159
67.75	1.434	3.583	9.775	42.161	85.120	99.927	135.771	2244.159
67.85	1.315	3.473	10.207	41.075	84.068	100.769	134.818	2244.159
67.95	1.366	3.972	9.405	42.161	87.132	101.260	136.439	2244.159
68.05	1.247	3.832	9.663	41.681	88.441	100.004	135.958	2244.159
68.15	1.440	3.528	9.538	41.921	84.956	98.747	135.742	2244.159
68.25	1.230	4.173	9.928	42.871	85.670	99.163	136.950	2244.159
68.35	1.394	3.510	9.796	40.574	85.834	100.113	136.036	2244.159
68.45	1.377	3.832	9.349	41.232	85.225	100.037	136.508	2244.159
68.55	1.553	3.668	9.342	41.399	86.184	98.758	135.408	2244.159
68.65	1.332	3.698	9.684	41.952	83.752	99.523	138.051	2244.159
68.75	1.423	3.856	9.907	41.921	85.027	99.676	137.176	2244.159
68.85	1.389	3.692	9.273	41.179	85.448	99.556	136.980	2244.159
68.95	1.326	3.850	10.005	41.837	86.102	101.107	137.520	2244.159
69.05	1.355	3.729	9.503	41.357	84.033	100.157	137.579	2244.159
69.15	1.564	3.668	9.719	40.480	85.927	99.042	136.832	2244.159
69.25	1.428	3.601	9.893	42.057	84.664	98.289	137.844	2244.159
69.35	1.366	3.643	9.573	41.618	84.875	100.244	135.152	2244.159
69.45	1.326	3.595	9.831	41.179	85.927	97.873	138.316	2244.159
69.55	1.269	3.674	9.586	42.621	85.494	101.162	136.970	2244.159
69.65	1.428	3.814	9.824	42.349	84.348	99.993	138.621	2244.159
69.75	1.355	3.637	9.614	41.232	86.301	99.053	137.186	2244.159
69.85	1.411	3.990	9.635	41.461	85.062	97.917	137.589	2244.159
69.95	1.275	3.954	9.712	41.148	84.886	99.501	136.646	2244.159
70.05	1.423	3.619	9.963	41.858	85.728	98.059	137.648	2244.159
70.15	1.292	3.899	9.831	42.464	86.360	96.715	134.150	2244.159
70.25	1.389	3.887	9.559	41.033	85.377	99.468	136.793	2244.159
70.35	1.247	3.826	9.677	41.054	86.523	98.813	134.779	2244.159
70.45	1.269	3.862	9.203	42.255	87.202	98.966	136.901	2244.159
70.55	1.417	3.759	9.789	41.138	85.728	99.632	133.610	2244.159
70.65	1.417	3.723	9.559	41.764	85.366	99.720	135.054	2244.159
70.75	1.372	3.808	9.893	41.451	85.997	99.261	135.643	2244.159
70.85	1.372	3.631	9.963	41.273	85.822	99.108	133.786	2244.159
70.95	1.247	3.643	9.670	41.482	84.068	99.556	134.592	2244.159
71.05	1.355	3.650	9.998	41.545	84.769	99.687	136.518	2244.159
71.15	1.264	3.510	9.754	40.083	85.015	98.354	134.484	2244.159
71.25	1.360	3.856	9.419	41.263	84.185	98.966	135.850	2244.159

Midpoint	103	30	10	3	1	0.34	0.1	Volume
71.35	1.457	3.662	9.468	41.232	85.681	100.015	137.009	2244.159
71.45	1.423	3.856	9.845	42.986	86.418	97.273	136.940	2244.159
71.55	1.315	3.723	9.559	41.044	86.933	98.168	136.223	2244.159
71.65	1.417	3.856	9.238	41.691	84.173	100.670	137.540	2244.159
71.75	1.332	3.552	10.054	42.182	84.313	99.414	137.962	2244.159
71.85	1.428	3.643	9.817	40.793	84.383	100.834	138.188	2244.159
71.95	1.292	3.741	9.349	40.417	85.600	100.572	135.221	2244.159
72.05	1.298	3.923	9.998	42.276	86.161	101.271	135.319	2244.159
72.15	1.360	3.783	9.698	42.673	84.652	98.846	134.474	2244.159
72.25	1.372	4.027	9.398	41.587	86.535	99.195	135.634	2244.159
72.35	1.372	3.680	10.005	41.430	85.471	97.819	133.384	2244.159
72.45	1.349	3.674	10.214	41.848	85.120	99.621	135.673	2244.159
72.55	1.411	3.558	9.496	41.534	85.939	99.217	138.444	2244.159
72.65	1.423	3.984	9.698	41.451	85.553	99.829	133.924	2244.159
72.75	1.479	3.771	10.054	41.085	87.026	99.982	136.184	2244.159
72.85	1.224	3.710	9.398	42.057	84.781	99.239	135.064	2244.159
72.95	1.389	3.546	9.635	42.746	87.716	99.337	134.464	2244.159
73.05	1.394	4.063	9.503	41.858	85.787	99.086	135.732	2244.159
73.15	1.536	3.686	9.566	41.252	85.249	99.829	135.693	2244.159
73.25	1.337	3.929	10.117	41.983	85.728	101.566	137.687	2244.159
73.35	1.542	3.844	9.921	42.307	85.202	101.380	138.237	2244.159
73.45	1.372	3.443	9.607	41.252	85.541	100.299	136.371	2244.159
73.55	1.298	3.534	9.182	41.221	84.302	100.626	135.810	2244.159
73.65	1.355	3.631	10.180	42.401	86.629	99.851	137.019	2244.159
73.75	1.406	3.759	9.607	41.190	85.635	100.310	138.463	2244.159
73.85	1.445	3.650	9.517	41.399	85.249	100.484	134.661	2244.159
73.95	1.355	3.923	9.559	41.211	85.448	98.813	136.980	2244.159
74.05	1.451	3.996	10.082	42.840	85.518	100.484	137.206	2244.159
74.15	1.394	3.735	10.019	42.433	88.114	100.091	136.901	2244.159
74.25	1.457	3.668	9.866	41.618	84.769	98.562	136.125	2244.159
74.35	1.332	3.680	10.312	41.889	85.974	99.425	136.508	2244.159
74.45	1.377	3.911	9.370	42.036	83.998	103.063	134.965	2244.159
74.55	1.298	4.075	9.273	42.057	85.155	98.769	137.314	2244.159
74.65	1.400	3.862	9.545	41.075	85.553	100.889	137.923	2244.159
74.75	1.230	3.376	9.307	41.649	86.067	98.201	136.980	2244.159
74.85	1.377	3.522	9.545	40.803	86.523	100.637	135.693	2244.159
74.95	1.349	3.935	9.810	41.555	87.178	99.589	135.329	2244.159
75.05	1.326	3.504	9.984	42.265	85.050	97.917	137.255	2244.159
75.15	1.445	3.589	9.161	41.994	88.196	101.817	137.363	2244.159
75.25	1.457	3.832	9.928	42.464	86.149	99.731	134.130	2244.159
75.35	1.281	3.674	10.054	41.513	87.681	99.916	135.083	2244.159
75.45	1.468	3.759	9.705	41.127	86.231	99.326	136.724	2244.159
75.55	1.383	3.613	9.726	41.889	86.161	100.539	136.361	2244.159
75.65	1.264	3.862	9.705	41.179	85.319	98.726	137.589	2244.159
75.75	1.434	3.862	9.984	40.281	87.108	100.878	136.115	2244.159

Midpoint	103	30	10	3	1	0.34	0.1	Volume
75.85	1.383	3.941	10.221	42.077	84.044	100.310	137.137	2244.159
75.95	1.213	3.783	10.047	43.028	85.261	99.829	134.602	2244.159
76.05	1.417	3.911	9.454	40.459	86.383	98.529	136.950	2244.159
76.15	1.218	3.838	9.782	41.315	87.260	99.425	137.805	2244.159
76.25	1.428	3.862	9.845	41.754	87.868	100.179	135.496	2244.159
76.35	1.485	3.504	9.677	41.419	85.424	99.250	138.041	2244.159
76.45	1.610	3.850	9.740	41.545	86.980	100.277	136.940	2244.159
76.55	1.451	3.844	9.503	41.357	86.804	100.190	136.950	2244.159
76.65	1.309	3.832	9.907	41.336	86.021	101.785	137.481	2244.159
76.75	1.519	3.753	10.103	41.461	83.015	99.545	136.734	2244.159
76.85	1.269	3.935	10.033	42.328	86.114	99.032	136.764	2244.159
76.95	1.502	3.583	9.538	42.474	84.863	99.763	136.606	2244.159
77.05	1.394	3.875	9.273	41.597	84.711	99.414	136.066	2244.159
77.15	1.349	3.808	9.705	42.318	86.325	99.305	135.241	2244.159
77.25	1.417	3.869	9.531	41.075	85.132	100.026	136.380	2244.159
77.35	1.417	3.577	10.277	42.224	86.219	99.851	135.958	2244.159
77.45	1.451	3.692	9.405	42.109	87.424	100.463	134.523	2244.159
77.55	1.525	3.911	10.256	42.255	85.880	99.206	138.473	2244.159
77.65	1.434	3.583	9.719	41.576	87.763	100.736	133.718	2244.159
77.75	1.332	3.789	9.761	40.960	85.366	99.829	136.832	2244.159
77.85	1.440	3.716	9.642	42.589	87.143	99.479	137.785	2244.159
77.95	1.258	3.862	10.131	42.067	84.500	99.305	137.805	2244.159
78.05	1.377	3.844	9.433	43.101	85.845	101.916	137.019	2244.159
78.15	1.298	3.309	9.698	41.587	86.313	99.731	137.510	2244.159
78.25	1.372	3.656	10.187	42.286	85.576	102.538	137.176	2244.159
78.35	1.445	3.808	9.873	42.547	86.102	100.812	137.668	2244.159
78.45	1.468	3.601	9.719	42.756	87.015	99.567	139.584	2244.159
78.55	1.428	3.716	9.810	41.493	85.693	99.261	135.781	2244.159
78.65	1.235	3.540	9.845	42.589	86.067	100.878	135.830	2244.159
78.75	1.377	3.850	9.761	42.109	84.454	101.697	139.063	2244.159
78.85	1.389	4.075	9.649	40.469	88.289	98.726	135.801	2244.159
78.95	1.479	3.619	9.719	42.067	86.430	100.495	135.516	2244.159
79.05	1.587	3.929	9.921	42.391	87.026	99.742	136.999	2244.159
79.15	1.326	3.954	10.005	41.942	87.775	101.238	137.157	2244.159
79.25	1.428	3.954	9.614	42.161	87.412	99.982	137.559	2244.159
79.35	1.440	3.735	9.447	43.404	84.021	100.266	137.422	2244.159
79.45	1.343	3.941	10.277	42.913	86.488	99.905	136.036	2244.159
79.55	1.377	3.783	10.040	41.879	86.477	100.255	138.198	2244.159
79.65	1.372	3.996	9.886	41.576	86.184	101.664	136.194	2244.159
79.75	1.513	3.674	9.733	42.349	85.190	98.671	137.864	2244.159
79.85	1.326	3.680	9.538	41.837	86.769	100.681	138.513	2244.159
79.95	1.440	3.802	9.831	41.712	86.629	99.228	137.324	2244.159
80.05	1.417	3.716	10.221	41.221	85.027	101.162	138.660	2244.159
80.15	1.411	3.832	10.103	40.427	87.003	102.211	138.856	2244.159
80.25	1.235	3.595	9.789	42.662	85.038	100.812	136.046	2244.159

Midpoint	103	30	10	3	1	0.34	0.1	Volume
80.35	1.434	3.510	9.663	41.712	85.635	101.042	136.872	2244.159
80.45	1.207	3.747	9.398	42.641	85.798	99.763	137.569	2244.159
80.55	1.326	3.637	9.977	42.808	88.242	101.009	137.255	2244.159
80.65	1.457	3.668	9.928	42.171	86.617	100.266	137.717	2244.159
80.75	1.496	3.960	9.928	42.777	86.290	101.959	137.127	2244.159
80.85	1.298	3.704	10.005	41.440	84.325	103.390	135.191	2244.159
80.95	1.355	3.789	10.075	42.318	84.898	100.747	138.100	2244.159
81.05	1.264	3.887	9.817	41.743	84.255	99.807	136.125	2244.159
81.15	1.485	3.583	9.810	41.440	86.547	103.423	136.832	2244.159
81.25	1.615	3.607	9.266	41.649	86.547	101.511	136.587	2244.159
81.35	1.315	3.777	9.656	42.130	86.827	101.905	135.604	2244.159
81.45	1.315	4.051	9.935	42.934	86.067	101.107	136.960	2244.159
81.55	1.315	3.777	10.361	42.506	85.869	102.877	136.243	2244.159
81.65	1.468	3.796	10.019	41.472	86.430	98.879	139.053	2244.159
81.75	1.349	3.643	10.145	42.485	86.254	101.861	137.402	2244.159
81.85	1.525	3.735	9.524	42.808	86.629	101.992	138.621	2244.159
81.95	1.394	3.911	10.124	42.902	85.342	101.992	138.483	2244.159
82.05	1.315	3.686	9.886	42.349	86.044	101.020	138.257	2244.159
82.15	1.303	3.984	10.242	41.983	86.980	100.474	136.036	2244.159
82.25	1.485	3.668	9.579	42.662	87.716	101.107	137.677	2244.159
82.35	1.451	4.154	10.019	42.871	85.483	101.697	135.820	2244.159
82.45	1.349	3.856	9.335	43.143	86.898	100.135	138.837	2244.159
82.55	1.377	3.583	9.670	42.683	85.073	101.380	137.049	2244.159
82.65	1.337	3.583	10.263	43.247	84.688	101.326	136.911	2244.159
82.75	1.400	3.704	9.866	42.088	87.190	100.124	137.609	2244.159
82.85	1.372	4.221	9.342	43.362	86.219	101.205	137.471	2244.159
82.95	1.315	3.692	9.907	42.808	85.401	99.709	135.899	2244.159
83.05	1.417	4.014	9.621	41.649	87.295	101.096	136.999	2244.159
83.15	1.326	4.021	9.642	41.910	86.687	102.615	137.196	2244.159
83.25	1.400	3.850	9.817	44.010	86.091	102.407	136.881	2244.159
83.35	1.400	3.808	9.782	43.007	87.751	100.211	137.461	2244.159
83.45	1.247	3.668	9.635	40.365	86.290	101.806	139.014	2244.159
83.55	1.462	3.796	9.991	44.010	86.161	101.304	138.110	2244.159
83.65	1.355	3.796	10.256	40.751	87.634	102.910	137.098	2244.159
83.75	1.269	3.869	9.621	41.910	86.465	102.090	138.807	2244.159
83.85	1.377	3.844	9.838	41.952	85.155	101.479	140.969	2244.159
83.95	1.332	3.941	9.314	43.195	85.950	101.850	140.085	2244.159
84.05	1.389	3.637	9.552	42.318	87.915	101.675	139.436	2244.159
84.15	1.513	3.905	10.256	41.806	86.126	101.457	138.581	2244.159
84.25	1.247	3.899	10.214	42.464	86.196	102.877	135.624	2244.159
84.35	1.394	4.087	9.293	41.983	87.085	102.079	137.677	2244.159
84.45	1.542	3.960	9.838	41.806	87.155	101.053	138.257	2244.159
84.55	1.621	3.862	9.552	42.735	86.395	100.321	138.670	2244.159
84.65	1.423	3.832	9.893	42.777	85.448	101.839	138.120	2244.159
84.75	1.411	3.917	9.279	41.942	87.085	99.927	136.508	2244.159

Midpoint	103	30	10	3	1	0.34	0.1	Volume
84.85	1.423	3.625	9.907	42.370	88.009	101.358	139.004	2244.159
84.95	1.349	3.467	10.138	41.900	87.436	100.943	137.353	2244.159
85.05	1.423	3.814	9.991	42.673	85.366	102.899	136.616	2244.159
85.15	1.389	3.747	9.607	44.062	86.781	100.834	138.129	2244.159
85.25	1.406	3.905	10.382	42.955	90.277	98.737	136.125	2244.159
85.35	1.406	3.808	9.635	42.944	86.617	100.408	138.572	2244.159
85.45	1.366	3.710	9.886	43.080	84.910	99.895	134.307	2244.159
85.55	1.315	3.869	9.782	41.096	87.342	102.101	135.457	2244.159
85.65	1.337	3.735	9.712	42.808	87.155	102.462	133.138	2244.159
85.75	1.491	3.668	9.873	43.738	86.956	103.161	138.346	2244.159
85.85	1.349	3.978	10.312	42.443	87.728	102.047	135.653	2244.159
85.95	1.434	3.820	9.782	42.453	87.085	102.604	136.449	2244.159
86.05	1.411	4.045	9.998	42.892	86.792	102.014	135.545	2244.159
86.15	1.462	3.814	9.817	41.869	86.944	99.731	138.572	2244.159
86.25	1.462	3.996	10.005	42.892	85.927	101.719	137.962	2244.159
86.35	1.440	3.619	10.075	41.963	85.611	101.380	137.471	2244.159
86.45	1.423	3.625	10.263	41.242	86.909	100.758	139.151	2244.159
86.55	1.372	3.668	9.845	43.059	85.529	101.129	135.182	2244.159
86.65	1.604	3.723	10.145	40.845	86.675	100.911	139.161	2244.159
86.75	1.337	4.014	9.810	41.221	85.904	101.653	138.237	2244.159
86.85	1.366	3.668	9.782	42.171	84.079	101.697	138.306	2244.159
86.95	1.320	3.935	9.970	43.654	87.599	100.758	135.673	2244.159
87.05	1.485	4.094	9.831	41.378	87.248	100.998	137.049	2244.159
87.15	1.281	3.643	10.180	42.923	87.541	101.282	136.744	2244.159
87.25	1.423	3.881	9.866	43.560	85.740	99.807	139.023	2244.159
87.35	1.445	3.814	9.593	43.174	86.757	101.184	137.933	2244.159
87.45	1.440	3.802	10.068	43.247	85.132	98.999	139.869	2244.159
87.55	1.355	4.002	10.228	41.754	86.173	100.310	138.070	2244.159
87.65	1.264	3.704	10.033	42.652	86.477	102.167	136.439	2244.159
87.75	1.303	3.862	9.586	41.409	85.261	100.900	135.889	2244.159
87.85	1.423	3.844	9.642	42.568	86.827	99.163	137.510	2244.159
87.95	1.417	3.601	9.775	41.315	86.442	100.179	136.164	2244.159
88.05	1.349	3.637	10.333	42.934	86.711	100.790	136.547	2244.159
88.15	1.457	3.850	10.033	42.130	85.611	102.516	135.879	2244.159
88.25	1.468	3.826	9.907	42.631	85.459	101.774	138.660	2244.159
88.35	1.445	3.881	9.949	42.339	85.284	102.757	136.764	2244.159
88.45	1.394	3.765	9.796	41.816	85.728	101.380	134.749	2244.159
88.55	1.372	3.832	9.663	42.537	84.173	101.162	134.651	2244.159
88.65	1.264	3.747	9.956	41.910	85.997	100.528	135.201	2244.159
88.75	1.423	3.783	10.277	42.788	86.442	101.402	136.754	2244.159
88.85	1.116	3.820	9.510	40.970	87.775	99.807	134.219	2244.159
88.95	1.428	4.215	10.068	42.767	85.214	99.905	135.781	2244.159
89.05	1.269	3.479	9.663	41.963	85.950	100.441	135.290	2244.159
89.15	1.491	3.546	10.221	42.057	84.231	100.026	137.638	2244.159
89.25	1.445	3.911	10.410	42.318	84.068	99.294	136.862	2244.159

Midpoint	103	30	10	3	1	0.34	0.1	Volume
89.35	1.389	3.978	10.326	42.401	85.693	100.266	136.312	2244.159
89.45	1.553	3.607	9.768	42.464	86.675	100.670	136.901	2244.159
89.55	1.332	3.765	10.061	41.900	85.202	98.452	135.820	2244.159
89.65	1.332	3.869	9.314	42.057	84.992	100.146	138.365	2244.159
89.75	1.417	3.802	9.538	41.273	86.301	100.353	134.730	2244.159
89.85	1.434	3.631	9.880	41.440	85.179	97.513	133.934	2244.159
89.95	1.383	3.485	9.740	40.657	84.664	101.358	134.985	2244.159
90.05	1.423	3.771	9.866	41.566	85.927	100.266	135.034	2244.159
90.15	1.366	3.656	9.342	42.245	86.079	98.726	135.643	2244.159
90.25	1.349	3.941	9.573	42.234	85.144	97.863	132.814	2244.159
90.35	1.428	3.899	10.005	40.636	84.992	99.600	135.791	2244.159
90.45	1.366	3.984	9.405	40.897	84.875	100.015	134.504	2244.159
90.55	1.513	3.747	9.405	41.566	85.284	99.512	134.238	2244.159
90.65	1.196	3.862	9.698	41.336	86.313	98.442	133.089	2244.159
90.75	1.213	3.650	9.838	42.036	83.787	97.721	131.468	2244.159
90.85	1.479	3.783	9.621	40.751	85.003	97.043	134.120	2244.159
90.95	1.479	3.808	9.503	41.722	82.162	97.830	133.511	2244.159
91.05	1.468	3.589	9.419	40.041	82.091	99.458	134.386	2244.159
91.15	1.406	3.534	9.705	41.482	82.852	96.235	132.185	2244.159
91.25	1.298	3.686	9.845	40.490	83.226	98.234	132.303	2244.159
91.35	1.309	3.723	9.984	41.054	82.758	99.523	130.927	2244.159
91.45	1.417	3.558	9.656	41.033	82.103	95.820	134.248	2244.159
91.55	1.281	3.899	9.914	40.396	82.477	98.223	134.160	2244.159
91.65	1.377	3.710	10.082	41.023	81.460	99.184	133.226	2244.159
91.75	1.411	3.613	9.503	40.866	83.904	98.267	133.295	2244.159
91.85	1.343	3.589	9.447	40.218	81.308	97.906	133.737	2244.159
91.95	1.269	3.595	9.126	42.046	82.033	98.125	132.362	2244.159
92.05	1.417	3.583	9.579	41.806	82.384	98.442	132.155	2244.159
92.15	1.337	3.656	9.161	40.793	83.682	97.808	132.018	2244.159
92.25	1.355	3.631	9.691	40.594	83.600	98.201	132.106	2244.159
92.35	1.411	3.656	9.586	40.312	82.360	95.896	128.225	2244.159
92.45	1.372	3.698	9.405	40.991	80.875	97.043	132.578	2244.159
92.55	1.547	3.668	9.677	40.010	82.512	94.432	130.986	2244.159
92.65	1.252	3.692	9.384	40.051	80.606	97.207	130.593	2244.159
92.75	1.366	3.479	9.649	39.456	80.676	94.367	129.925	2244.159
92.85	1.542	3.802	9.356	40.459	82.279	96.923	132.185	2244.159
92.95	1.343	3.911	9.607	39.644	83.296	95.667	132.273	2244.159
93.05	1.360	3.577	9.168	40.083	80.057	99.261	129.964	2244.159
93.15	1.275	3.558	9.266	40.010	82.267	96.158	130.573	2244.159
93.25	1.389	3.619	9.419	38.819	81.062	97.633	131.310	2244.159
93.35	1.406	4.021	9.189	39.268	81.179	97.065	135.083	2244.159
93.45	1.275	3.680	8.993	41.430	81.413	95.634	129.719	2244.159
93.55	1.406	3.449	9.796	41.096	81.402	93.635	132.637	2244.159
93.65	1.366	3.668	9.810	40.072	81.939	96.344	128.598	2244.159
93.75	1.241	3.783	9.761	40.427	79.870	96.879	130.446	2244.159

Midpoint	103	30	10	3	1	0.34	0.1	Volume
93.85	1.269	3.698	9.063	41.064	83.273	97.065	129.090	2244.159
93.95	1.394	3.832	9.286	39.832	82.162	95.405	129.846	2244.159
94.05	1.355	3.832	9.935	39.247	81.881	97.480	129.070	2244.159
94.15	1.360	3.491	9.782	39.947	81.191	97.699	131.684	2244.159
94.25	1.224	3.370	9.426	40.281	80.232	96.300	129.001	2244.159
94.35	1.417	3.382	9.656	40.782	83.121	96.825	130.387	2244.159
94.45	1.434	3.443	9.363	40.866	82.056	96.661	128.117	2244.159
94.55	1.400	3.607	9.670	39.581	81.331	95.547	128.048	2244.159
94.65	1.474	3.461	9.293	41.221	81.390	97.043	128.412	2244.159
94.75	1.298	3.832	9.056	40.699	81.893	94.782	128.824	2244.159
94.85	1.440	3.461	9.614	40.720	81.647	97.087	129.011	2244.159
94.95	1.252	3.668	9.391	40.897	80.443	96.464	128.490	2244.159
95.05	1.264	3.704	9.133	39.665	83.857	96.333	131.045	2244.159
95.15	1.377	3.467	9.684	39.341	81.787	97.589	128.520	2244.159
95.25	1.326	3.473	9.454	40.083	83.039	95.339	129.552	2244.159
95.35	1.337	3.643	9.733	40.678	81.706	97.131	129.827	2244.159
95.45	1.360	3.564	9.489	40.427	81.448	97.152	131.340	2244.159
95.55	1.457	3.668	9.321	40.542	82.524	96.825	127.783	2244.159
95.65	1.428	3.510	9.782	38.746	84.594	93.952	132.087	2244.159
95.75	1.332	3.650	9.133	39.874	83.296	96.759	129.326	2244.159
95.85	1.417	3.577	9.273	40.177	83.518	95.131	128.029	2244.159
95.95	1.326	3.522	9.259	40.824	81.273	95.219	130.534	2244.159
96.05	1.366	3.698	9.391	40.720	81.530	96.246	131.202	2244.159
96.15	1.326	3.692	9.559	39.832	83.565	97.305	128.461	2244.159
96.25	1.349	3.619	9.928	41.440	83.448	95.263	130.347	2244.159
96.35	1.559	3.358	9.363	39.790	82.279	96.126	129.846	2244.159
96.45	1.434	3.589	9.538	40.730	80.314	95.044	130.465	2244.159
96.55	1.167	3.656	10.075	40.542	82.980	95.263	131.497	2244.159
96.65	1.303	3.643	8.972	40.365	83.296	97.611	130.888	2244.159
96.75	1.190	3.625	9.782	40.814	83.097	95.492	130.721	2244.159
96.85	1.281	3.577	9.621	40.386	81.600	97.000	130.888	2244.159
96.95	1.360	3.613	9.705	39.780	83.565	97.972	128.716	2244.159
97.05	1.337	3.351	9.049	41.513	81.752	99.042	131.595	2244.159
97.15	1.207	3.552	10.005	41.409	81.542	96.890	127.370	2244.159
97.25	1.224	3.625	9.140	40.856	84.126	99.545	131.821	2244.159
97.35	1.417	3.692	9.859	40.605	82.337	96.115	128.589	2244.159
97.45	1.377	3.710	9.719	41.545	83.775	97.054	132.106	2244.159
97.55	1.366	3.783	9.252	41.117	84.652	98.321	130.524	2244.159
97.65	1.252	3.735	9.559	41.451	81.144	99.851	132.077	2244.159
97.75	1.162	3.759	9.314	41.889	83.939	98.289	127.999	2244.159
97.85	1.315	3.869	9.621	41.012	83.354	96.464	130.239	2244.159
97.95	1.474	3.783	9.593	41.346	82.512	95.874	132.303	2244.159
98.05	1.298	3.376	9.154	41.482	84.255	97.884	131.566	2244.159
98.15	1.298	3.935	9.691	40.887	83.366	98.922	131.045	2244.159
98.25	1.360	3.777	9.524	41.012	82.711	99.851	130.917	2244.159

Midpoint	103	30	10	3	1	0.34	0.1	Volume
98.35	1.485	3.382	9.524	40.594	83.553	97.852	132.126	2244.159
98.45	1.451	3.443	9.238	40.908	82.875	98.136	131.625	2244.159
98.55	1.252	3.650	9.475	40.386	83.237	97.338	132.028	2244.159
98.65	1.337	3.704	9.579	41.618	84.489	97.950	132.725	2244.159
98.75	1.292	3.382	10.173	42.255	83.004	98.879	132.204	2244.159
98.85	1.286	3.887	9.349	41.357	84.161	96.377	132.175	2244.159
98.95	1.468	3.814	9.586	41.367	83.694	98.769	130.652	2244.159
99.05	1.406	3.619	9.761	42.767	83.916	98.747	132.214	2244.159
99.15	1.485	3.826	9.838	41.409	86.114	97.873	130.809	2244.159
99.25	1.286	3.814	9.831	41.994	85.050	99.348	132.499	2244.159
99.35	1.343	3.789	9.245	41.493	82.641	97.480	133.108	2244.159
99.45	1.252	3.753	9.189	41.733	85.027	98.835	132.932	2244.159
99.55	1.355	3.698	9.545	39.707	84.348	99.272	131.959	2244.159
99.65	1.224	3.881	9.398	39.352	84.992	97.579	134.012	2244.159
99.75	1.389	3.546	9.838	41.931	82.852	97.994	134.631	2244.159
99.85	1.434	3.765	9.719	40.699	82.676	98.780	131.949	2244.159
99.95	0.272	0.517	1.884	24.271	16.184	55.606	24.063	2244.159

E.8. CavAb channel: Calcium and Manganese

Table of Mn^{2+} population data for simulation with Ca^{2+} and Mn^{2+} across bulk density ratios (#JJF-GRS12G8)

	Population in histogram bin at Mn^{2+}/Ca^{2+} ratio:					
Midpoint	10	3	1	0.33	0.1	Volume
-99.95	71.403	102.073	38.698	33.903	7.258	2245.929
-99.85	134.551	111.389	74.667	37.691	13.671	2245.929
-99.75	136.615	112.751	74.204	38.556	14.014	2245.929
-99.65	136.437	110.725	73.887	38.850	13.657	2245.929
-99.55	133.650	115.574	77.179	36.428	13.742	2245.929
-99.45	136.271	112.950	74.883	37.362	13.843	2245.929
-99.35	134.895	109.147	73.396	38.365	14.530	2245.929
-99.25	137.172	109.645	74.277	38.037	14.301	2245.929
-99.15	134.397	114.461	75.648	37.587	14.000	2245.929
-99.05	133.768	110.426	76.255	36.930	13.871	2245.929
-98.95	136.354	110.575	74.479	38.002	14.158	2245.929
-98.85	135.559	113.116	75.186	37.293	13.886	2245.929
-98.75	136.639	113.681	75.446	36.791	14.143	2245.929
-98.65	136.923	111.920	77.294	36.982	13.599	2245.929
-98.55	135.393	114.262	76.139	39.351	13.814	2245.929
-98.45	133.294	112.468	73.295	39.248	13.699	2245.929
-98.35	135.808	112.286	75.172	36.757	13.370	2245.929
-98.25	134.895	112.867	75.851	39.299	13.814	2245.929
-98.15	137.955	110.094	74.869	38.538	14.587	2245.929
-98.05	135.441	113.813	75.562	38.227	13.943	2245.929

Midpoint	10	3	1	0.33	0.1	Volume
-97.95	134.741	115.275	73.916	37.414	14.243	2245.929
-97.85	136.757	111.156	75.316	37.414	14.029	2245.929
-97.75	137.054	111.771	76.082	38.227	13.814	2245.929
-97.65	135.464	110.160	74.898	37.379	14.415	2245.929
-97.55	132.938	112.435	74.421	37.518	12.926	2245.929
-97.45	136.734	110.426	74.652	37.673	14.358	2245.929
-97.35	134.646	111.057	76.443	39.438	13.385	2245.929
-97.25	132.570	110.509	76.385	39.092	14.315	2245.929
-97.15	135.963	110.692	75.851	36.791	13.456	2245.929
-97.05	135.014	111.389	76.240	39.663	14.243	2245.929
-96.95	135.453	111.306	76.500	38.244	13.699	2245.929
-96.85	135.334	114.627	76.356	38.590	14.558	2245.929
-96.75	135.963	111.107	75.302	36.255	13.800	2245.929
-96.65	135.725	111.489	75.576	37.743	13.227	2245.929
-96.55	138.003	113.448	75.836	37.725	13.699	2245.929
-96.45	134.729	112.186	75.634	37.310	14.086	2245.929
-96.35	133.638	111.804	74.219	37.587	13.886	2245.929
-96.25	136.295	112.784	75.966	39.057	14.430	2245.929
-96.15	133.911	112.751	76.197	37.829	13.871	2245.929
-96.05	133.021	112.734	75.894	37.829	14.644	2245.929
-95.95	136.152	110.658	75.374	37.033	13.427	2245.929
-95.85	133.934	112.568	74.594	38.002	13.828	2245.929
-95.75	135.192	113.000	74.652	37.812	13.986	2245.929
-95.65	135.868	111.588	75.836	37.068	14.229	2245.929
-95.55	137.042	114.860	75.749	37.864	13.814	2245.929
-95.45	135.583	114.212	75.995	39.524	13.800	2245.929
-95.35	135.927	112.103	76.616	37.397	14.215	2245.929
-95.25	134.895	113.830	75.547	37.068	13.800	2245.929
-95.15	137.172	114.677	76.255	37.328	13.928	2245.929
-95.05	134.516	112.269	77.973	38.469	14.172	2245.929
-94.95	137.113	113.614	77.280	38.279	14.616	2245.929
-94.85	136.318	112.817	76.356	38.971	13.828	2245.929
-94.75	136.745	113.979	75.360	38.590	14.472	2245.929
-94.65	136.200	112.684	75.345	39.490	13.814	2245.929
-94.55	138.394	113.946	76.356	38.435	13.757	2245.929
-94.45	136.508	113.896	74.999	39.957	14.630	2245.929
-94.35	139.960	113.282	77.814	37.795	13.513	2245.929
-94.25	136.508	112.767	75.692	37.345	13.800	2245.929
-94.15	136.639	110.409	73.526	38.884	14.143	2245.929
-94.05	139.367	114.943	77.107	36.826	14.644	2245.929
-93.95	137.682	116.005	79.302	37.950	14.744	2245.929
-93.85	136.520	115.740	74.277	38.158	14.401	2245.929
-93.75	140.339	116.304	77.338	39.351	14.272	2245.929
-93.65	139.723	114.129	76.341	37.916	14.200	2245.929
-93.55	137.647	112.352	76.905	36.930	14.387	2245.929

Midpoint	10	3	1	0.33	0.1	Volume
-93.45	138.536	114.345	76.688	39.265	13.886	2245.929
-93.35	139.082	116.553	75.894	39.144	13.313	2245.929
-93.25	137.421	114.561	76.197	37.276	14.701	2245.929
-93.15	138.584	114.179	75.894	38.002	15.074	2245.929
-93.05	139.960	115.723	77.915	39.005	14.630	2245.929
-92.95	141.371	114.610	78.565	38.711	14.258	2245.929
-92.85	140.837	117.799	78.868	39.697	14.100	2245.929
-92.75	141.620	115.806	75.287	37.466	15.303	2245.929
-92.65	140.588	117.599	77.121	39.905	14.315	2245.929
-92.55	137.742	116.238	78.291	38.089	15.346	2245.929
-92.45	139.723	114.494	79.894	39.109	14.701	2245.929
-92.35	138.608	119.443	77.165	39.351	14.200	2245.929
-92.25	140.695	116.603	76.948	39.005	14.172	2245.929
-92.15	139.794	113.863	79.966	39.023	13.900	2245.929
-92.05	139.853	114.876	75.966	39.075	14.229	2245.929
-91.95	139.284	120.339	77.612	41.513	15.045	2245.929
-91.85	143.909	115.906	77.049	40.545	14.272	2245.929
-91.75	140.885	119.177	78.247	38.521	15.059	2245.929
-91.65	140.624	116.985	77.627	39.507	14.759	2245.929
-91.55	144.052	118.479	80.385	39.628	14.888	2245.929
-91.45	143.838	119.011	80.356	40.061	15.074	2245.929
-91.35	143.696	117.815	79.056	40.095	14.687	2245.929
-91.25	142.771	119.808	80.356	39.576	14.616	2245.929
-91.15	140.731	118.762	81.496	40.856	14.973	2245.929
-91.05	144.419	119.542	80.399	40.285	15.231	2245.929
-90.95	147.930	120.555	77.887	40.458	13.685	2245.929
-90.85	141.929	118.297	78.219	42.067	15.360	2245.929
-90.75	145.938	122.448	80.125	40.943	14.730	2245.929
-90.65	143.447	119.791	80.486	40.199	15.303	2245.929
-90.55	144.503	119.260	81.208	43.191	15.475	2245.929
-90.45	147.159	124.125	83.157	40.631	14.659	2245.929
-90.35	149.057	119.277	82.045	40.614	15.503	2245.929
-90.25	146.578	121.352	82.594	40.372	15.274	2245.929
-90.15	147.752	120.738	81.439	40.891	14.387	2245.929
-90.05	149.033	122.697	82.969	41.479	14.945	2245.929
-89.95	143.992	121.402	81.771	40.701	15.618	2245.929
-89.85	147.835	122.664	82.493	41.479	15.446	2245.929
-89.75	149.235	123.544	81.597	40.510	15.317	2245.929
-89.65	149.449	122.913	81.294	41.652	15.174	2245.929
-89.55	146.958	123.461	82.478	43.416	15.661	2245.929
-89.45	148.523	124.939	81.944	41.168	15.489	2245.929
-89.35	147.444	122.830	84.211	39.715	14.358	2245.929
-89.25	149.733	123.262	82.406	41.565	14.816	2245.929
-89.15	146.329	123.262	82.854	41.531	14.845	2245.929
-89.05	150.860	121.153	82.767	41.998	15.417	2245.929

Midpoint	10	3	1	0.33	0.1	Volume
-88.95	150.919	123.611	85.770	43.139	15.618	2245.929
-88.85	149.413	121.369	82.955	41.548	15.360	2245.929
-88.75	147.480	122.099	83.056	42.050	14.472	2245.929
-88.65	151.121	122.747	82.132	41.998	15.918	2245.929
-88.55	149.899	126.799	84.630	40.856	15.002	2245.929
-88.45	150.931	125.719	85.092	41.946	14.931	2245.929
-88.35	149.674	124.723	83.316	40.683	14.973	2245.929
-88.25	151.382	122.415	81.150	41.617	15.374	2245.929
-88.15	153.517	123.096	82.305	42.257	15.503	2245.929
-88.05	149.520	125.022	83.807	42.759	15.403	2245.929
-87.95	149.698	122.780	82.175	41.565	16.677	2245.929
-87.85	151.560	128.326	83.590	40.061	15.689	2245.929
-87.75	151.904	124.922	85.150	40.130	15.961	2245.929
-87.65	150.231	125.221	82.940	44.056	16.319	2245.929
-87.55	152.853	125.703	84.760	43.676	15.675	2245.929
-87.45	149.982	122.980	82.666	41.998	16.205	2245.929
-87.35	150.990	126.699	83.922	40.649	15.661	2245.929
-87.25	147.385	123.079	81.742	39.645	14.945	2245.929
-87.15	152.781	123.096	83.908	42.811	15.575	2245.929
-87.05	149.413	125.587	83.994	41.929	16.004	2245.929
-86.95	151.963	122.897	84.110	44.125	15.847	2245.929
-86.85	151.631	125.155	84.038	41.704	15.632	2245.929
-86.75	153.220	129.389	82.132	41.496	15.517	2245.929
-86.65	149.294	127.961	83.142	43.520	15.389	2245.929
-86.55	152.034	123.179	84.933	41.894	15.389	2245.929
-86.45	150.777	123.694	81.785	41.894	15.432	2245.929
-86.35	152.722	125.421	82.536	43.001	15.976	2245.929
-86.25	148.820	127.413	86.204	41.565	15.861	2245.929
-86.15	153.730	121.967	83.966	41.410	16.190	2245.929
-86.05	148.903	125.254	84.659	42.949	15.346	2245.929
-85.95	153.078	126.882	84.399	43.139	15.503	2245.929
-85.85	154.229	126.002	84.298	41.479	15.045	2245.929
-85.75	153.576	125.819	82.262	42.586	15.489	2245.929
-85.65	152.912	124.557	84.456	41.669	16.090	2245.929
-85.55	152.117	128.592	84.399	43.728	16.706	2245.929
-85.45	156.767	128.160	85.294	42.742	15.675	2245.929
-85.35	151.607	127.629	85.323	42.932	15.575	2245.929
-85.25	150.042	126.649	84.269	43.416	16.018	2245.929
-85.15	152.544	123.760	84.760	42.551	16.992	2245.929
-85.05	153.695	127.347	83.532	43.572	16.004	2245.929
-84.95	151.536	129.688	85.626	42.309	15.432	2245.929
-84.85	151.607	128.874	81.034	41.116	15.718	2245.929
-84.75	150.255	123.793	85.958	43.382	15.346	2245.929
-84.65	154.501	125.188	82.883	42.984	14.916	2245.929
-84.55	152.129	126.948	84.225	42.966	15.374	2245.929

Midpoint	10	3	1	0.33	0.1	Volume
-84.45	151.299	126.052	85.525	41.254	15.804	2245.929
-84.35	151.228	127.231	86.579	41.790	15.475	2245.929
-84.25	152.793	124.424	84.500	40.770	15.646	2245.929
-84.15	151.382	125.786	84.023	41.738	15.746	2245.929
-84.05	151.240	124.723	85.135	42.551	16.333	2245.929
-83.95	153.600	126.301	83.734	43.555	16.047	2245.929
-83.85	153.102	123.328	84.514	42.413	16.133	2245.929
-83.75	150.267	126.350	83.215	42.361	15.331	2245.929
-83.65	149.152	127.695	83.128	41.565	15.002	2245.929
-83.55	151.868	124.358	84.052	40.891	14.916	2245.929
-83.45	150.587	126.732	84.529	41.773	15.589	2245.929
-83.35	150.967	126.052	85.467	42.793	15.317	2245.929
-83.25	152.082	125.138	81.814	41.704	15.675	2245.929
-83.15	151.916	125.885	84.081	41.548	16.591	2245.929
-83.05	151.773	125.371	84.890	40.683	15.331	2245.929
-82.95	151.987	124.790	83.576	41.064	15.646	2245.929
-82.85	150.955	126.600	84.774	43.382	14.888	2245.929
-82.75	148.215	129.738	85.294	43.209	15.847	2245.929
-82.65	153.114	124.607	82.738	42.603	16.147	2245.929
-82.55	151.192	130.269	85.337	41.894	15.346	2245.929
-82.45	151.536	128.260	84.081	42.776	15.646	2245.929
-82.35	151.904	125.288	86.594	43.088	15.775	2245.929
-82.25	153.054	125.421	85.554	43.088	15.317	2245.929
-82.15	154.584	126.367	84.803	42.672	16.262	2245.929
-82.05	155.391	124.906	88.196	42.984	16.162	2245.929
-81.95	153.778	126.849	83.677	41.531	15.746	2245.929
-81.85	152.011	126.151	86.146	42.482	15.131	2245.929
-81.75	150.326	127.314	84.933	44.419	15.646	2245.929
-81.65	156.696	125.171	84.355	44.022	15.961	2245.929
-81.55	152.331	126.433	84.110	40.977	16.419	2245.929
-81.45	149.294	124.590	83.388	43.347	15.575	2245.929
-81.35	154.869	128.326	83.186	44.368	15.260	2245.929
-81.25	151.512	129.555	83.402	42.603	16.190	2245.929
-81.15	152.283	124.823	83.908	42.759	15.288	2245.929
-81.05	151.607	125.122	84.168	42.603	15.088	2245.929
-80.95	152.117	125.520	82.464	40.977	16.147	2245.929
-80.85	152.770	124.374	82.305	42.793	16.376	2245.929
-80.75	150.943	126.732	84.471	43.468	16.147	2245.929
-80.65	151.880	125.885	81.612	42.724	15.303	2245.929
-80.55	151.323	124.922	84.731	42.292	15.847	2245.929
-80.45	153.327	127.264	85.381	41.929	15.947	2245.929
-80.35	155.011	126.433	87.287	43.624	16.305	2245.929
-80.25	155.284	127.480	83.677	42.707	15.016	2245.929
-80.15	154.181	123.644	83.850	42.915	15.546	2245.929
-80.05	152.046	123.561	85.323	42.690	15.675	2245.929

Midpoint	10	3	1	0.33	0.1	Volume
-79.95	151.714	125.288	83.518	42.378	15.131	2245.929
-79.85	153.303	127.413	87.272	40.735	15.632	2245.929
-79.75	151.963	126.583	84.716	41.911	14.888	2245.929
-79.65	150.765	125.719	83.677	39.611	15.145	2245.929
-79.55	149.330	127.314	82.579	43.606	15.245	2245.929
-79.45	153.588	125.736	85.294	43.157	15.689	2245.929
-79.35	153.268	126.699	83.157	41.375	15.789	2245.929
-79.25	153.019	127.928	85.929	41.635	15.603	2245.929
-79.15	152.188	122.980	85.496	41.756	15.789	2245.929
-79.05	151.738	126.218	87.258	43.416	14.945	2245.929
-78.95	150.196	123.428	86.536	41.859	15.503	2245.929
-78.85	151.773	125.421	84.774	41.548	15.761	2245.929
-78.75	155.083	127.347	83.518	42.153	15.260	2245.929
-78.65	151.963	123.063	83.605	41.081	15.460	2245.929
-78.55	151.299	127.147	86.117	41.808	16.162	2245.929
-78.45	151.370	126.068	85.308	43.433	15.088	2245.929
-78.35	149.626	125.919	84.991	42.361	16.104	2245.929
-78.25	152.378	124.590	83.518	41.929	15.346	2245.929
-78.15	151.916	126.799	86.103	41.859	16.176	2245.929
-78.05	155.059	124.341	85.222	41.479	16.133	2245.929
-77.95	154.976	126.516	84.745	41.323	16.018	2245.929
-77.85	154.300	122.000	83.734	41.565	16.219	2245.929
-77.75	152.307	129.605	85.409	41.583	16.462	2245.929
-77.65	153.149	125.686	83.763	44.073	14.988	2245.929
-77.55	153.220	127.496	83.489	41.877	15.160	2245.929
-77.45	153.600	124.374	84.890	41.929	15.603	2245.929
-77.35	153.956	125.670	86.175	42.569	16.262	2245.929
-77.25	151.014	127.413	84.558	42.050	14.959	2245.929
-77.15	151.951	126.699	83.966	43.226	15.875	2245.929
-77.05	153.813	126.284	85.077	41.271	15.489	2245.929
-76.95	153.825	124.922	84.615	42.205	15.675	2245.929
-76.85	150.089	124.192	85.583	42.430	15.203	2245.929
-76.75	152.070	126.400	83.576	41.046	15.818	2245.929
-76.65	152.829	124.009	85.251	43.278	14.931	2245.929
-76.55	150.552	126.350	85.005	41.565	15.918	2245.929
-76.45	153.019	126.035	83.864	42.396	15.417	2245.929
-76.35	150.931	122.581	84.832	43.036	15.503	2245.929
-76.25	152.236	124.424	82.854	42.378	14.988	2245.929
-76.15	151.667	124.773	84.240	42.275	15.002	2245.929
-76.05	152.675	124.009	81.944	42.223	15.174	2245.929
-75.95	154.478	127.214	82.940	42.223	16.033	2245.929
-75.85	154.466	125.786	82.550	41.341	15.732	2245.929
-75.75	150.540	125.553	85.929	43.658	15.231	2245.929
-75.65	149.864	122.166	82.319	42.569	14.659	2245.929
-75.55	153.624	125.753	83.821	40.960	16.305	2245.929

Midpoint	10	3	1	0.33	0.1	Volume
-75.45	151.465	125.404	85.193	43.209	15.174	2245.929
-75.35	149.757	125.055	84.182	42.793	15.374	2245.929
-75.25	151.916	125.155	83.821	43.814	15.031	2245.929
-75.15	151.595	125.221	85.121	43.693	14.959	2245.929
-75.05	150.089	127.729	86.218	42.084	14.916	2245.929
-74.95	150.907	125.620	84.991	42.257	15.489	2245.929
-74.85	149.069	124.540	84.933	43.088	15.890	2245.929
-74.75	154.905	125.088	84.052	41.323	16.405	2245.929
-74.65	153.327	126.533	84.731	41.237	15.661	2245.929
-74.55	153.137	126.201	83.706	40.493	16.076	2245.929
-74.45	151.370	126.317	84.471	40.995	15.546	2245.929
-74.35	151.251	124.956	83.547	41.600	15.976	2245.929
-74.25	151.975	126.699	84.327	44.229	15.245	2245.929
-74.15	152.829	126.400	85.756	43.883	16.405	2245.929
-74.05	153.979	127.181	83.763	42.430	15.804	2245.929
-73.95	151.429	130.203	82.550	42.534	15.875	2245.929
-73.85	150.718	127.928	83.879	41.012	15.675	2245.929
-73.75	151.916	128.044	83.085	42.119	16.419	2245.929
-73.65	150.552	124.756	83.244	41.808	15.403	2245.929
-73.55	150.765	127.496	85.395	40.943	15.245	2245.929
-73.45	150.694	124.623	83.142	41.548	15.732	2245.929
-73.35	150.386	124.740	84.298	42.050	15.847	2245.929
-73.25	150.386	124.590	83.980	42.275	14.787	2245.929
-73.15	153.861	124.026	83.417	43.261	15.517	2245.929
-73.05	150.326	123.627	82.738	42.396	15.475	2245.929
-72.95	157.170	123.611	81.800	42.499	15.789	2245.929
-72.85	150.682	123.992	83.763	41.808	15.990	2245.929
-72.75	152.865	126.467	85.222	42.326	16.620	2245.929
-72.65	150.077	124.922	84.095	42.880	15.761	2245.929
-72.55	150.753	124.159	84.124	43.693	15.890	2245.929
-72.45	148.144	129.107	86.579	41.773	15.746	2245.929
-72.35	152.509	123.777	82.810	42.915	15.517	2245.929
-72.25	151.560	124.706	84.067	42.275	15.188	2245.929
-72.15	150.445	124.291	83.749	40.908	14.659	2245.929
-72.05	147.598	127.878	83.966	41.513	15.933	2245.929
-71.95	150.623	126.367	85.265	42.153	15.861	2245.929
-71.85	153.339	125.520	85.453	40.839	14.816	2245.929
-71.75	152.153	128.260	81.973	42.240	15.746	2245.929
-71.65	157.550	126.948	82.522	42.223	15.775	2245.929
-71.55	150.955	125.902	83.763	43.641	16.248	2245.929
-71.45	152.022	125.504	82.406	42.465	15.947	2245.929
-71.35	151.014	123.992	84.399	42.171	15.775	2245.929
-71.25	151.133	124.225	82.969	41.375	14.430	2245.929
-71.15	154.276	125.138	84.716	41.064	14.830	2245.929
-71.05	154.620	127.181	84.774	43.105	15.016	2245.929

Midpoint	10	3	1	0.33	0.1	Volume
-70.95	151.346	128.210	83.561	42.344	15.732	2245.929
-70.85	149.840	126.732	84.240	43.191	15.031	2245.929
-70.75	153.007	128.443	86.001	42.067	15.245	2245.929
-70.65	151.916	124.657	84.110	41.946	15.303	2245.929
-70.55	148.476	124.657	84.471	42.724	15.775	2245.929
-70.45	151.192	125.304	84.225	40.891	15.603	2245.929
-70.35	150.753	126.052	83.085	41.133	15.331	2245.929
-70.25	151.916	125.819	84.630	42.413	15.102	2245.929
-70.15	146.934	128.659	84.052	43.399	15.646	2245.929
-70.05	150.006	125.470	83.850	42.863	16.133	2245.929
-69.95	154.893	128.625	85.409	42.361	14.859	2245.929
-69.85	152.959	129.738	84.052	41.565	14.988	2245.929
-69.75	152.758	124.557	82.319	42.793	14.659	2245.929
-69.65	153.339	124.723	84.846	42.517	16.190	2245.929
-69.55	150.386	129.124	84.168	40.078	15.432	2245.929
-69.45	150.314	125.404	83.128	42.845	15.131	2245.929
-69.35	153.007	124.706	86.565	41.894	16.004	2245.929
-69.25	153.635	127.496	84.615	42.153	15.260	2245.929
-69.15	153.220	125.969	83.850	43.312	16.935	2245.929
-69.05	151.833	128.011	85.193	43.797	15.074	2245.929
-68.95	149.769	124.009	83.662	42.897	15.360	2245.929
-68.85	153.885	127.098	83.619	42.430	16.176	2245.929
-68.75	152.260	128.127	85.308	42.119	15.933	2245.929
-68.65	151.584	125.487	83.518	41.704	15.589	2245.929
-68.55	153.232	124.441	83.287	42.638	15.632	2245.929
-68.45	151.856	126.450	82.955	42.205	15.718	2245.929
-68.35	152.948	125.520	84.991	42.863	14.344	2245.929
-68.25	150.670	125.902	84.485	41.652	15.904	2245.929
-68.15	150.433	125.205	85.568	41.271	17.221	2245.929
-68.05	150.741	123.992	84.052	41.929	15.059	2245.929
-67.95	156.091	126.384	85.352	42.828	15.546	2245.929
-67.85	150.741	123.943	86.204	41.479	15.546	2245.929
-67.75	153.849	126.898	84.991	42.119	15.775	2245.929
-67.65	149.923	126.550	87.171	41.583	15.460	2245.929
-67.55	151.251	123.079	84.067	42.084	16.061	2245.929
-67.45	149.211	125.221	82.493	41.462	14.859	2245.929
-67.35	150.741	124.723	85.438	42.915	16.219	2245.929
-67.25	149.686	126.782	84.428	41.202	15.074	2245.929
-67.15	152.924	124.790	84.500	42.361	15.560	2245.929
-67.05	148.772	125.470	83.749	43.814	15.303	2245.929
-66.95	152.497	122.000	83.677	42.430	15.818	2245.929
-66.85	149.757	124.341	86.651	43.883	15.746	2245.929
-66.75	152.544	123.063	84.543	40.787	15.260	2245.929
-66.65	150.646	128.028	84.240	41.323	16.090	2245.929
-66.55	152.390	125.736	84.370	41.548	15.618	2245.929

Midpoint	10	3	1	0.33	0.1	Volume
-66.45	150.219	125.437	85.929	42.707	15.360	2245.929
-66.35	151.477	127.081	84.673	42.413	15.818	2245.929
-66.25	149.555	124.590	86.045	43.001	15.231	2245.929
-66.15	153.505	122.647	84.052	42.136	15.661	2245.929
-66.05	153.125	124.723	83.142	42.240	15.761	2245.929
-65.95	151.394	123.494	84.153	41.963	15.861	2245.929
-65.85	153.695	127.430	85.034	42.499	15.260	2245.929
-65.75	152.011	126.932	86.059	41.894	16.190	2245.929
-65.65	148.879	126.218	83.864	43.485	14.787	2245.929
-65.55	152.461	126.849	85.193	43.312	15.546	2245.929
-65.45	148.772	121.568	84.399	42.690	15.990	2245.929
-65.35	152.698	126.433	84.182	42.897	15.732	2245.929
-65.25	154.525	121.867	84.890	41.358	15.603	2245.929
-65.15	149.413	126.400	86.103	43.901	15.374	2245.929
-65.05	151.572	124.939	83.157	41.427	15.546	2245.929
-64.95	150.836	122.963	85.164	43.122	15.618	2245.929
-64.85	154.620	126.417	84.009	42.880	14.358	2245.929
-64.75	152.117	123.860	85.294	42.015	15.546	2245.929
-64.65	151.180	126.981	84.355	41.686	15.131	2245.929
-64.55	152.461	125.022	84.688	41.444	15.317	2245.929
-64.45	152.283	124.026	84.716	43.001	16.477	2245.929
-64.35	150.267	126.384	84.731	43.053	16.262	2245.929
-64.25	149.781	127.214	85.193	41.185	16.104	2245.929
-64.15	150.979	124.457	84.067	40.943	14.716	2245.929
-64.05	153.054	127.048	84.702	42.223	15.517	2245.929
-63.95	152.153	125.769	84.991	42.655	14.988	2245.929
-63.85	153.458	127.596	83.590	42.396	15.875	2245.929
-63.75	152.046	130.070	83.706	42.326	15.102	2245.929
-63.65	151.073	128.111	85.453	41.859	16.319	2245.929
-63.55	151.323	125.271	86.305	43.520	15.145	2245.929
-63.45	151.572	122.930	84.716	41.635	15.217	2245.929
-63.35	153.730	128.991	84.312	42.257	15.245	2245.929
-63.25	151.477	123.544	83.142	44.402	15.718	2245.929
-63.15	148.666	125.969	83.994	42.621	15.489	2245.929
-63.05	150.990	125.404	84.716	41.946	15.274	2245.929
-62.95	153.303	123.959	85.352	41.842	16.004	2245.929
-62.85	148.298	125.852	84.558	42.932	15.403	2245.929
-62.75	153.446	124.889	83.417	42.845	15.045	2245.929
-62.65	152.058	128.177	84.673	42.361	16.033	2245.929
-62.55	152.070	125.636	84.731	43.433	13.928	2245.929
-62.45	153.576	122.764	83.547	40.856	15.489	2245.929
-62.35	152.414	127.878	82.536	41.116	15.117	2245.929
-62.25	151.868	124.042	84.312	43.001	15.059	2245.929
-62.15	151.999	127.064	84.659	42.465	15.517	2245.929
-62.05	153.125	127.197	84.529	42.724	15.031	2245.929

Midpoint	10	3	1	0.33	0.1	Volume
-61.95	154.003	124.026	84.384	41.462	14.973	2245.929
-61.85	155.438	123.528	85.121	41.392	15.861	2245.929
-61.75	151.595	126.018	83.778	41.756	15.976	2245.929
-61.65	151.334	126.035	84.225	41.842	15.704	2245.929
-61.55	150.611	123.909	84.298	43.226	15.933	2245.929
-61.45	152.544	121.186	83.085	42.084	14.458	2245.929
-61.35	151.856	125.421	84.933	43.364	15.475	2245.929
-61.25	153.280	126.218	84.471	40.856	15.389	2245.929
-61.15	150.623	127.098	84.673	42.361	16.047	2245.929
-61.05	153.718	127.828	85.727	41.289	15.389	2245.929
-60.95	151.477	125.188	83.128	42.396	15.718	2245.929
-60.85	147.764	125.404	84.428	42.621	15.517	2245.929
-60.75	152.580	124.690	84.283	41.617	15.675	2245.929
-60.65	151.453	123.262	83.142	43.001	15.403	2245.929
-60.55	152.129	125.769	82.247	41.756	16.319	2245.929
-60.45	151.655	127.646	81.973	42.482	14.659	2245.929
-60.35	152.094	127.563	83.619	43.728	16.376	2245.929
-60.25	151.133	125.570	86.565	40.701	16.205	2245.929
-60.15	151.987	125.354	84.413	42.119	15.832	2245.929
-60.05	151.595	125.105	85.020	42.223	15.231	2245.929
-59.95	151.750	127.646	85.077	41.946	15.675	2245.929
-59.85	152.521	127.679	84.370	39.957	16.333	2245.929
-59.75	152.022	129.472	85.106	42.050	16.033	2245.929
-59.65	153.173	124.839	83.720	40.268	15.031	2245.929
-59.55	155.296	128.957	86.594	41.756	16.405	2245.929
-59.45	151.002	128.592	84.168	41.046	15.274	2245.929
-59.35	151.868	126.649	83.171	44.212	14.873	2245.929
-59.25	150.777	123.909	85.092	42.153	16.076	2245.929
-59.15	149.140	128.376	82.449	42.551	15.374	2245.929
-59.05	151.417	126.865	84.211	41.548	15.818	2245.929
-58.95	150.386	125.553	84.832	41.877	15.890	2245.929
-58.85	151.394	123.611	84.947	42.448	15.818	2245.929
-58.75	150.314	127.264	84.789	43.122	15.274	2245.929
-58.65	153.351	127.297	84.211	41.219	16.104	2245.929
-58.55	149.899	127.712	86.536	42.257	15.789	2245.929
-58.45	152.366	125.338	84.817	41.617	15.961	2245.929
-58.35	149.840	127.945	83.734	42.465	15.145	2245.929
-58.25	152.746	126.334	84.659	41.341	15.603	2245.929
-58.15	151.548	124.457	84.716	40.995	15.503	2245.929
-58.05	150.042	128.011	83.720	42.880	16.190	2245.929
-57.95	150.943	124.491	84.760	42.015	14.873	2245.929
-57.85	152.900	125.952	84.240	42.517	16.047	2245.929
-57.75	150.457	124.839	86.911	42.361	15.045	2245.929
-57.65	149.804	126.998	85.063	40.960	15.718	2245.929
-57.55	150.646	123.179	83.402	42.551	16.706	2245.929

Midpoint	10	3	1	0.33	0.1	Volume
-57.45	151.738	126.516	83.864	42.378	16.233	2245.929
-57.35	150.160	127.994	83.966	42.413	15.689	2245.929
-57.25	152.034	125.686	84.009	41.254	15.961	2245.929
-57.15	151.572	126.666	85.770	44.195	15.374	2245.929
-57.05	152.473	126.550	84.269	41.669	15.217	2245.929
-56.95	152.924	127.147	83.301	41.946	15.990	2245.929
-56.85	152.295	127.895	83.460	41.998	15.059	2245.929
-56.75	153.232	124.956	83.041	41.998	15.818	2245.929
-56.65	150.801	124.424	83.489	43.330	15.689	2245.929
-56.55	152.177	126.267	85.857	40.718	16.004	2245.929
-56.45	153.624	125.271	84.327	42.361	15.245	2245.929
-56.35	153.718	122.714	83.662	41.462	15.560	2245.929
-56.25	152.117	124.574	83.677	41.600	15.360	2245.929
-56.15	153.137	126.151	84.370	41.098	15.503	2245.929
-56.05	151.073	124.059	83.749	41.825	14.215	2245.929
-55.95	150.469	124.258	85.626	40.078	15.546	2245.929
-55.85	152.224	125.753	85.756	44.160	15.761	2245.929
-55.75	152.224	125.039	83.244	41.202	15.245	2245.929
-55.65	153.968	127.081	84.976	41.773	16.749	2245.929
-55.55	153.873	125.769	83.460	43.122	15.260	2245.929
-55.45	154.750	125.969	84.615	43.122	15.560	2245.929
-55.35	153.446	127.081	83.619	43.330	15.575	2245.929
-55.25	152.829	124.740	83.157	42.326	15.546	2245.929
-55.15	153.552	127.247	82.594	40.891	16.434	2245.929
-55.05	153.718	127.147	85.164	42.534	14.344	2245.929
-54.95	153.885	126.035	83.850	41.427	15.517	2245.929
-54.85	153.849	126.815	83.908	42.949	15.002	2245.929
-54.75	150.599	124.574	84.067	42.915	15.832	2245.929
-54.65	151.690	127.978	83.475	42.499	16.248	2245.929
-54.55	153.019	123.743	85.395	42.915	14.902	2245.929
-54.45	147.622	124.640	84.341	41.859	15.389	2245.929
-54.35	152.948	126.168	84.197	41.479	15.188	2245.929
-54.25	149.555	127.280	83.345	43.278	15.475	2245.929
-54.15	150.125	129.904	85.770	41.392	15.188	2245.929
-54.05	149.401	123.245	83.345	40.995	15.432	2245.929
-53.95	150.219	126.832	83.431	42.621	15.847	2245.929
-53.85	149.840	126.035	83.922	42.897	15.718	2245.929
-53.75	150.030	127.114	84.976	42.084	16.391	2245.929
-53.65	153.220	129.771	84.052	41.548	15.203	2245.929
-53.55	151.500	124.657	86.391	43.278	15.761	2245.929
-53.45	151.631	126.417	85.669	41.202	15.847	2245.929
-53.35	152.011	123.992	83.561	41.306	14.830	2245.929
-53.25	152.248	122.448	83.374	40.528	15.489	2245.929
-53.15	151.133	124.258	82.045	42.153	15.389	2245.929
-53.05	151.999	125.703	85.280	43.105	15.646	2245.929

Midpoint	10	3	1	0.33	0.1	Volume
-52.95	154.217	125.620	83.229	41.790	16.290	2245.929
-52.85	151.382	124.242	83.922	40.666	16.505	2245.929
-52.75	150.303	125.105	83.431	43.191	16.419	2245.929
-52.65	152.094	122.498	84.774	42.621	15.160	2245.929
-52.55	151.441	127.081	84.601	41.981	16.205	2245.929
-52.45	153.671	125.686	84.832	42.569	15.446	2245.929
-52.35	150.552	124.192	83.893	42.275	15.403	2245.929
-52.25	153.363	128.376	82.262	41.859	16.061	2245.929
-52.15	152.544	126.566	85.106	41.998	14.673	2245.929
-52.05	151.168	126.566	82.132	42.724	15.832	2245.929
-51.95	153.208	127.596	85.438	41.531	15.632	2245.929
-51.85	154.632	127.646	86.464	42.672	15.417	2245.929
-51.75	154.051	125.603	82.810	41.721	16.090	2245.929
-51.65	150.872	128.277	82.637	42.724	15.231	2245.929
-51.55	151.963	126.251	84.023	42.015	16.176	2245.929
-51.45	153.197	124.540	84.991	41.859	15.847	2245.929
-51.35	152.307	125.985	82.045	42.223	15.832	2245.929
-51.25	150.836	128.111	83.633	42.378	16.262	2245.929
-51.15	151.038	129.954	84.500	41.808	15.832	2245.929
-51.05	152.983	128.343	83.576	42.759	15.145	2245.929
-50.95	150.943	126.384	84.312	41.565	16.104	2245.929
-50.85	153.173	126.849	85.872	42.586	14.902	2245.929
-50.75	154.205	124.507	85.698	43.347	14.816	2245.929
-50.65	151.667	125.952	86.247	41.479	15.174	2245.929
-50.55	151.619	127.048	84.182	43.088	15.016	2245.929
-50.45	152.544	125.836	84.023	43.676	15.789	2245.929
-50.35	152.722	125.171	83.576	41.046	15.117	2245.929
-50.25	150.397	127.695	83.720	42.776	15.546	2245.929
-50.15	151.192	127.945	83.734	40.649	16.147	2245.929
-50.05	152.556	123.478	85.554	42.067	16.763	2245.929
-49.95	149.366	126.799	84.846	41.444	16.290	2245.929
-49.85	154.015	127.596	84.399	41.981	16.405	2245.929
-49.75	152.295	125.072	83.518	42.448	15.331	2245.929
-49.65	151.228	123.992	83.706	42.102	14.616	2245.929
-49.55	151.382	122.880	85.352	41.825	15.704	2245.929
-49.45	154.181	125.719	83.056	41.289	15.417	2245.929
-49.35	152.177	121.817	84.817	42.240	16.061	2245.929
-49.25	150.101	125.553	87.157	42.448	14.673	2245.929
-49.15	152.319	126.367	85.150	42.776	14.759	2245.929
-49.05	150.314	125.304	85.077	43.139	16.648	2245.929
-48.95	151.643	127.164	84.240	41.427	16.233	2245.929
-48.85	152.485	125.736	85.684	40.856	15.475	2245.929
-48.75	152.888	126.799	84.572	44.471	14.687	2245.929
-48.65	152.165	123.328	85.135	43.762	15.031	2245.929
-48.55	151.785	125.769	86.276	43.243	15.575	2245.929

Midpoint	10	3	1	0.33	0.1	Volume
-48.45	152.734	124.623	83.951	42.102	15.560	2245.929
-48.35	153.161	127.646	84.197	42.119	16.147	2245.929
-48.25	151.785	125.454	84.168	41.738	15.174	2245.929
-48.15	150.386	125.969	83.734	42.949	15.374	2245.929
-48.05	151.916	125.487	83.908	41.064	15.990	2245.929
-47.95	151.536	125.437	81.857	42.845	14.845	2245.929
-47.85	152.841	125.603	84.355	43.122	15.475	2245.929
-47.75	153.256	127.064	83.518	41.375	15.832	2245.929
-47.65	153.469	124.524	84.572	44.333	15.260	2245.929
-47.55	152.390	126.085	85.828	42.569	15.274	2245.929
-47.45	148.595	125.653	80.861	42.015	15.976	2245.929
-47.35	154.300	128.410	84.803	43.555	14.959	2245.929
-47.25	151.607	127.612	86.391	42.655	14.701	2245.929
-47.15	151.192	126.600	84.586	42.448	16.290	2245.929
-47.05	153.600	126.118	84.269	40.856	14.802	2245.929
-46.95	151.287	125.404	83.951	42.603	14.744	2245.929
-46.85	151.939	123.544	84.312	42.102	16.520	2245.929
-46.75	152.509	124.640	83.229	42.119	14.716	2245.929
-46.65	153.244	125.736	82.406	43.831	15.432	2245.929
-46.55	149.591	126.932	85.641	40.943	15.933	2245.929
-46.45	148.974	126.616	82.666	43.226	15.317	2245.929
-46.35	154.632	126.898	83.994	42.638	14.716	2245.929
-46.25	153.742	126.616	83.966	42.292	15.675	2245.929
-46.15	152.461	124.607	85.005	42.603	15.589	2245.929
-46.05	150.777	124.773	84.558	41.427	14.701	2245.929
-45.95	150.658	126.616	83.114	42.171	14.644	2245.929
-45.85	151.323	125.620	86.781	41.513	15.360	2245.929
-45.75	150.196	127.579	84.716	42.534	16.018	2245.929
-45.65	152.082	129.007	84.976	42.326	16.305	2245.929
-45.55	150.219	130.070	84.254	42.534	15.632	2245.929
-45.45	152.307	128.559	84.298	42.724	15.761	2245.929
-45.35	151.690	124.225	84.471	42.465	15.460	2245.929
-45.25	150.848	126.616	83.272	42.517	15.489	2245.929
-45.15	150.516	125.122	83.937	40.908	15.460	2245.929
-45.05	151.904	126.981	82.810	40.873	15.990	2245.929
-44.95	151.441	124.192	85.886	43.157	15.603	2245.929
-44.85	152.900	127.064	82.363	41.704	15.947	2245.929
-44.75	150.753	126.184	83.142	42.966	15.961	2245.929
-44.65	153.612	126.981	84.543	41.721	15.303	2245.929
-44.55	149.662	127.978	84.413	43.503	16.004	2245.929
-44.45	153.979	126.052	83.041	41.894	15.389	2245.929
-44.35	151.690	125.620	85.785	42.119	15.016	2245.929
-44.25	151.240	128.742	84.572	42.517	15.031	2245.929
-44.15	154.300	126.550	84.760	41.306	15.804	2245.929
-44.05	152.319	124.325	82.146	41.894	15.718	2245.929

Midpoint	10	3	1	0.33	0.1	Volume
-43.95	152.544	128.559	84.659	41.825	15.761	2245.929
-43.85	152.414	128.426	84.081	41.012	16.548	2245.929
-43.75	153.588	125.138	84.991	43.589	15.360	2245.929
-43.65	153.932	124.673	83.619	41.219	16.147	2245.929
-43.55	148.701	123.378	84.514	41.859	15.088	2245.929
-43.45	151.833	126.516	86.594	42.465	16.004	2245.929
-43.35	153.541	124.673	84.384	42.084	15.832	2245.929
-43.25	153.944	127.662	84.789	43.572	15.918	2245.929
-43.15	151.726	126.716	85.164	42.776	14.458	2245.929
-43.05	153.541	126.566	83.460	43.676	15.532	2245.929
-42.95	149.211	126.965	85.381	42.742	15.245	2245.929
-42.85	154.406	127.845	85.597	42.136	16.305	2245.929
-42.75	153.422	125.022	83.807	43.122	15.746	2245.929
-42.65	155.047	128.293	84.399	42.742	16.076	2245.929
-42.55	152.770	125.653	86.117	42.517	15.947	2245.929
-42.45	154.181	126.350	84.760	43.624	14.730	2245.929
-42.35	150.955	127.131	84.269	41.929	16.276	2245.929
-42.25	154.988	127.314	85.294	43.606	15.675	2245.929
-42.15	153.244	124.740	83.114	42.153	16.018	2245.929
-42.05	149.484	127.612	84.904	41.496	15.560	2245.929
-41.95	152.959	128.493	84.254	42.413	15.145	2245.929
-41.85	149.935	126.649	83.056	42.067	15.875	2245.929
-41.75	152.615	126.932	84.947	41.289	15.446	2245.929
-41.65	153.351	128.077	84.644	39.939	15.890	2245.929
-41.55	151.263	126.600	85.265	43.226	16.162	2245.929
-41.45	155.142	127.081	85.193	42.153	16.104	2245.929
-41.35	154.454	124.607	83.402	42.984	15.632	2245.929
-41.25	154.549	125.769	83.619	42.448	15.503	2245.929
-41.15	152.105	130.103	85.222	42.621	15.890	2245.929
-41.05	152.212	123.627	85.280	41.669	15.589	2245.929
-40.95	154.312	128.891	83.287	43.278	15.074	2245.929
-40.85	152.627	126.766	83.807	41.929	17.479	2245.929
-40.75	153.991	126.832	83.446	41.773	15.804	2245.929
-40.65	151.643	126.600	86.016	42.742	15.675	2245.929
-40.55	151.667	125.636	83.215	42.759	15.818	2245.929
-40.45	153.552	129.771	84.919	42.586	14.845	2245.929
-40.35	154.810	127.612	85.265	43.624	15.303	2245.929
-40.25	151.477	127.612	83.994	44.281	15.761	2245.929
-40.15	151.738	125.088	82.753	42.845	15.746	2245.929
-40.05	152.639	127.397	86.608	42.932	16.677	2245.929
-39.95	153.695	125.537	84.832	42.499	15.575	2245.929
-39.85	151.939	125.902	83.417	42.828	15.331	2245.929
-39.75	153.825	124.989	83.085	41.773	14.902	2245.929
-39.65	154.608	123.528	84.586	42.897	15.961	2245.929
-39.55	151.607	125.885	85.381	41.479	16.162	2245.929

Midpoint	10	3	1	0.33	0.1	Volume
-39.45	152.793	124.922	85.164	41.842	14.959	2245.929
-39.35	153.742	126.234	84.211	41.946	15.947	2245.929
-39.25	154.217	126.815	83.966	43.157	15.432	2245.929
-39.15	151.477	127.247	85.323	42.413	15.546	2245.929
-39.05	151.500	127.945	84.904	43.070	15.174	2245.929
-38.95	151.334	125.254	86.001	42.759	15.990	2245.929
-38.85	155.865	124.806	86.233	41.981	15.360	2245.929
-38.75	153.232	128.974	84.846	42.949	15.589	2245.929
-38.65	152.105	128.991	85.280	42.102	16.333	2245.929
-38.55	152.070	123.992	84.630	41.756	15.403	2245.929
-38.45	152.034	124.341	85.265	42.448	15.331	2245.929
-38.35	155.189	127.214	84.067	42.742	15.818	2245.929
-38.25	153.778	125.736	85.193	42.448	15.775	2245.929
-38.15	155.510	125.620	84.052	43.191	14.931	2245.929
-38.05	153.137	127.131	86.175	42.811	16.620	2245.929
-37.95	153.873	127.048	83.980	42.845	16.205	2245.929
-37.85	153.790	127.978	86.146	43.209	15.918	2245.929
-37.75	154.003	129.190	84.283	43.105	15.775	2245.929
-37.65	154.300	127.480	85.915	42.396	15.102	2245.929
-37.55	151.477	124.325	85.641	42.153	15.446	2245.929
-37.45	153.541	125.039	84.110	42.603	15.346	2245.929
-37.35	156.008	129.057	84.456	42.828	15.603	2245.929
-37.25	151.240	129.605	87.373	43.364	15.675	2245.929
-37.15	154.656	128.758	85.655	43.658	15.904	2245.929
-37.05	155.925	129.505	86.579	41.513	15.331	2245.929
-36.95	154.145	128.376	84.327	43.416	16.648	2245.929
-36.85	152.805	127.795	86.637	42.690	15.031	2245.929
-36.75	156.162	124.806	85.236	43.883	16.333	2245.929
-36.65	153.351	125.470	81.655	43.018	15.847	2245.929
-36.55	154.383	124.956	84.124	41.548	16.090	2245.929
-36.45	152.663	123.793	84.947	43.105	15.045	2245.929
-36.35	153.149	127.563	85.092	42.586	15.890	2245.929
-36.25	151.572	128.376	83.922	41.842	15.245	2245.929
-36.15	157.217	128.874	83.994	43.001	15.632	2245.929
-36.05	153.137	123.694	84.471	41.565	16.190	2245.929
-35.95	153.612	128.360	84.659	43.451	15.775	2245.929
-35.85	152.070	128.177	85.785	43.382	15.847	2245.929
-35.75	153.398	128.277	85.048	42.448	15.374	2245.929
-35.65	150.469	126.450	89.193	43.555	15.546	2245.929
-35.55	154.110	127.064	86.103	44.229	16.104	2245.929
-35.45	153.232	125.769	83.258	42.465	15.918	2245.929
-35.35	150.575	126.649	85.698	43.001	15.117	2245.929
-35.25	153.161	125.221	85.770	43.485	15.646	2245.929
-35.15	155.035	125.055	84.370	44.298	15.432	2245.929
-35.05	152.141	126.915	84.846	43.779	16.276	2245.929

Midpoint	10	3	1	0.33	0.1	Volume
-34.95	153.707	128.094	83.576	42.448	16.305	2245.929
-34.85	155.391	127.197	84.269	41.323	16.119	2245.929
-34.75	152.936	128.094	83.908	42.119	15.976	2245.929
-34.65	153.766	127.247	85.424	42.413	15.532	2245.929
-34.55	154.264	127.114	84.933	43.555	15.746	2245.929
-34.45	152.188	128.443	85.669	43.209	15.718	2245.929
-34.35	151.180	122.930	85.915	41.669	15.231	2245.929
-34.25	153.790	127.795	84.254	41.427	15.861	2245.929
-34.15	154.644	128.675	87.619	43.364	15.746	2245.929
-34.05	153.185	125.719	85.207	43.209	15.775	2245.929
-33.95	155.557	124.424	83.142	42.499	15.160	2245.929
-33.85	152.805	125.122	85.583	43.537	15.961	2245.929
-33.75	153.778	124.590	84.586	44.229	16.591	2245.929
-33.65	155.177	126.267	84.947	42.067	15.002	2245.929
-33.55	151.406	127.330	85.973	41.583	15.890	2245.929
-33.45	152.319	130.867	84.976	41.392	15.675	2245.929
-33.35	154.205	128.941	86.969	43.261	16.104	2245.929
-33.25	151.868	128.360	84.688	42.776	15.160	2245.929
-33.15	152.177	129.489	87.518	42.534	16.162	2245.929
-33.05	152.865	127.712	86.839	43.295	15.389	2245.929
-32.95	153.588	124.175	83.532	42.465	15.933	2245.929
-32.85	153.635	128.808	82.926	42.603	15.618	2245.929
-32.75	154.074	126.633	86.377	42.015	15.560	2245.929
-32.65	149.935	127.579	85.438	42.724	16.205	2245.929
-32.55	153.885	125.421	84.861	42.793	15.245	2245.929
-32.45	152.378	128.559	86.146	42.880	16.477	2245.929
-32.35	154.667	128.293	85.828	43.451	15.489	2245.929
-32.25	151.453	126.085	86.507	41.756	15.618	2245.929
-32.15	152.805	124.873	88.211	40.441	15.632	2245.929
-32.05	151.500	126.301	85.886	41.773	15.976	2245.929
-31.95	155.379	124.657	85.048	42.966	15.288	2245.929
-31.85	152.082	125.570	85.308	43.589	15.847	2245.929
-31.75	150.860	125.620	83.677	42.465	15.117	2245.929
-31.65	152.165	125.852	85.280	42.050	15.102	2245.929
-31.55	152.449	126.566	84.153	44.022	15.875	2245.929
-31.45	148.950	126.965	86.622	42.430	15.532	2245.929
-31.35	152.722	126.018	84.688	42.240	15.117	2245.929
-31.25	152.177	126.168	85.641	41.150	15.074	2245.929
-31.15	151.667	126.002	83.691	42.067	16.004	2245.929
-31.05	153.386	127.695	84.991	43.105	15.775	2245.929
-30.95	151.750	126.284	82.810	42.828	15.517	2245.929
-30.85	151.999	126.832	85.020	42.690	16.319	2245.929
-30.75	153.398	127.197	83.908	42.032	15.861	2245.929
-30.65	149.959	126.052	83.374	40.545	15.145	2245.929
-30.55	154.240	125.055	85.424	43.728	15.775	2245.929

Midpoint	10	3	1	0.33	0.1	Volume
-30.45	153.386	127.845	85.034	44.229	16.262	2245.929
-30.35	148.381	124.109	85.063	42.811	14.730	2245.929
-30.25	150.433	125.620	82.767	42.621	15.646	2245.929
-30.15	152.271	127.413	83.864	43.382	16.362	2245.929
-30.05	151.168	125.122	85.265	42.569	16.205	2245.929
-29.95	154.110	122.614	84.615	42.863	15.031	2245.929
-29.85	150.279	124.109	83.662	42.932	16.176	2245.929
-29.75	148.595	124.258	85.063	41.721	15.618	2245.929
-29.65	150.884	123.278	83.980	42.015	15.203	2245.929
-29.55	151.038	125.553	82.781	42.413	15.059	2245.929
-29.45	148.357	122.996	84.168	40.891	14.931	2245.929
-29.35	151.631	121.850	85.193	42.413	14.916	2245.929
-29.25	149.223	125.985	82.175	40.873	15.389	2245.929
-29.15	150.753	124.574	83.937	42.724	15.818	2245.929
-29.05	148.298	123.461	81.944	43.866	15.360	2245.929
-28.95	148.488	125.504	85.135	40.718	15.532	2245.929
-28.85	149.152	122.847	84.543	42.534	15.804	2245.929
-28.75	151.263	123.378	83.807	41.513	15.045	2245.929
-28.65	147.124	123.544	83.388	39.853	15.274	2245.929
-28.55	148.583	124.075	85.684	40.493	15.603	2245.929
-28.45	148.867	124.939	83.258	42.240	15.675	2245.929
-28.35	149.520	122.232	84.038	41.323	14.845	2245.929
-28.25	147.658	122.199	80.457	41.271	14.888	2245.929
-28.15	149.816	122.382	80.558	42.067	15.646	2245.929
-28.05	147.776	124.756	79.894	40.804	15.360	2245.929
-27.95	143.862	118.712	83.099	40.493	15.374	2245.929
-27.85	146.970	121.751	81.078	40.303	14.487	2245.929
-27.75	146.756	122.681	80.731	41.427	15.360	2245.929
-27.65	148.156	120.406	80.904	40.372	14.759	2245.929
-27.55	143.672	124.208	80.760	41.064	14.945	2245.929
-27.45	145.499	117.500	80.948	40.683	15.031	2245.929
-27.35	146.388	120.372	80.096	39.369	15.374	2245.929
-27.25	146.507	121.867	79.258	41.254	16.219	2245.929
-27.15	145.902	120.489	82.233	40.701	14.272	2245.929
-27.05	143.992	120.821	78.811	41.012	14.730	2245.929
-26.95	144.123	119.974	79.417	40.995	14.845	2245.929
-26.85	144.076	117.616	80.774	40.095	14.601	2245.929
-26.75	145.001	118.695	81.020	40.112	15.231	2245.929
-26.65	141.549	121.751	81.092	39.196	15.245	2245.929
-26.55	147.314	119.061	79.345	39.351	14.945	2245.929
-26.45	142.415	118.513	78.536	41.583	13.900	2245.929
-26.35	143.898	122.000	81.597	41.306	15.016	2245.929
-26.25	143.506	121.037	80.067	41.098	13.528	2245.929
-26.15	143.150	118.280	82.883	39.715	14.344	2245.929
-26.05	142.391	123.096	78.883	40.476	15.031	2245.929

Midpoint	10	3	1	0.33	0.1	Volume
-25.95	144.550	119.725	81.482	40.856	15.002	2245.929
-25.85	143.826	120.721	79.071	39.559	15.088	2245.929
-25.75	143.269	120.356	78.637	41.358	14.329	2245.929
-25.65	142.628	118.396	80.168	41.617	14.945	2245.929
-25.55	142.937	116.487	81.381	42.482	14.530	2245.929
-25.45	143.079	117.201	79.836	41.548	14.530	2245.929
-25.35	142.771	117.184	81.063	38.867	15.188	2245.929
-25.25	142.795	121.070	77.915	40.597	14.730	2245.929
-25.15	142.984	119.326	79.143	39.369	14.072	2245.929
-25.05	141.087	119.260	80.428	40.856	14.444	2245.929
-24.95	142.878	119.609	81.468	40.631	14.258	2245.929
-24.85	141.751	117.848	80.255	39.542	14.530	2245.929
-24.75	144.550	118.878	78.955	40.147	15.231	2245.929
-24.65	143.720	118.596	81.135	40.545	15.446	2245.929
-24.55	142.451	116.221	78.608	39.593	14.644	2245.929
-24.45	143.044	116.802	80.327	40.977	14.959	2245.929
-24.35	139.877	119.825	80.789	39.749	14.344	2245.929
-24.25	141.846	118.596	78.652	42.257	15.303	2245.929
-24.15	142.130	117.965	79.879	39.593	15.403	2245.929
-24.05	143.803	121.867	79.013	40.372	14.043	2245.929
-23.95	141.229	118.446	79.879	38.919	14.630	2245.929
-23.85	143.767	116.653	77.829	39.732	15.560	2245.929
-23.75	142.593	118.147	79.634	41.842	15.689	2245.929
-23.65	142.759	118.861	81.381	39.092	14.916	2245.929
-23.55	143.613	116.454	80.919	38.659	14.716	2245.929
-23.45	142.759	116.387	80.948	40.216	14.959	2245.929
-23.35	142.391	115.690	79.359	41.548	15.059	2245.929
-23.25	145.890	117.483	79.460	40.545	14.787	2245.929
-23.15	139.900	118.861	78.219	39.818	15.117	2245.929
-23.05	140.150	118.646	78.276	38.158	15.217	2245.929
-22.95	140.316	118.396	80.673	40.528	14.601	2245.929
-22.85	139.972	112.269	78.392	41.548	15.059	2245.929
-22.75	142.047	119.459	78.464	39.663	14.515	2245.929
-22.65	142.451	118.878	78.450	41.289	15.331	2245.929
-22.55	143.886	118.679	80.211	40.251	15.059	2245.929
-22.45	141.751	118.513	77.988	40.320	14.859	2245.929
-22.35	143.103	118.629	80.717	40.303	14.902	2245.929
-22.25	140.754	119.692	80.052	40.891	14.830	2245.929
-22.15	143.293	119.808	80.442	40.320	15.489	2245.929
-22.05	143.257	118.994	78.825	41.375	15.016	2245.929
-21.95	141.929	120.372	79.691	40.285	13.943	2245.929
-21.85	142.617	118.181	81.107	39.991	14.802	2245.929
-21.75	141.371	120.223	81.554	39.905	14.444	2245.929
-21.65	138.786	119.609	81.468	40.925	14.730	2245.929
-21.55	143.981	120.306	79.302	41.323	14.859	2245.929

Midpoint	10	3	1	0.33	0.1	Volume
-21.45	142.854	120.107	81.193	41.064	14.902	2245.929
-21.35	144.906	118.496	80.616	40.597	14.730	2245.929
-21.25	142.878	120.240	80.240	39.472	16.076	2245.929
-21.15	144.360	118.928	82.348	41.046	16.276	2245.929
-21.05	143.826	121.302	81.785	38.815	15.503	2245.929
-20.95	144.514	120.074	81.554	38.659	15.288	2245.929
-20.85	141.739	120.655	80.298	39.542	15.532	2245.929
-20.75	139.651	118.214	83.475	41.496	16.061	2245.929
-20.65	143.388	120.074	78.219	42.119	15.059	2245.929
-20.55	145.416	118.380	78.406	41.150	15.446	2245.929
-20.45	144.289	119.243	80.038	41.133	14.931	2245.929
-20.35	141.514	120.389	80.832	39.161	15.002	2245.929
-20.25	143.471	118.878	83.229	41.790	14.744	2245.929
-20.15	143.933	119.077	81.583	40.873	15.231	2245.929
-20.05	146.175	120.040	81.063	41.064	15.618	2245.929
-19.95	145.843	121.784	81.468	40.424	15.818	2245.929
-19.85	145.072	122.681	81.857	40.925	15.689	2245.929
-19.75	142.889	122.581	81.496	42.050	15.933	2245.929
-19.65	145.570	119.708	80.890	41.150	15.188	2245.929
-19.55	145.772	119.708	82.334	41.583	14.959	2245.929
-19.45	148.369	118.994	81.424	41.168	15.117	2245.929
-19.35	144.799	121.020	82.608	41.444	14.673	2245.929
-19.25	144.633	123.677	81.886	40.908	15.403	2245.929
-19.15	149.366	120.040	82.103	42.932	15.961	2245.929
-19.05	144.846	121.718	83.374	42.517	15.417	2245.929
-18.95	144.467	121.452	81.150	40.130	15.904	2245.929
-18.85	17.305	16.340	10.223	5.068	1.832	2245.929
-18.75	10.011	8.386	5.444	2.491	1.188	10313.629
-18.65	8.504	7.389	5.025	2.629	1.117	131.230
-18.55	9.868	7.589	5.213	2.629	0.988	123.728
-18.45	8.184	8.436	4.852	2.128	0.973	117.886
-18.35	7.816	6.626	5.126	2.439	0.888	112.997
-18.25	7.852	6.044	4.520	1.851	1.059	108.748
-18.15	7.733	6.609	4.447	2.197	0.759	104.969
-18.05	7.544	5.596	3.798	2.058	0.902	101.553
-17.95	7.733	6.044	4.216	2.179	0.873	98.430
-17.85	7.508	5.629	4.520	1.955	0.888	95.549
-17.75	6.274	5.912	4.187	1.937	0.730	92.873
-17.65	7.722	5.812	3.985	2.024	0.802	90.373
-17.55	6.441	5.480	3.567	1.782	0.716	88.026
-17.45	6.132	5.131	3.783	1.989	0.687	85.815
-17.35	6.737	4.932	3.220	1.816	0.716	83.724
-17.25	6.464	5.281	3.336	1.730	0.587	81.741
-17.15	5.705	4.915	3.321	1.626	0.673	79.857
-17.05	5.836	4.716	3.437	2.006	0.544	78.061

Midpoint	10	3	1	0.33	0.1	Volume
-16.95	6.025	5.579	3.610	1.764	0.644	76.347
-16.85	5.207	4.583	3.581	1.937	0.544	74.708
-16.75	5.409	4.749	3.249	2.006	0.759	73.138
-16.65	4.922	4.616	3.292	1.557	0.573	71.632
-16.55	5.646	4.799	2.787	1.661	0.501	70.186
-16.45	4.993	4.666	3.307	1.366	0.472	68.796
-16.35	5.077	4.600	2.830	1.626	0.401	67.457
-16.25	5.005	3.902	3.018	1.712	0.558	66.168
-16.15	4.804	4.351	3.234	1.505	0.644	64.924
-16.05	5.112	3.620	2.917	1.107	0.372	63.724
-15.95	5.029	4.052	2.787	1.401	0.544	62.565
-15.85	4.946	4.118	2.902	1.453	0.487	61.445
-15.75	4.792	4.317	2.512	1.090	0.587	60.361
-15.65	4.080	4.234	3.177	1.522	0.501	59.312
-15.55	4.483	4.218	2.902	1.401	0.501	58.297
-15.45	5.432	4.533	3.437	1.574	0.744	57.313
-15.35	6.713	6.227	3.465	1.557	0.587	56.359
-15.25	7.247	5.945	3.928	1.678	0.515	55.434
-15.15	6.986	6.244	3.725	1.712	0.458	54.537
-15.05	7.081	5.463	3.769	1.955	0.458	53.666
-14.95	7.140	5.812	3.812	1.920	0.601	52.820
-14.85	7.105	5.878	4.101	1.730	0.544	51.998
-14.75	7.152	5.696	4.014	1.453	0.515	51.200
-14.65	6.701	6.177	4.130	1.557	0.487	50.425
-14.55	7.413	5.945	4.274	1.937	0.558	49.670
-14.45	7.603	5.812	3.942	1.816	0.558	48.937
-14.35	7.674	5.297	3.740	1.816	0.644	48.223
-14.25	7.508	6.094	3.841	1.764	0.658	47.529
-14.15	7.128	5.745	3.697	1.885	0.573	46.853
-14.05	7.259	6.177	4.014	1.903	0.530	46.196
-13.95	7.745	6.493	3.682	1.712	0.587	45.555
-13.85	7.805	5.845	4.592	1.816	0.744	44.932
-13.75	7.852	6.011	3.870	1.851	0.558	44.324
-13.65	7.057	6.609	4.404	2.179	0.744	43.733
-13.55	8.208	6.094	4.678	1.955	0.673	43.156
-13.45	8.326	6.642	4.621	1.799	0.601	42.595
-13.35	8.445	6.277	4.722	2.110	0.816	42.048
-13.25	8.540	7.124	4.577	2.214	0.573	41.514
-13.15	8.492	7.057	4.866	2.006	0.644	40.994
-13.05	8.587	7.074	4.491	2.283	0.673	40.487
-12.95	9.536	6.659	4.361	2.681	0.630	39.993
-12.85	9.275	7.888	5.242	2.024	0.859	39.512
-12.75	8.908	7.854	4.982	2.318	0.802	39.042
-12.65	10.367	8.568	5.646	2.318	0.859	38.584
-12.55	9.868	7.971	5.343	2.733	0.845	38.137

Midpoint	10	3	1	0.33	0.1	Volume
-12.45	10.200	8.087	5.169	2.249	0.515	37.702
-12.35	10.734	9.133	6.036	2.802	0.816	37.277
-12.25	11.636	8.585	6.440	3.114	0.873	36.863
-12.15	10.817	9.631	6.397	3.304	0.945	36.459
-12.05	12.181	10.163	6.122	3.217	0.973	36.066
-11.95	12.039	9.299	6.844	3.511	0.945	35.682
-11.85	13.581	10.926	6.989	3.356	1.102	35.307
-11.75	13.522	11.175	7.610	3.805	1.117	34.942
-11.65	14.636	11.624	7.696	3.944	1.474	34.586
-11.55	14.352	11.790	8.230	3.978	1.088	34.239
-11.45	15.514	12.836	9.184	4.324	1.403	33.900
-11.35	17.270	12.985	9.140	4.515	1.675	33.570
-11.25	17.104	15.393	10.122	4.653	1.646	33.249
-11.15	18.527	15.426	10.613	5.397	1.589	32.935
-11.05	19.061	17.236	10.830	5.518	1.875	32.630
-10.95	20.662	18.764	10.902	5.916	1.847	32.332
-10.85	21.931	18.249	12.577	5.898	2.290	32.042
-10.75	23.592	19.810	13.544	6.019	2.147	31.759
-10.65	24.410	19.727	14.656	6.141	2.491	31.484
-10.55	26.924	22.650	14.411	7.576	2.233	31.216
-10.45	29.356	23.713	16.432	7.732	2.963	30.955
-10.35	28.870	24.891	17.688	8.199	3.035	30.701
-10.25	33.306	27.067	17.111	8.285	3.063	30.454
-10.15	34.100	29.242	19.883	10.119	3.049	30.213
-10.05	35.559	30.670	20.547	10.776	3.607	29.979
-9.95	38.904	33.227	21.962	11.572	3.865	29.751
-9.85	40.529	33.958	24.229	12.281	3.665	29.530
-9.75	42.000	35.120	24.778	12.506	4.566	29.315
-9.65	46.792	40.152	27.969	13.025	4.853	29.106
-9.55	48.523	42.560	28.446	14.824	5.268	28.903
-9.45	51.062	46.063	30.684	15.689	5.368	28.706
-9.35	53.351	49.932	32.503	16.104	6.227	28.515
-9.25	58.249	51.942	36.070	18.871	6.528	28.329
-9.15	63.029	51.493	38.582	18.612	6.714	28.150
-9.05	64.488	57.023	38.857	20.878	7.744	27.975
-8.95	67.952	56.242	42.943	22.798	7.873	27.807
-8.85	73.918	63.748	44.098	24.372	8.417	27.643
-8.75	77.381	67.302	48.719	25.219	8.346	27.485
-8.65	80.702	71.138	51.000	27.468	9.978	27.332
-8.55	84.213	73.678	52.978	28.108	9.949	27.185
-8.45	86.751	78.876	55.780	28.627	11.008	27.042
-8.35	89.978	83.824	57.382	30.962	12.110	26.905
-8.25	92.717	87.095	56.920	31.118	11.423	26.772
-8.15	91.223	83.110	59.057	32.052	12.926	26.645
-8.05	91.614	82.230	59.577	32.536	12.139	26.522

Midpoint	10	3	1	0.33	0.1	Volume
-7.95	93.951	83.874	59.317	34.266	13.456	26.405
-7.85	91.519	82.496	59.606	33.712	12.683	26.292
-7.75	94.034	80.055	58.841	34.162	11.967	26.183
-7.65	94.259	81.732	58.812	33.055	12.282	26.080
-7.55	88.815	74.957	57.845	31.014	12.569	25.981
-7.45	83.051	71.353	54.523	30.841	12.726	25.886
-7.35	80.168	68.497	51.982	30.720	11.953	25.797
-7.25	76.053	72.018	51.145	29.129	12.468	25.711
-7.15	74.428	69.377	49.859	28.783	12.025	25.631
-7.05	72.554	64.695	51.072	26.724	11.423	25.554
-6.95	68.497	58.883	47.333	25.462	11.223	25.482
-6.85	65.864	55.993	44.026	23.282	11.252	25.415
-6.75	57.810	57.853	42.799	22.417	10.078	25.352
-6.65	59.851	52.938	43.737	23.057	9.405	25.293
-6.55	59.673	50.680	42.207	22.953	9.219	25.238
-6.45	59.317	43.921	38.885	24.061	7.616	25.188
-6.35	54.300	44.469	35.738	21.258	7.258	25.142
-6.25	46.471	40.285	35.925	19.926	7.186	25.100
-6.15	45.878	37.096	34.120	20.221	7.515	25.063
-6.05	44.396	36.831	29.153	17.764	6.728	25.030
-5.95	39.972	36.266	27.117	15.879	6.342	25.000
-5.85	39.936	31.168	23.175	15.862	5.869	24.975
-5.75	36.152	30.803	20.966	14.772	6.184	24.955
-5.65	34.183	26.618	19.753	14.115	5.483	24.938
-5.55	31.906	23.032	18.468	12.125	5.125	24.926
-5.45	23.508	19.860	15.060	10.378	4.051	24.917
-5.35	15.977	14.397	10.570	7.040	3.550	24.913
-5.25	15.301	13.052	11.205	7.075	3.164	24.913
-5.15	14.886	12.354	10.945	5.985	2.777	24.913
-5.05	12.774	11.939	9.617	5.293	2.949	24.913
-4.95	12.252	11.425	8.606	5.501	2.519	24.913
-4.85	12.110	11.258	8.909	5.310	2.505	24.913
-4.75	12.086	10.993	9.357	5.362	2.434	24.913
-4.65	12.276	10.445	9.790	4.826	2.548	24.913
-4.55	11.897	11.491	9.631	5.535	2.362	24.913
-4.45	11.873	11.142	9.819	4.964	2.448	24.913
-4.35	12.537	10.279	8.924	5.950	2.391	24.913
-4.25	13.830	10.329	7.725	5.777	2.262	24.913
-4.15	13.711	11.557	8.259	5.120	2.205	24.913
-4.05	14.174	11.076	8.707	5.691	2.691	24.913
-3.95	14.055	9.830	7.971	5.328	2.562	24.913
-3.85	12.964	10.561	8.303	5.743	2.748	24.913
-3.75	14.115	10.445	9.804	5.691	2.462	24.913
-3.65	13.462	11.408	10.613	6.227	2.519	24.913
-3.55	13.818	12.354	11.234	6.106	2.548	24.913

Midpoint	10	3	1	0.33	0.1	Volume
-3.45	14.542	13.733	11.378	6.331	2.763	24.913
-3.35	15.443	14.447	11.104	5.916	3.178	24.913
-3.25	15.562	15.028	12.505	7.576	3.722	24.913
-3.15	16.178	17.934	12.606	6.971	3.779	24.913
-3.05	18.052	15.792	14.122	7.248	3.937	24.913
-2.95	18.906	18.415	15.161	8.510	3.908	24.913
-2.85	19.950	19.345	15.060	8.822	3.836	24.913
-2.75	21.030	18.532	15.826	8.804	4.023	24.913
-2.65	20.543	20.458	16.187	8.441	4.509	24.913
-2.55	21.753	21.969	15.840	9.929	4.738	24.913
-2.45	24.766	23.829	17.298	10.655	5.096	24.913
-2.35	27.138	26.469	19.262	11.641	5.225	24.913
-2.25	26.414	27.980	20.201	12.575	5.282	24.913
-2.15	29.380	28.296	21.948	12.852	5.883	24.913
-2.05	33.294	28.412	22.165	14.737	7.229	24.913
-1.95	34.112	30.355	25.717	16.000	7.973	24.913
-1.85	34.942	33.626	27.334	17.972	7.730	24.913
-1.75	36.698	36.565	29.413	18.456	8.532	24.913
-1.65	40.043	37.030	34.929	20.774	9.562	24.913
-1.55	43.589	40.501	36.806	23.334	11.209	24.913
-1.45	45.890	46.761	39.867	25.289	12.096	24.913
-1.35	52.378	51.228	43.015	27.762	11.939	24.913
-1.25	56.565	53.702	43.708	29.094	13.127	24.913
-1.15	59.910	62.337	46.538	32.830	14.630	24.913
-1.05	66.256	67.152	49.527	36.670	15.646	24.913
-0.95	70.739	68.514	55.982	38.486	16.477	24.913
-0.85	78.176	71.586	58.364	38.936	17.321	24.913
-0.75	76.990	73.230	60.935	41.046	17.665	24.913
-0.65	82.671	75.322	64.747	44.195	18.738	24.913
-0.55	85.221	79.889	67.259	43.330	18.581	24.913
-0.45	89.100	86.664	68.761	45.146	18.538	24.913
-0.35	87.404	86.282	72.443	43.814	18.252	24.913
-0.25	88.186	86.431	71.966	44.541	18.609	24.913
-0.05	93.014	83.658	67.880	45.215	18.796	24.913
0.05	91.448	81.632	68.097	43.658	18.910	24.913
0.25	88.566	78.925	64.342	41.341	17.951	24.913
0.35	85.375	75.687	59.996	41.289	16.677	24.913
0.45	81.449	72.964	57.108	39.023	15.704	24.913
0.55	79.350	71.038	54.292	36.584	13.757	24.913
0.65	75.448	65.193	53.426	32.692	14.258	24.913
0.75	69.719	63.167	49.354	31.706	12.325	24.913
0.85	64.761	56.873	44.531	28.056	12.239	24.913
0.95	55.308	52.025	40.503	25.756	11.423	24.913
1.05	54.454	48.836	36.662	23.922	9.992	24.913
1.15	49.603	45.432	33.759	22.002	9.247	24.913

Midpoint	10	3	1	0.33	0.1	Volume
1.25	43.411	39.969	30.525	19.165	8.059	24.913
1.35	41.739	34.655	27.348	17.574	7.444	24.913
1.45	37.196	32.347	26.309	15.948	7.773	24.913
1.55	34.658	29.690	23.146	14.910	6.284	24.913
1.65	31.135	27.532	21.038	13.821	5.454	24.913
1.75	29.000	25.107	20.013	12.886	5.769	24.913
1.85	27.648	24.094	18.930	12.177	4.982	24.913
1.95	26.474	22.351	16.865	10.828	4.209	24.913
2.05	25.050	20.906	16.360	10.655	3.779	24.913
2.15	22.370	19.794	14.873	9.704	3.908	24.913
2.25	20.413	19.030	14.093	9.029	3.536	24.913
2.35	18.966	16.174	13.703	7.957	3.378	24.913
2.45	19.250	16.024	12.764	7.334	3.078	24.913
2.55	18.420	14.862	11.133	7.057	2.877	24.913
2.65	16.902	14.364	11.451	7.559	2.963	24.913
2.75	16.226	13.716	10.469	7.351	2.519	24.913
2.85	15.668	14.115	10.685	7.023	2.462	24.913
2.95	16.297	13.284	10.165	6.123	2.706	24.913
3.05	14.814	13.500	9.905	6.071	2.162	24.913
3.15	14.198	12.454	9.660	6.538	2.462	24.913
3.25	13.664	13.052	9.530	5.622	2.419	24.913
3.35	14.221	11.292	9.285	5.397	2.290	24.913
3.45	14.589	12.022	9.227	5.431	2.133	24.913
3.55	13.616	12.653	8.418	5.397	2.333	24.913
3.65	13.462	11.192	9.212	5.189	2.205	24.913
3.75	13.462	12.288	9.010	5.258	1.975	24.913
3.85	13.533	11.740	8.548	5.414	1.847	24.913
3.95	12.573	11.458	8.115	4.930	2.305	24.913
4.05	13.118	11.491	8.620	4.757	1.789	24.913
4.15	13.000	10.910	8.360	4.688	2.162	24.913
4.25	13.000	11.690	8.418	5.085	1.661	24.913
4.35	13.344	11.142	8.375	4.774	1.832	24.913
4.45	12.964	11.009	8.274	4.826	1.832	24.913
4.55	12.834	11.541	8.389	4.272	1.933	24.913
4.65	12.786	10.744	8.317	5.120	1.689	24.913
4.75	12.407	11.275	8.245	4.480	1.589	24.913
4.85	12.786	11.491	8.360	4.497	1.489	24.913
4.95	12.739	11.009	7.740	4.774	1.603	24.913
5.05	12.691	10.910	8.577	4.601	1.661	24.913
5.15	13.106	11.009	7.653	4.445	1.360	24.913
5.25	12.810	10.777	7.667	4.705	1.675	24.913
5.35	12.537	11.840	7.971	4.636	1.575	24.913
5.45	25.584	22.168	15.941	7.957	3.106	24.917
5.55	27.589	23.165	17.573	9.496	3.350	24.926
5.65	27.446	23.331	17.096	9.790	3.450	24.938

Midpoint	10	3	1	0.33	0.1	Volume
5.75	28.051	23.845	17.919	8.925	3.622	24.955
5.85	26.723	25.323	17.313	9.721	3.779	24.975
5.95	28.846	25.091	17.530	9.652	3.722	25.000
6.05	28.597	23.995	17.631	9.150	3.235	25.030
6.15	29.166	24.725	18.006	9.617	3.421	25.063
6.25	28.561	25.290	18.179	9.565	3.507	25.100
6.35	28.739	24.626	17.775	9.704	3.793	25.142
6.45	29.320	27.000	18.483	9.756	3.693	25.188
6.55	30.518	25.406	18.483	9.911	3.650	25.238
6.65	29.593	27.283	18.714	9.635	3.393	25.293
6.75	30.400	26.237	18.150	10.430	3.679	25.352
6.85	32.025	26.369	18.742	10.603	3.708	25.415
6.95	31.740	27.748	19.999	10.448	4.194	25.482
7.05	32.713	27.665	19.695	10.482	3.765	25.554
7.15	33.187	30.272	18.872	10.949	3.793	25.631
7.25	34.183	28.412	20.446	10.292	3.292	25.711
7.35	34.278	29.757	20.807	10.690	3.679	25.797
7.45	34.954	29.425	19.926	10.413	3.980	25.886
7.55	33.472	30.006	20.937	10.188	3.536	25.981
7.65	33.353	29.607	20.793	10.119	3.793	26.080
7.75	33.495	28.329	19.551	10.655	3.407	26.183
7.85	33.365	28.395	20.013	9.375	4.538	26.292
7.95	32.748	29.408	19.508	9.635	3.922	26.405
8.05	32.131	27.615	19.508	9.998	3.335	26.522
8.15	33.104	28.744	19.176	10.015	3.106	26.645
8.25	31.218	26.901	19.017	9.237	2.978	26.772
8.35	30.079	25.141	18.020	8.960	2.834	26.905
8.45	29.024	24.842	17.977	8.545	3.178	27.042
8.55	28.431	23.663	16.793	8.164	2.677	27.185
8.65	26.853	22.351	15.320	7.628	2.877	27.332
8.75	24.434	21.272	14.483	7.888	2.147	27.485
8.85	23.983	20.059	13.819	7.040	2.104	27.643
8.95	22.382	18.648	13.544	6.435	2.247	27.807
9.05	21.207	17.917	12.086	5.950	2.119	27.975
9.15	20.057	16.821	11.422	5.881	2.348	28.150
9.25	18.883	15.526	11.552	5.708	1.804	28.329
9.35	18.313	15.161	9.905	4.930	1.632	28.515
9.45	17.281	14.131	9.256	4.964	1.732	28.706
9.55	16.475	13.567	9.386	3.996	1.503	28.903
9.65	15.763	12.238	8.476	4.497	1.188	29.106
9.75	14.779	12.437	8.779	3.926	1.231	29.315
9.85	14.589	11.956	7.436	3.840	1.288	29.530
9.95	13.735	10.976	7.277	3.736	1.446	29.751
10.05	13.711	9.963	7.162	3.615	0.988	29.979
10.15	13.249	9.448	6.888	3.650	1.002	30.213

Midpoint	10	3	1	0.33	0.1	Volume
10.25	11.790	10.163	6.830	3.304	0.945	30.454
10.35	11.659	10.295	6.685	2.491	0.988	30.701
10.45	11.387	9.199	5.992	3.044	1.002	30.955
10.55	10.900	8.668	6.281	2.612	0.959	31.216
10.65	10.165	8.269	5.805	2.474	0.873	31.484
10.75	10.117	7.987	5.386	2.802	0.816	31.759
10.85	9.690	7.174	5.126	2.491	0.830	32.042
10.95	9.346	7.572	5.097	2.301	0.816	32.332
11.05	8.694	6.974	5.285	2.560	0.959	32.630
11.15	8.730	6.709	4.289	2.162	0.601	32.935
11.25	8.564	6.609	4.548	2.214	0.773	33.249
11.35	8.220	6.343	4.736	2.024	0.616	33.570
11.45	8.374	6.493	4.491	1.868	0.630	33.900
11.55	7.342	6.343	4.779	1.955	0.644	34.239
11.65	7.662	6.144	3.595	1.920	0.515	34.586
11.75	6.903	6.576	4.043	1.782	0.544	34.942
11.85	7.045	5.862	4.000	1.851	0.644	35.307
11.95	6.773	6.011	3.812	1.782	0.630	35.682
12.05	7.022	5.364	3.884	1.972	0.573	36.066
12.15	6.879	5.596	3.899	1.868	0.444	36.459
12.25	6.441	5.546	3.379	1.661	0.601	36.863
12.35	6.417	5.330	3.668	1.591	0.630	37.277
12.45	6.784	5.729	3.047	1.661	0.458	37.702
12.55	6.524	5.546	3.191	1.609	0.487	38.137
12.65	6.097	4.633	3.032	1.539	0.487	38.584
12.75	5.764	4.251	3.191	1.609	0.444	39.042
12.85	6.215	4.749	2.989	1.868	0.515	39.512
12.95	6.357	5.214	3.162	1.695	0.587	39.993
13.05	5.824	4.683	2.917	1.349	0.444	40.487
13.15	5.847	4.301	3.018	1.574	0.501	40.994
13.25	5.978	5.164	3.018	1.401	0.372	41.514
13.35	5.563	5.015	2.946	1.470	0.315	42.048
13.45	5.575	4.666	3.422	1.522	0.458	42.595
13.55	5.610	4.450	2.873	1.332	0.530	43.156
13.65	6.108	4.733	3.220	1.539	0.286	43.733
13.75	5.551	4.683	3.177	1.453	0.415	44.324
13.85	5.551	4.500	2.873	1.695	0.358	44.932
13.95	5.990	4.185	3.292	1.176	0.401	45.555
14.05	5.729	4.218	2.787	1.228	0.415	46.196
14.15	5.207	4.151	3.263	1.297	0.401	46.853
14.25	5.515	4.102	3.003	1.522	0.487	47.529
14.35	5.100	4.832	3.321	1.245	0.415	48.223
14.45	5.456	4.434	2.960	1.539	0.444	48.937
14.55	5.587	4.151	2.960	1.470	0.329	49.670
14.65	5.670	5.048	2.859	1.263	0.487	50.425

Midpoint	10	3	1	0.33	0.1	Volume
14.75	5.409	4.168	2.556	1.263	0.415	51.200
14.85	5.741	4.782	3.220	1.280	0.530	51.998
14.95	5.705	4.185	3.018	1.539	0.487	52.820
15.05	5.966	4.932	3.307	1.297	0.630	53.666
15.15	5.800	4.882	2.975	1.401	0.587	54.537
15.25	6.156	4.782	3.119	1.488	0.401	55.434
15.35	5.515	4.566	2.845	1.349	0.329	56.359
15.45	4.685	4.118	3.076	1.470	0.530	57.313
15.55	3.938	3.487	2.440	1.055	0.401	58.297
15.65	4.009	3.388	2.383	1.124	0.387	59.312
15.75	4.139	3.437	2.339	1.626	0.501	60.361
15.85	3.807	3.703	2.512	1.055	0.515	61.445
15.95	4.317	3.819	2.195	1.332	0.387	62.565
16.05	4.934	3.404	2.859	1.176	0.372	63.724
16.15	4.068	3.504	2.873	1.280	0.487	64.924
16.25	4.827	3.487	2.512	1.159	0.558	66.168
16.35	4.744	3.985	2.888	1.228	0.487	67.457
16.45	4.922	4.234	2.614	1.228	0.401	68.796
16.55	4.650	4.118	3.278	1.315	0.644	70.186
16.65	4.816	4.550	2.787	1.436	0.487	71.632
16.75	4.875	4.882	3.003	1.332	0.544	73.138
16.85	4.863	4.500	3.076	1.695	0.658	74.708
16.95	4.673	3.985	2.931	1.505	0.530	76.347
17.05	5.302	4.334	3.003	1.418	0.458	78.061
17.15	5.551	5.297	3.220	1.574	0.701	79.857
17.25	5.124	4.699	3.090	1.885	0.487	81.741
17.35	6.263	4.882	3.090	1.920	0.816	83.724
17.45	5.444	4.965	3.379	1.920	0.716	85.815
17.55	6.156	5.031	3.104	1.661	0.701	88.026
17.65	6.452	5.397	3.278	1.937	0.730	90.373
17.75	7.093	5.148	3.003	1.937	0.644	92.873
17.85	6.512	5.745	3.711	2.041	0.816	95.549
17.95	6.085	6.410	4.130	1.834	0.601	98.430
18.05	6.725	6.393	3.884	2.093	0.816	101.553
18.15	7.069	5.912	4.520	1.816	0.845	104.969
18.25	7.283	6.692	4.693	2.301	0.787	108.748
18.35	7.472	6.841	4.404	2.456	0.644	112.997
18.45	7.698	6.825	4.996	2.283	0.816	117.886
18.55	8.398	7.705	4.650	2.301	0.873	123.728
18.65	9.169	7.805	5.400	2.128	0.973	131.230
18.75	9.667	8.519	5.083	2.802	0.973	10313.629
18.85	17.293	13.949	9.357	5.224	1.675	2245.929
18.95	140.648	120.456	81.164	43.105	15.961	2245.929
19.05	145.333	119.393	80.182	41.583	15.689	2245.929
19.15	146.329	118.479	81.150	39.351	16.104	2245.929

Midpoint	10	3	1	0.33	0.1	Volume
19.25	144.360	119.343	78.883	41.098	14.458	2245.929
19.35	148.108	118.313	81.482	41.929	15.217	2245.929
19.45	145.380	120.788	81.655	41.341	15.789	2245.929
19.55	142.617	121.701	81.150	40.199	15.389	2245.929
19.65	144.336	120.505	81.280	41.479	14.916	2245.929
19.75	143.838	121.884	81.626	41.462	15.432	2245.929
19.85	143.174	118.712	81.670	41.981	15.245	2245.929
19.95	142.700	118.845	81.352	39.593	15.174	2245.929
20.05	143.340	119.027	80.702	40.631	14.115	2245.929
20.15	142.842	119.808	80.514	42.084	14.501	2245.929
20.25	141.312	119.459	81.049	39.749	14.888	2245.929
20.35	145.404	116.155	81.280	38.919	15.818	2245.929
20.45	141.988	119.376	80.211	41.323	14.673	2245.929
20.55	143.399	118.131	79.302	39.178	15.131	2245.929
20.65	142.569	119.609	81.915	42.153	14.859	2245.929
20.75	141.395	119.991	79.879	40.925	16.104	2245.929
20.85	139.284	119.891	80.413	40.683	14.587	2245.929
20.95	146.554	119.791	77.684	40.493	14.859	2245.929
21.05	142.166	117.500	80.644	40.078	15.160	2245.929
21.15	142.118	121.037	78.897	40.545	14.973	2245.929
21.25	146.412	117.749	79.417	40.424	14.701	2245.929
21.35	140.648	115.690	80.558	39.888	15.274	2245.929
21.45	141.442	118.164	79.172	40.061	14.931	2245.929
21.55	143.079	118.413	81.828	39.455	15.160	2245.929
21.65	142.569	118.380	79.619	39.455	15.188	2245.929
21.75	142.617	116.869	79.099	39.905	14.802	2245.929
21.85	142.818	120.356	79.735	39.957	15.074	2245.929
21.95	142.190	120.622	79.273	40.009	14.931	2245.929
22.05	142.688	119.094	79.865	42.188	15.160	2245.929
22.15	142.451	118.015	79.980	40.873	14.802	2245.929
22.25	142.534	117.101	79.056	40.216	14.301	2245.929
22.35	141.822	118.396	79.504	40.528	14.988	2245.929
22.45	143.767	118.994	79.432	40.735	15.145	2245.929
22.55	143.103	115.424	80.428	38.573	14.115	2245.929
22.65	142.047	116.072	79.432	40.701	15.203	2245.929
22.75	143.423	118.546	78.840	41.202	14.129	2245.929
22.85	143.376	117.799	78.493	39.680	15.675	2245.929
22.95	140.161	118.280	78.883	38.296	14.243	2245.929
23.05	141.300	120.987	79.879	39.351	14.988	2245.929
23.15	141.015	121.136	79.460	39.351	14.859	2245.929
23.25	143.162	118.280	78.175	40.701	14.558	2245.929
23.35	141.822	116.636	80.052	38.054	14.802	2245.929
23.45	141.312	118.197	78.840	40.043	14.286	2245.929
23.55	139.058	114.079	80.413	40.095	15.446	2245.929
23.65	142.142	119.027	80.341	40.441	15.131	2245.929

Midpoint	10	3	1	0.33	0.1	Volume
23.75	142.284	116.653	80.500	40.891	15.331	2245.929
23.85	139.201	120.754	78.594	39.576	15.632	2245.929
23.95	144.336	117.085	80.471	39.593	15.346	2245.929
24.05	144.396	116.238	79.258	39.317	13.971	2245.929
24.15	141.431	116.122	79.201	40.873	14.673	2245.929
24.25	142.059	116.371	78.998	40.718	13.914	2245.929
24.35	140.826	115.540	80.948	39.005	13.843	2245.929
24.45	141.715	120.157	80.486	39.403	14.329	2245.929
24.55	140.031	114.810	79.273	40.147	15.117	2245.929
24.65	138.560	117.018	80.168	40.406	13.886	2245.929
24.75	142.000	118.679	80.168	38.953	14.215	2245.929
24.85	142.083	116.669	79.229	40.649	14.515	2245.929
24.95	142.059	117.566	79.432	39.766	14.430	2245.929
25.05	142.676	119.758	79.735	40.268	14.458	2245.929
25.15	139.616	116.105	78.955	40.355	15.317	2245.929
25.25	142.296	116.985	79.085	41.254	14.931	2245.929
25.35	143.257	119.360	81.699	39.213	15.203	2245.929
25.45	142.854	122.581	80.370	41.808	14.859	2245.929
25.55	143.044	119.526	79.330	40.631	15.059	2245.929
25.65	142.569	119.775	78.493	39.593	15.704	2245.929
25.75	143.826	119.492	79.071	40.130	14.072	2245.929
25.85	143.138	118.695	79.460	41.202	14.888	2245.929
25.95	143.044	119.775	80.009	40.199	14.616	2245.929
26.05	143.708	119.376	81.944	40.389	13.857	2245.929
26.15	143.364	119.725	80.009	40.441	15.360	2245.929
26.25	143.233	121.219	80.269	40.233	14.544	2245.929
26.35	144.823	120.206	80.962	38.971	15.231	2245.929
26.45	143.388	116.653	80.442	40.182	14.687	2245.929
26.55	142.652	121.120	80.558	40.856	15.059	2245.929
26.65	144.586	120.389	79.764	41.237	15.031	2245.929
26.75	145.262	120.754	80.269	41.150	15.933	2245.929
26.85	142.154	120.240	80.168	39.645	15.016	2245.929
26.95	145.297	117.400	80.746	41.756	14.988	2245.929
27.05	144.253	121.336	80.919	40.147	15.303	2245.929
27.15	149.366	120.970	81.915	41.323	14.945	2245.929
27.25	143.684	121.103	80.024	42.050	14.816	2245.929
27.35	144.170	121.967	81.395	41.116	15.231	2245.929
27.45	147.385	119.260	81.800	41.271	15.875	2245.929
27.55	148.559	121.917	82.247	41.877	14.057	2245.929
27.65	143.388	120.588	80.630	40.718	15.689	2245.929
27.75	148.891	119.110	81.569	42.275	14.945	2245.929
27.85	147.824	118.264	80.529	42.257	14.057	2245.929
27.95	146.056	124.441	81.843	42.759	15.360	2245.929
28.05	147.432	121.402	83.157	41.029	13.614	2245.929
28.15	146.578	124.590	81.886	42.621	14.787	2245.929

Midpoint	10	3	1	0.33	0.1	Volume
28.25	147.207	123.992	82.493	43.226	14.816	2245.929
28.35	152.117	124.291	80.962	40.406	15.761	2245.929
28.45	148.120	125.935	81.655	40.787	14.902	2245.929
28.55	146.839	123.063	81.944	40.873	14.773	2245.929
28.65	151.441	121.502	83.503	42.136	15.217	2245.929
28.75	152.544	125.553	84.197	41.773	15.890	2245.929
28.85	148.488	123.345	83.272	41.756	15.804	2245.929
28.95	148.393	124.109	84.168	41.652	15.245	2245.929
29.05	150.718	124.258	82.377	43.261	15.016	2245.929
29.15	150.077	127.347	83.850	42.188	15.918	2245.929
29.25	149.887	126.085	84.384	42.119	15.933	2245.929
29.35	151.809	124.059	82.493	43.364	15.059	2245.929
29.45	150.469	120.738	84.413	41.842	14.859	2245.929
29.55	149.935	125.470	84.370	41.410	14.630	2245.929
29.65	151.477	127.646	85.554	41.756	16.004	2245.929
29.75	150.931	125.653	80.919	42.742	15.317	2245.929
29.85	149.864	124.856	82.998	41.738	15.245	2245.929
29.95	151.062	124.673	86.464	43.088	15.575	2245.929
30.05	152.734	126.981	84.384	40.147	15.603	2245.929
30.15	151.833	122.946	83.157	42.534	16.648	2245.929
30.25	155.332	125.736	83.244	42.776	15.618	2245.929
30.35	153.351	124.773	84.586	42.759	16.047	2245.929
30.45	149.140	126.998	84.095	42.655	14.558	2245.929
30.55	154.786	125.171	84.110	41.911	16.004	2245.929
30.65	151.240	123.611	85.048	42.690	15.432	2245.929
30.75	152.354	126.135	86.723	39.715	15.918	2245.929
30.85	153.220	124.623	84.413	42.084	15.718	2245.929
30.95	151.287	126.998	84.919	40.009	15.789	2245.929
31.05	155.403	126.135	83.475	43.209	15.417	2245.929
31.15	150.160	127.895	85.164	44.108	15.102	2245.929
31.25	150.990	130.386	83.763	41.323	15.804	2245.929
31.35	152.473	125.885	84.529	42.205	15.074	2245.929
31.45	153.244	128.725	85.280	42.396	15.274	2245.929
31.55	154.312	125.852	84.745	42.811	16.219	2245.929
31.65	152.770	125.587	83.503	43.139	15.417	2245.929
31.75	151.441	127.778	84.355	43.641	16.190	2245.929
31.85	152.331	124.906	85.034	41.168	15.432	2245.929
31.95	152.817	126.815	85.496	41.963	16.548	2245.929
32.05	151.323	127.081	84.774	42.067	15.374	2245.929
32.15	153.446	127.579	85.698	43.676	15.732	2245.929
32.25	151.536	127.181	84.586	41.531	15.589	2245.929
32.35	152.876	126.965	84.471	42.482	15.546	2245.929
32.45	154.193	125.354	84.688	43.243	15.460	2245.929
32.55	151.275	126.600	86.579	43.503	15.317	2245.929
32.65	154.300	124.856	83.662	41.565	15.818	2245.929

Midpoint	10	3	1	0.33	0.1	Volume
32.75	152.580	126.882	84.601	41.721	16.491	2245.929
32.85	157.229	125.188	83.994	43.797	16.534	2245.929
32.95	153.908	124.408	86.955	41.842	16.119	2245.929
33.05	154.347	126.749	85.684	42.551	16.348	2245.929
33.15	151.856	126.732	84.067	40.804	15.174	2245.929
33.25	153.968	125.719	84.023	42.897	15.818	2245.929
33.35	153.220	124.524	86.608	42.050	15.417	2245.929
33.45	154.881	127.446	85.308	40.701	14.959	2245.929
33.55	153.054	126.284	86.478	42.638	16.348	2245.929
33.65	152.948	125.653	83.893	43.555	16.205	2245.929
33.75	156.221	131.083	83.518	42.845	16.763	2245.929
33.85	152.781	127.280	85.453	43.347	15.303	2245.929
33.95	154.264	128.874	85.438	41.686	14.773	2245.929
34.05	149.781	130.236	86.767	43.278	16.133	2245.929
34.15	153.066	126.566	87.547	42.793	15.804	2245.929
34.25	155.367	126.400	85.077	42.828	14.916	2245.929
34.35	150.777	123.478	84.153	43.295	15.360	2245.929
34.45	153.707	126.600	84.774	42.690	15.288	2245.929
34.55	157.941	129.007	85.381	42.811	15.517	2245.929
34.65	152.438	126.666	83.648	42.136	15.861	2245.929
34.75	150.575	127.629	83.879	40.285	15.475	2245.929
34.85	154.928	127.994	83.662	44.177	15.303	2245.929
34.95	154.240	130.136	83.388	40.320	16.018	2245.929
35.05	154.395	125.171	85.150	43.606	15.432	2245.929
35.15	152.473	124.192	86.406	44.333	14.888	2245.929
35.25	152.438	125.005	84.500	43.261	15.918	2245.929
35.35	150.670	127.197	84.355	40.389	15.059	2245.929
35.45	151.987	126.184	86.882	42.655	14.902	2245.929
35.55	150.196	126.533	85.034	43.001	15.260	2245.929
35.65	154.679	124.291	85.308	43.243	15.589	2245.929
35.75	155.367	123.627	84.370	43.036	16.648	2245.929
35.85	154.786	126.467	84.485	43.762	15.847	2245.929
35.95	154.003	127.314	83.229	42.984	15.933	2245.929
36.05	153.837	127.480	86.276	42.015	15.761	2245.929
36.15	150.409	128.410	83.763	42.136	15.245	2245.929
36.25	153.813	125.371	83.879	43.970	15.217	2245.929
36.35	152.260	126.018	83.994	42.067	15.632	2245.929
36.45	155.616	128.659	86.276	43.797	16.004	2245.929
36.55	152.271	124.706	85.814	41.081	15.832	2245.929
36.65	153.695	127.247	85.568	42.915	16.061	2245.929
36.75	152.971	127.662	83.417	43.987	16.104	2245.929
36.85	155.355	128.808	87.128	41.358	15.603	2245.929
36.95	153.090	125.885	83.142	41.513	16.391	2245.929
37.05	151.299	127.695	85.987	42.586	16.176	2245.929
37.15	150.563	124.441	86.449	42.517	14.787	2245.929

Midpoint	10	3	1	0.33	0.1	Volume
37.25	154.691	122.664	85.048	41.894	15.446	2245.929
37.35	154.454	127.098	84.197	41.773	15.174	2245.929
37.45	150.291	126.965	83.532	40.873	15.403	2245.929
37.55	152.414	125.902	85.308	41.565	15.632	2245.929
37.65	150.421	127.563	86.781	43.243	15.074	2245.929
37.75	153.778	127.297	84.803	42.759	15.918	2245.929
37.85	151.180	125.238	84.991	42.621	15.245	2245.929
37.95	153.268	130.767	82.868	42.984	15.890	2245.929
38.05	152.283	123.328	84.919	41.496	15.517	2245.929
38.15	153.102	124.192	84.615	44.385	14.487	2245.929
38.25	149.745	126.118	85.193	41.306	15.904	2245.929
38.35	153.339	124.756	85.742	43.001	16.333	2245.929
38.45	151.951	122.548	86.348	43.036	15.245	2245.929
38.55	154.893	125.421	82.897	43.451	15.990	2245.929
38.65	151.880	126.782	84.384	41.617	15.661	2245.929
38.75	152.995	124.159	83.908	42.880	16.147	2245.929
38.85	154.430	124.424	84.890	41.842	15.517	2245.929
38.95	152.034	123.544	83.908	42.724	14.730	2245.929
39.05	152.639	127.280	85.193	41.929	15.947	2245.929
39.15	151.109	126.433	85.409	42.361	15.160	2245.929
39.25	153.019	126.118	85.496	41.790	15.861	2245.929
39.35	150.789	129.605	85.944	42.897	16.090	2245.929
39.45	152.034	127.081	84.947	42.275	15.661	2245.929
39.55	155.343	126.849	85.150	42.845	15.847	2245.929
39.65	153.244	124.906	86.160	42.292	16.434	2245.929
39.75	149.781	126.035	86.305	41.911	15.203	2245.929
39.85	153.220	129.456	85.987	41.929	15.059	2245.929
39.95	152.283	127.164	83.330	40.873	15.432	2245.929
40.05	150.314	126.483	84.558	42.482	15.804	2245.929
40.15	151.477	124.291	85.727	42.292	15.274	2245.929
40.25	156.209	129.240	86.666	41.202	15.632	2245.929
40.35	152.212	124.889	84.572	41.323	15.818	2245.929
40.45	148.322	126.699	85.048	43.278	15.303	2245.929
40.55	151.346	126.633	84.197	41.738	14.931	2245.929
40.65	153.647	129.721	85.236	41.946	16.477	2245.929
40.75	150.445	126.500	85.193	42.966	15.403	2245.929
40.85	152.449	125.603	84.919	41.012	15.890	2245.929
40.95	154.478	128.061	86.146	42.223	14.888	2245.929
41.05	152.758	124.873	83.807	42.811	15.503	2245.929
41.15	154.691	128.609	84.384	42.517	15.575	2245.929
41.25	155.450	126.882	85.323	42.240	15.603	2245.929
41.35	150.943	126.118	85.554	41.237	16.520	2245.929
41.45	153.695	125.105	85.178	42.586	15.890	2245.929
41.55	150.184	128.194	83.677	43.001	15.904	2245.929
41.65	154.122	123.428	84.298	42.811	16.033	2245.929

Midpoint	10	3	1	0.33	0.1	Volume
41.75	151.631	125.520	85.337	43.364	15.532	2245.929
41.85	153.991	126.981	84.471	41.341	15.861	2245.929
41.95	152.734	125.238	85.337	42.257	15.575	2245.929
42.05	151.489	126.782	82.796	42.465	15.303	2245.929
42.15	151.417	128.991	83.114	43.330	16.405	2245.929
42.25	153.837	126.483	85.886	41.808	16.376	2245.929
42.35	153.908	125.802	83.893	42.672	16.348	2245.929
42.45	155.806	125.969	83.677	43.468	15.203	2245.929
42.55	150.919	128.791	85.424	40.960	15.288	2245.929
42.65	152.034	124.358	83.561	43.278	16.319	2245.929
42.75	153.422	126.002	85.236	41.531	15.145	2245.929
42.85	151.275	128.144	84.991	41.185	16.047	2245.929
42.95	149.793	129.572	84.543	41.427	15.446	2245.929
43.05	150.706	124.408	83.013	43.278	15.646	2245.929
43.15	151.738	125.686	86.998	43.036	15.818	2245.929
43.25	150.872	127.031	84.615	43.779	15.603	2245.929
43.35	154.134	128.393	82.608	43.831	15.947	2245.929
43.45	152.414	127.612	84.586	42.793	16.033	2245.929
43.55	149.733	126.915	85.308	43.433	15.489	2245.929
43.65	154.916	125.686	84.529	42.102	15.489	2245.929
43.75	151.702	126.732	87.604	42.344	15.861	2245.929
43.85	152.011	133.092	84.283	41.513	15.675	2245.929
43.95	152.058	124.175	85.048	40.770	15.446	2245.929
44.05	155.759	124.474	86.059	43.018	14.401	2245.929
44.15	152.271	126.683	85.814	43.295	15.489	2245.929
44.25	156.162	127.778	85.770	43.312	15.446	2245.929
44.35	154.371	127.015	84.471	43.728	16.405	2245.929
44.45	151.738	126.583	85.973	40.666	15.059	2245.929
44.55	154.596	124.242	84.456	43.606	15.517	2245.929
44.65	154.027	126.865	83.807	42.828	15.804	2245.929
44.75	152.615	121.087	84.861	42.292	15.288	2245.929
44.85	153.054	124.557	86.420	42.361	15.661	2245.929
44.95	150.469	126.500	85.597	40.735	16.219	2245.929
45.05	154.359	126.616	84.904	42.344	15.718	2245.929
45.15	152.698	127.297	86.940	41.669	15.231	2245.929
45.25	157.158	125.802	82.839	42.309	15.403	2245.929
45.35	152.260	123.146	85.323	42.742	16.061	2245.929
45.45	152.105	126.998	85.092	43.658	16.362	2245.929
45.55	154.406	125.753	84.225	43.157	15.317	2245.929
45.65	148.808	122.813	84.904	43.330	15.661	2245.929
45.75	150.587	125.636	84.081	42.621	15.560	2245.929
45.85	152.532	123.013	84.428	39.957	15.990	2245.929
45.95	150.658	124.640	82.291	41.531	15.775	2245.929
46.05	153.896	128.874	84.182	41.859	15.432	2245.929
46.15	151.667	126.898	85.900	42.828	16.906	2245.929

Midpoint	10	3	1	0.33	0.1	Volume
46.25	154.584	126.483	83.879	43.762	15.203	2245.929
46.35	152.948	127.778	86.926	41.686	15.532	2245.929
46.45	151.299	128.758	85.294	43.433	15.260	2245.929
46.55	152.319	128.924	82.161	43.001	15.603	2245.929
46.65	151.394	126.467	83.070	41.237	15.718	2245.929
46.75	152.544	123.909	83.807	42.361	15.575	2245.929
46.85	151.073	125.686	83.605	42.724	16.219	2245.929
46.95	153.054	127.662	83.171	41.548	16.033	2245.929
47.05	151.821	130.203	85.381	41.323	14.945	2245.929
47.15	149.816	125.902	85.308	42.586	15.603	2245.929
47.25	151.512	127.994	86.955	41.704	16.018	2245.929
47.35	150.646	127.463	84.370	44.004	14.988	2245.929
47.45	155.569	127.612	85.886	43.295	15.446	2245.929
47.55	151.833	126.433	85.482	42.793	15.832	2245.929
47.65	151.323	126.898	85.106	43.209	15.789	2245.929
47.75	150.469	127.380	83.720	41.150	15.002	2245.929
47.85	153.885	125.371	85.294	42.448	16.133	2245.929
47.95	152.853	127.231	84.312	41.081	15.446	2245.929
48.05	149.769	124.922	84.240	41.981	16.176	2245.929
48.15	152.900	126.516	86.218	41.825	15.832	2245.929
48.25	154.620	124.208	85.597	42.050	14.959	2245.929
48.35	152.153	126.849	84.673	42.638	15.059	2245.929
48.45	154.822	127.529	83.402	43.001	16.663	2245.929
48.55	151.773	128.559	84.688	41.462	15.818	2245.929
48.65	150.860	126.450	85.178	42.067	15.331	2245.929
48.75	151.216	124.026	86.839	42.966	15.775	2245.929
48.85	154.276	126.450	83.417	40.510	15.890	2245.929
48.95	149.828	125.753	84.933	43.918	15.389	2245.929
49.05	152.912	125.454	84.038	41.790	16.018	2245.929
49.15	153.991	124.989	84.904	41.600	15.661	2245.929
49.25	152.865	125.802	85.106	42.153	16.061	2245.929
49.35	149.342	124.972	85.987	42.966	15.618	2245.929
49.45	149.247	126.234	85.944	42.517	15.317	2245.929
49.55	149.626	124.972	85.048	42.569	15.503	2245.929
49.65	151.833	125.122	82.579	42.776	15.517	2245.929
49.75	153.410	129.240	86.001	40.337	16.205	2245.929
49.85	150.730	125.454	86.276	43.226	16.119	2245.929
49.95	155.521	123.577	84.615	42.499	15.160	2245.929
50.05	150.848	126.666	83.807	43.070	15.432	2245.929
50.15	151.240	126.085	83.475	42.448	14.945	2245.929
50.25	151.441	125.304	83.171	41.098	15.890	2245.929
50.35	151.465	129.041	85.366	41.756	15.818	2245.929
50.45	152.746	124.939	86.074	42.361	15.203	2245.929
50.55	149.259	125.819	84.312	40.960	15.360	2245.929
50.65	149.282	127.164	85.467	43.105	15.847	2245.929

Midpoint	10	3	1	0.33	0.1	Volume
50.75	151.453	127.496	86.290	41.825	16.319	2245.929
50.85	151.370	129.572	82.666	42.275	15.575	2245.929
50.95	156.304	127.114	83.850	43.312	16.319	2245.929
51.05	151.014	123.810	85.525	43.295	15.918	2245.929
51.15	153.280	123.909	83.633	44.748	15.875	2245.929
51.25	154.288	126.981	82.926	42.292	15.575	2245.929
51.35	152.354	126.483	83.056	40.822	15.002	2245.929
51.45	153.042	124.673	85.150	42.724	15.174	2245.929
51.55	154.205	124.424	83.980	41.842	15.746	2245.929
51.65	152.734	126.384	84.615	43.520	15.317	2245.929
51.75	152.663	123.511	85.612	42.742	15.303	2245.929
51.85	153.517	123.129	83.576	42.413	14.616	2245.929
51.95	150.718	126.683	85.164	41.583	14.716	2245.929
52.05	152.770	124.590	82.262	42.621	15.732	2245.929
52.15	155.213	124.839	84.803	41.150	15.603	2245.929
52.25	152.390	125.587	85.323	41.686	14.644	2245.929
52.35	153.707	125.138	83.994	44.056	15.732	2245.929
52.45	153.232	127.446	84.688	41.635	15.861	2245.929
52.55	152.805	125.587	83.330	40.856	16.391	2245.929
52.65	152.438	126.915	83.821	43.122	15.746	2245.929
52.75	152.781	123.793	84.673	41.531	16.018	2245.929
52.85	149.271	128.210	84.009	43.261	15.317	2245.929
52.95	154.442	127.015	83.836	42.603	15.360	2245.929
53.05	150.824	124.208	84.500	41.877	14.744	2245.929
53.15	150.741	126.749	83.648	42.586	16.076	2245.929
53.25	152.188	127.513	84.890	41.392	15.503	2245.929
53.35	152.307	127.363	82.926	43.382	16.262	2245.929
53.45	149.626	122.498	83.475	41.150	15.947	2245.929
53.55	153.114	124.839	82.363	41.375	16.104	2245.929
53.65	151.963	126.234	82.781	42.153	16.047	2245.929
53.75	151.584	123.079	82.406	42.621	15.346	2245.929
53.85	153.232	128.393	84.067	41.202	15.575	2245.929
53.95	150.611	126.799	83.518	41.721	14.444	2245.929
54.05	153.635	127.862	83.114	43.105	15.661	2245.929
54.15	151.453	125.304	83.417	42.257	15.503	2245.929
54.25	151.761	125.719	83.374	43.537	15.317	2245.929
54.35	149.520	123.345	84.745	42.240	15.260	2245.929
54.45	149.401	127.347	86.276	40.562	16.405	2245.929
54.55	150.611	121.253	82.753	41.963	15.575	2245.929
54.65	152.283	126.334	83.359	41.289	15.059	2245.929
54.75	151.619	126.882	86.030	43.139	15.560	2245.929
54.85	147.563	126.433	83.258	41.029	15.990	2245.929
54.95	153.802	124.424	85.698	42.292	15.317	2245.929
55.05	152.461	127.197	82.724	41.825	15.517	2245.929
55.15	153.161	121.286	83.619	41.859	15.589	2245.929

Midpoint	10	3	1	0.33	0.1	Volume
55.25	153.244	126.450	84.456	41.583	15.675	2245.929
55.35	152.046	127.098	83.417	42.517	15.260	2245.929
55.45	150.077	125.304	82.161	42.551	15.117	2245.929
55.55	152.592	126.068	82.738	42.413	15.560	2245.929
55.65	153.446	125.088	84.745	42.949	15.918	2245.929
55.75	151.323	122.548	84.659	41.375	15.131	2245.929
55.85	153.885	126.965	84.991	41.652	15.188	2245.929
55.95	150.730	124.507	83.258	42.413	15.947	2245.929
56.05	153.944	123.461	83.720	42.257	15.746	2245.929
56.15	151.678	122.349	83.417	42.949	16.505	2245.929
56.25	154.893	123.943	84.428	41.894	15.245	2245.929
56.35	152.271	125.520	81.612	41.894	15.031	2245.929
56.45	153.991	126.467	83.258	42.344	15.933	2245.929
56.55	153.920	127.496	84.919	41.738	15.517	2245.929
56.65	150.741	125.254	82.522	43.641	15.904	2245.929
56.75	151.595	126.732	85.077	43.191	15.832	2245.929
56.85	151.595	126.135	84.197	43.036	15.904	2245.929
56.95	152.710	127.081	83.301	42.378	14.973	2245.929
57.05	148.772	125.969	85.265	42.863	15.503	2245.929
57.15	150.943	126.981	85.164	42.465	15.947	2245.929
57.25	151.773	122.747	85.655	41.635	15.560	2245.929
57.35	151.157	124.972	83.532	42.759	16.262	2245.929
57.45	153.386	127.579	83.446	41.946	15.632	2245.929
57.55	150.552	122.747	83.893	42.828	15.217	2245.929
57.65	157.360	126.998	83.070	40.770	15.646	2245.929
57.75	151.512	125.188	86.695	41.064	14.802	2245.929
57.85	149.460	127.463	83.994	40.579	16.276	2245.929
57.95	151.133	123.694	84.139	42.897	15.818	2245.929
58.05	153.873	128.592	84.095	41.859	14.845	2245.929
58.15	151.560	125.055	82.377	42.966	15.775	2245.929
58.25	154.371	126.450	82.117	41.911	15.145	2245.929
58.35	151.382	126.516	86.074	41.929	15.145	2245.929
58.45	153.090	124.956	86.030	43.416	15.990	2245.929
58.55	154.300	126.633	83.258	42.499	15.446	2245.929
58.65	152.224	127.264	83.763	41.963	15.646	2245.929
58.75	151.038	126.849	83.200	41.946	15.475	2245.929
58.85	152.829	127.430	84.312	42.603	15.016	2245.929
58.95	152.390	124.823	84.269	41.894	15.847	2245.929
59.05	152.402	126.218	82.926	42.292	15.417	2245.929
59.15	151.833	125.421	83.648	41.929	14.802	2245.929
59.25	150.006	129.024	85.641	40.856	15.131	2245.929
59.35	152.544	127.845	86.074	41.929	15.661	2245.929
59.45	152.212	127.596	83.951	41.323	15.074	2245.929
59.55	152.485	124.092	83.258	40.943	15.188	2245.929
59.65	148.096	125.520	83.417	41.254	15.016	2245.929

Midpoint	10	3	1	0.33	0.1	Volume
59.75	153.659	125.404	85.987	42.102	16.033	2245.929
59.85	151.904	125.570	83.648	42.984	15.961	2245.929
59.95	152.402	127.247	84.139	43.503	14.931	2245.929
60.05	152.011	123.710	83.388	41.081	15.618	2245.929
60.15	152.497	126.500	82.088	42.378	15.804	2245.929
60.25	150.670	127.695	83.691	41.652	15.718	2245.929
60.35	151.204	123.511	83.200	42.448	15.374	2245.929
60.45	151.987	125.238	86.218	43.589	15.861	2245.929
60.55	151.750	126.932	83.215	42.205	15.990	2245.929
60.65	150.030	124.690	82.464	42.223	15.303	2245.929
60.75	153.149	125.421	85.121	43.157	15.918	2245.929
60.85	149.769	127.247	85.178	42.586	15.832	2245.929
60.95	151.050	123.694	84.197	41.116	14.773	2245.929
61.05	151.192	124.607	83.807	42.586	15.832	2245.929
61.15	151.773	126.981	84.428	43.693	14.630	2245.929
61.25	149.425	124.590	84.976	43.485	15.861	2245.929
61.35	150.718	125.338	82.031	42.205	15.288	2245.929
61.45	150.492	123.743	84.702	42.966	15.260	2245.929
61.55	153.078	124.873	85.511	41.496	15.260	2245.929
61.65	153.054	126.716	86.045	40.528	15.918	2245.929
61.75	151.678	125.719	83.951	42.569	15.832	2245.929
61.85	153.125	124.640	85.193	41.306	14.787	2245.929
61.95	153.327	128.991	83.922	43.451	15.102	2245.929
62.05	151.904	124.607	83.316	43.036	15.689	2245.929
62.15	150.018	123.478	83.720	41.565	15.360	2245.929
62.25	150.552	126.815	82.550	42.257	15.231	2245.929
62.35	151.394	125.171	84.023	40.995	16.391	2245.929
62.45	149.188	125.769	84.269	41.496	15.131	2245.929
62.55	153.339	125.802	86.810	44.039	14.945	2245.929
62.65	151.406	125.055	84.139	40.856	15.532	2245.929
62.75	150.077	122.000	84.211	42.361	15.217	2245.929
62.85	151.595	127.679	85.280	42.828	15.661	2245.929
62.95	150.196	126.981	84.327	41.289	15.117	2245.929
63.05	151.465	124.873	85.063	40.614	15.632	2245.929
63.15	148.927	120.655	84.341	42.863	15.360	2245.929
63.25	153.564	129.622	83.446	41.219	15.360	2245.929
63.35	147.705	123.478	83.171	41.790	15.961	2245.929
63.45	151.287	125.520	84.601	44.990	15.546	2245.929
63.55	153.066	128.974	83.359	40.925	15.832	2245.929
63.65	154.513	122.581	84.168	42.638	15.589	2245.929
63.75	151.275	125.919	84.110	41.981	16.047	2245.929
63.85	150.860	123.694	82.969	43.399	15.389	2245.929
63.95	150.077	126.965	83.706	43.382	16.076	2245.929
64.05	151.750	126.683	84.745	44.004	14.773	2245.929
64.15	148.476	124.657	83.301	41.548	15.360	2245.929

Midpoint	10	3	1	0.33	0.1	Volume
64.25	149.769	123.179	83.706	42.032	15.403	2245.929
64.35	152.414	122.332	84.456	40.666	16.061	2245.929
64.45	151.085	126.085	83.041	42.344	15.303	2245.929
64.55	150.113	125.902	84.428	41.427	14.329	2245.929
64.65	151.690	125.421	85.770	41.998	15.446	2245.929
64.75	151.014	123.810	84.225	42.084	15.331	2245.929
64.85	150.504	126.417	86.045	42.707	16.147	2245.929
64.95	153.825	128.957	85.612	41.185	15.689	2245.929
65.05	151.939	123.777	85.092	40.891	15.603	2245.929
65.15	149.555	123.577	84.442	39.801	14.916	2245.929
65.25	152.473	126.284	84.659	40.908	15.632	2245.929
65.35	152.212	126.284	85.973	42.205	16.205	2245.929
65.45	150.113	123.146	83.316	42.188	16.119	2245.929
65.55	153.220	122.946	83.893	42.171	15.475	2245.929
65.65	150.469	128.659	83.749	44.125	15.818	2245.929
65.75	152.485	123.179	84.110	41.427	15.389	2245.929
65.85	153.742	127.114	83.359	40.943	15.231	2245.929
65.95	151.453	126.483	83.605	41.462	15.546	2245.929
66.05	150.184	129.290	84.919	40.908	15.303	2245.929
66.15	152.319	124.773	81.915	42.724	15.689	2245.929
66.25	152.770	126.516	83.085	41.531	15.661	2245.929
66.35	151.251	125.869	84.543	42.205	16.119	2245.929
66.45	149.045	124.956	85.424	41.306	15.503	2245.929
66.55	153.576	125.055	83.966	41.600	15.575	2245.929
66.65	150.872	123.594	85.063	43.364	15.489	2245.929
66.75	152.319	122.465	84.283	42.482	15.560	2245.929
66.85	152.829	124.557	85.958	43.433	15.661	2245.929
66.95	151.690	125.271	84.529	43.070	15.804	2245.929
67.05	149.010	124.806	82.608	42.084	15.861	2245.929
67.15	154.383	129.539	85.511	41.704	16.162	2245.929
67.25	151.050	127.862	84.225	41.462	15.832	2245.929
67.35	153.837	126.716	84.456	41.323	15.016	2245.929
67.45	151.844	124.026	83.561	41.098	16.391	2245.929
67.55	154.122	126.400	82.218	43.745	15.589	2245.929
67.65	149.793	125.869	83.475	42.032	15.288	2245.929
67.75	151.584	123.528	82.810	41.185	14.644	2245.929
67.85	152.544	123.411	83.893	42.205	15.589	2245.929
67.95	151.833	127.862	82.854	42.811	15.560	2245.929
68.05	153.635	127.828	84.702	43.399	16.176	2245.929
68.15	152.236	127.147	84.052	43.347	15.689	2245.929
68.25	153.280	126.052	85.612	43.312	16.004	2245.929
68.35	152.509	124.839	82.652	43.537	15.346	2245.929
68.45	150.386	123.959	83.662	41.773	14.172	2245.929
68.55	152.722	128.526	86.276	42.326	15.317	2245.929
68.65	153.042	124.657	87.258	42.863	15.632	2245.929

Midpoint	10	3	1	0.33	0.1	Volume
68.75	151.524	123.677	85.337	42.915	15.117	2245.929
68.85	151.856	125.736	84.500	42.344	15.704	2245.929
68.95	154.371	125.371	84.038	42.690	15.890	2245.929
69.05	152.295	125.271	84.933	43.987	14.515	2245.929
69.15	151.714	125.437	84.832	42.672	16.233	2245.929
69.25	150.172	121.751	84.327	42.672	15.532	2245.929
69.35	152.485	126.566	84.384	43.762	15.360	2245.929
69.45	151.157	125.769	84.269	42.621	15.088	2245.929
69.55	151.714	126.367	85.612	42.880	15.503	2245.929
69.65	153.197	124.308	83.503	42.621	15.789	2245.929
69.75	150.196	124.242	84.009	42.067	15.174	2245.929
69.85	151.500	123.129	82.464	42.638	15.861	2245.929
69.95	150.504	126.500	84.716	43.312	15.746	2245.929
70.05	152.402	126.948	84.456	40.562	15.303	2245.929
70.15	151.477	125.155	83.489	40.925	15.317	2245.929
70.25	154.810	127.928	84.168	42.621	15.460	2245.929
70.35	151.856	126.101	84.168	43.261	16.004	2245.929
70.45	150.694	126.865	83.345	41.341	15.847	2245.929
70.55	151.572	124.640	85.193	40.822	15.646	2245.929
70.65	155.391	123.727	82.637	41.998	15.274	2245.929
70.75	152.710	123.229	84.341	40.009	15.074	2245.929
70.85	154.561	125.703	84.240	42.759	15.331	2245.929
70.95	152.354	127.181	84.904	42.932	15.203	2245.929
71.05	153.339	127.579	82.810	41.652	15.789	2245.929
71.15	151.868	124.242	83.619	40.372	16.663	2245.929
71.25	154.916	126.035	82.680	43.191	15.389	2245.929
71.35	154.217	125.072	86.059	42.603	16.190	2245.929
71.45	152.888	127.961	84.052	41.842	15.532	2245.929
71.55	153.090	125.885	83.215	41.237	16.233	2245.929
71.65	151.904	122.780	84.399	43.018	16.119	2245.929
71.75	150.148	124.673	84.298	43.122	16.205	2245.929
71.85	151.429	127.994	82.319	41.116	15.389	2245.929
71.95	153.197	126.533	83.691	42.396	16.047	2245.929
72.05	153.825	124.706	82.291	41.254	15.231	2245.929
72.15	150.575	127.297	85.511	42.707	15.432	2245.929
72.25	151.809	126.766	82.421	41.565	15.918	2245.929
72.35	153.161	124.225	85.048	42.292	15.417	2245.929
72.45	150.172	126.882	84.471	42.309	14.945	2245.929
72.55	151.216	127.563	81.828	43.416	14.988	2245.929
72.65	150.445	124.275	86.204	43.191	15.203	2245.929
72.75	152.580	126.118	83.374	43.209	15.517	2245.929
72.85	152.307	124.159	82.969	40.701	14.959	2245.929
72.95	150.836	123.694	83.605	41.877	17.250	2245.929
73.05	151.334	126.135	82.753	43.312	15.260	2245.929
73.15	149.923	123.494	84.976	41.686	15.875	2245.929

Midpoint	10	3	1	0.33	0.1	Volume
73.25	151.726	125.603	83.374	42.396	15.618	2245.929
73.35	152.449	126.334	84.500	41.617	15.031	2245.929
73.45	154.371	125.171	85.063	43.676	15.904	2245.929
73.55	152.793	123.461	83.070	42.569	15.389	2245.929
73.65	152.710	128.061	81.626	44.246	15.303	2245.929
73.75	151.667	124.424	83.446	41.981	14.931	2245.929
73.85	153.256	124.640	85.092	42.724	15.618	2245.929
73.95	152.236	125.205	83.070	42.292	15.331	2245.929
74.05	151.714	124.673	84.038	42.482	16.004	2245.929
74.15	150.990	127.978	83.633	42.655	14.773	2245.929
74.25	151.026	125.636	84.038	43.088	14.100	2245.929
74.35	153.244	125.288	84.890	42.413	15.532	2245.929
74.45	152.105	124.009	84.846	43.485	16.290	2245.929
74.55	153.825	124.258	84.947	42.067	14.687	2245.929
74.65	151.595	128.360	83.027	42.240	15.632	2245.929
74.75	152.414	123.129	85.352	43.606	16.033	2245.929
74.85	152.781	124.225	85.626	41.756	14.830	2245.929
74.95	152.912	128.758	85.164	41.669	15.618	2245.929
75.05	152.532	129.007	84.298	43.243	14.931	2245.929
75.15	150.836	127.347	83.922	42.551	15.818	2245.929
75.25	152.971	127.264	85.164	42.759	15.475	2245.929
75.35	151.761	129.240	84.211	40.977	15.231	2245.929
75.45	151.560	124.590	83.345	42.326	15.475	2245.929
75.55	150.765	123.694	83.763	42.050	14.372	2245.929
75.65	152.402	124.524	83.489	42.621	16.090	2245.929
75.75	153.280	125.719	84.254	41.392	15.117	2245.929
75.85	153.707	122.797	87.330	42.534	14.673	2245.929
75.95	152.544	124.889	83.013	42.672	15.789	2245.929
76.05	150.990	125.404	83.677	41.756	15.689	2245.929
76.15	152.876	125.885	83.951	40.372	16.076	2245.929
76.25	151.251	125.354	84.283	41.133	15.775	2245.929
76.35	153.042	127.247	86.810	41.098	15.732	2245.929
76.45	151.738	127.978	84.644	41.358	14.988	2245.929
76.55	151.987	125.985	82.449	41.496	15.102	2245.929
76.65	151.904	122.647	86.464	42.569	15.861	2245.929
76.75	152.331	124.972	84.485	43.001	15.961	2245.929
76.85	152.248	124.059	82.940	40.752	16.104	2245.929
76.95	149.152	125.520	84.139	42.240	16.076	2245.929
77.05	153.920	125.919	83.489	42.465	15.403	2245.929
77.15	153.837	124.275	85.467	42.949	15.475	2245.929
77.25	153.303	124.408	84.803	44.679	15.188	2245.929
77.35	152.568	123.810	83.200	42.396	15.646	2245.929
77.45	151.833	125.421	82.969	41.963	16.333	2245.929
77.55	149.116	124.075	85.583	41.859	14.888	2245.929
77.65	151.797	126.118	86.290	43.572	15.732	2245.929

Midpoint	10	3	1	0.33	0.1	Volume
77.75	151.097	126.400	84.456	41.116	16.162	2245.929
77.85	152.283	126.716	84.572	41.254	16.276	2245.929
77.95	151.477	125.254	83.821	43.849	15.947	2245.929
78.05	151.607	124.574	86.204	42.776	15.446	2245.929
78.15	153.019	126.732	84.413	43.433	14.873	2245.929
78.25	152.781	131.100	84.428	42.672	15.646	2245.929
78.35	149.425	125.587	84.659	43.001	14.802	2245.929
78.45	152.521	125.288	83.215	41.306	15.460	2245.929
78.55	153.778	126.334	84.225	41.981	15.475	2245.929
78.65	151.228	123.826	83.706	43.416	16.018	2245.929
78.75	152.426	122.780	82.493	43.347	16.491	2245.929
78.85	151.216	125.039	82.709	43.555	15.131	2245.929
78.95	152.912	123.096	84.254	42.188	15.804	2245.929
79.05	153.469	127.978	84.630	43.139	15.746	2245.929
79.15	152.260	125.952	83.186	43.226	15.904	2245.929
79.25	154.157	128.841	83.561	42.655	15.231	2245.929
79.35	152.236	127.812	84.529	42.413	15.804	2245.929
79.45	151.406	125.636	83.503	41.358	15.446	2245.929
79.55	153.232	127.181	83.662	41.289	14.945	2245.929
79.65	153.208	126.251	83.114	41.859	15.188	2245.929
79.75	153.351	124.790	84.716	43.399	16.076	2245.929
79.85	153.612	125.387	86.579	41.859	15.217	2245.929
79.95	152.473	124.507	86.752	43.399	15.288	2245.929
80.05	151.868	125.819	83.893	40.718	16.076	2245.929
80.15	154.976	126.683	83.778	41.738	15.245	2245.929
80.25	151.714	127.945	84.197	41.877	15.646	2245.929
80.35	153.042	126.915	83.807	43.606	14.558	2245.929
80.45	151.916	126.251	83.374	43.105	15.446	2245.929
80.55	151.180	128.326	86.218	42.344	14.959	2245.929
80.65	153.102	127.795	84.644	42.465	15.317	2245.929
80.75	149.911	127.446	83.417	41.946	15.933	2245.929
80.85	150.089	128.874	84.081	42.966	15.890	2245.929
80.95	154.157	126.467	84.384	42.102	15.432	2245.929
81.05	149.128	126.749	84.572	40.925	15.460	2245.929
81.15	150.231	122.448	85.698	43.018	16.162	2245.929
81.25	151.204	125.338	85.554	43.001	15.389	2245.929
81.35	150.255	125.520	84.586	41.929	15.890	2245.929
81.45	151.180	126.500	84.283	41.444	15.231	2245.929
81.55	153.849	125.271	84.095	43.001	15.546	2245.929
81.65	150.730	125.138	85.323	41.894	15.761	2245.929
81.75	153.291	123.278	85.308	42.067	16.276	2245.929
81.85	151.251	126.384	83.229	42.378	15.990	2245.929
81.95	151.785	127.147	82.550	42.707	16.434	2245.929
82.05	150.267	124.939	84.731	42.344	15.804	2245.929
82.15	152.117	123.494	84.688	41.929	15.961	2245.929

Midpoint	10	3	1	0.33	0.1	Volume
82.25	152.592	124.308	86.146	41.738	15.446	2245.929
82.35	150.291	125.520	84.991	41.652	15.160	2245.929
82.45	150.042	127.496	83.648	42.517	15.632	2245.929
82.55	151.394	127.795	84.716	41.444	14.988	2245.929
82.65	152.011	127.446	84.991	41.669	16.004	2245.929
82.75	151.512	126.865	84.124	41.219	15.274	2245.929
82.85	155.794	125.454	85.669	43.433	15.661	2245.929
82.95	150.184	126.052	84.586	43.157	15.403	2245.929
83.05	152.888	126.284	85.222	41.756	15.274	2245.929
83.15	152.627	125.304	85.612	42.102	15.818	2245.929
83.25	153.730	130.452	84.038	42.534	15.188	2245.929
83.35	153.363	124.507	85.106	41.929	15.646	2245.929
83.45	153.375	123.909	82.854	42.603	15.904	2245.929
83.55	153.137	127.015	85.597	42.621	14.816	2245.929
83.65	151.690	127.994	84.038	43.416	15.732	2245.929
83.75	151.085	125.470	85.612	41.911	16.162	2245.929
83.85	149.449	124.358	84.153	43.070	16.276	2245.929
83.95	151.631	125.819	84.846	42.897	15.618	2245.929
84.05	152.971	127.895	83.388	43.987	15.231	2245.929
84.15	152.509	125.171	85.193	43.883	15.661	2245.929
84.25	155.830	126.483	85.915	43.658	16.348	2245.929
84.35	151.382	125.570	86.074	43.312	15.303	2245.929
84.45	155.213	123.992	83.561	41.531	16.090	2245.929
84.55	149.128	127.413	83.215	43.901	16.248	2245.929
84.65	153.327	125.039	84.240	39.905	16.391	2245.929
84.75	154.466	127.081	84.168	41.773	15.775	2245.929
84.85	151.062	124.524	83.706	44.558	15.589	2245.929
84.95	150.030	126.533	83.605	42.396	15.489	2245.929
85.05	153.220	127.778	85.467	41.790	16.133	2245.929
85.15	151.251	128.111	84.615	43.018	16.176	2245.929
85.25	151.263	126.749	84.413	42.015	15.475	2245.929
85.35	150.386	125.885	84.471	41.635	16.591	2245.929
85.45	153.102	126.500	83.980	43.191	15.475	2245.929
85.55	154.762	127.463	83.734	43.191	15.517	2245.929
85.65	152.556	124.756	84.688	42.966	15.031	2245.929
85.75	153.517	125.653	85.727	41.998	15.933	2245.929
85.85	152.248	128.194	85.222	41.410	16.262	2245.929
85.95	150.765	124.790	83.431	41.462	16.348	2245.929
86.05	154.193	126.301	85.958	42.984	14.888	2245.929
86.15	153.197	123.810	83.489	41.548	16.047	2245.929
86.25	150.255	126.483	83.547	42.292	16.190	2245.929
86.35	154.347	125.819	84.023	43.209	15.704	2245.929
86.45	150.480	126.417	85.669	43.018	14.587	2245.929
86.55	154.608	129.256	84.529	42.326	15.904	2245.929
86.65	151.334	125.254	85.106	41.323	15.689	2245.929

Midpoint	10	3	1	0.33	0.1	Volume
86.75	152.271	124.673	86.983	41.704	16.047	2245.929
86.85	150.053	124.192	85.294	41.583	15.274	2245.929
86.95	152.948	128.277	84.630	42.378	15.546	2245.929
87.05	149.899	126.616	84.875	43.952	14.988	2245.929
87.15	150.148	126.284	83.778	41.704	15.861	2245.929
87.25	149.899	124.922	86.781	41.462	16.176	2245.929
87.35	149.176	125.338	83.287	42.240	15.575	2245.929
87.45	149.650	125.653	85.424	41.410	14.573	2245.929
87.55	150.077	125.769	85.265	41.254	15.274	2245.929
87.65	147.824	119.741	83.128	42.378	15.847	2245.929
87.75	147.290	123.727	83.157	41.202	15.918	2245.929
87.85	151.382	123.577	83.518	41.859	15.374	2245.929
87.95	153.659	120.904	84.298	42.119	15.317	2245.929
88.05	151.014	124.026	83.359	42.915	16.033	2245.929
88.15	150.848	124.109	82.753	43.330	15.818	2245.929
88.25	150.160	123.411	80.182	42.586	15.346	2245.929
88.35	151.595	126.417	84.442	41.531	14.902	2245.929
88.45	151.002	125.171	81.381	42.465	15.603	2245.929
88.55	149.140	121.551	82.926	42.223	15.503	2245.929
88.65	149.603	123.146	81.828	40.406	15.002	2245.929
88.75	150.208	123.860	84.197	41.271	16.190	2245.929
88.85	146.887	123.146	84.168	41.410	15.446	2245.929
88.95	149.674	123.992	82.522	41.929	15.675	2245.929
89.05	148.203	123.295	81.785	40.389	15.546	2245.929
89.15	150.670	123.860	82.334	42.742	14.687	2245.929
89.25	146.922	127.596	81.915	41.116	15.646	2245.929
89.35	148.689	125.005	80.529	41.185	15.260	2245.929
89.45	148.310	120.472	82.767	42.655	15.890	2245.929
89.55	145.712	121.485	80.529	40.182	14.830	2245.929
89.65	146.578	121.203	80.341	41.444	15.489	2245.929
89.75	148.239	123.179	82.897	40.977	15.245	2245.929
89.85	147.539	118.496	81.280	40.268	14.888	2245.929
89.95	148.013	120.157	81.020	40.977	15.618	2245.929
90.05	144.135	123.760	81.872	41.168	14.902	2245.929
90.15	147.918	123.677	81.164	40.476	14.329	2245.929
90.25	144.479	120.439	80.789	41.479	15.274	2245.929
90.35	145.546	120.688	80.168	40.199	15.532	2245.929
90.45	145.131	120.256	80.197	41.496	15.575	2245.929
90.55	146.044	117.516	79.821	40.078	14.759	2245.929
90.65	144.823	116.968	80.774	41.098	14.845	2245.929
90.75	143.838	119.310	80.529	40.666	14.959	2245.929
90.85	145.594	119.210	79.186	40.026	14.043	2245.929
90.95	144.929	121.834	80.991	39.974	15.460	2245.929
91.05	145.048	120.738	78.926	40.856	15.374	2245.929
91.15	142.249	120.804	78.132	40.130	15.646	2245.929

Midpoint	10	3	1	0.33	0.1	Volume
91.25	143.589	118.612	77.150	39.922	14.716	2245.929
91.35	140.909	115.275	79.533	40.147	14.916	2245.929
91.45	143.281	119.426	76.659	40.666	14.787	2245.929
91.55	140.292	116.935	78.450	39.023	14.773	2245.929
91.65	141.502	119.160	80.269	39.178	15.389	2245.929
91.75	140.565	114.992	77.092	40.424	15.331	2245.929
91.85	145.036	117.915	79.258	40.528	13.871	2245.929
91.95	141.668	115.640	77.006	40.112	15.002	2245.929
92.05	144.325	117.168	79.677	39.576	14.487	2245.929
92.15	139.556	116.736	77.294	38.556	15.059	2245.929
92.25	137.920	115.208	79.403	39.317	13.900	2245.929
92.35	138.216	119.061	77.107	38.521	14.258	2245.929
92.45	141.668	114.411	77.988	40.579	14.959	2245.929
92.55	138.702	116.404	76.399	38.348	14.616	2245.929
92.65	138.453	116.985	78.233	39.299	14.501	2245.929
92.75	140.956	114.245	77.049	39.057	13.556	2245.929
92.85	138.975	115.059	77.554	37.760	14.215	2245.929
92.95	139.011	114.943	78.045	36.238	13.928	2245.929
93.05	141.110	115.557	76.630	37.881	15.675	2245.929
93.15	138.975	115.872	77.150	37.604	14.100	2245.929
93.25	139.889	115.574	77.858	38.729	14.687	2245.929
93.35	136.342	114.029	76.558	38.763	14.301	2245.929
93.45	136.686	115.042	76.558	37.258	15.045	2245.929
93.55	139.236	113.764	78.782	39.524	15.217	2245.929
93.65	137.421	114.926	74.999	38.729	13.886	2245.929
93.75	138.501	115.756	74.609	38.244	14.229	2245.929
93.85	137.765	114.162	76.746	37.535	14.816	2245.929
93.95	137.991	113.697	77.367	38.175	13.270	2245.929
94.05	135.808	116.238	76.934	37.760	14.086	2245.929
94.15	136.627	113.681	76.125	39.213	13.943	2245.929
94.25	138.240	113.382	76.226	37.328	14.744	2245.929
94.35	136.081	113.431	76.486	40.164	13.542	2245.929
94.45	134.753	113.050	76.962	37.103	13.628	2245.929
94.55	137.030	113.597	76.991	39.818	14.258	2245.929
94.65	136.698	113.614	75.533	37.501	14.644	2245.929
94.75	138.003	114.926	75.879	38.296	14.472	2245.929
94.85	138.275	113.083	75.995	38.400	14.072	2245.929
94.95	135.381	113.066	75.461	39.369	13.470	2245.929
95.05	134.693	113.398	75.879	38.504	14.616	2245.929
95.15	136.390	113.299	75.316	37.051	14.086	2245.929
95.25	138.845	110.260	76.255	38.158	13.528	2245.929
95.35	136.034	115.823	76.702	37.570	14.129	2245.929
95.45	137.184	111.472	75.793	38.435	13.642	2245.929
95.55	137.599	114.461	75.923	37.846	14.859	2245.929
95.65	135.393	112.850	73.872	37.085	13.614	2245.929

Midpoint	10	3	1	0.33	0.1	Volume
95.75	140.066	111.339	74.768	38.054	13.871	2245.929
95.85	137.137	112.020	75.836	37.345	13.556	2245.929
95.95	135.322	110.061	75.230	38.175	14.515	2245.929
96.05	135.417	112.751	74.898	38.746	14.129	2245.929
96.15	134.148	111.439	76.702	36.688	14.014	2245.929
96.25	136.484	112.020	75.186	38.383	14.544	2245.929
96.35	137.161	114.561	75.027	37.933	13.943	2245.929
96.45	135.500	114.062	77.237	37.829	13.470	2245.929
96.55	136.105	112.086	76.067	38.832	14.243	2245.929
96.65	136.366	113.681	75.302	36.393	14.100	2245.929
96.75	137.860	113.382	75.663	37.535	14.243	2245.929
96.85	134.397	112.302	75.417	38.365	13.914	2245.929
96.95	134.943	110.343	73.482	37.795	14.716	2245.929
97.05	136.235	112.153	75.995	37.829	14.458	2245.929
97.15	136.176	112.419	74.739	37.708	13.385	2245.929
97.25	135.370	112.767	74.609	37.725	13.470	2245.929
97.35	135.927	114.826	74.941	38.175	14.086	2245.929
97.45	133.069	112.153	74.667	37.604	14.158	2245.929
97.55	134.100	109.263	75.923	36.463	13.213	2245.929
97.65	137.445	112.568	75.822	37.691	14.730	2245.929
97.75	135.310	112.335	75.071	36.463	14.458	2245.929
97.85	134.883	110.841	72.948	37.604	13.785	2245.929
97.95	136.010	112.535	75.793	37.068	13.742	2245.929
98.05	135.986	114.710	74.768	37.743	13.270	2245.929
98.15	135.939	114.461	75.706	37.379	13.757	2245.929
98.25	135.322	112.585	74.796	38.158	13.871	2245.929
98.35	134.290	110.359	74.768	39.715	14.329	2245.929
98.45	135.192	113.182	74.334	37.691	12.955	2245.929
98.55	136.484	111.173	76.746	37.864	13.614	2245.929
98.65	134.824	115.009	73.121	37.379	13.971	2245.929
98.75	135.144	111.372	74.161	37.760	14.229	2245.929
98.85	135.607	113.514	75.461	38.798	13.928	2245.929
98.95	136.152	112.850	76.255	38.071	14.186	2245.929
99.05	137.908	112.817	75.778	37.501	14.043	2245.929
99.15	135.524	113.166	76.096	36.584	13.456	2245.929
99.25	137.386	112.551	75.157	37.518	14.100	2245.929
99.35	132.357	112.800	74.291	37.864	13.141	2245.929
99.45	133.543	114.245	76.616	37.206	14.158	2245.929
99.55	136.627	109.778	75.576	37.881	13.886	2245.929
99.65	133.911	111.954	73.194	38.002	14.329	2245.929
99.75	135.441	111.057	74.392	38.556	13.657	2245.929
99.85	135.239	111.173	73.959	35.788	14.186	2245.929
99.95	73.230	103.053	40.070	34.819	6.914	2245.929

Table of Mn^{2+} population data for simulation with Ca^{2+} and Mn^{2+} across bulk density ratios (#JJF-GRS12G8)

	Population in histogram bin at Mn^{2+}/Ca^{2+} ratio:					
Midpoint	10	3	1	0.33	0.1	Volume
-99.95	0.000	0.000	0.000	0.000	0.000	2233.992
-99.85	0.000	0.000	0.000	0.000	0.000	2233.992
-99.75	3.582	24.991	19.898	77.284	36.976	2233.992
-99.65	13.711	38.342	73.786	116.739	141.089	2233.992
-99.55	13.699	35.967	76.154	112.709	138.340	2233.992
-99.45	13.166	37.346	75.490	114.318	139.127	2233.992
-99.35	14.221	37.761	75.923	115.200	137.868	2233.992
-99.25	14.648	37.678	78.204	115.442	141.647	2233.992
-99.15	13.818	38.740	76.702	116.705	137.639	2233.992
-99.05	13.759	37.063	75.908	111.187	138.913	2233.992
-98.95	13.545	37.844	78.118	113.280	137.553	2233.992
-98.85	13.711	38.508	76.659	113.972	139.256	2233.992
-98.75	13.379	37.047	77.352	115.753	141.260	2233.992
-98.65	13.711	38.325	77.641	112.346	136.952	2233.992
-98.55	13.866	36.930	75.230	112.294	136.451	2233.992
-98.45	14.245	38.491	77.988	114.595	140.487	2233.992
-98.35	14.150	39.039	76.934	113.470	138.440	2233.992
-98.25	14.447	37.047	75.331	113.920	138.770	2233.992
-98.15	14.245	38.076	77.655	112.847	138.455	2233.992
-98.05	14.755	36.499	73.598	111.879	138.827	2233.992
-97.95	13.949	34.788	75.215	115.096	141.947	2233.992
-97.85	13.545	38.458	74.753	115.978	139.628	2233.992
-97.75	14.281	37.628	74.017	114.629	138.770	2233.992
-97.65	14.304	39.620	76.226	114.352	138.927	2233.992
-97.55	13.699	38.392	74.580	113.609	138.226	2233.992
-97.45	13.059	35.419	77.338	112.415	138.913	2233.992
-97.35	13.972	36.831	76.385	111.100	139.729	2233.992
-97.25	14.542	37.844	76.414	113.712	136.723	2233.992
-97.15	14.008	37.860	75.013	117.155	139.356	2233.992
-97.05	14.494	38.740	76.529	110.755	136.866	2233.992
-96.95	14.494	39.272	73.150	113.315	138.183	2233.992
-96.85	13.960	37.744	74.450	115.010	136.622	2233.992
-96.75	14.387	38.010	77.035	116.687	137.767	2233.992
-96.65	14.055	38.906	76.385	113.799	135.678	2233.992
-96.55	13.771	37.312	76.746	111.239	139.414	2233.992
-96.45	14.435	37.927	74.176	112.398	140.101	2233.992
-96.35	13.688	38.309	75.013	115.667	139.886	2233.992
-96.25	13.854	38.259	76.775	115.304	140.001	2233.992
-96.15	14.055	38.790	75.952	113.453	140.258	2233.992
-96.05	13.711	37.478	76.024	115.044	139.843	2233.992
-95.95	14.281	37.578	75.446	115.823	138.812	2233.992
-95.85	13.949	37.561	75.013	113.660	140.244	2233.992

Midpoint	10	3	1	0.33	0.1	Volume
-95.75	14.470	37.047	75.721	113.297	137.338	2233.992
-95.65	13.996	38.774	75.201	113.107	140.029	2233.992
-95.55	13.711	36.548	74.392	113.660	138.626	2233.992
-95.45	13.937	37.096	76.601	111.879	139.986	2233.992
-95.35	14.909	38.541	76.645	115.459	140.487	2233.992
-95.25	13.557	38.325	73.396	114.629	137.424	2233.992
-95.15	13.664	37.296	76.168	113.678	138.369	2233.992
-95.05	13.261	36.416	76.630	111.862	139.428	2233.992
-94.95	15.111	38.541	77.511	115.926	139.757	2233.992
-94.85	14.055	37.561	75.778	113.453	139.213	2233.992
-94.75	13.937	37.678	76.385	114.525	140.588	2233.992
-94.65	13.664	37.894	75.634	111.187	138.941	2233.992
-94.55	13.782	38.325	75.417	114.370	139.800	2233.992
-94.45	13.711	36.798	74.623	115.321	135.448	2233.992
-94.35	14.126	36.715	76.861	114.819	134.718	2233.992
-94.25	13.901	36.615	75.634	113.764	137.939	2233.992
-94.15	14.304	37.661	76.356	117.155	139.528	2233.992
-94.05	14.482	38.624	74.825	113.730	139.185	2233.992
-93.95	14.316	37.960	77.670	112.830	136.622	2233.992
-93.85	14.032	37.910	75.071	113.332	139.385	2233.992
-93.75	13.652	38.940	76.630	113.280	138.498	2233.992
-93.65	14.352	36.847	75.663	116.480	139.943	2233.992
-93.55	14.625	37.860	76.760	114.802	138.039	2233.992
-93.45	14.743	38.558	75.865	113.574	138.469	2233.992
-93.35	14.209	39.205	74.551	116.428	138.855	2233.992
-93.25	14.743	38.558	77.367	115.407	139.170	2233.992
-93.15	14.150	37.611	77.757	115.944	139.299	2233.992
-93.05	14.826	38.940	77.251	117.967	137.309	2233.992
-92.95	14.447	38.226	76.240	115.684	139.786	2233.992
-92.85	13.984	39.870	76.370	113.436	141.761	2233.992
-92.75	14.826	37.827	76.731	115.598	140.788	2233.992
-92.65	14.447	38.093	76.818	115.753	139.256	2233.992
-92.55	14.387	38.591	75.822	114.473	142.291	2233.992
-92.45	13.972	38.325	76.645	114.629	140.330	2233.992
-92.35	14.803	37.711	78.320	115.425	140.688	2233.992
-92.25	14.008	39.255	78.623	118.106	137.295	2233.992
-92.15	14.126	37.113	77.497	116.307	140.630	2233.992
-92.05	14.387	38.259	75.360	115.200	140.645	2233.992
-91.95	14.292	39.056	77.222	117.673	144.667	2233.992
-91.85	15.253	38.126	78.320	116.826	140.874	2233.992
-91.75	15.087	38.441	78.984	115.926	143.508	2233.992
-91.65	14.625	40.633	76.702	118.538	140.444	2233.992
-91.55	14.387	40.069	76.009	117.431	142.162	2233.992
-91.45	14.506	40.451	78.392	118.296	143.780	2233.992
-91.35	14.625	39.371	75.822	120.995	143.637	2233.992

Midpoint	10	3	1	0.33	0.1	Volume
-91.25	14.257	37.362	78.710	116.065	142.248	2233.992
-91.15	14.221	39.703	79.345	118.607	143.637	2233.992
-91.05	14.969	39.039	77.612	120.060	142.950	2233.992
-90.95	14.767	39.687	78.233	116.670	146.027	2233.992
-90.85	14.874	39.405	79.489	119.490	144.710	2233.992
-90.75	14.803	40.301	78.435	121.773	143.551	2233.992
-90.65	14.969	41.148	78.493	121.410	143.164	2233.992
-90.55	14.957	39.106	80.240	119.784	147.616	2233.992
-90.45	15.099	40.069	79.258	118.694	149.663	2233.992
-90.35	15.099	39.803	81.005	121.998	147.616	2233.992
-90.25	14.672	40.667	78.840	122.742	147.903	2233.992
-90.15	14.494	41.397	79.995	125.838	150.121	2233.992
-90.05	14.032	39.222	79.561	122.690	146.399	2233.992
-89.95	14.625	39.156	80.255	124.713	145.498	2233.992
-89.85	15.455	41.646	76.139	122.811	146.858	2233.992
-89.75	15.040	39.189	80.442	122.932	145.784	2233.992
-89.65	15.301	42.609	81.208	123.485	147.044	2233.992
-89.55	14.387	40.135	80.052	123.831	150.322	2233.992
-89.45	14.518	40.002	80.399	121.029	148.432	2233.992
-89.35	14.518	39.488	81.410	123.503	148.232	2233.992
-89.25	14.992	40.916	81.323	123.485	145.025	2233.992
-89.15	14.198	40.152	82.695	124.852	151.410	2233.992
-89.05	15.028	41.314	81.424	124.817	150.193	2233.992
-88.95	14.838	41.331	80.962	124.402	150.107	2233.992
-88.85	15.301	41.198	83.417	123.191	150.236	2233.992
-88.75	15.099	42.892	80.904	123.624	147.015	2233.992
-88.65	15.146	41.364	81.727	122.932	149.005	2233.992
-88.55	15.657	41.198	81.280	122.067	151.238	2233.992
-88.45	14.980	41.663	83.749	122.205	151.324	2233.992
-88.35	15.989	41.165	84.124	123.174	151.925	2233.992
-88.25	15.538	41.862	83.186	122.793	152.741	2233.992
-88.15	14.992	41.447	80.977	126.184	151.195	2233.992
-88.05	15.135	41.846	82.810	123.970	151.109	2233.992
-87.95	15.645	40.882	84.543	123.555	150.708	2233.992
-87.85	15.230	42.078	81.771	123.416	151.710	2233.992
-87.75	14.909	40.285	82.680	123.364	152.913	2233.992
-87.65	16.072	42.427	82.002	122.811	151.095	2233.992
-87.55	15.277	41.397	82.637	125.129	148.618	2233.992
-87.45	16.297	42.078	83.518	125.872	151.338	2233.992
-87.35	15.241	40.650	82.969	127.118	150.050	2233.992
-87.25	15.301	42.344	83.532	125.388	152.125	2233.992
-87.15	15.704	41.779	82.276	120.995	151.839	2233.992
-87.05	14.957	43.058	82.767	126.633	149.606	2233.992
-86.95	15.526	41.198	81.800	125.474	151.753	2233.992
-86.85	15.206	41.132	84.514	122.482	151.367	2233.992

Midpoint	10	3	1	0.33	0.1	Volume
-86.75	16.095	40.882	82.435	128.588	149.305	2233.992
-86.65	15.751	41.414	85.482	124.731	149.821	2233.992
-86.55	15.004	42.892	81.612	126.530	152.569	2233.992
-86.45	14.672	42.925	82.189	124.022	154.015	2233.992
-86.35	15.158	40.135	82.810	126.460	151.080	2233.992
-86.25	14.803	41.547	79.735	124.990	149.119	2233.992
-86.15	15.111	41.879	83.850	124.160	155.547	2233.992
-86.05	15.194	41.563	83.041	123.260	156.205	2233.992
-85.95	16.155	41.696	83.547	126.236	152.698	2233.992
-85.85	15.692	39.504	83.908	124.627	152.841	2233.992
-85.75	15.645	41.215	82.464	124.056	154.960	2233.992
-85.65	15.965	43.606	83.128	123.122	151.782	2233.992
-85.55	15.372	42.775	84.442	128.726	155.633	2233.992
-85.45	14.779	41.098	83.287	126.737	152.140	2233.992
-85.35	15.609	41.945	83.763	124.160	152.254	2233.992
-85.25	16.854	43.008	83.114	120.752	151.367	2233.992
-85.15	15.289	40.849	83.186	125.838	151.438	2233.992
-85.05	15.384	40.152	82.926	125.250	150.923	2233.992
-84.95	15.704	42.859	86.045	125.129	151.825	2233.992
-84.85	15.028	42.493	83.142	126.236	152.541	2233.992
-84.75	14.494	41.181	83.272	126.097	151.095	2233.992
-84.65	15.716	41.480	85.886	127.896	151.496	2233.992
-84.55	15.562	42.410	82.435	127.792	152.154	2233.992
-84.45	15.526	43.074	83.316	126.789	151.252	2233.992
-84.35	15.277	41.663	83.417	125.250	153.156	2233.992
-84.25	15.882	42.742	83.099	126.962	153.142	2233.992
-84.15	15.324	42.028	81.872	124.108	153.113	2233.992
-84.05	15.372	42.692	82.507	126.685	150.350	2233.992
-83.95	16.238	41.912	84.067	127.204	153.142	2233.992
-83.85	15.111	41.115	83.836	126.893	154.044	2233.992
-83.75	15.407	42.095	81.525	123.606	151.939	2233.992
-83.65	15.301	43.108	82.695	125.976	152.684	2233.992
-83.55	16.427	41.895	83.677	125.890	149.864	2233.992
-83.45	15.407	43.290	82.868	127.913	153.543	2233.992
-83.35	16.036	42.095	82.319	123.295	151.624	2233.992
-83.25	15.052	43.323	82.449	127.827	154.344	2233.992
-83.15	16.048	43.606	85.337	127.031	153.485	2233.992
-83.05	15.704	41.597	82.161	125.111	148.790	2233.992
-82.95	15.941	41.895	83.272	123.001	152.040	2233.992
-82.85	15.526	42.344	81.987	128.017	152.254	2233.992
-82.75	15.811	40.949	83.301	126.270	150.093	2233.992
-82.65	15.063	42.576	83.734	124.627	151.496	2233.992
-82.55	15.751	42.775	84.197	124.869	154.573	2233.992
-82.45	15.526	41.132	84.904	125.647	153.070	2233.992
-82.35	15.075	42.377	83.576	123.797	152.168	2233.992

Midpoint	10	3	1	0.33	0.1	Volume
-82.25	14.969	42.526	84.904	127.222	151.309	2233.992
-82.15	16.380	41.082	84.139	128.657	153.213	2233.992
-82.05	15.668	42.526	84.197	125.907	152.841	2233.992
-81.95	16.131	41.912	83.937	126.253	155.475	2233.992
-81.85	14.850	43.108	85.020	126.391	152.354	2233.992
-81.75	15.170	40.982	84.976	126.322	155.260	2233.992
-81.65	16.250	42.692	83.171	125.942	153.571	2233.992
-81.55	15.396	41.497	81.958	125.959	154.745	2233.992
-81.45	15.811	41.663	82.074	127.412	155.676	2233.992
-81.35	15.550	40.484	83.431	122.032	153.442	2233.992
-81.25	16.309	40.318	84.240	126.772	152.340	2233.992
-81.15	15.099	41.879	81.886	124.956	150.093	2233.992
-81.05	15.158	41.895	82.088	127.308	152.025	2233.992
-80.95	15.834	40.102	87.835	125.111	153.256	2233.992
-80.85	15.692	42.925	84.659	126.339	155.160	2233.992
-80.75	14.957	40.251	85.150	123.209	154.373	2233.992
-80.65	15.407	42.643	84.471	124.160	154.430	2233.992
-80.55	15.550	42.626	83.258	125.215	151.567	2233.992
-80.45	15.419	43.257	84.586	123.814	152.269	2233.992
-80.35	16.012	42.327	82.998	128.380	154.244	2233.992
-80.25	16.214	43.589	82.478	127.152	150.794	2233.992
-80.15	15.799	42.078	84.139	126.893	152.999	2233.992
-80.05	15.941	42.394	84.586	126.789	151.553	2233.992
-79.95	15.799	41.497	84.976	127.152	153.013	2233.992
-79.85	15.301	41.746	84.601	124.886	150.637	2233.992
-79.75	15.502	42.228	83.691	123.139	151.624	2233.992
-79.65	15.811	42.012	84.413	125.596	150.680	2233.992
-79.55	16.226	42.311	83.590	128.138	148.389	2233.992
-79.45	15.704	41.248	85.872	124.834	153.571	2233.992
-79.35	15.206	42.277	82.637	127.222	151.138	2233.992
-79.25	15.692	42.726	83.691	125.596	152.813	2233.992
-79.15	15.538	41.796	83.633	124.367	154.631	2233.992
-79.05	16.000	40.434	84.182	128.450	152.097	2233.992
-78.95	15.052	42.261	81.150	123.831	153.528	2233.992
-78.85	15.645	41.331	83.966	128.017	152.870	2233.992
-78.75	15.763	41.995	82.377	128.778	154.802	2233.992
-78.65	15.016	41.397	83.388	125.474	149.291	2233.992
-78.55	16.238	41.364	84.919	124.523	151.352	2233.992
-78.45	15.573	41.663	83.475	126.478	155.117	2233.992
-78.35	15.728	42.311	81.641	122.742	152.397	2233.992
-78.25	16.178	42.892	81.958	125.025	153.829	2233.992
-78.15	16.380	41.680	83.229	126.876	153.256	2233.992
-78.05	15.870	42.327	81.670	123.036	151.753	2233.992
-77.95	15.016	43.423	82.117	124.904	149.506	2233.992
-77.85	15.301	42.161	83.706	124.281	152.269	2233.992

Midpoint	10	3	1	0.33	0.1	Volume
-77.75	16.095	41.895	84.312	124.177	153.943	2233.992
-77.65	15.052	41.812	81.987	126.720	149.635	2233.992
-77.55	14.933	41.713	83.864	126.582	151.610	2233.992
-77.45	15.028	41.646	84.095	127.014	152.097	2233.992
-77.35	15.597	42.460	82.247	125.129	152.340	2233.992
-77.25	15.917	41.032	82.016	122.292	150.708	2233.992
-77.15	15.099	41.895	84.601	125.734	153.471	2233.992
-77.05	15.016	42.427	84.774	126.564	152.827	2233.992
-76.95	15.514	42.028	82.940	126.997	155.289	2233.992
-76.85	15.111	43.921	82.088	125.215	151.281	2233.992
-76.75	15.740	41.929	83.951	126.789	152.397	2233.992
-76.65	15.052	41.480	83.763	125.146	154.330	2233.992
-76.55	16.060	41.812	86.117	124.610	153.557	2233.992
-76.45	15.111	41.945	83.070	125.267	149.277	2233.992
-76.35	15.052	41.115	82.926	123.382	153.285	2233.992
-76.25	15.146	42.726	83.056	125.907	152.054	2233.992
-76.15	15.609	42.859	84.962	126.824	151.009	2233.992
-76.05	16.214	41.580	84.558	128.329	153.442	2233.992
-75.95	15.514	43.340	83.734	122.845	151.539	2233.992
-75.85	14.613	42.211	81.843	125.907	151.181	2233.992
-75.75	15.479	42.443	83.908	122.534	151.610	2233.992
-75.65	15.253	41.962	83.345	125.042	151.195	2233.992
-75.55	15.929	41.613	84.009	127.014	153.929	2233.992
-75.45	15.645	40.866	81.756	125.094	153.657	2233.992
-75.35	15.763	41.696	84.789	124.073	151.281	2233.992
-75.25	15.040	40.600	83.503	125.111	150.551	2233.992
-75.15	15.621	42.360	82.189	125.319	153.843	2233.992
-75.05	14.933	42.560	85.828	125.993	150.236	2233.992
-74.95	15.052	43.257	83.200	126.114	152.254	2233.992
-74.85	15.621	41.646	84.182	126.391	153.199	2233.992
-74.75	15.989	40.019	84.009	126.478	149.763	2233.992
-74.65	15.538	41.879	81.309	127.325	155.819	2233.992
-74.55	15.633	42.161	82.854	126.080	151.911	2233.992
-74.45	15.230	41.812	85.684	122.949	151.467	2233.992
-74.35	15.218	42.244	81.872	127.481	152.197	2233.992
-74.25	15.740	42.477	83.041	126.011	150.995	2233.992
-74.15	15.004	41.829	85.669	125.129	151.080	2233.992
-74.05	15.313	41.397	82.247	123.658	153.414	2233.992
-73.95	15.431	42.144	83.402	125.492	151.009	2233.992
-73.85	15.870	43.025	84.384	125.077	152.369	2233.992
-73.75	15.609	41.829	82.984	122.690	151.238	2233.992
-73.65	14.577	42.078	82.911	127.862	150.952	2233.992
-73.55	15.241	41.264	82.738	126.080	148.761	2233.992
-73.45	16.167	40.202	84.745	126.754	154.602	2233.992
-73.35	15.277	43.506	82.883	127.637	151.610	2233.992

Midpoint	10	3	1	0.33	0.1	Volume
-73.25	15.158	42.261	82.680	125.250	153.357	2233.992
-73.15	15.550	39.737	82.709	123.330	150.680	2233.992
-73.05	16.036	42.061	81.135	124.385	152.397	2233.992
-72.95	15.941	41.430	83.605	126.616	151.424	2233.992
-72.85	14.933	41.181	83.691	123.831	153.600	2233.992
-72.75	16.060	42.543	84.182	124.990	152.254	2233.992
-72.65	15.301	41.696	82.911	124.696	153.800	2233.992
-72.55	16.178	41.995	83.374	124.073	151.739	2233.992
-72.45	15.550	40.982	83.662	126.979	152.383	2233.992
-72.35	15.775	40.966	83.633	123.209	152.498	2233.992
-72.25	16.072	41.414	82.002	125.336	152.884	2233.992
-72.15	15.751	42.543	83.951	124.592	149.620	2233.992
-72.05	14.909	42.095	82.810	126.979	152.626	2233.992
-71.95	15.111	41.430	82.810	124.938	150.436	2233.992
-71.85	15.917	42.045	85.554	126.772	152.626	2233.992
-71.75	15.384	41.215	84.976	128.450	148.404	2233.992
-71.65	15.419	42.244	84.269	124.834	152.541	2233.992
-71.55	15.135	40.418	84.471	123.693	152.770	2233.992
-71.45	15.455	41.098	84.644	124.575	150.866	2233.992
-71.35	15.775	40.451	82.421	123.779	150.193	2233.992
-71.25	16.084	41.430	84.038	126.997	150.222	2233.992
-71.15	15.313	41.563	84.529	124.437	150.551	2233.992
-71.05	15.989	41.248	82.825	123.485	154.373	2233.992
-70.95	14.874	42.178	83.287	123.745	152.054	2233.992
-70.85	15.372	42.709	82.392	124.592	148.761	2233.992
-70.75	15.538	42.095	82.464	124.817	149.864	2233.992
-70.65	15.087	42.327	83.056	123.970	152.197	2233.992
-70.55	15.917	42.261	84.197	123.641	151.510	2233.992
-70.45	15.846	41.464	83.215	125.042	152.283	2233.992
-70.35	14.909	40.882	83.821	124.281	152.226	2233.992
-70.25	16.344	41.862	79.966	127.066	152.755	2233.992
-70.15	15.099	41.945	83.114	124.644	155.532	2233.992
-70.05	15.562	41.729	83.388	127.533	149.248	2233.992
-69.95	15.004	42.875	82.839	125.855	153.156	2233.992
-69.85	15.431	42.095	81.107	125.769	153.700	2233.992
-69.75	15.443	41.098	82.493	126.754	150.995	2233.992
-69.65	14.957	42.958	84.875	125.232	149.348	2233.992
-69.55	15.633	41.680	82.377	125.388	151.066	2233.992
-69.45	15.372	40.617	84.644	124.662	150.078	2233.992
-69.35	15.633	41.945	81.511	123.226	151.567	2233.992
-69.25	15.645	42.244	82.550	124.575	152.054	2233.992
-69.15	15.443	41.198	82.781	124.748	150.508	2233.992
-69.05	15.965	42.609	82.334	126.426	152.140	2233.992
-68.95	15.751	41.746	83.085	122.430	155.919	2233.992
-68.85	15.336	41.513	84.919	128.553	152.469	2233.992

Midpoint	10	3	1	0.33	0.1	Volume
-68.75	15.419	43.340	84.269	124.143	148.933	2233.992
-68.65	15.787	41.513	82.522	123.260	153.600	2233.992
-68.55	15.763	41.181	82.016	123.243	151.882	2233.992
-68.45	16.036	41.430	82.883	126.218	150.365	2233.992
-68.35	15.657	40.351	82.998	123.797	149.491	2233.992
-68.25	15.182	40.600	84.615	126.011	150.622	2233.992
-68.15	15.799	41.430	83.215	127.429	154.187	2233.992
-68.05	15.075	41.713	82.839	127.256	152.813	2233.992
-67.95	15.206	40.567	84.067	122.845	149.105	2233.992
-67.85	14.719	40.584	84.615	123.036	154.101	2233.992
-67.75	15.479	42.178	82.753	125.855	149.649	2233.992
-67.65	15.799	41.962	82.377	123.364	155.189	2233.992
-67.55	15.336	39.820	84.803	126.979	152.340	2233.992
-67.45	14.708	41.513	82.016	129.782	149.849	2233.992
-67.35	15.194	42.045	82.926	124.402	149.391	2233.992
-67.25	15.502	41.264	84.428	127.118	150.522	2233.992
-67.15	15.407	40.949	83.244	126.339	154.573	2233.992
-67.05	15.965	40.351	81.843	125.129	152.197	2233.992
-66.95	15.929	41.364	82.767	122.707	150.808	2233.992
-66.85	15.728	41.547	82.897	124.160	156.778	2233.992
-66.75	15.787	41.696	82.940	124.852	153.357	2233.992
-66.65	16.321	42.759	83.359	123.641	156.162	2233.992
-66.55	15.550	42.178	84.095	124.073	152.326	2233.992
-66.45	16.024	41.298	82.204	126.426	152.698	2233.992
-66.35	15.490	40.434	81.684	127.619	151.510	2233.992
-66.25	15.799	42.775	83.171	126.409	150.107	2233.992
-66.15	15.372	42.144	85.135	124.662	149.993	2233.992
-66.05	15.621	42.045	82.363	124.108	152.054	2233.992
-65.95	15.929	40.301	83.561	127.706	154.230	2233.992
-65.85	15.111	41.264	82.738	125.163	154.702	2233.992
-65.75	15.443	40.600	83.215	125.820	149.778	2233.992
-65.65	15.716	42.061	82.550	122.430	150.465	2233.992
-65.55	15.657	42.211	84.731	124.367	153.643	2233.992
-65.45	15.775	41.563	81.771	124.056	154.774	2233.992
-65.35	15.158	40.982	82.767	125.492	152.125	2233.992
-65.25	16.190	43.523	81.583	123.520	153.013	2233.992
-65.15	14.980	41.248	83.200	123.987	153.256	2233.992
-65.05	15.906	40.816	82.435	128.294	155.633	2233.992
-64.95	15.621	43.191	83.590	122.638	153.314	2233.992
-64.85	15.502	41.364	83.489	125.699	153.442	2233.992
-64.75	15.479	42.692	83.792	125.665	152.469	2233.992
-64.65	15.514	40.268	83.590	123.952	151.166	2233.992
-64.55	15.680	41.198	81.439	123.330	152.626	2233.992
-64.45	15.241	41.929	84.601	123.485	150.751	2233.992
-64.35	14.814	42.211	83.460	126.703	149.835	2233.992

Midpoint	10	3	1	0.33	0.1	Volume
-64.25	15.455	42.443	82.536	125.423	150.494	2233.992
-64.15	14.672	42.028	84.890	123.157	151.553	2233.992
-64.05	15.384	41.746	80.890	124.108	153.557	2233.992
-63.95	15.787	42.012	83.691	125.457	149.506	2233.992
-63.85	15.004	40.301	81.381	123.935	148.590	2233.992
-63.75	14.435	42.742	82.911	126.772	153.056	2233.992
-63.65	15.241	40.750	84.413	125.094	152.970	2233.992
-63.55	16.012	42.394	83.085	127.222	155.203	2233.992
-63.45	15.360	42.908	83.402	128.190	151.324	2233.992
-63.35	15.158	40.849	82.117	123.849	151.123	2233.992
-63.25	16.404	42.676	81.468	125.059	151.410	2233.992
-63.15	15.716	41.231	81.121	125.942	153.714	2233.992
-63.05	15.550	40.534	85.178	127.118	149.663	2233.992
-62.95	15.431	41.846	85.323	123.399	148.704	2233.992
-62.85	15.135	41.580	84.038	123.451	151.868	2233.992
-62.75	15.692	42.643	84.745	124.904	152.669	2233.992
-62.65	15.977	42.244	83.244	125.042	152.340	2233.992
-62.55	15.799	41.713	83.345	126.374	150.952	2233.992
-62.45	15.016	41.895	83.287	124.592	152.541	2233.992
-62.35	14.648	41.862	83.099	125.855	151.395	2233.992
-62.25	14.945	40.617	82.652	127.619	154.903	2233.992
-62.15	15.099	42.493	80.644	125.942	150.436	2233.992
-62.05	16.060	42.892	83.099	125.440	150.966	2233.992
-61.95	15.324	41.696	82.753	123.036	152.483	2233.992
-61.85	16.238	40.966	84.197	127.723	153.485	2233.992
-61.75	14.399	43.074	83.951	123.001	152.913	2233.992
-61.65	14.874	41.513	83.330	126.253	150.651	2233.992
-61.55	15.289	41.846	82.449	123.382	151.553	2233.992
-61.45	15.870	42.809	81.785	124.523	150.293	2233.992
-61.35	15.680	41.181	81.872	122.465	150.035	2233.992
-61.25	15.490	43.672	82.695	124.039	149.907	2233.992
-61.15	15.894	40.882	82.103	123.503	149.706	2233.992
-61.05	15.265	41.447	82.262	125.371	150.823	2233.992
-60.95	15.704	41.132	84.745	124.765	151.768	2233.992
-60.85	15.834	41.630	83.937	125.284	152.741	2233.992
-60.75	15.182	41.646	84.471	124.160	154.058	2233.992
-60.65	15.407	42.360	82.262	127.308	151.009	2233.992
-60.55	15.894	40.799	82.666	126.218	152.741	2233.992
-60.45	15.324	40.866	81.569	124.956	151.896	2233.992
-60.35	15.573	42.311	83.532	127.291	156.177	2233.992
-60.25	15.075	41.597	85.973	122.845	151.911	2233.992
-60.15	15.917	42.161	82.911	122.569	153.228	2233.992
-60.05	15.787	41.314	81.655	123.918	151.853	2233.992
-59.95	16.060	41.862	81.872	125.820	152.483	2233.992
-59.85	14.767	41.812	83.576	123.555	153.958	2233.992

Midpoint	10	3	1	0.33	0.1	Volume
-59.75	15.538	41.414	83.489	125.232	150.479	2233.992
-59.65	15.953	40.202	82.493	127.412	149.391	2233.992
-59.55	15.455	42.144	84.327	125.959	152.526	2233.992
-59.45	15.052	42.543	82.334	127.775	150.694	2233.992
-59.35	14.494	41.397	85.337	126.547	148.876	2233.992
-59.25	15.894	42.477	84.644	126.460	151.539	2233.992
-59.15	15.455	42.477	84.341	126.893	151.624	2233.992
-59.05	15.621	41.115	82.955	124.644	154.960	2233.992
-58.95	15.811	40.584	84.168	122.032	154.473	2233.992
-58.85	15.906	40.517	83.605	124.039	149.792	2233.992
-58.75	15.728	42.045	87.676	126.201	153.199	2233.992
-58.65	15.965	42.692	84.456	126.512	147.359	2233.992
-58.55	15.407	42.244	83.547	127.083	148.633	2233.992
-58.45	15.016	42.742	81.742	125.319	150.909	2233.992
-58.35	15.313	41.447	83.460	126.426	149.649	2233.992
-58.25	15.063	42.161	82.161	123.970	152.312	2233.992
-58.15	15.585	41.879	80.327	123.503	149.534	2233.992
-58.05	14.838	41.862	81.944	124.644	153.428	2233.992
-57.95	14.340	41.580	82.753	126.011	154.044	2233.992
-57.85	15.111	41.680	83.287	125.838	150.494	2233.992
-57.75	15.573	42.775	84.731	127.187	152.011	2233.992
-57.65	15.645	43.141	82.868	125.267	151.381	2233.992
-57.55	14.980	40.633	84.774	125.647	151.882	2233.992
-57.45	14.921	43.290	83.749	123.970	153.457	2233.992
-57.35	15.277	42.742	84.038	124.022	151.481	2233.992
-57.25	16.036	41.497	82.998	122.707	149.406	2233.992
-57.15	14.565	42.261	83.633	126.979	151.238	2233.992
-57.05	16.344	40.932	82.868	128.052	150.136	2233.992
-56.95	16.297	42.061	86.464	125.924	155.074	2233.992
-56.85	15.846	42.327	82.926	126.582	150.694	2233.992
-56.75	15.467	40.916	83.561	122.275	148.890	2233.992
-56.65	16.084	42.942	83.186	124.108	154.788	2233.992
-56.55	15.740	43.656	84.355	123.278	149.763	2233.992
-56.45	15.799	41.929	84.933	125.146	151.524	2233.992
-56.35	15.443	40.982	84.312	125.474	151.009	2233.992
-56.25	15.099	41.945	82.753	124.229	151.410	2233.992
-56.15	15.858	41.779	81.872	126.703	152.956	2233.992
-56.05	15.182	41.331	86.146	127.049	154.244	2233.992
-55.95	16.060	41.597	84.529	125.838	153.729	2233.992
-55.85	14.933	42.958	82.276	122.257	155.289	2233.992
-55.75	15.218	42.061	83.374	126.218	153.085	2233.992
-55.65	14.945	41.978	83.229	125.094	151.696	2233.992
-55.55	15.633	42.560	81.236	129.989	151.968	2233.992
-55.45	15.146	41.148	84.052	124.904	147.087	2233.992
-55.35	14.625	41.995	83.966	121.686	154.831	2233.992

Midpoint	10	3	1	0.33	0.1	Volume
-55.25	15.870	41.464	81.713	124.195	154.344	2233.992
-55.15	15.301	43.755	80.457	124.195	151.968	2233.992
-55.05	15.550	40.683	83.821	126.685	148.003	2233.992
-54.95	14.980	42.277	83.720	126.495	152.082	2233.992
-54.85	15.360	41.680	83.186	125.578	151.868	2233.992
-54.75	15.218	41.397	84.630	127.533	151.982	2233.992
-54.65	15.668	41.646	84.774	123.312	149.377	2233.992
-54.55	15.965	42.709	84.500	126.772	155.704	2233.992
-54.45	15.751	41.879	82.796	125.526	152.727	2233.992
-54.35	15.313	41.563	83.244	127.291	154.115	2233.992
-54.25	16.238	41.796	81.424	129.124	153.042	2233.992
-54.15	15.633	41.812	85.409	124.073	150.465	2233.992
-54.05	15.348	41.298	83.417	127.585	151.782	2233.992
-53.95	15.609	40.384	83.200	123.122	152.598	2233.992
-53.85	16.273	40.584	80.904	127.464	149.033	2233.992
-53.75	15.609	41.895	84.976	124.056	148.260	2233.992
-53.65	16.522	39.770	84.254	126.166	152.125	2233.992
-53.55	15.372	42.394	83.446	127.222	154.101	2233.992
-53.45	16.427	43.473	82.652	126.391	149.821	2233.992
-53.35	15.811	43.523	82.594	125.440	154.058	2233.992
-53.25	15.479	42.244	81.915	124.385	150.007	2233.992
-53.15	15.170	43.440	84.514	125.578	150.350	2233.992
-53.05	15.206	40.716	84.976	126.460	152.269	2233.992
-52.95	15.692	41.812	82.984	124.973	152.140	2233.992
-52.85	14.814	41.198	83.807	124.298	155.160	2233.992
-52.75	15.929	41.929	83.532	124.783	150.164	2233.992
-52.65	15.526	40.600	83.648	124.177	154.602	2233.992
-52.55	15.253	42.012	83.619	125.353	152.283	2233.992
-52.45	15.941	41.132	83.374	123.952	152.240	2233.992
-52.35	15.396	41.464	85.005	121.444	152.254	2233.992
-52.25	15.740	41.314	83.402	127.775	153.242	2233.992
-52.15	15.657	42.261	84.023	124.869	151.567	2233.992
-52.05	15.704	41.547	83.215	125.717	153.256	2233.992
-51.95	14.613	42.045	83.821	127.222	151.138	2233.992
-51.85	15.111	41.829	84.456	125.163	153.399	2233.992
-51.75	16.024	42.128	83.345	121.237	154.831	2233.992
-51.65	15.490	40.932	82.724	124.143	152.841	2233.992
-51.55	15.882	42.775	81.987	125.855	150.594	2233.992
-51.45	15.550	40.285	84.485	124.696	152.283	2233.992
-51.35	16.368	40.534	80.977	126.651	154.187	2233.992
-51.25	15.206	41.829	83.374	126.132	154.645	2233.992
-51.15	15.265	41.829	85.597	126.045	151.309	2233.992
-51.05	16.273	41.962	83.301	128.467	147.588	2233.992
-50.95	15.455	41.995	85.294	126.616	148.217	2233.992
-50.85	15.526	41.763	83.200	126.339	147.201	2233.992

Midpoint	10	3	1	0.33	0.1	Volume
-50.75	15.775	42.111	83.330	125.890	150.479	2233.992
-50.65	15.230	40.849	82.522	126.703	152.526	2233.992
-50.55	14.791	41.929	83.099	125.042	148.547	2233.992
-50.45	16.297	41.763	83.460	126.132	152.326	2233.992
-50.35	14.921	42.061	82.074	125.440	153.328	2233.992
-50.25	15.040	40.550	85.077	121.392	153.428	2233.992
-50.15	16.226	40.152	84.197	125.232	151.052	2233.992
-50.05	15.799	42.194	83.287	126.772	152.784	2233.992
-49.95	15.787	42.809	83.691	122.447	150.365	2233.992
-49.85	16.167	41.364	83.980	124.558	153.242	2233.992
-49.75	16.012	42.095	84.442	126.789	151.281	2233.992
-49.65	15.573	40.434	83.099	127.118	151.138	2233.992
-49.55	15.894	40.567	82.319	125.319	150.751	2233.992
-49.45	15.182	41.198	83.691	128.830	149.950	2233.992
-49.35	15.253	41.414	85.453	124.056	155.046	2233.992
-49.25	15.692	41.547	83.417	124.921	153.442	2233.992
-49.15	15.633	42.028	83.330	125.682	153.085	2233.992
-49.05	15.811	42.228	82.117	126.287	149.262	2233.992
-48.95	15.834	43.722	85.525	123.987	150.064	2233.992
-48.85	15.265	41.879	82.709	124.748	152.269	2233.992
-48.75	15.502	42.510	83.085	127.187	151.252	2233.992
-48.65	14.969	41.846	84.962	126.772	152.226	2233.992
-48.55	15.633	41.065	83.041	124.575	150.121	2233.992
-48.45	15.704	41.098	84.803	125.302	153.385	2233.992
-48.35	15.111	42.692	85.395	124.367	155.461	2233.992
-48.25	14.980	41.132	84.789	126.357	152.011	2233.992
-48.15	15.419	40.999	80.529	126.080	152.354	2233.992
-48.05	15.372	41.895	81.554	123.278	155.060	2233.992
-47.95	15.253	41.397	84.688	126.616	150.995	2233.992
-47.85	15.799	42.261	83.836	127.429	149.348	2233.992
-47.75	15.965	41.563	81.670	125.440	152.440	2233.992
-47.65	15.289	40.949	81.713	127.308	148.432	2233.992
-47.55	15.230	41.597	83.518	124.731	155.046	2233.992
-47.45	15.431	41.364	84.023	124.073	147.903	2233.992
-47.35	15.040	43.074	82.955	128.034	151.639	2233.992
-47.25	15.135	40.401	82.608	127.031	155.175	2233.992
-47.15	15.277	42.277	82.363	126.132	154.917	2233.992
-47.05	15.514	41.530	82.637	124.886	152.555	2233.992
-46.95	14.577	41.397	82.781	124.091	155.361	2233.992
-46.85	15.929	41.862	83.951	125.198	151.696	2233.992
-46.75	15.146	42.344	82.377	122.828	148.790	2233.992
-46.65	16.024	41.480	82.550	123.433	151.939	2233.992
-46.55	15.573	41.464	82.117	126.011	152.727	2233.992
-46.45	15.609	40.135	84.760	123.468	154.588	2233.992
-46.35	15.597	41.215	82.623	124.212	152.097	2233.992

Midpoint	10	3	1	0.33	0.1	Volume
-46.25	15.751	41.696	82.666	126.460	150.723	2233.992
-46.15	15.194	42.377	83.301	126.582	150.365	2233.992
-46.05	14.886	41.447	84.240	123.658	153.085	2233.992
-45.95	15.740	40.916	83.186	123.122	154.845	2233.992
-45.85	16.178	42.012	82.132	125.474	151.553	2233.992
-45.75	15.692	42.510	83.186	124.091	153.271	2233.992
-45.65	16.368	41.829	81.525	122.499	151.095	2233.992
-45.55	15.704	41.846	83.561	124.904	152.870	2233.992
-45.45	15.372	42.061	83.186	126.582	151.882	2233.992
-45.35	14.909	41.049	85.280	128.329	152.183	2233.992
-45.25	15.787	41.829	84.991	125.613	153.328	2233.992
-45.15	15.099	41.513	85.337	128.052	150.780	2233.992
-45.05	15.336	44.453	84.529	126.962	152.598	2233.992
-44.95	15.562	41.862	81.468	123.468	152.541	2233.992
-44.85	16.368	41.032	81.208	123.641	150.837	2233.992
-44.75	14.886	43.440	82.623	126.772	149.763	2233.992
-44.65	16.155	42.178	85.063	127.222	153.342	2233.992
-44.55	15.787	42.410	83.215	127.550	151.066	2233.992
-44.45	16.107	42.294	84.052	125.025	153.858	2233.992
-44.35	14.198	42.095	84.789	125.734	154.416	2233.992
-44.25	15.953	41.812	84.327	128.380	154.344	2233.992
-44.15	15.763	42.327	83.980	125.976	150.866	2233.992
-44.05	15.941	41.347	83.215	127.481	152.426	2233.992
-43.95	16.238	39.521	84.485	124.056	152.197	2233.992
-43.85	14.660	40.999	81.886	126.789	153.500	2233.992
-43.75	15.170	41.713	84.500	126.841	153.414	2233.992
-43.65	15.609	42.676	83.099	126.063	155.232	2233.992
-43.55	15.419	42.942	83.027	122.690	152.526	2233.992
-43.45	15.870	42.859	81.468	125.872	153.042	2233.992
-43.35	15.004	42.809	84.991	128.882	149.978	2233.992
-43.25	15.657	43.606	84.327	122.932	153.085	2233.992
-43.15	14.850	40.633	85.063	125.855	150.508	2233.992
-43.05	15.645	42.626	84.659	123.520	151.395	2233.992
-42.95	15.597	42.659	83.807	125.423	153.027	2233.992
-42.85	16.487	41.513	82.218	127.100	151.352	2233.992
-42.75	16.202	41.198	81.554	124.627	157.551	2233.992
-42.65	15.490	41.181	82.406	123.485	154.802	2233.992
-42.55	16.060	43.739	84.586	124.644	151.281	2233.992
-42.45	15.740	40.750	81.612	123.226	155.532	2233.992
-42.35	16.285	41.298	82.161	125.302	154.115	2233.992
-42.25	15.135	41.563	83.966	124.696	151.309	2233.992
-42.15	14.708	41.978	81.843	124.592	150.808	2233.992
-42.05	15.858	42.095	81.092	125.336	154.373	2233.992
-41.95	16.178	43.141	83.922	123.312	153.385	2233.992
-41.85	14.352	42.842	82.753	125.198	155.189	2233.992

Midpoint	10	3	1	0.33	0.1	Volume
-41.75	14.886	42.377	82.464	125.734	153.442	2233.992
-41.65	15.218	43.490	81.930	122.966	153.113	2233.992
-41.55	15.313	41.663	83.330	125.232	152.183	2233.992
-41.45	16.024	42.045	83.041	127.498	153.714	2233.992
-41.35	14.933	41.995	84.659	125.613	154.201	2233.992
-41.25	15.218	41.829	85.698	124.592	152.226	2233.992
-41.15	15.135	43.556	84.673	125.890	154.960	2233.992
-41.05	16.095	42.676	83.229	127.567	155.146	2233.992
-40.95	15.834	41.198	85.150	124.644	151.811	2233.992
-40.85	15.158	42.277	82.088	124.679	153.972	2233.992
-40.75	16.072	42.443	82.724	123.572	149.177	2233.992
-40.65	16.048	40.667	84.760	125.751	151.138	2233.992
-40.55	15.787	42.942	84.861	127.689	155.232	2233.992
-40.45	15.348	43.008	83.879	125.959	150.250	2233.992
-40.35	15.977	41.630	84.312	125.336	148.103	2233.992
-40.25	15.479	41.945	81.901	126.011	152.498	2233.992
-40.15	15.704	43.490	84.688	128.398	153.085	2233.992
-40.05	14.803	42.859	82.074	129.072	156.449	2233.992
-39.95	15.846	42.659	83.937	125.319	153.170	2233.992
-39.85	16.084	41.165	82.637	126.668	153.271	2233.992
-39.75	15.194	40.750	84.731	124.471	153.757	2233.992
-39.65	15.052	42.128	83.374	123.693	150.107	2233.992
-39.55	15.668	40.700	84.153	129.747	151.352	2233.992
-39.45	15.087	42.360	84.500	123.658	153.657	2233.992
-39.35	15.467	41.132	81.049	124.091	153.886	2233.992
-39.25	15.692	40.550	82.652	127.429	154.573	2233.992
-39.15	15.479	43.041	82.161	127.222	154.903	2233.992
-39.05	15.573	40.799	82.652	124.454	151.768	2233.992
-38.95	15.028	43.207	83.316	124.143	150.980	2233.992
-38.85	15.846	40.882	84.023	125.388	153.357	2233.992
-38.75	15.123	42.809	82.421	124.073	154.430	2233.992
-38.65	15.455	42.742	85.756	128.432	153.471	2233.992
-38.55	15.372	41.945	84.803	127.325	155.074	2233.992
-38.45	16.000	42.991	82.608	126.357	153.256	2233.992
-38.35	14.933	41.298	81.771	127.343	157.279	2233.992
-38.25	16.463	41.779	84.803	124.800	155.275	2233.992
-38.15	15.787	41.929	83.720	126.893	154.172	2233.992
-38.05	15.633	44.104	84.644	123.624	152.684	2233.992
-37.95	15.265	39.936	83.836	126.184	151.911	2233.992
-37.85	15.787	40.484	83.922	123.243	155.289	2233.992
-37.75	15.562	42.593	83.359	126.080	151.481	2233.992
-37.65	15.265	41.414	83.547	126.011	153.858	2233.992
-37.55	15.455	42.659	83.157	127.394	152.455	2233.992
-37.45	16.368	42.194	84.124	124.316	155.203	2233.992
-37.35	15.763	40.102	85.669	126.374	153.972	2233.992

Midpoint	10	3	1	0.33	0.1	Volume
-37.25	15.609	42.012	84.312	124.160	150.021	2233.992
-37.15	15.775	41.879	83.605	127.723	152.841	2233.992
-37.05	15.289	43.722	85.828	125.129	152.512	2233.992
-36.95	16.416	39.836	84.976	126.478	153.714	2233.992
-36.85	15.277	42.194	85.539	126.668	151.324	2233.992
-36.75	15.407	42.360	83.330	128.052	153.357	2233.992
-36.65	15.633	42.493	86.723	128.346	152.941	2233.992
-36.55	15.431	40.766	83.850	128.450	153.872	2233.992
-36.45	15.443	43.025	84.890	127.083	150.551	2233.992
-36.35	14.838	42.128	84.283	125.353	152.068	2233.992
-36.25	15.052	41.015	84.009	126.166	153.743	2233.992
-36.15	15.384	42.991	84.067	125.942	152.383	2233.992
-36.05	15.751	42.277	84.832	125.440	151.367	2233.992
-35.95	15.562	42.526	84.225	127.100	153.528	2233.992
-35.85	15.657	41.713	86.348	126.287	151.768	2233.992
-35.75	15.858	43.656	83.994	124.886	152.297	2233.992
-35.65	15.502	43.589	84.875	124.938	155.346	2233.992
-35.55	15.384	42.128	85.539	125.371	152.727	2233.992
-35.45	16.012	42.825	83.734	127.481	152.211	2233.992
-35.35	15.170	44.038	85.092	127.170	149.248	2233.992
-35.25	15.597	42.593	85.150	125.526	155.618	2233.992
-35.15	15.740	41.729	84.327	126.045	153.571	2233.992
-35.05	15.301	41.779	83.677	127.291	152.412	2233.992
-34.95	16.131	43.224	84.370	123.468	154.402	2233.992
-34.85	14.945	40.866	83.720	125.492	154.373	2233.992
-34.75	15.277	42.659	83.792	124.523	154.473	2233.992
-34.65	16.119	42.576	81.973	128.951	155.532	2233.992
-34.55	16.024	42.294	82.464	127.273	153.242	2233.992
-34.45	15.431	43.722	82.854	125.838	152.956	2233.992
-34.35	15.989	42.609	84.919	125.699	153.285	2233.992
-34.25	15.502	40.418	84.485	126.357	150.121	2233.992
-34.15	15.123	41.995	83.085	128.623	155.031	2233.992
-34.05	15.550	41.065	84.803	128.173	155.017	2233.992
-33.95	16.155	41.895	86.189	129.003	153.643	2233.992
-33.85	15.763	43.888	84.558	126.564	153.614	2233.992
-33.75	15.775	41.530	84.673	125.302	154.444	2233.992
-33.65	16.107	42.061	84.211	126.530	153.815	2233.992
-33.55	15.384	42.792	85.467	128.034	151.610	2233.992
-33.45	15.502	43.108	84.384	127.913	151.997	2233.992
-33.35	15.502	42.759	82.796	127.066	154.974	2233.992
-33.25	15.538	41.082	83.807	124.143	151.811	2233.992
-33.15	16.427	40.999	84.009	128.605	155.804	2233.992
-33.05	16.273	41.945	82.738	125.232	151.524	2233.992
-32.95	16.024	42.908	85.641	124.558	157.307	2233.992
-32.85	15.372	41.231	82.753	128.173	152.927	2233.992

Midpoint	10	3	1	0.33	0.1	Volume
-32.75	15.277	42.842	84.355	126.616	152.512	2233.992
-32.65	15.313	42.294	83.605	125.319	155.146	2233.992
-32.55	15.324	42.643	84.688	128.156	153.815	2233.992
-32.45	16.285	42.427	83.417	122.361	151.768	2233.992
-32.35	15.324	42.128	84.240	126.720	153.042	2233.992
-32.25	15.170	42.593	83.706	128.899	154.158	2233.992
-32.15	15.562	41.680	84.240	128.536	155.976	2233.992
-32.05	15.479	41.331	82.291	123.745	151.524	2233.992
-31.95	15.230	43.705	84.630	130.716	153.314	2233.992
-31.85	15.431	42.709	84.442	129.314	156.220	2233.992
-31.75	15.989	41.796	82.883	126.685	153.170	2233.992
-31.65	15.253	42.377	84.485	122.845	154.273	2233.992
-31.55	15.396	41.547	83.879	128.329	152.770	2233.992
-31.45	15.609	40.102	83.590	124.108	154.988	2233.992
-31.35	16.143	41.381	82.680	128.121	153.786	2233.992
-31.25	15.609	42.443	83.908	124.160	153.385	2233.992
-31.15	15.289	42.477	83.475	126.339	155.060	2233.992
-31.05	15.834	41.630	85.178	126.876	155.676	2233.992
-30.95	15.929	40.351	83.114	127.360	156.449	2233.992
-30.85	14.767	42.892	83.677	126.512	155.103	2233.992
-30.75	15.396	39.288	85.756	128.744	150.379	2233.992
-30.65	15.811	43.124	85.424	128.329	153.958	2233.992
-30.55	15.407	42.261	81.699	127.187	152.397	2233.992
-30.45	14.826	42.394	81.626	123.641	151.853	2233.992
-30.35	15.597	41.613	82.377	126.097	156.592	2233.992
-30.25	14.648	41.049	83.605	125.578	150.365	2233.992
-30.15	14.743	41.480	85.900	127.100	151.753	2233.992
-30.05	15.123	41.978	82.926	126.028	151.267	2233.992
-29.95	15.550	41.364	84.355	126.910	154.631	2233.992
-29.85	15.490	41.630	83.258	123.710	153.256	2233.992
-29.75	15.324	40.849	83.085	123.710	151.782	2233.992
-29.65	15.834	41.547	82.955	124.800	153.271	2233.992
-29.55	16.119	41.430	82.825	125.993	150.336	2233.992
-29.45	15.846	42.045	84.586	125.215	149.305	2233.992
-29.35	15.941	39.537	82.883	124.817	149.048	2233.992
-29.25	15.348	42.427	82.536	123.312	149.993	2233.992
-29.15	14.850	40.633	80.659	124.731	151.624	2233.992
-29.05	15.004	40.501	83.345	124.973	150.651	2233.992
-28.95	15.336	41.248	80.659	123.018	150.078	2233.992
-28.85	15.194	39.936	81.453	125.942	147.645	2233.992
-28.75	15.123	40.069	81.684	127.170	150.307	2233.992
-28.65	14.921	40.318	81.525	119.075	150.293	2233.992
-28.55	15.882	41.680	82.810	120.666	149.749	2233.992
-28.45	15.372	41.812	81.236	119.593	151.453	2233.992
-28.35	14.506	41.530	79.980	125.284	148.933	2233.992

Midpoint	10	3	1	0.33	0.1	Volume
-28.25	14.660	39.421	83.013	121.721	148.332	2233.992
-28.15	15.241	42.144	81.208	122.275	146.500	2233.992
-28.05	15.372	40.849	79.850	122.378	150.007	2233.992
-27.95	14.980	39.305	81.540	120.251	149.592	2233.992
-27.85	14.435	39.454	79.403	122.984	147.645	2233.992
-27.75	15.455	41.447	81.034	120.147	145.183	2233.992
-27.65	14.126	39.654	80.471	123.503	143.565	2233.992
-27.55	14.601	38.491	79.605	122.690	146.170	2233.992
-27.45	14.850	37.678	80.500	120.579	146.457	2233.992
-27.35	14.945	40.285	79.605	121.548	147.301	2233.992
-27.25	14.980	40.783	81.049	120.683	151.009	2233.992
-27.15	15.692	41.480	78.912	122.136	146.256	2233.992
-27.05	14.613	41.198	79.807	119.836	144.810	2233.992
-26.95	14.779	40.833	80.125	119.887	145.841	2233.992
-26.85	14.719	40.135	77.858	120.493	149.091	2233.992
-26.75	14.459	40.019	79.432	119.455	144.581	2233.992
-26.65	14.969	40.982	79.995	120.977	144.052	2233.992
-26.55	14.648	40.799	80.702	119.455	146.686	2233.992
-26.45	15.230	39.853	79.533	119.680	146.600	2233.992
-26.35	15.230	39.969	79.417	118.417	144.338	2233.992
-26.25	14.043	39.886	78.941	121.427	147.401	2233.992
-26.15	14.803	38.641	81.208	120.268	144.080	2233.992
-26.05	14.198	39.537	80.067	121.202	145.798	2233.992
-25.95	14.387	39.305	79.316	119.905	146.385	2233.992
-25.85	14.233	39.471	76.775	120.476	144.080	2233.992
-25.75	14.743	39.222	77.670	120.527	146.986	2233.992
-25.65	13.972	40.916	77.612	118.902	142.864	2233.992
-25.55	14.850	37.910	80.081	119.524	145.168	2233.992
-25.45	14.838	40.368	79.403	120.700	145.612	2233.992
-25.35	14.672	40.334	78.926	118.417	146.915	2233.992
-25.25	14.625	38.126	78.031	120.666	144.825	2233.992
-25.15	14.767	40.916	77.872	118.521	144.395	2233.992
-25.05	14.636	39.039	79.374	120.614	143.765	2233.992
-24.95	13.640	38.956	79.634	118.348	143.808	2233.992
-24.85	14.648	39.870	78.883	118.054	144.553	2233.992
-24.75	15.016	38.591	79.056	119.697	144.023	2233.992
-24.65	15.431	37.694	76.962	117.846	145.125	2233.992
-24.55	14.565	39.803	79.850	119.697	144.338	2233.992
-24.45	15.075	39.587	79.533	121.790	144.467	2233.992
-24.35	14.696	40.251	77.569	120.631	146.242	2233.992
-24.25	14.209	38.923	78.738	120.406	146.657	2233.992
-24.15	14.625	40.916	80.312	121.150	142.663	2233.992
-24.05	15.241	39.537	78.926	119.697	142.506	2233.992
-23.95	14.376	39.969	79.561	120.510	145.970	2233.992
-23.85	14.672	39.554	79.836	120.891	148.532	2233.992

Midpoint	10	3	1	0.33	0.1	Volume
-23.75	14.767	38.807	78.002	121.133	145.698	2233.992
-23.65	14.613	39.637	78.565	119.801	144.295	2233.992
-23.55	14.091	39.056	79.980	120.078	147.215	2233.992
-23.45	15.324	38.691	80.370	117.552	146.399	2233.992
-23.35	14.803	39.488	79.460	121.773	142.091	2233.992
-23.25	14.376	38.890	80.904	119.957	147.817	2233.992
-23.15	14.530	38.807	79.706	120.233	147.330	2233.992
-23.05	14.459	40.700	79.201	122.084	147.831	2233.992
-22.95	14.518	39.919	78.782	120.251	148.446	2233.992
-22.85	14.577	38.591	78.955	119.836	145.855	2233.992
-22.75	14.625	40.135	79.475	118.988	146.514	2233.992
-22.65	14.340	38.691	80.486	119.161	143.279	2233.992
-22.55	13.984	38.392	80.298	122.067	146.299	2233.992
-22.45	15.704	40.185	80.644	122.932	147.960	2233.992
-22.35	14.814	38.890	79.605	118.279	147.545	2233.992
-22.25	14.281	39.886	80.673	119.663	146.872	2233.992
-22.15	14.779	39.604	79.403	120.476	147.573	2233.992
-22.05	14.909	39.753	80.948	118.504	147.917	2233.992
-21.95	15.158	40.716	79.460	120.977	146.142	2233.992
-21.85	14.969	40.268	82.449	122.465	145.125	2233.992
-21.75	15.514	40.019	80.847	122.603	148.547	2233.992
-21.65	14.992	40.683	80.688	121.150	150.794	2233.992
-21.55	14.862	40.766	81.872	121.358	147.473	2233.992
-21.45	15.289	38.259	82.204	122.880	147.602	2233.992
-21.35	14.897	40.932	80.356	119.369	145.698	2233.992
-21.25	14.352	39.471	81.049	123.053	147.731	2233.992
-21.15	15.075	40.484	81.323	120.112	149.134	2233.992
-21.05	15.158	39.471	80.731	120.476	149.177	2233.992
-20.95	15.111	40.501	81.482	121.894	146.772	2233.992
-20.85	15.063	39.571	81.135	122.326	149.950	2233.992
-20.75	14.209	40.052	81.771	122.275	149.477	2233.992
-20.65	15.206	39.787	82.291	123.572	151.682	2233.992
-20.55	15.123	40.152	81.742	125.163	149.735	2233.992
-20.45	14.909	40.069	81.005	122.240	149.191	2233.992
-20.35	15.360	41.215	82.623	124.679	148.776	2233.992
-20.25	13.498	41.846	81.078	121.929	147.817	2233.992
-20.15	15.419	40.019	82.810	120.960	152.426	2233.992
-20.05	15.360	40.949	81.713	125.198	149.148	2233.992
-19.95	15.135	39.903	82.088	123.900	150.293	2233.992
-19.85	14.459	41.945	79.561	124.143	150.164	2233.992
-19.75	14.969	42.975	81.193	124.195	151.739	2233.992
-19.65	15.621	40.085	81.626	124.471	155.031	2233.992
-19.55	14.886	41.530	81.063	123.900	150.408	2233.992
-19.45	15.941	42.311	80.182	122.897	152.884	2233.992
-19.35	14.791	41.098	83.215	127.533	154.631	2233.992

Midpoint	10	3	1	0.33	0.1	Volume
-19.25	14.803	40.849	83.576	126.962	155.547	2233.992
-19.15	12.146	32.862	67.764	102.175	127.217	2233.992
-19.05	1.210	3.022	5.906	9.617	11.237	12221.243
-18.95	1.008	2.823	5.314	8.683	10.779	139.219
-18.85	0.866	2.109	5.357	8.649	10.250	128.897
-18.75	1.044	2.424	5.501	7.870	10.564	121.873
-18.65	0.866	2.408	4.808	7.732	9.233	116.271
-18.55	0.890	2.325	4.534	6.625	9.434	111.530
-18.45	0.783	2.574	4.548	6.677	7.945	107.382
-18.35	0.557	2.209	4.072	6.832	8.303	103.676
-18.25	0.640	2.391	3.985	6.106	7.888	100.316
-18.15	0.700	1.910	4.014	5.968	8.059	97.236
-18.05	0.498	1.827	4.433	5.898	6.757	94.390
-17.95	0.629	1.993	3.668	5.795	8.074	91.743
-17.85	0.688	1.926	3.509	5.916	7.301	89.267
-17.75	0.724	2.242	3.336	6.141	7.286	86.940
-17.65	0.617	1.959	3.595	5.345	7.444	84.746
-17.55	0.818	1.943	4.101	6.175	6.198	82.670
-17.45	0.771	2.092	3.292	5.795	7.100	80.700
-17.35	0.605	1.544	2.960	5.414	6.327	78.826
-17.25	0.581	1.611	3.682	5.034	6.342	77.040
-17.15	0.522	1.594	3.047	5.137	6.227	75.335
-17.05	0.652	1.411	3.047	4.722	6.485	73.703
-16.95	0.498	1.644	3.162	5.258	6.499	72.140
-16.85	0.605	1.428	3.220	5.293	6.399	70.640
-16.75	0.439	1.345	3.220	4.791	6.442	69.199
-16.65	0.463	1.511	3.047	4.809	5.068	67.813
-16.55	0.546	1.428	3.003	4.238	6.256	66.479
-16.45	0.439	1.494	2.614	4.515	5.912	65.194
-16.35	0.640	1.445	2.902	5.120	5.311	63.953
-16.25	0.569	1.411	2.888	4.964	5.497	62.756
-16.15	0.356	1.611	2.787	4.238	5.526	61.600
-16.05	0.593	1.262	2.816	4.134	5.440	60.482
-15.95	0.534	1.312	3.119	4.272	4.681	59.400
-15.85	0.534	1.279	2.801	5.085	5.268	58.354
-15.75	0.415	1.245	2.556	4.566	4.624	57.340
-15.65	0.474	1.113	2.628	3.771	4.982	56.358
-15.55	0.593	1.395	2.772	4.463	4.509	55.405
-15.45	0.676	1.661	3.567	5.518	6.886	54.481
-15.35	0.593	1.777	4.650	6.919	8.389	53.585
-15.25	0.581	2.175	4.606	6.867	7.759	52.715
-15.15	0.617	1.976	4.476	7.628	8.489	51.870
-15.05	0.557	2.092	4.274	6.954	8.832	51.049
-14.95	0.474	1.876	4.303	7.057	7.587	50.252
-14.85	0.534	1.993	4.260	6.884	8.274	49.476

Midpoint	10	3	1	0.33	0.1	Volume
-14.75	0.451	1.777	4.476	6.694	8.474	48.723
-14.65	0.640	1.959	4.548	6.936	8.560	47.989
-14.55	0.486	2.042	4.274	7.092	8.403	47.276
-14.45	0.546	2.026	4.404	7.317	8.317	46.582
-14.35	0.557	2.142	4.491	6.383	8.503	45.906
-14.25	0.557	2.026	4.794	7.317	8.861	45.248
-14.15	0.617	2.225	4.765	7.248	8.503	44.608
-14.05	0.463	1.993	4.375	7.421	8.990	43.984
-13.95	0.569	1.710	4.866	7.092	8.618	43.377
-13.85	0.522	2.258	4.707	7.472	8.761	42.785
-13.75	0.557	2.458	4.953	7.005	9.133	42.208
-13.65	0.593	2.358	4.736	7.438	9.333	41.646
-13.55	0.593	2.242	5.256	7.853	9.648	41.098
-13.45	0.593	2.092	5.371	8.216	9.534	40.564
-13.35	0.640	2.441	5.213	8.061	9.834	40.043
-13.25	0.593	2.242	5.169	7.403	10.636	39.536
-13.15	0.581	1.777	4.953	7.369	10.106	39.041
-13.05	0.605	2.458	5.473	8.735	10.794	38.558
-12.95	0.688	2.491	5.819	8.528	10.020	38.088
-12.85	0.854	2.258	6.166	8.216	11.252	37.629
-12.75	0.842	2.341	5.790	8.735	11.051	37.181
-12.65	0.747	2.657	6.180	9.963	11.266	36.745
-12.55	0.581	2.474	6.223	8.943	11.538	36.319
-12.45	0.807	2.607	5.935	10.032	12.697	35.904
-12.35	0.747	2.840	6.527	10.759	12.454	35.499
-12.25	1.008	3.321	6.599	10.396	13.256	35.103
-12.15	0.724	3.089	7.682	10.845	13.356	34.718
-12.05	0.925	3.620	7.537	12.610	14.301	34.342
-11.95	0.771	3.221	8.057	11.589	13.900	33.976
-11.85	0.949	3.238	8.043	12.385	15.475	33.618
-11.75	1.032	3.022	8.332	13.526	16.677	33.269
-11.65	1.091	4.317	9.559	14.045	17.479	32.929
-11.55	1.257	4.002	9.458	15.274	18.266	32.597
-11.45	1.103	4.417	9.111	15.412	18.781	32.274
-11.35	1.198	4.699	10.266	16.605	19.740	31.959
-11.25	1.174	4.749	10.368	17.090	20.528	31.651
-11.15	1.483	5.065	11.739	18.214	22.618	31.351
-11.05	1.578	4.882	12.302	18.525	25.624	31.059
-10.95	1.423	5.895	12.519	20.030	24.106	30.775
-10.85	1.850	5.297	12.692	21.881	26.740	30.497
-10.75	1.637	5.712	14.194	23.109	28.057	30.227
-10.65	1.945	5.745	14.815	23.040	29.732	29.964
-10.55	2.088	6.891	15.840	25.168	31.894	29.707
-10.45	2.111	7.273	16.909	26.759	34.685	29.457
-10.35	2.325	8.120	17.169	27.433	34.986	29.214

Midpoint	10	3	1	0.33	0.1	Volume
-10.25	2.562	8.286	19.060	30.789	39.352	28.978
-10.15	2.550	8.984	19.421	30.443	38.765	28.747
-10.05	2.799	9.150	20.345	32.865	43.718	28.523
-9.95	2.479	9.150	21.298	33.072	44.434	28.306
-9.85	3.025	10.760	23.811	37.085	46.953	28.094
-9.75	2.858	10.677	23.782	40.406	48.170	27.888
-9.65	3.760	11.109	25.760	39.386	51.949	27.688
-9.55	3.843	11.856	27.262	43.364	55.800	27.493
-9.45	3.997	12.670	27.984	47.308	58.777	27.305
-9.35	3.523	13.401	28.504	46.668	59.679	27.122
-9.25	4.329	13.982	30.150	52.497	64.833	26.944
-9.15	4.519	15.161	33.615	54.210	67.338	26.772
-9.05	4.816	15.294	34.178	54.850	76.986	26.605
-8.95	4.863	15.642	36.185	63.360	80.665	26.443
-8.85	5.017	16.107	37.918	64.104	83.542	26.286
-8.75	5.278	17.668	39.824	65.678	88.610	26.135
-8.65	5.907	17.120	40.662	65.539	88.910	25.989
-8.55	5.468	18.266	39.391	64.259	92.375	25.847
-8.45	5.420	18.449	39.954	65.142	95.180	25.711
-8.35	5.634	17.801	41.903	70.192	92.632	25.579
-8.25	5.978	18.980	39.131	71.957	90.170	25.452
-8.15	5.420	16.821	38.929	70.832	87.007	25.330
-8.05	4.946	17.652	40.070	69.725	90.929	25.213
-7.95	5.136	16.356	37.846	66.249	86.534	25.100
-7.85	4.863	15.576	36.878	61.388	89.283	24.991
-7.75	4.709	15.078	37.196	61.942	85.933	24.888
-7.65	4.780	14.729	35.420	62.582	85.589	24.788
-7.55	4.400	13.600	32.200	59.139	77.530	24.694
-7.45	3.985	12.603	29.630	53.051	72.706	24.603
-7.35	3.796	14.198	25.933	47.775	68.927	24.517
-7.25	3.535	13.068	24.374	52.221	65.692	24.435
-7.15	3.440	11.292	25.211	50.525	59.565	24.358
-7.05	3.653	11.175	24.489	44.316	60.681	24.285
-6.95	3.262	9.548	23.522	44.506	54.354	24.216
-6.85	2.977	8.967	21.804	41.202	47.927	24.151
-6.75	2.870	7.472	18.959	35.961	42.630	24.091
-6.65	2.218	7.257	16.447	28.800	38.393	24.034
-6.55	2.348	6.177	16.504	26.067	36.246	23.982
-6.45	2.005	6.210	14.829	23.715	32.366	23.934
-6.35	1.981	4.932	14.165	21.172	32.524	23.890
-6.25	1.661	4.467	13.198	22.521	27.886	23.850
-6.15	1.411	3.985	11.393	20.272	29.059	23.814
-6.05	1.459	4.417	10.035	17.678	24.794	23.782
-5.95	1.257	4.284	8.895	15.083	24.378	23.754
-5.85	1.269	4.234	8.274	15.377	22.246	23.730

Midpoint	10	3	1	0.33	0.1	Volume
-5.75	1.234	3.620	7.985	13.786	18.810	23.710
-5.65	0.901	3.637	7.046	10.897	15.174	23.694
-5.55	0.640	2.723	5.285	12.125	15.002	23.682
-5.45	0.735	2.225	4.159	10.794	11.538	23.674
-5.35	0.439	1.345	2.715	5.570	8.117	23.670
-5.25	0.308	1.079	2.758	4.636	8.016	23.670
-5.15	0.237	0.880	2.960	4.186	8.002	23.670
-5.05	0.320	0.930	2.715	4.359	6.971	23.670
-4.95	0.356	0.830	2.657	4.809	8.360	23.670
-4.85	0.308	1.046	2.426	4.359	6.528	23.670
-4.75	0.237	0.963	2.671	4.030	6.442	23.670
-4.65	0.308	0.863	2.195	3.805	6.928	23.670
-4.55	0.178	0.947	1.964	3.321	6.313	23.670
-4.45	0.142	0.664	1.978	4.117	5.984	23.670
-4.35	0.285	0.980	1.993	3.754	6.742	23.670
-4.25	0.297	0.963	2.224	3.909	5.984	23.670
-4.15	0.261	0.780	2.094	4.151	5.654	23.670
-4.05	0.356	1.079	1.805	3.909	5.683	23.670
-3.95	0.368	0.714	2.050	3.892	5.468	23.670
-3.85	0.190	0.980	1.790	3.840	5.697	23.670
-3.75	0.308	0.714	2.267	4.342	6.585	23.670
-3.65	0.297	1.030	1.920	4.030	6.728	23.670
-3.55	0.285	0.980	2.281	4.688	5.984	23.670
-3.45	0.344	1.229	2.440	4.930	7.057	23.670
-3.35	0.273	1.079	2.339	5.708	7.659	23.670
-3.25	0.356	1.030	2.743	5.466	7.859	23.670
-3.15	0.285	1.196	2.498	5.414	8.174	23.670
-3.05	0.415	1.146	2.902	5.898	8.016	23.670
-2.95	0.486	1.262	3.206	6.435	9.534	23.670
-2.85	0.368	1.129	3.437	5.674	10.164	23.670
-2.75	0.439	1.777	3.076	6.659	9.892	23.670
-2.65	0.463	1.312	2.873	6.625	11.252	23.670
-2.55	0.463	1.411	3.826	7.092	11.867	23.670
-2.45	0.581	1.478	3.841	8.510	13.113	23.670
-2.35	0.415	1.611	4.187	8.026	11.996	23.670
-2.25	0.676	1.744	5.039	8.251	14.644	23.670
-2.15	0.807	1.760	4.823	8.908	16.061	23.670
-2.05	0.735	1.893	5.328	9.271	16.691	23.670
-1.95	0.712	2.092	6.166	10.586	17.307	23.670
-1.85	0.783	2.574	6.180	12.541	19.125	23.670
-1.75	0.878	2.491	6.541	12.575	20.571	23.670
-1.65	0.795	2.873	7.191	14.858	21.601	23.670
-1.55	1.032	3.338	7.768	15.827	22.432	23.670
-1.45	0.984	3.437	8.129	16.242	22.976	23.670
-1.35	1.056	3.620	8.938	18.318	26.898	23.670

Midpoint	10	3	1	0.33	0.1	Volume
-1.25	1.459	3.985	9.155	19.684	28.029	23.670
-1.15	1.435	4.832	10.281	21.258	31.736	23.670
-1.05	1.554	5.031	12.158	22.711	33.626	23.670
-0.95	1.589	5.613	11.595	22.850	36.174	23.670
-0.85	1.969	5.729	12.317	24.268	36.961	23.670
-0.75	1.838	5.563	13.775	27.849	41.327	23.670
-0.65	1.744	5.397	14.468	28.575	44.620	23.670
-0.55	1.921	5.812	15.638	31.325	48.814	23.670
-0.45	2.159	6.044	16.418	33.263	50.561	23.670
-0.35	2.088	6.310	18.020	31.602	53.610	23.670
-0.25	2.503	6.526	18.179	33.799	53.824	23.670
-0.05	2.692	7.024	17.891	34.162	54.154	23.670
0.05	2.775	7.174	18.714	32.830	54.454	23.670
0.25	2.515	7.140	18.165	35.425	50.475	23.670
0.35	2.491	6.908	16.981	32.640	54.741	23.670
0.45	2.242	6.360	16.591	32.553	51.448	23.670
0.55	2.206	6.858	16.143	31.723	48.284	23.670
0.65	1.969	6.609	15.566	31.550	46.581	23.670
0.75	2.230	5.712	14.829	28.627	43.646	23.670
0.85	1.921	5.862	15.335	29.250	41.127	23.670
0.95	1.803	5.513	13.544	26.603	37.820	23.670
1.05	1.910	4.965	12.274	24.787	36.102	23.670
1.15	1.755	5.048	11.682	23.369	32.466	23.670
1.25	1.661	4.965	10.743	21.552	29.732	23.670
1.35	1.234	4.450	9.718	20.082	28.444	23.670
1.45	1.245	3.686	8.664	18.162	26.082	23.670
1.55	0.984	3.836	8.288	16.294	23.749	23.670
1.65	0.925	3.221	7.610	14.616	22.775	23.670
1.75	0.961	2.823	7.148	14.495	19.483	23.670
1.85	1.079	2.856	7.061	13.319	19.297	23.670
1.95	1.056	2.441	6.036	12.298	17.593	23.670
2.05	1.044	2.474	6.195	11.745	16.147	23.670
2.15	0.700	2.076	5.603	11.537	15.546	23.670
2.25	0.759	2.076	5.617	10.240	14.258	23.670
2.35	0.712	2.209	4.881	9.635	13.599	23.670
2.45	0.700	1.611	4.895	8.735	13.399	23.670
2.55	0.747	1.843	4.332	9.531	11.924	23.670
2.65	0.557	1.993	4.043	8.493	11.853	23.670
2.75	0.557	1.578	4.144	7.922	11.137	23.670
2.85	0.640	1.843	4.115	8.251	12.025	23.670
2.95	0.629	1.578	4.187	8.095	11.080	23.670
3.05	0.676	1.677	4.029	7.680	11.137	23.670
3.15	0.557	1.511	3.668	7.922	10.908	23.670
3.25	0.534	1.245	3.509	7.386	10.622	23.670
3.35	0.391	1.644	4.346	7.749	10.393	23.670

Midpoint	10	3	1	0.33	0.1	Volume
3.45	0.510	1.793	4.144	6.746	9.777	23.670
3.55	0.581	1.827	3.942	6.936	9.763	23.670
3.65	0.617	1.478	3.552	7.386	10.564	23.670
3.75	0.522	1.611	4.332	7.178	10.464	23.670
3.85	0.427	1.411	4.289	7.161	9.978	23.670
3.95	0.546	1.578	4.057	8.095	10.564	23.670
4.05	0.557	1.694	3.812	7.213	10.207	23.670
4.15	0.557	1.461	3.783	7.524	10.550	23.670
4.25	0.403	1.677	4.433	8.251	10.622	23.670
4.35	0.486	1.843	4.577	8.458	11.194	23.670
4.45	0.581	1.843	4.274	7.645	11.065	23.670
4.55	0.510	1.627	3.942	7.611	11.796	23.670
4.65	0.415	2.042	4.534	8.372	11.037	23.670
4.75	0.605	2.358	4.404	8.424	11.810	23.670
4.85	0.510	1.677	4.317	8.303	11.638	23.670
4.95	0.546	1.644	4.722	7.628	11.581	23.670
5.05	0.581	1.910	4.404	7.680	11.137	23.670
5.15	0.557	1.959	4.837	8.216	12.039	23.670
5.25	0.569	1.893	4.736	9.029	11.710	23.670
5.35	0.546	2.076	4.462	8.718	11.724	23.670
5.45	1.281	4.650	10.642	16.882	24.307	23.674
5.55	1.376	4.882	10.700	18.595	25.996	23.682
5.65	1.352	5.281	11.422	18.871	26.969	23.694
5.75	1.578	4.849	12.721	19.027	27.442	23.710
5.85	1.483	4.733	11.768	19.719	26.826	23.730
5.95	1.566	5.181	12.201	20.393	27.270	23.754
6.05	1.400	4.915	12.071	20.999	27.513	23.782
6.15	1.483	6.127	13.154	22.590	26.898	23.814
6.25	1.708	5.031	12.952	20.428	30.376	23.850
6.35	1.838	5.397	13.024	22.019	29.603	23.890
6.45	1.921	5.696	13.486	22.279	29.804	23.934
6.55	1.506	5.762	13.891	21.725	30.763	23.982
6.65	1.637	5.762	13.530	24.977	31.507	24.034
6.75	1.506	5.696	14.194	23.974	31.808	24.091
6.85	1.898	6.559	14.555	25.462	32.924	24.151
6.95	1.755	6.991	15.566	25.064	33.640	24.216
7.05	1.803	6.941	15.753	24.856	35.673	24.285
7.15	2.135	6.924	16.158	26.776	36.102	24.358
7.25	1.862	6.543	17.169	27.676	36.017	24.435
7.35	1.921	7.539	16.735	28.454	36.804	24.517
7.45	2.135	7.788	16.981	27.779	38.164	24.603
7.55	2.325	7.223	17.587	27.589	38.178	24.694
7.65	2.337	8.054	18.353	30.443	37.620	24.788
7.75	2.526	7.954	18.483	30.495	39.781	24.888
7.85	2.621	7.406	17.530	29.457	38.708	24.991

Midpoint	10	3	1	0.33	0.1	Volume
7.95	2.325	7.755	18.555	30.097	38.608	25.100
8.05	2.301	7.340	17.876	30.633	39.094	25.213
8.15	2.408	7.605	19.017	31.568	39.194	25.330
8.25	2.301	7.605	18.179	29.544	39.080	25.452
8.35	2.360	7.489	17.587	28.610	36.861	25.579
8.45	2.467	7.954	16.836	28.177	35.702	25.711
8.55	2.111	7.638	17.140	27.174	35.415	25.847
8.65	2.099	7.722	15.421	26.292	34.084	25.989
8.75	1.981	6.493	16.244	26.275	32.567	26.135
8.85	2.016	6.476	16.129	25.496	31.751	26.286
8.95	1.720	6.509	14.757	23.593	29.790	26.443
9.05	1.708	6.327	13.588	22.590	28.644	26.605
9.15	1.494	5.696	12.822	20.463	26.125	26.772
9.25	1.696	5.247	12.302	19.235	25.882	26.944
9.35	1.530	5.812	11.999	18.958	23.935	27.122
9.45	1.352	4.517	11.797	18.439	22.847	27.305
9.55	1.483	4.899	10.555	17.021	21.172	27.493
9.65	1.186	4.102	10.324	16.830	20.227	27.688
9.75	1.293	3.852	9.458	16.138	19.125	27.888
9.85	1.210	4.317	8.909	14.115	18.581	28.094
9.95	1.067	4.118	9.054	13.994	17.794	28.306
10.05	1.091	3.786	8.115	12.938	16.319	28.523
10.15	0.830	3.670	7.754	13.526	15.059	28.747
10.25	0.842	3.321	8.057	12.177	14.744	28.978
10.35	0.961	3.188	7.711	11.693	14.659	29.214
10.45	0.984	3.055	7.581	11.606	12.798	29.457
10.55	0.973	2.989	7.032	11.053	13.342	29.707
10.65	0.901	3.138	6.700	9.704	12.497	29.964
10.75	0.783	2.740	6.195	9.981	11.524	30.227
10.85	0.842	2.790	6.267	8.908	12.125	30.497
10.95	0.640	2.424	6.036	8.631	11.695	30.775
11.05	0.795	2.375	5.588	8.839	10.522	31.059
11.15	0.593	2.142	5.213	8.061	11.567	31.351
11.25	0.617	2.507	4.722	8.424	10.135	31.651
11.35	0.557	2.507	5.242	8.285	10.092	31.959
11.45	0.569	2.275	4.751	8.320	9.634	32.274
11.55	0.605	2.242	5.010	7.715	9.491	32.597
11.65	0.522	2.209	5.270	7.369	9.247	32.929
11.75	0.546	2.441	4.650	7.749	8.961	33.269
11.85	0.557	1.827	4.779	7.075	8.861	33.618
11.95	0.688	1.910	4.462	6.781	8.217	33.976
12.05	0.534	2.125	4.216	6.504	8.618	34.342
12.15	0.463	1.644	4.289	6.538	8.102	34.718
12.25	0.498	1.793	3.899	6.123	7.387	35.103
12.35	0.510	2.109	4.187	6.798	7.616	35.499

Midpoint	10	3	1	0.33	0.1	Volume
12.45	0.439	2.092	3.494	5.950	8.045	35.904
12.55	0.629	2.009	3.956	6.141	7.630	36.319
12.65	0.498	2.142	4.072	5.829	7.100	36.745
12.75	0.486	2.009	3.754	6.158	7.086	37.181
12.85	0.534	1.843	4.043	6.089	7.315	37.629
12.95	0.510	1.461	4.173	5.743	6.642	38.088
13.05	0.380	1.627	3.624	5.743	6.900	38.558
13.15	0.581	1.827	3.292	5.466	6.957	39.041
13.25	0.415	1.727	3.798	5.552	6.499	39.536
13.35	0.320	1.428	3.595	5.846	6.485	40.043
13.45	0.534	1.362	3.783	5.328	6.384	40.564
13.55	0.356	1.594	3.769	6.054	6.513	41.098
13.65	0.427	2.026	3.307	5.241	5.569	41.646
13.75	0.391	1.461	3.552	5.085	6.470	42.208
13.85	0.498	1.544	3.234	5.431	6.771	42.785
13.95	0.498	1.843	3.364	5.258	6.571	43.377
14.05	0.439	1.279	3.465	5.276	6.384	43.984
14.15	0.415	1.627	3.234	5.189	6.399	44.608
14.25	0.391	1.445	3.697	5.656	6.170	45.248
14.35	0.344	1.661	3.552	5.155	6.270	45.906
14.45	0.403	1.777	3.090	5.276	6.284	46.582
14.55	0.403	1.578	3.653	5.103	6.957	47.276
14.65	0.427	1.478	3.783	5.120	5.955	47.989
14.75	0.344	1.793	3.653	5.431	7.000	48.723
14.85	0.356	1.445	3.364	5.950	6.098	49.476
14.95	0.356	1.578	3.855	4.791	6.814	50.252
15.05	0.463	1.494	3.480	5.656	6.327	51.049
15.15	0.356	1.611	3.697	5.051	6.542	51.870
15.25	0.534	1.710	4.130	5.483	6.642	52.715
15.35	0.320	1.827	3.494	5.933	6.184	53.585
15.45	0.510	1.146	2.801	4.151	5.583	54.481
15.55	0.308	0.947	2.224	3.529	4.481	55.405
15.65	0.403	1.063	2.599	3.615	4.380	56.358
15.75	0.308	1.146	2.411	3.286	4.452	57.340
15.85	0.463	1.030	2.166	3.944	4.481	58.354
15.95	0.427	1.262	2.729	3.529	4.753	59.400
16.05	0.557	0.980	2.888	3.494	4.939	60.482
16.15	0.356	1.279	2.354	3.459	4.509	61.600
16.25	0.510	1.146	2.368	3.823	4.638	62.756
16.35	0.640	1.395	2.585	4.324	4.280	63.953
16.45	0.463	1.212	2.065	4.203	5.125	65.194
16.55	0.474	1.129	2.960	3.944	4.910	66.479
16.65	0.498	1.046	2.541	4.151	4.438	67.813
16.75	0.486	1.079	2.989	4.203	4.910	69.199
16.85	0.546	1.345	2.859	4.515	5.125	70.640

Midpoint	10	3	1	0.33	0.1	Volume
16.95	0.463	1.129	3.177	4.463	5.025	72.140
17.05	0.557	1.661	3.336	5.328	5.626	73.703
17.15	0.415	1.578	2.657	4.653	5.998	75.335
17.25	0.451	1.561	2.960	4.099	5.468	77.040
17.35	0.439	1.727	3.090	4.411	5.855	78.826
17.45	0.688	1.528	3.480	4.307	6.528	80.700
17.55	0.569	1.661	2.917	5.379	5.841	82.670
17.65	0.664	1.644	3.480	4.982	6.284	84.746
17.75	0.688	1.611	3.364	5.397	7.057	86.940
17.85	0.534	1.744	3.278	5.345	6.356	89.267
17.95	0.605	1.395	3.364	4.826	6.699	91.743
18.05	0.759	2.258	3.668	5.414	7.229	94.390
18.15	0.818	1.760	3.451	5.518	7.587	97.236
18.25	0.771	2.175	3.798	5.777	6.928	100.316
18.35	0.878	1.843	4.592	6.469	7.029	103.676
18.45	0.724	2.059	4.159	6.141	7.787	107.382
18.55	0.878	2.076	4.346	6.227	9.305	111.530
18.65	0.676	2.209	4.418	6.867	8.145	116.271
18.75	0.818	2.657	5.112	7.144	9.978	121.873
18.85	0.842	2.225	4.303	7.801	9.577	128.897
18.95	0.913	2.756	5.097	8.476	9.419	139.219
19.05	1.234	2.690	5.920	9.064	10.579	12221.243
19.15	11.908	33.095	67.562	101.258	122.851	2233.992
19.25	15.585	39.288	81.496	122.499	149.005	2233.992
19.35	14.767	41.513	82.276	124.091	150.236	2233.992
19.45	15.206	40.683	82.493	123.295	150.035	2233.992
19.55	14.814	42.394	80.847	123.226	151.252	2233.992
19.65	14.553	41.929	83.244	123.243	148.976	2233.992
19.75	15.657	40.002	82.233	125.371	148.933	2233.992
19.85	15.324	41.065	82.594	122.880	147.559	2233.992
19.95	15.099	39.737	81.785	123.468	147.860	2233.992
20.05	15.087	42.526	80.847	123.693	148.446	2233.992
20.15	16.000	40.534	82.565	127.394	152.283	2233.992
20.25	15.158	40.251	81.236	126.668	148.890	2233.992
20.35	15.313	39.770	80.991	123.105	149.005	2233.992
20.45	14.601	40.069	80.832	122.430	150.136	2233.992
20.55	15.407	40.484	81.439	121.323	147.860	2233.992
20.65	14.791	39.620	80.746	122.032	148.761	2233.992
20.75	14.411	40.534	81.424	123.676	149.477	2233.992
20.85	14.803	38.541	78.392	119.334	147.716	2233.992
20.95	15.016	38.740	79.793	119.230	146.657	2233.992
21.05	14.269	40.683	79.287	120.441	148.876	2233.992
21.15	15.004	39.753	80.919	122.897	150.021	2233.992
21.25	15.123	40.152	78.536	119.922	149.391	2233.992
21.35	15.040	39.039	81.034	122.050	149.491	2233.992

Midpoint	10	3	1	0.33	0.1	Volume
21.45	14.684	40.501	79.186	119.420	148.604	2233.992
21.55	14.731	41.298	80.269	118.400	147.974	2233.992
21.65	15.075	39.072	80.746	118.971	147.788	2233.992
21.75	14.376	40.069	80.702	122.309	144.639	2233.992
21.85	15.396	39.504	80.298	119.472	149.678	2233.992
21.95	14.636	40.285	82.103	122.275	146.958	2233.992
22.05	15.526	39.006	80.529	120.060	147.545	2233.992
22.15	14.553	39.421	79.951	122.793	146.156	2233.992
22.25	14.696	40.401	79.374	120.320	149.778	2233.992
22.35	14.625	39.654	79.273	118.780	142.033	2233.992
22.45	14.150	39.687	78.118	120.095	146.256	2233.992
22.55	14.696	40.550	80.428	120.216	146.371	2233.992
22.65	14.387	39.986	81.828	124.056	144.367	2233.992
22.75	14.767	39.288	80.370	118.590	145.412	2233.992
22.85	14.281	40.135	79.287	121.444	146.442	2233.992
22.95	14.636	39.006	79.691	117.639	146.113	2233.992
23.05	14.138	39.936	80.688	119.386	143.179	2233.992
23.15	14.791	40.218	79.850	118.780	146.027	2233.992
23.25	14.067	38.358	79.908	121.825	145.927	2233.992
23.35	14.091	39.139	79.576	121.980	144.682	2233.992
23.45	14.969	41.032	79.489	120.476	145.884	2233.992
23.55	14.909	39.272	80.803	120.925	144.324	2233.992
23.65	15.443	39.338	80.081	120.389	143.909	2233.992
23.75	14.814	38.840	78.074	119.524	145.469	2233.992
23.85	14.292	40.434	79.287	118.210	147.616	2233.992
23.95	15.028	39.521	79.865	115.684	145.040	2233.992
24.05	14.696	41.630	80.413	118.521	142.806	2233.992
24.15	15.063	40.218	79.590	122.482	143.651	2233.992
24.25	14.459	39.056	80.096	118.244	146.299	2233.992
24.35	15.028	39.919	76.746	118.365	145.684	2233.992
24.45	15.194	40.600	77.150	116.255	146.142	2233.992
24.55	14.269	41.414	76.702	118.106	144.496	2233.992
24.65	14.245	39.421	78.738	117.085	148.046	2233.992
24.75	15.146	39.239	78.450	119.680	141.203	2233.992
24.85	14.862	38.989	78.883	117.881	145.440	2233.992
24.95	14.221	39.272	79.764	121.635	144.267	2233.992
25.05	14.340	39.787	79.330	118.971	144.181	2233.992
25.15	14.542	39.388	78.536	121.859	146.557	2233.992
25.25	14.542	40.185	78.623	119.939	146.929	2233.992
25.35	14.625	40.069	79.590	118.884	144.496	2233.992
25.45	15.135	38.989	77.468	118.106	145.097	2233.992
25.55	14.886	40.550	78.247	119.403	144.581	2233.992
25.65	14.838	37.960	78.738	120.787	144.009	2233.992
25.75	14.743	39.587	77.858	120.251	145.168	2233.992
25.85	14.459	39.488	78.608	119.455	146.070	2233.992

Midpoint	10	3	1	0.33	0.1	Volume
25.95	14.530	39.388	79.403	119.576	144.753	2233.992
26.05	14.138	39.853	78.450	116.532	146.099	2233.992
26.15	14.767	39.371	80.457	118.625	145.197	2233.992
26.25	14.886	39.853	78.536	120.925	145.011	2233.992
26.35	14.399	39.106	80.789	119.784	145.626	2233.992
26.45	14.553	40.550	78.421	121.756	144.825	2233.992
26.55	14.601	41.397	77.684	121.963	142.978	2233.992
26.65	14.470	39.388	79.966	123.797	145.569	2233.992
26.75	14.601	40.517	80.457	119.991	145.655	2233.992
26.85	13.676	40.584	80.601	119.593	145.841	2233.992
26.95	14.613	39.953	80.226	118.867	148.876	2233.992
27.05	14.459	39.239	78.825	120.303	147.631	2233.992
27.15	14.909	39.919	79.316	120.026	147.015	2233.992
27.25	14.506	42.194	80.500	121.704	143.436	2233.992
27.35	15.740	40.202	79.807	121.721	146.271	2233.992
27.45	15.490	41.430	79.518	122.136	146.414	2233.992
27.55	15.158	39.753	81.612	122.396	146.127	2233.992
27.65	15.324	39.571	79.951	124.056	146.170	2233.992
27.75	14.708	39.870	81.280	124.229	146.757	2233.992
27.85	14.708	40.849	79.836	122.707	151.438	2233.992
27.95	15.502	41.447	79.547	123.503	149.320	2233.992
28.05	15.230	41.132	80.486	120.320	149.134	2233.992
28.15	15.135	39.620	81.338	121.444	147.373	2233.992
28.25	15.040	41.314	79.071	120.510	149.577	2233.992
28.35	15.550	40.318	81.944	120.977	151.324	2233.992
28.45	15.158	41.895	83.085	123.762	149.262	2233.992
28.55	15.728	40.351	82.060	126.391	147.860	2233.992
28.65	15.253	41.281	79.894	124.506	147.888	2233.992
28.75	15.336	41.347	82.276	124.662	149.993	2233.992
28.85	14.862	41.298	81.872	123.883	148.747	2233.992
28.95	14.636	40.202	85.467	126.201	150.121	2233.992
29.05	15.514	41.215	82.291	127.135	149.678	2233.992
29.15	14.969	40.783	82.536	125.319	150.379	2233.992
29.25	15.597	42.045	80.514	123.987	151.739	2233.992
29.35	15.680	41.746	83.966	123.572	149.091	2233.992
29.45	15.431	40.982	81.699	125.561	152.283	2233.992
29.55	15.538	40.833	83.821	124.800	152.512	2233.992
29.65	15.846	41.049	83.215	126.184	152.798	2233.992
29.75	15.894	42.194	81.410	126.374	150.064	2233.992
29.85	14.435	41.746	82.652	121.375	149.878	2233.992
29.95	15.146	41.132	82.262	122.897	151.539	2233.992
30.05	15.099	43.091	84.615	126.530	151.453	2233.992
30.15	16.095	41.264	83.980	127.152	150.121	2233.992
30.25	15.004	41.829	82.984	122.534	149.878	2233.992
30.35	15.692	41.215	82.449	126.218	155.289	2233.992

Midpoint	10	3	1	0.33	0.1	Volume
30.45	15.431	41.331	83.966	124.921	152.426	2233.992
30.55	15.277	42.892	84.211	126.132	152.798	2233.992
30.65	15.467	42.111	82.305	125.129	154.988	2233.992
30.75	15.538	41.962	84.139	126.011	152.970	2233.992
30.85	15.206	41.862	83.778	127.533	149.978	2233.992
30.95	15.123	43.855	84.529	123.797	152.254	2233.992
31.05	16.261	43.008	84.355	124.938	154.573	2233.992
31.15	15.348	41.547	85.020	129.020	153.070	2233.992
31.25	15.396	41.846	82.348	123.745	153.571	2233.992
31.35	16.036	41.015	84.774	127.706	152.025	2233.992
31.45	15.230	41.264	83.446	125.371	152.312	2233.992
31.55	15.028	42.344	84.774	123.226	152.669	2233.992
31.65	15.989	41.447	84.991	121.998	153.056	2233.992
31.75	15.241	41.646	87.445	126.685	149.363	2233.992
31.85	15.336	40.866	82.911	130.473	152.297	2233.992
31.95	15.016	41.746	85.525	126.443	154.101	2233.992
32.05	16.250	42.360	82.969	127.740	153.958	2233.992
32.15	14.423	43.323	84.081	122.344	153.242	2233.992
32.25	15.526	43.074	84.471	126.979	151.009	2233.992
32.35	16.048	42.643	84.124	128.398	154.273	2233.992
32.45	15.265	42.194	84.832	124.973	152.712	2233.992
32.55	15.645	41.032	83.359	129.972	156.907	2233.992
32.65	14.565	42.078	86.666	125.959	155.647	2233.992
32.75	15.052	42.510	84.370	126.322	154.244	2233.992
32.85	16.107	39.537	84.514	124.610	154.588	2233.992
32.95	15.194	42.942	82.594	122.517	156.463	2233.992
33.05	15.419	42.726	83.619	127.706	153.900	2233.992
33.15	15.929	41.298	85.106	125.872	149.849	2233.992
33.25	15.538	42.161	84.558	127.862	154.774	2233.992
33.35	15.775	42.942	85.034	123.727	153.557	2233.992
33.45	15.597	43.672	83.272	125.250	152.727	2233.992
33.55	16.511	41.646	83.345	127.377	152.512	2233.992
33.65	15.799	43.456	83.099	122.153	153.328	2233.992
33.75	15.502	42.560	83.258	126.858	156.205	2233.992
33.85	15.740	41.132	85.251	127.550	152.813	2233.992
33.95	15.775	42.427	84.803	129.263	157.837	2233.992
34.05	16.226	42.028	86.059	126.063	155.103	2233.992
34.15	15.953	42.327	84.500	125.180	155.132	2233.992
34.25	15.253	42.975	83.085	124.834	154.244	2233.992
34.35	15.431	42.261	84.197	126.218	154.573	2233.992
34.45	15.846	42.228	85.294	125.647	157.608	2233.992
34.55	15.490	42.244	84.673	123.762	155.232	2233.992
34.65	15.277	41.414	84.413	128.415	150.164	2233.992
34.75	16.131	41.962	84.644	126.495	152.469	2233.992
34.85	15.633	42.261	83.518	125.267	152.813	2233.992

Midpoint	10	3	1	0.33	0.1	Volume
34.95	16.309	43.157	84.586	127.118	153.399	2233.992
35.05	15.977	43.274	82.348	127.394	152.541	2233.992
35.15	15.277	42.775	86.074	126.011	152.984	2233.992
35.25	15.621	41.912	84.962	126.841	154.201	2233.992
35.35	15.704	42.194	83.908	130.802	154.802	2233.992
35.45	16.570	42.775	86.045	127.170	151.825	2233.992
35.55	15.633	42.294	84.456	126.495	152.984	2233.992
35.65	16.060	42.244	83.893	125.353	152.784	2233.992
35.75	15.467	42.908	82.623	126.166	153.242	2233.992
35.85	15.277	41.082	83.518	128.294	153.915	2233.992
35.95	15.479	42.825	84.413	126.633	152.011	2233.992
36.05	15.241	42.078	85.323	128.277	155.246	2233.992
36.15	16.487	41.331	83.532	122.949	154.287	2233.992
36.25	15.811	41.929	82.363	125.474	152.455	2233.992
36.35	15.621	41.929	82.464	127.222	153.714	2233.992
36.45	16.380	42.543	84.456	127.481	154.731	2233.992
36.55	15.443	43.689	84.702	126.616	155.633	2233.992
36.65	15.763	41.763	85.294	127.343	152.455	2233.992
36.75	15.775	43.938	85.294	126.460	154.616	2233.992
36.85	15.360	43.108	86.666	125.976	151.410	2233.992
36.95	15.479	41.513	84.312	127.412	151.811	2233.992
37.05	15.751	42.161	83.013	128.207	152.612	2233.992
37.15	15.123	43.340	86.752	126.374	153.127	2233.992
37.25	16.012	41.015	83.749	126.339	153.629	2233.992
37.35	15.336	42.892	83.619	128.242	155.418	2233.992
37.45	15.787	42.709	85.121	127.446	151.882	2233.992
37.55	15.633	41.613	83.836	125.492	155.103	2233.992
37.65	16.333	42.543	83.633	127.273	153.858	2233.992
37.75	15.763	42.410	83.330	126.703	151.453	2233.992
37.85	15.004	41.796	86.868	126.305	151.825	2233.992
37.95	16.072	41.430	86.348	124.973	154.144	2233.992
38.05	15.170	42.012	82.247	130.370	154.459	2233.992
38.15	15.621	41.746	85.828	126.997	150.680	2233.992
38.25	14.719	40.833	82.218	124.731	152.870	2233.992
38.35	15.265	42.261	82.565	125.492	153.256	2233.992
38.45	15.277	42.277	84.182	127.619	153.629	2233.992
38.55	16.344	42.061	84.009	124.523	152.770	2233.992
38.65	15.941	42.078	83.792	125.630	151.023	2233.992
38.75	15.704	42.975	83.576	128.830	151.324	2233.992
38.85	15.146	41.796	83.229	127.689	154.015	2233.992
38.95	15.870	42.427	83.316	127.343	154.631	2233.992
39.05	14.969	42.526	83.475	127.152	154.359	2233.992
39.15	15.716	42.327	85.409	127.377	152.584	2233.992
39.25	15.431	41.447	81.597	126.754	152.383	2233.992
39.35	15.467	43.257	84.428	126.339	150.751	2233.992

Midpoint	10	3	1	0.33	0.1	Volume
39.45	15.479	41.729	85.496	124.817	150.980	2233.992
39.55	14.731	40.185	82.911	125.474	152.584	2233.992
39.65	15.301	40.418	84.009	125.786	154.674	2233.992
39.75	16.439	42.144	83.244	126.703	153.829	2233.992
39.85	16.748	41.729	84.933	123.710	150.608	2233.992
39.95	15.407	41.696	84.355	126.063	154.230	2233.992
40.05	15.704	40.982	84.919	126.910	151.868	2233.992
40.15	15.467	41.978	85.236	125.942	153.213	2233.992
40.25	15.704	43.091	83.619	125.094	151.882	2233.992
40.35	15.621	41.912	84.327	125.769	157.007	2233.992
40.45	15.324	42.526	85.669	124.489	154.960	2233.992
40.55	15.680	42.095	84.240	126.530	153.271	2233.992
40.65	15.704	40.135	83.648	126.132	155.260	2233.992
40.75	15.965	42.576	84.399	123.970	153.972	2233.992
40.85	15.787	42.958	85.886	124.471	150.837	2233.992
40.95	16.214	41.065	84.817	125.405	152.498	2233.992
41.05	15.716	42.095	83.576	126.097	155.447	2233.992
41.15	15.751	40.683	82.724	127.498	151.567	2233.992
41.25	16.439	41.929	83.864	125.405	151.739	2233.992
41.35	15.407	42.028	81.150	125.250	154.273	2233.992
41.45	16.973	41.862	81.843	125.993	152.727	2233.992
41.55	15.811	41.846	83.605	124.004	153.657	2233.992
41.65	14.755	43.041	84.716	125.405	153.714	2233.992
41.75	16.000	42.111	84.485	126.460	153.027	2233.992
41.85	15.419	40.899	85.973	129.505	152.999	2233.992
41.95	16.285	42.360	82.810	124.195	149.950	2233.992
42.05	15.763	42.942	84.124	125.924	154.015	2233.992
42.15	15.763	41.115	84.745	127.740	153.514	2233.992
42.25	15.941	41.082	84.442	122.915	154.402	2233.992
42.35	15.502	42.825	83.503	126.945	154.201	2233.992
42.45	15.763	41.646	84.904	127.187	152.584	2233.992
42.55	14.719	42.128	83.532	127.377	154.029	2233.992
42.65	15.965	42.859	81.886	129.401	155.074	2233.992
42.75	15.645	39.886	85.121	123.779	152.827	2233.992
42.85	16.155	40.916	82.579	126.322	150.579	2233.992
42.95	16.084	41.497	84.168	128.467	152.541	2233.992
43.05	14.814	40.617	85.164	126.426	149.950	2233.992
43.15	16.665	43.008	82.507	127.706	150.522	2233.992
43.25	15.479	43.440	86.637	126.080	150.565	2233.992
43.35	15.396	42.991	83.706	126.824	154.316	2233.992
43.45	15.111	40.351	81.655	126.011	152.999	2233.992
43.55	16.511	41.231	84.312	125.319	151.968	2233.992
43.65	15.419	42.975	83.316	124.350	153.199	2233.992
43.75	14.980	43.174	84.110	123.382	150.193	2233.992
43.85	15.230	41.098	82.132	126.270	153.815	2233.992

Midpoint	10	3	1	0.33	0.1	Volume
43.95	15.977	42.344	83.778	125.457	152.913	2233.992
44.05	15.301	42.526	84.745	128.744	156.205	2233.992
44.15	15.585	42.842	85.395	129.816	152.483	2233.992
44.25	14.874	41.729	82.276	124.592	153.772	2233.992
44.35	15.372	42.045	82.406	124.402	150.207	2233.992
44.45	15.799	42.244	82.854	126.045	152.297	2233.992
44.55	16.084	41.829	82.998	129.193	151.224	2233.992
44.65	15.146	41.879	84.485	127.723	152.254	2233.992
44.75	15.479	42.593	82.594	126.253	150.522	2233.992
44.85	15.621	41.779	83.171	127.567	152.312	2233.992
44.95	15.704	41.082	82.680	125.371	153.915	2233.992
45.05	15.585	41.663	85.352	126.460	152.970	2233.992
45.15	16.024	41.314	84.543	127.671	154.416	2233.992
45.25	15.206	41.978	83.648	130.266	153.886	2233.992
45.35	14.933	42.659	84.919	125.682	151.309	2233.992
45.45	16.060	42.493	83.489	125.734	152.340	2233.992
45.55	16.558	41.879	84.875	122.742	156.005	2233.992
45.65	15.467	41.381	84.168	125.820	150.350	2233.992
45.75	15.562	40.999	82.406	127.031	154.158	2233.992
45.85	15.917	41.696	84.731	126.201	151.896	2233.992
45.95	16.653	41.397	85.395	124.264	152.970	2233.992
46.05	14.909	40.866	83.070	124.817	150.723	2233.992
46.15	15.455	43.025	81.511	123.866	155.074	2233.992
46.25	15.479	40.882	82.522	126.045	151.982	2233.992
46.35	15.479	42.344	82.161	122.032	151.066	2233.992
46.45	16.095	40.517	83.749	127.689	154.860	2233.992
46.55	15.680	41.879	84.471	123.555	154.144	2233.992
46.65	16.084	42.875	83.518	125.734	150.966	2233.992
46.75	15.870	41.414	85.886	124.506	152.082	2233.992
46.85	15.396	41.049	82.507	124.350	153.915	2233.992
46.95	15.597	42.560	83.893	126.339	151.309	2233.992
47.05	15.657	41.414	84.572	125.890	150.708	2233.992
47.15	15.230	42.593	82.883	126.339	151.682	2233.992
47.25	15.514	42.028	87.027	125.526	150.293	2233.992
47.35	15.443	42.659	84.716	125.284	153.786	2233.992
47.45	15.870	40.949	81.958	124.298	149.406	2233.992
47.55	15.526	42.360	85.698	125.907	152.669	2233.992
47.65	15.751	41.962	85.669	125.717	155.604	2233.992
47.75	15.787	41.995	84.586	123.537	154.487	2233.992
47.85	15.965	41.945	81.121	125.180	154.845	2233.992
47.95	16.000	43.357	82.247	124.834	154.015	2233.992
48.05	15.324	41.663	83.778	125.353	153.299	2233.992
48.15	15.621	42.709	82.969	124.575	150.980	2233.992
48.25	15.846	40.949	83.475	125.129	150.307	2233.992
48.35	15.929	43.406	84.919	123.935	153.113	2233.992

Midpoint	10	3	1	0.33	0.1	Volume
48.45	14.316	42.277	84.731	126.754	153.815	2233.992
48.55	15.455	42.809	83.677	128.259	152.354	2233.992
48.65	15.728	42.510	81.655	126.703	150.565	2233.992
48.75	15.704	41.464	82.680	128.951	153.629	2233.992
48.85	14.945	42.028	83.374	128.986	154.373	2233.992
48.95	16.511	43.406	81.222	125.734	150.436	2233.992
49.05	15.467	40.949	84.456	124.938	151.009	2233.992
49.15	15.751	41.165	83.503	125.890	150.894	2233.992
49.25	16.178	42.543	83.590	122.361	152.455	2233.992
49.35	15.597	42.975	82.146	128.173	152.526	2233.992
49.45	15.823	41.215	85.511	127.689	149.105	2233.992
49.55	15.633	42.543	84.254	124.800	154.258	2233.992
49.65	15.751	41.563	83.475	126.685	151.796	2233.992
49.75	15.882	41.115	81.078	125.371	150.766	2233.992
49.85	14.613	41.364	84.110	123.364	150.594	2233.992
49.95	15.787	41.929	83.893	125.353	148.776	2233.992
50.05	15.194	41.414	84.630	125.976	153.571	2233.992
50.15	14.897	40.285	83.013	126.979	153.528	2233.992
50.25	15.941	43.639	83.749	126.322	150.064	2233.992
50.35	15.479	42.194	84.817	125.717	149.062	2233.992
50.45	15.479	41.414	81.901	127.498	151.882	2233.992
50.55	15.716	40.650	84.875	125.007	153.013	2233.992
50.65	14.174	42.609	82.623	125.025	152.770	2233.992
50.75	14.518	41.978	83.244	124.073	155.146	2233.992
50.85	14.909	43.257	83.171	126.080	149.864	2233.992
50.95	15.823	41.879	84.009	121.980	153.858	2233.992
51.05	16.226	42.078	83.041	123.087	149.420	2233.992
51.15	15.609	42.144	83.287	125.578	152.354	2233.992
51.25	15.135	40.484	82.897	126.011	150.236	2233.992
51.35	15.063	40.949	81.742	126.201	151.624	2233.992
51.45	15.775	41.513	83.778	124.091	150.121	2233.992
51.55	15.336	42.925	82.825	123.658	151.839	2233.992
51.65	15.087	42.394	85.323	125.561	152.598	2233.992
51.75	15.775	41.879	83.994	128.467	151.939	2233.992
51.85	16.119	41.829	82.305	125.665	150.021	2233.992
51.95	15.704	41.381	86.695	124.800	152.254	2233.992
52.05	16.261	40.301	81.713	126.703	148.876	2233.992
52.15	14.862	41.713	85.669	124.800	150.307	2233.992
52.25	15.372	41.630	84.442	126.979	149.162	2233.992
52.35	14.779	41.082	83.027	127.862	150.408	2233.992
52.45	16.321	41.198	84.038	126.737	153.528	2233.992
52.55	15.917	42.560	82.565	124.592	151.596	2233.992
52.65	15.775	42.228	84.240	128.536	154.215	2233.992
52.75	15.562	42.012	83.605	125.371	149.821	2233.992
52.85	16.143	41.264	84.168	124.904	151.968	2233.992

Midpoint	10	3	1	0.33	0.1	Volume
52.95	16.155	40.916	85.150	129.816	152.884	2233.992
53.05	15.858	41.945	84.947	126.028	150.866	2233.992
53.15	15.550	40.966	83.807	123.382	153.314	2233.992
53.25	15.348	42.277	83.951	124.350	154.387	2233.992
53.35	15.728	41.563	83.807	124.506	151.109	2233.992
53.45	15.716	41.480	83.720	126.460	151.768	2233.992
53.55	15.633	41.945	84.875	125.890	149.019	2233.992
53.65	16.048	42.012	82.247	123.935	151.882	2233.992
53.75	15.372	42.178	82.579	124.333	150.436	2233.992
53.85	15.633	41.098	82.594	127.083	150.465	2233.992
53.95	15.431	42.228	83.633	124.869	151.037	2233.992
54.05	15.194	40.334	82.103	123.555	148.117	2233.992
54.15	14.838	41.945	83.056	126.426	149.377	2233.992
54.25	15.265	40.783	82.883	124.990	152.569	2233.992
54.35	15.158	42.128	82.262	123.797	152.770	2233.992
54.45	15.633	41.513	83.388	128.242	152.183	2233.992
54.55	15.751	41.281	82.363	124.696	147.688	2233.992
54.65	15.063	42.742	85.785	123.918	153.256	2233.992
54.75	15.479	42.443	82.652	125.993	153.371	2233.992
54.85	15.455	43.573	83.994	125.215	153.586	2233.992
54.95	15.953	42.460	85.048	125.198	153.958	2233.992
55.05	15.467	41.248	83.879	122.032	150.680	2233.992
55.15	15.989	42.975	84.124	123.987	150.594	2233.992
55.25	15.123	42.609	84.009	127.256	154.874	2233.992
55.35	15.941	41.298	83.460	129.003	153.228	2233.992
55.45	15.087	40.052	84.370	126.011	151.753	2233.992
55.55	15.657	42.111	84.283	125.474	152.397	2233.992
55.65	16.333	41.763	83.807	124.817	150.465	2233.992
55.75	16.427	41.597	84.197	122.361	151.997	2233.992
55.85	15.419	42.759	84.745	125.613	153.786	2233.992
55.95	15.265	41.912	81.164	123.710	151.653	2233.992
56.05	15.360	41.513	84.904	126.132	150.150	2233.992
56.15	15.514	43.074	83.547	121.773	150.751	2233.992
56.25	15.668	41.812	84.731	126.045	149.620	2233.992
56.35	15.348	41.497	83.821	127.567	152.641	2233.992
56.45	15.087	40.932	82.955	125.907	152.283	2233.992
56.55	14.945	41.381	84.240	126.841	152.197	2233.992
56.65	15.360	41.364	83.605	125.457	153.085	2233.992
56.75	15.692	42.095	82.421	124.316	153.557	2233.992
56.85	16.048	41.098	86.001	126.426	151.653	2233.992
56.95	15.787	42.377	82.493	126.547	153.700	2233.992
57.05	15.787	41.729	83.532	127.481	151.524	2233.992
57.15	15.846	43.573	83.922	124.367	152.469	2233.992
57.25	15.763	41.580	83.691	125.474	150.379	2233.992
57.35	16.202	41.879	83.142	125.820	153.557	2233.992

Midpoint	10	3	1	0.33	0.1	Volume
57.45	15.621	41.298	84.023	124.125	149.921	2233.992
57.55	16.297	40.318	84.269	126.876	154.774	2233.992
57.65	15.324	41.298	83.244	123.001	150.007	2233.992
57.75	15.989	41.729	82.377	123.330	153.643	2233.992
57.85	15.692	41.347	85.612	126.633	152.684	2233.992
57.95	15.407	41.663	83.836	125.180	150.222	2233.992
58.05	16.119	41.563	84.716	124.852	148.532	2233.992
58.15	15.680	42.427	82.825	125.025	152.841	2233.992
58.25	15.324	42.028	84.399	124.039	151.052	2233.992
58.35	15.882	40.036	86.521	128.329	154.058	2233.992
58.45	15.692	41.314	84.038	120.770	153.528	2233.992
58.55	15.301	40.966	84.254	123.589	150.594	2233.992
58.65	15.882	40.866	83.041	126.720	152.097	2233.992
58.75	15.301	41.563	83.128	123.762	152.612	2233.992
58.85	15.846	42.443	85.453	125.976	150.365	2233.992
58.95	15.087	42.377	82.522	124.056	151.338	2233.992
59.05	14.980	42.095	84.688	125.890	154.387	2233.992
59.15	15.052	40.368	83.648	126.339	151.596	2233.992
59.25	15.716	41.115	85.092	126.391	150.952	2233.992
59.35	15.668	41.281	82.709	127.810	150.494	2233.992
59.45	15.313	41.962	82.406	123.900	154.044	2233.992
59.55	14.992	42.859	84.861	124.471	151.453	2233.992
59.65	15.538	40.866	83.460	124.886	153.471	2233.992
59.75	15.301	41.430	82.002	127.222	153.528	2233.992
59.85	15.467	42.925	83.922	125.855	152.054	2233.992
59.95	15.016	41.829	84.659	124.385	154.072	2233.992
60.05	15.289	41.032	82.493	125.630	154.831	2233.992
60.15	14.992	42.394	82.031	125.838	150.021	2233.992
60.25	15.502	40.750	83.518	122.915	149.019	2233.992
60.35	15.645	41.215	83.994	122.655	149.520	2233.992
60.45	15.799	40.633	85.438	126.443	152.813	2233.992
60.55	14.909	38.641	83.893	125.474	154.029	2233.992
60.65	15.182	41.829	82.464	127.533	154.172	2233.992
60.75	15.787	41.680	82.319	123.503	154.473	2233.992
60.85	15.657	41.779	79.966	125.163	149.921	2233.992
60.95	16.095	41.812	84.355	124.471	151.066	2233.992
61.05	15.052	41.696	85.178	124.246	151.152	2233.992
61.15	15.313	40.351	85.453	123.520	151.567	2233.992
61.25	15.075	41.763	82.854	122.586	149.763	2233.992
61.35	15.052	41.331	82.392	126.339	150.264	2233.992
61.45	16.451	43.871	85.265	124.489	149.706	2233.992
61.55	15.360	41.812	83.879	123.970	150.622	2233.992
61.65	15.668	42.593	83.850	127.325	150.035	2233.992
61.75	16.273	43.805	84.688	128.104	154.258	2233.992
61.85	15.751	40.799	82.998	126.824	152.698	2233.992

Midpoint	10	3	1	0.33	0.1	Volume
61.95	15.633	41.513	85.381	122.119	152.512	2233.992
62.05	15.040	43.091	86.117	126.080	152.999	2233.992
62.15	15.419	42.709	84.225	124.298	149.864	2233.992
62.25	15.336	41.513	83.056	121.721	152.483	2233.992
62.35	14.933	40.501	82.406	126.339	152.068	2233.992
62.45	15.929	40.550	81.150	124.921	153.213	2233.992
62.55	15.597	40.999	83.547	126.201	154.029	2233.992
62.65	15.455	42.111	82.565	125.613	153.213	2233.992
62.75	15.633	42.394	82.377	123.676	152.526	2233.992
62.85	14.743	42.012	81.958	122.915	151.138	2233.992
62.95	15.502	42.892	83.532	126.045	153.829	2233.992
63.05	15.396	40.899	80.746	125.976	149.892	2233.992
63.15	15.502	42.925	84.254	122.067	149.320	2233.992
63.25	16.107	41.513	82.421	123.174	151.066	2233.992
63.35	15.146	42.443	83.937	122.880	152.168	2233.992
63.45	16.368	42.327	83.792	125.423	152.140	2233.992
63.55	15.811	41.231	81.973	125.596	153.042	2233.992
63.65	15.858	42.477	83.374	127.135	150.136	2233.992
63.75	15.490	41.181	82.623	126.201	151.152	2233.992
63.85	15.977	41.397	82.262	124.558	152.025	2233.992
63.95	15.455	40.318	81.612	126.772	152.397	2233.992
64.05	15.384	41.397	82.868	126.218	154.545	2233.992
64.15	15.834	40.600	82.883	124.350	154.301	2233.992
64.25	14.874	42.626	82.031	124.869	151.109	2233.992
64.35	15.230	41.298	82.031	128.744	150.751	2233.992
64.45	15.479	42.692	83.330	125.578	152.641	2233.992
64.55	15.123	41.846	83.518	126.945	152.498	2233.992
64.65	15.206	41.381	84.586	125.751	155.404	2233.992
64.75	15.668	39.936	83.287	123.226	151.352	2233.992
64.85	15.882	42.709	84.197	125.059	152.340	2233.992
64.95	15.490	42.045	84.153	126.287	149.649	2233.992
65.05	15.384	40.816	85.641	126.011	152.999	2233.992
65.15	15.609	40.617	84.197	124.212	152.455	2233.992
65.25	15.870	41.862	83.792	124.886	154.201	2233.992
65.35	15.573	40.816	83.532	127.758	152.340	2233.992
65.45	15.692	40.002	83.590	123.952	152.054	2233.992
65.55	15.740	41.314	83.792	124.056	151.610	2233.992
65.65	15.929	41.879	83.734	123.779	153.242	2233.992
65.75	15.455	41.978	83.980	123.312	152.526	2233.992
65.85	15.467	41.513	82.536	125.232	151.882	2233.992
65.95	15.728	40.882	82.363	124.108	152.655	2233.992
66.05	15.704	43.772	83.619	126.409	150.350	2233.992
66.15	15.585	39.787	83.099	123.727	152.727	2233.992
66.25	15.799	39.903	82.204	128.052	155.475	2233.992
66.35	15.324	43.390	84.702	123.762	150.952	2233.992

Midpoint	10	3	1	0.33	0.1	Volume
66.45	16.368	41.397	84.009	126.668	151.037	2233.992
66.55	16.250	42.742	82.536	126.097	151.052	2233.992
66.65	15.728	42.261	83.677	125.232	150.393	2233.992
66.75	14.945	41.115	81.554	125.474	151.768	2233.992
66.85	14.648	41.547	83.374	121.825	152.125	2233.992
66.95	15.787	41.796	81.901	125.907	151.782	2233.992
67.05	15.657	41.995	83.301	123.745	152.197	2233.992
67.15	15.087	42.775	84.124	125.855	153.042	2233.992
67.25	16.024	40.567	82.579	126.443	150.551	2233.992
67.35	15.277	41.447	83.417	125.682	150.952	2233.992
67.45	15.763	43.207	84.745	126.166	151.524	2233.992
67.55	15.289	41.264	84.211	127.412	153.843	2233.992
67.65	14.826	43.240	82.955	123.520	153.399	2233.992
67.75	16.202	40.418	82.839	121.946	150.451	2233.992
67.85	15.301	43.240	83.446	126.789	153.027	2233.992
67.95	15.348	41.763	84.124	124.367	150.064	2233.992
68.05	15.514	41.248	82.421	124.558	152.669	2233.992
68.15	15.170	41.464	82.969	123.070	149.005	2233.992
68.25	15.075	40.600	82.247	125.457	152.183	2233.992
68.35	16.012	42.809	84.240	130.681	152.183	2233.992
68.45	15.858	41.680	82.348	124.610	150.995	2233.992
68.55	14.969	40.783	82.175	123.209	151.338	2233.992
68.65	15.324	40.916	84.269	124.073	152.369	2233.992
68.75	15.846	41.198	84.991	124.419	152.827	2233.992
68.85	16.119	41.264	83.763	125.492	152.082	2233.992
68.95	15.253	42.709	84.514	129.090	151.352	2233.992
69.05	16.226	41.215	83.359	124.783	153.757	2233.992
69.15	14.708	42.543	82.233	125.094	150.995	2233.992
69.25	15.016	40.799	83.980	125.405	154.187	2233.992
69.35	15.158	41.929	81.453	124.696	152.626	2233.992
69.45	15.396	40.949	81.699	124.125	152.541	2233.992
69.55	15.431	42.377	84.038	126.858	150.093	2233.992
69.65	15.597	42.327	82.724	123.745	150.365	2233.992
69.75	16.107	40.916	81.727	122.257	150.479	2233.992
69.85	15.668	41.580	83.171	120.649	152.011	2233.992
69.95	15.230	41.597	82.247	121.929	150.436	2233.992
70.05	14.945	42.144	80.904	125.215	153.457	2233.992
70.15	15.573	41.929	82.810	124.056	150.035	2233.992
70.25	15.324	40.932	82.262	124.765	154.015	2233.992
70.35	14.803	40.932	82.984	125.163	152.226	2233.992
70.45	15.313	40.949	83.301	126.080	150.264	2233.992
70.55	14.660	42.958	83.633	127.706	151.052	2233.992
70.65	15.585	41.680	84.153	122.586	149.721	2233.992
70.75	15.775	41.331	83.475	124.592	153.514	2233.992
70.85	15.170	42.560	84.832	125.544	153.800	2233.992

Midpoint	10	3	1	0.33	0.1	Volume
70.95	15.834	42.178	83.359	128.450	150.923	2233.992
71.05	15.870	42.294	81.872	122.413	152.269	2233.992
71.15	15.265	41.513	83.994	126.841	153.285	2233.992
71.25	15.657	41.995	84.572	123.503	151.267	2233.992
71.35	15.396	41.929	84.197	128.796	152.025	2233.992
71.45	15.526	41.530	83.316	123.018	152.555	2233.992
71.55	15.004	41.298	83.085	126.339	153.586	2233.992
71.65	15.906	42.443	83.619	126.547	151.567	2233.992
71.75	15.502	42.012	84.976	125.855	152.197	2233.992
71.85	15.751	42.344	82.218	123.209	150.179	2233.992
71.95	15.704	41.364	84.269	126.737	155.647	2233.992
72.05	15.680	42.892	82.175	126.962	151.896	2233.992
72.15	15.621	40.401	82.348	125.111	153.614	2233.992
72.25	15.751	41.746	84.283	124.004	151.496	2233.992
72.35	14.933	42.161	83.301	125.180	154.645	2233.992
72.45	16.119	42.144	82.507	123.883	155.160	2233.992
72.55	15.265	41.962	84.846	125.769	151.009	2233.992
72.65	15.075	40.501	83.792	125.872	149.892	2233.992
72.75	15.063	42.792	85.568	126.945	150.393	2233.992
72.85	15.799	43.174	83.128	123.243	150.121	2233.992
72.95	15.016	41.331	83.547	125.336	148.661	2233.992
73.05	15.716	41.763	83.258	126.097	151.911	2233.992
73.15	15.573	41.895	84.283	125.457	151.825	2233.992
73.25	15.894	42.277	85.568	125.924	150.336	2233.992
73.35	14.779	42.144	81.597	126.547	153.428	2233.992
73.45	15.906	42.360	82.911	125.526	152.927	2233.992
73.55	15.479	42.277	83.215	129.626	151.639	2233.992
73.65	15.313	41.480	83.792	128.986	151.295	2233.992
73.75	15.016	40.750	82.074	124.886	153.543	2233.992
73.85	15.645	42.792	84.428	123.710	148.761	2233.992
73.95	15.965	41.895	83.951	125.647	151.052	2233.992
74.05	15.763	42.460	84.543	127.810	154.759	2233.992
74.15	15.419	41.746	82.911	124.990	149.134	2233.992
74.25	15.597	43.855	83.893	124.644	153.600	2233.992
74.35	14.684	42.609	82.565	126.685	150.823	2233.992
74.45	15.099	42.443	82.998	123.952	150.551	2233.992
74.55	15.253	41.630	86.088	124.281	154.301	2233.992
74.65	15.799	43.788	82.117	126.218	152.898	2233.992
74.75	15.052	41.430	83.503	126.633	151.939	2233.992
74.85	15.313	40.982	83.879	125.163	151.123	2233.992
74.95	15.787	41.630	84.529	127.446	152.641	2233.992
75.05	15.182	41.298	83.561	125.561	153.886	2233.992
75.15	15.277	41.281	85.626	127.948	151.968	2233.992
75.25	15.182	42.244	84.976	126.080	152.698	2233.992
75.35	15.585	40.700	81.641	125.786	151.252	2233.992

Midpoint	10	3	1	0.33	0.1	Volume
75.45	15.550	41.895	83.879	128.277	151.138	2233.992
75.55	15.182	41.962	82.868	123.745	154.974	2233.992
75.65	14.470	41.049	82.637	124.783	153.199	2233.992
75.75	15.894	42.078	83.879	126.582	151.352	2233.992
75.85	15.645	42.410	83.619	127.464	152.956	2233.992
75.95	15.989	42.327	83.114	125.751	154.301	2233.992
76.05	14.791	42.925	83.763	125.976	151.581	2233.992
76.15	15.929	41.248	83.417	125.059	153.471	2233.992
76.25	15.407	42.775	83.677	124.765	150.365	2233.992
76.35	15.621	42.643	82.522	124.852	148.661	2233.992
76.45	15.585	42.228	83.691	124.956	152.211	2233.992
76.55	15.799	42.311	82.002	126.789	155.432	2233.992
76.65	15.562	42.942	83.446	126.651	151.267	2233.992
76.75	14.992	43.373	83.186	124.298	152.956	2233.992
76.85	15.170	41.796	81.872	124.264	149.778	2233.992
76.95	15.550	42.626	82.045	125.284	151.925	2233.992
77.05	16.392	42.360	84.428	124.644	154.187	2233.992
77.15	15.348	41.978	84.861	125.077	151.968	2233.992
77.25	14.921	41.812	84.745	126.876	149.935	2233.992
77.35	14.886	40.301	81.366	124.229	155.175	2233.992
77.45	16.617	42.228	84.370	126.876	154.001	2233.992
77.55	16.072	42.028	84.644	124.679	152.211	2233.992
77.65	15.158	42.759	85.005	126.339	151.281	2233.992
77.75	15.360	40.567	81.785	125.613	153.900	2233.992
77.85	15.538	41.879	83.691	121.686	154.172	2233.992
77.95	15.870	41.846	84.919	125.838	151.052	2233.992
78.05	15.941	42.659	83.301	122.171	148.933	2233.992
78.15	15.834	41.215	84.644	125.526	151.367	2233.992
78.25	15.787	41.929	83.937	122.499	150.064	2233.992
78.35	15.490	42.859	83.691	125.042	151.095	2233.992
78.45	15.396	41.165	83.157	127.464	151.352	2233.992
78.55	14.933	42.311	82.810	129.505	151.224	2233.992
78.65	15.894	42.692	82.319	125.647	152.068	2233.992
78.75	16.155	42.991	82.652	126.547	155.332	2233.992
78.85	15.775	41.812	84.197	126.478	153.142	2233.992
78.95	15.490	43.506	84.399	125.596	152.626	2233.992
79.05	15.882	42.128	83.720	128.363	153.485	2233.992
79.15	15.241	41.978	82.767	125.751	152.641	2233.992
79.25	15.562	41.132	82.421	126.910	153.314	2233.992
79.35	15.692	41.065	85.742	125.526	155.833	2233.992
79.45	14.601	41.314	83.128	125.111	153.986	2233.992
79.55	15.313	42.244	85.973	125.596	155.890	2233.992
79.65	15.419	42.643	81.468	123.849	155.532	2233.992
79.75	15.384	41.895	81.107	126.478	152.226	2233.992
79.85	16.261	42.394	84.890	123.676	153.299	2233.992

Midpoint	10	3	1	0.33	0.1	Volume
79.95	16.107	42.377	83.142	124.869	155.217	2233.992
80.05	15.384	41.929	83.951	123.520	154.444	2233.992
80.15	15.455	40.351	82.825	125.215	151.997	2233.992
80.25	15.206	41.331	84.399	123.658	152.712	2233.992
80.35	15.787	42.792	83.013	127.325	153.056	2233.992
80.45	15.906	40.019	83.763	125.769	153.170	2233.992
80.55	16.202	40.882	83.475	126.720	152.397	2233.992
80.65	15.597	42.111	85.236	127.429	150.866	2233.992
80.75	14.257	41.132	81.973	124.143	153.457	2233.992
80.85	15.016	41.397	83.345	124.506	153.414	2233.992
80.95	15.419	41.049	84.615	127.152	155.389	2233.992
81.05	15.538	41.746	82.132	126.633	153.514	2233.992
81.15	14.992	41.364	83.922	124.143	153.858	2233.992
81.25	15.170	42.178	84.760	123.468	150.794	2233.992
81.35	15.087	42.443	85.409	122.638	156.220	2233.992
81.45	15.775	43.141	82.478	127.014	152.455	2233.992
81.55	15.917	41.248	84.904	125.215	152.941	2233.992
81.65	15.075	42.128	82.594	124.246	150.980	2233.992
81.75	16.380	42.360	81.525	126.391	150.436	2233.992
81.85	15.265	42.012	81.641	126.530	153.886	2233.992
81.95	16.107	42.643	84.240	126.201	152.226	2233.992
82.05	15.324	40.584	82.796	126.979	156.148	2233.992
82.15	15.490	41.215	83.807	124.662	151.982	2233.992
82.25	15.479	40.567	84.298	124.662	151.395	2233.992
82.35	15.894	41.796	82.478	124.592	151.825	2233.992
82.45	15.170	41.082	82.940	124.108	150.279	2233.992
82.55	16.000	41.547	84.341	123.883	154.731	2233.992
82.65	15.704	42.144	83.258	125.059	150.665	2233.992
82.75	16.072	41.397	83.287	126.460	152.927	2233.992
82.85	14.992	41.148	86.738	122.638	150.422	2233.992
82.95	15.716	41.995	83.937	127.170	151.295	2233.992
83.05	15.407	40.401	84.875	124.956	152.168	2233.992
83.15	15.407	41.480	83.503	124.886	152.111	2233.992
83.25	15.170	40.650	83.085	126.582	150.837	2233.992
83.35	15.704	40.119	82.940	124.990	152.383	2233.992
83.45	15.218	44.021	82.348	125.526	154.187	2233.992
83.55	15.526	41.347	83.778	123.451	152.555	2233.992
83.65	15.016	41.364	82.291	124.765	155.132	2233.992
83.75	15.834	43.506	84.529	125.907	152.598	2233.992
83.85	15.490	43.523	85.020	125.769	154.502	2233.992
83.95	15.645	40.932	84.947	128.052	151.739	2233.992
84.05	15.633	41.480	83.229	126.478	152.440	2233.992
84.15	16.226	42.128	83.893	123.762	153.342	2233.992
84.25	16.107	42.593	82.031	125.353	151.853	2233.992
84.35	15.194	41.464	81.265	128.069	152.197	2233.992

Midpoint	10	3	1	0.33	0.1	Volume
84.45	15.657	42.311	81.583	125.371	152.999	2233.992
84.55	15.550	41.580	81.251	125.855	152.856	2233.992
84.65	15.277	41.912	81.626	125.786	154.301	2233.992
84.75	16.178	41.314	82.045	128.502	152.068	2233.992
84.85	16.487	42.842	80.861	125.198	150.995	2233.992
84.95	15.811	43.224	84.370	125.302	152.097	2233.992
85.05	15.170	41.198	82.291	125.578	153.629	2233.992
85.15	15.502	40.418	83.013	122.551	154.659	2233.992
85.25	14.459	41.978	83.922	125.042	151.667	2233.992
85.35	15.692	42.560	84.240	124.056	154.029	2233.992
85.45	16.226	41.530	82.724	127.896	152.612	2233.992
85.55	15.135	40.766	83.605	125.250	151.123	2233.992
85.65	14.364	42.045	83.994	125.838	152.397	2233.992
85.75	15.182	42.061	82.536	123.503	152.999	2233.992
85.85	16.321	41.082	82.435	125.423	151.925	2233.992
85.95	15.336	41.613	83.359	124.973	153.743	2233.992
86.05	16.107	39.820	85.482	126.824	149.005	2233.992
86.15	15.562	42.775	84.197	123.122	152.326	2233.992
86.25	15.384	42.161	84.341	126.668	152.068	2233.992
86.35	15.253	41.480	83.489	124.748	153.242	2233.992
86.45	15.633	41.846	81.583	124.419	150.923	2233.992
86.55	15.218	41.929	83.489	123.935	150.737	2233.992
86.65	16.072	41.929	83.171	124.575	153.815	2233.992
86.75	15.811	40.849	81.828	123.624	151.438	2233.992
86.85	15.573	41.430	83.807	124.800	154.287	2233.992
86.95	15.324	42.144	83.951	125.007	150.565	2233.992
87.05	15.135	41.962	81.280	124.765	148.404	2233.992
87.15	15.111	43.025	83.561	126.305	152.698	2233.992
87.25	15.170	42.942	84.803	123.105	150.422	2233.992
87.35	15.621	41.995	84.673	126.443	149.907	2233.992
87.45	15.004	40.932	85.207	127.862	152.741	2233.992
87.55	16.321	42.311	83.402	124.350	152.469	2233.992
87.65	15.336	40.683	82.045	125.526	150.035	2233.992
87.75	14.613	41.597	82.276	125.907	152.154	2233.992
87.85	15.550	42.510	82.579	123.555	148.876	2233.992
87.95	15.277	42.692	80.240	122.413	153.829	2233.992
88.05	15.158	40.501	80.312	124.229	146.085	2233.992
88.15	15.301	40.401	83.330	124.333	150.751	2233.992
88.25	16.261	40.501	83.316	124.506	146.714	2233.992
88.35	15.479	40.683	82.738	124.385	149.749	2233.992
88.45	15.799	40.932	81.265	123.831	147.359	2233.992
88.55	15.502	40.833	81.265	123.900	149.291	2233.992
88.65	15.253	42.111	82.305	122.603	150.007	2233.992
88.75	14.542	41.763	81.901	121.116	152.068	2233.992
88.85	15.146	42.144	82.781	121.012	150.680	2233.992

Midpoint	10	3	1	0.33	0.1	Volume
88.95	14.803	40.368	84.500	119.317	147.845	2233.992
89.05	15.099	41.846	80.688	123.555	151.009	2233.992
89.15	14.874	40.168	82.608	121.444	152.011	2233.992
89.25	14.245	41.165	80.558	122.638	150.307	2233.992
89.35	15.834	39.488	81.857	122.032	148.489	2233.992
89.45	15.419	40.750	83.417	123.520	148.046	2233.992
89.55	14.969	39.637	82.204	122.534	147.502	2233.992
89.65	15.052	40.052	80.197	123.762	148.003	2233.992
89.75	15.063	40.467	80.356	122.188	150.708	2233.992
89.85	14.838	40.633	83.056	122.863	148.962	2233.992
89.95	14.814	41.115	78.796	122.586	142.806	2233.992
90.05	14.945	40.567	81.193	121.254	149.592	2233.992
90.15	14.091	40.534	79.706	123.572	146.500	2233.992
90.25	15.692	41.796	79.330	120.579	147.802	2233.992
90.35	14.043	41.796	77.049	120.995	145.197	2233.992
90.45	15.087	39.172	80.197	117.933	144.066	2233.992
90.55	14.209	38.608	78.103	121.081	144.052	2233.992
90.65	14.636	40.816	80.919	119.161	145.541	2233.992
90.75	14.482	39.106	80.370	120.476	145.784	2233.992
90.85	15.099	40.069	81.265	118.452	147.917	2233.992
90.95	14.684	38.425	81.583	119.005	144.725	2233.992
91.05	15.277	39.305	78.666	118.521	141.647	2233.992
91.15	13.949	39.471	79.388	117.985	144.624	2233.992
91.25	14.352	38.608	79.764	115.373	141.504	2233.992
91.35	14.459	39.156	78.984	116.203	141.461	2233.992
91.45	14.364	40.301	77.208	118.054	143.651	2233.992
91.55	14.945	37.993	78.767	116.480	141.031	2233.992
91.65	13.996	38.890	76.009	119.420	144.610	2233.992
91.75	14.126	38.807	77.872	116.964	141.432	2233.992
91.85	14.316	38.325	78.219	118.884	141.318	2233.992
91.95	14.387	38.707	77.107	116.047	145.068	2233.992
92.05	14.162	39.023	75.663	118.521	141.389	2233.992
92.15	14.636	38.541	78.608	119.663	137.381	2233.992
92.25	14.340	38.242	77.222	118.244	143.422	2233.992
92.35	14.352	38.508	77.396	112.882	142.363	2233.992
92.45	14.103	38.807	77.757	113.609	141.776	2233.992
92.55	14.482	38.757	78.536	115.269	139.686	2233.992
92.65	13.889	38.807	77.251	116.117	140.788	2233.992
92.75	14.459	38.342	76.053	118.746	142.205	2233.992
92.85	14.411	38.043	77.670	114.664	140.759	2233.992
92.95	14.115	37.063	77.829	115.719	139.958	2233.992
93.05	13.972	39.554	77.078	119.749	140.029	2233.992
93.15	14.577	38.491	77.121	115.494	142.262	2233.992
93.25	13.723	38.973	76.197	114.456	137.997	2233.992
93.35	14.257	36.864	76.139	112.294	143.536	2233.992

Midpoint	10	3	1	0.33	0.1	Volume
93.45	13.782	37.894	76.341	114.940	134.847	2233.992
93.55	14.340	39.471	79.114	115.771	140.487	2233.992
93.65	13.960	38.425	77.612	114.491	141.475	2233.992
93.75	14.292	38.441	76.804	114.404	143.222	2233.992
93.85	14.043	36.748	79.388	114.404	137.853	2233.992
93.95	13.759	37.661	76.269	113.211	136.064	2233.992
94.05	14.530	37.761	76.876	116.238	139.256	2233.992
94.15	14.352	38.226	77.193	113.470	142.749	2233.992
94.25	13.771	39.023	75.576	114.127	137.209	2233.992
94.35	13.830	36.465	77.294	112.605	138.841	2233.992
94.45	13.925	37.495	75.071	115.667	137.710	2233.992
94.55	14.660	37.445	76.212	114.958	137.968	2233.992
94.65	14.470	38.143	75.085	113.349	136.479	2233.992
94.75	14.589	37.445	76.038	114.733	138.913	2233.992
94.85	13.913	37.644	78.219	113.332	138.884	2233.992
94.95	13.866	38.923	75.215	115.131	138.111	2233.992
95.05	14.387	37.943	74.176	115.321	140.401	2233.992
95.15	14.233	38.242	75.143	112.277	140.573	2233.992
95.25	14.364	38.840	74.739	117.033	139.843	2233.992
95.35	13.557	38.026	75.143	115.200	141.418	2233.992
95.45	13.652	37.445	77.107	116.480	136.966	2233.992
95.55	14.897	38.823	75.865	115.079	138.669	2233.992
95.65	13.213	38.076	75.952	115.719	136.121	2233.992
95.75	13.628	37.827	75.143	112.778	138.054	2233.992
95.85	14.589	38.292	74.941	113.505	139.156	2233.992
95.95	14.328	38.159	76.443	113.038	134.117	2233.992
96.05	13.889	37.379	75.360	112.761	137.266	2233.992
96.15	13.605	39.106	75.085	112.882	136.737	2233.992
96.25	14.186	37.844	76.125	112.588	135.821	2233.992
96.35	13.771	37.694	74.869	117.552	136.107	2233.992
96.45	13.711	37.794	74.378	113.539	138.311	2233.992
96.55	13.628	37.611	76.746	115.131	134.833	2233.992
96.65	13.842	37.744	76.645	113.833	140.058	2233.992
96.75	13.616	36.698	74.970	114.750	134.174	2233.992
96.85	13.877	36.897	75.244	113.609	140.459	2233.992
96.95	13.628	37.628	74.508	114.716	138.498	2233.992
97.05	13.972	37.196	75.302	114.837	136.021	2233.992
97.15	13.545	37.761	76.428	113.868	138.626	2233.992
97.25	14.209	37.910	75.013	116.376	138.082	2233.992
97.35	14.198	37.844	74.190	114.508	139.228	2233.992
97.45	14.067	38.807	74.233	115.373	134.160	2233.992
97.55	14.399	38.143	75.562	115.131	137.381	2233.992
97.65	13.450	38.176	76.067	114.923	138.755	2233.992
97.75	13.261	36.930	76.919	115.615	138.297	2233.992
97.85	14.115	38.026	76.067	114.923	138.569	2233.992

Midpoint	10	3	1	0.33	0.1	Volume
97.95	13.901	36.831	74.724	116.791	138.784	2233.992
98.05	13.450	37.462	77.280	114.681	139.729	2233.992
98.15	13.557	36.216	77.193	114.491	142.363	2233.992
98.25	14.565	36.665	75.417	116.497	138.240	2233.992
98.35	14.209	36.997	75.201	112.882	140.187	2233.992
98.45	13.711	37.910	75.201	115.459	138.755	2233.992
98.55	15.087	39.587	78.190	113.574	138.855	2233.992
98.65	13.996	38.408	76.009	114.422	139.385	2233.992
98.75	14.340	37.263	76.515	114.854	139.414	2233.992
98.85	14.257	38.342	75.345	115.010	137.224	2233.992
98.95	13.344	39.006	77.468	111.464	139.671	2233.992
99.05	13.782	37.047	75.518	114.352	139.285	2233.992
99.15	14.376	38.441	75.143	115.390	138.970	2233.992
99.25	14.328	36.997	74.869	116.203	138.154	2233.992
99.35	13.723	40.036	76.313	115.580	139.013	2233.992
99.45	13.676	38.358	76.500	110.720	136.522	2233.992
99.55	13.759	37.844	74.898	114.024	136.608	2233.992
99.65	13.782	38.956	76.009	114.283	138.068	2233.992
99.75	3.784	24.925	20.995	74.448	36.432	2233.992
99.85	0.000	0.000	0.000	0.000	0.000	2233.992
99.95	0.000	0.000	0.000	0.000	0.000	2233.992
